# Independent *Polled* Mutations Leading to Complex Gene Expression Differences in Cattle

**DOI:** 10.1371/journal.pone.0093435

**Published:** 2014-03-26

**Authors:** Natalie Wiedemar, Jens Tetens, Vidhya Jagannathan, Annie Menoud, Samuel Neuenschwander, Rémy Bruggmann, Georg Thaller, Cord Drögemüller

**Affiliations:** 1 Institute of Genetics, University of Bern, Bern, Switzerland; 2 Institute of Animal Breeding and Husbandry, Christian-Albrechts-University Kiel, Kiel, Germany; 3 Interfaculty Bioinformatics Unit, University of Bern, Bern, Switzerland; 4 Vital-IT, Swiss Institute of Bioinformatics, Lausanne, Switzerland; University of Queensland, Australia

## Abstract

The molecular regulation of horn growth in ruminants is still poorly understood. To investigate this process, we collected 1019 hornless (polled) animals from different cattle breeds. High-density SNP genotyping confirmed the presence of two different polled associated haplotypes in Simmental and Holstein cattle co-localized on BTA 1. We refined the critical region of the Simmental *polled* mutation to 212 kb and identified an overlapping region of 932 kb containing the Holstein *polled* mutation. Subsequently, whole genome sequencing of polled Simmental and Holstein cows was used to determine polled associated genomic variants. By genotyping larger cohorts of animals with known horn status we found a single perfectly associated insertion/deletion variant in Simmental and other beef cattle confirming the recently published possible *Celtic polled* mutation. We identified a total of 182 sequence variants as candidate mutations for polledness in Holstein cattle, including an 80 kb genomic duplication and three SNPs reported before. For the first time we showed that hornless cattle with scurs are obligate heterozygous for one of the *polled* mutations. This is in contrast to published complex inheritance models for the bovine scurs phenotype. Studying differential expression of the annotated genes and loci within the mapped region on BTA 1 revealed a locus *(LOC100848215*), known in cow and buffalo only, which is higher expressed in fetal tissue of wildtype horn buds compared to tissue of polled fetuses. This implicates that the presence of this long noncoding RNA is a prerequisite for horn bud formation. In addition, both transcripts associated with polledness in goat and sheep (*FOXL2* and *RXFP2*), show an overexpression in horn buds confirming their importance during horn development in cattle.

## Introduction

**Figure 1:**
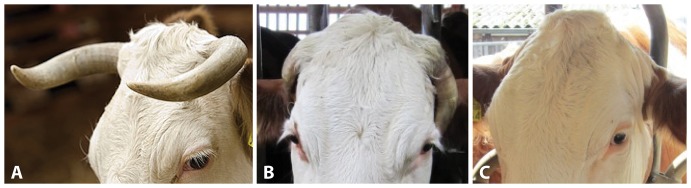
Horn phenotypes in Simmental cattle. Normally horned cow (**A**), cow with loosely attached small horns termed scurs (**B**), smooth polled cow showing the typical peaked shape of the proximal frontal bone (**C**).

Permanent horns (Figure 1A) are a typical feature of domesticated ruminants like cattle, sheep and goats. Consisting of an outer keratin-layer and a bony pneumatized core [1] they are important for the animal's self-defense in wildlife. Nevertheless there is evidence for the existence of hornless cattle until back to ancient times, as for example shown in several Old Egyptian tomb sceneries [2]. Besides the absence of horn growth (polledness, Figure 1C) these animals may show atypical eyelashes and defects of the genital tract in males [3].

Since there is no need for self-defense in modern intensive cattle production, and new housing systems as free stalls with headlock barriers have been established, horns have become an undesired trait in beef and dairy cattle. To avoid the risk of injury to humans and animals, most of the cattle are dehorned at a young age. Due to animal welfare [4] and economic reasons, efforts are taken to breed hornless (polled) cattle. There are several cattle breeds such as Angus and Galloway, which are fixed for the autosomal dominant *polled* allele [5]–[7]. In other breeds natural polledness has until now just been accomplished in a small part of the populations by cross breeding or selection of polled individuals.

The *polled* locus (*P*) has previously been mapped on the proximal end of BTA 1 [8]–[13] corresponding to a region on HSA 21 [14]. The apparent causative mutation for polledness in various beef or dual-purpose cattle breeds with a Celtic origin has been described as a structural sequence variant, a complex insertion-deletion affecting an intergenic region of BTA 1 [15]. Studying the *polled* mutation in cattle with a Friesian origin like Holstein and Jersey showed allelic heterogeneity. A second polled associated haplotype has been detected and four intergenic candidate variants, three SNPs and an 80 kb duplication, are discussed as possibly causative for the Friesian polledness [15]. Recently, a SNP within intron 3 of *IFGR2* has been described to be in perfect association with polledness in Holstein cattle by another group [16]. A further dominantly inherited polled phenotype accompanied by congenital malformations has been shown to be caused by a *ZEB2* mutation in French Charolais cattle [17]. This report confirms the assumption that other mutations beside the characterized *polled* alleles on BTA 1 may affect horn growth in cattle. In goats, the polled intersex syndrome (PIS) is caused by the deletion of an 11.7 kb DNA element located at goat chromosome 1q43, which affects the transcription of at least two flanking genes, *FOXL2* and a noncoding RNA [18], [19]. A polymorphism at *RXFP2* explains horn variability in sheep [20], [21].

A second locus affecting horn growth in cattle is called *Scurs* (*Sc*) [5], [6]. This interacting second mutation causes scurs in cattle which carry the *polled* mutation. Scurs are corneous growths of different sizes from crusts up to big horn-like formations, which develop in the same area as horns but are not firmly attached to the skull (Figure 1B). In Angus and Galloway scurs have been described to develop depending on the sex and *polled* genotype [6]. Whereas homozygous *polled* animals develop scurs only if they also carry the *Sc* mutation in a homozygous state, heterozygous *polled* females develop scurs only if they carry the *Sc* mutation in a homozygous state. Heterozygous *polled* males develop scurs in the presence of one or two *Sc* alleles [6]. The mode of inheritance of the *scurs* mutation is still under debate [6], [22]. One report stated that the *scurs* locus maps to BTA 19 in Canadian beef cattle based on linkage analysis [23]. This was not confirmed in French Charolais cattle showing polledness and scurs [22]. A recent study in French Charolais cattle showing a scurs-like phenotype revealed a dominantly inherited causative *TWIST1* mutation in the absence of the *polled* mutation [24].

Comparative studies were performed to uncover differential gene expression associated with the development of horns and scurs in cattle [3], [25]. Microarray based differential expression studies of epidermal and dermal tissues from the skull region of newborn calves revealed no differentially expressed gene in the region of the *polled* locus on BTA 1 [25]. Recently, differential gene expression analysis has been performed in horn buds and frontal skin of horned and polled fetuses 90 days post-coitum using quantitative RT-PCR [3]. This study was focussed on the expression of known genes involved in ruminant horn growth and annotated genes of the *polled* locus on BTA 1. An increased expression of a long intergenic noncoding RNA (*LincRNA#1*) located at BTA 1 and a reduced expression of *RXFP2* was shown in polled fetuses indicating their role during horn bud agenesis. In addition, *OLIG2* located at BTA 1 and *FOXL2* showed different expression levels between the horn buds and frontal skin in polled as well as in wildtype fetuses.

In the present study we describe our efforts to identify the causative variants for polledness in the Simmental and Holstein breed, which were carried out independently from the recently published studies [3], [15]. Furthermore, we analysed the gene expression to evaluate differentially expressed transcripts in wildtype and polled fetuses during development.

## Results

### Two different polled associated haplotypes are co-localized on BTA 1

**Figure 2:**
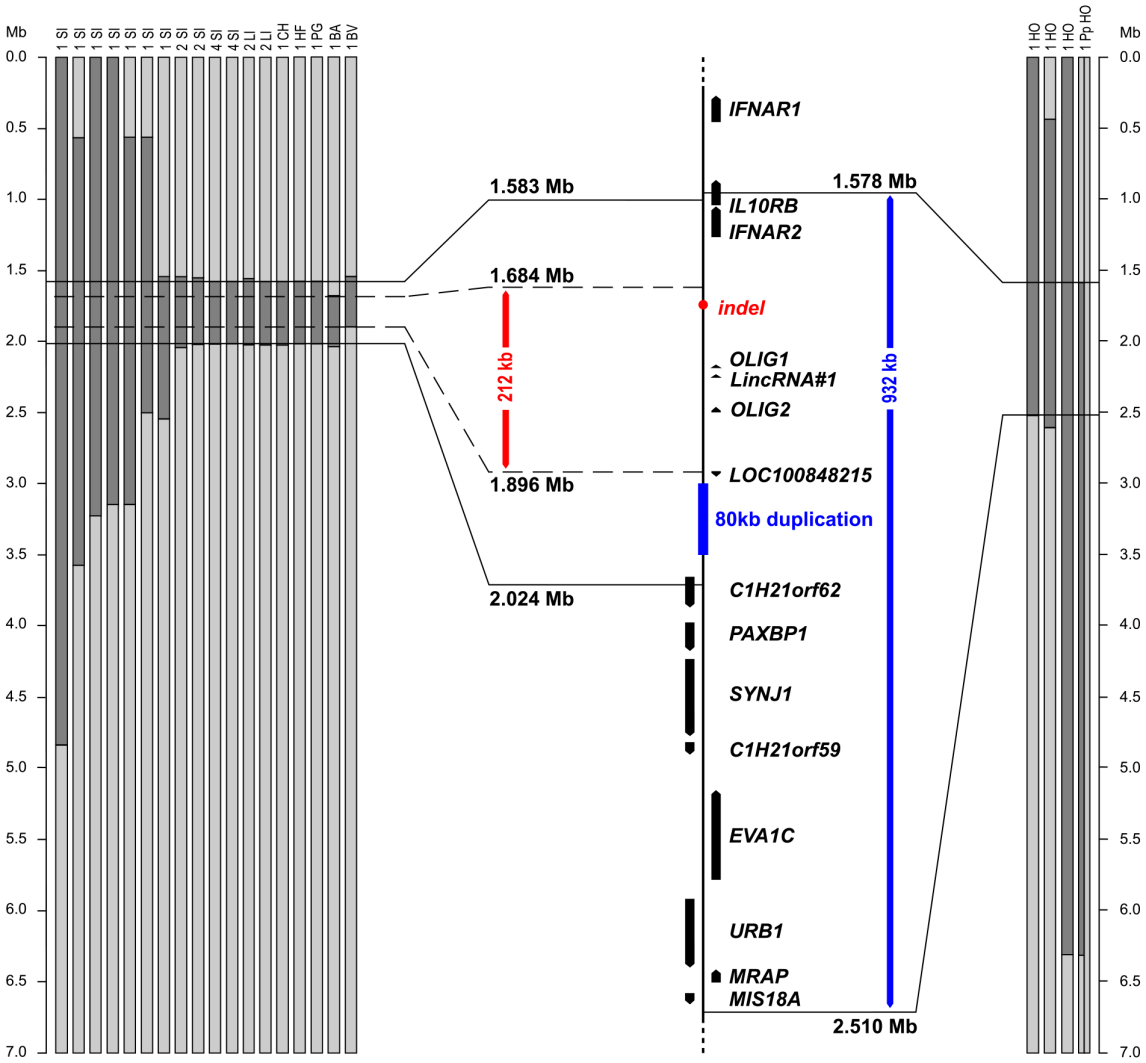
Homozygosity mapping on BTA 1. SNP genotypes of BTA 1 markers are presented as vertical bars. The dark grey segments represent homozygous blocks with shared alleles. A total of 28 progeny tested homozygous polled bulls belonging to beef and dual-purpose breeds of Celtic origin are shown on the left (SI: Simmental, LI: Limousin, CH: Charolais; HF: Hereford, PG: Pinzgauer; BA: Blonde d'Aquitaine, BV: Braunvieh). Some recombinations were observed in more than one animal, the number of animals is displayed above the chromosome bar. The haplotype analysis suggests the position of the Celtic *polled* mutation within a 212 kb interval shown in red. The annotated genes and loci on the BTA 1 segment (UMD3.1 assembly) are shown in the center. Three progeny tested homozygous polled bulls and a single heterozygous polled bull belonging to the Holstein (HO) breed of Friesian origin are shown on the right. The 932 kb critical region of the Friesian *polled* mutation is indicated in blue.

We performed genome-wide homozygosity mapping based on genotypes of 19 progeny tested homozygous polled (*PP*) Simmental bulls genotyped with illumina's bovine HD BeadChip comprising 777,962 SNP markers. Thereby, a 441 kb interval of shared homozygosity (BTA 1: 1,582,828–2,023,687; UMD 3.1 assembly) was identified within the previously mapped *polled* interval. We searched for possible recombinations by manual comparison of the SNP data of 108 genotyped obligate heterozygous polled Simmental cattle with the previously defined polled associated 441 kb haplotype present in the homozygous polled animals. We identified one copy of the entire polled haplotype in each animal and no evidence for further crossing over events. Subsequently, SNP genotypes of nine progeny tested *PP* bulls from other beef and dual-purpose breeds were included in the analysis. In seven out of nine bulls homozygosity for the identical 441 kb polled associated haplotype seen in Simmental was found. However, a polled Blonde d'Aquitaine bull carried a recombinant haplotype adjusting the proximal border of the critical interval to BTA 1: 1,684,495. A Braunvieh bull showed a recombination at BTA 1: 1,896,112 refining the distal border of the polled interval. Thus – assuming that it resides on the common haplotype block – the causative *polled* mutation in these beef breeds is located within a 212 kb interval on BTA 1 (Figure 2). The same approach was applied for three progeny tested homozygous polled (*PP*) and 29 obligate heterozygous polled (*Pp*) bulls of the Holstein breed. Within the previously mapped region on BTA 1 the three *PP* Holstein bulls shared a homozygous haplotype block of 1,606 kb (BTA 1: 903,971–2,509,966). Twenty-eight out of the 29 *Pp* bulls carried one copy of this haplotype. One of the heterozygous Holstein bulls had a recombinant copy of this polled associated haplotype and in comparison to the *PP* bulls it was homozygous for the opposite SNP alleles at several positions proximal of BTA 1: 1,578,430 (Figure 2). Thus, we mapped the *polled* mutation within the Holstein breed to a 932 kb region on BTA 1. Taken together, the analyses revealed two different polled associated haplotypes overlapping within the same chromosomal region on BTA 1.

### Whole genome re-sequencing reveals candidate causal mutations for polledness

We performed whole genome re-sequencing of one homozygous polled (*PP*) Simmental (39.3x mean coverage), one heterozygous (*Pp*) Simmental (21.8x mean coverage) and 1 horned Simmental cattle (23.7x mean coverage). SNPs, short insertions and deletions, and structural variants were called with respect to the UMD3.1 cow reference genome sequence of a horned Hereford cow. The *PP* cow carried 121 homozygous sequence variants within the mapped 212 kb interval. Subsequent filtering for variants that were heterozygous in the *Pp* cow and absent in the horned Simmental cow revealed four associated intergenic variants. The variants were further compared with 33 cow genomes of various horned breeds and 2 genomes of hornless Galloways, one *PP* and one *Pp*, that had been sequenced in our laboratory in the course of other ongoing studies. Assuming that the mutant allele of the causative variant should be completely absent in horned cattle and present in the polled Galloways, a single polled associated variant at BTA 1: 1,706,044 was identified. Sanger sequencing of this variant revealed a duplication of 208 bp (BTA 1: 1,705,837–1,706,044), inserted after 10 bp of the wildtype sequence at BTA 1: 1,706,054 in combination with a 6 bp deletion (BTA 1: 1,706,055–1,706,060) designated as complex insertion-deletion (*indel*, Figure S1A). Subsequently, this *indel* variant was genotyped in a larger cohort of polled and horned cattle from different breeds using fragment length analysis (Figure S1B). Genotyping 2,329 cattle from 30 breeds this complex *indel* was found to be in perfect association with the *polled* locus in various breeds like Angus, Galloway, Blonde d'Aquitaine, Braunvieh, Hereford, Norwegian Red and Pinzgauer (Table S1). Among 403 polled Simmental cattle we identified one single hornless cow with a crust at one side, that did not carry the variant *indel* allele. In addition, we observed two polled Limpurger animals with scurs and one polled Yak, which also did not carry the variant *indel* allele. A total of 239 out of 252 polled Limousin and Limousin crosses and 15 out of 16 polled Charolais cattle carried one or two copies of the variant *indel* allele. The remaining 14 polled Limousin and Charolais animals carried a copy of the second polled associated haplotype found in Holstein cattle (see below). In contrast, among polled Holstein cattle only six out of 167 genotyped animals were found to carry the variant *indel* allele.

To detect possible causal variants for the second polled associated haplotype the whole genome of a single homozygous *PP* Holstein cow was sequenced at 10.1x mean coverage. Within the critical 932 kb interval there were 1,769 variants. Subtracting variants observed in the 33 horned control cattle from different breeds, there were 191 intergenic variants left (Table S2). For subsequent genotyping of more than 400 horned and more than 80 polled Holstein cattle (Table S2) we focused on the 43 variants located within the critical region of 441 kb initially mapped in Simmental cattle, as we assumed that the second polled mutation in Holstein should affect the same chromosome segment as the Simmental mutation and as it is the segment within which the (Holstein) *polled* mutation had also been mapped in a previous publication [3]. This approach enabled us to exclude 9 variants as they occurred also in horned animals. Finally there were 34 variants left that showed perfect association with polledness in Holstein cattle: 31 SNPs, a 1 bp deletion, a short 5 bp deletion/12 bp insertion, and an 80 kb genomic duplication (Table S2). As the large duplication was identified by other groups before [15], we re-analyzed it by Sanger sequencing to determine its precise structure. It represents a tandem duplication of 80,129 bp (BTA 1: 1,909,352–1,989,480) combined with two 2 bp deletions (BTA 1: 1,909,354–1,909,355delTG and BTA 1: 1,909,396–1,909,397delTG) in the duplicated copy (Figure S2). In addition to these 34 variants there were still 148 variants located within the distal region of the mapped 932 kb haplotype. Beside in hornless Holstein cattle the polled Holstein haplotype was found in 20 *Pp* Limousin and Charolais beef cattle, the aforementioned 14 animals not carrying the Simmental *indel* plus six Limousin cattle carrying both polled mutations, the *indel* and the Holstein haplotype, thus being compound polled *PP* homozygotes. The three hornless cattle of the Simmental and Limpurger breed and the polled Yak mentioned above were tested negative for the presence of the sequence variants associated with polledness in Holstein. Finally, we genotyped a recently published *IFNGR2* SNP (BTA 1: 1,390,292, Table S3) supposed to be perfectly associated with the Holstein *polled* mutation [16] in 160 polled Holstein cattle and showed that 4 polled animals (one hornless cow with two polled offspring and one progeny-tested polled bull) did not carry the variant SNP allele. These animals were genotyped as heterozygous carriers of several polled associated Holstein variants and did not carry the previously identified *indel* of polled Simmental cattle. In addition, the variant *IFNGR2* allele was also present in heterozygous state in one of the sequenced control genomes of a horned Brown Swiss cow.

### Male and female cattle with scurs are always heterozygous *Pp*

In regard to a possible influence of the individual polled genotype on the expression of scurs we determined the polled genotype in 207 scurred animals. The animals were tested for the *indel* mutation found in beef and dual-purpose breeds and for the C>A SNP at BTA 1: 1′768′587 associated to the polled mutation in Holstein cattle. Animals were classified as scurred if they showed crusts or horn-like formations that were not firmly attached to the skull. This clearly revealed that regardless of the kind of *polled* mutation and regardless of the breed and sex, scurred animals were heterozygous *Pp* whereas all the homozygous polled animals were smoothly polled without any visible signs of scurs (Table 1).

**Table 1:** Relation of the polled genotype in regard to the expression of scurs in animals of different breeds carrying the *indel* variant or the variants associated with the hornless mutation derived in the Holstein breed.

breed	phenotype	male	male	female	female	total	total
		*Pp*	*PP*	*Pp*	*PP*	*Pp*	*PP*
**with ** ***indel*** ** variant**	**scurred**						
Angus		1		1		**2**	
Braunvieh		1				**1**	
Blonde d'Aquitaine		1					
Charolais		4				**4**	
Holstein		5				**5**	
Limousin		13		16		**29**	
Simmental		38		92		**130**	
**total**		**63**		**109**		**172**	
**with ** ***indel*** ** variant**	**polled**						
Angus				4		**4**	
Braunvieh		3	1			**3**	**1**
Charolais		2	1			**2**	**1**
Galloway		1	8	1		**2**	**8**
Holstein		1				**1**	
Limousin		42	11	110	29	**152**	**40**
Pinzgauer		6				**6**	
Simmental		23	51	119	68	**142**	**119**
**total**		**78**	**72**	**234**	**97**	**312**	**169**
**with Holstein ** ***polled*** ** variants**	scurred						
Holstein		30		3		**33**	
Limousin		1		1		**2**	
**total**		**31**		**4**		**35**	
**with Holstein ** ***polled*** ** variants**	polled						
Charolais			1				**1**
Pinzgauer		12	3	75	18	**87**	**21**
Limousin		2		8		**10**	
**total**		**14**	**4**	**83**	**18**	**97**	**22**

### Gene expression studies reveal known and new horn development specific candidates

Initially, we performed total mRNA sequencing (RNA-Seq) of skin biopsies derived from one polled and one horned fetus to study possible effects of the polled mutation on gene expression. The horned fetus with ∼32 cm crown-rump length, corresponding to an age of approximately 150 days post fertilization [26], and macroscopically visible horn buds was confirmed to be wildtype for both, the Simmental *indel* and the Holstein associated variants. The polled fetus, ∼35 cm from crown to rump and therefore approximately 158 days post fertilization, with a smooth skin without any visible horn buds, was homozygous *PP* for the Simmental *indel*. Both fetuses were tested wildtype for the C>A SNP at BTA 1: 1′768′587 and the 80 kb duplication associated with polledness in Holstein. Biopsies from the horn bud of the wildtype fetus and from that same skin area in the *PP* fetus were used. Mapping the obtained sequence reads to the UMD3.1 cow assembly showed a large number of differentially expressed genes (Table S5). Among the differentially expressed (P value <0.05) transcripts were three genes located within the critical region on BTA 1 (*OLIG1*, *OLIG2, C1H21orf62*) and two genes known to be involved in horn development of sheep and goat (*RXFP2* and *FOXL2*). Visual inspection of the mapped reads within the critical BTA 1 region harboring the *polled* mutations revealed evidence for a spliced transcript at position BTA 1: 1,898,100 to 1,899,400 (spliced between position BTA 1: 1,898,193 and 1,899,327; Figure S3). These reads were present in the horned fetus only and correspond to an uncharacterized locus annotated as *LOC100848215*. The splicing pattern of the obtained sequence reads are similar to those of three ESTs (EH130782, EH138227, EV693397) used for the computer predicted annotation at UCSC genome browser [27]. Cross-species dataset comparison revealed known transcripts of this locus in cow and buffalo only (Figure S4).

**Figure 3:**
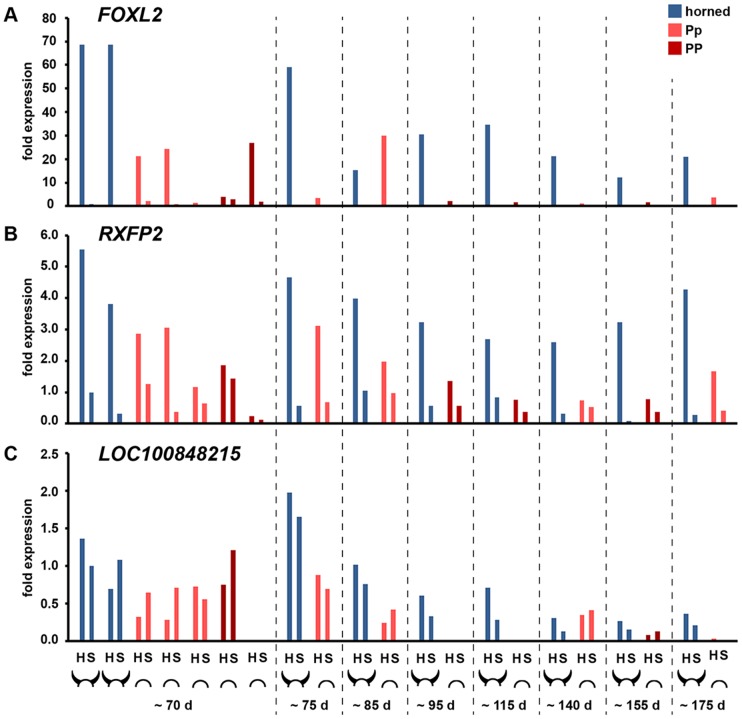
Gene expression study based on RT-PCR. Relative expression of *FOXL2* (A), *RXFP2* (B), and *LOC100848215* (C) transcripts in fetal skin and horn bud biopsies of different developmental stages and different polled genotypes. Different fetal stages are divides in eight groups of estimated age (d). Wildtype fetuses are marked with the shape of a horned cow head, fetuses carrying the *polled* mutation are marked with the shape of a polled cow head, whereas each icon designates one fetus. For each individual a biopsy of the horn bud area (H) and a biopsy of the frontal skin (S) were studied. Expression levels in wildtype fetuses are shown in blue, those of heterozygous *Pp* polled fetuses in orange and those of homozygous *PP* polled fetuses in dark red. Expression levels are normalized to the average of three control genes and shown as relative expression in relation to the frontal skin sample of the youngest horned fetus.

In a second experiment, the gene expression analysis was extended to samples of different developmental stages. Quantitative RT-PCR (qRT-PCR) was performed for six transcripts that were found to be differentially expressed in the aforementioned experiment. Biopsies were taken from horn bud tissue and frontal skin of 21 fetuses measuring 6.8 up to 44 cm from crown to rump (Figure 3). According to the developmental stages, the fetuses were divided into eight case-control groups of matching age. The youngest group comprised two wildtype fetuses and five fetuses carrying at least one copy of the Simmental *indel*, the older groups consisted of one wildtype and polled fetus each. All the polled fetuses were carrying a copy of the *indel* variants, none of the fetuses carried the variants associated with polledness in Holstein. Expression was detectable for five out of the six examined transcripts expression, while it was not possible to amplify the *OLIG1* transcripts in the younger fetuses using different sets of primers. *C1H21orf62* was found to be less expressed in horn buds compared to frontal skin regardless of the polled genotype (Figure S5). *OLIG2* was found to be higher expressed in the early developmental stages compared to older fetuses (Figure S6), whereas no visible difference between the genotypes could be observed across the different stages. *FOXL2* was found to be strongly over-expressed in all the wildtype horn buds compared to the respective wildtype frontal skin area (Figure 3A). The expression of *FOXL2* decreased with increasing age in wildtype fetuses. Within the polled fetuses *FOXL2* showed the same tendency to be higher expressed in the “horn bud” tissue biopsies compared to frontal skin, but the findings in the polled fetuses were not as strong and consistent as in the wildtype fetuses. In general, the expression levels of *FOXL2* were lower in the polled fetuses. As seen in the RNA-Seq experiment before, *RXFP2* was found to be highly expressed in wildtype horn buds. Expression was also detectable at lower levels in polled fetuses, where it was higher expressed in the area of the horn bud compared to frontal skin (Figure 3B). The *LOC100848215* was generally found to be less expressed in the polled fetuses than in the wildtype fetuses regardless of the tissue. In two individual fetuses, a 70 days old *PP* fetus and a 140 days old *Pp* fetus, the expression pattern was comparable with those of the age-matching wildtype fetuses. Expression levels in three out of five homozygous polled fetuses were below the detection threshold. In the horned fetuses we observed a trend of decreasing *LOC100848215* expression level with increasing age of the fetuses and a higher expression in the horn bud compared to the frontal skin (Figure 3C).

## Discussion

### Mapping and mutation analysis

Using high-density SNP genotyping to fine map the bovine *polled* mutation showed the presence of two independent polled associated haplotypes in different cattle breeds. Haplotype analyses revealed a 212 kb polled associated haplotype present in many beef and dual-purpose cattle breeds like Simmental, Angus, Galloway. Thus we were able to improve previous mappings based on illumina's bovineSNP50 BeadChip, where the *polled* locus had been mapped on a 381 kb interval in a mixed breed approach including Holstein and beef breeds [13], [15] and to a 400 kb interval including four different polled haplotypes from Holstein and Charolais cattle respectively [3]. The small size of the mapped haplotypes suggests that the *polled* mutation occurred a long time ago as stated by Allais-Bonnet et al. [3]. In contrast to previous studies [3], [15] we mapped the *polled* mutation in Holstein cattle separately and ended up with a 932 kb polled associated interval. This critical interval includes the mapped interval of 212 kb in polled beef cattle and contains 14 annotated genes and loci.

Whole genome re-sequencing of selected polled and horned individuals from the Simmental and Holstein breed was used for mutation analysis. Filtering of variants present in controls of different breeds was performed to identify sequence variants private to polled animals. In Simmental, we were left with only one single private variant, a complex *indel* within the critical interval. This confirms other recently published results [3], [15]. Using Sanger sequencing, for the first time the precise architecture of this sequence variant was determined (Figure S1). Genotyping more than 2,000 animals the *indel* was found to be in perfect association with polledness in Angus, Galloway, Blonde d'Aquitaine, Braunvieh, Hereford, Norwegian Red and Pinzgauer cattle. As the *indel* variant is present in polled Scottish beef breeds like Angus and Galloway, it is very likely that the *polled* allele in beef and related dual-purpose breeds originates in these Scottish breeds and was introduced through crossbreeding to other breeds long time ago. Therefore, we agree with Medugorac *et al*. who designated this *polled* mutation as Celtic [15]. In Limousin and Charolais cattle the majority of polled animals carry the *indel* variant, nevertheless there are some polled and scurred animals, which do not carry the *indel* but do carry the polled associated haplotype of Holstein cattle, indicating that polledness in these breeds obviously is due to different introgression events from different breeds. In Limousin, we found six polled animals being compound homozygotes carrying the Simmental *indel* as well as the Holstein polled haplotype. This indicates that both *polled* alleles segregate within these two breeds. In Simmental cattle, there was one hornless cow out of 403 polled animals which did not carry a copy of the *indel* variant. Based on pedigree data this cow was found to have horned parents and the owner confirmed that this animal never has been dehorned. Therefore, we speculate that its polledness represents a phenocopy e.g. due to mosaicism without an effect on the germ cell line as no transmission of the polled trait has been observed in a total of 22 offspring of this cow. In addition, two hornless Limpurger cattle, a bull with horned parents and its male offspring, both scurred neither carry the *indel* variant and nor the polled associated Holstein haplotype. Transmission of the polled phenotype from father to son suggests the presence of an inherited *de novo* mutation leading to polledness. Recently, also in the French Charolais cattle population independent polled phenotypes have been reported. Capitan et al. states to have additional cases of newly occurring horn anomalies, which cannot be explained by the known polled associated variants, indicating that *de novo* mutations affecting horn growth sporadically occur [17], [24]. The genotyped individual polled yak from Switzerland doesn't carry any of the polled variants reported in this paper. This indicates the presence of an independent *polled* mutation. According to a recent study the *polled* mutation in domestic yaks was mapped to the same genomic region on BTA 1 [28].

In the critical Holstein polled interval of 931 kb there are a total of 182 private associated candidate variants. Due to the publication of another group [3], the identical phenotype, the initial mapping and the findings in other breeds, we considered the overlapping BTA 1 segment more likely to contain the causal mutation assuming that this independent mutation affects the same gene or regulatory element. Therefore we focused on genotyping the variants within a 441 kb region originally mapped in Simmental and still ended up with a total of 34 variants associated with polledness in Holstein. The majority of these variants (Table S2) were detected and reported to be ruled out by Allais-Bonnet [3] by genotyping eight of them in recombinant animals. Nevertheless in our cohort we genotyped all of them and they were perfectly associated with polledness in more than 80 polled Holstein and not present in over 400 horned (mainly Holstein) cattle. At the moment it is impossible to conclusively prove, which variant is responsible for polledness in Holstein. However, we speculate that, due to its large structure and possible regulatory effect, an 80 kb duplication is most likely the causative variant. Nevertheless the causative variant in Holstein could theoretically also be one of the 181 further sequence variants. A recently published *IFNGR2* associated SNP [16] could be clearly ruled out as it was not perfectly associated with the polled phenotypes in our Holstein cohort (Table S3). In addition it maps outside of the critical region on BTA 1.

### Polled and scurs

Two types of horns are known in cattle: regular horns attached to the skull and so called scurs or “wiggle horns” in German, which refers to small loosely attached horns. For the first time we were able to compare the accurate polled genotype with recorded horn phenotype details. The fact that all 207 scurred animals are heterozygous for one of the *polled* mutations indicates epistasis of the *polled* and *scurs* loci. *Polled* is epistatic over *scurs* and in homozygous *P/P* animals *scurs* cannot be expressed. A total of 191 homozygous polled cattle showed no signs of scurs. Therefore, our data did not confirm previous models where homozygous polled cattle being homozygous for the *scurs* mutation were described to develop scurs [6]. Comparing the expression of scurs in heterozygous polled animals between male and female, it is conspicuous that especially in the Holstein breed more than two third of the male sampled *Pp* animals show scurs whereas in female almost all of the *Pp* animals are smooth polled. This tendency is apparent in the Simmental breed as well, but not as striking as in the Holstein breed (Table 1). This observation has to be taken with caution as the sampling was not representative for the whole population and there might be a bias, as we were specifically looking for scurred individuals. Although previous studies indicate an autosomal locus on BTA19 [23], an X-chromosomal recessive mode of inheritance would explain these observations. In different published inheritance models [5]–[7], female individuals are thought to develop scurs only if they are homozygous, but males also in the heterozygous state. It might be assumed that these males are in fact hemizygous. Nevertheless, there are still several inconsistencies with this inheritance model reported in the literature [6]. Many of those arise from the absence of scurs that would have been expected based on the pedigree. This might yet be explained by misclassified phenotypes or incomplete penetrance, especially in males. However, there is no clear evidence for X-linked inheritance. Alternatively, genetic heterogeneity, as reported or proposed for other cattle populations [22], [24], might also explain the observed mode of inheritance.

### Expression differences during development between polled and wildtype fetuses

The Simmental polled variant observed in almost all hornless beef and dual-purpose cattle is located in an intergenic region and therefore the question of the functional consequences remains open. In polled goats a genomic deletion affects the transcription of neighbouring genes including a previously unknown long noncoding RNA [19]. First histological changes of bovine horn buds take place at a neck-rump length of 5.3 cm, with approximately 70 days of gestation, as a thickening of the epidermis [29], indicating that key genes involved in horn development already have to be expressed in the first trimester of gestation. To study possible regulatory effects we collected samples of developing cattle fetuses of eight different developmental stages from 70 to 175 days with and without visible horn buds. Genotyping confirmed the presence of one or two copies of the *indel* mutation in the polled fetuses. In an initial RNA-Seq experiment we identified a large number of differentially expressed genes by comparing the expression patterns in horn bud tissue of a single polled and a single horned fetus, both at approximately 5 months of gestation. Subsequently we performed qRT-PCR based gene expression studies to evaluate differential expressed transcripts in wildtype and polled fetuses during development. We focused on transcripts of two genes (*FOXL2* and *RXFP2*) affected by polled causing mutations in small ruminants and the genes of the mapped *polled* region, that were found to be differentially expressed in the RNA-Seq experiment (*OLIG1*, *OLIG2, C1H21orf62*) plus a single uncharacterized locus (*LOC100848215*) of the polled associated region on BTA 1.

The genes *FOXL2* and *RXFP2* were clearly down-regulated in the polled horn bud compared to the wildtype horn bud in the RNA-Seq experiment. Quantitative RT-PCR confirmed these findings, as these two genes were higher expressed in wildtype horn buds compared to all the other tissues in different stages of the fetal development. Interestingly there was also a differential expression within the polled samples, where both of the genes were higher expressed in the polled horn bud than in the polled frontal skin. Allais-Bonnet et al. [3] showed the same tendency in 90 days old fetuses, where *RXFP2* was described to be up-regulated in wildtype horn buds compared to *Pp* horn buds and *FOXL2* was described to be up-regulated in horn buds of both genotypes compared to frontal skin. The fact that these genes are known to be involved in horn development in goat and sheep [19], [21] together with the expression patterns we found in wildtype and polled fetuses, suggests their involvement during horn bud formation in cattle.

The BTA 1 genes *OLIG1* and *OLIG2* encode transcription factors supposedly involved in oligodendrocyte differentiation and maturation [30]–[32]. We found them to be up-regulated in the wildtype horn bud in the RNA-Seq experiment. In further qRT-PCR expression studies in younger fetuses it was not possible to detect expression of *OLIG1*. For *OLIG2* we couldn't confirm the findings of the RNA-Seq experiment in younger fetuses. *OLIG2* was found to be higher expressed in younger stages generally, but there was no constant expression difference between genotypes or tissues across different stages. This stands in contrast to a recent report, where OLIG2 was described to be higher expressed in frontal skin than in horn buds from 90 days old fetuses [3]. The *C1H21orf62*, a protein coding gene of unknown function, was the only positional candidate shown to be up-regulated in the polled fetal tissue compared to the wildtype horn bud tissue in the RNA-Seq experiment. Quantitative RT-PCR didn't confirm this finding as no constant difference in expression pattern between polled and wildtype fetuses was observed across the studied developmental stages. This is in agreement with Allais-Bonnet et al. who also did not observe differential expression of *C1H21orf62* [3]. Nevertheless qRT-PCR indicates that *C1H21orf62* is generally higher expressed in frontal skin than in horn buds regardless of the *polled* genotype.

In a previous study Allais-Bonnet et al. [3] reported two long noncoding RNA (*LincRNA#1* and *LincRNA#2*) both located within the mapped polled region on BTA 1. The annotated locus *LOC100848368* corresponds perfectly to *LincRNA#1* whereas only 4.7 kb in the 3′ region of the 74 kb spanning *LincRNA#2* overlaps to the four annotated exons of *LOC100848215*. The *LincRNA#1* was described to be significantly overexpressed in horn bud of polled fetuses compared to frontal skin of the polled fetuses and compared to the horn bud and frontal skin of wildtype fetuses whereas the *LincRNA#2* was not reported to be differentially expressed using qRT-PCR [3]. In the fetal samples of our study *LincRNA#1* (or *LOC100848368*) was not detectable, neither by RNA-Seq nor by qRT-PCR using the published primers [3]. Interestingly, our RNA-Seq experiment revealed some spliced reads covering two exons of *LOC100848215* in the wildtype horn bud sample only. Quantitative RT-PCR confirmed the presence of *LOC100848215* expression in our samples, especially in both horn bud and frontal skin tissues of wildtype fetuses. In the samples of polled fetuses *LOC100848215* expression was usually lower and even not detectable at all in biopsies from three out of five homozygous polled (*PP*) fetuses. Allais-Bonnet *et al*. [3] designated the position of *LincRNA#2* based on several spliced ESTs, which span more than 74 kb. If primers of that study (whose sequences were not published) were designed within the 5′ region, the actual transcripts could possibly have been missed, as our RNA-Seq data shows reads in the distal segment of the last two annotated exons only (BTA 1: 1,898,100–1,899,400). Long noncoding RNAs have the potential to play important regulatory roles, as their sequence structure allows them to regulate transcription in an allele- and locus-specific manner [33]. A recent deep transcriptome sequencing study revealed a large number of long noncoding RNAs in bovine skin samples, revealing an unexplored reservoir of novel possibly functional RNAs [34]. So far there are no known functions of the *LOC100848215* and interestingly ESTs are found for cow and buffalo only (Figure S4). Therefore this RNA seems to be specific for horned ruminants. The physical proximity of *LOC100848215* to the sites of the different *polled* mutations (Figure 2) and its decreased or even absent expression in the presence of one or two copies of the *polled* allele together with its apparent ruminant specificity makes it a very interesting candidate. Therefore we speculate that this long noncoding RNA might play a significant role in horn growth. As the presence of the dominant *polled* allele inhibits horn bud formation probably by influencing the expression of genes required for horn development (*FOXL2*, *RXFP2*, *LOC100848215*) the functional effect could be due to haploinsufficiency of the involved genes. The performed RNA-Seq experiment indicates that numerous other transcripts are differently expressed between wildtype horned and polled fetuses. Therefore we plan to perform future more comprehensive RNA-Seq experiments comparing the expression pattern across different developmental stages and between different genotypes to specify these findings.

## Conclusion

We independently identified a complicated *indel* confirming the recently published Celtic *polled* mutation as the causative variant for polledness in Simmental and other beef and dual-purpose cattle. In addition, we confirmed the presence of an independent *polled* associated haplotype in Holstein cattle corresponding to the assumed Friesian *polled* allele. In addition to the four reported possible causative variants we provide a list of 182 sequence variants perfectly associated with the *polled* mutation in Holstein cattle. We found evidence for sporadic occurrence of de novo mutations leading to polledness. Comparing the genotypes of about 400 polled cattle with the recorded phenotype details we found that homozygous polled animals are always smooth polled. In contrast all animals showing scurs are heterozygous for one of the *polled* alleles. RNA-Seq of fetal horn bud tissue of a horned and a polled fetus revealed a large number of differentially expressed genes, including some of the previously known positional and functional candidates. Quantitative RT-PCR of skin and horn bud biopsies from different fetal stages implicates an important role of *RXFP2* and *FOXL2* in ruminant horn development and suggests a key role of the ruminant specific transcript *LOC100848215* for horn bud formation in cattle.

## Materials and Methods

### Ethics Statement

All animal work was conducted according to the national and international guidelines for animal welfare. The collection of fetal tissue was done at a local slaughterhouse, as a low number of pregnant cows are routinely slaughtered. Blood sampling was done with owner consent. The whole study was approved by the “Cantonal Committee for Animal Experiments” (Canton of Bern; permits BE78/12).

### Material

We collected blood, hair root, tissue or semen samples from 1,019 polled cattle belonging to 14 different breeds. For phenotyping we inspected and palpated the area on the cattle's forehead, where horns usually grow and recorded any scabs, corneous growths or horn-like formations. We observed the development of different forms of scurs in a total of 207 animals born as polled. The DNA of 1,501 horned cattle from 28 different breeds was taken from the archive of the Institute of Genetics.

DNA was either isolated from EDTA-blood using the Nucleon Bacc2 kit (GE Healthcare) or from sperm straw, hair roots or ear punch biopsies using QIAGEN's DNeasy kit according to the manufacturers' instruction.

A total of 23 fetuses (Table S4) were collected at a governmentally authorized slaughterhouse, inspecting the uteri of the slaughtered cows. Fetal horn buds were biopsied using a 4 mm or 6 mm biopsy punch and immediately stored in RNA-later (Ambion). After storage at 4°C for 48 hours they were kept at −20°C. Total RNA was isolated using Qiagen RNeasy Kit including a DNase treatment and subsequently stored at −80°C.

### SNP genotyping

High-density SNP genotyping was performed using the illumina BovineHD BeadChip with 777,962 SNP markers at GeneSeek [35].

### Whole genome re-sequencing

We prepared fragment libraries with 250 bp insert size and collected one to three lanes of illumina HiSeq2000 paired-end reads (2×100 bp) obtaining about 200 million tags per lane. We mapped the reads to the cow reference genome Bos_taurus_UMD_3.1 with the Burrows-Wheeler Aligner (BWA) version 0.5.9-r16 [36] with default settings. After sorting the mapped reads by the coordinates of the sequence with Picard tools, we labeled the PCR duplicates also with Picard tools [37]. We used the Genome Analysis Tool Kit (GATK version 0591, [38]) to perform local realignment and to produce a cleaned BAM file. Variants calls were then made with the unified genotyper module of GATK. Variant data for each sample were obtained in variant call format (version 4.0) as raw calls for all samples and sites flagged using the variant filtration module of GATK. Variant calls that failed to pass the following filters were labeled accordingly in the call set: (i) Hard to Validate MQ0≥4 & ((MQ0/(1.0 * DP)) >0.1); (ii) strand bias (low Quality scores) QUAL <30.0 || (Quality by depth) QD <5.0 || (homopolymer runs) HRun >5 || (strand bias) SB >0.00; (iii) SNP cluster window size 10. The snpEFF software [39] together with the recent ensembl cow genome annotation was used to predict the functional effects of detected variants. IGV-viewer software [40] was used for manual inspection of sequence variants.

### Genotyping of candidate variants

For genotyping of SNPs and small InDels we applied Sanger sequencing. Primers (Table S6) were designed with Primer3 software [41] after masking of repetitive sequences with RepeatMasker [42]. PCR products were amplified using AmpliTaqGold360Mastermix (LifeTechnologies) and directly sequenced on an ABI3730 capillary sequencer (LifeTechnologies) after treatment with exonuclease I and shrimp alkaline phosphatase. The sequence data were analyzed with Sequencher 5.1 software. For genotyping of the 80 kb duplication we set the forward primer at the end of the duplicated sequence and the reverse primer at the beginning of the duplicated sequence (Figure S2), therefore only in the mutant allele a PCR-product was amplified, which was detected on a 1% agarose gel. The polled associated *indel* detected in Simmental cattle was genotyped using fragment analysis, setting the forward primer at the end of the duplicated sequence and the fluorescently labeled reverse primer in the region afterwards (Figure S1). PCR products were amplified using QIAGEN Multiplex PCR Kit (Qiagen) and the fragment length of the PCR products was directly analyzed with an ABI 3730 capillary sequencer (LifeTechnologies) and the Genemapper-software (LifeTechnologies).

### RNA-Seq

We prepared two fragment libraries with 350 bp insert size following illumina's TruSeq Stranded mRNA Sample Preparation Guide and collected a quarter of a lane of illumina HiSeq2000 paired-end reads (2×100 bp) obtaining 70248709 (polled) and 85385646 (horned) tags per library. We mapped the reads to the cattle reference genome (Bostaurus_UMD3.1) using the spliced alignment program TopHat2 (version 2.0.4) with default parameters [43]. Read counting was carried out using HTSeq-count (version 0.5.3p9) and differential gene expression analysis was performed using DEseq software [44].

### Quantitative PCR of fetal tissue

cDNA was synthesized using the First Strand cDNA synthesis kit (GE Healthcare) and 1 μg total RNA. Primers were taken from the literature [3] or designed as described above spanning exon junctions if possible (Table S7). Quantitative RT-PCR was performed in triplicates using 10 μl Power Sybr Green reaction mix (LifeTechnologies), 6.4 μl H_2_O, 0.8 μl primer (10 pmol/μl) and 2 μl cDNA (∼16 ng). The reaction was performed on an ABI 7300 Real-Time PCR System (LifeTechnologies). Cycle threshold (Ct) values were normalized to three endogenous control genes (*GART*, *HPRT1* and *RPLP0*, Figure S7, Figure S8, Figure S9). Relative quantification was calculated using the 2^−ΔΔCt^ method [45].

## Supporting Information

Figure S1 **Characterization of the polled associated insertion-deletion (** ***indel*** **). Schematic representation of the duplication and deletion on BTA 1 (A).** Sequence details and primers used for genotyping are shown. Fragment length analysis showing three different genotypes (B).
(TIF)

Figure S2 **Characterization of the polled associated 80 kb duplication.** Region of the 80 kb duplication (taken from the UCSC genome browser), segments conserved in other species are shown as colored bars (**A**). Schematic illustration of the 80 kb duplication, both 2 bp deletions are indicated in red, primers used to determine the presence of the duplication are shown as black arrows (**B**).
(TIF)

Figure S3 **RNA-Seq data of horn bud tissue from a wildtype and polled fetus at** ***LOC100848215*** **.** Screenshot of the mapped reads displayed in the igv viewer BTA 1 UMD3.1: 1897536–1899936. Presence of spliced reads in the wildtype sample (above) in contrast absence of reads in the polled fetus (below).
(TIF)

Figure S4 **Cross-species comparison of** ***LOC100848215*** **associated EST's, showing expression of this sequence in ruminants only (buffalo).**
(TIF)

Figure S5 **Gene expression study of** ***C1H21orf62*** **based on RT-PCR.** Expression of *C1H21orf62* after normalization to *GART* (**A**), *HPRT1* (**B**) and *RPLP0* (**C**). Different fetal stages are divides in eight groups of estimated age (d). Wildtype fetuses are marked with the shape of a horned cow head, fetuses carrying the *polled* mutation are marked with the shape of a polled cow head, whereas each icon designates one fetus. For each individual a biopsy of the horn bud area (H) and a biopsy of the frontal skin (S) were studied. Expression levels in wildtype fetuses are shown in blue, those of heterozygous Pp polled fetuses in orange and those of homozygous PP polled fetuses in dark red. Expression levels are shown as relative expression in relation to the wildtype frontal skin of the youngest fetus.
(TIF)

Figure S6 **Gene expression study of** ***OLIG2*** **based on RT-PCR.** Expression of *OLIG2* after normalization to *GART* (**A**), *HPRT1* (**B**) and *RPLP0* (**C**). Different fetal stages are divides in eight groups of estimated age (d). Wildtype fetuses are marked with the shape of a horned cow head, fetuses carrying the *polled* mutation are marked with the shape of a polled cow head, whereas each icon designates one fetus. For each individual a biopsy of the horn bud area (H) and a biopsy of the frontal skin (S) were studied. Expression levels in wildtype fetuses are shown in blue, those of heterozygous Pp polled fetuses in orange and those of homozygous PP polled fetuses in dark red. Expression levels are shown as relative expression in relation to the wildtype frontal skin of the youngest fetus.
(TIF)

Figure S7 **Gene expression study of** ***FOXL2*** **based on RT-PCR.** Expression of *FOXL2* after normalization to *GART* (**A**), *HPRT1* (**B**) and *RPLP0* (**C**). Different fetal stages are divides in eight groups of estimated age (d). Wildtype fetuses are marked with the shape of a horned cow head, fetuses carrying the *polled* mutation are marked with the shape of a polled cow head, whereas each icon designates one fetus. For each individual a biopsy of the horn bud area (H) and a biopsy of the frontal skin (S) were studied. Expression levels in wildtype fetuses are shown in blue, those of heterozygous Pp polled fetuses in orange and those of homozygous PP polled fetuses in dark red. Expression levels are shown as relative expression in relation to the wildtype frontal skin of the youngest fetus.
(TIF)

Figure S8 **Gene expression study of** ***RXFP2*** **based on RT-PCR.** Expression of *RXFP2* after normalization to *GART* (**A**), *HPRT1* (**B**) and *RPLP0* (**C**). Different fetal stages are divides in eight groups of estimated age (d). Wildtype fetuses are marked with the shape of a horned cow head, fetuses carrying the *polled* mutation are marked with the shape of a polled cow head, whereas each icon designates one fetus. For each individual a biopsy of the horn bud area (H) and a biopsy of the frontal skin (S) were studied. Expression levels in wildtype fetuses are shown in blue, those of heterozygous Pp polled fetuses in orange and those of homozygous PP polled fetuses in dark red. Expression levels are shown as relative expression in relation to the wildtype frontal skin of the youngest fetus.
(TIF)

Figure S9 **Gene expression study of** ***LOC100848215*** **based on RT-PCR.** Expression of *LOC100848215* after normalization to *GART* (**A**), *HPRT1* (**B**) and *RPLP0* (**C**). Different fetal stages are divides in eight groups of estimated age (d). Wildtype fetuses are marked with the shape of a horned cow head, fetuses carrying the *polled* mutation are marked with the shape of a polled cow head, whereas each icon designates one fetus. For each individual a biopsy of the horn bud area (H) and a biopsy of the frontal skin (S) were studied. Expression levels in wildtype fetuses are shown in blue, those of heterozygous Pp polled fetuses in orange and those of homozygous PP polled fetuses in dark red. Expression levels are shown as relative expression in relation to the wildtype frontal skin of the youngest fetus.
(TIF)

Table S1 **Complex** ***indel*** **associated with polledness in Simmental cattle, genotyped in 2,329 animals.**
(DOCX)

Table S2 **Sequence variants of polled associated 932 kb haplotype in Holstein.** Animals carrying the polled associated *indel* found in Simmental are not included. The remaining associated variants are highlighted in grey and the excluded variants shown in red.
(PDF)

Table S3 **Genotypes of the** ***IFNGR2*** **SNP at BTA 1 UMD3.1: 1,390,292 (reference allele:** ***G*** **, variant allele:** ***A*** **).** Genotypes of 161 polled Holstein and 55 horned control cattle.
(DOCX)

Table S4 **Fetuses used for RNA-Seq and RT-PCR.** Age was estimated based on the relation between crown-rump length and time of gestation described by Schnorr and Kressin [26]. They were genotyped for the *indel* variant associated with polledness in beef and dual-purpose breeds and for the C>A SNP at BTA 1: 1′768′587 associated with polledness in the Holstein breed.
(PDF)

Table S5 **Differential gene expression in horn bud biopsies of a wildtype and a polled fetus.**
(PDF)

Table S6 **Primer used for genotyping of candidate variants.**
(PDF)

Table S7 **Primer used for qRT-PCR.**
(PDF)
