# Supplementary figures and images for: Independent Polled Mutations Leading to Complex Gene Expression Differences in Cattle
Source: PLoS One. 2014 Mar 26;9(3):e93435. doi: 10.1371/journal.pone.0093435 (PMC3966897; doi:10.1371/journal.pone.0093435)

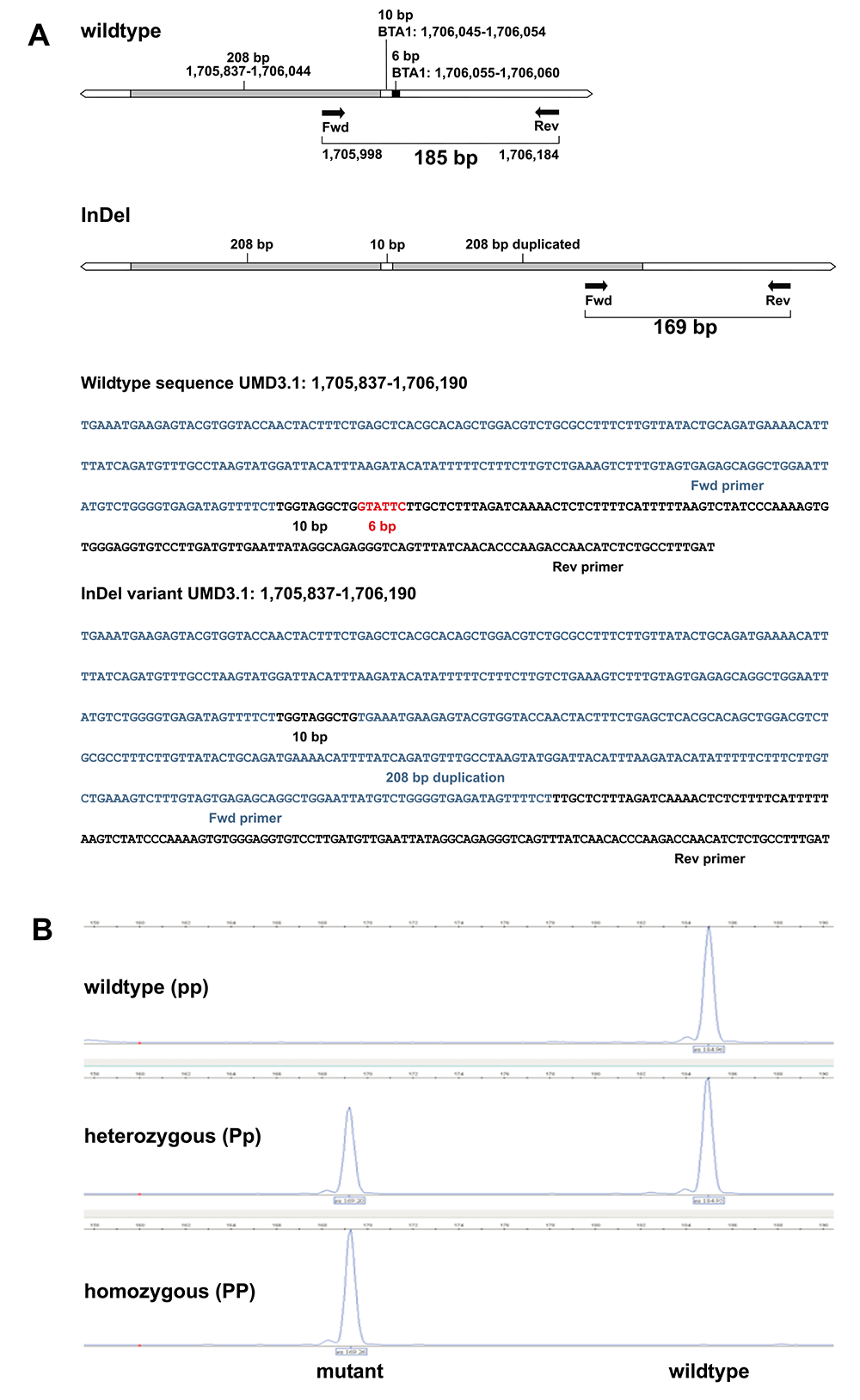

Supplement: Figure S1 — Characterization of the polled associated insertion-deletion ( indel ). Schematic representation of the duplication and deletion on BTA 1 (A). Sequence details and primers used for genotyping are shown. Fragment length analysis showing three different genotypes (B). (TIF) [file pone.0093435.s001.tif]

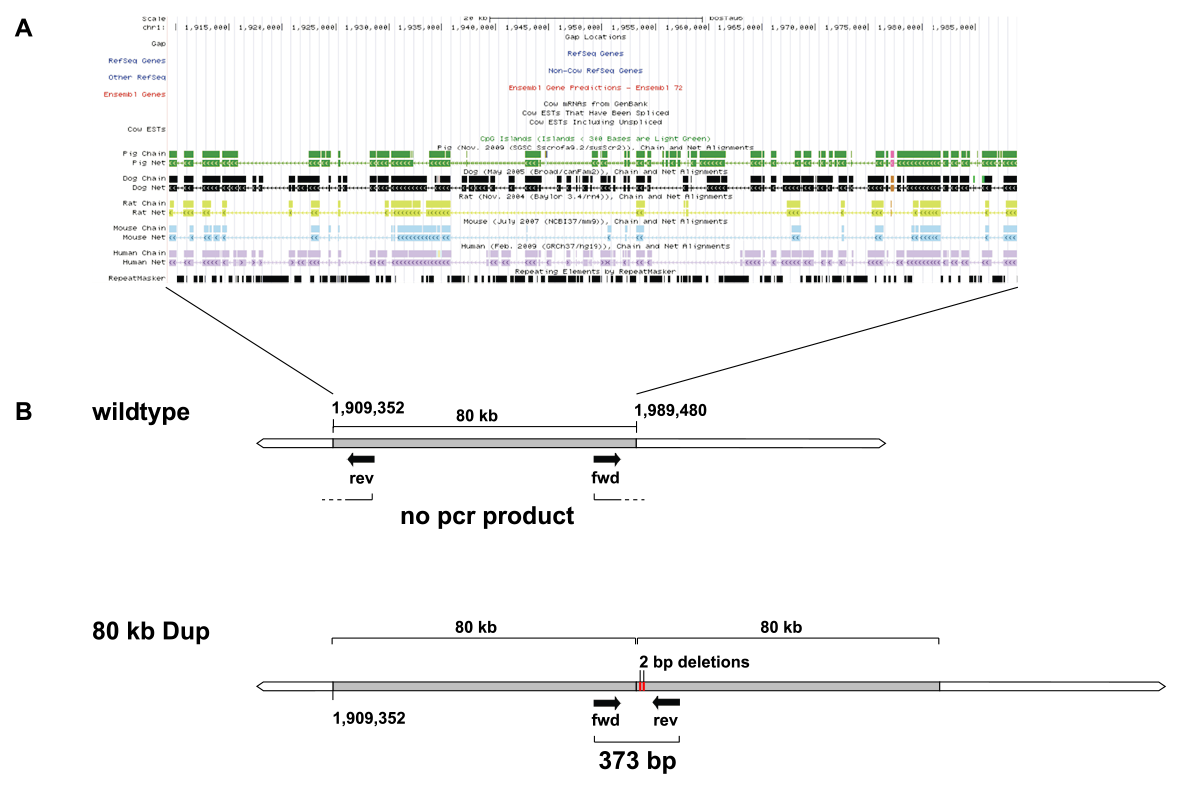

Supplement: Figure S2 — Characterization of the polled associated 80 kb duplication. Region of the 80 kb duplication (taken from the UCSC genome browser), segments conserved in other species are shown as colored bars (A). Schematic illustration of the 80 kb duplication, both 2 bp deletions are indicated in red, primers used to determine the presence of the duplication are shown as black arrows (B). (TIF) [file pone.0093435.s002.tif]

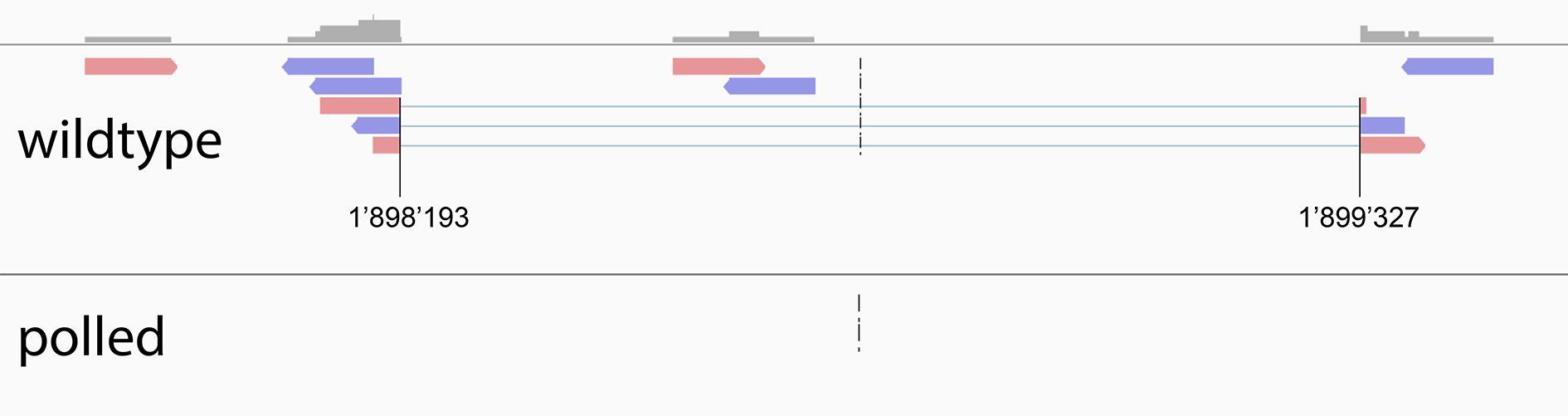

Supplement: Figure S3 — RNA-Seq data of horn bud tissue from a wildtype and polled fetus at LOC100848215 . Screenshot of the mapped reads displayed in the igv viewer BTA 1 UMD3.1: 1897536–1899936. Presence of spliced reads in the wildtype sample (above) in contrast absence of reads in the polled fetus (below). (TIF) [file pone.0093435.s003.tif]

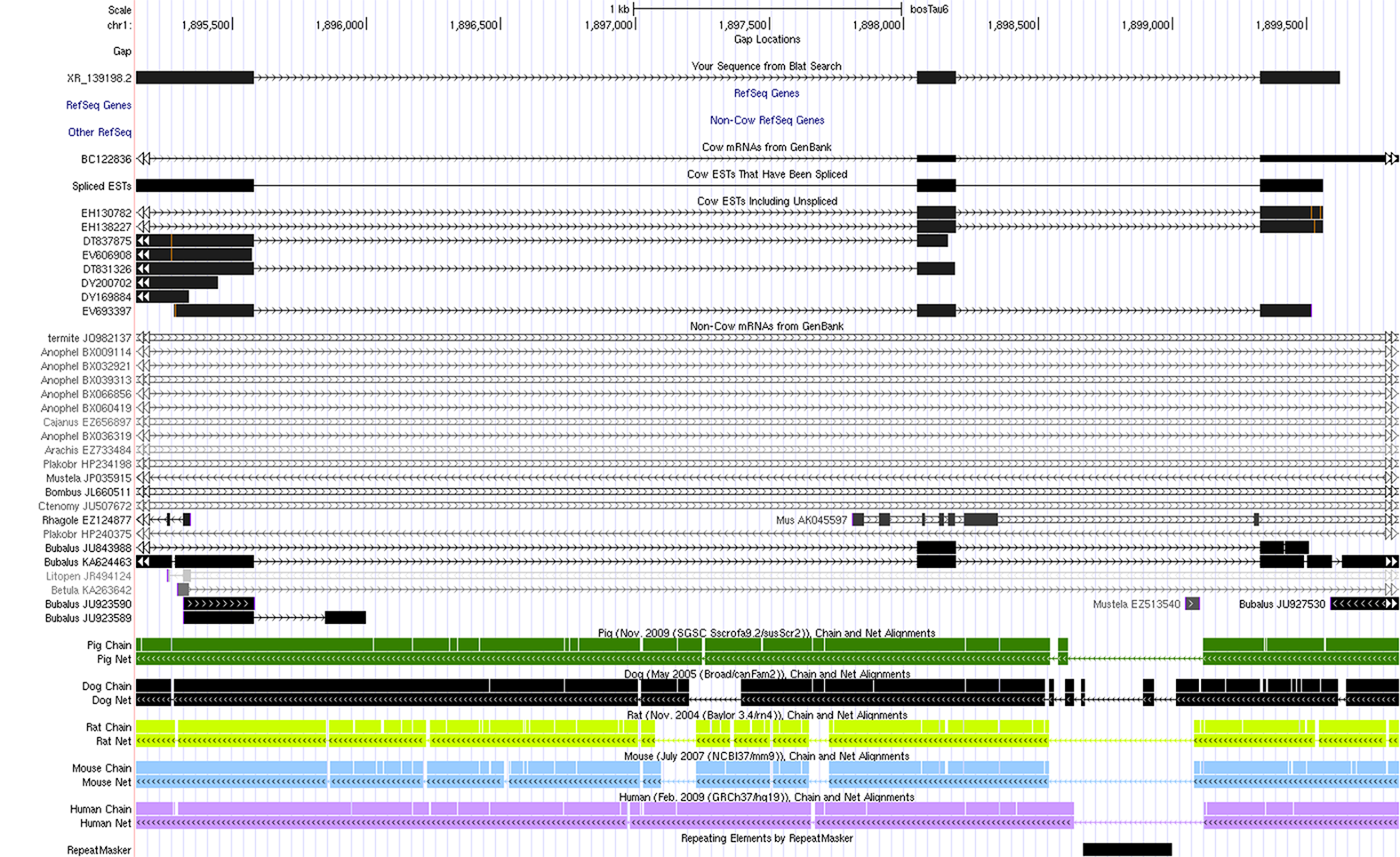

Supplement: Figure S4 — Cross-species comparison of LOC100848215 associated EST's, showing expression of this sequence in ruminants only (buffalo). (TIF) [file pone.0093435.s004.tif]

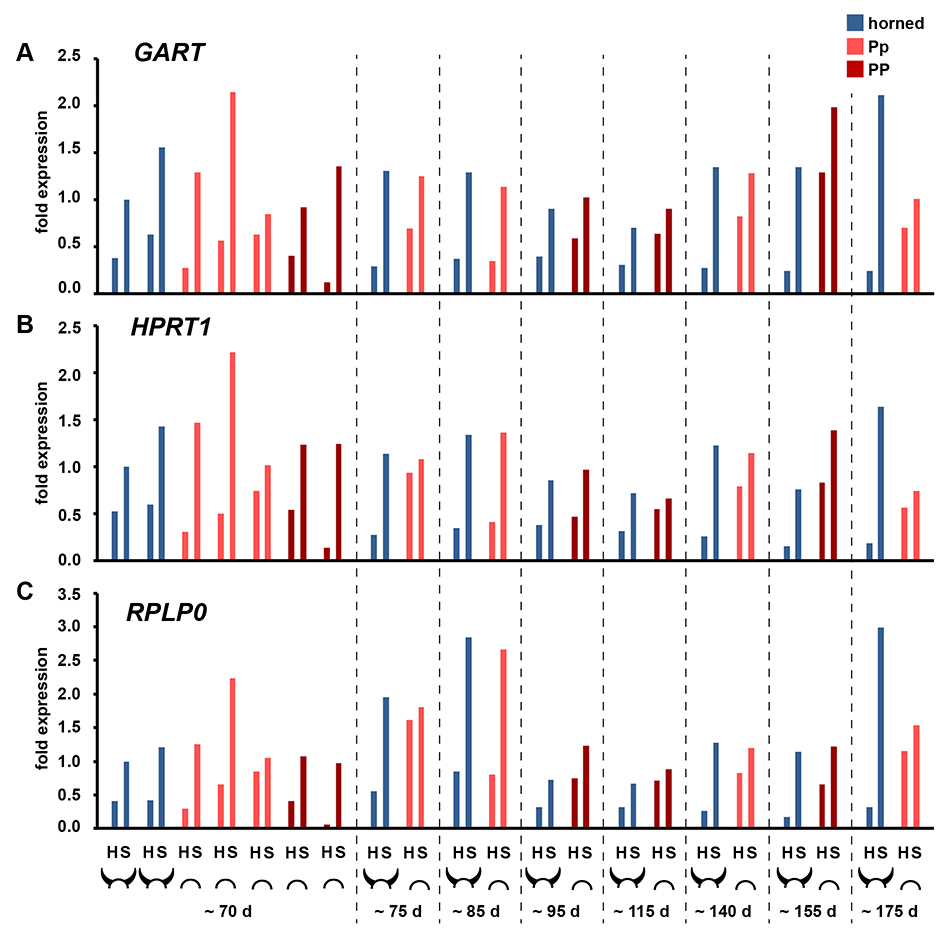

Supplement: Figure S5 — Gene expression study of C1H21orf62 based on RT-PCR. Expression of C1H21orf62 after normalization to GART (A), HPRT1 (B) and RPLP0 (C). Different fetal stages are divides in eight groups of estimated age (d). Wildtype fetuses are marked with the shape of a horned cow head, fetuses carrying the polled mutation are marked with the shape of a polled cow head, whereas each icon designates one fetus. For each individual a biopsy of the horn bud area (H) and a biopsy of the frontal skin (S) were studied. Expression levels in wildtype fetuses are shown in blue, those of heterozygous Pp polled fetuses in orange and those of homozygous PP polled fetuses in dark red. Expression levels are shown as relative expression in relation to the wildtype frontal skin of the youngest fetus. (TIF) [file pone.0093435.s005.tif]

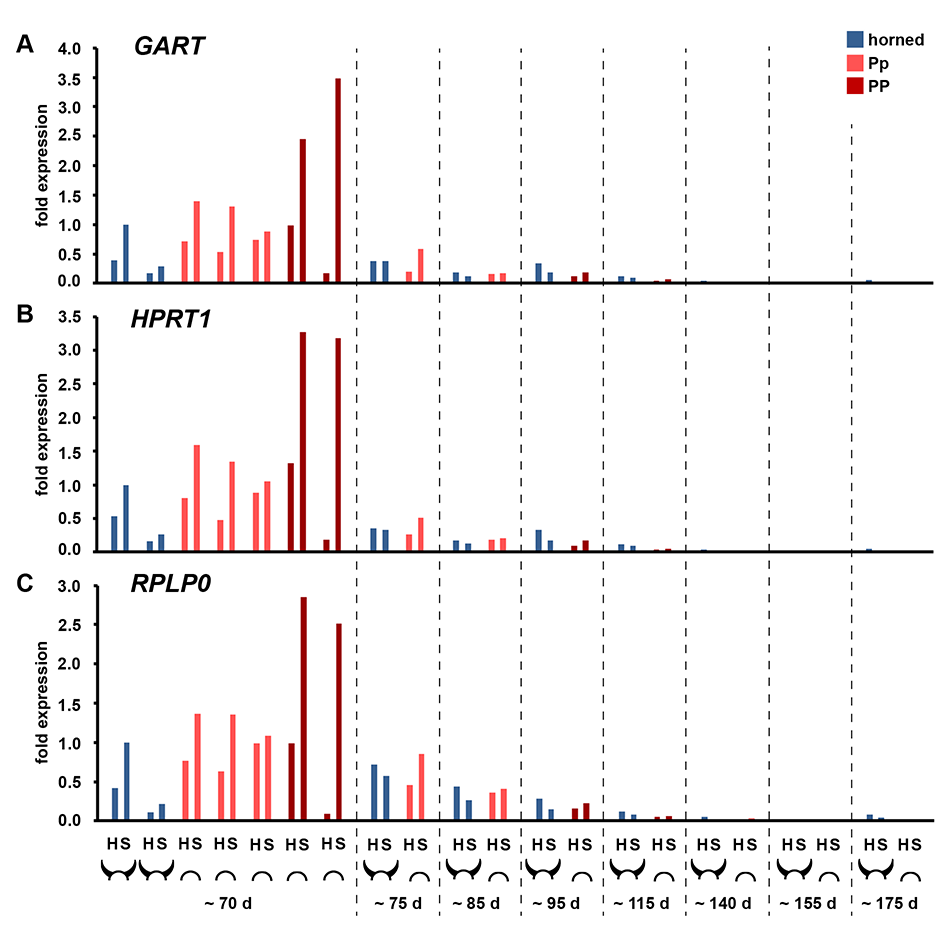

Supplement: Figure S6 — Gene expression study of OLIG2 based on RT-PCR. Expression of OLIG2 after normalization to GART (A), HPRT1 (B) and RPLP0 (C). Different fetal stages are divides in eight groups of estimated age (d). Wildtype fetuses are marked with the shape of a horned cow head, fetuses carrying the polled mutation are marked with the shape of a polled cow head, whereas each icon designates one fetus. For each individual a biopsy of the horn bud area (H) and a biopsy of the frontal skin (S) were studied. Expression levels in wildtype fetuses are shown in blue, those of heterozygous Pp polled fetuses in orange and those of homozygous PP polled fetuses in dark red. Expression levels are shown as relative expression in relation to the wildtype frontal skin of the youngest fetus. (TIF) [file pone.0093435.s006.tif]

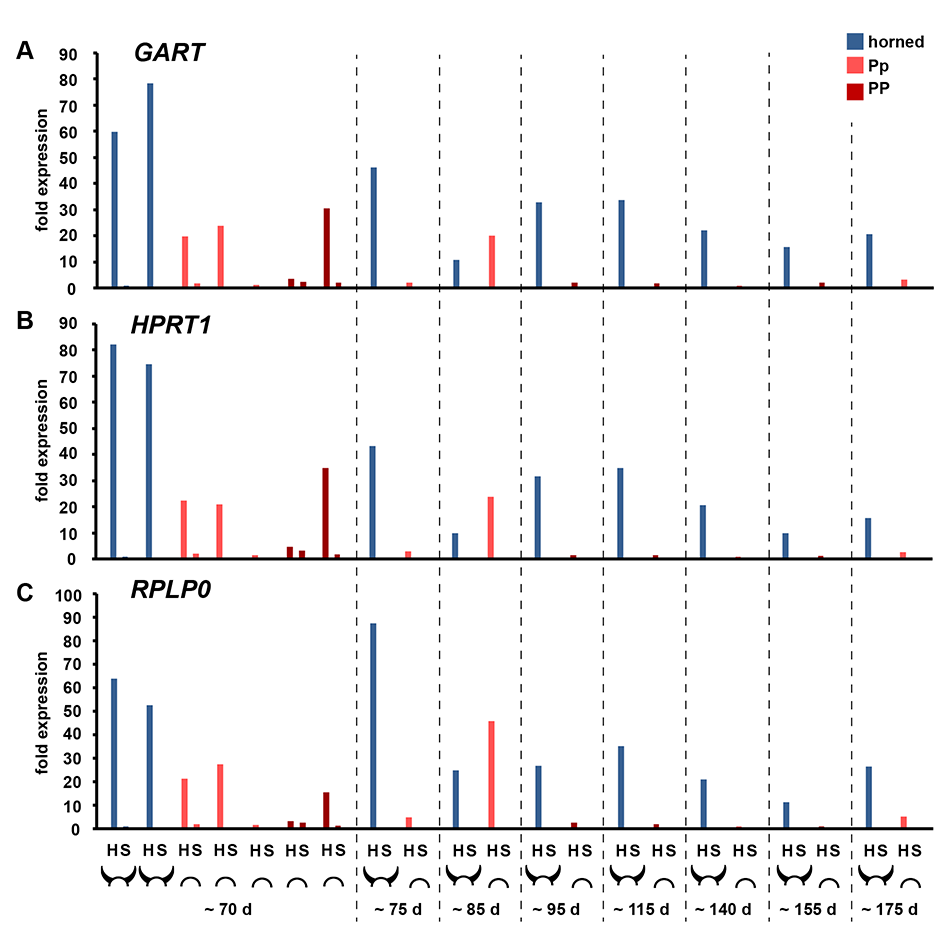

Supplement: Figure S7 — Gene expression study of FOXL2 based on RT-PCR. Expression of FOXL2 after normalization to GART (A), HPRT1 (B) and RPLP0 (C). Different fetal stages are divides in eight groups of estimated age (d). Wildtype fetuses are marked with the shape of a horned cow head, fetuses carrying the polled mutation are marked with the shape of a polled cow head, whereas each icon designates one fetus. For each individual a biopsy of the horn bud area (H) and a biopsy of the frontal skin (S) were studied. Expression levels in wildtype fetuses are shown in blue, those of heterozygous Pp polled fetuses in orange and those of homozygous PP polled fetuses in dark red. Expression levels are shown as relative expression in relation to the wildtype frontal skin of the youngest fetus. (TIF) [file pone.0093435.s007.tif]

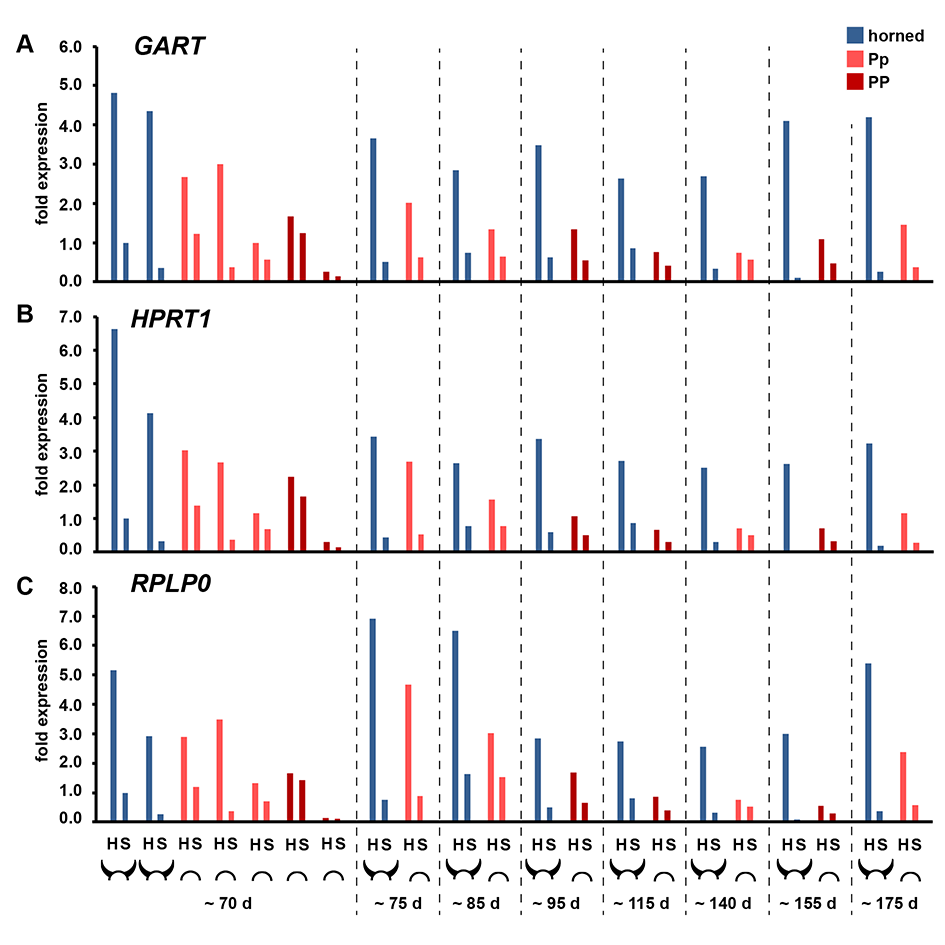

Supplement: Figure S8 — Gene expression study of RXFP2 based on RT-PCR. Expression of RXFP2 after normalization to GART (A), HPRT1 (B) and RPLP0 (C). Different fetal stages are divides in eight groups of estimated age (d). Wildtype fetuses are marked with the shape of a horned cow head, fetuses carrying the polled mutation are marked with the shape of a polled cow head, whereas each icon designates one fetus. For each individual a biopsy of the horn bud area (H) and a biopsy of the frontal skin (S) were studied. Expression levels in wildtype fetuses are shown in blue, those of heterozygous Pp polled fetuses in orange and those of homozygous PP polled fetuses in dark red. Expression levels are shown as relative expression in relation to the wildtype frontal skin of the youngest fetus. (TIF) [file pone.0093435.s008.tif]

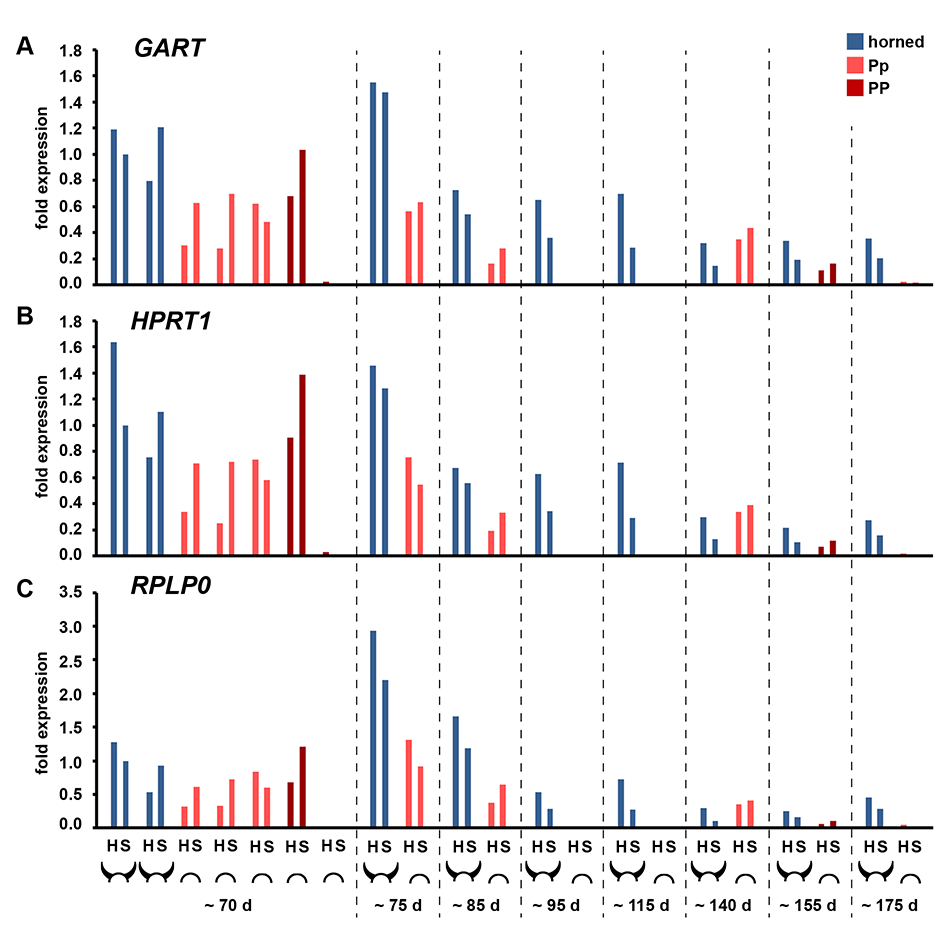

Supplement: Figure S9 — Gene expression study of LOC100848215 based on RT-PCR. Expression of LOC100848215 after normalization to GART (A), HPRT1 (B) and RPLP0 (C). Different fetal stages are divides in eight groups of estimated age (d). Wildtype fetuses are marked with the shape of a horned cow head, fetuses carrying the polled mutation are marked with the shape of a polled cow head, whereas each icon designates one fetus. For each individual a biopsy of the horn bud area (H) and a biopsy of the frontal skin (S) were studied. Expression levels in wildtype fetuses are shown in blue, those of heterozygous Pp polled fetuses in orange and those of homozygous PP polled fetuses in dark red. Expression levels are shown as relative expression in relation to the wildtype frontal skin of the youngest fetus. (TIF) [file pone.0093435.s009.tif]
