# Supplementary material for: Independent Polled Mutations Leading to Complex Gene Expression Differences in Cattle
Source: PLoS One. 2014 Mar 26;9(3):e93435. doi: 10.1371/journal.pone.0093435 (PMC3966897; doi:10.1371/journal.pone.0093435)
Supplement: Table S1 — Complex indel associated with polledness in Simmental cattle, genotyped in 2,329 animals. (DOCX) [file pone.0093435.s010.docx]

**Table S1:** Complex *indel* related with polledness in Simmental cattle, genotyped in 2,329 animals.

| **breed** | **ref/ref polled** | **ref/var polled** | **var/var**  **PP polled** | **total polled** |  | **ref/ref horned** |
| --- | --- | --- | --- | --- | --- | --- |
| Angler |  |  |  |  |  | **5** |
| Angus |  | 10 | 46 | **56** |  |  |
| Angus-crosses |  | 6 |  | **6** |  |  |
| Aubrac |  |  |  |  |  | **1** |
| Ayrshire |  |  |  |  |  | **1** |
| Belgium Blue |  |  |  |  |  | **3** |
| Blonde d'Aquitaine |  | 2 | 1 | **3** |  | **3** |
| Braunvieh |  | 7 | 1 | **8** |  | **204** |
| Charolais | 1* | 11 | 4 | **16** |  | **1** |
| Chianina |  |  |  |  |  | **2** |
| Dutch Belted |  |  |  |  |  | **1** |
| Eringer |  |  |  |  |  | **23** |
| Evolener |  |  |  |  |  | **7** |
| Galloway |  | 3 | 65 | **68** |  |  |
| Galloway-crosses |  | 2 |  | **2** |  |  |
| Gelbvieh |  |  |  |  |  | **1** |
| Grauvieh |  |  |  |  |  | **5** |
| Hereford |  | 4 | 4 | **8** |  | **1** |
| Highland Cattle |  |  |  |  |  | **8** |
| Holstein | 161* | 6 |  | **167** |  | **178** |
| Holstein-crosses | 2* |  |  | **2** |  | **2** |
| Jersey | 1* | 2 |  | **3** |  | **2** |
| Limousin | 13* | 186 | 47 | **246** |  | **75** |
| Limousin-crosses |  | 6 |  | **6** |  |  |
| Limpurger | 2** |  |  | **2** |  | **1** |
| Montbéliarde |  |  |  |  |  | **5** |
| Norwegian Red |  | 2 |  | **2** |  |  |
| Ongole |  |  |  |  |  | **2** |
| Pezzata Rossa |  |  |  |  |  | **1** |
| Pinzgauer |  | 13 | 1 | **14** |  | **6** |
| Pustertaler |  |  |  |  |  | **3** |
| Romagnola |  |  |  |  |  | **7** |
| Rotes Höhenvieh |  |  |  |  |  | **17** |
| Salers |  |  |  |  |  | **1** |
| Simmental | 1** | 281 | 121 | **403** |  | **647** |
| Simmental-crosses |  | 6 |  | **6** |  | **95** |
| Yak | 1** |  |  | **1** |  | **2** |
| **total** | **182** | **547** | **290** | **1019** |  | **1310** |
|  |  |  |  |  |  |  |
| * Animals carry the polled associated Holstein variants. | | | | | | |
| ** Three animals are probably hornless due to *de novo* mutations and the fourth is a polled Yak, in which polledness is presumably caused by another polled allele. | | | | | | |
