# Supplementary material for: Independent Polled Mutations Leading to Complex Gene Expression Differences in Cattle
Source: PLoS One. 2014 Mar 26;9(3):e93435. doi: 10.1371/journal.pone.0093435 (PMC3966897; doi:10.1371/journal.pone.0093435)
Supplement: Table S2 — Sequence variants of polled associated 932 kb haplotype in Holstein. Animals carrying the polled associated indel found in Simmental are not included. The remaining associated variants are highlighted in grey and the excluded variants shown in red. (PDF) [file pone.0093435.s011.pdf]

Table S2: Sequence variants of polled associated 932 kb haplotype in Holstein. Animals carrying the polled associated *indel* found in Simmental are not included. The remaining associated variants are highlighted in grey and the excluded variants shown in red.

UMD3.1 position	variant	reference allele	variant allele	ref/ref polled	ref/var polled	var/var polled	ref/ref horned	ref/var horned
1'583'414°	SNP	A	G		84	4	204	
1'584'650	SNP	C	T		1	2	55	1
1'587'758	SNP	C	T		76	4	200	1
1'596'777°	SNP	G	T		85	4	390	
1'603'747°	SNP	C	T		87	4	426	
1'606'620°	SNP	C	T		83	4	415	
1'607'675°	SNP	G	A			3*	41	
1'618'261°	SNP	T	C			3*	43	
1'623'157°	SNP	G	A		11	2	77	
1'623'573	SNP	G	C		77	4	368	
1'624'279°	SNP	C	T		82	2	417	
1'624'522°	SNP	T	G		85	2	411	
1'624'555°	SNP	T	C		81	1	408	
1'626'030°	SNP	T	C		84	4	425	
1'626'065°	SNP	G	C		84	4	421	
1'628'090°	SNP	A	G		60	4	384	
1'631'299°	SNP	A	G		86	4	424	
1'631'667°	SNP	C	T		87	4	429	
1'631'762°	SNP	G	A		87	4	429	
1'631'895°	SNP	T	C		87	4	427	
1'633'226°	SNP	T	A		77	4	420	
1'633'690°	SNP	C	T		79	4	422	
1'635'514°	SNP	G	C		76	4	414	
1'639'632°	SNP	A	G		82	3	396	
1'640'658°	SNP	G	T		86	4	432	
1'640'815°	SNP	T	C		85	3	420	
1642525° **	SNP	T	A			1	33	
1'645'083°	SNP	G	T		80	4	426	
1'645'296°	deletion	AT	A		76	4	422	
1645301°	SNP	T	A		75	4	423	
1'649'163 +	InDel	CGCATCA	TTCTCAGAATAG		85	3	415	
1'651'491°	SNP	C	T	6	12	3	203	
1'654'405 +	SNP	G	A		87	4	424	
1'655'463 +	SNP	C	T		86	4	429	
1'672'807	SNP	G	A		2	2	216	1
1'676'520	SNP	C	T		19	3	407	1
1'679'832	SNP	C	T		2	3	133	1
1'680'646	SNP	T	C		80	6	221	1
1'684'055°	SNP	G	C		85	4	416	
1'764'239°	SNP	T	C		85	4	409	
1'768'587 +	SNP	C	A	4***	152	25	443	
1'855'898°	SNP	G	A		87	4	408	
1'909'354+	80kbDup				105****		430	
2'028'953°	SNP	C	G			1	33	
2'248'958	SNP	G	A			1	33	
2'255'019	SNP	C	T			1	33	
2'261'830	SNP	T	C			1	33	
2'263'363	SNP	T	C			1	33	
2'267'616	deletion	CT	C			1	33	
2'268'463	SNP	G	A			1	33	
2'269'541	SNP	T	C			1	33	
2'270'224	SNP	C	T			1	33	
2'271'484	SNP	C	T			1	33	
2'274'411	SNP	C	T			1	33	
2'274'700	SNP	T	G			1	33	
2'275'244	SNP	G	A			1	33	
2'275'575	SNP	G	A			1	33	
2'275'606	SNP	C	G			1	33	
2'275'689	SNP	G	A			1	33	
2'278'133	SNP	A	C			1	33	
2'279'787	SNP	G	T			1	33	
2'279'861	SNP	A	G			1	33	

UMD3.1 position	variant	reference allele	variant allele	ref/ref polled	ref/var polled	var/var polled	ref/ref horned	ref/var horned
2'279'921	SNP	T	C			1	32	
2'279'996	SNP	G	A			1	32	
2'280'020	SNP	A	G			1	32	
2'280'142	SNP	C	T			1	32	
2'280'168	SNP	G	A			1	32	
2'280'378	SNP	T	C			1	32	
2'280'407	SNP	C	T			1	33	
2'280'428	InDel	CCT	CTCTAC			1	32	
2'280'522	SNP	T	C			1	33	
2'280'577	SNP	C	G			1	33	
2'280'612	SNP	C	T			1	33	
2'280'642	SNP	T	C			1	33	
2'280'673	SNP	C	A			1	33	
2'280'683	SNP	A	G			1	33	
2'280'702	SNP	C	T			1	33	
2'280'862	SNP	A	G			1	33	
2'280'879	SNP	A	G			1	33	
2'280'916	SNP	T	C			1	33	
2'280'926	SNP	G	C			1	33	
2'281'031	SNP	G	A			1	33	
2'281'058	SNP	C	G			1	33	
2'281'154	SNP	A	T			1	33	
2'281'262	SNP	T	C			1	33	
2'281'270	SNP	T	A			1	33	
2'281'440	SNP	A	T			1	33	
2'281'560	SNP	A	G			1	33	
2'281'590	SNP	T	C			1	33	
2'281'635	SNP	C	T			1	33	
2'282'082	SNP	A	G			1	33	
2'282'132	SNP	G	A			1	33	
2'282'187	SNP	T	C			1	33	
2'282'194	SNP	A	T			1	33	
2'282'315	SNP	A	G			1	33	
2'282'521	SNP	T	C			1	33	
2'282'761	SNP	A	G			1	33	
2'283'214	SNP	C	T			1	33	
2'283'224	SNP	G	A			1	33	
2'283'237	SNP	C	G			1	33	
2'283'539	SNP	G	A			1	33	
2'283'965	SNP	G	A			1	33	
2'283'992	SNP	C	A			1	33	
2'284'002	SNP	C	T			1	33	
2'284'720	SNP	C	G			1	33	
2'284'769	SNP	T	C			1	33	
2'285'147	insertion	G	GT			1	33	
2'285'345	SNP	G	A			1	33	
2'285'383	SNP	G	A			1	33	
2'286'301	SNP	T	G			1	33	
2'286'353	SNP	G	A			1	33	
2'286'400	SNP	T	G			1	33	
2'286'504	SNP	C	G			1	33	
2'286'523	SNP	C	A			1	33	
2'286'592	SNP	T	C			1	33	
2'286'604	SNP	G	A			1	33	
2'286'606	SNP	G	A			1	33	
2'286'988	SNP	A	G			1	33	
2'287'210	SNP	C	T			1	33	
2'287'370	SNP	A	G			1	33	
2'287'448	SNP	C	G			1	33	
2'287'529	insertion	A	AT			1	33	
2'287'596	insertion	C	CT			1	33	
2'287'746	SNP	T	C			1	33	
2'287'757	SNP	C	T			1	33	
2'287'765	SNP	T	C			1	33	
2'287'798	SNP	A	G			1	33	
2'287'996	SNP	G	C			1	33	
2'288'467	SNP	A	G			1	32	

UMD3.1 position	variant	reference allele	variant allele	ref/ref polled	ref/var polled	var/var polled	ref/ref horned	ref/var horned
2'288'990	SNP	A	G			1	32	
2'289'042	SNP	A	G			1	33	
2'289'346	SNP	G	A			1	33	
2'289'458	SNP	C	T			1	32	
2'289'716	SNP	C	T			1	33	
2'292'082	insertion	C	CACCT			1	33	
2'292'239	SNP	G	A			1	33	
2'292'304	SNP	G	A			1	33	
2'293'266	SNP	T	C			1	33	
2'293'846	SNP	G	A			1	33	
2'293'867	SNP	C	A			1	33	
2'293'870	SNP	T	C			1	33	
2'294'082	SNP	G	C			1	33	
2'294'184	SNP	C	T			1	33	
2'294'641	SNP	G	T			1	33	
2'294'647	SNP	T	C			1	33	
2'294'824	SNP	G	A			1	33	
2'294'855	SNP	C	T			1	33	
2'295'121	SNP	C	G			1	33	
2'295'487	SNP	G	A			1	33	
2'295'951	SNP	G	A			1	32	
2'297'070	SNP	C	T			1	33	
2'297'597	SNP	T	A			1	33	
2'298'042	SNP	A	G			1	33	
2'298'175	SNP	G	A			1	33	
2'298'226	SNP	C	A			1	33	
2'299'551	SNP	A	G			1	32	
2'299'669	SNP	C	G			1	33	
2'299'800	SNP	C	T			1	33	
2'299'901	SNP	A	G			1	33	
2'300'316	SNP	A	G			1	33	
2'300'391	deletion	TTAACTCACTGACAGTGAGTCGACAG	T			1	33	
2'300'451	SNP	C	T			1	33	
2'300'502	SNP	A	G			1	33	
2'300'761	SNP	G	A			1	33	
2'300'868	SNP	C	T			1	33	
2'300'871	SNP	G	A			1	33	
2'300'897	SNP	C	T			1	33	
2'300'924	SNP	G	A			1	33	
2'301'003	SNP	G	A			1	33	
2'301'036	SNP	T	C			1	33	
2'301'086	SNP	T	C			1	33	
2'301'104	SNP	T	A			1	33	
2'301'170	SNP	A	G			1	33	
2'301'257	SNP	T	C			1	33	
2'301'276	SNP	A	G			1	33	
2'301'300	SNP	A	G			1	33	
2'301'327	SNP	T	A			1	33	
2'301'358	SNP	G	A			1	33	
2'301'483	SNP	A	G			1	33	
2'301'520	deletion	AGAGAGAGC	A			1	33	
2'301'538	SNP	T	C			1	33	
2'301'708	SNP	G	A			1	32	
2'306'127	SNP	G	A			1	33	
2'306'341	SNP	G	A			1	31	
2'331'321	SNP	G	A			1	33	
2'335'134	insertion	A	AT			1	33	
2'336'578	SNP	T	G			1	33	
2'343'920	SNP	G	A			1	33	
2'354'603	SNP	T	A			1	33	
2'356'950	SNP	T	C			1	33	
2'381'155°	SNP	T	G			1	33	

* Variants were excluded because obligate heterozygous Pp polled animals carried two copies of the variant allele.
** Genotyping of additional animals for this variant was technially impossible.
*** One Simmental with polledness of unknown origin, two Limpurger with Scurs probably de novo mutation and one polled yak.
**** Differentiation between the heterozygous (ref/var) and homozygous (var/var) genotype not performed.
° Variants identified and ruled out by Allais-Bonnet et al. [3]
+ Variants identified by Allais-Bonnet et al. [3]
