# Supplementary material for: Independent Polled Mutations Leading to Complex Gene Expression Differences in Cattle
Source: PLoS One. 2014 Mar 26;9(3):e93435. doi: 10.1371/journal.pone.0093435 (PMC3966897; doi:10.1371/journal.pone.0093435)
Supplement: Table S3 — Genotypes of the IFNGR2 SNP at BTA 1 UMD3.1: 1,390,292 (reference allele: G , variant allele: A ). Genotypes of 161 polled Holstein and 55 horned control cattle. (DOCX) [file pone.0093435.s012.docx]

**Table S3:** Genotypes of the *IFNGR2* SNP at BTA 1 UMD3.1: 1,390,292 (reference allele: G, variant allele: A). Genotypes of 161 polled Holstein and 55 horned control cattle.

|  | **horned** | **polled *Pp*** | **polled *PP*** |
| --- | --- | --- | --- |
| ***GG*** | **54** | **4** |  |
| ***AG*** | **1** | **135** |  |
| ***AA*** |  |  | **22** |
| **Total** | **55** | **139** | **22** |
