# Supplementary material for: Independent Polled Mutations Leading to Complex Gene Expression Differences in Cattle
Source: PLoS One. 2014 Mar 26;9(3):e93435. doi: 10.1371/journal.pone.0093435 (PMC3966897; doi:10.1371/journal.pone.0093435)
Supplement: Table S4 — Fetuses used for RNA-Seq and RT-PCR. Age was estimated based on the relation between crown-rump length and time of gestation described by Schnorr and Kressin [26]. They were genotyped for the indel variant associated with polledness in beef and dual-purpose breeds and for the C>A SNP at BTA 1: 1′768′587 associated with polledness in the Holstein breed. (PDF) [file pone.0093435.s013.pdf]

Table S4: Fetuses used for RNA-Seq and RT-PCR. Age was estimated based on the relation between crown-rump length and time of gestation described by Schnorr and Kressin [26]. They were genotyped for the *indel* variant associated with polledness in beef and dual-purpose breeds and for the C>A SNP at BTA 1: 1'768'587 associated with polledness in the Holstein breed.

Sample ID	crown-rump length (cm)	sex	estimated age in days	*indel* genotype	genotype Holstein polledness
**Fetuses used for RNA-Seq**					
POL1067	32	f	150	wildtype	wildtype
POL1068	35	m	158	*PP*	wildtype
**Fetuses used for RT-PCR**					
~70 days					
POL1113	6.8	m	67	wildtype	wildtype
POL1114	6.8	m	67	*PP*	wildtype
POL1144	6.9	m	69	heterozygous	wildtype
POL1100	7.3	f	70	*PP*	wildtype
POL1151	7.7	m	70	wildtype	wildtype
POL1150	7.7	f	70	heterozygous	wildtype
POL1134	7.9	m	71	heterozygous	wildtype
~75 days					
POL1146	8.8	m	74	heterozygous	wildtype
POL1136	9.2	m	75	wildtype	wildtype
~85 days					
POL1099	11	m	83	heterozygous	wildtype
POL1152	11.3	f	83	wildtype	wildtype
~95 days					
POL1132	15.5	m	95	wildtype	wildtype
POL1108	15.5	f	96	*PP*	wildtype
~115 days					
POL1125	21.5	f	115	wildtype	wildtype
POL1149	21.8	f	115	*PP*	wildtype
~140 days					
POL1140	29.4	f	140	wildtype	wildtype
POL1147	30.3	m	143	heterozygous	wildtype
~155 days					
POL1106	34.5	f	155	wildtype	wildtype
POL1129	35	f	158	*PP*	wildtype
~175 days					
POL1104	42	f	172	wildtype	wildtype
POL1117	44	f	177	heterozygous	wildtype
