# Supplementary material for: Independent Polled Mutations Leading to Complex Gene Expression Differences in Cattle
Source: PLoS One. 2014 Mar 26;9(3):e93435. doi: 10.1371/journal.pone.0093435 (PMC3966897; doi:10.1371/journal.pone.0093435)
Supplement: Table S5 — Differential gene expression in horn bud biopsies of a wildtype and a polled fetus. (PDF) [file pone.0093435.s014.pdf]

Ensembl gene ID	geneName	counts wildtype horn bud	counts polled horn bud	baseMean	baseMean wildtype horn bud	baseMean polled horn bud	log2FoldChange	pval	padj
ENSBTAG00000000470	*IBSP*	20'115	5	8'713	17'420	6	-11.559	4.41E-21	8.60E-17
ENSBTAG00000015650	*TNN*	11'683	14	5'067	10'118	16	-9.290	7.86E-17	7.66E-13
ENSBTAG00000045544	*BT.89732*	0	1'378	796	0	1'591	Inf	4.14E-15	2.69E-11
ENSBTAG00000020099	*STMN2*	10'315	26	4'482	8'933	30	-8.217	9.67E-15	4.72E-11
ENSBTAG00000015059	*MMP13*	2'500	2	1'084	2'165	2	-9.873	3.22E-14	1.25E-10
ENSBTAG00000011451	*RTN1*	4'030	8	1'750	3'490	9	-8.562	5.05E-14	1.41E-10
ENSBTAG00000018703	*OSTN*	2'711	3	1'176	2'348	3	-9.405	4.58E-14	1.41E-10
ENSBTAG00000019394	*ANO5*	3'112	5	1'350	2'695	6	-8.867	7.44E-14	1.81E-10
ENSBTAG00000009433	*BGLAP*	12'284	62	5'355	10'638	72	-7.215	7.35E-13	1.59E-09
ENSBTAG00000011836	*OMD*	2'233	5	970	1'934	6	-8.388	1.91E-12	3.73E-09
ENSBTAG00000032997	*LOR*	1'633	2	708	1'414	2	-9.258	2.53E-12	4.49E-09
ENSBTAG00000048102	*protein_coding*	0	709	409	0	819	Inf	7.02E-12	1.14E-08
ENSBTAG00000020676	*MMP9*	1'839	5	799	1'593	6	-8.108	1.32E-11	1.97E-08
ENSBTAG00000026708	*PRSS35*	9'057	76	3'966	7'844	88	-6.482	3.50E-11	4.56E-08
ENSBTAG00000046123	*BT.78843*	0	616	356	0	711	Inf	3.43E-11	4.56E-08
ENSBTAG00000020809	*SLC36A2*	1'100	1	477	953	1	-9.688	4.13E-11	5.03E-08
ENSBTAG00000000564	*NSG2*	4'337	32	1'896	3'756	37	-6.667	5.76E-11	5.91E-08
ENSBTAG00000013662	*COL8A1*	15'615	151	6'849	13'523	174	-6.277	5.57E-11	5.91E-08
ENSBTAG00000045750	*DDX3Y*	0	589	340	0	680	Inf	5.67E-11	5.91E-08
ENSBTAG00000019132	*DMP1*	1'130	2	490	979	2	-8.727	1.18E-10	1.15E-07
ENSBTAG00000046725	*BT.90609*	52	3'327	1'943	45	3'842	6.415	1.42E-10	1.31E-07
ENSBTAG00000016158	*BT.92700*	21'424	266	9'430	18'554	307	-5.917	2.77E-10	2.45E-07
ENSBTAG00000011113	*pseudogene*	753	0	326	652	0		3.78E-10	3.21E-07
ENSBTAG00000046332	*ACTA1*	114	5'046	2'963	99	5'827	5.883	8.07E-10	6.56E-07
ENSBTAG00000015374	*COL22A1*	2'418	22	1'060	2'094	25	-6.365	1.09E-09	8.52E-07
ENSBTAG00000000033	*PHOSPHO1*	4'240	51	1'865	3'672	59	-5.962	1.28E-09	9.60E-07
ENSBTAG00000045494	*bta-mir-2904-2*	12'340	186	5'451	10'687	215	-5.637	1.51E-09	1.09E-06
ENSBTAG00000009639	*SMPD3*	3'074	35	1'351	2'662	40	-6.042	1.92E-09	1.34E-06
ENSBTAG00000009702	*BT.95556*	190	6'753	3'981	165	7'798	5.566	2.66E-09	1.79E-06
ENSBTAG00000021685	*EEF1A2*	5'814	89	2'569	5'035	103	-5.615	3.59E-09	2.33E-06
ENSBTAG00000017627	*STMN4*	1'043	5	455	903	6	-7.290	3.80E-09	2.39E-06
ENSBTAG00000021218	*MYLPF*	149	5'184	3'058	129	5'986	5.536	3.99E-09	2.43E-06
ENSBTAG00000013921	*CKM*	44	1'950	1'145	38	2'252	5.885	4.98E-09	2.94E-06
ENSBTAG00000000558	*NEUROD6*	547	0	237	474	0		1.09E-08	6.22E-06
ENSBTAG00000021013	*TUBB4A*	2'352	33	1'037	2'037	38	-5.740	1.36E-08	7.36E-06
ENSBTAG00000048000	*bta-mir-2904-3*	4'104	71	1'818	3'554	82	-5.438	1.36E-08	7.36E-06
ENSBTAG00000014078	*IGFBPL1*	767	3	334	664	3	-7.583	1.72E-08	9.06E-06
ENSBTAG00000048172	*BT.98214*	0	347	200	0	401	Inf	1.82E-08	9.35E-06
ENSBTAG00000014977	*BT.87394*	2'649	42	1'171	2'294	48	-5.564	2.00E-08	9.99E-06
ENSBTAG00000009707	*MYL1*	84	2'533	1'499	73	2'925	5.329	2.90E-08	1.38E-05
ENSBTAG00000021588	*SCG2*	1'165	11	511	1'009	13	-6.312	2.84E-08	1.38E-05
ENSBTAG00000039260	*RPTN*	1'247	13	547	1'080	15	-6.169	3.17E-08	1.47E-05
ENSBTAG00000047852	*bta-mir-2904-3*	3'738	73	1'661	3'237	84	-5.263	3.43E-08	1.56E-05
ENSBTAG00000007258	*GAD1*	634	2	276	549	2	-7.893	4.38E-08	1.94E-05
ENSBTAG00000038974	*STMN3*	1'565	23	691	1'355	27	-5.673	6.53E-08	2.83E-05
ENSBTAG00000014969	*CILP2*	1'770	29	783	1'533	33	-5.517	7.59E-08	3.22E-05
ENSBTAG00000017312	*SST*	434	0	188	376	0		1.10E-07	4.57E-05
ENSBTAG00000004231	*GPM6A*	2'220	44	987	1'923	51	-5.242	1.14E-07	4.62E-05
ENSBTAG00000024604	*RPL21*	1'447	23	640	1'253	27	-5.560	1.29E-07	5.14E-05
ENSBTAG00000005714	*ACTC1*	219	4'498	2'692	190	5'194	4.775	1.58E-07	6.15E-05
ENSBTAG00000011782	*CHGB*	415	0	180	359	0		1.70E-07	6.51E-05
ENSBTAG00000015132	*RXFP2*	4'513	125	2'026	3'908	144	-4.759	2.37E-07	8.89E-05
ENSBTAG00000046537	*bta-mir-2904-3*	3'949	107	1'772	3'420	124	-4.791	2.46E-07	9.06E-05
ENSBTAG00000003392	*GABRA5*	386	0	167	334	0		3.40E-07	0.000122649
ENSBTAG00000010741	*KBTBD10*	152	2'966	1'778	132	3'425	4.701	3.51E-07	0.000124354
ENSBTAG00000002066	*MYL7*	3	368	214	3	425	7.354	3.76E-07	0.000130783
ENSBTAG00000045532	*bta-mir-2904-3*	3'496	100	1'572	3'028	115	-4.713	4.12E-07	0.000140888
ENSBTAG00000019131	*TNRC4*	817	10	360	708	12	-5.937	4.92E-07	0.000165529
ENSBTAG00000009387	*BT.57732*	34	955	566	29	1'103	5.227	5.59E-07	0.000184684
ENSBTAG00000020869	*DDC*	575	4	251	498	5	-6.752	5.92E-07	0.000192471
ENSBTAG00000018432	*SLC6A15*	700	8	308	606	9	-6.036	8.61E-07	0.000275386
ENSBTAG00000002030	*SPP2*	783	11	345	678	13	-5.738	1.04E-06	0.000320895
ENSBTAG00000002661	*ISLR2*	964	17	427	835	20	-5.410	1.03E-06	0.000320895
ENSBTAG00000002898	*UNC45B*	3	329	191	3	380	7.192	1.09E-06	0.000330742
ENSBTAG00000011392	*MYBPC1*	185	2'965	1'792	160	3'424	4.417	1.21E-06	0.000363134
ENSBTAG00000021046	*ELAVL4*	529	4	231	458	5	-6.632	1.27E-06	0.000374218
ENSBTAG00000045657	*bta-mir-2904-3*	3'828	138	1'737	3'315	159	-4.379	1.52E-06	0.000442916
ENSBTAG00000000071	*PMP2*	470	3	205	407	3	-6.877	1.81E-06	5.18E-04
ENSBTAG00000000419	*PHEX*	372	1	162	322	1	-8.124	2.22E-06	0.000627012
ENSBTAG00000033224	*pseudogene*	64	1'196	718	55	1'381	4.639	2.45E-06	0.00068252
ENSBTAG00000009180	*NRSN1*	450	3	197	390	3	-6.814	2.66E-06	0.000730594
ENSBTAG00000011170	*JAKMIP1*	303	0	131	262	0		3.07E-06	0.000818813

Ensembl gene ID	geneName	counts wildtype horn bud	counts polled horn bud	baseMean	baseMean wildtype horn bud	baseMean polled horn bud	log2FoldChange	pval	padj
ENSBTAG00000015979	*BT.46235*	1'066	26	477	923	30	-4.943	3.06E-06	0.000818813
ENSBTAG00000021458	*DLX6*	401	2	175	347	2	-7.232	3.23E-06	0.000851532
ENSBTAG00000043991	*SLIT1*	1'328	15'401	9'467	1'150	17'784	3.951	3.88E-06	0.001009606
ENSBTAG00000032775	*RPL23A*	466	5'689	3'486	404	6'569	4.025	3.98E-06	0.00102063
ENSBTAG00000008495	*AP3B2*	1'265	38	570	1'096	44	-4.642	4.88E-06	0.001235899
ENSBTAG00000002123	*MYO3A*	1'172	34	527	1'015	39	-4.692	5.15E-06	0.001287596
ENSBTAG00000003045	*BAMBI*	4'794	219	2'202	4'152	253	-4.037	5.24E-06	0.001293795
ENSBTAG00000027080	*SLC8A3*	965	25	432	836	29	-4.855	5.71E-06	0.001392603
ENSBTAG00000045632	*bta-mir-2904-1*	2'487	102	1'136	2'154	118	-4.193	6.00E-06	0.001445571
ENSBTAG00000015335	*BAI3*	652	12	289	565	14	-5.349	6.85E-06	0.001628803
ENSBTAG00000011869	*CSRP3*	18	502	298	16	580	5.217	7.15E-06	0.001658981
ENSBTAG00000038347	*SLC17A6*	366	2	160	317	2	-7.101	7.13E-06	0.001658981
ENSBTAG00000008920	*ATP1B4*	19	514	305	16	594	5.173	7.29E-06	0.001671676
ENSBTAG00000016805	*SGMS2*	2'908	129	1'334	2'518	149	-4.080	7.56E-06	0.001714582
ENSBTAG00000011803	*BT.91205*	478	5'286	3'259	414	6'104	3.882	7.81E-06	0.001750999
ENSBTAG00000009409	*BT.104601*	427	4	187	370	5	-6.323	8.23E-06	0.001823736
ENSBTAG00000025246	*ZIC2*	318	1	138	275	1	-7.898	8.65E-06	0.00189482
ENSBTAG00000002717	*INA*	477	6	210	413	7	-5.898	9.53E-06	0.002065321
ENSBTAG00000021336	*BT.54994*	707	16	315	612	18	-5.051	1.06E-05	0.00226477
ENSBTAG00000010947	*PHYHIPL*	465	6	205	403	7	-5.861	1.18E-05	0.002496035
ENSBTAG00000004292	*CRMP1*	3'318	164	1'531	2'873	189	-3.924	1.22E-05	0.002557184
ENSBTAG00000018989	*GRM3*	638	14	284	553	16	-5.095	1.45E-05	0.002998397
ENSBTAG00000033292	*SP7*	5'915	330	2'752	5'123	381	-3.749	1.55E-05	0.00318745
ENSBTAG00000018576	*DPYSL5*	3'375	175	1'562	2'923	202	-3.854	1.59E-05	0.003229794
ENSBTAG00000037547	*OLIG1*	248	0	107	215	0		1.62E-05	0.003231629
ENSBTAG00000016886	*MT3*	248	0	107	215	0		1.64E-05	0.003295408
ENSBTAG00000007955	*SEZ6L2*	540	10	240	468	12	-5.340	1.68E-05	0.003311489
ENSBTAG00000047381	*bta-mir-2904-3*	1'977	91	909	1'712	105	-4.026	1.68E-05	0.003311489
ENSBTAG00000008023	*RHBDL2*	756	21	339	655	24	-4.755	1.85E-05	0.003613949
ENSBTAG00000003668	*CXORF57*	41	690	416	36	797	4.488	1.91E-05	0.003696393
ENSBTAG00000021302	*CHAD*	6'726	394	3'140	5'825	455	-3.678	1.95E-05	0.003727075
ENSBTAG00000004775	*BT.51704*	1'089	40	495	943	46	-4.352	1.98E-05	0.003743076
ENSBTAG00000006541	*BT.62768*	72	982	598	62	1'134	4.185	2.05E-05	0.003839772
ENSBTAG00000037768	*BT.18504*	238	0	103	206	0		2.27E-05	0.004222619
ENSBTAG00000002574	*MYOZ2*	41	663	401	36	766	4.430	2.59E-05	0.004773433
ENSBTAG00000027134	*DYNC1I1*	685	19	308	593	22	-4.757	2.66E-05	0.004804081
ENSBTAG00000032782	*INSC*	1'575	73	724	1'364	84	-4.016	2.65E-05	0.004804081
ENSBTAG00000038962	*BT.25073*	233	0	101	202	0		2.69E-05	0.004804091
ENSBTAG00000018645	*DLX5*	1'187	49	542	1'028	57	-4.183	2.76E-05	0.004900575
ENSBTAG00000005353	*DES*	379	3'535	2'205	328	4'082	3.636	2.90E-05	0.005102515
ENSBTAG00000017780	*DPYSL4*	969	37	441	839	43	-4.296	3.29E-05	0.005719931
ENSBTAG00000035392	*pseudogene*	254	2'403	1'497	220	2'775	3.657	3.70E-05	0.006381049
ENSBTAG00000000703	*ST6GAL2*	559	14	250	484	16	-4.904	4.06E-05	0.006952483
ENSBTAG00000006811	*BT.30722*	1'705	89	790	1'477	103	-3.845	4.26E-05	0.007226279
ENSBTAG00000008763	*CXHXorf64*	8	286	169	7	330	5.575	4.49E-05	0.007484664
ENSBTAG00000012918	*BT.49282*	5'265	345	2'479	4'560	398	-3.517	4.47E-05	0.007484664
ENSBTAG00000032702	*processed_pseudogene*	175	1'690	1'051	152	1'951	3.687	4.92E-05	0.008131887
ENSBTAG00000045979	*pseudogene*	252	2'260	1'414	218	2'610	3.580	5.35E-05	0.008771857
ENSBTAG00000014340	*KERA*	3'796	248	1'787	3'287	286	-3.521	5.55E-05	0.009019313
ENSBTAG00000033268	*BT.105445*	1'501	79	696	1'300	91	-3.833	5.60E-05	0.009021314
ENSBTAG00000047042	*bta-mir-2904-3*	1'841	104	857	1'594	120	-3.731	5.71E-05	0.009120442
ENSBTAG00000007090	*BT.91663*	53	695	424	46	803	4.128	5.87E-05	0.009307711
ENSBTAG00000010880	*TNNI2*	124	1'253	777	107	1'447	3.752	5.99E-05	0.009425332
ENSBTAG00000037526	*FABP4*	828	33	378	717	38	-4.234	6.27E-05	0.009776889
ENSBTAG00000015204	*SMPX*	12	311	185	10	359	5.111	7.76E-05	0.011920197
ENSBTAG00000004630	*BT.3034*	271	2	119	235	2	-6.667	7.93E-05	0.012071056
ENSBTAG00000009012	*BT.85209*	3'403	233	1'608	2'947	269	-3.453	7.99E-05	0.012071056
ENSBTAG00000009013	*pseudogene*	735	5'557	3'527	637	6'417	3.334	8.10E-05	0.012150972
ENSBTAG00000002317	*PTN*	39'757	3'159	19'039	34'431	3'648	-3.239	8.61E-05	0.012821142
ENSBTAG00000001755	*BT.104022*	199	0	86	172	0		8.78E-05	0.012969643
ENSBTAG00000014832	*TMEFF2*	500	14	225	433	16	-4.743	9.37E-05	0.013741702
ENSBTAG00000021526	*RPRM*	903	42	415	782	48	-4.011	9.66E-05	0.014061786
ENSBTAG00000008074	*BT.33105*	17'437	1'409	8'364	15'101	1'627	-3.214	0.000105583	0.015200736
ENSBTAG00000008700	*CRCT1*	231	1	101	200	1	-7.437	0.000106789	0.015200736
ENSBTAG00000014412	*ELAVL3*	286	3	126	248	3	-6.160	0.000106344	0.015200736
ENSBTAG00000014885	*MYOM3*	42	553	337	36	639	4.134	0.000109318	0.015447927
ENSBTAG00000026004	*CNTNAP4*	404	9	180	350	10	-5.073	0.000112836	0.01583039
ENSBTAG00000006451	*GAP43*	2'021	134	952	1'750	155	-3.500	0.00011717	0.016320971
ENSBTAG00000021721	*BT.93339*	433	11	194	375	13	-4.884	0.00012357	0.017090366
ENSBTAG00000010738	*CCL14*	1'578	100	741	1'367	115	-3.565	0.000132499	0.018196169
ENSBTAG00000001032	*BT.98113*	80	809	502	69	934	3.753	0.000136482	0.018612103
ENSBTAG00000012370	*MGP*	26'282	2'254	12'682	22'761	2'603	-3.128	0.000143813	0.0194757

Ensembl gene ID	geneName	counts wildtype horn bud	counts polled horn bud	baseMean	baseMean wildtype horn bud	baseMean polled horn bud	log2FoldChange	pval	padj
ENSBTAG00000021189	*ARHGAP36*	18	339	204	16	391	4.650	0.000150787	0.020003443
ENSBTAG00000021190	*BT.71620*	4'605	365	2'205	3'988	421	-3.242	0.000149193	0.020003443
ENSBTAG00000033897	*pseudogene*	655	4'531	2'900	567	5'232	3.205	0.000150307	0.020003443
ENSBTAG00000011274	*ENO2*	1'649	109	777	1'428	126	-3.504	0.000153497	0.020126195
ENSBTAG00000038128	*BOLA-DQA5*	526	18	238	456	21	-4.454	0.000153777	0.020126195
ENSBTAG00000004119	*NEUROD2*	183	0	79	158	0		0.000158685	0.020630082
ENSBTAG00000014912	*FMOD*	54'219	4'789	26'242	46'955	5'530	-3.086	0.000162892	0.020759748
ENSBTAG00000018137	*A2M*	15'358	1'325	7'415	13'300	1'530	-3.120	0.00016119	0.020759748
ENSBTAG00000021503	*pseudogene*	182	1'459	921	158	1'685	3.418	0.000163171	0.020759748
ENSBTAG00000027525	*pseudogene*	64	678	419	55	783	3.820	0.00016394	0.020759748
ENSBTAG00000018237	*MYO16*	182	1'444	913	158	1'667	3.403	0.000174588	0.021965397
ENSBTAG00000004561	*PAX6*	180	0	78	156	0		0.000177782	0.022223914
ENSBTAG00000009696	*BT.49327*	86	810	505	74	935	3.651	0.000190157	0.023433045
ENSBTAG00000012137	*NTN1*	638	4'261	2'736	553	4'920	3.155	0.000189922	0.023433045
ENSBTAG00000021916	*BT.22263*	87	816	509	75	942	3.645	0.00019106	0.023433045
MISSING	*OLIG2*	322	6	143	279	7	-5.330916878	0.000197066	0.023724606
ENSBTAG00000039657	*HIST1H2AA*	451	14	203	391	16	-4.595	0.000196429	0.023941
ENSBTAG00000019037	*AQP4*	1'681	118	796	1'456	136	-3.417	0.000202927	0.02457943
ENSBTAG00000002522	*SYT5*	1'080	65	505	935	75	-3.639	0.000205208	0.024702277
ENSBTAG00000016063	*DNER*	852	46	395	738	53	-3.796	0.000214576	0.025671475
ENSBTAG00000048210	*protein_coding*	20'028	1'827	9'727	17'345	2'110	-3.039	0.00021654	0.025748491
ENSBTAG00000020048	*MAPK10*	413	12	186	358	14	-4.690	0.000225317	0.026629691
ENSBTAG00000011500	*CASQ2*	77	724	451	67	836	3.648	0.000241973	0.028425981
ENSBTAG00000013534	*GFAP*	529	21	241	458	24	-4.240	0.000253807	0.029594534
ENSBTAG00000034077	*ASIP*	79	731	456	68	844	3.625	0.000254955	0.029594534
ENSBTAG00000003018	*FSTL3*	1'564	9'453	6'135	1'354	10'915	3.011	0.000265593	0.030466668
ENSBTAG00000046348	*ELAVL2*	203	1	88	176	1	-7.250	0.00026535	0.030466668
ENSBTAG00000008323	*SNAP25*	2'135	168	1'021	1'849	194	-3.253	0.000271694	0.0309842
ENSBTAG00000023037	*pseudogene*	851	5'230	3'388	737	6'039	3.035	0.000283317	0.032121835
ENSBTAG00000022699	*CAV3*	15	284	170	13	328	4.658	0.000295547	0.033314768
ENSBTAG00000018635	*ATP1A3*	1'437	106	683	1'244	122	-3.346	0.000327091	0.036658643
ENSBTAG00000026505	*protein_coding*	49	515	319	42	595	3.809	0.000336638	0.037513048
ENSBTAG00000007344	*FRMD7*	415	14	188	359	16	-4.475	0.000348285	0.038347949
ENSBTAG00000007782	*BT.33253*	14	269	161	12	311	4.679	0.00035003	0.038347949
ENSBTAG00000012411	*HAPLN1*	1'280	92	607	1'109	106	-3.383	0.000346131	0.038347949
ENSBTAG00000021692	*TBR1*	161	0	70	139	0		0.00037269	0.040602368
ENSBTAG00000011279	*MATN4*	6'880	38'004	24'921	5'958	43'883	2.881	0.000390442	0.042300088
ENSBTAG00000004680	*SLC13A5*	458	18	209	397	21	-4.254	0.000398192	0.042711792
ENSBTAG00000022886	*RYR2*	352	10	158	305	12	-4.722	0.000398623	0.042711792
ENSBTAG00000024545	*DCHS2*	752	44	351	651	51	-3.680	0.000407166	0.043388747
ENSBTAG00000047286	*pseudogene*	1'470	8'279	5'416	1'273	9'560	2.909	0.000413565	0.043831098
ENSBTAG00000023429	*PLS1*	1'963	165	945	1'700	191	-3.157	0.000420005	0.044273104
ENSBTAG00000004770	*BT.44059*	3	157	92	3	181	6.125	0.000430677	0.045153889
ENSBTAG00000014538	*HPCAL4*	635	34	295	550	39	-3.808	0.000441258	0.046015855
ENSBTAG00000002471	*CHL1*	927	62	437	803	72	-3.487	0.000450376	0.046716948
ENSBTAG00000000836	*BT.87815*	194	1'307	839	168	1'509	3.167	0.00045877	0.047038591
ENSBTAG00000001862	*PPP2R2B*	1'203	90	573	1'042	104	-3.326	0.000463125	0.047038591
ENSBTAG00000021127	*GNA14*	519	24	239	449	28	-4.020	0.000460805	0.047038591
ENSBTAG00000048169	*protein_coding*	12'753	1'304	6'275	11'044	1'506	-2.875	0.000458101	0.047038591
ENSBTAG00000017411	*AK5*	788	49	370	682	57	-3.592	0.0004688	0.047368216
ENSBTAG00000008571	*CUX2*	1'714	143	825	1'484	165	-3.168	0.000473591	0.047605616
ENSBTAG00000002294	*SIM1*	228	3	100	197	3	-5.833	0.000514749	0.051214886
ENSBTAG00000016847	*ARL4D*	1'076	79	512	932	91	-3.353	0.000513958	0.051214886
ENSBTAG00000012164	*CP*	1'825	158	881	1'580	182	-3.115	0.000529631	0.05242812
ENSBTAG00000000265	*SYNPR*	182	1	79	158	1	-7.093	0.000545186	0.053695348
ENSBTAG00000009998	*GALNTL1*	3'970	20'837	13'749	3'438	24'060	2.807	0.000548755	0.053775205
ENSBTAG00000047132	*protein_coding*	72	605	380	62	699	3.486	0.000580148	0.056567332
ENSBTAG00000018204	*MYH1*	9	209	125	8	241	4.952	0.000588683	0.057113954
ENSBTAG00000022890	*MBP*	1'166	93	559	1'010	107	-3.233	0.000655375	0.063269624
ENSBTAG00000032875	*BEX2*	219	3	97	190	3	-5.775	0.000667724	0.064144263
ENSBTAG00000010688	*BT.58115*	2'236	212	1'091	1'936	245	-2.984	0.000683891	0.0653753
ENSBTAG00000006273	*SULT4A1*	336	11	152	291	13	-4.518	0.000699955	0.066437843
ENSBTAG00000044035	*GPRIN1*	497	25	230	430	29	-3.898	0.00070182	0.066437843
ENSBTAG00000019327	*BT.43742*	76	608	384	66	702	3.415	0.00070618	0.066527576
ENSBTAG00000007506	*CACNG7*	251	5	112	217	6	-5.235	0.000710873	0.06656877
ENSBTAG00000008295	*ACTL6B*	335	11	151	290	13	-4.514	0.000713444	0.06656877
ENSBTAG00000003560	*RPL8*	4'543	22'655	15'047	3'934	26'160	2.733	0.000734034	0.067840732
ENSBTAG00000010103	*BT.91750*	417	18	191	361	21	-4.119	0.000733345	0.067840732
ENSBTAG00000006080	*MAST1*	594	35	277	514	40	-3.670	0.000754545	0.06908157
ENSBTAG00000040167	*protein_coding*	1'460	7'437	4'926	1'264	8'588	2.764	0.000753204	0.06908157
ENSBTAG00000022590	*BT.102557*	3'539	365	1'743	3'065	421	-2.862	0.000758295	0.069100545
ENSBTAG00000000601	*COL11A2*	3'679	382	1'814	3'186	441	-2.853	0.000768804	0.069732283

Ensembl gene ID	geneName	counts wildtype horn bud	counts polled horn bud	baseMean	baseMean wildtype horn bud	baseMean polled horn bud	log2FoldChange	pval	padj
ENSBTAG00000000414	*FUT5*	143	0	62	124	0		0.000777864	0.070227452
ENSBTAG00000026986	*TTN*	351	1'973	1'291	304	2'278	2.906	0.000784816	0.070528571
ENSBTAG00000011283	*RBPJL*	19	272	165	16	314	4.255	0.000797409	0.071331515
ENSBTAG00000031785	*H2B*	528	29	245	457	33	-3.771	0.000801417	0.07136269
ENSBTAG00000033803	*FABP7*	8'203	915	4'080	7'104	1'057	-2.749	0.000830674	0.073631724
ENSBTAG00000019628	*EDAR*	190	1'162	753	165	1'342	3.028	0.000851292	0.07511784
ENSBTAG00000018245	*SLC1A3*	3'165	331	1'562	2'741	382	-2.842	0.000874268	0.076797736
ENSBTAG00000000905	*RAB6B*	755	54	358	654	62	-3.390	0.000913038	0.079843754
ENSBTAG00000007383	*BT.37663*	5'630	627	2'800	4'876	724	-2.752	0.000925438	0.080566785
ENSBTAG00000005414	*protein_coding*	1'569	7'668	5'107	1'359	8'854	2.704	0.000945001	0.081904247
ENSBTAG00000005800	*GRIA1*	378	16	173	327	18	-4.147	0.000953331	0.082260648
ENSBTAG00000021261	*processed_pseudogene*	262	1'480	968	227	1'709	2.913	0.000975206	0.083777506
ENSBTAG00000006030	*MYOG*	9	193	115	8	223	4.838	0.000982054	0.083995771
ENSBTAG00000016514	*CPE*	14'145	1'664	7'086	12'250	1'921	-2.673	0.001005352	0.08524072
ENSBTAG00000037470	*TMSB4Y*	709	3'573	2'370	614	4'126	2.748	0.001000989	0.08524072
ENSBTAG00000033225	*CNTN4*	987	82	475	855	95	-3.174	0.001033093	0.087213596
ENSBTAG00000000310	*MFAP5*	1'869	8'928	5'964	1'619	10'309	2.671	0.001044032	0.087757228
ENSBTAG00000030678	*pseudogene*	657	3'303	2'191	569	3'814	2.745	0.001048897	0.087787746
ENSBTAG00000006738	*GPR68*	663	46	314	574	53	-3.434	0.001073827	0.089490171
ENSBTAG00000033835	*MPZ*	10'940	1'297	5'486	9'474	1'498	-2.661	0.001095995	0.09094895
ENSBTAG00000015217	*DLX1*	369	16	169	320	18	-4.112	0.001106676	0.091446171
ENSBTAG00000008544	*SALL1*	134	0	58	116	0		0.00113905	0.093724139
ENSBTAG00000007104	*JPH4*	592	39	279	513	45	-3.509	0.001148787	0.094128152
ENSBTAG00000031277	*FOXL2*	161	1	70	139	1	-6.916	0.001159536	0.094611364
ENSBTAG00000011530	*CDH15*	20	262	160	17	303	4.127	0.001172197	0.09524587
ENSBTAG00000005243	*BT.53990*	244	6	109	211	7	-4.931	0.001231013	0.098808961
ENSBTAG00000012870	*BT.29026*	473	27	220	410	31	-3.716	0.001237158	0.098808961
ENSBTAG00000013400	*VIP*	132	0	57	114	0		0.001241382	0.098808961
ENSBTAG00000020302	*FEZF2*	132	0	57	114	0		0.001241382	0.098808961
ENSBTAG00000047000	*protein_coding*	151	912	592	131	1'053	3.010	0.001227535	0.098808961
ENSBTAG00000027625	*BT.85254*	10'553	1'288	5'313	9'139	1'487	-2.619	0.001296681	0.102790951
ENSBTAG00000006990	*C11ORF9*	101	669	430	87	772	3.143	0.00131116	0.103166615
ENSBTAG00000024957	*SNCA*	737	57	352	638	66	-3.278	0.001312	0.103166615
ENSBTAG00000025213	*OMG*	178	2	78	154	2	-6.061	0.001331153	0.104252305
ENSBTAG00000021120	*BT.48781*	6	158	94	5	182	5.134	0.001426992	0.111311114
ENSBTAG00000001865	*SGCG*	1'072	99	521	928	114	-3.022	0.001440836	0.11194318
ENSBTAG00000005321	*BT.103652*	382	19	176	331	22	-3.914	0.001484628	0.114887829
ENSBTAG00000006256	*PTPRO*	438	25	204	379	29	-3.716	0.00152795	0.11777292
ENSBTAG00000005106	*BT.35117*	474	29	222	410	33	-3.616	0.001549168	0.11893829
ENSBTAG00000020985	*TYRP1*	396	21	184	343	24	-3.822	0.001617152	0.123670884
ENSBTAG00000000329	*BT.68694*	1'206	119	591	1'044	137	-2.926	0.001641508	0.125043176
ENSBTAG00000018800	*RPS4*	4'380	18'947	12'836	3'793	21'878	2.528	0.001663732	0.126242945
ENSBTAG00000002908	*GNG4*	647	50	309	560	58	-3.279	0.001706883	0.128517116
ENSBTAG00000038064	*protein_coding*	26	280	173	23	323	3.844	0.001704718	0.128517116
ENSBTAG00000003827	*RIMS2*	243	7	109	210	8	-4.702	0.001740884	0.130573016
ENSBTAG00000003684	*LRFN5*	499	33	235	432	38	-3.503	0.001765316	0.131007177
ENSBTAG00000005681	*ME1*	774	66	373	670	76	-3.137	0.00176302	0.131007177
ENSBTAG00000020223	*CASQ1*	60	448	285	52	517	3.316	0.001766827	0.131007177
ENSBTAG00000002319	*HMCN2*	716	3'261	2'193	620	3'765	2.602	0.001782207	0.131647064
ENSBTAG00000001094	*BT.51603*	81	544	349	70	628	3.163	0.001794247	0.132036292
ENSBTAG00000046394	*RPL23A*	2'011	8'684	5'884	1'742	10'027	2.525	0.001831495	0.13427065
ENSBTAG00000031669	*CTNNA2*	228	6	102	197	7	-4.833	0.001842432	0.134566568
ENSBTAG00000000238	*PSD2*	240	7	108	208	8	-4.684	0.001871967	0.136213522
ENSBTAG00000019416	*KCTD4*	184	3	81	159	3	-5.524	0.001916954	0.13896844
ENSBTAG00000001703	*SYT13*	713	60	343	617	69	-3.156	0.00194933	0.140792144
ENSBTAG00000006688	*RPS6KL1*	490	33	231	424	38	-3.477	0.001963248	0.141274157
ENSBTAG00000048312	*BT.103142*	931	88	454	806	102	-2.988	0.001970604	0.141282197
ENSBTAG00000040028	*MGC166429*	45	366	231	39	423	3.439	0.002063135	0.147374316
ENSBTAG00000033109	*pseudogene*	308	1'489	993	267	1'719	2.688	0.002087879	0.148597516
ENSBTAG00000017861	*BRSK2*	247	8	112	214	9	-4.533	0.002107621	0.149457161
ENSBTAG00000002593	*GDAP1L1*	819	75	398	709	87	-3.034	0.002127259	0.150303168
ENSBTAG00000000460	*BT.43006*	4'216	541	2'138	3'651	625	-2.547	0.002202385	0.154491796
ENSBTAG00000027213	*protein_coding*	4'995	20'515	14'007	4'326	23'689	2.453	0.002201217	0.154491796
ENSBTAG00000006031	*GPR98*	288	12	132	249	14	-4.170	0.002234776	0.156202039
ENSBTAG00000025220	*FAM155A*	143	1	62	124	1	-6.745	0.002279803	0.158780131
ENSBTAG00000015366	*SFRP4*	41'134	5'706	21'106	35'623	6'589	-2.435	0.002297587	0.159449261
ENSBTAG00000019021	*TRPC3*	178	3	79	154	3	-5.476	0.002313082	0.159955332
ENSBTAG00000006253	*FLNC*	6'037	24'456	16'734	5'228	28'239	2.433	0.002348228	0.161812027
ENSBTAG00000009338	*LRRC7*	177	3	78	153	3	-5.468	0.002387134	0.163913699
ENSBTAG00000007568	*MEPE*	117	0	51	101	0		0.002403265	0.16444236
ENSBTAG00000045727	*pseudogene*	1'086	4'560	3'103	941	5'265	2.485	0.002438551	0.166273393
ENSBTAG00000040210	*pseudogene*	193	980	649	167	1'132	2.759	0.002470679	0.167877057

Ensembl gene ID	geneName	counts wildtype horn bud	counts polled horn bud	baseMean	baseMean wildtype horn bud	baseMean polled horn bud	log2FoldChange	pval	padj
ENSBTAG00000014949	*SP9*	189	4	84	164	5	-5.147	0.002580142	0.174706091
ENSBTAG00000038783	*protein_coding*	1'660	6'750	4'616	1'438	7'794	2.439	0.002641088	0.178214028
ENSBTAG00000000575	*TNC*	53'540	7'657	27'604	46'367	8'842	-2.391	0.002686805	0.180673724
ENSBTAG00000001764	*NCAN*	5'122	696	2'620	4'436	804	-2.465	0.002760239	0.184973959
ENSBTAG00000015854	*TCHHL1*	156	2	69	135	2	-5.870	0.002812359	0.18782128
ENSBTAG00000021426	*BT.39045*	113	0	49	98	0		0.002879792	0.191668351
ENSBTAG00000020598	*PPP2R2C*	155	2	68	134	2	-5.861	0.002911951	0.193017782
ENSBTAG00000039839	*BT.56045*	1'132	125	562	980	144	-2.764	0.002919863	0.193017782
ENSBTAG00000000032	*ABI3*	678	62	329	587	72	-3.036	2.94E-03	1.94E-01
ENSBTAG00000013213	*OSR2*	1'888	7'485	5'139	1'635	8'643	2.402	0.002965774	0.194732544
ENSBTAG00000003540	*BT.53392*	136	1	59	118	1	-6.672	0.00298801	0.194880235
ENSBTAG00000012311	*CACNG5*	68	443	285	59	512	3.119	0.002984554	0.194880235
ENSBTAG00000045943	*CHRNG*	16	201	123	14	232	4.066	0.00305968	0.198889382
ENSBTAG00000001326	*SLC44A5*	153	2	67	133	2	-5.842	0.003122501	0.202298622
ENSBTAG00000044073	*CD248*	5'906	22'624	15'619	5'115	26'124	2.353	0.003189627	0.205963315
ENSBTAG00000005250	*BGN*	50'769	7'508	26'318	43'967	8'669	-2.342	0.003225392	0.207585364
ENSBTAG00000024420	*COL28A1*	259	1'184	796	224	1'367	2.608	0.003299381	0.211648789
ENSBTAG00000001599	*SV2A*	1'126	129	562	975	149	-2.711	0.003443309	0.220010765
ENSBTAG00000015356	*TAC1*	109	0	47	94	0		0.003457926	0.220010765
ENSBTAG00000019253	*RPL36A*	758	3'049	2'089	656	3'521	2.423	0.003463582	0.220010765
ENSBTAG00000001801	*SYT4*	5'042	718	2'598	4'367	829	-2.397	0.003527683	0.222632182
ENSBTAG00000015177	*PRSS23*	1'621	203	819	1'404	234	-2.582	0.003523767	0.222632182
ENSBTAG00000039530	*NEFM*	282	14	130	244	16	-3.917	0.003676842	0.231297108
ENSBTAG00000035643	*NAP1L1*	203	949	636	176	1'096	2.640	0.003693987	0.231628439
ENSBTAG00000032812	*protein_coding*	207	963	646	179	1'112	2.633	0.003717075	0.232329085
ENSBTAG00000000835	*BT.29499*	829	88	410	718	102	-2.821	0.003771069	0.234682144
ENSBTAG00000039967	*KRT78*	4'540	651	2'342	3'932	752	-2.387	0.00377879	0.234682144
ENSBTAG00000006733	*PPP1R3A*	6	133	79	5	154	4.885	0.003806292	0.234893994
ENSBTAG00000011390	*CHRND*	6	133	79	5	154	4.885	0.003806292	0.234893994
ENSBTAG00000034973	*protein_coding*	1'478	5'612	3'880	1'280	6'480	2.340	0.00391482	0.240829358
ENSBTAG00000018375	*ATCAY*	174	4	78	151	5	-5.028	0.004035594	0.24677783
ENSBTAG00000024773	*BT.75472*	198	6	89	171	7	-4.629	0.004036825	0.24677783
ENSBTAG00000018125	*KIF5C*	755	79	373	654	91	-2.842	0.004105532	0.2501937
ENSBTAG00000004824	*REEP1*	1'316	164	665	1'140	189	-2.589	0.004185497	0.253278331
ENSBTAG00000032022	*RPL23A*	6'577	23'919	16'658	5'696	27'619	2.278	0.004189705	0.253278331
ENSBTAG00000047699	*BT.64721*	2'965	10'899	7'576	2'568	12'585	2.293	0.004195113	0.253278331
ENSBTAG00000046871	*processed_pseudogene*	17	194	119	15	224	3.927	0.004305171	0.258323502
ENSBTAG00000047202	*GRIN1*	411	31	196	356	36	-3.314	0.004293255	0.258323502
ENSBTAG00000007754	*NDUFA3*	283	1'211	822	245	1'398	2.512	0.004373485	0.261617578
ENSBTAG00000035438	*protein_coding*	9	149	90	8	172	4.464	0.004390701	0.261844234
ENSBTAG00000012855	*LPL*	2'807	400	1'446	2'431	462	-2.396	0.004473469	0.265158439
ENSBTAG00000018563	*SFRP2*	161'696	25'524	84'753	140'033	29'473	-2.248	0.004467437	0.265158439
ENSBTAG00000008017	*UNC79*	194	6	87	168	7	-4.600	0.004494567	0.265601696
ENSBTAG00000039302	*ANGPTL6*	1'019	121	511	882	140	-2.659	0.0045111	0.265773274
ENSBTAG00000000535	*PCNXL2*	142	2	63	123	2	-5.735	0.004607452	0.269819572
ENSBTAG00000001099	*C5H12ORF53*	142	2	63	123	2	-5.735	0.004607452	0.269819572
ENSBTAG00000009355	*SNAP91*	576	55	281	499	64	-2.974	0.00462897	0.270268113
ENSBTAG00000008844	*EXTL3*	1'040	3'883	2'692	901	4'484	2.316	0.00465622	0.271047629
ENSBTAG00000013991	*NR2E1*	156	3	69	135	3	-5.285	0.004690642	0.272238702
ENSBTAG00000001401	*SLC45A1*	102	0	44	88	0		0.004787005	0.275999471
ENSBTAG00000015551	*processed_pseudogene*	2'335	8'417	5'871	2'022	9'719	2.265	0.004784339	0.275999471
ENSBTAG00000047613	*HTRA3*	2'525	9'080	6'336	2'187	10'485	2.261	0.004797899	0.275999471
ENSBTAG00000017509	*MYPN*	4	112	66	3	129	5.222	0.004946778	0.283726796
ENSBTAG00000033326	*DPEP1*	1'097	4'026	2'799	950	4'649	2.291	0.005024855	0.28660242
ENSBTAG00000038490	*CLEC4A*	399	31	191	346	36	-3.271	0.005026308	0.28660242
ENSBTAG00000011246	*FOXP2*	299	18	140	259	21	-3.639	0.005057505	0.286704677
ENSBTAG00000038180	*BT.99915*	647	67	319	560	77	-2.856	0.005054946	0.286704677
ENSBTAG00000010251	*GABRG2*	139	2	61	120	2	-5.704	0.005130887	0.289183312
ENSBTAG00000012119	*PTPRZ1*	6'473	1'005	3'383	5'606	1'160	-2.272	0.005116713	0.289183312
ENSBTAG00000014642	*BT.23360*	1'420	189	724	1'230	218	-2.494	0.005199421	0.292201477
ENSBTAG00000017044	*BT.96871*	188	6	85	163	7	-4.555	0.005286946	0.29626648
ENSBTAG00000005685	*BT.49451*	159	730	490	138	843	2.614	0.00534048	0.298408873
ENSBTAG00000003152	*BT.46545*	789	90	394	683	104	-2.717	0.005364181	0.298876857
ENSBTAG00000004622	*C13H20ORF12*	1'626	224	833	1'408	259	-2.445	0.005409243	0.299503402
ENSBTAG00000013301	*GGT7*	279	16	130	242	18	-3.709	0.005401711	0.299503402
ENSBTAG00000013866	*RPS27*	22'771	3'690	11'991	19'720	4'261	-2.210	0.005421502	0.299503402
ENSBTAG00000006363	*pseudogene*	132	631	421	114	729	2.672	0.005448554	0.30014763
ENSBTAG00000005535	*pseudogene*	27	231	145	23	267	3.512	0.005547908	0.304759883
ENSBTAG00000021649	*protein_coding*	2'304	8'072	5'658	1'995	9'321	2.224	0.005563881	0.304778751
ENSBTAG00000013422	*GABRB3*	175	5	79	152	6	-4.714	0.005587687	0.305225425
ENSBTAG00000004503	*NPY*	346	25	164	300	29	-3.376	0.005685193	0.309684198
ENSBTAG00000012624	*PDZRN4*	338	24	160	293	28	-3.401	0.005732327	0.310516994

Ensembl gene ID	geneName	counts wildtype horn bud	counts polled horn bud	baseMean	baseMean wildtype horn bud	baseMean polled horn bud	log2FoldChange	pval	padj
ENSBTAG00000018253	*CHRNA1*	32	251	159	28	290	3.387	0.005730847	0.310516994
ENSBTAG00000015749	*BT.87354*	379	30	181	328	35	-3.244	0.005965777	0.322267628
ENSBTAG00000005236	*CCKBR*	183	6	83	158	7	-4.516	0.006059944	0.325550881
ENSBTAG00000017719	*AKAP6*	521	51	255	451	59	-2.938	0.006056746	0.325550881
ENSBTAG00000002414	*KCNJ10*	341	25	162	295	29	-3.355	0.006128608	0.327435555
ENSBTAG00000003592	*CNTN6*	118	1	52	102	1	-6.468	0.006113274	0.327435555
ENSBTAG00000021904	*KIF26A*	771	2'771	1'934	668	3'200	2.261	0.006245686	0.332779025
ENSBTAG00000019385	*SCN3A*	330	24	157	286	28	-3.366	0.006482724	0.344467562
ENSBTAG00000020198	*BT.62723*	546	56	269	473	65	-2.870	0.006518876	0.344654083
ENSBTAG00000034433	*protein_coding*	2'730	9'239	6'516	2'364	10'668	2.174	0.006521581	0.344654083
ENSBTAG00000046512	*CMYA1*	5	113	67	4	130	4.913	0.006568683	0.346205099
ENSBTAG00000006999	*RYR1*	523	1'918	1'334	453	2'215	2.290	0.00662397	0.348177986
ENSBTAG00000005260	*SPP1*	115'150	19'642	61'202	99'723	22'681	-2.136	0.006712556	0.351885912
ENSBTAG00000009438	*EPHA5*	179	6	81	155	7	-4.484	0.006764041	0.353634206
ENSBTAG00000006563	*KBTBD5*	35	255	162	30	294	3.280	0.006790521	0.354069406
ENSBTAG00000016407	*IRX6*	643	2'296	1'604	557	2'651	2.251	0.006922955	0.360012118
ENSBTAG00000004354	*protein_coding*	224	11	103	194	13	-3.933	0.007037774	0.363015414
ENSBTAG00000009014	*UPK1B*	712	84	357	617	97	-2.668	0.007042297	0.363015414
ENSBTAG00000011115	*CH25H*	576	62	285	499	72	-2.801	0.007023902	0.363015414
ENSBTAG00000013250	*PCSK1N*	467	45	228	404	52	-2.960	0.007055169	0.363015414
ENSBTAG00000046120	*pseudogene*	3'573	11'836	8'381	3'094	13'667	2.143	0.007090458	0.363871093
ENSBTAG00000002390	*C5H12orf68*	232	12	107	201	14	-3.858	0.007113405	0.364090578
ENSBTAG00000009328	*BT.78357*	114	1	50	99	1	-6.418	0.007196354	0.365142222
ENSBTAG00000014824	*MMP14*	29'035	94'451	67'104	25'145	109'063	2.117	0.00721557	0.365142222
ENSBTAG00000018707	*BT.63558*	48	302	195	42	349	3.068	0.007227573	0.365142222
ENSBTAG00000030179	*MAPK8IP2*	586	64	291	507	74	-2.780	0.007181181	0.365142222
ENSBTAG00000039357	*HRNR*	1'140	156	584	987	180	-2.454	0.007182784	0.365142222
ENSBTAG00000006907	*NEB*	378	1'411	978	327	1'629	2.315	0.007251809	0.365419989
ENSBTAG00000001517	*BT.65129*	86	438	290	74	506	2.764	0.00733	0.368281601
ENSBTAG00000004750	*VSTM2L*	1'182	164	607	1'024	189	-2.434	0.007346369	0.368281601
ENSBTAG00000021444	*TWIST2*	3'146	10'354	7'340	2'725	11'956	2.134	0.007413954	0.370716686
ENSBTAG00000004557	*DHRS9*	113	1	50	98	1	-6.405	0.007497611	0.373940954
ENSBTAG00000001075	*BT.49430*	128	2	57	111	2	-5.585	0.007655069	0.38082017
ENSBTAG00000011275	*SCUBE1*	1'633	244	848	1'414	282	-2.328	0.007721317	0.383138432
ENSBTAG00000008332	*ENPEP*	1'044	3'530	2'490	904	4'076	2.173	0.007776173	0.384881096
ENSBTAG00000033319	*CD200*	1'060	146	543	918	169	-2.445	0.007931188	0.391559717
ENSBTAG00000047295	*MGC140151*	140	3	62	121	3	-5.129	0.007988113	0.393374242
ENSBTAG00000021092	*ATP6V0D2*	364	31	176	315	36	-3.139	0.008044744	0.395165127
ENSBTAG00000012919	*MMP15*	2'053	6'698	4'756	1'778	7'734	2.121	0.008148437	0.399252949
ENSBTAG00000012916	*pseudogene*	914	3'077	2'172	792	3'553	2.166	0.0082543	0.40282248
ENSBTAG00000038168	*SLITRK1*	139	3	62	120	3	-5.119	0.008262602	0.40282248
ENSBTAG00000004050	*pseudogene*	125	558	376	108	644	2.573	0.008320639	0.404215567
ENSBTAG00000014179	*SLC12A5*	208	10	96	180	12	-3.963	0.008332632	0.404215567
ENSBTAG00000005562	*GALNT13*	217	857	589	188	990	2.397	0.008367925	0.404823866
ENSBTAG00000013071	*GPR153*	1'014	3'383	2'392	878	3'906	2.153	0.00838669	0.404823866
ENSBTAG00000019066	*FAM167A*	1'335	197	692	1'156	227	-2.346	0.008539117	0.411163766
ENSBTAG00000034693	*SYT1*	372	33	180	322	38	-3.080	0.008631708	0.413579715
ENSBTAG00000046989	*MANEAL*	565	64	282	489	74	-2.727	0.008629828	0.413579715
ENSBTAG00000008924	*TNFSF11*	149	4	67	129	5	-4.804	0.008729279	0.417229574
ENSBTAG00000003485	*MAGOH*	81	405	269	70	468	2.737	0.008850541	0.420292881
ENSBTAG00000016026	*PCOLCE2*	780	101	396	675	117	-2.534	0.008816237	0.420292881
ENSBTAG00000037986	*BT.34345*	383	35	186	332	40	-3.037	0.008858026	0.420292881
ENSBTAG00000004046	*DTX4*	940	3'113	2'204	814	3'595	2.143	0.00889214	0.420887447
ENSBTAG00000011677	*HIST1H1C*	213	11	99	184	13	-3.860	0.008999185	0.423945485
ENSBTAG00000023895	*pseudogene*	385	1'374	960	333	1'587	2.250	0.009000227	0.423945485
ENSBTAG00000003234	*HSD17B2*	56	317	207	48	366	2.916	0.009077304	0.42552043
ENSBTAG00000013358	*RPS23*	2'800	8'878	6'338	2'425	10'251	2.080	0.009056407	0.42552043
ENSBTAG00000045585	*FKBP8*	401	1'421	994	347	1'641	2.240	0.009100361	0.42557829
ENSBTAG00000004400	*PRRC2B*	4'202	13'197	9'439	3'639	15'239	2.066	0.009169951	0.427806728
ENSBTAG00000000995	*FAM46A*	1'936	309	1'017	1'677	357	-2.232	0.009245773	0.429753316
ENSBTAG00000019498	*BT.103104*	1'590	5'099	3'632	1'377	5'888	2.096	0.009255751	0.429753316
ENSBTAG00000006631	*GLI1*	1'874	5'973	4'260	1'623	6'897	2.087	0.009287555	0.430205706
ENSBTAG00000002616	*pseudogene*	51	297	194	44	343	2.957	0.009425842	0.435433461
ENSBTAG00000005182	*BOLA*	836	2'755	1'953	724	3'181	2.136	0.009445072	0.435433461
ENSBTAG00000015789	*BCAN*	2'212	362	1'167	1'916	418	-2.196	0.009606789	0.44086242
ENSBTAG00000039122	*BT.84824*	107	1	47	93	1	-6.326	0.009608047	0.44086242
ENSBTAG00000021949	*NEFL*	166	6	75	144	7	-4.375	0.009712901	0.444627403
ENSBTAG00000021217	*COL11A1*	63'277	11'597	34'095	54'799	13'391	-2.033	0.009736574	0.44466726
ENSBTAG00000004599	*TAGLN3*	655	82	331	567	95	-2.583	0.009809373	0.446513063
ENSBTAG00000008272	*EBF3*	1'606	253	841	1'391	292	-2.251	0.009822784	0.446513063
ENSBTAG00000047712	*processed_pseudogene*	156	637	435	135	736	2.445	0.009929123	0.450297289
ENSBTAG00000015525	*SLC39A12*	106	1	46	92	1	-6.313	0.010016803	0.451126277

Ensembl gene ID	geneName	counts wildtype horn bud	counts polled horn bud	baseMean	baseMean wildtype horn bud	baseMean polled horn bud	log2FoldChange	pval	padj
ENSBTAG00000017369	*MAMDC2*	755	100	385	654	115	-2.501	0.00998558	0.451126277
ENSBTAG00000017473	*LAMC3*	1'114	3'570	2'544	965	4'122	2.095	0.010012509	0.451126277
ENSBTAG00000019422	*pseudogene*	71	361	239	61	417	2.761	0.010109153	0.454236394
ENSBTAG00000001615	*RUNDC3A*	238	15	112	206	17	-3.573	0.010227672	0.455882074
ENSBTAG00000008113	*OSR1*	903	2'915	2'074	782	3'366	2.106	0.010221253	0.455882074
ENSBTAG00000009476	*FIGF*	1'298	199	677	1'124	230	-2.290	0.01024665	0.455882074
ENSBTAG00000033143	*pseudogene*	191	742	511	165	857	2.373	0.010262665	0.455882074
ENSBTAG00000045825	*5_8S_rRNA*	3'597	624	1'918	3'115	721	-2.112	0.010261626	0.455882074
ENSBTAG00000001078	*SRL*	259	951	661	224	1'098	2.292	0.010327626	0.457379984
ENSBTAG00000005305	*NTS*	230	14	108	199	16	-3.623	0.010343294	0.457379984
ENSBTAG00000005048	*DHRS7C*	0	59	34	0	68	Inf	0.010374106	0.457704603
ENSBTAG00000000189	*BT.87346*	198	10	92	171	12	-3.892	1.05E-02	0.463589386
ENSBTAG00000011374	*S1PR3*	3'629	634	1'937	3'143	732	-2.102	0.01057206	0.46433727
ENSBTAG00000017071	*C1QTNF3*	11'129	2'037	5'995	9'638	2'352	-2.035	0.010596107	0.464347611
ENSBTAG00000018234	*BT.57043*	85	0	37	74	0		0.010845769	0.474222729
ENSBTAG00000024021	*BT.104105*	142	4	64	123	5	-4.735	0.010897406	0.474464466
ENSBTAG00000047122	*5S_rRNA*	342	31	166	296	36	-3.049	0.010899958	0.474464466
ENSBTAG00000027081	*ATP10A*	271	974	680	235	1'125	2.261	0.011071182	0.48084438
ENSBTAG00000021823	*HFE2*	0	58	33	0	67	Inf	0.011151322	0.483248725
ENSBTAG00000021669	*KIAA0889*	329	1'146	804	285	1'323	2.215	0.011224012	0.48532032
ENSBTAG00000001491	*KIAA1239*	103	1	45	89	1	-6.271	0.011356777	0.488892941
ENSBTAG00000014176	*REM1*	947	2'985	2'133	820	3'447	2.071	0.01134082	0.488892941
ENSBTAG00000019694	*ADAM23*	612	78	310	530	90	-2.557	0.01146965	0.492664404
ENSBTAG00000004591	*protein_coding*	416	44	206	360	51	-2.826	0.011632649	0.498534822
ENSBTAG00000017866	*CD36*	3'157	558	1'689	2'734	644	-2.085	0.011683012	0.498534822
ENSBTAG00000018303	*PAPPA2*	73	357	238	63	412	2.705	0.011659696	0.498534822
ENSBTAG00000017280	*BT.19562*	479	55	239	415	64	-2.707	0.011763615	0.500878283
ENSBTAG00000006432	*KCNE4*	1'473	239	776	1'276	276	-2.209	0.011808403	0.501689899
ENSBTAG00000031464	*pseudogene*	214	790	549	185	912	2.299	0.011834393	0.501701072
ENSBTAG00000011590	*GLIPR1*	285	23	137	247	27	-3.216	0.011886699	0.50282543
ENSBTAG00000004565	*pseudogene*	626	2'005	1'429	542	2'315	2.094	0.012060284	0.507964574
ENSBTAG00000006620	*SLC24A4*	37	234	151	32	270	3.076	0.012037787	0.507964574
ENSBTAG00000047231	*TNNI1*	11	131	80	10	151	3.989	0.012114834	0.509162447
ENSBTAG00000040279	*KRT24*	263	20	125	228	23	-3.302	0.012338955	0.517466577
ENSBTAG00000034854	*BT.26387*	138	4	62	120	5	-4.693	0.012384174	0.518248465
ENSBTAG00000006924	*BT.26336*	115	2	51	100	2	-5.430	0.012421731	0.518707025
ENSBTAG00000014721	*MUC4*	467	54	233	404	62	-2.697	0.012531278	0.521050011
ENSBTAG00000040386	*BT.29722*	484	57	242	419	66	-2.671	0.012531088	0.521050011
ENSBTAG00000031704	*MGC133804*	82	0	36	71	0		0.0125835	0.522108142
ENSBTAG00000040277	*H4*	248	18	118	215	21	-3.369	0.012688494	0.525346743
ENSBTAG00000001822	*SNX10*	566	72	287	490	83	-2.560	0.01271743	0.525429245
ENSBTAG00000008687	*PCSK2*	126	3	56	109	3	-4.977	0.012888998	0.529154404
ENSBTAG00000015576	*NPAS1*	126	3	56	109	3	-4.977	0.012888998	0.529154404
ENSBTAG00000022278	*RPL23A*	10'019	29'138	21'161	8'677	33'646	1.955	0.012864546	0.529154404
ENSBTAG00000016857	*ADAMTS15*	728	2'272	1'627	630	2'623	2.057	0.012930331	0.529736084
ENSBTAG00000007879	*SRRM3*	273	22	131	236	25	-3.218	0.013024929	0.531378969
ENSBTAG00000018926	*LEAP2*	260	20	124	225	23	-3.285	0.013009652	0.531378969
ENSBTAG00000011190	*FLNA*	29'675	85'681	62'318	25'699	98'936	1.945	0.013067693	0.532010626
ENSBTAG00000001874	*DOCK3*	322	30	157	279	35	-3.009	0.01325333	0.53620993
ENSBTAG00000010769	*BT.56814*	81	0	35	70	0		0.013226432	0.53620993
ENSBTAG00000016920	*EOMES*	81	0	35	70	0		0.013226432	0.53620993
ENSBTAG00000007635	*PLCL1*	369	38	182	320	44	-2.865	0.013386216	0.538236275
ENSBTAG00000011702	*BT.64987*	252	19	120	218	22	-3.314	0.013335296	0.538236275
ENSBTAG00000031278	*SNPH*	111	464	316	96	536	2.479	0.013385906	0.538236275
ENSBTAG00000010977	*FRZB*	942	144	491	816	166	-2.295	0.013426828	0.538758376
ENSBTAG00000008238	*S100A7*	2'661	479	1'429	2'304	553	-2.059	0.013512786	0.54109414
ENSBTAG00000008778	*CHST13*	320	30	156	277	35	-3.000	0.013640459	0.545087289
ENSBTAG00000016334	*BT.41615*	1'346	223	712	1'166	257	-2.179	0.013711817	0.546400726
ENSBTAG00000045896	*NPTX2*	151	581	401	131	671	2.359	0.013729365	0.546400726
ENSBTAG00000008652	*DUSP27*	15	145	90	13	167	3.688	0.013759441	0.546482401
ENSBTAG00000017136	*SSTR2*	313	29	152	271	33	-3.017	0.013827429	0.548066433
ENSBTAG00000043993	*C1H21orf62*	42	244	159	36	282	2.953	0.013878011	0.548955567
ENSBTAG00000001051	*OSCAR*	112	2	50	97	2	-5.392	0.013913789	0.549256698
ENSBTAG00000000595	*FBN3*	1'216	3'618	2'615	1'053	4'178	1.988	0.014106309	0.552383818
ENSBTAG00000002080	*NOV*	3'462	642	1'870	2'998	741	-2.016	0.014089652	0.552383818
ENSBTAG00000013711	*TMEM151B*	178	9	82	154	10	-3.891	0.014073432	0.552383818
ENSBTAG00000014525	*SLC6A13*	98	1	43	85	1	-6.200	0.014026374	0.552383818
ENSBTAG00000006505	*S100A9*	628	86	322	544	99	-2.453	0.014178853	0.55411186
ENSBTAG00000000061	*PCDH7*	755	111	391	654	128	-2.351	1.46E-02	5.60E-01
ENSBTAG00000003919	*KIAA0408*	4'003	754	2'169	3'467	871	-1.993	0.014511597	0.559969487
ENSBTAG00000011092	*KNDC1*	241	18	115	209	21	-3.328	0.014454504	0.559969487
ENSBTAG00000013550	*PRKCG*	286	25	138	248	29	-3.101	0.014364038	0.559969487

Ensembl gene ID	geneName	counts wildtype horn bud	counts polled horn bud	baseMean	baseMean wildtype horn bud	baseMean polled horn bud	log2FoldChange	pval	padj
ENSBTAG00000014960	*SCHIP1*	1'397	236	741	1'210	273	-2.150	0.014467043	0.559969487
ENSBTAG00000017976	*ABL1*	3'178	9'120	6'642	2'752	10'531	1.936	0.014598467	0.559969487
ENSBTAG00000019822	*TPPP3*	3'019	8'670	6'313	2'615	10'011	1.937	0.01461589	0.559969487
ENSBTAG00000026111	*LBH*	3'410	9'784	7'125	2'953	11'298	1.936	0.014522948	0.559969487
ENSBTAG00000027766	*C1QTNF5*	1'925	341	1'030	1'667	394	-2.082	0.014583004	0.559969487
ENSBTAG00000035493	*pseudogene*	2'579	7'448	5'417	2'233	8'600	1.945	0.014455579	0.559969487
ENSBTAG00000004954	*TOX*	1'020	163	536	883	188	-2.231	0.014800942	0.565947383
ENSBTAG00000040518	*processed_pseudogene*	3'196	9'125	6'652	2'768	10'537	1.929	0.014952743	0.570632944
ENSBTAG00000045507	*ZNF469*	2'110	6'074	4'420	1'827	7'014	1.940	0.015036741	0.572717736
ENSBTAG00000013919	*BT.88538*	2'094	378	1'125	1'813	436	-2.055	0.015167814	0.576583891
ENSBTAG00000033887	*BT.87855*	449	1'414	1'011	389	1'633	2.070	0.015211161	0.577106712
ENSBTAG00000006973	*PHF21B*	96	1	42	83	1	-6.170	0.015272367	0.578303727
ENSBTAG00000002492	*SYCP3*	369	40	183	320	46	-2.791	0.015453303	0.584021059
ENSBTAG00000040414	*pseudogene*	2'514	7'160	5'222	2'177	8'268	1.925	0.015495321	0.584476321
ENSBTAG00000005990	*S1PR1*	3'477	660	1'887	3'011	762	-1.982	0.015625179	0.588236713
ENSBTAG00000006963	*RPL12*	521	1'605	1'152	451	1'853	2.038	0.015724248	0.589689534
ENSBTAG00000013031	*TMEM62*	630	89	324	546	103	-2.408	0.01570472	0.589689534
ENSBTAG00000001028	*MAP6*	120	3	54	104	3	-4.907	0.015878346	0.591921453
ENSBTAG00000010551	*ATP1A2*	1'974	358	1'061	1'710	413	-2.048	0.015916114	0.591921453
ENSBTAG00000013183	*COL23A1*	798	2'372	1'715	691	2'739	1.987	0.015846486	0.591921453
ENSBTAG00000013462	*L1CAM*	2'325	429	1'254	2'014	495	-2.023	0.015909173	0.591921453
ENSBTAG00000018179	*PCDH8*	188	11	88	163	13	-3.680	0.01593553	0.591921453
ENSBTAG00000003514	*HSF4*	475	60	240	411	69	-2.570	0.016053776	0.593441435
ENSBTAG00000018901	*CTIF*	446	1'390	996	386	1'605	2.055	0.016035479	0.593441435
ENSBTAG00000038233	*protein_coding*	0	53	31	0	61	Inf	0.016074711	0.593441435
ENSBTAG00000046602	*BT.94420*	112	449	308	97	518	2.418	0.016098175	0.593441435
ENSBTAG00000000590	*BT.65618*	351	1'124	801	304	1'298	2.094	0.016131477	0.593547057
ENSBTAG00000033153	*GRIK2*	228	17	109	197	20	-3.330	0.0163346	0.59988895
ENSBTAG00000004035	*SDK1*	596	1'793	1'293	516	2'070	2.004	0.016612565	0.606626397
ENSBTAG00000017116	*RASD2*	275	906	642	238	1'046	2.135	0.016615209	0.606626397
ENSBTAG00000025531	*PSORS1C2*	214	15	101	185	17	-3.420	0.016595615	0.606626397
ENSBTAG00000039981	*pseudogene*	98	405	276	85	468	2.462	0.016642486	0.606626397
ENSBTAG00000021903	*KIAA0319*	138	5	63	120	6	-4.372	0.016775519	0.610334689
ENSBTAG00000005033	*BT.67294*	107	2	47	93	2	-5.326	0.016833256	0.611294841
ENSBTAG00000008717	*SERPINE2*	3'034	581	1'649	2'628	671	-1.970	0.01694457	0.613053918
ENSBTAG00000019008	*FBXL16*	427	52	215	370	60	-2.623	0.016921577	0.613053918
ENSBTAG00000047502	*FKBP5*	1'776	323	956	1'538	373	-2.044	0.016992068	0.613633926
ENSBTAG00000012036	*HR*	2'133	5'971	4'371	1'847	6'895	1.900	0.017151064	0.618230862
ENSBTAG00000003470	*TTYH1*	849	135	446	735	156	-2.238	0.017195972	0.618705987
ENSBTAG00000010645	*BOLA-DRA*	3'167	611	1'724	2'743	706	-1.959	0.017272052	0.620298882
ENSBTAG00000007698	*TMEM59L*	1'079	182	572	934	210	-2.153	0.017392629	0.620752178
ENSBTAG00000011082	*IGF-I*	262	23	127	227	27	-3.095	0.017412002	0.620752178
ENSBTAG00000021774	*CELF5*	93	1	41	81	1	-6.124	0.017364104	0.620752178
ENSBTAG00000024929	*PPP1R27*	3	79	47	3	91	5.134	0.017402532	0.620752178
ENSBTAG00000020573	*SCUBE2*	3'378	659	1'843	2'925	761	-1.943	0.017793229	0.633185699
ENSBTAG00000012684	*CCL19*	323	34	159	280	39	-2.833	0.018132159	0.64317177
ENSBTAG00000026461	*CACNA1H*	1'563	4'366	3'198	1'354	5'041	1.897	0.018139812	0.64317177
ENSBTAG00000026437	*ULBP3*	2	71	42	2	82	5.565	0.018225967	0.645053682
ENSBTAG00000018603	*RGMA*	939	2'674	1'950	813	3'088	1.925	0.01849712	0.653464392
ENSBTAG00000003938	*FNDC1*	14'193	38'158	28'176	12'291	44'061	1.842	0.018574143	0.654998831
ENSBTAG00000000910	*SLC6A7*	174	10	81	151	12	-3.706	0.018698639	0.658198857
ENSBTAG00000019033	*CD84*	363	42	181	314	48	-2.696	0.019038766	0.668963917
ENSBTAG00000021313	*RUNDC3B*	330	36	164	286	42	-2.781	0.019155557	0.671857029
ENSBTAG00000013366	*NRIP3*	871	144	460	754	166	-2.182	0.01936077	0.676620742
ENSBTAG00000018688	*CBR3*	462	1'378	996	400	1'591	1.992	0.019349819	0.676620742
ENSBTAG00000047491	*CACNA1S*	631	1'825	1'327	546	2'107	1.947	0.019534513	0.681471442
ENSBTAG00000027263	*pseudogene*	1'437	3'960	2'909	1'244	4'573	1.877	0.019614014	0.681805512
ENSBTAG00000046917	*GPR22*	114	3	51	99	3	-4.833	0.019601811	0.681805512
ENSBTAG00000007214	*ELL2*	1'299	234	698	1'125	270	-2.058	0.019651947	0.681908582
ENSBTAG00000010820	*BT.21876*	952	2'670	1'954	824	3'083	1.903	0.019786699	0.684149689
ENSBTAG00000016360	*FAM18A*	90	1	40	78	1	-6.077	0.019758917	0.684149689
ENSBTAG00000013507	*SPRR3*	2'646	520	1'446	2'292	600	-1.932	0.019855756	0.685322306
ENSBTAG00000009125	*ALDH1A3*	2'023	388	1'100	1'752	448	-1.967	0.019916094	0.686190363
ENSBTAG00000009274	*protein_coding*	24	166	106	21	192	3.205	0.020025434	0.688740716
ENSBTAG00000015939	*PVRL1*	600	1'732	1'260	520	2'000	1.944	0.020092238	0.689821714
ENSBTAG00000012761	*PTK7*	10'865	28'686	21'267	9'409	33'124	1.816	0.020340782	0.697127561
ENSBTAG00000044202	*CNKSR2*	210	16	100	182	18	-3.299	0.020524	0.70217286
ENSBTAG00000021177	*ADAMTS14*	525	1'524	1'107	455	1'760	1.953	0.020683897	0.706403986
ENSBTAG00000001404	*CACNG3*	72	0	31	62	0		0.020851257	0.709856305
ENSBTAG00000014312	*ATL1*	392	49	198	339	57	-2.585	0.020857785	0.709856305
ENSBTAG00000009292	*TMEM100*	1'384	256	747	1'199	296	-2.020	0.020962399	0.712173776
ENSBTAG00000047475	*BT.44249*	84	345	236	73	398	2.453	0.02110726	0.715848132

Ensembl gene ID	geneName	counts wildtype horn bud	counts polled horn bud	baseMean	baseMean wildtype horn bud	baseMean polled horn bud	log2FoldChange	pval	padj
ENSBTAG00000046056	*pseudogene*	8	102	62	7	118	4.087	0.021153598	0.716174169
ENSBTAG00000014972	*PTGER4*	1'650	314	896	1'429	363	-1.979	0.021275785	0.719062551
ENSBTAG00000006073	*PODNL1*	195	14	93	169	16	-3.385	0.021518353	0.724755862
ENSBTAG00000025837	*RFX4*	121	4	55	105	5	-4.504	0.021518571	0.724755862
ENSBTAG00000004645	*ADAMTSL3*	592	90	308	513	104	-2.303	0.021623713	0.727041418
ENSBTAG00000000442	*RBP4*	2'105	415	1'151	1'823	479	-1.928	0.021959163	0.733476306
ENSBTAG00000011290	*processed_pseudogene*	583	1'655	1'208	505	1'911	1.920	0.021943431	0.733476306
ENSBTAG00000015837	*P2RY12*	181	12	85	157	14	-3.500	0.021965549	0.733476306
ENSBTAG00000038281	*CSMD3*	71	0	31	61	0		0.021949585	0.733476306
ENSBTAG00000012413	*BT.37226*	487	69	251	422	80	-2.404	0.022069923	0.735701826
ENSBTAG00000004145	*ANO4*	137	6	63	119	7	-4.098	0.022316492	0.738869126
ENSBTAG00000010156	*TPT1*	16'040	41'444	30'873	13'891	47'855	1.785	0.022297681	0.738869126
ENSBTAG00000018465	*KCNAB1*	345	41	173	299	47	-2.658	0.022267837	0.738869126
ENSBTAG00000047167	*protein_coding*	187	13	88	162	15	-3.431	0.022248438	0.738869126
ENSBTAG00000007036	*SLC17A7*	406	53	206	352	61	-2.522	0.022368352	0.739330899
ENSBTAG00000005078	*UCHL1*	3'287	675	1'813	2'847	779	-1.869	0.022423204	0.739889848
ENSBTAG00000015829	*ENPP5*	280	29	138	242	33	-2.856	0.022615428	0.743715791
ENSBTAG00000046266	*TNFSF9*	110	3	49	95	3	-4.781	0.022583085	0.743715791
ENSBTAG00000002468	*RPS28*	1'390	3'711	2'744	1'204	4'285	1.832	0.022843113	0.744357446
ENSBTAG00000002923	*CX3CR1*	600	93	314	520	107	-2.275	0.022684421	0.744357446
ENSBTAG00000010693	*LMO7*	5'128	1'082	2'845	4'441	1'249	-1.830	0.022847127	0.744357446
ENSBTAG00000013798	*PTPRN*	144	7	66	125	8	-3.948	0.022863961	0.744357446
ENSBTAG00000015808	*BT.43552*	1'546	4'112	3'044	1'339	4'748	1.826	0.022812091	0.744357446
ENSBTAG00000018790	*BVES*	31	184	120	27	212	2.984	0.022782268	0.744357446
ENSBTAG00000002296	*TMEM173*	778	131	413	674	151	-2.155	0.022946068	0.744544557
ENSBTAG00000012638	*S100A12*	6'435	1'370	3'577	5'573	1'582	-1.817	0.022918873	0.744544557
ENSBTAG00000009830	*BT.20038*	378	48	191	327	55	-2.562	0.02305593	0.745628021
ENSBTAG00000032844	*BT.35529*	295	32	146	255	37	-2.790	0.023031065	0.745628021
ENSBTAG00000008504	*SYNGR3*	535	80	278	463	92	-2.326	0.02314676	0.746675581
ENSBTAG00000015470	*BT.43947*	30	180	117	26	208	3.000	0.02320319	0.746675581
ENSBTAG00000031750	*PLAC8*	321	37	160	278	43	-2.702	0.023180541	0.746675581
ENSBTAG00000006841	*LRRC4B*	885	155	473	766	179	-2.098	0.023540787	0.756291428
ENSBTAG00000003069	*MAN1C1*	1'530	296	833	1'325	342	-1.955	0.023653708	0.757423588
ENSBTAG00000012409	*BT.64746*	304'654	67'392	170'828	263'838	77'818	-1.761	0.023622265	0.757423588
ENSBTAG00000023941	*BT.62807*	118	4	53	102	5	-4.468	0.023756992	0.759483762
ENSBTAG00000017733	*BT.49731*	517	77	268	448	89	-2.332	0.023844546	0.76103519
ENSBTAG00000017430	*PNCK*	260	26	128	225	30	-2.907	0.023898918	0.761524171
ENSBTAG00000006608	*GGT5*	866	152	463	750	176	-2.095	0.024163396	0.768695579
ENSBTAG00000000588	*CHODL*	644	1'774	1'303	558	2'048	1.877	0.024236859	0.769776859
ENSBTAG00000000609	*pseudogene*	26'676	67'547	50'549	23'102	77'997	1.755	0.02429743	0.770445837
ENSBTAG00000021077	*BOLA-DQB*	312	36	156	270	42	-2.700	0.024384868	0.771963175
ENSBTAG00000045518	*5S_rRNA*	156	9	73	135	10	-3.700	0.024518769	0.774944117
ENSBTAG00000018326	*FXYD7*	213	18	103	184	21	-3.150	0.024574319	0.77544304
ENSBTAG00000019602	*SLCO1C1*	176	12	83	152	14	-3.459	0.024620709	0.775651776
ENSBTAG00000004799	*DENND2A*	4'068	10'395	7'763	3'523	12'003	1.769	0.024662135	0.775703696
ENSBTAG00000001303	*HSPB8*	742	2'013	1'484	643	2'324	1.855	0.024759949	0.777526185
ENSBTAG00000005857	*SLC6A1*	965	175	519	836	202	-2.048	0.024917278	0.781208742
ENSBTAG00000024137	*PTCH2*	2'466	6'343	4'730	2'136	7'324	1.778	0.024966465	0.781494422
ENSBTAG00000018307	*SEMA3F*	4'477	11'390	8'515	3'877	13'152	1.762	0.025009571	0.78158918
ENSBTAG00000012100	*PDE1A*	716	121	380	620	140	-2.150	0.025149838	0.783528921
ENSBTAG00000018267	*TRIM54*	3	72	43	3	83	5.000	0.025151998	0.783528921
ENSBTAG00000017043	*GPR1*	240	23	117	208	27	-2.968	0.025285506	0.786431662
ENSBTAG00000002174	*RERG*	1'280	246	696	1'109	284	-1.964	0.025519873	0.791369072
ENSBTAG00000032432	*BT.87860*	3'220	8'197	6'127	2'789	9'465	1.763	0.025525416	0.791369072
ENSBTAG00000014381	*BT.25906*	68	0	29	59	0		0.025627632	0.793276904
ENSBTAG00000010531	*CYP1B1*	493	74	256	427	85	-2.321	0.025910976	0.798465533
ENSBTAG00000014596	*EFHD1*	692	117	367	599	135	-2.149	0.026040733	0.798465533
ENSBTAG00000015127	*SDC4*	1'095	2'874	2'133	948	3'319	1.807	0.025906216	0.798465533
ENSBTAG00000017209	*GLRA2*	106	3	48	92	3	-4.728	0.026040925	0.798465533
ENSBTAG00000018225	*SIPA1L3*	989	2'609	1'935	856	3'013	1.814	0.025930146	0.798465533
ENSBTAG00000044033	*EDIL3*	1'877	381	1'033	1'626	440	-1.886	0.025979717	0.798465533
ENSBTAG00000002624	*PSTPIP2*	1'004	186	542	869	215	-2.017	0.026153767	0.800666587
ENSBTAG00000005110	*CADPS2*	762	133	407	660	154	-2.103	0.026506972	0.80830666
ENSBTAG00000011904	*HCFC1*	2'872	7'265	5'438	2'487	8'389	1.754	0.026527679	0.80830666
ENSBTAG00000016347	*BT.61659*	196	618	442	170	714	2.072	0.026467114	0.80830666
ENSBTAG00000002786	*protein_coding*	15	124	78	13	143	3.462	0.026701516	0.809807571
ENSBTAG00000003301	*NCAM2*	555	88	291	481	102	-2.242	0.026667731	0.809807571
ENSBTAG00000013502	*SLC1A6*	123	5	56	107	6	-4.206	0.026652033	0.809807571
ENSBTAG00000001829	*SLC17A9*	1'349	265	737	1'168	306	-1.933	0.026919766	0.809887623
ENSBTAG00000018309	*pseudogene*	438	1'222	895	379	1'411	1.895	0.026876634	0.809887623
ENSBTAG00000019060	*BT.71689*	284	32	141	246	37	-2.735	0.026993178	0.809887623
ENSBTAG00000024269	*TGFBR3*	6'300	15'723	11'806	5'456	18'155	1.734	0.026818003	0.809887623

Ensembl gene ID	geneName	counts wildtype horn bud	counts polled horn bud	baseMean	baseMean wildtype horn bud	baseMean polled horn bud	log2FoldChange	pval	padj
ENSBTAG00000027899	*protein_coding*	220	20	107	191	23	-3.044	0.026757499	0.809887623
ENSBTAG00000038913	*pseudogene*	1'212	3'137	2'336	1'050	3'622	1.787	0.02699487	0.809887623
ENSBTAG00000047548	*protein_coding*	280	827	599	242	955	1.977	0.02695031	0.809887623
ENSBTAG00000033389	*pseudogene*	2'360	5'965	4'466	2'044	6'888	1.753	0.027151754	0.813343084
ENSBTAG00000012297	*FAM65C*	1'651	4'213	3'147	1'430	4'865	1.767	0.027244839	0.814879756
ENSBTAG00000017641	*protein_coding*	32	179	117	28	207	2.899	0.027423236	0.81895947
ENSBTAG00000015527	*BT.22679*	2'097	437	1'160	1'816	505	-1.848	0.027622522	0.823649537
ENSBTAG00000013364	*LIPM*	550	88	289	476	102	-2.229	0.027721679	0.825344222
ENSBTAG00000021626	*MLC1*	82	1	36	71	1	-5.943	0.028000205	0.832365863
ENSBTAG00000040586	*BT.104539*	2'901	623	1'616	2'512	719	-1.804	0.028071404	0.83321226
ENSBTAG00000021497	*CDH23*	3'073	7'661	5'754	2'661	8'846	1.733	0.028158414	0.834524655
ENSBTAG00000003718	*BT.23353*	2'257	476	1'252	1'955	550	-1.830	0.028222324	0.835149543
ENSBTAG00000006133	*pseudogene*	1'719	4'325	3'241	1'489	4'994	1.746	0.028849359	0.84983587
ENSBTAG00000032150	*processed_pseudogene*	2'074	5'190	3'895	1'796	5'993	1.738	0.028823289	0.84983587
ENSBTAG00000048213	*PTCH1*	266	780	566	230	901	1.967	0.02879385	0.84983587
ENSBTAG00000009059	*PITX2*	1'073	2'749	2'052	929	3'174	1.772	0.028997786	0.852919795
ENSBTAG00000027524	*SMTNL1*	6	85	52	5	98	4.239	0.02917802	0.856928554
ENSBTAG00000014465	*SERPINE1*	787	2'049	1'524	682	2'366	1.796	0.029235052	0.857312394
ENSBTAG00000018732	*HSPA12B*	2'951	7'298	5'491	2'556	8'427	1.721	0.029290718	0.857655099
ENSBTAG00000001981	*SLC6A17*	98	356	248	85	411	2.276	0.029351807	0.858155306
ENSBTAG00000000253	*PTPRF*	5'556	13'572	10'242	4'812	15'672	1.704	0.029696088	0.859203864
ENSBTAG00000008868	*CAPN3*	66	271	185	57	313	2.453	0.029678796	0.859203864
ENSBTAG00000012653	*CAMK2B*	408	59	211	353	68	-2.375	0.029686769	0.859203864
ENSBTAG00000015405	*DCHS1*	6'648	16'219	12'243	5'757	18'728	1.702	0.029632903	0.859203864
ENSBTAG00000017222	*CTNND2*	596	100	316	516	115	-2.160	0.029537106	0.859203864
ENSBTAG00000019521	*COX6A2*	3	69	41	3	80	4.939	0.029502734	0.859203864
ENSBTAG00000024115	*FOXO6*	162	518	369	140	598	2.092	0.029494884	0.859203864
ENSBTAG00000000440	*HSPB2*	185	574	412	160	663	2.049	0.029808336	0.861173866
ENSBTAG00000002834	*CCDC69*	75	294	202	65	339	2.386	0.03009528	0.863565624
ENSBTAG00000014523	*DIRAS2*	161	11	76	139	13	-3.456	0.030101595	0.863565624
ENSBTAG00000020632	*NQO1*	539	88	284	467	102	-2.200	0.030201105	0.863565624
ENSBTAG00000020895	*LOXL4*	660	115	352	572	133	-2.106	0.030143438	0.863565624
ENSBTAG00000021259	*C2CD2*	215	647	467	186	747	2.004	0.030063903	0.863565624
ENSBTAG00000025903	*GLDC*	602	102	320	521	118	-2.146	0.030163432	0.863565624
ENSBTAG00000047008	*processed_pseudogene*	4'381	10'694	8'071	3'794	12'348	1.703	0.03017014	0.863565624
ENSBTAG00000014051	*ANGPT1*	519	84	273	449	97	-2.212	0.030676312	0.875869355
ENSBTAG00000017427	*FAM180A*	185	569	409	160	657	2.036	0.030945207	0.879682917
ENSBTAG00000020998	*RUVBL1*	3'037	7'418	5'598	2'630	8'566	1.703	0.03087911	0.879682917
ENSBTAG00000046232	*protein_coding*	1'178	2'961	2'220	1'020	3'419	1.745	0.030931711	0.879682917
ENSBTAG00000011465	*BT.54476*	150	482	343	130	557	2.099	0.031059439	0.880134188
ENSBTAG00000015317	*BT.37992*	299	37	151	259	43	-2.600	0.031111905	0.880134188
ENSBTAG00000015751	*MEOX1*	2'291	496	1'278	1'984	573	-1.793	0.031231878	0.880134188
ENSBTAG00000016692	*DSCAML1*	195	17	94	169	20	-3.105	0.031224332	0.880134188
ENSBTAG00000034529	*HMGA1*	1'491	3'705	2'785	1'291	4'278	1.728	0.031165222	0.880134188
ENSBTAG00000039504	*protein_coding*	5'181	1'175	2'922	4'487	1'357	-1.726	0.031189083	0.880134188
ENSBTAG00000020126	*BT.103339*	12'058	2'797	6'836	10'443	3'230	-1.693	0.031375068	0.88289352
ENSBTAG00000011762	*ANKRD52*	1'051	2'647	1'983	910	3'056	1.748	0.031420898	0.882909116
ENSBTAG00000002216	*MYOD1*	0	44	25	0	51	Inf	0.031648174	0.886739992
ENSBTAG00000018650	*HEPACAM*	341	46	174	295	53	-2.475	0.031645163	0.886739992
ENSBTAG00000003989	*BT.28194*	338	936	687	293	1'081	1.885	0.031793397	0.886985743
ENSBTAG00000005974	*APOBEC2*	103	362	254	89	418	2.228	0.031772664	0.886985743
ENSBTAG00000019172	*IGSF21*	146	9	68	126	10	-3.605	0.031704109	0.886985743
ENSBTAG00000005968	*BT.106457*	237	25	117	205	29	-2.830	0.03188952	0.888396474
ENSBTAG00000015595	*MCM5*	1'171	2'922	2'194	1'014	3'374	1.734	0.031981004	0.889674126
ENSBTAG00000004754	*DSCAM*	109	4	50	94	5	-4.353	0.03204881	0.890290376
ENSBTAG00000009037	*GALNT12*	363	51	187	314	59	-2.416	0.032203498	0.890780719
ENSBTAG00000013321	*protein_coding*	2'339	511	1'308	2'026	590	-1.779	0.032173678	0.890780719
ENSBTAG00000014449	*BT.73176*	334	924	678	289	1'067	1.883	0.032147171	0.890780719
ENSBTAG00000003490	*BT.16985*	2'037	440	1'136	1'764	508	-1.796	0.032264701	0.891209544
ENSBTAG00000003066	*NSA2*	4'283	973	2'416	3'709	1'124	-1.723	0.032462653	0.894007588
ENSBTAG00000011854	*SLC38A5*	458	72	240	397	83	-2.254	0.032549376	0.894007588
ENSBTAG00000021204	*TES*	1'223	3'036	2'282	1'059	3'506	1.727	0.032434444	0.894007588
ENSBTAG00000047547	*RPS23*	6'315	15'095	11'450	5'469	17'430	1.672	0.032516016	0.894007588
ENSBTAG00000009159	*PLXNA1*	2'185	5'308	4'011	1'892	6'129	1.696	0.032672438	0.89486688
ENSBTAG00000043250	*7SK*	881	169	479	763	195	-1.967	0.032658991	0.89486688
ENSBTAG00000005235	*DPP10*	275	33	138	238	38	-2.644	0.03306858	0.904446535
ENSBTAG00000005410	*RGS7*	192	17	93	166	20	-3.082	0.033152277	0.905465757
ENSBTAG00000019708	*ACSL6*	169	13	81	146	15	-3.285	0.033203376	0.905593067
ENSBTAG00000004888	*SLC10A4*	63	0	27	55	0		0.033280539	0.906429875
ENSBTAG00000021919	*NAV1*	2'672	6'431	4'870	2'314	7'426	1.682	0.033333243	0.906599116
ENSBTAG00000012793	*pseudogene*	357	970	715	309	1'120	1.857	0.033479015	0.909295646
ENSBTAG00000004475	*CDK5R1*	409	62	213	354	72	-2.307	0.033769213	0.915901844

Ensembl gene ID	geneName	counts wildtype horn bud	counts polled horn bud	baseMean	baseMean wildtype horn bud	baseMean polled horn bud	log2FoldChange	pval	padj
ENSBTAG00000022244	*protein_coding*	27	155	101	23	179	2.936	0.033854681	0.916944618
ENSBTAG00000002184	*FNDC5*	516	1'341	998	447	1'548	1.793	0.034110039	0.922579574
ENSBTAG00000006352	*MYT1*	115	5	53	100	6	-4.109	0.034244314	0.924928488
ENSBTAG00000007077	*ABHD1*	562	97	299	487	112	-2.119	0.034321408	0.925165368
ENSBTAG00000007444	*C1H21ORF7*	144	456	326	125	527	2.078	0.034347968	0.925165368
ENSBTAG00000012907	*BT.33408*	2'147	474	1'203	1'859	547	-1.764	0.034538946	0.929026193
ENSBTAG00000011662	*BT.19261*	743	1'868	1'400	643	2'157	1.745	0.034654006	0.930837156
ENSBTAG00000030855	*CHCHD6*	204	599	434	177	692	1.969	0.034772352	0.932731273
ENSBTAG00000008056	*GAD2*	62	0	27	54	0		0.035082458	0.93669683
ENSBTAG00000015775	*CHRNA7*	20	132	85	17	152	3.138	0.035112322	0.93669683
ENSBTAG00000020762	*HUNK*	431	1'133	841	373	1'308	1.809	0.035106144	0.93669683
ENSBTAG00000035654	*protein_coding*	254	717	524	220	828	1.912	0.034983754	0.93669683
ENSBTAG00000008103	*ALDH1A1*	554	96	295	480	111	-2.114	0.035263009	0.939431616
ENSBTAG00000003030	*KCNQ2*	114	5	52	99	6	-4.096	0.035340358	0.940207804
ENSBTAG00000008916	*CYFIP2*	514	87	273	445	100	-2.148	0.035522051	0.943754113
ENSBTAG00000047468	*FAM78B*	154	11	73	133	13	-3.392	0.035603262	0.94462477
ENSBTAG00000006645	*P2RY5*	3'997	927	2'266	3'462	1'070	-1.693	0.035858268	0.949698509
ENSBTAG00000006806	*KRT17*	12'404	28'847	22'026	10'742	33'310	1.633	0.035954021	0.949698509
ENSBTAG00000013849	*RGS13*	97	3	44	84	3	-4.600	0.035994369	0.949698509
ENSBTAG00000030973	*TUBA1D*	105	356	251	91	411	2.177	0.036037993	0.949698509
ENSBTAG00000039890	*FAM127A*	377	1'001	741	326	1'156	1.824	0.035904085	0.949698509
ENSBTAG00000011178	*IL17RB*	171	14	82	148	16	-3.195	0.036126093	0.950735411
ENSBTAG00000003871	*BT.17242*	1'068	219	589	925	253	-1.871	0.03629735	0.952392191
ENSBTAG00000008612	*C1R*	10'684	2'562	6'105	9'253	2'958	-1.645	0.036539211	0.952392191
ENSBTAG00000010411	*ADAMTS8*	343	918	679	297	1'060	1.835	0.036486992	0.952392191
ENSBTAG00000015533	*PTCHD2*	134	8	63	116	9	-3.651	0.036579752	0.952392191
ENSBTAG00000017512	*MAPT*	1'141	237	631	988	274	-1.852	0.036548434	0.952392191
ENSBTAG00000031401	*processed_pseudogene*	255	712	521	221	822	1.896	0.036535088	0.952392191
ENSBTAG00000033679	*HLCS*	1'123	2'728	2'061	973	3'150	1.696	0.036261347	0.952392191
ENSBTAG00000034885	*MGC148992*	1'004	204	553	869	236	-1.884	0.036528001	0.952392191
ENSBTAG00000045574	*pseudogene*	239	674	493	207	778	1.911	0.036632475	0.95249319
ENSBTAG00000047659	*processed_pseudogene*	266	736	540	230	850	1.883	0.036910583	0.958446445
ENSBTAG00000022158	*TNNT3*	1'283	3'083	2'336	1'111	3'560	1.680	0.037032291	0.960328056
ENSBTAG00000008966	*TSPAN7*	7'967	1'907	4'551	6'900	2'202	-1.648	0.037175849	0.962052548
ENSBTAG00000011310	*FAM155B*	79	289	201	68	334	2.286	0.037246791	0.962052548
ENSBTAG00000012608	*GDAP1*	1'301	277	723	1'127	320	-1.817	0.037234405	0.962052548
ENSBTAG00000039995	*BT.68530*	1'189	250	659	1'030	289	-1.835	0.037322266	0.962726876
ENSBTAG00000019977	*PCDH10*	372	56	193	322	65	-2.317	0.037452549	0.964811302
ENSBTAG00000009725	*BT.45005*	668	125	361	579	144	-2.003	0.037630875	0.968126237
ENSBTAG00000018880	*TRPV1*	83	298	208	72	344	2.259	0.037736261	0.969558412
ENSBTAG00000019347	*PLXDC1*	2'526	578	1'427	2'188	667	-1.713	0.037845353	0.971081886
ENSBTAG00000014135	*WDR17*	293	39	149	254	45	-2.494	0.038290815	0.981221009
ENSBTAG00000021191	*EHD2*	8'658	19'874	15'223	7'498	22'949	1.614	0.038396547	0.98263918
ENSBTAG00000004187	*WDR66*	504	87	268	436	100	-2.119	0.038475864	0.983378549
ENSBTAG00000001228	*CEND1*	215	23	106	186	27	-2.810	0.038981279	0.989774169
ENSBTAG00000012266	*pseudogene*	163	487	352	141	562	1.994	0.038967014	0.989774169
ENSBTAG00000012992	*CDH6*	380	988	735	329	1'141	1.794	0.039081386	0.989774169
ENSBTAG00000019081	*COL7A1*	15'529	35'408	27'167	13'449	40'886	1.604	0.039016539	0.989774169
ENSBTAG00000019339	*ITPKB*	762	1'861	1'404	660	2'149	1.703	0.038963042	0.989774169
ENSBTAG00000040103	*SYT8*	215	23	106	186	27	-2.810	0.038981279	0.989774169
ENSBTAG00000047668	*HMG20B*	161	482	348	139	557	1.997	0.039066292	0.989774169
ENSBTAG00000023367	*processed_pseudogene*	462	1'175	878	400	1'357	1.762	0.039169462	0.990718128
ENSBTAG00000027930	*protein_coding*	3'786	8'721	6'674	3'279	10'070	1.619	0.039255929	0.991618996
ENSBTAG00000003711	*EPAS1*	5'562	1'338	3'181	4'817	1'545	-1.640	0.039570714	0.996112468
ENSBTAG00000005150	*HMGB3*	232	645	473	201	745	1.890	0.039689215	0.996112468
ENSBTAG00000006138	*SEMA3C*	3'751	889	2'137	3'248	1'027	-1.662	0.039687408	0.996112468
ENSBTAG00000013391	*ANKH*	2'858	667	1'623	2'475	770	-1.684	0.039616189	0.996112468
ENSBTAG00000022902	*RPL17*	3'028	6'989	5'346	2'622	8'070	1.622	0.039556352	0.996112468
ENSBTAG00000019175	*BT.100127*	959	198	530	831	229	-1.861	0.039817819	0.996774438
ENSBTAG00000031723	*RPL6*	11'632	26'432	20'297	10'074	30'521	1.599	0.039813447	0.996774438
ENSBTAG00000000284	*NAALAD2*	187	539	392	162	622	1.942	0.040229645	0.999851874
ENSBTAG00000010020	*pseudogene*	483	1'216	911	418	1'404	1.747	0.040123361	0.999851874
ENSBTAG00000010228	*BT.84674*	44	195	132	38	225	2.563	0.04009014	0.999851874
ENSBTAG00000019543	*TCEB2*	1'032	2'457	1'865	894	2'837	1.666	0.040248383	0.999851874
ENSBTAG00000020817	*BT.37460*	94	3	42	81	3	-4.555	0.040132526	0.999851874
ENSBTAG00000021420	*EPHA7*	294	40	150	255	46	-2.463	0.040150199	0.999851874
ENSBTAG00000000005	*ADRBK2*	294	188	236	255	217	-0.230	8.25E-01	1.00E+00
ENSBTAG00000000008	*KCNJ1*	0	1	1	0	1	Inf	9.94E-01	1.00E+00
ENSBTAG00000000009	*FOXF1*	56	11	31	48	13	-1.933	3.97E-01	1.00E+00
ENSBTAG00000000010	*BMSC-UBP*	1'249	1'572	1'448	1'082	1'815	0.747	3.55E-01	1.00E+00
ENSBTAG00000000011	*TDH*	36	13	23	31	15	-1.054	6.94E-01	1.00E+00
ENSBTAG00000000012	*TTC33*	331	219	270	287	253	-0.181	8.59E-01	1.00E+00

Ensembl gene ID	geneName	counts wildtype horn bud	counts polled horn bud	baseMean	baseMean wildtype horn bud	baseMean polled horn bud	log2FoldChange	pval	padj
ENSBTAG00000000013	*BT.78334*	2'471	1'492	1'931	2'140	1'723	-0.313	6.94E-01	1.00E+00
ENSBTAG00000000014	*TXN2*	2'317	2'443	2'414	2'007	2'821	0.491	5.32E-01	1.00E+00
ENSBTAG00000000015	*FOXRED2*	119	325	239	103	375	1.865	7.26E-02	1.00E+00
ENSBTAG00000000016	*LTA*	23	8	15	20	9	-1.109	7.61E-01	1.00E+00
ENSBTAG00000000019	*BT.65236*	6'393	3'845	4'988	5'537	4'440	-0.318	6.81E-01	1.00E+00
ENSBTAG00000000020	*BT.46779*	68	24	43	59	28	-1.087	5.64E-01	1.00E+00
ENSBTAG00000000021	*CCDC53*	828	491	642	717	567	-0.339	6.95E-01	1.00E+00
ENSBTAG00000000022	*NUP37*	849	525	671	735	606	-0.278	7.47E-01	1.00E+00
ENSBTAG00000000023	*RNF2*	3'067	2'671	2'870	2'656	3'084	0.216	7.82E-01	1.00E+00
ENSBTAG00000000024	*DYM*	2'549	2'559	2'581	2'207	2'955	0.421	5.92E-01	1.00E+00
ENSBTAG00000000025	*BT.51816*	8'179	4'363	6'061	7'083	5'038	-0.492	5.24E-01	1.00E+00
ENSBTAG00000000026	*VPS33B*	1'586	1'381	1'484	1'374	1'595	0.215	7.88E-01	1.00E+00
ENSBTAG00000000027	*IGFL1*	2	0	1	2	0		9.75E-01	1.00E+00
ENSBTAG00000000029	*BT.59488*	6	1	3	5	1	-2.170	8.85E-01	1.00E+00
ENSBTAG00000000030	*RDM1*	248	188	216	215	217	0.015	9.93E-01	1.00E+00
ENSBTAG00000000031	*TRPV4*	827	1'006	939	716	1'162	0.698	4.01E-01	1.00E+00
ENSBTAG00000000037	*BT.37259*	1'073	567	792	929	655	-0.505	5.50E-01	1.00E+00
ENSBTAG00000000039	*SIRT7*	554	463	507	480	535	0.156	8.61E-01	1.00E+00
ENSBTAG00000000040	*MAFG*	262	318	297	227	367	0.694	4.74E-01	1.00E+00
ENSBTAG00000000042	*PYCR1*	11'537	11'084	11'395	9'991	12'799	0.357	6.40E-01	1.00E+00
ENSBTAG00000000044	*BT.48803*	1	34	20	1	39	5.503	1.20E-01	1.00E+00
ENSBTAG00000000046	*SURF2*	586	254	400	507	293	-0.791	3.91E-01	1.00E+00
ENSBTAG00000000049	*CCDC77*	1'236	595	879	1'070	687	-0.640	4.44E-01	1.00E+00
ENSBTAG00000000050	*PSMG3*	1'391	1'158	1'271	1'205	1'337	0.151	8.53E-01	1.00E+00
ENSBTAG00000000052	*BT.88764*	325	614	495	281	709	1.333	1.38E-01	1.00E+00
ENSBTAG00000000053	*FILIP1*	411	304	353	356	351	-0.020	9.87E-01	1.00E+00
ENSBTAG00000000054	*SNAPC4*	2'388	2'753	2'623	2'068	3'179	0.620	4.30E-01	1.00E+00
ENSBTAG00000000056	*STRADA*	2'360	2'164	2'271	2'044	2'499	0.290	7.12E-01	1.00E+00
ENSBTAG00000000057	*THBS3*	7'697	7'839	7'859	6'666	9'052	0.441	5.65E-01	1.00E+00
ENSBTAG00000000062	*BT.61705*	230	249	243	199	288	0.530	6.01E-01	1.00E+00
ENSBTAG00000000064	*FEN1*	741	800	783	642	924	0.526	5.33E-01	1.00E+00
ENSBTAG00000000065	*CRLS1*	1'719	725	1'163	1'489	837	-0.830	3.11E-01	1.00E+00
ENSBTAG00000000066	*LRRN4*	4	0	2	3	0		8.95E-01	1.00E+00
ENSBTAG00000000067	*SDF2L1*	165	149	157	143	172	0.268	8.16E-01	1.00E+00
ENSBTAG00000000072	*TFB2M*	1'146	697	899	992	805	-0.302	7.18E-01	1.00E+00
ENSBTAG00000000073	*BT.87657*	3'302	3'374	3'378	2'860	3'896	0.446	5.66E-01	1.00E+00
ENSBTAG00000000074	*NFIA*	616	723	684	533	835	0.646	4.50E-01	1.00E+00
ENSBTAG00000000076	*RECK*	4'195	3'014	3'557	3'633	3'480	-0.062	9.37E-01	1.00E+00
ENSBTAG00000000077	*ADSL*	2'774	2'478	2'632	2'402	2'861	0.252	7.47E-01	1
ENSBTAG00000000078	*GLIPR2*	3'941	5'957	5'146	3'413	6'879	1.011	1.93E-01	1
ENSBTAG00000000079	*C1ORF96*	385	218	293	333	252	-0.405	6.79E-01	1
ENSBTAG00000000080	*GRWD1*	872	1'479	1'231	755	1'708	1.177	1.51E-01	1
ENSBTAG00000000081	*COL4A3BP*	999	718	847	865	829	-0.061	9.44E-01	1
ENSBTAG00000000082	*BT.62324*	3	3	3	3	3	0.415	1.00E+00	1
ENSBTAG00000000084	*MRPS30*	644	487	560	558	562	0.012	9.91E-01	1
ENSBTAG00000000087	*HSD17B12*	3'682	1'890	2'686	3'189	2'182	-0.547	4.86E-01	1
ENSBTAG00000000088	*ALCAM*	2'099	2'094	2'118	1'818	2'418	0.412	6.02E-01	1
ENSBTAG00000000090	*SEC31B*	899	636	756	779	734	-0.084	9.23E-01	1
ENSBTAG00000000091	*NDUFB8*	1'494	1'249	1'368	1'294	1'442	0.157	8.46E-01	1
ENSBTAG00000000092	*HIF1AN*	1'502	1'734	1'652	1'301	2'002	0.622	4.37E-01	1
ENSBTAG00000000094	*ATPAF1*	772	519	634	669	599	-0.158	8.57E-01	1
ENSBTAG00000000095	*PD-L1*	14	4	8	12	5	-1.392	8.04E-01	1
ENSBTAG00000000097	*KIAA0494*	1'509	934	1'193	1'307	1'078	-0.277	7.35E-01	1
ENSBTAG00000000098	*SETDB1*	3'160	2'618	2'880	2'737	3'023	0.144	8.54E-01	1
ENSBTAG00000000099	*CERS2*	7'954	6'454	7'170	6'888	7'452	0.114	8.82E-01	1
ENSBTAG00000000100	*pseudogene*	1	0	0	1	0		1.00E+00	1
ENSBTAG00000000102	*GPR75*	166	21	84	144	24	-2.568	8.01E-02	1
ENSBTAG00000000103	*MRPL23*	347	282	313	301	326	0.116	9.06E-01	1
ENSBTAG00000000105	*RIT1*	7'336	5'332	6'255	6'353	6'157	-0.045	9.54E-01	1
ENSBTAG00000000106	*FAM69C*	744	515	619	644	595	-0.116	8.96E-01	1
ENSBTAG00000000108	*KIAA0907*	4'559	2'727	3'549	3'948	3'149	-0.326	6.75E-01	1
ENSBTAG00000000109	*NPTXR*	142	311	241	123	359	1.546	1.32E-01	1
ENSBTAG00000000111	*UGP2*	6'221	5'871	6'083	5'388	6'779	0.331	6.66E-01	1
ENSBTAG00000000113	*BT.100854*	4'446	5'405	5'046	3'850	6'241	0.697	3.68E-01	1
ENSBTAG00000000115	*VPS54*	2'030	1'042	1'481	1'758	1'203	-0.547	4.97E-01	1
ENSBTAG00000000120	*BT.24733*	133	119	126	115	137	0.255	8.37E-01	1
ENSBTAG00000000123	*HAO2*	3	1	2	3	1	-1.170	9.85E-01	1
ENSBTAG00000000124	*DHH*	105	41	69	91	47	-0.942	5.36E-01	1
ENSBTAG00000000125	*BT.95516*	461	510	494	399	589	0.561	5.28E-01	1
ENSBTAG00000000128	*BT.62474*	306	114	198	265	132	-1.009	3.45E-01	1
ENSBTAG00000000130	*TNFSF13*	156	115	134	135	133	-0.025	9.91E-01	1
ENSBTAG00000000131	*SENP3*	2'403	2'703	2'601	2'081	3'121	0.585	4.56E-01	1

Ensembl gene ID	geneName	counts wildtype horn bud	counts polled horn bud	baseMean	baseMean wildtype horn bud	baseMean polled horn bud	log2FoldChange	pval	padj
ENSBTAG00000000132	*EIF4A1*	21'732	20'101	21'016	18'820	23'211	0.302	6.91E-01	1
ENSBTAG00000000133	*CD68*	1'061	499	748	919	576	-0.673	4.28E-01	1
ENSBTAG00000000134	*MPDU1*	2'050	1'911	1'991	1'775	2'207	0.314	6.92E-01	1
ENSBTAG00000000137	*FRYL*	1'580	823	1'159	1'368	950	-0.526	5.20E-01	1
ENSBTAG00000000138	*C18ORF34*	4	2	3	3	2	-0.585	9.96E-01	1
ENSBTAG00000000139	*BT.65045*	1'970	1'509	1'724	1'706	1'742	0.030	9.70E-01	1
ENSBTAG00000000140	*EPHX1*	1'784	2'244	2'068	1'545	2'591	0.746	3.47E-01	1
ENSBTAG00000000141	*NFU1*	1'380	876	1'103	1'195	1'012	-0.241	7.70E-01	1
ENSBTAG00000000144	*BT.90060*	40	4	20	35	5	-2.907	3.44E-01	1
ENSBTAG00000000146	*FARP1*	5'610	8'754	7'483	4'858	10'108	1.057	1.72E-01	1
ENSBTAG00000000147	*RIC8B*	869	628	739	753	725	-0.054	9.52E-01	1
ENSBTAG00000000149	*BT.44648*	1'700	1'240	1'452	1'472	1'432	-0.040	9.62E-01	1
ENSBTAG00000000152	*RANBP2*	5'987	3'723	4'742	5'185	4'299	-0.270	7.27E-01	1
ENSBTAG00000000153	*LRFN3*	374	584	499	324	674	1.058	2.36E-01	1
ENSBTAG00000000154	*IRF2BP1*	1'099	1'482	1'332	952	1'711	0.846	2.97E-01	1
ENSBTAG00000000156	*LGALSL*	609	474	537	527	547	0.053	9.53E-01	1
ENSBTAG00000000157	*CCDC138*	574	343	447	497	396	-0.328	7.18E-01	1
ENSBTAG00000000158	*LY6K*	8	5	6	7	6	-0.263	9.97E-01	1
ENSBTAG00000000160	*CBS*	1'140	1'656	1'450	987	1'912	0.954	2.39E-01	1
ENSBTAG00000000161	*TMEM40*	159	25	83	138	29	-2.254	1.21E-01	1
ENSBTAG00000000162	*ANAPC16*	3'000	1'897	2'394	2'598	2'190	-0.246	7.55E-01	1
ENSBTAG00000000163	*DDIT4*	3'853	1'717	2'660	3'337	1'983	-0.751	3.40E-01	1
ENSBTAG00000000164	*GNRH1*	17	7	11	15	8	-0.865	8.49E-01	1
ENSBTAG00000000169	*ASZ1*	19	3	10	16	3	-2.248	6.40E-01	1
ENSBTAG00000000170	*GSTT4*	51	212	144	44	245	2.471	4.10E-02	1
ENSBTAG00000000172	*PFKFB1*	1	3	2	1	3	2.000	9.34E-01	1
ENSBTAG00000000175	*N4BP2*	1'342	608	932	1'162	702	-0.727	3.83E-01	1
ENSBTAG00000000176	*SP3*	5'318	3'192	4'146	4'606	3'686	-0.321	6.79E-01	1
ENSBTAG00000000177	*MSLN*	113	37	70	98	43	-1.196	4.29E-01	1
ENSBTAG00000000179	*RPUSD1*	308	428	380	267	494	0.890	3.38E-01	1
ENSBTAG00000000181	*SUSD3*	59	31	43	51	36	-0.513	7.91E-01	1
ENSBTAG00000000182	*SPINT2*	6'544	6'204	6'416	5'667	7'164	0.338	6.60E-01	1
ENSBTAG00000000183	*BT.53544*	23	1	11	20	1	-4.109	4.22E-01	1
ENSBTAG00000000184	*EIF2AK3*	3'845	1'922	2'775	3'330	2'219	-0.585	4.56E-01	1
ENSBTAG00000000185	*SLC6A14*	244	51	135	211	59	-1.843	1.28E-01	1
ENSBTAG00000000186	*FLAD1*	892	823	861	772	950	0.299	7.20E-01	1
ENSBTAG00000000188	*HS3ST5*	139	30	78	120	35	-1.797	2.22E-01	1
ENSBTAG00000000191	*SLC25A20*	1'291	1'134	1'214	1'118	1'309	0.228	7.79E-01	1
ENSBTAG00000000195	*protein_coding*	717	350	513	621	404	-0.620	4.85E-01	1
ENSBTAG00000000197	*RG9MTD2*	243	248	248	210	286	0.444	6.59E-01	1
ENSBTAG00000000198	*protein_coding*	0	1	1	0	1	Inf	0.993540919	1
ENSBTAG00000000199	*PDP1*	844	896	883	731	1'035	0.501	0.547525774	1
ENSBTAG00000000201	*BT.97325*	1'368	743	1'021	1'185	858	-0.466	0.572931703	1
ENSBTAG00000000202	*SLC25A19*	587	621	613	508	717	0.496	0.566177295	1
ENSBTAG00000000203	*ATP6V1G1*	8'041	6'983	7'513	6'964	8'063	0.212	0.782137494	1
ENSBTAG00000000204	*C8H9orf91*	1'073	997	1'040	929	1'151	0.309	0.706885671	1
ENSBTAG00000000205	*BT.52802*	2'199	1'279	1'691	1'904	1'477	-0.367	0.646728708	1
ENSBTAG00000000207	*HDHD1A*	676	537	603	585	620	0.083	0.92487498	1
ENSBTAG00000000210	*ARHGAP31*	1'882	2'643	2'341	1'630	3'052	0.905	0.252854525	1
ENSBTAG00000000211	*SHROOM2*	359	387	379	311	447	0.523	0.572129683	1
ENSBTAG00000000212	*BT.44647*	2'687	3'764	3'337	2'327	4'346	0.901	0.249618209	1
ENSBTAG00000000213	*TMEM39A*	3'318	1'963	2'570	2'873	2'267	-0.342	0.663511182	1
ENSBTAG00000000214	*protein_coding*	9	3	6	8	3	-1.170	0.890813994	1
ENSBTAG00000000215	*GNB1*	18'951	18'471	18'870	16'412	21'328	0.378	0.619271826	1
ENSBTAG00000000218	*MAP2K5*	1'034	655	826	895	756	-0.244	0.772843047	1
ENSBTAG00000000219	*GRIN2B*	9	4	6	8	5	-0.755	0.931866093	1
ENSBTAG00000000220	*NEK10*	50	25	36	43	29	-0.585	0.783230761	1
ENSBTAG00000000221	*RBM34*	423	223	312	366	257	-0.509	0.597745385	1
ENSBTAG00000000222	*ARID4B*	2'171	1'255	1'665	1'880	1'449	-0.376	0.639061312	1
ENSBTAG00000000223	*PPM1B*	2'952	1'303	2'031	2'557	1'505	-0.765	0.336155022	1
ENSBTAG00000000224	*TSPAN15*	635	309	453	550	357	-0.624	0.489668534	1
ENSBTAG00000000225	*POLB*	1'131	778	939	979	898	-0.125	0.882176961	1
ENSBTAG00000000231	*BT.48514*	5'199	3'604	4'332	4'502	4'162	-0.114	0.884142002	1
ENSBTAG00000000232	*OR51D1*	14	2	7	12	2	-2.392	0.704665469	1
ENSBTAG00000000233	*OR51E1*	23	21	22	20	24	0.284	0.93047699	1
ENSBTAG00000000236	*YWHAZ*	814	1'283	1'093	705	1'481	1.071	0.194396961	1
ENSBTAG00000000237	*BT.105896*	5	0	2	4	0		0.853268594	1
ENSBTAG00000000240	*AKAP7*	682	302	470	591	349	-0.760	0.397949472	1
ENSBTAG00000000241	*STAU1*	3'771	3'706	3'773	3'266	4'279	0.390	0.615054039	1
ENSBTAG00000000242	*processed_pseudogene*	2'346	1'894	2'109	2'032	2'187	0.106	0.892803647	1
ENSBTAG00000000243	*HEBP2*	2'458	2'327	2'408	2'129	2'687	0.336	0.668739788	1
ENSBTAG00000000244	*NRXN2*	1'977	561	1'180	1'712	648	-1.402	0.090300163	1

Ensembl gene ID	geneName	counts wildtype horn bud	counts polled horn bud	baseMean	baseMean wildtype horn bud	baseMean polled horn bud	log2FoldChange	pval	padj
ENSBTAG00000000245	*NHSL1*	577	418	491	500	483	-0.050	0.958342003	1
ENSBTAG00000000246	*BT.24594*	163	20	82	141	23	-2.612	0.078151099	1
ENSBTAG00000000250	*HDHD3*	533	466	500	462	538	0.221	0.803901072	1
ENSBTAG00000000251	*ALAD*	1'819	1'304	1'541	1'575	1'506	-0.065	0.936707184	1
ENSBTAG00000000252	*POLE3*	3'936	4'579	4'348	3'409	5'287	0.633	0.413885526	1
ENSBTAG00000000256	*TLE1*	1'768	1'176	1'445	1'531	1'358	-0.173	0.830827386	1
ENSBTAG00000000257	*MRAP2*	262	101	172	227	117	-0.960	0.386584607	1
ENSBTAG00000000258	*BT.28912*	238	77	148	206	89	-1.213	0.296617022	1
ENSBTAG00000000260	*ZNRF2*	298	122	199	258	141	-0.873	0.412213242	1
ENSBTAG00000000261	*C11H2orf84*	7	4	5	6	5	-0.392	0.98714753	1
ENSBTAG00000000264	*RFX6*	0	2	1	0	2	Inf	0.939077559	1
ENSBTAG00000000266	*NAB1*	3'475	1'561	2'406	3'009	1'802	-0.740	0.349131427	1
ENSBTAG00000000267	*SMG6*	1'398	2'510	2'055	1'211	2'898	1.259	0.115836623	1
ENSBTAG00000000269	*BT.87667*	851	320	553	737	370	-0.996	0.258786412	1
ENSBTAG00000000271	*BT.51770*	396	136	250	343	157	-1.127	0.265736207	1
ENSBTAG00000000274	*YKT6*	2'243	2'839	2'610	1'942	3'278	0.755	0.337189145	1
ENSBTAG00000000275	*PARP11*	772	784	787	669	905	0.437	0.603542437	1
ENSBTAG00000000277	*IL18*	1'079	524	770	934	605	-0.627	0.459059383	1
ENSBTAG00000000278	*RNF17*	1	0	0	1	0		1	1
ENSBTAG00000000279	*RAB26*	632	282	436	547	326	-0.749	0.409868253	1
ENSBTAG00000000281	*BT.30108*	330	246	285	286	284	-0.009	0.997847908	1
ENSBTAG00000000283	*CSF1*	562	524	546	487	605	0.314	0.720646716	1
ENSBTAG00000000285	*GUF1*	1'428	630	982	1'237	727	-0.766	0.356199036	1
ENSBTAG00000000286	*PFKM*	3'509	3'926	3'786	3'039	4'533	0.577	0.457636892	1
ENSBTAG00000000287	*GNPDA2*	1'580	822	1'159	1'368	949	-0.528	0.519032485	1
ENSBTAG00000000288	*UPF2*	2'834	1'568	2'132	2'454	1'811	-0.439	0.579434295	1
ENSBTAG00000000289	*ASB8*	2'062	1'518	1'769	1'786	1'753	-0.027	0.974587173	1
ENSBTAG00000000290	*ATP11B*	1'780	1'136	1'427	1'542	1'312	-0.233	0.773552999	1
ENSBTAG00000000291	*DHTKD1*	1'525	933	1'199	1'321	1'077	-0.294	0.719245628	1
ENSBTAG00000000292	*TCIRG1*	2'304	1'371	1'789	1'995	1'583	-0.334	0.675764128	1
ENSBTAG00000000295	*MGC139228*	2'140	1'400	1'735	1'853	1'617	-0.197	0.80571213	1
ENSBTAG00000000296	*IMPDH1*	1'845	2'593	2'296	1'598	2'994	0.906	0.252623292	1
ENSBTAG00000000297	*ZNF330*	1'151	681	892	997	786	-0.342	0.682371233	1
ENSBTAG00000000300	*KIAA2022*	37	14	24	32	16	-0.987	0.706068506	1
ENSBTAG00000000301	*protein_coding*	2	5	4	2	6	1.737	0.883335552	1
ENSBTAG00000000305	*LMO4*	4'183	3'794	4'002	3'623	4'381	0.274	0.722982484	1
ENSBTAG00000000306	*ITK*	31	20	25	27	23	-0.217	0.94785322	1
ENSBTAG00000000308	*ITCH*	5'904	2'729	4'132	5'113	3'151	-0.698	0.369292797	1
ENSBTAG00000000309	*PTPRT*	100	163	137	87	188	1.120	0.343043775	1
ENSBTAG00000000312	*GRINA*	2'396	3'086	2'819	2'075	3'563	0.780	0.320347993	1
ENSBTAG00000000313	*NUDT13*	268	137	195	232	158	-0.553	0.6060623	1
ENSBTAG00000000315	*ECD*	1'699	1'419	1'555	1'471	1'639	0.155	0.846283591	1
ENSBTAG00000000317	*BT.105291*	1'004	519	734	869	599	-0.537	0.527924456	1
ENSBTAG00000000320	*UBAP1*	1'426	1'348	1'396	1'235	1'557	0.334	0.678543013	1
ENSBTAG00000000321	*TBL2*	1'489	1'082	1'269	1'290	1'249	-0.046	0.95686849	1
ENSBTAG00000000322	*CD2AP*	1'761	1'056	1'372	1'525	1'219	-0.323	0.69026261	1
ENSBTAG00000000326	*ENTHD1*	2	0	1	2	0		0.974934741	1
ENSBTAG00000000328	*TPPP2*	18	12	15	16	14	-0.170	0.983826208	1
ENSBTAG00000000330	*BT.103955*	826	835	840	715	964	0.431	0.606834129	1
ENSBTAG00000000332	*ERF*	1'888	2'521	2'273	1'635	2'911	0.832	0.292986205	1
ENSBTAG00000000333	*ARHGAP20*	161	202	186	139	233	0.742	0.491640122	1
ENSBTAG00000000336	*BT.93633*	2	0	1	2	0		0.974934741	1
ENSBTAG00000000340	*bta-mir-6527*	7'892	5'214	6'428	6'835	6'021	-0.183	0.812555468	1
ENSBTAG00000000341	*ISOC1*	939	772	852	813	891	0.133	0.874468498	1
ENSBTAG00000000342	*PLOD3*	4'256	5'709	5'139	3'686	6'592	0.839	0.279133348	1
ENSBTAG00000000343	*ZNHIT1*	921	782	850	798	903	0.179	0.830739264	1
ENSBTAG00000000345	*CLDN15*	181	103	138	157	119	-0.398	0.738823647	1
ENSBTAG00000000347	*RHOG*	886	1'260	1'111	767	1'455	0.923	0.261778951	1
ENSBTAG00000000354	*PDE6G*	4	10	8	3	12	1.737	0.768040958	1
ENSBTAG00000000355	*OXLD1*	246	153	195	213	177	-0.270	0.804002146	1
ENSBTAG00000000356	*CCDC137*	593	616	612	514	711	0.470	0.586957177	1
ENSBTAG00000000357	*BT.45696*	777	1'574	1'245	673	1'817	1.433	0.082288623	1
ENSBTAG00000000359	*BT.63473*	2'038	1'539	1'771	1'765	1'777	0.010	0.990224969	1
ENSBTAG00000000362	*SPG11*	2'415	1'430	1'871	2'091	1'651	-0.341	0.668607535	1
ENSBTAG00000000363	*BDP1*	927	603	750	803	696	-0.205	0.809956305	1
ENSBTAG00000000365	*TDP2*	1'195	778	967	1'035	898	-0.204	0.806511187	1
ENSBTAG00000000369	*EPS8*	1'933	1'643	1'786	1'674	1'897	0.181	0.820310341	1
ENSBTAG00000000371	*DIS3L*	6'561	4'110	5'214	5'682	4'746	-0.260	0.736941419	1
ENSBTAG00000000372	*TIPIN*	1'319	851	1'062	1'142	983	-0.217	0.792722248	1
ENSBTAG00000000374	*LRRTM4*	52	8	27	45	9	-2.285	0.354195015	1
ENSBTAG00000000375	*SSRP1*	7'796	10'147	9'234	6'752	11'717	0.795	0.300834184	1
ENSBTAG00000000376	*P2RX3*	109	139	127	94	161	0.766	0.525602337	1

Ensembl gene ID	geneName	counts wildtype horn bud	counts polled horn bud	baseMean	baseMean wildtype horn bud	baseMean polled horn bud	log2FoldChange	pval	padj
ENSBTAG00000000377	*BMX*	98	26	57	85	30	-1.499	0.365394447	1
ENSBTAG00000000379	*GOLT1B*	1'674	1'103	1'362	1'450	1'274	-0.187	0.818267507	1
ENSBTAG00000000380	*C5H12orf39*	369	245	301	320	283	-0.176	0.859072905	1
ENSBTAG00000000381	*S1PR4*	2	1	1	2	1	-0.585	1	1
ENSBTAG00000000382	*MED27*	1'024	1'498	1'308	887	1'730	0.964	0.23657776	1
ENSBTAG00000000385	*ZNF238*	2'072	992	1'470	1'794	1'145	-0.648	0.422166452	1
ENSBTAG00000000386	*pseudogene*	2'098	2'698	2'466	1'817	3'115	0.778	0.323800945	1
ENSBTAG00000000387	*NICN1*	4'537	3'508	3'990	3'929	4'051	0.044	0.954412622	1
ENSBTAG00000000389	*ZNF212*	460	640	569	398	739	0.891	0.30887255	1
ENSBTAG00000000390	*TPST1*	6'059	5'652	5'887	5'247	6'526	0.315	0.682099101	1
ENSBTAG00000000392	*ASCC1*	2'432	820	1'527	2'106	947	-1.153	0.154868343	1
ENSBTAG00000000393	*SSPN*	162	85	119	140	98	-0.515	0.679204417	1
ENSBTAG00000000394	*BT.70619*	1'638	767	1'152	1'419	886	-0.680	0.406838068	1
ENSBTAG00000000395	*ADAMTS20*	253	75	153	219	87	-1.339	0.245352067	1
ENSBTAG00000000396	*PIM1*	548	710	647	475	820	0.789	0.360056947	1
ENSBTAG00000000397	*CNPY1*	1	0	0	1	0		1	1
ENSBTAG00000000399	*NANOS3*	60	16	35	52	18	-1.492	0.477198184	1
ENSBTAG00000000400	*KRT85*	172	287	240	149	331	1.154	0.257706499	1
ENSBTAG00000000401	*TTC24*	4	6	5	3	7	1.000	0.91481797	1
ENSBTAG00000000404	*APOA1BP*	2'002	2'333	2'214	1'734	2'694	0.636	0.420943029	1
ENSBTAG00000000405	*VKORC1*	3'102	2'217	2'623	2'686	2'560	-0.070	0.930429108	1
ENSBTAG00000000406	*GPATCH4*	637	492	560	552	568	0.042	0.962796225	1
ENSBTAG00000000409	*BT.35848*	2'118	1'537	1'805	1'834	1'775	-0.048	0.95374309	1
ENSBTAG00000000411	*HGS*	3'201	4'323	3'882	2'772	4'992	0.849	0.276265194	1
ENSBTAG00000000413	*BT.38701*	69	103	89	60	119	0.993	0.467233723	1
ENSBTAG00000000415	*ERBB2IP*	5'799	3'741	4'671	5'022	4'320	-0.217	0.779212453	1
ENSBTAG00000000417	*MRPL12*	1'252	1'672	1'507	1'084	1'931	0.832	0.30176847	1
ENSBTAG00000000418	*TOX3*	118	50	80	102	58	-0.824	0.566008323	1
ENSBTAG00000000421	*EEA1*	1'147	300	670	993	346	-1.520	0.081302353	1
ENSBTAG00000000422	*TSHZ1*	923	608	751	799	702	-0.187	0.826657336	1
ENSBTAG00000000423	*RBM4B*	2'732	1'632	2'125	2'366	1'884	-0.328	0.678606032	1
ENSBTAG00000000425	*RPL17*	71'851	81'498	78'165	62'225	94'106	0.597	0.432305651	1
ENSBTAG00000000428	*BAK1*	346	541	462	300	625	1.060	0.240516653	1
ENSBTAG00000000429	*BT.56883*	3'533	4'594	4'182	3'060	5'305	0.794	0.307198236	1
ENSBTAG00000000431	*TRD@*	64	9	33	55	10	-2.415	0.27905174	1
ENSBTAG00000000432	*BT.104278*	111	66	86	96	76	-0.335	0.815203229	1
ENSBTAG00000000434	*CRYAB*	659	669	672	571	772	0.437	0.609860773	1
ENSBTAG00000000435	*TARBP2*	1'919	1'640	1'778	1'662	1'894	0.188	0.812677948	1
ENSBTAG00000000436	*TNFAIP3*	192	128	157	166	148	-0.170	0.88631837	1
ENSBTAG00000000437	*O3FAR1*	3	0	1	3	0		0.936647693	1
ENSBTAG00000000439	*SFSWAP*	2'939	2'064	2'464	2'545	2'383	-0.095	0.905010276	1
ENSBTAG00000000441	*MCART6*	28	19	23	24	22	-0.144	0.975115816	1
ENSBTAG00000000445	*PDE6C*	88	15	47	76	17	-2.138	0.248403497	1
ENSBTAG00000000446	*ATP11A*	903	1'035	989	782	1'195	0.612	0.459042515	1
ENSBTAG00000000447	*C26H10ORF4*	1'526	605	1'010	1'322	699	-0.920	0.267521196	1
ENSBTAG00000000448	*BDH1*	605	256	410	524	296	-0.826	0.368701716	1
ENSBTAG00000000451	*TMEM126A*	389	470	440	337	543	0.688	0.447291329	1
ENSBTAG00000000454	*TMEM106C*	2'587	1'720	2'113	2'240	1'986	-0.174	0.826890632	1
ENSBTAG00000000455	*CREBZF*	7'742	2'810	4'975	6'705	3'245	-1.047	0.178791325	1
ENSBTAG00000000456	*PCPB*	3	0	1	3	0		0.936647693	1
ENSBTAG00000000457	*CCDC89*	8	0	3	7	0		0.72953145	1
ENSBTAG00000000458	*DYNC1LI2*	1'709	1'247	1'460	1'480	1'440	-0.040	0.962272605	1
ENSBTAG00000000459	*GTDC2*	1'020	851	933	883	983	0.154	0.853241947	1
ENSBTAG00000000462	*CCDC102A*	2'853	2'794	2'849	2'471	3'226	0.385	0.622259709	1
ENSBTAG00000000469	*PPP2CA*	7'535	5'349	6'351	6'526	6'176	-0.079	0.918657404	1
ENSBTAG00000000472	*ZNF570*	556	208	361	482	240	-1.003	0.285846142	1
ENSBTAG00000000473	*ATP10D*	1'286	789	1'012	1'114	911	-0.290	0.72632906	1
ENSBTAG00000000475	*IMMP1L*	453	382	417	392	441	0.169	0.854163817	1
ENSBTAG00000000476	*THPO*	480	316	390	416	365	-0.188	0.841111708	1
ENSBTAG00000000477	*TLR5*	604	227	393	523	262	-0.997	0.282156551	1
ENSBTAG00000000478	*C3ORF45*	45	73	62	39	84	1.113	0.483324034	1
ENSBTAG00000000480	*IFRD2*	953	1'173	1'090	825	1'354	0.715	0.384164049	1
ENSBTAG00000000483	*HYAL1*	574	713	660	497	823	0.728	0.396900811	1
ENSBTAG00000000484	*HYAL2*	1'716	2'501	2'187	1'486	2'888	0.958	0.227448472	1
ENSBTAG00000000489	*WDR54*	226	186	205	196	215	0.134	0.90204231	1
ENSBTAG00000000490	*RTKN*	1'483	1'716	1'633	1'284	1'981	0.626	0.434545626	1
ENSBTAG00000000492	*BT.65379*	3'441	2'233	2'779	2'980	2'578	-0.209	0.790458817	1
ENSBTAG00000000494	*PDE4D*	491	437	465	425	505	0.247	0.78373742	1
ENSBTAG00000000495	*BT.31273*	1'297	772	1'007	1'123	891	-0.333	0.686980243	1
ENSBTAG00000000496	*SLC12A8*	50	11	28	43	13	-1.769	0.458754393	1
ENSBTAG00000000497	*AKR1A1*	3'267	2'867	3'070	2'829	3'311	0.227	0.770994778	1
ENSBTAG00000000500	*FLJ20565*	337	349	347	292	403	0.466	0.620760961	1

Ensembl gene ID	geneName	counts wildtype horn bud	counts polled horn bud	baseMean	baseMean wildtype horn bud	baseMean polled horn bud	log2FoldChange	pval	padj
ENSBTAG00000000501	*KLHL13*	2'224	913	1'490	1'926	1'054	-0.869	0.281966106	1
ENSBTAG00000000504	*BT.39806*	1'923	1'257	1'558	1'665	1'451	-0.198	0.805654052	1
ENSBTAG00000000505	*CCBL2*	897	660	769	777	762	-0.028	0.976342415	1
ENSBTAG00000000507	*NR4A1*	918	396	626	795	457	-0.798	0.357265544	1
ENSBTAG00000000510	*ATG101*	1'247	1'712	1'528	1'080	1'977	0.872	0.279137477	1
ENSBTAG00000000511	*BT.81882*	450	408	430	390	471	0.274	0.763584807	1
ENSBTAG00000000512	*SNTA1*	701	482	582	607	557	-0.125	0.887914204	1
ENSBTAG00000000516	*OTOR*	19'128	30'798	26'064	16'565	35'562	1.102	0.151293807	1
ENSBTAG00000000517	*BT.89962*	463	241	340	401	278	-0.527	0.578388863	1
ENSBTAG00000000518	*protein_coding*	6	0	3	5	0		0.81115604	1
ENSBTAG00000000520	*ATP2B3*	99	51	72	86	59	-0.542	0.719823412	1
ENSBTAG00000000521	*PLEKHA8*	176	121	146	152	140	-0.126	0.920037654	1
ENSBTAG00000000522	*AHSG*	122	82	100	106	95	-0.158	0.911819112	1
ENSBTAG00000000524	*CSTB*	2'166	2'110	2'156	1'876	2'436	0.377	0.632486146	1
ENSBTAG00000000526	*TOPBP1*	2'977	2'354	2'648	2'578	2'718	0.076	0.92218255	1
ENSBTAG00000000527	*ZFAND3*	3'265	3'013	3'153	2'828	3'479	0.299	0.700740512	1
ENSBTAG00000000528	*MANBAL*	1'159	1'326	1'267	1'004	1'531	0.609	0.453053938	1
ENSBTAG00000000529	*BT.46996*	400	545	488	346	629	0.861	0.335028636	1
ENSBTAG00000000531	*protein_coding*	3	0	1	3	0		0.936647693	1
ENSBTAG00000000532	*DEM1*	667	162	382	578	187	-1.627	0.084678917	1
ENSBTAG00000000533	*ZNF684*	98	34	62	85	39	-1.112	0.485313243	1
ENSBTAG00000000534	*PNOC*	43	67	57	37	77	1.055	0.519900961	1
ENSBTAG00000000536	*ZNF395*	861	1'366	1'161	746	1'577	1.081	0.188707894	1
ENSBTAG00000000539	*BT.34089*	2'102	1'713	1'899	1'820	1'978	0.120	0.879839758	1
ENSBTAG00000000540	*RNF112*	544	873	740	471	1'008	1.097	0.198714221	1
ENSBTAG00000000541	*BT.55796*	31	38	35	27	44	0.709	0.735625157	1
ENSBTAG00000000542	*CCDC104*	3'938	1'896	2'800	3'410	2'189	-0.639	0.415377624	1
ENSBTAG00000000545	*SMEK2*	3'904	2'351	3'048	3'381	2'715	-0.317	0.685719452	1
ENSBTAG00000000546	*TOB1*	2'125	1'540	1'809	1'840	1'778	-0.049	0.951780917	1
ENSBTAG00000000548	*KLHDC10*	1'259	662	927	1'090	764	-0.512	0.538214958	1
ENSBTAG00000000550	*ATP5D*	4'801	6'579	5'877	4'158	7'597	0.870	0.261083467	1
ENSBTAG00000000551	*SMNDC1*	1'371	747	1'025	1'187	863	-0.461	0.57661522	1
ENSBTAG00000000552	*DISP2*	43	1	19	37	1	-5.011	0.165386276	1
ENSBTAG00000000555	*BT.53789*	3'911	2'942	3'392	3'387	3'397	0.004	0.995349057	1
ENSBTAG00000000559	*PLTP*	8'325	2'888	5'272	7'210	3'335	-1.112	0.153294838	1
ENSBTAG00000000560	*protein_coding*	47'906	76'596	64'967	41'488	88'445	1.092	0.154144502	1
ENSBTAG00000000561	*OCLN*	537	615	588	465	710	0.611	0.482653044	1
ENSBTAG00000000562	*TOMM20*	5'572	3'611	4'498	4'825	4'170	-0.211	0.785918768	1
ENSBTAG00000000563	*BT.48995*	2'719	2'116	2'399	2'355	2'443	0.053	0.945783774	1
ENSBTAG00000000565	*RFX7*	4'082	2'301	3'096	3'535	2'657	-0.412	0.598283017	1
ENSBTAG00000000566	*BT.72734*	1'330	626	937	1'152	723	-0.672	0.419265237	1
ENSBTAG00000000568	*CCDC154*	22	14	18	19	16	-0.237	0.958766648	1
ENSBTAG00000000569	*HES1*	2'438	2'702	2'616	2'111	3'120	0.563	0.472804904	1
ENSBTAG00000000570	*RET*	35	72	57	30	83	1.456	0.378436665	1
ENSBTAG00000000571	*BT.76342*	538	548	549	466	633	0.442	0.614478927	1
ENSBTAG00000000573	*protein_coding*	26	42	36	23	48	1.107	0.592394873	1
ENSBTAG00000000576	*GPR64*	635	132	351	550	152	-1.851	0.054712231	1
ENSBTAG00000000578	*BAT5*	4'372	3'426	3'871	3'786	3'956	0.063	0.934595532	1
ENSBTAG00000000579	*NIPBL*	4'773	2'737	3'647	4'134	3'160	-0.387	0.618907091	1
ENSBTAG00000000580	*BT.43761*	31	51	43	27	59	1.133	0.546243212	1
ENSBTAG00000000581	*DOCK10*	1'573	549	998	1'362	634	-1.104	0.185066332	1
ENSBTAG00000000582	*LY6G6E*	1'858	1'768	1'825	1'609	2'042	0.343	0.66550817	1
ENSBTAG00000000584	*BT.56245*	3	3	3	3	3	0.415	1	1
ENSBTAG00000000585	*LY6G6C*	2'649	805	1'612	2'294	930	-1.303	0.108039496	1
ENSBTAG00000000586	*C5ORF42*	4'357	1'905	2'986	3'773	2'200	-0.779	0.321178539	1
ENSBTAG00000000589	*CACNG4*	214	188	201	185	217	0.228	0.831721055	1
ENSBTAG00000000593	*ST8SIA1*	36	2	17	31	2	-3.755	0.293536605	1
ENSBTAG00000000597	*TMPRSS15*	13	13	13	11	15	0.415	0.928893337	1
ENSBTAG00000000598	*CST3*	42'262	12'227	25'359	36'600	14'119	-1.374	0.075389446	1
ENSBTAG00000000599	*CCNI*	15'569	10'693	12'915	13'483	12'347	-0.127	0.868543743	1
ENSBTAG00000000602	*RXRB*	2'159	2'777	2'538	1'870	3'207	0.778	0.323153629	1
ENSBTAG00000000603	*JAM2*	3'408	1'177	2'155	2'951	1'359	-1.119	0.160700052	1
ENSBTAG00000000604	*BT.99652*	4'972	5'044	5'065	4'306	5'824	0.436	0.57202207	1
ENSBTAG00000000605	*ATP5J*	3'825	2'257	2'959	3'313	2'606	-0.346	0.658567831	1
ENSBTAG00000000606	*SYT10*	29	55	44	25	64	1.338	0.469277852	1
ENSBTAG00000000607	*ABCF2*	1'846	2'109	2'017	1'599	2'435	0.607	0.443666948	1
ENSBTAG00000000608	*PRCC*	2'442	2'767	2'655	2'115	3'195	0.595	0.448029008	1
ENSBTAG00000000611	*C25H16orf90*	1	0	0	1	0		1	1
ENSBTAG00000000613	*ELAVL1*	3'543	3'407	3'501	3'068	3'934	0.359	0.644299364	1
ENSBTAG00000000616	*BT.42704*	306	288	299	265	333	0.328	0.735818496	1
ENSBTAG00000000619	*BT.54544*	13	3	7	11	3	-1.700	0.783725709	1
ENSBTAG00000000620	*C8ORF45*	29	5	15	25	6	-2.121	0.541892844	1

Ensembl gene ID	geneName	counts wildtype horn bud	counts polled horn bud	baseMean	baseMean wildtype horn bud	baseMean polled horn bud	log2FoldChange	pval	padj
ENSBTAG00000000621	*CATSPERG*	1'122	713	897	972	823	-0.239	0.775331371	1
ENSBTAG00000000622	*RPS17*	77'853	57'385	66'843	67'423	66'262	-0.025	0.974357148	1
ENSBTAG00000000623	*CPEB1*	202	183	193	175	211	0.273	0.800962843	1
ENSBTAG00000000625	*SMAD6*	790	418	583	684	483	-0.503	0.564283083	1
ENSBTAG00000000626	*BT.45170*	25	9	16	22	10	-1.059	0.75675668	1
ENSBTAG00000000628	*protein_coding*	162	244	211	140	282	1.006	0.337226493	1
ENSBTAG00000000629	*MMS22L*	147	153	152	127	177	0.473	0.680102833	1
ENSBTAG00000000630	*BT.40231*	5'369	4'045	4'660	4'650	4'671	0.007	0.992898696	1
ENSBTAG00000000632	*RAD54B*	638	168	373	553	194	-1.510	0.10980983	1
ENSBTAG00000000634	*BT.29245*	176	198	191	152	229	0.585	0.585840005	1
ENSBTAG00000000638	*CDT1*	443	901	712	384	1'040	1.439	0.095598191	1
ENSBTAG00000000639	*APRT*	2'230	2'798	2'581	1'931	3'231	0.742	0.345406932	1
ENSBTAG00000000640	*VIMP*	2'851	1'817	2'284	2'469	2'098	-0.235	0.766494731	1
ENSBTAG00000000641	*SKAP1*	42	6	22	36	7	-2.392	0.397151043	1
ENSBTAG00000000642	*INTS1*	3'141	5'449	4'506	2'720	6'292	1.210	0.121986475	1
ENSBTAG00000000644	*S100A5*	31	10	19	27	12	-1.217	0.684942058	1
ENSBTAG00000000646	*ANAPC4*	3'087	1'296	2'085	2'673	1'496	-0.837	0.292387502	1
ENSBTAG00000000647	*SELO*	1'134	1'726	1'488	982	1'993	1.021	0.20700961	1
ENSBTAG00000000648	*ADAMTS5*	256	131	186	222	151	-0.552	0.611365887	1
ENSBTAG00000000650	*TUBGCP6*	2'229	2'842	2'606	1'930	3'282	0.766	0.33057604	1
ENSBTAG00000000653	*PPP1R16B*	685	371	511	593	428	-0.470	0.597096604	1
ENSBTAG00000000654	*ARMC4*	258	154	201	223	178	-0.329	0.759009987	1
ENSBTAG00000000655	*MIPOL1*	200	67	125	173	77	-1.163	0.341236084	1
ENSBTAG00000000656	*BT.45162*	1'302	1'025	1'156	1'128	1'184	0.070	0.932027905	1
ENSBTAG00000000658	*KIAA1875*	271	227	248	235	262	0.159	0.876601237	1
ENSBTAG00000000660	*BT.28502*	426	316	367	369	365	-0.016	0.990532961	1
ENSBTAG00000000662	*TCF20*	2'620	3'470	3'138	2'269	4'007	0.820	0.294822054	1
ENSBTAG00000000664	*SLC22A15*	36	22	28	31	25	-0.295	0.913717147	1
ENSBTAG00000000665	*BORX*	8	0	3	7	0		0.72953145	1
ENSBTAG00000000666	*GHDC*	953	494	698	825	570	-0.533	0.533180818	1
ENSBTAG00000000667	*APOL3*	22	37	31	19	43	1.165	0.60091505	1
ENSBTAG00000000668	*SLC22A5*	631	442	528	546	510	-0.099	0.913680559	1
ENSBTAG00000000670	*RRP9*	473	546	520	410	630	0.622	0.481383858	1
ENSBTAG00000000671	*PARP3*	431	159	278	373	184	-1.024	0.300140452	1
ENSBTAG00000000672	*MGC143209*	1'096	588	814	949	679	-0.483	0.566013274	1
ENSBTAG00000000675	*PGS1*	1'604	1'878	1'779	1'389	2'169	0.643	0.420336116	1
ENSBTAG00000000676	*BT.21872*	1'853	1'075	1'423	1'605	1'241	-0.370	0.646501073	1
ENSBTAG00000000678	*BT.67516*	19'696	15'189	17'298	17'057	17'539	0.040	0.957332664	1
ENSBTAG00000000679	*BT.57470*	2'845	1'562	2'134	2'464	1'804	-0.450	0.569857647	1
ENSBTAG00000000682	*RAPGEF6*	589	399	485	510	461	-0.147	0.871782014	1
ENSBTAG00000000683	*WDR37*	653	667	668	566	770	0.446	0.602844054	1
ENSBTAG00000000684	*ARHGEF10L*	3'030	4'349	3'823	2'624	5'022	0.936	0.230409612	1
ENSBTAG00000000686	*NANP*	632	478	550	547	552	0.012	0.990640787	1
ENSBTAG00000000687	*WDR51B*	1'000	691	832	866	798	-0.118	0.88973113	1
ENSBTAG00000000690	*THEM5*	1'842	2'395	2'180	1'595	2'766	0.794	0.316296775	1
ENSBTAG00000000693	*BT.105130*	1'566	1'526	1'559	1'356	1'762	0.378	0.637146089	1
ENSBTAG00000000694	*TAF10*	1'846	1'607	1'727	1'599	1'856	0.215	0.787101853	1
ENSBTAG00000000695	*UBAP2*	2'499	2'817	2'708	2'164	3'253	0.588	0.453405675	1
ENSBTAG00000000696	*ILK*	6'258	6'469	6'445	5'420	7'470	0.463	0.546977172	1
ENSBTAG00000000697	*RRP8*	129	133	133	112	154	0.459	0.70135571	1
ENSBTAG00000000698	*MYO18B*	206	427	336	178	493	1.467	0.126226159	1
ENSBTAG00000000699	*C9ORF3*	145	134	140	126	155	0.301	0.799788857	1
ENSBTAG00000000700	*BT.104140*	2'354	2'471	2'446	2'039	2'853	0.485	0.537116568	1
ENSBTAG00000000704	*GUSB*	3'012	2'163	2'553	2'608	2'498	-0.063	0.937513732	1
ENSBTAG00000000705	*COX7A2L*	5'616	6'129	5'970	4'864	7'077	0.541	0.482209184	1
ENSBTAG00000000706	*ADAMTS1*	2'954	1'658	2'236	2'558	1'914	-0.418	0.596751914	1
ENSBTAG00000000707	*WISP1*	1'515	396	885	1'312	457	-1.521	0.073161451	1
ENSBTAG00000000710	*PIP5K1C*	875	1'612	1'310	758	1'861	1.297	0.113735479	1
ENSBTAG00000000711	*NDRG1*	4'809	3'198	3'929	4'165	3'693	-0.174	0.823722921	1
ENSBTAG00000000712	*FBXW2*	1'423	1'144	1'277	1'232	1'321	0.100	0.901777902	1
ENSBTAG00000000713	*AGFG2*	847	615	722	734	710	-0.047	0.958572985	1
ENSBTAG00000000715	*BT.64082*	20	7	13	17	8	-1.100	0.786059398	1
ENSBTAG00000000717	*PPAP2C*	385	680	559	333	785	1.236	0.161820813	1
ENSBTAG00000000718	*TMPRSS9*	28	28	28	24	32	0.415	0.868606267	1
ENSBTAG00000000719	*BT.50255*	21	42	33	18	48	1.415	0.507898338	1
ENSBTAG00000000720	*CTSL1*	50	62	57	43	72	0.725	0.659181229	1
ENSBTAG00000000721	*GPATCH1*	836	798	823	724	921	0.348	0.678173779	1
ENSBTAG00000000725	*USP42*	824	600	703	714	693	-0.043	0.962584602	1
ENSBTAG00000000727	*RNF169*	599	529	565	519	611	0.236	0.787747856	1
ENSBTAG00000000728	*ZNF597*	2	3	3	2	3	1.000	0.97171833	1
ENSBTAG00000000730	*GCG*	6	9	8	5	10	1.000	0.869259965	1
ENSBTAG00000000731	*BT.31782*	256	73	153	222	84	-1.395	0.226579542	1

Ensembl gene ID	geneName	counts wildtype horn bud	counts polled horn bud	baseMean	baseMean wildtype horn bud	baseMean polled horn bud	log2FoldChange	pval	padj
ENSBTAG00000000735	*BCL2A1*	22	2	11	19	2	-3.044	0.517475779	1
ENSBTAG00000000736	*LRP8*	433	86	237	375	99	-1.917	0.066581677	1
ENSBTAG00000000737	*DMXL2*	81	55	67	70	64	-0.143	0.936063372	1
ENSBTAG00000000738	*DAPK1*	1'265	677	939	1'096	782	-0.487	0.558203687	1
ENSBTAG00000000742	*BT.24748*	2'770	2'467	2'624	2'399	2'849	0.248	0.751386687	1
ENSBTAG00000000743	*MBOAT4*	2	0	1	2	0		0.974934741	1
ENSBTAG00000000744	*DCTN6*	1'517	1'281	1'396	1'314	1'479	0.171	0.831846043	1
ENSBTAG00000000745	*AQP1*	7'593	10'802	9'524	6'576	12'473	0.924	0.230372662	1
ENSBTAG00000000746	*BT.6431*	2'139	931	1'464	1'852	1'075	-0.785	0.331275774	1
ENSBTAG00000000748	*GAR1*	1'075	769	909	931	888	-0.068	0.936586085	1
ENSBTAG00000000749	*IFLTD1*	8	10	9	7	12	0.737	0.893887116	1
ENSBTAG00000000750	*MSL2*	941	654	785	815	755	-0.110	0.898274023	1
ENSBTAG00000000751	*RRH*	19	3	10	16	3	-2.248	0.640043503	1
ENSBTAG00000000752	*BT.28541*	409	423	421	354	488	0.464	0.610694434	1
ENSBTAG00000000753	*PIAS4*	649	946	827	562	1'092	0.959	0.255319066	1
ENSBTAG00000000754	*PPP2R5A*	4'664	2'304	3'350	4'039	2'660	-0.602	0.440485817	1
ENSBTAG00000000755	*ZFR2*	329	283	306	285	327	0.198	0.839008135	1
ENSBTAG00000000758	*TMEM206*	1'296	612	915	1'122	707	-0.667	0.423485354	1
ENSBTAG00000000759	*NENF*	5'258	5'375	5'380	4'554	6'207	0.447	0.56199723	1
ENSBTAG00000000764	*GIPC1*	2'846	3'110	3'028	2'465	3'591	0.543	0.486907099	1
ENSBTAG00000000767	*BT.49626*	169	180	177	146	208	0.506	0.644325872	1
ENSBTAG00000000770	*PGM2L1*	456	210	319	395	242	-0.704	0.463201146	1
ENSBTAG00000000771	*BT.100877*	3'004	2'561	2'779	2'602	2'957	0.185	0.812815032	1
ENSBTAG00000000772	*RGSL1*	1	0	0	1	0		1	1
ENSBTAG00000000773	*TTC9C*	1'707	1'102	1'375	1'478	1'272	-0.216	0.78984452	1
ENSBTAG00000000776	*protein_coding*	5	6	6	4	7	0.678	0.946418607	1
ENSBTAG00000000778	*HSP90AB1*	57'841	85'725	74'539	50'092	98'987	0.983	0.198735612	1
ENSBTAG00000000781	*HIP1*	1'109	1'113	1'123	960	1'285	0.420	0.607174666	1
ENSBTAG00000000782	*KDR*	10'863	5'208	7'711	9'408	6'014	-0.646	0.401614961	1
ENSBTAG00000000783	*TGFA*	33	31	32	29	36	0.325	0.89170339	1
ENSBTAG00000000784	*BT.89705*	1'379	1'062	1'210	1'194	1'226	0.038	0.962925748	1
ENSBTAG00000000785	*PASK*	541	582	570	469	672	0.520	0.550936852	1
ENSBTAG00000000786	*NQO2*	854	271	526	740	313	-1.241	0.163991107	1
ENSBTAG00000000789	*PPP1R7*	1'885	1'735	1'818	1'632	2'003	0.295	0.709955023	1
ENSBTAG00000000791	*BT.35938*	252	46	136	218	53	-2.039	0.094200343	1
ENSBTAG00000000792	*AJAP1*	52	15	31	45	17	-1.379	0.538076057	1
ENSBTAG00000000793	*LAMC2*	607	1'408	1'076	526	1'626	1.629	0.051777272	1
ENSBTAG00000000795	*NMNAT2*	467	250	347	404	289	-0.486	0.606740899	1
ENSBTAG00000000797	*MRPL9*	3'354	2'700	3'011	2'905	3'118	0.102	0.895563631	1
ENSBTAG00000000799	*BT.35115*	825	912	884	714	1'053	0.560	0.502015084	1
ENSBTAG00000000801	*BT.76599*	727	484	594	630	559	-0.172	0.845184934	1
ENSBTAG00000000802	*LYVE1*	875	938	920	758	1'083	0.515	0.534934275	1
ENSBTAG00000000803	*ZNF667*	1'552	990	1'244	1'344	1'143	-0.234	0.774809485	1
ENSBTAG00000000804	*MOB3B*	387	353	371	335	408	0.282	0.762068639	1
ENSBTAG00000000806	*ATAD1*	4'136	2'698	3'349	3'582	3'115	-0.201	0.796744835	1
ENSBTAG00000000807	*TMED3*	3'940	3'943	3'983	3'412	4'553	0.416	0.591155456	1
ENSBTAG00000000808	*COX14*	1'596	1'299	1'441	1'382	1'500	0.118	0.883446609	1
ENSBTAG00000000809	*IRX1*	1'070	1'988	1'611	927	2'296	1.309	0.106418875	1
ENSBTAG00000000810	*COPE*	7'997	6'214	7'050	6'926	7'175	0.051	0.946401479	1
ENSBTAG00000000811	*BT.61505*	1'103	1'244	1'196	955	1'436	0.589	0.47009762	1
ENSBTAG00000000812	*CD300A*	10	11	11	9	13	0.553	0.915317076	1
ENSBTAG00000000813	*HOMER3*	1'577	1'434	1'511	1'366	1'656	0.278	0.728902214	1
ENSBTAG00000000815	*EPHA2*	1'588	1'055	1'297	1'375	1'218	-0.175	0.830301941	1
ENSBTAG00000000816	*PRDM1*	1'444	1'385	1'425	1'251	1'599	0.355	0.659226641	1
ENSBTAG00000000817	*SYNJ2*	127	118	123	110	136	0.309	0.803222358	1
ENSBTAG00000000818	*PLEKHG7*	14	2	7	12	2	-2.392	0.704665469	1
ENSBTAG00000000819	*RFX3*	60	35	46	52	40	-0.363	0.850925472	1
ENSBTAG00000000820	*GNG11*	1'940	2'888	2'507	1'680	3'335	0.989	0.210982035	1
ENSBTAG00000000821	*GPR137C*	365	304	334	316	351	0.151	0.875028351	1
ENSBTAG00000000824	*TSEN54*	1'427	1'641	1'565	1'236	1'895	0.617	0.442085957	1
ENSBTAG00000000825	*MRPS26*	1'072	1'100	1'099	928	1'270	0.452	0.580721503	1
ENSBTAG00000000827	*TAOK1*	172	104	135	149	120	-0.311	0.797994483	1
ENSBTAG00000000828	*CAPN6*	16'893	7'397	11'586	14'630	8'541	-0.776	0.311956963	1
ENSBTAG00000000829	*CARKL*	154	121	137	133	140	0.067	0.96043756	1
ENSBTAG00000000830	*GPR137B*	469	242	343	406	279	-0.540	0.568634547	1
ENSBTAG00000000831	*CTNS*	1'005	884	946	870	1'021	0.230	0.781382649	1
ENSBTAG00000000833	*TAX1BP3*	2'884	4'089	3'610	2'498	4'722	0.919	0.239816488	1
ENSBTAG00000000837	*PARP1*	8'802	8'356	8'636	7'623	9'649	0.340	0.656628001	1
ENSBTAG00000000838	*BT.49778*	766	578	665	663	667	0.009	0.993164583	1
ENSBTAG00000000841	*LZTS1*	327	810	609	283	935	1.724	0.051492478	1
ENSBTAG00000000842	*UBASH3B*	83	53	67	72	61	-0.232	0.889176645	1
ENSBTAG00000000843	*NDRG2*	2'887	3'189	3'091	2'500	3'682	0.559	0.474334278	1

Ensembl gene ID	geneName	counts wildtype horn bud	counts polled horn bud	baseMean	baseMean wildtype horn bud	baseMean polled horn bud	log2FoldChange	pval	padj
ENSBTAG00000000845	*BT.104271*	20	0	9	17	0		0.367514426	1
ENSBTAG00000000848	*SNRNP200*	10'871	17'234	14'657	9'415	19'900	1.080	0.160691408	1
ENSBTAG00000000851	*CYP20A1*	2'792	1'890	2'300	2'418	2'182	-0.148	0.852093126	1
ENSBTAG00000000853	*pseudogene*	11	0	5	10	0		0.617662983	1
ENSBTAG00000000854	*BT.91470*	912	348	596	790	402	-0.975	0.264523138	1
ENSBTAG00000000855	*NXN*	4'903	6'679	5'979	4'246	7'712	0.861	0.265622022	1
ENSBTAG00000000856	*FBXL6*	882	962	937	764	1'111	0.540	0.514782504	1
ENSBTAG00000000857	*GPR172B*	2'091	2'265	2'213	1'811	2'615	0.530	0.501514032	1
ENSBTAG00000000859	*SLC38A1*	33	21	26	29	24	-0.237	0.93825302	1
ENSBTAG00000000861	*BT.106462*	3	0	1	3	0		0.936647693	1
ENSBTAG00000000868	*ZFP1*	341	169	245	295	195	-0.598	0.555310444	1
ENSBTAG00000000869	*RNF219*	612	293	434	530	338	-0.648	0.476390102	1
ENSBTAG00000000871	*TRIM27*	2'894	2'607	2'758	2'506	3'010	0.264	0.735095617	1
ENSBTAG00000000873	*BT.26578*	46	17	30	40	20	-1.021	0.658132137	1
ENSBTAG00000000874	*CAST*	11'158	6'947	8'842	9'663	8'022	-0.269	0.726422198	1
ENSBTAG00000000875	*AZI1*	2'056	2'367	2'257	1'781	2'733	0.618	0.43343156	1
ENSBTAG00000000877	*METTL20*	48	45	47	42	52	0.322	0.865884674	1
ENSBTAG00000000878	*BT.55953*	5'458	3'106	4'157	4'727	3'586	-0.398	0.60790005	1
ENSBTAG00000000879	*BT.97625*	864	962	930	748	1'111	0.570	0.492310727	1
ENSBTAG00000000880	*MGC133692*	5'038	2'641	3'706	4'363	3'050	-0.517	0.506874513	1
ENSBTAG00000000885	*BT.86945*	347	752	584	301	868	1.531	0.083589582	1
ENSBTAG00000000886	*BT.81241*	1	0	0	1	0		1	1
ENSBTAG00000000888	*SPTA1*	30	10	19	26	12	-1.170	0.701297322	1
ENSBTAG00000000892	*MB21D1*	213	109	155	184	126	-0.551	0.629677998	1
ENSBTAG00000000893	*BT.61878*	2	0	1	2	0		0.974934741	1
ENSBTAG00000000894	*PGK1*	11'179	8'653	9'836	9'681	9'992	0.046	0.951974014	1
ENSBTAG00000000895	*TAF9B*	313	208	256	271	240	-0.175	0.865334469	1
ENSBTAG00000000897	*IQGAP2*	2'017	3'860	3'102	1'747	4'457	1.351	0.087776498	1
ENSBTAG00000000898	*F2RL2*	1'073	720	880	929	831	-0.161	0.848976264	1
ENSBTAG00000000899	*TMEM17*	1'045	720	868	905	831	-0.122	0.885266784	1
ENSBTAG00000000900	*LRRC8C*	265	474	388	229	547	1.254	0.177976326	1
ENSBTAG00000000902	*OGT*	17'871	6'854	11'696	15'477	7'914	-0.968	0.20883727	1
ENSBTAG00000000908	*GPR81*	8	2	5	7	2	-1.585	0.87391615	1
ENSBTAG00000000911	*UBE2H*	420	366	393	364	423	0.216	0.815153412	1
ENSBTAG00000000913	*UQCR10*	2'855	2'759	2'829	2'473	3'186	0.366	0.63974518	1
ENSBTAG00000000914	*OPRK1*	2	0	1	2	0		0.974934741	1
ENSBTAG00000000916	*TCTE3*	7	3	5	6	3	-0.807	0.947428618	1
ENSBTAG00000000917	*BMP1*	14'831	16'982	16'227	12'844	19'609	0.610	0.423979836	1
ENSBTAG00000000918	*BT.32415*	1'648	660	1'095	1'427	762	-0.905	0.272201122	1
ENSBTAG00000000919	*HEY2*	1'987	757	1'297	1'721	874	-0.977	0.231047235	1
ENSBTAG00000000920	*DNAH17*	129	113	121	112	130	0.224	0.859382474	1
ENSBTAG00000000923	*BT.27555*	91	119	108	79	137	0.802	0.529231262	1
ENSBTAG00000000924	*BT.106533*	0	1	1	0	1	Inf	0.993540919	1
ENSBTAG00000000925	*GLT8D2*	6'577	4'545	5'472	5'696	5'248	-0.118	0.878963633	1
ENSBTAG00000000928	*SPECC1*	409	240	316	354	277	-0.354	0.713723079	1
ENSBTAG00000000930	*protein_coding*	1	0	0	1	0		1	1
ENSBTAG00000000933	*BT.37395*	241	221	232	209	255	0.290	0.778125049	1
ENSBTAG00000000936	*SLC39A2*	98	119	111	85	137	0.695	0.58221071	1
ENSBTAG00000000937	*SSFA2*	4'723	2'652	3'576	4'090	3'062	-0.418	0.591902677	1
ENSBTAG00000000939	*KIF16B*	977	955	974	846	1'103	0.382	0.643649181	1
ENSBTAG00000000940	*TBCC*	2'278	1'520	1'864	1'973	1'755	-0.169	0.832953257	1
ENSBTAG00000000941	*PLEKHB2*	4'127	1'937	2'905	3'574	2'237	-0.676	0.388704619	1
ENSBTAG00000000942	*SIPA1L2*	929	552	721	805	637	-0.336	0.69386922	1
ENSBTAG00000000943	*ZNF286A*	3'062	2'395	2'709	2'652	2'766	0.061	0.938083135	1
ENSBTAG00000000944	*LPCAT1*	1'528	1'689	1'637	1'323	1'950	0.560	0.484203449	1
ENSBTAG00000000945	*BT.8228*	1'572	1'060	1'293	1'361	1'224	-0.153	0.851074014	1
ENSBTAG00000000946	*KIAA1609*	423	586	521	366	677	0.885	0.317542582	1
ENSBTAG00000000948	*RAB2A*	10'523	8'454	9'438	9'113	9'762	0.099	0.896240371	1
ENSBTAG00000000949	*DNAJC9*	557	480	518	482	554	0.200	0.821288627	1
ENSBTAG00000000950	*MRPS16*	1'002	868	935	868	1'002	0.208	0.802119454	1
ENSBTAG00000000951	*JAKMIP3*	35	15	24	30	17	-0.807	0.762172534	1
ENSBTAG00000000954	*BT.56234*	26	5	14	23	6	-1.963	0.593870669	1
ENSBTAG00000000955	*RBM41*	152	75	109	132	87	-0.604	0.637400047	1
ENSBTAG00000000957	*BT.27095*	1'890	1'195	1'508	1'637	1'380	-0.246	0.76001311	1
ENSBTAG00000000958	*ZSWIM3*	46	35	40	40	40	0.021	1	1
ENSBTAG00000000959	*BT.42406*	193	153	172	167	177	0.080	0.946809066	1
ENSBTAG00000000960	*SPATA25*	16	5	10	14	6	-1.263	0.797689913	1
ENSBTAG00000000961	*NEURL2*	111	95	103	96	110	0.190	0.88868693	1
ENSBTAG00000000962	*STAP1*	43	8	23	37	9	-2.011	0.452137267	1
ENSBTAG00000000964	*FCRL5*	50	13	29	43	15	-1.528	0.51113213	1
ENSBTAG00000000965	*TNNI3K*	1	0	0	1	0		1	1
ENSBTAG00000000967	*BT.28783*	377	478	439	326	552	0.757	0.40310468	1

Ensembl gene ID	geneName	counts wildtype horn bud	counts polled horn bud	baseMean	baseMean wildtype horn bud	baseMean polled horn bud	log2FoldChange	pval	padj
ENSBTAG00000000970	*BT.58425*	193	117	151	167	135	-0.307	0.79283865	1
ENSBTAG00000000972	*KRTAP10-2*	1	0	0	1	0		1	1
ENSBTAG00000000973	*KCNJ15*	180	62	114	156	72	-1.123	0.373250002	1
ENSBTAG00000000974	*IARS*	4'225	2'454	3'246	3'659	2'834	-0.369	0.636760112	1
ENSBTAG00000000977	*CADM1*	1'293	681	953	1'120	786	-0.510	0.539180491	1
ENSBTAG00000000978	*ASH2L*	1'718	1'467	1'591	1'488	1'694	0.187	0.814906679	1
ENSBTAG00000000979	*C27H8orf40*	2'844	2'072	2'428	2'463	2'393	-0.042	0.958752413	1
ENSBTAG00000000981	*KLHDC8B*	1'029	632	810	891	730	-0.288	0.732852697	1
ENSBTAG00000000982	*MGC137053*	1	2	2	1	2	1.415	0.981979269	1
ENSBTAG00000000983	*ABCC8*	650	656	660	563	757	0.428	0.617478055	1
ENSBTAG00000000985	*RAB10*	4'562	3'136	3'786	3'951	3'621	-0.126	0.872201832	1
ENSBTAG00000000986	*USH1C*	16	5	10	14	6	-1.263	0.797689913	1
ENSBTAG00000000987	*OTOG*	6	5	5	5	6	0.152	1	1
ENSBTAG00000000988	*BRCA2*	680	440	548	589	508	-0.213	0.810155764	1
ENSBTAG00000000990	*PSMA2*	667	615	644	578	710	0.298	0.729176824	1
ENSBTAG00000000991	*MRPL32*	658	479	561	570	553	-0.043	0.963679645	1
ENSBTAG00000000993	*N4BP2L1*	2'954	1'665	2'240	2'558	1'923	-0.412	0.602072644	1
ENSBTAG00000000998	*BT.25759*	3'823	3'319	3'572	3'311	3'832	0.211	0.785404375	1
ENSBTAG00000001000	*CA13*	1'409	673	999	1'220	777	-0.651	0.431663722	1
ENSBTAG00000001001	*ANKRD55*	10	8	9	9	9	0.093	1	1
ENSBTAG00000001002	*TCF7*	1'106	409	715	958	472	-1.020	0.23381284	1
ENSBTAG00000001003	*CKMT2*	40	29	34	35	33	-0.049	0.998899204	1
ENSBTAG00000001004	*BT.54007*	1'461	928	1'168	1'265	1'072	-0.240	0.769999571	1
ENSBTAG00000001005	*BT.88981*	181	167	175	157	193	0.299	0.787635033	1
ENSBTAG00000001007	*SLC25A43*	296	135	206	256	156	-0.718	0.496749778	1
ENSBTAG00000001008	*BT.21975*	7	6	6	6	7	0.193	1	1
ENSBTAG00000001009	*HCLS1*	542	308	413	469	356	-0.400	0.662882729	1
ENSBTAG00000001010	*ADAMTS18*	617	600	614	534	693	0.375	0.664837604	1
ENSBTAG00000001013	*SLC30A5*	5'599	2'869	4'081	4'849	3'313	-0.550	0.479372268	1
ENSBTAG00000001014	*MAPK7*	1'999	2'872	2'524	1'731	3'316	0.938	0.235070213	1
ENSBTAG00000001015	*TM9SF4*	6'015	6'700	6'473	5'209	7'736	0.571	0.458305718	1
ENSBTAG00000001016	*NAA35*	1'249	490	824	1'082	566	-0.935	0.268017562	1
ENSBTAG00000001017	*SLK*	1'307	562	890	1'132	649	-0.803	0.337509696	1
ENSBTAG00000001019	*GDF10*	885	300	556	766	346	-1.146	0.194772902	1
ENSBTAG00000001020	*AKAP3*	14	10	12	12	12	-0.070	1	1
ENSBTAG00000001021	*CYP1A1*	49	41	45	42	47	0.158	0.942536116	1
ENSBTAG00000001022	*AMDHD2*	459	504	490	398	582	0.550	0.536848794	1
ENSBTAG00000001023	*ZNF598*	2'010	2'164	2'120	1'741	2'499	0.522	0.509325257	1
ENSBTAG00000001024	*ARID1A*	5'713	4'507	5'076	4'948	5'204	0.073	0.924174671	1
ENSBTAG00000001026	*BCMO1*	5	2	3	4	2	-0.907	0.965043111	1
ENSBTAG00000001027	*SERPINH1*	94'234	106'709	102'413	81'609	123'217	0.594	0.434014732	1
ENSBTAG00000001029	*BT.44411*	310	189	243	268	218	-0.299	0.770551259	1
ENSBTAG00000001030	*BT.43678*	5'359	4'722	5'047	4'641	5'452	0.232	0.762615897	1
ENSBTAG00000001034	*IL18R1*	24	9	16	21	10	-1.000	0.775206422	1
ENSBTAG00000001035	*SF1*	12'296	12'628	12'615	10'649	14'582	0.453	0.552419738	1
ENSBTAG00000001036	*HIBADH*	4'009	2'537	3'201	3'472	2'929	-0.245	0.753950876	1
ENSBTAG00000001037	*MAP4K2*	943	1'321	1'171	817	1'525	0.901	0.271281208	1
ENSBTAG00000001038	*SNRPB*	3'069	3'773	3'507	2'658	4'357	0.713	0.360464339	1
ENSBTAG00000001041	*SAO*	1	6	4	1	7	3.000	0.788284548	1
ENSBTAG00000001042	*MXD1*	614	349	467	532	403	-0.400	0.65697154	1
ENSBTAG00000001043	*MGC157332*	199	31	104	172	36	-2.267	0.090670897	1
ENSBTAG00000001044	*SUPV3L1*	1'432	804	1'084	1'240	928	-0.418	0.611444768	1
ENSBTAG00000001049	*BT.89202*	438	294	359	379	339	-0.160	0.867187996	1
ENSBTAG00000001050	*LDLRAP1*	617	773	713	534	893	0.740	0.385089183	1
ENSBTAG00000001052	*ADCY10*	35	10	21	30	12	-1.392	0.622743083	1
ENSBTAG00000001055	*HNRNPL*	15'415	11'492	13'310	13'350	13'270	-0.009	0.991724988	1
ENSBTAG00000001057	*ARFGAP3*	5'062	3'405	4'158	4'384	3'932	-0.157	0.840144335	1
ENSBTAG00000001058	*SRPK3*	325	412	379	281	476	0.757	0.414309591	1
ENSBTAG00000001059	*IDH3G*	4'390	3'876	4'139	3'802	4'476	0.235	0.760657669	1
ENSBTAG00000001060	*CXCR4*	3'635	4'536	4'193	3'148	5'238	0.735	0.344352145	1
ENSBTAG00000001061	*BT.62458*	403	464	442	349	536	0.618	0.493953857	1
ENSBTAG00000001062	*PTH2*	1	0	0	1	0		1	1
ENSBTAG00000001063	*BT.59423*	32	76	58	28	88	1.663	0.312589678	1
ENSBTAG00000001066	*BT.27151*	81	26	50	70	30	-1.224	0.485992574	1
ENSBTAG00000001067	*GRM2*	5	4	4	4	5	0.093	1	1
ENSBTAG00000001068	*ZCWPW1*	1'209	338	719	1'047	390	-1.424	0.099150047	1
ENSBTAG00000001069	*TP53*	3'984	5'647	4'985	3'450	6'521	0.918	0.236924962	1
ENSBTAG00000001071	*BT.20351*	1'279	1'373	1'347	1'108	1'585	0.517	0.522103811	1
ENSBTAG00000001074	*SAYSD1*	148	163	158	128	188	0.554	0.62404116	1
ENSBTAG00000001076	*NLRP6*	7	11	9	6	13	1.067	0.833633343	1
ENSBTAG00000001077	*OSBPL5*	2'579	4'376	3'643	2'233	5'053	1.178	0.13364905	1
ENSBTAG00000001079	*pseudogene*	641	608	629	555	702	0.339	0.694519667	1

Ensembl gene ID	geneName	counts wildtype horn bud	counts polled horn bud	baseMean	baseMean wildtype horn bud	baseMean polled horn bud	log2FoldChange	pval	padj
ENSBTAG00000001080	*SPAG17*	16	2	8	14	2	-2.585	0.65347607	1
ENSBTAG00000001081	*PALLD*	2'188	3'181	2'784	1'895	3'673	0.955	0.225334362	1
ENSBTAG00000001082	*SH2D5*	83	214	159	72	247	1.781	0.12052836	1
ENSBTAG00000001083	*BT.55601*	377	403	396	326	465	0.511	0.578292744	1
ENSBTAG00000001085	*FAM20A*	336	86	195	291	99	-1.551	0.152321683	1
ENSBTAG00000001086	*FHL2*	699	1'295	1'050	605	1'495	1.305	0.11691582	1
ENSBTAG00000001088	*SLC17A8*	5	1	3	4	1	-1.907	0.9188325	1
ENSBTAG00000001090	*C2ORF77*	103	61	80	89	70	-0.341	0.817883757	1
ENSBTAG00000001092	*PHOSPHO2*	1'064	675	850	921	779	-0.242	0.774178084	1
ENSBTAG00000001093	*KLHL23*	526	433	478	456	500	0.134	0.881703082	1
ENSBTAG00000001096	*PRKRA*	1'687	750	1'164	1'461	866	-0.754	0.357139473	1
ENSBTAG00000001097	*FKBP7*	11'015	4'916	7'608	9'539	5'677	-0.749	0.331189609	1
ENSBTAG00000001098	*BT.26062*	1'109	572	810	960	660	-0.540	0.521431178	1
ENSBTAG00000001100	*BT.49121*	70	30	48	61	35	-0.807	0.655317609	1
ENSBTAG00000001101	*IL28RA*	41	30	35	36	35	-0.036	1	1
ENSBTAG00000001102	*SERPINB10*	331	190	253	287	219	-0.386	0.702628887	1
ENSBTAG00000001104	*CMTM4*	1'490	881	1'154	1'290	1'017	-0.343	0.67531474	1
ENSBTAG00000001105	*ANXA4*	9'377	6'564	7'850	8'121	7'579	-0.100	0.897481866	1
ENSBTAG00000001107	*CACYBP*	4'848	3'519	4'131	4'198	4'063	-0.047	0.952400008	1
ENSBTAG00000001108	*BT.45372*	2'048	1'555	1'785	1'774	1'796	0.018	0.982317522	1
ENSBTAG00000001109	*MRPS14*	1'097	864	974	950	998	0.071	0.932451829	1
ENSBTAG00000001110	*SLC16A5*	430	697	589	372	805	1.112	0.204170504	1
ENSBTAG00000001112	*SMOX*	958	1'010	998	830	1'166	0.491	0.551487888	1
ENSBTAG00000001113	*BT.77215*	439	259	340	380	299	-0.346	0.716005288	1
ENSBTAG00000001114	*PRKD3*	3'102	2'221	2'626	2'686	2'565	-0.067	0.933068869	1
ENSBTAG00000001116	*P4HA2*	31'533	28'643	30'191	27'308	33'074	0.276	0.715594093	1
ENSBTAG00000001117	*ANKRD50*	1'839	1'218	1'500	1'593	1'406	-0.179	0.824446855	1
ENSBTAG00000001119	*ANKRD13B*	929	1'164	1'074	805	1'344	0.740	0.367961611	1
ENSBTAG00000001120	*CORO6*	335	161	238	290	186	-0.642	0.529068699	1
ENSBTAG00000001123	*SNRNP48*	1'646	746	1'143	1'425	861	-0.727	0.375562489	1
ENSBTAG00000001124	*GALC*	392	123	241	339	142	-1.257	0.219310254	1
ENSBTAG00000001125	*ADAMDEC1*	2	1	1	2	1	-0.585	1	1
ENSBTAG00000001126	*BMP2K*	464	257	349	402	297	-0.437	0.643324282	1
ENSBTAG00000001128	*RNF165*	29	28	29	25	32	0.364	0.885456337	1
ENSBTAG00000001131	*SLC12A9*	1'570	1'799	1'718	1'360	2'077	0.611	0.443755732	1
ENSBTAG00000001132	*GTDC1*	1'090	937	1'013	944	1'082	0.197	0.81125582	1
ENSBTAG00000001133	*KIAA0564*	725	661	696	628	763	0.282	0.741276665	1
ENSBTAG00000001134	*BT.20029*	7'621	6'803	7'228	6'600	7'855	0.251	0.742810566	1
ENSBTAG00000001135	*C1ORF194*	25	0	11	22	0		0.274776285	1
ENSBTAG00000001136	*KIAA1324*	49	13	29	42	15	-1.499	0.522603237	1
ENSBTAG00000001137	*CLTA*	6'001	6'507	6'355	5'197	7'514	0.532	0.489348478	1
ENSBTAG00000001138	*UFSP1*	154	184	173	133	212	0.672	0.541923685	1
ENSBTAG00000001139	*ACHE*	681	417	536	590	482	-0.293	0.741180422	1
ENSBTAG00000001140	*IAH1*	1'340	771	1'025	1'160	890	-0.382	0.643387802	1
ENSBTAG00000001141	*ADAM17*	855	1'032	966	740	1'192	0.686	0.407357376	1
ENSBTAG00000001142	*GNE*	1'078	1'012	1'051	934	1'169	0.324	0.693276123	1
ENSBTAG00000001143	*BT.24012*	164	120	140	142	139	-0.036	0.983490226	1
ENSBTAG00000001144	*NAA20*	2'700	1'856	2'241	2'338	2'143	-0.126	0.874376101	1
ENSBTAG00000001146	*HIVEP2*	1'914	1'706	1'814	1'658	1'970	0.249	0.753833534	1
ENSBTAG00000001150	*KCNE1*	5	0	2	4	0		0.853268594	1
ENSBTAG00000001151	*BT.56047*	3'111	2'976	3'065	2'694	3'436	0.351	0.652410218	1
ENSBTAG00000001152	*TLE6*	42	12	25	36	14	-1.392	0.582474487	1
ENSBTAG00000001153	*TLE2*	2'441	2'603	2'560	2'114	3'006	0.508	0.517662214	1
ENSBTAG00000001154	*DGAT2*	399	257	321	346	297	-0.220	0.820986088	1
ENSBTAG00000001156	*ST3GAL1*	275	309	297	238	357	0.583	0.547130052	1
ENSBTAG00000001160	*BT.35623*	1'226	804	995	1'062	928	-0.194	0.815915473	1
ENSBTAG00000001161	*FAM60A*	2'057	1'690	1'866	1'781	1'951	0.132	0.868293756	1
ENSBTAG00000001163	*H2AFY2*	1'171	1'564	1'410	1'014	1'806	0.833	0.303468558	1
ENSBTAG00000001164	*BT.91229*	1'269	906	1'073	1'099	1'046	-0.071	0.932775139	1
ENSBTAG00000001165	*AIFM2*	409	498	465	354	575	0.699	0.436289835	1
ENSBTAG00000001166	*HSF5*	11	5	8	10	6	-0.722	0.917926652	1
ENSBTAG00000001168	*BRAP*	683	490	579	591	566	-0.064	0.944118823	1
ENSBTAG00000001171	*OGFOD1*	2'040	1'317	1'644	1'767	1'521	-0.216	0.787743649	1
ENSBTAG00000001173	*PLXNA2*	2'414	656	1'424	2'091	757	-1.465	0.073900995	1
ENSBTAG00000001174	*MRPL15*	1'161	836	985	1'005	965	-0.059	0.945229523	1
ENSBTAG00000001176	*LRRN1*	775	1'407	1'148	671	1'625	1.275	0.122728032	1
ENSBTAG00000001178	*USPL1*	1'033	968	1'006	895	1'118	0.321	0.696610315	1
ENSBTAG00000001179	*TEP1*	2'768	2'357	2'559	2'397	2'722	0.183	0.81502592	1
ENSBTAG00000001181	*RPS6KC1*	2'729	2'071	2'377	2'363	2'391	0.017	0.982672384	1
ENSBTAG00000001182	*CDC10*	21'962	11'152	15'948	19'020	12'877	-0.563	0.461766578	1
ENSBTAG00000001183	*BT.21156*	44	10	25	38	12	-1.722	0.500505815	1
ENSBTAG00000001185	*BT.30964*	1'344	1'008	1'164	1'164	1'164	0.000	1	1

Ensembl gene ID	geneName	counts wildtype horn bud	counts polled horn bud	baseMean	baseMean wildtype horn bud	baseMean polled horn bud	log2FoldChange	pval	padj
ENSBTAG00000001186	*SH2D4A*	61	50	55	53	58	0.128	0.948786563	1
ENSBTAG00000001187	*RASL10A*	32	2	15	28	2	-3.585	0.345807076	1
ENSBTAG00000001188	*ROM1*	595	262	409	515	303	-0.768	0.402896631	1
ENSBTAG00000001189	*BT.35586*	2'669	3'338	3'083	2'311	3'854	0.738	0.345829331	1
ENSBTAG00000001192	*STX12*	3'717	2'416	3'004	3'219	2'790	-0.206	0.792253226	1
ENSBTAG00000001193	*BT.53643*	1'021	851	933	884	983	0.152	0.854577519	1
ENSBTAG00000001195	*KCNH4*	45	5	22	39	6	-2.755	0.327424431	1
ENSBTAG00000001197	*SLAMF7*	61	23	40	53	27	-0.992	0.615130035	1
ENSBTAG00000001198	*GIMAP7*	16	3	9	14	3	-2.000	0.709202358	1
ENSBTAG00000001199	*DAP3*	889	852	877	770	984	0.354	0.671328127	1
ENSBTAG00000001204	*BT.26095*	2'608	2'207	2'404	2'259	2'548	0.174	0.824314745	1
ENSBTAG00000001206	*BT.34282*	5	0	2	4	0		0.853268594	1
ENSBTAG00000001207	*SERPINB8*	1'003	488	716	869	563	-0.624	0.464247606	1
ENSBTAG00000001209	*PHLDB2*	4'684	5'204	5'033	4'056	6'009	0.567	0.462960117	1
ENSBTAG00000001212	*BT.49010*	20'712	20'979	21'081	17'937	24'224	0.434	0.568846084	1
ENSBTAG00000001213	*pseudogene*	3	1	2	3	1	-1.170	0.985253077	1
ENSBTAG00000001219	*BT.105273*	139	35	80	120	40	-1.575	0.274897885	1
ENSBTAG00000001223	*CAAP1*	762	645	702	660	745	0.175	0.838132898	1
ENSBTAG00000001224	*ATP9B*	2'153	1'462	1'776	1'865	1'688	-0.143	0.858265204	1
ENSBTAG00000001225	*BT.91172*	3'672	4'497	4'186	3'180	5'193	0.707	0.362259245	1
ENSBTAG00000001229	*SLC25A22*	1'800	1'670	1'744	1'559	1'928	0.307	0.699808217	1
ENSBTAG00000001231	*BT.97285*	244	107	167	211	124	-0.774	0.487846918	1
ENSBTAG00000001233	*MPZL3*	917	416	637	794	480	-0.725	0.401473105	1
ENSBTAG00000001235	*CD200R1*	205	199	204	178	230	0.372	0.725403112	1
ENSBTAG00000001241	*ISL1*	24	40	33	21	46	1.152	0.588971366	1
ENSBTAG00000001242	*BT.79981*	30	114	79	26	132	2.341	0.113666671	1
ENSBTAG00000001243	*AP3M2*	106	36	67	92	42	-1.143	0.459623889	1
ENSBTAG00000001244	*PLAT*	2'762	1'553	2'093	2'392	1'793	-0.416	0.599972669	1
ENSBTAG00000001246	*BT.54076*	13'105	11'259	12'175	11'349	13'001	0.196	0.796761479	1
ENSBTAG00000001247	*LECT2*	3	2	2	3	2	-0.170	1	1
ENSBTAG00000001249	*SULT1B1*	56	41	48	48	47	-0.035	0.999225712	1
ENSBTAG00000001250	*TFAP2A*	3'005	2'235	2'592	2'602	2'581	-0.012	0.988989083	1
ENSBTAG00000001252	*HIVEP1*	1'643	1'141	1'370	1'423	1'318	-0.111	0.892068482	1
ENSBTAG00000001254	*ANKRD46*	4'961	2'833	3'784	4'296	3'271	-0.393	0.613172717	1
ENSBTAG00000001255	*CRYBB3*	3	1	2	3	1	-1.170	0.985253077	1
ENSBTAG00000001257	*AGTPBP1*	1'157	674	890	1'002	778	-0.365	0.662767892	1
ENSBTAG00000001258	*TMEM50B*	2'447	1'427	1'883	2'119	1'648	-0.363	0.648464421	1
ENSBTAG00000001260	*BT.36570*	16	32	25	14	37	1.415	0.568981356	1
ENSBTAG00000001262	*IRGQ*	309	434	384	268	501	0.905	0.32878266	1
ENSBTAG00000001269	*TMEM165*	1'529	1'081	1'286	1'324	1'248	-0.085	0.917851458	1
ENSBTAG00000001271	*PLG*	11	2	6	10	2	-2.044	0.786625039	1
ENSBTAG00000001273	*NLRP3*	56	19	35	48	22	-1.144	0.585245408	1
ENSBTAG00000001274	*PPM1L*	93	64	77	81	74	-0.124	0.941516573	1
ENSBTAG00000001279	*MPP2*	237	338	298	205	390	0.927	0.339569189	1
ENSBTAG00000001280	*CLPB*	212	200	207	184	231	0.331	0.754114128	1
ENSBTAG00000001282	*BT.103355*	4'028	1'723	2'739	3'488	1'990	-0.810	0.303372209	1
ENSBTAG00000001283	*KIAA1432*	1'211	714	937	1'049	824	-0.347	0.676626285	1
ENSBTAG00000001286	*ELMO3*	1'156	1'654	1'456	1'001	1'910	0.932	0.249309753	1
ENSBTAG00000001287	*LRRC29*	63	32	46	55	37	-0.562	0.764157681	1
ENSBTAG00000001288	*MAOB*	113	19	60	98	22	-2.157	0.192832677	1
ENSBTAG00000001289	*MRPL18*	1'459	1'012	1'216	1'264	1'169	-0.113	0.891290351	1
ENSBTAG00000001290	*BT.25963*	407	265	329	352	306	-0.204	0.832973251	1
ENSBTAG00000001292	*LTF*	114	28	66	99	32	-1.610	0.304459837	1
ENSBTAG00000001293	*C3ORF15*	116	22	63	100	25	-1.984	0.218564221	1
ENSBTAG00000001294	*BT.59327*	874	1'000	956	757	1'155	0.609	0.462102447	1
ENSBTAG00000001295	*DEPDC5*	706	850	796	611	981	0.683	0.417677889	1
ENSBTAG00000001296	*TMEM50A*	4'628	3'221	3'864	4'008	3'719	-0.108	0.89032806	1
ENSBTAG00000001297	*protein_coding*	12	0	5	10	0		0.583618942	1
ENSBTAG00000001298	*STAMBPL1*	612	316	447	530	365	-0.539	0.551900119	1
ENSBTAG00000001299	*BT.58547*	153	76	110	133	88	-0.594	0.641790135	1
ENSBTAG00000001301	*LRRC32*	169	159	165	146	184	0.327	0.771520225	1
ENSBTAG00000001302	*MRPS28*	407	303	351	352	350	-0.011	0.995240782	1
ENSBTAG00000001305	*ATP2B2*	172	40	98	149	46	-1.689	0.209168088	1
ENSBTAG00000001306	*FXN*	1'128	976	1'052	977	1'127	0.206	0.801818813	1
ENSBTAG00000001308	*BT.29090*	12	52	35	10	60	2.531	0.236694237	1
ENSBTAG00000001310	*RHO*	5	2	3	4	2	-0.907	0.965043111	1
ENSBTAG00000001311	*MORF4L2*	22'753	14'939	18'477	19'705	17'250	-0.192	0.80164527	1
ENSBTAG00000001314	*TRMT6*	493	380	433	427	439	0.039	0.967326071	1
ENSBTAG00000001315	*DMRTA1*	4	4	4	3	5	0.415	0.994759363	1
ENSBTAG00000001320	*VPS16*	3'396	2'890	3'139	2'941	3'337	0.182	0.814704369	1
ENSBTAG00000001321	*IL1B*	14	7	10	12	8	-0.585	0.916699259	1
ENSBTAG00000001322	*FAM154A*	9	0	4	8	0		0.690676647	1

Ensembl gene ID	geneName	counts wildtype horn bud	counts polled horn bud	baseMean	baseMean wildtype horn bud	baseMean polled horn bud	log2FoldChange	pval	padj
ENSBTAG00000001323	*BT.50011*	2'442	1'113	1'700	2'115	1'285	-0.719	0.369778079	1
ENSBTAG00000001324	*BT.89425*	550	398	468	476	460	-0.052	0.957332383	1
ENSBTAG00000001325	*UPB1*	27	1	12	23	1	-4.340	0.350225008	1
ENSBTAG00000001328	*SLBP*	1'754	1'240	1'475	1'519	1'432	-0.085	0.916964865	1
ENSBTAG00000001329	*CLPTM1L*	4'729	5'145	5'018	4'095	5'941	0.537	0.487034154	1
ENSBTAG00000001331	*FAM194A*	8	1	4	7	1	-2.585	0.818599781	1
ENSBTAG00000001332	*MYO1C*	7'632	12'011	10'239	6'610	13'869	1.069	0.165830217	1
ENSBTAG00000001333	*PPARG*	1'076	692	865	932	799	-0.222	0.791996587	1
ENSBTAG00000001335	*GHR*	1'229	922	1'064	1'064	1'065	0.000	1	1
ENSBTAG00000001338	*BT.28967*	4	6	5	3	7	1.000	0.91481797	1
ENSBTAG00000001341	*WNT5B*	189	303	257	164	350	1.096	0.274735124	1
ENSBTAG00000001342	*RSL24D1*	2'341	1'726	2'010	2'027	1'993	-0.025	0.976540631	1
ENSBTAG00000001343	*DEPDC1*	339	189	256	294	218	-0.428	0.670766153	1
ENSBTAG00000001344	*protein_coding*	2	6	4	2	7	2.000	0.840116992	1
ENSBTAG00000001346	*STRA8*	182	27	94	158	31	-2.338	0.092904773	1
ENSBTAG00000001348	*COMMD8*	499	575	548	432	664	0.620	0.480168214	1
ENSBTAG00000001349	*KIF1C*	6'377	8'518	7'679	5'523	9'836	0.833	0.279890704	1
ENSBTAG00000001350	*protein_coding*	628	1'412	1'087	544	1'630	1.584	0.05808713	1
ENSBTAG00000001352	*SDCCAG3*	2'584	2'326	2'462	2'238	2'686	0.263	0.737111037	1
ENSBTAG00000001353	*PMPCA*	2'820	3'179	3'056	2'442	3'671	0.588	0.451725713	1
ENSBTAG00000001354	*INPP5E*	351	365	363	304	421	0.471	0.61357337	1
ENSBTAG00000001356	*BT.46819*	1'195	591	859	1'035	682	-0.601	0.473524457	1
ENSBTAG00000001357	*PRMT1*	1	0	0	1	0		1	1
ENSBTAG00000001359	*B3GNT7*	110	304	223	95	351	1.882	0.074672923	1
ENSBTAG00000001360	*RPS12*	53'988	56'259	55'859	46'755	64'962	0.474	0.531992531	1
ENSBTAG00000001361	*NMNAT1*	470	438	456	407	506	0.313	0.727979525	1
ENSBTAG00000001364	*ATPBD4*	371	319	345	321	368	0.197	0.835583015	1
ENSBTAG00000001365	*C1QTNF9*	23	10	16	20	12	-0.787	0.825334133	1
ENSBTAG00000001367	*C1QTNF2*	2'645	4'259	3'604	2'291	4'918	1.102	0.159878967	1
ENSBTAG00000001368	*LGALS3BP*	899	1'353	1'170	779	1'562	1.005	0.220868991	1
ENSBTAG00000001374	*WBSCR27*	140	26	76	121	30	-2.014	0.178254861	1
ENSBTAG00000001376	*RAD21L1*	0	1	1	0	1	Inf	0.993540919	1
ENSBTAG00000001382	*SLC26A9*	26	4	14	23	5	-2.285	0.549753943	1
ENSBTAG00000001385	*ZSCAN2*	1'035	1'852	1'517	896	2'139	1.254	0.12242662	1
ENSBTAG00000001387	*BT.42449*	476	306	383	412	353	-0.222	0.812559504	1
ENSBTAG00000001388	*NMB*	277	148	205	240	171	-0.489	0.644274978	1
ENSBTAG00000001390	*B4GALT4*	3'036	2'176	2'571	2'629	2'513	-0.065	0.934645862	1
ENSBTAG00000001391	*ANKRD42*	873	242	518	756	279	-1.436	0.109576207	1
ENSBTAG00000001392	*RDH16*	11	1	5	10	1	-3.044	0.722857941	1
ENSBTAG00000001393	*MTF2*	1'075	660	847	931	762	-0.289	0.731283436	1
ENSBTAG00000001394	*TMED9*	11'031	12'708	12'114	9'553	14'674	0.619	0.418265659	1
ENSBTAG00000001395	*C1H21ORF91*	440	309	369	381	357	-0.095	0.922362767	1
ENSBTAG00000001396	*ADAMTS7*	2'653	4'037	3'480	2'298	4'662	1.021	0.192782052	1
ENSBTAG00000001398	*ATP2A2*	4'862	4'062	4'451	4'211	4'690	0.156	0.83992953	1
ENSBTAG00000001400	*AKT2*	2'451	4'106	3'432	2'123	4'741	1.159	0.140259225	1
ENSBTAG00000001403	*PRKCI*	1'375	848	1'085	1'191	979	-0.282	0.731786393	1
ENSBTAG00000001405	*GLCE*	2'017	1'680	1'843	1'747	1'940	0.151	0.848803506	1
ENSBTAG00000001406	*MAB21L3*	4	2	3	3	2	-0.585	0.996239345	1
ENSBTAG00000001407	*AP3S2*	1'932	2'214	2'115	1'673	2'557	0.612	0.439511931	1
ENSBTAG00000001408	*CNRIP1*	3'262	1'524	2'292	2'825	1'760	-0.683	0.387840217	1
ENSBTAG00000001410	*GRAMD1B*	316	157	227	274	181	-0.594	0.564570941	1
ENSBTAG00000001412	*N6AMT1*	90	86	89	78	99	0.349	0.80260471	1
ENSBTAG00000001414	*KCTD12*	737	322	505	638	372	-0.780	0.381262035	1
ENSBTAG00000001415	*BT.59626*	398	536	482	345	619	0.845	0.34527138	1
ENSBTAG00000001417	*BT.96657*	432	125	259	374	144	-1.374	0.173408715	1
ENSBTAG00000001419	*MRPL4*	1'604	1'832	1'752	1'389	2'115	0.607	0.446810608	1
ENSBTAG00000001420	*ABHD12*	2'751	3'369	3'136	2'382	3'890	0.707	0.365586974	1
ENSBTAG00000001422	*HMGN3*	7'460	6'098	6'751	6'461	7'041	0.124	0.870960831	1
ENSBTAG00000001424	*BT.98842*	5'288	4'342	4'797	4'580	5'014	0.131	0.865098114	1
ENSBTAG00000001425	*FBXO11*	2'760	1'086	1'822	2'390	1'254	-0.931	0.245135014	1
ENSBTAG00000001429	*NEURL1B*	253	365	320	219	421	0.944	0.324323135	1
ENSBTAG00000001432	*protein_coding*	140	142	143	121	164	0.436	0.709920272	1
ENSBTAG00000001435	*ARL6IP6*	1'825	1'167	1'464	1'580	1'348	-0.230	0.77590575	1
ENSBTAG00000001439	*HESX1*	251	71	150	217	82	-1.407	0.225829463	1
ENSBTAG00000001440	*PMM2*	1'166	1'023	1'096	1'010	1'181	0.226	0.782303925	1
ENSBTAG00000001441	*CARHSP1*	967	1'488	1'278	837	1'718	1.037	0.20419704	1
ENSBTAG00000001443	*protein_coding*	25	1	11	22	1	-4.229	0.384445569	1
ENSBTAG00000001444	*TNXB*	7'799	17'484	13'471	6'754	20'189	1.580	0.042643062	1
ENSBTAG00000001446	*ABRACL*	528	328	418	457	379	-0.272	0.767756139	1
ENSBTAG00000001447	*HRH1*	361	135	234	313	156	-1.004	0.327637982	1
ENSBTAG00000001449	*SRA1*	3'600	2'020	2'725	3'118	2'332	-0.419	0.593831879	1
ENSBTAG00000001450	*APBB3*	2'878	2'104	2'461	2'492	2'429	-0.037	0.963771881	1

Ensembl gene ID	geneName	counts wildtype horn bud	counts polled horn bud	baseMean	baseMean wildtype horn bud	baseMean polled horn bud	log2FoldChange	pval	padj
ENSBTAG00000001457	*C20H5orf28*	273	158	209	236	182	-0.374	0.723948349	1
ENSBTAG00000001460	*LIN7C*	7'368	3'580	5'257	6'381	4'134	-0.626	0.418259701	1
ENSBTAG00000001462	*FLT4*	323	298	312	280	344	0.299	0.756433819	1
ENSBTAG00000001463	*TNKS*	365	376	375	316	434	0.458	0.621827102	1
ENSBTAG00000001464	*GPR156*	114	92	102	99	106	0.106	0.941827598	1
ENSBTAG00000001465	*P2RY1*	325	67	179	281	77	-1.863	0.095475434	1
ENSBTAG00000001468	*SAMD4A*	496	340	411	430	393	-0.130	0.890157216	1
ENSBTAG00000001470	*DTX2*	954	1'184	1'097	826	1'367	0.727	0.376119833	1
ENSBTAG00000001471	*POC1A*	370	539	471	320	622	0.958	0.286681354	1
ENSBTAG00000001473	*ARVCF*	922	619	757	798	715	-0.160	0.851970947	1
ENSBTAG00000001474	*NRGN*	1'101	721	893	953	833	-0.196	0.815717596	1
ENSBTAG00000001475	*BT.103442*	343	452	409	297	522	0.813	0.374885742	1
ENSBTAG00000001476	*BT.105339*	4	3	3	3	3	0.000	1	1
ENSBTAG00000001477	*pseudogene*	161	381	290	139	440	1.658	0.094438273	1
ENSBTAG00000001478	*PPPDE1*	286	253	270	248	292	0.238	0.811170349	1
ENSBTAG00000001481	*IGSF9*	1'503	2'676	2'196	1'302	3'090	1.247	0.118318795	1
ENSBTAG00000001483	*SRGAP2*	1'475	879	1'146	1'277	1'015	-0.332	0.685624111	1
ENSBTAG00000001484	*BT.49509*	2'449	1'082	1'685	2'121	1'249	-0.763	0.341071485	1
ENSBTAG00000001485	*PPIP5K2*	7'906	3'745	5'586	6'847	4'324	-0.663	0.39122725	1
ENSBTAG00000001486	*ZNF280D*	2'895	1'163	1'925	2'507	1'343	-0.901	0.259274619	1
ENSBTAG00000001489	*BT.53243*	36'109	31'105	33'594	31'271	35'917	0.200	0.791896092	1
ENSBTAG00000001492	*FYB*	270	80	163	234	92	-1.340	0.236352949	1
ENSBTAG00000001497	*MRAS*	3'575	4'731	4'279	3'096	5'463	0.819	0.291972928	1
ENSBTAG00000001498	*CYTH2*	2'746	3'025	2'936	2'378	3'493	0.555	0.478079268	1
ENSBTAG00000001499	*TRIM33*	500	334	409	433	386	-0.167	0.857910091	1
ENSBTAG00000001500	*BT.72793*	180	192	189	156	222	0.508	0.637108616	1
ENSBTAG00000001502	*protein_coding*	2	1	1	2	1	-0.585	1	1
ENSBTAG00000001503	*TRIM72*	14	105	67	12	121	3.322	0.044430868	1
ENSBTAG00000001504	*NOP14*	2'406	2'404	2'430	2'084	2'776	0.414	0.598321105	1
ENSBTAG00000001505	*GRK4*	604	644	633	523	744	0.508	0.555878946	1
ENSBTAG00000001506	*HTT*	3'304	7'453	5'734	2'861	8'606	1.589	0.043345916	1
ENSBTAG00000001507	*BT.93167*	81	100	93	70	115	0.719	0.593728191	1
ENSBTAG00000001508	*BT.44357*	403	303	349	349	350	0.004	0.999821352	1
ENSBTAG00000001509	*ELK3*	1'605	734	1'119	1'390	848	-0.714	0.384832103	1
ENSBTAG00000001510	*CDK17*	1'155	668	886	1'000	771	-0.375	0.653865465	1
ENSBTAG00000001511	*BCL6*	817	394	581	708	455	-0.637	0.465778008	1
ENSBTAG00000001512	*ZFP64*	945	1'082	1'034	818	1'249	0.610	0.458598133	1
ENSBTAG00000001513	*PDIK1L*	389	323	355	337	373	0.147	0.877212312	1
ENSBTAG00000001514	*ASB11*	498	233	350	431	269	-0.681	0.47011736	1
ENSBTAG00000001518	*ISG20L2*	2'004	1'826	1'922	1'736	2'108	0.281	0.72296146	1
ENSBTAG00000001519	*BT.46083*	619	434	519	536	501	-0.097	0.915157086	1
ENSBTAG00000001520	*MRPL24*	1'023	981	1'009	886	1'133	0.355	0.666908223	1
ENSBTAG00000001521	*UQCRB*	2'694	1'276	1'903	2'333	1'473	-0.663	0.405181186	1
ENSBTAG00000001522	*MTERFD1*	1'077	639	835	933	738	-0.338	0.687697621	1
ENSBTAG00000001523	*YES1*	5'460	3'646	4'469	4'728	4'210	-0.168	0.829249319	1
ENSBTAG00000001526	*protein_coding*	1	0	0	1	0		1	1
ENSBTAG00000001527	*TMEM213*	2	0	1	2	0		0.974934741	1
ENSBTAG00000001528	*CDK13*	2'257	1'249	1'698	1'955	1'442	-0.439	0.583518227	1
ENSBTAG00000001529	*BT.88726*	3'228	3'470	3'401	2'796	4'007	0.519	0.504664572	1
ENSBTAG00000001530	*FGF8*	0	2	1	0	2	Inf	0.939077559	1
ENSBTAG00000001533	*HACE1*	1'937	1'076	1'460	1'677	1'242	-0.433	0.591227167	1
ENSBTAG00000001535	*LIPH*	45	38	41	39	44	0.171	0.940017647	1
ENSBTAG00000001537	*TSKU*	4'409	5'127	4'869	3'818	5'920	0.633	0.413350571	1
ENSBTAG00000001538	*BT.61268*	21'529	25'684	24'151	18'645	29'657	0.670	0.379960887	1
ENSBTAG00000001539	*BT.61728*	2'004	1'364	1'655	1'736	1'575	-0.140	0.862110689	1
ENSBTAG00000001543	*BRI3BP*	17	33	26	15	38	1.372	0.572232262	1
ENSBTAG00000001545	*BT.61466*	26	14	19	23	16	-0.478	0.884547607	1
ENSBTAG00000001546	*MGAT1*	4'575	4'970	4'850	3'962	5'739	0.535	0.489042571	1
ENSBTAG00000001548	*UBQLN4*	1'336	1'865	1'655	1'157	2'154	0.896	0.264336066	1
ENSBTAG00000001551	*PLEKHG3*	1'711	2'568	2'224	1'482	2'965	1.001	0.207624792	1
ENSBTAG00000001552	*EPC1*	2'446	1'810	2'104	2'118	2'090	-0.019	0.981781338	1
ENSBTAG00000001553	*HNRNPA1*	24'712	13'328	18'396	21'401	15'390	-0.476	0.533268275	1
ENSBTAG00000001557	*CCL28*	14	3	8	12	3	-1.807	0.758305361	1
ENSBTAG00000001558	*PATL2*	15	65	44	13	75	2.531	0.186587477	1
ENSBTAG00000001562	*NFE2*	27	13	19	23	15	-0.639	0.839610081	1
ENSBTAG00000001564	*PDE4DIP*	2'637	1'795	2'178	2'284	2'073	-0.140	0.86039283	1
ENSBTAG00000001565	*MESDC2*	7'319	6'218	6'759	6'338	7'180	0.180	0.814269065	1
ENSBTAG00000001567	*LRRK1*	2'048	1'267	1'618	1'774	1'463	-0.278	0.729359386	1
ENSBTAG00000001568	*PPIC*	4'434	2'772	3'520	3'840	3'201	-0.263	0.736242352	1
ENSBTAG00000001570	*CSF2*	1	0	0	1	0		1	1
ENSBTAG00000001571	*LRRD1*	7	1	4	6	1	-2.392	0.851750348	1
ENSBTAG00000001572	*NSUN7*	58	39	48	50	45	-0.158	0.94239983	1

Ensembl gene ID	geneName	counts wildtype horn bud	counts polled horn bud	baseMean	baseMean wildtype horn bud	baseMean polled horn bud	log2FoldChange	pval	padj
ENSBTAG00000001573	*JMJD1C*	6'314	2'901	4'409	5'468	3'350	-0.707	0.362775385	1
ENSBTAG00000001574	*BT.25266*	983	713	837	851	823	-0.048	0.956259762	1
ENSBTAG00000001575	*BT.61778*	9'532	5'515	7'312	8'255	6'368	-0.374	0.626394283	1
ENSBTAG00000001576	*TMEM140*	213	307	269	184	354	0.942	0.341579256	1
ENSBTAG00000001578	*ADPGK*	2'232	1'821	2'018	1'933	2'103	0.121	0.877868156	1
ENSBTAG00000001579	*EIF2C2*	296	297	300	256	343	0.420	0.664555148	1
ENSBTAG00000001580	*CLGN*	283	338	318	245	390	0.671	0.482836251	1
ENSBTAG00000001582	*BT.47694*	1	2	2	1	2	1.415	0.981979269	1
ENSBTAG00000001585	*BT.56769*	5'972	2'105	3'801	5'172	2'431	-1.089	0.164477734	1
ENSBTAG00000001586	*BT.76892*	560	606	592	485	700	0.529	0.542582897	1
ENSBTAG00000001589	*TM9SF2*	10'489	8'532	9'468	9'084	9'852	0.117	0.877727086	1
ENSBTAG00000001592	*INSIG1*	1'271	778	1'000	1'101	898	-0.293	0.723569072	1
ENSBTAG00000001593	*OGFOD2*	381	390	390	330	450	0.449	0.626541213	1
ENSBTAG00000001594	*ARL6IP4*	1'369	1'286	1'335	1'186	1'485	0.325	0.687601396	1
ENSBTAG00000001595	*MT1E*	14	2	7	12	2	-2.392	0.704665469	1
ENSBTAG00000001597	*PITPNM2*	594	1'154	923	514	1'333	1.373	0.102864965	1
ENSBTAG00000001598	*MGC151537*	921	507	692	798	585	-0.446	0.602318471	1
ENSBTAG00000001600	*DCUN1D4*	339	234	282	294	270	-0.120	0.906774421	1
ENSBTAG00000001601	*PKM2*	18'054	34'969	28'007	15'635	40'379	1.369	0.076389148	1
ENSBTAG00000001602	*IL4R*	1'421	2'362	1'979	1'231	2'727	1.148	0.15133631	1
ENSBTAG00000001603	*BT.61469*	15	8	11	13	9	-0.492	0.927244877	1
ENSBTAG00000001604	*BT.9012*	141	239	199	122	276	1.176	0.269925894	1
ENSBTAG00000001605	*BT.63066*	13	0	6	11	0		0.551229012	1
ENSBTAG00000001607	*TMEM41B*	2'196	1'004	1'531	1'902	1'159	-0.714	0.375322031	1
ENSBTAG00000001608	*processed_pseudogene*	11	5	8	10	6	-0.722	0.917926652	1
ENSBTAG00000001609	*MAP2K6*	1'903	1'505	1'693	1'648	1'738	0.077	0.923583104	1
ENSBTAG00000001610	*BKB3*	27	13	19	23	15	-0.639	0.839610081	1
ENSBTAG00000001612	*LSM1*	872	737	803	755	851	0.172	0.837854362	1
ENSBTAG00000001614	*ZNF174*	1'087	1'063	1'084	941	1'227	0.383	0.640330267	1
ENSBTAG00000001616	*BT.25251*	1'760	2'364	2'127	1'524	2'730	0.841	0.289355625	1
ENSBTAG00000001617	*G2E3*	1'230	601	880	1'065	694	-0.618	0.459858943	1
ENSBTAG00000001618	*ALPK3*	354	398	383	307	460	0.584	0.527689522	1
ENSBTAG00000001619	*SERINC5*	956	1'112	1'056	828	1'284	0.633	0.441395509	1
ENSBTAG00000001621	*C17ORF57*	0	1	1	0	1	Inf	0.993540919	1
ENSBTAG00000001623	*pseudogene*	1	4	3	1	5	2.415	0.884888347	1
ENSBTAG00000001626	*CTPS*	2'271	1'531	1'867	1'967	1'768	-0.154	0.847575288	1
ENSBTAG00000001627	*protein_coding*	114	226	180	99	261	1.402	0.202162331	1
ENSBTAG00000001628	*SLFNL1*	12	14	13	10	16	0.637	0.876891072	1
ENSBTAG00000001629	*SCMH1*	666	817	760	577	943	0.710	0.401746294	1
ENSBTAG00000001631	*KIFC1*	1'063	1'391	1'263	921	1'606	0.803	0.323918313	1
ENSBTAG00000001632	*NBR1*	4'402	4'131	4'291	3'812	4'770	0.323	0.675618727	1
ENSBTAG00000001634	*BT.20328*	3'977	2'979	3'442	3'444	3'440	-0.002	0.999266188	1
ENSBTAG00000001635	*CUTA*	2'111	2'017	2'079	1'828	2'329	0.349	0.658300133	1
ENSBTAG00000001637	*FUNDC1*	832	391	586	721	451	-0.674	0.439713394	1
ENSBTAG00000001639	*TRPM1*	76	24	47	66	28	-1.248	0.491937234	1
ENSBTAG00000001640	*EPB41L1*	1'433	1'325	1'385	1'241	1'530	0.302	0.707861215	1
ENSBTAG00000001642	*TTC34*	89	79	84	77	91	0.243	0.867574688	1
ENSBTAG00000001644	*MDN1*	1'930	1'408	1'649	1'671	1'626	-0.040	0.961650371	1
ENSBTAG00000001645	*C20ORF4*	865	1'261	1'103	749	1'456	0.959	0.244288803	1
ENSBTAG00000001647	*PLEKHJ1*	1'696	1'176	1'413	1'469	1'358	-0.113	0.889667193	1
ENSBTAG00000001648	*RPL21*	15'914	10'104	12'725	13'782	11'667	-0.240	0.753500286	1
ENSBTAG00000001649	*ZFPM2*	469	333	395	406	385	-0.079	0.93502539	1
ENSBTAG00000001651	*SYF2*	894	665	771	774	768	-0.012	0.991263812	1
ENSBTAG00000001652	*SLCO3A1*	692	1'033	896	599	1'193	0.993	0.235658415	1
ENSBTAG00000001654	*SNTB2*	208	170	188	180	196	0.124	0.912050966	1
ENSBTAG00000001656	*C28H10orf57*	1'420	811	1'083	1'230	936	-0.393	0.632694854	1
ENSBTAG00000001657	*PICALM*	5'297	3'929	4'562	4'587	4'537	-0.016	0.984507341	1
ENSBTAG00000001658	*AKR1D1*	5	0	2	4	0		0.853268594	1
ENSBTAG00000001659	*VPS4A*	3'939	4'396	4'244	3'411	5'076	0.573	0.459346966	1
ENSBTAG00000001660	*SPTY2D1*	862	595	717	747	687	-0.120	0.890089916	1
ENSBTAG00000001662	*MGC139254*	1'467	699	1'039	1'270	807	-0.654	0.427791599	1
ENSBTAG00000001663	*PDF*	367	213	282	318	246	-0.370	0.707903621	1
ENSBTAG00000001665	*BT.56330*	1'282	1'559	1'455	1'110	1'800	0.697	0.387062087	1
ENSBTAG00000001666	*NIP7*	131	132	133	113	152	0.426	0.722136071	1
ENSBTAG00000001668	*WNT7A*	91	128	113	79	148	0.907	0.469542258	1
ENSBTAG00000001671	*RTF1*	2'810	2'544	2'686	2'434	2'938	0.272	0.728399522	1
ENSBTAG00000001672	*BT.103874*	3	5	4	3	6	1.152	0.920866718	1
ENSBTAG00000001673	*KIAA0528*	4'090	2'263	3'078	3'542	2'613	-0.439	0.574732874	1
ENSBTAG00000001675	*ITPKA*	18	11	14	16	13	-0.295	0.954936444	1
ENSBTAG00000001683	*FAM171B*	1'007	486	717	872	561	-0.636	0.455927705	1
ENSBTAG00000001686	*LRRC52*	1	0	0	1	0		1	1
ENSBTAG00000001687	*STC1*	300	188	238	260	217	-0.259	0.801953639	1

Ensembl gene ID	geneName	counts wildtype horn bud	counts polled horn bud	baseMean	baseMean wildtype horn bud	baseMean polled horn bud	log2FoldChange	pval	padj
ENSBTAG00000001692	*NDST1*	2'884	6'626	5'074	2'498	7'651	1.615	0.040510823	1
ENSBTAG00000001693	*RPAP1*	1'744	2'917	2'439	1'510	3'368	1.157	0.145031889	1
ENSBTAG00000001694	*TYRO3*	2'867	2'941	2'939	2'483	3'396	0.452	0.56293055	1
ENSBTAG00000001695	*pseudogene*	73	29	48	63	33	-0.917	0.608576302	1
ENSBTAG00000001696	*RBM46*	5	0	2	4	0		0.853268594	1
ENSBTAG00000001697	*TRA2B*	10'556	7'287	8'778	9'142	8'414	-0.120	0.876574955	1
ENSBTAG00000001698	*CHTF8*	4'086	4'328	4'268	3'539	4'998	0.498	0.520023526	1
ENSBTAG00000001700	*BT.56113*	6'220	5'658	5'960	5'387	6'533	0.278	0.716935712	1
ENSBTAG00000001702	*TMEM107*	538	316	415	466	365	-0.353	0.700974855	1
ENSBTAG00000001704	*BT.88057*	4	1	2	3	1	-1.585	0.952247636	1
ENSBTAG00000001706	*BT.61796*	109	30	65	94	35	-1.446	0.357966656	1
ENSBTAG00000001707	*BT.59061*	1'599	1'087	1'320	1'385	1'255	-0.142	0.862264562	1
ENSBTAG00000001708	*TTLL12*	3'667	5'314	4'656	3'176	6'136	0.950	0.221840685	1
ENSBTAG00000001710	*CMTM8*	355	181	258	307	209	-0.557	0.578455616	1
ENSBTAG00000001711	*C19H17orf59*	510	686	617	442	792	0.843	0.331043815	1
ENSBTAG00000001712	*CMTM7*	486	290	378	421	335	-0.330	0.723932458	1
ENSBTAG00000001714	*MFSD9*	225	257	246	195	297	0.607	0.547417537	1
ENSBTAG00000001717	*AURKB*	675	863	791	585	997	0.770	0.361885735	1
ENSBTAG00000001721	*GYG1*	1'449	1'014	1'213	1'255	1'171	-0.100	0.903772793	1
ENSBTAG00000001724	*BT.105959*	546	482	515	473	557	0.235	0.790862374	1
ENSBTAG00000001725	*CXCL10*	209	133	167	181	154	-0.237	0.835826167	1
ENSBTAG00000001727	*FAM213B*	3'086	3'643	3'440	2'673	4'207	0.654	0.401119087	1
ENSBTAG00000001728	*BT.26774*	9'275	3'355	5'953	8'032	3'874	-1.052	0.175553131	1
ENSBTAG00000001729	*DUSP10*	606	749	695	525	865	0.721	0.398961017	1
ENSBTAG00000001730	*BT.56902*	7	11	9	6	13	1.067	0.833633343	1
ENSBTAG00000001731	*GLYR1*	5'640	4'530	5'058	4'884	5'231	0.099	0.897534998	1
ENSBTAG00000001734	*MS4A10*	1	0	0	1	0		1	1
ENSBTAG00000001735	*ADAM11*	137	122	130	119	141	0.248	0.840066967	1
ENSBTAG00000001736	*UBN1*	1'200	1'492	1'381	1'039	1'723	0.729	0.367281583	1
ENSBTAG00000001737	*ZCCHC6*	1'971	1'008	1'435	1'707	1'164	-0.552	0.493866742	1
ENSBTAG00000001739	*BT.56646*	142	29	78	123	33	-1.877	0.201606901	1
ENSBTAG00000001740	*BT.41613*	2'140	2'602	2'429	1'853	3'005	0.697	0.376352772	1
ENSBTAG00000001741	*DLGAP4*	2'532	2'232	2'385	2'193	2'577	0.233	0.766537142	1
ENSBTAG00000001744	*BET1L*	3'902	4'533	4'307	3'379	5'234	0.631	0.415475808	1
ENSBTAG00000001745	*LUM*	259'257	210'233	233'640	224'523	242'756	0.113	0.8811568	1
ENSBTAG00000001747	*BT.31205*	688	539	609	596	622	0.063	0.943257863	1
ENSBTAG00000001748	*TRIM45*	1'049	488	736	908	563	-0.689	0.41812091	1
ENSBTAG00000001749	*SENP2*	4'100	2'107	2'992	3'551	2'433	-0.545	0.486115745	1
ENSBTAG00000001751	*CXXC4*	13	19	17	11	22	0.963	0.770262643	1
ENSBTAG00000001752	*FUCA2*	373	143	244	323	165	-0.968	0.340503534	1
ENSBTAG00000001753	*CLIC6*	32	8	18	28	9	-1.585	0.604898832	1
ENSBTAG00000001754	*AHCYL2*	2'040	1'048	1'488	1'767	1'210	-0.546	0.498076667	1
ENSBTAG00000001762	*RFXANK*	2'635	2'260	2'446	2'282	2'610	0.194	0.805041279	1
ENSBTAG00000001763	*NR2C2AP*	553	579	574	479	669	0.481	0.580938119	1
ENSBTAG00000001765	*HAPLN4*	10	1	5	9	1	-2.907	0.754028015	1
ENSBTAG00000001767	*B4GALT7*	1'810	2'177	2'041	1'568	2'514	0.681	0.390193617	1
ENSBTAG00000001768	*BT.21412*	276	183	225	239	211	-0.178	0.867133782	1
ENSBTAG00000001770	*ST8SIA5*	14	14	14	12	16	0.415	0.923601843	1
ENSBTAG00000001771	*DYRK1A*	1'535	1'120	1'311	1'329	1'293	-0.040	0.962574922	1
ENSBTAG00000001772	*PDCL2*	1	0	0	1	0		1	1
ENSBTAG00000001773	*C7ORF49*	364	331	349	315	382	0.278	0.768335462	1
ENSBTAG00000001774	*SPRY2*	1'465	869	1'136	1'269	1'003	-0.338	0.679798617	1
ENSBTAG00000001775	*RINL*	34	33	34	29	38	0.372	0.869906503	1
ENSBTAG00000001776	*BT.19604*	5'858	5'306	5'600	5'073	6'127	0.272	0.723180713	1
ENSBTAG00000001777	*RPLP2*	18'198	19'663	19'232	15'760	22'705	0.527	0.489415764	1
ENSBTAG00000001778	*BT.20178*	284	254	270	246	293	0.254	0.798716196	1
ENSBTAG00000001780	*SARS2*	963	1'354	1'199	834	1'563	0.907	0.267758042	1
ENSBTAG00000001781	*TOM40A*	1'316	1'617	1'503	1'140	1'867	0.712	0.376209825	1
ENSBTAG00000001782	*MRPS12*	632	692	673	547	799	0.546	0.523682094	1
ENSBTAG00000001783	*FBXO17*	387	269	323	335	311	-0.110	0.912185721	1
ENSBTAG00000001784	*LDLRAD2*	1	0	0	1	0		1	1
ENSBTAG00000001785	*TGM3*	28	12	19	24	14	-0.807	0.794158041	1
ENSBTAG00000001786	*AGPHD1*	571	345	446	495	398	-0.312	0.731552588	1
ENSBTAG00000001788	*BOVAGGRUS*	2'304	1'916	2'104	1'995	2'212	0.149	0.850170856	1
ENSBTAG00000001790	*SAFB2*	5'968	3'875	4'821	5'168	4'474	-0.208	0.788365703	1
ENSBTAG00000001792	*C19ORF70*	1'679	1'326	1'493	1'454	1'531	0.075	0.926165766	1
ENSBTAG00000001793	*HSD11B1L*	872	782	829	755	903	0.258	0.758366013	1
ENSBTAG00000001794	*RPL36*	1'197	1'500	1'384	1'037	1'732	0.741	0.359891195	1
ENSBTAG00000001795	*LONP1*	2'216	3'152	2'779	1'919	3'640	0.923	0.240805603	1
ENSBTAG00000001796	*CATSPERD*	920	489	681	797	565	-0.497	0.562401626	1
ENSBTAG00000001803	*FHL5*	5	2	3	4	2	-0.907	0.965043111	1
ENSBTAG00000001804	*GPR179*	23	6	13	20	7	-1.524	0.689823929	1

Ensembl gene ID	geneName	counts wildtype horn bud	counts polled horn bud	baseMean	baseMean wildtype horn bud	baseMean polled horn bud	log2FoldChange	pval	padj
ENSBTAG00000001805	*TWSG1*	11'235	7'534	9'215	9'730	8'700	-0.161	0.83358499	1
ENSBTAG00000001806	*IQCD*	91	50	68	79	58	-0.449	0.773828019	1
ENSBTAG00000001807	*AKAP8*	2'653	1'504	2'017	2'298	1'737	-0.404	0.610948699	1
ENSBTAG00000001808	*LACTB2*	438	221	317	379	255	-0.572	0.55145373	1
ENSBTAG00000001810	*SCAF11*	2'465	1'404	1'878	2'135	1'621	-0.397	0.618053193	1
ENSBTAG00000001812	*BT.36838*	21	7	13	18	8	-1.170	0.765843478	1
ENSBTAG00000001814	*PLXND1*	8'708	9'734	9'391	7'541	11'240	0.576	0.452409204	1
ENSBTAG00000001815	*BT.18132*	947	1'857	1'482	820	2'144	1.387	0.089140804	1
ENSBTAG00000001816	*BT.37567*	199	237	223	172	274	0.667	0.51786247	1
ENSBTAG00000001817	*TMEM161A*	1'385	1'333	1'369	1'199	1'539	0.360	0.655522204	1
ENSBTAG00000001818	*BT.61507*	179	216	202	155	249	0.686	0.516193092	1
ENSBTAG00000001821	*PRRC2C*	4'879	2'701	3'672	4'225	3'119	-0.438	0.573638695	1
ENSBTAG00000001823	*STC2*	44	17	29	38	20	-0.957	0.684136623	1
ENSBTAG00000001824	*BT.25025*	106	47	73	92	54	-0.758	0.611088895	1
ENSBTAG00000001825	*SP6*	71	118	99	61	136	1.148	0.383743463	1
ENSBTAG00000001826	*SASH1*	1'167	1'006	1'086	1'011	1'162	0.201	0.806407151	1
ENSBTAG00000001827	*GID8*	1'950	2'138	2'079	1'689	2'469	0.548	0.488675548	1
ENSBTAG00000001828	*SCRN2*	1'299	582	899	1'125	672	-0.743	0.373759638	1
ENSBTAG00000001830	*LRRC46*	39	16	26	34	18	-0.870	0.728438954	1
ENSBTAG00000001832	*MRPL10*	2'183	2'760	2'539	1'891	3'187	0.753	0.338642855	1
ENSBTAG00000001834	*PNKP*	1'207	1'271	1'256	1'045	1'468	0.490	0.546330809	1
ENSBTAG00000001835	*GJA1*	27'957	12'497	19'321	24'211	14'430	-0.747	0.329212824	1
ENSBTAG00000001836	*BT.75328*	2	22	14	2	25	3.874	0.336887961	1
ENSBTAG00000001838	*LRRC36*	16	4	9	14	5	-1.585	0.756102726	1
ENSBTAG00000001839	*OCIAD2*	1'507	629	1'016	1'305	726	-0.846	0.307360554	1
ENSBTAG00000001840	*INO80B*	880	1'119	1'027	762	1'292	0.762	0.356083624	1
ENSBTAG00000001842	*GSTM3*	1'501	1'166	1'323	1'300	1'346	0.051	0.950291591	1
ENSBTAG00000001843	*WBP1*	3'418	2'332	2'826	2'960	2'693	-0.137	0.862367612	1
ENSBTAG00000001844	*MOGS*	2'885	3'691	3'380	2'498	4'262	0.770	0.323881689	1
ENSBTAG00000001845	*EPS8L3*	2	0	1	2	0		0.974934741	1
ENSBTAG00000001847	*CNTLN*	1'227	331	722	1'063	382	-1.475	0.087743906	1
ENSBTAG00000001848	*KATNB1*	1'263	1'872	1'628	1'094	2'162	0.983	0.222058517	1
ENSBTAG00000001851	*BT.62268*	3	4	4	3	5	0.830	0.962490925	1
ENSBTAG00000001852	*BREH1*	118	85	100	102	98	-0.058	0.973870827	1
ENSBTAG00000001854	*CIAPIN1*	1'945	1'606	1'769	1'684	1'854	0.139	0.861497719	1
ENSBTAG00000001855	*COQ9*	1'739	2'135	1'986	1'506	2'465	0.711	0.37064287	1
ENSBTAG00000001856	*POLR2C*	3'932	3'202	3'551	3'405	3'697	0.119	0.87812686	1
ENSBTAG00000001857	*DOK4*	1'657	1'044	1'320	1'435	1'206	-0.251	0.757098993	1
ENSBTAG00000001858	*processed_pseudogene*	2'661	3'837	3'368	2'304	4'431	0.943	0.228582812	1
ENSBTAG00000001861	*SESTD1*	1'045	597	797	905	689	-0.393	0.641853467	1
ENSBTAG00000001864	*NR4A3*	39	6	20	34	7	-2.285	0.434300363	1
ENSBTAG00000001866	*BT.51193*	84	2	38	73	2	-4.977	0.041333156	1
ENSBTAG00000001867	*SACS*	1'402	1'014	1'193	1'214	1'171	-0.052	0.950457755	1
ENSBTAG00000001868	*PCYT2*	1'266	2'077	1'747	1'096	2'398	1.129	0.160312914	1
ENSBTAG00000001870	*CPNE6*	402	237	311	348	274	-0.347	0.719843053	1
ENSBTAG00000001871	*NRL*	1	1	1	1	1	0.415	1	1
ENSBTAG00000001872	*ORC6*	1'543	1'796	1'705	1'336	2'074	0.634	0.427373983	1
ENSBTAG00000001877	*HMGN5*	126	52	85	109	60	-0.862	0.539101485	1
ENSBTAG00000001879	*BT.58371*	273	158	209	236	182	-0.374	0.723948349	1
ENSBTAG00000001880	*VGLL1*	28	17	22	24	20	-0.305	0.926707752	1
ENSBTAG00000001881	*SLC10A1*	24	11	17	21	13	-0.710	0.836644854	1
ENSBTAG00000001882	*CD79A*	31	17	23	27	20	-0.452	0.876164144	1
ENSBTAG00000001885	*COPR5*	727	428	562	630	494	-0.349	0.690994947	1
ENSBTAG00000001886	*FAM122B*	271	171	216	235	197	-0.249	0.814247795	1
ENSBTAG00000001887	*BT.58902*	761	508	623	659	587	-0.168	0.847787328	1
ENSBTAG00000001888	*MED13L*	2'721	1'984	2'324	2'356	2'291	-0.041	0.960033844	1
ENSBTAG00000001889	*C22H3orf14*	261	101	171	226	117	-0.955	0.389627758	1
ENSBTAG00000001890	*BT.104559*	446	308	371	386	356	-0.119	0.901453705	1
ENSBTAG00000001892	*YAE1D1*	614	375	482	532	433	-0.296	0.741686147	1
ENSBTAG00000001894	*NEDD1*	2'720	2'060	2'367	2'356	2'379	0.014	0.985642225	1
ENSBTAG00000001895	*BT.17618*	889	507	678	770	585	-0.395	0.645330906	1
ENSBTAG00000001898	*CCDC112*	878	487	661	760	562	-0.435	0.613008155	1
ENSBTAG00000001899	*KCNH7*	14	0	6	12	0		0.520471303	1
ENSBTAG00000001900	*pseudogene*	36	58	49	31	67	1.103	0.53078159	1
ENSBTAG00000001901	*pseudogene*	4	25	16	3	29	3.059	0.372059915	1
ENSBTAG00000001902	*KIAA1731*	4'559	1'985	3'120	3'948	2'292	-0.785	0.316880194	1
ENSBTAG00000001903	*C2orf50*	3	0	1	3	0		0.936647693	1
ENSBTAG00000001904	*ZNF276*	1'771	1'406	1'579	1'534	1'624	0.082	0.918391119	1
ENSBTAG00000001906	*FANCA*	677	555	614	586	641	0.128	0.882857024	1
ENSBTAG00000001908	*BT.105088*	2'915	1'856	2'334	2'524	2'143	-0.236	0.764956005	1
ENSBTAG00000001911	*BT.89104*	31	72	55	27	83	1.631	0.332077134	1
ENSBTAG00000001912	*TRPV5*	1	8	5	1	9	3.415	0.697043054	1

Ensembl gene ID	geneName	counts wildtype horn bud	counts polled horn bud	baseMean	baseMean wildtype horn bud	baseMean polled horn bud	log2FoldChange	pval	padj
ENSBTAG00000001915	*APOH*	4	0	2	3	0		0.89545886	1
ENSBTAG00000001917	*C11ORF16*	12	2	6	10	2	-2.170	0.758651977	1
ENSBTAG00000001918	*STXBP5L*	21	10	15	18	12	-0.655	0.864027356	1
ENSBTAG00000001919	*ZNF652*	503	521	519	436	602	0.466	0.598035508	1
ENSBTAG00000001920	*POLQ*	135	96	114	117	111	-0.077	0.959430896	1
ENSBTAG00000001922	*AKIP1*	1'430	898	1'138	1'238	1'037	-0.256	0.755037986	1
ENSBTAG00000001926	*RAD23B*	6'527	5'930	6'250	5'653	6'847	0.277	0.718458722	1
ENSBTAG00000001927	*ATP6V1C2*	125	41	78	108	47	-1.193	0.41099054	1
ENSBTAG00000001928	*PDIA6*	10'782	8'311	9'467	9'337	9'597	0.040	0.95826181	1
ENSBTAG00000001931	*TRABD*	1'652	1'511	1'588	1'431	1'745	0.286	0.720299226	1
ENSBTAG00000001932	*MGC142781*	1'584	1'641	1'633	1'372	1'895	0.466	0.559849845	1
ENSBTAG00000001933	*PPP2R4*	4'002	6'155	5'287	3'466	7'107	1.036	0.182574279	1
ENSBTAG00000001936	*PCK1*	142	41	85	123	47	-1.377	0.32688897	1
ENSBTAG00000001937	*ANAPC11*	1'226	940	1'074	1'062	1'085	0.032	0.969597686	1
ENSBTAG00000001938	*CKS2*	1'658	1'124	1'367	1'436	1'298	-0.146	0.858059309	1
ENSBTAG00000001939	*SECISBP2*	1'509	822	1'128	1'307	949	-0.461	0.573611275	1
ENSBTAG00000001941	*SEMA4D*	782	967	897	677	1'117	0.721	0.387035254	1
ENSBTAG00000001942	*SNX27*	723	565	639	626	652	0.059	0.946217387	1
ENSBTAG00000001945	*BT.22399*	217	93	148	188	107	-0.807	0.485561222	1
ENSBTAG00000001947	*ZNF514*	71	37	52	61	43	-0.525	0.765499373	1
ENSBTAG00000001948	*VTI1B*	2'392	1'394	1'841	2'072	1'610	-0.364	0.647944898	1
ENSBTAG00000001949	*BT.55400*	627	421	515	543	486	-0.160	0.859210597	1
ENSBTAG00000001950	*BT.103133*	612	483	544	530	558	0.074	0.934588863	1
ENSBTAG00000001952	*PROM2*	187	231	214	162	267	0.720	0.489521412	1
ENSBTAG00000001956	*HINT3*	320	231	272	277	267	-0.055	0.960199686	1
ENSBTAG00000001957	*TRMT11*	492	236	349	426	273	-0.645	0.494052546	1
ENSBTAG00000001959	*TFAP2E*	187	226	211	162	261	0.688	0.510145252	1
ENSBTAG00000001961	*MAP1B*	1'328	927	1'110	1'150	1'070	-0.104	0.900942618	1
ENSBTAG00000001962	*MRPS27*	1'598	1'564	1'595	1'384	1'806	0.384	0.631140764	1
ENSBTAG00000001965	*ENPP7*	3	1	2	3	1	-1.170	0.985253077	1
ENSBTAG00000001966	*BT.47512*	16'416	7'562	11'474	14'217	8'732	-0.703	0.359369275	1
ENSBTAG00000001968	*TTC14*	6'199	2'033	3'858	5'368	2'348	-1.193	0.128409432	1
ENSBTAG00000001969	*CCDC39*	110	32	66	95	37	-1.366	0.379613131	1
ENSBTAG00000001975	*DRP2*	187	274	239	162	316	0.966	0.342257187	1
ENSBTAG00000001976	*PPP1R17*	18	0	8	16	0		0.412867528	1
ENSBTAG00000001977	*YIPF6*	2'053	1'824	1'942	1'778	2'106	0.244	0.757546314	1
ENSBTAG00000001979	*TAF7L*	18	6	11	16	7	-1.170	0.791784766	1
ENSBTAG00000001983	*SMAGP*	582	407	487	504	470	-0.101	0.912678102	1
ENSBTAG00000001985	*SRMS*	47	82	68	41	95	1.218	0.425089349	1
ENSBTAG00000001987	*HSPC321*	2'573	1'347	1'892	2'228	1'555	-0.519	0.514701926	1
ENSBTAG00000001988	*EIF3D*	8'290	9'338	8'981	7'179	10'783	0.587	0.444038576	1
ENSBTAG00000001992	*CYP51A1*	3'639	2'008	2'735	3'151	2'319	-0.443	0.572687967	1
ENSBTAG00000001994	*FAM187B*	1	2	2	1	2	1.415	0.981979269	1
ENSBTAG00000001996	*SNRNP70*	6'580	6'134	6'391	5'698	7'083	0.314	0.682633034	1
ENSBTAG00000001997	*HEATR7B2*	1	0	0	1	0		1	1
ENSBTAG00000001998	*ST6GALNAC1*	6	5	5	5	6	0.152	1	1
ENSBTAG00000001999	*MGC143285*	1'120	821	959	970	948	-0.033	0.970271892	1
ENSBTAG00000002000	*TIMM8B*	1'572	905	1'203	1'361	1'045	-0.382	0.640213698	1
ENSBTAG00000002001	*protein_coding*	10	5	7	9	6	-0.585	0.94357809	1
ENSBTAG00000002002	*BT.97864*	1'598	905	1'214	1'384	1'045	-0.405	0.619354149	1
ENSBTAG00000002004	*LLPH*	1'805	962	1'337	1'563	1'111	-0.493	0.543206548	1
ENSBTAG00000002006	*THBS1*	18'363	15'843	17'098	15'903	18'294	0.202	0.790166001	1
ENSBTAG00000002007	*PRG3*	2	0	1	2	0		0.974934741	1
ENSBTAG00000002009	*pseudogene*	102	299	217	88	345	1.967	0.064984789	1
ENSBTAG00000002010	*PI4K2A*	1'543	1'886	1'757	1'336	2'178	0.705	0.377508999	1
ENSBTAG00000002011	*KIAA0895*	77	32	52	67	37	-0.852	0.62333849	1
ENSBTAG00000002012	*FAM96A*	2'614	2'096	2'342	2'264	2'420	0.096	0.90225035	1
ENSBTAG00000002014	*SNX1*	4'909	3'825	4'334	4'251	4'417	0.055	0.942847166	1
ENSBTAG00000002015	*RPS6KA6*	1'586	614	1'041	1'374	709	-0.954	0.249210087	1
ENSBTAG00000002018	*EIF5A*	9'014	8'880	9'030	7'806	10'254	0.393	0.607008031	1
ENSBTAG00000002019	*SERPINB5*	953	1'377	1'208	825	1'590	0.946	0.247612274	1
ENSBTAG00000002020	*C5ORF41*	335	167	241	290	193	-0.589	0.562316029	1
ENSBTAG00000002021	*BT.102307*	605	519	562	524	599	0.194	0.825194528	1
ENSBTAG00000002023	*NOS1*	27	47	39	23	54	1.215	0.538169532	1
ENSBTAG00000002024	*ASB18*	0	2	1	0	2	Inf	0.939077559	1
ENSBTAG00000002025	*AMIGO2*	394	396	399	341	457	0.422	0.645754298	1
ENSBTAG00000002026	*RPL5*	18'043	19'598	19'128	15'626	22'630	0.534	0.48325165	1
ENSBTAG00000002027	*FAM167B*	493	183	319	427	211	-1.015	0.291279858	1
ENSBTAG00000002028	*MGC143035*	988	424	673	856	490	-0.805	0.348968135	1
ENSBTAG00000002029	*IGSF5*	14	5	9	12	6	-1.070	0.844263455	1
ENSBTAG00000002033	*UBXN2A*	375	264	315	325	305	-0.091	0.928063248	1
ENSBTAG00000002034	*MFSD2B*	85	67	75	74	77	0.072	0.969773395	1

Ensembl gene ID	geneName	counts wildtype horn bud	counts polled horn bud	baseMean	baseMean wildtype horn bud	baseMean polled horn bud	log2FoldChange	pval	padj
ENSBTAG00000002035	*ARL5C*	9	2	5	8	2	-1.755	0.844314416	1
ENSBTAG00000002036	*BT.37145*	1'285	1'275	1'293	1'113	1'472	0.404	0.617991873	1
ENSBTAG00000002037	*ATXN7L1*	468	412	441	405	476	0.231	0.799020228	1
ENSBTAG00000002038	*BT.49131*	56'016	51'176	53'802	48'511	59'093	0.285	0.707086855	1
ENSBTAG00000002039	*FRMD8*	1'138	1'194	1'182	986	1'379	0.484	0.552217463	1
ENSBTAG00000002041	*SASH3*	101	74	86	87	85	-0.034	0.991132283	1
ENSBTAG00000002042	*RIC8A*	5'001	4'860	4'971	4'331	5'612	0.374	0.627804925	1
ENSBTAG00000002044	*SIRT3*	328	177	244	284	204	-0.475	0.640229161	1
ENSBTAG00000002045	*BT.21635*	3'011	3'356	3'241	2'608	3'875	0.572	0.463624616	1
ENSBTAG00000002046	*COX8B*	557	625	602	482	722	0.581	0.502713586	1
ENSBTAG00000002048	*PTPN11*	1'617	1'811	1'746	1'400	2'091	0.579	0.468217401	1
ENSBTAG00000002049	*BT.58717*	2'988	2'776	2'897	2'588	3'205	0.309	0.692264548	1
ENSBTAG00000002050	*SELRC1*	230	170	198	199	196	-0.021	0.990505953	1
ENSBTAG00000002051	*ARAP1*	2'434	3'083	2'834	2'108	3'560	0.756	0.335283479	1
ENSBTAG00000002052	*PLOD1*	12'833	27'103	21'205	11'114	31'296	1.494	0.054143786	1
ENSBTAG00000002055	*ZFP28*	593	316	439	514	365	-0.493	0.587150371	1
ENSBTAG00000002056	*MARS2*	236	246	244	204	284	0.475	0.638586262	1
ENSBTAG00000002058	*ASF1A*	927	853	894	803	985	0.295	0.722990584	1
ENSBTAG00000002059	*MCM9*	934	622	764	809	718	-0.171	0.840955716	1
ENSBTAG00000002060	*BT.55679*	44'743	31'435	37'523	38'749	36'298	-0.094	0.901902849	1
ENSBTAG00000002062	*TRPA1*	430	83	234	372	96	-1.958	0.062054569	1
ENSBTAG00000002065	*TFAP2C*	1'210	785	977	1'048	906	-0.209	0.801571558	1
ENSBTAG00000002068	*TAGLN2*	6'181	3'516	4'706	5'353	4'060	-0.399	0.606467739	1
ENSBTAG00000002069	*BT.73422*	805	421	592	697	486	-0.520	0.550633063	1
ENSBTAG00000002070	*BT.54295*	284	390	348	246	450	0.873	0.354404578	1
ENSBTAG00000002072	*ERCC2*	1'572	2'954	2'386	1'361	3'411	1.325	0.096625757	1
ENSBTAG00000002073	*RAD17*	1'502	908	1'175	1'301	1'048	-0.311	0.70383806	1
ENSBTAG00000002075	*MME*	4'645	2'735	3'590	4'023	3'158	-0.349	0.654018985	1
ENSBTAG00000002076	*STAU2*	775	766	778	671	885	0.398	0.636664338	1
ENSBTAG00000002078	*KDM4A*	4'596	5'168	4'974	3'980	5'967	0.584	0.449572303	1
ENSBTAG00000002081	*BMPR1B*	437	394	417	378	455	0.266	0.771594063	1
ENSBTAG00000002082	*BT.95322*	27'071	16'861	21'457	23'444	19'469	-0.268	0.725344138	1
ENSBTAG00000002083	*AUTS2*	1'543	998	1'244	1'336	1'152	-0.214	0.793766965	1
ENSBTAG00000002084	*UNC5C*	211	146	176	183	169	-0.116	0.921383999	1
ENSBTAG00000002085	*BT.61828*	40	2	18	35	2	-3.907	0.248978493	1
ENSBTAG00000002086	*PRPF4B*	6'372	4'489	5'351	5'518	5'183	-0.090	0.907561593	1
ENSBTAG00000002087	*IL1F6*	1	0	0	1	0		1	1
ENSBTAG00000002089	*C6ORF146*	21	7	13	18	8	-1.170	0.765843478	1
ENSBTAG00000002090	*CPSF4*	973	816	892	843	942	0.161	0.846778072	1
ENSBTAG00000002092	*PI16*	9'840	4'330	6'761	8'522	5'000	-0.769	0.319024212	1
ENSBTAG00000002094	*ATP5J2*	3'999	3'101	3'522	3'463	3'581	0.048	0.950294092	1
ENSBTAG00000002095	*MEN1*	2'724	4'200	3'604	2'359	4'850	1.040	0.184410284	1
ENSBTAG00000002096	*FCGR3A*	106	42	70	92	48	-0.921	0.542920678	1
ENSBTAG00000002097	*TMC5*	13	9	11	11	10	-0.115	1	1
ENSBTAG00000002098	*CDC34*	2'112	2'169	2'167	1'829	2'505	0.453	0.565559407	1
ENSBTAG00000002100	*BT.31474*	1	0	0	1	0		1	1
ENSBTAG00000002101	*GDE1*	2'693	1'514	2'040	2'332	1'748	-0.416	0.60018586	1
ENSBTAG00000002103	*PLCG2*	264	165	210	229	191	-0.263	0.805417953	1
ENSBTAG00000002104	*RHPN1*	103	56	77	89	65	-0.464	0.753835641	1
ENSBTAG00000002105	*TEX261*	4'712	4'930	4'887	4'081	5'693	0.480	0.533875329	1
ENSBTAG00000002107	*FZD1*	4'683	2'985	3'751	4'056	3'447	-0.235	0.763172677	1
ENSBTAG00000002108	*YWHAQ*	18'972	13'621	16'079	16'430	15'728	-0.063	0.934833761	1
ENSBTAG00000002110	*DMGDH*	64	33	47	55	38	-0.541	0.770995834	1
ENSBTAG00000002112	*INSIG2*	1'739	968	1'312	1'506	1'118	-0.430	0.596124469	1
ENSBTAG00000002113	*ZNF41*	383	174	266	332	201	-0.723	0.467194481	1
ENSBTAG00000002115	*C20ORF96*	386	218	293	334	252	-0.409	0.675609782	1
ENSBTAG00000002116	*ZCCHC3*	3'207	2'642	2'914	2'777	3'051	0.135	0.861989199	1
ENSBTAG00000002117	*BT.97998*	384	252	312	333	291	-0.193	0.844147663	1
ENSBTAG00000002121	*CDK2AP2*	570	816	718	494	942	0.933	0.274776318	1
ENSBTAG00000002122	*FAM109A*	90	95	94	78	110	0.493	0.715200416	1
ENSBTAG00000002125	*BT.101003*	367	188	267	318	217	-0.550	0.580212405	1
ENSBTAG00000002126	*PFKFB2*	752	422	569	651	487	-0.418	0.632988959	1
ENSBTAG00000002127	*TCP11L2*	1'445	610	978	1'251	704	-0.829	0.318153323	1
ENSBTAG00000002128	*MPPE1*	2'086	1'740	1'908	1'807	2'009	0.153	0.846481849	1
ENSBTAG00000002129	*KLF5*	6'249	4'163	5'109	5'412	4'807	-0.171	0.825350369	1
ENSBTAG00000002130	*SMPD4*	3'554	3'632	3'636	3'078	4'194	0.446	0.565427911	1
ENSBTAG00000002132	*DRD3*	0	3	2	0	3	Inf	0.878646179	1
ENSBTAG00000002135	*CD69*	7	1	4	6	1	-2.392	0.851750348	1
ENSBTAG00000002136	*FCHO1*	196	44	110	170	51	-1.740	0.177525782	1
ENSBTAG00000002137	*SUGT1*	1'229	726	951	1'064	838	-0.344	0.678663922	1
ENSBTAG00000002138	*PADI1*	29	2	14	25	2	-3.443	0.390734455	1
ENSBTAG00000002139	*MIXL1*	9	12	11	8	14	0.830	0.858819499	1

Ensembl gene ID	geneName	counts wildtype horn bud	counts polled horn bud	baseMean	baseMean wildtype horn bud	baseMean polled horn bud	log2FoldChange	pval	padj
ENSBTAG00000002140	*IL3*	5	2	3	4	2	-0.907	0.965043111	1
ENSBTAG00000002143	*DTWD2*	76	56	65	66	65	-0.026	0.999479958	1
ENSBTAG00000002144	*ADRB2*	89	28	55	77	32	-1.253	0.457644612	1
ENSBTAG00000002145	*MMP17*	117	75	94	101	87	-0.227	0.873156264	1
ENSBTAG00000002147	*PPP4R2*	1'441	933	1'163	1'248	1'077	-0.212	0.796159372	1
ENSBTAG00000002148	*BT.20052*	850	1'001	946	736	1'156	0.651	0.432671094	1
ENSBTAG00000002151	*ASB15*	1	0	0	1	0		1	1
ENSBTAG00000002154	*GPR141*	8	2	5	7	2	-1.585	0.87391615	1
ENSBTAG00000002157	*BT.47472*	6	24	16	5	28	2.415	0.464285575	1
ENSBTAG00000002163	*PTGER2*	78	30	51	68	35	-0.963	0.580273764	1
ENSBTAG00000002164	*AXDND1*	12	9	10	10	10	0.000	1	1
ENSBTAG00000002166	*CRISP2*	23	8	15	20	9	-1.109	0.761253883	1
ENSBTAG00000002167	*NPHS2*	0	1	1	0	1	Inf	0.993540919	1
ENSBTAG00000002169	*pseudogene*	53	151	110	46	174	1.926	0.136236141	1
ENSBTAG00000002170	*NOVA1*	563	494	529	488	570	0.226	0.7977791	1
ENSBTAG00000002171	*FAM135A*	482	287	374	417	331	-0.333	0.721865002	1
ENSBTAG00000002175	*BT.61295*	1'950	1'465	1'690	1'689	1'692	0.002	0.997715094	1
ENSBTAG00000002176	*BT.43646*	3'875	3'448	3'669	3'356	3'981	0.247	0.750327747	1
ENSBTAG00000002177	*PIKFYVE*	496	304	390	430	351	-0.291	0.754223279	1
ENSBTAG00000002178	*PPL*	6'853	9'196	8'277	5'935	10'619	0.839	0.27573101	1
ENSBTAG00000002179	*C10H15ORF41*	562	554	563	487	640	0.394	0.651861169	1
ENSBTAG00000002180	*processed_pseudogene*	6	38	25	5	44	3.078	0.247557767	1
ENSBTAG00000002181	*KHDRBS3*	710	322	493	615	372	-0.726	0.416331498	1
ENSBTAG00000002182	*COX17*	1'368	874	1'097	1'185	1'009	-0.231	0.778907405	1
ENSBTAG00000002185	*RNF7*	2'738	1'337	1'958	2'371	1'544	-0.619	0.436335808	1
ENSBTAG00000002186	*NDN*	1'940	1'519	1'717	1'680	1'754	0.062	0.937933222	1
ENSBTAG00000002187	*SERINC3*	4'930	3'578	4'201	4'270	4'132	-0.047	0.952160905	1
ENSBTAG00000002188	*C16ORF89*	33	2	15	29	2	-3.629	0.331956808	1
ENSBTAG00000002189	*BT.76439*	686	689	695	594	796	0.421	0.621255773	1
ENSBTAG00000002190	*DOCK8*	359	205	274	311	237	-0.393	0.691919064	1
ENSBTAG00000002191	*RRP12*	2'100	2'981	2'630	1'819	3'442	0.920	0.243076062	1
ENSBTAG00000002192	*C14H8orf46*	182	23	92	158	27	-2.569	0.069917636	1
ENSBTAG00000002194	*GCOM1*	309	165	229	268	191	-0.490	0.63460399	1
ENSBTAG00000002196	*TCEB1*	2'313	1'747	2'010	2'003	2'017	0.010	0.989798798	1
ENSBTAG00000002199	*CORIN*	25	21	23	22	24	0.163	0.967026967	1
ENSBTAG00000002201	*NFXL1*	869	449	636	753	518	-0.538	0.533854407	1
ENSBTAG00000002202	*BT.84063*	742	843	808	643	973	0.599	0.476054806	1
ENSBTAG00000002203	*NDUFS2*	6'173	6'003	6'139	5'346	6'932	0.375	0.625736974	1
ENSBTAG00000002204	*pseudogene*	6	15	11	5	17	1.737	0.682513621	1
ENSBTAG00000002205	*CNGA1*	1	0	0	1	0		1	1
ENSBTAG00000002206	*ZC3H12B*	5	2	3	4	2	-0.907	0.965043111	1
ENSBTAG00000002209	*BT.76398*	4'318	4'285	4'344	3'739	4'948	0.404	0.601336814	1
ENSBTAG00000002210	*LAS1L*	9'720	4'824	6'994	8'418	5'570	-0.596	0.439320845	1
ENSBTAG00000002211	*MAPK8IP3*	2'771	3'322	3'118	2'400	3'836	0.677	0.386657551	1
ENSBTAG00000002214	*BT.68753*	75	3	34	65	3	-4.229	0.080714859	1
ENSBTAG00000002215	*GFPT2*	2'447	1'104	1'697	2'119	1'275	-0.733	0.360203903	1
ENSBTAG00000002219	*ATAD3*	1'211	1'549	1'419	1'049	1'789	0.770	0.340524342	1
ENSBTAG00000002220	*SPTLC1*	4'752	3'316	3'972	4'115	3'829	-0.104	0.894121299	1
ENSBTAG00000002223	*POP4*	829	608	710	718	702	-0.032	0.972301208	1
ENSBTAG00000002224	*UHRF1*	1'461	1'663	1'593	1'265	1'920	0.602	0.452606806	1
ENSBTAG00000002226	*FBXO31*	404	378	393	350	436	0.319	0.729438849	1
ENSBTAG00000002227	*LRIG3*	7'272	6'882	7'122	6'298	7'947	0.336	0.661582201	1
ENSBTAG00000002231	*SLC9A11*	6	3	4	5	3	-0.585	0.976586913	1
ENSBTAG00000002232	*TRPC1*	1'087	418	712	941	483	-0.964	0.260483759	1
ENSBTAG00000002233	*CPNE2*	864	304	550	748	351	-1.092	0.216822349	1
ENSBTAG00000002236	*TRPC4AP*	6'017	5'788	5'947	5'211	6'683	0.359	0.640379308	1
ENSBTAG00000002238	*ARL2*	1'565	1'498	1'543	1'355	1'730	0.352	0.660455895	1
ENSBTAG00000002240	*GPR18*	11	5	8	10	6	-0.722	0.917926652	1
ENSBTAG00000002242	*NIP30*	578	645	623	501	745	0.573	0.506873573	1
ENSBTAG00000002243	*SNX15*	698	749	735	604	865	0.517	0.542290391	1
ENSBTAG00000002248	*SAC3D1*	68	131	105	59	151	1.361	0.293032315	1
ENSBTAG00000002249	*NAALADL1*	181	200	194	157	231	0.559	0.600990135	1
ENSBTAG00000002251	*C12ORF63*	20	7	13	17	8	-1.100	0.786059398	1
ENSBTAG00000002252	*BT.68771*	0	1	1	0	1	Inf	0.993540919	1
ENSBTAG00000002255	*BHMT*	1	0	0	1	0		1	1
ENSBTAG00000002256	*YEATS4*	802	640	717	695	739	0.090	0.916958064	1
ENSBTAG00000002257	*KLK11*	309	141	215	268	163	-0.717	0.492559567	1
ENSBTAG00000002258	*APOA1*	5'389	2'019	3'499	4'667	2'331	-1.001	0.201496932	1
ENSBTAG00000002259	*protein_coding*	21	8	14	18	9	-0.977	0.800212154	1
ENSBTAG00000002260	*NCAPD3*	1'368	1'393	1'397	1'185	1'608	0.441	0.584144372	1
ENSBTAG00000002266	*NPL*	94	102	100	81	118	0.533	0.686192302	1
ENSBTAG00000002267	*ANKRD40*	2'344	2'168	2'267	2'030	2'503	0.302	0.700737038	1

Ensembl gene ID	geneName	counts wildtype horn bud	counts polled horn bud	baseMean	baseMean wildtype horn bud	baseMean polled horn bud	log2FoldChange	pval	padj
ENSBTAG00000002271	*CDADC1*	607	588	602	526	679	0.369	0.670223835	1
ENSBTAG00000002272	*KCTD18*	799	594	689	692	686	-0.013	0.990826128	1
ENSBTAG00000002275	*PTP4A1*	3'239	2'082	2'605	2'805	2'404	-0.223	0.777435385	1
ENSBTAG00000002277	*MLLT1*	1'370	2'739	2'175	1'186	3'163	1.415	0.077776911	1
ENSBTAG00000002278	*FBN1*	32'646	41'069	37'847	28'272	47'422	0.746	0.327785774	1
ENSBTAG00000002279	*LUC7L3*	13'908	4'854	8'825	12'045	5'605	-1.104	0.153696546	1
ENSBTAG00000002280	*BT.102163*	4'533	3'744	4'124	3'926	4'323	0.139	0.856922869	1
ENSBTAG00000002281	*ZNF354C*	676	507	585	585	585	0.000	1	1
ENSBTAG00000002282	*EIF1*	7'436	5'427	6'353	6'440	6'267	-0.039	0.960064611	1
ENSBTAG00000002283	*BT.105583*	894	1'369	1'178	774	1'581	1.030	0.209613554	1
ENSBTAG00000002286	*DNAJC18*	4'640	4'185	4'425	4'018	4'832	0.266	0.730245368	1
ENSBTAG00000002287	*CHD9*	2'298	1'551	1'891	1'990	1'791	-0.152	0.849139015	1
ENSBTAG00000002290	*BT.97261*	4	0	2	3	0		0.89545886	1
ENSBTAG00000002291	*ZBTB41*	107	46	73	93	53	-0.803	0.590274342	1
ENSBTAG00000002292	*SMARCA1*	9'690	4'354	6'710	8'392	5'028	-0.739	0.338227352	1
ENSBTAG00000002293	*ARL5A*	769	574	664	666	663	-0.007	0.996367068	1
ENSBTAG00000002295	*ATF2*	1'831	1'323	1'557	1'586	1'528	-0.054	0.947996449	1
ENSBTAG00000002297	*CACNB4*	12	1	6	10	1	-3.170	0.692545189	1
ENSBTAG00000002298	*CKAP2L*	779	810	805	675	935	0.471	0.57486987	1
ENSBTAG00000002299	*SEL1L3*	554	374	456	480	432	-0.152	0.868698437	1
ENSBTAG00000002300	*BT.93330*	81	75	78	70	87	0.304	0.8375644	1
ENSBTAG00000002302	*CD59*	2'900	1'344	2'032	2'511	1'552	-0.694	0.382116104	1
ENSBTAG00000002303	*GLG1*	3'001	4'725	4'027	2'599	5'456	1.070	0.171159444	1
ENSBTAG00000002305	*PCSK4*	251	318	292	217	367	0.756	0.436813131	1
ENSBTAG00000002306	*UTP11L*	2'260	1'455	1'819	1'957	1'680	-0.220	0.782814412	1
ENSBTAG00000002309	*BT.27313*	1'402	313	788	1'214	361	-1.748	0.042789212	1
ENSBTAG00000002311	*REEP6*	45	42	44	39	48	0.316	0.873626025	1
ENSBTAG00000002313	*BT.66624*	516	570	553	447	658	0.559	0.523809208	1
ENSBTAG00000002315	*RNF34*	874	973	940	757	1'124	0.570	0.492052093	1
ENSBTAG00000002316	*NIPA1*	64	40	51	55	46	-0.263	0.889778504	1
ENSBTAG00000002321	*AMT*	6'435	3'282	4'681	5'573	3'790	-0.556	0.472792104	1
ENSBTAG00000002323	*BT.24505*	4'102	2'348	3'132	3'552	2'711	-0.390	0.617960149	1
ENSBTAG00000002326	*LGALS3*	4'698	5'985	5'490	4'069	6'911	0.764	0.3229455	1
ENSBTAG00000002327	*TMEM208*	2'073	1'475	1'749	1'795	1'703	-0.076	0.925386881	1
ENSBTAG00000002328	*KDM2B*	1'253	1'069	1'160	1'085	1'234	0.186	0.819725595	1
ENSBTAG00000002329	*ASAP2*	962	660	798	833	762	-0.129	0.880487767	1
ENSBTAG00000002331	*DLGAP5*	945	809	876	818	934	0.191	0.819156566	1
ENSBTAG00000002332	*LSM5*	2'051	1'117	1'533	1'776	1'290	-0.462	0.565951274	1
ENSBTAG00000002333	*HOP*	1'514	375	872	1'311	433	-1.598	0.060493657	1
ENSBTAG00000002336	*RCOR1*	501	434	468	434	501	0.208	0.817392426	1
ENSBTAG00000002340	*STEAP4*	55	14	32	48	16	-1.559	0.481455921	1
ENSBTAG00000002341	*ETS1*	1'260	992	1'118	1'091	1'145	0.070	0.932125752	1
ENSBTAG00000002344	*ORAI3*	1'280	1'748	1'563	1'109	2'018	0.865	0.282749278	1
ENSBTAG00000002345	*SETD1A*	1'723	2'666	2'285	1'492	3'078	1.045	0.188267734	1
ENSBTAG00000002346	*HSD3B7*	763	775	778	661	895	0.438	0.603735618	1
ENSBTAG00000002347	*MAST3*	597	844	746	517	975	0.915	0.282219191	1
ENSBTAG00000002348	*SLC4A4*	89	34	58	77	39	-0.973	0.553114973	1
ENSBTAG00000002349	*STX1B*	278	156	210	241	180	-0.419	0.691697901	1
ENSBTAG00000002350	*PIK3R2*	8'137	13'292	11'198	7'047	15'348	1.123	0.145693293	1
ENSBTAG00000002352	*GGA2*	3'346	3'630	3'545	2'898	4'192	0.533	0.493481829	1
ENSBTAG00000002354	*pseudogene*	2	4	3	2	5	1.415	0.927250049	1
ENSBTAG00000002355	*protein_coding*	2	3	3	2	3	1.000	0.97171833	1
ENSBTAG00000002356	*BT.22438*	115	98	106	100	113	0.184	0.891086787	1
ENSBTAG00000002357	*TICAM2*	1'045	1'200	1'145	905	1'386	0.615	0.452172411	1
ENSBTAG00000002361	*TMCC2*	487	481	489	422	555	0.397	0.655763418	1
ENSBTAG00000002362	*APOLD1*	859	252	517	744	291	-1.354	0.130589471	1
ENSBTAG00000002363	*SESN2*	236	342	300	204	395	0.950	0.327238946	1
ENSBTAG00000002367	*HCFC1R1*	1'560	1'585	1'591	1'351	1'830	0.438	0.584158525	1
ENSBTAG00000002368	*BT.88263*	1'167	712	916	1'011	822	-0.298	0.721227916	1
ENSBTAG00000002369	*TMEM217*	2	1	1	2	1	-0.585	1	1
ENSBTAG00000002370	*ZNF792*	247	139	187	214	161	-0.414	0.703500778	1
ENSBTAG00000002374	*ARMC5*	498	705	623	431	814	0.917	0.290462814	1
ENSBTAG00000002376	*PPIL1*	1'378	1'016	1'183	1'193	1'173	-0.025	0.977733583	1
ENSBTAG00000002377	*PSMB2*	3'043	3'113	3'115	2'635	3'595	0.448	0.565693214	1
ENSBTAG00000002378	*NUCB1*	29'050	25'003	27'015	25'158	28'871	0.199	0.793276542	1
ENSBTAG00000002381	*ZDHHC5*	2'461	2'504	2'511	2'131	2'891	0.440	0.575021381	1
ENSBTAG00000002382	*DDX10*	808	944	895	700	1'090	0.639	0.442892712	1
ENSBTAG00000002385	*SLC35E4*	56	30	42	48	35	-0.485	0.80749413	1
ENSBTAG00000002389	*MED19*	1'213	922	1'058	1'050	1'065	0.019	0.981847432	1
ENSBTAG00000002391	*TGFB1I1*	1'411	1'905	1'711	1'222	2'200	0.848	0.289735737	1
ENSBTAG00000002392	*ANK2*	3'136	2'337	2'707	2'716	2'699	-0.009	0.991828353	1
ENSBTAG00000002393	*C6ORF89*	1'260	1'453	1'384	1'091	1'678	0.621	0.442241014	1

Ensembl gene ID	geneName	counts wildtype horn bud	counts polled horn bud	baseMean	baseMean wildtype horn bud	baseMean polled horn bud	log2FoldChange	pval	padj
ENSBTAG00000002394	*TMEM44*	1'410	948	1'158	1'221	1'095	-0.158	0.848192827	1
ENSBTAG00000002395	*HPS3*	1'267	444	805	1'097	513	-1.098	0.195511849	1
ENSBTAG00000002398	*BT.92826*	1'316	753	1'005	1'140	869	-0.390	0.636978392	1
ENSBTAG00000002401	*NPFF*	10	6	8	9	7	-0.322	0.979718036	1
ENSBTAG00000002402	*BT.4977*	7'637	13'787	11'267	6'614	15'920	1.267	0.101676236	1
ENSBTAG00000002404	*TMX2*	2'060	2'096	2'102	1'784	2'420	0.440	0.577538731	1
ENSBTAG00000002407	*TDRD5*	1	1	1	1	1	0.415	1	1
ENSBTAG00000002408	*FHAD1*	38	10	22	33	12	-1.511	0.579359679	1
ENSBTAG00000002411	*CTNND1*	9'088	7'737	8'402	7'870	8'934	0.183	0.810727952	1
ENSBTAG00000002412	*CYB5B*	1'279	1'226	1'262	1'108	1'416	0.354	0.66238525	1
ENSBTAG00000002413	*MCAT*	1'108	1'482	1'335	960	1'711	0.835	0.303811815	1
ENSBTAG00000002415	*BT.81335*	5'069	2'587	3'689	4'390	2'987	-0.555	0.475771663	1
ENSBTAG00000002416	*protein_coding*	53	66	61	46	76	0.732	0.647501135	1
ENSBTAG00000002417	*RAB30*	316	259	286	274	299	0.128	0.8979999	1
ENSBTAG00000002418	*C15ORF42*	410	461	444	355	532	0.584	0.517898322	1
ENSBTAG00000002419	*PIGM*	277	206	239	240	238	-0.012	0.996038051	1
ENSBTAG00000002422	*BCL9*	1'887	2'256	2'120	1'634	2'605	0.673	0.395485805	1
ENSBTAG00000002423	*PSMD12*	2'495	1'756	2'094	2'161	2'028	-0.092	0.908829556	1
ENSBTAG00000002425	*BT.61065*	305	97	188	264	112	-1.238	0.254592451	1
ENSBTAG00000002427	*pseudogene*	2	0	1	2	0		0.974934741	1
ENSBTAG00000002428	*PPA2*	1'286	985	1'126	1'114	1'137	0.030	0.97087604	1
ENSBTAG00000002430	*COL17A1*	17'195	25'011	21'886	14'891	28'880	0.956	0.212468706	1
ENSBTAG00000002431	*TRIM24*	1'559	736	1'100	1'350	850	-0.668	0.416509839	1
ENSBTAG00000002432	*ABHD13*	410	336	372	355	388	0.128	0.89233193	1
ENSBTAG00000002434	*WDR18*	612	831	745	530	960	0.856	0.313618054	1
ENSBTAG00000002435	*PTPRE*	319	257	287	276	297	0.103	0.918347719	1
ENSBTAG00000002440	*KIF7*	3'205	3'211	3'242	2'776	3'708	0.418	0.591599481	1
ENSBTAG00000002444	*MKI67*	2'223	3'141	2'776	1'925	3'627	0.914	0.24566847	1
ENSBTAG00000002445	*ZNF783*	216	370	307	187	427	1.192	0.218912595	1
ENSBTAG00000002447	*C19ORF44*	243	134	183	210	155	-0.444	0.685329149	1
ENSBTAG00000002448	*SNTG1*	19	4	11	16	5	-1.833	0.688634464	1
ENSBTAG00000002450	*FAM111A*	19	37	30	16	43	1.377	0.545853855	1
ENSBTAG00000002451	*TTC39A*	441	335	384	382	387	0.018	0.986576141	1
ENSBTAG00000002452	*ASCC2*	2'022	2'428	2'277	1'751	2'804	0.679	0.38980384	1
ENSBTAG00000002454	*ALG2*	757	391	554	656	451	-0.538	0.540332964	1
ENSBTAG00000002455	*KLHL8*	829	507	652	718	585	-0.294	0.733369037	1
ENSBTAG00000002457	*SEC61B*	7'497	5'457	6'397	6'493	6'301	-0.043	0.956080431	1
ENSBTAG00000002458	*NUP155*	2'623	1'346	1'913	2'272	1'554	-0.548	0.491447355	1
ENSBTAG00000002460	*ACTL6A*	1'598	1'470	1'541	1'384	1'697	0.295	0.713036923	1
ENSBTAG00000002462	*BT.24917*	1'433	669	1'007	1'241	772	-0.684	0.408524419	1
ENSBTAG00000002463	*NDUFB5*	3'452	2'816	3'121	2'990	3'252	0.121	0.876039343	1
ENSBTAG00000002464	*PLCH2*	973	2'133	1'653	843	2'463	1.547	0.057421261	1
ENSBTAG00000002467	*FAM173A*	818	520	654	708	600	-0.239	0.782846772	1
ENSBTAG00000002469	*BT.105935*	850	500	657	736	577	-0.350	0.6843536	1
ENSBTAG00000002470	*CCDC78*	303	198	246	262	229	-0.199	0.847623666	1
ENSBTAG00000002472	*CASP9*	1'169	557	828	1'012	643	-0.654	0.436529049	1
ENSBTAG00000002473	*ANGPTL4*	1'472	2'308	1'970	1'275	2'665	1.064	0.183016605	1
ENSBTAG00000002474	*MAD1L1*	413	699	582	358	807	1.174	0.181025008	1
ENSBTAG00000002475	*RAB11B*	3'606	4'196	3'984	3'123	4'845	0.634	0.41450915	1
ENSBTAG00000002476	*DNAJC16*	1'560	1'064	1'290	1'351	1'229	-0.137	0.867123301	1
ENSBTAG00000002477	*FTSJ2*	1'103	341	674	955	394	-1.279	0.139974264	1
ENSBTAG00000002478	*BT.104930*	8	2	5	7	2	-1.585	0.87391615	1
ENSBTAG00000002479	*BT.24745*	1'461	1'805	1'675	1'265	2'084	0.720	0.368336828	1
ENSBTAG00000002480	*NUDT1*	181	195	191	157	225	0.523	0.626493266	1
ENSBTAG00000002481	*HAGHL*	728	492	599	630	568	-0.150	0.864712931	1
ENSBTAG00000002483	*HES5*	94	133	117	81	154	0.916	0.459960851	1
ENSBTAG00000002484	*NARFL*	1'365	1'732	1'591	1'182	2'000	0.759	0.344780177	1
ENSBTAG00000002485	*TNS1*	4'948	10'525	8'219	4'285	12'153	1.504	0.054132721	1
ENSBTAG00000002487	*UBE3A*	6'562	4'398	5'381	5'683	5'078	-0.162	0.834010639	1
ENSBTAG00000002488	*BT.36084*	30	28	29	26	32	0.316	0.902058199	1
ENSBTAG00000002490	*CHPT1*	649	211	403	562	244	-1.206	0.19293223	1
ENSBTAG00000002493	*VPS35*	5'948	3'775	4'755	5'151	4'359	-0.241	0.75584905	1
ENSBTAG00000002495	*BRD4*	2'569	3'619	3'202	2'225	4'179	0.909	0.245954515	1
ENSBTAG00000002497	*ELOVL2*	73	35	52	63	40	-0.646	0.712044591	1
ENSBTAG00000002500	*FBXL15*	380	357	371	329	412	0.325	0.72729089	1
ENSBTAG00000002501	*CUEDC2*	1'430	1'408	1'432	1'238	1'626	0.393	0.625528697	1
ENSBTAG00000002503	*BSCL2*	917	1'001	975	794	1'156	0.541	0.512511263	1
ENSBTAG00000002504	*IREB2*	1'269	1'200	1'242	1'099	1'386	0.334	0.680301578	1
ENSBTAG00000002505	*GPR3*	3	10	7	3	12	2.152	0.727540088	1
ENSBTAG00000002507	*ATP5A1*	16'861	10'874	13'579	14'602	12'556	-0.218	0.775943285	1
ENSBTAG00000002508	*GNG3*	264	64	151	229	74	-1.629	0.161643734	1
ENSBTAG00000002510	*HNRNPUL2*	7'507	6'266	6'868	6'501	7'235	0.154	0.840103503	1

Ensembl gene ID	geneName	counts wildtype horn bud	counts polled horn bud	baseMean	baseMean wildtype horn bud	baseMean polled horn bud	log2FoldChange	pval	padj
ENSBTAG00000002512	*HAUS1*	747	429	571	647	495	-0.385	0.660342102	1
ENSBTAG00000002515	*DNAAF3*	40	1	18	35	1	-4.907	0.190349621	1
ENSBTAG00000002516	*BT.65196*	598	633	624	518	731	0.497	0.56467038	1
ENSBTAG00000002518	*NKX6-1*	6	1	3	5	1	-2.170	0.885240772	1
ENSBTAG00000002520	*CUGBP1*	1'310	1'040	1'168	1'134	1'201	0.082	0.920119961	1
ENSBTAG00000002524	*MRI1*	689	937	839	597	1'082	0.859	0.306919734	1
ENSBTAG00000002525	*C7H19orf53*	2'376	2'571	2'513	2'058	2'969	0.529	0.500777738	1
ENSBTAG00000002526	*BDH2*	2'017	1'249	1'594	1'747	1'442	-0.276	0.730844923	1
ENSBTAG00000002527	*ZSWIM4*	288	496	411	249	573	1.199	0.193082769	1
ENSBTAG00000002528	*EIF3A*	10'405	8'294	9'294	9'011	9'577	0.088	0.907955772	1
ENSBTAG00000002531	*ARHGAP10*	1'523	1'959	1'791	1'319	2'262	0.778	0.329773923	1
ENSBTAG00000002534	*EEF1E1*	862	762	813	747	880	0.237	0.777720756	1
ENSBTAG00000002539	*BT.56496*	2'544	3'812	3'302	2'203	4'402	0.998	0.203053575	1
ENSBTAG00000002542	*KRCC1*	3'020	1'566	2'212	2'615	1'808	-0.532	0.500863427	1
ENSBTAG00000002549	*PTMA*	15'695	24'421	20'896	13'592	28'199	1.053	0.170397384	1
ENSBTAG00000002550	*pseudogene*	6'954	7'930	7'590	6'022	9'157	0.605	0.43132513	1
ENSBTAG00000002551	*KCNJ8*	2'586	1'458	1'962	2'240	1'684	-0.412	0.604408964	1
ENSBTAG00000002554	*BT.44028*	12'509	7'004	9'460	10'833	8'088	-0.422	0.582449868	1
ENSBTAG00000002555	*PDZK1IP1*	39	6	20	34	7	-2.285	0.434300363	1
ENSBTAG00000002557	*MRPL19*	1'275	861	1'049	1'104	994	-0.151	0.855406832	1
ENSBTAG00000002561	*CCDC120*	1'425	1'407	1'429	1'234	1'625	0.397	0.622033134	1
ENSBTAG00000002562	*PRAF2*	3'065	2'913	3'009	2'654	3'364	0.342	0.661253	1
ENSBTAG00000002563	*WDR45*	1'152	1'125	1'148	998	1'299	0.381	0.640704125	1
ENSBTAG00000002564	*DLG4*	2'090	2'288	2'226	1'810	2'642	0.546	0.489251349	1
ENSBTAG00000002565	*protein_coding*	4	1	2	3	1	-1.585	0.952247636	1
ENSBTAG00000002566	*BT.30905*	1'698	1'771	1'758	1'471	2'045	0.476	0.550284823	1
ENSBTAG00000002568	*GPKOW*	1'062	1'245	1'179	920	1'438	0.644	0.429767926	1
ENSBTAG00000002570	*KLRAQ1*	856	411	608	741	475	-0.643	0.458934668	1
ENSBTAG00000002571	*PDCD2L*	280	176	223	242	203	-0.255	0.808552292	1
ENSBTAG00000002573	*UBA2*	4'456	3'307	3'839	3'859	3'819	-0.015	0.985439283	1
ENSBTAG00000002575	*SSU72*	1'150	993	1'071	996	1'147	0.203	0.804333696	1
ENSBTAG00000002576	*GLDN*	16	0	7	14	0		0.463690882	1
ENSBTAG00000002578	*ZMYND11*	11'839	7'478	9'444	10'253	8'635	-0.248	0.746723214	1
ENSBTAG00000002579	*FBL*	2'418	3'266	2'933	2'094	3'771	0.849	0.279583578	1
ENSBTAG00000002580	*MYH14*	1'561	3'498	2'696	1'352	4'039	1.579	0.048240393	1
ENSBTAG00000002581	*BOLA-DYA*	95	23	54	82	27	-1.631	0.337746585	1
ENSBTAG00000002582	*LYG2*	5	5	5	4	6	0.415	0.985023983	1
ENSBTAG00000002583	*DNALI1*	122	55	85	106	64	-0.734	0.601361337	1
ENSBTAG00000002585	*SLC16A2*	757	1'761	1'345	656	2'033	1.633	0.047883053	1
ENSBTAG00000002586	*BT.75685*	14'874	12'585	13'707	12'881	14'532	0.174	0.818974014	1
ENSBTAG00000002590	*EXOSC10*	4'007	2'542	3'203	3'470	2'935	-0.242	0.757420671	1
ENSBTAG00000002591	*GLTP*	4'130	2'245	3'084	3'577	2'592	-0.464	0.552658713	1
ENSBTAG00000002594	*ZNF436*	982	1'038	1'025	850	1'199	0.495	0.547644573	1
ENSBTAG00000002595	*ABCB5*	0	1	1	0	1	Inf	0.993540919	1
ENSBTAG00000002596	*VSTM1*	43	21	31	37	24	-0.619	0.790045288	1
ENSBTAG00000002599	*MGARP*	45	13	27	39	15	-1.376	0.570833419	1
ENSBTAG00000002600	*EFCAB7*	1'250	605	891	1'083	699	-0.632	0.449518294	1
ENSBTAG00000002603	*PRPF39*	8'719	2'812	5'399	7'551	3'247	-1.218	0.118661691	1
ENSBTAG00000002604	*SP8*	20	0	9	17	0		0.367514426	1
ENSBTAG00000002605	*protein_coding*	222	277	256	192	320	0.734	0.462671457	1
ENSBTAG00000002606	*LGR4*	1'917	2'310	2'164	1'660	2'667	0.684	0.387233348	1
ENSBTAG00000002608	*BT.20352*	14'611	12'946	13'801	12'653	14'949	0.240	0.751953201	1
ENSBTAG00000002609	*BT.49016*	1	1	1	1	1	0.415	1	1
ENSBTAG00000002610	*FKBP3*	4'001	3'044	3'490	3'465	3'515	0.021	0.978531947	1
ENSBTAG00000002612	*BT.103231*	681	272	452	590	314	-0.909	0.315562686	1
ENSBTAG00000002613	*MIS18BP1*	879	467	650	761	539	-0.497	0.563886443	1
ENSBTAG00000002614	*RUFY2*	891	406	620	772	469	-0.719	0.407076448	1
ENSBTAG00000002615	*LONRF3*	43	38	41	37	44	0.237	0.912715801	1
ENSBTAG00000002620	*DNA2*	275	191	229	238	221	-0.111	0.918727527	1
ENSBTAG00000002623	*SAMSN1*	85	39	59	74	45	-0.709	0.664692061	1
ENSBTAG00000002625	*MAP3K7*	2'857	1'450	2'074	2'474	1'674	-0.563	0.477471162	1
ENSBTAG00000002626	*ARHGAP6*	415	305	356	359	352	-0.029	0.979207158	1
ENSBTAG00000002627	*ZRANB2*	4'627	2'018	3'169	4'007	2'330	-0.782	0.318161191	1
ENSBTAG00000002628	*SLC44A2*	8'858	9'089	9'083	7'671	10'495	0.452	0.554646822	1
ENSBTAG00000002629	*C19ORF45*	4	2	3	3	2	-0.585	0.996239345	1
ENSBTAG00000002630	*MKL1*	1'121	2'084	1'689	971	2'406	1.310	0.105387025	1
ENSBTAG00000002631	*SCNN1A*	182	73	121	158	84	-0.903	0.46404952	1
ENSBTAG00000002633	*BT.105011*	4'880	8'644	7'104	4'226	9'981	1.240	0.11069881	1
ENSBTAG00000002634	*TMEM115*	2'493	3'325	2'999	2'159	3'839	0.831	0.289597818	1
ENSBTAG00000002635	*PGLYRP1*	54	96	79	47	111	1.245	0.386082612	1
ENSBTAG00000002639	*ZDHHC7*	849	1'100	1'003	735	1'270	0.789	0.340332226	1
ENSBTAG00000002640	*KALRN*	1'059	578	792	917	667	-0.459	0.587105412	1

Ensembl gene ID	geneName	counts wildtype horn bud	counts polled horn bud	baseMean	baseMean wildtype horn bud	baseMean polled horn bud	log2FoldChange	pval	padj
ENSBTAG00000002641	*BT.62656*	1'138	1'024	1'084	986	1'182	0.263	0.748457266	1
ENSBTAG00000002643	*VPS52*	5'761	4'543	5'117	4'989	5'246	0.072	0.924773331	1
ENSBTAG00000002644	*KCNQ4*	236	248	245	204	286	0.487	0.629934369	1
ENSBTAG00000002645	*SH3BP5L*	1'116	1'401	1'292	966	1'618	0.743	0.360225561	1
ENSBTAG00000002646	*BT.3906*	8'907	7'278	8'059	7'714	8'404	0.124	0.871235101	1
ENSBTAG00000002647	*RGS10*	624	199	385	540	230	-1.234	0.186327887	1
ENSBTAG00000002648	*RPS18*	59'080	72'393	67'379	51'165	83'592	0.708	0.352227503	1
ENSBTAG00000002650	*ZNF259*	1'939	1'638	1'785	1'679	1'891	0.172	0.829000201	1
ENSBTAG00000002651	*BT.21547*	232	224	230	201	259	0.364	0.723047642	1
ENSBTAG00000002654	*DNAJC7*	2'884	1'804	2'290	2'498	2'083	-0.262	0.740458366	1
ENSBTAG00000002655	*BT.28037*	307	663	516	266	766	1.526	0.089532599	1
ENSBTAG00000002657	*NKIRAS2*	2'540	3'560	3'155	2'200	4'111	0.902	0.249912534	1
ENSBTAG00000002658	*STAM*	824	697	759	714	805	0.174	0.837689094	1
ENSBTAG00000002660	*CCDC11*	144	78	107	125	90	-0.469	0.717255343	1
ENSBTAG00000002663	*BCAS3*	429	348	387	372	402	0.113	0.904231505	1
ENSBTAG00000002664	*WNT9B*	5	8	7	4	9	1.093	0.872919157	1
ENSBTAG00000002668	*ZNF711*	2'027	765	1'319	1'755	883	-0.991	0.224248238	1
ENSBTAG00000002669	*BT.74440*	271	134	195	235	155	-0.601	0.575117873	1
ENSBTAG00000002670	*C28H10ORF10*	513	295	392	444	341	-0.383	0.679098912	1
ENSBTAG00000002673	*FIGNL2*	125	73	96	108	84	-0.361	0.791396711	1
ENSBTAG00000002674	*GNGT1*	6	0	3	5	0		0.81115604	1
ENSBTAG00000002676	*SAE1*	6'004	7'002	6'642	5'200	8'085	0.637	0.408043705	1
ENSBTAG00000002678	*MED18*	442	545	506	383	629	0.717	0.418992022	1
ENSBTAG00000002680	*TMEM132E*	434	217	313	376	251	-0.585	0.543336226	1
ENSBTAG00000002682	*BT.43504*	1'277	1'729	1'551	1'106	1'996	0.852	0.289805525	1
ENSBTAG00000002683	*PFKP*	2'371	1'324	1'791	2'053	1'529	-0.426	0.593813848	1
ENSBTAG00000002685	*PITRM1*	3'030	2'849	2'957	2'624	3'290	0.326	0.67582498	1
ENSBTAG00000002687	*TSHZ3*	577	463	517	500	535	0.097	0.913462204	1
ENSBTAG00000002688	*ATP1B1*	1'791	1'080	1'399	1'551	1'247	-0.315	0.697293216	1
ENSBTAG00000002689	*NME7*	841	694	765	728	801	0.138	0.870844193	1
ENSBTAG00000002690	*BT.96927*	1'638	1'204	1'404	1'419	1'390	-0.029	0.97290219	1
ENSBTAG00000002691	*ELMOD1*	353	102	212	306	118	-1.376	0.193110012	1
ENSBTAG00000002693	*KANSL2*	3'906	2'190	2'956	3'383	2'529	-0.420	0.591920841	1
ENSBTAG00000002695	*MYO1H*	11	3	6	10	3	-1.459	0.83622464	1
ENSBTAG00000002697	*KCTD10*	5'487	3'582	4'444	4'752	4'136	-0.200	0.796479651	1
ENSBTAG00000002698	*UBE3B*	1'648	2'194	1'980	1'427	2'533	0.828	0.298070221	1
ENSBTAG00000002699	*KIT*	2'067	1'416	1'713	1'790	1'635	-0.131	0.871072117	1
ENSBTAG00000002701	*UGT3A1*	3	4	4	3	5	0.830	0.962490925	1
ENSBTAG00000002702	*TSPAN32*	51	22	35	44	25	-0.798	0.708195583	1
ENSBTAG00000002703	*ATP6V1A*	5'712	3'592	4'547	4'947	4'148	-0.254	0.743031609	1
ENSBTAG00000002704	*POMT2*	1'068	1'334	1'233	925	1'540	0.736	0.3663289	1
ENSBTAG00000002705	*REC8*	28	9	17	24	10	-1.222	0.70359948	1
ENSBTAG00000002706	*GSTZ1*	846	419	608	733	484	-0.599	0.49068576	1
ENSBTAG00000002707	*IPO4*	2'125	3'302	2'827	1'840	3'813	1.051	0.182651044	1
ENSBTAG00000002708	*MRPL42*	916	512	692	793	591	-0.424	0.620360366	1
ENSBTAG00000002709	*BT.49827*	4'545	4'470	4'549	3'936	5'162	0.391	0.612664179	1
ENSBTAG00000002710	*TSSK4*	30	15	22	26	17	-0.585	0.841963028	1
ENSBTAG00000002712	*MDP-1*	1'303	912	1'091	1'128	1'053	-0.100	0.904853665	1
ENSBTAG00000002714	*GNAI1*	4'464	2'723	3'505	3'866	3'144	-0.298	0.70217304	1
ENSBTAG00000002715	*BT.76123*	1'647	1'119	1'359	1'426	1'292	-0.143	0.861203709	1
ENSBTAG00000002716	*BT.91056*	1'524	1'210	1'359	1'320	1'397	0.082	0.91907992	1
ENSBTAG00000002718	*TINF2*	787	779	791	682	900	0.400	0.634344406	1
ENSBTAG00000002719	*ORC1*	192	237	220	166	274	0.719	0.48744427	1
ENSBTAG00000002720	*PRPF38A*	3'417	1'981	2'623	2'959	2'287	-0.371	0.636477923	1
ENSBTAG00000002721	*ZCCHC11*	2'816	1'254	1'943	2'439	1'448	-0.752	0.345094655	1
ENSBTAG00000002724	*HORMAD1*	9	1	4	8	1	-2.755	0.785977687	1
ENSBTAG00000002725	*RNGTT*	652	533	590	565	615	0.124	0.887178776	1
ENSBTAG00000002726	*CDC27*	3'794	2'214	2'921	3'286	2'557	-0.362	0.643950704	1
ENSBTAG00000002727	*PHACTR4*	5'163	2'659	3'771	4'471	3'070	-0.542	0.485976277	1
ENSBTAG00000002728	*ARID1B*	3'212	2'167	2'642	2'782	2'502	-0.153	0.846457627	1
ENSBTAG00000002730	*ELP3*	1'148	1'087	1'125	994	1'255	0.336	0.680673086	1
ENSBTAG00000002733	*DBX1*	0	7	4	0	8	Inf	0.646259203	1
ENSBTAG00000002734	*BT.49351*	6'107	7'862	7'184	5'289	9'078	0.779	0.311749578	1
ENSBTAG00000002735	*C25H16orf13*	1'158	961	1'056	1'003	1'110	0.146	0.859066813	1
ENSBTAG00000002736	*DNMT1*	3'042	3'099	3'106	2'634	3'578	0.442	0.57094333	1
ENSBTAG00000002737	*WFIKKN1*	192	42	107	166	48	-1.778	0.172744698	1
ENSBTAG00000002738	*S1PR2*	5'107	10'131	8'061	4'423	11'698	1.403	0.071695098	1
ENSBTAG00000002739	*PDE1C*	357	106	216	309	122	-1.337	0.203657447	1
ENSBTAG00000002742	*MSH2*	2'344	1'717	2'006	2'030	1'983	-0.034	0.967065923	1
ENSBTAG00000002743	*OSGEP*	1'993	1'115	1'507	1'726	1'287	-0.423	0.599394916	1
ENSBTAG00000002744	*MUSK*	21	37	30	18	43	1.232	0.582859983	1
ENSBTAG00000002745	*APEX1*	5'055	4'718	4'913	4'378	5'448	0.316	0.682263949	1

Ensembl gene ID	geneName	counts wildtype horn bud	counts polled horn bud	baseMean	baseMean wildtype horn bud	baseMean polled horn bud	log2FoldChange	pval	padj
ENSBTAG00000002746	*OSTF1*	3'433	1'391	2'290	2'973	1'606	-0.888	0.262472669	1
ENSBTAG00000002747	*BT.30635*	1'154	902	1'020	999	1'042	0.060	0.942797449	1
ENSBTAG00000002748	*TMEM55B*	1'670	1'320	1'485	1'446	1'524	0.076	0.924988781	1
ENSBTAG00000002749	*CRHR1*	14	5	9	12	6	-1.070	0.844263455	1
ENSBTAG00000002750	*KRIT1*	2'114	1'197	1'606	1'831	1'382	-0.406	0.613246387	1
ENSBTAG00000002751	*VSTM5*	29	21	25	25	24	-0.051	1	1
ENSBTAG00000002755	*ANKRD12*	2'648	1'635	2'091	2'293	1'888	-0.281	0.723528021	1
ENSBTAG00000002756	*protein_coding*	456	370	411	395	427	0.114	0.90279292	1
ENSBTAG00000002758	*THBD*	6'058	2'485	4'058	5'246	2'869	-0.871	0.264205852	1
ENSBTAG00000002763	*protein_coding*	3'134	6'232	4'955	2'714	7'196	1.407	0.072946636	1
ENSBTAG00000002764	*IGFLR1*	985	513	723	853	592	-0.526	0.536922497	1
ENSBTAG00000002765	*CYP24A1*	1	2	2	1	2	1.415	0.981979269	1
ENSBTAG00000002767	*GNB4*	858	762	811	743	880	0.244	0.771628687	1
ENSBTAG00000002768	*SLC35A2*	1'178	1'018	1'098	1'020	1'175	0.204	0.802868645	1
ENSBTAG00000002769	*AP1G1*	3'435	2'977	3'206	2'975	3'438	0.209	0.788471966	1
ENSBTAG00000002770	*TCN2*	6'142	4'338	5'164	5'319	5'009	-0.087	0.911406658	1
ENSBTAG00000002772	*GAL3ST3*	95	65	79	82	75	-0.132	0.936157094	1
ENSBTAG00000002773	*CD300LF*	23	18	20	20	21	0.061	1	1
ENSBTAG00000002774	*PTPN23*	4'439	5'841	5'294	3'844	6'745	0.811	0.294902169	1
ENSBTAG00000002775	*C12ORF71*	2	4	3	2	5	1.415	0.927250049	1
ENSBTAG00000002778	*SF3B2*	7'117	7'459	7'388	6'164	8'613	0.483	0.529268001	1
ENSBTAG00000002779	*GABRP*	588	397	484	509	458	-0.152	0.867587412	1
ENSBTAG00000002781	*GCNT4*	130	170	154	113	196	0.802	0.480704954	1
ENSBTAG00000002782	*ZC3H7B*	2'109	3'773	3'092	1'826	4'357	1.254	0.112330044	1
ENSBTAG00000002783	*BT.61834*	2'192	1'906	2'050	1'898	2'201	0.213	0.787063939	1
ENSBTAG00000002784	*COPS5*	2'450	1'685	2'034	2'122	1'946	-0.125	0.875637829	1
ENSBTAG00000002788	*RAB27B*	112	74	91	97	85	-0.183	0.900774247	1
ENSBTAG00000002791	*SEC24C*	5'395	6'434	6'051	4'672	7'429	0.669	0.385535666	1
ENSBTAG00000002792	*BT.103968*	576	394	477	499	455	-0.133	0.884575326	1
ENSBTAG00000002795	*NKTR*	5'540	2'077	3'598	4'798	2'398	-1.000	0.201630697	1
ENSBTAG00000002796	*KLHL3*	72	23	44	62	27	-1.231	0.508189508	1
ENSBTAG00000002798	*OBFC2B*	1'270	1'416	1'367	1'100	1'635	0.572	0.478909241	1
ENSBTAG00000002799	*SLC39A5*	107	72	88	93	83	-0.157	0.918024119	1
ENSBTAG00000002804	*BT.74208*	11'258	13'244	12'521	9'750	15'293	0.649	0.39599451	1
ENSBTAG00000002808	*PSMA3*	3'852	2'222	2'951	3'336	2'566	-0.379	0.62863754	1
ENSBTAG00000002809	*CAPN10*	448	559	517	388	645	0.734	0.406726763	1
ENSBTAG00000002810	*ZNF777*	876	1'307	1'134	759	1'509	0.992	0.227573367	1
ENSBTAG00000002813	*GAB1*	7'180	3'485	5'121	6'218	4'024	-0.628	0.417351255	1
ENSBTAG00000002816	*SARM1*	858	908	896	743	1'048	0.497	0.550644518	1
ENSBTAG00000002817	*SLC46A1*	1'226	1'231	1'242	1'062	1'421	0.421	0.604119886	1
ENSBTAG00000002820	*RBBP7*	14'034	7'871	10'621	12'154	9'089	-0.419	0.584209365	1
ENSBTAG00000002821	*CILP*	12'538	14'069	13'552	10'858	16'245	0.581	0.446792517	1
ENSBTAG00000002822	*C20ORF152*	2	6	4	2	7	2.000	0.840116992	1
ENSBTAG00000002823	*MPZL1*	13'362	6'493	9'535	11'572	7'497	-0.626	0.414826082	1
ENSBTAG00000002824	*DPEP3*	35	19	26	30	22	-0.466	0.860711567	1
ENSBTAG00000002826	*CLSPN*	455	444	453	394	513	0.380	0.673381876	1
ENSBTAG00000002827	*ACAT2*	2'339	1'972	2'151	2'026	2'277	0.169	0.830384	1
ENSBTAG00000002828	*RNF185*	2'629	3'476	3'145	2'277	4'014	0.818	0.296220289	1
ENSBTAG00000002829	*TCP1*	6'726	6'779	6'826	5'825	7'828	0.426	0.578604069	1
ENSBTAG00000002830	*U2AF2*	8'589	10'729	9'914	7'438	12'389	0.736	0.337565859	1
ENSBTAG00000002833	*FBXO22*	1'079	562	792	934	649	-0.526	0.533287228	1
ENSBTAG00000002835	*SMAD1*	4'854	3'512	4'129	4'204	4'055	-0.052	0.947605137	1
ENSBTAG00000002836	*TAF3*	469	379	422	406	438	0.108	0.907490501	1
ENSBTAG00000002837	*TOR1B*	583	600	599	505	693	0.457	0.598664117	1
ENSBTAG00000002842	*HEATR4*	57	21	37	49	24	-1.026	0.61717469	1
ENSBTAG00000002843	*MGC160092*	146	77	108	126	89	-0.508	0.694285511	1
ENSBTAG00000002844	*PPFIA3*	1'117	1'790	1'517	967	2'067	1.095	0.175990064	1
ENSBTAG00000002845	*BT.34193*	278	418	362	241	483	1.003	0.284572866	1
ENSBTAG00000002846	*BT.48370*	206	96	145	178	111	-0.687	0.555931552	1
ENSBTAG00000002847	*D2HGDH*	1'167	1'237	1'220	1'011	1'428	0.499	0.539387139	1
ENSBTAG00000002848	*C16H1orf74*	253	366	321	219	423	0.948	0.322166958	1
ENSBTAG00000002849	*IRF6*	1'977	1'771	1'879	1'712	2'045	0.256	0.746538158	1
ENSBTAG00000002850	*C17ORF66*	35	7	19	30	8	-1.907	0.525088239	1
ENSBTAG00000002851	*protein_coding*	1	0	0	1	0		1	1
ENSBTAG00000002853	*HRC*	100	239	181	87	276	1.672	0.130109752	1
ENSBTAG00000002854	*TMEM63A*	4'240	6'105	5'361	3'672	7'049	0.941	0.22516984	1
ENSBTAG00000002855	*BT.75974*	7	4	5	6	5	-0.392	0.98714753	1
ENSBTAG00000002856	*MEIG1*	4	2	3	3	2	-0.585	0.996239345	1
ENSBTAG00000002858	*ACBD7*	769	171	432	666	197	-1.754	0.058965484	1
ENSBTAG00000002859	*BT.101300*	3	3	3	3	3	0.415	1	1
ENSBTAG00000002863	*ACAA2*	2'094	1'631	1'848	1'813	1'883	0.055	0.945266703	1
ENSBTAG00000002865	*KCNIP1*	107	11	53	93	13	-2.867	0.112635176	1

Ensembl gene ID	geneName	counts wildtype horn bud	counts polled horn bud	baseMean	baseMean wildtype horn bud	baseMean polled horn bud	log2FoldChange	pval	padj
ENSBTAG00000002866	*BT.20216*	792	587	682	686	678	-0.017	0.9867229	1
ENSBTAG00000002868	*GPR6*	11	0	5	10	0		0.617662983	1
ENSBTAG00000002869	*ARL4A*	1'471	1'115	1'281	1'274	1'287	0.015	0.985349957	1
ENSBTAG00000002871	*COPS6*	4'767	4'452	4'635	4'128	5'141	0.316	0.681731798	1
ENSBTAG00000002874	*TMEM183*	2'964	2'062	2'474	2'567	2'381	-0.108	0.891249984	1
ENSBTAG00000002878	*CHRD*	865	970	935	749	1'120	0.580	0.484381039	1
ENSBTAG00000002879	*DCPS*	882	964	938	764	1'113	0.543	0.512420511	1
ENSBTAG00000002880	*SORBS1*	225	132	174	195	152	-0.354	0.750842745	1
ENSBTAG00000002881	*AP4M1*	1'971	2'397	2'237	1'707	2'768	0.697	0.377571548	1
ENSBTAG00000002882	*BT.17939*	2'322	2'804	2'624	2'011	3'238	0.687	0.381866181	1
ENSBTAG00000002883	*RPTOR*	812	1'343	1'127	703	1'551	1.141	0.166688892	1
ENSBTAG00000002885	*MSR1*	602	470	532	521	543	0.058	0.949005183	1
ENSBTAG00000002887	*BT.50938*	3	1	2	3	1	-1.170	0.985253077	1
ENSBTAG00000002888	*TMTC2*	929	573	733	805	662	-0.282	0.740902133	1
ENSBTAG00000002890	*RBM10*	8'112	10'010	9'292	7'025	11'559	0.718	0.349438698	1
ENSBTAG00000002891	*BT.40017*	5	0	2	4	0		0.853268594	1
ENSBTAG00000002894	*TNFSF4*	52	18	33	45	21	-1.115	0.608207447	1
ENSBTAG00000002895	*ATOH1*	63	36	48	55	42	-0.392	0.83416089	1
ENSBTAG00000002896	*BT.91155*	696	700	706	603	808	0.423	0.619054195	1
ENSBTAG00000002900	*processed_pseudogene*	4'077	4'922	4'607	3'531	5'683	0.687	0.375407953	1
ENSBTAG00000002902	*ANO6*	1'424	859	1'113	1'233	992	-0.314	0.702125785	1
ENSBTAG00000002904	*ZNF787*	350	292	320	303	337	0.154	0.87405619	1
ENSBTAG00000002907	*BT.74302*	1'308	948	1'114	1'133	1'095	-0.049	0.953729931	1
ENSBTAG00000002910	*ECHDC2*	1'304	860	1'061	1'129	993	-0.185	0.822700605	1
ENSBTAG00000002912	*INHBA*	1'233	619	891	1'068	715	-0.579	0.488124136	1
ENSBTAG00000002914	*GALNTL4*	765	667	716	663	770	0.217	0.798585934	1
ENSBTAG00000002915	*GPR63*	261	168	210	226	194	-0.221	0.837222419	1
ENSBTAG00000002917	*PRKAG2*	1'454	1'095	1'262	1'259	1'264	0.006	0.994597583	1
ENSBTAG00000002918	*RAD51*	925	843	887	801	973	0.281	0.735726767	1
ENSBTAG00000002920	*KAT6B*	2'563	1'953	2'237	2'220	2'255	0.023	0.976761213	1
ENSBTAG00000002921	*FAM82A2*	1'146	660	877	992	762	-0.381	0.648875467	1
ENSBTAG00000002922	*GAB2*	327	664	525	283	767	1.437	0.108207948	1
ENSBTAG00000002924	*DUPD1*	0	2	1	0	2	Inf	0.939077559	1
ENSBTAG00000002928	*AMTN*	0	14	8	0	16	Inf	0.360624746	1
ENSBTAG00000002929	*IRF4*	10	9	10	9	10	0.263	0.978917997	1
ENSBTAG00000002931	*BEST3*	7	5	6	6	6	-0.070	1	1
ENSBTAG00000002934	*BT.104598*	568	239	384	492	276	-0.834	0.369151051	1
ENSBTAG00000002935	*TMEM248*	1'881	1'571	1'722	1'629	1'814	0.155	0.845446414	1
ENSBTAG00000002936	*PRRX2*	4'030	4'313	4'235	3'490	4'980	0.513	0.507751292	1
ENSBTAG00000002937	*EMR3*	2	1	1	2	1	-0.585	1	1
ENSBTAG00000002938	*C7ORF58*	1'528	1'400	1'470	1'323	1'617	0.289	0.719116187	1
ENSBTAG00000002939	*FURIN*	2'296	4'455	3'566	1'988	5'144	1.371	0.082249819	1
ENSBTAG00000002940	*WNT16*	879	518	680	761	598	-0.348	0.685451929	1
ENSBTAG00000002941	*FES*	1'015	385	662	879	445	-0.984	0.254669525	1
ENSBTAG00000002942	*SLC2A10*	1'270	664	933	1'100	767	-0.521	0.531515717	1
ENSBTAG00000002943	*protein_coding*	25	7	15	22	8	-1.421	0.689157104	1
ENSBTAG00000002944	*ATP5G3*	6'395	5'734	6'080	5'538	6'621	0.258	0.737116341	1
ENSBTAG00000002947	*LY9*	178	91	130	154	105	-0.553	0.647749537	1
ENSBTAG00000002948	*SETD2*	4'752	4'672	4'755	4'115	5'395	0.391	0.612807099	1
ENSBTAG00000002949	*BT.62437*	1'129	1'345	1'265	978	1'553	0.668	0.411303027	1
ENSBTAG00000002950	*TMEM132D*	41	49	46	36	57	0.672	0.713693554	1
ENSBTAG00000002951	*CD244*	24	5	13	21	6	-1.848	0.630822808	1
ENSBTAG00000002952	*TBX19*	129	81	103	112	94	-0.256	0.849993475	1
ENSBTAG00000002953	*TXN*	4'181	1'838	2'872	3'621	2'122	-0.771	0.326582792	1
ENSBTAG00000002955	*ZNF487P*	54	19	34	47	22	-1.092	0.607609553	1
ENSBTAG00000002956	*ZNF674*	516	244	364	447	282	-0.665	0.477081177	1
ENSBTAG00000002959	*LRCH1*	833	631	725	721	729	0.014	0.987653881	1
ENSBTAG00000002960	*WDR82P1*	14'646	10'521	12'416	12'684	12'149	-0.062	0.935806397	1
ENSBTAG00000002962	*PPM1M*	3'126	2'813	2'978	2'707	3'248	0.263	0.735972196	1
ENSBTAG00000002964	*BT.63269*	11	62	41	10	72	2.910	0.150903648	1
ENSBTAG00000002965	*BT.42155*	7	8	8	6	9	0.608	0.932722577	1
ENSBTAG00000002966	*BT.20044*	4'358	2'528	3'347	3'774	2'919	-0.371	0.634789808	1
ENSBTAG00000002970	*INTS6*	2'921	1'189	1'951	2'530	1'373	-0.882	0.269050127	1
ENSBTAG00000002971	*CUTC*	777	486	617	673	561	-0.262	0.763569996	1
ENSBTAG00000002972	*CASC1*	159	88	120	138	102	-0.438	0.725739228	1
ENSBTAG00000002973	*BT.48969*	1'042	734	875	902	848	-0.090	0.915602501	1
ENSBTAG00000002974	*BT.25090*	1'133	954	1'041	981	1'102	0.167	0.839212284	1
ENSBTAG00000002976	*BT.78171*	82	121	105	71	140	0.976	0.447939604	1
ENSBTAG00000002977	*ECE1*	5'897	7'391	6'821	5'107	8'534	0.741	0.33638547	1
ENSBTAG00000002978	*PRKG2*	99	95	98	86	110	0.356	0.791330854	1
ENSBTAG00000002979	*PIK3R3*	245	451	366	212	521	1.295	0.168533717	1
ENSBTAG00000002980	*GPT2*	950	886	923	823	1'023	0.314	0.704793888	1

Ensembl gene ID	geneName	counts wildtype horn bud	counts polled horn bud	baseMean	baseMean wildtype horn bud	baseMean polled horn bud	log2FoldChange	pval	padj
ENSBTAG00000002981	*FAM64A*	638	580	611	553	670	0.278	0.748647167	1
ENSBTAG00000002982	*protein_coding*	289	472	398	250	545	1.123	0.224810234	1
ENSBTAG00000002983	*NT5C1A*	14	4	8	12	5	-1.392	0.803716462	1
ENSBTAG00000002985	*KCNMB1*	44	17	29	38	20	-0.957	0.684136623	1
ENSBTAG00000002988	*TMEM57*	3'084	2'263	2'642	2'671	2'613	-0.032	0.969111865	1
ENSBTAG00000002993	*SFT2D1*	3'254	1'249	2'130	2'818	1'442	-0.966	0.224630963	1
ENSBTAG00000002995	*GMPPA*	4'415	3'457	3'908	3'824	3'992	0.062	0.935733749	1
ENSBTAG00000002996	*SHROOM4*	358	369	368	310	426	0.459	0.622344857	1
ENSBTAG00000002997	*ADAMTSL1*	1'289	1'369	1'349	1'116	1'581	0.502	0.534513452	1
ENSBTAG00000002998	*MFI2*	19	16	17	16	18	0.167	0.9762745	1
ENSBTAG00000002999	*ANAPC15*	631	560	597	546	647	0.243	0.779997144	1
ENSBTAG00000003000	*LRTOMT*	4	2	3	3	2	-0.585	0.996239345	1
ENSBTAG00000003001	*LAMTOR1*	1'952	2'302	2'174	1'690	2'658	0.653	0.408915505	1
ENSBTAG00000003002	*BT.28680*	2'594	1'682	2'094	2'246	1'942	-0.210	0.791489787	1
ENSBTAG00000003012	*TRAF1*	402	258	323	348	298	-0.225	0.81650024	1
ENSBTAG00000003014	*TRPV2*	486	442	466	421	510	0.278	0.756971057	1
ENSBTAG00000003015	*SESN1*	3'671	1'713	2'579	3'179	1'978	-0.685	0.384671454	1
ENSBTAG00000003016	*BT.37382*	62	52	57	54	60	0.161	0.931216726	1
ENSBTAG00000003017	*TTC38*	981	617	781	850	712	-0.254	0.76468746	1
ENSBTAG00000003019	*CRYGN*	16	2	8	14	2	-2.585	0.65347607	1
ENSBTAG00000003020	*SRCRB4D*	8	6	7	7	7	0.000	1	1
ENSBTAG00000003021	*SP1*	2'802	2'594	2'711	2'427	2'995	0.304	0.697682931	1
ENSBTAG00000003022	*CATHL7*	0	2	1	0	2	Inf	0.939077559	1
ENSBTAG00000003025	*NME6*	562	540	555	487	624	0.357	0.683211954	1
ENSBTAG00000003027	*EMX2*	1'187	641	884	1'028	740	-0.474	0.570738342	1
ENSBTAG00000003033	*GADD45G*	335	230	278	290	266	-0.127	0.900794597	1
ENSBTAG00000003034	*GATAD2A*	1'794	2'776	2'380	1'554	3'205	1.045	0.187596842	1
ENSBTAG00000003035	*IMPACT*	2'240	1'284	1'711	1'940	1'483	-0.388	0.627736604	1
ENSBTAG00000003036	*PPFIA2*	514	141	304	445	163	-1.451	0.138190478	1
ENSBTAG00000003037	*BT.62616*	248	193	219	215	223	0.053	0.962935098	1
ENSBTAG00000003038	*BT.91437*	879	789	836	761	911	0.259	0.756973772	1
ENSBTAG00000003039	*PSMB8*	531	426	476	460	492	0.097	0.914890917	1
ENSBTAG00000003040	*RALGAPB*	4'254	2'653	3'374	3'684	3'063	-0.266	0.733123553	1
ENSBTAG00000003043	*GNG2*	1'622	530	1'008	1'405	612	-1.199	0.150427296	1
ENSBTAG00000003044	*SPIN1*	9'653	6'604	7'993	8'360	7'626	-0.133	0.863378684	1
ENSBTAG00000003047	*FAM170A*	110	42	72	95	48	-0.974	0.515378345	1
ENSBTAG00000003049	*BT.28275*	1'267	1'225	1'256	1'097	1'415	0.366	0.651419314	1
ENSBTAG00000003051	*FER*	481	529	514	417	611	0.552	0.532463988	1
ENSBTAG00000003052	*PCSK7*	1'843	2'080	1'999	1'596	2'402	0.590	0.457092452	1
ENSBTAG00000003054	*INSRR*	81	68	74	70	79	0.163	0.919365128	1
ENSBTAG00000003058	*ADRM1*	1'798	2'245	2'075	1'557	2'592	0.735	0.353775574	1
ENSBTAG00000003059	*C1H21ORF59*	1'881	1'128	1'466	1'629	1'303	-0.323	0.689161581	1
ENSBTAG00000003060	*BT.22873*	68	38	51	59	44	-0.424	0.813334901	1
ENSBTAG00000003061	*LAMA5*	3'777	7'740	6'104	3'271	8'937	1.450	0.063940741	1
ENSBTAG00000003062	*BT.103321*	846	594	709	733	686	-0.095	0.913223189	1
ENSBTAG00000003063	*BT.103203*	560	282	405	485	326	-0.575	0.531888	1
ENSBTAG00000003064	*BT.39483*	7'067	3'073	4'834	6'120	3'548	-0.786	0.310961817	1
ENSBTAG00000003067	*BT.56882*	5'521	4'087	4'750	4'781	4'719	-0.019	0.981509123	1
ENSBTAG00000003068	*SC4MOL*	5'209	3'219	4'114	4'511	3'717	-0.279	0.719104744	1
ENSBTAG00000003072	*ACADVL*	5'387	6'052	5'827	4'665	6'988	0.583	0.449401522	1
ENSBTAG00000003073	*NAT11*	359	417	396	311	482	0.631	0.492651723	1
ENSBTAG00000003074	*SLC16A6*	147	78	109	127	90	-0.499	0.698459767	1
ENSBTAG00000003075	*DVL2*	1'963	2'645	2'377	1'700	3'054	0.845	0.284790056	1
ENSBTAG00000003076	*NT5DC1*	209	192	201	181	222	0.293	0.783917889	1
ENSBTAG00000003077	*DPF2*	4'124	4'384	4'317	3'571	5'062	0.503	0.515608258	1
ENSBTAG00000003078	*COL10A1*	16	7	11	14	8	-0.778	0.871294281	1
ENSBTAG00000003079	*TIGD3*	48	41	44	42	47	0.188	0.929560875	1
ENSBTAG00000003081	*RWDD4A*	1'128	1'026	1'081	977	1'185	0.278	0.7341625	1
ENSBTAG00000003083	*SLC50A1*	476	387	430	412	447	0.116	0.899490048	1
ENSBTAG00000003084	*MGC166084*	632	651	650	547	752	0.458	0.594166822	1
ENSBTAG00000003086	*BT.87590*	1	2	2	1	2	1.415	0.981979269	1
ENSBTAG00000003087	*DCUN1D5*	1'372	671	981	1'188	775	-0.617	0.456699864	1
ENSBTAG00000003088	*LANCL3*	8	2	5	7	2	-1.585	0.87391615	1
ENSBTAG00000003089	*BT.38851*	470	293	373	407	338	-0.267	0.776412459	1
ENSBTAG00000003090	*LRRC9*	67	17	39	58	20	-1.564	0.433811969	1
ENSBTAG00000003092	*ANAPC5*	3'617	1'793	2'601	3'132	2'070	-0.597	0.447651284	1
ENSBTAG00000003093	*TSGA10IP*	10	0	4	9	0		0.653360153	1
ENSBTAG00000003094	*SART1*	3'037	3'523	3'349	2'630	4'068	0.629	0.419688012	1
ENSBTAG00000003097	*DENND4C*	2'508	1'432	1'913	2'172	1'654	-0.393	0.620903113	1
ENSBTAG00000003098	*MTDH*	5'730	3'766	4'655	4'962	4'349	-0.190	0.806049487	1
ENSBTAG00000003100	*SMTN*	2'645	5'046	4'059	2'291	5'827	1.347	0.086749643	1
ENSBTAG00000003101	*SALL4*	2	1	1	2	1	-0.585	1	1

Ensembl gene ID	geneName	counts wildtype horn bud	counts polled horn bud	baseMean	baseMean wildtype horn bud	baseMean polled horn bud	log2FoldChange	pval	padj
ENSBTAG00000003102	*GCC1*	253	294	279	219	339	0.632	0.519700742	1
ENSBTAG00000003103	*EIF4E1B*	1	0	0	1	0		1	1
ENSBTAG00000003108	*SRD5A2*	3	0	1	3	0		0.936647693	1
ENSBTAG00000003109	*ITM2B*	43'252	18'277	29'281	37'457	21'104	-0.828	0.279120071	1
ENSBTAG00000003110	*FAM19A4*	16	28	23	14	32	1.222	0.642324835	1
ENSBTAG00000003111	*ATM*	716	462	577	620	533	-0.217	0.805286175	1
ENSBTAG00000003112	*BT.22029*	1'516	1'033	1'253	1'313	1'193	-0.138	0.866050321	1
ENSBTAG00000003113	*RNF125*	90	43	64	78	50	-0.651	0.681795599	1
ENSBTAG00000003114	*AVL9*	262	151	201	227	174	-0.380	0.722597401	1
ENSBTAG00000003115	*KBTBD2*	3'519	2'137	2'758	3'048	2'468	-0.305	0.698026645	1
ENSBTAG00000003116	*MTMR6*	3'740	2'076	2'818	3'239	2'397	-0.434	0.579764926	1
ENSBTAG00000003120	*MGC139239*	24	1	11	21	1	-4.170	0.402732934	1
ENSBTAG00000003121	*FRMPD3*	146	16	72	126	18	-2.775	0.077049966	1
ENSBTAG00000003124	*HIATL1*	5'021	2'687	3'725	4'348	3'103	-0.487	0.531567896	1
ENSBTAG00000003126	*PEX16*	1'010	964	994	875	1'113	0.348	0.673270763	1
ENSBTAG00000003128	*BT.42861*	122	57	86	106	66	-0.683	0.625688563	1
ENSBTAG00000003130	*BT.63828*	447	893	709	387	1'031	1.413	0.101626015	1
ENSBTAG00000003131	*NDST4*	2	5	4	2	6	1.737	0.883335552	1
ENSBTAG00000003132	*CHRNB4*	27	79	57	23	91	1.964	0.238355555	1
ENSBTAG00000003135	*IQCJ*	4	0	2	3	0		0.89545886	1
ENSBTAG00000003137	*BT.24893*	136	38	81	118	44	-1.424	0.320711491	1
ENSBTAG00000003140	*CRYGS*	14	3	8	12	3	-1.807	0.758305361	1
ENSBTAG00000003143	*BT.18678*	795	521	645	688	602	-0.195	0.822863772	1
ENSBTAG00000003144	*KIAA0825*	243	172	205	210	199	-0.084	0.942367398	1
ENSBTAG00000003147	*BT.67031*	10'835	7'418	8'974	9'383	8'566	-0.132	0.864265366	1
ENSBTAG00000003148	*MCM3AP*	5'068	4'566	4'831	4'389	5'272	0.265	0.731347606	1
ENSBTAG00000003151	*DNAJB11*	1'715	1'517	1'618	1'485	1'752	0.238	0.765690225	1
ENSBTAG00000003155	*IFI27L2*	594	318	441	514	367	-0.486	0.592033449	1
ENSBTAG00000003160	*BT.91105*	1'496	1'447	1'483	1'296	1'671	0.367	0.647608941	1
ENSBTAG00000003161	*YBEY*	417	433	431	361	500	0.469	0.605036079	1
ENSBTAG00000003162	*CRYZ*	1'416	1'479	1'467	1'226	1'708	0.478	0.552273661	1
ENSBTAG00000003163	*C20ORF151*	205	373	304	178	431	1.279	0.188704251	1
ENSBTAG00000003164	*B3GALT1*	30	11	19	26	13	-1.032	0.731379921	1
ENSBTAG00000003165	*ADAMTS9*	1'422	770	1'060	1'231	889	-0.470	0.56823608	1
ENSBTAG00000003166	*AXL*	5'671	3'502	4'477	4'911	4'044	-0.280	0.717627822	1
ENSBTAG00000003168	*HNRNPUL1*	15'265	12'845	14'026	13'220	14'832	0.166	0.827039475	1
ENSBTAG00000003169	*FBXO24*	73	21	44	63	24	-1.382	0.461629122	1
ENSBTAG00000003171	*SHANK2*	206	35	109	178	40	-2.142	0.10228196	1
ENSBTAG00000003172	*MEIS2*	2'323	1'011	1'590	2'012	1'167	-0.785	0.329058308	1
ENSBTAG00000003174	*NDUFAF4*	352	297	324	305	343	0.170	0.86019102	1
ENSBTAG00000003176	*JAM3*	3'401	2'289	2'794	2'945	2'643	-0.156	0.842690022	1
ENSBTAG00000003177	*SLC25A33*	330	159	235	286	184	-0.638	0.532768022	1
ENSBTAG00000003178	*ATE1*	722	927	848	625	1'070	0.776	0.3550408	1
ENSBTAG00000003180	*BT.58035*	265	482	393	229	557	1.278	0.169169154	1
ENSBTAG00000003183	*NTN4*	814	449	612	705	518	-0.443	0.609712904	1
ENSBTAG00000003184	*GATA1*	10	4	7	9	5	-0.907	0.905171387	1
ENSBTAG00000003185	*BT.36811*	3'107	1'180	2'027	2'691	1'363	-0.982	0.218416264	1
ENSBTAG00000003186	*FAM105B*	668	525	592	579	606	0.068	0.939267148	1
ENSBTAG00000003189	*CCDC6*	642	496	564	556	573	0.043	0.962373294	1
ENSBTAG00000003191	*FSCN1*	10'012	18'464	14'996	8'671	21'320	1.298	0.09323104	1
ENSBTAG00000003192	*TBC1D16*	800	1'852	1'416	693	2'139	1.626	0.048134491	1
ENSBTAG00000003193	*RPUSD2*	281	308	300	243	356	0.547	0.57151372	1
ENSBTAG00000003196	*PAPSS2*	656	166	380	568	192	-1.567	0.096453324	1
ENSBTAG00000003197	*processed_pseudogene*	3'602	4'518	4'168	3'119	5'217	0.742	0.339609706	1
ENSBTAG00000003199	*SLC35C1*	235	216	226	204	249	0.293	0.776866892	1
ENSBTAG00000003200	*BT.44027*	630	660	654	546	762	0.482	0.574406696	1
ENSBTAG00000003201	*FNTA*	4'060	2'755	3'349	3'516	3'181	-0.144	0.853727891	1
ENSBTAG00000003202	*OPRD1*	2	0	1	2	0		0.974934741	1
ENSBTAG00000003205	*RPL35*	10'954	8'452	9'623	9'486	9'760	0.041	0.956754305	1
ENSBTAG00000003207	*SP5*	108	157	137	94	181	0.955	0.418027237	1
ENSBTAG00000003208	*RAB33B*	2'198	1'268	1'684	1'904	1'464	-0.379	0.636218307	1
ENSBTAG00000003209	*BLCAP*	2'823	2'373	2'592	2'445	2'740	0.165	0.833450573	1
ENSBTAG00000003212	*NNAT*	3'006	1'330	2'070	2'603	1'536	-0.761	0.337935957	1
ENSBTAG00000003215	*BT.69259*	2'769	1'741	2'204	2'398	2'010	-0.254	0.747957955	1
ENSBTAG00000003217	*CADM3*	771	207	453	668	239	-1.482	0.10537233	1
ENSBTAG00000003218	*RAB28*	1'965	1'416	1'668	1'702	1'635	-0.058	0.943862625	1
ENSBTAG00000003219	*FBXL7*	497	427	462	430	493	0.196	0.828106485	1
ENSBTAG00000003220	*BT.27077*	61	8	31	53	9	-2.516	0.276473094	1
ENSBTAG00000003221	*ATF7IP*	1'049	567	782	908	655	-0.473	0.576199576	1
ENSBTAG00000003222	*ASNS*	2'046	3'799	3'079	1'772	4'387	1.308	0.098219462	1
ENSBTAG00000003225	*IMP4*	1'354	1'367	1'376	1'173	1'578	0.429	0.595007195	1
ENSBTAG00000003228	*RPL3*	73'738	65'068	69'497	63'859	75'134	0.235	0.756563265	1

Ensembl gene ID	geneName	counts wildtype horn bud	counts polled horn bud	baseMean	baseMean wildtype horn bud	baseMean polled horn bud	log2FoldChange	pval	padj
ENSBTAG00000003229	*RPL23*	96'461	72'739	83'765	83'538	83'992	0.008	0.991109983	1
ENSBTAG00000003231	*LIM2*	5	2	3	4	2	-0.907	0.965043111	1
ENSBTAG00000003232	*RAB7L1*	140	60	95	121	69	-0.807	0.547318234	1
ENSBTAG00000003235	*DHDDS*	527	599	574	456	692	0.600	0.491715172	1
ENSBTAG00000003237	*IQSEC1*	1'471	2'280	1'953	1'274	2'633	1.047	0.189972848	1
ENSBTAG00000003238	*MEOX2*	2'947	1'425	2'099	2'552	1'645	-0.633	0.42462264	1
ENSBTAG00000003239	*UTP20*	1'620	936	1'242	1'403	1'081	-0.376	0.64408377	1
ENSBTAG00000003240	*C9ORF152*	29	20	24	25	23	-0.121	0.981341314	1
ENSBTAG00000003242	*ACAD9*	1'962	2'237	2'141	1'699	2'583	0.604	0.444746859	1
ENSBTAG00000003244	*protein_coding*	91	93	93	79	107	0.446	0.742531122	1
ENSBTAG00000003245	*BCAR3*	900	768	833	779	887	0.186	0.824334992	1
ENSBTAG00000003249	*protein_coding*	1	1	1	1	1	0.415	1	1
ENSBTAG00000003253	*NPPC*	85	12	44	74	14	-2.409	0.212307122	1
ENSBTAG00000003255	*CD4*	22	18	20	19	21	0.126	0.983779847	1
ENSBTAG00000003256	*TOMM34*	870	774	824	753	894	0.246	0.76902029	1
ENSBTAG00000003257	*STK4*	226	382	318	196	441	1.172	0.223112333	1
ENSBTAG00000003258	*pseudogene*	1	0	0	1	0		1	1
ENSBTAG00000003259	*TCERG1*	4'946	2'453	3'558	4'283	2'832	-0.597	0.444120275	1
ENSBTAG00000003260	*GPR151*	26	13	19	23	15	-0.585	0.857318411	1
ENSBTAG00000003261	*BT.44020*	1'408	623	969	1'219	719	-0.761	0.359340507	1
ENSBTAG00000003263	*TBCA*	2'832	2'096	2'436	2'453	2'420	-0.019	0.981836698	1
ENSBTAG00000003264	*BT.44371*	2'166	2'315	2'274	1'876	2'673	0.511	0.51672937	1
ENSBTAG00000003265	*ADD3*	6'720	4'362	5'428	5'820	5'037	-0.208	0.787499692	1
ENSBTAG00000003267	*ZNF132*	482	352	412	417	406	-0.038	0.970128584	1
ENSBTAG00000003268	*PELO*	1'052	740	883	911	854	-0.093	0.913580226	1
ENSBTAG00000003272	*protein_coding*	12	12	12	10	14	0.415	0.934466298	1
ENSBTAG00000003275	*ANK1*	84	237	173	73	274	1.911	0.089617027	1
ENSBTAG00000003276	*BT.22013*	904	419	633	783	484	-0.694	0.422097178	1
ENSBTAG00000003279	*NDUFA10*	1'812	2'126	2'012	1'569	2'455	0.646	0.415636041	1
ENSBTAG00000003280	*MGC137055*	21	10	15	18	12	-0.655	0.864027356	1
ENSBTAG00000003282	*SLCO1A2*	13	4	8	11	5	-1.285	0.828302954	1
ENSBTAG00000003288	*CCZ1*	2'162	1'494	1'799	1'872	1'725	-0.118	0.883182371	1
ENSBTAG00000003289	*BT.88304*	2	0	1	2	0		0.974934741	1
ENSBTAG00000003290	*SLC9A7*	65	42	52	56	48	-0.215	0.910857317	1
ENSBTAG00000003291	*RIMKLB*	1'410	890	1'124	1'221	1'028	-0.249	0.762152515	1
ENSBTAG00000003294	*MRPL43*	468	502	492	405	580	0.516	0.561787969	1
ENSBTAG00000003295	*BT.18123*	3'392	1'723	2'464	2'938	1'990	-0.562	0.475574713	1
ENSBTAG00000003296	*C26H10ORF2*	1'108	894	996	960	1'032	0.105	0.898673293	1
ENSBTAG00000003298	*LZTS2*	2'651	3'317	3'063	2'296	3'830	0.738	0.345490933	1
ENSBTAG00000003299	*LPAR4*	152	32	84	132	37	-1.833	0.198637922	1
ENSBTAG00000003300	*MFGE8*	6'296	7'962	7'323	5'452	9'194	0.754	0.327698012	1
ENSBTAG00000003303	*ICT1*	1'055	1'018	1'045	914	1'175	0.364	0.658154741	1
ENSBTAG00000003304	*HOXC4*	191	270	239	165	312	0.914	0.368697243	1
ENSBTAG00000003305	*NCF1*	142	44	87	123	51	-1.275	0.359463353	1
ENSBTAG00000003307	*IMMT*	3'582	2'138	2'785	3'102	2'469	-0.329	0.674527439	1
ENSBTAG00000003308	*GRID2IP*	10	1	5	9	1	-2.907	0.754028015	1
ENSBTAG00000003312	*CHST15*	847	518	666	734	598	-0.294	0.732711637	1
ENSBTAG00000003313	*BT.49346*	1	0	0	1	0		1	1
ENSBTAG00000003314	*SKA3*	644	456	542	558	527	-0.083	0.927488511	1
ENSBTAG00000003315	*MRP63*	715	458	574	619	529	-0.228	0.796032084	1
ENSBTAG00000003316	*SYAP1*	2'266	1'486	1'839	1'962	1'716	-0.194	0.808545058	1
ENSBTAG00000003319	*FLRT3*	5'289	7'373	6'547	4'580	8'514	0.894	0.247243736	1
ENSBTAG00000003320	*BT.63533*	1	0	0	1	0		1	1
ENSBTAG00000003321	*CXHXORF41*	31	13	21	27	15	-0.839	0.771927663	1
ENSBTAG00000003322	*TTLL7*	99	22	56	86	25	-1.755	0.299304267	1
ENSBTAG00000003323	*NOL6*	1'040	1'867	1'528	901	2'156	1.259	0.120935174	1
ENSBTAG00000003325	*SNRK*	862	632	738	747	730	-0.033	0.971677033	1
ENSBTAG00000003326	*NAIP*	80	31	53	69	36	-0.953	0.579620097	1
ENSBTAG00000003327	*MGC157327*	41	19	29	36	22	-0.695	0.772323841	1
ENSBTAG00000003328	*BT.25402*	243	187	213	210	216	0.037	0.975826008	1
ENSBTAG00000003329	*FST*	1'277	1'119	1'199	1'106	1'292	0.224	0.782598356	1
ENSBTAG00000003330	*ATL3*	1'095	1'064	1'088	948	1'229	0.374	0.648304803	1
ENSBTAG00000003332	*BT.83862*	227	155	188	197	179	-0.135	0.905304761	1
ENSBTAG00000003334	*ADAP2*	144	40	85	125	46	-1.433	0.30755305	1
ENSBTAG00000003336	*TPD52L1*	197	211	207	171	244	0.514	0.624902503	1
ENSBTAG00000003338	*LRRCC1*	5'097	1'369	2'997	4'414	1'581	-1.481	0.062416905	1
ENSBTAG00000003339	*ZBTB10*	670	430	538	580	497	-0.225	0.800201201	1
ENSBTAG00000003340	*HDDC2*	1'005	653	812	870	754	-0.207	0.806895637	1
ENSBTAG00000003341	*DOK2*	162	142	152	140	164	0.225	0.847278492	1
ENSBTAG00000003342	*AMD1*	1'937	1'398	1'646	1'677	1'614	-0.055	0.946157922	1
ENSBTAG00000003343	*BT.78062*	24	20	22	21	23	0.152	0.972384506	1
ENSBTAG00000003345	*FAT4*	5'263	4'381	4'808	4'558	5'059	0.150	0.845019293	1

Ensembl gene ID	geneName	counts wildtype horn bud	counts polled horn bud	baseMean	baseMean wildtype horn bud	baseMean polled horn bud	log2FoldChange	pval	padj
ENSBTAG00000003349	*BT.38595*	535	315	414	463	364	-0.349	0.704035582	1
ENSBTAG00000003352	*HLA-DQB2*	81	7	39	70	8	-3.117	0.139481627	1
ENSBTAG00000003353	*SLC6A2*	359	718	570	311	829	1.415	0.109779887	1
ENSBTAG00000003354	*SMCHD1*	2'747	1'832	2'247	2'379	2'115	-0.169	0.830842918	1
ENSBTAG00000003358	*BT.61911*	3'591	1'857	2'627	3'110	2'144	-0.536	0.495066528	1
ENSBTAG00000003359	*ELOVL5*	3'102	1'942	2'464	2'686	2'242	-0.261	0.740943931	1
ENSBTAG00000003361	*METTL15*	295	272	285	255	314	0.298	0.761538233	1
ENSBTAG00000003362	*HSP90B1*	35'807	30'540	33'137	31'010	35'265	0.185	0.806464671	1
ENSBTAG00000003365	*SMG8*	619	465	537	536	537	0.002	0.999669637	1
ENSBTAG00000003366	*DDX58*	425	253	330	368	292	-0.333	0.72775046	1
ENSBTAG00000003367	*protein_coding*	15	4	9	13	5	-1.492	0.779648463	1
ENSBTAG00000003369	*SYT12*	134	103	117	116	119	0.035	0.984141038	1
ENSBTAG00000003370	*BT.87812*	22	6	13	19	7	-1.459	0.70932461	1
ENSBTAG00000003371	*JAMP*	3'255	1'560	2'310	2'819	1'801	-0.646	0.413627174	1
ENSBTAG00000003372	*SLC35B4*	55	65	61	48	75	0.656	0.681878621	1
ENSBTAG00000003375	*TC2N*	252	168	206	218	194	-0.170	0.876279801	1
ENSBTAG00000003376	*ASB1*	98	312	223	85	360	2.086	0.049823396	1
ENSBTAG00000003378	*ACAP2*	517	322	410	448	372	-0.268	0.771646864	1
ENSBTAG00000003381	*PAPD7*	3'067	2'549	2'800	2'656	2'943	0.148	0.849414133	1
ENSBTAG00000003382	*RIMS4*	15	4	9	13	5	-1.492	0.779648463	1
ENSBTAG00000003383	*protein_coding*	412	920	710	357	1'062	1.574	0.069422182	1
ENSBTAG00000003384	*KIAA1009*	1'234	520	835	1'069	600	-0.832	0.323195722	1
ENSBTAG00000003386	*SUPT4H1*	3'022	3'085	3'090	2'617	3'562	0.445	0.568414698	1
ENSBTAG00000003387	*BT.37390*	12	9	10	10	10	0.000	1	1
ENSBTAG00000003388	*BT.20421*	1'163	1'138	1'161	1'007	1'314	0.384	0.637947423	1
ENSBTAG00000003389	*protein_coding*	1	0	0	1	0		1	1
ENSBTAG00000003390	*SF3A1*	6'440	9'636	8'352	5'577	11'127	0.996	0.196842443	1
ENSBTAG00000003393	*CCDC157*	71	89	82	61	103	0.741	0.599819476	1
ENSBTAG00000003394	*RNF215*	1'338	1'297	1'328	1'159	1'498	0.370	0.646943336	1
ENSBTAG00000003395	*ZRANB1*	2'433	1'356	1'836	2'107	1'566	-0.428	0.590965637	1
ENSBTAG00000003396	*BT.59779*	625	640	640	541	739	0.449	0.601644935	1
ENSBTAG00000003397	*CTBP2*	4'920	3'642	4'233	4'261	4'205	-0.019	0.981550014	1
ENSBTAG00000003398	*KCNG1*	75	20	44	65	23	-1.492	0.426126997	1
ENSBTAG00000003399	*BT.103096*	9'702	6'473	7'938	8'402	7'474	-0.169	0.826395118	1
ENSBTAG00000003401	*BT.100842*	3'735	3'056	3'382	3'235	3'529	0.126	0.871368578	1
ENSBTAG00000003403	*PADI2*	126	58	88	109	67	-0.704	0.610997678	1
ENSBTAG00000003404	*PFN4*	0	13	8	0	15	Inf	0.392334779	1
ENSBTAG00000003405	*TP53I3*	301	156	220	261	180	-0.533	0.608206048	1
ENSBTAG00000003407	*SF3B14*	3'566	1'948	2'669	3'088	2'249	-0.457	0.560475977	1
ENSBTAG00000003408	*IGKC*	35	8	20	30	9	-1.714	0.559280423	1
ENSBTAG00000003409	*FKBP1B*	163	53	101	141	61	-1.206	0.35898913	1
ENSBTAG00000003410	*EFTUD1*	1'317	1'321	1'333	1'141	1'525	0.419	0.603780667	1
ENSBTAG00000003414	*FAM154B*	92	42	64	80	48	-0.716	0.650199998	1
ENSBTAG00000003415	*ESD*	4'608	3'389	3'952	3'991	3'913	-0.028	0.971965867	1
ENSBTAG00000003417	*PHKG2*	873	1'055	987	756	1'218	0.688	0.405373793	1
ENSBTAG00000003418	*MSN*	13'154	10'526	11'773	11'392	12'154	0.093	0.90193743	1
ENSBTAG00000003419	*EAPP*	825	517	656	714	597	-0.259	0.764324321	1
ENSBTAG00000003421	*RNF40*	1'966	3'487	2'865	1'703	4'026	1.242	0.116626754	1
ENSBTAG00000003422	*SNX5*	3'744	3'173	3'453	3'242	3'664	0.176	0.820185016	1
ENSBTAG00000003423	*DDX24*	4'375	4'057	4'237	3'789	4'685	0.306	0.691996094	1
ENSBTAG00000003424	*BET1*	2'618	1'485	1'991	2'267	1'715	-0.403	0.611869827	1
ENSBTAG00000003425	*BT.23197*	757	708	737	656	818	0.318	0.707152239	1
ENSBTAG00000003432	*BT.62776*	146	145	147	126	167	0.405	0.727234223	1
ENSBTAG00000003434	*CERS4*	722	723	730	625	835	0.417	0.62301562	1
ENSBTAG00000003435	*FAM189A1*	24	16	20	21	18	-0.170	0.97319525	1
ENSBTAG00000003436	*RHBDF2*	381	441	420	330	509	0.626	0.49217171	1
ENSBTAG00000003438	*ZNF297B*	435	328	378	377	379	0.008	0.995961045	1
ENSBTAG00000003439	*AKAP10*	1'727	1'226	1'456	1'496	1'416	-0.079	0.92297717	1
ENSBTAG00000003440	*BT.76681*	670	447	548	580	516	-0.169	0.849607953	1
ENSBTAG00000003443	*USP46*	2'719	1'992	2'327	2'355	2'300	-0.034	0.966997406	1
ENSBTAG00000003444	*SYDE2*	243	226	236	210	261	0.310	0.761853774	1
ENSBTAG00000003446	*EPHB4*	2'773	5'484	4'367	2'401	6'332	1.399	0.075198948	1
ENSBTAG00000003447	*ZNF551*	827	386	581	716	446	-0.684	0.433559791	1
ENSBTAG00000003449	*BT.106165*	292	275	285	253	318	0.329	0.737552436	1
ENSBTAG00000003450	*BT.61946*	1'307	1'036	1'164	1'132	1'196	0.080	0.922339156	1
ENSBTAG00000003454	*RGS20*	88	10	44	76	12	-2.722	0.163766618	1
ENSBTAG00000003455	*ANKRD6*	908	664	777	786	767	-0.036	0.96788153	1
ENSBTAG00000003456	*ZNF606*	2'049	1'043	1'489	1'774	1'204	-0.559	0.487714003	1
ENSBTAG00000003457	*ATF5*	418	821	655	362	948	1.389	0.110464366	1
ENSBTAG00000003458	*CDCA7*	2'150	1'808	1'975	1'862	2'088	0.165	0.834664777	1
ENSBTAG00000003460	*TCEA1*	1'972	1'267	1'585	1'708	1'463	-0.223	0.781485887	1
ENSBTAG00000003462	*ZNF135*	645	522	581	559	603	0.110	0.900680675	1

Ensembl gene ID	geneName	counts wildtype horn bud	counts polled horn bud	baseMean	baseMean wildtype horn bud	baseMean polled horn bud	log2FoldChange	pval	padj
ENSBTAG00000003465	*ZNF329*	634	642	645	549	741	0.433	0.614432868	1
ENSBTAG00000003467	*SLC5A11*	4	11	8	3	13	1.874	0.733957485	1
ENSBTAG00000003469	*CHURC1*	3'340	1'888	2'536	2'893	2'180	-0.408	0.604117372	1
ENSBTAG00000003472	*ABCA12*	380	105	225	329	121	-1.441	0.167403959	1
ENSBTAG00000003474	*RAB15*	766	1'188	1'018	663	1'372	1.048	0.206470639	1
ENSBTAG00000003476	*FEM1A*	2'647	4'564	3'781	2'292	5'270	1.201	0.126043808	1
ENSBTAG00000003479	*BT.48763*	15	4	9	13	5	-1.492	0.779648463	1
ENSBTAG00000003481	*OTUD6B*	426	495	470	369	572	0.632	0.480861815	1
ENSBTAG00000003483	*NUDT18*	296	326	316	256	376	0.554	0.562483127	1
ENSBTAG00000003484	*C3H1ORF123*	3'578	2'783	3'156	3'099	3'214	0.053	0.945983421	1
ENSBTAG00000003489	*IPCEF1*	54	30	41	47	35	-0.433	0.831567814	1
ENSBTAG00000003491	*SLC25A23*	617	388	491	534	448	-0.254	0.7772162	1
ENSBTAG00000003492	*protein_coding*	1	0	0	1	0		1	1
ENSBTAG00000003494	*C20ORF194*	823	667	741	713	770	0.112	0.895644492	1
ENSBTAG00000003495	*JHDM1D*	148	70	105	128	81	-0.665	0.608802306	1
ENSBTAG00000003496	*AIG1*	968	714	831	838	824	-0.024	0.979431351	1
ENSBTAG00000003497	*pseudogene*	1	1	1	1	1	0.415	1	1
ENSBTAG00000003498	*BT.80964*	1'553	2'964	2'384	1'345	3'423	1.348	0.091296746	1
ENSBTAG00000003499	*ENPP4*	515	226	353	446	261	-0.773	0.411415477	1
ENSBTAG00000003500	*WDR68*	774	1'121	982	670	1'294	0.949	0.252956611	1
ENSBTAG00000003501	*SACM1L*	4'246	2'644	3'365	3'677	3'053	-0.268	0.731027062	1
ENSBTAG00000003502	*BT.61573*	199	46	113	172	53	-1.698	0.184468138	1
ENSBTAG00000003503	*C18H16orf87*	758	551	646	656	636	-0.045	0.960802289	1
ENSBTAG00000003504	*GSS*	715	870	812	619	1'005	0.698	0.406571828	1
ENSBTAG00000003505	*DCN*	294'359	153'510	216'090	254'922	177'258	-0.524	0.490540064	1
ENSBTAG00000003506	*STEAP2*	375	129	237	325	149	-1.124	0.27253346	1
ENSBTAG00000003508	*C7ORF63*	414	75	223	359	87	-2.050	0.054315107	1
ENSBTAG00000003509	*BT.22283*	202	170	186	175	196	0.166	0.881071918	1
ENSBTAG00000003510	*MMRN2*	323	244	281	280	282	0.010	0.995034255	1
ENSBTAG00000003511	*SLAIN1*	820	656	734	710	757	0.093	0.913337221	1
ENSBTAG00000003512	*MYH7B*	220	286	260	191	330	0.794	0.425899603	1
ENSBTAG00000003515	*SNCG*	47	17	30	41	20	-1.052	0.645458921	1
ENSBTAG00000003516	*TM2D2*	1'290	973	1'120	1'117	1'124	0.008	0.992586047	1
ENSBTAG00000003519	*NOL3*	692	384	521	599	443	-0.435	0.623870954	1
ENSBTAG00000003521	*KIAA0895L*	138	112	124	120	129	0.114	0.930987772	1
ENSBTAG00000003525	*STXBP1*	3'519	2'127	2'752	3'048	2'456	-0.311	0.691662531	1
ENSBTAG00000003526	*C14ORF49*	356	374	370	308	432	0.486	0.601235671	1
ENSBTAG00000003527	*CCDC62*	370	147	245	320	170	-0.917	0.366080138	1
ENSBTAG00000003528	*protein_coding*	1	0	0	1	0		1	1
ENSBTAG00000003529	*protein_coding*	53	18	33	46	21	-1.143	0.596664274	1
ENSBTAG00000003530	*BT.35580*	10'714	10'231	10'546	9'279	11'814	0.348	0.648051406	1
ENSBTAG00000003531	*ABCA13*	10	1	5	9	1	-2.907	0.754028015	1
ENSBTAG00000003532	*BT.44985*	1'134	553	810	982	639	-0.621	0.461141441	1
ENSBTAG00000003535	*DPM3*	814	611	705	705	706	0.001	1	1
ENSBTAG00000003536	*Mrz 06*	2'118	1'328	1'684	1'834	1'533	-0.258	0.747125994	1
ENSBTAG00000003539	*BT.22715*	10	3	6	9	3	-1.322	0.863266279	1
ENSBTAG00000003541	*ZNF614*	356	231	288	308	267	-0.209	0.833669694	1
ENSBTAG00000003543	*KEAP1*	2'037	2'697	2'439	1'764	3'114	0.820	0.298829022	1
ENSBTAG00000003544	*S1PR5*	291	176	228	252	203	-0.310	0.765490684	1
ENSBTAG00000003545	*TAF1D*	3'547	1'441	2'368	3'072	1'664	-0.884	0.263921172	1
ENSBTAG00000003546	*BT.102328*	679	453	556	588	523	-0.169	0.849316382	1
ENSBTAG00000003547	*BT.78724*	1'081	549	785	936	634	-0.562	0.505680579	1
ENSBTAG00000003548	*GSTP1*	5'844	7'660	6'953	5'061	8'845	0.805	0.296298853	1
ENSBTAG00000003550	*C29H11orf54*	878	670	767	760	774	0.025	0.977407351	1
ENSBTAG00000003552	*MED17*	1'899	1'397	1'629	1'645	1'613	-0.028	0.97372776	1
ENSBTAG00000003553	*ZFP36L2*	3'104	2'050	2'528	2'688	2'367	-0.183	0.816121691	1
ENSBTAG00000003555	*THADA*	1'077	864	965	933	998	0.097	0.906938988	1
ENSBTAG00000003556	*BT.21805*	1'245	1'079	1'162	1'078	1'246	0.209	0.798113085	1
ENSBTAG00000003557	*BT.89116*	3'664	2'172	2'841	3'173	2'508	-0.339	0.665132867	1
ENSBTAG00000003559	*PPP1R13B*	656	766	726	568	885	0.639	0.452313646	1
ENSBTAG00000003561	*BT.40236*	157	329	258	136	380	1.482	0.142417102	1
ENSBTAG00000003564	*HPX*	3	0	1	3	0		0.936647693	1
ENSBTAG00000003565	*TRIM3*	1'639	1'713	1'699	1'419	1'978	0.479	0.548457751	1
ENSBTAG00000003566	*ARFIP2*	2'962	3'221	3'142	2'565	3'719	0.536	0.4920929	1
ENSBTAG00000003567	*FXC1*	200	125	159	173	144	-0.263	0.820263372	1
ENSBTAG00000003568	*CLDN10*	167	169	170	145	195	0.432	0.69725861	1
ENSBTAG00000003569	*DZIP1*	5'445	3'089	4'141	4'716	3'567	-0.403	0.603901259	1
ENSBTAG00000003570	*DDX19A*	2'426	1'980	2'194	2'101	2'286	0.122	0.876891677	1
ENSBTAG00000003572	*F11*	1	0	0	1	0		1	1
ENSBTAG00000003574	*KIAA1644*	37	79	62	32	91	1.509	0.344211596	1
ENSBTAG00000003575	*ZKSCAN4*	168	108	135	145	125	-0.222	0.856739138	1
ENSBTAG00000003576	*POLL*	632	870	776	547	1'005	0.876	0.300782275	1

Ensembl gene ID	geneName	counts wildtype horn bud	counts polled horn bud	baseMean	baseMean wildtype horn bud	baseMean polled horn bud	log2FoldChange	pval	padj
ENSBTAG00000003577	*BT.22451*	3'910	2'392	3'074	3'386	2'762	-0.294	0.707171052	1
ENSBTAG00000003578	*DPCD*	455	348	398	394	402	0.028	0.977781925	1
ENSBTAG00000003579	*FBXW4*	781	802	801	676	926	0.453	0.589714792	1
ENSBTAG00000003580	*PSD*	1'389	1'148	1'264	1'203	1'326	0.140	0.862899154	1
ENSBTAG00000003581	*SETD7*	333	543	458	288	627	1.120	0.215837281	1
ENSBTAG00000003585	*BT.87076*	7'060	5'960	6'498	6'114	6'882	0.171	0.823637795	1
ENSBTAG00000003586	*MAN1A2*	371	221	288	321	255	-0.332	0.735602025	1
ENSBTAG00000003587	*BT.3469*	730	734	740	632	848	0.423	0.617638867	1
ENSBTAG00000003588	*ZFAND1*	2'159	1'368	1'725	1'870	1'580	-0.243	0.761279773	1
ENSBTAG00000003589	*BT.49090*	3'497	1'672	2'480	3'028	1'931	-0.650	0.409985778	1
ENSBTAG00000003593	*ANKMY1*	10	6	8	9	7	-0.322	0.979718036	1
ENSBTAG00000003594	*BT.56262*	4	0	2	3	0		0.89545886	1
ENSBTAG00000003595	*FAM216B*	46	13	27	40	15	-1.408	0.558417298	1
ENSBTAG00000003598	*LEPREL2*	7'852	9'155	8'686	6'800	10'571	0.637	0.406827031	1
ENSBTAG00000003600	*MGC151839*	459	161	292	398	186	-1.096	0.263075707	1
ENSBTAG00000003601	*ZMAT1*	162	34	90	140	39	-1.837	0.187048176	1
ENSBTAG00000003602	*RBPJ*	657	1'301	1'036	569	1'502	1.401	0.093397111	1
ENSBTAG00000003604	*ADAMTSL4*	10'758	17'436	14'725	9'317	20'133	1.112	0.148938469	1
ENSBTAG00000003606	*ZNF696*	764	602	678	662	695	0.071	0.934593905	1
ENSBTAG00000003607	*FAM20C*	3'452	2'490	2'932	2'990	2'875	-0.056	0.943700341	1
ENSBTAG00000003608	*SYCP2*	51	4	24	44	5	-3.257	0.235209398	1
ENSBTAG00000003609	*CXORF26*	1'353	899	1'105	1'172	1'038	-0.175	0.832419641	1
ENSBTAG00000003610	*DENND1A*	1'124	1'762	1'504	973	2'035	1.064	0.188790801	1
ENSBTAG00000003614	*AP4B1*	615	401	498	533	463	-0.202	0.822431998	1
ENSBTAG00000003616	*DCLRE1B*	223	145	180	193	167	-0.206	0.85473245	1
ENSBTAG00000003617	*ALKBH4*	871	1'248	1'098	754	1'441	0.934	0.25671816	1
ENSBTAG00000003619	*SEC24D*	9'839	4'660	6'951	8'521	5'381	-0.663	0.389610041	1
ENSBTAG00000003622	*BT.24488*	970	493	705	840	569	-0.561	0.511137608	1
ENSBTAG00000003624	*CNTF*	7	1	4	6	1	-2.392	0.851750348	1
ENSBTAG00000003625	*BT.66099*	416	210	301	360	242	-0.571	0.555931597	1
ENSBTAG00000003626	*BT.87149*	2	7	5	2	8	2.222	0.797845137	1
ENSBTAG00000003629	*BT.28878*	285	85	172	247	98	-1.330	0.232309628	1
ENSBTAG00000003632	*CYP39A1*	1'025	380	663	888	439	-1.017	0.239200818	1
ENSBTAG00000003633	*protein_coding*	2	0	1	2	0		0.974934741	1
ENSBTAG00000003634	*CISD1*	954	560	736	826	647	-0.354	0.677978392	1
ENSBTAG00000003635	*SLC35C2*	1'267	1'314	1'307	1'097	1'517	0.468	0.563461765	1
ENSBTAG00000003636	*BT.65903*	3'899	1'649	2'640	3'377	1'904	-0.826	0.294367701	1
ENSBTAG00000003638	*BT.68008*	116	13	58	100	15	-2.743	0.111780995	1
ENSBTAG00000003639	*BT.48490*	4'445	3'762	4'097	3'849	4'344	0.174	0.821401401	1
ENSBTAG00000003640	*GJD4*	8	6	7	7	7	0.000	1	1
ENSBTAG00000003642	*PRDX1*	7'274	7'816	7'662	6'299	9'025	0.519	0.499031716	1
ENSBTAG00000003644	*HSPBP1*	700	1'055	912	606	1'218	1.007	0.228616383	1
ENSBTAG00000003649	*BT.52423*	3'775	3'292	3'535	3'269	3'801	0.218	0.779090105	1
ENSBTAG00000003650	*NR4A2*	1'074	298	637	930	344	-1.435	0.101047388	1
ENSBTAG00000003651	*OTOP1*	145	52	93	126	60	-1.064	0.43195741	1
ENSBTAG00000003652	*TMEM128*	1'673	975	1'287	1'449	1'126	-0.364	0.654353238	1
ENSBTAG00000003653	*LYAR*	910	615	749	788	710	-0.150	0.861021703	1
ENSBTAG00000003658	*BT.82217*	1'233	409	770	1'068	472	-1.177	0.167591857	1
ENSBTAG00000003661	*BT.21079*	332	100	201	288	115	-1.316	0.218263453	1
ENSBTAG00000003665	*NUP210*	478	1'187	892	414	1'371	1.727	0.042682272	1
ENSBTAG00000003666	*ZPLD1*	1	0	0	1	0		1	1
ENSBTAG00000003667	*TLN2*	1'804	4'040	3'114	1'562	4'665	1.578	0.04742374	1
ENSBTAG00000003669	*BNC2*	236	259	252	204	299	0.549	0.584205035	1
ENSBTAG00000003671	*ANKRD43*	14	3	8	12	3	-1.807	0.758305361	1
ENSBTAG00000003675	*LPHN1*	3'598	5'111	4'509	3'116	5'902	0.921	0.236209898	1
ENSBTAG00000003676	*protein_coding*	8	26	18	7	30	2.115	0.486375379	1
ENSBTAG00000003679	*PAAF1*	1'379	938	1'139	1'194	1'083	-0.141	0.86454638	1
ENSBTAG00000003682	*DNAJB13*	46	2	21	40	2	-4.109	0.194365237	1
ENSBTAG00000003687	*FOXK2*	1'137	1'298	1'242	985	1'499	0.606	0.455962428	1
ENSBTAG00000003690	*NHLRC2*	380	394	392	329	455	0.467	0.61206385	1
ENSBTAG00000003691	*NTSR2*	5	0	2	4	0		0.853268594	1
ENSBTAG00000003692	*UCP2*	757	1'082	952	656	1'249	0.930	0.263622599	1
ENSBTAG00000003693	*UNC84B*	2'885	4'894	4'075	2'498	5'651	1.177	0.132796372	1
ENSBTAG00000003695	*BT.39758*	3'045	2'384	2'695	2'637	2'753	0.062	0.936680578	1
ENSBTAG00000003696	*CCDC64*	284	200	238	246	231	-0.091	0.933613955	1
ENSBTAG00000003697	*TARDBP*	6'600	3'185	4'697	5'716	3'678	-0.636	0.4119466	1
ENSBTAG00000003699	*ZFP3*	325	321	326	281	371	0.397	0.676770125	1
ENSBTAG00000003700	*SLC5A10*	45	58	53	39	67	0.781	0.646775613	1
ENSBTAG00000003701	*BT.56209*	232	268	255	201	309	0.623	0.533320921	1
ENSBTAG00000003702	*ANGEL2*	3'608	1'849	2'630	3'125	2'135	-0.549	0.484654444	1
ENSBTAG00000003705	*FAM83G*	223	281	259	193	324	0.749	0.453054362	1
ENSBTAG00000003707	*GRAP*	245	172	205	212	199	-0.095	0.933232222	1

Ensembl gene ID	geneName	counts wildtype horn bud	counts polled horn bud	baseMean	baseMean wildtype horn bud	baseMean polled horn bud	log2FoldChange	pval	padj
ENSBTAG00000003708	*BT.69980*	6'856	8'254	7'734	5'937	9'531	0.683	0.374505126	1
ENSBTAG00000003709	*ITGB8*	114	39	72	99	45	-1.132	0.449610965	1
ENSBTAG00000003710	*XPO4*	767	672	720	664	776	0.224	0.792088862	1
ENSBTAG00000003712	*MGC140823*	2'223	2'352	2'321	1'925	2'716	0.496	0.528414609	1
ENSBTAG00000003713	*BT.74445*	588	684	650	509	790	0.633	0.461529681	1
ENSBTAG00000003715	*ADAM21*	4	6	5	3	7	1.000	0.91481797	1
ENSBTAG00000003719	*TDRD7*	659	439	539	571	507	-0.171	0.848026492	1
ENSBTAG00000003721	*CHST1*	1'373	610	947	1'189	704	-0.755	0.363963156	1
ENSBTAG00000003724	*BT.51200*	0	1	1	0	1	Inf	0.993540919	1
ENSBTAG00000003726	*MTMR9*	415	292	348	359	337	-0.092	0.925674174	1
ENSBTAG00000003727	*BT.104277*	2'038	1'145	1'544	1'765	1'322	-0.417	0.60419153	1
ENSBTAG00000003728	*NDUFS4*	2'991	1'942	2'416	2'590	2'242	-0.208	0.792203159	1
ENSBTAG00000003731	*protein_coding*	2	7	5	2	8	2.222	0.797845137	1
ENSBTAG00000003733	*TM4SF5*	133	134	135	115	155	0.426	0.720930105	1
ENSBTAG00000003737	*OSTB*	61	4	29	53	5	-3.516	0.166206557	1
ENSBTAG00000003740	*KDM6A*	3'598	1'073	2'177	3'116	1'239	-1.331	0.096589686	1
ENSBTAG00000003741	*NEURL*	151	122	136	131	141	0.107	0.93292299	1
ENSBTAG00000003743	*protein_coding*	189	194	194	164	224	0.453	0.672461254	1
ENSBTAG00000003745	*BT.64382*	3'974	3'063	3'489	3'442	3'537	0.039	0.959278728	1
ENSBTAG00000003746	*SCP2*	7'086	4'188	5'486	6'137	4'836	-0.344	0.656295136	1
ENSBTAG00000003747	*PTGR2*	1'340	813	1'050	1'160	939	-0.306	0.710900937	1
ENSBTAG00000003748	*BT.30639*	4'160	2'980	3'522	3'603	3'441	-0.066	0.933104787	1
ENSBTAG00000003749	*KCNMB4*	2'138	1'412	1'741	1'852	1'630	-0.183	0.818999026	1
ENSBTAG00000003751	*MACC1*	9	15	13	8	17	1.152	0.772410391	1
ENSBTAG00000003752	*SLC25A24*	3'073	2'078	2'530	2'661	2'399	-0.149	0.850012907	1
ENSBTAG00000003754	*STARD9*	2'595	2'707	2'687	2'247	3'126	0.476	0.54341631	1
ENSBTAG00000003757	*DNAJA2*	2'313	1'360	1'787	2'003	1'570	-0.351	0.660025004	1
ENSBTAG00000003758	*TKT*	6'449	8'559	7'734	5'585	9'883	0.823	0.285169249	1
ENSBTAG00000003759	*PCNXL3*	3'340	5'583	4'670	2'893	6'447	1.156	0.138691812	1
ENSBTAG00000003760	*pseudogene*	5	5	5	4	6	0.415	0.985023983	1
ENSBTAG00000003762	*GNB5*	1'632	1'096	1'339	1'413	1'266	-0.159	0.845031261	1
ENSBTAG00000003763	*MYO5C*	247	164	202	214	189	-0.176	0.872641299	1
ENSBTAG00000003764	*AKIRIN2*	3'206	1'356	2'171	2'776	1'566	-0.826	0.297772915	1
ENSBTAG00000003766	*CNO*	703	370	518	609	427	-0.511	0.564393908	1
ENSBTAG00000003769	*processed_pseudogene*	1'245	1'301	1'290	1'078	1'502	0.479	0.55473683	1
ENSBTAG00000003770	*PTS*	503	295	388	436	341	-0.355	0.702453251	1
ENSBTAG00000003771	*C1QL2*	17	0	7	15	0		0.437564044	1
ENSBTAG00000003773	*SLC25A13*	680	249	438	589	288	-1.034	0.256463114	1
ENSBTAG00000003774	*BT.39896*	2	0	1	2	0		0.974934741	1
ENSBTAG00000003775	*BT.19507*	20	14	17	17	16	-0.100	0.998076173	1
ENSBTAG00000003776	*C4ORF44*	155	235	203	134	271	1.015	0.337475617	1
ENSBTAG00000003777	*TIE1*	2'421	1'608	1'977	2'097	1'857	-0.175	0.825953495	1
ENSBTAG00000003779	*ZC4H2*	689	519	598	597	599	0.006	0.995772343	1
ENSBTAG00000003781	*BT.26255*	0	1	1	0	1	Inf	0.993540919	1
ENSBTAG00000003784	*DNAJC2*	1'770	954	1'317	1'533	1'102	-0.477	0.556898263	1
ENSBTAG00000003786	*BT.27844*	145	30	80	126	35	-1.858	0.201694956	1
ENSBTAG00000003788	*RCC1*	400	451	434	346	521	0.588	0.516521209	1
ENSBTAG00000003789	*KIAA1429*	8'211	6'493	7'304	7'111	7'497	0.076	0.920166804	1
ENSBTAG00000003791	*LPAR3*	358	56	187	310	65	-2.261	0.043519474	1
ENSBTAG00000003793	*NGLY1*	2'758	1'795	2'231	2'388	2'073	-0.205	0.796206644	1
ENSBTAG00000003794	*BT.72155*	903	320	576	782	370	-1.082	0.218439643	1
ENSBTAG00000003797	*SLC17A3*	3	2	2	3	2	-0.170	1	1
ENSBTAG00000003798	*BT.20898*	4'221	4'228	4'269	3'655	4'882	0.417	0.589457324	1
ENSBTAG00000003799	*TARS2*	2'535	1'944	2'220	2'195	2'245	0.032	0.967465791	1
ENSBTAG00000003800	*LRRC27*	191	180	187	165	208	0.329	0.761800296	1
ENSBTAG00000003801	*MBD1*	1'776	1'361	1'555	1'538	1'572	0.031	0.969241345	1
ENSBTAG00000003802	*BT.26301*	106	59	80	92	68	-0.430	0.768339687	1
ENSBTAG00000003804	*BT.27130*	998	607	783	864	701	-0.302	0.721151644	1
ENSBTAG00000003806	*ECM1*	1'885	747	1'248	1'632	863	-0.920	0.260091422	1
ENSBTAG00000003807	*RQCD1*	2'910	1'958	2'391	2'520	2'261	-0.157	0.843165495	1
ENSBTAG00000003808	*HDAC9*	357	201	271	309	232	-0.414	0.677422065	1
ENSBTAG00000003809	*BT.45064*	814	288	519	705	333	-1.084	0.223617056	1
ENSBTAG00000003810	*protein_coding*	42	14	26	36	16	-1.170	0.635897554	1
ENSBTAG00000003813	*BCS1L*	542	647	608	469	747	0.671	0.439166511	1
ENSBTAG00000003815	*EDEM2*	1'218	899	1'046	1'055	1'038	-0.023	0.979605	1
ENSBTAG00000003817	*BT.45858*	1'392	1'299	1'353	1'206	1'500	0.315	0.696062984	1
ENSBTAG00000003819	*STK36*	916	1'004	976	793	1'159	0.547	0.507908202	1
ENSBTAG00000003820	*GRAMD1A*	2'697	2'801	2'785	2'336	3'234	0.470	0.548350673	1
ENSBTAG00000003822	*CROCC*	3'095	2'970	3'055	2'680	3'429	0.356	0.648272026	1
ENSBTAG00000003825	*PTPN12*	2'865	1'755	2'254	2'481	2'026	-0.292	0.711864031	1
ENSBTAG00000003826	*SCN1B*	93	72	82	81	83	0.046	0.983041915	1
ENSBTAG00000003829	*HPN*	190	327	271	165	378	1.198	0.227897165	1

Ensembl gene ID	geneName	counts wildtype horn bud	counts polled horn bud	baseMean	baseMean wildtype horn bud	baseMean polled horn bud	log2FoldChange	pval	padj
ENSBTAG00000003830	*PSMB3*	3'143	2'886	3'027	2'722	3'332	0.292	0.707905826	1
ENSBTAG00000003832	*BT.22115*	10'964	11'531	11'405	9'495	13'315	0.488	0.523242437	1
ENSBTAG00000003833	*TMEM22*	139	16	69	120	18	-2.704	0.089378299	1
ENSBTAG00000003835	*BMP4*	1'940	1'680	1'810	1'680	1'940	0.207	0.793953559	1
ENSBTAG00000003836	*BT.44707*	4'049	1'905	2'853	3'507	2'200	-0.673	0.391401903	1
ENSBTAG00000003837	*BT.27743*	731	401	548	633	463	-0.451	0.608239877	1
ENSBTAG00000003840	*GUCY1B1*	4'236	1'206	2'531	3'668	1'393	-1.397	0.07977993	1
ENSBTAG00000003842	*RABGEF1*	2'923	2'332	2'612	2'531	2'693	0.089	0.909183635	1
ENSBTAG00000003843	*SMARCAL1*	3'322	2'301	2'767	2'877	2'657	-0.115	0.884437986	1
ENSBTAG00000003845	*BT.102977*	310	432	384	268	499	0.894	0.334947816	1
ENSBTAG00000003846	*RPL37A*	69'701	48'630	58'258	60'363	56'153	-0.104	0.891332297	1
ENSBTAG00000003848	*BT.70303*	628	727	692	544	839	0.626	0.463407497	1
ENSBTAG00000003849	*BT.46638*	2'908	2'068	2'453	2'518	2'388	-0.077	0.92332678	1
ENSBTAG00000003851	*BT.53231*	5'918	1'903	3'661	5'125	2'197	-1.222	0.120225301	1
ENSBTAG00000003855	*FAM120C*	361	275	315	313	318	0.022	0.984296303	1
ENSBTAG00000003856	*pseudogene*	2	7	5	2	8	2.222	0.797845137	1
ENSBTAG00000003857	*KIAA0247*	313	325	323	271	375	0.469	0.622605925	1
ENSBTAG00000003861	*CCDC75*	511	379	440	443	438	-0.016	0.989453167	1
ENSBTAG00000003863	*PCED1A*	1'773	1'321	1'530	1'535	1'525	-0.010	0.992167726	1
ENSBTAG00000003864	*SLC13A4*	210	172	190	182	199	0.127	0.909457743	1
ENSBTAG00000003865	*CASR*	1	1	1	1	1	0.415	1	1
ENSBTAG00000003866	*PPP1R8*	2'491	1'978	2'221	2'157	2'284	0.082	0.916633335	1
ENSBTAG00000003872	*BT.35446*	819	419	597	709	484	-0.552	0.52614642	1
ENSBTAG00000003876	*MCU*	309	396	362	268	457	0.773	0.408257679	1
ENSBTAG00000003877	*ZCCHC24*	672	624	651	582	721	0.308	0.719919811	1
ENSBTAG00000003878	*BT.29175*	1'175	758	946	1'018	875	-0.217	0.79449058	1
ENSBTAG00000003880	*BT.42167*	6'019	13'135	10'190	5'213	15'167	1.541	0.048243705	1
ENSBTAG00000003882	*TMEM111*	2'723	1'959	2'310	2'358	2'262	-0.060	0.940432022	1
ENSBTAG00000003884	*FAM120B*	1'152	793	957	998	916	-0.124	0.882938653	1
ENSBTAG00000003885	*BT.30603*	1'150	656	877	996	757	-0.395	0.6370386	1
ENSBTAG00000003887	*ECHDC1*	1'936	1'876	1'921	1'677	2'166	0.370	0.640979768	1
ENSBTAG00000003889	*PER1*	3'589	4'231	3'997	3'108	4'886	0.652	0.400878515	1
ENSBTAG00000003891	*VAMP2*	2'220	1'472	1'811	1'923	1'700	-0.178	0.824267292	1
ENSBTAG00000003892	*CMAHP*	78	9	39	68	10	-2.700	0.192108355	1
ENSBTAG00000003893	*RPGR*	54	54	55	47	62	0.415	0.809758888	1
ENSBTAG00000003894	*NDRG4*	2'151	854	1'424	1'863	986	-0.918	0.257643232	1
ENSBTAG00000003895	*CYBA*	1'532	1'876	1'746	1'327	2'166	0.707	0.375867845	1
ENSBTAG00000003897	*C6H4orf52*	1'552	984	1'240	1'344	1'136	-0.242	0.766579607	1
ENSBTAG00000003898	*HMGCS2*	2	0	1	2	0		0.974934741	1
ENSBTAG00000003901	*BT.22977*	189	132	158	164	152	-0.103	0.933887665	1
ENSBTAG00000003902	*ZNF512*	3'358	3'036	3'207	2'908	3'506	0.270	0.728886875	1
ENSBTAG00000003904	*GPN1*	2'512	2'053	2'273	2'175	2'371	0.124	0.874729521	1
ENSBTAG00000003906	*SUPT7L*	535	432	481	463	499	0.107	0.906374717	1
ENSBTAG00000003907	*TSPAN14*	2'665	2'753	2'743	2'308	3'179	0.462	0.555098946	1
ENSBTAG00000003908	*BT.53583*	2'007	1'395	1'674	1'738	1'611	-0.110	0.891973012	1
ENSBTAG00000003910	*SH2D4B*	13	12	13	11	14	0.300	0.958035022	1
ENSBTAG00000003912	*pseudogene*	6	4	5	5	5	-0.170	1	1
ENSBTAG00000003914	*FAM125A*	1'831	2'499	2'236	1'586	2'886	0.864	0.275585035	1
ENSBTAG00000003915	*BT.44938*	1'372	1'771	1'617	1'188	2'045	0.783	0.329046354	1
ENSBTAG00000003916	*DPH3*	996	1'092	1'062	863	1'261	0.548	0.504841452	1
ENSBTAG00000003920	*TGM1*	187	103	140	162	119	-0.445	0.706837692	1
ENSBTAG00000003921	*BT.92277*	481	111	272	417	128	-1.700	0.091362115	1
ENSBTAG00000003922	*MPHOSPH8*	1'780	1'162	1'442	1'542	1'342	-0.200	0.80469397	1
ENSBTAG00000003923	*GPBAR1*	4	1	2	3	1	-1.585	0.952247636	1
ENSBTAG00000003925	*BT.68078*	2'237	1'303	1'721	1'937	1'505	-0.365	0.648331368	1
ENSBTAG00000003927	*AAMP*	4'125	4'935	4'635	3'572	5'698	0.674	0.38443204	1
ENSBTAG00000003928	*ATG16L1*	1'517	1'260	1'384	1'314	1'455	0.147	0.855119929	1
ENSBTAG00000003929	*PHF10*	3'018	2'234	2'597	2'614	2'580	-0.019	0.981981079	1
ENSBTAG00000003934	*PSPC1*	2'702	1'527	2'052	2'340	1'763	-0.408	0.606714571	1
ENSBTAG00000003935	*RECS1*	7'057	7'391	7'323	6'112	8'534	0.482	0.530159692	1
ENSBTAG00000003936	*PNKD*	721	860	809	624	993	0.669	0.426186756	1
ENSBTAG00000003937	*RPS13*	24'368	25'745	25'416	21'103	29'728	0.494	0.515878873	1
ENSBTAG00000003941	*MGC155012*	2'691	1'457	2'006	2'330	1'682	-0.470	0.553763256	1
ENSBTAG00000003942	*PIP4K2C*	1'119	883	994	969	1'020	0.073	0.929670414	1
ENSBTAG00000003943	*TTC39B*	81	15	44	70	17	-2.018	0.289374381	1
ENSBTAG00000003944	*C2ORF62*	18	2	9	16	2	-2.755	0.605203757	1
ENSBTAG00000003946	*BT.46978*	2'083	1'077	1'524	1'804	1'244	-0.537	0.504850315	1
ENSBTAG00000003947	*SSBP4*	3'995	2'803	3'348	3'460	3'237	-0.096	0.902623458	1
ENSBTAG00000003948	*BT.23182*	5'276	4'805	5'059	4'569	5'548	0.280	0.716008596	1
ENSBTAG00000003949	*RBM39*	22'370	10'751	15'894	19'373	12'414	-0.642	0.40136424	1
ENSBTAG00000003950	*PROC*	36	8	20	31	9	-1.755	0.544756671	1
ENSBTAG00000003952	*FBXL2*	445	214	316	385	247	-0.641	0.504441943	1

Ensembl gene ID	geneName	counts wildtype horn bud	counts polled horn bud	baseMean	baseMean wildtype horn bud	baseMean polled horn bud	log2FoldChange	pval	padj
ENSBTAG00000003953	*protein_coding*	596	1'022	848	516	1'180	1.193	0.15777297	1
ENSBTAG00000003954	*BT.58583*	5'533	4'111	4'769	4'792	4'747	-0.014	0.98700411	1
ENSBTAG00000003955	*MYO7A*	97	95	97	84	110	0.385	0.7746305	1
ENSBTAG00000003956	*SGCA*	383	443	422	332	512	0.625	0.492554118	1
ENSBTAG00000003957	*PRDM7*	14	13	14	12	15	0.308	0.951846517	1
ENSBTAG00000003958	*TMEM220*	447	299	366	387	345	-0.165	0.862441697	1
ENSBTAG00000003959	*BT.102104*	2'360	3'121	2'824	2'044	3'604	0.818	0.297530053	1
ENSBTAG00000003960	*CCP110*	890	884	896	771	1'021	0.405	0.626198582	1
ENSBTAG00000003961	*SNX20*	48	29	38	42	33	-0.312	0.888396056	1
ENSBTAG00000003962	*C20ORF144*	10	12	11	9	14	0.678	0.884992558	1
ENSBTAG00000003963	*FER1L5*	161	283	233	139	327	1.229	0.231812547	1
ENSBTAG00000003965	*LIPT1*	255	240	249	221	277	0.328	0.745764592	1
ENSBTAG00000003966	*DUSP3*	2'141	1'330	1'695	1'854	1'536	-0.272	0.734338415	1
ENSBTAG00000003967	*OTUB1*	3'086	3'311	3'248	2'673	3'823	0.517	0.507434459	1
ENSBTAG00000003968	*UBP1*	3'709	3'052	3'368	3'212	3'524	0.134	0.863094531	1
ENSBTAG00000003970	*MITD1*	805	374	565	697	432	-0.691	0.430757433	1
ENSBTAG00000003971	*BT.78028*	347	685	546	301	791	1.396	0.116387974	1
ENSBTAG00000003975	*LMAN2L*	772	854	827	669	986	0.561	0.503775951	1
ENSBTAG00000003977	*SLC52A3*	21	10	15	18	12	-0.655	0.864027356	1
ENSBTAG00000003981	*RRP1*	1'131	1'312	1'247	979	1'515	0.629	0.438947873	1
ENSBTAG00000003983	*TOM1L2*	1'180	1'326	1'277	1'022	1'531	0.583	0.472178882	1
ENSBTAG00000003985	*CCDC15*	1'152	673	887	998	777	-0.360	0.666436468	1
ENSBTAG00000003986	*CXXC5*	2'218	1'329	1'728	1'921	1'535	-0.324	0.685472166	1
ENSBTAG00000003987	*protein_coding*	4	1	2	3	1	-1.585	0.952247636	1
ENSBTAG00000003990	*ITPRIP*	1'274	1'614	1'484	1'103	1'864	0.756	0.347994181	1
ENSBTAG00000003992	*BT.60930*	0	1	1	0	1	Inf	0.993540919	1
ENSBTAG00000003994	*IGFBP3*	5'073	6'445	5'918	4'393	7'442	0.760	0.324875333	1
ENSBTAG00000003997	*BT.38237*	871	706	785	754	815	0.112	0.894796045	1
ENSBTAG00000004000	*SAP30L*	566	437	497	490	505	0.042	0.964118352	1
ENSBTAG00000004004	*CSDC2*	846	805	831	733	930	0.343	0.68187257	1
ENSBTAG00000004005	*WDR7*	537	392	459	465	453	-0.039	0.968756166	1
ENSBTAG00000004007	*MCF2*	28	6	16	24	7	-1.807	0.598818952	1
ENSBTAG00000004008	*FAM199X*	53	39	45	46	45	-0.027	1	1
ENSBTAG00000004009	*PMM1*	1'255	1'305	1'297	1'087	1'507	0.471	0.560489971	1
ENSBTAG00000004010	*PAPPA*	87	152	125	75	176	1.220	0.31619132	1
ENSBTAG00000004011	*GALK2*	624	479	547	540	553	0.034	0.971084499	1
ENSBTAG00000004013	*FGF7*	2'010	1'619	1'805	1'741	1'869	0.103	0.896917734	1
ENSBTAG00000004014	*FBLN2*	11'482	10'636	11'113	9'944	12'281	0.305	0.689612541	1
ENSBTAG00000004015	*KAT6A*	1'547	1'365	1'458	1'340	1'576	0.234	0.770417283	1
ENSBTAG00000004017	*PLEKHG2*	4'845	7'188	6'248	4'196	8'300	0.984	0.203911005	1
ENSBTAG00000004018	*DGKA*	3'133	1'374	2'150	2'713	1'587	-0.774	0.329247326	1
ENSBTAG00000004019	*PMEL17*	1'347	1'375	1'377	1'167	1'588	0.445	0.581441001	1
ENSBTAG00000004021	*CDK2*	1'355	1'134	1'241	1'173	1'309	0.158	0.845588285	1
ENSBTAG00000004022	*PLAG1*	51	32	41	44	37	-0.257	0.906140921	1
ENSBTAG00000004023	*KIAA1324L*	209	29	107	181	33	-2.434	0.068251779	1
ENSBTAG00000004024	*UBE2Q2*	2'795	1'980	2'353	2'421	2'286	-0.082	0.917845543	1
ENSBTAG00000004028	*POLK*	1'219	542	841	1'056	626	-0.754	0.369566011	1
ENSBTAG00000004029	*LCOR*	17	13	15	15	15	0.028	1	1
ENSBTAG00000004034	*SESN3*	470	178	306	407	206	-0.986	0.308886846	1
ENSBTAG00000004036	*GJC1*	804	675	738	696	779	0.163	0.848209314	1
ENSBTAG00000004037	*JUN*	5'894	3'845	4'772	5'104	4'440	-0.201	0.795182121	1
ENSBTAG00000004038	*BT.93332*	1'038	256	597	899	296	-1.605	0.069900346	1
ENSBTAG00000004039	*DHX35*	663	591	628	574	682	0.249	0.772954012	1
ENSBTAG00000004041	*BT.91133*	5'351	2'141	3'553	4'634	2'472	-0.906	0.246676084	1
ENSBTAG00000004043	*protein_coding*	146	134	141	126	155	0.291	0.806248973	1
ENSBTAG00000004048	*BT.37989*	10	14	12	9	16	0.900	0.826937278	1
ENSBTAG00000004051	*SBDS*	7'928	5'640	6'689	6'866	6'513	-0.076	0.921771947	1
ENSBTAG00000004052	*EPGN*	3	2	2	3	2	-0.170	1	1
ENSBTAG00000004054	*PAPOLA*	4'921	3'969	4'422	4'262	4'583	0.105	0.891670181	1
ENSBTAG00000004059	*SOAT1*	1'852	930	1'339	1'604	1'074	-0.579	0.475364671	1
ENSBTAG00000004061	*PP2D1*	87	40	61	75	46	-0.706	0.662602178	1
ENSBTAG00000004063	*SERPINA5*	103	40	68	89	46	-0.950	0.536383382	1
ENSBTAG00000004064	*BPNT1*	2'049	1'450	1'724	1'774	1'674	-0.084	0.917599976	1
ENSBTAG00000004066	*PARP8*	572	439	501	495	507	0.033	0.971863888	1
ENSBTAG00000004072	*CAPZA2*	4'970	3'763	4'325	4'304	4'345	0.014	0.985548221	1
ENSBTAG00000004073	*BT.63562*	1'094	1'116	1'118	947	1'289	0.444	0.587358948	1
ENSBTAG00000004075	*IDI1*	1'930	1'047	1'440	1'671	1'209	-0.467	0.562579127	1
ENSBTAG00000004076	*OXER1*	8	10	9	7	12	0.737	0.893887116	1
ENSBTAG00000004077	*YWHAG*	4'270	3'430	3'829	3'698	3'961	0.099	0.898012136	1
ENSBTAG00000004078	*KCNH2*	267	112	180	231	129	-0.838	0.4433362	1
ENSBTAG00000004079	*ZFP106*	756	665	711	655	768	0.230	0.78715319	1
ENSBTAG00000004080	*TIAL1*	8'643	4'467	6'322	7'485	5'158	-0.537	0.485961298	1

Ensembl gene ID	geneName	counts wildtype horn bud	counts polled horn bud	baseMean	baseMean wildtype horn bud	baseMean polled horn bud	log2FoldChange	pval	padj
ENSBTAG00000004081	*FAT3*	366	62	194	317	72	-2.146	0.052094357	1
ENSBTAG00000004082	*pseudogene*	453	333	388	392	385	-0.029	0.97885423	1
ENSBTAG00000004085	*ASF1B*	392	612	523	339	707	1.058	0.233492967	1
ENSBTAG00000004086	*SPINT1*	603	1'118	907	522	1'291	1.306	0.12087862	1
ENSBTAG00000004088	*CRYBB2*	1	0	0	1	0		1	1
ENSBTAG00000004091	*GTF3C4*	303	469	402	262	542	1.045	0.256954909	1
ENSBTAG00000004092	*BT.19921*	272	429	365	236	495	1.072	0.252474114	1
ENSBTAG00000004093	*TUBB2B*	6'200	2'035	3'860	5'369	2'350	-1.192	0.128776729	1
ENSBTAG00000004094	*SPARCL1*	22'999	9'908	15'679	19'918	11'441	-0.800	0.296711783	1
ENSBTAG00000004095	*FAM166A*	12	5	8	10	6	-0.848	0.892845055	1
ENSBTAG00000004097	*A2ML1*	2'340	852	1'505	2'026	984	-1.043	0.197945662	1
ENSBTAG00000004098	*BT.48133*	785	467	610	680	539	-0.334	0.700877986	1
ENSBTAG00000004099	*C6ORF97*	33	6	18	29	7	-2.044	0.51823266	1
ENSBTAG00000004100	*TTI1*	1'192	1'017	1'103	1'032	1'174	0.186	0.820326564	1
ENSBTAG00000004104	*RUNX2*	1'326	325	762	1'148	375	-1.614	0.061382329	1
ENSBTAG00000004106	*BT.62775*	214	195	205	185	225	0.281	0.791518414	1
ENSBTAG00000004108	*STRA13*	551	420	481	477	485	0.023	0.981002382	1
ENSBTAG00000004109	*pseudogene*	1	1	1	1	1	0.415	1	1
ENSBTAG00000004110	*protein_coding*	35	51	45	30	59	0.958	0.603427049	1
ENSBTAG00000004111	*LRRC45*	632	635	640	547	733	0.422	0.623968604	1
ENSBTAG00000004112	*GAMT*	1'257	1'335	1'315	1'089	1'542	0.502	0.535151385	1
ENSBTAG00000004114	*BT.27525*	1'425	1'888	1'707	1'234	2'180	0.821	0.305319767	1
ENSBTAG00000004115	*MYLIP*	3'384	1'422	2'286	2'931	1'642	-0.836	0.291430599	1
ENSBTAG00000004117	*AZI2*	2'783	1'351	1'985	2'410	1'560	-0.628	0.429824915	1
ENSBTAG00000004118	*ALAS1*	1'591	1'195	1'379	1'378	1'380	0.002	0.998271609	1
ENSBTAG00000004120	*BT.51775*	1'379	869	1'099	1'194	1'003	-0.251	0.760334209	1
ENSBTAG00000004124	*CPOX*	550	405	472	476	468	-0.026	0.979812537	1
ENSBTAG00000004126	*MLF1*	854	650	745	740	751	0.021	0.98105784	1
ENSBTAG00000004129	*CCL11*	3	6	5	3	7	1.415	0.880191876	1
ENSBTAG00000004130	*C11ORF87*	62	121	97	54	140	1.380	0.300748287	1
ENSBTAG00000004131	*RAP2C*	4'282	2'122	3'079	3'708	2'450	-0.598	0.444978951	1
ENSBTAG00000004135	*LMTK2*	514	512	518	445	591	0.409	0.643127245	1
ENSBTAG00000004136	*NFE2L3*	504	357	424	436	412	-0.082	0.931051788	1
ENSBTAG00000004138	*GLTSCR1*	814	967	911	705	1'117	0.664	0.425348669	1
ENSBTAG00000004139	*BT.84482*	625	595	614	541	687	0.344	0.690796885	1
ENSBTAG00000004140	*C9ORF96*	44	50	48	38	58	0.599	0.739680939	1
ENSBTAG00000004147	*BT.20171*	3'814	3'607	3'734	3'303	4'165	0.335	0.666093117	1
ENSBTAG00000004148	*MATN4*	16	32	25	14	37	1.415	0.568981356	1
ENSBTAG00000004150	*GGFBPP5*	84	7	40	73	8	-3.170	0.127764016	1
ENSBTAG00000004153	*SCGB3A2*	8	0	3	7	0		0.72953145	1
ENSBTAG00000004154	*SKIL*	563	311	423	488	359	-0.441	0.629258438	1
ENSBTAG00000004155	*SPATA20*	1'663	647	1'094	1'440	747	-0.947	0.251003997	1
ENSBTAG00000004159	*BT.86129*	112	24	62	97	28	-1.807	0.261585512	1
ENSBTAG00000004161	*UBE2D2*	5'729	2'625	3'996	4'961	3'031	-0.711	0.361094942	1
ENSBTAG00000004165	*CLYBL*	1'464	398	864	1'268	460	-1.464	0.084724798	1
ENSBTAG00000004167	*TTC29*	4	1	2	3	1	-1.585	0.952247636	1
ENSBTAG00000004168	*STON1*	3'510	3'577	3'585	3'040	4'130	0.442	0.569069523	1
ENSBTAG00000004170	*pseudogene*	1	1	1	1	1	0.415	1	1
ENSBTAG00000004171	*BT.53642*	802	578	681	695	667	-0.057	0.948865358	1
ENSBTAG00000004172	*SETD1B*	859	1'506	1'241	744	1'739	1.225	0.135751299	1
ENSBTAG00000004173	*UBXN8*	973	671	809	843	775	-0.121	0.887335924	1
ENSBTAG00000004175	*HPD*	5	3	4	4	3	-0.322	1	1
ENSBTAG00000004176	*PDC*	1	1	1	1	1	0.415	1	1
ENSBTAG00000004177	*TANC1*	1'723	999	1'323	1'492	1'154	-0.371	0.647209312	1
ENSBTAG00000004178	*BT.19539*	424	201	300	367	232	-0.662	0.495357582	1
ENSBTAG00000004179	*PSMD9*	1'722	1'496	1'609	1'491	1'727	0.212	0.790698352	1
ENSBTAG00000004184	*WDSUB1*	1'488	1'095	1'277	1'289	1'264	-0.027	0.974798718	1
ENSBTAG00000004188	*BT.61360*	674	757	729	584	874	0.583	0.492643702	1
ENSBTAG00000004189	*MLXIP*	395	925	705	342	1'068	1.643	0.058754491	1
ENSBTAG00000004190	*ARHGAP29*	2'913	1'670	2'226	2'523	1'928	-0.388	0.623925397	1
ENSBTAG00000004191	*JMJD4*	756	776	775	655	896	0.453	0.591386682	1
ENSBTAG00000004192	*LRRC43*	4	0	2	3	0		0.89545886	1
ENSBTAG00000004193	*VEZF1*	7'467	3'764	5'406	6'467	4'346	-0.573	0.458368014	1
ENSBTAG00000004194	*BT.42611*	2'009	2'694	2'425	1'740	3'111	0.838	0.288369374	1
ENSBTAG00000004196	*FMNL1*	608	427	510	527	493	-0.095	0.917564661	1
ENSBTAG00000004197	*B3GNT4*	4	0	2	3	0		0.89545886	1
ENSBTAG00000004199	*DIABLO*	2'430	1'638	1'998	2'104	1'891	-0.154	0.846925701	1
ENSBTAG00000004200	*GTPBP3*	1'563	1'253	1'400	1'354	1'447	0.096	0.905174277	1
ENSBTAG00000004203	*BT.59536*	1'681	1'207	1'425	1'456	1'394	-0.063	0.939339134	1
ENSBTAG00000004204	*GPR107*	353	653	530	306	754	1.302	0.143648709	1
ENSBTAG00000004206	*LRRC55*	27	13	19	23	15	-0.639	0.839610081	1
ENSBTAG00000004207	*CD93*	6'733	3'387	4'871	5'831	3'911	-0.576	0.456854575	1

Ensembl gene ID	geneName	counts wildtype horn bud	counts polled horn bud	baseMean	baseMean wildtype horn bud	baseMean polled horn bud	log2FoldChange	pval	padj
ENSBTAG00000004208	*PLEKHG6*	95	219	168	82	253	1.620	0.150476522	1
ENSBTAG00000004211	*TNFRSF1A*	5'020	4'942	5'027	4'347	5'707	0.392	0.610724646	1
ENSBTAG00000004212	*BT.106191*	1	0	0	1	0		1	1
ENSBTAG00000004215	*RARRES2*	1'468	1'873	1'717	1'271	2'163	0.767	0.337968947	1
ENSBTAG00000004216	*MAP3K2*	63	30	45	55	35	-0.655	0.727930941	1
ENSBTAG00000004218	*BT.54651*	42	7	22	36	8	-2.170	0.431693274	1
ENSBTAG00000004221	*ESM1*	512	103	281	443	119	-1.898	0.059307026	1
ENSBTAG00000004222	*SAT2*	625	308	448	541	356	-0.606	0.503123528	1
ENSBTAG00000004223	*SRR*	1'138	716	906	986	827	-0.253	0.76190554	1
ENSBTAG00000004224	*C19ORF38*	20	8	13	17	9	-0.907	0.820299743	1
ENSBTAG00000004225	*TSR1*	2'151	2'053	2'117	1'863	2'371	0.348	0.659488596	1
ENSBTAG00000004226	*SHBG*	230	95	154	199	110	-0.861	0.451251438	1
ENSBTAG00000004227	*TBCK*	888	920	916	769	1'062	0.466	0.574709067	1
ENSBTAG00000004230	*SCYE1*	4'710	3'462	4'038	4'079	3'998	-0.029	0.971078269	1
ENSBTAG00000004232	*TMIE*	6	0	3	5	0		0.81115604	1
ENSBTAG00000004237	*BTC*	26	7	15	23	8	-1.478	0.671006397	1
ENSBTAG00000004238	*TACC1*	419	393	408	363	454	0.323	0.724866752	1
ENSBTAG00000004240	*BT.103427*	6'492	4'430	5'369	5'622	5'115	-0.136	0.860390242	1
ENSBTAG00000004242	*ARPC1A*	6'025	5'322	5'682	5'218	6'145	0.236	0.758616744	1
ENSBTAG00000004243	*LYPLA1*	2'511	1'607	2'015	2'175	1'856	-0.229	0.773482558	1
ENSBTAG00000004246	*H6PD*	251	399	339	217	461	1.084	0.253808866	1
ENSBTAG00000004247	*RPH3A*	40	14	25	35	16	-1.100	0.66319922	1
ENSBTAG00000004248	*MLYCD*	229	272	256	198	314	0.663	0.506924133	1
ENSBTAG00000004249	*TANC2*	387	518	467	335	598	0.836	0.35247551	1
ENSBTAG00000004256	*ODC1*	3'757	3'011	3'365	3'254	3'477	0.096	0.901742772	1
ENSBTAG00000004257	*TAF4B*	117	73	93	101	84	-0.266	0.850582247	1
ENSBTAG00000004258	*EEF2*	92'937	159'796	132'501	80'486	184'517	1.197	0.118867784	1
ENSBTAG00000004259	*HPCAL1*	794	643	715	688	742	0.111	0.897122148	1
ENSBTAG00000004261	*BT.18280*	4'176	6'267	5'427	3'617	7'237	1.001	0.197485031	1
ENSBTAG00000004262	*ZNF454*	532	240	369	461	277	-0.733	0.432404509	1
ENSBTAG00000004263	*ATP6V0A4*	224	97	153	194	112	-0.792	0.489085059	1
ENSBTAG00000004266	*DDX25*	122	11	59	106	13	-3.056	0.077697444	1
ENSBTAG00000004267	*CCDC148*	9	6	7	8	7	-0.170	1	1
ENSBTAG00000004268	*BT.57991*	943	931	946	817	1'075	0.397	0.632063599	1
ENSBTAG00000004269	*SGK1*	3'334	988	2'014	2'887	1'141	-1.340	0.095421622	1
ENSBTAG00000004270	*FCER2*	18	5	11	16	6	-1.433	0.753075742	1
ENSBTAG00000004271	*UBAP2L*	4'067	4'420	4'313	3'522	5'104	0.535	0.4895297	1
ENSBTAG00000004272	*ISG12(B)*	26	1	12	23	1	-4.285	0.366952216	1
ENSBTAG00000004273	*CST7*	76	60	68	66	69	0.074	0.971027757	1
ENSBTAG00000004275	*DKK3*	7'257	4'024	5'466	6'285	4'647	-0.436	0.572656783	1
ENSBTAG00000004277	*EVC2*	2'374	1'530	1'911	2'056	1'767	-0.219	0.783784475	1
ENSBTAG00000004278	*APMAP*	2'301	2'460	2'417	1'993	2'841	0.511	0.515465389	1
ENSBTAG00000004279	*RHOA*	9'865	7'323	8'500	8'543	8'456	-0.015	0.985382185	1
ENSBTAG00000004280	*SRPX*	10'295	9'056	9'686	8'916	10'457	0.230	0.763021924	1
ENSBTAG00000004281	*ACSS1*	1'298	1'258	1'288	1'124	1'453	0.370	0.647800078	1
ENSBTAG00000004282	*AMBRA1*	1'343	2'402	1'968	1'163	2'774	1.254	0.118051897	1
ENSBTAG00000004283	*PPFIBP1*	3'065	1'692	2'304	2'654	1'954	-0.442	0.575492515	1
ENSBTAG00000004284	*UFD1L*	3'432	2'671	3'028	2'972	3'084	0.053	0.945201613	1
ENSBTAG00000004286	*CDC45L*	1'116	1'407	1'296	966	1'625	0.749	0.356213765	1
ENSBTAG00000004287	*BT.87537*	929	1'237	1'116	805	1'428	0.828	0.313176469	1
ENSBTAG00000004288	*GSTA4*	5'061	2'190	3'456	4'383	2'529	-0.793	0.310166279	1
ENSBTAG00000004290	*DDX28*	334	475	419	289	548	0.923	0.312725953	1
ENSBTAG00000004291	*Sep 06*	1'426	1'747	1'626	1'235	2'017	0.708	0.377088089	1
ENSBTAG00000004294	*EVI5L*	2'078	2'754	2'490	1'800	3'180	0.821	0.29766182	1
ENSBTAG00000004295	*NDUFA8*	1'315	1'409	1'383	1'139	1'627	0.515	0.523614849	1
ENSBTAG00000004296	*MORN5*	26	11	18	23	13	-0.826	0.800203209	1
ENSBTAG00000004297	*ACOXL*	2	2	2	2	2	0.415	1	1
ENSBTAG00000004300	*LRRC8E*	122	116	120	106	134	0.342	0.783859553	1
ENSBTAG00000004302	*C20ORF7*	378	214	287	327	247	-0.406	0.679515393	1
ENSBTAG00000004303	*SLC27A2*	39	0	17	34	0		0.123198114	1
ENSBTAG00000004305	*RGS16*	639	575	609	553	664	0.263	0.761762647	1
ENSBTAG00000004307	*BT.68703*	5'492	2'801	3'995	4'756	3'234	-0.556	0.474199908	1
ENSBTAG00000004310	*UBR7*	2'689	2'253	2'465	2'329	2'602	0.160	0.838431891	1
ENSBTAG00000004313	*HOXD13*	2	0	1	2	0		0.974934741	1
ENSBTAG00000004315	*HDLBP*	17'910	23'038	21'056	15'511	26'602	0.778	0.308546362	1
ENSBTAG00000004316	*BT.53074*	3'396	1'879	2'555	2'941	2'170	-0.439	0.576931999	1
ENSBTAG00000004318	*ARL3*	3'163	2'310	2'703	2'739	2'667	-0.038	0.962106274	1
ENSBTAG00000004321	*SFXN2*	1'957	1'165	1'520	1'695	1'345	-0.333	0.6788729	1
ENSBTAG00000004322	*FOS*	1'571	494	965	1'361	570	-1.254	0.134221721	1
ENSBTAG00000004327	*LONRF2*	25	13	18	22	15	-0.528	0.875356394	1
ENSBTAG00000004328	*CHST10*	2'601	2'402	2'513	2'253	2'774	0.300	0.701760948	1
ENSBTAG00000004329	*RELL1*	311	251	280	269	290	0.106	0.9167457	1

Ensembl gene ID	geneName	counts wildtype horn bud	counts polled horn bud	baseMean	baseMean wildtype horn bud	baseMean polled horn bud	log2FoldChange	pval	padj
ENSBTAG00000004330	*ENO4*	98	42	67	85	48	-0.807	0.602079634	1
ENSBTAG00000004331	*PYGO2*	3'395	3'336	3'396	2'940	3'852	0.390	0.616151918	1
ENSBTAG00000004333	*COPA*	28'301	16'478	21'768	24'509	19'027	-0.365	0.631992905	1
ENSBTAG00000004334	*BT.48812*	4'307	4'550	4'492	3'730	5'254	0.494	0.522794001	1
ENSBTAG00000004337	*PDE1B*	1'035	520	748	896	600	-0.578	0.496031359	1
ENSBTAG00000004339	*protein_coding*	86	61	72	74	70	-0.080	0.96775092	1
ENSBTAG00000004342	*CCDC87*	6	7	7	5	8	0.637	0.939319729	1
ENSBTAG00000004343	*BT.53065*	2'228	2'261	2'270	1'930	2'611	0.436	0.579668782	1
ENSBTAG00000004344	*ACSL1*	5'296	2'656	3'827	4'586	3'067	-0.581	0.45565472	1
ENSBTAG00000004345	*MEP1A*	5	1	3	4	1	-1.907	0.9188325	1
ENSBTAG00000004347	*GPR116*	3'205	965	1'945	2'776	1'114	-1.317	0.101560796	1
ENSBTAG00000004348	*MRPL21*	877	682	774	760	788	0.052	0.951502299	1
ENSBTAG00000004349	*DAZAP2*	6'842	4'827	5'750	5'925	5'574	-0.088	0.909562699	1
ENSBTAG00000004351	*IL31RA*	6	13	10	5	15	1.531	0.740992914	1
ENSBTAG00000004352	*SLC5A7*	3	0	1	3	0		0.936647693	1
ENSBTAG00000004353	*CNOT6L*	463	378	419	401	436	0.122	0.894695708	1
ENSBTAG00000004355	*ROBO3*	28	0	12	24	0		0.230947873	1
ENSBTAG00000004356	*BT.22502*	3'410	1'826	2'531	2'953	2'108	-0.486	0.536825116	1
ENSBTAG00000004358	*DARS2*	687	322	483	595	372	-0.678	0.448678414	1
ENSBTAG00000004361	*ZBTB37*	58	26	40	50	30	-0.743	0.70760791	1
ENSBTAG00000004362	*SERPINC1*	72	24	45	62	28	-1.170	0.526927042	1
ENSBTAG00000004364	*THNSL2*	76	69	73	66	80	0.276	0.858255635	1
ENSBTAG00000004367	*COQ10A*	719	731	733	623	844	0.439	0.604736785	1
ENSBTAG00000004368	*BT.105181*	1'286	1'254	1'281	1'114	1'448	0.379	0.640138926	1
ENSBTAG00000004371	*CS*	3'763	2'315	2'966	3'259	2'673	-0.286	0.715172009	1
ENSBTAG00000004374	*CNPY2*	3'730	2'273	2'927	3'230	2'625	-0.300	0.702229622	1
ENSBTAG00000004375	*ESRP2*	1'746	2'037	1'932	1'512	2'352	0.637	0.422310753	1
ENSBTAG00000004376	*PAN2*	2'773	2'038	2'377	2'401	2'353	-0.029	0.971595105	1
ENSBTAG00000004377	*BT.27838*	773	801	797	669	925	0.466	0.579247231	1
ENSBTAG00000004378	*IL23A*	473	310	384	410	358	-0.195	0.836089528	1
ENSBTAG00000004379	*ETHE1*	518	588	564	449	679	0.598	0.494042562	1
ENSBTAG00000004380	*STAT2*	2'947	2'321	2'616	2'552	2'680	0.071	0.928061186	1
ENSBTAG00000004381	*TACSTD2*	1'140	1'026	1'086	987	1'185	0.263	0.74816225	1
ENSBTAG00000004383	*FNBP1L*	4'535	2'828	3'596	3'927	3'265	-0.266	0.732577123	1
ENSBTAG00000004384	*APOF*	0	1	1	0	1	Inf	0.993540919	1
ENSBTAG00000004386	*SOCS1*	227	455	361	197	525	1.418	0.133769146	1
ENSBTAG00000004387	*MTPAP*	1'212	648	899	1'050	748	-0.488	0.55850899	1
ENSBTAG00000004388	*SLITRK6*	1'327	1'409	1'388	1'149	1'627	0.502	0.534108566	1
ENSBTAG00000004392	*BT.68933*	2'163	2'520	2'392	1'873	2'910	0.635	0.419884557	1
ENSBTAG00000004394	*PKIB*	1'115	401	714	966	463	-1.060	0.216252201	1
ENSBTAG00000004398	*IMMP2L*	217	155	183	188	179	-0.070	0.954333606	1
ENSBTAG00000004399	*LRRN3*	2'078	742	1'328	1'800	857	-1.071	0.189494796	1
ENSBTAG00000004401	*UGGT2*	1'956	891	1'361	1'694	1'029	-0.719	0.374985514	1
ENSBTAG00000004402	*BT.69099*	1'814	1'187	1'471	1'571	1'371	-0.197	0.807757177	1
ENSBTAG00000004403	*CD320*	746	1'022	913	646	1'180	0.869	0.297535378	1
ENSBTAG00000004405	*IQUB*	41	5	21	36	6	-2.621	0.372060946	1
ENSBTAG00000004406	*MSI2*	62	51	56	54	59	0.133	0.945609968	1
ENSBTAG00000004407	*KCNK2*	511	288	388	443	333	-0.412	0.656793073	1
ENSBTAG00000004409	*IVD*	3'177	2'593	2'873	2'751	2'994	0.122	0.875612044	1
ENSBTAG00000004411	*CRISPLD1*	2'849	1'605	2'160	2'467	1'853	-0.413	0.601942484	1
ENSBTAG00000004412	*TMPRSS11F*	1	2	2	1	2	1.415	0.981979269	1
ENSBTAG00000004413	*RHOBTB3*	1'885	1'005	1'396	1'632	1'160	-0.492	0.542578515	1
ENSBTAG00000004414	*SLC30A10*	5	1	3	4	1	-1.907	0.9188325	1
ENSBTAG00000004415	*processed_pseudogene*	102	73	86	88	84	-0.068	0.971159037	1
ENSBTAG00000004416	*NUDC*	4'031	4'040	4'078	3'491	4'665	0.418	0.589082596	1
ENSBTAG00000004420	*BAHD1*	894	911	913	774	1'052	0.442	0.594526105	1
ENSBTAG00000004421	*CHST14*	2'382	2'664	2'569	2'063	3'076	0.576	0.462906892	1
ENSBTAG00000004422	*ALG14*	871	799	838	754	923	0.291	0.728532891	1
ENSBTAG00000004423	*FLJ32810*	268	166	212	232	192	-0.276	0.79515974	1
ENSBTAG00000004425	*BBS10*	130	92	109	113	106	-0.084	0.955862625	1
ENSBTAG00000004426	*DNAJB14*	284	164	218	246	189	-0.377	0.718989319	1
ENSBTAG00000004427	*OSBPL8*	1'899	1'030	1'417	1'645	1'189	-0.468	0.562732492	1
ENSBTAG00000004428	*H2AFZ*	5'574	4'157	4'814	4'827	4'800	-0.008	0.992588563	1
ENSBTAG00000004429	*BT.86382*	18	3	10	16	3	-2.170	0.662488565	1
ENSBTAG00000004430	*SGPP1*	1'605	1'004	1'275	1'390	1'159	-0.262	0.747920381	1
ENSBTAG00000004432	*CHRDL1*	1'210	1'136	1'180	1'048	1'312	0.324	0.690725515	1
ENSBTAG00000004436	*PPT2*	1'711	1'518	1'617	1'482	1'753	0.242	0.761576006	1
ENSBTAG00000004437	*pseudogene*	0	3	2	0	3	Inf	0.878646179	1
ENSBTAG00000004438	*XPNPEP1*	3'207	2'752	2'978	2'777	3'178	0.194	0.803050409	1
ENSBTAG00000004440	*BT.19972*	127	45	81	110	52	-1.082	0.448469808	1
ENSBTAG00000004442	*AGPAT1*	4'194	4'338	4'321	3'632	5'009	0.464	0.54893223	1
ENSBTAG00000004443	*protein_coding*	1	0	0	1	0		1	1

Ensembl gene ID	geneName	counts wildtype horn bud	counts polled horn bud	baseMean	baseMean wildtype horn bud	baseMean polled horn bud	log2FoldChange	pval	padj
ENSBTAG00000004448	*CKAP5*	5'871	6'522	6'308	5'084	7'531	0.567	0.46151302	1
ENSBTAG00000004449	*DGKE*	211	129	166	183	149	-0.295	0.795465856	1
ENSBTAG00000004452	*IPO9*	4'212	6'491	5'571	3'648	7'495	1.039	0.181010971	1
ENSBTAG00000004456	*TECTA*	14	2	7	12	2	-2.392	0.704665469	1
ENSBTAG00000004457	*ORAI1*	697	770	746	604	889	0.559	0.509437178	1
ENSBTAG00000004458	*MORN3*	14	4	8	12	5	-1.392	0.803716462	1
ENSBTAG00000004459	*TMEM45A*	4'023	2'275	3'055	3'484	2'627	-0.407	0.602519536	1
ENSBTAG00000004460	*RFWD3*	1'030	1'139	1'104	892	1'315	0.560	0.49409947	1
ENSBTAG00000004461	*BT.105716*	575	536	558	498	619	0.314	0.72012732	1
ENSBTAG00000004462	*NPAS3*	118	19	62	102	22	-2.220	0.174619318	1
ENSBTAG00000004463	*KDELR1*	4'415	5'059	4'833	3'824	5'842	0.611	0.429134314	1
ENSBTAG00000004464	*C17ORF53*	106	132	122	92	152	0.732	0.5499545	1
ENSBTAG00000004468	*BARX1*	4	14	10	3	16	2.222	0.638389235	1
ENSBTAG00000004471	*ST3GAL6*	2'632	774	1'587	2'279	894	-1.351	0.096438288	1
ENSBTAG00000004472	*DYNLT1*	1'100	1'007	1'058	953	1'163	0.288	0.726060183	1
ENSBTAG00000004476	*BT.47646*	260	37	134	225	43	-2.398	0.05304698	1
ENSBTAG00000004484	*CCDC41*	446	230	326	386	266	-0.540	0.57167523	1
ENSBTAG00000004488	*MEI1*	107	38	68	93	44	-1.079	0.480852792	1
ENSBTAG00000004489	*APPBP2*	1'743	1'230	1'465	1'509	1'420	-0.088	0.914426303	1
ENSBTAG00000004490	*TRIM31*	9	4	6	8	5	-0.755	0.931866093	1
ENSBTAG00000004492	*C16H1ORF26*	914	418	637	792	483	-0.714	0.409047409	1
ENSBTAG00000004494	*GALGT*	851	310	547	737	358	-1.042	0.238484905	1
ENSBTAG00000004495	*UBA5*	3'163	1'763	2'387	2'739	2'036	-0.428	0.587064102	1
ENSBTAG00000004496	*FAM18B1*	5'265	2'799	3'896	4'560	3'232	-0.496	0.523143358	1
ENSBTAG00000004497	*BT.102077*	1'508	2'635	2'174	1'306	3'043	1.220	0.126435994	1
ENSBTAG00000004498	*ESR2*	3	3	3	3	3	0.415	1	1
ENSBTAG00000004499	*RGS3*	4'449	2'784	3'534	3'853	3'215	-0.261	0.737536537	1
ENSBTAG00000004502	*CLUAP1*	1'898	1'114	1'465	1'644	1'286	-0.354	0.661004969	1
ENSBTAG00000004505	*NLRC3*	28	5	15	24	6	-2.070	0.558765873	1
ENSBTAG00000004507	*FAM163B*	48	0	21	42	0		0.074622382	1
ENSBTAG00000004508	*DBH*	4	3	3	3	3	0.000	1	1
ENSBTAG00000004509	*SLX4*	1'980	2'176	2'114	1'715	2'513	0.551	0.485712688	1
ENSBTAG00000004510	*BT.45831*	729	1'197	1'007	631	1'382	1.130	0.174056389	1
ENSBTAG00000004511	*VAV2*	2'310	2'492	2'439	2'001	2'878	0.524	0.504754387	1
ENSBTAG00000004512	*MKRN2*	665	570	617	576	658	0.193	0.824167016	1
ENSBTAG00000004514	*RAF1*	938	1'697	1'386	812	1'960	1.270	0.1197499	1
ENSBTAG00000004515	*KCNK1*	626	470	542	542	543	0.002	1	1
ENSBTAG00000004517	*TARBP1*	3'405	1'364	2'262	2'949	1'575	-0.905	0.254057484	1
ENSBTAG00000004518	*GRHL2*	358	680	548	310	785	1.341	0.130986889	1
ENSBTAG00000004521	*LSM7*	1'494	1'327	1'413	1'294	1'532	0.244	0.761759618	1
ENSBTAG00000004524	*BT.35019*	1'057	1'456	1'298	915	1'681	0.877	0.281013731	1
ENSBTAG00000004526	*TPT1*	13'071	15'097	14'376	11'320	17'433	0.623	0.414957771	1
ENSBTAG00000004531	*CCT4*	8'632	8'975	8'919	7'476	10'363	0.471	0.538227801	1
ENSBTAG00000004532	*BT.44742*	647	332	472	560	383	-0.548	0.542109661	1
ENSBTAG00000004538	*BT.25852*	462	292	369	400	337	-0.247	0.793329764	1
ENSBTAG00000004539	*C6ORF15*	6	0	3	5	0		0.81115604	1
ENSBTAG00000004540	*NUB1*	1'498	1'078	1'271	1'297	1'245	-0.060	0.943036254	1
ENSBTAG00000004541	*LPAR5*	90	68	78	78	79	0.011	1	1
ENSBTAG00000004542	*C9ORF59*	157	115	134	136	133	-0.034	0.985083781	1
ENSBTAG00000004544	*TXNDC9*	2'973	1'518	2'164	2'575	1'753	-0.555	0.483537597	1
ENSBTAG00000004547	*OLR1*	25	1	11	22	1	-4.229	0.384445569	1
ENSBTAG00000004549	*BT.47162*	885	679	775	766	784	0.033	0.969962182	1
ENSBTAG00000004551	*BT.58492*	1'861	2'083	2'008	1'612	2'405	0.578	0.466112587	1
ENSBTAG00000004552	*PBXIP1*	2'966	3'031	3'034	2'569	3'500	0.446	0.567307874	1
ENSBTAG00000004553	*TPM4*	24'471	29'843	27'826	21'193	34'460	0.701	0.357774439	1
ENSBTAG00000004554	*CAPZB*	5'591	5'982	5'875	4'842	6'907	0.513	0.505611454	1
ENSBTAG00000004555	*LRP2*	178	134	154	154	155	0.005	1	1
ENSBTAG00000004556	*SLC2A3*	4'182	1'318	2'572	3'622	1'522	-1.251	0.11546393	1
ENSBTAG00000004558	*bta-mir-147*	54	13	31	47	15	-1.639	0.467492948	1
ENSBTAG00000004560	*CLEC4F*	14	26	21	12	30	1.308	0.637986085	1
ENSBTAG00000004562	*CD207*	13	12	13	11	14	0.300	0.958035022	1
ENSBTAG00000004564	*BT.103671*	854	464	638	740	536	-0.465	0.590374995	1
ENSBTAG00000004567	*C1D*	803	445	605	695	514	-0.437	0.615657297	1
ENSBTAG00000004568	*GALNS*	643	790	735	557	912	0.712	0.401885723	1
ENSBTAG00000004570	*PRRX1*	5'449	3'267	4'246	4'719	3'772	-0.323	0.677248134	1
ENSBTAG00000004571	*PRPF4*	2'914	1'690	2'238	2'524	1'951	-0.371	0.638874168	1
ENSBTAG00000004572	*RNF183*	4	0	2	3	0		0.89545886	1
ENSBTAG00000004574	*BT.63571*	440	224	320	381	259	-0.559	0.559869231	1
ENSBTAG00000004575	*FAM126A*	415	297	351	359	343	-0.068	0.946465936	1
ENSBTAG00000004578	*LGI3*	353	872	656	306	1'007	1.720	0.050064638	1
ENSBTAG00000004580	*BT.53744*	20	2	10	17	2	-2.907	0.559880543	1
ENSBTAG00000004581	*BT.59489*	0	1	1	0	1	Inf	0.993540919	1

Ensembl gene ID	geneName	counts wildtype horn bud	counts polled horn bud	baseMean	baseMean wildtype horn bud	baseMean polled horn bud	log2FoldChange	pval	padj
ENSBTAG00000004582	*COL20A1*	337	255	293	292	294	0.013	0.992822665	1
ENSBTAG00000004585	*CCDC30*	461	96	255	399	111	-1.849	0.071565105	1
ENSBTAG00000004586	*ALG13*	1'138	481	770	986	555	-0.827	0.329352902	1
ENSBTAG00000004587	*DUSP6*	1'009	359	644	874	415	-1.076	0.21481159	1
ENSBTAG00000004588	*KCNN4*	41	12	25	36	14	-1.358	0.595766107	1
ENSBTAG00000004590	*PPIH*	1'564	1'018	1'265	1'354	1'175	-0.204	0.802243222	1
ENSBTAG00000004592	*SNAPC3*	944	480	686	818	554	-0.561	0.512839849	1
ENSBTAG00000004593	*BT.105991*	21'572	14'237	17'561	18'682	16'439	-0.184	0.809276693	1
ENSBTAG00000004594	*MMP7*	35	28	31	30	32	0.093	0.98159261	1
ENSBTAG00000004595	*BT.53617*	2	0	1	2	0		0.974934741	1
ENSBTAG00000004597	*PIM2*	443	346	392	384	400	0.059	0.951504934	1
ENSBTAG00000004598	*GOLGA5*	3'432	2'276	2'800	2'972	2'628	-0.178	0.821405712	1
ENSBTAG00000004600	*OTUD5*	4'606	4'976	4'867	3'989	5'746	0.527	0.495504817	1
ENSBTAG00000004601	*ABHD10*	1'735	908	1'276	1'503	1'048	-0.519	0.523163568	1
ENSBTAG00000004602	*PITX1*	221	85	145	191	98	-0.963	0.408321507	1
ENSBTAG00000004603	*PHC2*	4'408	5'140	4'876	3'817	5'935	0.637	0.410429632	1
ENSBTAG00000004604	*CCDC124*	889	1'142	1'044	770	1'319	0.776	0.34638627	1
ENSBTAG00000004607	*GSG1L*	29	0	13	25	0		0.217991529	1
ENSBTAG00000004608	*BT.26763*	5	1	3	4	1	-1.907	0.9188325	1
ENSBTAG00000004612	*SORCS3*	41	7	22	36	8	-2.135	0.444042087	1
ENSBTAG00000004613	*BT.64547*	2'209	680	1'349	1'913	785	-1.285	0.116575874	1
ENSBTAG00000004617	*BT.19086*	370	444	417	320	513	0.678	0.457554648	1
ENSBTAG00000004620	*ZNF133*	2'142	1'988	2'075	1'855	2'296	0.307	0.697086714	1
ENSBTAG00000004624	*TUBB*	4'899	9'533	7'625	4'243	11'008	1.375	0.077482256	1
ENSBTAG00000004625	*PYGB*	1'366	2'096	1'802	1'183	2'420	1.033	0.197671829	1
ENSBTAG00000004629	*BT.29773*	61	76	70	53	88	0.732	0.626622065	1
ENSBTAG00000004631	*KIAA0586*	1'079	573	798	934	662	-0.498	0.554976193	1
ENSBTAG00000004632	*protein_coding*	1'584	2'008	1'845	1'372	2'319	0.757	0.342197924	1
ENSBTAG00000004633	*PRSS48*	29	19	24	25	22	-0.195	0.957962741	1
ENSBTAG00000004635	*LLGL1*	4'727	6'330	5'701	4'094	7'309	0.836	0.27972183	1
ENSBTAG00000004636	*PLEKHA1*	1'791	1'375	1'569	1'551	1'588	0.034	0.966592788	1
ENSBTAG00000004637	*Mrz 10*	46	33	39	40	38	-0.064	0.989986625	1
ENSBTAG00000004639	*HARBI1*	444	360	400	385	416	0.112	0.904194759	1
ENSBTAG00000004640	*BT.22967*	154	32	85	133	37	-1.852	0.192577229	1
ENSBTAG00000004641	*BT.54613*	543	338	430	470	390	-0.269	0.769147726	1
ENSBTAG00000004643	*WASL*	1'858	1'002	1'383	1'609	1'157	-0.476	0.556407809	1
ENSBTAG00000004647	*UCP1*	4	0	2	3	0		0.89545886	1
ENSBTAG00000004650	*ZNF516*	318	417	378	275	482	0.806	0.385130502	1
ENSBTAG00000004651	*NME1*	1'269	905	1'072	1'099	1'045	-0.073	0.931229315	1
ENSBTAG00000004652	*BT.105429*	325	707	549	281	816	1.536	0.084912428	1
ENSBTAG00000004653	*GPR125*	4'594	2'766	3'586	3'979	3'194	-0.317	0.684155967	1
ENSBTAG00000004654	*PWP1*	3'692	2'648	3'128	3'197	3'058	-0.064	0.93517258	1
ENSBTAG00000004657	*BT.34956*	76	35	53	66	40	-0.704	0.682955345	1
ENSBTAG00000004658	*WEE2*	1	2	2	1	2	1.415	0.981979269	1
ENSBTAG00000004659	*BT.30256*	1'180	505	803	1'022	583	-0.809	0.337981844	1
ENSBTAG00000004660	*BT.24874*	2'942	2'554	2'748	2'548	2'949	0.211	0.787062624	1
ENSBTAG00000004662	*SLC16A12*	33	14	22	29	16	-0.822	0.766955725	1
ENSBTAG00000004663	*BT.20335*	601	416	500	520	480	-0.116	0.898999468	1
ENSBTAG00000004664	*COQ4*	507	583	556	439	673	0.617	0.481480619	1
ENSBTAG00000004672	*RDX*	12'147	9'595	10'799	10'520	11'079	0.075	0.921432366	1
ENSBTAG00000004674	*HAAO*	30	21	25	26	24	-0.100	0.987328991	1
ENSBTAG00000004675	*ZNF18*	499	436	468	432	503	0.220	0.806578725	1
ENSBTAG00000004676	*TMEM184A*	21	22	22	18	25	0.482	0.86964816	1
ENSBTAG00000004679	*WARS*	2'999	2'781	2'904	2'597	3'211	0.306	0.694794579	1
ENSBTAG00000004685	*SRGAP3*	597	839	743	517	969	0.906	0.286876073	1
ENSBTAG00000004688	*DHCR24*	1'838	3'316	2'710	1'592	3'829	1.266	0.110354536	1
ENSBTAG00000004690	*CD300E*	9	0	4	8	0		0.690676647	1
ENSBTAG00000004692	*processed_pseudogene*	228	482	377	197	557	1.495	0.112007536	1
ENSBTAG00000004693	*CCDC60*	31	14	22	27	16	-0.732	0.799069086	1
ENSBTAG00000004694	*RPGRIP1*	69	69	70	60	80	0.415	0.787090927	1
ENSBTAG00000004695	*CDK9*	7'019	6'041	6'527	6'079	6'976	0.199	0.795505353	1
ENSBTAG00000004705	*ILDR1*	4	5	5	3	6	0.737	0.954105567	1
ENSBTAG00000004706	*BT.25870*	654	893	799	566	1'031	0.864	0.305885696	1
ENSBTAG00000004709	*MAPK9*	479	376	424	415	434	0.066	0.944265411	1
ENSBTAG00000004713	*pseudogene*	25	142	93	22	164	2.921	0.040540607	1
ENSBTAG00000004714	*C19ORF59*	17	3	9	15	3	-2.087	0.685542914	1
ENSBTAG00000004715	*AKIRIN1*	4'456	2'252	3'230	3'859	2'600	-0.570	0.466164851	1
ENSBTAG00000004716	*RETN*	47	8	25	41	9	-2.140	0.405865602	1
ENSBTAG00000004718	*PUS7L*	967	542	732	837	626	-0.420	0.621612424	1
ENSBTAG00000004720	*SULF1*	621	263	421	538	304	-0.824	0.367433284	1
ENSBTAG00000004721	*GSG1*	10	0	4	9	0		0.653360153	1
ENSBTAG00000004722	*THUMPD2*	909	547	709	787	632	-0.318	0.710324418	1

Ensembl gene ID	geneName	counts wildtype horn bud	counts polled horn bud	baseMean	baseMean wildtype horn bud	baseMean polled horn bud	log2FoldChange	pval	padj
ENSBTAG00000004723	*BT.103073*	1'098	883	985	951	1'020	0.101	0.903371749	1
ENSBTAG00000004727	*PAQR4*	464	808	667	402	933	1.215	0.160056467	1
ENSBTAG00000004729	*BT.43297*	572	705	655	495	814	0.717	0.404608573	1
ENSBTAG00000004732	*SPTB*	347	737	576	301	851	1.502	0.089961281	1
ENSBTAG00000004735	*BT.60810*	128	102	114	111	118	0.087	0.950624085	1
ENSBTAG00000004736	*GRB2*	2'042	1'718	1'876	1'768	1'984	0.166	0.83437445	1
ENSBTAG00000004738	*LRRC47*	2'345	3'044	2'773	2'031	3'515	0.791	0.313731148	1
ENSBTAG00000004739	*SLC18A2*	697	239	440	604	276	-1.129	0.215909891	1
ENSBTAG00000004741	*IL12B*	4	0	2	3	0		0.89545886	1
ENSBTAG00000004742	*RUNX1*	344	228	281	298	263	-0.178	0.859246279	1
ENSBTAG00000004743	*protein_coding*	1	0	0	1	0		1	1
ENSBTAG00000004745	*NAA15*	3'582	2'152	2'794	3'102	2'485	-0.320	0.683306134	1
ENSBTAG00000004746	*HELZ*	1'224	658	910	1'060	760	-0.480	0.56447105	1
ENSBTAG00000004747	*SPCS1*	1'709	1'263	1'469	1'480	1'458	-0.021	0.980548076	1
ENSBTAG00000004749	*NEK4*	1'573	708	1'090	1'362	818	-0.737	0.370716397	1
ENSBTAG00000004751	*SPTBN4*	529	688	626	458	794	0.794	0.358533864	1
ENSBTAG00000004753	*BT.26185*	1'059	756	895	917	873	-0.071	0.933854044	1
ENSBTAG00000004755	*SHKBP1*	1'456	1'856	1'702	1'261	2'143	0.765	0.338991803	1
ENSBTAG00000004757	*LTBP4*	14'227	21'462	18'552	12'321	24'782	1.008	0.189121416	1
ENSBTAG00000004760	*PPYR1*	9	5	7	8	6	-0.433	0.969858938	1
ENSBTAG00000004761	*FOXH1*	5	2	3	4	2	-0.907	0.965043111	1
ENSBTAG00000004766	*BT.37161*	75	39	55	65	45	-0.528	0.757942066	1
ENSBTAG00000004767	*SYK*	199	139	166	172	161	-0.103	0.932708124	1
ENSBTAG00000004768	*RAI1*	1'902	2'992	2'551	1'647	3'455	1.069	0.176926478	1
ENSBTAG00000004769	*NEIL2*	190	153	171	165	177	0.103	0.930440347	1
ENSBTAG00000004771	*processed_pseudogene*	182	436	331	158	503	1.675	0.083216602	1
ENSBTAG00000004772	*BT.10185*	647	368	493	560	425	-0.399	0.6552782	1
ENSBTAG00000004776	*DUSP23*	135	101	117	117	117	-0.004	1	1
ENSBTAG00000004777	*S100B*	2'171	919	1'471	1'880	1'061	-0.825	0.30725358	1
ENSBTAG00000004780	*CDKL1*	160	221	197	139	255	0.881	0.408094733	1
ENSBTAG00000004781	*BT.42818*	1'579	1'183	1'367	1'367	1'366	-0.002	1	1
ENSBTAG00000004782	*PTPRCAP*	595	428	505	515	494	-0.060	0.948881413	1
ENSBTAG00000004783	*BT.49709*	2'608	2'974	2'846	2'259	3'434	0.604	0.440104282	1
ENSBTAG00000004785	*CABP4*	4	0	2	3	0		0.89545886	1
ENSBTAG00000004786	*TMEM134*	261	193	224	226	223	-0.020	0.989987802	1
ENSBTAG00000004787	*METRNL*	4'838	3'599	4'173	4'190	4'156	-0.012	0.988899818	1
ENSBTAG00000004788	*SDR39U1*	873	488	660	756	563	-0.424	0.622301193	1
ENSBTAG00000004789	*LGR6*	300	797	590	260	920	1.825	0.04060299	1
ENSBTAG00000004790	*UBE2T*	1'572	965	1'238	1'361	1'114	-0.289	0.723143	1
ENSBTAG00000004791	*DSCR3*	2'025	2'400	2'262	1'754	2'771	0.660	0.403117433	1
ENSBTAG00000004792	*CHMP4C*	305	111	196	264	128	-1.043	0.330221413	1
ENSBTAG00000004793	*AMBN*	2	0	1	2	0		0.974934741	1
ENSBTAG00000004795	*RIPPLY2*	41	0	18	36	0		0.11009249	1
ENSBTAG00000004796	*GRP*	8	1	4	7	1	-2.585	0.818599781	1
ENSBTAG00000004797	*METTL19*	2'466	1'709	2'055	2'136	1'973	-0.114	0.886585894	1
ENSBTAG00000004801	*PDXDC1*	6'295	4'276	5'195	5'452	4'937	-0.143	0.85376066	1
ENSBTAG00000004802	*KIAA0284*	803	1'359	1'132	695	1'569	1.174	0.154845248	1
ENSBTAG00000004803	*NTAN1*	4'981	4'146	4'551	4'314	4'787	0.150	0.845280479	1
ENSBTAG00000004804	*RRN3*	2'689	1'539	2'053	2'329	1'777	-0.390	0.622892078	1
ENSBTAG00000004805	*ITFG1*	5'839	4'048	4'865	5'057	4'674	-0.113	0.883979105	1
ENSBTAG00000004806	*PHKB*	2'616	1'539	2'021	2'266	1'777	-0.350	0.658968027	1
ENSBTAG00000004813	*SUSD1*	32	20	25	28	23	-0.263	0.93150743	1
ENSBTAG00000004814	*INPP4A*	1'348	870	1'086	1'167	1'005	-0.217	0.792828468	1
ENSBTAG00000004815	*protein_coding*	1'215	825	1'002	1'052	953	-0.143	0.863526251	1
ENSBTAG00000004817	*pseudogene*	380	946	711	329	1'092	1.731	0.046792001	1
ENSBTAG00000004820	*BT.91103*	318	379	357	275	438	0.668	0.475574048	1
ENSBTAG00000004822	*SOX10*	1'404	1'438	1'438	1'216	1'660	0.450	0.576386808	1
ENSBTAG00000004823	*MRPL35*	1'013	525	742	877	606	-0.533	0.530359698	1
ENSBTAG00000004826	*ACP5*	4'625	1'484	2'859	4'005	1'714	-1.225	0.121742251	1
ENSBTAG00000004829	*YIF1A*	2'529	2'765	2'691	2'190	3'193	0.544	0.487864794	1
ENSBTAG00000004832	*BT.105308*	1'254	438	796	1'086	506	-1.102	0.194097971	1
ENSBTAG00000004833	*BT.56298*	1'477	1'116	1'284	1'279	1'289	0.011	0.98986782	1
ENSBTAG00000004834	*CNOT10*	1'677	1'460	1'569	1'452	1'686	0.215	0.78803364	1
ENSBTAG00000004835	*BT.105461*	374	461	428	324	532	0.717	0.430480076	1
ENSBTAG00000004836	*NRK*	2'647	639	1'515	2'292	738	-1.635	0.046187163	1
ENSBTAG00000004838	*BT.88227*	849	973	929	735	1'124	0.612	0.461395073	1
ENSBTAG00000004840	*C1S*	6'009	1'526	3'483	5'204	1'762	-1.562	0.048973015	1
ENSBTAG00000004842	*RSPRY1*	2'505	1'477	1'937	2'169	1'705	-0.347	0.662504137	1
ENSBTAG00000004844	*ARL2BP*	1'614	747	1'130	1'398	863	-0.696	0.396022609	1
ENSBTAG00000004848	*OLFML2A*	1'199	1'930	1'633	1'038	2'229	1.102	0.171882659	1
ENSBTAG00000004849	*IGSF3*	1'369	1'263	1'322	1'186	1'458	0.299	0.711630188	1
ENSBTAG00000004850	*KPNA3*	699	692	702	605	799	0.401	0.638218554	1

Ensembl gene ID	geneName	counts wildtype horn bud	counts polled horn bud	baseMean	baseMean wildtype horn bud	baseMean polled horn bud	log2FoldChange	pval	padj
ENSBTAG00000004851	*NCKAP5L*	3'032	3'976	3'608	2'626	4'591	0.806	0.301503916	1
ENSBTAG00000004852	*BT.20245*	2'168	2'676	2'484	1'878	3'090	0.719	0.361444048	1
ENSBTAG00000004854	*SLC24A5*	1'431	515	917	1'239	595	-1.059	0.206059149	1
ENSBTAG00000004855	*PRDX6*	3'739	4'347	4'129	3'238	5'019	0.632	0.415064956	1
ENSBTAG00000004856	*RNF130*	2'984	1'765	2'311	2'584	2'038	-0.343	0.664411072	1
ENSBTAG00000004860	*SLC27A6*	142	140	142	123	162	0.395	0.736753482	1
ENSBTAG00000004861	*EIF3F*	8'739	9'448	9'239	7'568	10'910	0.528	0.490915874	1
ENSBTAG00000004862	*TUB*	501	669	603	434	772	0.832	0.338311311	1
ENSBTAG00000004863	*RIC3*	294	156	217	255	180	-0.499	0.632613801	1
ENSBTAG00000004864	*BT.103320*	739	606	670	640	700	0.129	0.881177264	1
ENSBTAG00000004869	*CXADR*	459	471	471	398	544	0.452	0.613531213	1
ENSBTAG00000004870	*GALNT2*	2'021	2'872	2'533	1'750	3'316	0.922	0.242860255	1
ENSBTAG00000004871	*NDUFV2*	1'028	694	846	890	801	-0.152	0.857789386	1
ENSBTAG00000004872	*MRPL20*	1'969	1'242	1'570	1'705	1'434	-0.250	0.756239149	1
ENSBTAG00000004873	*BT.39199*	6'613	3'165	4'691	5'727	3'655	-0.648	0.403297978	1
ENSBTAG00000004874	*AURKAIP1*	1'151	1'048	1'103	997	1'210	0.280	0.73239682	1
ENSBTAG00000004875	*NAA60*	1'356	1'568	1'492	1'174	1'811	0.625	0.437412483	1
ENSBTAG00000004876	*RABL2B*	5'876	3'604	4'625	5'089	4'162	-0.290	0.707992254	1
ENSBTAG00000004877	*BT.102673*	130	140	137	113	162	0.522	0.658821322	1
ENSBTAG00000004878	*CAMKV*	847	206	486	734	238	-1.625	0.074086896	1
ENSBTAG00000004879	*FOXO4*	1'501	1'307	1'405	1'300	1'509	0.215	0.789115024	1
ENSBTAG00000004881	*MTHFD2*	1'332	957	1'129	1'154	1'105	-0.062	0.941352553	1
ENSBTAG00000004884	*SLC4A5*	78	27	49	68	31	-1.115	0.528356651	1
ENSBTAG00000004885	*BT.46641*	870	670	764	753	774	0.038	0.96489943	1
ENSBTAG00000004886	*ZAR1*	10	1	5	9	1	-2.907	0.754028015	1
ENSBTAG00000004887	*DCTN1*	6'735	9'293	8'282	5'833	10'731	0.880	0.253697022	1
ENSBTAG00000004891	*OXNAD1*	717	368	523	621	425	-0.547	0.536516832	1
ENSBTAG00000004893	*ACOX3*	1'863	1'020	1'396	1'613	1'178	-0.454	0.574436258	1
ENSBTAG00000004894	*BT.79637*	9	6	7	8	7	-0.170	1	1
ENSBTAG00000004895	*AFAP1L2*	4'138	1'956	2'921	3'584	2'259	-0.666	0.395808583	1
ENSBTAG00000004896	*ABLIM3*	709	207	427	614	239	-1.361	0.139439852	1
ENSBTAG00000004899	*ABLIM1*	642	670	665	556	774	0.477	0.578091264	1
ENSBTAG00000004901	*PIK3C2A*	124	64	91	107	74	-0.539	0.695379938	1
ENSBTAG00000004902	*BT.66889*	3'159	2'177	2'625	2'736	2'514	-0.122	0.877260679	1
ENSBTAG00000004903	*KRT15*	18'921	18'476	18'860	16'386	21'334	0.381	0.616804867	1
ENSBTAG00000004905	*KRT19*	437	391	415	378	451	0.255	0.78105952	1
ENSBTAG00000004906	*BT.37175*	908	391	619	786	451	-0.800	0.356400616	1
ENSBTAG00000004907	*MINK1*	6'812	7'603	7'339	5'899	8'779	0.574	0.455318515	1
ENSBTAG00000004908	*CHRNE*	220	108	158	191	125	-0.611	0.590763578	1
ENSBTAG00000004909	*GP1BA*	5	0	2	4	0		0.853268594	1
ENSBTAG00000004910	*SLC25A11*	3'417	3'577	3'545	2'959	4'130	0.481	0.535992045	1
ENSBTAG00000004911	*BIRC7*	1	0	0	1	0		1	1
ENSBTAG00000004912	*ANKRD17*	6'118	3'547	4'697	5'298	4'096	-0.371	0.631467189	1
ENSBTAG00000004913	*RNF167*	2'571	2'404	2'501	2'227	2'776	0.318	0.684956866	1
ENSBTAG00000004915	*PFN1*	3'455	5'777	4'831	2'992	6'671	1.157	0.138299907	1
ENSBTAG00000004916	*ARFGAP3*	2'980	2'928	2'981	2'581	3'381	0.390	0.6175067	1
ENSBTAG00000004917	*BT.24545*	5	0	2	4	0		0.853268594	1
ENSBTAG00000004920	*BT.88403*	1'596	689	1'089	1'382	796	-0.797	0.333248181	1
ENSBTAG00000004921	*FAM47E*	308	158	225	267	182	-0.548	0.596531911	1
ENSBTAG00000004922	*AGPAT5*	3'341	1'691	2'423	2'893	1'953	-0.567	0.471781871	1
ENSBTAG00000004924	*PENK*	914	1'519	1'273	792	1'754	1.148	0.160817345	1
ENSBTAG00000004925	*ZNF845*	319	176	240	276	203	-0.443	0.664507351	1
ENSBTAG00000004926	*PPIL5*	176	144	159	152	166	0.126	0.915680951	1
ENSBTAG00000004929	*C5ORF51*	406	314	357	352	363	0.044	0.964708785	1
ENSBTAG00000004930	*bta-mir-6517*	407	266	330	352	307	-0.199	0.837418099	1
ENSBTAG00000004931	*BT.28135*	393	190	280	340	219	-0.633	0.519864051	1
ENSBTAG00000004933	*KLHDC2*	5'315	2'122	3'527	4'603	2'450	-0.910	0.245161101	1
ENSBTAG00000004934	*NEMF*	2'011	840	1'356	1'742	970	-0.844	0.298495416	1
ENSBTAG00000004936	*TMIGD2*	12	5	8	10	6	-0.848	0.892845055	1
ENSBTAG00000004937	*SEC61A1*	6'269	8'975	7'896	5'429	10'363	0.933	0.226760398	1
ENSBTAG00000004939	*ZNF569*	1'101	442	732	953	510	-0.902	0.290668867	1
ENSBTAG00000004940	*BRUNOL4*	181	27	94	157	31	-2.330	0.094468831	1
ENSBTAG00000004941	*BT.54888*	4	1	2	3	1	-1.585	0.952247636	1
ENSBTAG00000004942	*EXOSC9*	2'191	1'021	1'538	1'897	1'179	-0.687	0.393749506	1
ENSBTAG00000004943	*BT.87491*	508	406	454	440	469	0.092	0.920437538	1
ENSBTAG00000004945	*BBS7*	772	359	542	669	415	-0.690	0.434080089	1
ENSBTAG00000004948	*FAM84A*	1'924	1'313	1'591	1'666	1'516	-0.136	0.866152289	1
ENSBTAG00000004949	*FDXACB1*	76	73	75	66	84	0.357	0.811692939	1
ENSBTAG00000004950	*BRN*	4'196	2'671	3'359	3'634	3'084	-0.237	0.76192924	1
ENSBTAG00000004951	*C15H11orf1*	4	4	4	3	5	0.415	0.994759363	1
ENSBTAG00000004952	*MFSD4*	366	181	263	317	209	-0.601	0.547032694	1
ENSBTAG00000004953	*ELK4*	94	86	90	81	99	0.287	0.837738195	1

Ensembl gene ID	geneName	counts wildtype horn bud	counts polled horn bud	baseMean	baseMean wildtype horn bud	baseMean polled horn bud	log2FoldChange	pval	padj
ENSBTAG00000004956	*CHEK2*	235	176	203	204	203	-0.002	1	1
ENSBTAG00000004958	*PTPRN2*	108	6	50	94	7	-3.755	0.052893299	1
ENSBTAG00000004961	*GOSR1*	515	300	396	446	346	-0.365	0.693566927	1
ENSBTAG00000004962	*BCORL1*	1'011	1'920	1'546	876	2'217	1.340	0.09918613	1
ENSBTAG00000004964	*PCGF5*	155	133	144	134	154	0.194	0.871163239	1
ENSBTAG00000004965	*BT.70137*	3'113	2'211	2'624	2'696	2'553	-0.079	0.92127325	1
ENSBTAG00000004966	*KIF1B*	1'199	1'000	1'097	1'038	1'155	0.153	0.851735992	1
ENSBTAG00000004969	*LRRC14*	615	766	709	533	885	0.732	0.390823255	1
ENSBTAG00000004970	*LRRC24*	480	448	466	416	517	0.316	0.72527262	1
ENSBTAG00000004971	*GRAMD1C*	817	521	655	708	602	-0.234	0.786918229	1
ENSBTAG00000004974	*ZDHHC23*	22	34	29	19	39	1.043	0.650839179	1
ENSBTAG00000004975	*KIAA1407*	164	193	182	142	223	0.650	0.54937873	1
ENSBTAG00000004976	*BT.61280*	1'764	2'354	2'123	1'528	2'718	0.831	0.294742683	1
ENSBTAG00000004977	*QTRTD1*	234	291	269	203	336	0.730	0.460743054	1
ENSBTAG00000004979	*BT.69496*	1'237	664	919	1'071	767	-0.483	0.562399314	1
ENSBTAG00000004982	*GPLD1*	17	15	16	15	17	0.234	0.961701465	1
ENSBTAG00000004987	*NUP210L*	1'058	469	729	916	542	-0.759	0.373403599	1
ENSBTAG00000004988	*CCKAR*	21	10	15	18	12	-0.655	0.864027356	1
ENSBTAG00000004989	*IRF5*	267	226	246	231	261	0.175	0.864978287	1
ENSBTAG00000004990	*NEO1*	6'244	3'327	4'625	5'407	3'842	-0.493	0.524401543	1
ENSBTAG00000004991	*IKBKAP*	805	762	789	697	880	0.336	0.689963463	1
ENSBTAG00000004992	*TNPO3*	2'110	1'847	1'980	1'827	2'133	0.223	0.778021344	1
ENSBTAG00000004993	*BT.69917*	367	367	371	318	424	0.415	0.655547272	1
ENSBTAG00000004994	*pseudogene*	3'396	7'306	5'689	2'941	8'436	1.520	0.052781019	1
ENSBTAG00000004995	*C1GALT1*	563	413	482	488	477	-0.032	0.974755038	1
ENSBTAG00000004996	*FAM206A*	1'552	889	1'185	1'344	1'027	-0.389	0.634196645	1
ENSBTAG00000004997	*CTNNAL1*	3'251	2'359	2'770	2'815	2'724	-0.048	0.952570401	1
ENSBTAG00000004999	*C5H12orf35*	2'652	1'061	1'761	2'297	1'225	-0.907	0.258098051	1
ENSBTAG00000005002	*GALE*	1'240	695	938	1'074	803	-0.420	0.613394754	1
ENSBTAG00000005003	*BT.56277*	3	1	2	3	1	-1.170	0.985253077	1
ENSBTAG00000005004	*BT.106544*	3	0	1	3	0		0.936647693	1
ENSBTAG00000005007	*processed_pseudogene*	66	144	112	57	166	1.541	0.226021637	1
ENSBTAG00000005008	*WSB1*	26'843	8'923	16'775	23'247	10'303	-1.174	0.127846203	1
ENSBTAG00000005009	*PRELID1*	2'490	3'022	2'823	2'156	3'490	0.694	0.375828469	1
ENSBTAG00000005010	*MXD3*	373	470	433	323	543	0.749	0.409682143	1
ENSBTAG00000005012	*HSPH1*	5'156	2'966	3'945	4'465	3'425	-0.383	0.622410286	1
ENSBTAG00000005014	*BT.63292*	240	424	349	208	490	1.236	0.19213255	1
ENSBTAG00000005015	*SFXN3*	3'369	2'228	2'745	2'918	2'573	-0.182	0.817522617	1
ENSBTAG00000005016	*AP3B1*	5'492	5'504	5'556	4'756	6'355	0.418	0.586992272	1
ENSBTAG00000005017	*ZNF169*	206	71	130	178	82	-1.122	0.352356011	1
ENSBTAG00000005018	*NFAM1*	25	12	18	22	14	-0.644	0.847278186	1
ENSBTAG00000005019	*PRSS50*	7	1	4	6	1	-2.392	0.851750348	1
ENSBTAG00000005021	*SEMA5A*	761	590	670	659	681	0.048	0.956543187	1
ENSBTAG00000005025	*FAM91A1*	1'717	1'196	1'434	1'487	1'381	-0.107	0.896038263	1
ENSBTAG00000005026	*MANEA*	473	384	427	410	443	0.114	0.901463714	1
ENSBTAG00000005027	*GLRX3*	3'683	3'171	3'426	3'190	3'662	0.199	0.797508674	1
ENSBTAG00000005028	*HIRIP3*	1'435	1'112	1'263	1'243	1'284	0.047	0.953963689	1
ENSBTAG00000005029	*TAL1*	162	83	118	140	96	-0.550	0.659783049	1
ENSBTAG00000005030	*INO80E*	487	543	524	422	627	0.572	0.516754621	1
ENSBTAG00000005031	*DOC2A*	82	46	62	71	53	-0.419	0.798232341	1
ENSBTAG00000005034	*CAPN5*	2'724	2'337	2'529	2'359	2'699	0.194	0.804405314	1
ENSBTAG00000005038	*ZBTB42*	87	175	139	75	202	1.423	0.229420285	1
ENSBTAG00000005039	*ARAF*	2'338	2'609	2'519	2'025	3'013	0.573	0.465675153	1
ENSBTAG00000005040	*SMEK1*	4'874	2'722	3'682	4'221	3'143	-0.425	0.584724681	1
ENSBTAG00000005042	*SYN1*	143	36	83	124	42	-1.575	0.269547531	1
ENSBTAG00000005043	*TIMP1*	7'766	3'911	5'621	6'726	4'516	-0.575	0.457016352	1
ENSBTAG00000005045	*GIP*	2	0	1	2	0		0.974934741	1
ENSBTAG00000005049	*BLK*	14	2	7	12	2	-2.392	0.704665469	1
ENSBTAG00000005052	*GLP2R*	7	4	5	6	5	-0.392	0.98714753	1
ENSBTAG00000005055	*TXK*	17	7	11	15	8	-0.865	0.849311167	1
ENSBTAG00000005057	*FAM204A*	3'184	1'438	2'209	2'757	1'660	-0.732	0.355686822	1
ENSBTAG00000005058	*C18H16orf70*	449	449	454	389	518	0.415	0.644890054	1
ENSBTAG00000005062	*TEC*	850	306	545	736	353	-1.059	0.231321247	1
ENSBTAG00000005063	*THEM6*	106	98	102	92	113	0.302	0.820203769	1
ENSBTAG00000005064	*WARS2*	606	780	713	525	901	0.779	0.36085048	1
ENSBTAG00000005066	*HSPBAP1*	394	183	276	341	211	-0.691	0.483651606	1
ENSBTAG00000005067	*UBTF*	5'735	6'344	6'146	4'967	7'325	0.561	0.46648766	1
ENSBTAG00000005068	*COMMD2*	1'137	1'352	1'273	985	1'561	0.665	0.41300706	1
ENSBTAG00000005069	*TM7SF2*	665	526	592	576	607	0.077	0.930771396	1
ENSBTAG00000005071	*DIRC2*	845	445	623	732	514	-0.510	0.555924469	1
ENSBTAG00000005072	*CUBN*	208	236	226	180	273	0.597	0.56134085	1
ENSBTAG00000005073	*ZNHIT2*	165	221	199	143	255	0.837	0.430680954	1

Ensembl gene ID	geneName	counts wildtype horn bud	counts polled horn bud	baseMean	baseMean wildtype horn bud	baseMean polled horn bud	log2FoldChange	pval	padj
ENSBTAG00000005075	*BT.21430*	2'745	2'759	2'782	2'377	3'186	0.422	0.589154652	1
ENSBTAG00000005076	*BT.54995*	2'134	2'148	2'164	1'848	2'480	0.424	0.59059144	1
ENSBTAG00000005077	*CXCL12*	11'662	10'658	11'203	10'100	12'307	0.285	0.70842125	1
ENSBTAG00000005082	*MED21*	2'900	2'313	2'591	2'511	2'671	0.089	0.90962185	1
ENSBTAG00000005083	*TULP4*	759	759	767	657	876	0.415	0.622956815	1
ENSBTAG00000005085	*TRIM63*	43	27	34	37	31	-0.256	0.916700934	1
ENSBTAG00000005086	*RPRD1B*	2'658	1'854	2'221	2'302	2'141	-0.105	0.895550394	1
ENSBTAG00000005089	*BT.56703*	1'130	957	1'042	979	1'105	0.175	0.831250055	1
ENSBTAG00000005090	*TMEM2*	3'449	1'449	2'330	2'987	1'673	-0.836	0.29091736	1
ENSBTAG00000005091	*DGKG*	11	27	20	10	31	1.710	0.547124159	1
ENSBTAG00000005092	*BT.56413*	2'674	4'009	3'472	2'316	4'629	0.999	0.202129478	1
ENSBTAG00000005093	*BT.20720*	185	131	156	160	151	-0.083	0.948434119	1
ENSBTAG00000005094	*LRP2BP*	61	36	47	53	42	-0.346	0.85674643	1
ENSBTAG00000005096	*COX7A2*	2'845	1'542	2'122	2'464	1'781	-0.469	0.554124205	1
ENSBTAG00000005098	*protein_coding*	1	0	0	1	0		1	1
ENSBTAG00000005099	*pseudogene*	1'375	2'080	1'796	1'191	2'402	1.012	0.206621012	1
ENSBTAG00000005100	*TMEM30A*	2'297	1'568	1'900	1'989	1'811	-0.136	0.865311327	1
ENSBTAG00000005102	*PHTF2*	947	639	779	820	738	-0.153	0.858312266	1
ENSBTAG00000005104	*BT.103136*	312	78	180	270	90	-1.585	0.152363971	1
ENSBTAG00000005105	*PAFAH2*	881	766	824	763	885	0.213	0.799452238	1
ENSBTAG00000005107	*CRADD*	126	72	96	109	83	-0.392	0.773054706	1
ENSBTAG00000005108	*SLIT2*	1'575	529	987	1'364	611	-1.159	0.164768662	1
ENSBTAG00000005111	*BMP2*	843	413	603	730	477	-0.614	0.47982591	1
ENSBTAG00000005115	*SLC31A2*	411	330	368	356	381	0.098	0.917828314	1
ENSBTAG00000005116	*FKBP15*	1'609	1'189	1'383	1'393	1'373	-0.021	0.980562261	1
ENSBTAG00000005119	*PSMD1*	4'875	3'661	4'225	4'222	4'227	0.002	0.997744679	1
ENSBTAG00000005120	*TESPA1*	45	15	28	39	17	-1.170	0.622112607	1
ENSBTAG00000005124	*PHF16*	208	243	230	180	281	0.639	0.532283276	1
ENSBTAG00000005127	*PSMA7*	6'471	5'985	6'257	5'604	6'911	0.302	0.693599578	1
ENSBTAG00000005129	*CEP55*	269	181	221	233	209	-0.157	0.884155438	1
ENSBTAG00000005133	*ANKS6*	397	278	332	344	321	-0.099	0.920619785	1
ENSBTAG00000005136	*ABI1*	1'557	1'183	1'357	1'348	1'366	0.019	0.981835281	1
ENSBTAG00000005137	*LPGAT1*	324	354	345	281	409	0.543	0.564453368	1
ENSBTAG00000005138	*BCL9L*	2'934	6'571	5'064	2'541	7'588	1.578	0.04509858	1
ENSBTAG00000005140	*NBL1*	2'205	1'627	1'894	1'910	1'879	-0.024	0.977783189	1
ENSBTAG00000005141	*PELI2*	269	620	474	233	716	1.620	0.075481477	1
ENSBTAG00000005142	*RPL37*	25'643	33'959	30'710	22'207	39'212	0.820	0.282855043	1
ENSBTAG00000005143	*protein_coding*	0	1	1	0	1	Inf	0.993540919	1
ENSBTAG00000005144	*BT.45215*	1'040	945	996	901	1'091	0.277	0.737128358	1
ENSBTAG00000005145	*BT.65579*	211	420	334	183	485	1.408	0.141883959	1
ENSBTAG00000005146	*BOLA*	144	32	81	125	37	-1.755	0.224842926	1
ENSBTAG00000005147	*HPS5*	853	618	726	739	714	-0.050	0.955553254	1
ENSBTAG00000005148	*PTX4*	26	4	14	23	5	-2.285	0.549753943	1
ENSBTAG00000005149	*AQP10*	3	1	2	3	1	-1.170	0.985253077	1
ENSBTAG00000005151	*WDPCP*	47	49	49	41	57	0.475	0.793101758	1
ENSBTAG00000005152	*TK2*	612	814	735	530	940	0.827	0.331204777	1
ENSBTAG00000005154	*APCDD1*	7'328	12'507	10'394	6'346	14'442	1.186	0.125054254	1
ENSBTAG00000005155	*BT.90528*	1'084	556	790	939	642	-0.548	0.516260501	1
ENSBTAG00000005156	*BT.32280*	143	78	107	124	90	-0.459	0.723616932	1
ENSBTAG00000005158	*NAPG*	2'856	1'902	2'335	2'473	2'196	-0.171	0.828563047	1
ENSBTAG00000005160	*GTF2H1*	2'099	1'187	1'594	1'818	1'371	-0.407	0.61178717	1
ENSBTAG00000005161	*ATP8B2*	5'529	7'134	6'513	4'788	8'238	0.783	0.310362781	1
ENSBTAG00000005162	*STX19*	252	147	194	218	170	-0.363	0.737359419	1
ENSBTAG00000005163	*BT.35032*	206	162	183	178	187	0.068	0.954224276	1
ENSBTAG00000005164	*BT.101478*	3	1	2	3	1	-1.170	0.985253077	1
ENSBTAG00000005165	*CSGALNACT2*	1'221	1'276	1'265	1'057	1'473	0.479	0.555149535	1
ENSBTAG00000005166	*FOP*	9'711	6'524	7'972	8'410	7'533	-0.159	0.836552056	1
ENSBTAG00000005168	*protein_coding*	14	3	8	12	3	-1.807	0.758305361	1
ENSBTAG00000005169	*RGS7BP*	115	94	104	100	109	0.124	0.929789724	1
ENSBTAG00000005170	*GPR114*	16	10	13	14	12	-0.263	0.967957861	1
ENSBTAG00000005174	*GDPGP1*	239	264	256	207	305	0.559	0.576402039	1
ENSBTAG00000005176	*BT.58072*	102	49	72	88	57	-0.643	0.668636317	1
ENSBTAG00000005178	*MMAB*	316	258	286	274	298	0.122	0.902619189	1
ENSBTAG00000005180	*RBMS1*	2'486	1'648	2'028	2'153	1'903	-0.178	0.823022086	1
ENSBTAG00000005181	*SYS1*	892	977	950	772	1'128	0.546	0.50962199	1
ENSBTAG00000005183	*BT.26959*	1'561	1'841	1'739	1'352	2'126	0.653	0.413342712	1
ENSBTAG00000005184	*TP53TG5*	43	10	24	37	12	-1.689	0.512943326	1
ENSBTAG00000005186	*DBNDD2*	1'179	1'286	1'253	1'021	1'485	0.540	0.505756602	1
ENSBTAG00000005189	*C11H9ORF9*	52	153	111	45	177	1.972	0.126757714	1
ENSBTAG00000005190	*TSC1*	860	1'322	1'136	745	1'527	1.035	0.208337134	1
ENSBTAG00000005191	*RPN1*	11'270	9'434	10'327	9'760	10'893	0.158	0.835131608	1
ENSBTAG00000005193	*CRTAM*	5	0	2	4	0		0.853268594	1

Ensembl gene ID	geneName	counts wildtype horn bud	counts polled horn bud	baseMean	baseMean wildtype horn bud	baseMean polled horn bud	log2FoldChange	pval	padj
ENSBTAG00000005195	*ZNF200*	453	371	410	392	428	0.127	0.8911261	1
ENSBTAG00000005196	*TYW3*	414	254	326	359	293	-0.290	0.763233842	1
ENSBTAG00000005197	*BAZ1B*	6'179	5'042	5'587	5'351	5'822	0.122	0.873940558	1
ENSBTAG00000005198	*FGF1*	44	14	27	38	16	-1.237	0.609587027	1
ENSBTAG00000005202	*C13H20orf79*	22	8	14	19	9	-1.044	0.780532613	1
ENSBTAG00000005203	*C3ORF33*	387	306	344	335	353	0.076	0.937830541	1
ENSBTAG00000005205	*BT.48522*	3'555	1'536	2'426	3'079	1'774	-0.796	0.313943583	1
ENSBTAG00000005206	*TMEM14A*	2'006	990	1'440	1'737	1'143	-0.604	0.454606754	1
ENSBTAG00000005207	*COPS7B*	1'775	1'596	1'690	1'537	1'843	0.262	0.742669604	1
ENSBTAG00000005208	*CLEC16A*	324	524	443	281	605	1.109	0.222869458	1
ENSBTAG00000005209	*VPS8*	1'842	1'726	1'794	1'595	1'993	0.321	0.686151335	1
ENSBTAG00000005210	*SNAPC5*	445	265	346	385	306	-0.333	0.725853726	1
ENSBTAG00000005211	*RPL4*	62'149	68'602	66'519	53'823	79'215	0.558	0.463044856	1
ENSBTAG00000005212	*C1H3orf70*	345	437	402	299	505	0.756	0.410486088	1
ENSBTAG00000005213	*ZWILCH*	1'037	638	817	898	737	-0.286	0.734851646	1
ENSBTAG00000005214	*BT.27048*	59	59	60	51	68	0.415	0.801576506	1
ENSBTAG00000005215	*IL10RA*	111	72	90	96	83	-0.209	0.885807172	1
ENSBTAG00000005217	*protein_coding*	20	75	52	17	87	2.322	0.187177021	1
ENSBTAG00000005218	*PDE3B*	358	106	216	310	122	-1.341	0.202116614	1
ENSBTAG00000005219	*KLHL4*	61	26	41	53	30	-0.815	0.674328314	1
ENSBTAG00000005221	*WNK1*	6'102	5'427	5'776	5'284	6'267	0.246	0.748825729	1
ENSBTAG00000005222	*C9ORF128*	5	5	5	4	6	0.415	0.985023983	1
ENSBTAG00000005225	*RAD52*	1'380	1'137	1'254	1'195	1'313	0.136	0.867360272	1
ENSBTAG00000005227	*ATF6*	419	553	501	363	639	0.815	0.359442997	1
ENSBTAG00000005229	*protein_coding*	1	0	0	1	0		1	1
ENSBTAG00000005230	*SHOX2*	562	402	475	487	464	-0.068	0.942207882	1
ENSBTAG00000005231	*C1orf222*	12	3	7	10	3	-1.585	0.809704871	1
ENSBTAG00000005234	*LYPD1*	97	93	96	84	107	0.354	0.793725295	1
ENSBTAG00000005238	*NCKAP5*	162	379	289	140	438	1.641	0.097782279	1
ENSBTAG00000005239	*UBL4A*	594	845	745	514	976	0.924	0.277635652	1
ENSBTAG00000005240	*BT.105380*	935	252	550	810	291	-1.477	0.097570943	1
ENSBTAG00000005244	*RASL11A*	175	74	119	152	85	-0.827	0.505540021	1
ENSBTAG00000005246	*DIP2B*	3'944	2'234	2'998	3'416	2'580	-0.405	0.604836283	1
ENSBTAG00000005247	*FRMD3*	83	32	54	72	37	-0.960	0.570423098	1
ENSBTAG00000005249	*RNF14*	2'192	1'203	1'644	1'898	1'389	-0.451	0.573911596	1
ENSBTAG00000005251	*protein_coding*	28	10	18	24	12	-1.070	0.735005286	1
ENSBTAG00000005252	*NUDT11*	183	172	179	158	199	0.326	0.767381523	1
ENSBTAG00000005257	*PEX13*	1'200	961	1'074	1'039	1'110	0.095	0.908487984	1
ENSBTAG00000005259	*UCP3*	0	1	1	0	1	Inf	0.993540919	1
ENSBTAG00000005261	*FCGBP*	15	26	22	13	30	1.209	0.660290471	1
ENSBTAG00000005263	*KLHL2*	2'989	1'169	1'969	2'589	1'350	-0.939	0.239186936	1
ENSBTAG00000005265	*BT.61596*	930	715	816	805	826	0.036	0.966895411	1
ENSBTAG00000005268	*PLA2G4D*	22	4	12	19	5	-2.044	0.62595401	1
ENSBTAG00000005269	*CCNB2*	565	697	647	489	805	0.718	0.40437718	1
ENSBTAG00000005272	*DOHH*	380	556	486	329	642	0.964	0.281560431	1
ENSBTAG00000005273	*IL1R1*	1'068	1'009	1'045	925	1'165	0.333	0.685190472	1
ENSBTAG00000005275	*PKIG*	651	479	558	564	553	-0.028	0.977863339	1
ENSBTAG00000005278	*WEE1*	2'626	1'355	1'919	2'274	1'565	-0.540	0.497691511	1
ENSBTAG00000005280	*ADA*	848	329	557	734	380	-0.951	0.280240461	1
ENSBTAG00000005284	*SERPINE3*	91	29	56	79	33	-1.235	0.45898586	1
ENSBTAG00000005285	*MRPS33*	2'978	2'148	2'530	2'579	2'480	-0.056	0.94397625	1
ENSBTAG00000005286	*BT.64617*	351	755	588	304	872	1.520	0.085482053	1
ENSBTAG00000005288	*GFRA3*	449	243	335	389	281	-0.471	0.620756309	1
ENSBTAG00000005289	*CHERP*	2'227	2'488	2'401	1'929	2'873	0.575	0.465129994	1
ENSBTAG00000005290	*CLDN2*	0	1	1	0	1	Inf	0.993540919	1
ENSBTAG00000005293	*CDC25C*	107	84	95	93	97	0.066	0.968374134	1
ENSBTAG00000005294	*BT.105913*	1'395	1'226	1'312	1'208	1'416	0.229	0.777288964	1
ENSBTAG00000005295	*SLC35E1*	1'013	969	998	877	1'119	0.351	0.670350885	1
ENSBTAG00000005296	*RPL13A*	74'397	75'405	75'750	64'430	87'070	0.434	0.566811129	1
ENSBTAG00000005299	*EDNRB*	976	558	745	845	644	-0.392	0.645024126	1
ENSBTAG00000005300	*TMEM51*	591	530	562	512	612	0.258	0.768399231	1
ENSBTAG00000005301	*KLHL32*	128	52	85	111	60	-0.885	0.526757888	1
ENSBTAG00000005304	*SRM*	1'245	1'126	1'189	1'078	1'300	0.270	0.739992127	1
ENSBTAG00000005306	*TRH*	50	17	31	43	20	-1.141	0.608722633	1
ENSBTAG00000005308	*ANGPTL5*	4'617	1'809	3'044	3'998	2'089	-0.937	0.233371767	1
ENSBTAG00000005309	*TGDS*	1'350	788	1'040	1'169	910	-0.362	0.661208046	1
ENSBTAG00000005310	*GPR180*	542	348	436	469	402	-0.224	0.806896601	1
ENSBTAG00000005311	*BT.55567*	324	310	319	281	358	0.351	0.713676889	1
ENSBTAG00000005312	*MED26*	792	865	842	686	999	0.542	0.517163622	1
ENSBTAG00000005313	*EPHB3*	3'467	7'459	5'808	3'003	8'613	1.520	0.05269955	1
ENSBTAG00000005314	*MFN2*	1'918	2'243	2'126	1'661	2'590	0.641	0.418002226	1
ENSBTAG00000005315	*BT.91603*	50'684	49'084	50'285	43'894	56'677	0.369	0.626810219	1

Ensembl gene ID	geneName	counts wildtype horn bud	counts polled horn bud	baseMean	baseMean wildtype horn bud	baseMean polled horn bud	log2FoldChange	pval	padj
ENSBTAG00000005316	*GDI2*	15'529	11'669	13'461	13'449	13'474	0.003	0.996553609	1
ENSBTAG00000005318	*RNF6*	551	380	458	477	439	-0.121	0.895801886	1
ENSBTAG00000005324	*protein_coding*	1	1	1	1	1	0.415	1	1
ENSBTAG00000005326	*CSNK1A1*	1'941	1'939	1'960	1'681	2'239	0.414	0.601638166	1
ENSBTAG00000005327	*SUSD5*	55	17	34	48	20	-1.279	0.551614403	1
ENSBTAG00000005328	*BT.43104*	936	513	701	811	592	-0.453	0.596596389	1
ENSBTAG00000005330	*KRTDAP*	13'959	6'587	9'847	12'089	7'606	-0.668	0.384051055	1
ENSBTAG00000005333	*MB*	128	221	183	111	255	1.203	0.269778115	1
ENSBTAG00000005337	*RAB3B*	95	30	58	82	35	-1.248	0.446094408	1
ENSBTAG00000005338	*EIF3M*	484	1'047	814	419	1'209	1.528	0.073676874	1
ENSBTAG00000005339	*VEGFA*	2'234	944	1'512	1'935	1'090	-0.828	0.305037359	1
ENSBTAG00000005340	*SERGEF*	1'109	1'439	1'311	960	1'662	0.791	0.330113817	1
ENSBTAG00000005343	*TPH1*	9	7	8	8	8	0.052	1	1
ENSBTAG00000005344	*GNPNAT1*	1'009	625	798	874	722	-0.276	0.744265405	1
ENSBTAG00000005345	*ARPC3*	5'545	4'340	4'907	4'802	5'011	0.062	0.935993203	1
ENSBTAG00000005347	*GPN3*	545	266	390	472	307	-0.620	0.50295348	1
ENSBTAG00000005349	*BT.23343*	14'690	22'208	19'183	12'722	25'644	1.011	0.187724292	1
ENSBTAG00000005351	*RFXAP*	870	483	656	753	558	-0.434	0.614429184	1
ENSBTAG00000005352	*CRYBA1*	17	10	13	15	12	-0.350	0.946275136	1
ENSBTAG00000005354	*LRRFIP1*	2'549	1'437	1'933	2'207	1'659	-0.412	0.604514927	1
ENSBTAG00000005355	*BAG2*	1'112	630	845	963	727	-0.405	0.629754881	1
ENSBTAG00000005356	*ST5*	3'084	4'795	4'104	2'671	5'537	1.052	0.178209374	1
ENSBTAG00000005357	*VPS11*	2'349	2'767	2'615	2'034	3'195	0.651	0.407030537	1
ENSBTAG00000005359	*TGFB2*	2'354	3'538	3'062	2'039	4'085	1.003	0.202032809	1
ENSBTAG00000005362	*RAB23*	355	408	389	307	471	0.616	0.50441455	1
ENSBTAG00000005363	*GGN*	25	16	20	22	18	-0.229	0.954816059	1
ENSBTAG00000005364	*HMBS*	889	876	891	770	1'012	0.394	0.636178667	1
ENSBTAG00000005365	*OVCH1*	18	7	12	16	8	-0.948	0.827785041	1
ENSBTAG00000005367	*VRK3*	554	604	589	480	697	0.540	0.534727609	1
ENSBTAG00000005370	*TMTC1*	145	70	103	126	81	-0.636	0.626613477	1
ENSBTAG00000005371	*DPAGT1*	1'125	1'024	1'078	974	1'182	0.279	0.733261996	1
ENSBTAG00000005372	*DLGAP1*	232	131	176	201	151	-0.410	0.711633453	1
ENSBTAG00000005373	*TPM1*	10'747	6'830	8'597	9'307	7'887	-0.239	0.755721907	1
ENSBTAG00000005376	*FBXO30*	332	304	319	288	351	0.288	0.764077913	1
ENSBTAG00000005377	*SOSTDC1*	2'637	3'043	2'899	2'284	3'514	0.622	0.427096081	1
ENSBTAG00000005378	*XKRX*	49	28	37	42	32	-0.392	0.855951411	1
ENSBTAG00000005379	*SLC41A1*	481	570	537	417	658	0.660	0.453288854	1
ENSBTAG00000005380	*MOCS2*	2'126	1'967	2'056	1'841	2'271	0.303	0.701411985	1
ENSBTAG00000005382	*CCDC85B*	447	386	416	387	446	0.203	0.824620559	1
ENSBTAG00000005384	*COPZ1*	8'157	8'641	8'521	7'064	9'978	0.498	0.515574433	1
ENSBTAG00000005385	*POP5*	692	425	545	599	491	-0.288	0.74430864	1
ENSBTAG00000005390	*GMFG*	365	201	274	316	232	-0.446	0.652889594	1
ENSBTAG00000005392	*KBTBD3*	219	98	151	190	113	-0.745	0.51678079	1
ENSBTAG00000005393	*TIPRL*	1'025	690	842	888	797	-0.156	0.85396878	1
ENSBTAG00000005394	*COX18*	269	145	200	233	167	-0.477	0.655174862	1
ENSBTAG00000005395	*BT.86924*	344	104	209	298	120	-1.311	0.215915115	1
ENSBTAG00000005396	*XKR6*	4	1	2	3	1	-1.585	0.952247636	1
ENSBTAG00000005397	*BT.68611*	11'288	6'049	8'380	9'776	6'985	-0.485	0.52776834	1
ENSBTAG00000005400	*ATG5*	401	364	384	347	420	0.275	0.766537943	1
ENSBTAG00000005403	*ADI1*	1'962	1'900	1'947	1'699	2'194	0.369	0.641606602	1
ENSBTAG00000005404	*MSC*	17	36	28	15	42	1.497	0.523052277	1
ENSBTAG00000005408	*CLK1*	30'184	8'225	17'819	26'140	9'497	-1.461	0.059672418	1
ENSBTAG00000005412	*NEDD4L*	1'825	1'283	1'531	1'580	1'481	-0.093	0.908767593	1
ENSBTAG00000005413	*NLRC5*	125	94	108	108	109	0.004	1	1
ENSBTAG00000005416	*PPIL3*	686	359	504	594	415	-0.519	0.559572043	1
ENSBTAG00000005419	*ABP1*	5	6	6	4	7	0.678	0.946418607	1
ENSBTAG00000005424	*KCNC1*	85	10	43	74	12	-2.672	0.176932861	1
ENSBTAG00000005426	*PSMC1*	3'909	4'506	4'294	3'385	5'203	0.620	0.423764324	1
ENSBTAG00000005427	*C14ORF102*	642	1'207	975	556	1'394	1.326	0.113300517	1
ENSBTAG00000005429	*BT.75316*	7	2	4	6	2	-1.392	0.903936064	1
ENSBTAG00000005430	*MB21D2*	302	192	242	262	222	-0.238	0.817386068	1
ENSBTAG00000005431	*LMCD1*	1'168	583	842	1'012	673	-0.587	0.48411271	1
ENSBTAG00000005432	*CAPG*	1'417	1'792	1'648	1'227	2'069	0.754	0.346954702	1
ENSBTAG00000005433	*C3ORF32*	65	34	48	56	39	-0.520	0.777662951	1
ENSBTAG00000005434	*PLVAP*	4'416	2'497	3'354	3'824	2'883	-0.408	0.601466453	1
ENSBTAG00000005436	*SHQ1*	611	542	577	529	626	0.242	0.781469398	1
ENSBTAG00000005437	*SMCP*	1	1	1	1	1	0.415	1	1
ENSBTAG00000005439	*FAM102B*	297	213	252	257	246	-0.065	0.953640926	1
ENSBTAG00000005441	*ZMAT2*	2'909	2'663	2'797	2'519	3'075	0.288	0.712758448	1
ENSBTAG00000005442	*WDR78*	116	100	108	100	115	0.201	0.879896757	1
ENSBTAG00000005443	*MIER1*	5'016	2'741	3'755	4'344	3'165	-0.457	0.557165054	1
ENSBTAG00000005444	*UGGT1*	537	539	544	465	622	0.420	0.632051035	1

Ensembl gene ID	geneName	counts wildtype horn bud	counts polled horn bud	baseMean	baseMean wildtype horn bud	baseMean polled horn bud	log2FoldChange	pval	padj
ENSBTAG00000005445	*SLC35D2*	129	143	138	112	165	0.564	0.632153232	1
ENSBTAG00000005446	*POLR2J*	704	684	700	610	790	0.373	0.661209728	1
ENSBTAG00000005448	*RASA4*	861	287	539	746	331	-1.170	0.187525556	1
ENSBTAG00000005450	*CYP3A5*	2	0	1	2	0		0.974934741	1
ENSBTAG00000005453	*FGD4*	79	31	52	68	36	-0.935	0.588407576	1
ENSBTAG00000005454	*BT.23151*	1'164	834	986	1'008	963	-0.066	0.938285031	1
ENSBTAG00000005455	*USP7*	7'923	5'829	6'796	6'862	6'731	-0.028	0.972039745	1
ENSBTAG00000005456	*TTK*	502	303	392	435	350	-0.313	0.735758079	1
ENSBTAG00000005458	*BT.97014*	1'303	1'969	1'701	1'128	2'274	1.011	0.208455022	1
ENSBTAG00000005461	*SOX15*	279	249	265	242	288	0.251	0.801959854	1
ENSBTAG00000005462	*FXR2*	3'115	2'954	3'054	2'698	3'411	0.338	0.664068413	1
ENSBTAG00000005464	*BT.61413*	5'273	7'273	6'482	4'567	8'398	0.879	0.255381428	1
ENSBTAG00000005465	*NDUFA9*	1'879	1'972	1'952	1'627	2'277	0.485	0.540909772	1
ENSBTAG00000005466	*C8H8ORF58*	118	160	143	102	185	0.854	0.462509297	1
ENSBTAG00000005469	*KIAA1967*	8'967	11'202	10'350	7'766	12'935	0.736	0.337315241	1
ENSBTAG00000005470	*BIN3*	972	810	889	842	935	0.152	0.855495327	1
ENSBTAG00000005471	*RHCG*	15	2	8	13	2	-2.492	0.678711229	1
ENSBTAG00000005474	*CREB1*	399	345	372	346	398	0.205	0.826424769	1
ENSBTAG00000005475	*BT.40997*	276	299	292	239	345	0.531	0.58533848	1
ENSBTAG00000005476	*METTL21A*	452	341	393	391	394	0.008	0.995141584	1
ENSBTAG00000005477	*LAPTM5*	2'820	1'463	2'066	2'442	1'689	-0.532	0.50258637	1
ENSBTAG00000005478	*PATZ1*	2'170	3'038	2'694	1'879	3'508	0.900	0.252939704	1
ENSBTAG00000005479	*LIPC*	26	0	11	23	0		0.259291126	1
ENSBTAG00000005481	*ADAM10*	2'489	2'422	2'476	2'156	2'797	0.376	0.63215657	1
ENSBTAG00000005482	*BT.40036*	319	130	213	276	150	-0.880	0.400902656	1
ENSBTAG00000005483	*BT.36307*	4'284	4'758	4'602	3'710	5'494	0.566	0.464066847	1
ENSBTAG00000005488	*ARIH2*	1'549	1'590	1'589	1'341	1'836	0.453	0.571624781	1
ENSBTAG00000005489	*WDR36*	1'340	1'011	1'164	1'160	1'167	0.009	0.992119201	1
ENSBTAG00000005490	*P4HTM*	739	786	774	640	908	0.504	0.550243726	1
ENSBTAG00000005492	*CCNY*	3'286	3'225	3'285	2'846	3'724	0.388	0.618029736	1
ENSBTAG00000005493	*TBC1D14*	3'704	3'344	3'535	3'208	3'861	0.268	0.730224468	1
ENSBTAG00000005495	*protein_coding*	2'660	1'573	2'060	2'304	1'816	-0.343	0.665547779	1
ENSBTAG00000005496	*RP2*	533	599	577	462	692	0.583	0.503303523	1
ENSBTAG00000005497	*RPRD1A*	1'893	973	1'381	1'639	1'124	-0.545	0.500512984	1
ENSBTAG00000005498	*SQLE*	3'200	2'364	2'750	2'771	2'730	-0.022	0.978975513	1
ENSBTAG00000005499	*BT.54157*	15	1	7	13	1	-3.492	0.607205542	1
ENSBTAG00000005501	*COBLL1*	170	73	116	147	84	-0.805	0.520381307	1
ENSBTAG00000005503	*PRMT2*	1'700	1'876	1'819	1'472	2'166	0.557	0.48385246	1
ENSBTAG00000005504	*TP53INP2*	31	17	23	27	20	-0.452	0.876164144	1
ENSBTAG00000005507	*pseudogene*	9	3	6	8	3	-1.170	0.890813994	1
ENSBTAG00000005514	*BT.45586*	5'798	4'150	4'907	5'021	4'792	-0.067	0.931310242	1
ENSBTAG00000005517	*NOP58*	4'808	2'320	3'421	4'164	2'679	-0.636	0.41508377	1
ENSBTAG00000005519	*RNF38*	621	210	390	538	242	-1.149	0.216626801	1
ENSBTAG00000005522	*PLSCR5*	2	0	1	2	0		0.974934741	1
ENSBTAG00000005523	*XYLT1*	503	525	521	436	606	0.477	0.589168818	1
ENSBTAG00000005524	*HBP1*	6'665	5'897	6'291	5'772	6'809	0.238	0.755908918	1
ENSBTAG00000005525	*LHX6*	278	477	396	241	551	1.194	0.197726962	1
ENSBTAG00000005526	*COG5*	2'063	1'534	1'779	1'787	1'771	-0.012	0.98906877	1
ENSBTAG00000005527	*GIPR*	24	5	13	21	6	-1.848	0.630822808	1
ENSBTAG00000005530	*BT.35560*	471	591	545	408	682	0.742	0.398330047	1
ENSBTAG00000005532	*BT.44757*	1'826	2'405	2'179	1'581	2'777	0.812	0.305235512	1
ENSBTAG00000005533	*BT.64650*	3'026	4'621	3'978	2'621	5'336	1.026	0.189227059	1
ENSBTAG00000005534	*ENO3*	729	1'487	1'174	631	1'717	1.443	0.081416281	1
ENSBTAG00000005536	*MEA1*	865	1'451	1'212	749	1'675	1.161	0.157378713	1
ENSBTAG00000005540	*CBX3*	169	56	106	146	65	-1.178	0.362698719	1
ENSBTAG00000005542	*EPS15*	4'961	3'260	4'030	4'296	3'764	-0.191	0.806382581	1
ENSBTAG00000005547	*MGC159550*	551	396	467	477	457	-0.062	0.94849957	1
ENSBTAG00000005548	*HYAL4*	10	0	4	9	0		0.653360153	1
ENSBTAG00000005550	*SOS2*	1'446	658	1'006	1'252	760	-0.721	0.383851093	1
ENSBTAG00000005556	*CYGB*	834	222	489	722	256	-1.494	0.098896991	1
ENSBTAG00000005557	*MLLT4*	3'199	2'564	2'866	2'770	2'961	0.096	0.902123276	1
ENSBTAG00000005559	*pseudogene*	0	3	2	0	3	Inf	0.878646179	1
ENSBTAG00000005560	*ST18*	32	1	14	28	1	-4.585	0.277113151	1
ENSBTAG00000005564	*PIAS1*	805	556	670	697	642	-0.119	0.891826039	1
ENSBTAG00000005565	*BT.60745*	1'262	1'233	1'258	1'093	1'424	0.381	0.638043059	1
ENSBTAG00000005567	*BEND4*	9	1	4	8	1	-2.755	0.785977687	1
ENSBTAG00000005568	*FRMPD2*	1	0	0	1	0		1	1
ENSBTAG00000005571	*PHF20*	1'676	1'170	1'401	1'451	1'351	-0.103	0.899306102	1
ENSBTAG00000005572	*ZNF205*	1'332	1'418	1'395	1'154	1'637	0.505	0.530932857	1
ENSBTAG00000005573	*SCAND1*	1'960	2'143	2'086	1'697	2'475	0.544	0.491784297	1
ENSBTAG00000005574	*CLU*	25'951	20'536	23'094	22'474	23'713	0.077	0.91829838	1
ENSBTAG00000005576	*CLEC11A*	3'495	4'542	4'136	3'027	5'245	0.793	0.307805681	1

Ensembl gene ID	geneName	counts wildtype horn bud	counts polled horn bud	baseMean	baseMean wildtype horn bud	baseMean polled horn bud	log2FoldChange	pval	padj
ENSBTAG00000005577	*RNASEH2C*	1'248	1'431	1'367	1'081	1'652	0.612	0.44860547	1
ENSBTAG00000005578	*KAT5*	1'632	1'866	1'784	1'413	2'155	0.608	0.445266099	1
ENSBTAG00000005580	*C1ORF84*	1'735	3'735	2'908	1'503	4'313	1.521	0.056117605	1
ENSBTAG00000005581	*CCL25*	196	144	168	170	166	-0.030	0.985579061	1
ENSBTAG00000005583	*HYI*	684	490	579	592	566	-0.066	0.942172241	1
ENSBTAG00000005584	*processed_pseudogene*	1'381	1'765	1'617	1'196	2'038	0.769	0.337866858	1
ENSBTAG00000005585	*ANXA13*	76	59	67	66	68	0.050	0.984253269	1
ENSBTAG00000005586	*GATM*	2'605	2'328	2'472	2'256	2'688	0.253	0.7471172	1
ENSBTAG00000005587	*SKIV2L*	2'284	3'536	3'031	1'978	4'083	1.046	0.183946011	1
ENSBTAG00000005588	*BT.28712*	618	672	656	535	776	0.536	0.532486739	1
ENSBTAG00000005589	*STK19*	3'168	2'240	2'665	2'744	2'587	-0.085	0.914663712	1
ENSBTAG00000005592	*BT.26509*	1'644	1'176	1'391	1'424	1'358	-0.068	0.934078457	1
ENSBTAG00000005595	*TRMU*	1'365	1'233	1'303	1'182	1'424	0.268	0.740132971	1
ENSBTAG00000005596	*IGFBP2*	838	929	899	726	1'073	0.564	0.498269188	1
ENSBTAG00000005600	*protein_coding*	0	1	1	0	1	Inf	0.993540919	1
ENSBTAG00000005603	*CXCL11*	16	8	12	14	9	-0.585	0.904936474	1
ENSBTAG00000005604	*POPDC2*	1	5	3	1	6	2.737	0.836148221	1
ENSBTAG00000005607	*ERCC6L*	294	255	275	255	294	0.210	0.833144936	1
ENSBTAG00000005609	*protein_coding*	1	0	0	1	0		1	1
ENSBTAG00000005614	*UXS1*	2'786	2'180	2'465	2'413	2'517	0.061	0.937731165	1
ENSBTAG00000005615	*BT.88249*	823	271	513	713	313	-1.188	0.183915835	1
ENSBTAG00000005616	*BRS3*	2	0	1	2	0		0.974934741	1
ENSBTAG00000005617	*SLC41A3*	919	1'142	1'057	796	1'319	0.728	0.376208427	1
ENSBTAG00000005620	*RPS3*	69'655	97'738	86'591	60'323	112'858	0.904	0.236495959	1
ENSBTAG00000005622	*LITAF*	3'667	1'956	2'717	3'176	2'259	-0.492	0.531184074	1
ENSBTAG00000005623	*BT.45169*	9	5	7	8	6	-0.433	0.969858938	1
ENSBTAG00000005626	*NEUROD4*	3	0	1	3	0		0.936647693	1
ENSBTAG00000005627	*PAFAH1B2*	3'038	3'110	3'111	2'631	3'591	0.449	0.564859096	1
ENSBTAG00000005628	*CD52*	222	94	150	192	109	-0.825	0.473751599	1
ENSBTAG00000005629	*AIM1L*	697	1'158	970	604	1'337	1.147	0.169043756	1
ENSBTAG00000005630	*BT.41977*	83	112	101	72	129	0.847	0.516922503	1
ENSBTAG00000005631	*VARS*	3'159	5'045	4'281	2'736	5'825	1.090	0.162735465	1
ENSBTAG00000005633	*RGNEF*	912	551	713	790	636	-0.312	0.715230773	1
ENSBTAG00000005634	*LSM2*	1'988	1'833	1'919	1'722	2'117	0.298	0.706906359	1
ENSBTAG00000005635	*TTC39C*	421	317	365	365	366	0.006	0.997834587	1
ENSBTAG00000005638	*CABYR*	148	63	100	128	73	-0.817	0.534513226	1
ENSBTAG00000005639	*processed_pseudogene*	392	561	494	339	648	0.932	0.296470542	1
ENSBTAG00000005644	*WBSCR17*	49	33	40	42	38	-0.155	0.950170709	1
ENSBTAG00000005647	*GPR132*	157	23	81	136	27	-2.356	0.109683853	1
ENSBTAG00000005650	*SKAP2*	1'420	1'161	1'285	1'230	1'341	0.125	0.877941765	1
ENSBTAG00000005652	*ALG3*	778	818	809	674	945	0.487	0.561770424	1
ENSBTAG00000005653	*BT.87340*	127	51	84	110	59	-0.901	0.520880239	1
ENSBTAG00000005654	*TMSB10*	51'610	39'301	45'038	44'696	45'381	0.022	0.976276437	1
ENSBTAG00000005657	*CAMK2N2*	309	82	181	268	95	-1.499	0.174316586	1
ENSBTAG00000005658	*BT.70166*	1'472	1'555	1'535	1'275	1'796	0.494	0.537812045	1
ENSBTAG00000005660	*PSMD2*	8'819	10'553	9'912	7'637	12'186	0.674	0.37941045	1
ENSBTAG00000005661	*SNAP23*	2'490	1'189	1'765	2'156	1'373	-0.651	0.415099852	1
ENSBTAG00000005663	*CEP27*	3'141	2'215	2'639	2'720	2'558	-0.089	0.910799488	1
ENSBTAG00000005664	*YWHAE*	22'352	16'219	19'043	19'357	18'728	-0.048	0.950787192	1
ENSBTAG00000005665	*CRK*	9'640	4'622	6'843	8'348	5'337	-0.645	0.402379741	1
ENSBTAG00000005666	*LRRC20*	185	171	179	160	197	0.302	0.784401974	1
ENSBTAG00000005667	*FAM75D1*	9	0	4	8	0		0.690676647	1
ENSBTAG00000005668	*SLC39A8*	340	309	326	294	357	0.277	0.771931414	1
ENSBTAG00000005670	*ARHGEF19*	2'299	3'331	2'919	1'991	3'846	0.950	0.227039624	1
ENSBTAG00000005671	*UBQLNL*	1	2	2	1	2	1.415	0.981979269	1
ENSBTAG00000005672	*CDH22*	60	39	48	52	45	-0.206	0.918903796	1
ENSBTAG00000005674	*NEU1*	801	680	739	694	785	0.179	0.833313293	1
ENSBTAG00000005675	*SLC44A4*	5	4	4	4	5	0.093	1	1
ENSBTAG00000005676	*EHMT2*	6'056	8'572	7'571	5'245	9'898	0.916	0.235128568	1
ENSBTAG00000005678	*AACS*	2'352	1'852	2'088	2'037	2'139	0.070	0.929071296	1
ENSBTAG00000005679	*BT.62785*	629	145	356	545	167	-1.702	0.075318578	1
ENSBTAG00000005682	*UBAC2*	1'435	1'364	1'409	1'243	1'575	0.342	0.671172608	1
ENSBTAG00000005683	*ELAC1*	1'177	650	885	1'019	751	-0.442	0.597299658	1
ENSBTAG00000005688	*MRPS2*	778	990	908	674	1'143	0.763	0.360156074	1
ENSBTAG00000005691	*BT.66957*	1'220	525	831	1'057	606	-0.801	0.341119811	1
ENSBTAG00000005693	*CCDC43*	1'492	1'395	1'451	1'292	1'611	0.318	0.692305916	1
ENSBTAG00000005694	*BT.65037*	5'016	3'090	3'956	4'344	3'568	-0.284	0.714970307	1
ENSBTAG00000005695	*NUDT6*	90	64	76	78	74	-0.077	0.968683753	1
ENSBTAG00000005696	*DBF4B*	145	111	127	126	128	0.030	0.987132036	1
ENSBTAG00000005697	*MDGA2*	19	10	14	16	12	-0.511	0.904299589	1
ENSBTAG00000005699	*SCML4*	4	2	3	3	2	-0.585	0.996239345	1
ENSBTAG00000005700	*MAP3K9*	58	50	54	50	58	0.201	0.914002993	1

Ensembl gene ID	geneName	counts wildtype horn bud	counts polled horn bud	baseMean	baseMean wildtype horn bud	baseMean polled horn bud	log2FoldChange	pval	padj
ENSBTAG00000005704	*IL1RL2*	162	68	109	140	79	-0.837	0.511697103	1
ENSBTAG00000005708	*BT.95439*	528	245	370	457	283	-0.693	0.458023288	1
ENSBTAG00000005709	*ABHD3*	370	109	223	320	126	-1.348	0.196367681	1
ENSBTAG00000005710	*BT.40343*	3'176	2'734	2'954	2'750	3'157	0.199	0.798595947	1
ENSBTAG00000005711	*NSG1*	495	196	328	429	226	-0.922	0.334935181	1
ENSBTAG00000005712	*BT.61487*	1'432	1'232	1'331	1'240	1'423	0.198	0.806412946	1
ENSBTAG00000005715	*C26H10ORF125*	1'124	1'097	1'120	973	1'267	0.380	0.642050115	1
ENSBTAG00000005716	*CRABP2*	6'461	12'343	9'924	5'595	14'252	1.349	0.082371001	1
ENSBTAG00000005717	*GJB5*	126	231	188	109	267	1.290	0.234348459	1
ENSBTAG00000005718	*PLIN2*	3'087	2'697	2'894	2'673	3'114	0.220	0.777688041	1
ENSBTAG00000005719	*GJB4*	33	65	52	29	75	1.393	0.41789017	1
ENSBTAG00000005722	*processed_pseudogene*	237	464	371	205	536	1.384	0.141239264	1
ENSBTAG00000005723	*C3ORF17*	704	416	545	610	480	-0.344	0.69668398	1
ENSBTAG00000005724	*SYTL4*	390	388	393	338	448	0.408	0.658193153	1
ENSBTAG00000005725	*IDO2*	66	51	58	57	59	0.043	0.990205177	1
ENSBTAG00000005726	*HNRNPA2B1*	32'278	12'532	21'212	27'954	14'471	-0.950	0.215565327	1
ENSBTAG00000005728	*CDHR4*	27	21	24	23	24	0.052	1	1
ENSBTAG00000005729	*FBXL4*	624	406	505	540	469	-0.205	0.819363262	1
ENSBTAG00000005730	*AGPAT6*	2'719	4'012	3'494	2'355	4'633	0.976	0.212448738	1
ENSBTAG00000005732	*TRAPPC3*	895	708	796	775	818	0.077	0.927889354	1
ENSBTAG00000005733	*CHD6*	1'106	1'240	1'195	958	1'432	0.580	0.476571648	1
ENSBTAG00000005734	*GATA6*	44	40	42	38	46	0.278	0.892766234	1
ENSBTAG00000005735	*ATP5G2*	6'455	8'328	7'603	5'590	9'616	0.783	0.3095123	1
ENSBTAG00000005736	*GPR113*	54	34	43	47	39	-0.252	0.90478782	1
ENSBTAG00000005738	*ATP8B1*	784	438	592	679	506	-0.425	0.626033136	1
ENSBTAG00000005741	*BT.87151*	158	23	82	137	27	-2.365	0.107715825	1
ENSBTAG00000005742	*CYYR1*	133	62	93	115	72	-0.686	0.612429031	1
ENSBTAG00000005743	*SMN1*	597	547	574	517	632	0.289	0.740666763	1
ENSBTAG00000005744	*BT.87535*	1'158	496	788	1'003	573	-0.808	0.339544352	1
ENSBTAG00000005745	*HPSE*	149	113	130	129	130	0.016	0.996029719	1
ENSBTAG00000005748	*SOX17*	125	69	94	108	80	-0.442	0.7461877	1
ENSBTAG00000005750	*TCF15*	25	17	21	22	20	-0.141	0.979928407	1
ENSBTAG00000005751	*CDAN1*	4'051	3'116	3'553	3'508	3'598	0.036	0.962269502	1
ENSBTAG00000005753	*BT.4192*	3'573	1'692	2'524	3'094	1'954	-0.663	0.399837094	1
ENSBTAG00000005754	*PPM1K*	3'312	2'052	2'619	2'868	2'369	-0.276	0.725990348	1
ENSBTAG00000005756	*ZDHHC24*	114	80	96	99	92	-0.096	0.951671776	1
ENSBTAG00000005757	*FUS*	18'922	16'751	17'865	16'387	19'342	0.239	0.752849125	1
ENSBTAG00000005760	*TBC1D4*	670	380	510	580	439	-0.403	0.650400514	1
ENSBTAG00000005761	*DEDD2*	488	681	604	423	786	0.896	0.303008982	1
ENSBTAG00000005762	*LYNX1*	141	194	173	122	224	0.875	0.426896282	1
ENSBTAG00000005763	*BT.52156*	884	1'075	1'003	766	1'241	0.697	0.398686949	1
ENSBTAG00000005765	*SYNGR1*	419	431	430	363	498	0.456	0.615574344	1
ENSBTAG00000005766	*IL4I1*	60	77	70	52	89	0.775	0.60604604	1
ENSBTAG00000005769	*NPHP3*	410	177	280	355	204	-0.797	0.418408271	1
ENSBTAG00000005772	*SPDYC*	7	7	7	6	8	0.415	0.967915805	1
ENSBTAG00000005773	*PGM2*	271	306	294	235	353	0.590	0.543232605	1
ENSBTAG00000005776	*ZNF185*	250	218	234	217	252	0.217	0.833309639	1
ENSBTAG00000005779	*BT.56226*	0	1	1	0	1	Inf	0.993540919	1
ENSBTAG00000005780	*LDB1*	5'122	5'653	5'482	4'436	6'528	0.557	0.469861667	1
ENSBTAG00000005782	*BT.99183*	18	12	15	16	14	-0.170	0.983826208	1
ENSBTAG00000005784	*CSMD2*	124	32	72	107	37	-1.539	0.306246072	1
ENSBTAG00000005785	*DPY19L1*	3'246	1'538	2'294	2'811	1'776	-0.663	0.401998166	1
ENSBTAG00000005786	*ATXN3*	219	97	151	190	112	-0.760	0.508947514	1
ENSBTAG00000005787	*IFT57*	1'667	1'376	1'516	1'444	1'589	0.138	0.863124337	1
ENSBTAG00000005788	*VANGL1*	135	117	126	117	135	0.209	0.867532257	1
ENSBTAG00000005791	*UROS*	388	308	346	336	356	0.082	0.932926706	1
ENSBTAG00000005792	*BCCIP*	1'844	2'078	1'998	1'597	2'399	0.587	0.458746936	1
ENSBTAG00000005793	*PEA15*	13'234	10'567	11'831	11'461	12'202	0.090	0.905191187	1
ENSBTAG00000005795	*DHX32*	1'374	906	1'118	1'190	1'046	-0.186	0.821735541	1
ENSBTAG00000005796	*DCAF8*	2'777	2'764	2'798	2'405	3'192	0.408	0.601542539	1
ENSBTAG00000005797	*AGXT*	10	28	20	9	32	1.900	0.502905125	1
ENSBTAG00000005798	*TRIM50*	2	0	1	2	0		0.974934741	1
ENSBTAG00000005799	*KIRREL*	956	1'680	1'384	828	1'940	1.228	0.132140958	1
ENSBTAG00000005803	*PTPDC1*	358	197	269	310	227	-0.447	0.653492378	1
ENSBTAG00000005805	*BT.63275*	3	3	3	3	3	0.415	1	1
ENSBTAG00000005807	*MICU1*	2'076	2'435	2'305	1'798	2'812	0.645	0.413481336	1
ENSBTAG00000005808	*TMEM14C*	5'255	3'450	4'267	4'551	3'984	-0.192	0.804802511	1
ENSBTAG00000005809	*GPR89*	674	419	534	584	484	-0.271	0.760172691	1
ENSBTAG00000005810	*PDZK1*	398	175	273	345	202	-0.770	0.436172221	1
ENSBTAG00000005812	*TP73*	562	1'081	867	487	1'248	1.359	0.108258477	1
ENSBTAG00000005814	*PSME2*	2'583	2'386	2'496	2'237	2'755	0.301	0.701472777	1
ENSBTAG00000005815	*BT.43766*	1'396	1'844	1'669	1'209	2'129	0.817	0.308390778	1

Ensembl gene ID	geneName	counts wildtype horn bud	counts polled horn bud	baseMean	baseMean wildtype horn bud	baseMean polled horn bud	log2FoldChange	pval	padj
ENSBTAG00000005816	*BT.92462*	826	617	714	715	712	-0.006	0.997185985	1
ENSBTAG00000005819	*CCDC61*	1'019	705	848	882	814	-0.116	0.891201694	1
ENSBTAG00000005824	*SPOP*	2'918	2'577	2'751	2'527	2'976	0.236	0.762826674	1
ENSBTAG00000005825	*BT.37835*	130	125	128	113	144	0.358	0.768267938	1
ENSBTAG00000005826	*PTAR1*	127	65	93	110	75	-0.551	0.68635303	1
ENSBTAG00000005827	*FIG4*	1'139	1'107	1'132	986	1'278	0.374	0.647083592	1
ENSBTAG00000005828	*MERTK*	638	367	488	553	424	-0.383	0.668986725	1
ENSBTAG00000005830	*STRN4*	3'271	4'336	3'920	2'833	5'007	0.822	0.29145515	1
ENSBTAG00000005832	*ADRBK1*	2'064	2'663	2'431	1'787	3'075	0.783	0.321136376	1
ENSBTAG00000005833	*ETNK1*	457	206	317	396	238	-0.735	0.444349977	1
ENSBTAG00000005835	*PYCR2*	1'076	1'138	1'123	932	1'314	0.496	0.544270974	1
ENSBTAG00000005838	*ULK3*	2'717	2'064	2'368	2'353	2'383	0.018	0.981180055	1
ENSBTAG00000005841	*protein_coding*	38	12	23	33	14	-1.248	0.637252626	1
ENSBTAG00000005842	*ABCC10*	925	1'306	1'155	801	1'508	0.913	0.26584814	1
ENSBTAG00000005843	*REXO2*	1'648	1'270	1'447	1'427	1'466	0.039	0.961399635	1
ENSBTAG00000005844	*SCAMP2*	7'536	6'020	6'739	6'526	6'951	0.091	0.905133474	1
ENSBTAG00000005845	*MPI*	412	466	447	357	538	0.593	0.511277619	1
ENSBTAG00000005846	*C15ORF17*	740	902	841	641	1'042	0.701	0.403364717	1
ENSBTAG00000005847	*ROCK2*	1'829	690	1'190	1'584	797	-0.991	0.227032395	1
ENSBTAG00000005848	*PRTG*	1	1	1	1	1	0.415	1	1
ENSBTAG00000005850	*DLK2*	215	192	204	186	222	0.252	0.813447999	1
ENSBTAG00000005851	*BT.21128*	2'305	1'517	1'874	1'996	1'752	-0.189	0.813429281	1
ENSBTAG00000005852	*KLF8*	32	29	31	28	33	0.273	0.914612032	1
ENSBTAG00000005854	*COPS8*	2'684	2'214	2'440	2'324	2'557	0.137	0.860966944	1
ENSBTAG00000005855	*pseudogene*	35	26	30	30	30	-0.014	1	1
ENSBTAG00000005858	*BT.106291*	20	15	17	17	17	0.000	1	1
ENSBTAG00000005859	*LALBA*	7	1	4	6	1	-2.392	0.851750348	1
ENSBTAG00000005861	*CCNT1*	425	396	413	368	457	0.313	0.732294061	1
ENSBTAG00000005862	*SMC4*	3'269	1'143	2'075	2'831	1'320	-1.101	0.167952994	1
ENSBTAG00000005863	*ATF7*	671	1'395	1'096	581	1'611	1.471	0.0773874	1
ENSBTAG00000005865	*MAPK6*	3'199	1'890	2'476	2'770	2'182	-0.344	0.662089424	1
ENSBTAG00000005866	*ZNF81*	11	8	9	10	9	-0.044	1	1
ENSBTAG00000005868	*HEBP1*	1'222	2'126	1'757	1'058	2'455	1.214	0.131809593	1
ENSBTAG00000005869	*SENP6*	9'269	3'115	5'812	8'027	3'597	-1.158	0.136807623	1
ENSBTAG00000005870	*FANCL*	1'373	653	972	1'189	754	-0.657	0.428331634	1
ENSBTAG00000005871	*MECOM*	591	242	396	512	279	-0.873	0.344834427	1
ENSBTAG00000005877	*FAM83A*	58	52	55	50	60	0.257	0.885158787	1
ENSBTAG00000005882	*ZNF333*	772	958	887	669	1'106	0.726	0.384165343	1
ENSBTAG00000005884	*WDR31*	41	21	30	36	24	-0.550	0.817917018	1
ENSBTAG00000005888	*MDGA1*	786	1'162	1'011	681	1'342	0.979	0.237632451	1
ENSBTAG00000005891	*FCRL1*	49	6	25	42	7	-2.615	0.321667608	1
ENSBTAG00000005892	*ZAP70*	677	521	594	586	602	0.037	0.967238396	1
ENSBTAG00000005893	*TMEM68*	2'228	1'947	2'089	1'930	2'248	0.221	0.77988887	1
ENSBTAG00000005895	*pseudogene*	10	19	15	9	22	1.341	0.695457515	1
ENSBTAG00000005897	*FNTB*	1'176	1'064	1'124	1'018	1'229	0.271	0.740495188	1
ENSBTAG00000005898	*TGS1*	1'791	1'220	1'480	1'551	1'409	-0.139	0.864131182	1
ENSBTAG00000005903	*BT.68746*	3'639	6'006	5'043	3'151	6'935	1.138	0.144355379	1
ENSBTAG00000005904	*RBBP4*	9'666	4'281	6'657	8'371	4'943	-0.760	0.324966982	1
ENSBTAG00000005905	*TOPORS*	2'923	2'102	2'479	2'531	2'427	-0.061	0.939623099	1
ENSBTAG00000005907	*NDUFB6*	1'683	1'585	1'644	1'458	1'830	0.328	0.680734524	1
ENSBTAG00000005908	*BT.24455*	881	381	601	763	440	-0.794	0.361708838	1
ENSBTAG00000005909	*LEPROT*	4'605	3'269	3'881	3'988	3'775	-0.079	0.919494857	1
ENSBTAG00000005910	*OB-R*	30	4	15	26	5	-2.492	0.481680465	1
ENSBTAG00000005912	*DMAP1*	993	1'305	1'183	860	1'507	0.809	0.322291533	1
ENSBTAG00000005913	*PATL1*	3'332	2'482	2'876	2'886	2'866	-0.010	0.991147315	1
ENSBTAG00000005915	*SFMBT2*	106	61	81	92	70	-0.382	0.793530765	1
ENSBTAG00000005916	*ADNP2*	1'555	1'111	1'315	1'347	1'283	-0.070	0.932649243	1
ENSBTAG00000005917	*BT.88578*	1'550	963	1'227	1'342	1'112	-0.272	0.73936454	1
ENSBTAG00000005922	*CCDC48*	124	98	110	107	113	0.076	0.959151226	1
ENSBTAG00000005923	*ABTB2*	455	1'025	789	394	1'184	1.587	0.064488356	1
ENSBTAG00000005925	*SLC10A2*	1	0	0	1	0		1	1
ENSBTAG00000005928	*MINPP1*	604	317	445	523	366	-0.515	0.569840343	1
ENSBTAG00000005929	*SPTBN2*	2'462	4'132	3'452	2'132	4'771	1.162	0.139321658	1
ENSBTAG00000005932	*FAM184B*	328	57	175	284	66	-2.110	0.062954806	1
ENSBTAG00000005933	*CTNNBIP1*	595	481	535	515	555	0.108	0.903292684	1
ENSBTAG00000005934	*TTYH3*	3'102	5'927	4'765	2'686	6'844	1.349	0.085259534	1
ENSBTAG00000005936	*BT.50841*	38	44	42	33	51	0.627	0.745347708	1
ENSBTAG00000005937	*BT.53551*	50	19	33	43	22	-0.981	0.654631811	1
ENSBTAG00000005940	*PRKAB1*	1'914	2'238	2'121	1'658	2'584	0.641	0.418191954	1
ENSBTAG00000005945	*BT.40405*	123	31	71	107	36	-1.573	0.298793736	1
ENSBTAG00000005946	*USP13*	129	111	120	112	128	0.198	0.876670877	1
ENSBTAG00000005947	*PLAU*	1'888	1'702	1'800	1'635	1'965	0.265	0.738355453	1

Ensembl gene ID	geneName	counts wildtype horn bud	counts polled horn bud	baseMean	baseMean wildtype horn bud	baseMean polled horn bud	log2FoldChange	pval	padj
ENSBTAG00000005950	*BT.88811*	11	1	5	10	1	-3.044	0.722857941	1
ENSBTAG00000005951	*HIC2*	93	158	131	81	182	1.180	0.324935703	1
ENSBTAG00000005952	*BT.87788*	1	0	0	1	0		1	1
ENSBTAG00000005954	*BT.51298*	0	1	1	0	1	Inf	0.993540919	1
ENSBTAG00000005955	*RSPH3*	439	293	359	380	338	-0.168	0.860245029	1
ENSBTAG00000005957	*BT.21493*	3'786	3'604	3'720	3'279	4'162	0.344	0.657329238	1
ENSBTAG00000005958	*PTK2B*	486	357	417	421	412	-0.030	0.977462345	1
ENSBTAG00000005960	*EPB41L2*	9'706	4'656	6'891	8'406	5'376	-0.645	0.402866245	1
ENSBTAG00000005961	*CDC42SE2*	2'638	1'683	2'114	2'285	1'943	-0.233	0.76865282	1
ENSBTAG00000005963	*BT.55380*	39	43	42	34	50	0.556	0.774979973	1
ENSBTAG00000005964	*BT.105430*	429	432	435	372	499	0.425	0.639010058	1
ENSBTAG00000005965	*GPR123*	46	6	23	40	7	-2.524	0.352195564	1
ENSBTAG00000005966	*C9ORF79*	1	0	0	1	0		1	1
ENSBTAG00000005967	*UBR2*	1'353	822	1'060	1'172	949	-0.304	0.71245726	1
ENSBTAG00000005969	*EIF2S2*	4'941	3'951	4'421	4'279	4'562	0.092	0.904382867	1
ENSBTAG00000005970	*XBP1*	10'170	6'481	8'146	8'807	7'484	-0.235	0.759779726	1
ENSBTAG00000005971	*PRPH2*	11	11	11	10	13	0.415	0.940352837	1
ENSBTAG00000005972	*pseudogene*	47	12	27	41	14	-1.555	0.519882605	1
ENSBTAG00000005973	*OVGP1*	256	103	170	222	119	-0.898	0.418716399	1
ENSBTAG00000005975	*C23H6orf130*	2'694	1'873	2'248	2'333	2'163	-0.109	0.890773663	1
ENSBTAG00000005976	*HSD17B7*	1'499	1'007	1'230	1'298	1'163	-0.159	0.846361804	1
ENSBTAG00000005978	*PIK3R5*	141	192	172	122	222	0.860	0.435629708	1
ENSBTAG00000005979	*HELLS*	1'066	809	929	923	934	0.017	0.984380429	1
ENSBTAG00000005980	*RREB1*	1'260	1'659	1'503	1'091	1'916	0.812	0.313724613	1
ENSBTAG00000005984	*DAAM1*	2'915	1'849	2'330	2'524	2'135	-0.242	0.759689028	1
ENSBTAG00000005985	*CBLN2*	575	606	599	498	700	0.491	0.571513258	1
ENSBTAG00000005986	*SFT2D3*	123	82	101	107	95	-0.170	0.904356714	1
ENSBTAG00000005987	*WDR33*	2'969	1'916	2'392	2'571	2'212	-0.217	0.783666807	1
ENSBTAG00000005989	*LAP3*	2'233	2'023	2'135	1'934	2'336	0.273	0.729644986	1
ENSBTAG00000005994	*SPESP1*	8	40	27	7	46	2.737	0.274893759	1
ENSBTAG00000005996	*LRRC18*	10	4	7	9	5	-0.907	0.905171387	1
ENSBTAG00000005997	*ABCB1*	276	127	193	239	147	-0.705	0.511721101	1
ENSBTAG00000005998	*CCRN4L*	622	332	461	539	383	-0.491	0.586224696	1
ENSBTAG00000005999	*FSD1*	308	76	177	267	88	-1.604	0.14950225	1
ENSBTAG00000006001	*STAP2*	393	477	446	340	551	0.694	0.442097113	1
ENSBTAG00000006002	*DIDO1*	2'496	1'917	2'188	2'162	2'214	0.034	0.965275647	1
ENSBTAG00000006003	*SAP130*	2'013	1'232	1'583	1'743	1'423	-0.293	0.71511517	1
ENSBTAG00000006004	*LRRC69*	25	10	17	22	12	-0.907	0.788134871	1
ENSBTAG00000006005	*MPND*	727	720	730	630	831	0.401	0.636352128	1
ENSBTAG00000006007	*SH3GL1*	8'336	9'362	9'015	7'219	10'810	0.582	0.447328114	1
ENSBTAG00000006008	*CAMSAP1*	3'133	4'281	3'828	2'713	4'943	0.865	0.267095944	1
ENSBTAG00000006010	*STAB2*	13	15	14	11	17	0.621	0.873093979	1
ENSBTAG00000006013	*NT5DC3*	65	152	116	56	176	1.641	0.192945519	1
ENSBTAG00000006014	*BT.103245*	5'853	6'524	6'301	5'069	7'533	0.572	0.457703432	1
ENSBTAG00000006015	*BT.91277*	593	450	517	514	520	0.017	0.986494445	1
ENSBTAG00000006016	*GTPBP2*	2'170	1'857	2'012	1'879	2'144	0.190	0.809712509	1
ENSBTAG00000006017	*BT.20288*	1'683	2'225	2'013	1'458	2'569	0.818	0.303583304	1
ENSBTAG00000006019	*FAM178B*	16	14	15	14	16	0.222	0.967786569	1
ENSBTAG00000006021	*CEP250*	1'342	2'081	1'783	1'162	2'403	1.048	0.191491795	1
ENSBTAG00000006022	*ATP6V0E2*	791	616	698	685	711	0.054	0.950237131	1
ENSBTAG00000006024	*ISLR*	5'847	3'676	4'654	5'064	4'245	-0.255	0.74257349	1
ENSBTAG00000006025	*AHSA2*	692	360	507	599	416	-0.528	0.55276687	1
ENSBTAG00000006026	*NGFRAP1*	2'242	1'056	1'580	1'942	1'219	-0.671	0.403730233	1
ENSBTAG00000006027	*BT.104999*	8'202	4'703	6'267	7'103	5'431	-0.387	0.615216816	1
ENSBTAG00000006029	*OGDH*	3'988	6'249	5'335	3'454	7'216	1.063	0.171625074	1
ENSBTAG00000006033	*NDUFA1*	2'365	1'318	1'785	2'048	1'522	-0.428	0.591362021	1
ENSBTAG00000006034	*RNF113A*	762	676	720	660	781	0.242	0.775753033	1
ENSBTAG00000006035	*PPP1R1B*	141	92	114	122	106	-0.201	0.879103316	1
ENSBTAG00000006036	*ERP27*	11	4	7	10	5	-1.044	0.879025484	1
ENSBTAG00000006037	*WISP2*	695	154	390	602	178	-1.759	0.06240277	1
ENSBTAG00000006039	*ARHGDIB*	1'621	869	1'204	1'404	1'003	-0.484	0.552810893	1
ENSBTAG00000006040	*UHRF1BP1L*	1'253	796	1'002	1'085	919	-0.240	0.77282442	1
ENSBTAG00000006042	*C10ORF128*	33	25	29	29	29	0.014	1	1
ENSBTAG00000006043	*BT.61939*	54	45	49	47	52	0.152	0.941451331	1
ENSBTAG00000006044	*KLHL5*	3'236	1'278	2'139	2'802	1'476	-0.925	0.24459971	1
ENSBTAG00000006045	*P4HB*	41'859	36'326	39'098	36'251	41'946	0.211	0.781010978	1
ENSBTAG00000006046	*pseudogene*	18'863	25'243	22'742	16'336	29'148	0.835	0.274695025	1
ENSBTAG00000006048	*DCAKD*	496	717	629	430	828	0.947	0.274576475	1
ENSBTAG00000006049	*BZW1*	9'206	5'990	7'445	7'973	6'917	-0.205	0.789994108	1
ENSBTAG00000006050	*BOLA1*	293	238	264	254	275	0.115	0.910417427	1
ENSBTAG00000006051	*BT.88362*	1'676	1'770	1'748	1'451	2'044	0.494	0.535477669	1
ENSBTAG00000006052	*PLCD3*	1'181	678	903	1'023	783	-0.386	0.644087228	1

Ensembl gene ID	geneName	counts wildtype horn bud	counts polled horn bud	baseMean	baseMean wildtype horn bud	baseMean polled horn bud	log2FoldChange	pval	padj
ENSBTAG00000006054	*ACBD4*	564	594	587	488	686	0.490	0.573203822	1
ENSBTAG00000006056	*HEXIM1*	2'782	1'949	2'330	2'409	2'251	-0.098	0.901695473	1
ENSBTAG00000006059	*TSN*	1'668	1'602	1'647	1'445	1'850	0.357	0.654945557	1
ENSBTAG00000006060	*DCDC5*	16	11	13	14	13	-0.126	0.9977793	1
ENSBTAG00000006063	*TMEM230*	1'555	1'123	1'322	1'347	1'297	-0.055	0.947912302	1
ENSBTAG00000006064	*C19ORF57*	111	86	98	96	99	0.047	0.979517845	1
ENSBTAG00000006065	*PCNA*	4'843	4'600	4'753	4'194	5'312	0.341	0.658645505	1
ENSBTAG00000006066	*CDS2*	3'585	2'879	3'215	3'105	3'324	0.099	0.898892285	1
ENSBTAG00000006067	*VMAC*	510	819	694	442	946	1.098	0.201255115	1
ENSBTAG00000006068	*CC2D1A*	2'402	3'449	3'031	2'080	3'983	0.937	0.232853491	1
ENSBTAG00000006069	*CAPS*	804	685	744	696	791	0.184	0.82842847	1
ENSBTAG00000006070	*RANBP3*	3'318	4'420	3'989	2'873	5'104	0.829	0.287176467	1
ENSBTAG00000006071	*CTTN*	10'286	8'981	9'639	8'908	10'370	0.219	0.773749573	1
ENSBTAG00000006072	*LRRC59*	2'966	2'571	2'769	2'569	2'969	0.209	0.78912889	1
ENSBTAG00000006075	*SUCLG1*	3'971	4'004	4'031	3'439	4'623	0.427	0.581465415	1
ENSBTAG00000006077	*RTBDN*	4	0	2	3	0		0.89545886	1
ENSBTAG00000006078	*IL15RA*	71	41	54	61	47	-0.377	0.830470253	1
ENSBTAG00000006081	*PPM1J*	61	41	50	53	47	-0.158	0.940064916	1
ENSBTAG00000006082	*DNASE2*	2'397	2'060	2'227	2'076	2'379	0.196	0.802881755	1
ENSBTAG00000006083	*KLF1*	10	7	8	9	8	-0.100	1	1
ENSBTAG00000006084	*PINK1*	1'953	2'592	2'342	1'691	2'993	0.823	0.297507487	1
ENSBTAG00000006085	*DDOST*	11'198	9'348	10'246	9'698	10'794	0.155	0.839196115	1
ENSBTAG00000006086	*MMP28*	227	91	151	197	105	-0.904	0.43219469	1
ENSBTAG00000006087	*COG1*	3'105	3'555	3'397	2'689	4'105	0.610	0.433517659	1
ENSBTAG00000006088	*BT.88388*	497	391	441	430	451	0.069	0.940991311	1
ENSBTAG00000006089	*BT.44486*	815	759	791	706	876	0.312	0.710585392	1
ENSBTAG00000006093	*protein_coding*	14	23	19	12	27	1.131	0.701095973	1
ENSBTAG00000006095	*PRDM13*	0	1	1	0	1	Inf	0.993540919	1
ENSBTAG00000006099	*PIP5K1A*	5'539	4'238	4'845	4'797	4'894	0.029	0.969839787	1
ENSBTAG00000006101	*PSMD4*	4'771	4'218	4'501	4'132	4'871	0.237	0.758358041	1
ENSBTAG00000006103	*XRCC6*	2'816	2'385	2'596	2'439	2'754	0.175	0.822639161	1
ENSBTAG00000006104	*NHP2L1*	7'564	7'353	7'521	6'551	8'491	0.374	0.625295328	1
ENSBTAG00000006107	*ANKRD32*	1'222	369	742	1'058	426	-1.313	0.126246633	1
ENSBTAG00000006108	*MMP11*	1'118	1'458	1'326	968	1'684	0.798	0.325409896	1
ENSBTAG00000006110	*BT.100721*	32	14	22	28	16	-0.778	0.782869547	1
ENSBTAG00000006111	*EXOC8*	476	379	425	412	438	0.086	0.926162979	1
ENSBTAG00000006112	*SPRTN*	1'739	1'011	1'337	1'506	1'167	-0.367	0.650455802	1
ENSBTAG00000006114	*ZCCHC8*	2'837	1'686	2'202	2'457	1'947	-0.336	0.671283457	1
ENSBTAG00000006116	*SF4*	2'234	2'351	2'325	1'935	2'715	0.489	0.534802262	1
ENSBTAG00000006117	*FLYWCH1*	239	331	295	207	382	0.885	0.362835048	1
ENSBTAG00000006118	*RSRC2*	10'836	4'596	7'346	9'384	5'307	-0.822	0.286591936	1
ENSBTAG00000006121	*SBK2*	41	3	19	36	3	-3.358	0.288139309	1
ENSBTAG00000006122	*BT.101023*	12'072	7'821	9'743	10'455	9'031	-0.211	0.783125892	1
ENSBTAG00000006124	*MAU2*	1'798	2'329	2'123	1'557	2'689	0.788	0.320082539	1
ENSBTAG00000006125	*PLK3*	538	349	434	466	403	-0.209	0.819749553	1
ENSBTAG00000006126	*GTF3C1*	5'674	10'277	8'390	4'914	11'867	1.272	0.101348019	1
ENSBTAG00000006129	*KIAA0556*	1'355	1'886	1'676	1'173	2'178	0.892	0.26627156	1
ENSBTAG00000006130	*CLEC14A*	2'571	1'278	1'851	2'227	1'476	-0.593	0.456633494	1
ENSBTAG00000006132	*DENND3*	347	457	414	301	528	0.812	0.374540709	1
ENSBTAG00000006134	*DYNLRB1*	2'188	2'015	2'111	1'895	2'327	0.296	0.707385742	1
ENSBTAG00000006135	*MAP1LC3A*	3'645	2'494	3'018	3'157	2'880	-0.132	0.866239185	1
ENSBTAG00000006136	*CDA*	47	29	37	41	33	-0.282	0.901599712	1
ENSBTAG00000006139	*TRPM4*	999	1'106	1'071	865	1'277	0.562	0.493755844	1
ENSBTAG00000006140	*PEX11A*	505	624	579	437	721	0.720	0.408945294	1
ENSBTAG00000006141	*WDR93*	81	49	63	70	57	-0.310	0.851677552	1
ENSBTAG00000006142	*DHX36*	2'415	1'346	1'823	2'091	1'554	-0.428	0.591122831	1
ENSBTAG00000006143	*BT.39650*	6'040	6'342	6'277	5'231	7'323	0.485	0.527878357	1
ENSBTAG00000006147	*GPR149*	5	3	4	4	3	-0.322	1	1
ENSBTAG00000006150	*MRPS9*	822	747	787	712	863	0.277	0.742267172	1
ENSBTAG00000006152	*processed_pseudogene*	298	594	472	258	686	1.410	0.119981726	1
ENSBTAG00000006155	*CCRL2*	22	17	19	19	20	0.043	1	1
ENSBTAG00000006156	*BST1*	8	1	4	7	1	-2.585	0.818599781	1
ENSBTAG00000006157	*HS6ST1*	528	703	635	457	812	0.828	0.337913551	1
ENSBTAG00000006158	*DZIP3*	1'385	640	969	1'199	739	-0.699	0.39994303	1
ENSBTAG00000006159	*BT.87160*	71	6	34	61	7	-3.150	0.16403829	1
ENSBTAG00000006160	*SUOX*	1'567	1'026	1'271	1'357	1'185	-0.196	0.810342038	1
ENSBTAG00000006161	*MET*	919	965	955	796	1'114	0.486	0.55756408	1
ENSBTAG00000006162	*RAB11FIP5*	1'380	2'082	1'800	1'195	2'404	1.008	0.208282527	1
ENSBTAG00000006165	*PRDX4*	9'396	5'170	7'053	8'137	5'970	-0.447	0.561452827	1
ENSBTAG00000006166	*BT.88577*	2'010	2'373	2'240	1'741	2'740	0.655	0.407255524	1
ENSBTAG00000006167	*BT.26364*	1'023	374	659	886	432	-1.037	0.230504554	1
ENSBTAG00000006168	*RBM45*	2'608	1'559	2'029	2'259	1'800	-0.327	0.680108698	1

Ensembl gene ID	geneName	counts wildtype horn bud	counts polled horn bud	baseMean	baseMean wildtype horn bud	baseMean polled horn bud	log2FoldChange	pval	padj
ENSBTAG00000006170	*ZNF76*	2'043	1'828	1'940	1'769	2'111	0.255	0.747781078	1
ENSBTAG00000006173	*CCDC116*	24	9	16	21	10	-1.000	0.775206422	1
ENSBTAG00000006174	*TMEM212*	2	0	1	2	0		0.974934741	1
ENSBTAG00000006175	*KDM5B*	5'544	6'519	6'164	4'801	7'527	0.649	0.399911963	1
ENSBTAG00000006176	*BT.54980*	141	161	154	122	186	0.606	0.594486817	1
ENSBTAG00000006177	*ZDHHC20*	1'021	905	965	884	1'045	0.241	0.770730473	1
ENSBTAG00000006182	*pseudogene*	1'666	1'836	1'781	1'443	2'120	0.555	0.485796678	1
ENSBTAG00000006185	*BT.67115*	660	499	574	572	576	0.012	0.990964054	1
ENSBTAG00000006186	*KCTD3*	3'041	2'047	2'499	2'634	2'364	-0.156	0.843507761	1
ENSBTAG00000006187	*MFAP4*	13'791	25'164	20'500	11'943	29'057	1.283	0.09652097	1
ENSBTAG00000006188	*USH2A*	18	9	13	16	10	-0.585	0.894065104	1
ENSBTAG00000006189	*ACTG1*	96'404	105'221	102'494	83'488	121'499	0.541	0.475893502	1
ENSBTAG00000006191	*SERPINA12*	46	9	25	40	10	-1.939	0.447293074	1
ENSBTAG00000006193	*IRAK2*	593	207	376	514	239	-1.103	0.237944865	1
ENSBTAG00000006194	*BT.17885*	6	6	6	5	7	0.415	0.976119138	1
ENSBTAG00000006195	*BT.20334*	1'472	1'871	1'718	1'275	2'160	0.761	0.34138114	1
ENSBTAG00000006197	*ZDHHC21*	56	32	43	48	37	-0.392	0.844443139	1
ENSBTAG00000006199	*DRAP1*	3'417	3'389	3'436	2'959	3'913	0.403	0.603992543	1
ENSBTAG00000006200	*SLC25A17*	1'693	1'403	1'543	1'466	1'620	0.144	0.857380969	1
ENSBTAG00000006201	*DTWD1*	656	397	513	568	458	-0.310	0.728160519	1
ENSBTAG00000006202	*ST13*	9'686	10'102	10'027	8'388	11'665	0.476	0.533964378	1
ENSBTAG00000006204	*BT.54391*	273	262	269	236	303	0.356	0.71978576	1
ENSBTAG00000006205	*BT.87475*	2	0	1	2	0		0.974934741	1
ENSBTAG00000006208	*ADCY7*	1'292	577	893	1'119	666	-0.748	0.37106854	1
ENSBTAG00000006209	*EXT1*	3'073	2'209	2'606	2'661	2'551	-0.061	0.938917168	1
ENSBTAG00000006212	*GTF2IRD1*	990	1'768	1'449	857	2'042	1.252	0.124185506	1
ENSBTAG00000006213	*BT.87445*	528	762	669	457	880	0.944	0.272500605	1
ENSBTAG00000006214	*LOXL2*	4'129	5'853	5'167	3'576	6'758	0.918	0.236578937	1
ENSBTAG00000006218	*FOXI2*	0	1	1	0	1	Inf	0.993540919	1
ENSBTAG00000006221	*GPR97*	5	0	2	4	0		0.853268594	1
ENSBTAG00000006222	*TFDP2*	354	459	418	307	530	0.790	0.387060647	1
ENSBTAG00000006223	*C1ORF38*	67	55	61	58	64	0.130	0.944410229	1
ENSBTAG00000006225	*RPA2*	560	603	591	485	696	0.522	0.548190893	1
ENSBTAG00000006227	*IDH3A*	2'512	1'427	1'912	2'175	1'648	-0.401	0.614403371	1
ENSBTAG00000006231	*ACSBG1*	213	84	141	184	97	-0.927	0.430001307	1
ENSBTAG00000006232	*WDR86*	213	186	200	184	215	0.219	0.83854951	1
ENSBTAG00000006233	*BT.88406*	123	75	97	107	87	-0.299	0.828206237	1
ENSBTAG00000006234	*BT.76843*	591	332	448	512	383	-0.417	0.645476022	1
ENSBTAG00000006235	*INTS3*	3'899	5'777	5'024	3'377	6'671	0.982	0.206298347	1
ENSBTAG00000006236	*pseudogene*	2	0	1	2	0		0.974934741	1
ENSBTAG00000006239	*BT.88677*	40	31	35	35	36	0.047	0.996958386	1
ENSBTAG00000006240	*TLR4*	460	165	294	398	191	-1.064	0.276245437	1
ENSBTAG00000006241	*MANB*	8'033	8'448	8'356	6'957	9'755	0.488	0.524491074	1
ENSBTAG00000006242	*USP9X*	7'602	3'794	5'482	6'584	4'381	-0.588	0.447104608	1
ENSBTAG00000006243	*GCNT7*	9	3	6	8	3	-1.170	0.890813994	1
ENSBTAG00000006244	*WDR83OS*	2'664	2'049	2'337	2'307	2'366	0.036	0.963026667	1
ENSBTAG00000006245	*MORG1*	935	953	955	810	1'100	0.443	0.592860996	1
ENSBTAG00000006246	*BT.44024*	627	590	612	543	681	0.327	0.705304339	1
ENSBTAG00000006247	*DHPS*	1'814	1'876	1'869	1'571	2'166	0.464	0.559443427	1
ENSBTAG00000006250	*CASC3*	7'550	5'707	6'564	6'538	6'590	0.011	0.987811672	1
ENSBTAG00000006251	*BT.106624*	2	1	1	2	1	-0.585	1	1
ENSBTAG00000006252	*RBMX2*	347	205	269	301	237	-0.344	0.730325966	1
ENSBTAG00000006255	*BT.36413*	382	154	254	331	178	-0.896	0.373180317	1
ENSBTAG00000006259	*MOBKL1B*	2'179	1'814	1'991	1'887	2'095	0.151	0.848995643	1
ENSBTAG00000006260	*KIAA1614*	528	1'102	865	457	1'272	1.477	0.081891078	1
ENSBTAG00000006261	*GPR17*	361	212	279	313	245	-0.353	0.721585351	1
ENSBTAG00000006262	*LIMS2*	781	396	567	676	457	-0.565	0.519156446	1
ENSBTAG00000006263	*BT.67549*	5	2	3	4	2	-0.907	0.965043111	1
ENSBTAG00000006268	*IKBKG*	687	894	814	595	1'032	0.795	0.345062847	1
ENSBTAG00000006270	*HSP90AA1*	44'694	40'809	42'914	38'706	47'122	0.284	0.708012658	1
ENSBTAG00000006272	*NTHL1*	1'119	924	1'018	969	1'067	0.139	0.866401865	1
ENSBTAG00000006276	*C1QTNF1*	2'984	2'800	2'909	2'584	3'233	0.323	0.678742022	1
ENSBTAG00000006277	*ENGASE*	1'195	992	1'090	1'035	1'145	0.146	0.858301712	1
ENSBTAG00000006278	*FAM83C*	162	64	107	140	74	-0.925	0.471879256	1
ENSBTAG00000006279	*PNPLA5*	2	0	1	2	0		0.974934741	1
ENSBTAG00000006280	*BT.51613*	314	154	225	272	178	-0.613	0.553425084	1
ENSBTAG00000006282	*POU2AF1*	9	2	5	8	2	-1.755	0.844314416	1
ENSBTAG00000006287	*NEDD9*	1'405	1'346	1'385	1'217	1'554	0.353	0.66128559	1
ENSBTAG00000006288	*VSTM4*	2'195	2'933	2'644	1'901	3'387	0.833	0.289896351	1
ENSBTAG00000006291	*CYLD*	1'956	1'031	1'442	1'694	1'190	-0.509	0.528385024	1
ENSBTAG00000006293	*PLAC1*	9	5	7	8	6	-0.433	0.969858938	1
ENSBTAG00000006295	*TSHB*	6	0	3	5	0		0.81115604	1

Ensembl gene ID	geneName	counts wildtype horn bud	counts polled horn bud	baseMean	baseMean wildtype horn bud	baseMean polled horn bud	log2FoldChange	pval	padj
ENSBTAG00000006296	*VMA21*	2'212	2'070	2'153	1'916	2'390	0.319	0.685530974	1
ENSBTAG00000006305	*AK1*	1'041	434	701	902	501	-0.847	0.322611941	1
ENSBTAG00000006307	*HSD17B11*	1'088	472	744	942	545	-0.790	0.353280114	1
ENSBTAG00000006320	*DBT*	1'073	879	972	929	1'015	0.127	0.877892526	1
ENSBTAG00000006321	*RTCA*	1'609	676	1'087	1'393	781	-0.836	0.310307242	1
ENSBTAG00000006322	*DENND5A*	4'136	3'532	3'830	3'582	4'078	0.187	0.808727071	1
ENSBTAG00000006323	*SNX9*	3'392	2'854	3'117	2'938	3'296	0.166	0.831104369	1
ENSBTAG00000006324	*NAB2*	5'097	5'211	5'216	4'414	6'017	0.447	0.562059465	1
ENSBTAG00000006325	*NUDCD3*	2'338	3'306	2'921	2'025	3'817	0.915	0.244395477	1
ENSBTAG00000006326	*ALDH1L2*	2'841	1'768	2'251	2'460	2'042	-0.269	0.733535908	1
ENSBTAG00000006328	*RBM6*	6'053	3'490	4'636	5'242	4'030	-0.379	0.62425828	1
ENSBTAG00000006330	*RBM5*	25'457	12'662	18'334	22'046	14'621	-0.593	0.438121287	1
ENSBTAG00000006335	*STAT6*	4'116	5'857	5'164	3'565	6'763	0.924	0.233815417	1
ENSBTAG00000006338	*MYCL1*	75	93	86	65	107	0.725	0.600844189	1
ENSBTAG00000006339	*HMGN1*	19	3	10	16	3	-2.248	0.640043503	1
ENSBTAG00000006341	*SLC35F4*	11	0	5	10	0		0.617662983	1
ENSBTAG00000006342	*ATPIF1*	3'505	3'215	3'374	3'035	3'712	0.290	0.708524006	1
ENSBTAG00000006343	*IL1R2*	50	35	42	43	40	-0.100	0.972970357	1
ENSBTAG00000006345	*BT.88084*	3	10	7	3	12	2.152	0.727540088	1
ENSBTAG00000006346	*DAP*	16'223	15'330	15'876	14'050	17'702	0.333	0.661394079	1
ENSBTAG00000006347	*CIRH1A*	1'292	935	1'099	1'119	1'080	-0.052	0.951677667	1
ENSBTAG00000006349	*EXTL1*	233	44	126	202	51	-1.990	0.109648094	1
ENSBTAG00000006350	*BT.29906*	2	0	1	2	0		0.974934741	1
ENSBTAG00000006353	*TXNL4B*	1'322	823	1'048	1'145	950	-0.269	0.744932582	1
ENSBTAG00000006354	*HP*	26	3	13	23	3	-2.700	0.499863335	1
ENSBTAG00000006355	*SLC30A2*	4	3	3	3	3	0.000	1	1
ENSBTAG00000006357	*CRIP3*	33	28	30	29	32	0.178	0.950448967	1
ENSBTAG00000006359	*EPHA1*	1'287	1'690	1'533	1'115	1'951	0.808	0.315510219	1
ENSBTAG00000006360	*BT.22214*	13	0	6	11	0		0.551229012	1
ENSBTAG00000006364	*C3H1orf85*	985	1'142	1'086	853	1'319	0.628	0.443844387	1
ENSBTAG00000006366	*NFATC4*	6'509	8'703	7'843	5'637	10'049	0.834	0.278955837	1
ENSBTAG00000006367	*CTGF*	6'168	6'676	6'525	5'342	7'709	0.529	0.491311291	1
ENSBTAG00000006368	*N4BP3*	172	377	292	149	435	1.547	0.116844838	1
ENSBTAG00000006369	*MRPS35*	684	645	669	592	745	0.330	0.699764056	1
ENSBTAG00000006370	*BT.28845*	10'291	12'897	11'902	8'912	14'892	0.741	0.333801559	1
ENSBTAG00000006371	*RMND5B*	2'810	1'964	2'351	2'434	2'268	-0.102	0.898245404	1
ENSBTAG00000006372	*KLHDC5*	189	121	152	164	140	-0.228	0.846852389	1
ENSBTAG00000006373	*BT.53541*	24	24	24	21	28	0.415	0.881615086	1
ENSBTAG00000006374	*NHP2*	1'234	1'293	1'281	1'069	1'493	0.482	0.551705497	1
ENSBTAG00000006377	*MYO1G*	51	23	35	44	27	-0.734	0.729360125	1
ENSBTAG00000006378	*RIPK1*	1'540	1'125	1'316	1'334	1'299	-0.038	0.964275716	1
ENSBTAG00000006379	*BPHL*	758	391	554	656	451	-0.540	0.538858143	1
ENSBTAG00000006383	*protein_coding*	96'846	123'504	113'241	83'871	142'610	0.766	0.314587729	1
ENSBTAG00000006384	*protein_coding*	1	0	0	1	0		1	1
ENSBTAG00000006385	*BAI1*	802	744	777	695	859	0.307	0.71604366	1
ENSBTAG00000006386	*protein_coding*	427	804	649	370	928	1.328	0.126820923	1
ENSBTAG00000006387	*TSNAXIP1*	24	4	13	21	5	-2.170	0.58681328	1
ENSBTAG00000006388	*FBXO45*	146	109	126	126	126	-0.007	1	1
ENSBTAG00000006391	*BT.87145*	977	537	733	846	620	-0.448	0.598253026	1
ENSBTAG00000006392	*TTC7B*	626	355	476	542	410	-0.403	0.653404925	1
ENSBTAG00000006393	*FECH*	1'460	1'521	1'510	1'264	1'756	0.474	0.55474917	1
ENSBTAG00000006395	*TUBGCP2*	3'073	4'352	3'843	2'661	5'025	0.917	0.239966049	1
ENSBTAG00000006396	*GPI*	7'241	8'758	8'192	6'271	10'113	0.689	0.369584126	1
ENSBTAG00000006397	*pseudogene*	4'756	5'709	5'356	4'119	6'592	0.679	0.379855105	1
ENSBTAG00000006398	*BT.70495*	900	221	517	779	255	-1.611	0.073972217	1
ENSBTAG00000006403	*C1ORF115*	199	86	136	172	99	-0.795	0.503162675	1
ENSBTAG00000006404	*CENPT*	2'151	1'946	2'055	1'863	2'247	0.271	0.731965505	1
ENSBTAG00000006405	*PALB2*	1'082	515	766	937	595	-0.656	0.438851897	1
ENSBTAG00000006408	*C1ORF129*	3	1	2	3	1	-1.170	0.985253077	1
ENSBTAG00000006409	*LRRC34*	384	75	210	333	87	-1.941	0.071413367	1
ENSBTAG00000006410	*MGC3248*	1'642	1'279	1'449	1'422	1'477	0.055	0.946053497	1
ENSBTAG00000006411	*THAP11*	2'605	2'483	2'562	2'256	2'867	0.346	0.658998948	1
ENSBTAG00000006413	*C15ORF29*	252	188	218	218	217	-0.008	1	1
ENSBTAG00000006414	*NUTF2*	3'312	2'894	3'105	2'868	3'342	0.220	0.777022392	1
ENSBTAG00000006415	*LRRIQ4*	1	0	0	1	0		1	1
ENSBTAG00000006416	*EMC4*	2'931	2'270	2'580	2'538	2'621	0.046	0.952695118	1
ENSBTAG00000006417	*EDC4*	3'408	5'250	4'507	2'951	6'062	1.038	0.182841103	1
ENSBTAG00000006419	*TNNT1*	36	21	28	31	24	-0.363	0.891403315	1
ENSBTAG00000006420	*BT.25715*	925	594	743	801	686	-0.224	0.793101288	1
ENSBTAG00000006422	*TRIM28*	12'913	20'190	17'248	11'183	23'313	1.060	0.16801325	1
ENSBTAG00000006423	*PHF5A*	2'038	1'645	1'832	1'765	1'899	0.106	0.893820895	1
ENSBTAG00000006424	*TNNI3*	63	3	29	55	3	-3.977	0.126325513	1

Ensembl gene ID	geneName	counts wildtype horn bud	counts polled horn bud	baseMean	baseMean wildtype horn bud	baseMean polled horn bud	log2FoldChange	pval	padj
ENSBTAG00000006428	*ZNF354A*	1'213	510	820	1'050	589	-0.835	0.322122885	1
ENSBTAG00000006429	*ACO2*	4'347	7'612	6'277	3'765	8'790	1.223	0.116001192	1
ENSBTAG00000006434	*SYNPO2*	296	296	299	256	342	0.415	0.668352837	1
ENSBTAG00000006438	*BT.51053*	38	81	63	33	94	1.507	0.339729353	1
ENSBTAG00000006439	*DOCK11*	1'272	1'137	1'207	1'102	1'313	0.253	0.755514834	1
ENSBTAG00000006440	*IGDCC4*	4'144	4'973	4'666	3'589	5'742	0.678	0.381279459	1
ENSBTAG00000006441	*ATP5F1*	7'661	5'532	6'511	6'635	6'388	-0.055	0.944107066	1
ENSBTAG00000006443	*PRDM11*	201	355	292	174	410	1.236	0.2071835	1
ENSBTAG00000006446	*BT.86327*	113	29	66	98	33	-1.547	0.322741527	1
ENSBTAG00000006447	*ACSM3*	215	81	140	186	94	-0.993	0.399038855	1
ENSBTAG00000006448	*ERI2*	331	147	228	287	170	-0.756	0.462983004	1
ENSBTAG00000006449	*MGC134577*	1'074	925	999	930	1'068	0.200	0.808885056	1
ENSBTAG00000006452	*CD3D*	34	9	20	29	10	-1.503	0.606713166	1
ENSBTAG00000006453	*CD3G*	41	12	25	36	14	-1.358	0.595766107	1
ENSBTAG00000006457	*AHSP*	33	10	20	29	12	-1.307	0.653206662	1
ENSBTAG00000006463	*DLST*	2'792	2'691	2'763	2'418	3'107	0.362	0.643472851	1
ENSBTAG00000006464	*GGA1*	1'790	2'307	2'107	1'550	2'664	0.781	0.324653821	1
ENSBTAG00000006466	*CD53*	359	146	240	311	169	-0.883	0.386190441	1
ENSBTAG00000006468	*PIWIL1*	16	11	13	14	13	-0.126	0.9977793	1
ENSBTAG00000006470	*TTBK2*	154	95	122	133	110	-0.282	0.82348477	1
ENSBTAG00000006471	*BT.27563*	1'367	707	1'000	1'184	816	-0.536	0.516899967	1
ENSBTAG00000006472	*GPR143*	144	146	147	125	169	0.435	0.707862693	1
ENSBTAG00000006474	*EPCAM*	1'896	1'697	1'801	1'642	1'960	0.255	0.748189036	1
ENSBTAG00000006478	*BT.21936*	450	252	340	390	291	-0.421	0.657048025	1
ENSBTAG00000006479	*CAPN7*	2'829	1'408	2'038	2'450	1'626	-0.592	0.456117695	1
ENSBTAG00000006481	*BT.52712*	1'591	2'337	2'038	1'378	2'699	0.970	0.223406846	1
ENSBTAG00000006482	*PTCD3*	1'819	1'100	1'423	1'575	1'270	-0.311	0.700768186	1
ENSBTAG00000006485	*TREML1*	5	12	9	4	14	1.678	0.738147499	1
ENSBTAG00000006486	*TSEN34*	1'549	2'135	1'903	1'341	2'465	0.878	0.270998419	1
ENSBTAG00000006487	*RPS9*	26'429	25'654	26'255	22'888	29'623	0.372	0.624282037	1
ENSBTAG00000006489	*MYO5A*	1'145	631	860	992	729	-0.445	0.595714412	1
ENSBTAG00000006490	*HLA-DOA*	30	1	14	26	1	-4.492	0.304362174	1
ENSBTAG00000006491	*AGL*	310	213	257	268	246	-0.126	0.903602031	1
ENSBTAG00000006492	*BT.25341*	1'217	749	959	1'054	865	-0.285	0.7315841	1
ENSBTAG00000006493	*CLP1*	710	558	630	615	644	0.067	0.938737652	1
ENSBTAG00000006494	*CLDN16*	5	6	6	4	7	0.678	0.946418607	1
ENSBTAG00000006495	*GNB2*	8'584	10'652	9'867	7'434	12'300	0.726	0.343826486	1
ENSBTAG00000006497	*pseudogene*	327	76	185	283	88	-1.690	0.125109354	1
ENSBTAG00000006499	*PIP4K2B*	1'170	1'461	1'350	1'013	1'687	0.735	0.363878523	1
ENSBTAG00000006500	*RBM15B*	1'608	2'642	2'222	1'393	3'051	1.131	0.155350412	1
ENSBTAG00000006501	*VPRBP*	2'142	1'939	2'047	1'855	2'239	0.271	0.731199121	1
ENSBTAG00000006504	*BT.43244*	1'302	1'002	1'142	1'128	1'157	0.037	0.964120932	1
ENSBTAG00000006506	*GIT2*	1'961	1'439	1'680	1'698	1'662	-0.031	0.970038446	1
ENSBTAG00000006507	*ADAMTS3*	226	63	134	196	73	-1.428	0.234478053	1
ENSBTAG00000006508	*MAN2B2*	0	1	1	0	1	Inf	0.993540919	1
ENSBTAG00000006510	*YRDC*	885	572	713	766	660	-0.215	0.802504367	1
ENSBTAG00000006511	*MTF1*	285	348	324	247	402	0.703	0.460608262	1
ENSBTAG00000006515	*ESPN*	105	24	59	91	28	-1.714	0.296160751	1
ENSBTAG00000006517	*DRG2*	1'957	2'376	2'219	1'695	2'744	0.695	0.379354694	1
ENSBTAG00000006519	*BT.30010*	1'152	897	1'017	998	1'036	0.054	0.948169316	1
ENSBTAG00000006520	*MYO15A*	127	71	96	110	82	-0.424	0.754764285	1
ENSBTAG00000006523	*SOD2*	476	464	474	412	536	0.378	0.672594669	1
ENSBTAG00000006525	*BT.64647*	645	859	775	559	992	0.828	0.327597606	1
ENSBTAG00000006526	*BCL2L1*	1'715	2'226	2'028	1'485	2'570	0.791	0.319203618	1
ENSBTAG00000006528	*BBS9*	711	347	508	616	401	-0.620	0.485534891	1
ENSBTAG00000006529	*BT.52184*	659	308	463	571	356	-0.682	0.448739703	1
ENSBTAG00000006530	*processed_pseudogene*	934	547	720	809	632	-0.357	0.67582471	1
ENSBTAG00000006531	*DIP2C*	1'659	2'075	1'916	1'437	2'396	0.738	0.353770118	1
ENSBTAG00000006532	*CDK5RAP2*	2'502	2'362	2'447	2'167	2'727	0.332	0.6723203	1
ENSBTAG00000006533	*NSFL1C*	3'721	2'409	3'002	3'222	2'782	-0.212	0.786595194	1
ENSBTAG00000006534	*GTPBP8*	64	61	63	55	70	0.346	0.83169858	1
ENSBTAG00000006535	*IRS4*	23	113	75	20	130	2.712	0.076334291	1
ENSBTAG00000006536	*CGN1*	1	0	0	1	0		1	1
ENSBTAG00000006538	*PTHLH*	280	416	361	242	480	0.986	0.292877116	1
ENSBTAG00000006539	*SIRPB2*	14	7	10	12	8	-0.585	0.916699259	1
ENSBTAG00000006542	*RABEP2*	1'345	1'246	1'302	1'165	1'439	0.305	0.706411812	1
ENSBTAG00000006543	*EIF3C*	8'246	7'364	7'822	7'141	8'503	0.252	0.741975909	1
ENSBTAG00000006546	*BT.73429*	272	149	204	236	172	-0.453	0.669764947	1
ENSBTAG00000006547	*CCDC25*	791	658	722	685	760	0.149	0.860879827	1
ENSBTAG00000006548	*MCCC1*	1'142	744	924	989	859	-0.203	0.808177193	1
ENSBTAG00000006549	*FAM26F*	13	2	7	11	2	-2.285	0.731320841	1
ENSBTAG00000006550	*BT.101926*	4'938	3'127	3'944	4'276	3'611	-0.244	0.75362736	1

Ensembl gene ID	geneName	counts wildtype horn bud	counts polled horn bud	baseMean	baseMean wildtype horn bud	baseMean polled horn bud	log2FoldChange	pval	padj
ENSBTAG00000006551	*ESCO2*	404	392	401	350	453	0.372	0.685849034	1
ENSBTAG00000006552	*LAMP3*	67	23	42	58	27	-1.127	0.554514656	1
ENSBTAG00000006555	*ZMYM1*	822	549	673	712	634	-0.167	0.847020951	1
ENSBTAG00000006556	*COPB1*	11'841	9'012	10'330	10'255	10'406	0.021	0.97736098	1
ENSBTAG00000006557	*BT.22959*	21	18	19	18	21	0.193	0.964623995	1
ENSBTAG00000006558	*SOHLH1*	9	0	4	8	0		0.690676647	1
ENSBTAG00000006560	*VANGL2*	408	793	635	353	916	1.374	0.115547292	1
ENSBTAG00000006561	*RASGEF1B*	1'935	2'290	2'160	1'676	2'644	0.658	0.405429976	1
ENSBTAG00000006562	*pseudogene*	0	7	4	0	8	Inf	0.646259203	1
ENSBTAG00000006564	*PSMA1*	3'630	2'836	3'209	3'144	3'275	0.059	0.939404566	1
ENSBTAG00000006566	*GPR43*	1	1	1	1	1	0.415	1	1
ENSBTAG00000006567	*BT.46336*	139	364	270	120	420	1.804	0.073950841	1
ENSBTAG00000006568	*TBC1D8B*	583	281	415	505	324	-0.638	0.486150493	1
ENSBTAG00000006569	*SEZ6L*	287	46	151	249	53	-2.226	0.060919205	1
ENSBTAG00000006570	*SEC22C*	125	144	137	108	166	0.619	0.599650325	1
ENSBTAG00000006572	*BT.26197*	246	138	186	213	159	-0.419	0.700758544	1
ENSBTAG00000006573	*KLHL18*	981	983	992	850	1'135	0.418	0.612422611	1
ENSBTAG00000006574	*ARL8A*	1'083	900	989	938	1'039	0.148	0.857937161	1
ENSBTAG00000006578	*SPEF1*	192	172	182	166	199	0.256	0.815759923	1
ENSBTAG00000006579	*P4HA3*	5'201	2'201	3'523	4'504	2'541	-0.826	0.290945457	1
ENSBTAG00000006581	*BT.57634*	2'525	1'189	1'780	2'187	1'373	-0.671	0.400716805	1
ENSBTAG00000006582	*BT.95277*	99	30	60	86	35	-1.307	0.419020486	1
ENSBTAG00000006586	*KPNB1*	6'155	5'751	5'986	5'330	6'641	0.317	0.679766419	1
ENSBTAG00000006587	*ZNF367*	361	266	310	313	307	-0.026	0.98338627	1
ENSBTAG00000006588	*CBY1*	2'398	1'785	2'069	2'077	2'061	-0.011	0.990428106	1
ENSBTAG00000006589	*CFTR*	122	61	88	106	70	-0.585	0.673823195	1
ENSBTAG00000006590	*NUDT16L1*	1'011	1'025	1'030	876	1'184	0.435	0.597091311	1
ENSBTAG00000006591	*CCNF*	509	705	627	441	814	0.885	0.306724744	1
ENSBTAG00000006592	*SGSM1*	383	346	366	332	400	0.268	0.774189129	1
ENSBTAG00000006593	*ANKS3*	848	1'066	983	734	1'231	0.745	0.368174835	1
ENSBTAG00000006594	*TMEM211*	20	19	20	17	22	0.341	0.920476198	1
ENSBTAG00000006595	*BT.33223*	231	198	214	200	229	0.193	0.855870758	1
ENSBTAG00000006601	*KSR1*	666	776	736	577	896	0.636	0.453876612	1
ENSBTAG00000006606	*FAM101B*	202	96	143	175	111	-0.658	0.57387824	1
ENSBTAG00000006607	*CCNG1*	12'343	7'589	9'726	10'689	8'763	-0.287	0.708511561	1
ENSBTAG00000006609	*ERRFI1*	1'260	621	904	1'091	717	-0.606	0.467868662	1
ENSBTAG00000006610	*BT.27277*	104	60	80	90	69	-0.379	0.797082497	1
ENSBTAG00000006611	*NUP93*	2'646	2'702	2'706	2'292	3'120	0.445	0.56956066	1
ENSBTAG00000006612	*SCAF1*	2'343	3'452	3'008	2'029	3'986	0.974	0.215257662	1
ENSBTAG00000006613	*BT.35194*	1'448	1'242	1'344	1'254	1'434	0.194	0.810493091	1
ENSBTAG00000006614	*TMCC1*	767	471	604	664	544	-0.288	0.740841216	1
ENSBTAG00000006615	*BT.62821*	289	301	299	250	348	0.474	0.624637977	1
ENSBTAG00000006616	*PTPN1*	750	900	844	650	1'039	0.678	0.418409203	1
ENSBTAG00000006618	*BT.39657*	129	75	99	112	87	-0.367	0.78517519	1
ENSBTAG00000006619	*BT.24440*	987	601	774	855	694	-0.301	0.722910396	1
ENSBTAG00000006624	*SLC36A3*	10	7	8	9	8	-0.100	1	1
ENSBTAG00000006625	*TMEM48*	555	444	497	481	513	0.093	0.917945596	1
ENSBTAG00000006626	*TMEM132B*	57	8	29	49	9	-2.418	0.308761478	1
ENSBTAG00000006630	*BT.27296*	9	1	4	8	1	-2.755	0.785977687	1
ENSBTAG00000006633	*IRF3*	4'645	4'402	4'553	4'023	5'083	0.338	0.661951129	1
ENSBTAG00000006635	*DBX2*	39	17	27	34	20	-0.783	0.753120629	1
ENSBTAG00000006638	*BCL2L12*	927	799	863	803	923	0.201	0.810275808	1
ENSBTAG00000006639	*CSTF1*	1'594	982	1'257	1'380	1'134	-0.284	0.727626806	1
ENSBTAG00000006640	*BT.68871*	2'675	864	1'657	2'317	998	-1.215	0.132710753	1
ENSBTAG00000006642	*PRKACA*	1'491	2'114	1'866	1'291	2'441	0.919	0.250081465	1
ENSBTAG00000006643	*BT.22022*	93	39	63	81	45	-0.839	0.597554793	1
ENSBTAG00000006644	*SPCS2*	5'190	3'196	4'093	4'495	3'690	-0.284	0.714245878	1
ENSBTAG00000006646	*HRMT1L2*	7'175	9'966	8'861	6'214	11'508	0.889	0.248315177	1
ENSBTAG00000006647	*RCBTB2*	6'839	3'018	4'704	5'923	3'485	-0.765	0.324340588	1
ENSBTAG00000006649	*TSSK6*	2	3	3	2	3	1.000	0.97171833	1
ENSBTAG00000006650	*ZNF668*	253	252	255	219	291	0.409	0.683007844	1
ENSBTAG00000006651	*INSL6*	39	9	22	34	10	-1.700	0.535008866	1
ENSBTAG00000006654	*TANK*	1'820	1'166	1'461	1'576	1'346	-0.227	0.778527784	1
ENSBTAG00000006656	*RANBP1*	1'183	833	993	1'025	962	-0.091	0.913995753	1
ENSBTAG00000006657	*ZDHHC8*	856	1'033	967	741	1'193	0.686	0.407514187	1
ENSBTAG00000006659	*BT.53553*	20	10	14	17	12	-0.585	0.883959502	1
ENSBTAG00000006661	*RALA*	1'074	1'212	1'165	930	1'399	0.589	0.470254314	1
ENSBTAG00000006662	*BT.56101*	709	586	645	614	677	0.140	0.871244225	1
ENSBTAG00000006663	*CYTH1*	1'021	900	962	884	1'039	0.233	0.778201568	1
ENSBTAG00000006665	*ERP29*	6'058	5'676	5'900	5'246	6'554	0.321	0.676053981	1
ENSBTAG00000006666	*NAA25*	784	489	622	679	565	-0.266	0.759758081	1
ENSBTAG00000006667	*BT.87209*	926	944	946	802	1'090	0.443	0.592931267	1

Ensembl gene ID	geneName	counts wildtype horn bud	counts polled horn bud	baseMean	baseMean wildtype horn bud	baseMean polled horn bud	log2FoldChange	pval	padj
ENSBTAG00000006670	*ERGIC3*	6'753	6'199	6'503	5'848	7'158	0.292	0.703883615	1
ENSBTAG00000006671	*DDX27*	1'906	1'316	1'585	1'651	1'520	-0.119	0.882806895	1
ENSBTAG00000006672	*SYNCRIP*	4'421	4'218	4'350	3'829	4'871	0.347	0.653184777	1
ENSBTAG00000006673	*TMEM179*	40	7	21	35	8	-2.100	0.456712191	1
ENSBTAG00000006675	*PCSK6*	979	729	845	848	842	-0.010	0.992486174	1
ENSBTAG00000006676	*FIBIN*	4'454	1'902	3'027	3'857	2'196	-0.813	0.30049309	1
ENSBTAG00000006677	*pseudogene*	44	28	35	38	32	-0.237	0.922699543	1
ENSBTAG00000006678	*GATAD2B*	586	783	706	507	904	0.833	0.329389895	1
ENSBTAG00000006679	*BT.29882*	328	561	466	284	648	1.189	0.18838913	1
ENSBTAG00000006680	*NKIRAS1*	1'048	704	860	908	813	-0.159	0.85080564	1
ENSBTAG00000006683	*ZFYVE28*	17	4	10	15	5	-1.672	0.733083131	1
ENSBTAG00000006685	*IL10*	8	7	8	7	8	0.222	0.99532173	1
ENSBTAG00000006686	*NPNT*	2'146	1'587	1'846	1'858	1'833	-0.020	0.981066438	1
ENSBTAG00000006689	*TECTB*	3	0	1	3	0		0.936647693	1
ENSBTAG00000006690	*ARCN1*	9'340	8'003	8'665	8'089	9'241	0.192	0.801256602	1
ENSBTAG00000006691	*GABRA3*	483	134	287	418	155	-1.435	0.147222628	1
ENSBTAG00000006692	*IL19*	1	0	0	1	0		1	1
ENSBTAG00000006693	*MTX2*	1'832	1'160	1'463	1'587	1'339	-0.244	0.762397328	1
ENSBTAG00000006694	*CXCL14*	36'125	36'239	36'565	31'285	41'845	0.420	0.580582893	1
ENSBTAG00000006695	*VCPIP1*	728	429	563	630	495	-0.348	0.69209708	1
ENSBTAG00000006697	*RICTOR*	3'147	1'212	2'062	2'725	1'399	-0.962	0.227532324	1
ENSBTAG00000006702	*EIF3E*	34'247	31'063	32'764	29'659	35'868	0.274	0.717590309	1
ENSBTAG00000006703	*PTGDR*	201	60	122	174	69	-1.329	0.282500274	1
ENSBTAG00000006704	*BT.54401*	2	0	1	2	0		0.974934741	1
ENSBTAG00000006707	*ACSL5*	1'159	371	716	1'004	428	-1.228	0.153182441	1
ENSBTAG00000006708	*ARIH1*	1'894	1'212	1'520	1'640	1'399	-0.229	0.776431986	1
ENSBTAG00000006712	*KIAA0368*	5'336	4'403	4'853	4'621	5'084	0.138	0.857844087	1
ENSBTAG00000006713	*ZDHHC6*	1'934	1'463	1'682	1'675	1'689	0.012	0.987781963	1
ENSBTAG00000006714	*VTI1A*	196	156	175	170	180	0.086	0.942217308	1
ENSBTAG00000006715	*FBXO8*	1'146	722	913	992	834	-0.251	0.763526027	1
ENSBTAG00000006716	*PTGS1*	1'091	649	847	945	749	-0.334	0.690631615	1
ENSBTAG00000006718	*NVL*	1'729	1'465	1'594	1'497	1'692	0.176	0.825760581	1
ENSBTAG00000006719	*BT.45827*	283	256	270	245	296	0.270	0.78568469	1
ENSBTAG00000006720	*BT.103132*	618	450	527	535	520	-0.043	0.964471926	1
ENSBTAG00000006721	*TWISTNB*	550	462	505	476	533	0.163	0.854654258	1
ENSBTAG00000006722	*FGF16*	38	3	18	33	3	-3.248	0.322153449	1
ENSBTAG00000006724	*PPP1R2*	1'327	838	1'058	1'149	968	-0.248	0.763837135	1
ENSBTAG00000006726	*CCDC102B*	69	12	37	60	14	-2.109	0.311053786	1
ENSBTAG00000006727	*pseudogene*	1	1	1	1	1	0.415	1	1
ENSBTAG00000006729	*ARID5B*	491	796	672	425	919	1.112	0.197310722	1
ENSBTAG00000006730	*BT.53247*	1'148	720	913	994	831	-0.258	0.757545033	1
ENSBTAG00000006731	*LAT*	1'601	1'597	1'615	1'387	1'844	0.411	0.606795926	1
ENSBTAG00000006732	*NRCAM*	136	13	66	118	15	-2.972	0.070353782	1
ENSBTAG00000006734	*BICD1*	62	38	49	54	44	-0.291	0.879262475	1
ENSBTAG00000006735	*BT.32379*	812	298	524	703	344	-1.031	0.245992434	1
ENSBTAG00000006739	*KRT81*	7	3	5	6	3	-0.807	0.947428618	1
ENSBTAG00000006740	*BT.91117*	3'259	4'518	4'020	2'822	5'217	0.886	0.255394853	1
ENSBTAG00000006742	*BT.87971*	58	1	26	50	1	-5.443	0.082423998	1
ENSBTAG00000006743	*RBBP8*	2'109	1'422	1'734	1'826	1'642	-0.154	0.848350688	1
ENSBTAG00000006744	*TMEM131*	3'754	2'378	2'998	3'251	2'746	-0.244	0.755804365	1
ENSBTAG00000006745	*FGG*	3	0	1	3	0		0.936647693	1
ENSBTAG00000006747	*LTBP3*	19'179	26'252	23'461	16'610	30'313	0.868	0.256609491	1
ENSBTAG00000006748	*DMXL1*	1'672	749	1'156	1'448	865	-0.743	0.36431517	1
ENSBTAG00000006751	*PAPD4*	1'599	775	1'140	1'385	895	-0.630	0.442205891	1
ENSBTAG00000006752	*PFKFB4*	285	469	394	247	542	1.134	0.221081568	1
ENSBTAG00000006754	*DBP*	796	587	684	689	678	-0.024	0.979893666	1
ENSBTAG00000006755	*SMAP*	10'709	6'841	8'587	9'274	7'899	-0.232	0.76311054	1
ENSBTAG00000006756	*BT.71626*	25'206	17'351	20'932	21'829	20'035	-0.124	0.87153258	1
ENSBTAG00000006757	*CA11*	411	192	289	356	222	-0.683	0.484919576	1
ENSBTAG00000006758	*pseudogene*	32	30	31	28	35	0.322	0.895053951	1
ENSBTAG00000006759	*BT.91195*	2'645	2'339	2'496	2'291	2'701	0.238	0.761728922	1
ENSBTAG00000006760	*OTX1*	215	233	228	186	269	0.531	0.605195683	1
ENSBTAG00000006762	*SH3RF2*	574	545	563	497	629	0.340	0.697177616	1
ENSBTAG00000006765	*LARS*	2'875	2'380	2'619	2'490	2'748	0.142	0.855465521	1
ENSBTAG00000006767	*MRPL13*	1'443	958	1'178	1'250	1'106	-0.176	0.830463154	1
ENSBTAG00000006768	*BT.29990*	436	291	357	378	336	-0.168	0.860440456	1
ENSBTAG00000006769	*HSD3B*	8	6	7	7	7	0.000	1	1
ENSBTAG00000006770	*MTBP*	655	572	614	567	660	0.220	0.800034427	1
ENSBTAG00000006771	*FRY*	1'929	1'175	1'514	1'671	1'357	-0.300	0.709421818	1
ENSBTAG00000006775	*SLC12A7*	2'000	1'406	1'678	1'732	1'624	-0.093	0.908234068	1
ENSBTAG00000006776	*KIAA0930*	468	423	447	405	488	0.269	0.76604131	1
ENSBTAG00000006777	*TLCD1*	254	218	236	220	252	0.195	0.850794592	1

Ensembl gene ID	geneName	counts wildtype horn bud	counts polled horn bud	baseMean	baseMean wildtype horn bud	baseMean polled horn bud	log2FoldChange	pval	padj
ENSBTAG00000006779	*LDHD*	22	6	13	19	7	-1.459	0.70932461	1
ENSBTAG00000006780	*BT.33843*	61	16	36	53	18	-1.516	0.467470811	1
ENSBTAG00000006784	*BT.103342*	610	746	695	528	861	0.705	0.408957621	1
ENSBTAG00000006785	*ARF2*	848	897	885	734	1'036	0.496	0.551594932	1
ENSBTAG00000006786	*MED6*	1'327	711	985	1'149	821	-0.485	0.557993587	1
ENSBTAG00000006789	*FGD5*	2'143	1'414	1'744	1'856	1'633	-0.185	0.817684345	1
ENSBTAG00000006790	*SPTBN5*	1'022	620	800	885	716	-0.306	0.717245463	1
ENSBTAG00000006792	*EHD4*	645	589	619	559	680	0.284	0.742564211	1
ENSBTAG00000006795	*GCSH*	1'074	824	941	930	951	0.033	0.969159839	1
ENSBTAG00000006797	*Mrz 03*	666	368	501	577	425	-0.441	0.620882724	1
ENSBTAG00000006800	*FGF6*	1	2	2	1	2	1.415	0.981979269	1
ENSBTAG00000006801	*TMEM106A*	899	675	779	779	779	0.002	0.999572072	1
ENSBTAG00000006804	*KIRREL3*	101	30	61	87	35	-1.336	0.406070553	1
ENSBTAG00000006805	*ZNF180*	549	419	480	475	484	0.025	0.979392577	1
ENSBTAG00000006807	*BT.54548*	20	15	17	17	17	0.000	1	1
ENSBTAG00000006812	*FAM181B*	194	71	125	168	82	-1.035	0.396509026	1
ENSBTAG00000006813	*pseudogene*	1'611	2'264	2'005	1'395	2'614	0.906	0.255171514	1
ENSBTAG00000006815	*processed_pseudogene*	3'732	3'978	3'913	3'232	4'593	0.507	0.51326605	1
ENSBTAG00000006816	*FAM108B1*	1'331	849	1'067	1'153	980	-0.234	0.777210789	1
ENSBTAG00000006817	*CBL*	91	163	134	79	188	1.256	0.292936471	1
ENSBTAG00000006818	*TMEM185A*	987	1'024	1'019	855	1'182	0.468	0.569737053	1
ENSBTAG00000006819	*CTAGE5*	2'521	1'042	1'693	2'183	1'203	-0.860	0.284196292	1
ENSBTAG00000006820	*FATE1*	3	1	2	3	1	-1.170	0.985253077	1
ENSBTAG00000006821	*PECR*	577	517	548	500	597	0.257	0.77021213	1
ENSBTAG00000006823	*CMYA5*	330	328	332	286	379	0.406	0.668646562	1
ENSBTAG00000006824	*APPL1*	1'435	1'134	1'276	1'243	1'309	0.075	0.926100696	1
ENSBTAG00000006828	*BT.96694*	1'644	1'680	1'682	1'424	1'940	0.446	0.575984991	1
ENSBTAG00000006831	*C8H9ORF85*	17	21	19	15	24	0.720	0.811878868	1
ENSBTAG00000006832	*BT.29369*	873	867	879	756	1'001	0.405	0.62694573	1
ENSBTAG00000006833	*LMAN1*	3'129	1'678	2'324	2'710	1'938	-0.484	0.539833708	1
ENSBTAG00000006835	*MCAM*	6'087	3'918	4'898	5'271	4'524	-0.221	0.775802595	1
ENSBTAG00000006836	*FBXO33*	447	209	314	387	241	-0.682	0.478396197	1
ENSBTAG00000006837	*BT.37042*	3'109	1'660	2'305	2'692	1'917	-0.490	0.534695261	1
ENSBTAG00000006838	*AIFM1*	1'199	762	959	1'038	880	-0.239	0.774171077	1
ENSBTAG00000006839	*JOSD2*	582	599	598	504	692	0.457	0.598682361	1
ENSBTAG00000006840	*ASPDH*	4	0	2	3	0		0.89545886	1
ENSBTAG00000006843	*UTP15*	1'570	957	1'232	1'360	1'105	-0.299	0.71385237	1
ENSBTAG00000006844	*LEF1*	1'562	1'743	1'683	1'353	2'013	0.573	0.473056663	1
ENSBTAG00000006846	*LGALS9*	1'020	427	688	883	493	-0.841	0.326951824	1
ENSBTAG00000006848	*DHX34*	456	594	540	395	686	0.796	0.365749421	1
ENSBTAG00000006851	*BT.30066*	125	15	63	108	17	-2.644	0.110296954	1
ENSBTAG00000006852	*ACYP2*	117	59	85	101	68	-0.573	0.685031147	1
ENSBTAG00000006853	*IFNK*	0	6	3	0	7	Inf	0.700309343	1
ENSBTAG00000006859	*protein_coding*	11	6	8	10	7	-0.459	0.954298496	1
ENSBTAG00000006860	*BT.62819*	356	193	266	308	223	-0.468	0.638686359	1
ENSBTAG00000006861	*C11H2orf73*	16	4	9	14	5	-1.585	0.756102726	1
ENSBTAG00000006862	*MEIS3*	3'826	2'818	3'284	3'313	3'254	-0.026	0.974340788	1
ENSBTAG00000006864	*protein_coding*	682	275	454	591	318	-0.895	0.32249515	1
ENSBTAG00000006868	*OTUD7A*	11	7	9	10	8	-0.237	0.988863286	1
ENSBTAG00000006869	*PHF20L1*	1'172	749	940	1'015	865	-0.231	0.782004214	1
ENSBTAG00000006870	*RASGEF1A*	236	97	158	204	112	-0.868	0.444353537	1
ENSBTAG00000006872	*processed_pseudogene*	327	219	268	283	253	-0.163	0.872792786	1
ENSBTAG00000006876	*PMPCB*	1'592	808	1'156	1'379	933	-0.563	0.491287023	1
ENSBTAG00000006877	*MMP-16*	12'804	3'649	7'651	11'089	4'214	-1.396	0.073341967	1
ENSBTAG00000006878	*BT.105107*	1'564	1'089	1'306	1'354	1'257	-0.107	0.896145845	1
ENSBTAG00000006881	*SLC35B1*	1'690	1'343	1'507	1'464	1'551	0.083	0.917235269	1
ENSBTAG00000006882	*IQGAP3*	724	922	846	627	1'065	0.764	0.362434424	1
ENSBTAG00000006883	*BT.77209*	12'811	22'248	18'392	11'095	25'690	1.211	0.116127312	1
ENSBTAG00000006884	*BT.63774*	10'166	4'018	6'722	8'804	4'640	-0.924	0.232283242	1
ENSBTAG00000006886	*FAM117A*	2'028	1'553	1'775	1'756	1'793	0.030	0.969972033	1
ENSBTAG00000006893	*BT.76404*	337	397	375	292	458	0.651	0.482854867	1
ENSBTAG00000006894	*BT.23126*	175	108	138	152	125	-0.281	0.815925049	1
ENSBTAG00000006896	*BT.62468*	444	154	281	385	178	-1.113	0.25976165	1
ENSBTAG00000006898	*EPRS*	10'011	8'536	9'263	8'670	9'857	0.185	0.808271855	1
ENSBTAG00000006899	*JPH3*	76	5	36	66	6	-3.511	0.119724572	1
ENSBTAG00000006901	*BT.66996*	3'075	2'069	2'526	2'663	2'389	-0.157	0.842828416	1
ENSBTAG00000006902	*KCNJ5*	2	0	1	2	0		0.974934741	1
ENSBTAG00000006903	*protein_coding*	0	1	1	0	1	Inf	0.993540919	1
ENSBTAG00000006904	*TENC1*	8'386	4'957	6'493	7'262	5'724	-0.343	0.655654109	1
ENSBTAG00000006905	*KLHDC4*	1'345	1'419	1'402	1'165	1'639	0.492	0.541404204	1
ENSBTAG00000006909	*PIK3CB*	732	339	513	634	391	-0.696	0.433486048	1
ENSBTAG00000006910	*ASRGL1*	1'417	929	1'150	1'227	1'073	-0.194	0.813453843	1

Ensembl gene ID	geneName	counts wildtype horn bud	counts polled horn bud	baseMean	baseMean wildtype horn bud	baseMean polled horn bud	log2FoldChange	pval	padj
ENSBTAG00000006911	*NUP107*	2'478	1'594	1'993	2'146	1'841	-0.221	0.780726208	1
ENSBTAG00000006912	*CCDC33*	3	0	1	3	0		0.936647693	1
ENSBTAG00000006914	*BT.34383*	997	1'172	1'108	863	1'353	0.648	0.428935997	1
ENSBTAG00000006916	*TAF15*	4'273	2'253	3'151	3'701	2'602	-0.508	0.515506082	1
ENSBTAG00000006918	*TLK1*	2'774	1'478	2'055	2'402	1'707	-0.493	0.534003322	1
ENSBTAG00000006919	*SMAD4*	5'786	4'503	5'105	5'011	5'200	0.053	0.944394617	1
ENSBTAG00000006920	*ABCF3*	2'276	2'563	2'465	1'971	2'959	0.586	0.455930685	1
ENSBTAG00000006921	*ABCA6*	519	480	502	449	554	0.302	0.733649047	1
ENSBTAG00000006925	*CENPL*	1'091	731	894	945	844	-0.163	0.84674206	1
ENSBTAG00000006927	*ABCF1*	2'668	2'410	2'547	2'311	2'783	0.268	0.731982318	1
ENSBTAG00000006928	*BT.49448*	15'448	12'411	13'855	13'378	14'331	0.099	0.895847837	1
ENSBTAG00000006933	*BT.27784*	1'979	2'427	2'258	1'714	2'802	0.709	0.369261213	1
ENSBTAG00000006934	*BT.104995*	52	10	28	45	12	-1.963	0.410438585	1
ENSBTAG00000006936	*BT.88532*	661	545	601	572	629	0.137	0.875611639	1
ENSBTAG00000006937	*ABCA10*	122	46	79	106	53	-0.992	0.490250179	1
ENSBTAG00000006938	*PPM1F*	401	522	475	347	603	0.795	0.374662384	1
ENSBTAG00000006939	*FGD3*	365	396	387	316	457	0.533	0.564018965	1
ENSBTAG00000006940	*USP48*	4'564	2'475	3'405	3'953	2'858	-0.468	0.54865466	1
ENSBTAG00000006941	*BT.25712*	2'309	1'713	1'989	2'000	1'978	-0.016	0.985592641	1
ENSBTAG00000006943	*BT.56213*	10	0	4	9	0		0.653360153	1
ENSBTAG00000006945	*BT.43337*	726	347	515	629	401	-0.650	0.463853488	1
ENSBTAG00000006947	*BT.53546*	7	0	3	6	0		0.769774961	1
ENSBTAG00000006948	*BT.26841*	1'534	982	1'231	1'328	1'134	-0.228	0.779803385	1
ENSBTAG00000006949	*BT.24857*	67	22	42	58	25	-1.192	0.53496564	1
ENSBTAG00000006950	*COPS4*	4'042	3'033	3'501	3'500	3'502	0.001	0.999016147	1
ENSBTAG00000006951	*LMO2*	1'814	1'131	1'438	1'571	1'306	-0.267	0.741591379	1
ENSBTAG00000006954	*ZNF432*	734	357	524	636	412	-0.625	0.48026264	1
ENSBTAG00000006955	*CPNE1*	2'840	2'611	2'737	2'460	3'015	0.294	0.70707049	1
ENSBTAG00000006957	*SLC2A8*	480	533	516	416	615	0.566	0.522039598	1
ENSBTAG00000006958	*BT.24727*	604	388	486	523	448	-0.223	0.804265381	1
ENSBTAG00000006960	*DHX16*	2'595	2'408	2'514	2'247	2'781	0.307	0.6952269	1
ENSBTAG00000006961	*NLRP13*	5	2	3	4	2	-0.907	0.965043111	1
ENSBTAG00000006962	*NFS1*	1'450	892	1'143	1'256	1'030	-0.286	0.72743649	1
ENSBTAG00000006966	*NRM*	1'338	1'948	1'704	1'159	2'249	0.957	0.233175947	1
ENSBTAG00000006969	*TUBB5*	44'039	41'785	43'194	38'139	48'249	0.339	0.654643447	1
ENSBTAG00000006970	*OLA1*	956	887	926	828	1'024	0.307	0.711401575	1
ENSBTAG00000006971	*BRD9*	3'358	3'783	3'638	2'908	4'368	0.587	0.45040988	1
ENSBTAG00000006972	*TRIP13*	513	450	482	444	520	0.226	0.800773599	1
ENSBTAG00000006974	*PLEKHA7*	496	510	509	430	589	0.455	0.607263058	1
ENSBTAG00000006977	*PLP1*	2'722	1'336	1'950	2'357	1'543	-0.612	0.441872053	1
ENSBTAG00000006978	*HSD17B4*	4'686	3'936	4'302	4'058	4'545	0.163	0.832234816	1
ENSBTAG00000006982	*RAB5C*	5'252	5'686	5'557	4'548	6'566	0.530	0.492035019	1
ENSBTAG00000006984	*BT.91089*	3'218	3'837	3'609	2'787	4'431	0.669	0.390351071	1
ENSBTAG00000006985	*TM7SF3*	3'929	2'720	3'272	3'403	3'141	-0.116	0.88303691	1
ENSBTAG00000006987	*LASS3*	1'251	600	888	1'083	693	-0.645	0.44030214	1
ENSBTAG00000006988	*C2ORF65*	11	8	9	10	9	-0.044	1	1
ENSBTAG00000006989	*SEMA4F*	1'010	413	676	875	477	-0.875	0.30898663	1
ENSBTAG00000006991	*ADH6*	6	6	6	5	7	0.415	0.976119138	1
ENSBTAG00000006995	*SPTBN1*	28'542	17'952	22'724	24'718	20'729	-0.254	0.739229251	1
ENSBTAG00000006998	*RASGRP4*	306	200	248	265	231	-0.198	0.847474973	1
ENSBTAG00000007000	*ENOSF1*	387	210	289	335	242	-0.467	0.633613926	1
ENSBTAG00000007001	*SLC26A1*	32	42	38	28	48	0.807	0.687347776	1
ENSBTAG00000007002	*IDUA*	2'078	2'227	2'186	1'800	2'572	0.515	0.514166559	1
ENSBTAG00000007003	*TYMS*	3'055	2'196	2'591	2'646	2'536	-0.061	0.938891619	1
ENSBTAG00000007007	*WDR20*	1'206	960	1'076	1'044	1'109	0.086	0.916912511	1
ENSBTAG00000007008	*MOK*	260	149	199	225	172	-0.388	0.717495254	1
ENSBTAG00000007010	*pseudogene*	0	2	1	0	2	Inf	0.939077559	1
ENSBTAG00000007011	*C10ORF122*	27	5	15	23	6	-2.018	0.576089911	1
ENSBTAG00000007012	*ZNF3*	2'552	2'004	2'262	2'210	2'314	0.066	0.932789423	1
ENSBTAG00000007013	*BT.42415*	1'133	728	911	981	841	-0.223	0.789826747	1
ENSBTAG00000007014	*CEP63*	711	298	480	616	344	-0.839	0.349428062	1
ENSBTAG00000007015	*BT.26976*	919	514	695	796	594	-0.423	0.620977099	1
ENSBTAG00000007016	*CNPY4*	4'289	2'971	3'572	3'714	3'431	-0.115	0.883621582	1
ENSBTAG00000007019	*LRIT3*	8	5	6	7	6	-0.263	0.996869191	1
ENSBTAG00000007020	*BT.41431*	382	248	309	331	286	-0.208	0.831742787	1
ENSBTAG00000007022	*protein_coding*	18	12	15	16	14	-0.170	0.983826208	1
ENSBTAG00000007024	*pseudogene*	2'693	5'800	4'515	2'332	6'697	1.522	0.053482404	1
ENSBTAG00000007031	*BT.21584*	181	175	179	157	202	0.366	0.738332104	1
ENSBTAG00000007035	*CCER1*	1	0	0	1	0		1	1
ENSBTAG00000007039	*FBXO25*	1'145	1'135	1'151	992	1'311	0.402	0.621886051	1
ENSBTAG00000007044	*CALHM1*	1	0	0	1	0		1	1
ENSBTAG00000007047	*FZD10*	1'671	2'236	2'015	1'447	2'582	0.835	0.293505334	1

Ensembl gene ID	geneName	counts wildtype horn bud	counts polled horn bud	baseMean	baseMean wildtype horn bud	baseMean polled horn bud	log2FoldChange	pval	padj
ENSBTAG00000007049	*MBL*	1	0	0	1	0		1	1
ENSBTAG00000007051	*CLDN23*	105	42	70	91	48	-0.907	0.550014133	1
ENSBTAG00000007052	*BT.62541*	63	47	54	55	54	-0.008	1	1
ENSBTAG00000007053	*ZFHX2*	218	284	258	189	328	0.797	0.424912885	1
ENSBTAG00000007058	*ATG2A*	143	318	246	124	367	1.568	0.125582547	1
ENSBTAG00000007061	*PPP2R5B*	2'192	1'951	2'076	1'898	2'253	0.247	0.754373439	1
ENSBTAG00000007062	*IGFBP5*	6'661	1'855	3'955	5'769	2'142	-1.429	0.06990814	1
ENSBTAG00000007063	*GPHA2*	10	1	5	9	1	-2.907	0.754028015	1
ENSBTAG00000007065	*TRAPPC6A*	351	231	285	304	267	-0.189	0.850434389	1
ENSBTAG00000007066	*TMTC4*	2'878	1'218	1'949	2'492	1'406	-0.826	0.300396972	1
ENSBTAG00000007067	*ZC3H13*	4'844	3'554	4'149	4'195	4'104	-0.032	0.968335459	1
ENSBTAG00000007068	*SH3BGR*	851	2'011	1'530	737	2'322	1.656	0.043476915	1
ENSBTAG00000007070	*BLOC1S3*	38	46	43	33	53	0.691	0.715410825	1
ENSBTAG00000007071	*RAI14*	3'876	1'751	2'689	3'357	2'022	-0.731	0.352630902	1
ENSBTAG00000007073	*CPB2*	3	0	1	3	0		0.936647693	1
ENSBTAG00000007074	*BT.100182*	783	432	588	678	499	-0.443	0.611662034	1
ENSBTAG00000007075	*BT.65666*	97	199	157	84	230	1.452	0.203976004	1
ENSBTAG00000007079	*BT.49547*	687	376	515	595	434	-0.455	0.60863342	1
ENSBTAG00000007080	*PREB*	2'513	1'607	2'016	2'176	1'856	-0.230	0.772366856	1
ENSBTAG00000007083	*C17ORF46*	3	0	1	3	0		0.936647693	1
ENSBTAG00000007084	*BT.48108*	898	1'330	1'157	778	1'536	0.982	0.231862575	1
ENSBTAG00000007089	*FRG1*	1'903	961	1'379	1'648	1'110	-0.571	0.480783085	1
ENSBTAG00000007092	*BT.106521*	4	2	3	3	2	-0.585	0.996239345	1
ENSBTAG00000007093	*DDX11*	987	1'504	1'296	855	1'737	1.023	0.209909626	1
ENSBTAG00000007094	*KIAA1024*	25	14	19	22	16	-0.421	0.90258764	1
ENSBTAG00000007096	*BT.51458*	1'147	1'043	1'099	993	1'204	0.278	0.734204685	1
ENSBTAG00000007097	*THTPA*	684	575	628	592	664	0.165	0.849296082	1
ENSBTAG00000007099	*AP1G2*	2'030	2'175	2'135	1'758	2'511	0.515	0.514854477	1
ENSBTAG00000007100	*TRAPPC10*	621	543	582	538	627	0.221	0.79969071	1
ENSBTAG00000007101	*F3*	6'935	1'900	4'100	6'006	2'194	-1.453	0.065360949	1
ENSBTAG00000007102	*BT.91314*	730	722	733	632	834	0.399	0.637867989	1
ENSBTAG00000007103	*BT.64660*	102	76	88	88	88	-0.009	1	1
ENSBTAG00000007105	*PWP2*	1'013	1'565	1'342	877	1'807	1.043	0.200403454	1
ENSBTAG00000007106	*ES1*	1'596	1'565	1'595	1'382	1'807	0.387	0.628725593	1
ENSBTAG00000007107	*TMEM143*	357	355	360	309	410	0.407	0.663597374	1
ENSBTAG00000007108	*LGALS12*	2	1	1	2	1	-0.585	1	1
ENSBTAG00000007109	*BT.44353*	95	182	146	82	210	1.353	0.245077461	1
ENSBTAG00000007110	*RCOR3*	1'602	982	1'261	1'387	1'134	-0.291	0.720898366	1
ENSBTAG00000007111	*STEAP3*	114	128	123	99	148	0.582	0.633814119	1
ENSBTAG00000007112	*C3H1ORF41*	518	354	429	449	409	-0.134	0.885473223	1
ENSBTAG00000007113	*TRRAP*	5'107	6'148	5'761	4'423	7'099	0.683	0.376424064	1
ENSBTAG00000007114	*MGC157319*	870	455	639	753	525	-0.520	0.546973342	1
ENSBTAG00000007115	*GSR*	569	618	603	493	714	0.534	0.537681537	1
ENSBTAG00000007116	*ARRDC3*	2'304	2'711	2'563	1'995	3'130	0.650	0.40847636	1
ENSBTAG00000007117	*CTC1*	1'620	2'087	1'906	1'403	2'410	0.780	0.327044456	1
ENSBTAG00000007118	*SMURF1*	897	1'225	1'096	777	1'415	0.865	0.293226408	1
ENSBTAG00000007120	*ARFGEF2*	744	552	641	644	637	-0.016	0.988351372	1
ENSBTAG00000007121	*TK1*	688	1'373	1'091	596	1'585	1.412	0.08968104	1
ENSBTAG00000007122	*ZFP2*	1'023	539	754	886	622	-0.509	0.54820321	1
ENSBTAG00000007123	*ENSA*	6'087	5'897	6'040	5'271	6'809	0.369	0.630843774	1
ENSBTAG00000007125	*AFMID*	151	119	134	131	137	0.071	0.957814124	1
ENSBTAG00000007127	*processed_pseudogene*	2'337	4'144	3'404	2'024	4'785	1.241	0.115014959	1
ENSBTAG00000007128	*ATP13A1*	2'601	3'960	3'413	2'253	4'573	1.021	0.192665301	1
ENSBTAG00000007129	*MRVI1*	535	183	337	463	211	-1.133	0.234856605	1
ENSBTAG00000007130	*ESF1*	1'836	708	1'204	1'590	818	-0.960	0.241627456	1
ENSBTAG00000007131	*BT.54500*	49	56	54	42	65	0.608	0.722034383	1
ENSBTAG00000007133	*CUL2*	2'027	1'175	1'556	1'755	1'357	-0.372	0.6438033	1
ENSBTAG00000007136	*ADAM22*	191	94	137	165	109	-0.608	0.608643431	1
ENSBTAG00000007137	*RFC5*	4'818	2'823	3'716	4'173	3'260	-0.356	0.647203378	1
ENSBTAG00000007139	*WSB2*	2'645	1'851	2'214	2'291	2'137	-0.100	0.900327849	1
ENSBTAG00000007141	*GULP1*	996	459	696	863	530	-0.703	0.411736	1
ENSBTAG00000007142	*CPPED1*	350	329	342	303	380	0.326	0.730520006	1
ENSBTAG00000007144	*KRT83*	1	1	1	1	1	0.415	1	1
ENSBTAG00000007145	*KRT82*	1	0	0	1	0		1	1
ENSBTAG00000007146	*CPVL*	6	3	4	5	3	-0.585	0.976586913	1
ENSBTAG00000007147	*BT.62349*	3'264	3'625	3'506	2'827	4'186	0.566	0.466747575	1
ENSBTAG00000007148	*F2*	51	72	64	44	83	0.913	0.560133448	1
ENSBTAG00000007149	*NYNRIN*	3'695	7'174	5'742	3'200	8'284	1.372	0.079281255	1
ENSBTAG00000007152	*OS9*	8'995	8'013	8'521	7'790	9'253	0.248	0.745264334	1
ENSBTAG00000007153	*C1QA*	1'426	1'641	1'565	1'235	1'895	0.618	0.441349399	1
ENSBTAG00000007156	*AGAP2*	696	260	451	603	300	-1.006	0.267607958	1
ENSBTAG00000007158	*TSPAN31*	4'745	2'778	3'659	4'109	3'208	-0.357	0.646259583	1

Ensembl gene ID	geneName	counts wildtype horn bud	counts polled horn bud	baseMean	baseMean wildtype horn bud	baseMean polled horn bud	log2FoldChange	pval	padj
ENSBTAG00000007159	*BT.32558*	4	2	3	3	2	-0.585	0.996239345	1
ENSBTAG00000007160	*CDK4*	3'785	5'094	4'580	3'278	5'882	0.844	0.277438345	1
ENSBTAG00000007163	*SLC44A1*	2'564	1'044	1'713	2'220	1'206	-0.881	0.272122398	1
ENSBTAG00000007164	*FGFR3*	2'468	2'148	2'309	2'137	2'480	0.215	0.784735631	1
ENSBTAG00000007166	*CAMKK1*	237	506	395	205	584	1.509	0.106114745	1
ENSBTAG00000007167	*ZMYM5*	2'533	1'266	1'828	2'194	1'462	-0.586	0.462864767	1
ENSBTAG00000007169	*P2RX1*	9	15	13	8	17	1.152	0.772410391	1
ENSBTAG00000007170	*SLC38A7*	815	984	921	706	1'136	0.687	0.4089062	1
ENSBTAG00000007172	*GOT2*	4'179	4'209	4'240	3'619	4'860	0.425	0.582489495	1
ENSBTAG00000007173	*BT.67124*	19'453	18'417	19'056	16'847	21'266	0.336	0.658500505	1
ENSBTAG00000007175	*AVPR1A*	682	1'000	873	591	1'155	0.967	0.248933322	1
ENSBTAG00000007176	*BT.46878*	471	278	364	408	321	-0.346	0.71294969	1
ENSBTAG00000007177	*EMP2*	1'356	1'750	1'598	1'174	2'021	0.783	0.32951245	1
ENSBTAG00000007181	*KIFC3*	1'397	1'162	1'276	1'210	1'342	0.149	0.853877313	1
ENSBTAG00000007184	*NUDT7*	710	751	741	615	867	0.496	0.558237269	1
ENSBTAG00000007186	*ARHGAP39*	1'036	1'308	1'204	897	1'510	0.751	0.357166315	1
ENSBTAG00000007187	*INF2*	2'549	2'949	2'806	2'207	3'405	0.625	0.424821801	1
ENSBTAG00000007189	*RCHY1*	1'631	928	1'242	1'412	1'072	-0.399	0.624666908	1
ENSBTAG00000007190	*THAP6*	160	179	173	139	207	0.577	0.600853407	1
ENSBTAG00000007191	*CCL5*	29	6	16	25	7	-1.858	0.581888091	1
ENSBTAG00000007192	*BT.20099*	2'515	3'461	3'087	2'178	3'996	0.876	0.264127034	1
ENSBTAG00000007193	*CCL16*	295	203	245	255	234	-0.124	0.906570332	1
ENSBTAG00000007195	*GUCA2A*	6	0	3	5	0		0.81115604	1
ENSBTAG00000007196	*TAGLN*	2'442	1'804	2'099	2'115	2'083	-0.022	0.979327791	1
ENSBTAG00000007199	*pseudogene*	2	3	3	2	3	1.000	0.97171833	1
ENSBTAG00000007200	*COX6B2*	69	94	84	60	109	0.861	0.53776249	1
ENSBTAG00000007201	*C9H6ORF35*	1'100	811	945	953	936	-0.025	0.978365685	1
ENSBTAG00000007202	*BT.33239*	2'176	2'196	2'210	1'884	2'536	0.428	0.587006894	1
ENSBTAG00000007203	*STT3A*	16'591	13'584	15'027	14'368	15'685	0.127	0.867513886	1
ENSBTAG00000007204	*KRT80*	2'705	2'690	2'724	2'343	3'106	0.407	0.602958648	1
ENSBTAG00000007206	*FAM40B*	473	168	302	410	194	-1.078	0.267652872	1
ENSBTAG00000007208	*BT.35872*	728	791	772	630	913	0.535	0.526326334	1
ENSBTAG00000007210	*BT.37907*	271	37	139	235	43	-2.458	0.045568279	1
ENSBTAG00000007211	*ASB12*	85	43	62	74	50	-0.568	0.72572557	1
ENSBTAG00000007213	*SIRPA*	7'812	5'764	6'711	6'765	6'656	-0.024	0.976388893	1
ENSBTAG00000007215	*RNPEPL1*	1'130	1'696	1'468	979	1'958	1.001	0.216290796	1
ENSBTAG00000007216	*DPF1*	563	136	322	488	157	-1.634	0.092812518	1
ENSBTAG00000007217	*KIAA0195*	2'175	3'533	2'982	1'884	4'080	1.115	0.157295932	1
ENSBTAG00000007220	*CASKIN2*	1'917	2'464	2'253	1'660	2'845	0.777	0.325810086	1
ENSBTAG00000007223	*BT.88141*	312	289	302	270	334	0.305	0.753426381	1
ENSBTAG00000007228	*CBX7*	98	98	99	85	113	0.415	0.7550013	1
ENSBTAG00000007230	*FANCF*	171	162	168	148	187	0.337	0.763534336	1
ENSBTAG00000007231	*SPATA9*	56	40	47	48	46	-0.070	0.982919613	1
ENSBTAG00000007233	*WDR25*	225	274	256	195	316	0.699	0.484409822	1
ENSBTAG00000007234	*PLRG1*	2'456	1'743	2'070	2'127	2'013	-0.080	0.92095343	1
ENSBTAG00000007235	*NTNG2*	860	613	726	745	708	-0.073	0.933405283	1
ENSBTAG00000007236	*PCM1*	8'334	4'181	6'023	7'217	4'828	-0.580	0.452241848	1
ENSBTAG00000007237	*BT.25661*	1'521	1'132	1'312	1'317	1'307	-0.011	0.99083732	1
ENSBTAG00000007239	*TSG-6*	2'048	1'528	1'769	1'774	1'764	-0.008	0.993972706	1
ENSBTAG00000007240	*ZCCHC4*	277	185	227	240	214	-0.167	0.874993705	1
ENSBTAG00000007241	*SLAMF9*	3	0	1	3	0		0.936647693	1
ENSBTAG00000007243	*CCDC147*	1	0	0	1	0		1	1
ENSBTAG00000007244	*NID1*	13'021	4'096	8'003	11'277	4'730	-1.254	0.10655608	1
ENSBTAG00000007245	*IFT140*	1'686	2'102	1'944	1'460	2'427	0.733	0.35648201	1
ENSBTAG00000007246	*TMEM204*	1'152	960	1'053	998	1'109	0.152	0.853358215	1
ENSBTAG00000007247	*NUF2*	995	590	771	862	681	-0.339	0.689229689	1
ENSBTAG00000007253	*BT.53698*	343	59	183	297	68	-2.124	0.058345098	1
ENSBTAG00000007256	*DYRK2*	1'210	1'041	1'125	1'048	1'202	0.198	0.80862076	1
ENSBTAG00000007259	*ELFN2*	456	117	265	395	135	-1.547	0.125039999	1
ENSBTAG00000007262	*ZNF536*	169	378	291	146	436	1.576	0.110543844	1
ENSBTAG00000007266	*BT.74315*	886	433	634	767	500	-0.618	0.474776129	1
ENSBTAG00000007268	*BT.19195*	5'266	5'182	5'272	4'560	5'984	0.392	0.610980829	1
ENSBTAG00000007269	*EIF2B1*	952	746	843	824	861	0.063	0.940453408	1
ENSBTAG00000007270	*BT.103768*	1'072	734	888	928	848	-0.131	0.876437011	1
ENSBTAG00000007271	*TCTN2*	1'829	825	1'268	1'584	953	-0.734	0.367798931	1
ENSBTAG00000007272	*ATP6V0A2*	1'813	1'162	1'456	1'570	1'342	-0.227	0.779149262	1
ENSBTAG00000007273	*TF*	42	5	21	36	6	-2.655	0.36039356	1
ENSBTAG00000007275	*TREM2*	191	92	136	165	106	-0.639	0.591261557	1
ENSBTAG00000007280	*BT.87198*	8	4	6	7	5	-0.585	0.95915875	1
ENSBTAG00000007281	*SEMA6C*	1'986	4'112	3'234	1'720	4'748	1.465	0.064652988	1
ENSBTAG00000007284	*CCNA1*	60	73	68	52	84	0.698	0.647506976	1
ENSBTAG00000007286	*pseudogene*	6	1	3	5	1	-2.170	0.885240772	1

Ensembl gene ID	geneName	counts wildtype horn bud	counts polled horn bud	baseMean	baseMean wildtype horn bud	baseMean polled horn bud	log2FoldChange	pval	padj
ENSBTAG00000007288	*CDK19*	514	615	578	445	710	0.674	0.439735498	1
ENSBTAG00000007295	*protein_coding*	1	0	0	1	0		1	1
ENSBTAG00000007296	*protein_coding*	678	967	852	587	1'117	0.927	0.269705008	1
ENSBTAG00000007298	*TEX29*	1	0	0	1	0		1	1
ENSBTAG00000007299	*SF3A3*	3'286	2'853	3'070	2'846	3'294	0.211	0.786166218	1
ENSBTAG00000007300	*FHL3*	1'291	1'339	1'332	1'118	1'546	0.468	0.562911361	1
ENSBTAG00000007302	*protein_coding*	7	5	6	6	6	-0.070	1	1
ENSBTAG00000007303	*RAD21*	12'090	10'184	11'115	10'470	11'759	0.168	0.825801505	1
ENSBTAG00000007304	*CLK3*	3'283	2'919	3'107	2'843	3'371	0.245	0.752465225	1
ENSBTAG00000007305	*VPS41*	3'100	2'603	2'845	2'685	3'006	0.163	0.834510268	1
ENSBTAG00000007306	*BT.91125*	10	1	5	9	1	-2.907	0.754028015	1
ENSBTAG00000007307	*SELE*	257	92	164	223	106	-1.067	0.342408757	1
ENSBTAG00000007308	*FLJ21128*	929	1'592	1'321	805	1'838	1.192	0.144735211	1
ENSBTAG00000007309	*ST6GALNAC5*	35	2	16	30	2	-3.714	0.305838928	1
ENSBTAG00000007312	*CD209*	28	95	67	24	110	2.178	0.165426079	1
ENSBTAG00000007315	*BT.59370*	1'745	2'195	2'023	1'511	2'535	0.746	0.347401339	1
ENSBTAG00000007316	*BT.93595*	581	165	347	503	191	-1.401	0.141902073	1
ENSBTAG00000007318	*ZNF687*	1'291	2'203	1'831	1'118	2'544	1.186	0.139939175	1
ENSBTAG00000007319	*JAG2*	2'221	4'634	3'637	1'923	5'351	1.476	0.061966032	1
ENSBTAG00000007320	*PI4KB*	4'968	6'362	5'824	4'302	7'346	0.772	0.31782501	1
ENSBTAG00000007321	*SREK1*	2'598	1'036	1'723	2'250	1'196	-0.911	0.256149619	1
ENSBTAG00000007323	*BT.27146*	2'323	1'607	1'934	2'012	1'856	-0.117	0.884319306	1
ENSBTAG00000007324	*LRRC4C*	528	282	391	457	326	-0.490	0.596479773	1
ENSBTAG00000007325	*TPSB1*	265	152	203	229	176	-0.387	0.717012393	1
ENSBTAG00000007329	*BT.31874*	250	144	191	217	166	-0.381	0.725356074	1
ENSBTAG00000007330	*BT.27527*	3'516	1'616	2'455	3'045	1'866	-0.706	0.370573674	1
ENSBTAG00000007331	*PLOD2*	9'680	3'973	6'485	8'383	4'588	-0.870	0.260793772	1
ENSBTAG00000007332	*ATP5L*	1'399	2'355	1'965	1'212	2'719	1.166	0.145216085	1
ENSBTAG00000007333	*AKNAD1*	110	36	68	95	42	-1.196	0.434126415	1
ENSBTAG00000007334	*NFKBID*	559	464	510	484	536	0.146	0.869786464	1
ENSBTAG00000007335	*TPD52*	1'317	1'001	1'148	1'141	1'156	0.019	0.981712018	1
ENSBTAG00000007336	*HCST*	15	3	8	13	3	-1.907	0.733459861	1
ENSBTAG00000007338	*TYROBP*	397	175	273	344	202	-0.767	0.438482763	1
ENSBTAG00000007340	*protein_coding*	1	0	0	1	0		1	1
ENSBTAG00000007343	*PDLIM7*	4'829	4'344	4'599	4'182	5'016	0.262	0.733746252	1
ENSBTAG00000007346	*COMMD4*	1'766	1'531	1'649	1'529	1'768	0.209	0.793368551	1
ENSBTAG00000007347	*BT.30826*	46	0	20	40	0		0.083327455	1
ENSBTAG00000007348	*STRA6*	126	53	85	109	61	-0.834	0.551156676	1
ENSBTAG00000007349	*PCDHB14*	150	83	113	130	96	-0.439	0.730923681	1
ENSBTAG00000007350	*BT.63172*	195	91	137	169	105	-0.684	0.563725575	1
ENSBTAG00000007351	*pseudogene*	4	1	2	3	1	-1.585	0.952247636	1
ENSBTAG00000007352	*KCNC4*	264	481	392	229	555	1.281	0.16855034	1
ENSBTAG00000007353	*DLX4*	135	82	106	117	95	-0.304	0.818542209	1
ENSBTAG00000007354	*processed_pseudogene*	443	960	746	384	1'109	1.531	0.075615303	1
ENSBTAG00000007356	*ELF1*	2'791	2'628	2'726	2'417	3'035	0.328	0.674683447	1
ENSBTAG00000007357	*BT.17704*	5'822	2'384	3'897	5'042	2'753	-0.873	0.26325549	1
ENSBTAG00000007359	*PROP1*	0	1	1	0	1	Inf	0.993540919	1
ENSBTAG00000007360	*TMEM43*	7'189	5'334	6'193	6'226	6'159	-0.016	0.984793354	1
ENSBTAG00000007361	*EHD1*	2'454	3'302	2'969	2'125	3'813	0.843	0.282501579	1
ENSBTAG00000007362	*XPC*	3'886	3'379	3'634	3'365	3'902	0.213	0.783070125	1
ENSBTAG00000007363	*LSM3*	2'920	1'864	2'341	2'529	2'152	-0.233	0.76855451	1
ENSBTAG00000007364	*PMS2*	306	324	320	265	374	0.497	0.60257951	1
ENSBTAG00000007365	*NEIL1*	474	432	455	410	499	0.281	0.755230025	1
ENSBTAG00000007366	*HICE1*	1'017	791	897	881	913	0.052	0.95039909	1
ENSBTAG00000007367	*AIMP2*	1'123	965	1'043	973	1'114	0.196	0.811332484	1
ENSBTAG00000007368	*BT.20028*	2'112	1'765	1'934	1'829	2'038	0.156	0.843707016	1
ENSBTAG00000007369	*PDLIM2*	765	578	665	663	667	0.011	0.99140223	1
ENSBTAG00000007370	*BT.88674*	2'720	2'120	2'402	2'356	2'448	0.055	0.943555394	1
ENSBTAG00000007371	*SCAMP1*	838	786	817	726	908	0.323	0.700625044	1
ENSBTAG00000007372	*ANKRD61*	23	36	31	20	42	1.061	0.635060227	1
ENSBTAG00000007374	*LHFPL2*	9'539	14'194	12'325	8'261	16'390	0.988	0.198866987	1
ENSBTAG00000007375	*MIF*	2'775	4'514	3'808	2'403	5'212	1.117	0.153990503	1
ENSBTAG00000007378	*CLIP4*	364	338	353	315	390	0.308	0.743229029	1
ENSBTAG00000007379	*ALK*	78	89	85	68	103	0.605	0.664699199	1
ENSBTAG00000007382	*SCAPER*	1'484	865	1'142	1'285	999	-0.364	0.657183155	1
ENSBTAG00000007384	*CYTH3*	1'397	893	1'120	1'210	1'031	-0.231	0.779279348	1
ENSBTAG00000007385	*C10H15ORF24*	2'276	1'214	1'686	1'971	1'402	-0.492	0.538966938	1
ENSBTAG00000007386	*BT.35837*	1'251	876	1'047	1'083	1'012	-0.099	0.905816251	1
ENSBTAG00000007387	*ENY2*	2'612	1'685	2'104	2'262	1'946	-0.217	0.784231581	1
ENSBTAG00000007388	*ZC3H12D*	14	3	8	12	3	-1.807	0.758305361	1
ENSBTAG00000007389	*IFI35*	608	733	686	527	846	0.685	0.423288893	1
ENSBTAG00000007390	*VAT1*	14'078	25'698	20'933	12'192	29'673	1.283	0.096345138	1

Ensembl gene ID	geneName	counts wildtype horn bud	counts polled horn bud	baseMean	baseMean wildtype horn bud	baseMean polled horn bud	log2FoldChange	pval	padj
ENSBTAG00000007393	*BT.34221*	484	625	570	419	722	0.784	0.370047316	1
ENSBTAG00000007394	*BT.69963*	2'314	2'511	2'452	2'004	2'899	0.533	0.497864441	1
ENSBTAG00000007395	*ALS2*	1'583	1'180	1'367	1'371	1'363	-0.009	0.993019933	1
ENSBTAG00000007397	*BT.40283*	734	386	541	636	446	-0.512	0.561261327	1
ENSBTAG00000007398	*MRPS24*	434	512	484	376	591	0.653	0.464099939	1
ENSBTAG00000007399	*BT.104573*	10'130	6'810	8'318	8'773	7'864	-0.158	0.837445689	1
ENSBTAG00000007402	*ZFAND2A*	246	238	244	213	275	0.367	0.71707383	1
ENSBTAG00000007403	*BT.63215*	45	25	34	39	29	-0.433	0.84851981	1
ENSBTAG00000007409	*BT.68917*	213	85	141	184	98	-0.910	0.437927427	1
ENSBTAG00000007413	*CEP41*	390	260	319	338	300	-0.170	0.862205679	1
ENSBTAG00000007414	*PAPOLG*	1'393	965	1'160	1'206	1'114	-0.115	0.889908297	1
ENSBTAG00000007415	*SLC7A8*	514	758	660	445	875	0.975	0.257874617	1
ENSBTAG00000007417	*GDF11*	232	277	260	201	320	0.671	0.500628215	1
ENSBTAG00000007421	*BT.64659*	2'372	1'303	1'779	2'054	1'505	-0.449	0.573561798	1
ENSBTAG00000007422	*BT.19916*	2'094	1'441	1'739	1'813	1'664	-0.124	0.877434525	1
ENSBTAG00000007423	*TNFRSF11B*	216	48	121	187	55	-1.755	0.160672871	1
ENSBTAG00000007424	*LIF*	149	36	85	129	42	-1.634	0.247005946	1
ENSBTAG00000007427	*PPRC1*	3'680	5'251	4'625	3'187	6'063	0.928	0.232759235	1
ENSBTAG00000007428	*CDKL5*	83	87	86	72	100	0.483	0.729693475	1
ENSBTAG00000007429	*SPNS2*	1'149	267	652	995	308	-1.690	0.054330242	1
ENSBTAG00000007430	*MYBBP1A*	1'580	3'514	2'713	1'368	4'058	1.568	0.049690874	1
ENSBTAG00000007431	*KIAA1199*	852	305	545	738	352	-1.067	0.227797105	1
ENSBTAG00000007433	*protein_coding*	1'105	676	869	957	781	-0.294	0.726015288	1
ENSBTAG00000007434	*CD300LG*	735	358	525	637	413	-0.623	0.481604348	1
ENSBTAG00000007435	*NOLC1*	2'778	2'121	2'427	2'406	2'449	0.026	0.973757408	1
ENSBTAG00000007436	*GGT6*	953	1'164	1'085	825	1'344	0.704	0.391700097	1
ENSBTAG00000007437	*TEKT1*	37	10	22	32	12	-1.472	0.593518052	1
ENSBTAG00000007438	*STT3B*	4'821	2'677	3'633	4'175	3'091	-0.434	0.577568073	1
ENSBTAG00000007439	*ODZ4*	936	1'010	988	811	1'166	0.525	0.525079525	1
ENSBTAG00000007440	*MLANA*	266	176	217	230	203	-0.181	0.86616673	1
ENSBTAG00000007441	*SEC23IP*	2'356	2'052	2'205	2'040	2'369	0.216	0.784100866	1
ENSBTAG00000007442	*AKAP9*	4'390	2'952	3'605	3'802	3'409	-0.157	0.840214765	1
ENSBTAG00000007443	*pseudogene*	1'866	2'622	2'322	1'616	3'028	0.906	0.252576443	1
ENSBTAG00000007445	*CD28*	1	0	0	1	0		1	1
ENSBTAG00000007446	*NGF*	289	232	259	250	268	0.098	0.924560269	1
ENSBTAG00000007447	*NUDT4*	6'260	2'045	3'891	5'421	2'361	-1.199	0.126585626	1
ENSBTAG00000007449	*HS3ST1*	322	208	260	279	240	-0.215	0.832371209	1
ENSBTAG00000007450	*C2*	396	370	385	343	427	0.317	0.732001407	1
ENSBTAG00000007453	*RDBP*	2'359	2'991	2'748	2'043	3'454	0.757	0.334820997	1
ENSBTAG00000007454	*RPL10*	16'474	24'583	21'326	14'267	28'386	0.993	0.195686758	1
ENSBTAG00000007455	*DNASE1L1*	242	106	166	210	122	-0.776	0.487973222	1
ENSBTAG00000007456	*TAZ*	1'269	963	1'105	1'099	1'112	0.017	0.984026315	1
ENSBTAG00000007457	*BT.87844*	32	0	14	28	0		0.183444185	1
ENSBTAG00000007460	*RAB11FIP2*	1'008	492	721	873	568	-0.620	0.467251354	1
ENSBTAG00000007461	*C20H5orf34*	950	455	674	823	525	-0.647	0.451136485	1
ENSBTAG00000007462	*ASTE1*	260	161	206	225	186	-0.276	0.796481316	1
ENSBTAG00000007467	*MIB1*	1'166	808	971	1'010	933	-0.114	0.891987791	1
ENSBTAG00000007470	*TRIM58*	6	1	3	5	1	-2.170	0.885240772	1
ENSBTAG00000007473	*protein_coding*	69	74	73	60	85	0.516	0.730366189	1
ENSBTAG00000007474	*EIF3B*	6'069	6'309	6'270	5'256	7'285	0.471	0.540165866	1
ENSBTAG00000007476	*BT.25271*	1'081	859	964	936	992	0.083	0.92015563	1
ENSBTAG00000007477	*protein_coding*	50	62	57	43	72	0.725	0.659181229	1
ENSBTAG00000007479	*CCDC90B*	1'714	951	1'291	1'484	1'098	-0.435	0.592506984	1
ENSBTAG00000007480	*CIRBP*	18'419	20'099	19'580	15'951	23'208	0.541	0.477801353	1
ENSBTAG00000007482	*pseudogene*	8	5	6	7	6	-0.263	0.996869191	1
ENSBTAG00000007483	*UNC119B*	427	686	581	370	792	1.099	0.210052933	1
ENSBTAG00000007484	*BT.49540*	1'301	825	1'040	1'127	953	-0.242	0.769734667	1
ENSBTAG00000007485	*GRHL1*	5'097	2'155	3'451	4'414	2'488	-0.827	0.290422615	1
ENSBTAG00000007486	*CEL*	8	1	4	7	1	-2.585	0.818599781	1
ENSBTAG00000007488	*ZFP90*	1'627	747	1'136	1'409	863	-0.708	0.388101883	1
ENSBTAG00000007489	*NCOA3*	987	882	937	855	1'018	0.253	0.760473881	1
ENSBTAG00000007490	*SULF2*	5'317	4'430	4'860	4'605	5'115	0.152	0.843648863	1
ENSBTAG00000007492	*BT.52241*	320	293	308	277	338	0.288	0.765811322	1
ENSBTAG00000007493	*BT.30403*	74	24	46	64	28	-1.209	0.509156749	1
ENSBTAG00000007494	*SMARCA2*	15'117	10'203	12'437	13'092	11'781	-0.152	0.84269594	1
ENSBTAG00000007496	*P2RX2*	82	5	38	71	6	-3.621	0.098518384	1
ENSBTAG00000007497	*ARFRP1*	1'988	2'491	2'299	1'722	2'876	0.740	0.348592388	1
ENSBTAG00000007498	*ZGPAT*	1'369	1'633	1'536	1'186	1'886	0.669	0.404753833	1
ENSBTAG00000007499	*HNRPH2*	4'112	2'844	3'423	3'561	3'284	-0.117	0.881498888	1
ENSBTAG00000007501	*LIME1*	170	129	148	147	149	0.017	0.994338331	1
ENSBTAG00000007502	*CKMT1*	207	152	177	179	176	-0.031	0.984432515	1
ENSBTAG00000007503	*STRC*	11	2	6	10	2	-2.044	0.786625039	1

Ensembl gene ID	geneName	counts wildtype horn bud	counts polled horn bud	baseMean	baseMean wildtype horn bud	baseMean polled horn bud	log2FoldChange	pval	padj
ENSBTAG00000007507	*BT.20123*	1'000	521	734	866	602	-0.526	0.536684251	1
ENSBTAG00000007508	*SRBD1*	2'376	1'738	2'032	2'058	2'007	-0.036	0.96499501	1
ENSBTAG00000007509	*bta-mir-935*	40	0	17	35	0		0.116452486	1
ENSBTAG00000007510	*PXMP2*	643	361	487	557	417	-0.418	0.6406588	1
ENSBTAG00000007512	*PLA2G15*	361	458	421	313	529	0.758	0.405635257	1
ENSBTAG00000007513	*PGAM5*	1'636	1'497	1'573	1'417	1'729	0.287	0.719863956	1
ENSBTAG00000007514	*CPNE5*	411	568	506	356	656	0.882	0.321387098	1
ENSBTAG00000007515	*UBE2Q1*	4'247	2'805	3'458	3'678	3'239	-0.183	0.814402905	1
ENSBTAG00000007516	*processed_pseudogene*	960	1'089	1'044	831	1'257	0.597	0.468137748	1
ENSBTAG00000007517	*CHRNB2*	35	9	20	30	10	-1.544	0.591735698	1
ENSBTAG00000007519	*ADAR*	2'177	2'799	2'559	1'885	3'232	0.778	0.323389753	1
ENSBTAG00000007520	*MAPRE2*	1'910	2'203	2'099	1'654	2'544	0.621	0.432747687	1
ENSBTAG00000007522	*BT.65168*	779	507	630	675	585	-0.205	0.814345296	1
ENSBTAG00000007523	*STX4*	1'315	1'170	1'245	1'139	1'351	0.246	0.761297592	1
ENSBTAG00000007530	*BT.26590*	9	5	7	8	6	-0.433	0.969858938	1
ENSBTAG00000007531	*NCF4*	64	51	57	55	59	0.087	0.968097632	1
ENSBTAG00000007534	*GGH*	341	120	217	295	139	-1.092	0.296464069	1
ENSBTAG00000007537	*PEX19*	1'584	1'906	1'786	1'372	2'201	0.682	0.392509827	1
ENSBTAG00000007540	*GLUD1*	6'840	5'572	6'179	5'924	6'434	0.119	0.876218106	1
ENSBTAG00000007543	*TAF1B*	1'008	411	674	873	475	-0.879	0.306883081	1
ENSBTAG00000007544	*MARVELD3*	48	42	45	42	48	0.222	0.913281698	1
ENSBTAG00000007545	*PHLPP2*	213	176	194	184	203	0.140	0.899380039	1
ENSBTAG00000007546	*CSNK1E*	546	571	566	473	659	0.480	0.582902199	1
ENSBTAG00000007547	*CACNG1*	3	37	23	3	43	4.040	0.169907182	1
ENSBTAG00000007550	*OLFM2*	2'825	3'621	3'314	2'447	4'181	0.773	0.322456036	1
ENSBTAG00000007553	*BT.53878*	80	18	45	69	21	-1.737	0.351202531	1
ENSBTAG00000007554	*IFI6*	2'039	2'361	2'246	1'766	2'726	0.627	0.427399261	1
ENSBTAG00000007556	*DYRK4*	8	9	9	7	10	0.585	0.926558417	1
ENSBTAG00000007558	*IL9R*	2	12	8	2	14	3.000	0.606634849	1
ENSBTAG00000007559	*BT.28504*	3'652	2'605	3'085	3'163	3'008	-0.072	0.927135305	1
ENSBTAG00000007562	*TRAM1*	4'346	3'098	3'671	3'764	3'577	-0.073	0.925764738	1
ENSBTAG00000007564	*BBS12*	576	388	473	499	448	-0.155	0.865090817	1
ENSBTAG00000007566	*CEP170*	3'463	1'656	2'456	2'999	1'912	-0.649	0.410302996	1
ENSBTAG00000007567	*TMX3*	3'332	2'612	2'951	2'886	3'016	0.064	0.934604526	1
ENSBTAG00000007569	*TNFRSF11A*	194	84	133	168	97	-0.793	0.507984379	1
ENSBTAG00000007570	*NDUFAF6*	361	270	312	313	312	-0.004	1	1
ENSBTAG00000007572	*ATP6V1F*	1'485	2'194	1'910	1'286	2'533	0.978	0.220762176	1
ENSBTAG00000007577	*UBXN7*	559	528	547	484	610	0.333	0.704644736	1
ENSBTAG00000007578	*BT.69928*	4'222	1'399	2'636	3'656	1'615	-1.178	0.137152235	1
ENSBTAG00000007580	*ZSWIM2*	4	0	2	3	0		0.89545886	1
ENSBTAG00000007581	*ADORA2B*	203	133	165	176	154	-0.195	0.866533742	1
ENSBTAG00000007583	*BT.91072*	47'002	43'743	45'607	40'705	50'510	0.311	0.681254419	1
ENSBTAG00000007584	*INPP1*	989	507	721	856	585	-0.549	0.51951669	1
ENSBTAG00000007586	*BT.87823*	926	776	849	802	896	0.160	0.848514411	1
ENSBTAG00000007588	*BT.28958*	4'989	3'430	4'141	4'321	3'961	-0.126	0.872125056	1
ENSBTAG00000007589	*BT.61870*	543	249	379	470	288	-0.710	0.445237103	1
ENSBTAG00000007591	*CHUK*	2'175	1'514	1'816	1'884	1'748	-0.108	0.893614389	1
ENSBTAG00000007592	*RARG*	7'347	10'089	9'006	6'363	11'650	0.873	0.256984818	1
ENSBTAG00000007593	*AIDA*	412	290	346	357	335	-0.092	0.926261438	1
ENSBTAG00000007594	*CWF19L1*	1'711	998	1'317	1'482	1'152	-0.363	0.654985633	1
ENSBTAG00000007595	*BROX*	1'450	1'017	1'215	1'256	1'174	-0.097	0.906951574	1
ENSBTAG00000007596	*GEM*	3'794	2'528	3'102	3'286	2'919	-0.171	0.827659661	1
ENSBTAG00000007599	*IKBKB*	1'063	1'077	1'082	921	1'244	0.434	0.596530614	1
ENSBTAG00000007602	*ITGA8*	1'092	810	941	946	935	-0.016	0.986834576	1
ENSBTAG00000007605	*FKBP4*	5'225	8'942	7'425	4'525	10'325	1.190	0.125114267	1
ENSBTAG00000007606	*HNRNPU*	19'594	10'877	14'764	16'969	12'560	-0.434	0.570082784	1
ENSBTAG00000007608	*C7ORF60*	284	218	249	246	252	0.033	0.977138359	1
ENSBTAG00000007609	*ITFG2*	1'233	1'193	1'223	1'068	1'378	0.367	0.651057143	1
ENSBTAG00000007611	*NRIP2*	261	370	327	226	427	0.919	0.33552529	1
ENSBTAG00000007614	*BBS4*	3'885	1'992	2'832	3'365	2'300	-0.549	0.484230895	1
ENSBTAG00000007615	*CNTN3*	351	115	218	304	133	-1.195	0.25322049	1
ENSBTAG00000007616	*TMED6*	217	149	180	188	172	-0.127	0.912521801	1
ENSBTAG00000007617	*TERF2*	2'756	2'007	2'352	2'387	2'317	-0.042	0.958171391	1
ENSBTAG00000007618	*BT.24946*	16	0	7	14	0		0.463690882	1
ENSBTAG00000007619	*PUSL1*	347	267	304	301	308	0.037	0.972434385	1
ENSBTAG00000007620	*BT.75461*	2'314	2'658	2'537	2'004	3'069	0.615	0.433948832	1
ENSBTAG00000007622	*CATD*	13'627	13'785	13'859	11'801	15'918	0.432	0.571346757	1
ENSBTAG00000007623	*GLTPD1*	1'062	1'058	1'071	920	1'222	0.410	0.617507198	1
ENSBTAG00000007624	*FRMD4B*	2'430	1'638	1'998	2'104	1'891	-0.154	0.846925701	1
ENSBTAG00000007626	*IL2RG*	301	133	207	261	154	-0.763	0.469196836	1
ENSBTAG00000007629	*THAP1*	382	264	318	331	305	-0.118	0.905552893	1
ENSBTAG00000007630	*RNF170*	1'483	1'299	1'392	1'284	1'500	0.224	0.781064728	1

Ensembl gene ID	geneName	counts wildtype horn bud	counts polled horn bud	baseMean	baseMean wildtype horn bud	baseMean polled horn bud	log2FoldChange	pval	padj
ENSBTAG00000007632	*FAM151B*	800	635	713	693	733	0.082	0.924245562	1
ENSBTAG00000007633	*ANKRD34B*	11	13	12	10	15	0.656	0.880852037	1
ENSBTAG00000007634	*HOOK3*	287	208	244	249	240	-0.049	0.966161568	1
ENSBTAG00000007636	*IRAKM*	373	240	300	323	277	-0.221	0.822245751	1
ENSBTAG00000007638	*PIF1*	35	43	40	30	50	0.712	0.717092181	1
ENSBTAG00000007639	*BT.19687*	1'588	1'124	1'337	1'375	1'298	-0.084	0.919245629	1
ENSBTAG00000007642	*ADORA3*	286	193	235	248	223	-0.152	0.885466775	1
ENSBTAG00000007643	*TRIM39-RPP21*	384	272	323	333	314	-0.082	0.935052192	1
ENSBTAG00000007644	*GNG7*	1'621	2'460	2'122	1'404	2'841	1.017	0.201402057	1
ENSBTAG00000007646	*PIGA*	276	172	219	239	199	-0.267	0.800081166	1
ENSBTAG00000007647	*OR13H1*	0	2	1	0	2	Inf	0.939077559	1
ENSBTAG00000007648	*NET1*	9'021	8'135	8'603	7'812	9'393	0.266	0.72789312	1
ENSBTAG00000007649	*TM2D3*	680	481	572	589	555	-0.084	0.92552319	1
ENSBTAG00000007650	*BT.65378*	1	1	1	1	1	0.415	1	1
ENSBTAG00000007651	*TARSL2*	684	139	376	592	161	-1.884	0.048092897	1
ENSBTAG00000007652	*RFTN2*	3'784	2'259	2'943	3'277	2'608	-0.329	0.674264846	1
ENSBTAG00000007654	*CHST7*	168	91	125	145	105	-0.469	0.702227008	1
ENSBTAG00000007657	*SCARA3*	2'582	2'508	2'566	2'236	2'896	0.373	0.634080363	1
ENSBTAG00000007658	*BT.43180*	1'701	1'812	1'783	1'473	2'092	0.506	0.52487807	1
ENSBTAG00000007659	*CORO2A*	214	69	133	185	80	-1.218	0.310576881	1
ENSBTAG00000007660	*BT.27825*	1'233	1'099	1'168	1'068	1'269	0.249	0.75989962	1
ENSBTAG00000007661	*MYO1F*	333	197	258	288	227	-0.342	0.734194022	1
ENSBTAG00000007662	*GRP78*	18'902	18'400	18'808	16'370	21'246	0.376	0.620959341	1
ENSBTAG00000007665	*NPR3*	115	108	112	100	125	0.324	0.799916547	1
ENSBTAG00000007666	*IGF2BP2*	1'001	692	833	867	799	-0.118	0.890327478	1
ENSBTAG00000007668	*AGPAT4*	464	290	368	402	335	-0.263	0.779931383	1
ENSBTAG00000007674	*BT.63602*	0	1	1	0	1	Inf	0.993540919	1
ENSBTAG00000007675	*NPHP1*	663	647	661	574	747	0.380	0.657852169	1
ENSBTAG00000007678	*MKX*	1'879	1'450	1'651	1'627	1'674	0.041	0.959041701	1
ENSBTAG00000007680	*THSD7A*	134	51	87	116	59	-0.979	0.479706625	1
ENSBTAG00000007681	*DHFR*	1'271	1'442	1'383	1'101	1'665	0.597	0.459634948	1
ENSBTAG00000007683	*DCAF17*	384	216	291	333	249	-0.415	0.671673018	1
ENSBTAG00000007684	*BT.90094*	2'134	1'318	1'685	1'848	1'522	-0.280	0.726543075	1
ENSBTAG00000007685	*PSMB1*	4'117	3'705	3'922	3'565	4'278	0.263	0.734047134	1
ENSBTAG00000007686	*TBP*	161	139	150	139	161	0.203	0.863186597	1
ENSBTAG00000007687	*BT.29849*	66	1	29	57	1	-5.629	0.0572057	1
ENSBTAG00000007689	*LPIN1*	236	196	215	204	226	0.147	0.89060146	1
ENSBTAG00000007692	*pseudogene*	4'631	8'530	6'930	4'011	9'850	1.296	0.095914499	1
ENSBTAG00000007693	*SLC45A3*	187	74	124	162	85	-0.922	0.451153527	1
ENSBTAG00000007694	*KIF25*	79	11	41	68	13	-2.429	0.225857322	1
ENSBTAG00000007695	*CSN1S1*	3	0	1	3	0		0.936647693	1
ENSBTAG00000007696	*protein_coding*	3'133	1'234	2'069	2'713	1'425	-0.929	0.243280219	1
ENSBTAG00000007700	*PHYH*	1'599	1'220	1'397	1'385	1'409	0.025	0.975792902	1
ENSBTAG00000007702	*BT.24490*	373	268	316	323	309	-0.062	0.952658826	1
ENSBTAG00000007703	*NMT2*	1'221	974	1'091	1'057	1'125	0.089	0.913849842	1
ENSBTAG00000007704	*BT.20013*	75	85	82	65	98	0.596	0.675191642	1
ENSBTAG00000007705	*COLEC12*	11'891	8'058	9'801	10'298	9'305	-0.146	0.848964631	1
ENSBTAG00000007708	*NLGN3*	2'008	1'586	1'785	1'739	1'831	0.075	0.925208322	1
ENSBTAG00000007709	*SHOC2*	3'117	1'864	2'426	2'699	2'152	-0.327	0.678513151	1
ENSBTAG00000007712	*BT.53768*	2'253	1'926	2'088	1'951	2'224	0.189	0.810904025	1
ENSBTAG00000007714	*ZNF235*	670	405	524	580	468	-0.311	0.725974151	1
ENSBTAG00000007718	*TGIF1*	2'086	2'159	2'150	1'807	2'493	0.465	0.556156288	1
ENSBTAG00000007719	*MFSD6*	735	475	593	637	548	-0.215	0.806629195	1
ENSBTAG00000007720	*NKG2A*	3	0	1	3	0		0.936647693	1
ENSBTAG00000007721	*DPM1*	4'843	2'232	3'386	4'194	2'577	-0.703	0.368634494	1
ENSBTAG00000007722	*MOCS3*	625	521	571	541	602	0.152	0.862093106	1
ENSBTAG00000007723	*BT.53389*	703	608	655	609	702	0.206	0.811134578	1
ENSBTAG00000007724	*SAMD13*	1	1	1	1	1	0.415	1	1
ENSBTAG00000007725	*ARF1*	10'712	7'746	9'111	9'277	8'944	-0.053	0.945933988	1
ENSBTAG00000007728	*PES1*	2'136	2'571	2'409	1'850	2'969	0.682	0.386470228	1
ENSBTAG00000007730	*ZFX*	2'241	1'074	1'590	1'941	1'240	-0.646	0.421242424	1
ENSBTAG00000007731	*protein_coding*	348	308	329	301	356	0.239	0.802730762	1
ENSBTAG00000007732	*BT.34295*	357	129	229	309	149	-1.054	0.307175928	1
ENSBTAG00000007734	*MEMO1*	1'256	635	910	1'088	733	-0.569	0.494977371	1
ENSBTAG00000007736	*BT.66714*	15	0	6	13	0		0.491308095	1
ENSBTAG00000007737	*UBA52*	28'781	19'646	23'805	24'925	22'685	-0.136	0.858910198	1
ENSBTAG00000007739	*C7H19ORF60*	1'332	1'080	1'200	1'154	1'247	0.112	0.890245716	1
ENSBTAG00000007740	*BMK*	8'364	9'221	8'945	7'243	10'647	0.556	0.468337338	1
ENSBTAG00000007741	*CRLF1*	884	407	618	766	470	-0.704	0.417052922	1
ENSBTAG00000007743	*BT.69197*	775	625	696	671	722	0.105	0.903059474	1
ENSBTAG00000007746	*AHR*	4'875	1'833	3'169	4'222	2'117	-0.996	0.204959533	1
ENSBTAG00000007749	*TONSL*	871	1'261	1'105	754	1'456	0.949	0.249070712	1

Ensembl gene ID	geneName	counts wildtype horn bud	counts polled horn bud	baseMean	baseMean wildtype horn bud	baseMean polled horn bud	log2FoldChange	pval	padj
ENSBTAG00000007750	*pseudogene*	2	8	5	2	9	2.415	0.75674087	1
ENSBTAG00000007753	*KIFC2*	1'560	1'132	1'329	1'351	1'307	-0.048	0.954689436	1
ENSBTAG00000007755	*APOBEC3Z3*	71	47	58	61	54	-0.180	0.923088677	1
ENSBTAG00000007756	*TFPT*	430	692	586	372	799	1.101	0.20860721	1
ENSBTAG00000007757	*PRPF31*	1'738	2'213	2'030	1'505	2'555	0.764	0.33620351	1
ENSBTAG00000007758	*PDE10A*	223	141	178	193	163	-0.246	0.826045398	1
ENSBTAG00000007759	*BT.32772*	1'399	2'157	1'851	1'212	2'491	1.040	0.194185457	1
ENSBTAG00000007761	*ZBTB5*	861	991	945	746	1'144	0.618	0.456278247	1
ENSBTAG00000007762	*ACCN3*	45	48	47	39	55	0.508	0.781570227	1
ENSBTAG00000007763	*SLC1A4*	3'024	4'348	3'820	2'619	5'021	0.939	0.229182773	1
ENSBTAG00000007765	*TFCP2L1*	13	5	9	11	6	-0.963	0.868297704	1
ENSBTAG00000007766	*CDK5*	1'360	1'734	1'590	1'178	2'002	0.766	0.340434424	1
ENSBTAG00000007767	*TBX15*	2'764	3'055	2'961	2'394	3'528	0.559	0.474183831	1
ENSBTAG00000007768	*KCNS2*	22	56	42	19	65	1.763	0.357224328	1
ENSBTAG00000007772	*SLC29A4*	396	158	263	343	182	-0.911	0.361953189	1
ENSBTAG00000007773	*VCAM1*	3'779	2'414	3'030	3'273	2'787	-0.232	0.767549203	1
ENSBTAG00000007776	*BRPF1*	1'444	2'077	1'824	1'251	2'398	0.939	0.240190062	1
ENSBTAG00000007777	*OGG1*	660	686	682	572	792	0.471	0.581710945	1
ENSBTAG00000007778	*TRPM3*	91	89	91	79	103	0.383	0.78128012	1
ENSBTAG00000007779	*GTF3C3*	654	708	692	566	818	0.529	0.534976634	1
ENSBTAG00000007780	*CAMK1*	1'763	945	1'309	1'527	1'091	-0.485	0.550489992	1
ENSBTAG00000007783	*MYBL2*	1'345	1'745	1'590	1'165	2'015	0.791	0.32499731	1
ENSBTAG00000007784	*NAA50*	5'593	3'702	4'559	4'844	4'275	-0.180	0.816353913	1
ENSBTAG00000007785	*PKD2L2*	218	70	135	189	81	-1.224	0.305647106	1
ENSBTAG00000007786	*FBXL14*	1'144	957	1'048	991	1'105	0.158	0.848118111	1
ENSBTAG00000007787	*HIBCH*	880	563	706	762	650	-0.229	0.789352261	1
ENSBTAG00000007788	*GRIPAP1*	3'184	1'745	2'386	2'757	2'015	-0.453	0.565999836	1
ENSBTAG00000007791	*KCNQ5*	43	13	26	37	15	-1.311	0.596402998	1
ENSBTAG00000007793	*SERTAD2*	135	84	107	117	97	-0.269	0.839358789	1
ENSBTAG00000007794	*KRT20*	28	7	16	24	8	-1.585	0.635899565	1
ENSBTAG00000007796	*BT.32570*	94	66	79	81	76	-0.095	0.95734325	1
ENSBTAG00000007797	*IGBP1*	2'490	1'895	2'172	2'156	2'188	0.021	0.978635163	1
ENSBTAG00000007798	*DGAT2L6*	12	4	8	10	5	-1.170	0.853405557	1
ENSBTAG00000007799	*FAM54A*	148	153	152	128	177	0.463	0.686198068	1
ENSBTAG00000007800	*BT.17207*	41	40	41	36	46	0.379	0.851299942	1
ENSBTAG00000007802	*BCLAF1*	6'089	3'754	4'804	5'273	4'335	-0.283	0.714980892	1
ENSBTAG00000007804	*RILPL2*	1'360	810	1'057	1'178	935	-0.333	0.686669509	1
ENSBTAG00000007806	*MTPN*	2'874	2'993	2'972	2'489	3'456	0.474	0.544225082	1
ENSBTAG00000007807	*pseudogene*	2'040	1'715	1'874	1'767	1'980	0.165	0.835476034	1
ENSBTAG00000007808	*ANTXR1*	5'566	7'818	6'924	4'820	9'027	0.905	0.241272616	1
ENSBTAG00000007809	*PPP1R36*	493	171	312	427	197	-1.113	0.249921886	1
ENSBTAG00000007812	*NDUFA13*	3'437	3'344	3'419	2'977	3'861	0.375	0.629035138	1
ENSBTAG00000007813	*MTCP1NB*	537	243	373	465	281	-0.729	0.434349384	1
ENSBTAG00000007814	*WWTR1*	3'176	2'075	2'573	2'750	2'396	-0.199	0.800587312	1
ENSBTAG00000007816	*processed_pseudogene*	334	476	419	289	550	0.926	0.311055809	1
ENSBTAG00000007817	*BT.58955*	457	282	361	396	326	-0.281	0.765567574	1
ENSBTAG00000007818	*POFUT2*	8'225	10'071	9'376	7'123	11'629	0.707	0.356881184	1
ENSBTAG00000007820	*processed_pseudogene*	570	902	768	494	1'042	1.077	0.205287008	1
ENSBTAG00000007821	*NPHS1*	6	1	3	5	1	-2.170	0.885240772	1
ENSBTAG00000007823	*BT.88019*	30	88	64	26	102	1.968	0.216188787	1
ENSBTAG00000007825	*MARK2*	1'500	2'442	2'059	1'299	2'820	1.118	0.16137262	1
ENSBTAG00000007826	*KIRREL2*	10	7	8	9	8	-0.100	1	1
ENSBTAG00000007827	*RCOR2*	1'787	1'050	1'380	1'548	1'212	-0.352	0.663541233	1
ENSBTAG00000007828	*SLA*	339	65	184	294	75	-1.968	0.077159686	1
ENSBTAG00000007829	*CSAD*	1'930	918	1'366	1'671	1'060	-0.657	0.417455012	1
ENSBTAG00000007830	*PLEKHM3*	88	66	76	76	76	0.000	1	1
ENSBTAG00000007833	*PIAS2*	1'270	817	1'022	1'100	943	-0.221	0.789412128	1
ENSBTAG00000007834	*PPP1R16A*	739	1'081	944	640	1'248	0.964	0.247469009	1
ENSBTAG00000007835	*GPT*	550	638	607	476	737	0.629	0.467895931	1
ENSBTAG00000007836	*PPA1*	1'831	1'397	1'599	1'586	1'613	0.025	0.975502428	1
ENSBTAG00000007837	*EPT1*	254	142	192	220	164	-0.424	0.694935053	1
ENSBTAG00000007838	*MFSD3*	443	386	415	384	446	0.216	0.81362608	1
ENSBTAG00000007840	*HMGCR*	4'013	3'178	3'572	3'475	3'670	0.078	0.919174134	1
ENSBTAG00000007841	*WTIP*	1'046	1'120	1'100	906	1'293	0.514	0.530593299	1
ENSBTAG00000007842	*GCLM*	413	213	302	358	246	-0.540	0.57749269	1
ENSBTAG00000007843	*BT.18317*	20	2	10	17	2	-2.907	0.559880543	1
ENSBTAG00000007844	*CETN2*	3'872	2'920	3'362	3'353	3'372	0.008	0.991626223	1
ENSBTAG00000007846	*ITIH3*	9	4	6	8	5	-0.755	0.931866093	1
ENSBTAG00000007847	*EED*	2'519	1'173	1'768	2'182	1'354	-0.688	0.389726783	1
ENSBTAG00000007850	*ITIH4*	202	159	179	175	184	0.070	0.953579141	1
ENSBTAG00000007851	*IL24*	3	2	2	3	2	-0.170	1	1
ENSBTAG00000007855	*VSIG10*	268	174	217	232	201	-0.208	0.845364365	1

Ensembl gene ID	geneName	counts wildtype horn bud	counts polled horn bud	baseMean	baseMean wildtype horn bud	baseMean polled horn bud	log2FoldChange	pval	padj
ENSBTAG00000007859	*BT.28172*	10	1	5	9	1	-2.907	0.754028015	1
ENSBTAG00000007860	*ASPM*	1'129	1'281	1'228	978	1'479	0.597	0.462826587	1
ENSBTAG00000007863	*BT.64156*	4'977	2'655	3'688	4'310	3'066	-0.492	0.527816394	1
ENSBTAG00000007865	*GNPDA1*	1'361	955	1'141	1'179	1'103	-0.096	0.908039124	1
ENSBTAG00000007866	*HS3ST3B1*	62	107	89	54	124	1.202	0.381080335	1
ENSBTAG00000007867	*BT.33292*	7'878	4'763	6'161	6'823	5'500	-0.311	0.686716368	1
ENSBTAG00000007870	*FKBP14*	7'421	3'783	5'398	6'427	4'368	-0.557	0.471112408	1
ENSBTAG00000007871	*SNX12*	3'199	3'301	3'291	2'770	3'812	0.460	0.554379606	1
ENSBTAG00000007872	*PSIP1*	5'613	3'195	4'275	4'861	3'689	-0.398	0.608010213	1
ENSBTAG00000007875	*BT.106000*	11'373	5'275	7'970	9'849	6'091	-0.693	0.367755969	1
ENSBTAG00000007876	*MAPK8*	2'998	1'680	2'268	2'596	1'940	-0.421	0.594524615	1
ENSBTAG00000007878	*ARHGAP22*	144	54	94	125	62	-1.000	0.458905992	1
ENSBTAG00000007880	*RAB40C*	1'261	1'621	1'482	1'092	1'872	0.777	0.334915808	1
ENSBTAG00000007881	*IFIT1*	121	115	119	105	133	0.342	0.784878782	1
ENSBTAG00000007882	*CACNA1B*	556	444	497	482	513	0.091	0.920269332	1
ENSBTAG00000007883	*pseudogene*	192	213	206	166	246	0.565	0.591435677	1
ENSBTAG00000007884	*SREBF1*	3'879	4'214	4'113	3'359	4'866	0.535	0.49042228	1
ENSBTAG00000007887	*GRIN2A*	2	1	1	2	1	-0.585	1	1
ENSBTAG00000007888	*EFR3B*	211	31	109	183	36	-2.352	0.075274917	1
ENSBTAG00000007890	*SEC14L5*	53	41	47	46	47	0.045	0.993138314	1
ENSBTAG00000007893	*SCPEP1*	4'726	3'685	4'174	4'093	4'255	0.056	0.941858627	1
ENSBTAG00000007895	*SLC20A1*	3'719	2'159	2'857	3'221	2'493	-0.370	0.637333583	1
ENSBTAG00000007896	*NAGPA*	494	443	470	428	512	0.258	0.774041161	1
ENSBTAG00000007897	*POMC*	181	155	168	157	179	0.191	0.866624762	1
ENSBTAG00000007898	*CYBRD1*	808	790	806	700	912	0.383	0.648847407	1
ENSBTAG00000007900	*FIS1*	3'075	2'876	2'992	2'663	3'321	0.319	0.68289715	1
ENSBTAG00000007901	*EMR1*	2	0	1	2	0		0.974934741	1
ENSBTAG00000007904	*KRT72*	12	1	6	10	1	-3.170	0.692545189	1
ENSBTAG00000007906	*ZACN*	517	678	615	448	783	0.806	0.352381767	1
ENSBTAG00000007909	*NOTCH2*	4'258	4'426	4'399	3'688	5'111	0.471	0.542693846	1
ENSBTAG00000007910	*BT.49465*	2'691	3'507	3'190	2'330	4'050	0.797	0.308347011	1
ENSBTAG00000007913	*CWC15*	7'082	4'332	5'568	6'133	5'002	-0.294	0.703317445	1
ENSBTAG00000007914	*pseudogene*	14	5	9	12	6	-1.070	0.844263455	1
ENSBTAG00000007916	*FOXJ1*	48	1	21	42	1	-5.170	0.130942353	1
ENSBTAG00000007917	*TSHZ2*	431	406	421	373	469	0.329	0.71841611	1
ENSBTAG00000007920	*PANK2*	1'139	547	809	986	632	-0.643	0.445479158	1
ENSBTAG00000007921	*DAPP1*	153	34	86	133	39	-1.755	0.214120938	1
ENSBTAG00000007922	*RNF24*	123	121	123	107	140	0.391	0.75062078	1
ENSBTAG00000007923	*LAMTOR3*	3'483	2'113	2'728	3'016	2'440	-0.306	0.696748026	1
ENSBTAG00000007927	*SLAMF1*	8	4	6	7	5	-0.585	0.95915875	1
ENSBTAG00000007930	*NCOA6*	3'379	2'112	2'683	2'926	2'439	-0.263	0.737963188	1
ENSBTAG00000007931	*SLC38A6*	493	329	403	427	380	-0.168	0.857050857	1
ENSBTAG00000007932	*BT.44996*	265	85	164	229	98	-1.225	0.277165708	1
ENSBTAG00000007933	*C3ORF58*	2'533	1'332	1'866	2'194	1'538	-0.512	0.520191901	1
ENSBTAG00000007934	*MRPS17*	737	610	671	638	704	0.142	0.868751215	1
ENSBTAG00000007935	*CALCOCO2*	5'379	3'292	4'230	4'658	3'801	-0.293	0.705489578	1
ENSBTAG00000007937	*PRIM2*	787	775	788	682	895	0.393	0.640738698	1
ENSBTAG00000007939	*CHMP2B*	3'951	2'347	3'066	3'422	2'710	-0.336	0.667201236	1
ENSBTAG00000007942	*PAPD5*	532	271	387	461	313	-0.558	0.546922929	1
ENSBTAG00000007943	*AZIN1*	10'579	5'360	7'675	9'162	6'189	-0.566	0.461962648	1
ENSBTAG00000007946	*PIPOX*	42	4	20	36	5	-2.977	0.320832869	1
ENSBTAG00000007948	*SORCS1*	90	17	49	78	20	-1.989	0.270816526	1
ENSBTAG00000007952	*BT.48853*	3'236	5'353	4'492	2'802	6'181	1.141	0.144074528	1
ENSBTAG00000007953	*BT.24006*	975	500	711	844	577	-0.548	0.520520197	1
ENSBTAG00000007954	*CDIPT*	1'902	2'152	2'066	1'647	2'485	0.593	0.453727278	1
ENSBTAG00000007956	*FRAT1*	22	9	15	19	10	-0.874	0.813237758	1
ENSBTAG00000007958	*BT.28890*	1'032	1'658	1'404	894	1'914	1.099	0.176421598	1
ENSBTAG00000007960	*BT.39255*	2'884	2'351	2'606	2'498	2'715	0.120	0.877792234	1
ENSBTAG00000007961	*TADA3L*	4'867	3'703	4'245	4'215	4'276	0.021	0.978310047	1
ENSBTAG00000007962	*ATP9A*	978	1'199	1'116	847	1'384	0.709	0.387161794	1
ENSBTAG00000007963	*KIDINS220*	8'935	6'346	7'533	7'738	7'328	-0.079	0.919188364	1
ENSBTAG00000007964	*ARPC4*	2'899	3'890	3'501	2'511	4'492	0.839	0.282648405	1
ENSBTAG00000007966	*BT.88472*	2'862	1'702	2'222	2'479	1'965	-0.335	0.672068676	1
ENSBTAG00000007968	*RPUSD3*	2'468	1'689	2'044	2'137	1'950	-0.132	0.868488579	1
ENSBTAG00000007969	*CIDEC*	1	0	0	1	0		1	1
ENSBTAG00000007970	*C3ORF38*	1'204	839	1'006	1'043	969	-0.106	0.899397226	1
ENSBTAG00000007971	*OR6Q1*	0	1	1	0	1	Inf	0.993540919	1
ENSBTAG00000007974	*WTAP*	1'920	1'537	1'719	1'663	1'775	0.094	0.906040759	1
ENSBTAG00000007975	*ALDH8A1*	6	3	4	5	3	-0.585	0.976586913	1
ENSBTAG00000007976	*FAM3C*	6'815	3'164	4'778	5'902	3'653	-0.692	0.372266301	1
ENSBTAG00000007977	*LAMP5*	85	7	41	74	8	-3.187	0.124082372	1
ENSBTAG00000007979	*BT.88395*	1'809	1'655	1'739	1'567	1'911	0.287	0.718723447	1

Ensembl gene ID	geneName	counts wildtype horn bud	counts polled horn bud	baseMean	baseMean wildtype horn bud	baseMean polled horn bud	log2FoldChange	pval	padj
ENSBTAG00000007981	*GRK5*	976	1'142	1'082	845	1'319	0.642	0.434483809	1
ENSBTAG00000007983	*ZNF775*	111	103	108	96	119	0.307	0.813646839	1
ENSBTAG00000007986	*WHSC1*	1'912	3'726	2'979	1'656	4'302	1.378	0.08234478	1
ENSBTAG00000007988	*STX2*	1'249	955	1'092	1'082	1'103	0.028	0.973421204	1
ENSBTAG00000007989	*FUT9*	12	0	5	10	0		0.583618942	1
ENSBTAG00000007990	*EPYC*	14	25	20	12	29	1.252	0.658456692	1
ENSBTAG00000007992	*HAS3*	253	313	290	219	361	0.722	0.458422991	1
ENSBTAG00000007993	*CORO1C*	3'977	2'714	3'289	3'444	3'134	-0.136	0.862048174	1
ENSBTAG00000007994	*OSCP1*	84	91	89	73	105	0.531	0.700069632	1
ENSBTAG00000007996	*BT.102056*	115	84	98	100	97	-0.038	0.986648023	1
ENSBTAG00000007998	*TMCO4*	777	575	668	673	664	-0.019	0.984722329	1
ENSBTAG00000008001	*NUCKS1*	4'562	3'955	4'259	3'951	4'567	0.209	0.786560924	1
ENSBTAG00000008003	*SMG7*	4'418	3'325	3'833	3'826	3'839	0.005	0.994564584	1
ENSBTAG00000008004	*NCF2*	72	17	41	62	20	-1.667	0.391968253	1
ENSBTAG00000008005	*CCDC135*	4	0	2	3	0		0.89545886	1
ENSBTAG00000008006	*RASGRP3*	729	325	503	631	375	-0.750	0.399416507	1
ENSBTAG00000008008	*TLR2*	490	377	430	424	435	0.037	0.969707215	1
ENSBTAG00000008009	*PLD6*	1	1	1	1	1	0.415	1	1
ENSBTAG00000008010	*FLCN*	4'259	2'873	3'503	3'688	3'317	-0.153	0.844940311	1
ENSBTAG00000008013	*LRRC2*	49	29	38	42	33	-0.342	0.875360027	1
ENSBTAG00000008014	*BT.55643*	2'067	2'489	2'332	1'790	2'874	0.683	0.386600483	1
ENSBTAG00000008016	*STX17*	350	240	290	303	277	-0.129	0.898280049	1
ENSBTAG00000008020	*BT.7193*	3	15	10	3	17	2.737	0.564172985	1
ENSBTAG00000008021	*BT.42450*	87	37	59	75	43	-0.818	0.61640588	1
ENSBTAG00000008022	*PTGFRN*	4'840	6'032	5'578	4'192	6'965	0.733	0.343059129	1
ENSBTAG00000008024	*UCHL3*	919	445	655	796	514	-0.631	0.463661993	1
ENSBTAG00000008025	*UBE3C*	2'199	2'287	2'273	1'904	2'641	0.472	0.549408865	1
ENSBTAG00000008026	*OXT*	1	3	2	1	3	2.000	0.933774563	1
ENSBTAG00000008027	*AVP*	7	0	3	6	0		0.769774961	1
ENSBTAG00000008028	*CHN1*	1'222	661	911	1'058	763	-0.471	0.571699879	1
ENSBTAG00000008032	*BT.45948*	1'743	1'309	1'510	1'509	1'512	0.002	0.998358454	1
ENSBTAG00000008033	*PPP6C*	1'630	1'171	1'382	1'412	1'352	-0.062	0.940231788	1
ENSBTAG00000008034	*LMAN2*	5'648	5'263	5'484	4'891	6'077	0.313	0.683897728	1
ENSBTAG00000008035	*processed_pseudogene*	85	65	74	74	75	0.028	0.994589873	1
ENSBTAG00000008036	*CELSR1*	755	938	868	654	1'083	0.728	0.383960247	1
ENSBTAG00000008040	*SPG20*	6'937	5'630	6'254	6'008	6'501	0.114	0.881713588	1
ENSBTAG00000008047	*ZDHHC15*	117	68	90	101	79	-0.368	0.792918932	1
ENSBTAG00000008048	*GCFC2*	263	146	198	228	169	-0.434	0.685365173	1
ENSBTAG00000008053	*WDFY2*	349	454	413	302	524	0.795	0.385156923	1
ENSBTAG00000008054	*MGC133632*	3'172	1'831	2'431	2'747	2'114	-0.378	0.631698803	1
ENSBTAG00000008057	*MGC148449*	113	71	90	98	82	-0.255	0.85850073	1
ENSBTAG00000008059	*CHRM3*	28	8	17	24	9	-1.392	0.670673444	1
ENSBTAG00000008060	*SNRNP35*	820	536	665	710	619	-0.198	0.818850503	1
ENSBTAG00000008061	*BT.62192*	1'992	1'788	1'895	1'725	2'065	0.259	0.743696258	1
ENSBTAG00000008062	*DMRT2*	392	389	394	339	449	0.404	0.660891238	1
ENSBTAG00000008063	*PPARA*	619	437	520	536	505	-0.087	0.924100439	1
ENSBTAG00000008064	*VPS37C*	206	288	255	178	333	0.898	0.369776458	1
ENSBTAG00000008065	*CDPF1*	937	944	951	811	1'090	0.426	0.607061045	1
ENSBTAG00000008066	*PKDREJ*	106	74	89	92	85	-0.103	0.949233465	1
ENSBTAG00000008068	*ERLEC1*	6'722	3'610	4'995	5'821	4'168	-0.482	0.533393559	1
ENSBTAG00000008072	*FUSIP1*	5'981	3'090	4'374	5'180	3'568	-0.538	0.488231681	1
ENSBTAG00000008075	*TRAPPC4*	2'167	1'404	1'749	1'877	1'621	-0.211	0.792068616	1
ENSBTAG00000008076	*SLC35B2*	2'457	2'998	2'795	2'128	3'462	0.702	0.370722195	1
ENSBTAG00000008077	*BT.58907*	1'184	926	1'047	1'025	1'069	0.060	0.941819592	1
ENSBTAG00000008078	*MGC137058*	205	265	242	178	306	0.785	0.438154222	1
ENSBTAG00000008079	*NRBP2*	5'495	3'141	4'193	4'759	3'627	-0.392	0.613629875	1
ENSBTAG00000008082	*CEP350*	1'009	604	786	874	697	-0.325	0.700706886	1
ENSBTAG00000008083	*SEL1L*	1'001	943	978	867	1'089	0.329	0.690430559	1
ENSBTAG00000008084	*ZNF382*	934	395	632	809	456	-0.827	0.339893148	1
ENSBTAG00000008088	*KIF18B*	496	739	641	430	853	0.990	0.252346385	1
ENSBTAG00000008089	*AMMECR1L*	516	544	538	447	628	0.491	0.576333008	1
ENSBTAG00000008090	*CCM2*	1'601	1'609	1'622	1'387	1'858	0.422	0.597333016	1
ENSBTAG00000008091	*SELENBP1*	667	394	516	578	455	-0.344	0.698379037	1
ENSBTAG00000008092	*RAB37*	18	15	16	16	17	0.152	0.982518029	1
ENSBTAG00000008093	*NACAD*	714	801	772	618	925	0.581	0.491423913	1
ENSBTAG00000008095	*ACER1*	27	24	26	23	28	0.245	0.934933033	1
ENSBTAG00000008096	*EDN1*	560	161	335	485	186	-1.383	0.149278778	1
ENSBTAG00000008097	*WNT2*	100	118	111	87	136	0.654	0.604707243	1
ENSBTAG00000008098	*DCLK2*	981	510	719	850	589	-0.529	0.53512301	1
ENSBTAG00000008099	*CCR5*	71	59	65	61	68	0.148	0.932844744	1
ENSBTAG00000008100	*GOLGA7B*	201	24	101	174	28	-2.651	0.054114811	1
ENSBTAG00000008101	*PCSK5*	725	686	710	628	792	0.335	0.693595748	1

Ensembl gene ID	geneName	counts wildtype horn bud	counts polled horn bud	baseMean	baseMean wildtype horn bud	baseMean polled horn bud	log2FoldChange	pval	padj
ENSBTAG00000008102	*CRTAC1*	285	132	200	247	152	-0.695	0.513627041	1
ENSBTAG00000008105	*RBM38*	68	140	110	59	162	1.457	0.253431113	1
ENSBTAG00000008109	*TMEM97*	1'854	2'162	2'051	1'606	2'496	0.637	0.421629858	1
ENSBTAG00000008110	*IFT20*	2'986	1'653	2'247	2'586	1'909	-0.438	0.579334937	1
ENSBTAG00000008111	*ESYT3*	148	66	102	128	76	-0.750	0.566454295	1
ENSBTAG00000008112	*SHPRH*	842	689	762	729	796	0.126	0.882270968	1
ENSBTAG00000008114	*CD99*	7'615	8'740	8'343	6'595	10'092	0.614	0.42382923	1
ENSBTAG00000008115	*TNFAIP1*	1'410	1'601	1'535	1'221	1'849	0.598	0.456137666	1
ENSBTAG00000008116	*POLDIP2*	2'479	2'704	2'635	2'147	3'122	0.540	0.490851723	1
ENSBTAG00000008118	*BXDC5*	2'183	1'610	1'875	1'891	1'859	-0.024	0.977109956	1
ENSBTAG00000008120	*TMEM199*	1'648	914	1'241	1'427	1'055	-0.435	0.592938904	1
ENSBTAG00000008121	*BT.24212*	940	489	689	814	565	-0.528	0.537673576	1
ENSBTAG00000008122	*GNG5*	1'238	1'523	1'415	1'072	1'759	0.714	0.376654237	1
ENSBTAG00000008124	*GK*	259	192	223	224	222	-0.017	0.992859199	1
ENSBTAG00000008125	*KCNAB2*	135	247	201	117	285	1.287	0.227374739	1
ENSBTAG00000008126	*NPHP4*	922	580	734	798	670	-0.254	0.766397798	1
ENSBTAG00000008129	*CLSTN3*	1'039	586	788	900	677	-0.411	0.626545685	1
ENSBTAG00000008130	*MED10*	644	521	580	558	602	0.109	0.901187584	1
ENSBTAG00000008131	*BT.46389*	871	509	671	754	588	-0.360	0.675504865	1
ENSBTAG00000008132	*SOX13*	803	675	737	695	779	0.165	0.84655299	1
ENSBTAG00000008133	*ALKBH1*	1'462	865	1'132	1'266	999	-0.342	0.676553628	1
ENSBTAG00000008134	*BDNF*	156	343	266	135	396	1.552	0.123001848	1
ENSBTAG00000008135	*SLIRP*	1'713	1'249	1'463	1'484	1'442	-0.041	0.961212142	1
ENSBTAG00000008136	*SNW1*	4'888	3'709	4'258	4'233	4'283	0.017	0.982307943	1
ENSBTAG00000008137	*BT.90476*	761	971	890	659	1'121	0.767	0.358550227	1
ENSBTAG00000008138	*BT.87350*	8	14	12	7	16	1.222	0.772505073	1
ENSBTAG00000008139	*HOXA3*	50	128	96	43	148	1.771	0.190038899	1
ENSBTAG00000008140	*FAP*	10'709	4'030	6'964	9'274	4'653	-0.995	0.198755375	1
ENSBTAG00000008142	*IFIH1*	411	204	296	356	236	-0.596	0.540674092	1
ENSBTAG00000008143	*BT.52411*	1'266	741	976	1'096	856	-0.358	0.666282061	1
ENSBTAG00000008144	*PPIL6*	58	33	44	50	38	-0.399	0.838832744	1
ENSBTAG00000008145	*SMPD2*	1'198	1'281	1'258	1'037	1'479	0.512	0.528395289	1
ENSBTAG00000008147	*MICAL1*	2'762	6'205	4'778	2'392	7'165	1.583	0.044736803	1
ENSBTAG00000008148	*GCM-1*	1	1	1	1	1	0.415	1	1
ENSBTAG00000008150	*PKIA*	2'149	606	1'280	1'861	700	-1.411	0.086636572	1
ENSBTAG00000008151	*MAP3K3*	1'394	1'752	1'615	1'207	2'023	0.745	0.353146572	1
ENSBTAG00000008153	*CAMSAP2*	2'866	2'033	2'415	2'482	2'348	-0.080	0.919697161	1
ENSBTAG00000008154	*LIMD2*	810	1'151	1'015	701	1'329	0.922	0.265522833	1
ENSBTAG00000008158	*CCDC68*	52	50	51	45	58	0.358	0.841743321	1
ENSBTAG00000008159	*ZC2HC1B*	91	20	51	79	23	-1.771	0.314185735	1
ENSBTAG00000008160	*MBOAT2*	653	1'088	911	566	1'256	1.152	0.169740071	1
ENSBTAG00000008161	*CLCA1*	5	4	4	4	5	0.093	1	1
ENSBTAG00000008165	*ITGA2B*	510	327	410	442	378	-0.226	0.807132558	1
ENSBTAG00000008167	*BT.85213*	13'897	7'395	10'287	12'035	8'539	-0.495	0.518372911	1
ENSBTAG00000008168	*STARD6*	7	0	3	6	0		0.769774961	1
ENSBTAG00000008169	*C18ORF54*	205	132	165	178	152	-0.220	0.848600513	1
ENSBTAG00000008170	*POLA1*	1'267	1'191	1'236	1'097	1'375	0.326	0.688163156	1
ENSBTAG00000008172	*EGLN3*	942	564	734	816	651	-0.325	0.702935624	1
ENSBTAG00000008173	*TRIM13*	1'730	1'158	1'418	1'498	1'337	-0.164	0.839854099	1
ENSBTAG00000008175	*ZNF713*	503	325	405	436	375	-0.215	0.816913512	1
ENSBTAG00000008176	*KCNRG*	16	2	8	14	2	-2.585	0.65347607	1
ENSBTAG00000008180	*BT.42242*	607	466	532	526	538	0.034	0.97110917	1
ENSBTAG00000008181	*CHAF1A*	741	1'287	1'064	642	1'486	1.212	0.144206148	1
ENSBTAG00000008182	*FOSB*	132	29	74	114	33	-1.771	0.237218691	1
ENSBTAG00000008183	*MBTPS2*	439	224	319	380	259	-0.556	0.562287145	1
ENSBTAG00000008184	*CCT6A*	3'758	3'393	3'586	3'255	3'918	0.268	0.730027918	1
ENSBTAG00000008185	*BT.20228*	404	299	348	350	345	-0.019	0.988015448	1
ENSBTAG00000008186	*UBXN6*	1'513	1'618	1'589	1'310	1'868	0.512	0.522681874	1
ENSBTAG00000008188	*C16ORF52*	190	161	175	165	186	0.176	0.875934582	1
ENSBTAG00000008190	*SUMF2*	986	832	907	854	961	0.170	0.838176561	1
ENSBTAG00000008191	*SLC39A7*	12'268	14'160	13'487	10'624	16'351	0.622	0.415870368	1
ENSBTAG00000008192	*LPPR2*	1'078	307	644	934	354	-1.397	0.109548097	1
ENSBTAG00000008193	*MICALCL*	17	16	17	15	18	0.328	0.935078883	1
ENSBTAG00000008195	*PHKG1*	221	183	201	191	211	0.143	0.895906714	1
ENSBTAG00000008196	*SWSAP1*	225	177	200	195	204	0.069	0.952344174	1
ENSBTAG00000008197	*EPOR*	59	34	45	51	39	-0.380	0.844956551	1
ENSBTAG00000008198	*RGL3*	8	6	7	7	7	0.000	1	1
ENSBTAG00000008201	*CCDC151*	95	51	71	82	59	-0.482	0.753146549	1
ENSBTAG00000008202	*PRKCSH*	7'946	10'625	9'575	6'881	12'269	0.834	0.277930619	1
ENSBTAG00000008203	*SEPW1*	6'053	6'804	6'549	5'242	7'857	0.584	0.448024518	1
ENSBTAG00000008204	*C9ORF41*	282	171	221	244	197	-0.307	0.77001312	1
ENSBTAG00000008213	*ATMIN*	1'717	908	1'268	1'487	1'048	-0.504	0.535422911	1

Ensembl gene ID	geneName	counts wildtype horn bud	counts polled horn bud	baseMean	baseMean wildtype horn bud	baseMean polled horn bud	log2FoldChange	pval	padj
ENSBTAG00000008216	*RRM2*	2'970	2'433	2'691	2'572	2'809	0.127	0.87053164	1
ENSBTAG00000008218	*BT.45355*	3'211	1'796	2'427	2'781	2'074	-0.423	0.591238969	1
ENSBTAG00000008219	*RBM7*	2'204	1'591	1'873	1'909	1'837	-0.055	0.945991802	1
ENSBTAG00000008220	*C15H11orf71*	54	66	61	47	76	0.705	0.658910011	1
ENSBTAG00000008222	*FAM86A*	624	406	505	540	469	-0.205	0.819363262	1
ENSBTAG00000008223	*NLRC4*	51	14	30	44	16	-1.450	0.524935296	1
ENSBTAG00000008224	*PAIP2*	9'413	6'198	7'654	8'152	7'157	-0.188	0.807215068	1
ENSBTAG00000008227	*BT.35250*	52	34	42	45	39	-0.198	0.929711843	1
ENSBTAG00000008228	*BEND6*	458	233	333	397	269	-0.560	0.556184649	1
ENSBTAG00000008232	*BT.100456*	1'185	2'321	1'853	1'026	2'680	1.385	0.086032951	1
ENSBTAG00000008236	*OR51E2*	2	0	1	2	0		0.974934741	1
ENSBTAG00000008237	*TOLLIP*	2'900	3'678	3'379	2'511	4'247	0.758	0.331754641	1
ENSBTAG00000008240	*GALNTL2*	41	16	27	36	18	-0.943	0.700293423	1
ENSBTAG00000008241	*MRPS5*	1'356	1'461	1'431	1'174	1'687	0.523	0.516392387	1
ENSBTAG00000008243	*LRRC40*	1'155	672	888	1'000	776	-0.366	0.661271387	1
ENSBTAG00000008248	*DMD*	849	693	768	735	800	0.122	0.88555585	1
ENSBTAG00000008250	*SPRY4*	324	210	262	281	242	-0.211	0.835925676	1
ENSBTAG00000008251	*SNRPN*	5'425	3'410	4'318	4'698	3'938	-0.255	0.742667846	1
ENSBTAG00000008253	*EXPH5*	66	27	44	57	31	-0.874	0.640748997	1
ENSBTAG00000008255	*BT.87490*	15	15	15	13	17	0.415	0.918564425	1
ENSBTAG00000008257	*protein_coding*	361	597	501	313	689	1.141	0.202081063	1
ENSBTAG00000008259	*IRG1*	6	4	5	5	5	-0.170	1	1
ENSBTAG00000008260	*UBE2W*	1'182	887	1'024	1'024	1'024	0.001	0.999912765	1
ENSBTAG00000008267	*C2ORF29*	1'657	1'243	1'435	1'435	1'435	0.000	1	1
ENSBTAG00000008269	*RNF149*	1'290	585	896	1'117	675	-0.726	0.385103058	1
ENSBTAG00000008271	*C12H13orf33*	3'365	1'184	2'141	2'914	1'367	-1.092	0.170892552	1
ENSBTAG00000008274	*MUC5B*	27	16	21	23	18	-0.340	0.919037185	1
ENSBTAG00000008275	*GREB1L*	144	40	85	125	46	-1.433	0.30755305	1
ENSBTAG00000008278	*TMEM187*	103	36	65	89	42	-1.102	0.479658881	1
ENSBTAG00000008279	*FRMD4A*	2'320	2'202	2'276	2'009	2'543	0.340	0.665970772	1
ENSBTAG00000008280	*HNF4G*	40	1	18	35	1	-4.907	0.190349621	1
ENSBTAG00000008283	*BT.67771*	564	283	408	488	327	-0.580	0.527825238	1
ENSBTAG00000008285	*BT.106609*	5'109	2'995	3'941	4'425	3'458	-0.355	0.647418951	1
ENSBTAG00000008287	*YLPM1*	5'309	3'325	4'219	4'598	3'839	-0.260	0.737685745	1
ENSBTAG00000008288	*BT.77481*	838	444	619	726	513	-0.501	0.563002281	1
ENSBTAG00000008289	*ZBTB4*	2'185	2'024	2'115	1'892	2'337	0.305	0.699469777	1
ENSBTAG00000008291	*PROCR*	179	136	156	155	157	0.019	0.99261102	1
ENSBTAG00000008292	*SNRPD1*	2'292	1'399	1'800	1'985	1'615	-0.297	0.709706592	1
ENSBTAG00000008293	*GPCPD1*	2'185	587	1'285	1'892	678	-1.481	0.072547744	1
ENSBTAG00000008294	*KCNJ2*	405	108	238	351	125	-1.492	0.148182676	1
ENSBTAG00000008296	*N6AMT2*	1'389	1'016	1'188	1'203	1'173	-0.036	0.966455553	1
ENSBTAG00000008299	*IL20RB*	899	587	728	779	678	-0.200	0.815539896	1
ENSBTAG00000008300	*BT.23418*	146'865	137'233	142'826	127'189	158'463	0.317	0.675246567	1
ENSBTAG00000008301	*WNK2*	917	1'192	1'085	794	1'376	0.793	0.334625002	1
ENSBTAG00000008302	*RINT1*	1'916	1'166	1'503	1'659	1'346	-0.301	0.708293746	1
ENSBTAG00000008303	*FKBP1A*	7'676	8'202	8'059	6'648	9'471	0.511	0.505442072	1
ENSBTAG00000008306	*BT.67498*	1'918	1'347	1'608	1'661	1'555	-0.095	0.907023509	1
ENSBTAG00000008307	*PLCH1*	127	71	96	110	82	-0.424	0.754764285	1
ENSBTAG00000008308	*protein_coding*	7	4	5	6	5	-0.392	0.98714753	1
ENSBTAG00000008309	*ATP13A2*	1'904	1'763	1'842	1'649	2'036	0.304	0.701718289	1
ENSBTAG00000008310	*TMEM9B*	2'337	1'786	2'043	2'024	2'062	0.027	0.972642549	1
ENSBTAG00000008313	*KLF15*	106	128	120	92	148	0.687	0.576975524	1
ENSBTAG00000008314	*SDHB*	4'190	4'279	4'285	3'629	4'941	0.445	0.56487447	1
ENSBTAG00000008318	*AIPL1*	7	4	5	6	5	-0.392	0.98714753	1
ENSBTAG00000008319	*BT.88816*	45	49	48	39	57	0.538	0.76707008	1
ENSBTAG00000008320	*Sep 05*	4'039	3'157	3'572	3'498	3'645	0.060	0.938513496	1
ENSBTAG00000008321	*RNF8*	2'083	1'389	1'704	1'804	1'604	-0.170	0.832797958	1
ENSBTAG00000008324	*protein_coding*	1	0	0	1	0		1	1
ENSBTAG00000008325	*pseudogene*	84	171	135	73	197	1.441	0.227733097	1
ENSBTAG00000008327	*CD300LB*	6	5	5	5	6	0.152	1	1
ENSBTAG00000008328	*protein_coding*	0	3	2	0	3	Inf	0.878646179	1
ENSBTAG00000008329	*CYTIP*	101	32	62	87	37	-1.243	0.435337138	1
ENSBTAG00000008330	*RNF19B*	1'508	997	1'229	1'306	1'151	-0.182	0.824192983	1
ENSBTAG00000008331	*TMEM54*	146	117	131	126	135	0.096	0.941944749	1
ENSBTAG00000008333	*ETV4*	293	423	371	254	488	0.945	0.311135281	1
ENSBTAG00000008336	*HPCA*	154	46	93	133	53	-1.328	0.327527208	1
ENSBTAG00000008338	*PLCB1*	554	203	357	480	234	-1.033	0.272811869	1
ENSBTAG00000008339	*DHX8*	4'041	4'348	4'260	3'500	5'021	0.521	0.501363506	1
ENSBTAG00000008340	*ATOX1*	2'587	1'238	1'835	2'240	1'430	-0.648	0.416488035	1
ENSBTAG00000008341	*ARSB*	4'847	3'558	4'153	4'198	4'108	-0.031	0.969086842	1
ENSBTAG00000008342	*TMPRSS13*	348	429	398	301	495	0.717	0.435596203	1
ENSBTAG00000008343	*protein_coding*	162	57	103	140	66	-1.092	0.402472908	1

Ensembl gene ID	geneName	counts wildtype horn bud	counts polled horn bud	baseMean	baseMean wildtype horn bud	baseMean polled horn bud	log2FoldChange	pval	padj
ENSBTAG00000008346	*CRYBG3*	1'798	2'040	1'956	1'557	2'356	0.597	0.45172542	1
ENSBTAG00000008347	*MRPL48*	772	609	686	669	703	0.073	0.932953627	1
ENSBTAG00000008348	*C20ORF112*	500	408	452	433	471	0.122	0.893939365	1
ENSBTAG00000008349	*ZNF311*	393	207	290	340	239	-0.510	0.602243999	1
ENSBTAG00000008350	*BT.51832*	1'361	1'723	1'584	1'179	1'990	0.755	0.346958268	1
ENSBTAG00000008351	*C12ORF60*	17	4	10	15	5	-1.672	0.733083131	1
ENSBTAG00000008352	*BT.28487*	43	56	51	37	65	0.796	0.646425744	1
ENSBTAG00000008353	*CDKN1A*	1'208	1'859	1'596	1'046	2'147	1.037	0.198490956	1
ENSBTAG00000008355	*CPSF1*	5'309	7'491	6'624	4'598	8'650	0.912	0.238201041	1
ENSBTAG00000008361	*PON2*	2'034	1'412	1'696	1'761	1'630	-0.112	0.890104485	1
ENSBTAG00000008362	*CTDSP1*	2'809	3'686	3'344	2'433	4'256	0.807	0.301848278	1
ENSBTAG00000008363	*EXD1*	14	33	25	12	38	1.652	0.509598682	1
ENSBTAG00000008365	*ANKRD34A*	70	20	42	61	23	-1.392	0.468172319	1
ENSBTAG00000008366	*STAC2*	75	95	87	65	110	0.756	0.583443769	1
ENSBTAG00000008367	*LIX1L*	7'360	4'385	5'719	6'374	5'063	-0.332	0.666966936	1
ENSBTAG00000008368	*protein_coding*	2	0	1	2	0		0.974934741	1
ENSBTAG00000008369	*RBM8A*	2'927	2'248	2'565	2'535	2'596	0.034	0.965000469	1
ENSBTAG00000008370	*GNRHR2*	1'837	1'327	1'562	1'591	1'532	-0.054	0.94762173	1
ENSBTAG00000008371	*FBXL20*	1'038	743	878	899	858	-0.067	0.937698033	1
ENSBTAG00000008372	*PEX11B*	982	694	826	850	801	-0.086	0.920608259	1
ENSBTAG00000008373	*ITGA10*	2'004	594	1'211	1'736	686	-1.339	0.104598715	1
ENSBTAG00000008374	*AQP2*	2	0	1	2	0		0.974934741	1
ENSBTAG00000008376	*SMRP1*	11	13	12	10	15	0.656	0.880852037	1
ENSBTAG00000008378	*FEM1B*	2'088	1'261	1'632	1'808	1'456	-0.313	0.696815354	1
ENSBTAG00000008380	*BT.56405*	7'936	8'054	8'086	6'873	9'300	0.436	0.569008623	1
ENSBTAG00000008382	*C12ORF26*	131	87	107	113	100	-0.175	0.898304474	1
ENSBTAG00000008384	*SLC25A47*	5	3	4	4	3	-0.322	1	1
ENSBTAG00000008385	*BT.26807*	349	662	533	302	764	1.339	0.132838706	1
ENSBTAG00000008386	*CLPX*	2'584	1'876	2'202	2'238	2'166	-0.047	0.953852648	1
ENSBTAG00000008388	*BT.91058*	906	602	740	785	695	-0.175	0.838491513	1
ENSBTAG00000008389	*HTRA1*	25'756	34'722	31'199	22'305	40'094	0.846	0.268214543	1
ENSBTAG00000008390	*BT.88705*	939	1'026	999	813	1'185	0.543	0.510626315	1
ENSBTAG00000008394	*MYL3*	15	19	17	13	22	0.756	0.816016334	1
ENSBTAG00000008395	*APBA3*	1'308	1'284	1'308	1'133	1'483	0.388	0.631221476	1
ENSBTAG00000008396	*BT.30696*	1'366	1'403	1'402	1'183	1'620	0.454	0.573549927	1
ENSBTAG00000008397	*BT.39755*	446	193	305	386	223	-0.793	0.412414083	1
ENSBTAG00000008399	*ATRIP*	515	660	604	446	762	0.773	0.373417894	1
ENSBTAG00000008401	*PFKFB3*	1'120	885	996	970	1'022	0.075	0.927750032	1
ENSBTAG00000008403	*ROCK1*	3'854	1'413	2'485	3'338	1'632	-1.033	0.19245167	1
ENSBTAG00000008406	*TREX1*	398	390	398	345	450	0.386	0.674935934	1
ENSBTAG00000008409	*MYC*	1'843	1'009	1'381	1'596	1'165	-0.454	0.574621879	1
ENSBTAG00000008410	*OVOL2*	187	320	266	162	370	1.190	0.232988969	1
ENSBTAG00000008411	*PLEKHO2*	1'254	1'269	1'276	1'086	1'465	0.432	0.593840407	1
ENSBTAG00000008412	*BCL7C*	1'022	941	986	885	1'087	0.296	0.719963585	1
ENSBTAG00000008414	*ZFR*	4'000	2'403	3'119	3'464	2'775	-0.320	0.682241493	1
ENSBTAG00000008416	*ABHD5*	647	357	486	560	412	-0.443	0.620735628	1
ENSBTAG00000008417	*BT.48523*	3'160	1'606	2'296	2'737	1'854	-0.561	0.477289372	1
ENSBTAG00000008419	*PDE2A*	3'157	1'584	2'282	2'734	1'829	-0.580	0.463026119	1
ENSBTAG00000008420	*BT.37469*	174	20	87	151	23	-2.706	0.063332563	1
ENSBTAG00000008421	*SCRIB*	3'745	5'059	4'542	3'243	5'842	0.849	0.274501386	1
ENSBTAG00000008423	*TIMM22*	603	550	579	522	635	0.282	0.746122521	1
ENSBTAG00000008424	*ABR*	8'082	9'412	8'934	6'999	10'868	0.635	0.407945898	1
ENSBTAG00000008426	*pseudogene*	1	3	2	1	3	2.000	0.933774563	1
ENSBTAG00000008428	*BT.20327*	73	65	69	63	75	0.248	0.876590883	1
ENSBTAG00000008429	*BT.103632*	6'178	11'961	9'581	5'350	13'811	1.368	0.078318094	1
ENSBTAG00000008430	*RANBP3L*	76	4	35	66	5	-3.833	0.098743301	1
ENSBTAG00000008431	*MOGAT1*	0	3	2	0	3	Inf	0.878646179	1
ENSBTAG00000008432	*NUP98*	3'976	3'206	3'573	3'443	3'702	0.104	0.892609077	1
ENSBTAG00000008433	*PGAP2*	2'352	1'856	2'090	2'037	2'143	0.073	0.925931968	1
ENSBTAG00000008434	*GCAT*	558	359	449	483	415	-0.221	0.808500324	1
ENSBTAG00000008435	*BT.41732*	3'112	2'303	2'677	2'695	2'659	-0.019	0.981573755	1
ENSBTAG00000008436	*CDC25B*	1'133	1'347	1'268	981	1'555	0.665	0.41329601	1
ENSBTAG00000008437	*PCDH12*	1'333	1'151	1'242	1'154	1'329	0.203	0.802282408	1
ENSBTAG00000008438	*BT.28270*	3'835	1'874	2'743	3'321	2'164	-0.618	0.431372331	1
ENSBTAG00000008439	*C20ORF29*	904	922	924	783	1'065	0.443	0.593107588	1
ENSBTAG00000008441	*SOCS3*	149	103	124	129	119	-0.118	0.930386213	1
ENSBTAG00000008442	*NSF*	1'232	1'288	1'277	1'067	1'487	0.479	0.554452471	1
ENSBTAG00000008443	*LARP1*	2'989	3'473	3'299	2'589	4'010	0.632	0.418138541	1
ENSBTAG00000008446	*TYW1*	208	268	245	180	309	0.781	0.439615439	1
ENSBTAG00000008448	*MEIS1*	1'905	1'565	1'728	1'650	1'807	0.131	0.86891072	1
ENSBTAG00000008449	*DNAH14*	5	1	3	4	1	-1.907	0.9188325	1
ENSBTAG00000008452	*BT.22104*	16	0	7	14	0		0.463690882	1

Ensembl gene ID	geneName	counts wildtype horn bud	counts polled horn bud	baseMean	baseMean wildtype horn bud	baseMean polled horn bud	log2FoldChange	pval	padj
ENSBTAG00000008453	*LBR*	1'027	1'226	1'153	889	1'416	0.671	0.412128712	1
ENSBTAG00000008457	*SLU7*	2'687	1'829	2'219	2'327	2'112	-0.140	0.860255111	1
ENSBTAG00000008461	*LSG1*	2'505	1'491	1'946	2'169	1'722	-0.333	0.674935164	1
ENSBTAG00000008462	*FAM43A*	545	323	422	472	373	-0.340	0.710813239	1
ENSBTAG00000008463	*SLC22A14*	2	0	1	2	0		0.974934741	1
ENSBTAG00000008464	*ABCB9*	70	58	64	61	67	0.144	0.935655725	1
ENSBTAG00000008465	*ACS*	1'633	1'138	1'364	1'414	1'314	-0.106	0.897021529	1
ENSBTAG00000008466	*CCNB1IP1*	210	87	141	182	100	-0.856	0.465661674	1
ENSBTAG00000008467	*HENMT1*	275	82	166	238	95	-1.331	0.236829766	1
ENSBTAG00000008468	*AGAP3*	2'318	3'449	2'995	2'007	3'983	0.988	0.208804531	1
ENSBTAG00000008470	*BT.47690*	1'627	877	1'211	1'409	1'013	-0.477	0.559138787	1
ENSBTAG00000008471	*MX2*	37	10	22	32	12	-1.472	0.593518052	1
ENSBTAG00000008472	*CRNKL1*	1'598	1'453	1'531	1'384	1'678	0.278	0.728800503	1
ENSBTAG00000008479	*CXCL13*	68	12	36	59	14	-2.087	0.318514895	1
ENSBTAG00000008480	*CTBP1*	3'334	3'846	3'664	2'887	4'441	0.621	0.424591123	1
ENSBTAG00000008482	*BT.65645*	19'480	10'033	14'228	16'870	11'585	-0.542	0.478453229	1
ENSBTAG00000008483	*CALCRL*	2'435	1'023	1'645	2'109	1'181	-0.836	0.298123722	1
ENSBTAG00000008484	*TMEM161B*	1'213	578	859	1'050	667	-0.654	0.435052074	1
ENSBTAG00000008485	*TCEAL1*	1'716	1'153	1'409	1'486	1'331	-0.159	0.845248646	1
ENSBTAG00000008487	*VPS37D*	394	259	320	341	299	-0.190	0.84530094	1
ENSBTAG00000008490	*CRYZL1*	1'615	969	1'259	1'399	1'119	-0.322	0.692611959	1
ENSBTAG00000008491	*pseudogene*	275	426	365	238	492	1.046	0.264021964	1
ENSBTAG00000008492	*ZMYM3*	5'693	5'961	5'907	4'930	6'883	0.481	0.531650008	1
ENSBTAG00000008493	*AQP3*	5'721	4'644	5'158	4'955	5'362	0.114	0.881818421	1
ENSBTAG00000008494	*TMEM171*	4	3	3	3	3	0.000	1	1
ENSBTAG00000008497	*RGS14*	76	25	47	66	29	-1.189	0.510019275	1
ENSBTAG00000008498	*ALYREF*	1'891	1'792	1'853	1'638	2'069	0.337	0.670727988	1
ENSBTAG00000008499	*TROAP*	1'078	1'164	1'139	934	1'344	0.526	0.519941459	1
ENSBTAG00000008501	*FOXD3*	113	35	69	98	40	-1.276	0.402558895	1
ENSBTAG00000008507	*SPHK1*	251	265	262	217	306	0.493	0.620167138	1
ENSBTAG00000008509	*SLC38A3*	933	1'932	1'519	808	2'231	1.465	0.072618116	1
ENSBTAG00000008510	*FBXO42*	1'014	912	966	878	1'053	0.262	0.751290909	1
ENSBTAG00000008512	*BT.55466*	101	17	54	87	20	-2.156	0.215552149	1
ENSBTAG00000008513	*C1ORF186*	34	8	19	29	9	-1.672	0.574142194	1
ENSBTAG00000008517	*protein_coding*	10	64	41	9	74	3.093	0.126842851	1
ENSBTAG00000008518	*BT.12647*	2'163	2'772	2'537	1'873	3'201	0.773	0.326411768	1
ENSBTAG00000008519	*protein_coding*	1	0	0	1	0		1	1
ENSBTAG00000008520	*NFIC*	1'958	2'934	2'542	1'696	3'388	0.999	0.206514591	1
ENSBTAG00000008523	*TFE3*	2'548	2'965	2'815	2'207	3'424	0.634	0.41865111	1
ENSBTAG00000008525	*LRIG2*	488	278	372	423	321	-0.397	0.671213295	1
ENSBTAG00000008527	*BT.38355*	43'335	17'963	29'136	37'529	20'742	-0.855	0.263498734	1
ENSBTAG00000008528	*SLC25A1*	3'323	4'443	4'004	2'878	5'130	0.834	0.284096488	1
ENSBTAG00000008530	*SLC36A4*	169	101	131	146	117	-0.328	0.788493949	1
ENSBTAG00000008533	*GLCCI1*	258	142	194	223	164	-0.446	0.678599284	1
ENSBTAG00000008535	*SOCS7*	147	226	194	127	261	1.036	0.333447402	1
ENSBTAG00000008537	*C9ORF25*	178	161	170	154	186	0.270	0.809592179	1
ENSBTAG00000008538	*DNAI1*	43	12	26	37	14	-1.426	0.569445429	1
ENSBTAG00000008539	*ENHO*	768	749	765	665	865	0.379	0.653615301	1
ENSBTAG00000008541	*MGST1*	2'212	1'407	1'770	1'916	1'625	-0.238	0.766315451	1
ENSBTAG00000008542	*protein_coding*	210	82	138	182	95	-0.942	0.425474361	1
ENSBTAG00000008543	*GTF2H5*	1'253	913	1'070	1'085	1'054	-0.042	0.961424168	1
ENSBTAG00000008545	*ATF3*	350	145	235	303	167	-0.856	0.402663152	1
ENSBTAG00000008546	*BT.50244*	1	4	3	1	5	2.415	0.884888347	1
ENSBTAG00000008548	*BT.61805*	4'073	2'737	3'344	3'527	3'160	-0.158	0.839563165	1
ENSBTAG00000008550	*BT.20772*	49	10	27	42	12	-1.878	0.442313125	1
ENSBTAG00000008551	*TUSC1*	132	100	115	114	115	0.014	0.998110968	1
ENSBTAG00000008552	*PLXNA3*	5'442	7'202	6'515	4'713	8'316	0.819	0.28857148	1
ENSBTAG00000008553	*B4GALNT3*	933	1'438	1'234	808	1'660	1.039	0.204193374	1
ENSBTAG00000008554	*NINJ2*	71	31	49	61	36	-0.781	0.663129412	1
ENSBTAG00000008555	*P2RY10*	28	7	16	24	8	-1.585	0.635899565	1
ENSBTAG00000008556	*POU2F2*	46	15	29	40	17	-1.202	0.609435046	1
ENSBTAG00000008557	*pseudogene*	8'112	12'956	10'993	7'025	14'960	1.091	0.15750016	1
ENSBTAG00000008562	*POGLUT1*	545	417	477	472	482	0.029	0.976147961	1
ENSBTAG00000008564	*BT.54173*	0	2	1	0	2	Inf	0.939077559	1
ENSBTAG00000008567	*DLEC1*	358	123	226	310	142	-1.126	0.276990103	1
ENSBTAG00000008568	*BT.66004*	11	10	11	10	12	0.278	0.971523508	1
ENSBTAG00000008570	*RPS14*	71'639	57'728	64'350	62'041	66'659	0.104	0.890741868	1
ENSBTAG00000008573	*ZFP36*	1'785	1'334	1'543	1'546	1'540	-0.005	0.996543348	1
ENSBTAG00000008575	*CGNL1*	3'457	4'108	3'869	2'994	4'744	0.664	0.393039975	1
ENSBTAG00000008576	*PLEKHD1*	9	8	9	8	9	0.245	0.986825286	1
ENSBTAG00000008577	*GRSF1*	3'223	2'095	2'605	2'791	2'419	-0.206	0.793256396	1
ENSBTAG00000008578	*LSM4*	1'360	1'316	1'349	1'178	1'520	0.368	0.648898422	1

Ensembl gene ID	geneName		counts wildtype horn bud	counts polled horn bud	baseMean	baseMean wildtype horn bud	baseMean polled horn bud	log2FoldChange	pval	padj
ENSBTAG00000008579	*RCC2*		2'332	5'130	3'972	2'020	5'924	1.552	0.049638741	1
ENSBTAG00000008583	*BT.105215*		494	623	574	428	719	0.750	0.390738262	1
ENSBTAG00000008584	*CLN8*		306	316	315	265	365	0.461	0.630183855	1
ENSBTAG00000008585	*ARHGEF10*		2'685	2'357	2'523	2'325	2'722	0.227	0.771948768	1
ENSBTAG00000008587	*GSTT3*		34	36	36	29	42	0.497	0.816294415	1
ENSBTAG00000008590	*BT.24734*		2'949	2'480	2'709	2'554	2'864	0.165	0.832572328	1
ENSBTAG00000008591	*CAMTA2*		4'684	4'635	4'704	4'056	5'352	0.400	0.604458623	1
ENSBTAG00000008592	*BT.54393*		255	153	199	221	177	-0.322	0.765021622	1
ENSBTAG00000008593	*INCA1*		92	46	66	80	53	-0.585	0.708403414	1
ENSBTAG00000008595	*PPHLN1*		1'936	1'281	1'578	1'677	1'479	-0.181	0.822575497	1
ENSBTAG00000008596	*BT.57449*		3'716	1'671	2'574	3'218	1'930	-0.738	0.348992272	1
ENSBTAG00000008597	*SLC45A4*		183	255	226	158	294	0.894	0.385263988	1
ENSBTAG00000008600	*BT.87724*		664	468	558	575	540	-0.090	0.921091697	1
ENSBTAG00000008603	*BT.31012*		51	18	32	44	21	-1.087	0.619948893	1
ENSBTAG00000008605	*DNAH2*		6	2	4	5	2	-1.170	0.934316631	1
ENSBTAG00000008606	*EXOC3*		3'713	4'574	4'249	3'216	5'282	0.716	0.356448471	1
ENSBTAG00000008607	*ARID3A*		272	241	257	236	278	0.240	0.811450576	1
ENSBTAG00000008609		*Sep 08*	3'276	3'977	3'715	2'837	4'592	0.695	0.372079878	1
ENSBTAG00000008611	*IGFBP4*		35'671	19'327	26'604	30'892	22'317	-0.469	0.538345111	1
ENSBTAG00000008613	*TCP11L1*		597	441	513	517	509	-0.022	0.983477751	1
ENSBTAG00000008614	*BT.66800*		108	90	99	94	104	0.152	0.914280881	1
ENSBTAG00000008617	*BT.61678*		2'296	1'375	1'788	1'988	1'588	-0.325	0.684261009	1
ENSBTAG00000008619	*SAMD8*		302	229	263	262	264	0.016	0.990946183	1
ENSBTAG00000008621	*PRKAR1A*		6'518	5'666	6'094	5'645	6'543	0.213	0.78131904	1
ENSBTAG00000008624	*MESDC1*		649	800	743	562	924	0.717	0.39824399	1
ENSBTAG00000008625	*bta-mir-2901*		439	313	371	380	361	-0.073	0.941101374	1
ENSBTAG00000008626	*KCNN1*		112	249	192	97	288	1.568	0.148464124	1
ENSBTAG00000008629	*MTFR1*		1'929	1'618	1'769	1'671	1'868	0.161	0.839154383	1
ENSBTAG00000008631	*CORO1A*		708	361	515	613	417	-0.557	0.530323772	1
ENSBTAG00000008632	*BOLA2B*		1'072	667	849	928	770	-0.270	0.748625937	1
ENSBTAG00000008633	*SLX1A*		1'255	1'263	1'273	1'087	1'458	0.424	0.600717987	1
ENSBTAG00000008634	*WBP2*		2'252	2'580	2'465	1'950	2'979	0.611	0.437212511	1
ENSBTAG00000008635	*SULT1A1*		7'855	10'796	9'634	6'803	12'466	0.874	0.256006166	1
ENSBTAG00000008636	*PDE4B*		1'399	322	792	1'212	372	-1.704	0.047904549	1
ENSBTAG00000008638	*FAM110A*		291	449	385	252	518	1.041	0.262287842	1
ENSBTAG00000008639	*TRIML1*		0	2	1	0	2	Inf	0.939077559	1
ENSBTAG00000008641	*GNAT3*		4	0	2	3	0		0.89545886	1
ENSBTAG00000008642	*POLR1D*		3'163	3'559	3'424	2'739	4'110	0.585	0.452423338	1
ENSBTAG00000008644	*KCNK4*		229	318	283	198	367	0.889	0.364721509	1
ENSBTAG00000008645	*ESRRA*		810	958	904	701	1'106	0.657	0.430082529	1
ENSBTAG00000008646	*TRMT112*		1'419	1'624	1'552	1'229	1'875	0.610	0.447388982	1
ENSBTAG00000008647	*KLHL1*		40	0	17	35	0		0.116452486	1
ENSBTAG00000008648	*PRDX5*		3'831	3'591	3'732	3'318	4'147	0.322	0.678135271	1
ENSBTAG00000008649	*ETV3*		472	554	524	409	640	0.646	0.464206545	1
ENSBTAG00000008650	*CAMK1D*		233	112	166	202	129	-0.642	0.566790051	1
ENSBTAG00000008654	*HOOK2*		1'289	1'155	1'225	1'116	1'334	0.257	0.751997211	1
ENSBTAG00000008656	*KBTBD6*		120	63	88	104	73	-0.515	0.7118111	1
ENSBTAG00000008661	*C11ORF52*		13	17	15	11	20	0.802	0.820295848	1
ENSBTAG00000008664	*EIF2B2*		1'686	1'517	1'606	1'460	1'752	0.263	0.742403685	1
ENSBTAG00000008665	*DIXDC1*		541	557	556	469	643	0.457	0.601573727	1
ENSBTAG00000008672	*GABRD*		43	45	45	37	52	0.481	0.799630016	1
ENSBTAG00000008673	*ICA1L*		86	102	96	74	118	0.661	0.619520702	1
ENSBTAG00000008674	*FAM136A*		1'905	1'676	1'793	1'650	1'935	0.230	0.771989499	1
ENSBTAG00000008676	*SRRM4*		15	1	7	13	1	-3.492	0.607205542	1
ENSBTAG00000008682	*TLR3*		1'259	724	963	1'090	836	-0.383	0.64437281	1
ENSBTAG00000008683	*LDHA*		2'704	1'804	2'212	2'342	2'083	-0.169	0.831485935	1
ENSBTAG00000008684	*protein_coding*		11	1	5	10	1	-3.044	0.722857941	1
ENSBTAG00000008685	*BT.100304*		734	1'133	972	636	1'308	1.041	0.211002071	1
ENSBTAG00000008686	*SLC46A3*		53	48	51	46	55	0.272	0.883445432	1
ENSBTAG00000008688	*BT.34222*		2'044	2'712	2'451	1'770	3'132	0.823	0.29697668	1
ENSBTAG00000008690	*SLC6A9*		1'076	919	997	932	1'061	0.187	0.820315397	1
ENSBTAG00000008692	*UQCR11*		2'153	2'011	2'093	1'865	2'322	0.317	0.688394564	1
ENSBTAG00000008693	*EFCAB1*		92	71	81	80	82	0.041	0.985892645	1
ENSBTAG00000008695	*TCF3*		3'164	4'002	3'681	2'740	4'621	0.754	0.333212463	1
ENSBTAG00000008696	*BT.47865*		4'089	2'424	3'170	3'541	2'799	-0.339	0.664149488	1
ENSBTAG00000008699	*FEM1C*		2'579	1'767	2'137	2'233	2'040	-0.130	0.869888554	1
ENSBTAG00000008703	*EIF2AK2*		361	283	320	313	327	0.064	0.949333943	1
ENSBTAG00000008704	*FIGN*		94	44	66	81	51	-0.680	0.662834413	1
ENSBTAG00000008705	*BT.25997*		625	808	737	541	933	0.786	0.355349015	1
ENSBTAG00000008707	*SULT6B1*		32	14	22	28	16	-0.778	0.782869547	1
ENSBTAG00000008708	*BT.93891*		83	27	52	72	31	-1.205	0.487069052	1
ENSBTAG00000008709	*KDELC2*		1'216	531	833	1'053	613	-0.780	0.353803466	1

Ensembl gene ID	geneName	counts wildtype horn bud	counts polled horn bud	baseMean	baseMean wildtype horn bud	baseMean polled horn bud	log2FoldChange	pval	padj
ENSBTAG00000008710	KCNH1	30	10	19	26	12	-1.170	0.701297322	1
ENSBTAG00000008711	BOK	414	330	370	359	381	0.088	0.926801593	1
ENSBTAG00000008713	WDR21A	1'306	977	1'130	1'131	1'128	-0.004	0.998384439	1
ENSBTAG00000008714	CLSTN2	163	31	88	141	36	-1.979	0.159312055	1
ENSBTAG00000008716	SSB	4'354	2'242	3'180	3'771	2'589	-0.543	0.487666745	1
ENSBTAG00000008718	WBSCR17	544	531	542	471	613	0.380	0.665198418	1
ENSBTAG00000008719	PRKCD	718	698	714	622	806	0.374	0.659831846	1
ENSBTAG00000008720	C18H16orf78	1	0	0	1	0		1	1
ENSBTAG00000008721	MANSC1	1'027	586	783	889	677	-0.394	0.640936455	1
ENSBTAG00000008723	CCBE1	37	144	99	32	166	2.376	0.080948653	1
ENSBTAG00000008726	BT.40187	417	417	421	361	482	0.415	0.648646629	1
ENSBTAG00000008728	MKL2	1'124	958	1'040	973	1'106	0.184	0.822548078	1
ENSBTAG00000008729	BT.69011	3'892	2'591	3'181	3'371	2'992	-0.172	0.826253329	1
ENSBTAG00000008730	SFXN4	449	408	430	389	471	0.277	0.760911978	1
ENSBTAG00000008731	PRDX3	2'513	1'983	2'233	2'176	2'290	0.073	0.92573838	1
ENSBTAG00000008732	ZC3H12C	73	56	64	63	65	0.033	0.994144462	1
ENSBTAG00000008733	BT.62984	21'611	20'509	21'199	18'716	23'682	0.340	0.655080986	1
ENSBTAG00000008734	BT.42206	156	57	100	135	66	-1.037	0.430355418	1
ENSBTAG00000008735	VASH1	565	973	806	489	1'124	1.199	0.157575405	1
ENSBTAG00000008736	CUL1	2'942	2'715	2'841	2'548	3'135	0.299	0.70157725	1
ENSBTAG00000008737	DR1	6'132	4'388	5'189	5'310	5'067	-0.068	0.930865954	1
ENSBTAG00000008739	SAMD11	477	300	380	413	346	-0.254	0.786295791	1
ENSBTAG00000008743	ALDH2	9'173	4'029	6'298	7'944	4'652	-0.772	0.317821306	1
ENSBTAG00000008744	PDK2	1'136	1'309	1'248	984	1'512	0.620	0.445955253	1
ENSBTAG00000008747	DCXR	507	220	347	439	254	-0.789	0.40337803	1
ENSBTAG00000008752	NME5	48	13	28	42	15	-1.469	0.534305566	1
ENSBTAG00000008753	BRD8	4'266	2'816	3'473	3'694	3'252	-0.184	0.813589961	1
ENSBTAG00000008755	Mrz 07	7'271	2'842	4'789	6'297	3'282	-0.940	0.2268359	1
ENSBTAG00000008756	ELF3	230	247	242	199	285	0.518	0.609016609	1
ENSBTAG00000008758	KIF20A	1'888	2'238	2'110	1'635	2'584	0.660	0.404213386	1
ENSBTAG00000008759	CDC23	849	561	692	735	648	-0.183	0.832338173	1
ENSBTAG00000008761	TRAFD1	3'902	3'377	3'639	3'379	3'899	0.207	0.789779384	1
ENSBTAG00000008762	BT.33136	1'557	1'106	1'313	1'348	1'277	-0.078	0.924421701	1
ENSBTAG00000008764	SMYD5	1'417	1'893	1'707	1'227	2'186	0.833	0.298435142	1
ENSBTAG00000008765	C11H2ORF7	542	811	703	469	936	0.996	0.244793435	1
ENSBTAG00000008766	SLC9A1	1'158	1'922	1'611	1'003	2'219	1.146	0.155971965	1
ENSBTAG00000008771	MYEF2	769	366	544	666	423	-0.656	0.45635423	1
ENSBTAG00000008772	SMC2	1'437	1'025	1'214	1'244	1'184	-0.072	0.930742666	1
ENSBTAG00000008773	BT.91781	100	127	117	87	147	0.760	0.540767681	1
ENSBTAG00000008774	UROC1	334	482	423	289	557	0.944	0.301221997	1
ENSBTAG00000008777	BT.28236	14	15	15	12	17	0.515	0.896151294	1
ENSBTAG00000008779	ALOX15B	43	7	23	37	8	-2.204	0.419659754	1
ENSBTAG00000008783	HTR7	17	25	22	15	29	0.971	0.723825949	1
ENSBTAG00000008787	C13H10ORF38	1'917	697	1'232	1'660	805	-1.045	0.202413712	1
ENSBTAG00000008788	SLC13A2	12	1	6	10	1	-3.170	0.692545189	1
ENSBTAG00000008789	HMG20B	1'222	1'758	1'544	1'058	2'030	0.940	0.243890207	1
ENSBTAG00000008792	RNASE6	2'107	1'090	1'542	1'825	1'259	-0.536	0.505201952	1
ENSBTAG00000008793	RNASE1	6	2	4	5	2	-1.170	0.934316631	1
ENSBTAG00000008794	ATF6B	4'286	4'638	4'534	3'712	5'356	0.529	0.494127172	1
ENSBTAG00000008797	protein_coding	211	52	121	183	60	-1.606	0.197305825	1
ENSBTAG00000008798	LRRC48	48	17	31	42	20	-1.082	0.633001393	1
ENSBTAG00000008800	XKR8	226	291	266	196	336	0.780	0.431898574	1
ENSBTAG00000008801	ATPAF2	527	513	524	456	592	0.376	0.669856962	1
ENSBTAG00000008802	BT.105098	1'087	880	979	941	1'016	0.110	0.894184673	1
ENSBTAG00000008804	BT.52594	449	519	494	389	599	0.624	0.483060751	1
ENSBTAG00000008805	USP3	884	754	818	766	871	0.186	0.82526028	1
ENSBTAG00000008807	FBXL22	46	43	45	40	50	0.318	0.870987872	1
ENSBTAG00000008808	CCDC17	2'231	1'571	1'873	1'932	1'814	-0.091	0.910073939	1
ENSBTAG00000008809	BT.63447	562	218	369	487	252	-0.951	0.309432762	1
ENSBTAG00000008810	BT.39833	4'146	2'786	3'404	3'591	3'217	-0.158	0.839456127	1
ENSBTAG00000008812	PFDN4	2'629	1'089	1'767	2'277	1'257	-0.856	0.284949609	1
ENSBTAG00000008814	GPR124	4'770	6'501	5'819	4'131	7'507	0.862	0.265428683	1
ENSBTAG00000008815	WDR89	720	438	565	624	506	-0.302	0.731231177	1
ENSBTAG00000008816	TRIM2	11'300	5'704	8'186	9'786	6'586	-0.571	0.457408467	1
ENSBTAG00000008817	LAMA4	16'061	12'560	14'206	13'909	14'503	0.060	0.936347626	1
ENSBTAG00000008819	BT.50274	11	1	5	10	1	-3.044	0.722857941	1
ENSBTAG00000008821	SEPHS1	2'333	2'300	2'338	2'020	2'656	0.394	0.615973593	1
ENSBTAG00000008825	BT.27185	1'681	742	1'156	1'456	857	-0.765	0.350850936	1
ENSBTAG00000008826	BT.32989	256	179	214	222	207	-0.101	0.9278332	1
ENSBTAG00000008827	SPOCK2	2'996	5'059	4'218	2'595	5'842	1.171	0.134657335	1
ENSBTAG00000008828	BT.61706	226	597	443	196	689	1.816	0.049721657	1
ENSBTAG00000008832	CCL1	10	1	5	9	1	-2.907	0.754028015	1

Ensembl gene ID	geneName	counts wildtype horn bud	counts polled horn bud	baseMean	baseMean wildtype horn bud	baseMean polled horn bud	log2FoldChange	pval	padj
ENSBTAG00000008833	*APOM*	299	190	239	259	219	-0.239	0.817294872	1
ENSBTAG00000008835	*BAT4*	2'363	3'070	2'796	2'046	3'545	0.793	0.312862424	1
ENSBTAG00000008836	*ZNF782*	413	161	272	358	186	-0.944	0.341235038	1
ENSBTAG00000008837	*CSNK2B*	4'384	4'837	4'691	3'797	5'585	0.557	0.471410931	1
ENSBTAG00000008839	*FAM123B*	270	367	329	234	424	0.858	0.3674728	1
ENSBTAG00000008840	*HM13*	3'586	3'530	3'591	3'106	4'076	0.392	0.613326441	1
ENSBTAG00000008841	*DCAF15*	1'550	1'829	1'727	1'342	2'112	0.654	0.412948213	1
ENSBTAG00000008842	*JPH1*	440	267	345	381	308	-0.306	0.747858182	1
ENSBTAG00000008845	*BT.91042*	1'014	921	971	878	1'063	0.276	0.73819377	1
ENSBTAG00000008849	*SORT1*	1'510	698	1'057	1'308	806	-0.698	0.397112719	1
ENSBTAG00000008851	*BT.88266*	10	14	12	9	16	0.900	0.826937278	1
ENSBTAG00000008852	*SIGLEC10*	360	373	371	312	431	0.466	0.616110878	1
ENSBTAG00000008853	*HNRNPF*	17'984	10'862	14'058	15'575	12'542	-0.312	0.682819439	1
ENSBTAG00000008854	*RFX1*	720	734	736	624	848	0.443	0.601421505	1
ENSBTAG00000008857	*GALR3*	2	1	1	2	1	-0.585	1	1
ENSBTAG00000008858	*BT.23349*	14	9	11	12	10	-0.222	0.982048045	1
ENSBTAG00000008860	*FAM65A*	4'110	4'777	4'538	3'559	5'516	0.632	0.414472427	1
ENSBTAG00000008862	*GOLGB1*	3'897	2'649	3'217	3'375	3'059	-0.142	0.856428133	1
ENSBTAG00000008863	*TMEM87A*	3'988	1'984	2'872	3'454	2'291	-0.592	0.450158081	1
ENSBTAG00000008864	*MD2*	871	760	816	754	878	0.218	0.794905998	1
ENSBTAG00000008866	*GANC*	62	28	43	54	32	-0.732	0.701650784	1
ENSBTAG00000008869	*GPC2*	507	163	314	439	188	-1.222	0.20679288	1
ENSBTAG00000008871	*BT.35994*	3	1	2	3	1	-1.170	0.985253077	1
ENSBTAG00000008873	*NCBP2*	2'771	2'368	2'567	2'400	2'734	0.188	0.809904927	1
ENSBTAG00000008876	*EFCAB9*	5	0	2	4	0		0.853268594	1
ENSBTAG00000008877	*APBA1*	203	320	273	176	370	1.072	0.279233137	1
ENSBTAG00000008880	*KDELC1*	3'416	2'057	2'667	2'958	2'375	-0.317	0.686868348	1
ENSBTAG00000008881	*PURA*	174	87	126	151	100	-0.585	0.632093482	1
ENSBTAG00000008883	*BT.39424*	4'789	3'692	4'205	4'147	4'263	0.040	0.958705956	1
ENSBTAG00000008884	*MAPRE3*	1'503	1'309	1'407	1'302	1'512	0.216	0.788824468	1
ENSBTAG00000008886	*STOX2*	145	160	155	126	185	0.557	0.624288871	1
ENSBTAG00000008887	*PAQR3*	416	445	437	360	514	0.512	0.571545479	1
ENSBTAG00000008888	*BT.25237*	1'847	1'308	1'555	1'600	1'510	-0.083	0.919151822	1
ENSBTAG00000008892	*OR52W1*	2	2	2	2	2	0.415	1	1
ENSBTAG00000008893	*BT.39606*	1'473	960	1'192	1'276	1'109	-0.203	0.804805919	1
ENSBTAG00000008895	*BT.63406*	256	253	257	222	292	0.398	0.690887227	1
ENSBTAG00000008896	*DHX15*	8'080	5'177	6'488	6'997	5'978	-0.227	0.768192216	1
ENSBTAG00000008897	*BIN2*	297	126	201	257	145	-0.822	0.439050325	1
ENSBTAG00000008898	*BT.106426*	1	2	2	1	2	1.415	0.981979269	1
ENSBTAG00000008900	*CELA1*	80	40	58	69	46	-0.585	0.725701763	1
ENSBTAG00000008902	*BT.41970*	955	631	778	827	729	-0.183	0.830093072	1
ENSBTAG00000008906	*PCP2*	20	6	12	17	7	-1.322	0.749655668	1
ENSBTAG00000008908	*GZF1*	1'151	854	991	997	986	-0.016	0.987084139	1
ENSBTAG00000008909	*PNPT1*	1'686	657	1'109	1'460	759	-0.945	0.251661967	1
ENSBTAG00000008910	*SSTR3*	2	0	1	2	0		0.974934741	1
ENSBTAG00000008911	*protein_coding*	152	237	203	132	274	1.056	0.318953899	1
ENSBTAG00000008913	*BT.34416*	1'675	1'120	1'372	1'451	1'293	-0.166	0.838702091	1
ENSBTAG00000008915	*SF3B1*	57'540	22'119	37'686	49'831	25'541	-0.964	0.207977177	1
ENSBTAG00000008921	*NEXN*	728	960	869	630	1'109	0.814	0.330823548	1
ENSBTAG00000008922	*APOL6*	3	0	1	3	0		0.936647693	1
ENSBTAG00000008923	*BT.88909*	81	146	119	70	169	1.265	0.306788671	1
ENSBTAG00000008925	*BT.28672*	1'021	576	775	884	665	-0.411	0.627454319	1
ENSBTAG00000008926	*BT.19933*	7'118	5'340	6'165	6'164	6'166	0.000	0.999146796	1
ENSBTAG00000008931	*BT.47829*	2'052	739	1'315	1'777	853	-1.058	0.194809853	1
ENSBTAG00000008932	*SHISA7*	46	189	129	40	218	2.454	0.049791805	1
ENSBTAG00000008933	*MFSD5*	1'093	1'259	1'200	947	1'454	0.619	0.447483738	1
ENSBTAG00000008934	*ESPL1*	846	1'569	1'272	733	1'812	1.306	0.111795612	1
ENSBTAG00000008935	*PFDN5*	15'369	12'832	14'064	13'310	14'817	0.155	0.838553052	1
ENSBTAG00000008936	*ABCC2*	22	10	15	19	12	-0.722	0.844489371	1
ENSBTAG00000008937	*CELF2*	341	262	299	295	303	0.035	0.974355705	1
ENSBTAG00000008938	*BT.61976*	1'014	1'432	1'266	878	1'654	0.913	0.262794021	1
ENSBTAG00000008939	*DNMBP*	3'113	2'060	2'537	2'696	2'379	-0.181	0.81891495	1
ENSBTAG00000008940	*NPTX1*	56	33	43	48	38	-0.348	0.862557075	1
ENSBTAG00000008942	*BT.23388*	484	277	370	419	320	-0.390	0.676812752	1
ENSBTAG00000008943	*ZSCAN12*	607	336	457	526	388	-0.438	0.62755974	1
ENSBTAG00000008944	*CRB1*	183	23	93	158	27	-2.577	0.06867782	1
ENSBTAG00000008945	*SDSL*	3	0	1	3	0		0.936647693	1
ENSBTAG00000008946	*BT.29297*	1	1	1	1	1	0.415	1	1
ENSBTAG00000008947	*TKTL2*	97	61	77	84	70	-0.254	0.86845568	1
ENSBTAG00000008948	*P2RX7*	54	12	30	47	14	-1.755	0.442422088	1
ENSBTAG00000008950	*BT.85406*	75	80	79	65	92	0.508	0.725677399	1
ENSBTAG00000008951	*ALPL*	7'672	3'789	5'510	6'644	4'375	-0.603	0.435514635	1

Ensembl gene ID	geneName	counts wildtype horn bud	counts polled horn bud	baseMean	baseMean wildtype horn bud	baseMean polled horn bud	log2FoldChange	pval	padj
ENSBTAG00000008952	*RAP1GAP*	538	304	408	466	351	-0.408	0.656903905	1
ENSBTAG00000008953	*TAP1*	231	140	181	200	162	-0.307	0.781266494	1
ENSBTAG00000008954	*PSMB9*	108	49	75	94	57	-0.725	0.623103273	1
ENSBTAG00000008956	*CD8B*	14	14	14	12	16	0.415	0.923601843	1
ENSBTAG00000008958	*NSMAF*	1'325	922	1'106	1'147	1'065	-0.108	0.896570091	1
ENSBTAG00000008959	*MGC126945*	27	16	21	23	18	-0.340	0.919037185	1
ENSBTAG00000008962	*MRPL50*	806	599	695	698	692	-0.013	0.990337908	1
ENSBTAG00000008963	*CIT*	1'549	826	1'148	1'341	954	-0.492	0.547856685	1
ENSBTAG00000008964	*DTD1*	1'186	1'470	1'362	1'027	1'697	0.725	0.370602796	1
ENSBTAG00000008967	*RAP1B*	5'913	2'832	4'195	5'121	3'270	-0.647	0.405079001	1
ENSBTAG00000008969	*SLC9A8*	261	308	291	226	356	0.654	0.501617389	1
ENSBTAG00000008973	*BT.48981*	822	1'189	1'042	712	1'373	0.948	0.251713785	1
ENSBTAG00000008977	*TOMM6*	1'086	1'116	1'115	941	1'289	0.454	0.578580498	1
ENSBTAG00000008978	*USP22*	12'214	18'008	15'686	10'578	20'794	0.975	0.204156438	1
ENSBTAG00000008981	*BT.31964*	79	53	65	68	61	-0.161	0.928076088	1
ENSBTAG00000008985	*PCBP1*	9'002	8'104	8'577	7'796	9'358	0.263	0.730318964	1
ENSBTAG00000008987	*BT.58314*	1'772	1'674	1'734	1'535	1'933	0.333	0.6758247	1
ENSBTAG00000008989	*LOH12CR1*	565	425	490	489	491	0.004	0.998163934	1
ENSBTAG00000008990	*DRG1*	527	681	621	456	786	0.785	0.364560852	1
ENSBTAG00000008991	*EIF4ENIF1*	2'482	1'990	2'224	2'149	2'298	0.096	0.902578531	1
ENSBTAG00000008993	*DDX26B*	1'902	595	1'167	1'647	687	-1.262	0.126626395	1
ENSBTAG00000008994	*BT.21011*	421	614	537	365	709	0.959	0.277412612	1
ENSBTAG00000008996	*SFI1*	1'006	1'042	1'037	871	1'203	0.466	0.571151169	1
ENSBTAG00000008997	*ENG*	3'872	3'786	3'862	3'353	4'372	0.383	0.621494099	1
ENSBTAG00000008998	*PISD*	1'503	1'399	1'459	1'302	1'615	0.312	0.698165914	1
ENSBTAG00000009002	*BT.49309*	199	308	264	172	356	1.045	0.294328565	1
ENSBTAG00000009005	*DUSP15*	404	426	421	350	492	0.492	0.589393741	1
ENSBTAG00000009006	*PRR14L*	826	926	892	715	1'069	0.580	0.486395792	1
ENSBTAG00000009019	*SH3PXD2B*	2'202	5'025	3'855	1'907	5'802	1.605	0.042789292	1
ENSBTAG00000009020	*CRIM1*	2'374	2'515	2'480	2'056	2'904	0.498	0.525903354	1
ENSBTAG00000009021	*FEZ2*	2'046	1'475	1'738	1'772	1'703	-0.057	0.944335061	1
ENSBTAG00000009022	*DNAJC30*	219	311	274	190	359	0.921	0.350712221	1
ENSBTAG00000009023	*TMTC3*	713	508	602	617	587	-0.074	0.934562077	1
ENSBTAG00000009024	*KCND1*	609	859	760	527	992	0.911	0.283062763	1
ENSBTAG00000009026	*BT.105034*	2'925	4'951	4'125	2'533	5'717	1.174	0.13371351	1
ENSBTAG00000009029	*BT.54912*	169	41	97	146	47	-1.628	0.226581155	1
ENSBTAG00000009030	*NOX5*	3	1	2	3	1	-1.170	0.985253077	1
ENSBTAG00000009032	*TMEM80*	732	391	543	634	451	-0.490	0.578447255	1
ENSBTAG00000009033	*BT.61831*	2	9	6	2	10	2.585	0.716971424	1
ENSBTAG00000009034	*AP3D1*	8'085	8'131	8'195	7'002	9'389	0.423	0.580563368	1
ENSBTAG00000009035	*CENPE*	749	407	559	649	470	-0.465	0.596415313	1
ENSBTAG00000009036	*SUPT6H*	4'363	4'204	4'316	3'778	4'854	0.361	0.640031666	1
ENSBTAG00000009042	*ATXN7L2*	383	460	431	332	531	0.679	0.454278931	1
ENSBTAG00000009046	*TBX6*	67	77	73	58	89	0.616	0.677887512	1
ENSBTAG00000009047	*BT.85344*	2'226	2'222	2'247	1'928	2'566	0.412	0.600597061	1
ENSBTAG00000009048	*EFHD2*	1'592	1'620	1'625	1'379	1'871	0.440	0.581850267	1
ENSBTAG00000009049	*MGC142702*	2'158	1'675	1'902	1'869	1'934	0.050	0.950235715	1
ENSBTAG00000009050	*ADIPOR2*	700	887	815	606	1'024	0.757	0.368534505	1
ENSBTAG00000009051	*MMP19*	272	48	145	236	55	-2.087	0.080567363	1
ENSBTAG00000009055	*BT.66635*	1'014	531	746	878	613	-0.518	0.541754219	1
ENSBTAG00000009057	*BT.35354*	12	0	5	10	0		0.583618942	1
ENSBTAG00000009058	*TMEM177*	122	174	153	106	201	0.927	0.416284677	1
ENSBTAG00000009061	*FAR1*	3'760	1'749	2'638	3'256	2'020	-0.689	0.381183012	1
ENSBTAG00000009062	*DMRTB1*	1	0	0	1	0		1	1
ENSBTAG00000009064	*CSF2RB*	132	76	101	114	88	-0.381	0.77524373	1
ENSBTAG00000009065	*BT.31917*	870	401	608	753	463	-0.702	0.418960334	1
ENSBTAG00000009067	*BT.101954*	15'267	17'064	16'463	13'222	19'704	0.576	0.450680328	1
ENSBTAG00000009070	*NPFFR2*	12	1	6	10	1	-3.170	0.692545189	1
ENSBTAG00000009071	*ZNF699*	52	27	38	45	31	-0.531	0.798375367	1
ENSBTAG00000009072	*BT.26401*	492	257	361	426	297	-0.522	0.577818855	1
ENSBTAG00000009075	*BT.98029*	891	933	924	772	1'077	0.481	0.561871748	1
ENSBTAG00000009076	*BT.57494*	337	311	325	292	359	0.299	0.754128425	1
ENSBTAG00000009077	*SNRPA*	1'426	1'312	1'375	1'235	1'515	0.295	0.714602783	1
ENSBTAG00000009078	*MIA*	946	273	567	819	315	-1.378	0.119796917	1
ENSBTAG00000009079	*MIA*	1'461	642	1'003	1'265	741	-0.771	0.351824886	1
ENSBTAG00000009080	*ITGB6*	137	289	226	119	334	1.492	0.151754219	1
ENSBTAG00000009084	*ATG3*	2'644	1'399	1'953	2'290	1'615	-0.503	0.526646099	1
ENSBTAG00000009085	*SLC35A5*	5'632	2'461	3'860	4'877	2'842	-0.779	0.317482318	1
ENSBTAG00000009086	*LOXL1*	4'390	6'353	5'569	3'802	7'336	0.948	0.22137608	1
ENSBTAG00000009087	*GNG10*	6'640	5'609	6'114	5'750	6'477	0.172	0.822868037	1
ENSBTAG00000009091	*RNASEL*	114	249	193	99	288	1.542	0.15444695	1
ENSBTAG00000009093	*GAL3ST2*	5	3	4	4	3	-0.322	1	1

Ensembl gene ID	geneName	counts wildtype horn bud	counts polled horn bud	baseMean	baseMean wildtype horn bud	baseMean polled horn bud	log2FoldChange	pval	padj
ENSBTAG00000009097	*FANCI*	646	643	651	559	742	0.408	0.634515167	1
ENSBTAG00000009098	*POLG*	2'089	2'221	2'187	1'809	2'565	0.503	0.523558744	1
ENSBTAG00000009099	*PRR14*	1'370	1'591	1'512	1'186	1'837	0.631	0.432594226	1
ENSBTAG00000009101	*SH2B3*	492	294	383	426	339	-0.328	0.725012483	1
ENSBTAG00000009103	*UBLCP1*	2'166	1'336	1'709	1'876	1'543	-0.282	0.724561584	1
ENSBTAG00000009104	*SERP1*	1'949	1'595	1'765	1'688	1'842	0.126	0.874268622	1
ENSBTAG00000009105	*BT.61542*	11	45	31	10	52	2.447	0.284792696	1
ENSBTAG00000009106	*BT.23259*	6'662	3'962	5'172	5'769	4'575	-0.335	0.665044311	1
ENSBTAG00000009107	*CBX8*	764	750	764	662	866	0.388	0.645601665	1
ENSBTAG00000009109	*CASD1*	2'048	1'020	1'476	1'774	1'178	-0.591	0.463861704	1
ENSBTAG00000009110	*THOC7*	1'392	908	1'127	1'206	1'048	-0.201	0.806785458	1
ENSBTAG00000009111	*LRRN5*	1'187	1'887	1'603	1'028	2'179	1.084	0.179243779	1
ENSBTAG00000009112	*TRAM2*	506	686	615	438	792	0.854	0.324799555	1
ENSBTAG00000009115	*SPSB4*	119	67	90	103	77	-0.414	0.766341382	1
ENSBTAG00000009117	*THNSL1*	78	49	62	68	57	-0.256	0.881006005	1
ENSBTAG00000009121	*STAG2*	5'860	3'882	4'779	5'075	4'483	-0.179	0.817407773	1
ENSBTAG00000009123	*BT.97182*	434	641	558	376	740	0.978	0.266186449	1
ENSBTAG00000009124	*FEZ1*	2'251	1'625	1'913	1'949	1'876	-0.055	0.945989707	1
ENSBTAG00000009126	*YBX2*	77	43	58	67	50	-0.425	0.801129772	1
ENSBTAG00000009127	*TSPYL4*	3'513	2'367	2'888	3'042	2'733	-0.155	0.844123126	1
ENSBTAG00000009129	*BT.24784*	2'585	1'157	1'787	2'239	1'336	-0.745	0.351586555	1
ENSBTAG00000009131	*TTC37*	2'163	2'099	2'148	1'873	2'424	0.372	0.637527711	1
ENSBTAG00000009132	*TMPRSS2*	59	109	88	51	126	1.301	0.344369498	1
ENSBTAG00000009137	*NKG7*	133	126	130	115	145	0.337	0.781073552	1
ENSBTAG00000009138	*UBXN2B*	98	122	113	85	141	0.731	0.560655922	1
ENSBTAG00000009139	*C18ORF1*	369	241	299	320	278	-0.200	0.839909854	1
ENSBTAG00000009140	*CXORF22*	3	0	1	3	0		0.936647693	1
ENSBTAG00000009141	*FAM210A*	511	337	416	443	389	-0.186	0.841528276	1
ENSBTAG00000009142	*BT.43997*	731	532	624	633	614	-0.043	0.962616701	1
ENSBTAG00000009144	*BPIFA2A*	79	2	35	68	2	-4.889	0.050474476	1
ENSBTAG00000009145	*SLC7A4*	113	38	71	98	44	-1.157	0.442509611	1
ENSBTAG00000009148	*BT.42118*	1	1	1	1	1	0.415	1	1
ENSBTAG00000009150	*SPON1*	16'328	7'411	11'349	14'140	8'557	-0.725	0.345126968	1
ENSBTAG00000009151	*PYGO1*	312	235	271	270	271	0.006	0.998678698	1
ENSBTAG00000009152	*ZNF391*	47	19	31	41	22	-0.892	0.691980358	1
ENSBTAG00000009153	*MLXIPL*	9	6	7	8	7	-0.170	1	1
ENSBTAG00000009154	*U2SURP*	5'758	2'376	3'865	4'987	2'744	-0.862	0.269386843	1
ENSBTAG00000009155	*SCN5A*	8	20	15	7	23	1.737	0.615556808	1
ENSBTAG00000009156	*CAMK2N1*	1'305	484	845	1'130	559	-1.016	0.228238044	1
ENSBTAG00000009157	*SLC35B3*	1'206	635	889	1'044	733	-0.510	0.541256221	1
ENSBTAG00000009161	*TIGD4*	56	19	35	48	22	-1.144	0.585245408	1
ENSBTAG00000009163	*TMEM234*	706	568	634	611	656	0.101	0.907384402	1
ENSBTAG00000009165	*LPIN3*	677	747	724	586	863	0.557	0.512033823	1
ENSBTAG00000009166	*GSX1*	2	0	1	2	0		0.974934741	1
ENSBTAG00000009167	*BT.52155*	1'557	1'557	1'573	1'348	1'798	0.415	0.604152037	1
ENSBTAG00000009169	*XPR1*	608	578	597	527	667	0.342	0.693537997	1
ENSBTAG00000009171	*protein_coding*	2	2	2	2	2	0.415	1	1
ENSBTAG00000009172	*BCAT2*	1'192	1'650	1'469	1'032	1'905	0.884	0.273773729	1
ENSBTAG00000009174	*HSD17B14*	203	160	180	176	185	0.072	0.952052721	1
ENSBTAG00000009175	*RPS6KB2*	1'607	1'666	1'658	1'392	1'924	0.467	0.558704044	1
ENSBTAG00000009176	*ANKRD49*	658	395	513	570	456	-0.321	0.71825492	1
ENSBTAG00000009177	*PLEKHA4*	1'813	1'543	1'676	1'570	1'782	0.182	0.819057334	1
ENSBTAG00000009178	*BT.20299*	615	657	646	533	759	0.510	0.552842707	1
ENSBTAG00000009181	*INPP5A*	1'397	1'246	1'324	1'210	1'439	0.250	0.757040708	1
ENSBTAG00000009182	*BT.45373*	1	10	6	1	12	3.737	0.613387923	1
ENSBTAG00000009183	*SHISA5*	2'734	2'069	2'378	2'368	2'389	0.013	0.986772618	1
ENSBTAG00000009186	*PAX9*	3	11	8	3	13	2.290	0.692284022	1
ENSBTAG00000009187	*DNAJC28*	269	187	224	233	216	-0.110	0.920247687	1
ENSBTAG00000009188	*GART*	3'541	2'521	2'989	3'067	2'911	-0.075	0.924405389	1
ENSBTAG00000009190	*SLC2A4*	61	95	81	53	110	1.054	0.456852313	1
ENSBTAG00000009191	*BT.63339*	3'115	1'724	2'344	2'698	1'991	-0.438	0.578435989	1
ENSBTAG00000009192	*SKINT1*	40	57	50	35	66	0.926	0.595180945	1
ENSBTAG00000009194	*BT.28311*	592	545	571	513	629	0.296	0.734908863	1
ENSBTAG00000009195	*pseudogene*	0	2	1	0	2	Inf	0.939077559	1
ENSBTAG00000009198	*BT.69555*	335	213	268	290	246	-0.238	0.81300054	1
ENSBTAG00000009199	*GLIS2*	1'580	1'588	1'601	1'368	1'834	0.422	0.597499531	1
ENSBTAG00000009200	*BT.30804*	396	390	397	343	450	0.393	0.669258598	1
ENSBTAG00000009201	*BT.24770*	861	922	905	746	1'065	0.514	0.536738826	1
ENSBTAG00000009206	*BT.24663*	213	106	153	184	122	-0.592	0.605788568	1
ENSBTAG00000009207	*MLL5*	8'522	5'984	7'145	7'380	6'910	-0.095	0.902226968	1
ENSBTAG00000009208	*BT.88183*	2'087	1'106	1'542	1'807	1'277	-0.501	0.533182655	1
ENSBTAG00000009209	*BT.26788*	1'818	1'772	1'810	1'574	2'046	0.378	0.634308818	1

Ensembl gene ID	geneName	counts wildtype horn bud	counts polled horn bud	baseMean	baseMean wildtype horn bud	baseMean polled horn bud	log2FoldChange	pval	padj
ENSBTAG00000009210	*ZBTB44*	1'104	725	897	956	837	-0.192	0.819448244	1
ENSBTAG00000009211	*UBE2S*	1'546	2'266	1'978	1'339	2'617	0.967	0.225449473	1
ENSBTAG00000009213	*TOM40B*	901	923	923	780	1'066	0.450	0.587852826	1
ENSBTAG00000009214	*ETS2*	883	1'001	960	765	1'156	0.596	0.471739333	1
ENSBTAG00000009215	*NR1I3*	39	12	24	34	14	-1.285	0.623151531	1
ENSBTAG00000009216	*HVCN1*	596	449	517	516	518	0.006	0.996022083	1
ENSBTAG00000009217	*WNT10A*	132	259	207	114	299	1.387	0.190915604	1
ENSBTAG00000009218	*ANLN*	1'293	1'120	1'207	1'120	1'293	0.208	0.798321235	1
ENSBTAG00000009219	*CADM2*	171	15	83	148	17	-3.096	0.040740321	1
ENSBTAG00000009223	*SFN*	4'218	5'121	4'783	3'653	5'913	0.695	0.369502048	1
ENSBTAG00000009226	*SCUBE3*	238	387	326	206	447	1.116	0.243275855	1
ENSBTAG00000009228	*SRP68*	3'944	3'879	3'947	3'416	4'479	0.391	0.613686055	1
ENSBTAG00000009230	*FBLN7*	968	541	732	838	625	-0.424	0.618164436	1
ENSBTAG00000009231	*NSDHL*	1'277	1'156	1'220	1'106	1'335	0.271	0.738318565	1
ENSBTAG00000009232	*PIK3CA*	1'202	564	846	1'041	651	-0.677	0.420310551	1
ENSBTAG00000009233	*RAC1*	7'500	6'268	6'866	6'495	7'238	0.156	0.838264709	1
ENSBTAG00000009235	*KCNMB3*	82	11	42	71	13	-2.483	0.209667604	1
ENSBTAG00000009236	*DAGLB*	1'873	1'124	1'460	1'622	1'298	-0.322	0.690166662	1
ENSBTAG00000009237	*BT.44241*	10'905	7'906	9'287	9'444	9'129	-0.049	0.949810178	1
ENSBTAG00000009238	*BT.105698*	11	43	30	10	50	2.382	0.306653802	1
ENSBTAG00000009239	*SLC16A10*	37	26	31	32	30	-0.094	0.983564376	1
ENSBTAG00000009242	*ZNF638*	12'129	5'773	8'585	10'504	6'666	-0.656	0.393531267	1
ENSBTAG00000009243	*KIAA1919*	15	7	11	13	8	-0.684	0.893749952	1
ENSBTAG00000009245	*PPP2CB*	2'019	1'160	1'544	1'749	1'339	-0.384	0.632523569	1
ENSBTAG00000009246	*DPY30*	2'669	1'613	2'087	2'311	1'863	-0.312	0.694422976	1
ENSBTAG00000009251	*C2H1orf144*	1'004	1'476	1'287	869	1'704	0.971	0.233666923	1
ENSBTAG00000009252	*KLRA1*	10	5	7	9	6	-0.585	0.94357809	1
ENSBTAG00000009254	*CCDC129*	5	3	4	4	3	-0.322	1	1
ENSBTAG00000009255	*DNHD1*	148	81	111	128	94	-0.455	0.723069436	1
ENSBTAG00000009256	*ITGA4*	359	138	235	311	159	-0.964	0.346505603	1
ENSBTAG00000009257	*CERKL*	16	10	13	14	12	-0.263	0.967957861	1
ENSBTAG00000009258	*NEK11*	10	3	6	9	3	-1.322	0.863266279	1
ENSBTAG00000009260	*GPM6B*	2'525	833	1'574	2'187	962	-1.185	0.143520581	1
ENSBTAG00000009263	*MFSD1*	3'362	1'714	2'445	2'912	1'979	-0.557	0.479814396	1
ENSBTAG00000009265	*BT.97978*	102	51	74	88	59	-0.585	0.695678411	1
ENSBTAG00000009266	*CD226*	6	2	4	5	2	-1.170	0.934316631	1
ENSBTAG00000009267	*UHRF1BP1*	327	334	334	283	386	0.446	0.63819829	1
ENSBTAG00000009269	*GRIK3*	162	243	210	140	281	1.000	0.340364437	1
ENSBTAG00000009271	*PNMA1*	596	132	334	516	152	-1.760	0.069487051	1
ENSBTAG00000009272	*C14ORF43*	1'240	999	1'114	1'074	1'154	0.103	0.899819821	1
ENSBTAG00000009273	*protein_coding*	1	0	0	1	0		1	1
ENSBTAG00000009275	*BT.20306*	1'255	756	980	1'087	873	-0.316	0.703110829	1
ENSBTAG00000009277	*RGS17*	14	5	9	12	6	-1.070	0.844263455	1
ENSBTAG00000009278	*MSL3*	3'376	2'492	2'901	2'924	2'878	-0.023	0.977716945	1
ENSBTAG00000009279	*TNKS2*	3'321	2'131	2'668	2'876	2'461	-0.225	0.774800878	1
ENSBTAG00000009281	*SPRYD4*	676	422	536	585	487	-0.265	0.765255328	1
ENSBTAG00000009282	*PORCN*	1'131	813	959	979	939	-0.061	0.942993364	1
ENSBTAG00000009284	*GLS2*	174	69	115	151	80	-0.919	0.463325747	1
ENSBTAG00000009286	*ZC3H4*	1'382	2'195	1'866	1'197	2'535	1.083	0.176647545	1
ENSBTAG00000009287	*EBP*	525	474	501	455	547	0.268	0.763495613	1
ENSBTAG00000009288	*TBC1D25*	537	817	704	465	943	1.020	0.233715483	1
ENSBTAG00000009289	*MS4A8B*	380	383	386	329	442	0.426	0.644504552	1
ENSBTAG00000009290	*FAM161A*	419	154	270	363	178	-1.029	0.300623212	1
ENSBTAG00000009291	*C20ORF26*	44	57	52	38	66	0.788	0.646610793	1
ENSBTAG00000009293	*EVL*	7'223	7'482	7'447	6'255	8'639	0.466	0.543659993	1
ENSBTAG00000009294	*DEGS2*	32	40	37	28	46	0.737	0.718711377	1
ENSBTAG00000009296	*SLC26A5*	14	3	8	12	3	-1.807	0.758305361	1
ENSBTAG00000009297	*BT.35408*	115	50	79	100	58	-0.787	0.586330824	1
ENSBTAG00000009298	*XYLT2*	1'777	2'225	2'054	1'539	2'569	0.739	0.351359256	1
ENSBTAG00000009299	*AFTPH*	1'828	1'363	1'578	1'583	1'574	-0.008	0.993204511	1
ENSBTAG00000009302	*RCAN2*	779	464	605	675	536	-0.332	0.702646154	1
ENSBTAG00000009304	*PDCD5*	1'127	521	789	976	602	-0.698	0.408854223	1
ENSBTAG00000009305	*BT.81275*	4'087	2'329	3'114	3'539	2'689	-0.396	0.612225444	1
ENSBTAG00000009306	*MORN1*	99	98	99	86	113	0.400	0.763257777	1
ENSBTAG00000009307	*RER1*	4'362	4'541	4'511	3'778	5'243	0.473	0.540625772	1
ENSBTAG00000009308	*TDRD3*	1'744	1'434	1'583	1'510	1'656	0.133	0.86828043	1
ENSBTAG00000009309	*PEX10*	3'091	2'437	2'745	2'677	2'814	0.072	0.926369467	1
ENSBTAG00000009310	*UTP3*	1'065	491	745	922	567	-0.702	0.408886417	1
ENSBTAG00000009314	*C1ORF168*	15	17	16	13	20	0.596	0.865938341	1
ENSBTAG00000009315	*CDON*	3'767	3'169	3'461	3'262	3'659	0.166	0.830852571	1
ENSBTAG00000009327	*BT.42463*	13	7	10	11	8	-0.478	0.940171262	1
ENSBTAG00000009330	*SIN3B*	2'527	4'386	3'626	2'188	5'065	1.211	0.123502665	1

Ensembl gene ID	geneName	counts wildtype horn bud	counts polled horn bud	baseMean	baseMean wildtype horn bud	baseMean polled horn bud	log2FoldChange	pval	padj
ENSBTAG00000009331	CPAMD8	780	960	892	675	1'109	0.715	0.391701779	1
ENSBTAG00000009332	TTLL6	3	1	2	3	1	-1.170	0.985253077	1
ENSBTAG00000009334	NDUFA5	752	566	652	651	654	0.005	0.99663318	1
ENSBTAG00000009336	BT.77236	1'486	681	1'037	1'287	786	-0.711	0.389483003	1
ENSBTAG00000009337	PNMAL1	482	322	395	417	372	-0.167	0.858927297	1
ENSBTAG00000009340	KATNAL1	338	204	264	293	236	-0.313	0.75497453	1
ENSBTAG00000009341	CCDC146	75	7	37	65	8	-3.006	0.166246817	1
ENSBTAG00000009343	AAK1	142	82	109	123	95	-0.377	0.771639598	1
ENSBTAG00000009345	BT.49063	3'546	2'463	2'957	3'071	2'844	-0.111	0.888244214	1
ENSBTAG00000009346	MAPKAPK5	915	566	723	792	654	-0.278	0.744997305	1
ENSBTAG00000009348	protein_coding	133	55	89	115	64	-0.859	0.531865481	1
ENSBTAG00000009349	MMEL1	7	10	9	6	12	0.930	0.865678914	1
ENSBTAG00000009350	PLA2G12B	4	2	3	3	2	-0.585	0.996239345	1
ENSBTAG00000009351	BT.22019	8'143	6'719	7'405	7'052	7'758	0.138	0.856927856	1
ENSBTAG00000009353	EVI2B	177	56	109	153	65	-1.245	0.331199307	1
ENSBTAG00000009354	EVI2A	306	47	160	265	54	-2.288	0.050814779	1
ENSBTAG00000009357	IL34	469	217	328	406	251	-0.697	0.464978491	1
ENSBTAG00000009358	MTSS1L	3'687	4'731	4'328	3'193	5'463	0.775	0.318542707	1
ENSBTAG00000009359	processed_pseudogene	8'721	8'267	8'549	7'553	9'546	0.338	0.658650687	1
ENSBTAG00000009362	SYNE1	4'307	2'701	3'424	3'730	3'119	-0.258	0.740782297	1
ENSBTAG00000009363	GINS3	597	474	532	517	547	0.082	0.926945522	1
ENSBTAG00000009364	BT.102250	0	1	1	0	1	Inf	0.993540919	1
ENSBTAG00000009366	ARF5	1'409	1'522	1'489	1'220	1'757	0.526	0.512501826	1
ENSBTAG00000009368	RRAGC	1'329	948	1'123	1'151	1'095	-0.072	0.931254462	1
ENSBTAG00000009371	CCDC36	40	23	31	35	27	-0.383	0.876135313	1
ENSBTAG00000009372	TTC5	1'583	1'036	1'284	1'371	1'196	-0.197	0.809581736	1
ENSBTAG00000009374	PARP2	1'888	1'448	1'654	1'635	1'672	0.032	0.967925128	1
ENSBTAG00000009376	BT.48886	789	1'342	1'116	683	1'550	1.181	0.152789825	1
ENSBTAG00000009378	BT.86811	675	262	444	585	303	-0.950	0.295689333	1
ENSBTAG00000009381	BT.74007	333	91	197	288	105	-1.457	0.17698171	1
ENSBTAG00000009382	KLK13	2	0	1	2	0		0.974934741	1
ENSBTAG00000009383	BT.37915	1'754	1'209	1'458	1'519	1'396	-0.122	0.880996625	1
ENSBTAG00000009384	AGTRAP	812	639	721	703	738	0.069	0.935822488	1
ENSBTAG00000009386	C1ORF167	368	280	321	319	323	0.021	0.98561824	1
ENSBTAG00000009389	BT.49851	59'851	24'991	40'345	51'832	28'857	-0.845	0.268944017	1
ENSBTAG00000009390	BT.64822	735	833	799	637	962	0.596	0.479105148	1
ENSBTAG00000009391	ADPRH	234	206	220	203	238	0.231	0.825379768	1
ENSBTAG00000009392	PLA1A	108	55	79	94	64	-0.558	0.701583704	1
ENSBTAG00000009393	GAL	49	2	22	42	2	-4.200	0.171720345	1
ENSBTAG00000009394	BT.49772	522	437	478	452	505	0.159	0.860144302	1
ENSBTAG00000009396	EXO1	143	127	135	124	147	0.244	0.840400935	1
ENSBTAG00000009400	FASTKD3	200	196	200	173	226	0.386	0.716952766	1
ENSBTAG00000009401	BT.48691	577	437	502	500	505	0.014	0.989177755	1
ENSBTAG00000009405	TRPC4	145	37	84	126	43	-1.555	0.272110602	1
ENSBTAG00000009406	protein_coding	1	0	0	1	0		1	1
ENSBTAG00000009407	MBTD1	954	809	880	826	934	0.177	0.831899688	1
ENSBTAG00000009410	TBRG1	1'856	921	1'335	1'607	1'063	-0.596	0.462500871	1
ENSBTAG00000009412	SIAE	635	320	460	550	370	-0.574	0.524587424	1
ENSBTAG00000009414	BT.48909	2'865	2'419	2'637	2'481	2'793	0.171	0.826985884	1
ENSBTAG00000009415	NUFIP1	587	283	418	508	327	-0.638	0.485921694	1
ENSBTAG00000009416	BT.49365	484	305	386	419	352	-0.251	0.788091099	1
ENSBTAG00000009417	ZFAND5	5'982	3'338	4'517	5'181	3'854	-0.427	0.581985658	1
ENSBTAG00000009418	FAM131B	159	73	111	138	84	-0.708	0.577526616	1
ENSBTAG00000009419	BT.56841	4'898	10'745	8'325	4'242	12'407	1.548	0.047624041	1
ENSBTAG00000009420	SCML2	493	402	446	427	464	0.121	0.895109942	1
ENSBTAG00000009421	BT.49600	78	7	38	68	8	-3.063	0.152277607	1
ENSBTAG00000009423	NIPAL1	67	54	60	58	62	0.104	0.958339314	1
ENSBTAG00000009426	EZH2	3'027	2'302	2'640	2'621	2'658	0.020	0.979448718	1
ENSBTAG00000009427	PPM1D	876	454	641	759	524	-0.533	0.536777521	1
ENSBTAG00000009428	GAN	48	48	48	42	55	0.415	0.820595375	1
ENSBTAG00000009430	MYRIP	106	18	56	92	21	-2.143	0.207848965	1
ENSBTAG00000009431	SLC25A44	1'828	1'031	1'387	1'583	1'190	-0.411	0.611227031	1
ENSBTAG00000009432	PMF1	770	908	858	667	1'048	0.653	0.435155648	1
ENSBTAG00000009434	PAQR6	827	744	788	716	859	0.262	0.755386831	1
ENSBTAG00000009435	SMG5	3'866	5'550	4'878	3'348	6'409	0.937	0.227997002	1
ENSBTAG00000009436	TMEM79	1'100	993	1'050	953	1'147	0.267	0.744765123	1
ENSBTAG00000009439	C10ORF137	1'605	978	1'260	1'390	1'129	-0.300	0.712998196	1
ENSBTAG00000009441	RBBP6	5'953	2'676	4'123	5'155	3'090	-0.739	0.342595006	1
ENSBTAG00000009443	GCNT3	197	56	118	171	65	-1.400	0.263700617	1
ENSBTAG00000009446	USP10	2'528	2'382	2'470	2'189	2'750	0.329	0.674772562	1
ENSBTAG00000009447	PRMT8	59	15	34	51	17	-1.561	0.464398265	1
ENSBTAG00000009449	BT.65001	327	450	401	283	520	0.876	0.34117791	1

Ensembl gene ID	geneName	counts wildtype horn bud	counts polled horn bud	baseMean	baseMean wildtype horn bud	baseMean polled horn bud	log2FoldChange	pval	padj
ENSBTAG00000009451	*BT.59251*	1'783	1'544	1'663	1'544	1'783	0.207	0.79484274	1
ENSBTAG00000009453	*URGCP*	1'097	1'382	1'273	950	1'596	0.748	0.35743585	1
ENSBTAG00000009455	*IL12RB2*	10	2	5	9	2	-1.907	0.815196829	1
ENSBTAG00000009457	*TEX13A*	0	1	1	0	1	Inf	0.993540919	1
ENSBTAG00000009458	*METTL2*	1'845	1'276	1'536	1'598	1'473	-0.117	0.885391637	1
ENSBTAG00000009459	*CCDC165*	884	797	843	766	920	0.266	0.750997515	1
ENSBTAG00000009460	*ZNF550*	124	83	102	107	96	-0.164	0.907540798	1
ENSBTAG00000009461	*STYXL1*	20	20	20	17	23	0.415	0.896464675	1
ENSBTAG00000009462	*MDH2*	5'648	5'854	5'825	4'891	6'760	0.467	0.544261382	1
ENSBTAG00000009468	*FAM159B*	19	4	11	16	5	-1.833	0.688634464	1
ENSBTAG00000009469	*SREK1IP1*	160	67	108	139	77	-0.841	0.511915967	1
ENSBTAG00000009470	*CLIC4*	5'206	4'874	5'068	4'509	5'628	0.320	0.677859787	1
ENSBTAG00000009471	*CEP135*	385	149	253	333	172	-0.955	0.343520139	1
ENSBTAG00000009472	*C4H7orf25*	488	327	400	423	378	-0.163	0.862352894	1
ENSBTAG00000009475	*PLXDC2*	1'935	1'784	1'868	1'676	2'060	0.298	0.707348543	1
ENSBTAG00000009477	*PIR*	319	181	243	276	209	-0.403	0.69304474	1
ENSBTAG00000009478	*GDF9*	24	3	12	21	3	-2.585	0.53693803	1
ENSBTAG00000009479	*UQCRQ*	2'184	1'590	1'864	1'891	1'836	-0.043	0.958313458	1
ENSBTAG00000009481	*C1ORF87*	2	0	1	2	0		0.974934741	1
ENSBTAG00000009482	*AFF4*	613	471	537	531	544	0.035	0.969946117	1
ENSBTAG00000009483	*POLR2G*	3'308	3'472	3'437	2'865	4'009	0.485	0.533117427	1
ENSBTAG00000009484	*TAF6L*	575	716	662	498	827	0.731	0.394453413	1
ENSBTAG00000009486	*TMEM179B*	559	491	526	484	567	0.228	0.796641494	1
ENSBTAG00000009487	*TMEM223*	674	556	613	584	642	0.137	0.87462272	1
ENSBTAG00000009488	*NXF1*	3'424	2'917	3'167	2'965	3'368	0.184	0.813094167	1
ENSBTAG00000009489	*CACNA2D2*	61	15	35	53	17	-1.609	0.445110861	1
ENSBTAG00000009490	*STX5*	2'026	2'407	2'267	1'755	2'779	0.664	0.400621866	1
ENSBTAG00000009491	*WDR74*	988	913	955	856	1'054	0.301	0.715935079	1
ENSBTAG00000009493	*BCL3*	681	471	567	590	544	-0.117	0.896021727	1
ENSBTAG00000009494	*CBLC*	347	504	441	301	582	0.954	0.293447516	1
ENSBTAG00000009495	*BCAM*	9'238	20'580	15'882	8'000	23'764	1.571	0.043587898	1
ENSBTAG00000009496	*STAT5A*	1'702	1'399	1'545	1'474	1'615	0.132	0.868937427	1
ENSBTAG00000009498	*PTEN*	1'774	1'030	1'363	1'536	1'189	-0.369	0.648387142	1
ENSBTAG00000009500	*KDM1A*	4'678	3'979	4'323	4'051	4'595	0.182	0.813962654	1
ENSBTAG00000009501	*KLKB1*	1	1	1	1	1	0.415	1	1
ENSBTAG00000009502	*LUZP1*	591	1'127	907	512	1'301	1.346	0.110089486	1
ENSBTAG00000009504	*REEP4*	1'164	1'741	1'509	1'008	2'010	0.996	0.217864872	1
ENSBTAG00000009506	*NAGA*	3'953	4'549	4'338	3'423	5'253	0.618	0.425486099	1
ENSBTAG00000009507	*FAM109B*	6'186	7'203	6'837	5'357	8'317	0.635	0.409512629	1
ENSBTAG00000009508	*C5H22orf32*	1'778	1'392	1'574	1'540	1'607	0.062	0.938430357	1
ENSBTAG00000009509	*NDUFA6*	1'572	988	1'251	1'361	1'141	-0.255	0.754584354	1
ENSBTAG00000009510	*ADRA2B*	51	70	62	44	81	0.872	0.580894334	1
ENSBTAG00000009511	*processed_pseudogene*	184	228	211	159	263	0.724	0.488348665	1
ENSBTAG00000009512	*EPS8L1*	189	35	102	164	40	-2.018	0.131302236	1
ENSBTAG00000009513	*TGFBI*	18'124	10'354	13'826	15'696	11'956	-0.393	0.60754112	1
ENSBTAG00000009514	*PPWD1*	3'210	1'530	2'273	2'780	1'767	-0.654	0.408229504	1
ENSBTAG00000009515	*CA6*	8	0	3	7	0		0.72953145	1
ENSBTAG00000009516	*RNF20*	3'853	3'493	3'685	3'337	4'033	0.274	0.724164986	1
ENSBTAG00000009517	*DBI*	6'219	3'707	4'833	5'386	4'280	-0.331	0.668529187	1
ENSBTAG00000009518	*ALKBH3*	764	595	674	662	687	0.054	0.950421392	1
ENSBTAG00000009519	*BT.55196*	365	279	319	316	322	0.027	0.980050363	1
ENSBTAG00000009520	*ADCY1*	127	47	82	110	54	-1.019	0.472647532	1
ENSBTAG00000009521	*ZNF565*	566	286	410	490	330	-0.570	0.534635046	1
ENSBTAG00000009522	*EIF4E*	4'571	3'050	3'740	3'959	3'522	-0.169	0.828818885	1
ENSBTAG00000009523	*SLC9A6*	2'258	1'504	1'846	1'955	1'737	-0.171	0.830528635	1
ENSBTAG00000009526	*TMEM59*	6'092	2'495	4'078	5'276	2'881	-0.873	0.262920663	1
ENSBTAG00000009527	*BT.35742*	608	381	483	527	440	-0.259	0.773368608	1
ENSBTAG00000009530	*pseudogene*	351	115	218	304	133	-1.195	0.25322049	1
ENSBTAG00000009532	*WNT7B*	87	159	129	75	184	1.285	0.286806254	1
ENSBTAG00000009533	*RPL3L*	16	58	40	14	67	2.273	0.25035166	1
ENSBTAG00000009534	*NDUFB10*	3'693	4'983	4'476	3'198	5'754	0.847	0.275566894	1
ENSBTAG00000009535	*RPS2*	67'435	83'574	77'452	58'400	96'503	0.725	0.341235057	1
ENSBTAG00000009536	*RNF151*	1	5	3	1	6	2.737	0.836148221	1
ENSBTAG00000009537	*CLEC9A*	22	3	11	19	3	-2.459	0.576368753	1
ENSBTAG00000009539	*pseudogene*	11	66	43	10	76	3.000	0.129864539	1
ENSBTAG00000009541	*SUCLG2*	3'833	3'798	3'853	3'319	4'386	0.402	0.604216265	1
ENSBTAG00000009542	*ZC3H10*	1'037	902	970	898	1'042	0.214	0.795985584	1
ENSBTAG00000009543	*FAM62A*	4'961	5'742	5'463	4'296	6'630	0.626	0.4173948	1
ENSBTAG00000009545	*BT.48801*	198	62	122	171	72	-1.260	0.307833417	1
ENSBTAG00000009547	*ZDHHC4*	3'110	2'797	2'962	2'693	3'230	0.262	0.736810824	1
ENSBTAG00000009548	*MAD2L1BP*	497	296	386	430	342	-0.333	0.72069812	1
ENSBTAG00000009549	*RSPH9*	211	114	157	183	132	-0.473	0.67864366	1

Ensembl gene ID	geneName	counts wildtype horn bud	counts polled horn bud	baseMean	baseMean wildtype horn bud	baseMean polled horn bud	log2FoldChange	pval	padj
ENSBTAG00000009552	*ATP2B1*	4'836	4'426	4'649	4'188	5'111	0.287	0.709591206	1
ENSBTAG00000009554	*EHBP1L1*	6'071	6'770	6'537	5'258	7'817	0.572	0.456974637	1
ENSBTAG00000009560	*BT.62744*	812	606	701	703	700	-0.007	0.996013043	1
ENSBTAG00000009562	*ZNF593*	313	302	310	271	349	0.363	0.705837303	1
ENSBTAG00000009565	*BT.102247*	4'154	1'835	2'858	3'597	2'119	-0.764	0.331018072	1
ENSBTAG00000009566	*CCNH*	922	432	649	798	499	-0.679	0.431331763	1
ENSBTAG00000009568	*BT.39403*	2'748	2'595	2'688	2'380	2'996	0.332	0.670941032	1
ENSBTAG00000009569	*DOCK6*	4'459	8'306	6'726	3'862	9'591	1.312	0.092077143	1
ENSBTAG00000009570	*C19ORF80*	4	28	18	3	32	3.222	0.319626435	1
ENSBTAG00000009573	*BT.34346*	694	714	713	601	824	0.456	0.591842006	1
ENSBTAG00000009574	*ARFGAP2*	3'166	4'603	4'028	2'742	5'315	0.955	0.220927093	1
ENSBTAG00000009575	*C6ORF170*	301	142	212	261	164	-0.669	0.523444799	1
ENSBTAG00000009576	*PACSIN3*	1'175	1'587	1'425	1'018	1'833	0.849	0.294016321	1
ENSBTAG00000009578	*BT.63222*	6'539	4'763	5'581	5'663	5'500	-0.042	0.957245453	1
ENSBTAG00000009579	*CEP85*	932	697	806	807	805	-0.004	0.998534393	1
ENSBTAG00000009580	*SH3BGRL3*	7'402	7'916	7'775	6'410	9'141	0.512	0.504590504	1
ENSBTAG00000009581	*UBXN11*	200	164	181	173	189	0.129	0.909583763	1
ENSBTAG00000009583	*SLC22A2*	1	0	0	1	0		1	1
ENSBTAG00000009584	*SAPS3*	6'205	4'551	5'314	5'374	5'255	-0.032	0.967596565	1
ENSBTAG00000009585	*CDH16*	2	0	1	2	0		0.974934741	1
ENSBTAG00000009586	*CDC25A*	388	449	427	336	518	0.626	0.491182438	1
ENSBTAG00000009587	*NRG3*	17	0	7	15	0		0.437564044	1
ENSBTAG00000009595	*NGDN*	1'599	831	1'172	1'385	960	-0.529	0.517486058	1
ENSBTAG00000009596	*BT.49847*	30	7	17	26	8	-1.684	0.602356086	1
ENSBTAG00000009598	*CHM*	297	161	222	257	186	-0.468	0.652508701	1
ENSBTAG00000009599	*LCN1*	7	0	3	6	0		0.769774961	1
ENSBTAG00000009600	*STAMBP*	1'076	844	953	932	975	0.065	0.938286164	1
ENSBTAG00000009602	*TMEM120B*	996	974	994	863	1'125	0.383	0.642578718	1
ENSBTAG00000009603	*UQCRH*	13'883	7'418	10'294	12'023	8'566	-0.489	0.523383263	1
ENSBTAG00000009607	*GYLTL1B*	2'982	3'479	3'300	2'582	4'017	0.637	0.413855822	1
ENSBTAG00000009611	*PHF21A*	1'748	1'320	1'519	1'514	1'524	0.010	0.990435037	1
ENSBTAG00000009614	*BT.49396*	190	136	161	165	157	-0.067	0.958952119	1
ENSBTAG00000009615	*ANXA2*	42'680	48'295	46'364	36'962	55'766	0.593	0.435308633	1
ENSBTAG00000009617	*SLC2A1*	3'797	3'496	3'663	3'288	4'037	0.296	0.702746623	1
ENSBTAG00000009618	*NEK2*	201	294	257	174	339	0.964	0.335916164	1
ENSBTAG00000009620	*WDR27*	432	191	297	374	221	-0.762	0.433010328	1
ENSBTAG00000009621	*pseudogene*	2'802	4'122	3'593	2'427	4'760	0.972	0.21415014	1
ENSBTAG00000009622	*GPRC6A*	1	0	0	1	0		1	1
ENSBTAG00000009624	*SGSM3*	989	1'059	1'040	856	1'223	0.514	0.532257726	1
ENSBTAG00000009625	*DMBX1*	0	2	1	0	2	Inf	0.939077559	1
ENSBTAG00000009631	*ZNF683*	9	1	4	8	1	-2.755	0.785977687	1
ENSBTAG00000009632	*LARP7*	2'159	1'009	1'517	1'870	1'165	-0.682	0.396956435	1
ENSBTAG00000009634	*BT.93629*	16	19	18	14	22	0.663	0.837933827	1
ENSBTAG00000009635	*HEATR6*	2'205	1'742	1'961	1'910	2'011	0.075	0.92450121	1
ENSBTAG00000009636	*SLC7A6OS*	2'547	1'328	1'870	2'206	1'533	-0.525	0.510211426	1
ENSBTAG00000009637	*SLC12A2*	1'221	634	895	1'057	732	-0.530	0.525206449	1
ENSBTAG00000009638	*BT.21927*	1'529	1'537	1'549	1'324	1'775	0.423	0.597921421	1
ENSBTAG00000009641	*MTHFD1*	2'317	2'566	2'485	2'007	2'963	0.562	0.474396549	1
ENSBTAG00000009642	*AKAP5*	382	135	243	331	156	-1.086	0.286224845	1
ENSBTAG00000009643	*ZBTB25*	78	46	60	68	53	-0.347	0.836798561	1
ENSBTAG00000009646	*BT.22086*	7'944	8'938	8'600	6'880	10'321	0.585	0.445514639	1
ENSBTAG00000009647	*PHAX*	3'095	1'741	2'345	2'680	2'010	-0.415	0.598911925	1
ENSBTAG00000009649	*CCDC164*	190	49	111	165	57	-1.540	0.229863426	1
ENSBTAG00000009650	*BT.95227*	15	3	8	13	3	-1.907	0.733459861	1
ENSBTAG00000009653	*TCL1B*	1	0	0	1	0		1	1
ENSBTAG00000009654	*LPAR1*	2'675	2'027	2'329	2'317	2'341	0.015	0.984878881	1
ENSBTAG00000009655	*TNS3*	2'797	4'809	3'988	2'422	5'553	1.197	0.126857049	1
ENSBTAG00000009656	*BT.105763*	2	0	1	2	0		0.974934741	1
ENSBTAG00000009657	*MTMR1*	519	519	524	449	599	0.415	0.63802464	1
ENSBTAG00000009658	*PLEK*	86	43	62	74	50	-0.585	0.716746717	1
ENSBTAG00000009661	*RNASEH2A*	1'845	2'018	1'964	1'598	2'330	0.544	0.492449354	1
ENSBTAG00000009663	*CSDA*	5'657	4'759	5'197	4'899	5'495	0.166	0.829339743	1
ENSBTAG00000009664	*BT.59240*	431	258	336	373	298	-0.325	0.733317293	1
ENSBTAG00000009665	*UTRN*	3'250	3'935	3'679	2'815	4'544	0.691	0.374798126	1
ENSBTAG00000009667	*SLC25A46*	585	311	433	507	359	-0.496	0.585400164	1
ENSBTAG00000009668	*TMEM184B*	4'892	4'332	4'619	4'237	5'002	0.240	0.755933138	1
ENSBTAG00000009673	*NHEDC1*	49	14	29	42	16	-1.392	0.54797818	1
ENSBTAG00000009674	*BT.71188*	7	0	3	6	0		0.769774961	1
ENSBTAG00000009675	*protein_coding*	1	0	0	1	0		1	1
ENSBTAG00000009676	*BT.23261*	3'493	3'919	3'775	3'025	4'525	0.581	0.454561885	1
ENSBTAG00000009677	*PARP10*	597	1'099	893	517	1'269	1.295	0.12422823	1
ENSBTAG00000009679	*PLEKHG1*	721	419	554	624	484	-0.368	0.675809843	1

Ensembl gene ID	geneName	counts wildtype horn bud	counts polled horn bud	baseMean	baseMean wildtype horn bud	baseMean polled horn bud	log2FoldChange	pval	padj
ENSBTAG00000009680	*FAM177A1*	2'161	1'422	1'757	1'871	1'642	-0.189	0.813784204	1
ENSBTAG00000009681	*PPP2R3C*	988	479	704	856	553	-0.629	0.461376954	1
ENSBTAG00000009682	*KIAA0391*	548	395	465	475	456	-0.057	0.952320833	1
ENSBTAG00000009683	*PSMA6*	3'150	2'331	2'710	2'728	2'692	-0.019	0.981482921	1
ENSBTAG00000009685	*THEX1*	450	364	405	390	420	0.109	0.906960225	1
ENSBTAG00000009686	*FBXO15*	138	218	186	120	252	1.075	0.321372162	1
ENSBTAG00000009687	*C24H18ORF55*	1'615	947	1'246	1'399	1'094	-0.355	0.662939843	1
ENSBTAG00000009689	*RWDD1*	4'007	2'418	3'131	3'470	2'792	-0.314	0.688288761	1
ENSBTAG00000009691	*SH2B2*	406	478	452	352	552	0.651	0.470446088	1
ENSBTAG00000009693	*TBC1D12*	1'594	619	1'048	1'380	715	-0.950	0.25117345	1
ENSBTAG00000009694	*PUS10*	552	195	352	478	225	-1.086	0.250739159	1
ENSBTAG00000009698	*ABCD3*	3'681	2'005	2'752	3'188	2'315	-0.461	0.55651401	1
ENSBTAG00000009701	*IL25*	8	6	7	7	7	0.000	1	1
ENSBTAG00000009703	*BT.102832*	359	450	415	311	520	0.741	0.417375189	1
ENSBTAG00000009704	*CECR2*	75	80	79	65	92	0.508	0.725677399	1
ENSBTAG00000009705	*SERPINF1*	68'538	27'517	45'565	59'356	31'774	-0.902	0.238434491	1
ENSBTAG00000009708	*SMYD4*	465	292	370	403	337	-0.256	0.785421239	1
ENSBTAG00000009709	*TAF5*	679	329	484	588	380	-0.630	0.48123593	1
ENSBTAG00000009711	*RPA1*	4'211	4'187	4'241	3'647	4'835	0.407	0.598988657	1
ENSBTAG00000009713	*USMG5*	244	99	163	211	114	-0.886	0.430853695	1
ENSBTAG00000009714	*ARHGAP33*	1'053	838	940	912	968	0.086	0.918281426	1
ENSBTAG00000009715	*BT.23141*	2'679	2'871	2'818	2'320	3'315	0.515	0.510515861	1
ENSBTAG00000009717	*FGL2*	1'426	609	969	1'235	703	-0.812	0.328269626	1
ENSBTAG00000009719	*CALHM2*	1'334	1'393	1'382	1'155	1'608	0.477	0.553939373	1
ENSBTAG00000009726	*KLHL12*	947	986	979	820	1'139	0.473	0.566728062	1
ENSBTAG00000009727	*ADIPOR1*	1'982	1'663	1'818	1'716	1'920	0.162	0.8384735	1
ENSBTAG00000009732	*NCBP1*	3'715	2'414	3'002	3'217	2'787	-0.207	0.791844156	1
ENSBTAG00000009733	*FBP1*	21	22	22	18	25	0.482	0.86964816	1
ENSBTAG00000009734	*XPA*	647	320	465	560	370	-0.601	0.504542327	1
ENSBTAG00000009735	*A1BG*	0	2	1	0	2	Inf	0.939077559	1
ENSBTAG00000009736	*CDC42BPG*	3'905	4'694	4'401	3'382	5'420	0.681	0.380144112	1
ENSBTAG00000009737	*LTC4S*	131	43	82	113	50	-1.192	0.402685033	1
ENSBTAG00000009738	*MGAT4B*	3'713	3'954	3'891	3'216	4'566	0.506	0.514449054	1
ENSBTAG00000009739	*CSMD1*	135	26	73	117	30	-1.961	0.194232071	1
ENSBTAG00000009742	*DOK7*	110	100	105	95	115	0.278	0.833316553	1
ENSBTAG00000009743	*PDRG1*	1'433	717	1'034	1'241	828	-0.584	0.479190967	1
ENSBTAG00000009744	*FAM193A*	1'501	2'000	1'805	1'300	2'309	0.829	0.299327108	1
ENSBTAG00000009746	*MPHOSPH6*	919	703	804	796	812	0.028	0.973874245	1
ENSBTAG00000009747	*RNFT1*	4'608	3'132	3'804	3'991	3'617	-0.142	0.85563484	1
ENSBTAG00000009748	*BT.74298*	3	1	2	3	1	-1.170	0.985253077	1
ENSBTAG00000009749	*USP2*	103	113	110	89	130	0.549	0.666277686	1
ENSBTAG00000009750	*RAX2*	3	4	4	3	5	0.830	0.962490925	1
ENSBTAG00000009752	*CD70*	1	0	0	1	0		1	1
ENSBTAG00000009755	*GOLGA3*	1'701	1'594	1'657	1'473	1'841	0.321	0.687210819	1
ENSBTAG00000009757	*RPSA*	124'587	103'582	113'751	107'896	119'606	0.149	0.843885442	1
ENSBTAG00000009760	*BT.88469*	226	167	194	196	193	-0.021	0.990369671	1
ENSBTAG00000009761	*BT.86861*	9'781	6'729	8'120	8'471	7'770	-0.125	0.871621416	1
ENSBTAG00000009762	*BEAN1*	77	64	70	67	74	0.148	0.92953737	1
ENSBTAG00000009764	*protein_coding*	0	1	1	0	1	Inf	0.993540919	1
ENSBTAG00000009765	*MTMR14*	978	1'273	1'158	847	1'470	0.795	0.33124903	1
ENSBTAG00000009768	*IFIT3*	773	717	749	669	828	0.307	0.717227297	1
ENSBTAG00000009769	*processed_pseudogene*	24	59	44	21	68	1.713	0.356613297	1
ENSBTAG00000009770	*GPD2*	396	235	307	343	271	-0.338	0.727920951	1
ENSBTAG00000009772	*LIX1*	343	250	293	297	289	-0.041	0.97078499	1
ENSBTAG00000009773	*KREMEN1*	1'503	2'787	2'260	1'302	3'218	1.306	0.102072036	1
ENSBTAG00000009774	*MOBP*	6	4	5	5	5	-0.170	1	1
ENSBTAG00000009775	*PRRG3*	187	205	199	162	237	0.548	0.605890363	1
ENSBTAG00000009777	*NUP35*	1'305	852	1'057	1'130	984	-0.200	0.808925985	1
ENSBTAG00000009778	*KRAS*	600	524	562	520	605	0.220	0.802145893	1
ENSBTAG00000009780	*GTF2I*	12'912	8'171	10'309	11'182	9'435	-0.245	0.749190252	1
ENSBTAG00000009782	*CBX4*	748	651	700	648	752	0.215	0.801467124	1
ENSBTAG00000009783	*protein_coding*	2'611	5'703	4'423	2'261	6'585	1.542	0.050592315	1
ENSBTAG00000009784	*RAB32*	1'124	1'497	1'351	973	1'729	0.828	0.307012733	1
ENSBTAG00000009785	*SAMD4B*	2'411	3'106	2'837	2'088	3'586	0.780	0.320061444	1
ENSBTAG00000009786	*SCIN*	282	146	206	244	169	-0.535	0.613040778	1
ENSBTAG00000009788	*ACOT6*	24	10	16	21	12	-0.848	0.806552039	1
ENSBTAG00000009789	*BT.87672*	497	474	489	430	547	0.347	0.697293605	1
ENSBTAG00000009795	*SF3B5*	2'607	2'479	2'560	2'258	2'863	0.342	0.662170959	1
ENSBTAG00000009796	*PIGV*	656	410	521	568	473	-0.263	0.767670781	1
ENSBTAG00000009797	*RIMBP2*	166	63	108	144	73	-0.983	0.442958316	1
ENSBTAG00000009798	*DCDC2*	2	0	1	2	0		0.974934741	1
ENSBTAG00000009800	*BT.61112*	317	329	327	275	380	0.469	0.622286128	1

Ensembl gene ID	geneName	counts wildtype horn bud	counts polled horn bud	baseMean	baseMean wildtype horn bud	baseMean polled horn bud	log2FoldChange	pval	padj
ENSBTAG00000009803	*BT.21126*	146	21	75	126	24	-2.382	0.116735601	1
ENSBTAG00000009804	*ERP44*	4'226	2'687	3'381	3'660	3'103	-0.238	0.760260864	1
ENSBTAG00000009805	*BT.26633*	3'198	4'673	4'083	2'770	5'396	0.962	0.217362476	1
ENSBTAG00000009806	*INVS*	1'985	1'557	1'758	1'719	1'798	0.065	0.935278278	1
ENSBTAG00000009811	*BT.3020*	1'249	1'342	1'316	1'082	1'550	0.519	0.521655192	1
ENSBTAG00000009812	*CXCL6*	83	41	60	72	47	-0.602	0.713471911	1
ENSBTAG00000009813	*ELOVL1*	3'856	3'380	3'621	3'339	3'903	0.225	0.771630967	1
ENSBTAG00000009816	*BT.41596*	63	13	35	55	15	-1.862	0.381530631	1
ENSBTAG00000009818	*pseudogene*	70	48	58	61	55	-0.129	0.948571971	1
ENSBTAG00000009819	*CDC20*	840	1'352	1'144	727	1'561	1.102	0.180973491	1
ENSBTAG00000009822	*PPP4R1*	2'782	3'572	3'267	2'409	4'125	0.776	0.321108381	1
ENSBTAG00000009823	*pseudogene*	3'150	1'308	2'119	2'728	1'510	-0.853	0.283133323	1
ENSBTAG00000009824	*BT.36467*	574	177	351	497	204	-1.282	0.176881944	1
ENSBTAG00000009826	*GPD1L*	567	439	499	491	507	0.046	0.960443888	1
ENSBTAG00000009828	*FXYD2*	22	12	16	19	14	-0.459	0.903495485	1
ENSBTAG00000009831	*OCA2*	127	116	122	110	134	0.284	0.819766058	1
ENSBTAG00000009832	*NOBOX*	84	41	60	73	47	-0.620	0.704401664	1
ENSBTAG00000009833	*DTHD1*	9	0	4	8	0		0.690676647	1
ENSBTAG00000009834	*CNGA3*	53	10	29	46	12	-1.991	0.400294731	1
ENSBTAG00000009835	*CACNA1G*	10'002	21'744	16'885	8'662	25'108	1.535	0.048242201	1
ENSBTAG00000009836	*CHGA*	572	230	380	495	266	-0.899	0.333795954	1
ENSBTAG00000009837	*ANKS4B*	8	1	4	7	1	-2.585	0.818599781	1
ENSBTAG00000009838	*RRP15*	554	435	491	480	502	0.066	0.942320112	1
ENSBTAG00000009839	*BT.64477*	1'520	1'110	1'299	1'316	1'282	-0.038	0.963819876	1
ENSBTAG00000009840	*OR2AE1*	2	1	1	2	1	-0.585	1	1
ENSBTAG00000009841	*CNDP2*	2'483	3'263	2'959	2'150	3'768	0.809	0.302206556	1
ENSBTAG00000009842	*CRYM*	1'070	831	943	927	960	0.050	0.952169727	1
ENSBTAG00000009844	*CYR61*	3'315	1'388	2'237	2'871	1'603	-0.841	0.28886976	1
ENSBTAG00000009845	*ITPK1*	472	682	598	409	788	0.946	0.277675754	1
ENSBTAG00000009846	*MTHFD1L*	1'059	1'138	1'116	917	1'314	0.519	0.526042166	1
ENSBTAG00000009848	*GPR171*	43	19	30	37	22	-0.763	0.744681388	1
ENSBTAG00000009849	*PLAC8*	45	21	32	39	24	-0.684	0.763007177	1
ENSBTAG00000009850	*GIMAP7*	7	5	6	6	6	-0.070	1	1
ENSBTAG00000009851	*ROBO1*	6'403	4'191	5'192	5'545	4'839	-0.196	0.799682488	1
ENSBTAG00000009852	*NAV3*	938	475	680	812	548	-0.567	0.508781069	1
ENSBTAG00000009854	*BT.52586*	453	553	515	392	639	0.703	0.427215614	1
ENSBTAG00000009855	*FAN1*	1'702	1'653	1'691	1'474	1'909	0.373	0.640035579	1
ENSBTAG00000009856	*ACAP3*	8'302	6'850	7'550	7'190	7'910	0.138	0.856934722	1
ENSBTAG00000009859	*SUMO1*	4'031	4'190	4'165	3'491	4'838	0.471	0.543159701	1
ENSBTAG00000009861	*FRS3*	192	184	189	166	212	0.354	0.743606416	1
ENSBTAG00000009862	*C14ORF105*	1	0	0	1	0		1	1
ENSBTAG00000009863	*BHLHE40*	1'272	415	790	1'102	479	-1.201	0.15827914	1
ENSBTAG00000009864	*B4GALNT2*	3	0	1	3	0		0.936647693	1
ENSBTAG00000009867	*FAM71F1*	6	3	4	5	3	-0.585	0.976586913	1
ENSBTAG00000009868	*VGF*	24	0	10	21	0		0.291206645	1
ENSBTAG00000009870	*HES6*	396	70	212	343	81	-2.085	0.053329809	1
ENSBTAG00000009871	*RAB18*	7'906	4'658	6'113	6'847	5'379	-0.348	0.651512539	1
ENSBTAG00000009872	*SPHK2*	780	1'285	1'080	675	1'484	1.135	0.170057818	1
ENSBTAG00000009873	*NRN1*	612	1'188	951	530	1'372	1.372	0.102332752	1
ENSBTAG00000009874	*ZXDC*	1'255	1'990	1'692	1'087	2'298	1.080	0.179536566	1
ENSBTAG00000009876	*C4BPA*	1	2	2	1	2	1.415	0.981979269	1
ENSBTAG00000009877	*MCMBP*	2'080	1'824	1'954	1'801	2'106	0.226	0.775651532	1
ENSBTAG00000009879	*PCGF6*	232	186	208	201	215	0.096	0.930643621	1
ENSBTAG00000009881	*BT.54429*	867	972	937	751	1'122	0.580	0.484573056	1
ENSBTAG00000009885	*BT.88373*	70	76	74	61	88	0.534	0.718748325	1
ENSBTAG00000009886	*KDELR3*	12'973	7'779	10'109	11'235	8'982	-0.323	0.67361291	1
ENSBTAG00000009887	*DDX17*	16'901	10'222	13'220	14'637	11'803	-0.310	0.684859599	1
ENSBTAG00000009888	*DRAM2*	793	686	739	687	792	0.206	0.808298996	1
ENSBTAG00000009889	*BT.59542*	1'432	755	1'056	1'240	872	-0.508	0.537103748	1
ENSBTAG00000009891	*PPP4R4*	323	81	187	280	94	-1.581	0.149645921	1
ENSBTAG00000009895	*ZNF599*	96	51	71	83	59	-0.497	0.744693345	1
ENSBTAG00000009897	*AGPS*	1'043	717	866	903	828	-0.126	0.882206778	1
ENSBTAG00000009899	*CHID1*	5'629	5'914	5'852	4'875	6'829	0.486	0.527569358	1
ENSBTAG00000009900	*pseudogene*	14	33	25	12	38	1.652	0.509598682	1
ENSBTAG00000009902	*AKR1B1*	3'698	2'256	2'904	3'203	2'605	-0.298	0.703818004	1
ENSBTAG00000009903	*PTCH1*	1'263	2'402	1'934	1'094	2'774	1.342	0.095153228	1
ENSBTAG00000009904	*BT.86769*	5	2	3	4	2	-0.907	0.965043111	1
ENSBTAG00000009905	*NFYA*	1'295	1'208	1'258	1'122	1'395	0.315	0.697908516	1
ENSBTAG00000009906	*DDX1*	8'889	5'992	7'309	7'698	6'919	-0.154	0.841702311	1
ENSBTAG00000009907	*MAPK4*	27	16	21	23	18	-0.340	0.919037185	1
ENSBTAG00000009908	*RPS3A*	30'327	22'525	26'137	26'264	26'010	-0.014	0.985998353	1
ENSBTAG00000009911	*HDC*	80	41	58	69	47	-0.549	0.741297114	1

Ensembl gene ID	geneName	counts wildtype horn bud	counts polled horn bud	baseMean	baseMean wildtype horn bud	baseMean polled horn bud	log2FoldChange	pval	padj
ENSBTAG00000009912	*BT.17386*	1'400	722	1'023	1'212	834	-0.540	0.512938611	1
ENSBTAG00000009913	*UPK3A*	11	0	5	10	0		0.617662983	1
ENSBTAG00000009914	*NDUFS6*	1'515	1'424	1'478	1'312	1'644	0.326	0.685027914	1
ENSBTAG00000009915	*AP2A2*	7'177	10'213	9'004	6'215	11'793	0.924	0.230424471	1
ENSBTAG00000009916	*WDYHV1*	1'530	1'205	1'358	1'325	1'391	0.071	0.930566189	1
ENSBTAG00000009917	*PIP5K1B*	115	30	67	100	35	-1.524	0.325515821	1
ENSBTAG00000009918	*KIAA0182*	886	830	863	767	958	0.321	0.700742316	1
ENSBTAG00000009923	*KIAA1737*	2'138	1'073	1'545	1'852	1'239	-0.580	0.471111427	1
ENSBTAG00000009924	*MR1*	252	296	280	218	342	0.647	0.50929716	1
ENSBTAG00000009928	*MLST8*	1'141	1'391	1'297	988	1'606	0.701	0.387843895	1
ENSBTAG00000009933	*DTX3L*	447	453	455	387	523	0.434	0.629442941	1
ENSBTAG00000009937	*protein_coding*	2	3	3	2	3	1.000	0.97171833	1
ENSBTAG00000009938	*XCL2*	1	0	0	1	0		1	1
ENSBTAG00000009939	*CAND1*	5'656	4'204	4'876	4'898	4'854	-0.013	0.987563912	1
ENSBTAG00000009941	*MBIP*	2'397	1'376	1'832	2'076	1'589	-0.386	0.628482302	1
ENSBTAG00000009942	*PLCL2*	2'243	1'073	1'591	1'942	1'239	-0.649	0.419359215	1
ENSBTAG00000009943	*BT.98003*	6	0	3	5	0		0.81115604	1
ENSBTAG00000009945	*C16ORF79*	18	20	19	16	23	0.567	0.856141506	1
ENSBTAG00000009948	*TRIM25*	538	680	626	466	785	0.753	0.383847093	1
ENSBTAG00000009949	*BT.48837*	4'738	3'587	4'123	4'103	4'142	0.014	0.985720685	1
ENSBTAG00000009950	*BT.97999*	60	28	42	52	32	-0.684	0.723678078	1
ENSBTAG00000009951	*PGP*	262	316	296	227	365	0.685	0.479788481	1
ENSBTAG00000009952	*C2ORF69*	360	138	236	312	159	-0.968	0.344319544	1
ENSBTAG00000009956	*CENPM*	575	652	625	498	753	0.596	0.489765494	1
ENSBTAG00000009958	*TRIM35*	196	226	215	170	261	0.621	0.550944211	1
ENSBTAG00000009959	*BT.88817*	1'211	2'023	1'692	1'049	2'336	1.155	0.151716566	1
ENSBTAG00000009960	*FLOT1*	4'457	5'438	5'070	3'860	6'279	0.702	0.36415899	1
ENSBTAG00000009961	*E4F1*	1'340	1'412	1'395	1'160	1'630	0.491	0.542950701	1
ENSBTAG00000009962	*PDCL3*	2'173	1'926	2'053	1'882	2'224	0.241	0.760301224	1
ENSBTAG00000009963	*MORC2*	2'571	1'819	2'163	2'227	2'100	-0.084	0.916305505	1
ENSBTAG00000009964	*DNASE1L2*	26	11	18	23	13	-0.826	0.800203209	1
ENSBTAG00000009965	*ECI1*	635	825	751	550	953	0.793	0.350128307	1
ENSBTAG00000009966	*XRCC3*	782	936	879	677	1'081	0.674	0.419281823	1
ENSBTAG00000009968	*TBX4*	6	0	3	5	0		0.81115604	1
ENSBTAG00000009969	*RNPS1*	4'615	4'923	4'841	3'997	5'685	0.508	0.51053594	1
ENSBTAG00000009972	*INHA*	50	46	48	43	53	0.295	0.876157524	1
ENSBTAG00000009974	*TSNARE1*	374	424	407	324	490	0.596	0.515136722	1
ENSBTAG00000009975	*PBX4*	40	47	44	35	54	0.648	0.728718344	1
ENSBTAG00000009976	*pseudogene*	835	1'568	1'267	723	1'811	1.324	0.107190147	1
ENSBTAG00000009978	*TDRD6*	283	92	176	245	106	-1.206	0.275389406	1
ENSBTAG00000009979	*BT.70281*	2	2	2	2	2	0.415	1	1
ENSBTAG00000009982	*IGSF8*	2'287	1'685	1'963	1'981	1'946	-0.026	0.975558231	1
ENSBTAG00000009983	*KIF23*	1'584	918	1'216	1'372	1'060	-0.372	0.648441424	1
ENSBTAG00000009984	*SGPL1*	3'217	3'167	3'221	2'786	3'657	0.392	0.614219432	1
ENSBTAG00000009985	*SIN3A*	2'254	2'015	2'139	1'952	2'327	0.253	0.747994987	1
ENSBTAG00000009987	*INTB3*	294	150	214	255	173	-0.556	0.595657639	1
ENSBTAG00000009988	*SMARCB1*	2'950	4'228	3'718	2'555	4'882	0.934	0.231737236	1
ENSBTAG00000009990	*EPS15L1*	2'507	1'837	2'146	2'171	2'121	-0.034	0.967404463	1
ENSBTAG00000009991	*CANT1*	579	504	542	501	582	0.215	0.807319772	1
ENSBTAG00000009994	*EML5*	518	304	400	449	351	-0.354	0.701801968	1
ENSBTAG00000009995	*CPEB4*	2'098	707	1'317	1'817	816	-1.154	0.158092081	1
ENSBTAG00000009996	*DOT1L*	1'490	2'638	2'168	1'290	3'046	1.239	0.120840643	1
ENSBTAG00000009997	*ANO1*	348	272	308	301	314	0.060	0.953414133	1
ENSBTAG00000010001	*BT.38815*	2'444	1'143	1'718	2'117	1'320	-0.681	0.394653535	1
ENSBTAG00000010002	*IRF2*	966	852	910	837	984	0.234	0.778472409	1
ENSBTAG00000010003	*TLX3*	11	8	9	10	9	-0.044	1	1
ENSBTAG00000010006	*EMC2*	2'657	1'634	2'094	2'301	1'887	-0.286	0.718038121	1
ENSBTAG00000010007	*MAPK13*	921	1'103	1'036	798	1'274	0.675	0.412530863	1
ENSBTAG00000010008	*TTC12*	77	8	38	67	9	-2.852	0.177117668	1
ENSBTAG00000010009	*TMCO1*	2'042	1'251	1'606	1'768	1'445	-0.292	0.716247814	1
ENSBTAG00000010010	*pseudogene*	3	0	1	3	0		0.936647693	1
ENSBTAG00000010012	*BTF3*	17'565	13'862	15'609	15'212	16'006	0.073	0.922575389	1
ENSBTAG00000010013	*DNAJA3*	1'313	1'402	1'378	1'137	1'619	0.510	0.527717545	1
ENSBTAG00000010015	*NMRAL1*	1'593	1'203	1'384	1'380	1'389	0.010	0.990515061	1
ENSBTAG00000010016	*BT.102218*	6	5	5	5	6	0.152	1	1
ENSBTAG00000010018	*ATP7A*	156	57	100	135	66	-1.037	0.430355418	1
ENSBTAG00000010023	*BT.87967*	1'017	950	989	881	1'097	0.317	0.701103801	1
ENSBTAG00000010024	*MMP24*	123	10	59	107	12	-3.206	0.066644642	1
ENSBTAG00000010026	*BT.31482*	130	45	82	113	52	-1.115	0.431700126	1
ENSBTAG00000010027	*BT.18996*	30	9	18	26	10	-1.322	0.669937554	1
ENSBTAG00000010029	*BT.17237*	103	51	74	89	59	-0.599	0.687788992	1
ENSBTAG00000010030	*BT.103480*	1'427	1'164	1'290	1'236	1'344	0.121	0.881202098	1

Ensembl gene ID	geneName	counts wildtype horn bud	counts polled horn bud	baseMean	baseMean wildtype horn bud	baseMean polled horn bud	log2FoldChange	pval	padj
ENSBTAG00000010032	*NTM*	285	88	174	247	102	-1.280	0.248590912	1
ENSBTAG00000010033	*BT.80259*	146	46	90	126	53	-1.251	0.362517512	1
ENSBTAG00000010035	*pseudogene*	0	1	1	0	1	Inf	0.993540919	1
ENSBTAG00000010036	*BT.20991*	2'856	2'119	2'460	2'473	2'447	-0.016	0.985456914	1
ENSBTAG00000010037	*TMEM241*	212	142	174	184	164	-0.163	0.887418326	1
ENSBTAG00000010042	*RIOK3*	4'294	1'941	2'980	3'719	2'241	-0.730	0.351699571	1
ENSBTAG00000010046	*BT.40961*	641	374	493	555	432	-0.362	0.685396092	1
ENSBTAG00000010047	*TIAM2*	1'201	637	888	1'040	736	-0.500	0.549673781	1
ENSBTAG00000010048	*SPC25*	126	107	116	109	124	0.179	0.890401133	1
ENSBTAG00000010050	*COL16A1*	85'027	145'832	121'014	73'636	168'392	1.193	0.119973635	1
ENSBTAG00000010051	*LGSN*	2	0	1	2	0		0.974934741	1
ENSBTAG00000010052	*EXD2*	223	266	250	193	307	0.669	0.505266995	1
ENSBTAG00000010059	*C8H9orf72*	1'800	703	1'185	1'559	812	-0.941	0.251100504	1
ENSBTAG00000010060	*SLC43A3*	322	171	238	279	197	-0.498	0.625902402	1
ENSBTAG00000010062	*SLC26A4*	6	1	3	5	1	-2.170	0.885240772	1
ENSBTAG00000010063	*KDM3B*	4'250	3'157	3'663	3'681	3'645	-0.014	0.986830625	1
ENSBTAG00000010065	*BT.29713*	2	2	2	2	2	0.415	1	1
ENSBTAG00000010068	*REEP2*	750	265	478	650	306	-1.086	0.228038147	1
ENSBTAG00000010069	*EGR1*	2'356	1'749	2'030	2'040	2'020	-0.015	0.986507893	1
ENSBTAG00000010070	*BT.62411*	233	75	144	202	87	-1.220	0.297054989	1
ENSBTAG00000010071	*FBXO3*	2'938	2'093	2'481	2'544	2'417	-0.074	0.925853687	1
ENSBTAG00000010073	*FLOT2*	1'258	2'002	1'701	1'089	2'312	1.085	0.177382238	1
ENSBTAG00000010077	*FANCD2*	918	691	796	795	798	0.005	0.996079153	1
ENSBTAG00000010082	*COL15A1*	13'136	3'540	7'732	11'376	4'088	-1.477	0.058656674	1
ENSBTAG00000010083	*HADHB*	3'514	3'296	3'425	3'043	3'806	0.323	0.677900392	1
ENSBTAG00000010085	*SLC7A2*	294	107	189	255	124	-1.043	0.334832903	1
ENSBTAG00000010089	*CALC3*	3	3	3	3	3	0.415	1	1
ENSBTAG00000010091	*ARL6*	656	331	475	568	382	-0.572	0.523904913	1
ENSBTAG00000010094	*C22H3orf24*	6	2	4	5	2	-1.170	0.934316631	1
ENSBTAG00000010096	*BT.62759*	876	745	809	759	860	0.181	0.829359647	1
ENSBTAG00000010100	*MED20*	1'437	1'206	1'319	1'244	1'393	0.162	0.841045525	1
ENSBTAG00000010101	*BYSL*	480	517	506	416	597	0.522	0.555777973	1
ENSBTAG00000010105	*TMEM20*	110	26	63	95	30	-1.666	0.298016202	1
ENSBTAG00000010106	*BT.29057*	1'598	1'624	1'630	1'384	1'875	0.438	0.583391456	1
ENSBTAG00000010107	*DFNB31*	118	169	149	102	195	0.933	0.417474913	1
ENSBTAG00000010109	*CDK1*	789	778	791	683	898	0.395	0.639007729	1
ENSBTAG00000010111	*BT.44249*	1'464	2'962	2'344	1'268	3'420	1.432	0.073558712	1
ENSBTAG00000010112	*BPIFB6*	7	17	13	6	20	1.695	0.660205265	1
ENSBTAG00000010113	*ZBTB24*	651	725	700	564	837	0.570	0.503488231	1
ENSBTAG00000010114	*protein_coding*	2'296	955	1'546	1'988	1'103	-0.851	0.291500074	1
ENSBTAG00000010116	*AKD1*	102	65	82	88	75	-0.235	0.875935869	1
ENSBTAG00000010118	*HAT1*	2'651	1'788	2'180	2'296	2'065	-0.153	0.847168158	1
ENSBTAG00000010119	*ALDH1A2*	20	1	9	17	1	-3.907	0.484363729	1
ENSBTAG00000010120	*HERC3*	1'010	549	754	875	634	-0.464	0.584106845	1
ENSBTAG00000010123	*APOE*	4'794	2'180	3'334	4'152	2'517	-0.722	0.355887279	1
ENSBTAG00000010124	*BT.31619*	1'044	553	771	904	639	-0.502	0.553365696	1
ENSBTAG00000010125	*STAT5B*	954	822	888	826	949	0.200	0.810228393	1
ENSBTAG00000010126	*BT.24835*	2'109	3'680	3'038	1'826	4'249	1.218	0.122904696	1
ENSBTAG00000010127	*MIP*	8	3	5	7	3	-1.000	0.918861955	1
ENSBTAG00000010128	*NAP1L5*	499	647	590	432	747	0.790	0.364575671	1
ENSBTAG00000010129	*SLITRK5*	204	31	106	177	36	-2.303	0.083894781	1
ENSBTAG00000010130	*ZNF335*	1'640	2'432	2'114	1'420	2'808	0.983	0.216362051	1
ENSBTAG00000010131	*CBFA2T2*	425	408	420	368	471	0.356	0.696177134	1
ENSBTAG00000010132	*NAE1*	2'552	1'188	1'791	2'210	1'372	-0.688	0.389135561	1
ENSBTAG00000010134	*OSTM1*	1'379	558	919	1'194	644	-0.890	0.286600999	1
ENSBTAG00000010135	*TMEM189*	949	1'061	1'023	822	1'225	0.576	0.48448547	1
ENSBTAG00000010136	*CMAS*	2'486	1'210	1'775	2'153	1'397	-0.624	0.434901916	1
ENSBTAG00000010138	*SEMA3B*	10'447	9'429	9'968	9'047	10'888	0.267	0.726302641	1
ENSBTAG00000010144	*MTERFD3*	758	287	494	656	331	-0.986	0.270671587	1
ENSBTAG00000010145	*SLC12A1*	6	0	3	5	0		0.81115604	1
ENSBTAG00000010146	*PLXNB3*	623	288	436	540	333	-0.698	0.442451514	1
ENSBTAG00000010148	*processed_pseudogene*	1	8	5	1	9	3.415	0.697043054	1
ENSBTAG00000010149	*CRY1*	1'335	792	1'035	1'156	915	-0.338	0.682082232	1
ENSBTAG00000010151	*CA5A*	16	5	10	14	6	-1.263	0.797689913	1
ENSBTAG00000010152	*MID1*	1'916	1'068	1'446	1'659	1'233	-0.428	0.595683443	1
ENSBTAG00000010153	*ANXA3*	804	428	595	696	494	-0.495	0.570150178	1
ENSBTAG00000010155	*protein_coding*	552	581	574	478	671	0.489	0.574937167	1
ENSBTAG00000010157	*TOMM22*	4'272	4'062	4'195	3'700	4'690	0.342	0.657998391	1
ENSBTAG00000010158	*BT.106399*	293	597	472	254	689	1.442	0.112207523	1
ENSBTAG00000010161	*BT.26536*	403	327	363	349	378	0.114	0.90505565	1
ENSBTAG00000010163	*BT.17819*	50	17	31	43	20	-1.141	0.608722633	1
ENSBTAG00000010164	*ESPNL*	299	410	366	259	473	0.871	0.351322314	1

Ensembl gene ID	geneName	counts wildtype horn bud	counts polled horn bud	baseMean	baseMean wildtype horn bud	baseMean polled horn bud	log2FoldChange	pval	padj
ENSBTAG00000010165	*C22H3ORF18*	319	329	328	276	380	0.460	0.62889533	1
ENSBTAG00000010166	*BT.29684*	124	31	72	107	36	-1.585	0.294151816	1
ENSBTAG00000010167	*TEX14*	165	74	114	143	85	-0.742	0.555468766	1
ENSBTAG00000010169	*C1ORF9*	2'107	1'552	1'808	1'825	1'792	-0.026	0.975357626	1
ENSBTAG00000010170	*MBTPS1*	7'884	8'017	8'042	6'828	9'257	0.439	0.566530653	1
ENSBTAG00000010171	*JOSD1*	2'264	2'439	2'388	1'961	2'816	0.522	0.506681759	1
ENSBTAG00000010174	*C3H1orf111*	17	9	13	15	10	-0.503	0.915336184	1
ENSBTAG00000010175	*COL8A2*	1'511	518	953	1'309	598	-1.129	0.176765174	1
ENSBTAG00000010176	*ILKAP*	3'175	2'864	3'028	2'750	3'307	0.266	0.73248514	1
ENSBTAG00000010177	*GOLT1A*	2	1	1	2	1	-0.585	1	1
ENSBTAG00000010178	*PTPRD*	3'840	1'091	2'293	3'326	1'260	-1.400	0.080202407	1
ENSBTAG00000010179	*COL5A3*	9'280	8'349	8'839	8'037	9'641	0.263	0.731129769	1
ENSBTAG00000010180	*BT.30956*	261	384	335	226	443	0.972	0.306290668	1
ENSBTAG00000010181	*GRPEL1*	1'501	1'370	1'441	1'300	1'582	0.283	0.724574405	1
ENSBTAG00000010182	*ACR*	5	3	4	4	3	-0.322	1	1
ENSBTAG00000010185	*BT.18422*	185	233	215	160	269	0.748	0.472715056	1
ENSBTAG00000010188	*RDH8*	1	4	3	1	5	2.415	0.884888347	1
ENSBTAG00000010191	*PAK1*	4'138	2'421	3'190	3'584	2'796	-0.358	0.646540015	1
ENSBTAG00000010192	*BT.52985*	115	65	87	100	75	-0.408	0.772482285	1
ENSBTAG00000010193	*BT.41314*	5'265	5'620	5'525	4'560	6'489	0.509	0.508813797	1
ENSBTAG00000010195	*PKN3*	1'235	740	962	1'070	854	-0.324	0.696654519	1
ENSBTAG00000010196	*NUP43*	1'455	1'217	1'333	1'260	1'405	0.157	0.845649956	1
ENSBTAG00000010198	*ATF7IP2*	18	3	10	16	3	-2.170	0.662488565	1
ENSBTAG00000010204	*PCMT1*	2'536	2'177	2'355	2'196	2'514	0.195	0.804063628	1
ENSBTAG00000010206	*UAP1*	8'638	4'675	6'439	7'481	5'398	-0.471	0.541280107	1
ENSBTAG00000010207	*NUDT21*	1'006	735	860	871	849	-0.038	0.966146613	1
ENSBTAG00000010208	*ZER1*	978	1'537	1'311	847	1'775	1.067	0.190788765	1
ENSBTAG00000010210	*SUV39H2*	357	375	371	309	433	0.486	0.601210113	1
ENSBTAG00000010215	*CREB3L3*	2	0	1	2	0		0.974934741	1
ENSBTAG00000010217	*ZNF318*	3'115	2'076	2'547	2'698	2'397	-0.170	0.829055122	1
ENSBTAG00000010219	*KRBA1*	784	867	840	679	1'001	0.560	0.50352934	1
ENSBTAG00000010220	*DCLRE1C*	562	315	425	487	364	-0.420	0.645541098	1
ENSBTAG00000010221	*BT.24278*	885	670	770	766	774	0.014	0.988272236	1
ENSBTAG00000010223	*NTF3*	460	250	344	398	289	-0.465	0.623613002	1
ENSBTAG00000010225	*POLR2D*	1'156	977	1'065	1'001	1'128	0.172	0.833801676	1
ENSBTAG00000010227	*CPSF2*	2'099	1'832	1'967	1'818	2'115	0.219	0.782180111	1
ENSBTAG00000010229	*LAMA2*	9'101	6'325	7'593	7'882	7'303	-0.110	0.886787838	1
ENSBTAG00000010230	*CAPN1*	7'126	11'169	9'534	6'171	12'897	1.063	0.16839498	1
ENSBTAG00000010232	*NDUFS5*	6'121	5'404	5'770	5'301	6'240	0.235	0.759297054	1
ENSBTAG00000010234	*DHRS1*	2'619	2'227	2'420	2'268	2'572	0.181	0.817394793	1
ENSBTAG00000010235	*C10H14orf21*	908	886	905	786	1'023	0.380	0.647887889	1
ENSBTAG00000010236	*CIDEB*	442	288	358	383	333	-0.203	0.831149839	1
ENSBTAG00000010238	*LTB4R*	181	102	137	157	118	-0.412	0.730062199	1
ENSBTAG00000010239	*BT.54344*	44	5	22	38	6	-2.722	0.338083518	1
ENSBTAG00000010241	*UNC5D*	24	7	14	21	8	-1.363	0.707711751	1
ENSBTAG00000010242	*LAMP1*	13'044	15'758	14'746	11'296	18'196	0.688	0.368459791	1
ENSBTAG00000010243	*RBM33*	1'306	1'379	1'362	1'131	1'592	0.494	0.541114491	1
ENSBTAG00000010244	*CLIC5*	27	18	22	23	21	-0.170	0.968634064	1
ENSBTAG00000010245	*SPRY3*	33	13	22	29	15	-0.929	0.739894541	1
ENSBTAG00000010246	*GRTP1*	239	299	276	207	345	0.738	0.453130063	1
ENSBTAG00000010247	*FKTN*	93	67	79	81	77	-0.058	0.978500785	1
ENSBTAG00000010249	*TMEM222*	1'541	1'540	1'556	1'335	1'778	0.414	0.605170974	1
ENSBTAG00000010252	*BT.28733*	2'703	1'822	2'222	2'341	2'104	-0.154	0.84620266	1
ENSBTAG00000010253	*SYTL1*	624	785	723	540	906	0.746	0.380638359	1
ENSBTAG00000010254	*MAP3K6*	541	667	619	469	770	0.717	0.407263445	1
ENSBTAG00000010255	*ZNF24*	920	655	777	797	756	-0.075	0.931243398	1
ENSBTAG00000010256	*TMEM170B*	17	8	12	15	9	-0.672	0.883107327	1
ENSBTAG00000010264	*FBXL18*	138	312	240	120	360	1.592	0.122079417	1
ENSBTAG00000010265	*MGST3*	4'310	3'115	3'665	3'733	3'597	-0.053	0.946173964	1
ENSBTAG00000010270	*BT.37893*	92	106	101	80	122	0.619	0.63598649	1
ENSBTAG00000010271	*MAPKAP1*	1'686	1'302	1'482	1'460	1'503	0.042	0.95833095	1
ENSBTAG00000010273	*EREG*	9	1	4	8	1	-2.755	0.785977687	1
ENSBTAG00000010275	*KIAA0196*	2'778	2'128	2'432	2'406	2'457	0.030	0.968922387	1
ENSBTAG00000010276	*RECQL4*	342	541	460	296	625	1.077	0.233545546	1
ENSBTAG00000010277	*BT.48094*	37	3	18	32	3	-3.209	0.334323923	1
ENSBTAG00000010278	*STPG1*	19	26	23	16	30	0.868	0.744004518	1
ENSBTAG00000010279	*DUSP14*	571	392	474	495	453	-0.128	0.88935853	1
ENSBTAG00000010284	*AGXT2L1*	26	7	15	23	8	-1.478	0.671006397	1
ENSBTAG00000010285	*MMRN1*	457	166	294	396	192	-1.046	0.284628356	1
ENSBTAG00000010286	*NPAT*	1'559	1'207	1'372	1'350	1'394	0.046	0.954939393	1
ENSBTAG00000010289	*GEMIN8*	714	834	791	618	963	0.639	0.448192882	1
ENSBTAG00000010290	*GGNBP2*	3'481	1'712	2'496	3'015	1'977	-0.609	0.439708458	1

Ensembl gene ID	geneName	counts wildtype horn bud	counts polled horn bud	baseMean	baseMean wildtype horn bud	baseMean polled horn bud	log2FoldChange	pval	padj
ENSBTAG00000010291	*RBAK*	956	415	654	828	479	-0.789	0.360397772	1
ENSBTAG00000010292	*BT.48292*	1'256	1'029	1'138	1'088	1'188	0.127	0.876182579	1
ENSBTAG00000010293	*PARD3B*	55	75	67	48	87	0.862	0.573377085	1
ENSBTAG00000010297	*DHRS11*	1'395	1'372	1'396	1'208	1'584	0.391	0.627459832	1
ENSBTAG00000010298	*GTF2A2*	2'462	1'580	1'978	2'132	1'824	-0.225	0.777520543	1
ENSBTAG00000010299	*BT.39878*	619	124	340	536	143	-1.905	0.049819423	1
ENSBTAG00000010300	*BT.91596*	4	4	4	3	5	0.415	0.994759363	1
ENSBTAG00000010303	*ICAM1*	583	837	736	505	966	0.937	0.271507934	1
ENSBTAG00000010304	*CHKA*	564	175	345	488	202	-1.273	0.18106472	1
ENSBTAG00000010306	*RXFP1*	59	0	26	51	0		0.041133735	1
ENSBTAG00000010309	*XPO6*	2'939	3'293	3'174	2'545	3'802	0.579	0.458012676	1
ENSBTAG00000010311	*MRM1*	313	328	325	271	379	0.483	0.61241963	1
ENSBTAG00000010312	*MAPK1*	1'718	2'153	1'987	1'488	2'486	0.741	0.351209591	1
ENSBTAG00000010313	*DDX52*	1'321	1'028	1'166	1'144	1'187	0.053	0.948310469	1
ENSBTAG00000010316	*BT.34883*	31	5	16	27	6	-2.217	0.509474881	1
ENSBTAG00000010318	*FDX1L*	579	638	619	501	737	0.555	0.520721341	1
ENSBTAG00000010321	*TTC1*	1'230	1'140	1'191	1'065	1'316	0.305	0.707438515	1
ENSBTAG00000010322	*BT.39906*	2'185	4'322	3'441	1'892	4'991	1.399	0.076688693	1
ENSBTAG00000010324	*GTPBP1*	1'734	3'533	2'791	1'502	4'080	1.442	0.069999413	1
ENSBTAG00000010326	*AADAT*	529	440	483	458	508	0.149	0.868221705	1
ENSBTAG00000010328	*KCNK17*	12	3	7	10	3	-1.585	0.809704871	1
ENSBTAG00000010330	*LYZL1*	9	9	9	8	10	0.415	0.953225767	1
ENSBTAG00000010334	*SYTL3*	61	19	37	53	22	-1.268	0.532532503	1
ENSBTAG00000010336	*TALDO1*	4'098	5'207	4'781	3'549	6'013	0.761	0.326451415	1
ENSBTAG00000010337	*ALG11*	1'228	1'126	1'182	1'063	1'300	0.290	0.721772767	1
ENSBTAG00000010338	*FBXO36*	134	97	114	116	112	-0.051	0.976090334	1
ENSBTAG00000010339	*ABHD11*	1'410	1'191	1'298	1'221	1'375	0.172	0.832207895	1
ENSBTAG00000010341	*PWWP2A*	819	566	681	709	654	-0.118	0.892372985	1
ENSBTAG00000010343	*BT.19274*	203	210	209	176	242	0.464	0.658518605	1
ENSBTAG00000010344	*BT.40559*	69	37	51	60	43	-0.484	0.786188552	1
ENSBTAG00000010345	*BT.88804*	126	202	171	109	233	1.096	0.322519744	1
ENSBTAG00000010346	*ENAM*	1	0	0	1	0		1	1
ENSBTAG00000010347	*EZR*	6'639	4'854	5'677	5'750	5'605	-0.037	0.96283313	1
ENSBTAG00000010348	*PDDC1*	2'401	2'087	2'245	2'079	2'410	0.213	0.786778428	1
ENSBTAG00000010349	*IL1A*	10	3	6	9	3	-1.322	0.863266279	1
ENSBTAG00000010350	*BT.95206*	2'320	704	1'411	2'009	813	-1.305	0.110031231	1
ENSBTAG00000010351	*SNX25*	1'142	407	729	989	470	-1.073	0.20975771	1
ENSBTAG00000010352	*KCNK16*	1	0	0	1	0		1	1
ENSBTAG00000010353	*BT.28678*	329	247	285	285	285	0.001	1	1
ENSBTAG00000010355	*VPS37A*	1'558	785	1'128	1'349	906	-0.574	0.484004002	1
ENSBTAG00000010356	*RAB12*	3'152	1'623	2'302	2'730	1'874	-0.543	0.492112603	1
ENSBTAG00000010357	*BT.91730*	329	228	274	285	263	-0.114	0.912075826	1
ENSBTAG00000010359	*THG1L*	1'920	1'166	1'505	1'663	1'346	-0.305	0.705488679	1
ENSBTAG00000010360	*LRIG1*	6'877	10'325	8'939	5'956	11'922	1.001	0.194374269	1
ENSBTAG00000010361	*DLL4*	766	522	633	663	603	-0.138	0.874751692	1
ENSBTAG00000010362	*BT.17865*	190	57	115	165	66	-1.322	0.293806986	1
ENSBTAG00000010363	*LSM11*	85	53	67	74	61	-0.266	0.870232519	1
ENSBTAG00000010365	*SQRDL*	546	190	346	473	219	-1.108	0.242875677	1
ENSBTAG00000010366	*HCRTR1*	154	170	165	133	196	0.558	0.617774088	1
ENSBTAG00000010367	*TTC13*	848	372	582	734	430	-0.774	0.376091784	1
ENSBTAG00000010368	*BT.65031*	944	655	787	818	756	-0.112	0.895994288	1
ENSBTAG00000010370	*C21H14orf79*	144	134	140	125	155	0.311	0.7933106	1
ENSBTAG00000010371	*CHAC1*	38	32	35	33	37	0.167	0.948891151	1
ENSBTAG00000010372	*BT.91802*	505	126	291	437	145	-1.588	0.108862296	1
ENSBTAG00000010373	*C19ORF29*	1'456	1'960	1'762	1'261	2'263	0.844	0.291442882	1
ENSBTAG00000010375	*MSL1*	4'131	2'596	3'288	3'578	2'998	-0.255	0.743958008	1
ENSBTAG00000010376	*TMEM34*	4'225	3'067	3'600	3'659	3'541	-0.047	0.952704678	1
ENSBTAG00000010378	*PEF1*	3'262	3'054	3'176	2'825	3'526	0.320	0.681009103	1
ENSBTAG00000010379	*CRTC1*	586	974	816	507	1'125	1.148	0.175139257	1
ENSBTAG00000010380	*INO80*	1'188	866	1'014	1'029	1'000	-0.041	0.962237457	1
ENSBTAG00000010381	*MGC151858*	2'145	1'091	1'559	1'858	1'260	-0.560	0.485750637	1
ENSBTAG00000010382	*MUC13*	1	1	1	1	1	0.415	1	1
ENSBTAG00000010383	*YTHDF1*	1'597	1'682	1'663	1'383	1'942	0.490	0.53968983	1
ENSBTAG00000010384	*CDC6*	375	437	415	325	505	0.636	0.486280066	1
ENSBTAG00000010386	*ARPC2*	12'443	9'438	10'837	10'776	10'898	0.016	0.982474842	1
ENSBTAG00000010387	*FAM188A*	2'002	697	1'269	1'734	805	-1.107	0.176332855	1
ENSBTAG00000010388	*MGAT4A*	95	47	68	82	54	-0.600	0.697391208	1
ENSBTAG00000010389	*STBD1*	763	749	763	661	865	0.388	0.645675196	1
ENSBTAG00000010390	*SRI*	2'080	1'675	1'868	1'801	1'934	0.103	0.897067234	1
ENSBTAG00000010392	*ESRRG*	22	21	22	19	24	0.348	0.911694943	1
ENSBTAG00000010393	*TRNAU1AP*	841	485	644	728	560	-0.379	0.660723816	1
ENSBTAG00000010394	*MCF2L2*	47	5	23	41	6	-2.818	0.307057447	1

Ensembl gene ID	geneName	counts wildtype horn bud	counts polled horn bud	baseMean	baseMean wildtype horn bud	baseMean polled horn bud	log2FoldChange	pval	padj
ENSBTAG00000010395	*DOCK9*	4'855	2'446	3'514	4'205	2'824	-0.574	0.461681959	1
ENSBTAG00000010397	*B3GNT5*	371	269	316	321	311	-0.049	0.963701773	1
ENSBTAG00000010399	*TXNDC16*	263	256	262	228	296	0.376	0.706098307	1
ENSBTAG00000010401	*UBE4B*	3'630	2'398	2'956	3'144	2'769	-0.183	0.815533002	1
ENSBTAG00000010402	*MYH9*	17'408	16'141	16'857	15'076	18'638	0.306	0.687438033	1
ENSBTAG00000010403	*FAAP24*	436	423	433	378	488	0.371	0.682324383	1
ENSBTAG00000010407	*BT.106434*	207	444	346	179	513	1.516	0.112486984	1
ENSBTAG00000010408	*BT.54851*	558	418	483	483	483	-0.002	1	1
ENSBTAG00000010413	*TMEM169*	111	43	73	96	50	-0.953	0.522057868	1
ENSBTAG00000010414	*DMRTA2*	29	7	17	25	8	-1.636	0.618934669	1
ENSBTAG00000010416	*RIN3*	746	1'661	1'282	646	1'918	1.570	0.057484292	1
ENSBTAG00000010419	*BT.28622*	172	141	156	149	163	0.128	0.914344216	1
ENSBTAG00000010420	*PCDH17*	219	53	125	190	61	-1.632	0.185716861	1
ENSBTAG00000010422	*MDM2*	1'560	1'552	1'572	1'351	1'792	0.408	0.610632446	1
ENSBTAG00000010423	*LIFR*	119	76	95	103	88	-0.232	0.869105985	1
ENSBTAG00000010426	*BT.88138*	1'853	1'786	1'834	1'605	2'062	0.362	0.648635934	1
ENSBTAG00000010427	*BT.22323*	344	116	216	298	134	-1.153	0.271066456	1
ENSBTAG00000010428	*USP15*	2'831	1'408	2'039	2'452	1'626	-0.593	0.455336894	1
ENSBTAG00000010431	*LURAP1L*	639	344	475	553	397	-0.478	0.594049061	1
ENSBTAG00000010432	*BT.59019*	1'946	1'274	1'578	1'685	1'471	-0.196	0.807677215	1
ENSBTAG00000010433	*M-SAA3.2*	0	2	1	0	2	Inf	0.939077559	1
ENSBTAG00000010437	*MKLN1*	644	386	502	558	446	-0.323	0.717218915	1
ENSBTAG00000010439	*AKAP8L*	4'520	3'462	3'956	3'914	3'998	0.030	0.968454244	1
ENSBTAG00000010442	*PANK1*	784	552	658	679	637	-0.091	0.917779696	1
ENSBTAG00000010444	*ERBB3*	3'419	2'636	3'002	2'961	3'044	0.040	0.959071213	1
ENSBTAG00000010445	*BT.95252*	0	1	1	0	1	Inf	0.993540919	1
ENSBTAG00000010447	*LSP1*	860	589	712	745	680	-0.131	0.879660491	1
ENSBTAG00000010448	*CPNE4*	123	40	76	107	46	-1.206	0.409734327	1
ENSBTAG00000010449	*MOCS1*	391	541	482	339	625	0.883	0.323725936	1
ENSBTAG00000010450	*WIZ*	1'808	2'994	2'511	1'566	3'457	1.143	0.149580597	1
ENSBTAG00000010451	*PA2G4*	7'544	7'477	7'583	6'533	8'634	0.402	0.599772991	1
ENSBTAG00000010452	*PODXL*	663	313	468	574	361	-0.668	0.457764115	1
ENSBTAG00000010455	*KIAA1698*	653	1'004	862	566	1'159	1.036	0.218024498	1
ENSBTAG00000010456	*TADA1*	2'033	1'163	1'552	1'761	1'343	-0.391	0.626905226	1
ENSBTAG00000010457	*NUAK1*	473	358	412	410	413	0.013	0.990851208	1
ENSBTAG00000010458	*BT.45096*	52	25	37	45	29	-0.642	0.758368091	1
ENSBTAG00000010460	*MARK1*	2'512	1'321	1'850	2'175	1'525	-0.512	0.520387335	1
ENSBTAG00000010462	*ROBO2*	2'602	2'719	2'697	2'253	3'140	0.478	0.541267575	1
ENSBTAG00000010463	*BT.72941*	178	45	103	154	52	-1.569	0.232911152	1
ENSBTAG00000010464	*MN1*	1'254	844	1'030	1'086	975	-0.156	0.851024421	1
ENSBTAG00000010465	*MGC134282*	882	813	851	764	939	0.298	0.721914673	1
ENSBTAG00000010467	*BT.43451*	7'056	7'721	7'513	6'111	8'915	0.545	0.477796957	1
ENSBTAG00000010470	*C29H11orf83*	1'120	1'035	1'083	970	1'195	0.301	0.713201473	1
ENSBTAG00000010472	*TNFAIP8L3*	438	361	398	379	417	0.136	0.883789734	1
ENSBTAG00000010475	*MLNR*	1	0	0	1	0		1	1
ENSBTAG00000010477	*TTC21A*	460	243	339	398	281	-0.506	0.593949394	1
ENSBTAG00000010478	*AIP*	1'240	1'426	1'360	1'074	1'647	0.617	0.445625463	1
ENSBTAG00000010480	*BT.49082*	1'502	1'478	1'504	1'301	1'707	0.392	0.625344037	1
ENSBTAG00000010481	*UBXN1*	2'043	2'235	2'175	1'769	2'581	0.545	0.490427826	1
ENSBTAG00000010482	*PITPNM1*	916	1'129	1'048	793	1'304	0.717	0.384215178	1
ENSBTAG00000010483	*DLL3*	123	11	60	107	13	-3.068	0.07580492	1
ENSBTAG00000010484	*LRRN4CL*	1'226	1'454	1'370	1'062	1'679	0.661	0.413562358	1
ENSBTAG00000010485	*MFN1*	5'249	2'371	3'642	4'546	2'738	-0.732	0.348490475	1
ENSBTAG00000010487	*MGC128424*	1'809	781	1'234	1'567	902	-0.797	0.329143481	1
ENSBTAG00000010490	*PEX5*	2'063	2'521	2'349	1'787	2'911	0.704	0.372026466	1
ENSBTAG00000010492	*VPS4B*	2'701	1'773	2'193	2'339	2'047	-0.192	0.808450164	1
ENSBTAG00000010493	*SERTAD1*	700	784	756	606	905	0.579	0.494073468	1
ENSBTAG00000010496	*BT.45335*	1'147	483	776	993	558	-0.833	0.325941825	1
ENSBTAG00000010497	*TRAF2*	1'116	1'262	1'212	966	1'457	0.592	0.466843531	1
ENSBTAG00000010498	*SLC40A1*	5'263	2'546	3'749	4'558	2'940	-0.633	0.416721983	1
ENSBTAG00000010500	*BT.76502*	1'031	527	751	893	609	-0.553	0.514606577	1
ENSBTAG00000010501	*BT.51555*	26	3	13	23	3	-2.700	0.499863335	1
ENSBTAG00000010502	*SERTAD3*	1'900	1'964	1'957	1'645	2'268	0.463	0.559231095	1
ENSBTAG00000010503	*TIMM9*	939	412	644	813	476	-0.773	0.370542656	1
ENSBTAG00000010504	*TBRG4*	1'326	1'658	1'531	1'148	1'914	0.737	0.359235772	1
ENSBTAG00000010505	*INTS4*	1'400	1'167	1'280	1'212	1'348	0.152	0.850838496	1
ENSBTAG00000010506	*C3H1ORF149*	2'991	2'213	2'573	2'590	2'555	-0.020	0.981318795	1
ENSBTAG00000010507	*SLC22A16*	54	45	49	47	52	0.152	0.941451331	1
ENSBTAG00000010508	*BLVRB*	1'132	1'300	1'241	980	1'501	0.615	0.44967409	1
ENSBTAG00000010509	*CDK3*	1'106	640	848	958	739	-0.374	0.655787285	1
ENSBTAG00000010510	*DHX29*	4'296	2'061	3'050	3'720	2'380	-0.645	0.410474083	1
ENSBTAG00000010511	*BT.77188*	2'844	2'341	2'583	2'463	2'703	0.134	0.863755751	1

Ensembl gene ID	geneName	counts wildtype horn bud	counts polled horn bud	baseMean	baseMean wildtype horn bud	baseMean polled horn bud	log2FoldChange	pval	padj
ENSBTAG00000010513	*GPS2*	2'056	1'524	1'770	1'781	1'760	-0.017	0.984523442	1
ENSBTAG00000010514	*ASB7*	286	223	253	248	257	0.056	0.958820056	1
ENSBTAG00000010515	*FBXW5*	1'727	1'865	1'825	1'496	2'154	0.526	0.50849385	1
ENSBTAG00000010517	*EVPL*	3'662	4'381	4'115	3'171	5'059	0.674	0.38557818	1
ENSBTAG00000010518	*BT.62842*	1'689	1'066	1'347	1'463	1'231	-0.249	0.759148724	1
ENSBTAG00000010519	*NEURL4*	3'345	4'624	4'118	2'897	5'339	0.882	0.257334129	1
ENSBTAG00000010520	*C8G*	813	203	469	704	234	-1.587	0.082266536	1
ENSBTAG00000010521	*PVRIG*	12	7	9	10	8	-0.363	0.964206651	1
ENSBTAG00000010522	*protein_coding*	70	14	38	61	16	-1.907	0.346286566	1
ENSBTAG00000010524	*BCKDK*	1'360	1'753	1'601	1'178	2'024	0.781	0.330546401	1
ENSBTAG00000010526	*PPAP2A*	7'313	4'534	5'784	6'333	5'235	-0.275	0.721961451	1
ENSBTAG00000010527	*ACAP1*	1'671	1'204	1'419	1'447	1'390	-0.058	0.944329275	1
ENSBTAG00000010529	*BT.104004*	1'382	1'155	1'265	1'197	1'334	0.156	0.847316197	1
ENSBTAG00000010532	*BT.24709*	1'264	1'271	1'281	1'095	1'468	0.423	0.601591472	1
ENSBTAG00000010533	*HMGXB4*	877	1'002	958	760	1'157	0.607	0.463516926	1
ENSBTAG00000010534	*M-RIP*	8'633	13'037	11'265	7'476	15'054	1.010	0.189831109	1
ENSBTAG00000010536	*protein_coding*	4	2	3	3	2	-0.585	0.996239345	1
ENSBTAG00000010541	*ZNF346*	890	511	680	771	590	-0.385	0.653366951	1
ENSBTAG00000010542	*SPIRE1*	501	372	432	434	430	-0.014	0.990977455	1
ENSBTAG00000010543	*FGFR4*	391	974	732	339	1'125	1.732	0.046040818	1
ENSBTAG00000010544	*pseudogene*	8	10	9	7	12	0.737	0.893887116	1
ENSBTAG00000010545	*BT.87557*	2'051	1'890	1'979	1'776	2'182	0.297	0.707304867	1
ENSBTAG00000010546	*LIN54*	1'273	694	952	1'102	801	-0.460	0.579548424	1
ENSBTAG00000010547	*CEP76*	726	432	564	629	499	-0.334	0.70394412	1
ENSBTAG00000010548	*LRRC6*	103	123	116	89	142	0.671	0.590453381	1
ENSBTAG00000010549	*IFRD1*	1'984	927	1'394	1'718	1'070	-0.683	0.398972256	1
ENSBTAG00000010550	*EIF5A2*	42	55	50	36	64	0.804	0.646219093	1
ENSBTAG00000010552	*PSMG2*	1'306	1'001	1'143	1'131	1'156	0.031	0.969863612	1
ENSBTAG00000010555	*BT.35621*	22	20	21	19	23	0.278	0.935151797	1
ENSBTAG00000010559	*BT.89455*	924	885	911	800	1'022	0.353	0.671066145	1
ENSBTAG00000010562	*CD34*	5'126	7'620	6'619	4'439	8'799	0.987	0.202267763	1
ENSBTAG00000010563	*PTPN2*	2'148	1'508	1'801	1'860	1'741	-0.095	0.905919208	1
ENSBTAG00000010564	*MGC139109*	828	568	686	717	656	-0.129	0.882352261	1
ENSBTAG00000010565	*CYM*	0	1	1	0	1	Inf	0.993540919	1
ENSBTAG00000010566	*TMEM11*	671	743	720	581	858	0.562	0.508490705	1
ENSBTAG00000010568	*ZBED5*	2'446	1'034	1'656	2'118	1'194	-0.827	0.303095249	1
ENSBTAG00000010571	*PPAT*	1'648	764	1'155	1'427	882	-0.694	0.396928777	1
ENSBTAG00000010573	*FAM227A*	91	21	52	79	24	-1.700	0.330447843	1
ENSBTAG00000010576	*MAP2K3*	2'465	2'967	2'780	2'135	3'426	0.682	0.38423788	1
ENSBTAG00000010577	*BT.64565*	2'012	2'120	2'095	1'742	2'448	0.490	0.534947954	1
ENSBTAG00000010578	*IL17B*	7	43	28	6	50	3.034	0.220180886	1
ENSBTAG00000010579	*BT.19318*	524	261	378	454	301	-0.590	0.525562027	1
ENSBTAG00000010581	*MAGI1*	1'089	1'022	1'062	943	1'180	0.323	0.693469405	1
ENSBTAG00000010582	*BT.74262*	2'098	2'478	2'339	1'817	2'861	0.655	0.406052226	1
ENSBTAG00000010584	*AP2S1*	2'276	2'672	2'528	1'971	3'085	0.646	0.411028847	1
ENSBTAG00000010587	*SH3BGRL*	7'038	4'061	5'392	6'095	4'689	-0.378	0.624318287	1
ENSBTAG00000010590	*FAM118A*	1'385	753	1'034	1'199	869	-0.464	0.573765746	1
ENSBTAG00000010591	*ZNF365*	184	150	166	159	173	0.120	0.918225832	1
ENSBTAG00000010593	*SRP72*	3'594	2'485	2'991	3'112	2'869	-0.117	0.881550293	1
ENSBTAG00000010595	*WDR92*	872	629	741	755	726	-0.056	0.949448417	1
ENSBTAG00000010597	*GGCT*	476	429	454	412	495	0.265	0.769023291	1
ENSBTAG00000010598	*EIF4E2*	445	288	359	385	333	-0.213	0.822845329	1
ENSBTAG00000010599	*C2H2orf76*	478	280	369	414	323	-0.357	0.703602808	1
ENSBTAG00000010601	*DOLK*	697	803	765	604	927	0.619	0.4637557	1
ENSBTAG00000010602	*DDX56*	1'173	1'766	1'528	1'016	2'039	1.005	0.213309505	1
ENSBTAG00000010604	*PNO1*	1'075	740	893	931	854	-0.124	0.883708539	1
ENSBTAG00000010605	*BT.106472*	2	0	1	2	0		0.974934741	1
ENSBTAG00000010606	*PPP1R3B*	749	512	620	649	591	-0.134	0.879192628	1
ENSBTAG00000010609	*STK31*	73	38	54	63	44	-0.527	0.761669062	1
ENSBTAG00000010610	*DDIT4L*	1'882	1'073	1'434	1'630	1'239	-0.396	0.62415021	1
ENSBTAG00000010611	*OCIAD1*	8'681	3'610	5'843	7'518	4'168	-0.851	0.271894732	1
ENSBTAG00000010612	*TMED4*	5'881	5'941	5'977	5'093	6'860	0.430	0.57637061	1
ENSBTAG00000010613	*NUP188*	2'360	3'344	2'953	2'044	3'861	0.918	0.24274201	1
ENSBTAG00000010615	*ARL9*	72	4	33	62	5	-3.755	0.113420428	1
ENSBTAG00000010616	*MBD6*	2'417	2'994	2'775	2'093	3'457	0.724	0.356328369	1
ENSBTAG00000010617	*KRTCAP2*	3'099	1'913	2'446	2'684	2'209	-0.281	0.721565998	1
ENSBTAG00000010619	*PPP3R1*	1'689	1'417	1'549	1'463	1'636	0.162	0.839974587	1
ENSBTAG00000010620	*ATP6V1B1*	605	995	836	524	1'149	1.133	0.179840521	1
ENSBTAG00000010622	*TRIM46*	223	44	122	193	51	-1.926	0.124834837	1
ENSBTAG00000010623	*ANKRD53*	40	31	35	35	36	0.047	0.996958386	1
ENSBTAG00000010624	*DCTN2*	14'595	12'563	13'573	12'640	14'507	0.199	0.793827959	1
ENSBTAG00000010626	*SH3GLB2*	3'941	4'837	4'499	3'413	5'585	0.711	0.35945157	1

Ensembl gene ID	geneName	counts wildtype horn bud	counts polled horn bud	baseMean	baseMean wildtype horn bud	baseMean polled horn bud	log2FoldChange	pval	padj
ENSBTAG00000010627	*SF3B3*	9'829	9'477	9'728	8'512	10'943	0.362	0.635278653	1
ENSBTAG00000010630	*MGC165715*	588	333	447	509	385	-0.405	0.654897643	1
ENSBTAG00000010632	*FABP6*	5	2	3	4	2	-0.907	0.965043111	1
ENSBTAG00000010634	*BT.61452*	1'061	677	850	919	782	-0.233	0.781852278	1
ENSBTAG00000010635	*RAB3A*	697	224	431	604	259	-1.223	0.182335556	1
ENSBTAG00000010637	*BT.103824*	499	832	696	432	961	1.153	0.180193643	1
ENSBTAG00000010638	*DCST2*	84	75	80	73	87	0.252	0.865958285	1
ENSBTAG00000010639	*MAP2K7*	730	982	883	632	1'134	0.843	0.313590119	1
ENSBTAG00000010640	*protein_coding*	3	1	2	3	1	-1.170	0.985253077	1
ENSBTAG00000010641	*APH1B*	78	65	71	68	75	0.152	0.92692965	1
ENSBTAG00000010642	*ALKBH8*	791	657	722	685	759	0.147	0.862930994	1
ENSBTAG00000010644	*CHST11*	152	90	118	132	104	-0.341	0.787875017	1
ENSBTAG00000010647	*NTRK2*	879	1'623	1'318	761	1'874	1.300	0.112730352	1
ENSBTAG00000010649	*MEF2A*	8'339	4'240	6'059	7'222	4'896	-0.561	0.467362833	1
ENSBTAG00000010652	*PDE4C*	29	27	28	25	31	0.312	0.905723745	1
ENSBTAG00000010653	*FAM73B*	1'028	1'353	1'226	890	1'562	0.811	0.319897275	1
ENSBTAG00000010655	*DCST1*	3	0	1	3	0		0.936647693	1
ENSBTAG00000010657	*BAI2*	2'613	3'343	3'062	2'263	3'860	0.770	0.325159632	1
ENSBTAG00000010658	*PFKL*	3'025	5'463	4'464	2'620	6'308	1.268	0.105608133	1
ENSBTAG00000010659	*CUX1*	941	1'048	1'013	815	1'210	0.570	0.48906097	1
ENSBTAG00000010660	*CACNA1C*	1'658	1'105	1'356	1'436	1'276	-0.170	0.83423316	1
ENSBTAG00000010661	*DPYSL3*	7'077	6'308	6'706	6'129	7'284	0.249	0.745166708	1
ENSBTAG00000010662	*BT.48450*	2'607	3'367	3'073	2'258	3'888	0.784	0.31671727	1
ENSBTAG00000010663	*ADAM15*	2'454	3'181	2'899	2'125	3'673	0.789	0.314316334	1
ENSBTAG00000010664	*PRKCQ*	63	32	46	55	37	-0.562	0.764157681	1
ENSBTAG00000010665	*BT.54882*	40	15	26	35	17	-1.000	0.689043011	1
ENSBTAG00000010666	*KHNYN*	2'730	3'436	3'166	2'364	3'968	0.747	0.339594768	1
ENSBTAG00000010667	*PIK3IP1*	819	763	795	709	881	0.313	0.709987095	1
ENSBTAG00000010668	*GEMIN7*	353	283	316	306	327	0.096	0.922349564	1
ENSBTAG00000010670	*VSIG8*	493	153	302	427	177	-1.273	0.192226678	1
ENSBTAG00000010671	*GLI3*	1'256	1'122	1'192	1'088	1'296	0.252	0.756566494	1
ENSBTAG00000010672	*BT.55189*	1'408	1'146	1'271	1'219	1'323	0.118	0.884392659	1
ENSBTAG00000010673	*EFNA4*	945	1'546	1'302	818	1'785	1.125	0.168469854	1
ENSBTAG00000010674	*protein_coding*	1	0	0	1	0		1	1
ENSBTAG00000010676	*RBM44*	8	7	8	7	8	0.222	0.99532173	1
ENSBTAG00000010677	*LIMCH1*	1'995	922	1'396	1'728	1'065	-0.699	0.388192849	1
ENSBTAG00000010679	*LRRC31*	4	0	2	3	0		0.89545886	1
ENSBTAG00000010680	*protein_coding*	1	1	1	1	1	0.415	1	1
ENSBTAG00000010681	*NR1H3*	849	918	898	735	1'060	0.528	0.526051423	1
ENSBTAG00000010682	*DDR1*	7'365	9'371	8'599	6'378	10'821	0.763	0.321266962	1
ENSBTAG00000010689	*NRG2*	92	28	56	80	32	-1.301	0.436004647	1
ENSBTAG00000010691	*USP39*	2'121	1'696	1'898	1'837	1'958	0.092	0.907169777	1
ENSBTAG00000010692	*SND1*	7'016	8'878	8'164	6'076	10'251	0.755	0.326533948	1
ENSBTAG00000010694	*BICC1*	2'906	1'129	1'910	2'517	1'304	-0.949	0.235100056	1
ENSBTAG00000010696	*DVL1*	1'901	1'809	1'868	1'646	2'089	0.343	0.665116855	1
ENSBTAG00000010698	*VARS2*	1'513	1'652	1'609	1'310	1'908	0.542	0.498452274	1
ENSBTAG00000010701	*NACA*	16'295	8'339	11'870	14'112	9'629	-0.551	0.471573702	1
ENSBTAG00000010704	*BT.53375*	153	73	108	133	84	-0.653	0.611056312	1
ENSBTAG00000010709	*DLAT*	710	551	626	615	636	0.049	0.955691081	1
ENSBTAG00000010711	*protein_coding*	382	920	697	331	1'062	1.683	0.053366276	1
ENSBTAG00000010715	*MRPL37*	1'600	1'971	1'831	1'386	2'276	0.716	0.369094072	1
ENSBTAG00000010716	*FRAS1*	343	615	504	297	710	1.257	0.160402273	1
ENSBTAG00000010717	*BT.45850*	2'587	3'147	2'937	2'240	3'634	0.698	0.372997019	1
ENSBTAG00000010718	*RALGPS2*	320	521	439	277	602	1.118	0.219493457	1
ENSBTAG00000010719	*ANGPTL1*	10'241	7'216	8'601	8'869	8'332	-0.090	0.907161982	1
ENSBTAG00000010720	*MIB2*	3'448	4'188	3'911	2'986	4'836	0.696	0.371005845	1
ENSBTAG00000010721	*MCM3*	2'841	3'888	3'475	2'460	4'489	0.868	0.266975715	1
ENSBTAG00000010722	*processed_pseudogene*	275	628	482	238	725	1.606	0.077183975	1
ENSBTAG00000010723	*PFDN6*	1'224	951	1'079	1'060	1'098	0.051	0.950913433	1
ENSBTAG00000010724	*pseudogene*	2	45	27	2	52	4.907	0.083668923	1
ENSBTAG00000010726	*F8*	625	357	477	541	412	-0.393	0.661775712	1
ENSBTAG00000010727	*ATG4D*	797	1'250	1'067	690	1'443	1.064	0.198118088	1
ENSBTAG00000010728	*RAB44*	3	5	4	3	6	1.152	0.920866718	1
ENSBTAG00000010729	*KRI1*	959	1'012	1'000	831	1'169	0.493	0.550349487	1
ENSBTAG00000010730	*protein_coding*	1	0	0	1	0		1	1
ENSBTAG00000010731	*CDKN2D*	583	286	418	505	330	-0.612	0.503232314	1
ENSBTAG00000010732	*MMP23B*	4'775	3'954	4'350	4'135	4'566	0.143	0.853000301	1
ENSBTAG00000010734	*NOP16*	1'283	1'027	1'148	1'111	1'186	0.094	0.908628058	1
ENSBTAG00000010735	*HIGD2A*	1'064	1'163	1'132	921	1'343	0.543	0.506287179	1
ENSBTAG00000010736	*BBS5*	712	373	524	617	431	-0.518	0.558712148	1
ENSBTAG00000010737	*BT.32779*	4'278	3'602	3'932	3'705	4'159	0.167	0.829059342	1
ENSBTAG00000010739	*BLOC1S2*	875	503	669	758	581	-0.384	0.655443622	1

Ensembl gene ID	geneName	counts wildtype horn bud	counts polled horn bud	baseMean	baseMean wildtype horn bud	baseMean polled horn bud	log2FoldChange	pval	padj
ENSBTAG00000010740	*CLTB*	1'867	1'755	1'822	1'617	2'026	0.326	0.681701881	1
ENSBTAG00000010742	*PKD2L1*	182	118	147	158	136	-0.210	0.861033874	1
ENSBTAG00000010743	*FASTKD1*	1'010	416	678	875	480	-0.865	0.314599381	1
ENSBTAG00000010745	*THRA*	4'984	8'976	7'340	4'316	10'365	1.264	0.104024362	1
ENSBTAG00000010756	*ST7*	1'319	939	1'113	1'142	1'084	-0.075	0.928513515	1
ENSBTAG00000010758	*C19H17orf109*	198	89	137	171	103	-0.739	0.532987193	1
ENSBTAG00000010760	*BT.38709*	4'453	2'443	3'339	3'856	2'821	-0.451	0.563237556	1
ENSBTAG00000010763	*DUSP16*	301	303	305	261	350	0.425	0.659834118	1
ENSBTAG00000010765	*C1ORF131*	595	297	429	515	343	-0.587	0.519073957	1
ENSBTAG00000010766	*OIP5*	78	57	67	68	66	-0.037	0.992806173	1
ENSBTAG00000010772	*TPGS1*	554	637	608	480	736	0.616	0.476797689	1
ENSBTAG00000010773	*GNPAT*	3'699	2'996	3'331	3'203	3'459	0.111	0.886267386	1
ENSBTAG00000010774	*NUSAP1*	1'671	1'194	1'413	1'447	1'379	-0.070	0.932432409	1
ENSBTAG00000010775	*POGK*	4'670	2'651	3'553	4'044	3'061	-0.402	0.605993715	1
ENSBTAG00000010777	*CARS*	1'978	2'105	2'072	1'713	2'431	0.505	0.523317287	1
ENSBTAG00000010778	*NDUFAF1*	307	341	330	266	394	0.567	0.550747873	1
ENSBTAG00000010782	*KIAA1210*	4	1	2	3	1	-1.585	0.952247636	1
ENSBTAG00000010784	*ABT1*	872	658	757	755	760	0.009	0.992799433	1
ENSBTAG00000010785	*FAS*	277	197	234	240	227	-0.077	0.945217737	1
ENSBTAG00000010786	*TACC2*	3'194	2'291	2'706	2'766	2'645	-0.064	0.935635399	1
ENSBTAG00000010787	*RFC3*	1'029	816	917	891	942	0.080	0.923392193	1
ENSBTAG00000010788	*PPM1E*	120	10	58	104	12	-3.170	0.071944303	1
ENSBTAG00000010789	*ANKRD54*	959	788	870	831	910	0.132	0.874982974	1
ENSBTAG00000010790	*EIF3L*	7'226	8'174	7'848	6'258	9'439	0.593	0.440000524	1
ENSBTAG00000010791	*BT.106425*	1	0	0	1	0		1	1
ENSBTAG00000010792	*SEH1L*	3'395	2'397	2'854	2'940	2'768	-0.087	0.912305807	1
ENSBTAG00000010793	*CCDC80*	43'920	80'177	65'308	38'036	92'580	1.283	0.095362436	1
ENSBTAG00000010795	*ATG14*	1'725	1'024	1'338	1'494	1'182	-0.337	0.677453966	1
ENSBTAG00000010796	*SPADH1*	2	1	1	2	1	-0.585	1	1
ENSBTAG00000010798	*SLC23A1*	186	51	110	161	59	-1.452	0.257615453	1
ENSBTAG00000010799	*MYL6*	31'226	19'907	25'015	27'043	22'987	-0.234	0.758524792	1
ENSBTAG00000010801	*CMBL*	960	290	583	831	335	-1.312	0.13665299	1
ENSBTAG00000010802	*KCNK7*	183	160	172	158	185	0.221	0.844108811	1
ENSBTAG00000010803	*CHML*	107	47	73	93	54	-0.772	0.603737414	1
ENSBTAG00000010805	*POMT1*	3'365	2'740	3'039	2'914	3'164	0.119	0.878816204	1
ENSBTAG00000010809	*C19H17orf28*	1'268	1'024	1'140	1'098	1'182	0.107	0.896283456	1
ENSBTAG00000010810	*UCK1*	1'945	2'058	2'030	1'684	2'376	0.497	0.53048326	1
ENSBTAG00000010812	*P2RX4*	1'030	741	874	892	856	-0.060	0.944701809	1
ENSBTAG00000010813	*protein_coding*	293	230	260	254	266	0.066	0.950610329	1
ENSBTAG00000010814	*BT.52760*	1	0	0	1	0		1	1
ENSBTAG00000010815	*CAMKK2*	793	539	655	687	622	-0.142	0.870773839	1
ENSBTAG00000010818	*AEBP2*	3'244	1'397	2'211	2'809	1'613	-0.800	0.312719578	1
ENSBTAG00000010819	*HMBOX1*	186	214	204	161	247	0.617	0.558250256	1
ENSBTAG00000010821	*ACER2*	62	19	38	54	22	-1.291	0.522519899	1
ENSBTAG00000010822	*BT.89131*	19	2	9	16	2	-2.833	0.582174212	1
ENSBTAG00000010826	*C11ORF30*	1'151	782	950	997	903	-0.143	0.86497374	1
ENSBTAG00000010829	*EBF2*	58	27	41	50	31	-0.688	0.727084192	1
ENSBTAG00000010830	*LRP1*	39'938	87'739	67'950	34'587	101'312	1.550	0.045307471	1
ENSBTAG00000010832	*JAGN1*	1'215	1'283	1'267	1'052	1'481	0.494	0.542835232	1
ENSBTAG00000010833	*IL17RE*	416	695	581	360	803	1.155	0.18800775	1
ENSBTAG00000010835	*CRELD1*	899	665	773	779	768	-0.020	0.983612111	1
ENSBTAG00000010836	*PRRT3*	159	59	103	138	68	-1.015	0.436305978	1
ENSBTAG00000010837	*HTR4*	2	2	2	2	2	0.415	1	1
ENSBTAG00000010838	*TMEM181*	1'159	699	905	1'004	807	-0.314	0.706540878	1
ENSBTAG00000010841	*FMO5*	1'691	968	1'291	1'464	1'118	-0.390	0.631459632	1
ENSBTAG00000010843	*PGRMC2*	4'459	2'128	3'159	3'862	2'457	-0.652	0.404569081	1
ENSBTAG00000010846	*CNNM3*	1'785	2'082	1'975	1'546	2'404	0.637	0.422126381	1
ENSBTAG00000010847	*FOXN4*	10	0	4	9	0		0.653360153	1
ENSBTAG00000010849	*ANKRD23*	449	242	334	389	279	-0.477	0.616432182	1
ENSBTAG00000010850	*SERTAD4*	6'452	3'126	4'599	5'588	3'610	-0.630	0.416343701	1
ENSBTAG00000010851	*SEPHS2*	989	933	967	856	1'077	0.331	0.688901365	1
ENSBTAG00000010852	*BTN*	7	18	13	6	21	1.778	0.63434103	1
ENSBTAG00000010856	*SLC8A2*	229	212	222	198	245	0.304	0.77030767	1
ENSBTAG00000010858	*ABCC11*	22	12	16	19	14	-0.459	0.903495485	1
ENSBTAG00000010859	*HYLS1*	395	261	322	342	301	-0.183	0.851303609	1
ENSBTAG00000010860	*DRD2*	19	0	8	16	0		0.389538827	1
ENSBTAG00000010863	*DCP1A*	2'895	2'116	2'475	2'507	2'443	-0.037	0.963464368	1
ENSBTAG00000010865	*CACNG2*	11	0	5	10	0		0.617662983	1
ENSBTAG00000010866	*BMPER*	1'476	1'283	1'380	1'278	1'481	0.213	0.791735315	1
ENSBTAG00000010867	*LONP2*	3'193	2'226	2'668	2'765	2'570	-0.105	0.894014882	1
ENSBTAG00000010868	*BT.37798*	3'054	1'631	2'264	2'645	1'883	-0.490	0.535236703	1
ENSBTAG00000010871	*BT.60688*	5'357	3'068	4'091	4'639	3'543	-0.389	0.616324128	1

Ensembl gene ID	geneName	counts wildtype horn bud	counts polled horn bud	baseMean	baseMean wildtype horn bud	baseMean polled horn bud	log2FoldChange	pval	padj
ENSBTAG00000010873	*BHLHE23*	1	1	1	1	1	0.415	1	1
ENSBTAG00000010875	*MSX1*	314	90	188	272	104	-1.388	0.202931274	1
ENSBTAG00000010877	*ARMC12*	79	8	39	68	9	-2.889	0.167492805	1
ENSBTAG00000010878	*BT.27409*	72	2	32	62	2	-4.755	0.066927369	1
ENSBTAG00000010881	*AKAP14*	14	4	8	12	5	-1.392	0.803716462	1
ENSBTAG00000010882	*BT.88457*	80	94	89	69	109	0.648	0.636832044	1
ENSBTAG00000010884	*NKAP*	367	263	311	318	304	-0.066	0.949725162	1
ENSBTAG00000010885	*TSPYL1*	3'399	2'806	3'092	2'944	3'240	0.138	0.858712602	1
ENSBTAG00000010888	*MGC151567*	1'069	1'386	1'263	926	1'600	0.790	0.331922357	1
ENSBTAG00000010890	*PRMT5*	2'707	2'666	2'711	2'344	3'078	0.393	0.615493022	1
ENSBTAG00000010896	*BT.106450*	13	29	22	11	33	1.573	0.557224015	1
ENSBTAG00000010897	*RESP18*	54	7	27	47	8	-2.532	0.30637419	1
ENSBTAG00000010898	*SLC30A6*	801	471	619	694	544	-0.351	0.685971895	1
ENSBTAG00000010899	*BT.52974*	12'992	13'527	13'436	11'251	15'620	0.473	0.535134998	1
ENSBTAG00000010904	*PA1*	1'257	1'267	1'276	1'089	1'463	0.426	0.598722113	1
ENSBTAG00000010906	*RPS6KA5*	1'739	906	1'276	1'506	1'046	-0.526	0.517966937	1
ENSBTAG00000010907	*PPP1R1A*	502	114	283	435	132	-1.724	0.084725988	1
ENSBTAG00000010909	*SLC30A1*	205	114	155	178	132	-0.432	0.707446576	1
ENSBTAG00000010910	*MYEOV2*	3'871	1'953	2'804	3'352	2'255	-0.572	0.466056451	1
ENSBTAG00000010913	*SRXN1*	72	32	50	62	37	-0.755	0.67074827	1
ENSBTAG00000010915	*CRTC3*	558	693	642	483	800	0.728	0.398556131	1
ENSBTAG00000010916	*LHFPL4*	252	34	129	218	39	-2.475	0.049324238	1
ENSBTAG00000010919	*BT.44956*	6'642	3'860	5'105	5'752	4'457	-0.368	0.634117515	1
ENSBTAG00000010922	*TRAPPC2*	707	324	493	612	374	-0.711	0.426033579	1
ENSBTAG00000010923	*RAB9A*	2'005	1'318	1'629	1'736	1'522	-0.190	0.81307297	1
ENSBTAG00000010924	*BT.24718*	1'308	644	938	1'133	744	-0.607	0.465401449	1
ENSBTAG00000010927	*CBFA2T3*	709	864	806	614	998	0.700	0.405450885	1
ENSBTAG00000010928	*CSRNP2*	572	469	518	495	542	0.129	0.885363263	1
ENSBTAG00000010931	*BT.90251*	1'072	524	767	928	605	-0.618	0.465966031	1
ENSBTAG00000010932	*DNAJC12*	458	82	246	397	95	-2.067	0.047308976	1
ENSBTAG00000010934	*RAE1*	2'009	1'449	1'707	1'740	1'673	-0.056	0.945069837	1
ENSBTAG00000010935	*EML4*	2'869	2'209	2'518	2'485	2'551	0.038	0.961343159	1
ENSBTAG00000010937	*BT.27908*	607	412	501	526	476	-0.144	0.873685801	1
ENSBTAG00000010938	*C2H1orf64*	29	1	13	25	1	-4.443	0.318957573	1
ENSBTAG00000010939	*MGC165780*	29	4	15	25	5	-2.443	0.497961729	1
ENSBTAG00000010940	*HSPB7*	7	54	34	6	62	3.363	0.136104444	1
ENSBTAG00000010943	*SLC22A23*	310	380	354	268	439	0.709	0.449966697	1
ENSBTAG00000010944	*LRRC49*	471	296	375	408	342	-0.255	0.785863685	1
ENSBTAG00000010945	*SEC24B*	3'921	1'777	2'724	3'396	2'052	-0.727	0.355457412	1
ENSBTAG00000010948	*CLIC2*	370	167	257	320	193	-0.733	0.465014919	1
ENSBTAG00000010949	*PROSER1*	812	527	656	703	609	-0.209	0.809788759	1
ENSBTAG00000010951	*NHLRC3*	1'061	649	834	919	749	-0.294	0.726847057	1
ENSBTAG00000010952	*BT.106282*	3	1	2	3	1	-1.170	0.985253077	1
ENSBTAG00000010954	*ART3*	43	5	22	37	6	-2.689	0.349070496	1
ENSBTAG00000010955	*BT.52474*	919	653	775	796	754	-0.078	0.928569668	1
ENSBTAG00000010956	*SCARB2*	10'919	6'167	8'289	9'456	7'121	-0.409	0.594180911	1
ENSBTAG00000010957	*LHPP*	782	1'052	946	677	1'215	0.843	0.310769266	1
ENSBTAG00000010958	*C12ORF51*	64	45	54	55	52	-0.093	0.96895552	1
ENSBTAG00000010959	*HINT1*	3'787	2'299	2'967	3'280	2'655	-0.305	0.696928869	1
ENSBTAG00000010961	*BT.51888*	165	112	136	143	129	-0.144	0.90964025	1
ENSBTAG00000010963	*BT.26527*	598	117	326	518	135	-1.939	0.047696778	1
ENSBTAG00000010964	*STARD5*	333	221	272	288	255	-0.176	0.861809542	1
ENSBTAG00000010967	*C10ORF107*	191	134	160	165	155	-0.096	0.938236527	1
ENSBTAG00000010968	*TMED1*	2'125	1'906	2'021	1'840	2'201	0.258	0.743990153	1
ENSBTAG00000010970	*protein_coding*	1	5	3	1	6	2.737	0.836148221	1
ENSBTAG00000010971	*MGC142792*	1'572	875	1'186	1'361	1'010	-0.430	0.598492721	1
ENSBTAG00000010976	*KANK1*	2'814	1'833	2'277	2'437	2'117	-0.203	0.797243045	1
ENSBTAG00000010978	*PROX1*	17	23	21	15	27	0.851	0.766760787	1
ENSBTAG00000010979	*CDKL3*	5	1	3	4	1	-1.907	0.9188325	1
ENSBTAG00000010980	*ANKMY2*	1'793	881	1'285	1'553	1'017	-0.610	0.452991563	1
ENSBTAG00000010981	*CIB2*	448	265	347	388	306	-0.342	0.717911697	1
ENSBTAG00000010982	*UBE2B*	3'358	2'086	2'658	2'908	2'409	-0.272	0.729507893	1
ENSBTAG00000010984	*CDKN2AIPNL*	2'441	1'566	1'961	2'114	1'808	-0.225	0.777138782	1
ENSBTAG00000010986	*KCNQ1*	108	88	98	94	102	0.120	0.934674312	1
ENSBTAG00000010987	*BT.68839*	800	199	461	693	230	-1.592	0.08201165	1
ENSBTAG00000010988	*BT.86870*	5'437	4'872	5'167	4'709	5'626	0.257	0.738658321	1
ENSBTAG00000010989	*PIK3R1*	2'143	2'113	2'148	1'856	2'440	0.395	0.61692555	1
ENSBTAG00000010990	*LHX2*	1'402	1'439	1'438	1'214	1'662	0.453	0.573806946	1
ENSBTAG00000010991	*TTR*	1'058	756	895	916	873	-0.070	0.935164742	1
ENSBTAG00000010992	*CTSH*	1'463	668	1'019	1'267	771	-0.716	0.386593636	1
ENSBTAG00000010993	*ZNF839*	540	462	501	468	533	0.190	0.831370989	1
ENSBTAG00000010994	*CTSF*	7'134	4'815	5'869	6'178	5'560	-0.152	0.844045956	1

Ensembl gene ID	geneName	counts wildtype horn bud	counts polled horn bud	baseMean	baseMean wildtype horn bud	baseMean polled horn bud	log2FoldChange	pval	padj
ENSBTAG00000010995	*CINP*	1'606	1'161	1'366	1'391	1'341	-0.053	0.949211586	1
ENSBTAG00000010998	*FLIP*	703	429	552	609	495	-0.298	0.735892735	1
ENSBTAG00000010999	*IL17RD*	100	102	102	87	118	0.444	0.735259452	1
ENSBTAG00000011000	*HDAC10*	1'700	1'885	1'824	1'472	2'177	0.564	0.478424757	1
ENSBTAG00000011001	*BT.45313*	2'393	1'532	1'921	2'072	1'769	-0.228	0.774428298	1
ENSBTAG00000011002	*CCDC136*	141	98	118	122	113	-0.110	0.937097266	1
ENSBTAG00000011003	*BT.62405*	3	1	2	3	1	-1.170	0.985253077	1
ENSBTAG00000011007	*TTYH2*	1'607	3'362	2'637	1'392	3'882	1.480	0.063623724	1
ENSBTAG00000011010	*PRND*	1'149	443	753	995	512	-0.960	0.259613137	1
ENSBTAG00000011011	*SSH2*	236	166	198	204	192	-0.093	0.936136215	1
ENSBTAG00000011012	*protein_coding*	0	2	1	0	2	Inf	0.939077559	1
ENSBTAG00000011014	*CHST3*	437	691	588	378	798	1.076	0.218838979	1
ENSBTAG00000011017	*ABI2*	657	664	668	569	767	0.430	0.615356419	1
ENSBTAG00000011019	*protein_coding*	2	1	1	2	1	-0.585	1	1
ENSBTAG00000011021	*CES1*	1	2	2	1	2	1.415	0.981979269	1
ENSBTAG00000011022	*ARPP19*	3'996	2'122	2'955	3'461	2'450	-0.498	0.524765244	1
ENSBTAG00000011024	*TXNDC12*	3'330	2'346	2'796	2'884	2'709	-0.090	0.909184556	1
ENSBTAG00000011025	*BT.92009*	3'473	1'586	2'420	3'008	1'831	-0.716	0.364612855	1
ENSBTAG00000011027	*BT.69126*	3'203	1'745	2'394	2'774	2'015	-0.461	0.558614382	1
ENSBTAG00000011028	*BT.21593*	1'169	1'020	1'095	1'012	1'178	0.218	0.789783975	1
ENSBTAG00000011032	*MCPH1*	732	667	702	634	770	0.281	0.741758257	1
ENSBTAG00000011034	*ANGPT2*	883	412	620	765	476	-0.685	0.429633921	1
ENSBTAG00000011036	*BT.105859*	9	5	7	8	6	-0.433	0.969858938	1
ENSBTAG00000011037	*RBPMS2*	112	84	97	97	97	0.000	1	1
ENSBTAG00000011038	*MC2R*	4	1	2	3	1	-1.585	0.952247636	1
ENSBTAG00000011041	*ZFYVE1*	1'962	1'258	1'576	1'699	1'453	-0.226	0.778725767	1
ENSBTAG00000011042	*TMEM129*	1'358	1'508	1'459	1'176	1'741	0.566	0.481756652	1
ENSBTAG00000011043	*RAC2*	579	158	342	501	182	-1.459	0.127727535	1
ENSBTAG00000011044	*TACC3*	1'720	1'888	1'835	1'490	2'180	0.549	0.489692562	1
ENSBTAG00000011045	*MRPS36*	716	408	546	620	471	-0.396	0.652931588	1
ENSBTAG00000011046	*CDK7*	586	278	414	507	321	-0.661	0.470735181	1
ENSBTAG00000011048	*C9ORF68*	50	6	25	43	7	-2.644	0.312062188	1
ENSBTAG00000011050	*PPAPDC2*	78	42	58	68	48	-0.478	0.77592708	1
ENSBTAG00000011051	*pseudogene*	61	161	119	53	186	1.815	0.147783932	1
ENSBTAG00000011052	*protein_coding*	688	288	464	596	333	-0.841	0.350705029	1
ENSBTAG00000011056	*IDS*	719	729	732	623	842	0.435	0.608048832	1
ENSBTAG00000011057	*GGNBP1*	1	2	2	1	2	1.415	0.981979269	1
ENSBTAG00000011059	*CDKN2C*	872	629	741	755	726	-0.056	0.949448417	1
ENSBTAG00000011062	*TDO2*	2	0	1	2	0		0.974934741	1
ENSBTAG00000011063	*BT.28045*	292	263	278	253	304	0.264	0.789281554	1
ENSBTAG00000011064	*ADCK5*	2'404	1'913	2'145	2'082	2'209	0.085	0.913659298	1
ENSBTAG00000011067	*IL17RA*	649	1'123	929	562	1'297	1.206	0.150195479	1
ENSBTAG00000011068	*MON2*	7'636	2'919	4'992	6'613	3'371	-0.972	0.211264222	1
ENSBTAG00000011070	*C5H22orf28*	2'368	2'592	2'522	2'051	2'993	0.545	0.487454851	1
ENSBTAG00000011071	*BPIFC*	47	19	31	41	22	-0.892	0.691980358	1
ENSBTAG00000011072	*ADK*	2'215	1'162	1'630	1'918	1'342	-0.516	0.5201124	1
ENSBTAG00000011074	*BRD2*	11'715	10'584	11'183	10'145	12'221	0.269	0.724627078	1
ENSBTAG00000011075	*PROSC*	1'519	1'474	1'509	1'315	1'702	0.372	0.643136034	1
ENSBTAG00000011076	*BT.41409*	292	237	263	253	274	0.114	0.911426156	1
ENSBTAG00000011079	*MGC137708*	1'576	2'057	1'870	1'365	2'375	0.799	0.316044986	1
ENSBTAG00000011080	*BT.106473*	10	8	9	9	9	0.093	1	1
ENSBTAG00000011081	*KBTBD8*	109	66	85	94	76	-0.309	0.830974705	1
ENSBTAG00000011083	*POLD1*	1'056	1'740	1'462	915	2'009	1.136	0.161852483	1
ENSBTAG00000011087	*ARID2*	1'674	1'294	1'472	1'450	1'494	0.044	0.956947257	1
ENSBTAG00000011088	*SLC6A6*	125	98	111	108	113	0.064	0.966533104	1
ENSBTAG00000011091	*SGIP1*	60	3	28	52	3	-3.907	0.141363976	1
ENSBTAG00000011094	*processed_pseudogene*	1'478	1'271	1'374	1'280	1'468	0.197	0.806663783	1
ENSBTAG00000011095	*FAR2*	646	152	367	559	176	-1.672	0.078637098	1
ENSBTAG00000011096	*ERGIC2*	3'441	1'957	2'620	2'980	2'260	-0.399	0.6115584	1
ENSBTAG00000011097	*TCTEX1D1*	9	1	4	8	1	-2.755	0.785977687	1
ENSBTAG00000011098	*TTLL8*	1	0	0	1	0		1	1
ENSBTAG00000011100	*BT.49573*	3'039	1'949	2'441	2'632	2'251	-0.226	0.774730578	1
ENSBTAG00000011101	*EML6*	176	82	124	152	95	-0.687	0.575292491	1
ENSBTAG00000011102	*TPCN1*	1'128	966	1'046	977	1'115	0.191	0.815946164	1
ENSBTAG00000011103	*SLC24A6*	940	1'017	994	814	1'174	0.529	0.521894623	1
ENSBTAG00000011104	*RTN4*	16'142	7'541	11'343	13'979	8'708	-0.683	0.373327245	1
ENSBTAG00000011105	*SLC38A2*	15'002	9'231	11'826	12'992	10'659	-0.286	0.709127515	1
ENSBTAG00000011106	*PACRG*	28	1	13	24	1	-4.392	0.334236041	1
ENSBTAG00000011108	*USP54*	1'815	958	1'339	1'572	1'106	-0.507	0.531799914	1
ENSBTAG00000011110	*ACOT12*	87	16	47	75	18	-2.028	0.271033221	1
ENSBTAG00000011111	*BT.42676*	1'696	1'782	1'763	1'469	2'058	0.486	0.541386002	1
ENSBTAG00000011112	*BT.88525*	77	159	125	67	184	1.461	0.232518683	1

Ensembl gene ID	geneName	counts wildtype horn bud	counts polled horn bud	baseMean	baseMean wildtype horn bud	baseMean polled horn bud	log2FoldChange	pval	padj
ENSBTAG00000011116	*PAQR9*	2	0	1	2	0		0.974934741	1
ENSBTAG00000011120	*GBX1*	3	3	3	3	3	0.415	1	1
ENSBTAG00000011121	*CLCN4*	303	118	199	262	136	-0.945	0.375059653	1
ENSBTAG00000011124	*LHX3*	10	20	16	9	23	1.415	0.671359693	1
ENSBTAG00000011125	*MYO9B*	6'822	10'576	9'060	5'908	12'212	1.048	0.174888061	1
ENSBTAG00000011126	*STK38*	4'912	3'512	4'155	4'254	4'055	-0.069	0.929967005	1
ENSBTAG00000011127	*NUP205*	2'487	2'177	2'334	2'154	2'514	0.223	0.776566673	1
ENSBTAG00000011129	*QSOX2*	1'213	1'249	1'246	1'050	1'442	0.457	0.573267724	1
ENSBTAG00000011131	*NMUR2*	3	2	2	3	2	-0.170	1	1
ENSBTAG00000011132	*CCDC83*	4	0	2	3	0		0.89545886	1
ENSBTAG00000011133	*AP1S3*	327	176	243	283	203	-0.479	0.637873515	1
ENSBTAG00000011134	*USE1*	1'749	1'137	1'414	1'515	1'313	-0.206	0.79912303	1
ENSBTAG00000011135	*OCEL1*	394	397	400	341	458	0.426	0.642825646	1
ENSBTAG00000011136	*NR2F6*	1'014	919	970	878	1'061	0.273	0.741087446	1
ENSBTAG00000011137	*MORN4*	322	327	328	279	378	0.437	0.645678742	1
ENSBTAG00000011138	*CCDC58*	605	499	550	524	576	0.137	0.876647139	1
ENSBTAG00000011139	*BCHE*	2'256	4'552	3'605	1'954	5'256	1.428	0.070721571	1
ENSBTAG00000011140	*FAM162A*	1'662	1'170	1'395	1'439	1'351	-0.091	0.911258869	1
ENSBTAG00000011143	*KPNA1*	1'260	1'278	1'283	1'091	1'476	0.436	0.5908811	1
ENSBTAG00000011145	*NDUFA4*	5'967	4'378	5'111	5'168	5'055	-0.032	0.968161204	1
ENSBTAG00000011146	*BT.38707*	5'523	2'408	3'782	4'783	2'781	-0.783	0.315733733	1
ENSBTAG00000011147	*RNF13*	4'295	2'276	3'174	3'720	2'628	-0.501	0.521412437	1
ENSBTAG00000011149	*SIGIRR*	703	612	658	609	707	0.215	0.802485099	1
ENSBTAG00000011150	*PFN2*	15'182	6'494	10'323	13'148	7'499	-0.810	0.292039918	1
ENSBTAG00000011153	*MGAT4C*	134	25	72	116	29	-2.007	0.187127185	1
ENSBTAG00000011154	*SDC1*	5'654	6'011	5'919	4'897	6'941	0.503	0.513180379	1
ENSBTAG00000011155	*PUM2*	2'317	2'051	2'187	2'007	2'368	0.239	0.761478669	1
ENSBTAG00000011156	*BT.36109*	500	115	283	433	133	-1.705	0.087991571	1
ENSBTAG00000011161	*processed_pseudogene*	758	890	842	656	1'028	0.647	0.440269096	1
ENSBTAG00000011162	*ZKSCAN2*	228	215	223	197	248	0.330	0.750164879	1
ENSBTAG00000011163	*CYTL1*	1	0	0	1	0		1	1
ENSBTAG00000011164	*CES5A*	29	25	27	25	29	0.201	0.947508293	1
ENSBTAG00000011171	*PIEZO2*	7'544	9'164	8'557	6'533	10'582	0.696	0.365091298	1
ENSBTAG00000011172	*ALMS1*	2'832	1'304	1'979	2'453	1'506	-0.704	0.376303043	1
ENSBTAG00000011173	*FAM189A2*	184	150	166	159	173	0.120	0.918225832	1
ENSBTAG00000011176	*BT.49047*	968	689	817	838	796	-0.075	0.930481559	1
ENSBTAG00000011179	*PDCD2*	753	504	617	652	582	-0.164	0.851466263	1
ENSBTAG00000011180	*ACTR8*	1'188	777	963	1'029	897	-0.198	0.812792006	1
ENSBTAG00000011182	*PDLIM1*	1'132	1'319	1'252	980	1'523	0.636	0.434231462	1
ENSBTAG00000011184	*BT.87494*	29'910	22'876	26'159	25'903	26'415	0.028	0.969730841	1
ENSBTAG00000011185	*BT.58576*	269	238	254	233	275	0.238	0.813584586	1
ENSBTAG00000011186	*BEST1*	124	80	100	107	92	-0.217	0.875543744	1
ENSBTAG00000011187	*FAM13A*	2'348	2'026	2'186	2'033	2'339	0.202	0.797352438	1
ENSBTAG00000011189	*TJAP1*	1'916	2'159	2'076	1'659	2'493	0.587	0.458108572	1
ENSBTAG00000011192	*GPR87*	495	572	545	429	660	0.624	0.477662902	1
ENSBTAG00000011193	*C1QC*	1'006	801	898	871	925	0.086	0.917951202	1
ENSBTAG00000011195	*BT.46987*	24	17	20	21	20	-0.082	0.998309887	1
ENSBTAG00000011196	*C1QB*	1'334	862	1'075	1'155	995	-0.215	0.794617903	1
ENSBTAG00000011197	*BT.25055*	252	126	182	218	145	-0.585	0.592261014	1
ENSBTAG00000011198	*PPP1CC*	9'386	5'680	7'344	8'129	6'559	-0.310	0.687290721	1
ENSBTAG00000011200	*ZNF644*	5'707	2'735	4'050	4'942	3'158	-0.646	0.40606339	1
ENSBTAG00000011202	*C8H9ORF89*	2'136	1'196	1'615	1'850	1'381	-0.422	0.599073033	1
ENSBTAG00000011203	*RP1*	6	0	3	5	0		0.81115604	1
ENSBTAG00000011204	*NINJ1*	600	524	562	520	605	0.220	0.802145893	1
ENSBTAG00000011205	*BT.42457*	64	29	44	55	33	-0.727	0.698783445	1
ENSBTAG00000011206	*GALNT1*	3'274	1'308	2'173	2'835	1'510	-0.909	0.252777087	1
ENSBTAG00000011207	*CNN1*	120	226	182	104	261	1.328	0.224505245	1
ENSBTAG00000011209	*SYT11*	3'492	1'061	2'125	3'024	1'225	-1.304	0.103649317	1
ENSBTAG00000011212	*EIF3K*	7'290	8'471	8'047	6'313	9'781	0.632	0.410786693	1
ENSBTAG00000011213	*protein_coding*	78	94	88	68	109	0.684	0.618998413	1
ENSBTAG00000011214	*THAP3*	1'976	1'449	1'692	1'711	1'673	-0.032	0.969017097	1
ENSBTAG00000011215	*ACTN4*	12'937	18'039	16'017	11'204	20'830	0.895	0.243299726	1
ENSBTAG00000011216	*protein_coding*	0	2	1	0	2	Inf	0.939077559	1
ENSBTAG00000011217	*DNAJC11*	1'593	2'111	1'909	1'380	2'438	0.821	0.302690053	1
ENSBTAG00000011224	*CITED2*	2'813	967	1'776	2'436	1'117	-1.125	0.161698948	1
ENSBTAG00000011225	*TMX1*	4'286	2'897	3'528	3'712	3'345	-0.150	0.847819436	1
ENSBTAG00000011226	*SLC4A2*	5'440	7'177	6'499	4'711	8'287	0.815	0.291201287	1
ENSBTAG00000011227	*BT.30924*	275	49	147	238	57	-2.074	0.081294565	1
ENSBTAG00000011228	*FASTK*	3'370	3'919	3'722	2'919	4'525	0.633	0.415865407	1
ENSBTAG00000011229	*TMUB1*	487	539	522	422	622	0.561	0.524825393	1
ENSBTAG00000011234	*FOXO3*	418	483	460	362	558	0.624	0.487863378	1
ENSBTAG00000011236	*STX11*	52	22	35	45	25	-0.826	0.696065225	1

Ensembl gene ID	geneName	counts wildtype horn bud	counts polled horn bud	baseMean	baseMean wildtype horn bud	baseMean polled horn bud	log2FoldChange	pval	padj
ENSBTAG00000011237	*PKHD1*	3	1	2	3	1	-1.170	0.985253077	1
ENSBTAG00000011238	*CD48*	172	44	100	149	51	-1.552	0.243042022	1
ENSBTAG00000011239	*SEC11A*	3'625	2'633	3'090	3'139	3'040	-0.046	0.953829502	1
ENSBTAG00000011241	*HPDL*	21	21	21	18	24	0.415	0.892551358	1
ENSBTAG00000011242	*MUTYH*	1'002	858	929	868	991	0.191	0.817887564	1
ENSBTAG00000011243	*TOE1*	1'212	1'249	1'246	1'050	1'442	0.458	0.572281835	1
ENSBTAG00000011244	*TESK2*	403	355	379	349	410	0.232	0.803157974	1
ENSBTAG00000011245	*PLXNC1*	494	280	376	428	323	-0.404	0.664932523	1
ENSBTAG00000011247	*ZC3HC1*	2'463	2'327	2'410	2'133	2'687	0.333	0.671443581	1
ENSBTAG00000011248	*C11ORF61*	2'289	1'090	1'620	1'982	1'259	-0.655	0.414216644	1
ENSBTAG00000011249	*BT.67104*	801	798	808	694	921	0.410	0.625768266	1
ENSBTAG00000011250	*AFG3L2*	3'824	3'162	3'481	3'312	3'651	0.141	0.855861617	1
ENSBTAG00000011252	*RAD50*	3'254	1'300	2'160	2'818	1'501	-0.909	0.252884402	1
ENSBTAG00000011256	*MYO1B*	12'778	6'244	9'138	11'066	7'210	-0.618	0.421013162	1
ENSBTAG00000011257	*ASAH1*	3'709	2'612	3'114	3'212	3'016	-0.091	0.908283951	1
ENSBTAG00000011258	*ACOT9*	1'479	759	1'079	1'281	876	-0.547	0.505731393	1
ENSBTAG00000011262	*ZNF548*	260	227	244	225	262	0.219	0.830292805	1
ENSBTAG00000011263	*EIF6*	2'661	2'544	2'621	2'304	2'938	0.350	0.654767756	1
ENSBTAG00000011266	*ZBTB16*	38	89	68	33	103	1.643	0.285177426	1
ENSBTAG00000011267	*CLRN1*	0	2	1	0	2	Inf	0.939077559	1
ENSBTAG00000011268	*TMEM64*	343	257	297	297	297	-0.001	1	1
ENSBTAG00000011271	*SNX31*	7	1	4	6	1	-2.392	0.851750348	1
ENSBTAG00000011273	*LRRC23*	25	2	12	22	2	-3.229	0.459170232	1
ENSBTAG00000011277	*FAM133A*	25	5	14	22	6	-1.907	0.61211339	1
ENSBTAG00000011278	*XYLB*	112	101	107	97	117	0.266	0.839702128	1
ENSBTAG00000011280	*TBKBP1*	941	1'028	1'001	815	1'187	0.543	0.510761198	1
ENSBTAG00000011284	*SLC39A1*	5'716	6'265	6'092	4'950	7'234	0.547	0.47710663	1
ENSBTAG00000011285	*TRMT1L*	2'166	795	1'397	1'876	918	-1.031	0.204772966	1
ENSBTAG00000011287	*ATXN7*	200	230	219	173	266	0.617	0.551618256	1
ENSBTAG00000011291	*DLG3*	2'819	1'586	2'136	2'441	1'831	-0.415	0.600426379	1
ENSBTAG00000011292	*LZTR1*	2'193	2'363	2'314	1'899	2'729	0.523	0.50692801	1
ENSBTAG00000011298	*EYA1*	144	20	74	125	23	-2.433	0.112679313	1
ENSBTAG00000011299	*pseudogene*	22	25	24	19	29	0.599	0.823092306	1
ENSBTAG00000011300	*RHAG*	19	3	10	16	3	-2.248	0.640043503	1
ENSBTAG00000011304	*XRN2*	4'078	3'702	3'903	3'532	4'275	0.275	0.72191481	1
ENSBTAG00000011305	*BT.28359*	4	2	3	3	2	-0.585	0.996239345	1
ENSBTAG00000011307	*DNM1*	6'085	5'151	5'609	5'270	5'948	0.175	0.820041672	1
ENSBTAG00000011311	*CCDC12*	863	820	847	747	947	0.341	0.683156195	1
ENSBTAG00000011313	*BT.102649*	1'240	826	1'014	1'074	954	-0.171	0.837024941	1
ENSBTAG00000011314	*PTP*	1	0	0	1	0		1	1
ENSBTAG00000011315	*GJA10*	14	4	8	12	5	-1.392	0.803716462	1
ENSBTAG00000011316	*ZC3H12A*	233	227	232	202	262	0.377	0.712869569	1
ENSBTAG00000011317	*BT.52458*	8'454	7'557	8'024	7'321	8'726	0.253	0.740539068	1
ENSBTAG00000011319	*SLTM*	8'257	3'074	5'350	7'151	3'550	-1.010	0.193647172	1
ENSBTAG00000011321	*C8ORF80*	2	1	1	2	1	-0.585	1	1
ENSBTAG00000011322	*BT.41831*	1'035	736	873	896	850	-0.077	0.928667559	1
ENSBTAG00000011324	*EMILIN1*	18'893	26'302	23'366	16'362	30'371	0.892	0.243669241	1
ENSBTAG00000011325	*BT.59986*	504	300	391	436	346	-0.333	0.719401266	1
ENSBTAG00000011327	*OLFML3*	18'282	5'134	10'880	15'833	5'928	-1.417	0.068258325	1
ENSBTAG00000011328	*CGREF1*	7'621	1'916	4'406	6'600	2'212	-1.577	0.045853513	1
ENSBTAG00000011330	*protein_coding*	432	134	264	374	155	-1.274	0.20407466	1
ENSBTAG00000011332	*TRIM68*	400	231	307	346	267	-0.377	0.697442866	1
ENSBTAG00000011333	*CLASP2*	4'614	1'121	2'645	3'996	1'294	-1.626	0.042358192	1
ENSBTAG00000011334	*NADKD1*	636	279	436	551	322	-0.774	0.394791122	1
ENSBTAG00000011336	*OTOGL*	121	26	67	105	30	-1.803	0.246943291	1
ENSBTAG00000011337	*ANKRD33B*	6	6	6	5	7	0.415	0.976119138	1
ENSBTAG00000011338	*C10H5orf13*	34'080	22'581	27'794	29'514	26'074	-0.179	0.814712757	1
ENSBTAG00000011339	*TAF8*	694	713	712	601	823	0.454	0.593515462	1
ENSBTAG00000011340	*NSL1*	981	709	834	850	819	-0.053	0.951335851	1
ENSBTAG00000011343	*BT.33242*	109	17	57	94	20	-2.266	0.182487163	1
ENSBTAG00000011344	*TMEM110*	447	531	500	387	613	0.663	0.455223784	1
ENSBTAG00000011345	*BT.103068*	989	721	845	856	833	-0.041	0.963211109	1
ENSBTAG00000011349	*BT.38359*	492	779	663	426	900	1.078	0.211794008	1
ENSBTAG00000011350	*BT.21329*	705	548	622	611	633	0.052	0.953583468	1
ENSBTAG00000011351	*C19ORF6*	3'795	4'465	4'221	3'287	5'156	0.650	0.402383506	1
ENSBTAG00000011352	*TBC1D30*	244	95	161	211	110	-0.946	0.402660606	1
ENSBTAG00000011354	*NUDT9*	1'903	917	1'353	1'648	1'059	-0.638	0.431055876	1
ENSBTAG00000011356	*CAMSAP3*	567	630	609	491	727	0.567	0.512558768	1
ENSBTAG00000011358	*IER3*	893	600	733	773	693	-0.159	0.853529748	1
ENSBTAG00000011359	*CD7*	11	4	7	10	5	-1.044	0.879025484	1
ENSBTAG00000011360	*XAB2*	1'254	2'112	1'762	1'086	2'439	1.167	0.146910213	1
ENSBTAG00000011363	*EVI5*	513	256	370	444	296	-0.588	0.528912565	1

Ensembl gene ID	geneName	counts wildtype horn bud	counts polled horn bud	baseMean	baseMean wildtype horn bud	baseMean polled horn bud	log2FoldChange	pval	padj
ENSBTAG00000011366	*BT.36116*	1	1	1	1	1	0.415	1	1
ENSBTAG00000011367	*LIN7A*	107	138	126	93	159	0.782	0.51835398	1
ENSBTAG00000011368	*NOC3L*	1'494	972	1'208	1'294	1'122	-0.205	0.802241168	1
ENSBTAG00000011370	*pseudogene*	821	892	870	711	1'030	0.535	0.521763201	1
ENSBTAG00000011373	*MAML3*	184	227	211	159	262	0.718	0.492447948	1
ENSBTAG00000011375	*SHC3*	37	4	18	32	5	-2.794	0.380561288	1
ENSBTAG00000011377	*KCNMB3*	57	10	30	49	12	-2.096	0.362009999	1
ENSBTAG00000011379	*KCNH5*	1	0	0	1	0		1	1
ENSBTAG00000011381	*SLC30A3*	6	4	5	5	5	-0.170	1	1
ENSBTAG00000011382	*SLC9A3R1*	1'445	870	1'128	1'251	1'005	-0.317	0.69933276	1
ENSBTAG00000011383	*SNX4*	7'334	3'743	5'337	6'351	4'322	-0.555	0.472526797	1
ENSBTAG00000011384	*TBX5*	32	6	17	28	7	-2.000	0.533547462	1
ENSBTAG00000011387	*BT.103269*	1'912	1'505	1'697	1'656	1'738	0.070	0.930368323	1
ENSBTAG00000011388	*STOML2*	4'279	4'164	4'257	3'706	4'808	0.376	0.627044666	1
ENSBTAG00000011389	*BT.45467*	170	222	202	147	256	0.800	0.449421746	1
ENSBTAG00000011394	*FAM214B*	1'480	1'086	1'268	1'282	1'254	-0.032	0.970744587	1
ENSBTAG00000011395	*DNM1L*	3'248	2'143	2'644	2'813	2'475	-0.185	0.814430916	1
ENSBTAG00000011396	*HNRPC*	12'793	10'595	11'657	11'079	12'234	0.143	0.850764367	1
ENSBTAG00000011397	*UNC13B*	1'751	899	1'277	1'516	1'038	-0.547	0.501304081	1
ENSBTAG00000011398	*PRR7*	229	158	190	198	182	-0.120	0.916122694	1
ENSBTAG00000011399	*YARS2*	544	389	460	471	449	-0.069	0.942156997	1
ENSBTAG00000011400	*DBN1*	10'681	15'696	13'687	9'250	18'124	0.970	0.206705031	1
ENSBTAG00000011401	*PDZD8*	1'949	904	1'366	1'688	1'044	-0.693	0.392331847	1
ENSBTAG00000011402	*protein_coding*	2	2	2	2	2	0.415	1	1
ENSBTAG00000011403	*BT.22507*	2'396	2'165	2'287	2'075	2'500	0.269	0.732540844	1
ENSBTAG00000011405	*CEP72*	362	412	395	314	476	0.602	0.513254132	1
ENSBTAG00000011406	*TESK1*	2'763	3'361	3'137	2'393	3'881	0.698	0.372139126	1
ENSBTAG00000011409	*CD72*	12	11	12	10	13	0.290	0.964579659	1
ENSBTAG00000011411	*SIT1*	29	11	19	25	13	-0.984	0.748093269	1
ENSBTAG00000011412	*LAMB1*	37'040	18'347	26'631	32'078	21'185	-0.599	0.432827087	1
ENSBTAG00000011413	*CCDC107*	766	464	600	663	536	-0.308	0.72390711	1
ENSBTAG00000011414	*EIF2B5*	3'794	2'844	3'285	3'286	3'284	-0.001	1	1
ENSBTAG00000011415	*ETF1*	2'878	2'652	2'777	2'492	3'062	0.297	0.703807851	1
ENSBTAG00000011416	*ARHGEF39*	1'019	575	773	882	664	-0.410	0.627788608	1
ENSBTAG00000011417	*CNTRL*	2'099	1'656	1'865	1'818	1'912	0.073	0.926667278	1
ENSBTAG00000011419	*HSPA9*	5'391	4'269	4'799	4'669	4'929	0.078	0.918677658	1
ENSBTAG00000011420	*CA9*	87	52	68	75	60	-0.327	0.837754989	1
ENSBTAG00000011421	*CD37*	436	691	588	378	798	1.079	0.217495683	1
ENSBTAG00000011423	*SERPINI2*	4	0	2	3	0		0.89545886	1
ENSBTAG00000011424	*TPM2*	3'851	8'188	6'395	3'335	9'455	1.503	0.054961975	1
ENSBTAG00000011425	*PTPRA*	6'482	4'614	5'471	5'614	5'328	-0.075	0.922916271	1
ENSBTAG00000011427	*MGC151671*	15	0	6	13	0		0.491308095	1
ENSBTAG00000011429	*CREB3*	2'096	1'660	1'866	1'815	1'917	0.079	0.921108777	1
ENSBTAG00000011431	*BT.105015*	5'080	6'152	5'752	4'399	7'104	0.691	0.3705191	1
ENSBTAG00000011433	*BT.33487*	3'404	2'805	3'093	2'948	3'239	0.136	0.861367337	1
ENSBTAG00000011434	*NPR2*	2'790	2'546	2'678	2'416	2'940	0.283	0.717486708	1
ENSBTAG00000011435	*NPEPPS*	6'258	4'268	5'174	5'420	4'928	-0.137	0.859683774	1
ENSBTAG00000011437	*SIK1*	425	397	413	368	458	0.317	0.729224344	1
ENSBTAG00000011439	*EPB42*	11	5	8	10	6	-0.722	0.917926652	1
ENSBTAG00000011442	*BT.36669*	176	88	127	152	102	-0.585	0.630799669	1
ENSBTAG00000011444	*HINT2*	438	504	481	379	582	0.618	0.48930729	1
ENSBTAG00000011445	*BT.31084*	897	1'014	974	777	1'171	0.592	0.474264102	1
ENSBTAG00000011446	*SEMA7A*	642	230	411	556	266	-1.066	0.247184842	1
ENSBTAG00000011447	*FAM171A2*	925	1'073	1'020	801	1'239	0.629	0.445419879	1
ENSBTAG00000011448	*C10H14orf38*	27	0	12	23	0		0.244698433	1
ENSBTAG00000011454	*FKBP10*	17'211	17'153	17'356	14'905	19'807	0.410	0.590086913	1
ENSBTAG00000011455	*BT.37506*	667	748	721	578	864	0.580	0.494768468	1
ENSBTAG00000011456	*NT5C3L*	4'336	1'531	2'761	3'755	1'768	-1.087	0.169041385	1
ENSBTAG00000011458	*CPXM*	8'836	14'286	12'074	7'652	16'496	1.108	0.150739075	1
ENSBTAG00000011459	*BT.57716*	1	1	1	1	1	0.415	1	1
ENSBTAG00000011460	*KLHL11*	152	146	150	132	169	0.357	0.757656203	1
ENSBTAG00000011461	*ADORA1*	42	13	26	36	15	-1.277	0.609563219	1
ENSBTAG00000011463	*MID1IP1*	1'082	731	891	937	844	-0.151	0.858097388	1
ENSBTAG00000011466	*CARKD*	1'165	1'264	1'234	1'009	1'460	0.533	0.512191258	1
ENSBTAG00000011467	*BT.88775*	20	21	21	17	24	0.485	0.873025031	1
ENSBTAG00000011470	*protein_coding*	14	6	10	12	7	-0.807	0.881616426	1
ENSBTAG00000011471	*BT.21918*	1'461	1'747	1'641	1'265	2'017	0.673	0.400691582	1
ENSBTAG00000011473	*MYL9*	3'101	4'693	4'052	2'686	5'419	1.013	0.194617861	1
ENSBTAG00000011476	*HOXA9*	3	0	1	3	0		0.936647693	1
ENSBTAG00000011478	*C3ORF75*	257	290	279	223	335	0.589	0.548275002	1
ENSBTAG00000011479	*INPP5K*	1'593	1'849	1'757	1'380	2'135	0.630	0.429646265	1
ENSBTAG00000011480	*PITPNA*	1'416	1'212	1'313	1'226	1'399	0.191	0.813688994	1

Ensembl gene ID	geneName	counts wildtype horn bud	counts polled horn bud	baseMean	baseMean wildtype horn bud	baseMean polled horn bud	log2FoldChange	pval	padj
ENSBTAG00000011481	*IL12RB1*	5	2	3	4	2	-0.907	0.965043111	1
ENSBTAG00000011482	*SLC43A2*	544	476	510	471	550	0.222	0.802303158	1
ENSBTAG00000011483	*SCARF1*	1'238	541	848	1'072	625	-0.779	0.353653032	1
ENSBTAG00000011484	*ZDHHC3*	1'736	1'895	1'846	1'503	2'188	0.541	0.495900248	1
ENSBTAG00000011487	*C10ORF81*	6	23	16	5	27	2.354	0.485077849	1
ENSBTAG00000011488	*PRPF8*	18'713	17'958	18'471	16'206	20'736	0.356	0.640137572	1
ENSBTAG00000011489	*KAZALD1*	2'433	1'837	2'114	2'107	2'121	0.010	0.990248897	1
ENSBTAG00000011490	*TLCD2*	81	54	66	70	62	-0.170	0.922325667	1
ENSBTAG00000011491	*WDR81*	1'867	2'507	2'256	1'617	2'895	0.840	0.288507484	1
ENSBTAG00000011494	*PYGL*	1'684	1'372	1'521	1'458	1'584	0.119	0.88163298	1
ENSBTAG00000011495	*DIP2A*	2'047	1'485	1'744	1'773	1'715	-0.048	0.953381752	1
ENSBTAG00000011496	*protein_coding*	2	0	1	2	0		0.974934741	1
ENSBTAG00000011498	*ZNF184*	1'527	1'291	1'407	1'322	1'491	0.173	0.830085004	1
ENSBTAG00000011504	*ZP2*	30	2	14	26	2	-3.492	0.375178219	1
ENSBTAG00000011505	*RABEP1*	6'289	3'019	4'466	5'446	3'486	-0.644	0.406877415	1
ENSBTAG00000011507	*NUP88*	3'397	2'437	2'878	2'942	2'814	-0.064	0.935717905	1
ENSBTAG00000011509	*SERAC1*	235	153	190	204	177	-0.204	0.853821913	1
ENSBTAG00000011511	*AIM2*	1'350	593	927	1'169	685	-0.772	0.354557243	1
ENSBTAG00000011512	*PIGP*	430	427	433	372	493	0.405	0.65531593	1
ENSBTAG00000011514	*MRPL44*	1'065	709	870	922	819	-0.172	0.83839519	1
ENSBTAG00000011515	*SELL*	49	44	47	42	51	0.260	0.894361484	1
ENSBTAG00000011516	*LRP12*	1'637	1'089	1'338	1'418	1'257	-0.173	0.831811496	1
ENSBTAG00000011517	*CCDC106*	1'240	1'271	1'271	1'074	1'468	0.451	0.578301013	1
ENSBTAG00000011518	*RARB*	393	154	259	340	178	-0.937	0.349964098	1
ENSBTAG00000011524	*XRN1*	351	180	256	304	208	-0.548	0.58495443	1
ENSBTAG00000011525	*SMCT1*	13	2	7	11	2	-2.285	0.731320841	1
ENSBTAG00000011527	*NT5C*	1'049	724	872	908	836	-0.120	0.887571407	1
ENSBTAG00000011528	*FAM165B*	936	761	845	811	879	0.116	0.889814593	1
ENSBTAG00000011529	*ATR*	1'526	631	1'025	1'322	729	-0.859	0.299445408	1
ENSBTAG00000011531	*SS18*	8'447	5'144	6'628	7'315	5'940	-0.301	0.696388492	1
ENSBTAG00000011532	*MLLT6*	1'128	2'276	1'802	977	2'628	1.428	0.077429736	1
ENSBTAG00000011533	*MGC157263*	106	64	83	92	74	-0.313	0.830719846	1
ENSBTAG00000011534	*BT.35948*	413	304	354	358	351	-0.027	0.981152678	1
ENSBTAG00000011537	*pseudogene*	0	2	1	0	2	Inf	0.939077559	1
ENSBTAG00000011538	*KIF1A*	2'362	3'082	2'802	2'046	3'559	0.799	0.309079988	1
ENSBTAG00000011539	*BT.62403*	4'017	5'578	4'960	3'479	6'441	0.889	0.252205695	1
ENSBTAG00000011540	*SPG21*	2'365	1'376	1'819	2'048	1'589	-0.366	0.645988149	1
ENSBTAG00000011541	*RBL1*	566	622	604	490	718	0.551	0.524818796	1
ENSBTAG00000011543	*PDCD1*	1'005	1'402	1'245	870	1'619	0.895	0.272527215	1
ENSBTAG00000011544	*GAPVD1*	1'994	1'477	1'716	1'727	1'705	-0.018	0.983552413	1
ENSBTAG00000011545	*GDA*	123	124	125	107	143	0.427	0.727191005	1
ENSBTAG00000011547	*KBTBD12*	11	13	12	10	15	0.656	0.880852037	1
ENSBTAG00000011548	*AMPD1*	21	124	81	18	143	2.977	0.048141524	1
ENSBTAG00000011549	*TRPC2*	219	426	341	190	492	1.375	0.149704269	1
ENSBTAG00000011551	*BT.67284*	81	29	52	70	33	-1.067	0.537315028	1
ENSBTAG00000011553	*FAHD2A*	1'626	2'647	2'232	1'408	3'056	1.118	0.160089369	1
ENSBTAG00000011554	*SURF6*	596	655	636	516	756	0.551	0.522267968	1
ENSBTAG00000011556	*MED22*	641	798	738	555	921	0.731	0.389283353	1
ENSBTAG00000011558	*GPAT2*	24	7	14	21	8	-1.363	0.707711751	1
ENSBTAG00000011559	*RPL7A*	40'715	52'277	47'812	35'260	60'364	0.776	0.309017812	1
ENSBTAG00000011562	*STK32C*	249	186	215	216	215	-0.006	1	1
ENSBTAG00000011563	*BT.24855*	12	8	10	10	9	-0.170	0.99739059	1
ENSBTAG00000011567	*VPS18*	949	1'431	1'237	822	1'652	1.008	0.217944883	1
ENSBTAG00000011569	*BT.58935*	1	0	0	1	0		1	1
ENSBTAG00000011571	*ACIN1*	16'512	12'162	14'172	14'300	14'043	-0.026	0.973468812	1
ENSBTAG00000011572	*TSPYL5*	1'057	547	774	915	632	-0.535	0.527001749	1
ENSBTAG00000011573	*ENPP6*	32	6	17	28	7	-2.000	0.533547462	1
ENSBTAG00000011575	*RFNG*	4'446	3'138	3'737	3'850	3'623	-0.088	0.911071632	1
ENSBTAG00000011578	*CD44*	10'551	17'132	14'460	9'137	19'782	1.114	0.148036908	1
ENSBTAG00000011579	*CHRM1*	34	11	21	29	13	-1.213	0.667678963	1
ENSBTAG00000011580	*BT.88492*	4'234	8'240	6'591	3'667	9'515	1.376	0.07797853	1
ENSBTAG00000011581	*BSN*	74	82	79	64	95	0.563	0.695504452	1
ENSBTAG00000011582	*BT.87206*	525	680	620	455	785	0.788	0.362656275	1
ENSBTAG00000011583	*APEH*	3'775	4'793	4'402	3'269	5'534	0.759	0.327887704	1
ENSBTAG00000011584	*BT.36123*	5'279	3'132	4'094	4'572	3'617	-0.338	0.663247224	1
ENSBTAG00000011585	*BT.60953*	305	299	305	264	345	0.386	0.689063938	1
ENSBTAG00000011586	*HABP4*	747	426	569	647	492	-0.395	0.652070207	1
ENSBTAG00000011587	*C10ORF90*	10	5	7	9	6	-0.585	0.94357809	1
ENSBTAG00000011588	*RNF123*	2'504	3'639	3'185	2'169	4'202	0.954	0.223819751	1
ENSBTAG00000011589	*CDC14B*	709	589	647	614	680	0.148	0.864424939	1
ENSBTAG00000011591	*KRR1*	2'460	1'155	1'732	2'130	1'334	-0.676	0.398381619	1
ENSBTAG00000011593	*QKI*	2'855	1'621	2'172	2'473	1'872	-0.402	0.61182696	1

Ensembl gene ID	geneName	counts wildtype horn bud	counts polled horn bud	baseMean	baseMean wildtype horn bud	baseMean polled horn bud	log2FoldChange	pval	padj
ENSBTAG00000011595	*IP6K1*	941	2'042	1'586	815	2'358	1.533	0.060250334	1
ENSBTAG00000011596	*SFRS5*	11'350	5'211	7'923	9'829	6'017	-0.708	0.357801136	1
ENSBTAG00000011597	*PDS5B*	2'333	1'560	1'911	2'020	1'801	-0.166	0.83576859	1
ENSBTAG00000011598	*SOX2*	340	140	228	294	162	-0.865	0.401294113	1
ENSBTAG00000011600	*MAP3K8*	177	121	147	153	140	-0.134	0.914240751	1
ENSBTAG00000011601	*BT.52500*	995	1'131	1'084	862	1'306	0.600	0.46474619	1
ENSBTAG00000011602	*RASAL3*	57	15	33	49	17	-1.511	0.484450212	1
ENSBTAG00000011608	*P33MONOX*	4'413	3'966	4'201	3'822	4'580	0.261	0.735558981	1
ENSBTAG00000011611	*OSTC*	8'995	4'785	6'658	7'790	5'525	-0.496	0.519979895	1
ENSBTAG00000011613	*PLS3*	9'581	5'401	7'267	8'297	6'237	-0.412	0.592274136	1
ENSBTAG00000011614	*PDE7A*	397	374	388	344	432	0.329	0.721977559	1
ENSBTAG00000011616	*MPO*	3	3	3	3	3	0.415	1	1
ENSBTAG00000011617	*MAMSTR*	944	435	660	818	502	-0.703	0.414373832	1
ENSBTAG00000011619	*RASIP1*	1'878	1'344	1'589	1'626	1'552	-0.068	0.934123326	1
ENSBTAG00000011620	*PIGU*	1'545	1'390	1'472	1'338	1'605	0.263	0.743729676	1
ENSBTAG00000011621	*IZUMO1*	5	7	6	4	8	0.900	0.909121035	1
ENSBTAG00000011622	*C24H18ORF21*	724	520	614	627	600	-0.062	0.945097112	1
ENSBTAG00000011623	*VMP1*	2'126	1'220	1'625	1'841	1'409	-0.386	0.630050237	1
ENSBTAG00000011624	*FGF21*	8	7	8	7	8	0.222	0.99532173	1
ENSBTAG00000011625	*BT.23510*	786	844	828	681	975	0.518	0.536853513	1
ENSBTAG00000011626	*ATP2C1*	6'295	3'252	4'603	5'452	3'755	-0.538	0.487714363	1
ENSBTAG00000011628	*EGFR*	348	419	393	301	484	0.683	0.458648396	1
ENSBTAG00000011632	*MAP1LC3B*	2'319	1'160	1'674	2'008	1'339	-0.584	0.465647387	1
ENSBTAG00000011633	*CHCHD5*	421	174	283	365	201	-0.860	0.381806738	1
ENSBTAG00000011634	*ACPP*	74	13	40	64	15	-2.094	0.296592536	1
ENSBTAG00000011635	*BT.64608*	117	111	115	101	128	0.339	0.789069191	1
ENSBTAG00000011636	*C11ORF82*	585	228	385	507	263	-0.944	0.309480642	1
ENSBTAG00000011638	*protein_coding*	9	2	5	8	2	-1.755	0.844314416	1
ENSBTAG00000011639	*STK11*	1'455	1'633	1'573	1'260	1'886	0.582	0.468211437	1
ENSBTAG00000011640	*PPAP2B*	7'613	3'936	5'569	6'593	4'545	-0.537	0.487186927	1
ENSBTAG00000011642	*RBM27*	668	784	742	579	905	0.646	0.446178388	1
ENSBTAG00000011643	*SOS1*	972	555	741	842	641	-0.393	0.643613436	1
ENSBTAG00000011644	*BT.38726*	1'124	2'438	1'894	973	2'815	1.532	0.058231411	1
ENSBTAG00000011645	*U2AF1*	3'562	3'277	3'434	3'085	3'784	0.295	0.704305486	1
ENSBTAG00000011646	*ZNF512B*	2'042	3'930	3'153	1'768	4'538	1.360	0.085800174	1
ENSBTAG00000011647	*SLC25A15*	828	471	630	717	544	-0.399	0.644954124	1
ENSBTAG00000011648	*TMLHE*	280	165	217	242	191	-0.348	0.740679415	1
ENSBTAG00000011649	*FARSB*	343	461	415	297	532	0.842	0.357616736	1
ENSBTAG00000011650	*protein_coding*	94	87	91	81	100	0.303	0.827507382	1
ENSBTAG00000011651	*HECTD2*	281	80	168	243	92	-1.397	0.213669584	1
ENSBTAG00000011654	*protein_coding*	21	1	10	18	1	-3.977	0.462631882	1
ENSBTAG00000011655	*SAMD10*	89	47	66	77	54	-0.506	0.748842834	1
ENSBTAG00000011656	*PRPF6*	3'759	5'286	4'680	3'255	6'104	0.907	0.243260915	1
ENSBTAG00000011657	*protein_coding*	57	122	95	49	141	1.513	0.260646086	1
ENSBTAG00000011658	*PTPN18*	984	527	730	852	609	-0.486	0.568167702	1
ENSBTAG00000011659	*PIGZ*	461	506	492	399	584	0.549	0.537027729	1
ENSBTAG00000011661	*CEP152*	283	202	239	245	233	-0.071	0.948974742	1
ENSBTAG00000011666	*THRSP*	113	31	67	98	36	-1.451	0.349291207	1
ENSBTAG00000011667	*PM20D2*	136	131	135	118	151	0.361	0.763118829	1
ENSBTAG00000011668	*KCTD14*	15	13	14	13	15	0.209	0.974190924	1
ENSBTAG00000011671	*pseudogene*	17	0	7	15	0		0.437564044	1
ENSBTAG00000011672	*GABRR1*	9	4	6	8	5	-0.755	0.931866093	1
ENSBTAG00000011680	*PAPLN*	1'604	1'538	1'583	1'389	1'776	0.354	0.657756271	1
ENSBTAG00000011682	*GLI2*	1'250	2'759	2'134	1'083	3'186	1.557	0.053208138	1
ENSBTAG00000011683	*BT.64612*	2'038	1'197	1'574	1'765	1'382	-0.353	0.660648017	1
ENSBTAG00000011684	*RAB11FIP1*	328	304	318	284	351	0.305	0.750315774	1
ENSBTAG00000011689	*LENG8*	20'562	15'107	17'626	17'807	17'444	-0.030	0.96961192	1
ENSBTAG00000011692	*MGC140681*	583	284	416	505	328	-0.623	0.496391931	1
ENSBTAG00000011693	*LENG9*	262	319	298	227	368	0.699	0.470611086	1
ENSBTAG00000011694	*TIMM23*	1'133	720	906	981	831	-0.239	0.775157062	1
ENSBTAG00000011698	*BT.23803*	3'111	2'814	2'972	2'694	3'249	0.270	0.728807619	1
ENSBTAG00000011699	*CDC37*	2'002	2'025	2'036	1'734	2'338	0.432	0.585389171	1
ENSBTAG00000011700	*BT.60335*	93	43	65	81	50	-0.698	0.65657602	1
ENSBTAG00000011704	*protein_coding*	206	280	251	178	323	0.858	0.393505815	1
ENSBTAG00000011706	*TNR*	25	5	14	22	6	-1.907	0.61211339	1
ENSBTAG00000011709	*IMPA1*	2'925	1'567	2'171	2'533	1'809	-0.485	0.539651719	1
ENSBTAG00000011713	*MYO15B*	15	19	17	13	22	0.756	0.816016334	1
ENSBTAG00000011715	*RECQL5*	807	985	918	699	1'137	0.703	0.398490306	1
ENSBTAG00000011717	*SAP30BP*	1'898	1'970	1'959	1'644	2'275	0.469	0.554213995	1
ENSBTAG00000011719	*BT.88515*	4	3	3	3	3	0.000	1	1
ENSBTAG00000011720	*HSD17B6*	4	0	2	3	0		0.89545886	1
ENSBTAG00000011721	*MED13*	1'163	934	1'043	1'007	1'078	0.099	0.904785712	1

Ensembl gene ID	geneName	counts wildtype horn bud	counts polled horn bud	baseMean	baseMean wildtype horn bud	baseMean polled horn bud	log2FoldChange	pval	padj
ENSBTAG00000011723	*GRIK5*	1'187	1'552	1'410	1'028	1'792	0.802	0.321424432	1
ENSBTAG00000011726	*CRYL1*	989	1'747	1'437	856	2'017	1.236	0.129053066	1
ENSBTAG00000011727	*ETAA1*	2'552	1'250	1'827	2'210	1'443	-0.615	0.441007973	1
ENSBTAG00000011729	*STARD3*	1'867	1'910	1'911	1'617	2'205	0.448	0.572332528	1
ENSBTAG00000011730	*TCAP*	903	1'041	992	782	1'202	0.620	0.452856416	1
ENSBTAG00000011731	*PNMT*	3	0	1	3	0		0.936647693	1
ENSBTAG00000011732	*PGAP3*	3'102	2'920	3'029	2'686	3'372	0.328	0.674083242	1
ENSBTAG00000011733	*GIPC2*	59	12	32	51	14	-1.883	0.393742464	1
ENSBTAG00000011734	*ANKRD1*	40	38	39	35	44	0.341	0.870633653	1
ENSBTAG00000011735	*protein_coding*	5	0	2	4	0		0.853268594	1
ENSBTAG00000011736	*IGF2BP1*	153	17	76	133	20	-2.755	0.073203449	1
ENSBTAG00000011738	*TFR2*	60	47	53	52	54	0.063	0.982074816	1
ENSBTAG00000011741	*COL13A1*	1'810	659	1'164	1'568	761	-1.043	0.204966629	1
ENSBTAG00000011742	*TNIK*	526	493	512	456	569	0.322	0.716546936	1
ENSBTAG00000011743	*TLL2*	6	7	7	5	8	0.637	0.939319729	1
ENSBTAG00000011744	*USP31*	238	159	195	206	184	-0.167	0.880546861	1
ENSBTAG00000011748	*SGMS1*	1'440	980	1'189	1'247	1'132	-0.140	0.864824753	1
ENSBTAG00000011750	*protein_coding*	1	0	0	1	0		1	1
ENSBTAG00000011752	*SYNM*	1'151	754	934	997	871	-0.195	0.815445656	1
ENSBTAG00000011753	*WBSCR16*	1'326	1'376	1'369	1'148	1'589	0.468	0.561663871	1
ENSBTAG00000011754	*LAMTOR2*	1'577	1'305	1'436	1'366	1'507	0.142	0.860004997	1
ENSBTAG00000011756	*FLG2*	188	60	116	163	69	-1.233	0.325677958	1
ENSBTAG00000011757	*PSEN1*	4'534	3'514	3'992	3'927	4'058	0.047	0.950892486	1
ENSBTAG00000011758	*protein_coding*	621	647	642	538	747	0.474	0.581469964	1
ENSBTAG00000011759	*C1QL1*	22	5	12	19	6	-1.722	0.669657938	1
ENSBTAG00000011760	*MRPL14*	1'209	1'088	1'152	1'047	1'256	0.263	0.74723535	1
ENSBTAG00000011761	*BT.60913*	920	475	673	797	548	-0.539	0.530423275	1
ENSBTAG00000011763	*BT.19348*	1'990	1'407	1'674	1'723	1'625	-0.085	0.916468042	1
ENSBTAG00000011764	*pseudogene*	284	389	348	246	449	0.869	0.356570861	1
ENSBTAG00000011765	*GABARAPL1*	5'688	6'927	6'462	4'926	7'999	0.699	0.364199628	1
ENSBTAG00000011766	*BT.69954*	837	177	465	725	204	-1.826	0.047291507	1
ENSBTAG00000011767	*MPP5*	1'040	760	889	901	878	-0.037	0.966283246	1
ENSBTAG00000011770	*BT.29711*	2'295	1'461	1'837	1'988	1'687	-0.237	0.76705051	1
ENSBTAG00000011771	*FICD*	157	142	150	136	164	0.270	0.816742455	1
ENSBTAG00000011772	*PPP1R12B*	238	282	266	206	326	0.660	0.505778454	1
ENSBTAG00000011779	*MAL2*	801	530	653	694	612	-0.181	0.83524568	1
ENSBTAG00000011780	*SLITRK4*	25	41	34	22	47	1.129	0.590740393	1
ENSBTAG00000011783	*BT.59524*	2	1	1	2	1	-0.585	1	1
ENSBTAG00000011784	*FGR*	269	194	228	233	224	-0.057	0.961441485	1
ENSBTAG00000011785	*WDR26*	4'542	2'033	3'140	3'933	2'348	-0.745	0.341782361	1
ENSBTAG00000011786	*KIAA0664*	958	1'856	1'486	830	2'143	1.369	0.093049192	1
ENSBTAG00000011787	*ISCU*	5'529	3'057	4'159	4'788	3'530	-0.440	0.570974117	1
ENSBTAG00000011788	*CXORF38*	395	292	340	342	337	-0.021	0.986726337	1
ENSBTAG00000011789	*MGC157163*	2'992	2'355	2'655	2'591	2'719	0.070	0.928916377	1
ENSBTAG00000011790	*MED14*	3'289	1'227	2'133	2'848	1'417	-1.007	0.205823819	1
ENSBTAG00000011793	*FDX1*	1'101	613	831	953	708	-0.430	0.609169018	1
ENSBTAG00000011795	*BT.44465*	1'569	646	1'052	1'359	746	-0.865	0.295055318	1
ENSBTAG00000011796	*TRMT5*	882	831	862	764	960	0.329	0.693447782	1
ENSBTAG00000011798	*STK38L*	1'530	1'005	1'243	1'325	1'160	-0.191	0.81506849	1
ENSBTAG00000011800	*ABCG4*	177	88	127	153	102	-0.593	0.625573465	1
ENSBTAG00000011802	*BT.23508*	90'776	150'463	126'177	78'614	173'740	1.144	0.135630285	1
ENSBTAG00000011804	*ARNTL2*	7	2	4	6	2	-1.392	0.903936064	1
ENSBTAG00000011805	*M6PRBP1*	1'487	1'869	1'723	1'288	2'158	0.745	0.351558722	1
ENSBTAG00000011808	*MSTN*	15	84	55	13	97	2.900	0.099002374	1
ENSBTAG00000011809	*NUDT8*	130	103	116	113	119	0.079	0.955740904	1
ENSBTAG00000011810	*ANGPTL2*	34'184	54'250	46'123	29'604	62'643	1.081	0.158388105	1
ENSBTAG00000011811	*TNPO2*	3'455	6'415	5'200	2'992	7'407	1.308	0.094451316	1
ENSBTAG00000011812	*DIMT1*	2'182	1'157	1'613	1'890	1'336	-0.500	0.532883761	1
ENSBTAG00000011813	*TYR*	66	90	81	57	104	0.862	0.544036654	1
ENSBTAG00000011814	*CENPP*	96	90	94	83	104	0.322	0.814582866	1
ENSBTAG00000011815	*BT.22161*	1'315	1'260	1'297	1'139	1'455	0.353	0.662347077	1
ENSBTAG00000011816	*TBX10*	4	1	2	3	1	-1.585	0.952247636	1
ENSBTAG00000011817	*GABRA2*	15	6	10	13	7	-0.907	0.858439879	1
ENSBTAG00000011818	*BT.34702*	914	1'172	1'072	792	1'353	0.774	0.347041913	1
ENSBTAG00000011819	*PHF14*	2'855	2'141	2'472	2'473	2'472	0.000	1	1
ENSBTAG00000011820	*DAPK2*	124	79	99	107	91	-0.235	0.864744887	1
ENSBTAG00000011822	*C3H1orf228*	13	8	10	11	9	-0.285	0.973425765	1
ENSBTAG00000011823	*CLSTN1*	9'698	12'872	11'631	8'399	14'863	0.824	0.2832644	1
ENSBTAG00000011824	*OGN*	92'916	44'857	66'132	80'468	51'796	-0.636	0.404090678	1
ENSBTAG00000011825	*C7H19ORF43*	2'506	2'369	2'453	2'170	2'735	0.334	0.670473438	1
ENSBTAG00000011826	*PEX26*	200	254	233	173	293	0.760	0.456814928	1
ENSBTAG00000011828	*ARID3B*	92	112	105	80	129	0.699	0.588320736	1

Ensembl gene ID	geneName	counts wildtype horn bud	counts polled horn bud	baseMean	baseMean wildtype horn bud	baseMean polled horn bud	log2FoldChange	pval	padj
ENSBTAG00000011829	*BT.105571*	1'582	1'273	1'420	1'370	1'470	0.102	0.899749214	1
ENSBTAG00000011830	*pseudogene*	14	3	8	12	3	-1.807	0.758305361	1
ENSBTAG00000011831	*BT.105478*	1'291	1'064	1'173	1'118	1'229	0.136	0.867560771	1
ENSBTAG00000011832	*ALDH18A1*	6'950	5'926	6'431	6'019	6'843	0.185	0.809107603	1
ENSBTAG00000011833	*GRIA3*	336	302	320	291	349	0.261	0.7856494	1
ENSBTAG00000011834	*CCDC47*	2'125	1'634	1'864	1'840	1'887	0.036	0.963888251	1
ENSBTAG00000011837	*ASNA1*	2'181	3'336	2'870	1'889	3'852	1.028	0.191910092	1
ENSBTAG00000011838	*FAM113B*	183	175	180	158	202	0.351	0.749127371	1
ENSBTAG00000011839	*HMGCS1*	4'013	3'260	3'620	3'475	3'764	0.115	0.881642855	1
ENSBTAG00000011841	*TCTN3*	600	400	491	520	462	-0.170	0.851064598	1
ENSBTAG00000011842	*DUS3L*	753	998	902	652	1'152	0.821	0.325022384	1
ENSBTAG00000011843	*BT.62921*	35'010	33'456	34'476	30'320	38'632	0.350	0.645091455	1
ENSBTAG00000011844	*protein_coding*	0	3	2	0	3	Inf	0.878646179	1
ENSBTAG00000011846	*BT.21690*	1'149	1'469	1'346	995	1'696	0.769	0.342435038	1
ENSBTAG00000011847	*ASPN*	82'978	29'880	53'182	71'861	34'502	-1.059	0.167279714	1
ENSBTAG00000011849	*HDAC2*	6'146	4'051	5'000	5'323	4'678	-0.186	0.809936574	1
ENSBTAG00000011850	*BT.91994*	1'505	943	1'196	1'303	1'089	-0.259	0.751191427	1
ENSBTAG00000011851	*FYN*	6'297	4'447	5'294	5'453	5'135	-0.087	0.91120466	1
ENSBTAG00000011855	*EXOSC8*	836	493	647	724	569	-0.347	0.687988672	1
ENSBTAG00000011857	*PPM1H*	191	96	138	165	111	-0.577	0.625979455	1
ENSBTAG00000011859	*IPPK*	191	175	184	165	202	0.289	0.791903757	1
ENSBTAG00000011860	*BT.52606*	4'356	1'756	2'900	3'772	2'028	-0.896	0.254870459	1
ENSBTAG00000011861	*C21H15ORF26*	102	48	72	88	55	-0.672	0.655005836	1
ENSBTAG00000011862	*PCDH20*	388	540	480	336	624	0.892	0.319444721	1
ENSBTAG00000011864	*RGMB*	1'408	1'722	1'604	1'219	1'988	0.705	0.379071207	1
ENSBTAG00000011865	*MTA2*	5'550	6'598	6'213	4'806	7'619	0.665	0.388568038	1
ENSBTAG00000011866	*PCBD1*	647	452	541	560	522	-0.102	0.909863785	1
ENSBTAG00000011872	*EML3*	2'566	2'558	2'588	2'222	2'954	0.411	0.600448944	1
ENSBTAG00000011873	*KCNE3*	9	7	8	8	8	0.052	1	1
ENSBTAG00000011875	*CLIP2*	1'838	2'718	2'365	1'592	3'138	0.979	0.216294693	1
ENSBTAG00000011876	*MORC3*	1'902	1'411	1'638	1'647	1'629	-0.016	0.985830623	1
ENSBTAG00000011879	*B4GALT2*	5'136	6'138	5'768	4'448	7'088	0.672	0.383745226	1
ENSBTAG00000011880	*CHAF1B*	513	842	708	444	972	1.130	0.187949414	1
ENSBTAG00000011881	*TDRD10*	64	88	79	55	102	0.874	0.542513433	1
ENSBTAG00000011882	*SLC24A3*	297	218	254	257	252	-0.031	0.980430748	1
ENSBTAG00000011883	*ARL1*	4'673	3'677	4'146	4'047	4'246	0.069	0.92836195	1
ENSBTAG00000011885	*NNT*	3'992	3'269	3'616	3'457	3'775	0.127	0.86991696	1
ENSBTAG00000011887	*CD22*	33	4	17	29	5	-2.629	0.435665511	1
ENSBTAG00000011888	*CCDC24*	227	79	144	197	91	-1.108	0.343360191	1
ENSBTAG00000011889	*NOC4L*	305	416	372	264	480	0.863	0.35421315	1
ENSBTAG00000011890	*RSPH4A*	25	0	11	22	0		0.274776285	1
ENSBTAG00000011892	*SRGAP1*	662	639	656	573	738	0.364	0.671494273	1
ENSBTAG00000011893	*BT.49569*	658	523	587	570	604	0.084	0.92440674	1
ENSBTAG00000011894	*C22H3orf62*	301	166	226	261	192	-0.444	0.668448099	1
ENSBTAG00000011895	*PANK3*	429	348	387	372	402	0.113	0.904231505	1
ENSBTAG00000011896	*protein_coding*	231	109	163	200	126	-0.669	0.552442971	1
ENSBTAG00000011898	*ORAOV1*	374	432	411	324	499	0.623	0.495584435	1
ENSBTAG00000011899	*USP4*	3'111	2'904	3'024	2'694	3'353	0.316	0.685449966	1
ENSBTAG00000011902	*WASH1*	1'614	1'830	1'755	1'398	2'113	0.596	0.454655685	1
ENSBTAG00000011905	*RRAS2*	695	562	625	602	649	0.109	0.900776598	1
ENSBTAG00000011908	*CPQ*	1'265	825	1'024	1'096	953	-0.202	0.807945012	1
ENSBTAG00000011909	*ACVR1*	5'823	3'031	4'271	5'043	3'500	-0.527	0.49719845	1
ENSBTAG00000011910	*STK33*	32	14	22	28	16	-0.778	0.782869547	1
ENSBTAG00000011911	*NCOA7*	1'213	712	936	1'050	822	-0.354	0.670970701	1
ENSBTAG00000011912	*SLC25A25*	961	973	978	832	1'124	0.433	0.600200573	1
ENSBTAG00000011913	*CKAP4*	8'663	8'477	8'645	7'502	9'788	0.384	0.616025889	1
ENSBTAG00000011914	*protein_coding*	221	325	283	191	375	0.971	0.32210873	1
ENSBTAG00000011916	*USP8*	6'269	3'498	4'734	5'429	4'039	-0.427	0.581591266	1
ENSBTAG00000011917	*GPAM*	2'118	1'339	1'690	1'834	1'546	-0.247	0.758411943	1
ENSBTAG00000011918	*BLOC1S1*	1'928	1'831	1'892	1'670	2'114	0.341	0.667592171	1
ENSBTAG00000011921	*TIGIT*	0	1	1	0	1	Inf	0.993540919	1
ENSBTAG00000011922	*PLEC*	13'721	30'216	23'387	11'883	34'890	1.554	0.045382848	1
ENSBTAG00000011924	*BT.62206*	82	78	81	71	90	0.343	0.813828757	1
ENSBTAG00000011926	*protein_coding*	1'850	819	1'274	1'602	946	-0.761	0.350429081	1
ENSBTAG00000011927	*RDH5*	405	145	259	351	167	-1.067	0.287827171	1
ENSBTAG00000011928	*ZBTB20*	16	4	9	14	5	-1.585	0.756102726	1
ENSBTAG00000011929	*BT.42555*	38	19	27	33	22	-0.585	0.815468474	1
ENSBTAG00000011930	*DDX54*	1'507	2'376	2'024	1'305	2'744	1.072	0.179325922	1
ENSBTAG00000011931	*CD63*	32'560	26'693	29'510	28'198	30'822	0.128	0.865173707	1
ENSBTAG00000011932	*PRG4*	179	74	120	155	85	-0.859	0.486762031	1
ENSBTAG00000011933	*FAM110B*	527	451	489	456	521	0.190	0.831624144	1
ENSBTAG00000011934	*PCK2*	969	972	981	839	1'122	0.419	0.611464246	1

Ensembl gene ID	geneName	counts wildtype horn bud	counts polled horn bud	baseMean	baseMean wildtype horn bud	baseMean polled horn bud	log2FoldChange	pval	padj
ENSBTAG00000011935	*RASSF6*	6	5	5	5	6	0.152	1	1
ENSBTAG00000011936	*ATP8B4*	624	797	730	540	920	0.768	0.366487977	1
ENSBTAG00000011937	*RITA*	498	461	482	431	532	0.304	0.734028334	1
ENSBTAG00000011938	*processed_pseudogene*	2	0	1	2	0		0.974934741	1
ENSBTAG00000011939	*BT.104308*	136	27	74	118	31	-1.918	0.2012735	1
ENSBTAG00000011940	*ZNF831*	2	3	3	2	3	1.000	0.97171833	1
ENSBTAG00000011941	*LYZ1*	4	0	2	3	0		0.89545886	1
ENSBTAG00000011943	*TPR*	7'039	4'113	5'423	6'096	4'749	-0.360	0.641014057	1
ENSBTAG00000011944	*BT.64166*	23	2	11	20	2	-3.109	0.497345932	1
ENSBTAG00000011945	*TAF7*	6'916	4'950	5'853	5'989	5'716	-0.067	0.930995667	1
ENSBTAG00000011946	*C1ORF27*	1'349	591	925	1'168	682	-0.776	0.352277702	1
ENSBTAG00000011950	*KIAA0090*	5'443	6'006	5'824	4'714	6'935	0.557	0.469684941	1
ENSBTAG00000011951	*MRTO4*	2'834	3'807	3'425	2'454	4'396	0.841	0.282005687	1
ENSBTAG00000011952	*SULT1E1*	16	2	8	14	2	-2.585	0.65347607	1
ENSBTAG00000011953	*BT.69152*	6'197	3'621	4'774	5'367	4'181	-0.360	0.641762898	1
ENSBTAG00000011954	*SEC11C*	1'504	821	1'125	1'303	948	-0.458	0.576200488	1
ENSBTAG00000011956	*CNNM1*	8	3	5	7	3	-1.000	0.918861955	1
ENSBTAG00000011957	*CREBL2*	1'896	1'216	1'523	1'642	1'404	-0.226	0.779498337	1
ENSBTAG00000011959	*BT.31596*	105	32	64	91	37	-1.299	0.409678213	1
ENSBTAG00000011960	*GOT1*	1'739	1'608	1'681	1'506	1'857	0.302	0.704820885	1
ENSBTAG00000011961	*PF4*	55	25	38	48	29	-0.722	0.722367236	1
ENSBTAG00000011962	*PLA2G3*	30	13	20	26	15	-0.791	0.788388247	1
ENSBTAG00000011963	*RPS19*	7'340	11'294	9'699	6'357	13'041	1.037	0.179017784	1
ENSBTAG00000011964	*DIS3L2*	440	711	601	381	821	1.107	0.204839725	1
ENSBTAG00000011966	*LAMC1*	9'738	8'065	8'873	8'433	9'313	0.143	0.851125623	1
ENSBTAG00000011969	*HSPB1*	9'650	12'574	11'438	8'357	14'519	0.797	0.298985474	1
ENSBTAG00000011970	*FNIP2*	480	403	441	416	465	0.163	0.858326721	1
ENSBTAG00000011971	*NRP2*	2'411	2'147	2'284	2'088	2'479	0.248	0.75276086	1
ENSBTAG00000011973	*BT.94996*	167	117	140	145	135	-0.098	0.940156177	1
ENSBTAG00000011975	*SERPINB1*	425	231	317	368	267	-0.465	0.628890065	1
ENSBTAG00000011976	*CYP4B1*	41	4	20	36	5	-2.943	0.332018627	1
ENSBTAG00000011981	*MYBL1*	110	64	85	95	74	-0.366	0.798863049	1
ENSBTAG00000011982	*BT.42411*	31	16	23	27	18	-0.539	0.851125253	1
ENSBTAG00000011984	*BT.81161*	602	245	402	521	283	-0.882	0.338748402	1
ENSBTAG00000011985	*BT.87837*	80	50	64	69	58	-0.263	0.875777074	1
ENSBTAG00000011986	*PLSCR4*	2'114	1'254	1'639	1'831	1'448	-0.338	0.672956828	1
ENSBTAG00000011987	*C10H14ORF1*	360	274	314	312	316	0.021	0.98537176	1
ENSBTAG00000011988	*KIAA0100*	2'408	2'486	2'478	2'085	2'871	0.461	0.557170405	1
ENSBTAG00000011990	*BT.2598*	46	24	34	40	28	-0.524	0.814306895	1
ENSBTAG00000011991	*DRGX*	21	11	15	18	13	-0.518	0.894018059	1
ENSBTAG00000011992	*BT.54789*	79	13	42	68	15	-2.188	0.264270692	1
ENSBTAG00000011994	*NDEL1*	2'777	1'788	2'235	2'405	2'065	-0.220	0.781002884	1
ENSBTAG00000011995	*protein_coding*	2	0	1	2	0		0.974934741	1
ENSBTAG00000011997	*ZMIZ2*	1'636	2'221	1'991	1'417	2'565	0.856	0.282026007	1
ENSBTAG00000011998	*DUT*	1'508	1'755	1'666	1'306	2'026	0.634	0.428049715	1
ENSBTAG00000012002	*BCAR1*	2'533	1'481	1'952	2'194	1'710	-0.359	0.651345073	1
ENSBTAG00000012003	*PPIA*	9'031	10'134	9'761	7'821	11'702	0.581	0.447927208	1
ENSBTAG00000012004	*TGFB3*	7'569	5'698	6'567	6'555	6'579	0.005	0.993948547	1
ENSBTAG00000012005	*BT.18569*	887	809	851	768	934	0.282	0.735667785	1
ENSBTAG00000012007	*SOCS2*	1'684	1'150	1'393	1'458	1'328	-0.135	0.868189333	1
ENSBTAG00000012010	*PANX1*	342	275	307	296	318	0.100	0.919311437	1
ENSBTAG00000012012	*CYB5A*	1'894	1'711	1'808	1'640	1'976	0.268	0.735428922	1
ENSBTAG00000012014	*CDX1*	2	1	1	2	1	-0.585	1	1
ENSBTAG00000012015	*FSIP1*	42	2	19	36	2	-3.977	0.229264678	1
ENSBTAG00000012016	*LMTK3*	316	65	174	274	75	-1.866	0.097496989	1
ENSBTAG00000012019	*IRS2*	189	314	263	164	363	1.147	0.250777907	1
ENSBTAG00000012020	*TRAF5*	874	903	900	757	1'043	0.462	0.578541395	1
ENSBTAG00000012022	*TBC1D2B*	2'456	3'331	2'987	2'127	3'846	0.855	0.276028826	1
ENSBTAG00000012023	*BT.27962*	9	0	4	8	0		0.690676647	1
ENSBTAG00000012024	*SLC29A2*	431	193	298	373	223	-0.744	0.443921338	1
ENSBTAG00000012025	*LMX1A*	143	91	114	124	105	-0.237	0.855847569	1
ENSBTAG00000012026	*BT.61364*	44	50	48	38	58	0.599	0.739680939	1
ENSBTAG00000012029	*KLRB1*	7	0	3	6	0		0.769774961	1
ENSBTAG00000012030	*TTLL1*	1'076	713	878	932	823	-0.179	0.831969324	1
ENSBTAG00000012031	*protein_coding*	817	244	495	708	282	-1.328	0.140253621	1
ENSBTAG00000012032	*PDE4A*	825	885	868	714	1'022	0.516	0.536218982	1
ENSBTAG00000012034	*KRT4*	26'162	20'560	23'199	22'657	23'741	0.067	0.928739843	1
ENSBTAG00000012035	*BT.26113*	342	146	232	296	169	-0.813	0.428062168	1
ENSBTAG00000012037	*DGUOK*	1'970	1'385	1'653	1'706	1'599	-0.093	0.908407871	1
ENSBTAG00000012038	*TRIM56*	165	219	198	143	253	0.824	0.438576931	1
ENSBTAG00000012039	*PHACTR1*	844	711	776	731	821	0.168	0.842819692	1
ENSBTAG00000012040	*GIT1*	2'358	2'597	2'520	2'042	2'999	0.554	0.480440878	1

Ensembl gene ID	geneName	counts wildtype horn bud	counts polled horn bud	baseMean	baseMean wildtype horn bud	baseMean polled horn bud	log2FoldChange	pval	padj
ENSBTAG00000012041	*SPG7*	3'140	3'693	3'492	2'719	4'264	0.649	0.404779063	1
ENSBTAG00000012043	*PADI3*	1	1	1	1	1	0.415	1	1
ENSBTAG00000012044	*BT.22248*	44'200	47'231	46'408	38'278	54'538	0.511	0.501460832	1
ENSBTAG00000012046	*JUNB*	764	499	619	662	576	-0.199	0.819357942	1
ENSBTAG00000012047	*BT.25287*	1'213	869	1'027	1'050	1'003	-0.066	0.93785691	1
ENSBTAG00000012048	*CARM1*	2'586	4'611	3'782	2'240	5'324	1.249	0.111859302	1
ENSBTAG00000012049	*WIPI2*	2'057	2'546	2'361	1'781	2'940	0.723	0.359670649	1
ENSBTAG00000012050	*ORAI2*	550	551	556	476	636	0.418	0.633274745	1
ENSBTAG00000012052	*PADI4*	7	0	3	6	0		0.769774961	1
ENSBTAG00000012053	*ACCSL*	14	3	8	12	3	-1.807	0.758305361	1
ENSBTAG00000012054	*ALDH3B2*	521	263	377	451	304	-0.571	0.539231834	1
ENSBTAG00000012057	*GIF*	4	1	2	3	1	-1.585	0.952247636	1
ENSBTAG00000012058	*GMDS*	1'643	2'521	2'167	1'423	2'911	1.033	0.19421871	1
ENSBTAG00000012059	*MVD*	1'259	2'301	1'874	1'090	2'657	1.285	0.110176193	1
ENSBTAG00000012060	*LPIN2*	1'344	1'614	1'514	1'164	1'864	0.679	0.398392901	1
ENSBTAG00000012061	*BT.95772*	69	41	54	60	47	-0.336	0.851483306	1
ENSBTAG00000012062	*PRDX2*	11'172	12'018	11'776	9'675	13'877	0.520	0.495944065	1
ENSBTAG00000012063	*E2F4*	2'160	2'228	2'222	1'871	2'573	0.460	0.559815494	1
ENSBTAG00000012064	*ZNF473*	641	488	559	555	563	0.022	0.981889095	1
ENSBTAG00000012065	*BT.68903*	1'859	1'976	1'946	1'610	2'282	0.503	0.525817393	1
ENSBTAG00000012066	*BT.59680*	7'447	3'470	5'228	6'449	4'007	-0.687	0.37511587	1
ENSBTAG00000012067	*EPG5*	414	254	326	359	293	-0.290	0.763233842	1
ENSBTAG00000012068	*BRIP1*	292	192	237	253	222	-0.190	0.855894841	1
ENSBTAG00000012069	*CCDC167*	656	676	674	568	781	0.458	0.592145741	1
ENSBTAG00000012070	*INTS2*	1'011	496	724	876	573	-0.612	0.47233991	1
ENSBTAG00000012071	*ASGR1*	2	0	1	2	0		0.974934741	1
ENSBTAG00000012072	*NDUFS8*	1'255	1'052	1'151	1'087	1'215	0.160	0.844189519	1
ENSBTAG00000012073	*VOPP1*	1'407	1'931	1'724	1'218	2'230	0.872	0.276494886	1
ENSBTAG00000012074	*BT.68596*	338	321	332	293	371	0.341	0.72015144	1
ENSBTAG00000012077	*C26H10ORF6*	3'222	1'958	2'526	2'790	2'261	-0.304	0.699815372	1
ENSBTAG00000012078	*TMEM176A*	23	19	21	20	22	0.139	0.977962804	1
ENSBTAG00000012079	*MYLK4*	22	91	62	19	105	2.463	0.132569337	1
ENSBTAG00000012081	*BT.65675*	2'560	2'491	2'547	2'217	2'876	0.376	0.631873954	1
ENSBTAG00000012082	*BT.20111*	285	320	308	247	370	0.582	0.545071089	1
ENSBTAG00000012083	*IRX3*	731	866	817	633	1'000	0.660	0.432564732	1
ENSBTAG00000012086	*CNOT8*	1'583	1'365	1'474	1'371	1'576	0.201	0.802078098	1
ENSBTAG00000012087	*NHLRC1*	49	37	43	42	43	0.010	1	1
ENSBTAG00000012088	*BT.61392*	23'926	37'866	32'222	20'721	43'724	1.077	0.160240148	1
ENSBTAG00000012089	*AMELX*	6	5	5	5	6	0.152	1	1
ENSBTAG00000012090	*GGTA1*	1'449	1'248	1'348	1'255	1'441	0.200	0.804737148	1
ENSBTAG00000012094	*BT.49274*	2'841	2'868	2'886	2'460	3'312	0.429	0.583203337	1
ENSBTAG00000012095	*GEMIN5*	1'014	1'041	1'040	878	1'202	0.453	0.581679835	1
ENSBTAG00000012096	*BCKDHB*	883	549	699	765	634	-0.271	0.752455475	1
ENSBTAG00000012097	*RSPH6A*	14	6	10	12	7	-0.807	0.881616426	1
ENSBTAG00000012099	*TMEM87B*	581	476	526	503	550	0.127	0.886140116	1
ENSBTAG00000012101	*SYMPK*	2'101	3'620	3'000	1'820	4'180	1.200	0.128595471	1
ENSBTAG00000012102	*BT.59682*	2'357	2'944	2'720	2'041	3'399	0.736	0.348809445	1
ENSBTAG00000012103	*MAP2K4*	2'919	2'033	2'438	2'528	2'348	-0.107	0.892963003	1
ENSBTAG00000012104	*FOXA3*	4	3	3	3	3	0.000	1	1
ENSBTAG00000012106	*BT.63691*	250	279	269	217	322	0.573	0.561992592	1
ENSBTAG00000012107	*BT.20517*	3'516	2'135	2'755	3'045	2'465	-0.305	0.697921079	1
ENSBTAG00000012109	*EDN3*	240	143	186	208	165	-0.332	0.761812092	1
ENSBTAG00000012111	*PRDM5*	329	176	244	285	203	-0.487	0.631365024	1
ENSBTAG00000012112	*SLC7A6*	1'867	1'182	1'491	1'617	1'365	-0.244	0.761957111	1
ENSBTAG00000012113	*HCCS*	369	332	351	320	383	0.263	0.780599879	1
ENSBTAG00000012117	*ATP6AP1*	4'707	6'347	5'703	4'076	7'329	0.846	0.274095098	1
ENSBTAG00000012120	*TIPARP*	1'189	747	946	1'030	863	-0.256	0.759095643	1
ENSBTAG00000012121	*BT.28781*	1'296	1'152	1'226	1'122	1'330	0.245	0.762820058	1
ENSBTAG00000012124	*GPBP1*	9'507	6'472	7'853	8'233	7'473	-0.140	0.8560858	1
ENSBTAG00000012125	*GDI1*	14'967	13'830	14'466	12'962	15'970	0.301	0.692491341	1
ENSBTAG00000012126	*PNPLA7*	905	1'087	1'019	784	1'255	0.679	0.410209165	1
ENSBTAG00000012128	*BT.39765*	578	505	542	501	583	0.220	0.802572437	1
ENSBTAG00000012131	*protein_coding*	1	0	0	1	0		1	1
ENSBTAG00000012132	*processed_pseudogene*	2'300	2'566	2'477	1'992	2'963	0.573	0.466184803	1
ENSBTAG00000012135	*LARP1B*	482	164	303	417	189	-1.140	0.241122643	1
ENSBTAG00000012139	*SIX1*	166	56	104	144	65	-1.153	0.375243508	1
ENSBTAG00000012140	*BT.29666*	5	7	6	4	8	0.900	0.909121035	1
ENSBTAG00000012141	*RAD9A*	567	902	766	491	1'042	1.085	0.202240802	1
ENSBTAG00000012142	*RAD18*	457	311	377	396	359	-0.140	0.8829218	1
ENSBTAG00000012143	*TECPR2*	571	793	705	495	916	0.889	0.298510083	1
ENSBTAG00000012144	*ZMYND19*	482	570	538	417	658	0.657	0.455284216	1
ENSBTAG00000012146	*BT.48845*	3'655	4'967	4'450	3'165	5'735	0.858	0.269915042	1

Ensembl gene ID	geneName	counts wildtype horn bud	counts polled horn bud	baseMean	baseMean wildtype horn bud	baseMean polled horn bud	log2FoldChange	pval	padj
ENSBTAG00000012147	*ARRDC1*	1'492	1'326	1'412	1'292	1'531	0.245	0.760973724	1
ENSBTAG00000012148	*EHMT1*	4'867	5'316	5'177	4'215	6'138	0.542	0.482235987	1
ENSBTAG00000012150	*PLK5*	86	24	51	74	28	-1.426	0.413638792	1
ENSBTAG00000012152	*BT.37860*	9'205	8'783	9'057	7'972	10'142	0.347	0.649596225	1
ENSBTAG00000012156	*NAIF1*	589	422	499	510	487	-0.066	0.943813835	1
ENSBTAG00000012157	*CCDC158*	155	43	92	134	50	-1.435	0.29364479	1
ENSBTAG00000012158	*C16H1orf158*	8	0	3	7	0		0.72953145	1
ENSBTAG00000012159	*CNBP*	8'449	5'804	7'009	7'317	6'702	-0.127	0.869667086	1
ENSBTAG00000012161	*pseudogene*	39	49	45	34	57	0.744	0.686334536	1
ENSBTAG00000012163	*ITGB1BP2*	241	150	191	209	173	-0.269	0.805858403	1
ENSBTAG00000012168	*RANBP6*	1'436	997	1'197	1'244	1'151	-0.111	0.892761542	1
ENSBTAG00000012169	*BT.21568*	556	414	480	482	478	-0.010	0.994159252	1
ENSBTAG00000012170	*BT.20945*	5'225	2'902	3'938	4'525	3'351	-0.433	0.577143148	1
ENSBTAG00000012172	*MIDN*	1'042	1'323	1'215	902	1'528	0.759	0.351751819	1
ENSBTAG00000012176	*QPCTL*	770	800	795	667	924	0.470	0.576245249	1
ENSBTAG00000012177	*SNRPD2*	5'987	5'185	5'586	5'185	5'987	0.208	0.786990486	1
ENSBTAG00000012178	*NR1D1*	259	262	263	224	303	0.432	0.664347657	1
ENSBTAG00000012180	*BMF*	127	128	129	110	148	0.426	0.724615948	1
ENSBTAG00000012181	*GNA11*	1'285	2'333	1'903	1'113	2'694	1.275	0.112525683	1
ENSBTAG00000012182	*DIRAS3*	1'207	316	705	1'045	365	-1.518	0.079923381	1
ENSBTAG00000012184	*PTTG1*	286	320	309	248	370	0.577	0.548457795	1
ENSBTAG00000012185	*CLEC4E*	5	1	3	4	1	-1.907	0.9188325	1
ENSBTAG00000012186	*DKKL1*	910	854	887	788	986	0.323	0.697751343	1
ENSBTAG00000012189	*TMEM86B*	192	177	185	166	204	0.298	0.785023608	1
ENSBTAG00000012191	*WWC2*	990	802	892	857	926	0.111	0.894193644	1
ENSBTAG00000012192	*BT.87447*	5	3	4	4	3	-0.322	1	1
ENSBTAG00000012193	*TTC28*	1'217	1'338	1'299	1'054	1'545	0.552	0.495858479	1
ENSBTAG00000012194	*DENND2C*	3'239	2'846	3'046	2'805	3'286	0.228	0.769271065	1
ENSBTAG00000012197	*KIAA0415*	1'862	1'467	1'653	1'613	1'694	0.071	0.929145055	1
ENSBTAG00000012199	*IMP3*	742	541	634	643	625	-0.041	0.964982688	1
ENSBTAG00000012200	*ANKRD13D*	1'099	1'206	1'172	952	1'393	0.549	0.500843054	1
ENSBTAG00000012201	*CYP3A5*	60	22	39	52	25	-1.032	0.605444956	1
ENSBTAG00000012205	*CPT1C*	926	276	560	802	319	-1.331	0.133017469	1
ENSBTAG00000012206	*SNX33*	528	864	727	457	998	1.126	0.188420756	1
ENSBTAG00000012207	*CLDN22*	1	0	0	1	0		1	1
ENSBTAG00000012208	*BT.62645*	204	149	174	177	172	-0.038	0.978944359	1
ENSBTAG00000012210	*BT.24055*	3	1	2	3	1	-1.170	0.985253077	1
ENSBTAG00000012211	*HOXA5*	3	10	7	3	12	2.152	0.727540088	1
ENSBTAG00000012212	*CYP26B1*	1'028	825	921	890	953	0.098	0.906827515	1
ENSBTAG00000012213	*WAPAL*	2'528	1'697	2'074	2'189	1'960	-0.160	0.840732212	1
ENSBTAG00000012215	*CPNE7*	244	46	132	211	53	-1.992	0.104177264	1
ENSBTAG00000012216	*MLKL*	79	44	60	68	51	-0.429	0.796921139	1
ENSBTAG00000012217	*PLA2G2F*	19	42	32	16	48	1.559	0.472468741	1
ENSBTAG00000012219	*CSPG4*	3'103	5'254	4'377	2'687	6'067	1.175	0.13309553	1
ENSBTAG00000012222	*FA2H*	256	139	191	222	161	-0.466	0.666275157	1
ENSBTAG00000012223	*BT.64160*	6	5	5	5	6	0.152	1	1
ENSBTAG00000012225	*KPNA2*	2'631	2'273	2'452	2'279	2'625	0.204	0.79472354	1
ENSBTAG00000012228	*SEMA4A*	1'194	1'452	1'355	1'034	1'677	0.697	0.388994923	1
ENSBTAG00000012229	*protein_coding*	12	2	6	10	2	-2.170	0.758651977	1
ENSBTAG00000012232	*CYC1*	3'935	4'142	4'095	3'408	4'783	0.489	0.527950484	1
ENSBTAG00000012234	*RHBG*	581	501	541	503	579	0.201	0.819423325	1
ENSBTAG00000012235	*SHARPIN*	894	896	904	774	1'035	0.418	0.614901692	1
ENSBTAG00000012237	*BT.103111*	42'406	25'319	32'980	36'725	29'236	-0.329	0.665740275	1
ENSBTAG00000012239	*SLC37A3*	3'964	2'469	3'142	3'433	2'851	-0.268	0.731856789	1
ENSBTAG00000012241	*POLD2*	1'535	1'747	1'673	1'329	2'017	0.602	0.451624845	1
ENSBTAG00000012242	*MAF1*	3'200	3'745	3'548	2'771	4'324	0.642	0.409738258	1
ENSBTAG00000012243	*POLR1E*	1'030	996	1'021	892	1'150	0.367	0.656014345	1
ENSBTAG00000012244	*TUBA1B*	30'661	32'307	31'929	26'553	37'305	0.490	0.51884181	1
ENSBTAG00000012246	*ANO2*	16	25	21	14	29	1.059	0.702607494	1
ENSBTAG00000012247	*MXRA8*	16'584	8'838	12'284	14'362	10'205	-0.493	0.519591762	1
ENSBTAG00000012249	*PRR15L*	201	164	182	174	189	0.122	0.914844078	1
ENSBTAG00000012250	*PLDN*	2'112	1'644	1'864	1'829	1'898	0.054	0.946139766	1
ENSBTAG00000012251	*BT.47673*	284	198	237	246	229	-0.105	0.922247863	1
ENSBTAG00000012252	*BT.64749*	231	253	246	200	292	0.546	0.58811212	1
ENSBTAG00000012253	*EIF2C1*	664	1'115	931	575	1'287	1.163	0.164928187	1
ENSBTAG00000012254	*LAPTM4B*	1	0	0	1	0		1	1
ENSBTAG00000012259	*PNPO*	280	235	257	242	271	0.162	0.873312336	1
ENSBTAG00000012260	*MTRF1*	253	217	235	219	251	0.194	0.851687572	1
ENSBTAG00000012261	*RAB19*	24	26	25	21	30	0.531	0.839166484	1
ENSBTAG00000012262	*BT.65534*	410	548	494	355	633	0.834	0.349813353	1
ENSBTAG00000012263	*ASAP3*	3'290	2'522	2'881	2'849	2'912	0.032	0.967613649	1
ENSBTAG00000012265	*VWF*	11'693	6'841	9'013	10'126	7'899	-0.358	0.640477914	1

Ensembl gene ID	geneName	counts wildtype horn bud	counts polled horn bud	baseMean	baseMean wildtype horn bud	baseMean polled horn bud	log2FoldChange	pval	padj
ENSBTAG00000012267	*ZFC3H1*	6'007	2'368	3'968	5'202	2'734	-0.928	0.234628213	1
ENSBTAG00000012271	*THAP2*	859	417	613	744	482	-0.628	0.4696441	1
ENSBTAG00000012272	*TMEM19*	1'581	1'537	1'572	1'369	1'775	0.374	0.640019805	1
ENSBTAG00000012273	*BT.26122*	655	639	653	567	738	0.379	0.658664792	1
ENSBTAG00000012274	*CIZ1*	3'258	3'884	3'653	2'822	4'485	0.669	0.390394258	1
ENSBTAG00000012275	*ENDOU*	1'018	508	734	882	587	-0.588	0.489623256	1
ENSBTAG00000012276	*BT.87694*	41'305	31'989	36'354	35'771	36'938	0.046	0.950738107	1
ENSBTAG00000012277	*SRRD*	917	1'011	981	794	1'167	0.556	0.501253731	1
ENSBTAG00000012278	*MTMR12*	466	253	348	404	292	-0.466	0.621694006	1
ENSBTAG00000012279	*BT.105682*	742	729	742	643	842	0.390	0.645555017	1
ENSBTAG00000012280	*PGLYRP2*	28	12	19	24	14	-0.807	0.794158041	1
ENSBTAG00000012284	*BT.76984*	829	735	783	718	849	0.241	0.774625895	1
ENSBTAG00000012285	*ESRRB*	0	3	2	0	3	Inf	0.878646179	1
ENSBTAG00000012288	*BT.56280*	1'259	1'791	1'579	1'090	2'068	0.924	0.251433125	1
ENSBTAG00000012289	*AKR7A3*	1'426	1'164	1'290	1'235	1'344	0.122	0.880217261	1
ENSBTAG00000012290	*SCNN1B*	21	10	15	18	12	-0.655	0.864027356	1
ENSBTAG00000012291	*BT.44820*	780	971	898	675	1'121	0.731	0.380705852	1
ENSBTAG00000012293	*BT.58992*	4'431	2'247	3'216	3'837	2'595	-0.565	0.470054228	1
ENSBTAG00000012295	*NKD1*	1'031	1'329	1'214	893	1'535	0.781	0.338252903	1
ENSBTAG00000012296	*BT.76310*	19	2	9	16	2	-2.833	0.582174212	1
ENSBTAG00000012299	*REPS1*	3'049	1'887	2'410	2'641	2'179	-0.277	0.725262762	1
ENSBTAG00000012302	*RTN4RL1*	115	149	136	100	172	0.789	0.504724238	1
ENSBTAG00000012305	*LPHN2*	11'569	5'915	8'425	10'019	6'830	-0.553	0.471880769	1
ENSBTAG00000012307	*DTNA*	1'079	809	934	934	934	0.000	1	1
ENSBTAG00000012312	*ROR1*	675	557	614	585	643	0.138	0.874180727	1
ENSBTAG00000012314	*LDLR*	2'430	2'892	2'722	2'104	3'339	0.666	0.395912038	1
ENSBTAG00000012317	*PNP*	2'352	1'280	1'757	2'037	1'478	-0.463	0.562353513	1
ENSBTAG00000012319	*KCNE1L*	167	38	94	145	44	-1.721	0.206873318	1
ENSBTAG00000012320	*CETN1*	1	0	0	1	0		1	1
ENSBTAG00000012321	*ZFAND6*	900	707	798	779	816	0.067	0.937440397	1
ENSBTAG00000012322	*FAM212A*	1'304	1'580	1'477	1'129	1'824	0.692	0.390224367	1
ENSBTAG00000012330	*B2M*	18'189	11'766	14'669	15'752	13'586	-0.213	0.780219036	1
ENSBTAG00000012332	*C11H2orf49*	1'798	1'298	1'528	1'557	1'499	-0.055	0.94678758	1
ENSBTAG00000012333	*pseudogene*	364	426	404	315	492	0.642	0.483965962	1
ENSBTAG00000012334	*BT.72247*	4	0	2	3	0		0.89545886	1
ENSBTAG00000012335	*UBA7*	926	725	820	802	837	0.062	0.941832461	1
ENSBTAG00000012338	*FAH*	329	252	288	285	291	0.030	0.978374472	1
ENSBTAG00000012341	*BT.64603*	1'908	1'547	1'719	1'652	1'786	0.112	0.88773818	1
ENSBTAG00000012342	*LIMA1*	9'649	8'776	9'245	8'356	10'134	0.278	0.715650342	1
ENSBTAG00000012343	*TSPAN5*	624	664	654	540	767	0.505	0.556711863	1
ENSBTAG00000012344	*RPL26*	10'254	19'223	15'539	8'880	22'197	1.322	0.087523401	1
ENSBTAG00000012347	*OSTA*	3	0	1	3	0		0.936647693	1
ENSBTAG00000012348	*C3H1ORF92*	556	608	592	482	702	0.544	0.531200311	1
ENSBTAG00000012349	*LAX1*	97	52	72	84	60	-0.484	0.749950155	1
ENSBTAG00000012350	*MAT2B*	1'562	1'595	1'597	1'353	1'842	0.445	0.577909534	1
ENSBTAG00000012351	*BT.62516*	439	341	387	380	394	0.051	0.958520475	1
ENSBTAG00000012352	*PARP16*	1'591	1'456	1'530	1'378	1'681	0.287	0.720106768	1
ENSBTAG00000012353	*BT.27106*	3'966	1'984	2'863	3'435	2'291	-0.584	0.456306326	1
ENSBTAG00000012355	*RNF220*	4'387	5'220	4'913	3'799	6'028	0.666	0.389435618	1
ENSBTAG00000012357	*BT.37863*	38	54	48	33	62	0.922	0.605988843	1
ENSBTAG00000012361	*ARHGEF11*	3'333	2'723	3'015	2'886	3'144	0.123	0.873985402	1
ENSBTAG00000012363	*BT.102548*	1	0	0	1	0		1	1
ENSBTAG00000012365	*ANP32A*	7'827	8'785	8'461	6'778	10'144	0.582	0.448301199	1
ENSBTAG00000012366	*MRPS23*	1'891	1'423	1'640	1'638	1'643	0.005	0.995381258	1
ENSBTAG00000012371	*CPD*	1'240	969	1'096	1'074	1'119	0.059	0.942728497	1
ENSBTAG00000012372	*TMEM53*	115	179	153	100	207	1.053	0.356541886	1
ENSBTAG00000012374	*C2ORF54*	375	453	424	325	523	0.688	0.450016297	1
ENSBTAG00000012375	*BT.19212*	3'534	2'088	2'736	3'061	2'411	-0.344	0.661075268	1
ENSBTAG00000012376	*SNRPD1*	96	103	101	83	119	0.517	0.693943356	1
ENSBTAG00000012377	*ECHDC3*	575	380	468	498	439	-0.183	0.841061791	1
ENSBTAG00000012380	*HK1*	2'907	3'858	3'486	2'518	4'455	0.823	0.291793987	1
ENSBTAG00000012382	*KCTD9*	1'811	1'101	1'420	1'568	1'271	-0.303	0.707875093	1
ENSBTAG00000012383	*BT.48958*	4'313	2'767	3'465	3'735	3'195	-0.225	0.772804761	1
ENSBTAG00000012384	*TFEB*	356	593	497	308	685	1.151	0.198626386	1
ENSBTAG00000012385	*NFX1*	2'982	1'689	2'266	2'582	1'950	-0.405	0.608131518	1
ENSBTAG00000012387	*PAM*	29'148	14'022	20'717	25'243	16'191	-0.641	0.401756647	1
ENSBTAG00000012390	*ZNF483*	3	2	2	3	2	-0.170	1	1
ENSBTAG00000012391	*FAM63B*	435	263	340	377	304	-0.311	0.744194907	1
ENSBTAG00000012393	*AGT*	41	10	24	36	12	-1.621	0.538651466	1
ENSBTAG00000012394	*CCDC85A*	180	58	111	156	67	-1.219	0.337666412	1
ENSBTAG00000012397	*DCK*	590	407	490	511	470	-0.121	0.894939824	1
ENSBTAG00000012398	*APOC3*	2	0	1	2	0		0.974934741	1

Ensembl gene ID	geneName	counts wildtype horn bud	counts polled horn bud	baseMean	baseMean wildtype horn bud	baseMean polled horn bud	log2FoldChange	pval	padj
ENSBTAG00000012401	*pseudogene*	812	798	812	703	921	0.390	0.642266844	1
ENSBTAG00000012403	*ARG1*	71	4	33	61	5	-3.735	0.117423754	1
ENSBTAG00000012405	*PEAR1*	4'518	3'179	3'792	3'913	3'671	-0.092	0.906485634	1
ENSBTAG00000012406	*ZBP1*	25	12	18	22	14	-0.644	0.847278186	1
ENSBTAG00000012408	*RAB27A*	141	65	99	122	75	-0.702	0.596608876	1
ENSBTAG00000012412	*URB1*	969	1'285	1'161	839	1'484	0.822	0.315270239	1
ENSBTAG00000012414	*protein_coding*	0	1	1	0	1	Inf	0.993540919	1
ENSBTAG00000012416	*ZNF511*	691	588	639	598	679	0.182	0.832959983	1
ENSBTAG00000012417	*C15H11orf46*	2'336	1'434	1'839	2'023	1'656	-0.289	0.717133484	1
ENSBTAG00000012418	*MED23*	2'431	1'522	1'931	2'105	1'757	-0.261	0.743467247	1
ENSBTAG00000012419	*BCL2L15*	17	8	12	15	9	-0.672	0.883107327	1
ENSBTAG00000012421	*C17H22ORF25*	190	137	161	165	158	-0.057	0.966506003	1
ENSBTAG00000012423	*SENP1*	831	476	635	720	550	-0.389	0.653069321	1
ENSBTAG00000012425	*LDOC1L*	278	410	357	241	473	0.976	0.299033695	1
ENSBTAG00000012426	*INO80C*	877	727	799	760	839	0.144	0.864089461	1
ENSBTAG00000012432	*FDFT1*	3'245	3'580	3'472	2'810	4'134	0.557	0.474392185	1
ENSBTAG00000012433	*DENND1B*	112	70	89	97	81	-0.263	0.854704795	1
ENSBTAG00000012434	*ENOX1*	1'362	331	781	1'180	382	-1.626	0.058916807	1
ENSBTAG00000012436	*HES7*	27	26	27	23	30	0.361	0.892345382	1
ENSBTAG00000012439	*GUCA1B*	3	3	3	3	3	0.415	1	1
ENSBTAG00000012441	*SMAD7*	623	365	480	540	421	-0.356	0.691467069	1
ENSBTAG00000012442	*CATB*	26'726	21'252	23'843	23'145	24'540	0.084	0.910993461	1
ENSBTAG00000012443	*DIAPH3*	655	578	617	567	667	0.235	0.786414725	1
ENSBTAG00000012444	*BT.29890*	991	881	938	858	1'017	0.245	0.767337691	1
ENSBTAG00000012446	*MTHFSD*	422	333	375	365	385	0.073	0.939164178	1
ENSBTAG00000012447	*BT.101937*	18'996	12'370	15'367	16'451	14'284	-0.204	0.789827442	1
ENSBTAG00000012448	*CCDC91*	1'118	594	827	968	686	-0.497	0.55420094	1
ENSBTAG00000012449	*AFF3*	195	245	226	169	283	0.744	0.469358237	1
ENSBTAG00000012450	*RAPGEF2*	3'809	1'867	2'727	3'299	2'156	-0.614	0.434735342	1
ENSBTAG00000012451	*HLA-DMB*	262	107	175	227	124	-0.877	0.426282174	1
ENSBTAG00000012454	*SLC35A3*	1'649	696	1'116	1'428	804	-0.829	0.313215695	1
ENSBTAG00000012456	*HEPACAM2*	230	116	167	199	134	-0.572	0.609159666	1
ENSBTAG00000012458	*BT.87546*	23	29	27	20	33	0.749	0.760676063	1
ENSBTAG00000012460	*ELF5*	463	202	317	401	233	-0.782	0.41571842	1
ENSBTAG00000012462	*MAP1LC3C*	429	956	738	372	1'104	1.571	0.068832267	1
ENSBTAG00000012463	*ZMAT3*	50	29	38	43	33	-0.371	0.862489734	1
ENSBTAG00000012464	*AFAP1*	497	746	646	430	861	1.001	0.246978798	1
ENSBTAG00000012465	*FOXC2*	366	57	191	317	66	-2.268	0.041840223	1
ENSBTAG00000012467	*MASP1*	2'308	3'819	3'204	1'999	4'410	1.142	0.14701854	1
ENSBTAG00000012470	*SNAPIN*	1'510	902	1'175	1'308	1'042	-0.328	0.688204701	1
ENSBTAG00000012471	*BT.18113*	7'014	7'190	7'188	6'074	8'302	0.451	0.556878733	1
ENSBTAG00000012475	*BT.48104*	288	114	191	249	132	-0.922	0.392464353	1
ENSBTAG00000012476	*NRBF2*	911	766	837	789	885	0.165	0.844176368	1
ENSBTAG00000012478	*ALOXE3*	148	50	93	128	58	-1.151	0.395719656	1
ENSBTAG00000012480	*TBC1D13*	1'038	1'637	1'395	899	1'890	1.072	0.18713033	1
ENSBTAG00000012481	*RANGAP1*	1'485	2'312	1'978	1'286	2'670	1.054	0.187084496	1
ENSBTAG00000012482	*CDCP1*	214	449	352	185	518	1.484	0.118835399	1
ENSBTAG00000012484	*ENDOG*	534	651	607	462	752	0.701	0.418976701	1
ENSBTAG00000012485	*C9ORF114*	394	489	453	341	565	0.727	0.420242149	1
ENSBTAG00000012489	*C21H15orf27*	231	289	267	200	334	0.738	0.456330505	1
ENSBTAG00000012490	*ETFA*	2'615	1'589	2'050	2'265	1'835	-0.304	0.701995459	1
ENSBTAG00000012491	*BT.23474*	2	0	1	2	0		0.974934741	1
ENSBTAG00000012495	*SF3B4*	1'511	2'444	2'065	1'309	2'822	1.109	0.164782128	1
ENSBTAG00000012496	*BT.49269*	1'190	501	805	1'031	579	-0.833	0.324072506	1
ENSBTAG00000012497	*KHDRBS1*	6'374	4'718	5'484	5'520	5'448	-0.019	0.981277346	1
ENSBTAG00000012498	*BT.61625*	0	1	1	0	1	Inf	0.993540919	1
ENSBTAG00000012499	*RPGRIP1L*	789	472	614	683	545	-0.326	0.707527688	1
ENSBTAG00000012500	*RARA*	4'357	6'569	5'679	3'773	7'585	1.007	0.194244372	1
ENSBTAG00000012501	*FTO*	4'287	4'155	4'255	3'713	4'798	0.370	0.632371618	1
ENSBTAG00000012503	*MGAT3*	161	170	168	139	196	0.494	0.657465754	1
ENSBTAG00000012504	*BT.20690*	1'315	1'579	1'481	1'139	1'823	0.679	0.399063431	1
ENSBTAG00000012505	*ARHGEF17*	3'430	6'410	5'186	2'970	7'402	1.317	0.092202851	1
ENSBTAG00000012507	*PDZD3*	60	76	70	52	88	0.756	0.616182109	1
ENSBTAG00000012508	*OSGIN1*	7	16	12	6	18	1.608	0.686923859	1
ENSBTAG00000012509	*DYRK1B*	1'008	1'468	1'284	873	1'695	0.957	0.240225424	1
ENSBTAG00000012510	*PLCB3*	2'302	3'014	2'737	1'994	3'480	0.804	0.30648685	1
ENSBTAG00000012511	*BT.55802*	1'055	945	1'002	914	1'091	0.256	0.755999388	1
ENSBTAG00000012512	*GPR137*	1'157	1'244	1'219	1'002	1'436	0.520	0.522883453	1
ENSBTAG00000012514	*PODN*	1'454	433	880	1'259	500	-1.333	0.114926821	1
ENSBTAG00000012516	*SLC1A7*	20	51	38	17	59	1.766	0.378791921	1
ENSBTAG00000012518	*PCDH1*	989	1'091	1'058	856	1'260	0.557	0.498121425	1
ENSBTAG00000012519	*XDH*	754	304	502	653	351	-0.895	0.315523963	1

Ensembl gene ID	geneName	counts wildtype horn bud	counts polled horn bud	baseMean	baseMean wildtype horn bud	baseMean polled horn bud	log2FoldChange	pval	padj
ENSBTAG00000012522	*ZNF283*	488	202	328	423	233	-0.857	0.36917704	1
ENSBTAG00000012525	*BT.29579*	83	42	60	72	48	-0.568	0.728784387	1
ENSBTAG00000012526	*APBB1IP*	1'018	978	1'005	882	1'129	0.357	0.664667457	1
ENSBTAG00000012533	*protein_coding*	1	0	0	1	0		1	1
ENSBTAG00000012534	*BEND3*	202	244	228	175	282	0.688	0.502760779	1
ENSBTAG00000012535	*SCRG1*	88	9	43	76	10	-2.874	0.146958576	1
ENSBTAG00000012537	*BT.48879*	1'544	1'319	1'430	1'337	1'523	0.188	0.815406742	1
ENSBTAG00000012538	*KLK14*	3	7	5	3	8	1.637	0.840440358	1
ENSBTAG00000012541	*BT.28305*	2'618	1'023	1'724	2'267	1'181	-0.941	0.241376246	1
ENSBTAG00000012543	*EDA*	96	68	81	83	79	-0.082	0.96396539	1
ENSBTAG00000012544	*BT.49564*	2'615	1'903	2'231	2'265	2'197	-0.043	0.957274606	1
ENSBTAG00000012545	*CASC4*	14'356	6'020	9'692	12'433	6'951	-0.839	0.275770349	1
ENSBTAG00000012550	*TAOK3*	1'402	583	944	1'214	673	-0.851	0.307153114	1
ENSBTAG00000012552	*FMR1*	2'985	1'432	2'119	2'585	1'654	-0.645	0.41618362	1
ENSBTAG00000012554	*ANKAR*	55	33	43	48	38	-0.322	0.874633247	1
ENSBTAG00000012555	*RBM15*	261	205	231	226	237	0.067	0.951684236	1
ENSBTAG00000012557	*OSGEPL1*	394	257	319	341	297	-0.201	0.836189525	1
ENSBTAG00000012558	*ADAMTS12*	314	69	176	272	80	-1.771	0.113967957	1
ENSBTAG00000012560	*SFTPC*	44	67	58	38	77	1.022	0.531752303	1
ENSBTAG00000012561	*ORMDL1*	2'556	2'221	2'389	2'214	2'565	0.212	0.786744113	1
ENSBTAG00000012562	*RNF26*	463	807	666	401	932	1.217	0.159680941	1
ENSBTAG00000012564	*KIAA1267*	2'036	1'591	1'800	1'763	1'837	0.059	0.940637077	1
ENSBTAG00000012565	*PCNX*	1'306	917	1'095	1'131	1'059	-0.095	0.909258512	1
ENSBTAG00000012566	*KIAA1383*	478	184	313	414	212	-0.962	0.318320184	1
ENSBTAG00000012567	*BT.60929*	216	468	364	187	540	1.531	0.106160105	1
ENSBTAG00000012575	*BT.36159*	2'215	1'771	1'982	1'918	2'045	0.092	0.907105238	1
ENSBTAG00000012577	*UVSSA*	191	238	220	165	275	0.732	0.479190841	1
ENSBTAG00000012581	*BT.102071*	656	242	424	568	279	-1.024	0.263779197	1
ENSBTAG00000012582	*IARS2*	7'692	4'536	5'950	6'661	5'238	-0.347	0.652859463	1
ENSBTAG00000012584	*GJB3*	228	427	345	197	493	1.320	0.165085072	1
ENSBTAG00000012585	*RAB3GAP2*	3'325	1'934	2'556	2'880	2'233	-0.367	0.641077896	1
ENSBTAG00000012586	*BT.89909*	3'158	3'083	3'147	2'735	3'560	0.380	0.625345797	1
ENSBTAG00000012587	*BABAM1*	1'586	1'897	1'782	1'374	2'190	0.673	0.398542732	1
ENSBTAG00000012589	*HSPE1*	5'967	3'332	4'508	5'168	3'847	-0.426	0.582909915	1
ENSBTAG00000012590	*BT.62304*	29	24	26	25	28	0.142	0.969049213	1
ENSBTAG00000012592	*ANKLE1*	27	56	44	23	65	1.468	0.429679004	1
ENSBTAG00000012594	*MRPS6*	3'088	2'663	2'875	2'674	3'075	0.201	0.796214429	1
ENSBTAG00000012595	*HRSP12*	496	366	426	430	423	-0.023	0.983089212	1
ENSBTAG00000012596	*POP1*	551	375	455	477	433	-0.140	0.879030223	1
ENSBTAG00000012599	*SMAD3*	524	687	624	454	793	0.806	0.351867834	1
ENSBTAG00000012600	*BT.105073*	493	421	457	427	486	0.187	0.836050595	1
ENSBTAG00000012601	*TEKT4*	2	0	1	2	0		0.974934741	1
ENSBTAG00000012604	*C1ORF130*	47	23	34	41	27	-0.616	0.780031245	1
ENSBTAG00000012605	*GPR157*	22	14	18	19	16	-0.237	0.958766648	1
ENSBTAG00000012606	*ZNF541*	27	25	26	23	29	0.304	0.91341554	1
ENSBTAG00000012607	*BT.21459*	2'609	3'054	2'893	2'259	3'526	0.642	0.412078841	1
ENSBTAG00000012609	*SCO2*	911	972	956	789	1'122	0.509	0.539057918	1
ENSBTAG00000012615	*ZEB2*	9'105	9'371	9'353	7'885	10'821	0.457	0.550704052	1
ENSBTAG00000012618	*SLC23A3*	19	25	23	16	29	0.811	0.764792716	1
ENSBTAG00000012619	*C2H2ORF24*	1'306	2'088	1'771	1'131	2'411	1.092	0.173970792	1
ENSBTAG00000012620	*C2ORF17*	6'182	9'342	8'070	5'354	10'787	1.011	0.190780258	1
ENSBTAG00000012621	*RTN4RL2*	66	65	66	57	75	0.393	0.803339648	1
ENSBTAG00000012622	*TRA2A*	13'147	4'936	8'543	11'386	5'700	-0.998	0.196223473	1
ENSBTAG00000012623	*NDP*	229	85	148	198	98	-1.015	0.380644864	1
ENSBTAG00000012625	*pseudogene*	5	46	29	4	53	3.617	0.149490941	1
ENSBTAG00000012626	*B4GALT5*	395	239	309	342	276	-0.310	0.749715345	1
ENSBTAG00000012628	*SLC1A2*	5	5	5	4	6	0.415	0.985023983	1
ENSBTAG00000012629	*ZNF362*	2'461	3'158	2'889	2'131	3'647	0.775	0.323283648	1
ENSBTAG00000012630	*PAMR1*	1'112	837	965	963	966	0.005	0.995765231	1
ENSBTAG00000012632	*TECR*	3'065	3'522	3'361	2'654	4'067	0.616	0.429727286	1
ENSBTAG00000012634	*NDUFB7*	1'491	1'425	1'468	1'291	1'645	0.350	0.663300676	1
ENSBTAG00000012636	*IKZF4*	184	147	165	159	170	0.091	0.939596402	1
ENSBTAG00000012637	*ECRG4*	4'408	1'983	3'054	3'817	2'290	-0.737	0.346858495	1
ENSBTAG00000012640	*S100A8*	84	34	56	73	39	-0.890	0.594419392	1
ENSBTAG00000012642	*TRADD*	1'061	905	982	919	1'045	0.186	0.822321847	1
ENSBTAG00000012644	*UROD*	1'278	938	1'095	1'107	1'083	-0.031	0.971553542	1
ENSBTAG00000012646	*PLCXD2*	21	13	17	18	15	-0.277	0.951008818	1
ENSBTAG00000012647	*ERBB4*	5	2	3	4	2	-0.907	0.965043111	1
ENSBTAG00000012648	*ZSWIM5*	323	70	180	280	81	-1.791	0.107767238	1
ENSBTAG00000012649	*NAA38*	1'866	1'195	1'498	1'616	1'380	-0.228	0.777672692	1
ENSBTAG00000012654	*CHCHD1*	1'291	844	1'046	1'118	975	-0.198	0.810922496	1
ENSBTAG00000012656	*OVOL1*	360	261	307	312	301	-0.049	0.963919659	1

Ensembl gene ID	geneName	counts wildtype horn bud	counts polled horn bud	baseMean	baseMean wildtype horn bud	baseMean polled horn bud	log2FoldChange	pval	padj
ENSBTAG00000012657	*BT.22294*	5'097	7'627	6'611	4'414	8'807	0.997	0.198039746	1
ENSBTAG00000012658	*BT.49045*	991	785	882	858	906	0.079	0.925212082	1
ENSBTAG00000012659	*BT.46048*	2'142	680	1'320	1'855	785	-1.240	0.129905969	1
ENSBTAG00000012664	*NDST2*	3'675	3'554	3'643	3'183	4'104	0.367	0.636468152	1
ENSBTAG00000012667	*BT.103020*	997	1'109	1'072	863	1'281	0.569	0.488542196	1
ENSBTAG00000012668	*PILRA*	8	3	5	7	3	-1.000	0.918861955	1
ENSBTAG00000012671	*TNIP1*	1'515	2'031	1'829	1'312	2'345	0.838	0.294004941	1
ENSBTAG00000012672	*ZNHIT3*	537	317	416	465	366	-0.345	0.706873499	1
ENSBTAG00000012673	*PCTK3*	388	431	417	336	498	0.567	0.534442569	1
ENSBTAG00000012674	*BT.34176*	2'667	2'819	2'782	2'310	3'255	0.495	0.52706329	1
ENSBTAG00000012675	*BT.58356*	1'376	1'121	1'243	1'192	1'294	0.119	0.883264545	1
ENSBTAG00000012676	*KRT79*	559	281	404	484	324	-0.577	0.530235835	1
ENSBTAG00000012677	*ARHGAP12*	3'144	1'930	2'476	2'723	2'229	-0.289	0.71380763	1
ENSBTAG00000012678	*GLT25D1*	5'102	9'581	7'741	4'418	11'063	1.324	0.088761907	1
ENSBTAG00000012681	*CORO2B*	857	1'141	1'030	742	1'318	0.828	0.316116235	1
ENSBTAG00000012682	*UNC13A*	96	6	45	83	7	-3.585	0.07615354	1
ENSBTAG00000012683	*SRRM2*	37'537	30'600	33'921	32'508	35'334	0.120	0.873552918	1
ENSBTAG00000012687	*BT.63550*	1'121	1'009	1'068	971	1'165	0.263	0.748338921	1
ENSBTAG00000012688	*BT.20860*	33	2	15	29	2	-3.629	0.331956808	1
ENSBTAG00000012691	*BT.20425*	2'026	1'263	1'606	1'755	1'458	-0.267	0.739844453	1
ENSBTAG00000012692	*protein_coding*	5	7	6	4	8	0.900	0.909121035	1
ENSBTAG00000012693	*BT.78082*	56	32	43	48	37	-0.392	0.844443139	1
ENSBTAG00000012694	*UHMK1*	64	25	42	55	29	-0.941	0.623224919	1
ENSBTAG00000012695	*LCK*	143	175	163	124	202	0.706	0.528200898	1
ENSBTAG00000012697	*PGAM1*	8'520	8'859	8'804	7'379	10'229	0.471	0.538218289	1
ENSBTAG00000012698	*HDAC1*	6'259	5'053	5'628	5'420	5'835	0.106	0.889734002	1
ENSBTAG00000012699	*EXOSC1*	937	613	760	811	708	-0.197	0.817292069	1
ENSBTAG00000012700	*CD247*	730	435	567	632	502	-0.332	0.705474475	1
ENSBTAG00000012702	*ZDHHC16*	1'290	1'062	1'172	1'117	1'226	0.134	0.869119563	1
ENSBTAG00000012703	*GLO1*	3'996	3'249	3'606	3'461	3'752	0.116	0.880385391	1
ENSBTAG00000012704	*MMS19*	3'398	3'865	3'703	2'943	4'463	0.601	0.439676719	1
ENSBTAG00000012705	*BT.49359*	6'659	3'474	4'889	5'767	4'011	-0.524	0.498735856	1
ENSBTAG00000012706	*RTP1*	6	1	3	5	1	-2.170	0.885240772	1
ENSBTAG00000012708	*BT.19231*	724	145	397	627	167	-1.905	0.043901628	1
ENSBTAG00000012712	*AQR*	1'401	1'025	1'198	1'213	1'184	-0.036	0.966732978	1
ENSBTAG00000012715	*KIF26B*	137	159	151	119	184	0.630	0.582322827	1
ENSBTAG00000012718	*XK*	37	17	26	32	20	-0.707	0.78236821	1
ENSBTAG00000012719	*UBTD1*	798	820	819	691	947	0.454	0.588162681	1
ENSBTAG00000012720	*ANKRD2*	114	98	106	99	113	0.197	0.883346827	1
ENSBTAG00000012721	*HOGA1*	247	144	190	214	166	-0.363	0.738166384	1
ENSBTAG00000012722	*AGBL4*	18	12	15	16	14	-0.170	0.983826208	1
ENSBTAG00000012723	*HAUS4*	1'203	989	1'092	1'042	1'142	0.132	0.871758436	1
ENSBTAG00000012724	*AJUBA*	1'932	3'033	2'588	1'673	3'502	1.066	0.177894188	1
ENSBTAG00000012725	*C10H14ORF93*	457	440	452	396	508	0.360	0.689363387	1
ENSBTAG00000012726	*PSMB5*	3'489	3'549	3'560	3'022	4'098	0.440	0.571469167	1
ENSBTAG00000012728	*PSMB11*	3	2	2	3	2	-0.170	1	1
ENSBTAG00000012729	*ARHGEF9*	712	394	536	617	455	-0.439	0.619369046	1
ENSBTAG00000012735	*C9ORF117*	150	163	159	130	188	0.535	0.635778667	1
ENSBTAG00000012736	*PTRH1*	103	176	146	89	203	1.188	0.305954447	1
ENSBTAG00000012737	*GPR65*	13	6	9	11	7	-0.700	0.90529819	1
ENSBTAG00000012738	*ZNF827*	381	378	383	330	436	0.404	0.662672516	1
ENSBTAG00000012739	*C1QBP*	2'245	1'800	2'011	1'944	2'078	0.096	0.903003805	1
ENSBTAG00000012740	*PIGB*	1'355	558	909	1'173	644	-0.865	0.300836986	1
ENSBTAG00000012741	*CCPG1*	3'909	1'955	2'821	3'385	2'257	-0.585	0.456230555	1
ENSBTAG00000012742	*BT.27443*	22	19	20	19	22	0.204	0.959171191	1
ENSBTAG00000012744	*UBE2M*	1'992	2'353	2'221	1'725	2'717	0.655	0.406863922	1
ENSBTAG00000012745	*PPAN-P2RY11*	495	675	604	429	779	0.862	0.321155653	1
ENSBTAG00000012746	*KLHDC3*	7'486	5'655	6'506	6'483	6'530	0.010	0.988775468	1
ENSBTAG00000012747	*FAM3A*	1'071	1'021	1'053	928	1'179	0.346	0.673408218	1
ENSBTAG00000012748	*BT.85776*	5	0	2	4	0		0.853268594	1
ENSBTAG00000012749	*BT.103213*	13'331	19'110	16'806	11'545	22'066	0.935	0.223100215	1
ENSBTAG00000012750	*UPK2*	58	6	29	50	7	-2.858	0.244531603	1
ENSBTAG00000012751	*BTBD6*	1'795	2'068	1'971	1'555	2'388	0.619	0.435145816	1
ENSBTAG00000012752	*MRPL2*	773	843	821	669	973	0.540	0.519770782	1
ENSBTAG00000012753	*BRF1*	1'225	1'869	1'610	1'061	2'158	1.025	0.203629119	1
ENSBTAG00000012755	*KLC4*	1'856	2'001	1'959	1'607	2'311	0.524	0.509076239	1
ENSBTAG00000012756	*CCT7*	6'466	7'697	7'244	5'600	8'888	0.666	0.386280415	1
ENSBTAG00000012757	*BT.91783*	341	234	283	295	270	-0.128	0.899768386	1
ENSBTAG00000012758	*YIPF5*	4'784	3'889	4'317	4'143	4'491	0.116	0.880124258	1
ENSBTAG00000012759	*FBXO41*	94	60	75	81	69	-0.233	0.881841501	1
ENSBTAG00000012760	*NDUFB3*	1'790	797	1'235	1'550	920	-0.752	0.356608204	1
ENSBTAG00000012762	*C7H5orf24*	1'123	700	890	973	808	-0.267	0.7499508	1

Ensembl gene ID	geneName	counts wildtype horn bud	counts polled horn bud	baseMean	baseMean wildtype horn bud	baseMean polled horn bud	log2FoldChange	pval	padj
ENSBTAG00000012763	*protein_coding*	0	5	3	0	6	Inf	0.757530434	1
ENSBTAG00000012765	*protein_coding*	260	287	278	225	331	0.558	0.570180928	1
ENSBTAG00000012768	*HMGXB3*	2'172	2'827	2'573	1'881	3'264	0.795	0.312550605	1
ENSBTAG00000012771	*CSF1R*	3'586	2'456	2'971	3'106	2'836	-0.131	0.867711096	1
ENSBTAG00000012772	*GFI1B*	1	1	1	1	1	0.415	1	1
ENSBTAG00000012774	*RAB7B*	1'425	651	993	1'234	752	-0.715	0.388070276	1
ENSBTAG00000012777	*BT.51656*	1'009	1'684	1'409	874	1'945	1.154	0.156134408	1
ENSBTAG00000012778	*CAPN2*	10'212	6'001	7'887	8'844	6'929	-0.352	0.646910105	1
ENSBTAG00000012779	*FAM49A*	246	84	155	213	97	-1.135	0.32105629	1
ENSBTAG00000012780	*LPO*	93	7	44	81	8	-3.317	0.098232795	1
ENSBTAG00000012781	*TP53BP2*	3'132	2'502	2'801	2'712	2'889	0.091	0.907045995	1
ENSBTAG00000012782	*AQP6*	20	2	10	17	2	-2.907	0.559880543	1
ENSBTAG00000012784	*RACGAP1*	2'161	2'973	2'652	1'871	3'433	0.875	0.266503369	1
ENSBTAG00000012785	*TEPP*	3	3	3	3	3	0.415	1	1
ENSBTAG00000012787	*C16H1ORF55*	1'077	838	950	933	968	0.053	0.949529701	1
ENSBTAG00000012788	*COX6A1*	5'142	4'321	4'721	4'453	4'989	0.164	0.831246144	1
ENSBTAG00000012789	*PRKD1*	2'048	1'151	1'551	1'774	1'329	-0.416	0.604508834	1
ENSBTAG00000012790	*TRIAP1*	1'730	1'084	1'375	1'498	1'252	-0.259	0.749001935	1
ENSBTAG00000012791	*GATC*	552	509	533	478	588	0.298	0.735209154	1
ENSBTAG00000012792	*SFRS9*	1'753	1'395	1'564	1'518	1'611	0.085	0.915053182	1
ENSBTAG00000012794	*PAH*	40	0	17	35	0		0.116452486	1
ENSBTAG00000012796	*ZNF428*	2'492	2'435	2'485	2'158	2'812	0.382	0.626713983	1
ENSBTAG00000012797	*DCUN1D1*	1'672	802	1'187	1'448	926	-0.645	0.43017601	1
ENSBTAG00000012798	*KCNH8*	67	16	38	58	18	-1.651	0.412844723	1
ENSBTAG00000012800	*MMAA*	646	403	512	559	465	-0.266	0.765862576	1
ENSBTAG00000012801	*BT.17653*	11	0	5	10	0		0.617662983	1
ENSBTAG00000012803	*SRP19*	2'366	1'833	2'083	2'049	2'117	0.047	0.95272145	1
ENSBTAG00000012804	*BT.67755*	14'761	10'606	12'515	12'783	12'247	-0.062	0.936140772	1
ENSBTAG00000012805	*TSPAN13*	4'309	1'605	2'792	3'732	1'853	-1.010	0.200590216	1
ENSBTAG00000012808	*MASP2*	1'633	617	1'063	1'414	712	-0.989	0.231741356	1
ENSBTAG00000012809	*KIAA1045*	53	42	47	46	48	0.079	0.976807471	1
ENSBTAG00000012815	*CLEC12A*	217	49	122	188	57	-1.732	0.164825114	1
ENSBTAG00000012816	*protein_coding*	8'744	8'660	8'786	7'573	10'000	0.401	0.600137275	1
ENSBTAG00000012817	*JAG1*	6'675	4'933	5'738	5'781	5'696	-0.021	0.978886927	1
ENSBTAG00000012818	*BT.67505*	620	524	571	537	605	0.172	0.844149198	1
ENSBTAG00000012820	*STX3*	369	742	588	320	857	1.423	0.106572329	1
ENSBTAG00000012822	*BT.50441*	25	20	22	22	23	0.093	0.990498651	1
ENSBTAG00000012823	*GUCA1A*	30	21	25	26	24	-0.100	0.987328991	1
ENSBTAG00000012824	*INTU*	202	120	157	175	139	-0.336	0.770430888	1
ENSBTAG00000012826	*BT.50967*	6	2	4	5	2	-1.170	0.934316631	1
ENSBTAG00000012827	*TTLL11*	25	32	29	22	37	0.771	0.740099979	1
ENSBTAG00000012828	*TBC1D15*	2'220	1'484	1'818	1'923	1'714	-0.166	0.835738757	1
ENSBTAG00000012829	*EBI3*	188	67	120	163	77	-1.073	0.385671108	1
ENSBTAG00000012830	*CCDC94*	448	621	553	388	717	0.886	0.313481439	1
ENSBTAG00000012833	*ATG2B*	894	551	705	774	636	-0.283	0.740983072	1
ENSBTAG00000012834	*ARSI*	273	304	294	236	351	0.570	0.557112111	1
ENSBTAG00000012837	*COL6A6*	5'377	2'404	3'716	4'657	2'776	-0.746	0.338663069	1
ENSBTAG00000012838	*RDH12*	577	183	356	500	211	-1.242	0.189563655	1
ENSBTAG00000012844	*LRSAM1*	718	945	856	622	1'091	0.811	0.333089089	1
ENSBTAG00000012845	*SDF2*	3'468	2'557	2'978	3'003	2'953	-0.025	0.976006829	1
ENSBTAG00000012846	*C11ORF41*	178	83	125	154	96	-0.686	0.57448765	1
ENSBTAG00000012847	*FAM129B*	6'494	9'459	8'273	5'624	10'922	0.958	0.214567802	1
ENSBTAG00000012848	*PTPRU*	913	499	683	791	576	-0.457	0.594366721	1
ENSBTAG00000012849	*BT.32410*	43'307	48'484	46'745	37'505	55'985	0.578	0.447226803	1
ENSBTAG00000012850	*BT.103897*	31	2	15	27	2	-3.539	0.360208319	1
ENSBTAG00000012851	*SGLT1*	7	6	6	6	7	0.193	1	1
ENSBTAG00000012852	*BT.45343*	936	615	760	811	710	-0.191	0.823036082	1
ENSBTAG00000012854	*GSDMB*	227	259	248	197	299	0.605	0.547727585	1
ENSBTAG00000012856	*ARR3*	1	1	1	1	1	0.415	1	1
ENSBTAG00000012857	*CNNM2*	260	213	236	225	246	0.127	0.903424486	1
ENSBTAG00000012858	*NT5C2*	4'083	3'076	3'544	3'536	3'552	0.006	0.993098451	1
ENSBTAG00000012860	*PDZD11*	3'639	3'360	3'516	3'151	3'880	0.300	0.699152849	1
ENSBTAG00000012861	*BT.24872*	717	825	787	621	953	0.617	0.463876192	1
ENSBTAG00000012863	*BT.37948*	125	30	71	108	35	-1.644	0.277648394	1
ENSBTAG00000012865	*DEK*	13'538	8'352	10'684	11'724	9'644	-0.282	0.71303549	1
ENSBTAG00000012866	*THBS4*	1'601	802	1'156	1'387	926	-0.582	0.476901276	1
ENSBTAG00000012867	*TXLNA*	1'594	2'540	2'157	1'380	2'933	1.087	0.172236173	1
ENSBTAG00000012868	*ZNF77*	472	430	453	409	497	0.281	0.755896447	1
ENSBTAG00000012873	*BT.65854*	1'730	1'181	1'431	1'498	1'364	-0.136	0.867469655	1
ENSBTAG00000012876	*processed_pseudogene*	1'260	1'460	1'389	1'091	1'686	0.628	0.437115928	1
ENSBTAG00000012878	*BMS1*	2'735	2'067	2'378	2'369	2'387	0.011	0.988727349	1
ENSBTAG00000012880	*DNLZ*	454	511	492	393	590	0.586	0.510582657	1

Ensembl gene ID	geneName	counts wildtype horn bud	counts polled horn bud	baseMean	baseMean wildtype horn bud	baseMean polled horn bud	log2FoldChange	pval	padj
ENSBTAG00000012881	*EIF4G1*	13'899	17'470	16'105	12'037	20'173	0.745	0.33012542	1
ENSBTAG00000012882	*BT.58905*	722	444	569	625	513	-0.286	0.744525659	1
ENSBTAG00000012885	*ACAT1*	6'280	4'418	5'270	5'439	5'101	-0.092	0.905511126	1
ENSBTAG00000012887	*FCER1A*	183	116	146	158	134	-0.243	0.838850292	1
ENSBTAG00000012888	*C2ORF80*	6	1	3	5	1	-2.170	0.885240772	1
ENSBTAG00000012889	*NETO1*	57	1	25	49	1	-5.418	0.086303335	1
ENSBTAG00000012890	*SLC25A3*	7'448	5'752	6'546	6'450	6'642	0.042	0.955653381	1
ENSBTAG00000012892	*C17H22orf31*	38	48	44	33	55	0.752	0.686533501	1
ENSBTAG00000012894	*SAMD9*	34	40	38	29	46	0.650	0.749299661	1
ENSBTAG00000012896	*METTL7B*	0	1	1	0	1	Inf	0.993540919	1
ENSBTAG00000012897	*ITGA7*	3'483	2'422	2'907	3'016	2'797	-0.109	0.889973923	1
ENSBTAG00000012898	*RPS27L*	519	272	382	449	314	-0.517	0.577703698	1
ENSBTAG00000012899	*IFNGR2*	3'284	3'254	3'301	2'844	3'757	0.402	0.6055938	1
ENSBTAG00000012900	*TMEM229B*	3'029	1'657	2'268	2'623	1'913	-0.455	0.564455443	1
ENSBTAG00000012902	*BT.95450*	1'880	1'045	1'417	1'628	1'207	-0.432	0.592644061	1
ENSBTAG00000012904	*BT.55540*	1'132	724	908	980	836	-0.230	0.783689923	1
ENSBTAG00000012908	*HSPA13*	2'019	1'662	1'834	1'749	1'919	0.134	0.865632712	1
ENSBTAG00000012909	*CRABP1*	17'641	17'878	17'961	15'278	20'644	0.434	0.56844814	1
ENSBTAG00000012912	*BT.74143*	9	0	4	8	0		0.690676647	1
ENSBTAG00000012914	*BT.62809*	19	5	11	16	6	-1.511	0.7314974	1
ENSBTAG00000012915	*BT.62017*	312	62	171	270	72	-1.916	0.091152831	1
ENSBTAG00000012920	*TMEM70*	1'363	830	1'069	1'180	958	-0.301	0.715328181	1
ENSBTAG00000012921	*BT.26923*	294	273	285	255	315	0.308	0.753514212	1
ENSBTAG00000012922	*PHF2*	4'919	5'116	5'084	4'260	5'907	0.472	0.540902304	1
ENSBTAG00000012925	*BT.52849*	779	749	770	675	865	0.358	0.671058752	1
ENSBTAG00000012926	*BT.91422*	965	429	666	836	495	-0.755	0.380582377	1
ENSBTAG00000012927	*ALDOA*	12'520	18'540	16'125	10'843	21'408	0.981	0.201229015	1
ENSBTAG00000012928	*PPP4C*	4'623	4'114	4'377	4'004	4'750	0.247	0.749220003	1
ENSBTAG00000012929	*CEP85L*	174	98	132	151	113	-0.413	0.73291831	1
ENSBTAG00000012931	*BT.43673*	119	51	81	103	59	-0.807	0.571917208	1
ENSBTAG00000012936	*THYN1*	1'865	1'773	1'831	1'615	2'047	0.342	0.666695061	1
ENSBTAG00000012937	*BT.36400*	3'693	1'653	2'553	3'198	1'909	-0.745	0.344817922	1
ENSBTAG00000012938	*JARID2*	2'270	1'511	1'855	1'966	1'745	-0.172	0.829556356	1
ENSBTAG00000012939	*BT.38575*	929	580	737	805	670	-0.265	0.756461796	1
ENSBTAG00000012940	*TMEM25*	1'538	891	1'180	1'332	1'029	-0.373	0.648618119	1
ENSBTAG00000012941	*IFT46*	1'728	1'273	1'483	1'496	1'470	-0.026	0.975975752	1
ENSBTAG00000012943	*PPARGC1B*	49	174	122	42	201	2.243	0.075980127	1
ENSBTAG00000012944	*USP35*	281	420	364	243	485	0.995	0.288096774	1
ENSBTAG00000012945	*PDE6A*	19	2	9	16	2	-2.833	0.582174212	1
ENSBTAG00000012946	*HSF2*	2'206	1'084	1'581	1'910	1'252	-0.610	0.447661325	1
ENSBTAG00000012949	*FOV*	1	0	0	1	0		1	1
ENSBTAG00000012953	*ASTN1*	112	17	58	97	20	-2.305	0.171430834	1
ENSBTAG00000012954	*ERAS*	218	144	178	189	166	-0.183	0.871963958	1
ENSBTAG00000012955	*BT.57021*	129	75	99	112	87	-0.367	0.78517519	1
ENSBTAG00000012957	*SERBP1*	14'312	15'836	15'340	12'395	18'286	0.561	0.462311986	1
ENSBTAG00000012961	*PAPSS1*	7'442	5'893	6'625	6'445	6'805	0.078	0.918230435	1
ENSBTAG00000012962	*SARS*	5'227	3'989	4'566	4'527	4'606	0.025	0.97372743	1
ENSBTAG00000012963	*VILL*	23	81	57	20	94	2.231	0.186137338	1
ENSBTAG00000012966	*BT.25320*	244	259	255	211	299	0.501	0.616602431	1
ENSBTAG00000012968	*FLJ10803*	1'215	756	963	1'052	873	-0.269	0.745944847	1
ENSBTAG00000012969	*DKK2*	1'970	516	1'151	1'706	596	-1.518	0.067988175	1
ENSBTAG00000012970	*FAM53B*	1'091	1'158	1'141	945	1'337	0.501	0.539648836	1
ENSBTAG00000012972	*BT.56804*	1'623	1'355	1'485	1'406	1'565	0.155	0.847235047	1
ENSBTAG00000012973	*GFRAL*	6	0	3	5	0		0.81115604	1
ENSBTAG00000012975	*VDAC2*	2'309	2'652	2'531	2'000	3'062	0.615	0.434086686	1
ENSBTAG00000012976	*WLS*	6'785	7'497	7'266	5'876	8'657	0.559	0.466791995	1
ENSBTAG00000012977	*COMTD1*	408	472	449	353	545	0.625	0.488192564	1
ENSBTAG00000012979	*ZMYND12*	18	3	10	16	3	-2.170	0.662488565	1
ENSBTAG00000012980	*BT.33961*	811	450	611	702	520	-0.435	0.616690851	1
ENSBTAG00000012981	*HEXA*	2'714	2'459	2'595	2'350	2'839	0.273	0.727622988	1
ENSBTAG00000012982	*PPCS*	823	422	600	713	487	-0.549	0.528308262	1
ENSBTAG00000012984	*BT.87862*	78	31	52	68	36	-0.916	0.5973151	1
ENSBTAG00000012985	*FUK*	2'185	1'844	2'011	1'892	2'129	0.170	0.829452879	1
ENSBTAG00000012987	*ULK1*	2'211	3'589	3'030	1'915	4'144	1.114	0.157471564	1
ENSBTAG00000012988	*COG4*	2'497	2'443	2'492	2'162	2'821	0.383	0.625024839	1
ENSBTAG00000012989	*UBE2L6*	696	752	736	603	868	0.527	0.534549956	1
ENSBTAG00000012990	*PUS1*	2'303	1'583	1'911	1'994	1'828	-0.126	0.875187206	1
ENSBTAG00000012991	*PRUNE2*	758	199	443	656	230	-1.514	0.099404467	1
ENSBTAG00000012994	*LOX*	17'592	11'061	14'004	15'235	12'772	-0.254	0.739382802	1
ENSBTAG00000012995	*CCDC109B*	1'206	1'192	1'210	1'044	1'376	0.398	0.624323008	1
ENSBTAG00000012996	*RNF11*	2'344	1'439	1'846	2'030	1'662	-0.289	0.717173643	1
ENSBTAG00000012997	*SMPDL3B*	34	17	25	29	20	-0.585	0.828099259	1

Ensembl gene ID	geneName	counts wildtype horn bud	counts polled horn bud	baseMean	baseMean wildtype horn bud	baseMean polled horn bud	log2FoldChange	pval	padj
ENSBTAG00000012998	*NAA30*	518	244	365	449	282	-0.671	0.473215562	1
ENSBTAG00000012999	*BT.105664*	1'039	770	894	900	889	-0.017	0.985733443	1
ENSBTAG00000013001	*protein_coding*	806	383	570	698	442	-0.658	0.45212465	1
ENSBTAG00000013002	*GABRQ*	28	10	18	24	12	-1.070	0.735005286	1
ENSBTAG00000013004	*BT.43334*	1'332	1'079	1'200	1'154	1'246	0.111	0.89155132	1
ENSBTAG00000013007	*C13H20ORF108*	1'998	994	1'439	1'730	1'148	-0.592	0.463295875	1
ENSBTAG00000013008	*PMCH*	107	51	76	93	59	-0.654	0.657013061	1
ENSBTAG00000013009	*AURKA*	447	398	423	387	460	0.248	0.786339196	1
ENSBTAG00000013010	*CCDC126*	672	295	461	582	341	-0.773	0.391504956	1
ENSBTAG00000013011	*PLAA*	1'916	1'416	1'647	1'659	1'635	-0.021	0.980339306	1
ENSBTAG00000013013	*GMPS*	2'058	1'893	1'984	1'782	2'186	0.294	0.709742814	1
ENSBTAG00000013014	*BT.25609*	17	1	8	15	1	-3.672	0.555170792	1
ENSBTAG00000013015	*CCDC13*	18	24	22	16	28	0.830	0.765788213	1
ENSBTAG00000013016	*GNAI3*	5'766	4'448	5'065	4'994	5'136	0.041	0.957567016	1
ENSBTAG00000013017	*GNAT2*	167	115	139	145	133	-0.123	0.923242199	1
ENSBTAG00000013018	*AMPD2*	2'890	2'993	2'979	2'503	3'456	0.466	0.550997167	1
ENSBTAG00000013020	*pseudogene*	32	21	26	28	24	-0.193	0.954356549	1
ENSBTAG00000013023	*MAP4K4*	5'369	4'953	5'184	4'650	5'719	0.299	0.698021079	1
ENSBTAG00000013025	*HAX1*	2'385	1'301	1'784	2'065	1'502	-0.459	0.564930991	1
ENSBTAG00000013027	*CCK*	85	12	44	74	14	-2.409	0.212307122	1
ENSBTAG00000013029	*ARNTL*	718	484	590	622	559	-0.154	0.861621796	1
ENSBTAG00000013030	*LLRC8A*	2'095	2'486	2'342	1'814	2'871	0.662	0.401273848	1
ENSBTAG00000013032	*BT.41048*	3'165	1'962	2'503	2'741	2'266	-0.275	0.727157922	1
ENSBTAG00000013033	*BTBD10*	2'720	1'801	2'218	2'356	2'080	-0.180	0.820677227	1
ENSBTAG00000013036	*YDJC*	171	116	141	148	134	-0.145	0.907792484	1
ENSBTAG00000013038	*BT.34832*	3'087	2'588	2'831	2'673	2'988	0.161	0.836810914	1
ENSBTAG00000013042	*EXOC5*	4'546	2'048	3'151	3'937	2'365	-0.735	0.347774681	1
ENSBTAG00000013043	*AP5M1*	362	298	329	314	344	0.134	0.889520778	1
ENSBTAG00000013044	*GAB3*	142	41	85	123	47	-1.377	0.32688897	1
ENSBTAG00000013045	*DKC1*	4'315	3'594	3'943	3'737	4'150	0.151	0.844810211	1
ENSBTAG00000013046	*MPP1*	1'719	2'206	2'018	1'489	2'547	0.775	0.329307407	1
ENSBTAG00000013047	*GRM7*	118	9	56	102	10	-3.298	0.065990796	1
ENSBTAG00000013048	*NIPAL3*	233	251	246	202	290	0.522	0.604691673	1
ENSBTAG00000013050	*BT.65232*	4'294	1'526	2'740	3'719	1'762	-1.078	0.172756808	1
ENSBTAG00000013052	*SDR42E2*	22	2	11	19	2	-3.044	0.517475779	1
ENSBTAG00000013054	*MFSD2*	268	84	165	232	97	-1.259	0.264006359	1
ENSBTAG00000013056	*SPAG4L*	1	0	0	1	0		1	1
ENSBTAG00000013057	*CBLB*	400	438	426	346	506	0.546	0.548086967	1
ENSBTAG00000013059	*pseudogene*	2	1	1	2	1	-0.585	1	1
ENSBTAG00000013060	*IQGAP1*	17'530	15'201	16'367	15'181	17'553	0.209	0.782855062	1
ENSBTAG00000013063	*ITGB1BP1*	1'666	1'213	1'422	1'443	1'401	-0.043	0.959262348	1
ENSBTAG00000013064	*RGS18*	5	4	4	4	5	0.093	1	1
ENSBTAG00000013065	*BT.37142*	275	260	269	238	300	0.334	0.736370147	1
ENSBTAG00000013066	*IGF2*	329'307	177'204	244'903	285'188	204'618	-0.479	0.528576082	1
ENSBTAG00000013068	*GGPS1*	1'139	808	960	986	933	-0.080	0.924598341	1
ENSBTAG00000013069	*IQCB1*	1'133	692	890	981	799	-0.296	0.723313464	1
ENSBTAG00000013070	*EAF2*	101	44	69	87	51	-0.784	0.6073699	1
ENSBTAG00000013072	*FUBP3*	3'517	1'972	2'661	3'046	2'277	-0.420	0.593193922	1
ENSBTAG00000013074	*PPP1R15B*	1'685	813	1'199	1'459	939	-0.636	0.435927418	1
ENSBTAG00000013076	*SNX19*	1'624	3'257	2'584	1'406	3'761	1.419	0.075081325	1
ENSBTAG00000013078	*DNAH11*	160	161	162	139	186	0.424	0.706556711	1
ENSBTAG00000013081	*PSPH*	516	194	335	447	224	-0.996	0.295536144	1
ENSBTAG00000013084	*NAPA*	2'290	2'935	2'686	1'983	3'389	0.773	0.325441876	1
ENSBTAG00000013085	*BT.87229*	1	5	3	1	6	2.737	0.836148221	1
ENSBTAG00000013090	*BT.88746*	5	1	3	4	1	-1.907	0.9188325	1
ENSBTAG00000013093	*ALDH3B1*	462	185	307	400	214	-0.905	0.349388382	1
ENSBTAG00000013095	*FOXN1*	124	248	197	107	286	1.415	0.187826497	1
ENSBTAG00000013096	*UNC119*	1'635	1'729	1'706	1'416	1'996	0.496	0.534371518	1
ENSBTAG00000013098	*BT.49068*	4'399	3'751	4'070	3'810	4'331	0.185	0.810612478	1
ENSBTAG00000013099	*ALDOC*	1'510	489	936	1'308	565	-1.212	0.14856908	1
ENSBTAG00000013100	*SPAG5*	1'164	1'174	1'182	1'008	1'356	0.427	0.599786829	1
ENSBTAG00000013103	*COL1A1*	4'794'217	3'573'821	4'139'303	4'151'914	4'126'693	-0.009	0.991416729	1
ENSBTAG00000013105	*SYT3*	125	42	78	108	48	-1.158	0.42323836	1
ENSBTAG00000013106	*C19ORF81*	5	1	3	4	1	-1.907	0.9188325	1
ENSBTAG00000013107	*SHANK1*	653	987	853	566	1'140	1.011	0.229435925	1
ENSBTAG00000013108	*HK2*	394	260	321	341	300	-0.185	0.849843006	1
ENSBTAG00000013109	*BT.31041*	1'576	2'154	1'926	1'365	2'487	0.866	0.277346225	1
ENSBTAG00000013110	*FARP2*	320	260	289	277	300	0.115	0.908142789	1
ENSBTAG00000013111	*RRM1*	4'940	4'100	4'506	4'278	4'734	0.146	0.849541314	1
ENSBTAG00000013112	*C7H5ORF15*	6'727	6'196	6'490	5'826	7'155	0.296	0.699202438	1
ENSBTAG00000013113	*VDAC1*	6'950	6'048	6'501	6'019	6'984	0.214	0.779574932	1
ENSBTAG00000013114	*ZMYND8*	4'105	1'965	2'912	3'555	2'269	-0.648	0.408793902	1

Ensembl gene ID	geneName	counts wildtype horn bud	counts polled horn bud	baseMean	baseMean wildtype horn bud	baseMean polled horn bud	log2FoldChange	pval	padj
ENSBTAG00000013116	*BT.4552*	860	197	486	745	227	-1.711	0.060550164	1
ENSBTAG00000013117	*CACNA2D3*	519	92	278	449	106	-2.081	0.040395707	1
ENSBTAG00000013118	*CD86*	784	457	603	679	528	-0.364	0.676211262	1
ENSBTAG00000013120	*C10ORF76*	657	734	708	569	848	0.575	0.499595987	1
ENSBTAG00000013123	*PHB2*	3'013	4'289	3'781	2'609	4'953	0.924	0.236421366	1
ENSBTAG00000013124	*LRTM1*	2	9	6	2	10	2.585	0.716971424	1
ENSBTAG00000013125	*PLAUR*	812	374	568	703	432	-0.703	0.422196043	1
ENSBTAG00000013126	*BT.26573*	1'247	1'101	1'176	1'080	1'271	0.235	0.772618306	1
ENSBTAG00000013127	*BT.106554*	544	643	607	471	742	0.656	0.449030468	1
ENSBTAG00000013128	*ACBD3*	5'555	2'897	4'078	4'811	3'345	-0.524	0.499851844	1
ENSBTAG00000013131	*FAM110D*	221	59	130	191	68	-1.490	0.220208683	1
ENSBTAG00000013132	*MTX3*	41	45	44	36	52	0.549	0.772261136	1
ENSBTAG00000013133	*TBC1D10C*	37	27	32	32	31	-0.040	1	1
ENSBTAG00000013134	*SOST*	249	71	149	216	82	-1.395	0.230341942	1
ENSBTAG00000013136	*EFCAB11*	230	133	176	199	154	-0.375	0.735232167	1
ENSBTAG00000013138	*TDP1*	393	439	424	340	507	0.575	0.527610686	1
ENSBTAG00000013141	*ARPM1*	244	165	201	211	191	-0.149	0.89270294	1
ENSBTAG00000013142	*MYNN*	1'641	1'147	1'373	1'421	1'324	-0.102	0.901219801	1
ENSBTAG00000013143	*BT.27376*	597	526	562	517	607	0.232	0.790863732	1
ENSBTAG00000013145	*BT.105310*	4'209	2'348	3'178	3'645	2'711	-0.427	0.584732809	1
ENSBTAG00000013147	*NEFH*	521	995	800	451	1'149	1.348	0.113482869	1
ENSBTAG00000013148	*CLDN1*	1'415	2'000	1'767	1'225	2'309	0.914	0.25354114	1
ENSBTAG00000013150	*THOC5*	2'940	3'113	3'070	2'546	3'595	0.498	0.523765294	1
ENSBTAG00000013152	*BT.103053*	2'711	2'191	2'439	2'348	2'530	0.108	0.890609473	1
ENSBTAG00000013153	*NF2*	1'131	1'798	1'528	979	2'076	1.084	0.180325622	1
ENSBTAG00000013155	*COL2A1*	3'442	1'986	2'637	2'981	2'293	-0.378	0.630180134	1
ENSBTAG00000013157	*NOVA2*	165	54	103	143	62	-1.196	0.360259822	1
ENSBTAG00000013159	*PHF11*	2'907	1'677	2'227	2'518	1'936	-0.379	0.632009142	1
ENSBTAG00000013160	*GFRA4*	371	316	343	321	365	0.184	0.847143493	1
ENSBTAG00000013162	*HSPA8*	64'381	61'205	63'215	55'756	70'673	0.342	0.651761704	1
ENSBTAG00000013163	*ADAM33*	12'147	18'910	16'177	10'520	21'835	1.054	0.170635631	1
ENSBTAG00000013165	*ENPP2*	8'063	6'379	7'174	6'983	7'366	0.077	0.919476789	1
ENSBTAG00000013166	*SMYD2*	371	168	258	321	194	-0.728	0.467507544	1
ENSBTAG00000013167	*SIGLEC1*	313	633	501	271	731	1.431	0.111808159	1
ENSBTAG00000013168	*RPA3*	2'494	1'584	1'994	2'160	1'829	-0.240	0.762964997	1
ENSBTAG00000013169	*LCMT1*	1'978	1'943	1'978	1'713	2'244	0.389	0.62293547	1
ENSBTAG00000013173	*Sep 03*	1'927	817	1'306	1'669	943	-0.823	0.311934313	1
ENSBTAG00000013175	*KIAA0355*	1'654	1'113	1'359	1'432	1'285	-0.156	0.847693689	1
ENSBTAG00000013176	*BT.63041*	2'764	794	1'655	2'394	917	-1.385	0.087953577	1
ENSBTAG00000013177	*APEX2*	921	643	770	798	742	-0.103	0.90463842	1
ENSBTAG00000013178	*ALAS2*	138	137	139	120	158	0.405	0.732254953	1
ENSBTAG00000013180	*POT1*	783	717	753	678	828	0.288	0.733548559	1
ENSBTAG00000013181	*C14ORF166B*	1	0	0	1	0		1	1
ENSBTAG00000013184	*CEP120*	4'504	1'993	3'101	3'901	2'301	-0.761	0.331423652	1
ENSBTAG00000013185	*TIMD4*	124	130	129	107	150	0.483	0.689105454	1
ENSBTAG00000013187	*DLG5*	7'808	12'916	10'838	6'762	14'914	1.141	0.139546009	1
ENSBTAG00000013191	*AGRN*	9'586	18'723	14'961	8'302	21'619	1.381	0.074717783	1
ENSBTAG00000013192	*CEP19*	660	349	487	572	403	-0.504	0.572841869	1
ENSBTAG00000013196	*BT.45655*	360	153	244	312	177	-0.819	0.419138601	1
ENSBTAG00000013197	*PSEN2*	906	643	764	785	742	-0.080	0.927081727	1
ENSBTAG00000013198	*C4ORF21*	1'107	547	795	959	632	-0.602	0.475738017	1
ENSBTAG00000013201	*ALOX5AP*	238	119	172	206	137	-0.585	0.598100447	1
ENSBTAG00000013202	*PLCZ1*	1	0	0	1	0		1	1
ENSBTAG00000013203	*GP5*	49	12	28	42	14	-1.615	0.496582696	1
ENSBTAG00000013204	*BT.20559*	1'544	534	977	1'337	617	-1.117	0.180735646	1
ENSBTAG00000013205	*BT.28708*	258	243	252	223	281	0.329	0.744280977	1
ENSBTAG00000013208	*SLC25A4*	1'757	845	1'249	1'522	976	-0.641	0.431354544	1
ENSBTAG00000013210	*ADAMTS4*	2'882	3'049	3'008	2'496	3'521	0.496	0.525021964	1
ENSBTAG00000013211	*BT.52665*	2'723	1'864	2'255	2'358	2'152	-0.132	0.868303842	1
ENSBTAG00000013212	*COPG1*	12'037	16'290	14'617	10'424	18'810	0.852	0.26666745	1
ENSBTAG00000013215	*MREG*	150	135	143	130	156	0.263	0.824383387	1
ENSBTAG00000013218	*GORASP2*	7'002	6'504	6'787	6'064	7'510	0.309	0.687333443	1
ENSBTAG00000013219	*PEX5L*	27	3	13	23	3	-2.755	0.482187496	1
ENSBTAG00000013220	*R3HDML*	0	4	2	0	5	Inf	0.817359038	1
ENSBTAG00000013221	*RTTN*	809	488	632	701	563	-0.314	0.716948317	1
ENSBTAG00000013222	*CD109*	5'077	2'976	3'917	4'397	3'436	-0.356	0.647358019	1
ENSBTAG00000013224	*BT.52934*	739	278	480	640	321	-0.995	0.268061862	1
ENSBTAG00000013225	*NBN*	3'023	1'033	1'905	2'618	1'193	-1.134	0.157265171	1
ENSBTAG00000013226	*BT.43192*	799	568	674	692	656	-0.077	0.930469229	1
ENSBTAG00000013227	*SNAI2*	5'471	4'131	4'754	4'738	4'770	0.010	0.989579363	1
ENSBTAG00000013231	*C9ORF40*	586	361	462	507	417	-0.284	0.753837059	1
ENSBTAG00000013234	*BT.62522*	3	0	1	3	0		0.936647693	1

Ensembl gene ID	geneName	counts wildtype horn bud	counts polled horn bud	baseMean	baseMean wildtype horn bud	baseMean polled horn bud	log2FoldChange	pval	padj
ENSBTAG00000013235	TINAGL1	774	708	744	670	818	0.286	0.735246614	1
ENSBTAG00000013236	MED1	1'501	1'943	1'772	1'300	2'244	0.787	0.324412353	1
ENSBTAG00000013238	CDK12	1'229	1'185	1'216	1'064	1'368	0.362	0.655619874	1
ENSBTAG00000013239	CAPN12	30	17	23	26	20	-0.404	0.89272818	1
ENSBTAG00000013240	SLC3A2	3'696	3'968	3'891	3'201	4'582	0.517	0.504803588	1
ENSBTAG00000013241	BSND	6	5	5	5	6	0.152	1	1
ENSBTAG00000013242	TMEM8C	25	93	65	22	107	2.310	0.149256673	1
ENSBTAG00000013244	BT.76413	4'842	4'521	4'707	4'193	5'220	0.316	0.681958484	1
ENSBTAG00000013245	ITPR3	3'266	6'366	5'090	2'828	7'351	1.378	0.078669016	1
ENSBTAG00000013247	BT.36110	11	11	11	10	13	0.415	0.940352837	1
ENSBTAG00000013248	protein_coding	1	0	0	1	0		1	1
ENSBTAG00000013249	SALL2	1'596	1'551	1'587	1'382	1'791	0.374	0.640340025	1
ENSBTAG00000013251	HDGFRP2	4'236	4'805	4'608	3'668	5'548	0.597	0.440559452	1
ENSBTAG00000013253	BT.21910	1'093	1'435	1'302	947	1'657	0.808	0.320180043	1
ENSBTAG00000013254	XPO1	14'372	7'840	10'750	12'447	9'053	-0.459	0.548861627	1
ENSBTAG00000013256	LGR5	1'013	678	830	877	783	-0.164	0.846367317	1
ENSBTAG00000013259	POLR3A	5'021	3'346	4'106	4'348	3'864	-0.170	0.826591965	1
ENSBTAG00000013260	SPIN2	614	284	430	532	328	-0.697	0.443986796	1
ENSBTAG00000013263	HOXA1	23	42	34	20	48	1.284	0.542352263	1
ENSBTAG00000013264	RPS24	104'486	46'451	72'062	90'488	53'637	-0.754	0.322514217	1
ENSBTAG00000013265	BT.42896	1'813	2'026	1'955	1'570	2'339	0.575	0.468410913	1
ENSBTAG00000013266	PMFBP1	6	2	4	5	2	-1.170	0.934316631	1
ENSBTAG00000013270	TTC19	1'301	935	1'103	1'127	1'080	-0.062	0.941889171	1
ENSBTAG00000013271	NCOR1	4'719	4'077	4'397	4'087	4'708	0.204	0.791381554	1
ENSBTAG00000013273	PIGL	911	978	959	789	1'129	0.517	0.531900097	1
ENSBTAG00000013274	CENPV	919	940	941	796	1'085	0.448	0.589094323	1
ENSBTAG00000013275	BT.30106	1'064	1'469	1'309	921	1'696	0.880	0.278993861	1
ENSBTAG00000013277	CCDC22	1'028	1'641	1'393	890	1'895	1.090	0.180224147	1
ENSBTAG00000013278	MAGIX	78	101	92	68	117	0.788	0.559754198	1
ENSBTAG00000013279	FOXP3	45	31	37	39	36	-0.123	0.966636791	1
ENSBTAG00000013281	SPATA21	96	100	99	83	115	0.474	0.720331896	1
ENSBTAG00000013282	NECAP2	2'799	2'545	2'681	2'424	2'939	0.278	0.722452977	1
ENSBTAG00000013283	PRR19	169	123	144	146	142	-0.043	0.977762674	1
ENSBTAG00000013284	SGK3	1'103	586	816	955	677	-0.497	0.554642319	1
ENSBTAG00000013287	SMO	8'478	12'006	10'603	7'342	13'863	0.917	0.233225502	1
ENSBTAG00000013288	SUGP2	4'582	3'626	4'078	3'968	4'187	0.077	0.919951991	1
ENSBTAG00000013289	PHF8	1'228	1'033	1'128	1'063	1'193	0.166	0.839542732	1
ENSBTAG00000013290	DYSF	2'121	2'050	2'102	1'837	2'367	0.366	0.643055266	1
ENSBTAG00000013291	ARMC6	611	735	689	529	849	0.682	0.425248087	1
ENSBTAG00000013292	SIDT1	43	7	23	37	8	-2.204	0.419659754	1
ENSBTAG00000013294	BT.45227	377	265	316	326	306	-0.094	0.926124121	1
ENSBTAG00000013298	PLA2G4A	2'347	1'191	1'704	2'033	1'375	-0.564	0.4811889	1
ENSBTAG00000013300	KCNMA1	945	442	664	818	510	-0.681	0.428390311	1
ENSBTAG00000013302	R3HDM1	1'409	808	1'077	1'220	933	-0.387	0.637950136	1
ENSBTAG00000013303	ACSS2	501	647	590	434	747	0.784	0.367973046	1
ENSBTAG00000013305	BT.99178	0	2	1	0	2	Inf	0.939077559	1
ENSBTAG00000013306	ZBTB7C	344	187	257	298	216	-0.464	0.643959803	1
ENSBTAG00000013308	RBMX	7'886	8'245	8'175	6'829	9'521	0.479	0.531755262	1
ENSBTAG00000013309	SFRS4	10'304	5'180	7'452	8'924	5'981	-0.577	0.453278192	1
ENSBTAG00000013311	processed_pseudogene	597	515	556	517	595	0.202	0.818251809	1
ENSBTAG00000013314	BT.74403	937	267	560	811	308	-1.396	0.115711944	1
ENSBTAG00000013315	ATP5B	17'888	20'874	19'797	15'491	24'103	0.638	0.403235409	1
ENSBTAG00000013316	BRPF3	1'380	1'252	1'320	1'195	1'446	0.275	0.734047514	1
ENSBTAG00000013317	BT.89177	1'509	914	1'181	1'307	1'055	-0.308	0.706277464	1
ENSBTAG00000013319	BT.60977	4	4	4	3	5	0.415	0.994759363	1
ENSBTAG00000013320	TSPAN1	16	29	24	14	33	1.273	0.623253784	1
ENSBTAG00000013322	POMGNT1	3'146	4'160	3'764	2'725	4'804	0.818	0.293967927	1
ENSBTAG00000013326	ANAPC10	363	259	307	314	299	-0.072	0.944643997	1
ENSBTAG00000013329	AP1M1	2'959	3'796	3'473	2'563	4'383	0.774	0.321119906	1
ENSBTAG00000013330	TBC1D20	4'672	3'164	3'850	4'046	3'653	-0.147	0.850293849	1
ENSBTAG00000013333	GYS2	5	0	2	4	0		0.853268594	1
ENSBTAG00000013334	CSF3R	36	17	25	31	20	-0.667	0.797360051	1
ENSBTAG00000013336	EYA2	318	105	198	275	121	-1.184	0.268949791	1
ENSBTAG00000013337	DHDH	276	247	262	239	285	0.255	0.799263546	1
ENSBTAG00000013338	SLC7A9	3	4	4	3	5	0.830	0.962490925	1
ENSBTAG00000013339	NEBL	202	91	140	175	105	-0.735	0.532109517	1
ENSBTAG00000013340	BAX	759	658	709	657	760	0.209	0.806335805	1
ENSBTAG00000013341	GYG2	961	1'362	1'202	832	1'573	0.918	0.261727617	1
ENSBTAG00000013343	FTL	6'537	8'224	7'579	5'661	9'496	0.746	0.332274579	1
ENSBTAG00000013346	BT.26127	2'102	2'849	2'555	1'820	3'290	0.854	0.278860163	1
ENSBTAG00000013347	DMPK	1'228	844	1'019	1'063	975	-0.126	0.880118051	1
ENSBTAG00000013353	ZNF274	396	174	272	343	201	-0.771	0.436091847	1

Ensembl gene ID	geneName	counts wildtype horn bud	counts polled horn bud	baseMean	baseMean wildtype horn bud	baseMean polled horn bud	log2FoldChange	pval	padj
ENSBTAG00000013356	*CATHL1*	2	0	1	2	0		0.974934741	1
ENSBTAG00000013357	*KIAA0020*	1'235	915	1'063	1'070	1'057	-0.018	0.984877142	1
ENSBTAG00000013362	*BT.18482*	4'209	5'085	4'758	3'645	5'872	0.688	0.374409419	1
ENSBTAG00000013363	*CAP1*	5'921	5'293	5'620	5'128	6'112	0.253	0.741690687	1
ENSBTAG00000013367	*BT.101768*	8'528	5'649	6'954	7'385	6'523	-0.179	0.816181482	1
ENSBTAG00000013368	*ANKRD22*	66	18	39	57	21	-1.459	0.463542229	1
ENSBTAG00000013369	*COL14A1*	51'517	36'590	43'433	44'615	42'250	-0.079	0.918256545	1
ENSBTAG00000013371	*KIAA0226L*	469	441	458	406	509	0.326	0.717056326	1
ENSBTAG00000013378	*AADACL2*	12	2	6	10	2	-2.170	0.758651977	1
ENSBTAG00000013380	*CEP192*	2'013	1'243	1'589	1'743	1'435	-0.280	0.727055152	1
ENSBTAG00000013387	*CHMP4B*	3'228	2'041	2'576	2'796	2'357	-0.246	0.754359614	1
ENSBTAG00000013390	*PSMB6*	3'437	3'128	3'294	2'977	3'612	0.279	0.719533473	1
ENSBTAG00000013392	*PLD2*	4'317	2'962	3'579	3'739	3'420	-0.128	0.869632693	1
ENSBTAG00000013393	*GEMIN4*	992	1'045	1'033	859	1'207	0.490	0.551371096	1
ENSBTAG00000013395	*DBIL5*	6	24	16	5	28	2.415	0.464285575	1
ENSBTAG00000013401	*ARHGEF40*	7'509	11'303	9'777	6'503	13'052	1.005	0.192376304	1
ENSBTAG00000013402	*CTDSPL*	488	642	582	423	741	0.811	0.35286513	1
ENSBTAG00000013403	*AGBL5*	671	574	622	581	663	0.190	0.826596327	1
ENSBTAG00000013405	*FAM92A1*	889	777	834	770	897	0.221	0.792271485	1
ENSBTAG00000013406	*CSRP2*	1'231	559	856	1'066	645	-0.724	0.388298905	1
ENSBTAG00000013407	*PLXNB1*	2'424	5'242	4'076	2'099	6'053	1.528	0.053087684	1
ENSBTAG00000013408	*RBM12B*	1'796	1'229	1'487	1'555	1'419	-0.132	0.870551808	1
ENSBTAG00000013410	*MARK3*	3'532	2'517	2'983	3'059	2'906	-0.074	0.925817829	1
ENSBTAG00000013411	*BT.22783*	19'280	26'961	23'914	16'697	31'132	0.899	0.240289784	1
ENSBTAG00000013412	*NFAT5*	175	70	116	152	81	-0.907	0.46809843	1
ENSBTAG00000013413	*CCDC51*	1'119	931	1'022	969	1'075	0.150	0.855934053	1
ENSBTAG00000013414	*BLVRA*	1'036	566	775	897	654	-0.457	0.589042646	1
ENSBTAG00000013416	*BT.36448*	4	0	2	3	0		0.89545886	1
ENSBTAG00000013419	*HTATIP2*	145	71	104	126	82	-0.615	0.637264738	1
ENSBTAG00000013420	*FSD1L*	70	49	59	61	57	-0.100	0.963299849	1
ENSBTAG00000013421	*PRMT3*	1'170	928	1'042	1'013	1'072	0.081	0.922172122	1
ENSBTAG00000013423	*GLRX5*	1'001	1'140	1'092	867	1'316	0.603	0.46247287	1
ENSBTAG00000013425	*BT.37059*	743	715	735	643	826	0.360	0.671457877	1
ENSBTAG00000013426	*C5ORF35*	388	229	300	336	264	-0.346	0.722994728	1
ENSBTAG00000013429	*CLEC4G*	21	12	16	18	14	-0.392	0.922971022	1
ENSBTAG00000013436	*HAUS5*	6'136	4'201	5'082	5'314	4'851	-0.132	0.865417514	1
ENSBTAG00000013439	*ARHGEF26*	1'191	1'110	1'157	1'031	1'282	0.313	0.70071142	1
ENSBTAG00000013440	*BT.87291*	1'641	2'048	1'893	1'421	2'365	0.735	0.356069975	1
ENSBTAG00000013442	*GPR62*	12	15	14	10	17	0.737	0.84931155	1
ENSBTAG00000013443	*SDR9C7*	83	78	81	72	90	0.325	0.823222396	1
ENSBTAG00000013444	*BT.47419*	164	123	142	142	142	0.000	1	1
ENSBTAG00000013449	*RAB3GAP1*	4'974	4'045	4'489	4'308	4'671	0.117	0.879458718	1
ENSBTAG00000013451	*BT.53393*	29	17	22	25	20	-0.355	0.909573651	1
ENSBTAG00000013452	*BT.19592*	3	6	5	3	7	1.415	0.880191876	1
ENSBTAG00000013454	*TAB2*	4'002	2'476	3'162	3'466	2'859	-0.278	0.722488137	1
ENSBTAG00000013455	*SPOPL*	324	202	257	281	233	-0.267	0.79266641	1
ENSBTAG00000013456	*TMEM207*	5	0	2	4	0		0.853268594	1
ENSBTAG00000013460	*ZBTB11*	1'935	1'128	1'489	1'676	1'303	-0.364	0.651867748	1
ENSBTAG00000013461	*RPL24*	37'253	37'642	37'864	32'262	43'465	0.430	0.571214702	1
ENSBTAG00000013463	*DISP1*	702	177	406	608	204	-1.573	0.091827462	1
ENSBTAG00000013464	*CEP97*	284	176	225	246	203	-0.275	0.792669375	1
ENSBTAG00000013465	*FAM55C*	1'003	1'038	1'034	869	1'199	0.465	0.572275912	1
ENSBTAG00000013468	*AHNAK*	66'826	76'622	73'174	57'873	88'475	0.612	0.420506948	1
ENSBTAG00000013469	*SRCIN1*	470	552	522	407	637	0.647	0.463810221	1
ENSBTAG00000013472	*COL1A2*	4'781'780	1'761'353	3'087'489	4'141'143	2'033'835	-1.026	0.179805579	1
ENSBTAG00000013473	*MGC127766*	3	1	2	3	1	-1.170	0.985253077	1
ENSBTAG00000013475	*TRAF3*	299	250	274	259	289	0.157	0.875787191	1
ENSBTAG00000013476	*BT.46077*	365	213	281	316	246	-0.362	0.714118214	1
ENSBTAG00000013477	*GCHFR*	234	239	239	203	276	0.446	0.661116361	1
ENSBTAG00000013478	*MARVELD1*	8'790	13'049	11'340	7'612	15'068	0.985	0.200653725	1
ENSBTAG00000013479	*BT.67331*	2'718	2'955	2'883	2'354	3'412	0.536	0.493418497	1
ENSBTAG00000013480	*DNAJC17*	863	758	811	747	875	0.228	0.78626737	1
ENSBTAG00000013483	*TAF1A*	1'066	479	738	923	553	-0.739	0.385166923	1
ENSBTAG00000013485	*ZFYVE19*	430	364	396	372	420	0.175	0.850663244	1
ENSBTAG00000013486	*C12ORF66*	51	49	50	44	57	0.357	0.843880519	1
ENSBTAG00000013488	*PPP1R14D*	75	50	61	65	58	-0.170	0.925677636	1
ENSBTAG00000013489	*CYP27A1*	1'629	665	1'089	1'411	768	-0.878	0.287089059	1
ENSBTAG00000013491	*EML1*	1'314	1'154	1'235	1'138	1'333	0.228	0.779110067	1
ENSBTAG00000013492	*PRKAG3*	31	29	30	27	33	0.319	0.898503662	1
ENSBTAG00000013493	*BNIP2*	1'025	593	786	888	685	-0.374	0.657846781	1
ENSBTAG00000013495	*WDFY3*	1'713	1'355	1'524	1'484	1'565	0.077	0.923793167	1
ENSBTAG00000013496	*CPM*	167	90	124	145	104	-0.477	0.698488748	1

Ensembl gene ID	geneName	counts wildtype horn bud	counts polled horn bud	baseMean	baseMean wildtype horn bud	baseMean polled horn bud	log2FoldChange	pval	padj
ENSBTAG00000013498	*HTR2A*	199	206	205	172	238	0.465	0.659429848	1
ENSBTAG00000013501	*DHRS13*	111	247	191	96	285	1.569	0.149000527	1
ENSBTAG00000013505	*IQCA1*	131	43	82	113	50	-1.192	0.402685033	1
ENSBTAG00000013509	*TTC16*	16	20	18	14	23	0.737	0.813929384	1
ENSBTAG00000013510	*TOR2A*	357	352	358	309	406	0.395	0.673435806	1
ENSBTAG00000013511	*VWA5B1*	35	16	24	30	18	-0.714	0.787699325	1
ENSBTAG00000013513	*ATP6V1C1*	2'168	1'352	1'719	1'878	1'561	-0.266	0.739442712	1
ENSBTAG00000013515	*C5H12ORF11*	1'173	782	959	1'016	903	-0.170	0.83888916	1
ENSBTAG00000013523	*OSBPL7*	2'069	1'725	1'892	1'792	1'992	0.153	0.847219853	1
ENSBTAG00000013524	*CMIP*	1'882	2'204	2'087	1'630	2'545	0.643	0.416875556	1
ENSBTAG00000013525	*C6ORF25*	93	72	82	81	83	0.046	0.983041915	1
ENSBTAG00000013526	*EFTUD2*	4'014	5'875	5'130	3'476	6'784	0.965	0.214267081	1
ENSBTAG00000013527	*PGD*	4'214	4'635	4'501	3'649	5'352	0.552	0.475314742	1
ENSBTAG00000013528	*CHMP7*	3'057	3'549	3'373	2'647	4'098	0.630	0.418769836	1
ENSBTAG00000013529	*CCDC103*	10	2	5	9	2	-1.907	0.815196829	1
ENSBTAG00000013530	*DDAH2*	7'134	9'784	8'738	6'178	11'298	0.871	0.258119343	1
ENSBTAG00000013531	*APITD1*	1'287	692	957	1'115	799	-0.480	0.563048065	1
ENSBTAG00000013532	*FAM187A*	19	0	8	16	0		0.389538827	1
ENSBTAG00000013533	*CLIC1*	8'153	8'349	8'351	7'061	9'641	0.449	0.557489026	1
ENSBTAG00000013535	*DFFA*	784	686	736	679	792	0.222	0.793340537	1
ENSBTAG00000013536	*P2RY11*	11	3	6	10	3	-1.459	0.83622464	1
ENSBTAG00000013537	*FER1L6*	28	9	17	24	10	-1.222	0.70359948	1
ENSBTAG00000013538	*BT.103198*	626	986	840	542	1'139	1.070	0.204267645	1
ENSBTAG00000013541	*LMO3*	209	34	110	181	39	-2.205	0.092649096	1
ENSBTAG00000013544	*LGALS8*	445	382	413	385	441	0.195	0.832196656	1
ENSBTAG00000013545	*BT.45349*	502	513	514	435	592	0.446	0.61389115	1
ENSBTAG00000013548	*BT.53645*	6'110	2'921	4'332	5'291	3'373	-0.650	0.402865976	1
ENSBTAG00000013555	*ACVR1B*	643	1'043	881	557	1'204	1.113	0.185454193	1
ENSBTAG00000013556	*UNC13D*	723	703	719	626	812	0.375	0.659351578	1
ENSBTAG00000013557	*BT.28338*	951	626	773	824	723	-0.188	0.825171759	1
ENSBTAG00000013560	*RABIF*	131	118	125	113	136	0.264	0.831393423	1
ENSBTAG00000013562	*SKP2*	400	330	364	346	381	0.138	0.884554356	1
ENSBTAG00000013563	*TSG101*	1'231	531	840	1'066	613	-0.798	0.342749046	1
ENSBTAG00000013568	*UEVLD*	354	249	297	307	288	-0.093	0.928054086	1
ENSBTAG00000013569	*CD38*	149	18	75	129	21	-2.634	0.086893624	1
ENSBTAG00000013573	*BIRC5*	1'102	1'091	1'107	954	1'260	0.401	0.624420763	1
ENSBTAG00000013574	*ANKRD60*	1	0	0	1	0		1	1
ENSBTAG00000013577	*SLC1A5*	2'794	3'139	3'022	2'420	3'625	0.583	0.455627043	1
ENSBTAG00000013578	*BT.103113*	0	1	1	0	1	Inf	0.993540919	1
ENSBTAG00000013579	*TMEM66*	4'984	3'421	4'133	4'316	3'950	-0.128	0.869744608	1
ENSBTAG00000013580	*KDM3A*	3'717	2'724	3'182	3'219	3'145	-0.033	0.96695089	1
ENSBTAG00000013581	*LEPROTL1*	1'553	1'042	1'274	1'345	1'203	-0.161	0.844281003	1
ENSBTAG00000013585	*protein_coding*	1	0	0	1	0		1	1
ENSBTAG00000013586	*BT.63814*	29	6	16	25	7	-1.858	0.581888091	1
ENSBTAG00000013587	*BT.22492*	939	809	874	813	934	0.200	0.81062649	1
ENSBTAG00000013588	*ZNF532*	5'804	5'001	5'401	5'026	5'775	0.200	0.794447258	1
ENSBTAG00000013589	*CHMP3*	4'046	3'847	3'973	3'504	4'442	0.342	0.65841467	1
ENSBTAG00000013591	*CYB5R4*	536	350	434	464	404	-0.200	0.827978611	1
ENSBTAG00000013592	*ZNF248*	359	238	293	311	275	-0.178	0.858166524	1
ENSBTAG00000013593	*SNRPA1*	1'334	559	900	1'155	645	-0.840	0.315328808	1
ENSBTAG00000013596	*BT.48865*	1	0	0	1	0		1	1
ENSBTAG00000013598	*RSPO2*	10	3	6	9	3	-1.322	0.863266279	1
ENSBTAG00000013600	*PSMG1*	2'032	1'688	1'854	1'760	1'949	0.147	0.852550736	1
ENSBTAG00000013602	*BRWD1*	2'913	1'466	2'108	2'523	1'693	-0.576	0.467751921	1
ENSBTAG00000013606	*FKBP2*	2'522	1'469	1'940	2'184	1'696	-0.365	0.646484941	1
ENSBTAG00000013607	*BT.75467*	6'928	10'052	8'803	6'000	11'607	0.952	0.216924495	1
ENSBTAG00000013611	*SCLT1*	455	214	321	394	247	-0.673	0.482227295	1
ENSBTAG00000013612	*MBLAC2*	883	400	613	765	462	-0.727	0.402221174	1
ENSBTAG00000013613	*C19ORF42*	1'881	1'482	1'670	1'629	1'711	0.071	0.929072171	1
ENSBTAG00000013614	*TMEM38A*	307	382	353	266	441	0.730	0.436382907	1
ENSBTAG00000013615	*CHORDC1*	2'707	1'188	1'858	2'344	1'372	-0.773	0.332829045	1
ENSBTAG00000013616	*AP4E1*	303	209	252	262	241	-0.121	0.908601634	1
ENSBTAG00000013619	*BT.61074*	4	1	2	3	1	-1.585	0.952247636	1
ENSBTAG00000013620	*UCHL5*	2'184	1'225	1'653	1'891	1'415	-0.419	0.600815394	1
ENSBTAG00000013621	*DPY19L4*	491	271	369	425	313	-0.442	0.63612904	1
ENSBTAG00000013623	*TIMM13*	1'612	1'769	1'719	1'396	2'043	0.549	0.491300852	1
ENSBTAG00000013624	*BT.80892*	1'123	2'130	1'716	973	2'460	1.339	0.097881446	1
ENSBTAG00000013627	*HNRNPAB*	7'480	5'546	6'441	6'478	6'404	-0.017	0.983717721	1
ENSBTAG00000013628	*BT.105480*	848	447	625	734	516	-0.509	0.556795346	1
ENSBTAG00000013629	*WRB*	2'319	1'722	1'998	2'008	1'988	-0.014	0.98692423	1
ENSBTAG00000013631	*BT.64559*	9'966	8'844	9'421	8'631	10'212	0.243	0.750486685	1
ENSBTAG00000013632	*GRM4*	96	62	77	83	72	-0.216	0.889836755	1

Ensembl gene ID	geneName	counts wildtype horn bud	counts polled horn bud	baseMean	baseMean wildtype horn bud	baseMean polled horn bud	log2FoldChange	pval	padj
ENSBTAG00000013635	*PDE6H*	1	0	0	1	0		1	1
ENSBTAG00000013636	*DGKZ*	1'927	2'379	2'208	1'669	2'747	0.719	0.363288407	1
ENSBTAG00000013638	*IQCH*	20	15	17	17	17	0.000	1	1
ENSBTAG00000013640	*KLRG1*	8	2	5	7	2	-1.585	0.87391615	1
ENSBTAG00000013641	*BT.65578*	1'711	1'116	1'385	1'482	1'289	-0.201	0.803976036	1
ENSBTAG00000013642	*BT.28372*	14	0	6	12	0		0.520471303	1
ENSBTAG00000013644	*BT.62923*	13	0	6	11	0		0.551229012	1
ENSBTAG00000013645	*RFFL*	404	288	341	350	333	-0.073	0.942045243	1
ENSBTAG00000013648	*BT.74490*	2	0	1	2	0		0.974934741	1
ENSBTAG00000013650	*PQLC2*	763	549	647	661	634	-0.060	0.947069082	1
ENSBTAG00000013651	*BT.39960*	1'047	1'279	1'192	907	1'477	0.704	0.388459832	1
ENSBTAG00000013652	*BT.87840*	410	189	287	355	218	-0.702	0.473414289	1
ENSBTAG00000013653	*NFE2L1*	9'069	8'648	8'920	7'854	9'986	0.346	0.650465159	1
ENSBTAG00000013657	*CHRNA5*	189	146	166	164	169	0.043	0.974657716	1
ENSBTAG00000013658	*ERC1*	737	1'387	1'120	638	1'602	1.327	0.109248801	1
ENSBTAG00000013663	*C1H3orf26*	559	310	421	484	358	-0.436	0.633971156	1
ENSBTAG00000013664	*FILIP1L*	2'913	2'318	2'600	2'523	2'677	0.085	0.912991468	1
ENSBTAG00000013665	*KCNG2*	83	21	48	72	24	-1.568	0.383684453	1
ENSBTAG00000013666	*SLC25A29*	1'689	2'405	2'120	1'463	2'777	0.925	0.244452265	1
ENSBTAG00000013667	*TMEM30C*	43	20	30	37	23	-0.689	0.767580939	1
ENSBTAG00000013669	*KIF22*	816	1'195	1'043	707	1'380	0.965	0.243013709	1
ENSBTAG00000013670	*TSCOT*	433	219	314	375	253	-0.568	0.554671536	1
ENSBTAG00000013671	*UTP14A*	7'134	10'191	8'973	6'178	11'768	0.930	0.227693009	1
ENSBTAG00000013674	*EDNRA*	4'399	4'654	4'592	3'810	5'374	0.496	0.520851087	1
ENSBTAG00000013675	*BT.75805*	5	1	3	4	1	-1.907	0.9188325	1
ENSBTAG00000013676	*DYNC2LI1*	1'959	1'075	1'469	1'697	1'241	-0.451	0.576074885	1
ENSBTAG00000013677	*SEC14L1*	1'777	1'999	1'924	1'539	2'308	0.585	0.461393459	1
ENSBTAG00000013678	*OSGIN2*	601	323	447	520	373	-0.481	0.59557713	1
ENSBTAG00000013680	*ATP1B2*	636	520	576	551	600	0.125	0.887357979	1
ENSBTAG00000013686	*WSCD2*	146	197	177	126	227	0.847	0.439048218	1
ENSBTAG00000013688	*PGF*	266	91	168	230	105	-1.132	0.311346028	1
ENSBTAG00000013689	*BT.28177*	162	139	150	140	161	0.194	0.869287629	1
ENSBTAG00000013693	*protein_coding*	38	18	27	33	21	-0.663	0.791807555	1
ENSBTAG00000013697	*CLASRP*	2'031	1'524	1'759	1'759	1'760	0.001	0.999439724	1
ENSBTAG00000013698	*TTC25*	44	35	39	38	40	0.085	0.978986637	1
ENSBTAG00000013699	*TBC1D1*	2'760	2'694	2'750	2'390	3'111	0.380	0.626941949	1
ENSBTAG00000013700	*protein_coding*	4	14	10	3	16	2.222	0.638389235	1
ENSBTAG00000013702	*ZNF296*	43	39	41	37	45	0.274	0.895766536	1
ENSBTAG00000013705	*NFKBIE*	516	525	527	447	606	0.440	0.61774455	1
ENSBTAG00000013706	*MEGF9*	531	295	400	460	341	-0.433	0.63877254	1
ENSBTAG00000013708	*SNX8*	674	865	791	584	999	0.775	0.358469801	1
ENSBTAG00000013715	*FAM5B*	84	109	99	73	126	0.791	0.547193844	1
ENSBTAG00000013716	*NR2C2*	766	747	763	663	863	0.379	0.653782305	1
ENSBTAG00000013717	*PRAP1*	288	153	213	249	177	-0.497	0.635508554	1
ENSBTAG00000013718	*BT.53584*	5	0	2	4	0		0.853268594	1
ENSBTAG00000013721	*PSMD10*	609	638	632	527	737	0.482	0.57587907	1
ENSBTAG00000013722	*AARS2*	1'132	1'322	1'253	980	1'527	0.639	0.431841101	1
ENSBTAG00000013723	*MRPS25*	381	436	417	330	503	0.610	0.504044919	1
ENSBTAG00000013724	*ATG4A*	458	492	482	397	568	0.518	0.561318854	1
ENSBTAG00000013725	*ZFYVE20*	861	881	881	746	1'017	0.448	0.59071472	1
ENSBTAG00000013726	*RNPEP*	1'473	1'540	1'527	1'276	1'778	0.479	0.550261258	1
ENSBTAG00000013727	*UMPS*	1'269	989	1'120	1'099	1'142	0.055	0.946403214	1
ENSBTAG00000013730	*CD5*	58	44	51	50	51	0.016	1	1
ENSBTAG00000013732	*GPR37*	467	161	295	404	186	-1.121	0.251452478	1
ENSBTAG00000013734	*ATP6V1E2*	15	2	8	13	2	-2.492	0.678711229	1
ENSBTAG00000013735	*TBCE*	1'814	938	1'327	1'571	1'083	-0.536	0.508349297	1
ENSBTAG00000013736	*PROM1*	443	252	337	384	291	-0.399	0.675042727	1
ENSBTAG00000013737	*C20ORF132*	2	0	1	2	0		0.974934741	1
ENSBTAG00000013740	*SP2*	381	502	455	330	580	0.813	0.367373658	1
ENSBTAG00000013741	*CPLX2*	136	15	68	118	17	-2.766	0.086715053	1
ENSBTAG00000013744	*SYNPO*	1'075	2'257	1'769	931	2'606	1.485	0.066882941	1
ENSBTAG00000013745	*BT.27462*	7'304	10'110	9'000	6'325	11'674	0.884	0.250879016	1
ENSBTAG00000013749	*BT.61817*	852	801	831	738	925	0.326	0.697163222	1
ENSBTAG00000013750	*B3GALNT2*	1'804	981	1'348	1'562	1'133	-0.464	0.567006696	1
ENSBTAG00000013753	*BT.22808*	1'309	895	1'084	1'134	1'033	-0.133	0.872254988	1
ENSBTAG00000013755	*ITGB5*	9'715	16'790	13'900	8'413	19'387	1.204	0.118760252	1
ENSBTAG00000013756	*BT.105714*	3	2	2	3	2	-0.170	1	1
ENSBTAG00000013757	*CTCF*	6'546	5'273	5'879	5'669	6'089	0.103	0.892939332	1
ENSBTAG00000013759	*TSPAN9*	1'006	1'772	1'459	871	2'046	1.232	0.129968911	1
ENSBTAG00000013760	*COL4A6*	1'536	1'820	1'716	1'330	2'102	0.660	0.408873051	1
ENSBTAG00000013761	*STMN1*	22'576	15'522	18'737	19'551	17'923	-0.125	0.869817371	1
ENSBTAG00000013763	*C26H10orf82*	9	0	4	8	0		0.690676647	1

Ensembl gene ID	geneName	counts wildtype horn bud	counts polled horn bud	baseMean	baseMean wildtype horn bud	baseMean polled horn bud	log2FoldChange	pval	padj
ENSBTAG00000013764	*OPTC*	188	41	105	163	47	-1.782	0.17497621	1
ENSBTAG00000013766	*BT.54340*	8	16	13	7	18	1.415	0.716950431	1
ENSBTAG00000013769	*PVRL3*	1'009	859	933	874	992	0.183	0.825693387	1
ENSBTAG00000013772	*SRRM1*	7'293	3'408	5'126	6'316	3'935	-0.683	0.378139972	1
ENSBTAG00000013773	*PKP4*	4'386	3'463	3'899	3'798	3'999	0.074	0.923412191	1
ENSBTAG00000013774	*C15ORF52*	332	321	329	288	371	0.366	0.700179488	1
ENSBTAG00000013776	*ITPA*	1'624	1'271	1'437	1'406	1'468	0.061	0.939294797	1
ENSBTAG00000013778	*BT.74588*	19	7	12	16	8	-1.026	0.806704154	1
ENSBTAG00000013779	*HSPA12A*	171	21	86	148	24	-2.610	0.072921423	1
ENSBTAG00000013781	*MIOS*	1'287	938	1'099	1'115	1'083	-0.041	0.961652043	1
ENSBTAG00000013782	*DNAJB2*	2'632	2'714	2'707	2'279	3'134	0.459	0.557493061	1
ENSBTAG00000013784	*CSNK2A2*	1'167	1'029	1'099	1'011	1'188	0.233	0.775489687	1
ENSBTAG00000013790	*MAP3K1*	1'916	1'216	1'532	1'659	1'404	-0.241	0.764968665	1
ENSBTAG00000013791	*GPR41*	1	0	0	1	0		1	1
ENSBTAG00000013792	*FAM100B*	350	379	370	303	438	0.530	0.568873067	1
ENSBTAG00000013793	*NEDD4*	581	408	487	503	471	-0.095	0.918069476	1
ENSBTAG00000013799	*pseudogene*	1'494	2'164	1'896	1'294	2'499	0.950	0.234457189	1
ENSBTAG00000013801	*PBX1*	690	863	797	598	997	0.738	0.381454125	1
ENSBTAG00000013802	*DAB1*	59	69	65	51	80	0.641	0.680570837	1
ENSBTAG00000013806	*EFHB*	155	93	121	134	107	-0.322	0.798165326	1
ENSBTAG00000013810	*GABBR2*	220	74	138	191	85	-1.157	0.328901917	1
ENSBTAG00000013812	*TROVE2*	616	353	471	533	408	-0.388	0.666182828	1
ENSBTAG00000013813	*KLHL17*	1'848	1'432	1'627	1'600	1'654	0.047	0.953109721	1
ENSBTAG00000013819	*BT.99676*	7	0	3	6	0		0.769774961	1
ENSBTAG00000013820	*protein_coding*	1	0	0	1	0		1	1
ENSBTAG00000013821	*BT.89457*	687	673	686	595	777	0.385	0.651827563	1
ENSBTAG00000013822	*GAS2L3*	510	273	378	442	315	-0.487	0.601016548	1
ENSBTAG00000013824	*WWC3*	816	807	819	707	932	0.399	0.634261203	1
ENSBTAG00000013825	*BCAT1*	361	113	222	313	130	-1.261	0.226966461	1
ENSBTAG00000013830	*LEO1*	1'495	875	1'153	1'295	1'010	-0.358	0.662232403	1
ENSBTAG00000013831	*DSG1*	6'304	4'495	5'325	5'459	5'190	-0.073	0.925510483	1
ENSBTAG00000013832	*TRIT1*	2'229	1'415	1'782	1'930	1'634	-0.241	0.763490658	1
ENSBTAG00000013834	*TRIP10*	1'549	970	1'231	1'341	1'120	-0.260	0.749925061	1
ENSBTAG00000013836	*BT.37952*	134	46	85	116	53	-1.127	0.421823608	1
ENSBTAG00000013837	*TUBE1*	699	376	520	605	434	-0.480	0.588496624	1
ENSBTAG00000013838	*ALLC*	4	0	2	3	0		0.89545886	1
ENSBTAG00000013841	*MGC134427*	2	1	1	2	1	-0.585	1	1
ENSBTAG00000013842	*BT.43242*	632	625	635	547	722	0.399	0.643274226	1
ENSBTAG00000013843	*BT.28710*	3'004	2'199	2'570	2'602	2'539	-0.035	0.965621305	1
ENSBTAG00000013848	*BT.22380*	2'221	2'669	2'503	1'923	3'082	0.680	0.387456506	1
ENSBTAG00000013851	*BCL10*	1'181	904	1'033	1'023	1'044	0.029	0.972054086	1
ENSBTAG00000013854	*CALML5*	1'355	1'174	1'265	1'173	1'356	0.208	0.797340694	1
ENSBTAG00000013855	*ORMDL3*	3'346	3'540	3'493	2'898	4'088	0.496	0.52332786	1
ENSBTAG00000013856	*SSBP2*	5'049	3'123	3'989	4'373	3'606	-0.278	0.720571698	1
ENSBTAG00000013857	*BT.28221*	40	53	48	35	61	0.821	0.645734906	1
ENSBTAG00000013858	*C16ORF72*	1'104	687	875	956	793	-0.269	0.748131775	1
ENSBTAG00000013859	*BT.21542*	2'413	1'758	2'060	2'090	2'030	-0.042	0.959122248	1
ENSBTAG00000013860	*GADD45A*	1'313	1'363	1'355	1'137	1'574	0.469	0.56145789	1
ENSBTAG00000013861	*SLC8A1*	225	321	283	195	371	0.928	0.344299702	1
ENSBTAG00000013863	*BT.85182*	4'835	1'556	2'992	4'187	1'797	-1.221	0.122524984	1
ENSBTAG00000013867	*DLGAP3*	203	22	101	176	25	-2.791	0.044150023	1
ENSBTAG00000013869	*BT.66090*	1'875	3'272	2'701	1'624	3'778	1.218	0.124167487	1
ENSBTAG00000013871	*C18ORF26*	8	8	8	7	9	0.415	0.960311941	1
ENSBTAG00000013873	*MSX2*	299	297	301	259	343	0.405	0.675304474	1
ENSBTAG00000013874	*C18H16orf80*	1'543	1'258	1'394	1'336	1'453	0.120	0.88128267	1
ENSBTAG00000013875	*VAX1*	3	0	1	3	0		0.936647693	1
ENSBTAG00000013877	*BT.54223*	19	6	12	16	7	-1.248	0.770493406	1
ENSBTAG00000013879	*DGKH*	112	86	98	97	99	0.034	0.987462066	1
ENSBTAG00000013880	*WWC1*	616	401	498	533	463	-0.204	0.820345875	1
ENSBTAG00000013881	*BT.61260*	516	510	518	447	589	0.398	0.652319672	1
ENSBTAG00000013882	*CHMP2A*	3'099	2'763	2'937	2'684	3'190	0.249	0.749002925	1
ENSBTAG00000013884	*SIPA1*	2'384	2'823	2'662	2'065	3'260	0.659	0.401372309	1
ENSBTAG00000013885	*ADSS*	1'717	910	1'269	1'487	1'051	-0.501	0.537969779	1
ENSBTAG00000013888	*BT.88447*	7	6	6	6	7	0.193	1	1
ENSBTAG00000013889	*TMEM141*	477	408	442	413	471	0.190	0.834840565	1
ENSBTAG00000013895	*BT.26220*	3'335	4'406	3'988	2'888	5'088	0.817	0.294094878	1
ENSBTAG00000013898	*NPB*	7	18	13	6	21	1.778	0.63434103	1
ENSBTAG00000013899	*LARP4B*	1'015	931	977	879	1'075	0.290	0.725151648	1
ENSBTAG00000013900	*TRIM21*	280	321	307	242	371	0.612	0.524950577	1
ENSBTAG00000013901	*PTDSS1*	2'906	2'430	2'661	2'517	2'806	0.157	0.840848255	1
ENSBTAG00000013905	*SMC3*	8'076	3'708	5'638	6'994	4'282	-0.708	0.360019081	1
ENSBTAG00000013906	*ZNF594*	5	2	3	4	2	-0.907	0.965043111	1

Ensembl gene ID	geneName	counts wildtype horn bud	counts polled horn bud	baseMean	baseMean wildtype horn bud	baseMean polled horn bud	log2FoldChange	pval	padj
ENSBTAG00000013907	*CRP*	1	1	1	1	1	0.415	1	1
ENSBTAG00000013909	*BOC*	7'167	9'013	8'307	6'207	10'407	0.746	0.3321492	1
ENSBTAG00000013910	*POLR3GL*	1'206	616	878	1'044	711	-0.554	0.507576967	1
ENSBTAG00000013912	*TXNRD1*	2'949	2'645	2'804	2'554	3'054	0.258	0.74102187	1
ENSBTAG00000013914	*PET112L*	1'002	1'135	1'089	868	1'311	0.595	0.468311834	1
ENSBTAG00000013915	*DSN1*	1'184	929	1'049	1'025	1'073	0.065	0.937276302	1
ENSBTAG00000013916	*HERC2*	4'795	3'402	4'040	4'153	3'928	-0.080	0.918593091	1
ENSBTAG00000013917	*SRCAP*	4'908	11'075	8'519	4'250	12'788	1.589	0.04225072	1
ENSBTAG00000013918	*LPHN3*	648	354	485	561	409	-0.457	0.609501937	1
ENSBTAG00000013920	*WDR52*	219	68	134	190	79	-1.272	0.288109534	1
ENSBTAG00000013922	*MOSPD1*	566	607	596	490	701	0.516	0.552269413	1
ENSBTAG00000013923	*QPCT*	1'360	914	1'117	1'178	1'055	-0.158	0.848029176	1
ENSBTAG00000013924	*RPS11*	89'092	85'877	88'159	77'156	99'162	0.362	0.63283723	1
ENSBTAG00000013925	*KIN*	496	347	415	430	401	-0.100	0.915656881	1
ENSBTAG00000013926	*FCGRT*	22'272	13'397	17'379	19'288	15'470	-0.318	0.676780629	1
ENSBTAG00000013927	*BT.87504*	80	121	105	69	140	1.012	0.432987768	1
ENSBTAG00000013928	*WFDC2*	7	5	6	6	6	-0.070	1	1
ENSBTAG00000013929	*RRAD*	129	87	106	112	100	-0.153	0.912639041	1
ENSBTAG00000013930	*ATP5C1*	5'766	5'284	5'547	4'994	6'101	0.289	0.706916534	1
ENSBTAG00000013931	*FAM96B*	1'280	1'066	1'170	1'109	1'231	0.151	0.853044913	1
ENSBTAG00000013932	*CCDC71*	677	1'119	939	586	1'292	1.140	0.172876234	1
ENSBTAG00000013935	*BT.23506*	2'465	2'759	2'660	2'135	3'186	0.578	0.461523544	1
ENSBTAG00000013937	*BT.52886*	1'363	952	1'140	1'180	1'099	-0.103	0.901567404	1
ENSBTAG00000013938	*PHC3*	104	69	85	90	80	-0.177	0.907547238	1
ENSBTAG00000013941	*TMCO7*	825	598	702	714	691	-0.049	0.956418017	1
ENSBTAG00000013942	*DAGLA*	293	468	397	254	540	1.091	0.238185288	1
ENSBTAG00000013943	*ZNF703*	391	592	511	339	684	1.013	0.254725514	1
ENSBTAG00000013946	*BT.60840*	6'718	3'590	4'982	5'818	4'145	-0.489	0.52736491	1
ENSBTAG00000013947	*APTX*	416	348	381	360	402	0.158	0.86632859	1
ENSBTAG00000013949	*AHCTF1*	3'266	1'777	2'440	2'828	2'052	-0.463	0.55674404	1
ENSBTAG00000013951	*CHFR*	857	899	890	742	1'038	0.484	0.561055178	1
ENSBTAG00000013952	*HNRNPD*	15'969	9'058	12'144	13'830	10'459	-0.403	0.598498599	1
ENSBTAG00000013953	*BT.59726*	9'187	5'531	7'171	7'956	6'387	-0.317	0.6802752	1
ENSBTAG00000013955	*BT.36508*	728	1'436	1'144	630	1'658	1.395	0.092335123	1
ENSBTAG00000013956	*BT.65691*	3'357	3'948	3'733	2'907	4'559	0.649	0.404085116	1
ENSBTAG00000013957	*GALNT3*	284	76	167	246	88	-1.487	0.187468808	1
ENSBTAG00000013958	*BT.28427*	1'283	1'477	1'408	1'111	1'705	0.618	0.443590635	1
ENSBTAG00000013959	*GPR142*	2	1	1	2	1	-0.585	1	1
ENSBTAG00000013960	*PSAT1*	2'555	1'926	2'218	2'213	2'224	0.007	0.992563078	1
ENSBTAG00000013961	*MYSM1*	482	234	344	417	270	-0.627	0.506962807	1
ENSBTAG00000013971	*PI4KA*	5'037	7'110	6'286	4'362	8'210	0.912	0.238246958	1
ENSBTAG00000013973	*SERPIND1*	29	29	29	25	33	0.415	0.865591787	1
ENSBTAG00000013974	*TMEM215*	24	5	13	21	6	-1.848	0.630822808	1
ENSBTAG00000013976	*BT.63246*	95	34	61	82	39	-1.067	0.507012711	1
ENSBTAG00000013977	*PRSS38*	3	38	23	3	44	4.078	0.160732072	1
ENSBTAG00000013979	*BT.51319*	17	7	11	15	8	-0.865	0.849311167	1
ENSBTAG00000013980	*ECSOD*	25	4	13	22	5	-2.229	0.568026072	1
ENSBTAG00000013981	*SPTSSA*	2'119	1'263	1'647	1'835	1'458	-0.331	0.679205394	1
ENSBTAG00000013982	*BT.26227*	7'130	5'797	6'434	6'175	6'694	0.116	0.879010349	1
ENSBTAG00000013983	*FAM164C*	11	6	8	10	7	-0.459	0.954298496	1
ENSBTAG00000013984	*KL*	26	22	24	23	25	0.174	0.96187418	1
ENSBTAG00000013985	*FKRP*	1'190	597	860	1'031	689	-0.580	0.48874846	1
ENSBTAG00000013988	*BID1*	2'628	2'488	2'574	2'276	2'873	0.336	0.667981024	1
ENSBTAG00000013989	*C11ORF63*	5	5	5	4	6	0.415	0.985023983	1
ENSBTAG00000013990	*WNT6*	359	784	608	311	905	1.542	0.080048119	1
ENSBTAG00000013992	*ACYP1*	518	173	324	449	200	-1.167	0.224572543	1
ENSBTAG00000013994	*BT.106481*	1	0	0	1	0		1	1
ENSBTAG00000013996	*SH3BP2*	906	990	964	785	1'143	0.543	0.511761303	1
ENSBTAG00000013999	*BT.88154*	469	458	468	406	529	0.381	0.671098847	1
ENSBTAG00000014001	*MAP1A*	4'042	5'999	5'214	3'500	6'927	0.985	0.204909831	1
ENSBTAG00000014002	*CEP95*	3'343	1'341	2'222	2'895	1'548	-0.903	0.255402805	1
ENSBTAG00000014003	*MYF6*	6	58	36	5	67	3.688	0.098914803	1
ENSBTAG00000014005	*PPIP5K1*	645	289	446	559	334	-0.743	0.412061741	1
ENSBTAG00000014006	*TOR3A*	2'018	1'787	1'906	1'748	2'063	0.240	0.762316897	1
ENSBTAG00000014007	*ELMOD2*	3'286	1'768	2'444	2'846	2'042	-0.479	0.54307814	1
ENSBTAG00000014011	*TMOD2*	72	124	103	62	143	1.199	0.356571571	1
ENSBTAG00000014014	*UBIAD1*	126	174	155	109	201	0.881	0.438415382	1
ENSBTAG00000014015	*PLA2G6*	637	722	693	552	834	0.596	0.485283319	1
ENSBTAG00000014016	*BT.93003*	26	9	16	23	10	-1.115	0.73867587	1
ENSBTAG00000014017	*PCGF3*	1'045	924	986	905	1'067	0.237	0.773615914	1
ENSBTAG00000014020	*RTKN2*	45	16	29	39	18	-1.077	0.646840141	1
ENSBTAG00000014021	*BT.43088*	156	110	131	135	127	-0.089	0.948117288	1

Ensembl gene ID	geneName	counts wildtype horn bud	counts polled horn bud	baseMean	baseMean wildtype horn bud	baseMean polled horn bud	log2FoldChange	pval	padj
ENSBTAG00000014023	*SIRT1*	3'353	1'641	2'399	2'904	1'895	-0.616	0.43508014	1
ENSBTAG00000014024	*POMP*	6'180	3'805	4'873	5'352	4'394	-0.285	0.713040435	1
ENSBTAG00000014026	*PAG1*	17	3	9	15	3	-2.087	0.685542914	1
ENSBTAG00000014027	*TRIM36*	230	171	198	199	197	-0.013	0.99692527	1
ENSBTAG00000014028	*PSTK*	151	99	123	131	114	-0.194	0.880310965	1
ENSBTAG00000014029	*HERC4*	4'580	1'828	3'039	3'966	2'111	-0.910	0.24678913	1
ENSBTAG00000014030	*BT.87397*	513	255	369	444	294	-0.593	0.525067754	1
ENSBTAG00000014031	*BT.45021*	176	46	103	152	53	-1.521	0.247448829	1
ENSBTAG00000014032	*EIF3H*	9'116	10'718	10'135	7'895	12'376	0.649	0.3973793	1
ENSBTAG00000014034	*ACPL2*	900	489	672	779	565	-0.465	0.588181647	1
ENSBTAG00000014038	*CDKL2*	161	48	97	139	55	-1.331	0.318804011	1
ENSBTAG00000014041	*G3BP2*	3'740	3'605	3'701	3'239	4'163	0.362	0.640692114	1
ENSBTAG00000014042	*TLR10*	39	17	27	34	20	-0.783	0.753120629	1
ENSBTAG00000014043	*BT.39059*	2'889	2'176	2'507	2'502	2'513	0.006	0.993654826	1
ENSBTAG00000014044	*VEZT*	2'979	1'132	1'944	2'580	1'307	-0.981	0.219589108	1
ENSBTAG00000014046	*BPI*	32	23	27	28	27	-0.061	0.998655064	1
ENSBTAG00000014048	*C13H20orf196*	188	126	154	163	145	-0.162	0.892430804	1
ENSBTAG00000014050	*CHRNB3*	0	1	1	0	1	Inf	0.993540919	1
ENSBTAG00000014053	*MAK*	14	8	11	12	9	-0.392	0.950061617	1
ENSBTAG00000014054	*C9H6orf165*	16	1	8	14	1	-3.585	0.580698833	1
ENSBTAG00000014057	*SLC35A1*	3'421	1'526	2'362	2'963	1'762	-0.750	0.342943374	1
ENSBTAG00000014058	*LDB2*	1'043	568	780	903	656	-0.462	0.585072598	1
ENSBTAG00000014059	*ATP2B4*	1'199	1'203	1'214	1'038	1'389	0.420	0.605574601	1
ENSBTAG00000014060	*LSM6*	3'051	1'458	2'163	2'642	1'684	-0.650	0.411797747	1
ENSBTAG00000014062	*FRMD1*	1	3	2	1	3	2.000	0.933774563	1
ENSBTAG00000014063	*DNAH8*	13	5	9	11	6	-0.963	0.868297704	1
ENSBTAG00000014064	*FGFR2*	15'288	9'105	11'877	13'240	10'514	-0.333	0.663858227	1
ENSBTAG00000014068	*VDAC5P*	5'148	5'336	5'310	4'458	6'161	0.467	0.544799899	1
ENSBTAG00000014069	*BT.102008*	1'622	949	1'250	1'405	1'096	-0.358	0.660016369	1
ENSBTAG00000014072	*protein_coding*	10	1	5	9	1	-2.907	0.754028015	1
ENSBTAG00000014074	*SNX13*	302	313	311	262	361	0.467	0.627044804	1
ENSBTAG00000014075	*BT.23843*	21	5	12	18	6	-1.655	0.689790335	1
ENSBTAG00000014076	*FAM175A*	615	538	577	533	621	0.222	0.799341929	1
ENSBTAG00000014079	*GIGYF2*	4'436	3'075	3'696	3'842	3'551	-0.114	0.884553416	1
ENSBTAG00000014080	*BT.67542*	2'019	1'328	1'641	1'749	1'533	-0.189	0.813843457	1
ENSBTAG00000014081	*NBAS*	2'023	1'773	1'900	1'752	2'047	0.225	0.77672748	1
ENSBTAG00000014082	*CHST9*	20	5	12	17	6	-1.585	0.710402816	1
ENSBTAG00000014083	*AMIGO1*	963	1'192	1'105	834	1'376	0.723	0.378355991	1
ENSBTAG00000014087	*SUV420H2*	635	826	752	550	954	0.794	0.349041481	1
ENSBTAG00000014088	*MBNL3*	10	5	7	9	6	-0.585	0.94357809	1
ENSBTAG00000014089	*MDS018*	538	524	535	466	605	0.377	0.668315769	1
ENSBTAG00000014090	*BT.45843*	574	756	685	497	873	0.812	0.342973427	1
ENSBTAG00000014091	*ARHGEF3*	1'687	1'286	1'473	1'461	1'485	0.023	0.976944734	1
ENSBTAG00000014092	*CELF6*	118	83	99	102	96	-0.093	0.952798265	1
ENSBTAG00000014093	*IDH2*	4'232	5'228	4'851	3'665	6'037	0.720	0.352539667	1
ENSBTAG00000014095	*UCHL5IP*	1'162	1'202	1'197	1'006	1'388	0.464	0.568762918	1
ENSBTAG00000014096	*C14ORF135*	2'402	941	1'583	2'080	1'087	-0.937	0.245203034	1
ENSBTAG00000014099	*YTHDC2*	1'686	578	1'064	1'460	667	-1.129	0.173123451	1
ENSBTAG00000014100	*BAIAP3*	336	680	538	291	785	1.432	0.108227506	1
ENSBTAG00000014101	*C25H16ORF42*	655	692	683	567	799	0.494	0.562926068	1
ENSBTAG00000014102	*BT.101639*	1'179	1'158	1'179	1'021	1'337	0.389	0.632841463	1
ENSBTAG00000014103	*SH3GL2*	470	123	275	407	142	-1.519	0.129016691	1
ENSBTAG00000014104	*protein_coding*	59	66	64	51	76	0.577	0.715083597	1
ENSBTAG00000014105	*SRPR*	4'560	5'362	5'070	3'949	6'192	0.649	0.401347481	1
ENSBTAG00000014106	*IPO8*	1'589	1'324	1'452	1'376	1'529	0.152	0.850214161	1
ENSBTAG00000014111	*INPP4B*	148	54	95	128	62	-1.040	0.438326292	1
ENSBTAG00000014112	*EXOC4*	1'796	2'344	2'131	1'555	2'707	0.799	0.31346458	1
ENSBTAG00000014113	*CCL8*	116	20	62	100	23	-2.121	0.193870182	1
ENSBTAG00000014114	*KLHL26*	173	260	225	150	300	1.003	0.33130408	1
ENSBTAG00000014117	*CCDC9*	1'231	1'959	1'664	1'066	2'262	1.085	0.177847929	1
ENSBTAG00000014118	*GNPTG*	2'384	1'470	1'881	2'065	1'697	-0.283	0.722909666	1
ENSBTAG00000014119	*PRKCZ*	979	729	845	848	842	-0.010	0.992486174	1
ENSBTAG00000014122	*FOXRED1*	1'463	1'188	1'319	1'267	1'372	0.115	0.887379853	1
ENSBTAG00000014123	*ZNF507*	264	259	264	229	299	0.387	0.697067054	1
ENSBTAG00000014124	*MLLT3*	1'070	783	915	927	904	-0.035	0.96806614	1
ENSBTAG00000014126	*UNKL*	422	445	440	365	514	0.492	0.586822294	1
ENSBTAG00000014127	*BT.104982*	392	149	256	339	172	-0.981	0.329419474	1
ENSBTAG00000014129	*BT.105383*	2'002	2'206	2'141	1'734	2'547	0.555	0.48249894	1
ENSBTAG00000014130	*COX6C*	6'647	4'192	5'298	5'756	4'841	-0.250	0.746388937	1
ENSBTAG00000014132	*SNED1*	156	55	99	135	64	-1.089	0.409942825	1
ENSBTAG00000014133	*protein_coding*	1	0	0	1	0		1	1
ENSBTAG00000014136	*C16H1orf86*	628	572	602	544	660	0.280	0.746670111	1

Ensembl gene ID	geneName	counts wildtype horn bud	counts polled horn bud	baseMean	baseMean wildtype horn bud	baseMean polled horn bud	log2FoldChange	pval	padj
ENSBTAG00000014137	*CEP164*	1'066	1'199	1'154	923	1'384	0.585	0.47412213	1
ENSBTAG00000014138	*MTM1*	208	151	177	180	174	-0.047	0.972251637	1
ENSBTAG00000014140	*ATP1B3*	3'540	1'844	2'597	3'066	2'129	-0.526	0.503686003	1
ENSBTAG00000014143	*ASB5*	7	55	35	6	64	3.389	0.130273065	1
ENSBTAG00000014144	*CCDC113*	7	0	3	6	0		0.769774961	1
ENSBTAG00000014145	*SKOR1*	1	1	1	1	1	0.415	1	1
ENSBTAG00000014146	*SPCS3*	576	268	404	499	309	-0.689	0.453957211	1
ENSBTAG00000014147	*KLKBL4*	8	0	3	7	0		0.72953145	1
ENSBTAG00000014149	*BT.61865*	188	436	333	163	503	1.629	0.091310142	1
ENSBTAG00000014150	*C11H9orf16*	1'819	1'568	1'693	1'575	1'811	0.201	0.801042186	1
ENSBTAG00000014151	*BT.49434*	691	318	483	598	367	-0.705	0.431349445	1
ENSBTAG00000014153	*PKNOX1*	510	613	575	442	708	0.680	0.435597021	1
ENSBTAG00000014154	*NKG2A*	1	0	0	1	0		1	1
ENSBTAG00000014156	*BT.70677*	1'962	678	1'241	1'699	783	-1.118	0.1728691	1
ENSBTAG00000014158	*BT.36651*	6'273	3'022	4'461	5'433	3'490	-0.639	0.410599643	1
ENSBTAG00000014159	*BT.52587*	429	611	539	372	706	0.925	0.294457885	1
ENSBTAG00000014161	*ARMC10*	2'110	1'486	1'772	1'827	1'716	-0.091	0.910541223	1
ENSBTAG00000014162	*BT.91151*	43	40	42	37	46	0.311	0.879087605	1
ENSBTAG00000014166	*POLR1B*	1'924	1'483	1'689	1'666	1'712	0.039	0.960656994	1
ENSBTAG00000014167	*BT.21603*	1'306	1'997	1'718	1'131	2'306	1.028	0.200800684	1
ENSBTAG00000014169	*PCDH19*	33	72	56	29	83	1.541	0.355282107	1
ENSBTAG00000014170	*MAP7D3*	1'450	1'068	1'244	1'256	1'233	-0.026	0.976152867	1
ENSBTAG00000014171	*NAPEPLD*	739	358	527	640	413	-0.631	0.475925998	1
ENSBTAG00000014172	*FGD2*	137	65	97	119	75	-0.661	0.621114431	1
ENSBTAG00000014175	*STRAP*	5'964	3'549	4'632	5'165	4'098	-0.334	0.666490319	1
ENSBTAG00000014177	*C6*	99	4	45	86	5	-4.214	0.044883223	1
ENSBTAG00000014178	*BT.24515*	304	310	311	263	358	0.443	0.644690329	1
ENSBTAG00000014181	*RNF111*	2'719	1'394	1'982	2'355	1'610	-0.549	0.489748183	1
ENSBTAG00000014182	*BT.63787*	20'052	16'646	18'293	17'366	19'221	0.146	0.846821055	1
ENSBTAG00000014186	*NCOA5*	1'850	2'005	1'959	1'602	2'315	0.531	0.503018618	1
ENSBTAG00000014187	*BT.37213*	54	42	48	47	48	0.052	0.989137037	1
ENSBTAG00000014189	*RGS6*	36	19	27	31	22	-0.507	0.84539111	1
ENSBTAG00000014191	*BT.106212*	10'095	8'494	9'275	8'743	9'808	0.166	0.827735819	1
ENSBTAG00000014192	*LRRTM2*	124	110	117	107	127	0.242	0.849320858	1
ENSBTAG00000014194	*ADARB2*	17	1	8	15	1	-3.672	0.555170792	1
ENSBTAG00000014197	*ATA3*	92	147	125	80	170	1.091	0.370019495	1
ENSBTAG00000014199	*TBX21*	6	1	3	5	1	-2.170	0.885240772	1
ENSBTAG00000014202	*BT.58641*	2'726	1'748	2'190	2'361	2'018	-0.226	0.775439055	1
ENSBTAG00000014204	*S100A16*	5'541	4'327	4'898	4'799	4'996	0.058	0.939387255	1
ENSBTAG00000014205	*PRKAR2A*	797	759	783	690	876	0.345	0.682535155	1
ENSBTAG00000014207	*ADAMTS10*	5'601	5'826	5'789	4'851	6'727	0.472	0.539899351	1
ENSBTAG00000014208	*RPL35A*	24'607	10'153	16'517	21'310	11'724	-0.862	0.260967041	1
ENSBTAG00000014211	*FOXP4*	936	1'730	1'404	811	1'998	1.301	0.111001909	1
ENSBTAG00000014215	*BT.44090*	577	761	689	500	879	0.814	0.341493579	1
ENSBTAG00000014217	*HHEX*	320	85	188	277	98	-1.497	0.170713288	1
ENSBTAG00000014218	*DKK1*	795	692	744	688	799	0.215	0.799995477	1
ENSBTAG00000014220	*protein_coding*	20	1	9	17	1	-3.907	0.484363729	1
ENSBTAG00000014221	*RSG1*	435	537	498	377	620	0.719	0.41886527	1
ENSBTAG00000014223	*RAN*	1'560	1'055	1'285	1'351	1'218	-0.149	0.855245913	1
ENSBTAG00000014224	*IGSF9B*	16	27	23	14	31	1.170	0.661901171	1
ENSBTAG00000014225	*SLC5A9*	57	54	56	49	62	0.337	0.845381871	1
ENSBTAG00000014226	*BT.49114*	23'742	20'693	22'228	20'561	23'894	0.217	0.775118903	1
ENSBTAG00000014227	*BT.25129*	2'419	1'662	2'007	2'095	1'919	-0.126	0.874260695	1
ENSBTAG00000014228	*BT.70823*	167	64	109	145	74	-0.969	0.448016314	1
ENSBTAG00000014229	*SYT17*	798	342	543	691	395	-0.807	0.360030938	1
ENSBTAG00000014230	*BT.11497*	779	505	629	675	583	-0.210	0.809236798	1
ENSBTAG00000014232	*BT.77813*	98	22	55	85	25	-1.740	0.304866026	1
ENSBTAG00000014233	*CCT8*	1'473	2'010	1'798	1'276	2'321	0.863	0.280077901	1
ENSBTAG00000014235	*C3H1orf212*	1'046	1'039	1'053	906	1'200	0.405	0.621591072	1
ENSBTAG00000014237	*CYTH4*	359	229	288	311	264	-0.234	0.813806824	1
ENSBTAG00000014238	*ATP6V1E1*	4'210	3'785	4'008	3'646	4'371	0.262	0.735287713	1
ENSBTAG00000014239	*CCNB1*	301	492	414	261	568	1.124	0.22131868	1
ENSBTAG00000014246	*CENPH*	284	264	275	246	305	0.310	0.75403936	1
ENSBTAG00000014247	*FAM83F*	141	295	231	122	341	1.480	0.152764528	1
ENSBTAG00000014248	*MIER3*	636	618	632	551	714	0.374	0.664665024	1
ENSBTAG00000014249	*BT.106092*	9'608	5'455	7'310	8'321	6'299	-0.402	0.601524168	1
ENSBTAG00000014250	*ZC3HAV1L*	367	311	338	318	359	0.176	0.853712472	1
ENSBTAG00000014251	*MMP20*	0	1	1	0	1	Inf	0.993540919	1
ENSBTAG00000014252	*SPTLC3*	39	41	41	34	47	0.487	0.806665995	1
ENSBTAG00000014253	*TBC1D22B*	835	928	897	723	1'072	0.567	0.49560396	1
ENSBTAG00000014255	*BT.25465*	2'012	1'383	1'670	1'742	1'597	-0.126	0.876082863	1
ENSBTAG00000014259	*KCTD1*	1'599	1'275	1'429	1'385	1'472	0.088	0.912691157	1

Ensembl gene ID	geneName	counts wildtype horn bud	counts polled horn bud	baseMean	baseMean wildtype horn bud	baseMean polled horn bud	log2FoldChange	pval	padj
ENSBTAG00000014261	*TARS*	5'897	4'182	4'968	5'107	4'829	-0.081	0.91753927	1
ENSBTAG00000014262	*BZW2*	3'697	3'228	3'465	3'202	3'727	0.219	0.777429135	1
ENSBTAG00000014265	*SREBF2*	4'032	7'326	5'976	3'492	8'459	1.277	0.101600733	1
ENSBTAG00000014267	*ZCCHC14*	672	428	538	582	494	-0.236	0.790494888	1
ENSBTAG00000014269	*SCARB1*	3'361	2'211	2'732	2'911	2'553	-0.189	0.809990248	1
ENSBTAG00000014270	*UNC5B*	1'845	897	1'317	1'598	1'036	-0.625	0.441106913	1
ENSBTAG00000014272	*MUT*	3'878	2'125	2'906	3'358	2'454	-0.453	0.563268909	1
ENSBTAG00000014273	*NRF1*	598	575	591	518	664	0.358	0.679969937	1
ENSBTAG00000014278	*TBX2*	768	234	468	665	270	-1.300	0.152033145	1
ENSBTAG00000014284	*ALPK2*	6	30	20	5	35	2.737	0.35564536	1
ENSBTAG00000014286	*BT.36147*	1'304	932	1'103	1'129	1'076	-0.070	0.934131889	1
ENSBTAG00000014288	*GPRC5D*	6	4	5	5	5	-0.170	1	1
ENSBTAG00000014289	*ZFYVE9*	2'227	2'314	2'300	1'929	2'672	0.470	0.550343572	1
ENSBTAG00000014291	*WNT2B*	296	136	207	256	157	-0.707	0.502866595	1
ENSBTAG00000014294	*ST7L*	1'830	798	1'253	1'585	921	-0.782	0.337416822	1
ENSBTAG00000014295	*CAPZA1*	12'428	8'090	10'052	10'763	9'342	-0.204	0.789959714	1
ENSBTAG00000014296	*NCCRP1*	31	2	15	27	2	-3.539	0.360208319	1
ENSBTAG00000014297	*MOV10*	2'102	1'878	1'994	1'820	2'169	0.252	0.749535044	1
ENSBTAG00000014299	*RHOC*	6'878	6'435	6'694	5'957	7'430	0.319	0.677479306	1
ENSBTAG00000014300	*BT.88761*	4	4	4	3	5	0.415	0.994759363	1
ENSBTAG00000014302	*TOMM70A*	3'817	3'396	3'613	3'306	3'921	0.246	0.750580168	1
ENSBTAG00000014304	*TMEM47*	496	378	433	430	436	0.023	0.981828899	1
ENSBTAG00000014306	*DOPEY2*	682	530	601	591	612	0.051	0.954135586	1
ENSBTAG00000014310	*HEATR5B*	2'024	1'158	1'545	1'753	1'337	-0.391	0.627150466	1
ENSBTAG00000014313	*BT.53596*	308	424	378	267	490	0.876	0.345612694	1
ENSBTAG00000014314	*HPS1*	1'359	1'559	1'489	1'177	1'800	0.613	0.445900318	1
ENSBTAG00000014315	*PBLD*	53	10	29	46	12	-1.991	0.400294731	1
ENSBTAG00000014316	*HNRNPH3*	8'514	4'481	6'274	7'373	5'174	-0.511	0.507467673	1
ENSBTAG00000014318	*SLC33A1*	1'871	1'094	1'442	1'620	1'263	-0.359	0.656384388	1
ENSBTAG00000014319	*BT.92539*	136	81	106	118	94	-0.333	0.801231116	1
ENSBTAG00000014320	*BT.55613*	587	184	360	508	212	-1.259	0.182703398	1
ENSBTAG00000014322	*KIAA1467*	528	471	501	457	544	0.250	0.778569566	1
ENSBTAG00000014324	*ANTXR2*	6'079	5'838	6'003	5'265	6'741	0.357	0.642542091	1
ENSBTAG00000014325	*MGC166151*	1'054	609	808	913	703	-0.376	0.655410486	1
ENSBTAG00000014326	*CDCA8*	891	964	942	772	1'113	0.529	0.523669403	1
ENSBTAG00000014328	*BT.27532*	18	17	18	16	20	0.333	0.929998919	1
ENSBTAG00000014329	*BT.61986*	2	2	2	2	2	0.415	1	1
ENSBTAG00000014331	*BT.48960*	537	612	586	465	707	0.604	0.487859374	1
ENSBTAG00000014332	*BT.63430*	1	0	0	1	0		1	1
ENSBTAG00000014333	*SLC10A3*	702	897	822	608	1'036	0.769	0.360666685	1
ENSBTAG00000014334	*ZFYVE26*	895	1'213	1'088	775	1'401	0.854	0.299567435	1
ENSBTAG00000014335	*BT.65130*	110	116	115	95	134	0.492	0.6954648	1
ENSBTAG00000014336	*C10ORF26*	471	474	478	408	547	0.424	0.635037769	1
ENSBTAG00000014337	*EIF2S3*	3'845	2'977	3'384	3'330	3'438	0.046	0.952633535	1
ENSBTAG00000014339	*BT.93854*	2'238	1'069	1'586	1'938	1'234	-0.651	0.417882494	1
ENSBTAG00000014343	*BT.48915*	1'072	890	978	928	1'028	0.147	0.859393912	1
ENSBTAG00000014349	*RNF126*	1'419	1'123	1'263	1'229	1'297	0.078	0.924083431	1
ENSBTAG00000014351	*EIF2AK4*	3'417	2'051	2'664	2'959	2'368	-0.321	0.682534783	1
ENSBTAG00000014353	*SRP14*	3'502	1'925	2'628	3'033	2'223	-0.448	0.568400667	1
ENSBTAG00000014354	*FXYD6*	6'812	7'011	6'997	5'899	8'096	0.457	0.551985195	1
ENSBTAG00000014355	*MYCBPAP*	154	95	122	133	110	-0.282	0.82348477	1
ENSBTAG00000014357	*SDC2*	4'914	3'673	4'248	4'256	4'241	-0.005	0.995986678	1
ENSBTAG00000014358	*FAM176B*	1'338	1'579	1'491	1'159	1'823	0.654	0.416352497	1
ENSBTAG00000014359	*C16H1orf187*	181	294	248	157	339	1.115	0.270275467	1
ENSBTAG00000014361	*FAM160B2*	1'042	1'800	1'490	902	2'078	1.204	0.138257562	1
ENSBTAG00000014365	*protein_coding*	11	3	6	10	3	-1.459	0.83622464	1
ENSBTAG00000014366	*PRPF19*	3'533	5'265	4'570	3'060	6'079	0.991	0.203321947	1
ENSBTAG00000014367	*PRKX*	148	162	158	128	187	0.545	0.630023756	1
ENSBTAG00000014368	*SLAMF6*	21	2	10	18	2	-2.977	0.538317156	1
ENSBTAG00000014370	*NETO2*	148	11	70	128	13	-3.335	0.041149326	1
ENSBTAG00000014371	*bta-mir-671*	1'757	2'035	1'936	1'522	2'350	0.627	0.429884591	1
ENSBTAG00000014372	*SMARCD3*	3'964	4'274	4'184	3'433	4'935	0.524	0.499083256	1
ENSBTAG00000014373	*CETN3*	2'969	1'607	2'213	2'571	1'856	-0.471	0.551792016	1
ENSBTAG00000014374	*CARD6*	455	358	404	394	413	0.069	0.941875303	1
ENSBTAG00000014375	*BT.43570*	414	310	358	359	358	-0.002	1	1
ENSBTAG00000014376	*ANKRD13A*	2'396	1'507	1'908	2'075	1'740	-0.254	0.749941834	1
ENSBTAG00000014377	*BT.45569*	27'034	30'667	29'412	23'412	35'411	0.597	0.433065264	1
ENSBTAG00000014380	*MCM2*	2'847	6'029	4'714	2'466	6'962	1.498	0.057062667	1
ENSBTAG00000014382	*KANK4*	947	859	906	820	992	0.274	0.741424527	1
ENSBTAG00000014384	*ZDHHC22*	14	0	6	12	0		0.520471303	1
ENSBTAG00000014387	*PRKAB2*	191	50	112	165	58	-1.519	0.234767952	1
ENSBTAG00000014388	*EIF3I*	6'110	6'736	6'535	5'291	7'778	0.556	0.469970664	1

Ensembl gene ID	geneName	counts wildtype horn bud	counts polled horn bud	baseMean	baseMean wildtype horn bud	baseMean polled horn bud	log2FoldChange	pval	padj
ENSBTAG00000014389	*SP4*	367	245	300	318	283	-0.168	0.865593055	1
ENSBTAG00000014390	*MTMR9*	2'352	2'007	2'177	2'037	2'317	0.186	0.813143743	1
ENSBTAG00000014391	*CRB2*	32	2	15	28	2	-3.585	0.345807076	1
ENSBTAG00000014392	*ACTR10*	4'038	2'643	3'274	3'497	3'052	-0.196	0.801713861	1
ENSBTAG00000014393	*CLK2*	4'907	3'451	4'117	4'250	3'985	-0.093	0.905554623	1
ENSBTAG00000014395	*GLRA1*	3	0	1	3	0		0.936647693	1
ENSBTAG00000014396	*BT.101967*	3'432	2'473	2'914	2'972	2'856	-0.058	0.942182826	1
ENSBTAG00000014397	*BT.87808*	29	4	15	25	5	-2.443	0.497961729	1
ENSBTAG00000014399	*QSER1*	2'445	1'521	1'937	2'117	1'756	-0.270	0.734642666	1
ENSBTAG00000014400	*E2F2*	35	119	84	30	137	2.181	0.129069596	1
ENSBTAG00000014401	*SORBS3*	6'425	4'996	5'667	5'564	5'769	0.052	0.94556111	1
ENSBTAG00000014402	*GIMAP8*	115	50	79	100	58	-0.787	0.586330824	1
ENSBTAG00000014405	*OSBPL6*	1'373	725	1'013	1'189	837	-0.506	0.540158548	1
ENSBTAG00000014406	*LATS2*	799	692	746	692	799	0.208	0.806585445	1
ENSBTAG00000014407	*INO80D*	24	25	25	21	29	0.474	0.860158361	1
ENSBTAG00000014409	*YSK4*	14	0	6	12	0		0.520471303	1
ENSBTAG00000014411	*C5H12orf23*	7'109	4'644	5'760	6'157	5'362	-0.199	0.796499079	1
ENSBTAG00000014415	*ZNF653*	798	843	832	691	973	0.494	0.555295186	1
ENSBTAG00000014417	*BT.45558*	594	1'196	948	514	1'381	1.425	0.090295882	1
ENSBTAG00000014418	*FHIT*	98	72	84	85	83	-0.030	0.993857432	1
ENSBTAG00000014420	*AGER*	882	642	753	764	741	-0.043	0.961698454	1
ENSBTAG00000014421	*PBX2*	3'308	3'373	3'380	2'865	3'895	0.443	0.568950168	1
ENSBTAG00000014422	*MTMR10*	1'422	1'076	1'237	1'231	1'242	0.013	0.98787289	1
ENSBTAG00000014423	*protein_coding*	40'907	24'698	31'973	35'427	28'519	-0.313	0.681201074	1
ENSBTAG00000014424	*DDN*	109	18	58	94	21	-2.183	0.195565566	1
ENSBTAG00000014426	*PRKAG1*	2'172	1'771	1'963	1'881	2'045	0.121	0.87887494	1
ENSBTAG00000014429	*MLL2*	3'609	7'424	5'849	3'125	8'572	1.456	0.06313889	1
ENSBTAG00000014430	*CDC26*	1'271	746	981	1'101	861	-0.354	0.669688397	1
ENSBTAG00000014432	*HNMT*	1'370	456	856	1'186	527	-1.172	0.165154	1
ENSBTAG00000014433	*UBE2G1*	2'847	2'207	2'507	2'466	2'548	0.048	0.951388104	1
ENSBTAG00000014434	*BT.25908*	1'150	1'520	1'376	996	1'755	0.817	0.312885368	1
ENSBTAG00000014435	*TCF19*	1'299	1'121	1'210	1'125	1'294	0.202	0.803418187	1
ENSBTAG00000014438	*SS18L1*	702	352	507	608	406	-0.581	0.513513231	1
ENSBTAG00000014439	*KMO*	1	0	0	1	0		1	1
ENSBTAG00000014440	*PSMA4*	3'075	2'502	2'776	2'663	2'889	0.118	0.880256152	1
ENSBTAG00000014441	*BT.19988*	55	24	38	48	28	-0.781	0.702128407	1
ENSBTAG00000014442	*GTPBP5*	356	442	409	308	510	0.727	0.427073992	1
ENSBTAG00000014443	*BT.61126*	2	0	1	2	0		0.974934741	1
ENSBTAG00000014447	*RPS6KA1*	559	688	639	484	794	0.715	0.407192959	1
ENSBTAG00000014448	*VKORC1L1*	188	122	152	163	141	-0.209	0.860404568	1
ENSBTAG00000014450	*NOSIP*	1'880	1'951	1'940	1'628	2'253	0.469	0.55457588	1
ENSBTAG00000014451	*YEATS2*	3'552	2'285	2'857	3'076	2'638	-0.221	0.7778893	1
ENSBTAG00000014453	*PLK1*	1'165	1'172	1'181	1'009	1'353	0.424	0.602955921	1
ENSBTAG00000014454	*PRR12*	2'977	4'743	4'027	2'578	5'477	1.087	0.164572696	1
ENSBTAG00000014455	*PDZD2*	1'469	603	984	1'272	696	-0.870	0.295131816	1
ENSBTAG00000014456	*ERN2*	0	2	1	0	2	Inf	0.939077559	1
ENSBTAG00000014458	*HEATR7A*	4'944	5'100	5'085	4'282	5'889	0.460	0.551049486	1
ENSBTAG00000014459	*MAP6D1*	615	312	446	533	360	-0.564	0.533385889	1
ENSBTAG00000014460	*PARL*	2'480	1'669	2'037	2'148	1'927	-0.156	0.844476336	1
ENSBTAG00000014461	*ANAPC13*	1'773	1'307	1'522	1'535	1'509	-0.025	0.976849861	1
ENSBTAG00000014463	*CAMK2D*	3'912	1'927	2'806	3'388	2'225	-0.607	0.439667184	1
ENSBTAG00000014464	*SEC14L3*	7	2	4	6	2	-1.392	0.903936064	1
ENSBTAG00000014466	*VTCN1*	30	16	22	26	18	-0.492	0.867667507	1
ENSBTAG00000014467	*GLIS3*	76	69	73	66	80	0.276	0.858255635	1
ENSBTAG00000014468	*AP1S1*	2'104	1'897	2'006	1'822	2'190	0.266	0.736909628	1
ENSBTAG00000014469	*NBEAL1*	166	63	108	144	73	-0.983	0.442958316	1
ENSBTAG00000014470	*BT.52387*	32	6	17	28	7	-2.000	0.533547462	1
ENSBTAG00000014471	*BT.75887*	7'753	8'790	8'432	6'714	10'150	0.596	0.437147799	1
ENSBTAG00000014472	*LCMT2*	167	129	147	145	149	0.043	0.976343907	1
ENSBTAG00000014474	*API5*	1'881	1'752	1'826	1'629	2'023	0.313	0.693923816	1
ENSBTAG00000014476	*BT.28556*	488	340	408	423	393	-0.106	0.910765561	1
ENSBTAG00000014478	*ZSCAN29*	791	370	556	685	427	-0.681	0.438161513	1
ENSBTAG00000014479	*TUBGCP4*	2'009	1'171	1'546	1'740	1'352	-0.364	0.65103994	1
ENSBTAG00000014482	*FAF1*	416	418	421	360	483	0.422	0.643158343	1
ENSBTAG00000014484	*TMEM26*	261	321	298	226	371	0.714	0.461256241	1
ENSBTAG00000014485	*POF1B*	239	126	176	207	145	-0.509	0.644910884	1
ENSBTAG00000014486	*BT.63059*	35	7	19	30	8	-1.907	0.525088239	1
ENSBTAG00000014488	*CDH7*	884	827	860	766	955	0.319	0.70256573	1
ENSBTAG00000014490	*DDX39B*	16'779	13'874	15'276	14'531	16'020	0.141	0.85284241	1
ENSBTAG00000014491	*ATP6V1G2*	302	98	187	262	113	-1.209	0.266148439	1
ENSBTAG00000014492	*NFKBIL1*	596	600	604	516	693	0.425	0.623999572	1
ENSBTAG00000014493	*BT.58152*	2'222	1'667	1'925	1'924	1'925	0.000	0.99964833	1

Ensembl gene ID	geneName	counts wildtype horn bud	counts polled horn bud	baseMean	baseMean wildtype horn bud	baseMean polled horn bud	log2FoldChange	pval	padj
ENSBTAG00000014494	*RNASET2*	2'432	2'229	2'340	2'106	2'574	0.289	0.712798074	1
ENSBTAG00000014495	*BT.24497*	838	684	758	726	790	0.122	0.885761092	1
ENSBTAG00000014496	*BT.106551*	11	7	9	10	8	-0.237	0.988863286	1
ENSBTAG00000014497	*GPR31*	2	0	1	2	0		0.974934741	1
ENSBTAG00000014498	*UNC93A*	20	3	10	17	3	-2.322	0.618209332	1
ENSBTAG00000014501	*FERMT2*	9'936	5'164	7'284	8'605	5'963	-0.529	0.491690603	1
ENSBTAG00000014502	*BRAT1*	734	618	675	636	714	0.167	0.845923964	1
ENSBTAG00000014503	*IQCE*	1'521	1'993	1'809	1'317	2'301	0.805	0.313395904	1
ENSBTAG00000014505	*NGRN*	4'562	2'988	3'701	3'951	3'450	-0.195	0.802057502	1
ENSBTAG00000014506	*ZNF503*	1'245	1'704	1'523	1'078	1'968	0.868	0.281651336	1
ENSBTAG00000014508	*FBXO40*	8	4	6	7	5	-0.585	0.95915875	1
ENSBTAG00000014511	*FLJ20584*	273	190	228	236	219	-0.108	0.92119143	1
ENSBTAG00000014512	*WDR19*	4'989	2'509	3'609	4'321	2'897	-0.577	0.459378975	1
ENSBTAG00000014514	*SEC1*	25	48	39	22	55	1.356	0.493814537	1
ENSBTAG00000014515	*RFC1*	3'573	1'769	2'568	3'094	2'043	-0.599	0.446494363	1
ENSBTAG00000014517	*BT.25033*	5	1	3	4	1	-1.907	0.9188325	1
ENSBTAG00000014518	*RPL9*	8'120	7'418	7'799	7'032	8'566	0.285	0.709963681	1
ENSBTAG00000014520	*LIAS*	1'213	503	816	1'050	581	-0.855	0.311039623	1
ENSBTAG00000014521	*UGDH*	3'187	2'809	3'002	2'760	3'244	0.233	0.764993466	1
ENSBTAG00000014522	*C6H4orf34*	1'339	1'118	1'225	1'160	1'291	0.155	0.848977873	1
ENSBTAG00000014526	*MOB3A*	1'392	2'064	1'794	1'206	2'383	0.983	0.219645141	1
ENSBTAG00000014529	*GBP4*	14	3	8	12	3	-1.807	0.758305361	1
ENSBTAG00000014530	*MGC139448*	840	443	619	727	512	-0.508	0.557773313	1
ENSBTAG00000014533	*KEL*	1	0	0	1	0		1	1
ENSBTAG00000014534	*EEF1A1*	172'033	183'647	180'521	148'985	212'057	0.509	0.502039055	1
ENSBTAG00000014536	*SFXN1*	793	768	787	687	887	0.369	0.661371795	1
ENSBTAG00000014537	*PLEKHN1*	221	82	143	191	95	-1.015	0.385506831	1
ENSBTAG00000014540	*C16H1orf170*	113	63	85	98	73	-0.428	0.763526261	1
ENSBTAG00000014541	*BT.18780*	2'024	3'498	2'896	1'753	4'039	1.204	0.127619006	1
ENSBTAG00000014543	*PLEX2*	176	388	300	152	448	1.556	0.113035491	1
ENSBTAG00000014546	*BT.49447*	93	18	51	81	21	-1.954	0.2704511	1
ENSBTAG00000014547	*BT.23217*	77	217	159	67	251	1.910	0.097849117	1
ENSBTAG00000014548	*FAM129A*	1'050	824	930	909	951	0.065	0.937775034	1
ENSBTAG00000014549	*GNL3*	4'159	1'984	2'946	3'602	2'291	-0.653	0.405065917	1
ENSBTAG00000014550	*GLT8D1*	10'231	5'286	7'482	8'860	6'104	-0.538	0.484622907	1
ENSBTAG00000014551	*TNFAIP8*	1'344	1'013	1'167	1'164	1'170	0.007	0.993537544	1
ENSBTAG00000014553	*ATP6V0D1*	5'616	8'949	7'599	4'864	10'333	1.087	0.160226234	1
ENSBTAG00000014554	*BT.61987*	1'066	786	915	923	908	-0.025	0.97859045	1
ENSBTAG00000014555	*CASS4*	17	13	15	15	15	0.028	1	1
ENSBTAG00000014556	*AGRP*	2	1	1	2	1	-0.585	1	1
ENSBTAG00000014558	*DDX21*	1'720	1'618	1'679	1'490	1'868	0.327	0.681921095	1
ENSBTAG00000014560	*HLX*	433	337	382	375	389	0.053	0.956187227	1
ENSBTAG00000014561	*KBP*	2'353	1'291	1'764	2'038	1'491	-0.451	0.572230343	1
ENSBTAG00000014564	*XXYLT1*	541	271	391	469	313	-0.582	0.528951982	1
ENSBTAG00000014567	*BT.105316*	6'277	2'223	4'001	5'436	2'567	-1.083	0.166602363	1
ENSBTAG00000014574	*WDR96*	64	6	31	55	7	-3.000	0.203436893	1
ENSBTAG00000014575	*COL4A5*	3'446	3'823	3'699	2'984	4'414	0.565	0.467391513	1
ENSBTAG00000014576	*GUCY1A1*	319	92	191	276	106	-1.379	0.203702551	1
ENSBTAG00000014579	*MRPL34*	773	735	759	669	849	0.342	0.685425314	1
ENSBTAG00000014580	*FCRLB*	4	4	4	3	5	0.415	0.994759363	1
ENSBTAG00000014581	*BT.92106*	85	43	62	74	50	-0.568	0.72572557	1
ENSBTAG00000014583	*CALM*	11'460	12'613	12'244	9'925	14'564	0.553	0.469075139	1
ENSBTAG00000014585	*BT.45031*	22	84	58	19	97	2.348	0.161811258	1
ENSBTAG00000014587	*CAGE1*	167	81	119	145	94	-0.629	0.612788935	1
ENSBTAG00000014588	*RSL1D1*	2'294	2'036	2'169	1'987	2'351	0.243	0.757888012	1
ENSBTAG00000014592	*LACE1*	212	130	167	184	150	-0.291	0.798131355	1
ENSBTAG00000014593	*protein_coding*	184	172	179	159	199	0.318	0.772802026	1
ENSBTAG00000014595	*LIG1*	2'895	3'929	3'522	2'507	4'537	0.856	0.273413141	1
ENSBTAG00000014597	*C19ORF68*	162	132	146	140	152	0.120	0.922375589	1
ENSBTAG00000014598	*BPTF*	3'720	2'325	2'953	3'222	2'685	-0.263	0.737109031	1
ENSBTAG00000014599	*LRRC66*	31	26	28	27	30	0.161	0.959438006	1
ENSBTAG00000014600	*BT.24461*	95	39	64	82	45	-0.869	0.581752366	1
ENSBTAG00000014601	*SGCB*	3'213	1'191	2'079	2'783	1'375	-1.017	0.202225501	1
ENSBTAG00000014602	*CAMTA1*	516	240	362	447	277	-0.689	0.461957863	1
ENSBTAG00000014603	*BT.35951*	484	432	459	419	499	0.251	0.780615977	1
ENSBTAG00000014605	*BT.44079*	329	368	355	285	425	0.577	0.538288345	1
ENSBTAG00000014607	*EXOSC4*	528	635	595	457	733	0.681	0.433090537	1
ENSBTAG00000014608	*KDM1B*	771	485	614	668	560	-0.254	0.77099391	1
ENSBTAG00000014609	*BT.41853*	1'948	1'041	1'445	1'687	1'202	-0.489	0.544532952	1
ENSBTAG00000014610	*GPAA1*	3'695	3'267	3'486	3'200	3'772	0.237	0.759600272	1
ENSBTAG00000014611	*SORL1*	1'865	1'307	1'562	1'615	1'509	-0.098	0.904150427	1
ENSBTAG00000014612	*protein_coding*	181	75	122	157	87	-0.856	0.486730026	1

Ensembl gene ID	geneName	counts wildtype horn bud	counts polled horn bud	baseMean	baseMean wildtype horn bud	baseMean polled horn bud	log2FoldChange	pval	padj
ENSBTAG00000014614	*ACTA2*	8'559	3'435	5'689	7'412	3'966	-0.902	0.24459051	1
ENSBTAG00000014615	*BT.41181*	268	134	193	232	155	-0.585	0.586110737	1
ENSBTAG00000014616	*SEPSECS*	135	80	105	117	92	-0.340	0.79751033	1
ENSBTAG00000014619	*WASF3*	498	361	424	431	417	-0.049	0.960463388	1
ENSBTAG00000014620	*FOXI1*	2	2	2	2	2	0.415	1	1
ENSBTAG00000014623	*MCM8*	201	121	157	174	140	-0.317	0.783482139	1
ENSBTAG00000014626	*RARS*	3'843	2'757	3'256	3'328	3'184	-0.064	0.935457914	1
ENSBTAG00000014628	*BT.41610*	14	7	10	12	8	-0.585	0.916699259	1
ENSBTAG00000014633	*ABCD4*	1'613	1'482	1'554	1'397	1'711	0.293	0.714536262	1
ENSBTAG00000014636	*BT.36331*	1'232	320	718	1'067	370	-1.530	0.077201861	1
ENSBTAG00000014638	*TRPC7*	16	0	7	14	0		0.463690882	1
ENSBTAG00000014643	*EEF1D*	14'637	17'603	16'501	12'676	20'326	0.681	0.372650251	1
ENSBTAG00000014644	*DUS2L*	398	449	432	345	518	0.589	0.516238796	1
ENSBTAG00000014645	*CNTN2*	88	103	98	76	119	0.642	0.627906736	1
ENSBTAG00000014646	*MZT2*	454	397	426	393	458	0.221	0.808396844	1
ENSBTAG00000014648	*RPN2*	14'939	7'053	10'541	12'938	8'144	-0.668	0.38428134	1
ENSBTAG00000014649	*CPT2*	1'258	948	1'092	1'089	1'095	0.007	0.99391468	1
ENSBTAG00000014650	*NFATC2IP*	440	369	404	381	426	0.161	0.861819744	1
ENSBTAG00000014651	*SPATA19*	1	0	0	1	0		1	1
ENSBTAG00000014652	*TRPM8*	30	27	29	26	31	0.263	0.922347889	1
ENSBTAG00000014653	*SPNS1*	2'204	3'179	2'790	1'909	3'671	0.943	0.230807824	1
ENSBTAG00000014655	*MYO1A*	122	81	100	106	94	-0.176	0.901130491	1
ENSBTAG00000014659	*BT.38710*	141	129	136	122	149	0.287	0.811599766	1
ENSBTAG00000014660	*WDR53*	589	381	475	510	440	-0.213	0.813629954	1
ENSBTAG00000014661	*CHSY3*	459	171	297	398	197	-1.009	0.300309357	1
ENSBTAG00000014662	*protein_coding*	37	5	19	32	6	-2.472	0.422338869	1
ENSBTAG00000014665	*ADAMTS2*	17'894	11'185	14'206	15'497	12'915	-0.263	0.730994165	1
ENSBTAG00000014666	*GP9*	1	0	0	1	0		1	1
ENSBTAG00000014667	*PAIP2B*	42	39	41	36	45	0.308	0.881916376	1
ENSBTAG00000014668	*NAGK*	3'119	2'775	2'953	2'701	3'204	0.246	0.751905366	1
ENSBTAG00000014669	*NDUFA12*	593	146	341	514	169	-1.607	0.094728026	1
ENSBTAG00000014670	*BEX5*	957	509	708	829	588	-0.496	0.561478557	1
ENSBTAG00000014674	*CHRM2*	50	1	22	43	1	-5.229	0.119305888	1
ENSBTAG00000014675	*PTCD2*	1'074	822	940	930	949	0.029	0.972550641	1
ENSBTAG00000014676	*SHROOM1*	318	387	361	275	447	0.698	0.454971623	1
ENSBTAG00000014677	*TADA2A*	3'568	2'219	2'826	3'090	2'562	-0.270	0.730583428	1
ENSBTAG00000014679	*DHX57*	4'570	2'459	3'399	3'958	2'839	-0.479	0.539117591	1
ENSBTAG00000014680	*ZBTB49*	359	313	336	311	361	0.217	0.819749709	1
ENSBTAG00000014682	*AANAT*	2	0	1	2	0		0.974934741	1
ENSBTAG00000014685	*HPRT1*	1'727	1'811	1'793	1'496	2'091	0.484	0.543421212	1
ENSBTAG00000014687	*TP53I13*	2'755	2'694	2'748	2'386	3'111	0.383	0.624588518	1
ENSBTAG00000014689	*CSPP1*	2'707	838	1'656	2'344	968	-1.277	0.114754758	1
ENSBTAG00000014691	*ARFGEF1*	5'547	2'774	4'003	4'804	3'203	-0.585	0.452041643	1
ENSBTAG00000014692	*BT.17691*	386	196	280	334	226	-0.563	0.567592142	1
ENSBTAG00000014693	*TMEM88*	605	326	450	524	376	-0.477	0.5980462	1
ENSBTAG00000014694	*JTB*	2'820	1'792	2'256	2'442	2'069	-0.239	0.762520937	1
ENSBTAG00000014695	*LSMD1*	1'014	847	928	878	978	0.155	0.851685342	1
ENSBTAG00000014696	*BT.106432*	210	116	158	182	134	-0.441	0.699231953	1
ENSBTAG00000014697	*SMARCC2*	6'754	6'406	6'623	5'849	7'397	0.339	0.658867961	1
ENSBTAG00000014698	*CARD11*	350	226	282	303	261	-0.216	0.828712559	1
ENSBTAG00000014699	*FAM81A*	239	52	134	207	60	-1.785	0.141462388	1
ENSBTAG00000014700	*DYNC1LI1*	2'698	2'287	2'489	2'337	2'641	0.177	0.821688269	1
ENSBTAG00000014705	*HES4*	220	182	200	191	210	0.141	0.897091197	1
ENSBTAG00000014707	*ISG15*	143	35	82	124	40	-1.616	0.259136827	1
ENSBTAG00000014710	*RAP1A*	1'541	1'181	1'349	1'335	1'364	0.031	0.969508843	1
ENSBTAG00000014711	*BT.17683*	573	288	414	496	333	-0.577	0.528463957	1
ENSBTAG00000014712	*CLPP*	1'004	1'006	1'016	869	1'162	0.418	0.611831852	1
ENSBTAG00000014713	*RARRES1*	1'629	492	989	1'411	568	-1.312	0.116835303	1
ENSBTAG00000014714	*BT.103306*	2'260	1'092	1'609	1'957	1'261	-0.634	0.429453613	1
ENSBTAG00000014718	*CST6*	471	651	580	408	752	0.882	0.312850697	1
ENSBTAG00000014719	*BT.101239*	1'433	881	1'129	1'241	1'017	-0.287	0.726850071	1
ENSBTAG00000014722	*TSTD2*	1'115	990	1'054	966	1'143	0.243	0.766839803	1
ENSBTAG00000014724	*EIF4A2*	21'635	13'412	17'112	18'736	15'487	-0.275	0.718971141	1
ENSBTAG00000014725	*CD27*	8	1	4	7	1	-2.585	0.818599781	1
ENSBTAG00000014726	*VAMP3*	4'341	2'856	3'529	3'759	3'298	-0.189	0.808725944	1
ENSBTAG00000014727	*RFC4*	824	739	783	714	853	0.258	0.759572848	1
ENSBTAG00000014728	*BT.98677*	1'062	1'368	1'250	920	1'580	0.780	0.337962547	1
ENSBTAG00000014729	*MRPL51*	2'289	1'828	2'047	1'982	2'111	0.091	0.908677694	1
ENSBTAG00000014730	*BT.76801*	2'702	3'780	3'352	2'340	4'365	0.899	0.250551762	1
ENSBTAG00000014731	*G3PDH*	21'066	27'211	24'832	18'244	31'421	0.784	0.304550236	1
ENSBTAG00000014733	*BT.53770*	2'439	2'673	2'599	2'112	3'087	0.547	0.485609	1
ENSBTAG00000014734	*CHD4*	10'486	8'102	9'218	9'081	9'355	0.043	0.954716305	1

Ensembl gene ID	geneName	counts wildtype horn bud	counts polled horn bud	baseMean	baseMean wildtype horn bud	baseMean polled horn bud	log2FoldChange	pval	padj
ENSBTAG00000014735	*HOXA13*	4	6	5	3	7	1.000	0.91481797	1
ENSBTAG00000014737	*VEGFC*	448	216	319	388	249	-0.637	0.506294034	1
ENSBTAG00000014742	*DKFZP434K1815*	2'065	1'813	1'941	1'788	2'093	0.227	0.774058174	1
ENSBTAG00000014744	*TXNDC15*	2'127	988	1'491	1'842	1'141	-0.691	0.391379522	1
ENSBTAG00000014746	*CATSPER3*	14	14	14	12	16	0.415	0.923601843	1
ENSBTAG00000014748	*protein_coding*	1	0	0	1	0		1	1
ENSBTAG00000014749	*ZIC4*	33	22	27	29	25	-0.170	0.960619294	1
ENSBTAG00000014750	*BT.32278*	1'356	636	954	1'174	734	-0.677	0.41509553	1
ENSBTAG00000014751	*ZIC1*	92	127	113	80	147	0.880	0.48306117	1
ENSBTAG00000014752	*AKAP11*	1'296	731	983	1'122	844	-0.411	0.619793598	1
ENSBTAG00000014758	*BT.63013*	3'930	977	2'266	3'403	1'128	-1.593	0.047730839	1
ENSBTAG00000014759	*BT.18030*	3'493	2'428	2'914	3'025	2'804	-0.110	0.889390101	1
ENSBTAG00000014760	*FOXE1*	15	9	12	13	10	-0.322	0.959295425	1
ENSBTAG00000014761	*PGA*	22	28	26	19	32	0.763	0.761718009	1
ENSBTAG00000014762	*ISG20*	6	3	4	5	3	-0.585	0.976586913	1
ENSBTAG00000014764	*CD9*	6'826	6'161	6'513	5'911	7'114	0.267	0.727542653	1
ENSBTAG00000014765	*pseudogene*	164	210	192	142	242	0.772	0.471067667	1
ENSBTAG00000014766	*SRSF1*	21'829	10'116	15'293	18'904	11'681	-0.695	0.364333732	1
ENSBTAG00000014768	*ZNF786*	939	752	841	813	868	0.095	0.910549955	1
ENSBTAG00000014769	*GMEB2*	1'099	1'421	1'296	952	1'641	0.786	0.333537696	1
ENSBTAG00000014771	*BT.54455*	2'838	1'066	1'844	2'458	1'231	-0.998	0.213009488	1
ENSBTAG00000014772	*NUDCD2*	1'147	648	871	993	748	-0.409	0.625372006	1
ENSBTAG00000014773	*BT.12993*	1'122	674	875	972	778	-0.320	0.702281062	1
ENSBTAG00000014774	*ANKRD24*	382	185	272	331	214	-0.631	0.523934188	1
ENSBTAG00000014775	*GUK1*	3'478	2'860	3'157	3'012	3'302	0.133	0.864324438	1
ENSBTAG00000014777	*FBXO6*	898	806	854	778	931	0.259	0.756592219	1
ENSBTAG00000014779	*NISCH*	6'396	9'704	8'372	5'539	11'205	1.016	0.188142543	1
ENSBTAG00000014781	*pseudogene*	1	1	1	1	1	0.415	1	1
ENSBTAG00000014782	*STAB1*	3'779	3'446	3'626	3'273	3'979	0.282	0.716150598	1
ENSBTAG00000014784	*BT.51610*	5'299	7'815	6'807	4'589	9'024	0.976	0.207283846	1
ENSBTAG00000014786	*PBRM1*	2'353	1'629	1'959	2'038	1'881	-0.115	0.8853478	1
ENSBTAG00000014788	*AKAP12*	7'089	4'649	5'754	6'139	5'368	-0.194	0.802140834	1
ENSBTAG00000014790	*ZBTB2*	283	272	280	245	314	0.358	0.716003291	1
ENSBTAG00000014791	*CTH*	17	22	20	15	25	0.787	0.789040458	1
ENSBTAG00000014792	*MAP4K5*	3'214	1'509	2'263	2'783	1'742	-0.676	0.393001765	1
ENSBTAG00000014793	*C1ORF112*	405	325	363	351	375	0.098	0.918776382	1
ENSBTAG00000014794	*SCYL3*	2'010	1'011	1'454	1'741	1'167	-0.576	0.475033494	1
ENSBTAG00000014795	*BT.69436*	5	0	2	4	0		0.853268594	1
ENSBTAG00000014797	*SEC22A*	312	227	266	270	262	-0.044	0.969681829	1
ENSBTAG00000014798	*CXCR3*	5	2	3	4	2	-0.907	0.965043111	1
ENSBTAG00000014799	*ARMC7*	928	1'075	1'022	804	1'241	0.627	0.446764236	1
ENSBTAG00000014803	*CPZ*	10'660	7'842	9'143	9'232	9'055	-0.028	0.971765915	1
ENSBTAG00000014804	*BT.69182*	21'977	10'649	15'665	19'033	12'296	-0.630	0.410073551	1
ENSBTAG00000014805	*ENOPH1*	4'451	3'286	3'825	3'855	3'794	-0.023	0.97764566	1
ENSBTAG00000014806	*BT.62377*	565	877	751	489	1'013	1.049	0.217983489	1
ENSBTAG00000014807	*DPP8*	1'568	932	1'217	1'358	1'076	-0.335	0.680998768	1
ENSBTAG00000014809	*ANXA6*	12'108	25'725	20'095	10'486	29'705	1.502	0.052878049	1
ENSBTAG00000014813	*BT.56086*	40	3	19	35	3	-3.322	0.299073666	1
ENSBTAG00000014814	*KIAA0240*	687	553	617	595	639	0.102	0.907029251	1
ENSBTAG00000014818	*MYLK3*	92	56	72	80	65	-0.301	0.847076578	1
ENSBTAG00000014819	*CRYBA2*	0	1	1	0	1	Inf	0.993540919	1
ENSBTAG00000014820	*OXA1L*	2'872	2'828	2'876	2'487	3'265	0.393	0.615074261	1
ENSBTAG00000014821	*SLC7A7*	1'436	1'226	1'330	1'244	1'416	0.187	0.817065199	1
ENSBTAG00000014822	*MRPL52*	2'023	2'275	2'189	1'752	2'627	0.584	0.459372789	1
ENSBTAG00000014823	*BT.103066*	2'657	1'902	2'249	2'301	2'196	-0.067	0.93322663	1
ENSBTAG00000014825	*NLK*	1'300	579	897	1'126	669	-0.752	0.368376265	1
ENSBTAG00000014826	*ZC3H7A*	5'104	2'607	3'715	4'420	3'010	-0.554	0.476633852	1
ENSBTAG00000014827	*BT.92088*	3'161	1'517	2'245	2'738	1'752	-0.644	0.41553213	1
ENSBTAG00000014828	*CACNA1A*	947	486	691	820	561	-0.547	0.522607016	1
ENSBTAG00000014829	*BT.51933*	55	39	46	48	45	-0.081	0.97858379	1
ENSBTAG00000014830	*BT.25683*	13	0	6	11	0		0.551229012	1
ENSBTAG00000014831	*PPP1R3C*	2'449	633	1'426	2'121	731	-1.537	0.061187627	1
ENSBTAG00000014833	*NEU4*	40	4	20	35	5	-2.907	0.343574329	1
ENSBTAG00000014834	*ZP3*	3	2	2	3	2	-0.170	1	1
ENSBTAG00000014835	*SPARC*	1'257'826	588'549	884'454	1'089'309	679'598	-0.681	0.371173425	1
ENSBTAG00000014836	*protein_coding*	1	1	1	1	1	0.415	1	1
ENSBTAG00000014838	*VPS26B*	2'791	4'057	3'551	2'417	4'685	0.955	0.222386755	1
ENSBTAG00000014840	*BT.58650*	952	953	962	824	1'100	0.417	0.614504619	1
ENSBTAG00000014841	*GBA*	1'805	2'242	2'076	1'563	2'589	0.728	0.358668003	1
ENSBTAG00000014843	*COPB2*	9'903	8'278	9'067	8'576	9'559	0.156	0.837419243	1
ENSBTAG00000014847	*BBOX1*	486	188	319	421	217	-0.955	0.320170272	1
ENSBTAG00000014849	*GPATCH3*	492	630	577	426	727	0.772	0.376799071	1

Ensembl gene ID	geneName	counts wildtype horn bud	counts polled horn bud	baseMean	baseMean wildtype horn bud	baseMean polled horn bud	log2FoldChange	pval	padj
ENSBTAG00000014850	*GPN2*	429	428	433	372	494	0.412	0.649930187	1
ENSBTAG00000014855	*MICAL2*	228	120	168	197	139	-0.511	0.647803418	1
ENSBTAG00000014857	*BT.34069*	71	60	65	61	69	0.172	0.919660076	1
ENSBTAG00000014858	*PRPSAP2*	1'793	1'376	1'571	1'553	1'589	0.033	0.967150598	1
ENSBTAG00000014859	*CAPS2*	30	3	15	26	3	-2.907	0.432480205	1
ENSBTAG00000014861	*BT.21424*	985	587	765	853	678	-0.332	0.695780441	1
ENSBTAG00000014862	*HMGB1*	8'075	9'492	8'977	6'993	10'960	0.648	0.398138189	1
ENSBTAG00000014863	*GYPC*	635	387	498	550	447	-0.299	0.737872097	1
ENSBTAG00000014869	*pseudogene*	46	4	22	40	5	-3.109	0.279592358	1
ENSBTAG00000014871	*CCT8L2*	6	2	4	5	2	-1.170	0.934316631	1
ENSBTAG00000014872	*CAPNS1*	10'564	15'175	13'336	9'149	17'523	0.938	0.222278291	1
ENSBTAG00000014873	*COA5*	952	596	756	824	688	-0.261	0.759412661	1
ENSBTAG00000014874	*FYTTD1*	2'720	2'723	2'750	2'356	3'144	0.417	0.594346774	1
ENSBTAG00000014877	*UNC50*	2'002	1'195	1'557	1'734	1'380	-0.329	0.682024349	1
ENSBTAG00000014878	*COX7A1*	1'025	902	965	888	1'042	0.231	0.78041401	1
ENSBTAG00000014879	*FAM185A*	211	153	180	183	177	-0.049	0.970818508	1
ENSBTAG00000014880	*ICOSLG*	163	360	278	141	416	1.558	0.117896158	1
ENSBTAG00000014881	*BT.68767*	1'897	2'484	2'256	1'643	2'868	0.804	0.309576134	1
ENSBTAG00000014882	*DNMT3L*	5	1	3	4	1	-1.907	0.9188325	1
ENSBTAG00000014883	*GABARAP*	18'490	15'246	16'809	16'013	17'605	0.137	0.8569211	1
ENSBTAG00000014884	*HIPK3*	1'135	794	950	983	917	-0.100	0.905311274	1
ENSBTAG00000014886	*MEX3C*	1'683	1'495	1'592	1'458	1'726	0.244	0.760077087	1
ENSBTAG00000014889	*DCBLD2*	547	229	369	474	264	-0.841	0.368200177	1
ENSBTAG00000014890	*CYP19A1*	1	1	1	1	1	0.415	1	1
ENSBTAG00000014891	*SRSF7*	10'588	5'322	7'657	9'169	6'145	-0.577	0.45297039	1
ENSBTAG00000014897	*UNC5A*	136	198	173	118	229	0.957	0.38534987	1
ENSBTAG00000014898	*BT.41815*	106	65	83	92	75	-0.291	0.842953049	1
ENSBTAG00000014899	*TTC36*	1	0	0	1	0		1	1
ENSBTAG00000014900	*CNOT3*	2'236	2'836	2'606	1'936	3'275	0.758	0.335332152	1
ENSBTAG00000014903	*ABCA3*	707	426	552	612	492	-0.316	0.720146212	1
ENSBTAG00000014904	*SIRT5*	2'490	1'518	1'955	2'156	1'753	-0.299	0.706999154	1
ENSBTAG00000014906	*VCAN*	21'204	20'863	21'227	18'363	24'091	0.392	0.606564078	1
ENSBTAG00000014907	*IL11RA*	5'482	6'191	5'948	4'748	7'149	0.591	0.443460675	1
ENSBTAG00000014908	*CCL27*	87	24	52	75	28	-1.443	0.40648508	1
ENSBTAG00000014910	*PIWIL4*	23	7	14	20	8	-1.301	0.726674667	1
ENSBTAG00000014913	*SRD5A3*	400	210	294	346	242	-0.515	0.597647145	1
ENSBTAG00000014915	*BT.70784*	1'089	699	875	943	807	-0.225	0.789187148	1
ENSBTAG00000014916	*BT.35166*	44	43	44	38	50	0.382	0.844218622	1
ENSBTAG00000014917	*GTPBP4*	4'747	3'003	3'789	4'111	3'468	-0.246	0.752418776	1
ENSBTAG00000014918	*IBA57*	250	334	301	217	386	0.833	0.389360719	1
ENSBTAG00000014920	*SEMA3E*	217	272	251	188	314	0.741	0.460648091	1
ENSBTAG00000014921	*IL6*	49	2	22	42	2	-4.200	0.171720345	1
ENSBTAG00000014922	*BT.34341*	490	728	632	424	841	0.986	0.255021958	1
ENSBTAG00000014923	*BT.41087*	2'960	1'842	2'345	2'563	2'127	-0.269	0.733090214	1
ENSBTAG00000014925	*NPAS4*	10	0	4	9	0		0.653360153	1
ENSBTAG00000014926	*SNAPC2*	557	462	508	482	533	0.145	0.870811912	1
ENSBTAG00000014927	*OXSR1*	800	802	809	693	926	0.419	0.618112336	1
ENSBTAG00000014930	*MYLK2*	27	67	50	23	77	1.726	0.324759626	1
ENSBTAG00000014931	*BT.31582*	5'487	6'936	6'380	4'752	8'009	0.753	0.328941934	1
ENSBTAG00000014932	*ARSE*	995	998	1'007	862	1'152	0.419	0.610815265	1
ENSBTAG00000014933	*TRAK2*	609	741	692	527	856	0.698	0.414046811	1
ENSBTAG00000014934	*DCAF10*	202	180	191	175	208	0.249	0.818910935	1
ENSBTAG00000014936	*MCART1*	1'672	1'294	1'471	1'448	1'494	0.045	0.955234985	1
ENSBTAG00000014938	*protein_coding*	2	1	1	2	1	-0.585	1	1
ENSBTAG00000014940	*BT.35661*	454	424	441	393	490	0.316	0.726731215	1
ENSBTAG00000014943	*SMCX*	8'781	10'386	9'799	7'605	11'993	0.657	0.391333286	1
ENSBTAG00000014944	*TBXA2R*	717	840	795	621	970	0.643	0.444909731	1
ENSBTAG00000014945	*IQSEC2*	994	1'264	1'160	861	1'460	0.762	0.351843805	1
ENSBTAG00000014947	*BT.64781*	2'174	1'531	1'825	1'883	1'768	-0.091	0.910326788	1
ENSBTAG00000014948	*C15ORF55*	0	1	1	0	1	Inf	0.993540919	1
ENSBTAG00000014953	*BT.92118*	3	1	2	3	1	-1.170	0.985253077	1
ENSBTAG00000014955	*AMH*	97	71	83	84	82	-0.035	0.990880685	1
ENSBTAG00000014956	*JSRP1*	13	101	64	11	117	3.373	0.045474245	1
ENSBTAG00000014958	*PRKAR2B*	2'227	1'274	1'700	1'929	1'471	-0.391	0.625293227	1
ENSBTAG00000014964	*GALK1*	1'115	1'278	1'221	966	1'476	0.612	0.452202628	1
ENSBTAG00000014966	*PLXNB2*	13'361	15'650	14'821	11'571	18'071	0.643	0.399999298	1
ENSBTAG00000014967	*PAX7*	0	18	10	0	21	Inf	0.257475655	1
ENSBTAG00000014968	*YJEFN3*	36	20	27	31	23	-0.433	0.86863557	1
ENSBTAG00000014970	*HBXIP*	1'638	992	1'282	1'419	1'145	-0.308	0.704544912	1
ENSBTAG00000014971	*SEC61G*	5'646	2'801	4'062	4'890	3'234	-0.596	0.443056737	1
ENSBTAG00000014974	*GRAMD3*	891	466	655	772	538	-0.520	0.545926346	1
ENSBTAG00000014975	*SLC4A3*	1'271	1'508	1'421	1'101	1'741	0.662	0.412155698	1

Ensembl gene ID	geneName	counts wildtype horn bud	counts polled horn bud	baseMean	baseMean wildtype horn bud	baseMean polled horn bud	log2FoldChange	pval	padj
ENSBTAG00000014979	*MFSD12*	463	370	414	401	427	0.092	0.921898828	1
ENSBTAG00000014981	*BT.27676*	318	236	274	275	273	-0.015	0.992779959	1
ENSBTAG00000014982	*BT.50288*	93	74	83	81	85	0.085	0.959760885	1
ENSBTAG00000014983	*BT.48219*	1	2	2	1	2	1.415	0.981979269	1
ENSBTAG00000014984	*RPRD2*	1'814	1'415	1'602	1'571	1'634	0.057	0.943619009	1
ENSBTAG00000014988	*protein_coding*	192	388	307	166	448	1.430	0.142147225	1
ENSBTAG00000014990	*SLC18A3*	4	3	3	3	3	0.000	1	1
ENSBTAG00000014991	*PARD3*	2'823	2'601	2'724	2'445	3'003	0.297	0.704149697	1
ENSBTAG00000014992	*GPR37L1*	126	42	79	109	48	-1.170	0.417682045	1
ENSBTAG00000014995	*BT.45455*	469	475	477	406	548	0.433	0.62775059	1
ENSBTAG00000014996	*WDR62*	388	648	542	336	748	1.155	0.192027212	1
ENSBTAG00000015000	*BT.49331*	18'090	15'139	16'574	15'666	17'481	0.158	0.834949051	1
ENSBTAG00000015001	*OVOL3*	3	3	3	3	3	0.415	1	1
ENSBTAG00000015002	*POLR2I*	804	558	670	696	644	-0.112	0.898284325	1
ENSBTAG00000015004	*TBCB*	2'280	2'276	2'301	1'975	2'628	0.413	0.600214367	1
ENSBTAG00000015005	*MGC159566*	6'539	3'693	4'964	5'663	4'264	-0.409	0.596784692	1
ENSBTAG00000015006	*MRPL11*	2'536	2'771	2'698	2'196	3'200	0.543	0.488516375	1
ENSBTAG00000015007	*BT.55996*	1'449	1'874	1'709	1'255	2'164	0.786	0.326021118	1
ENSBTAG00000015008	*CPA3*	397	225	302	344	260	-0.404	0.677593921	1
ENSBTAG00000015009	*C25H7orf59*	3'250	2'954	3'113	2'815	3'411	0.277	0.721739561	1
ENSBTAG00000015010	*TBC1D23*	3'261	2'048	2'594	2'824	2'365	-0.256	0.744885958	1
ENSBTAG00000015011	*C7ORF43*	822	791	813	712	913	0.360	0.668387002	1
ENSBTAG00000015012	*PTRHD1*	447	353	397	387	408	0.074	0.937441899	1
ENSBTAG00000015013	*C2CD2L*	1'297	1'602	1'487	1'123	1'850	0.720	0.371520599	1
ENSBTAG00000015014	*CENPO*	620	668	654	537	771	0.523	0.542761857	1
ENSBTAG00000015015	*BT.43786*	1'329	1'708	1'562	1'151	1'972	0.777	0.333777907	1
ENSBTAG00000015016	*BT.28477*	5'305	5'700	5'588	4'594	6'582	0.519	0.500929666	1
ENSBTAG00000015017	*GAL3ST4*	1'182	994	1'086	1'024	1'148	0.165	0.840431397	1
ENSBTAG00000015018	*FSD2*	111	96	103	96	111	0.206	0.87901421	1
ENSBTAG00000015019	*OBFC1*	1'137	1'154	1'159	985	1'333	0.436	0.592590884	1
ENSBTAG00000015022	*KIF2A*	2'083	1'275	1'638	1'804	1'472	-0.293	0.714787149	1
ENSBTAG00000015023	*TRAPPC6B*	1'740	684	1'148	1'507	790	-0.932	0.256813612	1
ENSBTAG00000015024	*GXYLT1*	328	274	300	284	316	0.156	0.874244614	1
ENSBTAG00000015025	*BT.43331*	631	265	426	546	306	-0.837	0.359523156	1
ENSBTAG00000015026	*BT.62162*	56	62	60	48	72	0.562	0.729318361	1
ENSBTAG00000015027	*protein_coding*	42	39	41	36	45	0.308	0.881916376	1
ENSBTAG00000015032	*CD14*	1'006	417	676	871	482	-0.855	0.319805734	1
ENSBTAG00000015034	*IK*	6'636	4'071	5'224	5'747	4'701	-0.290	0.707658218	1
ENSBTAG00000015036	*RAD51C*	765	420	574	663	485	-0.450	0.607089906	1
ENSBTAG00000015037	*FAM59B*	261	495	399	226	572	1.338	0.149517075	1
ENSBTAG00000015038	*HADHA*	8'522	11'849	10'531	7'380	13'682	0.891	0.246801602	1
ENSBTAG00000015040	*BT.72262*	39	36	38	34	42	0.300	0.890830578	1
ENSBTAG00000015041	*NDUFA2*	693	621	659	600	717	0.257	0.764943639	1
ENSBTAG00000015042	*WDR55*	3'683	2'698	3'152	3'190	3'115	-0.034	0.966370714	1
ENSBTAG00000015043	*MFNG*	403	259	324	349	299	-0.223	0.818026333	1
ENSBTAG00000015044	*CARD10*	579	651	627	501	752	0.584	0.498575145	1
ENSBTAG00000015045	*BT.62716*	4	7	6	3	8	1.222	0.876605346	1
ENSBTAG00000015046	*MST1R*	791	596	687	685	688	0.007	0.995044342	1
ENSBTAG00000015049	*ECSIT*	518	742	653	449	857	0.934	0.279194619	1
ENSBTAG00000015050	*HCN2*	267	198	230	231	229	-0.016	0.993041858	1
ENSBTAG00000015051	*POLRMT*	857	1'315	1'130	742	1'518	1.033	0.209616401	1
ENSBTAG00000015052	*PROX2*	179	88	128	155	102	-0.609	0.615237178	1
ENSBTAG00000015053	*CFL2*	1'537	1'016	1'252	1'331	1'173	-0.182	0.823727535	1
ENSBTAG00000015056	*RBP2*	11	0	5	10	0		0.617662983	1
ENSBTAG00000015058	*CDH4*	188	159	173	163	184	0.173	0.878388659	1
ENSBTAG00000015060	*GBP5*	195	130	159	169	150	-0.170	0.885723048	1
ENSBTAG00000015061	*BT.37782*	17	6	11	15	7	-1.087	0.813534849	1
ENSBTAG00000015062	*KCNK18*	1	0	0	1	0		1	1
ENSBTAG00000015065	*TMPRSS7*	4	0	2	3	0		0.89545886	1
ENSBTAG00000015066	*BT.70066*	6'591	4'607	5'514	5'708	5'320	-0.102	0.89586307	1
ENSBTAG00000015067	*BT.46270*	1	0	0	1	0		1	1
ENSBTAG00000015069	*PCNP*	9'548	6'034	7'618	8'269	6'967	-0.247	0.748029168	1
ENSBTAG00000015072	*FZD8*	519	171	323	449	197	-1.187	0.217256448	1
ENSBTAG00000015073	*ISPD*	203	125	160	176	144	-0.285	0.80480978	1
ENSBTAG00000015074	*PTGDS*	3'804	1'412	2'462	3'294	1'630	-1.015	0.20024224	1
ENSBTAG00000015075	*BT.67787*	264	275	273	229	318	0.474	0.630926528	1
ENSBTAG00000015076	*LATS1*	1'682	1'072	1'347	1'457	1'238	-0.235	0.772472851	1
ENSBTAG00000015077	*BT.103876*	322	608	490	279	702	1.332	0.139069551	1
ENSBTAG00000015080	*BT.86915*	573	163	342	496	188	-1.399	0.143523019	1
ENSBTAG00000015083	*BT.68975*	2'982	1'720	2'284	2'582	1'986	-0.379	0.631475922	1
ENSBTAG00000015085	*CLIC3*	648	872	784	561	1'007	0.843	0.318508176	1
ENSBTAG00000015086	*HSD11B1*	93	77	85	81	89	0.143	0.925639832	1

Ensembl gene ID	geneName	counts wildtype horn bud	counts polled horn bud	baseMean	baseMean wildtype horn bud	baseMean polled horn bud	log2FoldChange	pval	padj
ENSBTAG00000015087	*KLHL35*	24	18	21	21	21	0.000	1	1
ENSBTAG00000015089	*LGALS1*	15'787	17'684	17'046	13'672	20'420	0.579	0.44812511	1
ENSBTAG00000015090	*SGTA*	2'918	4'084	3'621	2'527	4'716	0.900	0.249318378	1
ENSBTAG00000015091	*protein_coding*	2	0	1	2	0		0.974934741	1
ENSBTAG00000015092	*NOL12*	689	704	705	597	813	0.446	0.600346366	1
ENSBTAG00000015093	*ADAMTS13*	71	12	38	61	14	-2.150	0.296630072	1
ENSBTAG00000015094	*VNN1*	121	37	74	105	43	-1.294	0.383276347	1
ENSBTAG00000015095	*TTC6*	1	1	1	1	1	0.415	1	1
ENSBTAG00000015097	*MGC139698*	1'525	1'687	1'634	1'321	1'948	0.561	0.483362526	1
ENSBTAG00000015098	*RABEPK*	718	473	584	622	546	-0.187	0.831823076	1
ENSBTAG00000015099	*BT.19498*	2'758	2'093	2'403	2'388	2'417	0.017	0.982665179	1
ENSBTAG00000015100	*ATP6V0E1*	3'548	2'234	2'826	3'073	2'580	-0.252	0.747793732	1
ENSBTAG00000015101	*HMGB2*	1'746	1'618	1'690	1'512	1'868	0.305	0.70181947	1
ENSBTAG00000015103	*BT.26801*	952	898	931	824	1'037	0.331	0.689976396	1
ENSBTAG00000015104	*CCDC28B*	3'312	1'731	2'434	2'868	1'999	-0.521	0.508535497	1
ENSBTAG00000015105	*HERPUD2*	2'289	1'150	1'655	1'982	1'328	-0.578	0.470700034	1
ENSBTAG00000015106	*DSP*	32'099	29'615	30'998	27'799	34'196	0.299	0.693648783	1
ENSBTAG00000015107	*SLC16A1*	1'741	1'063	1'368	1'508	1'227	-0.297	0.714229259	1
ENSBTAG00000015108	*BT.64703*	470	347	404	407	401	-0.023	0.984081296	1
ENSBTAG00000015109	*TOB2*	4'597	3'405	3'956	3'981	3'932	-0.018	0.982519372	1
ENSBTAG00000015112	*BT.88479*	2'794	1'911	2'313	2'420	2'207	-0.133	0.866956799	1
ENSBTAG00000015113	*CITED4*	109	125	119	94	144	0.613	0.619723665	1
ENSBTAG00000015114	*CALR*	31'980	33'601	33'247	27'695	38'799	0.486	0.522282143	1
ENSBTAG00000015115	*ACPT*	210	93	145	182	107	-0.760	0.514188621	1
ENSBTAG00000015116	*RAD23A*	2'238	2'770	2'568	1'938	3'199	0.723	0.358289727	1
ENSBTAG00000015117	*GADD45GIP1*	445	460	458	385	531	0.463	0.606650741	1
ENSBTAG00000015118	*PNPLA1*	398	280	334	345	323	-0.092	0.926210744	1
ENSBTAG00000015119	*SIK3*	2'202	1'667	1'916	1'907	1'925	0.013	0.986501511	1
ENSBTAG00000015124	*BT.45117*	9'432	3'400	6'047	8'168	3'926	-1.057	0.173451236	1
ENSBTAG00000015125	*KLK5*	17	5	10	15	6	-1.350	0.775139346	1
ENSBTAG00000015126	*BT.63640*	343	390	374	297	450	0.600	0.518044999	1
ENSBTAG00000015128	*BT.106542*	27	6	15	23	7	-1.755	0.616167115	1
ENSBTAG00000015129	*KLK10*	2'506	2'200	2'355	2'170	2'540	0.227	0.772418703	1
ENSBTAG00000015130	*HDGFL1*	3	0	1	3	0		0.936647693	1
ENSBTAG00000015131	*SLC29A1*	3'338	2'626	2'962	2'891	3'032	0.069	0.929379103	1
ENSBTAG00000015133	*CCR7*	25	9	16	22	10	-1.059	0.75675668	1
ENSBTAG00000015136	*AARSD1*	1'634	1'766	1'727	1'415	2'039	0.527	0.508647231	1
ENSBTAG00000015138	*MGA*	1'478	1'172	1'317	1'280	1'353	0.080	0.921048784	1
ENSBTAG00000015139	*protein_coding*	1	2	2	1	2	1.415	0.981979269	1
ENSBTAG00000015140	*RUNDC1*	241	186	212	209	215	0.041	0.972659422	1
ENSBTAG00000015142	*MAPKBP1*	1'777	2'759	2'362	1'539	3'186	1.050	0.185699933	1
ENSBTAG00000015144	*BT.53629*	1'212	1'728	1'522	1'050	1'995	0.927	0.250713401	1
ENSBTAG00000015145	*BT.3750*	14'235	10'138	12'017	12'328	11'706	-0.075	0.922872226	1
ENSBTAG00000015146	*TOX4*	3'887	3'006	3'419	3'366	3'471	0.044	0.954348117	1
ENSBTAG00000015147	*S100A10*	17'487	20'643	19'490	15'144	23'836	0.654	0.391205859	1
ENSBTAG00000015148	*METTL3*	2'063	1'234	1'606	1'787	1'425	-0.326	0.684304614	1
ENSBTAG00000015149	*pseudogene*	882	1'389	1'184	764	1'604	1.070	0.192452012	1
ENSBTAG00000015150	*IL12A*	14	6	10	12	7	-0.807	0.881616426	1
ENSBTAG00000015151	*FOXN2*	111	179	151	96	207	1.104	0.335637051	1
ENSBTAG00000015154	*MCL1*	10'473	5'595	7'765	9'070	6'461	-0.489	0.524355262	1
ENSBTAG00000015155	*PTPLAD1*	5'145	4'287	4'703	4'456	4'950	0.152	0.843644253	1
ENSBTAG00000015156	*NDUFB1*	1'041	698	854	902	806	-0.162	0.848393796	1
ENSBTAG00000015157	*GRAP2*	18	13	15	16	15	-0.054	1	1
ENSBTAG00000015158	*C10H15orf44*	862	752	807	747	868	0.218	0.795371957	1
ENSBTAG00000015160	*PLD4*	298	124	201	258	143	-0.850	0.424139302	1
ENSBTAG00000015163	*TM4SF1*	5'029	1'688	3'152	4'355	1'949	-1.160	0.141191789	1
ENSBTAG00000015164	*BT.21212*	400	433	423	346	500	0.529	0.560699846	1
ENSBTAG00000015165	*DPY19L3*	303	295	302	262	341	0.376	0.697324564	1
ENSBTAG00000015166	*PNLDC1*	99	126	116	86	145	0.763	0.540304553	1
ENSBTAG00000015169	*UBXN4*	7'278	4'055	5'493	6'303	4'682	-0.429	0.578716711	1
ENSBTAG00000015170	*LCT*	0	2	1	0	2	Inf	0.939077559	1
ENSBTAG00000015171	*CCRK*	1'324	1'116	1'218	1'147	1'289	0.168	0.835860298	1
ENSBTAG00000015172	*BT.56763*	3'195	3'894	3'632	2'767	4'496	0.700	0.368466491	1
ENSBTAG00000015175	*OR2W1*	6	6	6	5	7	0.415	0.976119138	1
ENSBTAG00000015176	*C6ORF201*	240	162	197	208	187	-0.152	0.891281921	1
ENSBTAG00000015178	*BT.47386*	1'228	1'064	1'146	1'063	1'229	0.208	0.798654672	1
ENSBTAG00000015180	*protein_coding*	26	34	31	23	39	0.802	0.721601178	1
ENSBTAG00000015181	*EPM2A*	266	115	182	230	133	-0.795	0.466467963	1
ENSBTAG00000015182	*STARD10*	265	415	354	229	479	1.062	0.259488027	1
ENSBTAG00000015183	*TPCN2*	1'406	1'837	1'669	1'218	2'121	0.801	0.317719752	1
ENSBTAG00000015184	*SYDE1*	957	1'415	1'231	829	1'634	0.979	0.231069645	1
ENSBTAG00000015185	*BT.24347*	1'960	3'114	2'647	1'697	3'596	1.083	0.170803524	1

Ensembl gene ID	geneName	counts wildtype horn bud	counts polled horn bud	baseMean	baseMean wildtype horn bud	baseMean polled horn bud	log2FoldChange	pval	padj
ENSBTAG00000015186	*BT.24640*	1'444	1'884	1'713	1'251	2'175	0.799	0.31832688	1
ENSBTAG00000015187	*DDX41*	2'771	3'323	3'118	2'400	3'837	0.677	0.386353188	1
ENSBTAG00000015188	*KLF6*	3'404	1'740	2'479	2'948	2'009	-0.553	0.482608416	1
ENSBTAG00000015190	*C18ORF8*	908	532	700	786	614	-0.356	0.677272289	1
ENSBTAG00000015191	*ABCC6*	9	0	4	8	0		0.690676647	1
ENSBTAG00000015192	*FAM193B*	2'793	2'971	2'925	2'419	3'431	0.504	0.518850167	1
ENSBTAG00000015193	*BOLA3*	584	391	479	506	451	-0.164	0.857081395	1
ENSBTAG00000015194	*CBLN1*	8	0	3	7	0		0.72953145	1
ENSBTAG00000015195	*NPC1*	1'430	1'070	1'237	1'238	1'236	-0.003	0.998570962	1
ENSBTAG00000015196	*PHF6*	420	342	379	364	395	0.119	0.89984869	1
ENSBTAG00000015197	*KLHDC8A*	83	52	66	72	60	-0.260	0.875215291	1
ENSBTAG00000015198	*DZIP1L*	516	406	458	447	469	0.069	0.940402323	1
ENSBTAG00000015200	*L2HGDH*	596	210	379	516	242	-1.090	0.242986186	1
ENSBTAG00000015202	*ATP5S*	589	315	437	510	364	-0.488	0.591410358	1
ENSBTAG00000015205	*NCK2*	1'453	1'079	1'252	1'258	1'246	-0.014	0.987771038	1
ENSBTAG00000015208	*LRRC16B*	247	101	165	214	117	-0.875	0.434738703	1
ENSBTAG00000015209	*MXD4*	1'137	1'606	1'420	985	1'854	0.913	0.259393724	1
ENSBTAG00000015210	*LOXHD1*	1	1	1	1	1	0.415	1	1
ENSBTAG00000015212	*IFNAR2*	2'142	1'352	1'708	1'855	1'561	-0.249	0.756074381	1
ENSBTAG00000015214	*CA3*	2'396	747	1'469	2'075	863	-1.266	0.119925758	1
ENSBTAG00000015218	*pseudogene*	21	20	21	18	23	0.345	0.916000044	1
ENSBTAG00000015220	*EIF2C4*	614	499	554	532	576	0.116	0.895845144	1
ENSBTAG00000015221	*PCCB*	1'472	1'058	1'248	1'275	1'222	-0.061	0.941393973	1
ENSBTAG00000015222	*C1orf63*	6'374	1'965	3'895	5'520	2'269	-1.283	0.102803019	1
ENSBTAG00000015224	*TCOF1*	2'966	3'564	3'342	2'569	4'115	0.680	0.38346537	1
ENSBTAG00000015225	*NUPL1*	3'493	1'901	2'610	3'025	2'195	-0.463	0.556152807	1
ENSBTAG00000015226	*BRSK1*	2'030	1'572	1'787	1'758	1'815	0.046	0.95377415	1
ENSBTAG00000015228	*BT.46809*	5'922	1'796	3'601	5'129	2'074	-1.306	0.097395306	1
ENSBTAG00000015229	*DNAJC5B*	1	0	0	1	0		1	1
ENSBTAG00000015230	*PLA2G12A*	2'087	1'332	1'673	1'807	1'538	-0.233	0.771654359	1
ENSBTAG00000015232	*RAPGEFL1*	2'724	3'302	3'086	2'359	3'813	0.693	0.375787329	1
ENSBTAG00000015235	*HMCN1*	2'622	2'814	2'760	2'271	3'249	0.517	0.509074726	1
ENSBTAG00000015238	*DSC3*	742	978	886	643	1'129	0.813	0.330439225	1
ENSBTAG00000015240	*UBR4*	6'320	13'785	10'695	5'473	15'918	1.540	0.048252409	1
ENSBTAG00000015241	*BT.87687*	2'141	873	1'431	1'854	1'008	-0.879	0.277744538	1
ENSBTAG00000015242	*C6ORF192*	1'341	578	914	1'161	667	-0.799	0.338479351	1
ENSBTAG00000015246	*TAS1R2*	1	5	3	1	6	2.737	0.836148221	1
ENSBTAG00000015248	*BT.89243*	714	184	415	618	212	-1.541	0.097055598	1
ENSBTAG00000015249	*B4GALT1*	8'343	6'056	7'109	7'225	6'993	-0.047	0.95184419	1
ENSBTAG00000015251	*CXORF45*	1'664	558	1'043	1'441	644	-1.161	0.16219207	1
ENSBTAG00000015252	*BT.106486*	57	190	134	49	219	2.152	0.078065174	1
ENSBTAG00000015253	*COX19*	3'083	3'025	3'081	2'670	3'493	0.388	0.61897567	1
ENSBTAG00000015254	*GLB1*	3'302	3'387	3'385	2'860	3'911	0.452	0.561495051	1
ENSBTAG00000015255	*ST6GALNAC2*	872	329	568	755	380	-0.991	0.259554034	1
ENSBTAG00000015258	*P2RX5*	145	381	283	126	440	1.809	0.070603601	1
ENSBTAG00000015259	*BT.106329*	95	62	77	82	72	-0.201	0.898596989	1
ENSBTAG00000015260	*protein_coding*	1	6	4	1	7	3.000	0.788284548	1
ENSBTAG00000015261	*CARD14*	145	80	109	126	92	-0.443	0.731670851	1
ENSBTAG00000015263	*CPSF7*	1'901	2'159	2'070	1'646	2'493	0.599	0.449589925	1
ENSBTAG00000015264	*BT.40523*	1'182	936	1'052	1'024	1'081	0.078	0.924375014	1
ENSBTAG00000015265	*TIGD2*	410	193	289	355	223	-0.672	0.491940861	1
ENSBTAG00000015266	*SDHAF2*	1'270	1'150	1'214	1'100	1'328	0.272	0.738017532	1
ENSBTAG00000015267	*SGSH*	2'002	2'176	2'123	1'734	2'513	0.535	0.498270281	1
ENSBTAG00000015268	*PRKCABP*	1'078	777	915	934	897	-0.057	0.947033508	1
ENSBTAG00000015271	*BT.34596*	1'567	831	1'158	1'357	960	-0.500	0.541140932	1
ENSBTAG00000015272	*TSC22D4*	774	990	907	670	1'143	0.770	0.355614767	1
ENSBTAG00000015273	*CAND2*	683	853	788	591	985	0.736	0.383300704	1
ENSBTAG00000015274	*PRL*	3	0	1	3	0		0.936647693	1
ENSBTAG00000015275	*SLC16A8*	2	1	1	2	1	-0.585	1	1
ENSBTAG00000015276	*SLC26A11*	1'451	1'405	1'439	1'257	1'622	0.369	0.6467679	1
ENSBTAG00000015277	*PCF11*	5'706	2'583	3'962	4'942	2'983	-0.728	0.349600053	1
ENSBTAG00000015278	*ARPC5L*	1'464	1'068	1'251	1'268	1'233	-0.040	0.962480482	1
ENSBTAG00000015280	*KIF2C*	541	651	610	469	752	0.682	0.431203188	1
ENSBTAG00000015283	*RPL32*	59'107	55'698	57'751	51'188	64'315	0.329	0.663894804	1
ENSBTAG00000015285	*BT.65634*	136'955	143'685	142'260	118'607	165'913	0.484	0.523225118	1
ENSBTAG00000015286	*GOLGA1*	998	815	903	864	941	0.123	0.883021241	1
ENSBTAG00000015288	*BEST4*	13	8	10	11	9	-0.285	0.973425765	1
ENSBTAG00000015290	*BAIAP2L2*	167	56	105	145	65	-1.161	0.371016098	1
ENSBTAG00000015291	*ORC4*	3'416	1'629	2'420	2'958	1'881	-0.653	0.407682268	1
ENSBTAG00000015293	*NXPH3*	178	205	195	154	237	0.619	0.561682781	1
ENSBTAG00000015294	*COX10*	439	610	542	380	704	0.890	0.312750124	1
ENSBTAG00000015296	*PTPRB*	1'146	545	811	992	629	-0.657	0.435488931	1

Ensembl gene ID	geneName	counts wildtype horn bud	counts polled horn bud	baseMean	baseMean wildtype horn bud	baseMean polled horn bud	log2FoldChange	pval	padj
ENSBTAG00000015297	*BANK1*	149	33	84	129	38	-1.760	0.217686079	1
ENSBTAG00000015301	*MRPS10*	1'717	1'404	1'554	1'487	1'621	0.125	0.876291611	1
ENSBTAG00000015303	*MPP6*	290	104	186	251	120	-1.064	0.327554154	1
ENSBTAG00000015304	*BT.49340*	331	268	298	287	309	0.110	0.911615933	1
ENSBTAG00000015307	*FBN2*	10'500	13'799	12'513	9'093	15'934	0.809	0.291341339	1
ENSBTAG00000015308	*BT.66106*	7'255	6'248	6'749	6'283	7'215	0.199	0.794511033	1
ENSBTAG00000015309	*BT.30546*	425	87	234	368	100	-1.873	0.073410411	1
ENSBTAG00000015310	*SLC25A36*	1'973	922	1'387	1'709	1'065	-0.683	0.399274145	1
ENSBTAG00000015311	*PTPRR*	423	106	244	366	122	-1.582	0.123821437	1
ENSBTAG00000015312	*LTBR*	3'660	3'544	3'631	3'170	4'092	0.369	0.634814347	1
ENSBTAG00000015313	*BT.45461*	592	471	528	513	544	0.085	0.924333321	1
ENSBTAG00000015314	*ASL*	473	279	366	410	322	-0.347	0.712004906	1
ENSBTAG00000015316	*BT.61659*	2'742	5'330	4'265	2'375	6'155	1.374	0.080492218	1
ENSBTAG00000015318	*PVRL2*	962	1'442	1'249	833	1'665	0.999	0.221535086	1
ENSBTAG00000015319	*BT.51446*	1'118	514	781	968	594	-0.706	0.404026224	1
ENSBTAG00000015321	*TRERF1*	578	606	600	501	700	0.483	0.577306274	1
ENSBTAG00000015325	*MTOR*	2'182	1'951	2'071	1'890	2'253	0.254	0.748061713	1
ENSBTAG00000015326	*DFNA5*	242	178	208	210	206	-0.028	0.984692874	1
ENSBTAG00000015327	*BT.64208*	24'891	32'911	29'779	21'556	38'002	0.818	0.284223388	1
ENSBTAG00000015329	*FAM63A*	1'912	1'860	1'902	1'656	2'148	0.375	0.636062702	1
ENSBTAG00000015330	*DPP4*	920	450	658	797	520	-0.617	0.473755335	1
ENSBTAG00000015333	*TAF2*	2'427	2'176	2'307	2'102	2'513	0.258	0.743220841	1
ENSBTAG00000015334	*ZHX1*	5'228	2'505	3'710	4'528	2'893	-0.646	0.406805271	1
ENSBTAG00000015336	*PRUNE*	1'595	1'043	1'293	1'381	1'204	-0.198	0.808360786	1
ENSBTAG00000015337	*TSPAN12*	672	345	490	582	398	-0.547	0.540433956	1
ENSBTAG00000015338	*DSCC1*	1'286	921	1'089	1'114	1'063	-0.067	0.937057004	1
ENSBTAG00000015339	*ENDOV*	982	517	724	850	597	-0.511	0.549014716	1
ENSBTAG00000015340	*ANGPTL7*	18	7	12	16	8	-0.948	0.827785041	1
ENSBTAG00000015341	*BT.28648*	111	77	93	96	89	-0.113	0.942464941	1
ENSBTAG00000015343	*WDR34*	1'041	1'185	1'135	902	1'368	0.602	0.461734883	1
ENSBTAG00000015345	*BNIPL*	1'686	1'176	1'409	1'460	1'358	-0.105	0.898084221	1
ENSBTAG00000015346	*NASP*	7'805	7'448	7'680	6'759	8'600	0.347	0.650032061	1
ENSBTAG00000015347	*WNT10B*	74	131	108	64	151	1.239	0.333344643	1
ENSBTAG00000015348	*ZNF189*	1'790	1'204	1'470	1'550	1'390	-0.157	0.846339726	1
ENSBTAG00000015349	*BT.51884*	22	12	16	19	14	-0.459	0.903495485	1
ENSBTAG00000015350	*PLIN*	491	445	470	425	514	0.273	0.760951907	1
ENSBTAG00000015351	*BT.63725*	29	17	22	25	20	-0.355	0.909573651	1
ENSBTAG00000015352	*MTMR4*	3'628	2'161	2'819	3'142	2'495	-0.332	0.671642363	1
ENSBTAG00000015354	*ZDHHC9*	3'630	4'454	4'143	3'144	5'143	0.710	0.360527701	1
ENSBTAG00000015355	*PHKA2*	637	746	707	552	861	0.643	0.450628742	1
ENSBTAG00000015358	*ALDOB*	106	57	79	92	66	-0.480	0.742839917	1
ENSBTAG00000015361	*CNTFR*	2'763	1'548	2'090	2'393	1'787	-0.421	0.595456816	1
ENSBTAG00000015362	*BMP7*	777	1'272	1'071	673	1'469	1.126	0.17368773	1
ENSBTAG00000015363	*CDC42SE1*	5'892	4'804	5'325	5'103	5'547	0.121	0.875217643	1
ENSBTAG00000015364	*BT.101628*	1	0	0	1	0		1	1
ENSBTAG00000015368	*Sep 04*	1'743	719	1'170	1'509	830	-0.862	0.292926761	1
ENSBTAG00000015369	*MLLT11*	4'122	1'321	2'548	3'570	1'525	-1.227	0.122581733	1
ENSBTAG00000015371	*GABPB2*	217	270	250	188	312	0.730	0.467546682	1
ENSBTAG00000015373	*AADACL3*	19	2	9	16	2	-2.833	0.582174212	1
ENSBTAG00000015375	*SH2D3C*	1'238	907	1'060	1'072	1'047	-0.034	0.969123713	1
ENSBTAG00000015376	*FBXW11*	1'131	1'171	1'166	979	1'352	0.465	0.568367886	1
ENSBTAG00000015377	*EPHB1*	459	142	281	398	164	-1.278	0.197139457	1
ENSBTAG00000015378	*C9ORF174*	3	0	1	3	0		0.936647693	1
ENSBTAG00000015379	*UBOX5*	932	927	939	807	1'070	0.407	0.623113561	1
ENSBTAG00000015380	*SLC16A4*	32	15	23	28	17	-0.678	0.809212694	1
ENSBTAG00000015381	*ARHGAP18*	1'516	601	1'003	1'313	694	-0.920	0.267721684	1
ENSBTAG00000015384	*PHF13*	576	641	619	499	740	0.569	0.510064514	1
ENSBTAG00000015385	*CTDSPL2*	1'113	1'086	1'109	964	1'254	0.380	0.642596475	1
ENSBTAG00000015386	*REL*	39	22	30	34	25	-0.411	0.868871187	1
ENSBTAG00000015387	*RAB40B*	138	62	96	120	72	-0.739	0.581489701	1
ENSBTAG00000015388	*RPL18*	48'895	42'642	45'792	42'344	49'239	0.218	0.773748858	1
ENSBTAG00000015390	*FN3KRP*	3'351	2'911	3'132	2'902	3'361	0.212	0.785280162	1
ENSBTAG00000015392	*TTF2*	865	777	823	749	897	0.260	0.7563672	1
ENSBTAG00000015394	*FN3K*	222	139	176	192	161	-0.260	0.816346249	1
ENSBTAG00000015395	*pseudogene*	4	2	3	3	2	-0.585	0.996239345	1
ENSBTAG00000015396	*RBM24*	26	26	26	23	30	0.415	0.874909083	1
ENSBTAG00000015398	*BT.29712*	4'128	2'931	3'480	3'575	3'384	-0.079	0.920052443	1
ENSBTAG00000015401	*ATG4B*	1'855	1'936	1'921	1'606	2'236	0.477	0.547889464	1
ENSBTAG00000015402	*GREB1*	88	4	40	76	5	-4.044	0.065304408	1
ENSBTAG00000015403	*BT.35140*	1'517	1'439	1'488	1'314	1'662	0.339	0.672878308	1
ENSBTAG00000015406	*ZNF750*	939	643	778	813	742	-0.131	0.878240845	1
ENSBTAG00000015408	*BT.36762*	577	736	675	500	850	0.766	0.371614858	1

Ensembl gene ID	geneName	counts wildtype horn bud	counts polled horn bud	baseMean	baseMean wildtype horn bud	baseMean polled horn bud	log2FoldChange	pval	padj
ENSBTAG00000015409	*STK32B*	32	8	18	28	9	-1.585	0.604898832	1
ENSBTAG00000015412	*BT.62528*	1	2	2	1	2	1.415	0.981979269	1
ENSBTAG00000015413	*RASA2*	581	370	465	503	427	-0.236	0.794675188	1
ENSBTAG00000015414	*TBCD*	2'416	3'460	3'044	2'092	3'995	0.933	0.23466481	1
ENSBTAG00000015415	*UNC80*	67	10	35	58	12	-2.329	0.280869574	1
ENSBTAG00000015416	*PEBP4*	385	175	268	333	202	-0.722	0.467142147	1
ENSBTAG00000015419	*ARHGEF37*	352	315	334	305	364	0.255	0.78899241	1
ENSBTAG00000015424	*WDR12*	1'382	923	1'131	1'197	1'066	-0.167	0.839222157	1
ENSBTAG00000015426	*PDLIM4*	1'470	1'818	1'686	1'273	2'099	0.722	0.367194894	1
ENSBTAG00000015427	*CFDP1*	3'552	3'166	3'366	3'076	3'656	0.249	0.74845533	1
ENSBTAG00000015428	*TMEM170A*	2'215	1'153	1'625	1'918	1'331	-0.527	0.511161462	1
ENSBTAG00000015432	*TMEM231*	1'016	518	739	880	598	-0.557	0.512464088	1
ENSBTAG00000015433	*APC2*	461	209	320	399	241	-0.726	0.448550816	1
ENSBTAG00000015434	*DSTN*	8'498	5'391	6'792	7'359	6'225	-0.242	0.753834299	1
ENSBTAG00000015436	*SLC27A4*	354	528	458	307	610	0.992	0.272124551	1
ENSBTAG00000015437	*BT.29263*	371	611	513	321	706	1.135	0.202921563	1
ENSBTAG00000015438	*RRBP1*	29'380	17'559	22'860	25'444	20'275	-0.328	0.667488915	1
ENSBTAG00000015439	*BT.106463*	8	6	7	7	7	0.000	1	1
ENSBTAG00000015441	*ACTB*	795	611	697	688	706	0.035	0.968129514	1
ENSBTAG00000015444	*LETM2*	940	427	654	814	493	-0.723	0.401368952	1
ENSBTAG00000015449	*PPEF2*	17	9	13	15	10	-0.503	0.915336184	1
ENSBTAG00000015450	*CPEB3*	609	194	376	527	224	-1.235	0.187580575	1
ENSBTAG00000015453	*PCDHB16*	216	93	147	187	107	-0.801	0.489557358	1
ENSBTAG00000015457	*FGFR1*	18'091	23'599	21'459	15'667	27'250	0.798	0.296269082	1
ENSBTAG00000015459	*BT.47448*	5	1	3	4	1	-1.907	0.9188325	1
ENSBTAG00000015460	*TP63*	2'755	3'570	3'254	2'386	4'122	0.789	0.313049166	1
ENSBTAG00000015462	*BNC1*	336	614	500	291	709	1.285	0.152125145	1
ENSBTAG00000015463	*C27H8ORF41*	901	471	662	780	544	-0.521	0.544888356	1
ENSBTAG00000015464	*BT.104738*	2	1	1	2	1	-0.585	1	1
ENSBTAG00000015465	*C7H5orf46*	40	6	21	35	7	-2.322	0.421576376	1
ENSBTAG00000015466	*NSUN2*	3'183	2'706	2'941	2'757	3'125	0.181	0.816511398	1
ENSBTAG00000015467	*FAM184A*	410	74	220	355	85	-2.055	0.054325964	1
ENSBTAG00000015473	*RPS27A*	85'900	48'957	65'461	74'392	56'531	-0.396	0.602516454	1
ENSBTAG00000015474	*SFRS11*	9'358	3'611	6'137	8'104	4'170	-0.959	0.216085637	1
ENSBTAG00000015478	*SRD5A1*	682	677	686	591	782	0.404	0.635786258	1
ENSBTAG00000015479	*BT.24633*	1'091	1'182	1'155	945	1'365	0.531	0.515711624	1
ENSBTAG00000015481	*MTIF2*	597	317	442	517	366	-0.498	0.582940529	1
ENSBTAG00000015482	*ANKRD13C*	1'945	1'418	1'661	1'684	1'637	-0.041	0.960670069	1
ENSBTAG00000015483	*BT.30825*	8	3	5	7	3	-1.000	0.918861955	1
ENSBTAG00000015485	*MGC134132*	22	0	10	19	0		0.327125567	1
ENSBTAG00000015486	*CD2BP2*	2'067	2'666	2'434	1'790	3'078	0.782	0.321403507	1
ENSBTAG00000015487	*KDM4B*	1'995	3'583	2'933	1'728	4'137	1.260	0.111304065	1
ENSBTAG00000015490	*HS1BP3*	370	531	467	320	613	0.936	0.298195212	1
ENSBTAG00000015497	*NUS1*	442	210	313	383	242	-0.659	0.49385715	1
ENSBTAG00000015498	*ELOVL4*	1'019	248	584	882	286	-1.624	0.067479029	1
ENSBTAG00000015499	*C11ORF2*	1'530	2'369	2'030	1'325	2'735	1.046	0.189879853	1
ENSBTAG00000015504	*ZNF131*	1'775	1'144	1'429	1'537	1'321	-0.219	0.787068983	1
ENSBTAG00000015505	*FADS2*	2'668	921	1'687	2'311	1'063	-1.119	0.164950117	1
ENSBTAG00000015509	*BT.33608*	972	810	889	842	935	0.152	0.855495327	1
ENSBTAG00000015510	*EPHB6*	348	567	478	301	655	1.119	0.213465271	1
ENSBTAG00000015511	*FADS3*	660	466	555	572	538	-0.087	0.923470648	1
ENSBTAG00000015512	*BT.56197*	2'820	2'614	2'730	2'442	3'018	0.306	0.69587823	1
ENSBTAG00000015513	*DOCK5*	434	451	448	376	521	0.470	0.601940775	1
ENSBTAG00000015516	*CCDC28A*	366	163	253	317	188	-0.752	0.454943696	1
ENSBTAG00000015518	*KRT12*	2	5	4	2	6	1.737	0.883335552	1
ENSBTAG00000015519	*GFM2*	1'485	1'000	1'220	1'286	1'155	-0.155	0.849807218	1
ENSBTAG00000015520	*SLC11A1*	47	23	34	41	27	-0.616	0.780031245	1
ENSBTAG00000015521	*GEMIN6*	589	373	470	510	431	-0.244	0.78734557	1
ENSBTAG00000015522	*MRPS31*	670	396	519	580	457	-0.344	0.698888511	1
ENSBTAG00000015526	*PIWIL3*	1	1	1	1	1	0.415	1	1
ENSBTAG00000015529	*CHCHD4*	758	537	638	656	620	-0.082	0.926382005	1
ENSBTAG00000015532	*NR0B1*	30	6	16	26	7	-1.907	0.565369481	1
ENSBTAG00000015534	*LYPLAL1*	534	305	407	462	352	-0.393	0.669380201	1
ENSBTAG00000015535	*NEK8*	540	481	512	468	555	0.248	0.779740932	1
ENSBTAG00000015536	*CPNE3*	2'005	1'572	1'776	1'736	1'815	0.064	0.935873498	1
ENSBTAG00000015537	*RASAL2*	293	162	220	254	187	-0.440	0.673029625	1
ENSBTAG00000015538	*LEMD2*	2'465	2'734	2'646	2'135	3'157	0.564	0.47178462	1
ENSBTAG00000015539	*RAPGEF5*	168	268	227	145	309	1.089	0.290892239	1
ENSBTAG00000015541	*BT.24236*	2'734	2'342	2'536	2'368	2'704	0.192	0.80656086	1
ENSBTAG00000015543	*ARHGAP35*	2'441	3'322	2'975	2'114	3'836	0.860	0.273357135	1
ENSBTAG00000015546	*MINA*	1'270	747	981	1'100	863	-0.351	0.672393711	1
ENSBTAG00000015547	*SLC26A3*	22	5	12	19	6	-1.722	0.669657938	1

Ensembl gene ID	geneName	counts wildtype horn bud	counts polled horn bud	baseMean	baseMean wildtype horn bud	baseMean polled horn bud	log2FoldChange	pval	padj
ENSBTAG00000015549	*BT.69526*	6'035	6'931	6'615	5'226	8'003	0.615	0.424423089	1
ENSBTAG00000015550	*MLN*	4	5	5	3	6	0.737	0.954105567	1
ENSBTAG00000015553	*SCAI*	46	41	44	40	47	0.249	0.903212579	1
ENSBTAG00000015554	*TMF1*	1'983	830	1'338	1'717	958	-0.841	0.300554024	1
ENSBTAG00000015556	*UBA3*	3'318	1'877	2'520	2'873	2'167	-0.407	0.605174488	1
ENSBTAG00000015557	*protein_coding*	1	5	3	1	6	2.737	0.836148221	1
ENSBTAG00000015559	*ERI3*	2'455	2'198	2'332	2'126	2'538	0.256	0.745077836	1
ENSBTAG00000015562	*PLBD1*	1'365	1'019	1'179	1'182	1'177	-0.007	0.995352722	1
ENSBTAG00000015563	*PDE12*	205	132	165	178	152	-0.220	0.848600513	1
ENSBTAG00000015565	*BT.96606*	2	0	1	2	0		0.974934741	1
ENSBTAG00000015566	*CTR9*	2'865	2'945	2'941	2'481	3'401	0.455	0.560374106	1
ENSBTAG00000015567	*TIMM44*	1'264	1'372	1'339	1'095	1'584	0.533	0.509534211	1
ENSBTAG00000015569	*ENKUR*	91	26	54	79	30	-1.392	0.41117439	1
ENSBTAG00000015571	*GCLC*	507	357	426	439	412	-0.091	0.923472527	1
ENSBTAG00000015572	*BT.48251*	8'571	5'541	6'910	7'423	6'398	-0.214	0.780881589	1
ENSBTAG00000015575	*BT.27987*	7	3	5	6	3	-0.807	0.947428618	1
ENSBTAG00000015578	*SAP30*	541	185	341	469	214	-1.133	0.233755445	1
ENSBTAG00000015579	*TMEM160*	343	377	366	297	435	0.551	0.554063243	1
ENSBTAG00000015580	*TLE3*	3'916	6'094	5'214	3'391	7'037	1.053	0.175750965	1
ENSBTAG00000015581	*BT.10803*	473	157	295	410	181	-1.176	0.229410023	1
ENSBTAG00000015582	*HMOX1*	1'374	2'062	1'785	1'190	2'381	1.001	0.211851539	1
ENSBTAG00000015584	*BMI1*	1'077	1'212	1'166	933	1'399	0.585	0.473239804	1
ENSBTAG00000015587	*TTC32*	312	198	249	270	229	-0.241	0.813960334	1
ENSBTAG00000015588	*TMEM209*	916	874	901	793	1'009	0.347	0.676186182	1
ENSBTAG00000015590	*ICK*	2'234	1'141	1'626	1'935	1'318	-0.554	0.489461197	1
ENSBTAG00000015591	*BT.105440*	5'916	6'275	6'185	5'123	7'246	0.500	0.515694229	1
ENSBTAG00000015592	*GPR84*	9	5	7	8	6	-0.433	0.969858938	1
ENSBTAG00000015593	*KIAA0753*	1'006	640	805	871	739	-0.237	0.779048516	1
ENSBTAG00000015596	*SLCO2B1*	1'912	2'210	2'104	1'656	2'552	0.624	0.430451869	1
ENSBTAG00000015598	*BT.85322*	37'140	41'602	40'101	32'164	48'038	0.579	0.446770928	1
ENSBTAG00000015599	*bta-mir-2897*	1'898	3'009	2'559	1'644	3'474	1.080	0.172476289	1
ENSBTAG00000015602	*BT.49522*	673	455	554	583	525	-0.150	0.866640105	1
ENSBTAG00000015604	*ZNF385A*	1'008	2'100	1'649	873	2'425	1.474	0.069814468	1
ENSBTAG00000015606	*KAZN*	339	239	285	294	276	-0.089	0.931562326	1
ENSBTAG00000015607	*ERMP1*	612	321	450	530	371	-0.516	0.568414086	1
ENSBTAG00000015609	*PREX1*	2'390	3'346	2'967	2'070	3'864	0.900	0.251565035	1
ENSBTAG00000015611	*BT.102962*	6'771	10'916	9'234	5'864	12'605	1.104	0.153154618	1
ENSBTAG00000015612	*UTP6*	6'859	3'957	5'255	5'940	4'569	-0.379	0.62423064	1
ENSBTAG00000015614	*LNX2*	639	441	531	553	509	-0.120	0.894204324	1
ENSBTAG00000015615	*THEMIS*	1	0	0	1	0		1	1
ENSBTAG00000015618	*GDF15*	14	7	10	12	8	-0.585	0.916699259	1
ENSBTAG00000015625	*DCTN4*	2'386	1'561	1'934	2'066	1'802	-0.197	0.804761762	1
ENSBTAG00000015628	*SMPD1*	1'699	1'235	1'449	1'471	1'426	-0.045	0.956849843	1
ENSBTAG00000015630	*RLIM*	897	543	702	777	627	-0.309	0.71817805	1
ENSBTAG00000015632	*TNFRSF18*	197	232	219	171	268	0.651	0.529750827	1
ENSBTAG00000015635	*TNFRSF4*	282	185	229	244	214	-0.193	0.854662031	1
ENSBTAG00000015636	*C7H19ORF66*	442	408	427	383	471	0.300	0.74214294	1
ENSBTAG00000015637	*IMPAD1*	1'283	874	1'060	1'111	1'009	-0.139	0.867375395	1
ENSBTAG00000015638	*IL20RA*	220	273	253	191	315	0.726	0.468715591	1
ENSBTAG00000015639	*ERAL1*	1'926	1'993	1'985	1'668	2'301	0.464	0.557691751	1
ENSBTAG00000015642	*BT.49428*	5'041	3'912	4'441	4'366	4'517	0.049	0.948825857	1
ENSBTAG00000015644	*EID2*	226	53	128	196	61	-1.677	0.17103378	1
ENSBTAG00000015646	*RASSF3*	273	156	208	236	180	-0.392	0.711124114	1
ENSBTAG00000015647	*BT.44981*	1	0	0	1	0		1	1
ENSBTAG00000015648	*DNAJC21*	1'464	790	1'090	1'268	912	-0.475	0.563323321	1
ENSBTAG00000015649	*BT.53706*	102	23	57	88	27	-1.734	0.297740623	1
ENSBTAG00000015651	*BT.39002*	498	507	508	431	585	0.441	0.618703086	1
ENSBTAG00000015654	*PON1*	0	3	2	0	3	Inf	0.878646179	1
ENSBTAG00000015655	*APBB1*	2'045	1'473	1'736	1'771	1'701	-0.058	0.943084615	1
ENSBTAG00000015656	*WSCD1*	221	240	234	191	277	0.534	0.600689785	1
ENSBTAG00000015659	*CIAO1*	2'556	1'480	1'961	2'214	1'709	-0.373	0.63860425	1
ENSBTAG00000015662	*IMPG2*	61	53	57	53	61	0.212	0.905645201	1
ENSBTAG00000015663	*STIP1*	1'520	2'541	2'125	1'316	2'934	1.156	0.147330187	1
ENSBTAG00000015666	*protein_coding*	55	19	35	48	22	-1.118	0.596333837	1
ENSBTAG00000015667	*TMEM127*	394	623	530	341	719	1.076	0.224803671	1
ENSBTAG00000015669	*ADAT1*	156	93	121	135	107	-0.331	0.79198456	1
ENSBTAG00000015670	*PAK3*	95	37	62	82	43	-0.945	0.552062788	1
ENSBTAG00000015676	*AMBP*	2	0	1	2	0		0.974934741	1
ENSBTAG00000015677	*KARS*	13'499	18'777	16'686	11'690	21'682	0.891	0.245016647	1
ENSBTAG00000015678	*TMEM194B*	347	172	250	301	199	-0.597	0.55388635	1
ENSBTAG00000015681	*RGR*	24	30	28	21	35	0.737	0.759630273	1
ENSBTAG00000015683	*BT.64806*	8'267	6'185	7'151	7'159	7'142	-0.004	0.997200755	1

Ensembl gene ID	geneName	counts wildtype horn bud	counts polled horn bud	baseMean	baseMean wildtype horn bud	baseMean polled horn bud	log2FoldChange	pval	padj
ENSBTAG00000015685	*KIF12*	56	94	79	48	109	1.162	0.418690096	1
ENSBTAG00000015686	*TERF2IP*	2'432	1'236	1'767	2'106	1'427	-0.561	0.482118791	1
ENSBTAG00000015690	*PLIN4*	43	104	79	37	120	1.689	0.244132368	1
ENSBTAG00000015692	*BT.96746*	299	175	231	259	202	-0.358	0.729447878	1
ENSBTAG00000015694	*BT.48588*	944	679	801	818	784	-0.060	0.945018187	1
ENSBTAG00000015698	*FYCO1*	875	1'185	1'063	758	1'368	0.853	0.300985794	1
ENSBTAG00000015700	*ELOVL3*	476	201	322	412	232	-0.829	0.386913436	1
ENSBTAG00000015702	*BT.74425*	5	15	11	4	17	2.000	0.647640022	1
ENSBTAG00000015704	*TMCO3*	3'352	3'259	3'333	2'903	3'763	0.374	0.630188117	1
ENSBTAG00000015707	*TREML2*	8	1	4	7	1	-2.585	0.818599781	1
ENSBTAG00000015708	*CXCR6*	22	18	20	19	21	0.126	0.983779847	1
ENSBTAG00000015710	*CD3E*	88	28	54	76	32	-1.237	0.465077655	1
ENSBTAG00000015711	*BTG2*	495	258	363	429	298	-0.525	0.57515328	1
ENSBTAG00000015712	*POU3F4*	26	3	13	23	3	-2.700	0.499863335	1
ENSBTAG00000015713	*TLK2*	1'629	1'142	1'365	1'411	1'319	-0.097	0.905476035	1
ENSBTAG00000015715	*SPOCD1*	2	0	1	2	0		0.974934741	1
ENSBTAG00000015716	*ERO1L*	574	662	631	497	764	0.621	0.471773913	1
ENSBTAG00000015717	*HEY1*	725	227	445	628	262	-1.260	0.167538145	1
ENSBTAG00000015718	*CASP8*	804	477	624	696	551	-0.338	0.69671343	1
ENSBTAG00000015719	*BT.31254*	228	209	219	197	241	0.290	0.781510297	1
ENSBTAG00000015720	*WWP1*	2'376	1'389	1'831	2'058	1'604	-0.359	0.652095719	1
ENSBTAG00000015721	*RAVER1*	2'339	4'078	3'367	2'026	4'709	1.217	0.122232235	1
ENSBTAG00000015723	*CRIPT*	2'412	1'803	2'085	2'089	2'082	-0.005	0.996566292	1
ENSBTAG00000015724	*BPIFB1*	1	2	2	1	2	1.415	0.981979269	1
ENSBTAG00000015727	*IFI47*	67	69	69	58	80	0.457	0.766234287	1
ENSBTAG00000015728	*NSUN3*	1'613	784	1'151	1'397	905	-0.626	0.444844701	1
ENSBTAG00000015730	*BOLA-DMA*	363	201	273	314	232	-0.438	0.658948196	1
ENSBTAG00000015731	*TXNL1*	3'133	2'316	2'694	2'713	2'674	-0.021	0.979952478	1
ENSBTAG00000015732	*BT.22570*	1'878	1'249	1'534	1'626	1'442	-0.173	0.830040007	1
ENSBTAG00000015734	*FAM82B*	467	395	430	404	456	0.173	0.849577342	1
ENSBTAG00000015737	*KPNA7*	0	2	1	0	2	Inf	0.939077559	1
ENSBTAG00000015738	*GIGYF1*	3'299	4'967	4'296	2'857	5'735	1.005	0.197309192	1
ENSBTAG00000015739	*MRC2*	20'125	29'506	25'750	17'429	34'071	0.967	0.206859081	1
ENSBTAG00000015741	*pseudogene*	300	359	337	260	415	0.674	0.476144491	1
ENSBTAG00000015742	*ICAM3*	195	85	134	169	98	-0.783	0.512163187	1
ENSBTAG00000015743	*GMPR*	122	110	116	106	127	0.266	0.834687121	1
ENSBTAG00000015744	*CCNC*	2'178	1'322	1'706	1'886	1'527	-0.305	0.702911247	1
ENSBTAG00000015745	*RAB3IL1*	2'849	2'282	2'551	2'467	2'635	0.095	0.90347319	1
ENSBTAG00000015750	*BT.49011*	19	7	12	16	8	-1.026	0.806704154	1
ENSBTAG00000015752	*SP110*	197	143	168	171	165	-0.047	0.972901696	1
ENSBTAG00000015753	*RSBN1L*	1'131	605	839	979	699	-0.488	0.561514152	1
ENSBTAG00000015757	*PHYHIP*	217	185	201	188	214	0.185	0.864259682	1
ENSBTAG00000015758	*C18H16orf57*	764	668	716	662	771	0.221	0.794892807	1
ENSBTAG00000015759	*pseudogene*	2	0	1	2	0		0.974934741	1
ENSBTAG00000015761	*COQ5*	933	676	794	808	781	-0.050	0.955075642	1
ENSBTAG00000015763	*BT.50868*	5	1	3	4	1	-1.907	0.9188325	1
ENSBTAG00000015764	*EML2*	868	1'053	984	752	1'216	0.694	0.401752315	1
ENSBTAG00000015766	*ZFPM1*	382	866	665	331	1'000	1.596	0.067648953	1
ENSBTAG00000015767	*RNF10*	9'634	7'203	8'330	8'343	8'317	-0.004	0.996176371	1
ENSBTAG00000015769	*VSIG4*	453	432	446	392	499	0.347	0.701330247	1
ENSBTAG00000015771	*YTHDF2*	2'758	1'876	2'277	2'388	2'166	-0.141	0.859086839	1
ENSBTAG00000015772	*STOML1*	487	460	476	422	531	0.333	0.709972481	1
ENSBTAG00000015773	*LRGUK*	6	3	4	5	3	-0.585	0.976586913	1
ENSBTAG00000015776	*HIAT1*	4'144	2'877	3'455	3'589	3'322	-0.111	0.887017061	1
ENSBTAG00000015778	*SASS6*	842	409	601	729	472	-0.627	0.471315467	1
ENSBTAG00000015779	*PML*	626	947	818	542	1'094	1.012	0.230621983	1
ENSBTAG00000015780	*RCN2*	7'848	5'463	6'552	6'797	6'308	-0.108	0.889420092	1
ENSBTAG00000015781	*CCDC76*	2'672	879	1'665	2'314	1'015	-1.189	0.141052666	1
ENSBTAG00000015782	*BT.18085*	1'803	3'240	2'651	1'561	3'741	1.261	0.112172807	1
ENSBTAG00000015784	*HAPLN2*	11	2	6	10	2	-2.044	0.786625039	1
ENSBTAG00000015786	*LRRC39*	9	7	8	8	8	0.052	1	1
ENSBTAG00000015788	*C9ORF93*	152	97	122	132	112	-0.233	0.855092672	1
ENSBTAG00000015792	*C12ORF63*	98	12	49	85	14	-2.615	0.155351317	1
ENSBTAG00000015793	*BT.27433*	540	490	517	468	566	0.275	0.756216945	1
ENSBTAG00000015794	*NES*	13'836	18'351	16'586	11'982	21'190	0.822	0.282770553	1
ENSBTAG00000015796	*DNAJC5*	442	800	653	383	924	1.271	0.143159556	1
ENSBTAG00000015798	*UCKL1*	2'891	2'793	2'864	2'504	3'225	0.365	0.63999083	1
ENSBTAG00000015800	*protein_coding*	25	13	18	22	15	-0.528	0.875356394	1
ENSBTAG00000015801	*EFNB1*	3'268	4'105	3'785	2'830	4'740	0.744	0.339280347	1
ENSBTAG00000015802	*BT.85514*	729	778	765	631	898	0.509	0.546843398	1
ENSBTAG00000015804	*SIGMAR1*	1'628	1'780	1'733	1'410	2'055	0.544	0.495315585	1
ENSBTAG00000015805	*SIPA1L1*	2'697	2'185	2'429	2'336	2'523	0.111	0.887088179	1

Ensembl gene ID	geneName	counts wildtype horn bud	counts polled horn bud	baseMean	baseMean wildtype horn bud	baseMean polled horn bud	log2FoldChange	pval	padj
ENSBTAG00000015806	*BT.101881*	6'108	7'321	6'872	5'290	8'454	0.676	0.379629598	1
ENSBTAG00000015807	*GALT*	1'237	1'327	1'302	1'071	1'532	0.516	0.523757851	1
ENSBTAG00000015810	*PLET1*	1'779	909	1'295	1'541	1'050	-0.554	0.495529481	1
ENSBTAG00000015811	*DPPA4*	1	0	0	1	0		1	1
ENSBTAG00000015815	*CFP*	509	582	556	441	672	0.608	0.487246775	1
ENSBTAG00000015817	*ELK1*	1'657	1'560	1'618	1'435	1'801	0.328	0.681415273	1
ENSBTAG00000015818	*MMP1*	1	2	2	1	2	1.415	0.981979269	1
ENSBTAG00000015820	*UXT*	711	607	658	616	701	0.187	0.828054349	1
ENSBTAG00000015821	*AMPD3*	120	33	71	104	38	-1.447	0.338248149	1
ENSBTAG00000015824	*RNF141*	370	318	344	320	367	0.197	0.83620663	1
ENSBTAG00000015825	*CCDC65*	81	37	56	70	43	-0.715	0.669001698	1
ENSBTAG00000015828	*FKBP11*	3'658	1'771	2'606	3'168	2'045	-0.631	0.422271394	1
ENSBTAG00000015830	*BT.27351*	2	1	1	2	1	-0.585	1	1
ENSBTAG00000015831	*RPL18A*	20'387	28'686	25'390	17'656	33'124	0.908	0.235621275	1
ENSBTAG00000015833	*BT.24588*	4'357	5'943	5'318	3'773	6'862	0.863	0.265456395	1
ENSBTAG00000015834	*ARHGEF4*	792	451	603	686	521	-0.397	0.647960344	1
ENSBTAG00000015835	*PIWIL2*	0	2	1	0	2	Inf	0.939077559	1
ENSBTAG00000015836	*EXOC3L2*	138	106	121	120	122	0.034	0.98443927	1
ENSBTAG00000015839	*MAP4*	11'293	18'230	15'415	9'780	21'050	1.106	0.150902886	1
ENSBTAG00000015840	*HOXD1*	14	33	25	12	38	1.652	0.509598682	1
ENSBTAG00000015841	*CCDC115*	1'064	741	889	921	856	-0.107	0.899754571	1
ENSBTAG00000015843	*SLC6A3*	32	43	39	28	50	0.841	0.672122619	1
ENSBTAG00000015844	*TFPI2*	4'884	2'090	3'321	4'230	2'413	-0.810	0.301056493	1
ENSBTAG00000015848	*PHKA1*	1'288	1'037	1'156	1'115	1'197	0.102	0.900428403	1
ENSBTAG00000015849	*BT.80762*	113	41	73	98	47	-1.048	0.482340059	1
ENSBTAG00000015853	*SDHC*	1'717	1'224	1'450	1'487	1'413	-0.073	0.92896073	1
ENSBTAG00000015856	*C1ORF192*	581	170	350	503	196	-1.358	0.153555381	1
ENSBTAG00000015857	*TPRG1L*	3'339	3'856	3'672	2'892	4'453	0.623	0.423396072	1
ENSBTAG00000015858	*TSR2*	2'415	1'959	2'177	2'091	2'262	0.113	0.8857729	1
ENSBTAG00000015859	*WRAP73*	954	800	875	826	924	0.161	0.847170271	1
ENSBTAG00000015860	*FGD1*	2'574	2'570	2'598	2'229	2'968	0.413	0.598402074	1
ENSBTAG00000015865	*CAPN14*	6	5	5	5	6	0.152	1	1
ENSBTAG00000015866	*ZNF211*	450	221	322	390	255	-0.611	0.523286906	1
ENSBTAG00000015868	*LIG4*	823	642	727	713	741	0.057	0.947670605	1
ENSBTAG00000015872	*PROKR2*	11	22	17	10	25	1.415	0.651185912	1
ENSBTAG00000015874	*CASP3*	2'467	1'611	1'998	2'136	1'860	-0.200	0.801859782	1
ENSBTAG00000015875	*FOXM1*	224	404	330	194	466	1.266	0.186170053	1
ENSBTAG00000015878	*C5H12orf32*	996	1'021	1'021	863	1'179	0.451	0.584008811	1
ENSBTAG00000015879	*TULP3*	2'472	2'958	2'778	2'141	3'416	0.674	0.390128536	1
ENSBTAG00000015880	*PRIMA1*	39	45	43	34	52	0.621	0.744380926	1
ENSBTAG00000015882	*UBE2J2*	1'256	1'179	1'225	1'088	1'361	0.324	0.69018958	1
ENSBTAG00000015885	*PPFIA4*	2'464	1'587	1'983	2'134	1'833	-0.220	0.782544819	1
ENSBTAG00000015887	*FOXJ3*	1'027	1'179	1'125	889	1'361	0.614	0.453020804	1
ENSBTAG00000015888	*PDE7B*	461	262	351	399	303	-0.400	0.67176344	1
ENSBTAG00000015889	*CLCN7*	2'597	3'715	3'269	2'249	4'290	0.932	0.23456807	1
ENSBTAG00000015891	*NSUN4*	322	240	278	279	277	-0.009	0.997791577	1
ENSBTAG00000015892	*BT.49223*	2'231	1'360	1'751	1'932	1'570	-0.299	0.708326678	1
ENSBTAG00000015893	*BT.59510*	69	35	50	60	40	-0.564	0.752515335	1
ENSBTAG00000015894	*WWOX*	181	229	211	157	264	0.754	0.470919765	1
ENSBTAG00000015895	*HGD*	11	2	6	10	2	-2.044	0.786625039	1
ENSBTAG00000015896	*CC2D1B*	1'107	1'225	1'187	959	1'415	0.561	0.491150521	1
ENSBTAG00000015897	*pseudogene*	1	1	1	1	1	0.415	1	1
ENSBTAG00000015898	*RABL3*	1'157	691	900	1'002	798	-0.329	0.694105256	1
ENSBTAG00000015899	*protein_coding*	1'799	976	1'342	1'558	1'127	-0.467	0.564284295	1
ENSBTAG00000015900	*BT.28584*	1'156	1'745	1'508	1'001	2'015	1.009	0.211926971	1
ENSBTAG00000015901	*ARMC1*	4'151	2'239	3'090	3'595	2'585	-0.476	0.543132259	1
ENSBTAG00000015902	*FP*	836	881	871	724	1'017	0.491	0.556495584	1
ENSBTAG00000015904	*RORA*	59	38	47	51	44	-0.220	0.91388753	1
ENSBTAG00000015905	*ARHGAP32*	681	336	489	590	388	-0.604	0.498972051	1
ENSBTAG00000015907	*IRX4*	732	1'173	994	634	1'354	1.095	0.187952675	1
ENSBTAG00000015908	*MBOAT7*	1'097	1'369	1'265	950	1'581	0.735	0.366358019	1
ENSBTAG00000015909	*PDE8A*	890	490	668	771	566	-0.446	0.60383627	1
ENSBTAG00000015910	*ITGB1*	46'020	31'151	37'912	39'854	35'970	-0.148	0.846196914	1
ENSBTAG00000015911	*RWDD2A*	140	97	117	121	112	-0.114	0.934389033	1
ENSBTAG00000015912	*BT.23455*	4'825	3'396	4'050	4'179	3'921	-0.092	0.906754821	1
ENSBTAG00000015913	*MFHAS1*	551	640	608	477	739	0.631	0.466426052	1
ENSBTAG00000015915	*SBSN*	3'052	1'618	2'256	2'643	1'868	-0.501	0.526514404	1
ENSBTAG00000015917	*GAPDHS*	492	278	374	426	321	-0.409	0.661708799	1
ENSBTAG00000015919	*DKFZP564O0823*	294	226	258	255	261	0.036	0.975135094	1
ENSBTAG00000015920	*TMEM147*	3'859	3'652	3'779	3'342	4'217	0.335	0.665101428	1
ENSBTAG00000015924	*CALB1*	7	0	3	6	0		0.769774961	1
ENSBTAG00000015925	*ACN9*	286	196	237	248	226	-0.130	0.902739592	1

Ensembl gene ID	geneName	counts wildtype horn bud	counts polled horn bud	baseMean	baseMean wildtype horn bud	baseMean polled horn bud	log2FoldChange	pval	padj
ENSBTAG00000015926	*ABLIM2*	148	141	145	128	163	0.345	0.767829868	1
ENSBTAG00000015929	*pseudogene*	2	1	1	2	1	-0.585	1	1
ENSBTAG00000015930	*DNAL1*	148	76	108	128	88	-0.546	0.671598066	1
ENSBTAG00000015931	*TTC4*	1'585	1'176	1'365	1'373	1'358	-0.016	0.986360354	1
ENSBTAG00000015935	*IYD*	8	0	3	7	0		0.72953145	1
ENSBTAG00000015936	*BT.35142*	546	355	441	473	410	-0.206	0.822141899	1
ENSBTAG00000015938	*ARAP3*	1'530	1'077	1'284	1'325	1'244	-0.091	0.911683052	1
ENSBTAG00000015940	*SCGB2A2*	1	32	19	1	37	5.415	0.137492753	1
ENSBTAG00000015942	*BT.18516*	648	542	594	561	626	0.157	0.85696886	1
ENSBTAG00000015943	*EFHA1*	2'193	1'095	1'582	1'899	1'264	-0.587	0.464963159	1
ENSBTAG00000015944	*WDR61*	3'369	2'277	2'773	2'918	2'629	-0.150	0.848795399	1
ENSBTAG00000015945	*BT.27224*	118	76	95	102	88	-0.220	0.876693964	1
ENSBTAG00000015946	*RBBP5*	1'856	1'842	1'867	1'607	2'127	0.404	0.610697402	1
ENSBTAG00000015950	*ISOC2*	559	713	654	484	823	0.766	0.373319958	1
ENSBTAG00000015951	*NOL11*	1'915	1'483	1'685	1'658	1'712	0.046	0.953892957	1
ENSBTAG00000015952	*BT.62790*	586	1'093	885	507	1'262	1.314	0.119272246	1
ENSBTAG00000015953	*IL13*	12	2	6	10	2	-2.170	0.758651977	1
ENSBTAG00000015955	*ERGIC1*	5'700	5'555	5'675	4'936	6'414	0.378	0.623293456	1
ENSBTAG00000015957	*IL4*	3	0	1	3	0		0.936647693	1
ENSBTAG00000015958	*GOSR2*	2'410	1'946	2'167	2'087	2'247	0.107	0.892429802	1
ENSBTAG00000015961	*TSPAN2*	139	84	109	120	97	-0.312	0.812122677	1
ENSBTAG00000015962	*MMADHC*	7'691	4'783	6'092	6'661	5'523	-0.270	0.726056852	1
ENSBTAG00000015963	*MGC127919*	495	470	486	429	543	0.340	0.702907568	1
ENSBTAG00000015965	*ZC3H8*	880	425	626	762	491	-0.635	0.463257883	1
ENSBTAG00000015968	*ACSF3*	204	375	305	177	433	1.293	0.183585741	1
ENSBTAG00000015969	*STK40*	1'060	1'321	1'222	918	1'525	0.733	0.368726997	1
ENSBTAG00000015972	*GLRX2*	515	330	414	446	381	-0.227	0.806059121	1
ENSBTAG00000015973	*TRAF3IP1*	1'250	873	1'045	1'083	1'008	-0.103	0.902163472	1
ENSBTAG00000015974	*FLVCR1*	625	395	499	541	456	-0.247	0.782991846	1
ENSBTAG00000015976	*PHF12*	2'940	2'841	2'913	2'546	3'281	0.366	0.639513405	1
ENSBTAG00000015977	*SIKE1*	584	411	490	506	475	-0.092	0.920799876	1
ENSBTAG00000015978	*BT.70631*	11'980	4'693	7'897	10'375	5'419	-0.937	0.225042127	1
ENSBTAG00000015980	*BT.30099*	8'194	13'893	11'569	7'096	16'042	1.177	0.127708161	1
ENSBTAG00000015981	*ETV1*	285	68	163	247	79	-1.652	0.147531655	1
ENSBTAG00000015982	*BT.44040*	639	408	512	553	471	-0.232	0.795065314	1
ENSBTAG00000015984	*CADM4*	1'733	1'048	1'355	1'501	1'210	-0.311	0.701609907	1
ENSBTAG00000015985	*GPR20*	7	3	5	6	3	-0.807	0.947428618	1
ENSBTAG00000015986	*NDE1*	3'334	4'193	3'864	2'887	4'842	0.746	0.337928504	1
ENSBTAG00000015987	*protein_coding*	3	0	1	3	0		0.936647693	1
ENSBTAG00000015988	*BT.100599*	1'664	929	1'257	1'441	1'073	-0.426	0.600780971	1
ENSBTAG00000015989	*RPS5*	43'378	46'369	45'554	37'566	53'542	0.511	0.501052444	1
ENSBTAG00000015991	*BT.64827*	2'391	2'607	2'540	2'071	3'010	0.540	0.491818735	1
ENSBTAG00000015994	*LRRC63*	7	3	5	6	3	-0.807	0.947428618	1
ENSBTAG00000015996	*GPC1*	11'734	6'521	8'846	10'162	7'530	-0.432	0.573082728	1
ENSBTAG00000016000	*BT.37093*	536	241	371	464	278	-0.738	0.428909739	1
ENSBTAG00000016002	*FAM169A*	213	229	224	184	264	0.520	0.61426391	1
ENSBTAG00000016004	*METAP1*	783	960	893	678	1'109	0.709	0.395293154	1
ENSBTAG00000016005	*PPP3CA*	3'280	1'567	2'325	2'841	1'809	-0.651	0.410233265	1
ENSBTAG00000016006	*ANKRD11*	5'206	5'284	5'305	4'509	6'101	0.436	0.571092001	1
ENSBTAG00000016007	*ADH5*	6'114	5'255	5'681	5'295	6'068	0.197	0.797880325	1
ENSBTAG00000016010	*KLHL7*	1'607	836	1'179	1'392	965	-0.528	0.518481447	1
ENSBTAG00000016012	*BT.88369*	414	315	361	359	364	0.021	0.984900278	1
ENSBTAG00000016013	*BT.18153*	254	56	142	220	65	-1.766	0.137751647	1
ENSBTAG00000016014	*BT.61283*	280	148	207	242	171	-0.505	0.633118836	1
ENSBTAG00000016017	*CCDC40*	177	128	151	153	148	-0.053	0.970624742	1
ENSBTAG00000016021	*GAA*	727	704	721	630	813	0.369	0.664292117	1
ENSBTAG00000016023	*EIF4A3*	6'256	5'454	5'858	5'418	6'298	0.217	0.777289622	1
ENSBTAG00000016024	*MYL9*	20'644	17'669	19'140	17'878	20'402	0.191	0.801741925	1
ENSBTAG00000016028	*BT.87313*	465	600	548	403	693	0.783	0.373148492	1
ENSBTAG00000016030	*HOXD10*	58	63	61	50	73	0.534	0.739845162	1
ENSBTAG00000016032	*protein_coding*	274	153	207	237	177	-0.426	0.687922099	1
ENSBTAG00000016033	*HOXD9*	154	389	291	133	449	1.752	0.077641368	1
ENSBTAG00000016034	*EXOSC5*	314	378	354	272	436	0.683	0.466629189	1
ENSBTAG00000016037	*BCKDHA*	1'902	2'395	2'206	1'647	2'766	0.748	0.344776728	1
ENSBTAG00000016038	*GCC2*	629	403	505	545	465	-0.227	0.799823002	1
ENSBTAG00000016041	*B3GNT8*	117	132	127	101	152	0.589	0.626468796	1
ENSBTAG00000016042	*TM6SF2*	14	7	10	12	8	-0.585	0.916699259	1
ENSBTAG00000016043	*GNB3*	1'408	1'278	1'348	1'219	1'476	0.275	0.733092953	1
ENSBTAG00000016045	*TRIB2*	2'283	958	1'542	1'977	1'106	-0.838	0.298759973	1
ENSBTAG00000016046	*GOPC*	1'214	781	977	1'051	902	-0.221	0.790224061	1
ENSBTAG00000016048	*CPT1B*	291	298	298	252	344	0.449	0.642861409	1
ENSBTAG00000016050	*BT.49081*	3'259	2'213	2'689	2'822	2'555	-0.143	0.855731526	1

Ensembl gene ID	geneName	counts wildtype horn bud	counts polled horn bud	baseMean	baseMean wildtype horn bud	baseMean polled horn bud	log2FoldChange	pval	padj
ENSBTAG00000016052	*protein_coding*	1	1	1	1	1	0.415	1	1
ENSBTAG00000016053	*ARSA*	1'071	1'193	1'153	928	1'378	0.571	0.484740343	1
ENSBTAG00000016057	*CSRP1*	4'487	6'410	5'644	3'886	7'402	0.930	0.23034361	1
ENSBTAG00000016058	*FAM83B*	855	844	858	740	975	0.396	0.635121108	1
ENSBTAG00000016060	*CREM*	319	201	254	276	232	-0.251	0.805071232	1
ENSBTAG00000016061	*RSAD2*	213	80	138	184	92	-0.998	0.398461786	1
ENSBTAG00000016062	*C21H14orf28*	147	107	125	127	124	-0.043	0.979866211	1
ENSBTAG00000016069	*ARF4*	11'890	7'484	9'469	10'297	8'642	-0.253	0.741731595	1
ENSBTAG00000016070	*LINGO2*	54	62	59	47	72	0.614	0.706210226	1
ENSBTAG00000016071	*HHIP*	202	429	335	175	495	1.502	0.117859729	1
ENSBTAG00000016072	*SHC2*	391	455	432	339	525	0.634	0.484906656	1
ENSBTAG00000016073	*UGCG*	579	296	422	501	342	-0.553	0.545032302	1
ENSBTAG00000016074	*ZNF143*	884	751	816	766	867	0.180	0.830655525	1
ENSBTAG00000016075	*BT.43161*	952	762	852	824	880	0.094	0.911165237	1
ENSBTAG00000016076	*TRAK1*	524	435	478	454	502	0.146	0.870907792	1
ENSBTAG00000016077	*ZNF317*	802	647	721	695	747	0.105	0.90218871	1
ENSBTAG00000016078	*BT.63069*	178	208	197	154	240	0.640	0.547560676	1
ENSBTAG00000016079	*COX4I1*	8'469	7'481	7'986	7'334	8'638	0.236	0.757492215	1
ENSBTAG00000016080	*VPS13D*	1'931	1'383	1'635	1'672	1'597	-0.067	0.935117706	1
ENSBTAG00000016081	*PTMA*	11'064	17'370	14'819	9'582	20'057	1.066	0.166060287	1
ENSBTAG00000016085	*IRAK1*	2'310	2'147	2'240	2'001	2'479	0.309	0.694266872	1
ENSBTAG00000016091	*KLHL25*	472	533	512	409	615	0.590	0.504811289	1
ENSBTAG00000016092	*SPATS2L*	8'638	4'337	6'244	7'481	5'008	-0.579	0.452906844	1
ENSBTAG00000016093	*PLP2*	4'969	5'329	5'228	4'303	6'153	0.516	0.503611238	1
ENSBTAG00000016094	*SLC36A1*	113	139	129	98	161	0.714	0.552455725	1
ENSBTAG00000016095	*LMO6*	493	600	560	427	693	0.698	0.425145284	1
ENSBTAG00000016096	*SYP*	1'326	710	984	1'148	820	-0.486	0.557260944	1
ENSBTAG00000016097	*CACNA1F*	1	0	0	1	0		1	1
ENSBTAG00000016098	*C22H3ORF10*	4'388	2'976	3'618	3'800	3'436	-0.145	0.85265141	1
ENSBTAG00000016101	*BT.79817*	533	337	425	462	389	-0.246	0.788690738	1
ENSBTAG00000016103	*CBFB*	1'637	888	1'222	1'418	1'025	-0.467	0.566475683	1
ENSBTAG00000016104	*BTAF1*	2'078	1'168	1'574	1'800	1'349	-0.416	0.604376276	1
ENSBTAG00000016105	*H2AFY*	6'509	4'683	5'522	5'637	5'407	-0.060	0.938831342	1
ENSBTAG00000016107	*PCYT1B*	163	63	107	141	73	-0.956	0.457118063	1
ENSBTAG00000016108	*BTBD2*	4'740	6'241	5'656	4'105	7'206	0.812	0.293859693	1
ENSBTAG00000016109	*DHRS12*	217	117	162	188	135	-0.476	0.674225127	1
ENSBTAG00000016112	*BT.62537*	2	3	3	2	3	1.000	0.97171833	1
ENSBTAG00000016113	*ELSPBP1*	6	1	3	5	1	-2.170	0.885240772	1
ENSBTAG00000016116	*pseudogene*	770	1'086	960	667	1'254	0.911	0.273158414	1
ENSBTAG00000016121	*KRT75*	348	706	558	301	815	1.436	0.105771493	1
ENSBTAG00000016122	*PIGT*	1'814	1'932	1'901	1'571	2'231	0.506	0.523896928	1
ENSBTAG00000016124	*PACSIN1*	375	187	270	325	216	-0.589	0.552733973	1
ENSBTAG00000016125	*KCNA7*	2	8	5	2	9	2.415	0.75674087	1
ENSBTAG00000016126	*NUP85*	2'365	2'064	2'216	2'048	2'383	0.219	0.781221725	1
ENSBTAG00000016127	*BT.72701*	219	125	167	190	144	-0.394	0.726402218	1
ENSBTAG00000016128	*BT.21158*	2'948	2'797	2'891	2'553	3'230	0.339	0.663922201	1
ENSBTAG00000016131	*NCAPG2*	822	625	717	712	722	0.020	0.982601056	1
ENSBTAG00000016133	*MRPS7*	1'220	1'070	1'146	1'057	1'236	0.226	0.782074085	1
ENSBTAG00000016137	*ZNF608*	3'010	1'822	2'355	2'607	2'104	-0.309	0.695207874	1
ENSBTAG00000016138	*CGA*	5	0	2	4	0		0.853268594	1
ENSBTAG00000016139	*TMEM120A*	1'785	893	1'289	1'546	1'031	-0.584	0.472311725	1
ENSBTAG00000016140	*PPFIBP2*	1'878	1'249	1'534	1'626	1'442	-0.173	0.830040007	1
ENSBTAG00000016142	*CYB5R2*	330	174	243	286	201	-0.508	0.61693536	1
ENSBTAG00000016145	*ADAMTS19*	583	728	673	505	841	0.735	0.391063248	1
ENSBTAG00000016147	*CETN4*	1'054	523	758	913	604	-0.596	0.48219828	1
ENSBTAG00000016148	*LCN6*	7	6	6	6	7	0.193	1	1
ENSBTAG00000016149	*C9*	1	0	0	1	0		1	1
ENSBTAG00000016151	*VTN*	0	1	1	0	1	Inf	0.993540919	1
ENSBTAG00000016152	*DAB2*	14'011	7'788	10'563	12'134	8'993	-0.432	0.572705626	1
ENSBTAG00000016155	*BT.49306*	525	439	481	455	507	0.157	0.861521973	1
ENSBTAG00000016156	*MAPK3*	2'864	3'443	3'228	2'480	3'976	0.681	0.383442755	1
ENSBTAG00000016161	*FLII*	4'391	8'528	6'825	3'803	9'847	1.373	0.078464937	1
ENSBTAG00000016163	*BT.45038*	2	1	1	2	1	-0.585	1	1
ENSBTAG00000016164	*LMBRD1*	2'461	1'630	2'007	2'131	1'882	-0.179	0.821861555	1
ENSBTAG00000016165	*KRT7*	261	423	357	226	488	1.112	0.237688974	1
ENSBTAG00000016166	*protein_coding*	6	1	3	5	1	-2.170	0.885240772	1
ENSBTAG00000016167	*ATL2*	2'069	1'513	1'769	1'792	1'747	-0.036	0.964900912	1
ENSBTAG00000016168	*APLP2*	17'772	22'596	20'741	15'391	26'092	0.761	0.319006963	1
ENSBTAG00000016169	*ID1*	1'877	740	1'240	1'626	854	-0.928	0.256490619	1
ENSBTAG00000016170	*KCNJ11*	58	103	85	50	119	1.244	0.37365426	1
ENSBTAG00000016171	*COX4I2*	165	128	145	143	148	0.049	0.972162656	1
ENSBTAG00000016174	*BT.79166*	9'849	9'138	9'541	8'529	10'552	0.307	0.687781399	1

Ensembl gene ID	geneName	counts wildtype horn bud	counts polled horn bud	baseMean	baseMean wildtype horn bud	baseMean polled horn bud	log2FoldChange	pval	padj
ENSBTAG00000016175	*SPNS3*	115	161	143	100	186	0.900	0.439502695	1
ENSBTAG00000016177	*FGL1*	483	161	302	418	186	-1.170	0.229748414	1
ENSBTAG00000016179	*CIB3*	2	5	4	2	6	1.737	0.883335552	1
ENSBTAG00000016183	*Mrz 04*	45	3	21	39	3	-3.492	0.248154783	1
ENSBTAG00000016185	*ENAH*	1'663	1'170	1'396	1'440	1'351	-0.092	0.910401236	1
ENSBTAG00000016186	*protein_coding*	8	2	5	7	2	-1.585	0.87391615	1
ENSBTAG00000016187	*GRM6*	3	0	1	3	0		0.936647693	1
ENSBTAG00000016189	*pseudogene*	29	4	15	25	5	-2.443	0.497961729	1
ENSBTAG00000016190	*ZNF394*	1'245	789	995	1'078	911	-0.243	0.769707654	1
ENSBTAG00000016191	*ZNF32*	1'952	1'486	1'703	1'690	1'716	0.022	0.978601244	1
ENSBTAG00000016192	*MDH1B*	61	19	37	53	22	-1.268	0.532532503	1
ENSBTAG00000016193	*BT.28280*	737	684	714	638	790	0.307	0.717836466	1
ENSBTAG00000016194	*FBXO32*	90	90	91	78	104	0.415	0.7626997	1
ENSBTAG00000016197	*BT.105081*	6'210	6'687	6'550	5'378	7'721	0.522	0.497344619	1
ENSBTAG00000016198	*EP300*	3'458	2'570	2'981	2'995	2'968	-0.013	0.987756662	1
ENSBTAG00000016199	*CLINT1*	6'648	4'727	5'608	5'757	5'458	-0.077	0.921254205	1
ENSBTAG00000016202	*TREH*	0	2	1	0	2	Inf	0.939077559	1
ENSBTAG00000016203	*ZNF16*	741	800	783	642	924	0.526	0.53290079	1
ENSBTAG00000016204	*C1RL*	25	8	15	22	9	-1.229	0.723874526	1
ENSBTAG00000016206	*MAOA*	591	438	509	512	506	-0.017	0.987803041	1
ENSBTAG00000016208	*TGM2*	994	495	716	861	572	-0.591	0.488535573	1
ENSBTAG00000016209	*SLURP1*	3	0	1	3	0		0.936647693	1
ENSBTAG00000016210	*LYPD2*	24	0	10	21	0		0.291206645	1
ENSBTAG00000016211	*GCDH*	1'732	1'757	1'764	1'500	2'029	0.436	0.584158684	1
ENSBTAG00000016213	*SYCE2*	334	271	301	289	313	0.113	0.908885417	1
ENSBTAG00000016217	*RBM43*	1'345	1'070	1'200	1'165	1'236	0.085	0.917022679	1
ENSBTAG00000016218	*BT.34207*	621	922	801	538	1'065	0.985	0.243983136	1
ENSBTAG00000016220	*WIPF3*	371	293	330	321	338	0.075	0.939899391	1
ENSBTAG00000016221	*AMOT*	893	798	847	773	921	0.253	0.762548831	1
ENSBTAG00000016223	*SCRN1*	2'456	969	1'623	2'127	1'119	-0.927	0.249723139	1
ENSBTAG00000016224	*RPS7*	93'750	81'389	87'585	81'190	93'980	0.211	0.780169182	1
ENSBTAG00000016225	*COLEC11*	23	3	12	20	3	-2.524	0.556355022	1
ENSBTAG00000016227	*ASCL1*	54	0	23	47	0		0.053795713	1
ENSBTAG00000016228	*NMD3*	2'800	1'594	2'133	2'425	1'841	-0.398	0.615488013	1
ENSBTAG00000016229	*KLF9*	149	23	78	129	27	-2.281	0.126805778	1
ENSBTAG00000016230	*BRWD3*	232	159	192	201	184	-0.130	0.908594218	1
ENSBTAG00000016231	*POFUT1*	815	771	798	706	890	0.335	0.690384687	1
ENSBTAG00000016234	*DUOX2*	109	33	66	94	38	-1.309	0.399196644	1
ENSBTAG00000016235	*RNASEH1*	1'087	565	797	941	652	-0.529	0.530715809	1
ENSBTAG00000016236	*SLC12A6*	703	501	594	609	579	-0.074	0.935032823	1
ENSBTAG00000016239	*DUOXA2*	28	3	14	24	3	-2.807	0.465072461	1
ENSBTAG00000016240	*RNF157*	532	587	569	461	678	0.557	0.52347733	1
ENSBTAG00000016242	*BT.74243*	483	323	396	418	373	-0.165	0.860137953	1
ENSBTAG00000016243	*RBL2*	4'065	2'328	3'104	3'520	2'688	-0.389	0.618706654	1
ENSBTAG00000016244	*VWA3A*	56	2	25	48	2	-4.392	0.128674481	1
ENSBTAG00000016246	*DUOX1*	756	636	695	655	734	0.166	0.846502994	1
ENSBTAG00000016247	*BT.68754*	3	1	2	3	1	-1.170	0.985253077	1
ENSBTAG00000016249	*NAGS*	35	43	40	30	50	0.712	0.717092181	1
ENSBTAG00000016250	*AKTIP*	1'290	767	1'001	1'117	886	-0.335	0.685722184	1
ENSBTAG00000016251	*TMEM101*	1'519	1'979	1'800	1'315	2'285	0.797	0.318445607	1
ENSBTAG00000016252	*LSM12*	1'729	1'341	1'523	1'497	1'548	0.048	0.952025917	1
ENSBTAG00000016253	*G6PC3*	2'122	1'409	1'732	1'838	1'627	-0.176	0.82664194	1
ENSBTAG00000016254	*HDAC5*	4'900	6'910	6'111	4'244	7'979	0.911	0.239133837	1
ENSBTAG00000016255	*PLEK2*	315	386	359	273	446	0.708	0.449019562	1
ENSBTAG00000016257	*ZNF74*	741	938	862	642	1'083	0.755	0.367026126	1
ENSBTAG00000016259	*KIAA1958*	101	77	88	87	89	0.024	0.995066997	1
ENSBTAG00000016260	*LRRK2*	1'108	691	879	960	798	-0.266	0.750907344	1
ENSBTAG00000016263	*BT.66194*	429	156	276	372	180	-1.044	0.291518141	1
ENSBTAG00000016264	*BT.87555*	5'912	9'297	7'928	5'120	10'735	1.068	0.167320324	1
ENSBTAG00000016265	*DNAJA1*	3'158	1'984	2'513	2'735	2'291	-0.256	0.745641983	1
ENSBTAG00000016266	*SDHD*	2'410	2'017	2'208	2'087	2'329	0.158	0.840702439	1
ENSBTAG00000016267	*BT.22969*	10'965	3'389	6'705	9'496	3'913	-1.279	0.100549133	1
ENSBTAG00000016268	*XRCC1*	1'790	2'102	1'989	1'550	2'427	0.647	0.414972413	1
ENSBTAG00000016269	*ME2*	752	672	714	651	776	0.253	0.766501539	1
ENSBTAG00000016271	*SNRPF*	2'549	1'882	2'190	2'207	2'173	-0.023	0.978451026	1
ENSBTAG00000016272	*TEX11*	7	1	4	6	1	-2.392	0.851750348	1
ENSBTAG00000016274	*CCDC38*	50	31	40	43	36	-0.275	0.900392623	1
ENSBTAG00000016275	*AMDHD1*	534	202	348	462	233	-0.987	0.296612604	1
ENSBTAG00000016276	*HAL*	8	2	5	7	2	-1.585	0.87391615	1
ENSBTAG00000016277	*MOSC2*	1'559	891	1'189	1'350	1'029	-0.392	0.631276274	1
ENSBTAG00000016278	*RPL30*	49'408	41'055	45'097	42'789	47'406	0.148	0.84491696	1
ENSBTAG00000016279	*EMILIN3*	4'127	5'698	5'077	3'574	6'579	0.880	0.256387867	1

Ensembl gene ID	geneName	counts wildtype horn bud	counts polled horn bud	baseMean	baseMean wildtype horn bud	baseMean polled horn bud	log2FoldChange	pval	padj
ENSBTAG00000016281	*PREP*	2'109	2'302	2'242	1'826	2'658	0.541	0.492507031	1
ENSBTAG00000016282	*BT.69225*	12'289	9'055	10'549	10'643	10'456	-0.026	0.974142456	1
ENSBTAG00000016283	*TMEM63C*	43	13	26	37	15	-1.311	0.596402998	1
ENSBTAG00000016284	*IL7*	19	12	15	16	14	-0.248	0.963115943	1
ENSBTAG00000016285	*NGB*	2	0	1	2	0		0.974934741	1
ENSBTAG00000016287	*UNK*	1'521	1'976	1'799	1'317	2'282	0.793	0.320916456	1
ENSBTAG00000016290	*MOB1B*	157	121	138	136	140	0.039	0.979469982	1
ENSBTAG00000016291	*BT.61724*	155	197	181	134	227	0.761	0.484333098	1
ENSBTAG00000016293	*RNF18*	1	1	1	1	1	0.415	1	1
ENSBTAG00000016294	*BT.35916*	1'067	363	672	924	419	-1.140	0.18699681	1
ENSBTAG00000016295	*RPP25L*	1'309	905	1'089	1'134	1'045	-0.117	0.887678875	1
ENSBTAG00000016296	*GPD1*	1'010	892	952	875	1'030	0.236	0.77583263	1
ENSBTAG00000016297	*BT.102239*	355	209	274	307	241	-0.349	0.725265981	1
ENSBTAG00000016298	*TM9SF3*	13'699	8'785	11'004	11'864	10'144	-0.226	0.768139212	1
ENSBTAG00000016299	*ZNF784*	554	373	455	480	431	-0.156	0.865326951	1
ENSBTAG00000016302	*BT.96895*	453	179	300	392	207	-0.925	0.341667995	1
ENSBTAG00000016305	*ATP13A4*	210	60	126	182	69	-1.392	0.25597198	1
ENSBTAG00000016306	*PFDN4*	5	2	3	4	2	-0.907	0.965043111	1
ENSBTAG00000016307	*CDC7L1*	4'096	3'274	3'664	3'547	3'780	0.092	0.905413235	1
ENSBTAG00000016309	*ATP6V1D*	2'861	1'783	2'268	2'478	2'059	-0.267	0.735436585	1
ENSBTAG00000016311	*EIF2S1*	1'137	670	879	985	774	-0.348	0.677610444	1
ENSBTAG00000016312	*BT.62733*	912	549	712	790	634	-0.317	0.710670265	1
ENSBTAG00000016313	*BT.58971*	2'805	1'625	2'153	2'429	1'876	-0.373	0.637952418	1
ENSBTAG00000016315	*COTL1*	2'397	2'499	2'481	2'076	2'886	0.475	0.545188582	1
ENSBTAG00000016316	*ZZEF1*	802	1'108	987	695	1'279	0.881	0.287896454	1
ENSBTAG00000016319	*CYB5D2*	375	239	300	325	276	-0.235	0.811081042	1
ENSBTAG00000016320	*LMO1*	353	714	565	306	824	1.431	0.1062672	1
ENSBTAG00000016323	*SLC7A14*	6	0	3	5	0		0.81115604	1
ENSBTAG00000016324	*FBXL13*	14	16	15	12	18	0.608	0.869447069	1
ENSBTAG00000016326	*C3H1orf52*	813	373	567	704	431	-0.709	0.41851777	1
ENSBTAG00000016327	*SLC28A2*	0	1	1	0	1	Inf	0.993540919	1
ENSBTAG00000016328	*SFPQ*	13'804	7'703	10'425	11'955	8'895	-0.427	0.577762786	1
ENSBTAG00000016330	*BT.95042*	3	11	8	3	13	2.290	0.692284022	1
ENSBTAG00000016331	*ROGDI*	2'072	1'277	1'634	1'794	1'475	-0.283	0.724087234	1
ENSBTAG00000016332	*ING3*	693	693	700	600	800	0.415	0.626195268	1
ENSBTAG00000016335	*BT.22521*	824	774	804	714	894	0.325	0.699199218	1
ENSBTAG00000016336	*MGEA5*	9'167	5'550	7'174	7'939	6'409	-0.309	0.688007845	1
ENSBTAG00000016337	*THEM2*	1'935	1'759	1'853	1'676	2'031	0.277	0.726593584	1
ENSBTAG00000016338	*BT.59273*	2'606	2'178	2'386	2'257	2'515	0.156	0.84222173	1
ENSBTAG00000016341	*RGS5*	593	240	395	514	277	-0.890	0.335752107	1
ENSBTAG00000016343	*BT.24395*	812	181	456	703	209	-1.750	0.057383364	1
ENSBTAG00000016344	*PIK3R6*	153	111	130	133	128	-0.048	0.976076987	1
ENSBTAG00000016345	*IL2RB*	16	34	27	14	39	1.503	0.535161027	1
ENSBTAG00000016348	*XKR7*	11	0	5	10	0		0.617662983	1
ENSBTAG00000016349	*TEAD2*	2'818	5'158	4'198	2'440	5'956	1.287	0.100949659	1
ENSBTAG00000016350	*NACC1*	1'700	2'501	2'180	1'472	2'888	0.972	0.221127112	1
ENSBTAG00000016352	*STX10*	1'532	1'368	1'453	1'327	1'580	0.252	0.754120783	1
ENSBTAG00000016354	*IER2*	1'787	1'641	1'721	1'548	1'895	0.292	0.713802224	1
ENSBTAG00000016355	*UVRAG*	463	391	426	401	451	0.171	0.851787138	1
ENSBTAG00000016357	*BT.42521*	29	20	24	25	23	-0.121	0.981341314	1
ENSBTAG00000016359	*SLCO4C1*	32	14	22	28	16	-0.778	0.782869547	1
ENSBTAG00000016362	*BT.46466*	371	305	337	321	352	0.132	0.890598924	1
ENSBTAG00000016363	*BT.39597*	5'584	3'725	4'569	4'836	4'301	-0.169	0.827686441	1
ENSBTAG00000016365	*BT.45011*	40	0	17	35	0		0.116452486	1
ENSBTAG00000016366	*BT.29087*	15	1	7	13	1	-3.492	0.607205542	1
ENSBTAG00000016367	*RBM18*	1'109	1'068	1'097	960	1'233	0.361	0.659493189	1
ENSBTAG00000016368	*LRPPRC*	3'422	2'443	2'892	2'964	2'821	-0.071	0.928541363	1
ENSBTAG00000016369	*TSEN15*	2'115	1'455	1'756	1'832	1'680	-0.125	0.876926801	1
ENSBTAG00000016370	*pseudogene*	2	0	1	2	0		0.974934741	1
ENSBTAG00000016373	*TTC26*	291	298	298	252	344	0.449	0.642861409	1
ENSBTAG00000016374	*OGFRL1*	182	152	167	158	176	0.155	0.892959374	1
ENSBTAG00000016377	*IPP*	1'049	518	753	908	598	-0.603	0.477373461	1
ENSBTAG00000016378	*LRP10*	3'861	5'139	4'639	3'344	5'934	0.828	0.286411486	1
ENSBTAG00000016381	*REM2*	50	28	38	43	32	-0.421	0.843116681	1
ENSBTAG00000016384	*KRT32*	20	35	29	17	40	1.222	0.59690349	1
ENSBTAG00000016385	*F10*	1	0	0	1	0		1	1
ENSBTAG00000016387	*GDAP2*	912	633	760	790	731	-0.112	0.896829306	1
ENSBTAG00000016388	*SLCO4A1*	39	26	32	34	30	-0.170	0.953742426	1
ENSBTAG00000016389	*UBL4B*	4	11	8	3	13	1.874	0.733957485	1
ENSBTAG00000016391	*RPL37*	37'632	21'687	28'816	32'590	25'042	-0.380	0.617880535	1
ENSBTAG00000016396	*PDE11A*	11	0	5	10	0		0.617662983	1
ENSBTAG00000016397	*CDYL*	1'587	1'108	1'327	1'374	1'279	-0.103	0.899848718	1

Ensembl gene ID	geneName	counts wildtype horn bud	counts polled horn bud	baseMean	baseMean wildtype horn bud	baseMean polled horn bud	log2FoldChange	pval	padj
ENSBTAG00000016398	*DNAJC22*	4	4	4	3	5	0.415	0.994759363	1
ENSBTAG00000016399	*AKAP4*	48	30	38	42	35	-0.263	0.907543901	1
ENSBTAG00000016401	*OPTN*	2'179	1'415	1'760	1'887	1'634	-0.208	0.795192906	1
ENSBTAG00000016406	*MCM10*	370	206	279	320	238	-0.430	0.663330414	1
ENSBTAG00000016411	*BT.35049*	925	1'831	1'458	801	2'114	1.400	0.086445795	1
ENSBTAG00000016412	*BT.45725*	1'565	751	1'111	1'355	867	-0.644	0.43273078	1
ENSBTAG00000016413	*DUSP26*	546	518	535	473	598	0.339	0.70004949	1
ENSBTAG00000016414	*BT.61412*	213	255	239	184	294	0.675	0.506121903	1
ENSBTAG00000016415	*LTA4H*	2'248	1'824	2'026	1'947	2'106	0.114	0.885765986	1
ENSBTAG00000016420	*CTNNB1*	33'675	19'998	26'128	29'163	23'092	-0.337	0.658571696	1
ENSBTAG00000016421	*pseudogene*	428	483	464	371	558	0.589	0.511322876	1
ENSBTAG00000016424	*MMP25*	111	50	77	96	58	-0.736	0.614454798	1
ENSBTAG00000016427	*COMMD7*	1'942	1'297	1'590	1'682	1'498	-0.167	0.835618699	1
ENSBTAG00000016429	*TMEM205*	141	143	144	122	165	0.435	0.709399264	1
ENSBTAG00000016430	*METTL8*	156	106	129	135	122	-0.142	0.912579746	1
ENSBTAG00000016432	*BT.20969*	658	423	529	570	488	-0.222	0.802785206	1
ENSBTAG00000016433	*FANCB*	7	8	8	6	9	0.608	0.932722577	1
ENSBTAG00000016435	*NOM1*	1'309	1'698	1'547	1'134	1'961	0.790	0.325833006	1
ENSBTAG00000016438	*CCDC79*	38	8	21	33	9	-1.833	0.516703694	1
ENSBTAG00000016441	*BT.29313*	1'600	1'481	1'548	1'386	1'710	0.304	0.704656666	1
ENSBTAG00000016442	*MAP4K3*	3'780	1'577	2'547	3'274	1'821	-0.846	0.283707291	1
ENSBTAG00000016444	*FAM134B*	489	376	429	423	434	0.036	0.970507345	1
ENSBTAG00000016445	*YME1L1*	6'923	5'505	6'176	5'995	6'357	0.084	0.912092025	1
ENSBTAG00000016446	*GOT1L1*	11	13	12	10	15	0.656	0.880852037	1
ENSBTAG00000016448	*ZBTB40*	1'186	2'030	1'686	1'027	2'344	1.190	0.140037422	1
ENSBTAG00000016450	*CDNF*	27	40	35	23	46	0.982	0.639111274	1
ENSBTAG00000016451	*HSPA14*	2'761	1'873	2'277	2'391	2'163	-0.145	0.855217854	1
ENSBTAG00000016455	*CACHD1*	2'869	2'583	2'734	2'485	2'983	0.264	0.735964311	1
ENSBTAG00000016456	*CDC42BPB*	5'425	7'623	6'750	4'698	8'802	0.906	0.241130478	1
ENSBTAG00000016457	*FXR1*	5'912	4'126	4'942	5'120	4'764	-0.104	0.893791723	1
ENSBTAG00000016462	*TCF7L2*	2'095	3'881	3'148	1'814	4'481	1.305	0.098851265	1
ENSBTAG00000016465	*BT.65963*	992	1'312	1'187	859	1'515	0.818	0.316802985	1
ENSBTAG00000016467	*SLC22A17*	8'801	7'979	8'418	7'622	9'213	0.274	0.720440794	1
ENSBTAG00000016469	*TNKS1BP1*	8'514	14'357	11'976	7'373	16'578	1.169	0.130113418	1
ENSBTAG00000016470	*NADSYN1*	1'465	1'043	1'237	1'269	1'204	-0.075	0.927964274	1
ENSBTAG00000016471	*BT.65397*	779	982	904	675	1'134	0.749	0.368863558	1
ENSBTAG00000016472	*BT.87276*	269	182	222	233	210	-0.149	0.890219322	1
ENSBTAG00000016473	*NAT1*	338	329	336	293	380	0.376	0.691410753	1
ENSBTAG00000016477	*C19ORF35*	3	0	1	3	0		0.936647693	1
ENSBTAG00000016478	*LINGO3*	10	5	7	9	6	-0.585	0.94357809	1
ENSBTAG00000016481	*BT.87360*	14'927	15'587	15'463	12'927	17'998	0.477	0.531176746	1
ENSBTAG00000016483	*pseudogene*	1	2	2	1	2	1.415	0.981979269	1
ENSBTAG00000016484	*ATP11C*	563	353	448	488	408	-0.258	0.776645286	1
ENSBTAG00000016485	*OPN1SW*	0	1	1	0	1	Inf	0.993540919	1
ENSBTAG00000016486	*SORBS2*	108	41	70	94	47	-0.982	0.515355881	1
ENSBTAG00000016494	*RELT*	554	275	399	480	318	-0.595	0.518310694	1
ENSBTAG00000016495	*LINS*	709	520	607	614	600	-0.032	0.973134661	1
ENSBTAG00000016496	*ZNF641*	67	76	73	58	88	0.597	0.688421252	1
ENSBTAG00000016497	*FAM168A*	201	237	224	174	274	0.653	0.526573549	1
ENSBTAG00000016498	*PHACTR2*	171	70	114	148	81	-0.874	0.486838801	1
ENSBTAG00000016499	*OPN5*	1	0	0	1	0		1	1
ENSBTAG00000016501	*ARHGAP1*	3'694	4'199	4'024	3'199	4'849	0.600	0.439518098	1
ENSBTAG00000016502	*BT.38521*	1'169	1'051	1'113	1'012	1'214	0.262	0.74911707	1
ENSBTAG00000016504	*BT.38302*	987	1'030	1'022	855	1'189	0.477	0.562726397	1
ENSBTAG00000016505	*TMC7*	43	27	34	37	31	-0.256	0.916700934	1
ENSBTAG00000016506	*ST3GAL-IV*	1'289	1'096	1'191	1'116	1'266	0.181	0.824058767	1
ENSBTAG00000016507	*SERHL2*	137	159	151	119	184	0.630	0.582322827	1
ENSBTAG00000016508	*CTDSP2*	7'196	9'739	8'739	6'232	11'246	0.852	0.268577156	1
ENSBTAG00000016510	*RRP7A*	403	478	450	349	552	0.661	0.463401475	1
ENSBTAG00000016511	*POLDIP3*	7'732	9'929	9'081	6'696	11'465	0.776	0.312739325	1
ENSBTAG00000016512	*BT.67827*	2'835	1'812	2'274	2'455	2'092	-0.231	0.770554535	1
ENSBTAG00000016513	*ZNF331*	555	382	461	481	441	-0.124	0.893153812	1
ENSBTAG00000016515	*EFNA5*	635	403	508	550	465	-0.241	0.787698847	1
ENSBTAG00000016516	*CYB5R3*	16'437	18'984	18'078	14'235	21'921	0.623	0.414404792	1
ENSBTAG00000016519	*MBOAT1*	897	622	748	777	718	-0.113	0.89574903	1
ENSBTAG00000016520	*TPSB1*	300	111	194	260	128	-1.019	0.342631276	1
ENSBTAG00000016522	*C21H15ORF40*	1'220	726	947	1'057	838	-0.334	0.688174229	1
ENSBTAG00000016523	*DNAH10*	71	27	46	61	31	-0.980	0.591535315	1
ENSBTAG00000016524	*RALY*	8'716	10'804	10'012	7'548	12'475	0.725	0.344800035	1
ENSBTAG00000016525	*BT.83070*	531	211	352	460	244	-0.916	0.331327441	1
ENSBTAG00000016526	*BT.87920*	1'489	993	1'218	1'290	1'147	-0.169	0.83630765	1
ENSBTAG00000016527	*C28H10ORF35*	801	735	771	694	849	0.291	0.730263839	1

Ensembl gene ID	geneName	counts wildtype horn bud	counts polled horn bud	baseMean	baseMean wildtype horn bud	baseMean polled horn bud	log2FoldChange	pval	padj
ENSBTAG00000016528	*NOC2L*	1'984	2'337	2'208	1'718	2'699	0.651	0.409839731	1
ENSBTAG00000016529	*BT.77460*	691	674	688	598	778	0.379	0.656983389	1
ENSBTAG00000016532	*MAPKAPK3*	917	801	860	794	925	0.220	0.792468494	1
ENSBTAG00000016533	*FOXP1*	1'264	913	1'074	1'095	1'054	-0.054	0.949116346	1
ENSBTAG00000016534	*BCL11A*	834	791	818	722	913	0.339	0.686447146	1
ENSBTAG00000016541	*BT.30982*	171	103	134	148	119	-0.316	0.794859618	1
ENSBTAG00000016542	*LAMB3*	1'787	3'135	2'584	1'548	3'620	1.226	0.122414698	1
ENSBTAG00000016544	*SLC47A1*	190	112	147	165	129	-0.347	0.767431298	1
ENSBTAG00000016546	*PARP12*	1'027	924	978	889	1'067	0.263	0.750591008	1
ENSBTAG00000016547	*BT.20927*	937	695	807	811	803	-0.016	0.987214808	1
ENSBTAG00000016548	*TEX12*	111	19	59	96	22	-2.131	0.200635808	1
ENSBTAG00000016549	*LRRC16A*	1'129	567	816	978	655	-0.579	0.491966241	1
ENSBTAG00000016550	*BT.25557*	12	4	8	10	5	-1.170	0.853405557	1
ENSBTAG00000016551	*protein_coding*	264	142	196	229	164	-0.480	0.654725444	1
ENSBTAG00000016552	*NME3*	1'175	1'012	1'093	1'018	1'169	0.200	0.807524913	1
ENSBTAG00000016553	*EME2*	426	471	456	369	544	0.560	0.533684372	1
ENSBTAG00000016554	*C3ORF52*	106	127	119	92	147	0.676	0.583917271	1
ENSBTAG00000016555	*BT.101877*	301	276	290	261	319	0.290	0.767002884	1
ENSBTAG00000016556	*GCET2*	2	1	1	2	1	-0.585	1	1
ENSBTAG00000016557	*BT.28705*	2'346	2'057	2'203	2'032	2'375	0.225	0.774716355	1
ENSBTAG00000016558	*SPSB3*	2'663	2'493	2'592	2'306	2'879	0.320	0.682979651	1
ENSBTAG00000016559	*NUDT22*	787	806	806	682	931	0.449	0.592670552	1
ENSBTAG00000016560	*DNAJC4*	484	402	442	419	464	0.147	0.871893247	1
ENSBTAG00000016561	*NUBP2*	909	812	862	787	938	0.252	0.762657242	1
ENSBTAG00000016562	*SEC63*	8'100	5'601	6'741	7'015	6'467	-0.117	0.879489883	1
ENSBTAG00000016563	*GOLGA4*	5'093	1'712	3'194	4'411	1'977	-1.158	0.141763762	1
ENSBTAG00000016564	*NPRL3*	478	547	523	414	632	0.610	0.489920697	1
ENSBTAG00000016566	*ITGA9*	623	981	836	540	1'133	1.070	0.204637415	1
ENSBTAG00000016567	*HBQ1*	40	43	42	35	50	0.519	0.789098641	1
ENSBTAG00000016568	*BT.39454*	13'778	5'892	9'368	11'932	6'803	-0.810	0.292255548	1
ENSBTAG00000016571	*ITFG3*	2'239	2'626	2'486	1'939	3'032	0.645	0.412308332	1
ENSBTAG00000016572	*USP37*	203	162	181	176	187	0.090	0.938615097	1
ENSBTAG00000016573	*LHCGR*	6	0	3	5	0		0.81115604	1
ENSBTAG00000016574	*BT.26392*	275	49	147	238	57	-2.074	0.081294565	1
ENSBTAG00000016575	*BT.51496*	102	51	74	88	59	-0.585	0.695678411	1
ENSBTAG00000016577	*AXIN1*	2'057	3'037	2'644	1'781	3'507	0.977	0.215633851	1
ENSBTAG00000016578	*HNRNPR*	10'119	9'515	9'875	8'763	10'987	0.326	0.669238953	1
ENSBTAG00000016580	*TEF*	394	958	724	341	1'106	1.697	0.050628114	1
ENSBTAG00000016581	*MRPL28*	1'493	1'651	1'600	1'293	1'906	0.560	0.484246947	1
ENSBTAG00000016582	*GTF3C6*	465	461	468	403	532	0.403	0.653432425	1
ENSBTAG00000016584	*BT.47903*	453	459	461	392	530	0.434	0.628968589	1
ENSBTAG00000016588	*TMEM8*	442	448	450	383	517	0.434	0.629845921	1
ENSBTAG00000016589	*PRPF40B*	5'151	4'005	4'543	4'461	4'625	0.052	0.945957192	1
ENSBTAG00000016590	*BT.74407*	4'108	4'121	4'158	3'558	4'759	0.420	0.587738849	1
ENSBTAG00000016591	*RAB11FIP3*	1'629	2'367	2'072	1'411	2'733	0.954	0.230530331	1
ENSBTAG00000016592	*SOLH*	826	1'287	1'101	715	1'486	1.055	0.201048429	1
ENSBTAG00000016593	*FMNL3*	5'659	6'526	6'218	4'901	7'536	0.621	0.420389026	1
ENSBTAG00000016594	*PIGQ*	1'482	2'066	1'835	1'283	2'386	0.894	0.263053381	1
ENSBTAG00000016595	*CYSTM1*	796	695	746	689	803	0.219	0.795883626	1
ENSBTAG00000016596	*PFDN1*	2'956	2'007	2'439	2'560	2'317	-0.144	0.856062126	1
ENSBTAG00000016598	*DYNC1H1*	20'075	42'287	33'107	17'385	48'829	1.490	0.054368163	1
ENSBTAG00000016599	*MRPL53*	2'175	1'625	1'880	1'884	1'876	-0.006	0.995913442	1
ENSBTAG00000016600	*CCDC142*	473	427	451	410	493	0.267	0.767182854	1
ENSBTAG00000016602	*BT.106435*	6	0	3	5	0		0.81115604	1
ENSBTAG00000016603	*MGC152337*	162	118	138	140	136	-0.042	0.979153596	1
ENSBTAG00000016606	*BT.52040*	974	589	762	844	680	-0.311	0.714509178	1
ENSBTAG00000016607	*TLX2*	0	4	2	0	5	Inf	0.817359038	1
ENSBTAG00000016609	*DQX1*	176	106	137	152	122	-0.316	0.792796841	1
ENSBTAG00000016612	*NEK9*	2'612	1'867	2'209	2'262	2'156	-0.069	0.931109437	1
ENSBTAG00000016613	*KCNH6*	12	2	6	10	2	-2.170	0.758651977	1
ENSBTAG00000016615	*ABHD6*	374	262	313	324	303	-0.098	0.922199361	1
ENSBTAG00000016618	*DSTYK*	1'881	1'181	1'496	1'629	1'364	-0.256	0.75053502	1
ENSBTAG00000016619	*MIS18A*	201	195	200	174	225	0.371	0.727401024	1
ENSBTAG00000016620	*SIGLEC15*	14	1	7	12	1	-3.392	0.634690491	1
ENSBTAG00000016621	*C17ORF85*	1'868	1'566	1'713	1'618	1'808	0.161	0.84017215	1
ENSBTAG00000016622	*BT.66908*	765	686	727	663	792	0.258	0.761514274	1
ENSBTAG00000016625	*BT.21222*	2'348	1'418	1'835	2'033	1'637	-0.313	0.695117041	1
ENSBTAG00000016627	*WASF1*	773	337	529	669	389	-0.783	0.376378791	1
ENSBTAG00000016629	*ADCY9*	238	516	401	206	596	1.531	0.100427602	1
ENSBTAG00000016630	*MEX3B*	639	401	508	553	463	-0.257	0.773531551	1
ENSBTAG00000016633	*NPY2R*	65	5	31	56	6	-3.285	0.171296947	1
ENSBTAG00000016635	*BT.49137*	5'134	2'965	3'935	4'446	3'424	-0.377	0.627608439	1

Ensembl gene ID	geneName	counts wildtype horn bud	counts polled horn bud	baseMean	baseMean wildtype horn bud	baseMean polled horn bud	log2FoldChange	pval	padj
ENSBTAG00000016637	*WBP4*	2'428	1'123	1'700	2'103	1'297	-0.697	0.38397217	1
ENSBTAG00000016640	*HEATR3*	1'909	1'014	1'412	1'653	1'171	-0.498	0.53790784	1
ENSBTAG00000016643	*CYB561D1*	131	81	103	113	94	-0.279	0.835820114	1
ENSBTAG00000016644	*AQPEP*	2'291	2'046	2'173	1'984	2'363	0.252	0.749245966	1
ENSBTAG00000016645	*GABRA4*	47	0	20	41	0		0.078848742	1
ENSBTAG00000016646	*KCNA1*	19	7	12	16	8	-1.026	0.806704154	1
ENSBTAG00000016648	*BSG*	12'533	12'378	12'573	10'854	14'293	0.397	0.602667257	1
ENSBTAG00000016649	*CCND2*	448	583	531	388	673	0.795	0.367739226	1
ENSBTAG00000016650	*TIGAR*	57	33	44	49	38	-0.373	0.850623995	1
ENSBTAG00000016651	*ISL2*	19	4	11	16	5	-1.833	0.688634464	1
ENSBTAG00000016656	*BT.69491*	987	513	724	855	592	-0.529	0.534609174	1
ENSBTAG00000016658	*ABHD4*	1'347	2'019	1'749	1'167	2'331	0.999	0.213085704	1
ENSBTAG00000016661	*USP18*	135	92	112	117	106	-0.138	0.920357453	1
ENSBTAG00000016662	*CPS1*	1	0	0	1	0		1	1
ENSBTAG00000016663	*PIGN*	2'511	1'103	1'724	2'175	1'274	-0.772	0.335333888	1
ENSBTAG00000016664	*SLC2A4RG*	1'722	2'082	1'948	1'491	2'404	0.689	0.385947082	1
ENSBTAG00000016666	*KIAA1468*	3'445	1'466	2'338	2'983	1'693	-0.818	0.301549383	1
ENSBTAG00000016667	*ZBTB46*	134	82	105	116	95	-0.293	0.82543121	1
ENSBTAG00000016675	*ANAPC7*	1'434	1'084	1'247	1'242	1'252	0.011	0.989274725	1
ENSBTAG00000016676	*PTER*	613	364	476	531	420	-0.337	0.708023127	1
ENSBTAG00000016677	*C1QL3*	33	6	18	29	7	-2.044	0.51823266	1
ENSBTAG00000016679	*ETFDH*	892	681	779	772	786	0.026	0.976707863	1
ENSBTAG00000016680	*PPID*	3'533	2'375	2'901	3'060	2'742	-0.158	0.840770283	1
ENSBTAG00000016683	*BIKBA*	1'524	1'276	1'397	1'320	1'473	0.159	0.843775471	1
ENSBTAG00000016684	*ZNF202*	654	897	801	566	1'036	0.871	0.302207444	1
ENSBTAG00000016685	*FMO4*	372	238	298	322	275	-0.229	0.815806243	1
ENSBTAG00000016687	*ZNF646*	294	611	480	255	706	1.470	0.104655271	1
ENSBTAG00000016688	*processed_pseudogene*	2'898	2'559	2'732	2'510	2'955	0.236	0.763063695	1
ENSBTAG00000016691	*LACC1*	735	367	530	637	424	-0.587	0.506592816	1
ENSBTAG00000016694	*KIAA0319L*	965	852	910	836	984	0.235	0.777099683	1
ENSBTAG00000016695	*KCNG4*	2	12	8	2	14	3.000	0.606634849	1
ENSBTAG00000016696	*NCDN*	1'511	1'287	1'397	1'309	1'486	0.184	0.819779815	1
ENSBTAG00000016698	*SLAMF8*	31	4	16	27	5	-2.539	0.465876696	1
ENSBTAG00000016703	*CDC73*	1'538	1'625	1'604	1'332	1'876	0.494	0.536668785	1
ENSBTAG00000016704	*SLC37A2*	797	170	443	690	196	-1.814	0.050303824	1
ENSBTAG00000016705	*BCAS2*	297	191	239	257	221	-0.222	0.830711752	1
ENSBTAG00000016707	*FKBP9*	21'643	15'289	18'199	18'743	17'654	-0.086	0.91039869	1
ENSBTAG00000016708	*BT.44506*	19'143	13'113	15'860	16'578	15'142	-0.131	0.864422996	1
ENSBTAG00000016709	*NT5C3*	595	340	454	515	393	-0.392	0.664554814	1
ENSBTAG00000016710	*PTRH2*	671	465	559	581	537	-0.114	0.898818965	1
ENSBTAG00000016711	*PPIF*	274	291	287	237	336	0.502	0.607198341	1
ENSBTAG00000016712	*CLCA3*	56	26	39	48	30	-0.692	0.730588934	1
ENSBTAG00000016713	*TMEM30B*	61	25	41	53	29	-0.872	0.654842133	1
ENSBTAG00000016714	*RGS12*	957	1'512	1'287	829	1'746	1.075	0.188180092	1
ENSBTAG00000016716	*BT.24869*	539	402	465	467	464	-0.008	0.996391739	1
ENSBTAG00000016717	*CEP68*	359	279	317	311	322	0.051	0.959989681	1
ENSBTAG00000016720	*RAB1A*	12'504	6'670	9'265	10'829	7'702	-0.492	0.52174589	1
ENSBTAG00000016721	*ZNF791*	295	79	173	255	91	-1.486	0.183141181	1
ENSBTAG00000016722	*ATHL1*	528	811	697	457	936	1.034	0.228095638	1
ENSBTAG00000016723	*GFI1*	57	17	34	49	20	-1.330	0.530152091	1
ENSBTAG00000016724	*NPEPL1*	550	475	512	476	548	0.204	0.818804406	1
ENSBTAG00000016725	*KIAA1143*	1'439	852	1'115	1'246	984	-0.341	0.677819507	1
ENSBTAG00000016726	*BT.41914*	841	637	732	728	736	0.014	0.987753501	1
ENSBTAG00000016728	*PLEKHG5*	3'157	4'969	4'236	2'734	5'738	1.069	0.170893296	1
ENSBTAG00000016729	*KCNT2*	176	42	100	152	48	-1.652	0.214134168	1
ENSBTAG00000016730	*BT.76114*	1'363	1'344	1'366	1'180	1'552	0.395	0.624633565	1
ENSBTAG00000016731	*TMEM42*	900	529	695	779	611	-0.352	0.681483709	1
ENSBTAG00000016732	*DNAI2*	20	28	25	17	32	0.900	0.723545146	1
ENSBTAG00000016733	*NOL9*	1'189	1'352	1'295	1'030	1'561	0.600	0.458990587	1
ENSBTAG00000016734	*TAS1R1*	1	1	1	1	1	0.415	1	1
ENSBTAG00000016735	*ZBTB48*	899	1'182	1'072	779	1'365	0.810	0.325255052	1
ENSBTAG00000016736	*HIGD1B*	63	34	47	55	39	-0.475	0.799561763	1
ENSBTAG00000016737	*CDK8*	551	320	423	477	370	-0.369	0.686826741	1
ENSBTAG00000016738	*DTX1*	1'325	453	835	1'147	523	-1.133	0.180202424	1
ENSBTAG00000016739	*RASAL1*	710	196	421	615	226	-1.442	0.118922888	1
ENSBTAG00000016740	*ACLY*	9'607	6'285	7'789	8'320	7'257	-0.197	0.797770897	1
ENSBTAG00000016741	*TOX2*	69	25	44	60	29	-1.050	0.573552686	1
ENSBTAG00000016742	*WFDC3*	191	161	176	165	186	0.169	0.88136695	1
ENSBTAG00000016743	*DNTTIP1*	1'214	1'228	1'235	1'051	1'418	0.432	0.595139572	1
ENSBTAG00000016744	*BT.69966*	14'981	12'046	13'442	12'974	13'910	0.100	0.894604479	1
ENSBTAG00000016746	*UBE2C*	1'303	1'557	1'463	1'128	1'798	0.672	0.40422972	1
ENSBTAG00000016747	*NAT10*	2'534	2'144	2'335	2'195	2'476	0.174	0.824756776	1

Ensembl gene ID	geneName	counts wildtype horn bud	counts polled horn bud	baseMean	baseMean wildtype horn bud	baseMean polled horn bud	log2FoldChange	pval	padj
ENSBTAG00000016748	*protein_coding*	0	2	1	0	2	Inf	0.939077559	1
ENSBTAG00000016750	*PTBP3*	371	270	317	321	312	-0.043	0.968183401	1
ENSBTAG00000016751	*MYO6*	1'159	401	733	1'004	463	-1.116	0.192269181	1
ENSBTAG00000016752	*ASB10*	2	5	4	2	6	1.737	0.883335552	1
ENSBTAG00000016754	*KLHL38*	13	23	19	11	27	1.238	0.677916987	1
ENSBTAG00000016757	*ZKSCAN1*	246	474	380	213	547	1.361	0.14601761	1
ENSBTAG00000016758	*BT.30133*	2'308	1'477	1'852	1'999	1'705	-0.229	0.774251272	1
ENSBTAG00000016760	*BT.46948*	1'420	1'145	1'276	1'230	1'322	0.104	0.897565972	1
ENSBTAG00000016761	*NOX1*	40	10	23	35	12	-1.585	0.551930545	1
ENSBTAG00000016762	*SLC25A12*	2'630	1'409	1'952	2'278	1'627	-0.485	0.541464002	1
ENSBTAG00000016763	*BCDIN3*	2'251	3'087	2'757	1'949	3'565	0.871	0.268398104	1
ENSBTAG00000016764	*UBA1*	11'481	13'564	12'803	9'943	15'662	0.656	0.391504219	1
ENSBTAG00000016765	*BT.56205*	28	6	16	24	7	-1.807	0.598818952	1
ENSBTAG00000016766	*TMEM176B*	16	18	17	14	21	0.585	0.86255723	1
ENSBTAG00000016768	*SCN3B*	81	10	41	70	12	-2.603	0.19613437	1
ENSBTAG00000016769	*PCTK1*	5'313	5'372	5'402	4'601	6'203	0.431	0.575836106	1
ENSBTAG00000016770	*GAPT*	90	32	57	78	37	-1.077	0.513860637	1
ENSBTAG00000016771	*PLK2*	6'379	2'389	4'141	5'524	2'759	-1.002	0.199528124	1
ENSBTAG00000016772	*USP11*	6'700	7'734	7'366	5'802	8'930	0.622	0.418320179	1
ENSBTAG00000016773	*NXNL1*	1	3	2	1	3	2.000	0.933774563	1
ENSBTAG00000016774	*MRGPRD*	4	0	2	3	0		0.89545886	1
ENSBTAG00000016775	*SLC27A1*	1'930	3'824	3'044	1'671	4'416	1.402	0.077150742	1
ENSBTAG00000016776	*BAHCC1*	934	2'035	1'579	809	2'350	1.539	0.059379983	1
ENSBTAG00000016777	*TEX101*	2	0	1	2	0		0.974934741	1
ENSBTAG00000016779	*CLIP1*	5'151	2'389	3'610	4'461	2'759	-0.693	0.374078243	1
ENSBTAG00000016782	*SLC39A10*	1'869	1'203	1'504	1'619	1'389	-0.221	0.784621856	1
ENSBTAG00000016783	*BT.60804*	1'562	2'256	1'979	1'353	2'605	0.945	0.235621293	1
ENSBTAG00000016784	*BT.36236*	44	3	21	38	3	-3.459	0.257611707	1
ENSBTAG00000016785	*BT.104139*	1	0	0	1	0		1	1
ENSBTAG00000016791	*PEX7*	669	362	499	579	418	-0.471	0.597277562	1
ENSBTAG00000016793	*SLC35D3*	12	10	11	10	12	0.152	0.995696634	1
ENSBTAG00000016794	*BT.52969*	33	14	22	29	16	-0.822	0.766955725	1
ENSBTAG00000016795	*RUFY3*	889	251	530	770	290	-1.409	0.114938773	1
ENSBTAG00000016797	*CSNK1G2*	2'475	3'499	3'092	2'143	4'040	0.915	0.243788126	1
ENSBTAG00000016799	*ERN1*	66	90	81	57	104	0.862	0.544036654	1
ENSBTAG00000016800	*SEMA6D*	4'781	1'550	2'965	4'140	1'790	-1.210	0.125831229	1
ENSBTAG00000016801	*RXRG*	295	371	342	255	428	0.746	0.429578423	1
ENSBTAG00000016804	*LYST*	1'645	930	1'249	1'425	1'074	-0.408	0.616523866	1
ENSBTAG00000016806	*PAFAH1B1*	3'371	2'940	3'157	2'919	3'395	0.218	0.779597808	1
ENSBTAG00000016810	*PYCRL*	2'058	2'628	2'408	1'782	3'035	0.768	0.330481778	1
ENSBTAG00000016813	*SH3D19*	3'637	3'169	3'404	3'150	3'659	0.216	0.780494083	1
ENSBTAG00000016814	*CHAT*	1	0	0	1	0		1	1
ENSBTAG00000016817	*BT.59135*	3'324	2'047	2'621	2'879	2'364	-0.284	0.717636667	1
ENSBTAG00000016818	*ZCCHC17*	2'088	1'223	1'610	1'808	1'412	-0.357	0.656673566	1
ENSBTAG00000016819	*FABP3*	772	382	555	669	441	-0.600	0.494634269	1
ENSBTAG00000016820	*BDA20*	4	2	3	3	2	-0.585	0.996239345	1
ENSBTAG00000016821	*SNX22*	157	55	100	136	64	-1.098	0.405293379	1
ENSBTAG00000016822	*PPIB*	40'636	39'216	40'237	35'192	45'283	0.364	0.631662454	1
ENSBTAG00000016823	*CSNK1G1*	234	268	256	203	309	0.611	0.541189706	1
ENSBTAG00000016824	*C6ORF11*	1'413	1'785	1'642	1'224	2'061	0.752	0.348032764	1
ENSBTAG00000016826	*GPR176*	647	270	436	560	312	-0.846	0.352651377	1
ENSBTAG00000016827	*RGL2*	7'646	7'305	7'528	6'622	8'435	0.349	0.64849527	1
ENSBTAG00000016828	*TAPBP*	4'489	4'470	4'525	3'888	5'162	0.409	0.596608571	1
ENSBTAG00000016829	*ZBTB22*	260	421	356	225	486	1.110	0.238589361	1
ENSBTAG00000016830	*DAXX*	2'621	2'550	2'607	2'270	2'944	0.375	0.63178779	1
ENSBTAG00000016833	*CLPS*	4	0	2	3	0		0.89545886	1
ENSBTAG00000016834	*LHFPL5*	23	11	16	20	13	-0.649	0.855398161	1
ENSBTAG00000016836	*PDK1*	1'647	1'525	1'594	1'426	1'761	0.304	0.703781541	1
ENSBTAG00000016838	*SRPK1*	2'162	1'627	1'876	1'872	1'879	0.005	0.995183184	1
ENSBTAG00000016839	*MTO1*	439	260	340	380	300	-0.341	0.720343446	1
ENSBTAG00000016841	*ATP10B*	122	35	73	106	40	-1.386	0.352974716	1
ENSBTAG00000016844	*C6ORF103*	146	75	107	126	87	-0.546	0.673397599	1
ENSBTAG00000016845	*GALNT7*	785	528	645	680	610	-0.157	0.857128592	1
ENSBTAG00000016846	*YWHAB*	17'077	15'652	16'431	14'789	18'073	0.289	0.703634858	1
ENSBTAG00000016848	*GTF2E1*	550	405	472	476	468	-0.026	0.979812537	1
ENSBTAG00000016849	*PABPC1L*	239	168	200	207	194	-0.094	0.935150675	1
ENSBTAG00000016851	*BT.41950*	1'498	858	1'144	1'297	991	-0.389	0.634936162	1
ENSBTAG00000016852	*SNIP1*	540	507	527	468	585	0.324	0.713406941	1
ENSBTAG00000016854	*AK7*	200	50	115	173	58	-1.585	0.210378368	1
ENSBTAG00000016855	*BUD13*	1'469	1'163	1'308	1'272	1'343	0.078	0.923361073	1
ENSBTAG00000016858	*SAR1A*	3'146	3'679	3'486	2'725	4'248	0.641	0.410734501	1
ENSBTAG00000016867	*HSD17B3*	9	1	4	8	1	-2.755	0.785977687	1

Ensembl gene ID	geneName	counts wildtype horn bud	counts polled horn bud	baseMean	baseMean wildtype horn bud	baseMean polled horn bud	log2FoldChange	pval	padj
ENSBTAG00000016869	*POLD3*	1'447	1'380	1'423	1'253	1'593	0.347	0.666640966	1
ENSBTAG00000016873	*C13H20orf111*	2'025	1'439	1'708	1'754	1'662	-0.078	0.923645044	1
ENSBTAG00000016874	*DNAJB1*	4'559	3'826	4'183	3'948	4'418	0.162	0.833600734	1
ENSBTAG00000016876	*protein_coding*	167	320	257	145	370	1.353	0.179531345	1
ENSBTAG00000016877	*PLA2G2C*	2	2	2	2	2	0.415	1	1
ENSBTAG00000016878	*WDR59*	1'504	875	1'156	1'303	1'010	-0.366	0.654480219	1
ENSBTAG00000016879	*KIAA1147*	140	219	187	121	253	1.061	0.326710509	1
ENSBTAG00000016880	*BT.76071*	2	0	1	2	0		0.974934741	1
ENSBTAG00000016881	*BT.27994*	19'911	14'537	17'015	17'243	16'786	-0.039	0.960120387	1
ENSBTAG00000016882	*VPS26A*	7'291	5'005	6'047	6'314	5'779	-0.128	0.868917741	1
ENSBTAG00000016883	*protein_coding*	481	318	392	417	367	-0.182	0.846213624	1
ENSBTAG00000016885	*LRRTM3*	64	50	57	55	58	0.059	0.98268546	1
ENSBTAG00000016887	*TAF11*	1'232	814	1'003	1'067	940	-0.183	0.825993137	1
ENSBTAG00000016890	*ANKS1A*	1'436	1'370	1'413	1'244	1'582	0.347	0.666310842	1
ENSBTAG00000016891	*BT.50314*	37	17	26	32	20	-0.707	0.78236821	1
ENSBTAG00000016894	*CYFIP1*	6'478	5'995	6'266	5'610	6'922	0.303	0.692780075	1
ENSBTAG00000016895	*NIPA2*	2'593	1'706	2'108	2'246	1'970	-0.189	0.812001472	1
ENSBTAG00000016896	*BT.105012*	3'371	1'415	2'277	2'919	1'634	-0.837	0.290612789	1
ENSBTAG00000016899	*MED8*	919	692	797	796	799	0.006	0.995585183	1
ENSBTAG00000016900	*C20H5orf44*	1'238	838	1'020	1'072	968	-0.148	0.859019125	1
ENSBTAG00000016902	*SGTB*	56	39	47	48	45	-0.107	0.966395922	1
ENSBTAG00000016903	*BT.40554*	968	423	663	838	488	-0.779	0.365406624	1
ENSBTAG00000016904	*Mrz 09*	672	849	781	582	980	0.752	0.373137677	1
ENSBTAG00000016906	*BT.61430*	52	53	53	45	61	0.443	0.799297411	1
ENSBTAG00000016908	*METTL1*	710	907	831	615	1'047	0.768	0.360396282	1
ENSBTAG00000016910	*METTL21B*	808	419	592	700	484	-0.532	0.541275591	1
ENSBTAG00000016911	*ARHGAP23*	1'734	2'200	2'021	1'502	2'540	0.758	0.33954445	1
ENSBTAG00000016912	*TSFM*	1'006	663	818	871	766	-0.187	0.82578075	1
ENSBTAG00000016913	*AVIL*	451	119	264	391	137	-1.507	0.135104158	1
ENSBTAG00000016915	*FLT1*	642	387	501	556	447	-0.315	0.724198414	1
ENSBTAG00000016918	*MYOF*	6'375	3'519	4'792	5'521	4'063	-0.442	0.567823919	1
ENSBTAG00000016924	*BT.31580*	629	354	477	545	409	-0.414	0.644490078	1
ENSBTAG00000016926	*C13H20ORF85*	1	0	0	1	0		1	1
ENSBTAG00000016928	*PDZD7*	55	44	49	48	51	0.093	0.969328009	1
ENSBTAG00000016929	*BT.87607*	3'533	3'546	3'577	3'060	4'095	0.420	0.588362136	1
ENSBTAG00000016931	*E2F7*	117	55	82	101	64	-0.674	0.635587312	1
ENSBTAG00000016932	*SENP7*	2'160	1'407	1'748	1'871	1'625	-0.203	0.799590009	1
ENSBTAG00000016933	*IL27RA*	2'512	2'580	2'577	2'175	2'979	0.454	0.563017925	1
ENSBTAG00000016934	*C2ORF71*	0	6	3	0	7	Inf	0.700309343	1
ENSBTAG00000016936	*BT.61523*	155	153	155	134	177	0.396	0.728577086	1
ENSBTAG00000016937	*C19ORF67*	26	12	18	23	14	-0.700	0.829238712	1
ENSBTAG00000016939	*C11H2orf42*	1'015	569	768	879	657	-0.420	0.620092893	1
ENSBTAG00000016943	*NEK7*	609	562	588	527	649	0.299	0.730936968	1
ENSBTAG00000016944	*BT.56667*	109	35	67	94	40	-1.224	0.426660841	1
ENSBTAG00000016948	*BT.41304*	5'711	7'439	6'768	4'946	8'590	0.796	0.301842527	1
ENSBTAG00000016950	*B3GAT3*	2'201	1'867	2'031	1'906	2'156	0.178	0.822128145	1
ENSBTAG00000016951	*WDFY1*	1'055	1'024	1'048	914	1'182	0.372	0.650629575	1
ENSBTAG00000016952	*PSMD5*	3'116	1'927	2'462	2'699	2'225	-0.278	0.724011067	1
ENSBTAG00000016954	*TMEM39B*	1'139	1'007	1'075	986	1'163	0.237	0.772260004	1
ENSBTAG00000016956	*BT.42031*	14'695	17'756	16'615	12'726	20'503	0.688	0.367938835	1
ENSBTAG00000016957	*BT.91498*	649	581	616	562	671	0.255	0.767982171	1
ENSBTAG00000016958	*PHF19*	1'181	1'286	1'254	1'023	1'485	0.538	0.507656198	1
ENSBTAG00000016959	*LAPTM4B*	2'275	2'761	2'579	1'970	3'188	0.694	0.377209547	1
ENSBTAG00000016961	*RAP2B*	1'954	1'522	1'725	1'692	1'757	0.055	0.945448718	1
ENSBTAG00000016963	*ADAMTS6*	807	243	490	699	281	-1.317	0.144271104	1
ENSBTAG00000016967	*RARS2*	1'100	747	908	953	863	-0.143	0.864895963	1
ENSBTAG00000016968	*CHST4*	71	2	32	61	2	-4.735	0.069695007	1
ENSBTAG00000016969	*BT.24267*	1'667	977	1'286	1'444	1'128	-0.356	0.66164062	1
ENSBTAG00000016972	*RAB11FIP4*	182	230	212	158	266	0.753	0.471374114	1
ENSBTAG00000016973	*BT.46193*	328	506	434	284	584	1.040	0.25344313	1
ENSBTAG00000016975	*H2AFV*	5'484	4'323	4'871	4'749	4'992	0.072	0.925388453	1
ENSBTAG00000016977	*FUNDC2*	1'602	1'420	1'514	1'387	1'640	0.241	0.763662382	1
ENSBTAG00000016979	*PCYT1A*	487	660	592	422	762	0.854	0.327284567	1
ENSBTAG00000016980	*PPIL4*	2'589	1'682	2'092	2'242	1'942	-0.207	0.794216404	1
ENSBTAG00000016981	*BT.88405*	994	475	705	861	548	-0.650	0.446708058	1
ENSBTAG00000016982	*MCOLN3*	141	29	78	122	33	-1.867	0.204914808	1
ENSBTAG00000016984	*PTPN9*	3'283	2'118	2'644	2'843	2'446	-0.217	0.782482189	1
ENSBTAG00000016987	*C2ORF44*	229	249	243	198	288	0.536	0.596379789	1
ENSBTAG00000016988	*PIN1*	4'285	5'450	5'002	3'711	6'293	0.762	0.325164071	1
ENSBTAG00000016990	*BT.43047*	1'292	1'180	1'241	1'119	1'363	0.284	0.726168946	1
ENSBTAG00000016991	*BT.80870*	997	949	980	863	1'096	0.344	0.677112427	1
ENSBTAG00000016995	*CECR5*	708	625	667	613	722	0.235	0.783999551	1

Ensembl gene ID	geneName	counts wildtype horn bud	counts polled horn bud	baseMean	baseMean wildtype horn bud	baseMean polled horn bud	log2FoldChange	pval	padj
ENSBTAG00000016997	*protein_coding*	157	113	133	136	130	-0.059	0.967912114	1
ENSBTAG00000016998	*ARL11*	10	12	11	9	14	0.678	0.884992558	1
ENSBTAG00000016999	*RYK*	6'120	3'353	4'586	5'300	3'872	-0.453	0.558730282	1
ENSBTAG00000017001	*RBM28*	860	894	889	745	1'032	0.471	0.571727482	1
ENSBTAG00000017002	*C20ORF18*	1'760	2'209	2'037	1'524	2'551	0.743	0.349294591	1
ENSBTAG00000017005	*LCN8*	1	0	0	1	0		1	1
ENSBTAG00000017007	*TRIB3*	82	76	79	71	88	0.305	0.835874272	1
ENSBTAG00000017009	*CMC2*	989	722	845	856	834	-0.039	0.965120907	1
ENSBTAG00000017010	*C1H21ORF70*	454	361	405	393	417	0.084	0.928527614	1
ENSBTAG00000017011	*CCDC125*	346	149	236	300	172	-0.800	0.433633082	1
ENSBTAG00000017012	*protein_coding*	10	0	4	9	0		0.653360153	1
ENSBTAG00000017015	*TEKT5*	1	0	0	1	0		1	1
ENSBTAG00000017016	*H3F3C*	252	174	210	218	201	-0.119	0.914392565	1
ENSBTAG00000017017	*CTPS2*	1'942	1'652	1'795	1'682	1'908	0.182	0.819111787	1
ENSBTAG00000017019	*PRKDC*	1'150	1'084	1'124	996	1'252	0.330	0.686527344	1
ENSBTAG00000017020	*S100G*	5	2	3	4	2	-0.907	0.965043111	1
ENSBTAG00000017021	*BT.62006*	804	770	793	696	889	0.353	0.675094137	1
ENSBTAG00000017023	*INPP5J*	45	66	58	39	76	0.968	0.554239054	1
ENSBTAG00000017024	*PPARGC1A*	270	187	225	234	216	-0.115	0.916026527	1
ENSBTAG00000017026	*DEPDC1B*	89	74	81	77	85	0.149	0.923673732	1
ENSBTAG00000017027	*MMACHC*	503	310	397	436	358	-0.283	0.760181485	1
ENSBTAG00000017028	*USO1*	8'148	4'018	5'848	7'056	4'640	-0.605	0.433434745	1
ENSBTAG00000017029	*CCDC163P*	36	36	36	31	42	0.415	0.846633381	1
ENSBTAG00000017031	*C1ORF173*	32	2	15	28	2	-3.585	0.345807076	1
ENSBTAG00000017032	*FREM2*	160	254	216	139	293	1.082	0.29989859	1
ENSBTAG00000017033	*LSR*	380	426	410	329	492	0.580	0.52597252	1
ENSBTAG00000017035	*USF2*	2'689	3'247	3'039	2'329	3'749	0.687	0.379811237	1
ENSBTAG00000017037	*BT.104342*	4'914	4'724	4'855	4'256	5'455	0.358	0.642347497	1
ENSBTAG00000017038	*BT.65365*	1'125	1'337	1'259	974	1'544	0.664	0.413880164	1
ENSBTAG00000017039	*ARHGEF33*	16	5	10	14	6	-1.263	0.797689913	1
ENSBTAG00000017040	*LY6E*	770	1'138	990	667	1'314	0.979	0.238590578	1
ENSBTAG00000017041	*BT.106423*	48	44	46	42	51	0.290	0.881446192	1
ENSBTAG00000017048	*CENPJ*	628	331	463	544	382	-0.509	0.572114466	1
ENSBTAG00000017051	*ACP6*	722	444	569	625	513	-0.286	0.744525659	1
ENSBTAG00000017053	*ABCB10*	530	326	418	459	376	-0.286	0.755785726	1
ENSBTAG00000017055	*HCN3*	28	1	13	24	1	-4.392	0.334236041	1
ENSBTAG00000017056	*PKLR*	48	27	36	42	31	-0.415	0.849214196	1
ENSBTAG00000017060	*ITGB2*	331	262	295	287	303	0.078	0.93886935	1
ENSBTAG00000017061	*SMARCAD1*	2'156	1'257	1'659	1'867	1'451	-0.363	0.650151272	1
ENSBTAG00000017062	*ADM2*	0	1	1	0	1	Inf	0.993540919	1
ENSBTAG00000017063	*EPB41L4B*	383	448	424	332	517	0.641	0.480987766	1
ENSBTAG00000017064	*BT.89463*	4'381	3'513	3'925	3'794	4'056	0.096	0.900533419	1
ENSBTAG00000017067	*WBSCR22*	2'032	1'730	1'879	1'760	1'998	0.183	0.817542535	1
ENSBTAG00000017068	*DDA1*	588	549	572	509	634	0.316	0.717330505	1
ENSBTAG00000017069	*BT.36924*	302	164	225	262	189	-0.466	0.652916604	1
ENSBTAG00000017072	*BT.45809*	3'107	2'996	3'075	2'691	3'459	0.363	0.641792346	1
ENSBTAG00000017073	*HPGDS*	16	6	10	14	7	-1.000	0.835750236	1
ENSBTAG00000017074	*ANO8*	1'977	1'872	1'937	1'712	2'162	0.336	0.671174022	1
ENSBTAG00000017075	*STX1A*	709	465	575	614	537	-0.194	0.826385997	1
ENSBTAG00000017077	*CTSL1*	12'500	6'772	9'322	10'825	7'820	-0.469	0.540786113	1
ENSBTAG00000017078	*FAM114A2*	2'540	1'567	2'005	2'200	1'809	-0.282	0.722856557	1
ENSBTAG00000017079	*MFAP3*	4'177	2'449	3'223	3'617	2'828	-0.355	0.64926164	1
ENSBTAG00000017081	*SMCR7*	245	285	271	212	329	0.633	0.521464322	1
ENSBTAG00000017082	*POR*	2'744	3'051	2'950	2'376	3'523	0.568	0.467516915	1
ENSBTAG00000017083	*TEX15*	54	8	28	47	9	-2.340	0.335309709	1
ENSBTAG00000017085	*AGA*	1'389	745	1'032	1'203	860	-0.484	0.557796671	1
ENSBTAG00000017086	*BT.24356*	1'932	1'623	1'774	1'673	1'874	0.164	0.83696328	1
ENSBTAG00000017087	*BT.103042*	2'194	3'652	3'059	1'900	4'217	1.150	0.144585059	1
ENSBTAG00000017090	*SMCR8*	294	511	422	255	590	1.213	0.186486929	1
ENSBTAG00000017091	*FTSJD2*	2'844	4'733	3'964	2'463	5'465	1.150	0.142130584	1
ENSBTAG00000017094	*SHMT1*	286	349	325	248	403	0.702	0.460928036	1
ENSBTAG00000017095	*DENND4A*	1'593	773	1'136	1'380	893	-0.628	0.443536009	1
ENSBTAG00000017096	*ERICH1*	369	240	298	320	277	-0.206	0.835093552	1
ENSBTAG00000017097	*PTCRA*	9	3	6	8	3	-1.170	0.890813994	1
ENSBTAG00000017102	*pseudogene*	2	23	14	2	27	3.939	0.31720337	1
ENSBTAG00000017103	*BT.78411*	181	175	179	157	202	0.366	0.738332104	1
ENSBTAG00000017104	*MUC1*	102	98	101	88	113	0.357	0.787848476	1
ENSBTAG00000017105	*FSTL4*	59	56	58	51	65	0.340	0.841294218	1
ENSBTAG00000017108	*OTUD3*	340	261	298	294	301	0.034	0.975450688	1
ENSBTAG00000017112	*BT.48688*	90	247	182	78	285	1.872	0.091970277	1
ENSBTAG00000017113	*ANKRD33*	94	20	52	81	23	-1.818	0.296594003	1
ENSBTAG00000017115	*TMEM41A*	634	391	500	549	451	-0.282	0.752379471	1

Ensembl gene ID	geneName	counts wildtype horn bud	counts polled horn bud	baseMean	baseMean wildtype horn bud	baseMean polled horn bud	log2FoldChange	pval	padj
ENSBTAG00000017118	*FAM8A1*	590	336	449	511	388	-0.397	0.661077423	1
ENSBTAG00000017120	*PHB*	3'698	2'976	3'319	3'203	3'436	0.102	0.895706797	1
ENSBTAG00000017121	*ALB*	39	102	76	34	118	1.802	0.222169993	1
ENSBTAG00000017122	*HSPG2*	24'669	36'910	31'992	21'364	42'620	0.996	0.193373703	1
ENSBTAG00000017123	*BT.46423*	1'848	597	1'145	1'600	689	-1.215	0.141333968	1
ENSBTAG00000017124	*GPSM2*	2'714	1'971	2'313	2'350	2'276	-0.046	0.954194635	1
ENSBTAG00000017125	*PABPC4*	8'286	4'496	6'184	7'176	5'192	-0.467	0.544686498	1
ENSBTAG00000017128	*NUP153*	2'644	1'912	2'249	2'290	2'208	-0.053	0.948037232	1
ENSBTAG00000017129	*CLCC1*	3'799	2'570	3'129	3'290	2'968	-0.149	0.849561633	1
ENSBTAG00000017132	*TTC22*	256	270	267	222	312	0.492	0.619775648	1
ENSBTAG00000017133	*GINS4*	227	200	214	197	231	0.232	0.825832245	1
ENSBTAG00000017135	*CTSS*	5'285	2'400	3'674	4'577	2'771	-0.724	0.353431507	1
ENSBTAG00000017137	*CDC37L1*	950	585	749	823	675	-0.284	0.738234909	1
ENSBTAG00000017138	*TMEM71*	25	4	13	22	5	-2.229	0.568026072	1
ENSBTAG00000017139	*STX7*	5'437	5'338	5'436	4'709	6'164	0.389	0.613806959	1
ENSBTAG00000017140	*BT.61032*	571	345	446	495	398	-0.312	0.731552588	1
ENSBTAG00000017141	*CGRRF1*	688	390	523	596	450	-0.404	0.648588585	1
ENSBTAG00000017143	*BT.23128*	12'331	14'786	13'876	10'679	17'073	0.677	0.376106563	1
ENSBTAG00000017146	*GRIK4*	49	37	43	42	43	0.010	1	1
ENSBTAG00000017147	*AK3*	2'223	2'102	2'176	1'925	2'427	0.334	0.671523418	1
ENSBTAG00000017150	*EHF*	239	271	260	207	313	0.596	0.54948671	1
ENSBTAG00000017151	*SH3TC2*	576	366	461	499	423	-0.239	0.792204645	1
ENSBTAG00000017155	*TRIM32*	933	870	906	808	1'005	0.314	0.705450464	1
ENSBTAG00000017157	*CCDC110*	76	21	45	66	24	-1.441	0.436818231	1
ENSBTAG00000017158	*VSNL1*	383	77	210	332	89	-1.899	0.077068236	1
ENSBTAG00000017160	*BT.21021*	656	638	652	568	737	0.375	0.662440533	1
ENSBTAG00000017161	*TM2D1*	964	691	816	835	798	-0.065	0.940151451	1
ENSBTAG00000017162	*STK39*	165	114	137	143	132	-0.118	0.926839683	1
ENSBTAG00000017164	*BT.19425*	392	453	431	339	523	0.624	0.491938221	1
ENSBTAG00000017165	*BT.65258*	7'486	6'354	6'910	6'483	7'337	0.179	0.815562924	1
ENSBTAG00000017167	*LRAT*	71	17	41	61	20	-1.647	0.400022529	1
ENSBTAG00000017169	*protein_coding*	70	198	145	61	229	1.915	0.106112134	1
ENSBTAG00000017170	*protein_coding*	17	21	19	15	24	0.720	0.811878868	1
ENSBTAG00000017174	*SCN11A*	5	4	4	4	5	0.093	1	1
ENSBTAG00000017175	*RNF216*	1'427	1'290	1'363	1'236	1'490	0.269	0.738385649	1
ENSBTAG00000017177	*RBM25*	6'052	2'659	4'156	5'241	3'070	-0.771	0.321572683	1
ENSBTAG00000017179	*USP12*	2'814	1'449	2'055	2'437	1'673	-0.543	0.494063103	1
ENSBTAG00000017180	*SLITRK3*	140	27	76	121	31	-1.959	0.188150019	1
ENSBTAG00000017181	*MACROD1*	649	915	809	562	1'057	0.911	0.280516509	1
ENSBTAG00000017183	*PDLIM3*	953	631	777	825	729	-0.180	0.832920669	1
ENSBTAG00000017184	*MGC155143*	1'902	1'611	1'754	1'647	1'860	0.175	0.825411677	1
ENSBTAG00000017187	*MGC142908*	596	327	447	516	378	-0.451	0.618670341	1
ENSBTAG00000017188	*C20ORF82*	791	540	654	685	624	-0.136	0.876605592	1
ENSBTAG00000017189	*POLE4*	3'885	2'751	3'271	3'365	3'177	-0.083	0.916215636	1
ENSBTAG00000017191	*protein_coding*	1'077	1'395	1'272	933	1'611	0.788	0.33257421	1
ENSBTAG00000017194	*ASPG*	18	84	56	16	97	2.637	0.125233805	1
ENSBTAG00000017195	*FANCC*	840	616	719	727	711	-0.032	0.972088303	1
ENSBTAG00000017196	*PDIA3*	26'693	14'193	19'753	23'117	16'389	-0.496	0.51565331	1
ENSBTAG00000017198	*BT.35525*	22	2	11	19	2	-3.044	0.517475779	1
ENSBTAG00000017200	*RHOJ*	3'031	1'437	2'142	2'625	1'659	-0.662	0.403843171	1
ENSBTAG00000017201	*AP4S1*	271	135	195	235	156	-0.590	0.581715978	1
ENSBTAG00000017204	*protein_coding*	1	0	0	1	0		1	1
ENSBTAG00000017205	*WISP3*	45	13	27	39	15	-1.376	0.570833419	1
ENSBTAG00000017211	*SERINC4*	2'146	1'225	1'636	1'858	1'415	-0.394	0.623188201	1
ENSBTAG00000017212	*C15ORF63*	4'102	2'188	3'039	3'552	2'526	-0.492	0.529774614	1
ENSBTAG00000017213	*MFAP1*	2'362	2'105	2'238	2'046	2'431	0.249	0.751867706	1
ENSBTAG00000017214	*protein_coding*	0	3	2	0	3	Inf	0.878646179	1
ENSBTAG00000017215	*WDR76*	855	726	789	740	838	0.179	0.831890394	1
ENSBTAG00000017216	*FRMD5*	79	17	44	68	20	-1.801	0.339723788	1
ENSBTAG00000017218	*ACER3*	1'204	834	1'003	1'043	963	-0.115	0.891122864	1
ENSBTAG00000017219	*DDX51*	1'051	1'130	1'108	910	1'305	0.520	0.525651976	1
ENSBTAG00000017221	*protein_coding*	868	824	852	752	951	0.340	0.68416671	1
ENSBTAG00000017223	*BT.22540*	24'248	13'155	18'095	20'999	15'190	-0.467	0.540622251	1
ENSBTAG00000017225	*BT.65952*	2'093	1'397	1'713	1'813	1'613	-0.168	0.834101579	1
ENSBTAG00000017228	*TP53I11*	957	283	578	829	327	-1.343	0.128383914	1
ENSBTAG00000017232	*KIF13B*	499	523	518	432	604	0.483	0.584763159	1
ENSBTAG00000017233	*RNF213*	1	0	0	1	0		1	1
ENSBTAG00000017239	*GABBR1*	5'696	4'046	4'802	4'933	4'672	-0.078	0.920002738	1
ENSBTAG00000017240	*DGKQ*	700	829	782	606	957	0.659	0.434779649	1
ENSBTAG00000017241	*pseudogene*	1	0	0	1	0		1	1
ENSBTAG00000017242	*FADS6*	309	396	362	268	457	0.773	0.408257679	1
ENSBTAG00000017243	*GATA3*	706	894	822	611	1'032	0.756	0.368787736	1

Ensembl gene ID	geneName	counts wildtype horn bud	counts polled horn bud	baseMean	baseMean wildtype horn bud	baseMean polled horn bud	log2FoldChange	pval	padj
ENSBTAG00000017244	*PRPF18*	1'800	1'194	1'469	1'559	1'379	-0.177	0.826807897	1
ENSBTAG00000017245	*COPG2*	2'755	2'973	2'909	2'386	3'433	0.525	0.501965181	1
ENSBTAG00000017246	*UBB*	41'996	27'946	34'319	36'370	32'269	-0.173	0.820938664	1
ENSBTAG00000017248	*protein_coding*	318	390	363	275	450	0.709	0.44746887	1
ENSBTAG00000017249	*SBSPON*	193	160	176	167	185	0.145	0.898879881	1
ENSBTAG00000017251	*BT.86001*	2	2	2	2	2	0.415	1	1
ENSBTAG00000017252	*USH1G*	30	11	19	26	13	-1.032	0.731379921	1
ENSBTAG00000017253	*BT.18851*	340	454	409	294	524	0.832	0.363936258	1
ENSBTAG00000017255	*ATAD2B*	544	253	382	471	292	-0.689	0.457805157	1
ENSBTAG00000017256	*CD2*	24	4	13	21	5	-2.170	0.58681328	1
ENSBTAG00000017258	*ACSL3*	2'735	2'053	2'370	2'369	2'371	0.001	0.998696127	1
ENSBTAG00000017260	*PDE3A*	135	105	119	117	121	0.052	0.972731403	1
ENSBTAG00000017263	*MXI1*	1'832	1'231	1'504	1'587	1'421	-0.159	0.844700895	1
ENSBTAG00000017265	*BT.20583*	1'359	938	1'130	1'177	1'083	-0.120	0.885008586	1
ENSBTAG00000017266	*BT.102259*	5'874	2'811	4'166	5'087	3'246	-0.648	0.404288441	1
ENSBTAG00000017267	*COX5A*	2'475	2'312	2'407	2'143	2'670	0.317	0.686666806	1
ENSBTAG00000017268	*PROCA1*	389	262	320	337	303	-0.155	0.874404924	1
ENSBTAG00000017271	*BT.87962*	276	214	243	239	247	0.048	0.965783168	1
ENSBTAG00000017273	*LRRC25*	140	112	125	121	129	0.093	0.94466945	1
ENSBTAG00000017275	*BT.38955*	1'973	1'495	1'717	1'709	1'726	0.015	0.985332694	1
ENSBTAG00000017276	*TXNDC2*	3	0	1	3	0		0.936647693	1
ENSBTAG00000017277	*BT.82861*	119	34	71	103	39	-1.392	0.356096145	1
ENSBTAG00000017278	*BT.87957*	957	615	769	829	710	-0.223	0.793338326	1
ENSBTAG00000017279	*VAPA*	4'627	3'697	4'138	4'007	4'269	0.091	0.905693856	1
ENSBTAG00000017281	*OPLAH*	675	349	494	585	403	-0.537	0.5476155	1
ENSBTAG00000017282	*COL25A1*	311	419	377	269	484	0.845	0.363158663	1
ENSBTAG00000017283	*GTF3C2*	3'224	2'756	2'987	2'792	3'182	0.189	0.808520036	1
ENSBTAG00000017284	*RHBDL1*	2'102	1'017	1'497	1'820	1'174	-0.632	0.432595867	1
ENSBTAG00000017285	*FGF19*	2	14	9	2	16	3.222	0.540921467	1
ENSBTAG00000017287	*BT.26851*	690	330	489	598	381	-0.649	0.467582582	1
ENSBTAG00000017289	*MCF2L*	1'190	972	1'076	1'031	1'122	0.123	0.880908028	1
ENSBTAG00000017291	*BT.53314*	925	1'148	1'063	801	1'326	0.727	0.377195798	1
ENSBTAG00000017294	*AGP*	32	6	17	28	7	-2.000	0.533547462	1
ENSBTAG00000017296	*POLN*	46	19	31	40	22	-0.861	0.704839446	1
ENSBTAG00000017298	*C29H11orf67*	1'430	1'270	1'352	1'238	1'466	0.244	0.762542398	1
ENSBTAG00000017299	*KLC1*	4'486	2'266	3'251	3'885	2'617	-0.570	0.465510634	1
ENSBTAG00000017305	*VPREB*	18	2	9	16	2	-2.755	0.605203757	1
ENSBTAG00000017306	*AKNA*	357	692	554	309	799	1.370	0.122484186	1
ENSBTAG00000017310	*BT.3923*	80	21	47	69	24	-1.515	0.405666834	1
ENSBTAG00000017313	*CECR1*	134	54	89	116	62	-0.896	0.514389908	1
ENSBTAG00000017318	*TMEM178*	704	227	436	610	262	-1.218	0.183278	1
ENSBTAG00000017321	*SUMO3*	4'303	4'340	4'369	3'727	5'011	0.427	0.580463843	1
ENSBTAG00000017325	*ATG13*	2'961	3'204	3'132	2'564	3'700	0.529	0.497889553	1
ENSBTAG00000017326	*OMA1*	478	292	376	414	337	-0.296	0.751897964	1
ENSBTAG00000017328	*BT.28518*	1'562	674	1'065	1'353	778	-0.798	0.33360152	1
ENSBTAG00000017329	*GMNN*	1'671	1'041	1'325	1'447	1'202	-0.268	0.741760101	1
ENSBTAG00000017330	*IER3IP1*	855	789	826	740	911	0.299	0.721218671	1
ENSBTAG00000017333	*PRX*	1'166	546	820	1'010	630	-0.680	0.419641357	1
ENSBTAG00000017335	*HGFAC*	237	312	283	205	360	0.812	0.407381484	1
ENSBTAG00000017338	*MGC134066*	11	1	5	10	1	-3.044	0.722857941	1
ENSBTAG00000017339	*BT.90652*	932	638	772	807	737	-0.132	0.877903684	1
ENSBTAG00000017343	*COLEC10*	11	2	6	10	2	-2.044	0.786625039	1
ENSBTAG00000017346	*BT.92246*	285	631	488	247	729	1.562	0.084856219	1
ENSBTAG00000017348	*FGF5*	0	20	12	0	23	Inf	0.217717436	1
ENSBTAG00000017349	*PCDHGB4*	16'730	25'659	22'059	14'489	29'628	1.032	0.178723303	1
ENSBTAG00000017350	*PCLO*	242	65	142	210	75	-1.481	0.209725575	1
ENSBTAG00000017352	*ZC3H11A*	8'897	4'807	6'628	7'705	5'551	-0.473	0.539019972	1
ENSBTAG00000017354	*MPP7*	143	121	132	124	140	0.174	0.888634029	1
ENSBTAG00000017355	*ERCC1*	1'316	1'701	1'552	1'140	1'964	0.785	0.328880202	1
ENSBTAG00000017357	*CLCA4*	11	5	8	10	6	-0.722	0.917926652	1
ENSBTAG00000017360	*BT.22129*	1'894	1'470	1'669	1'640	1'697	0.049	0.950717283	1
ENSBTAG00000017361	*BAG5*	1'074	898	984	930	1'037	0.157	0.849585514	1
ENSBTAG00000017362	*CNOT6*	1'905	1'284	1'566	1'650	1'483	-0.154	0.848689328	1
ENSBTAG00000017363	*SAT1*	6'976	4'609	5'682	6'041	5'322	-0.183	0.812964823	1
ENSBTAG00000017365	*ZBTB47*	3'961	6'782	5'631	3'430	7'831	1.191	0.126319514	1
ENSBTAG00000017366	*GRB7*	249	456	371	216	527	1.288	0.170024536	1
ENSBTAG00000017367	*IFIT5*	5'774	4'480	5'087	5'000	5'173	0.049	0.948938094	1
ENSBTAG00000017368	*YBX1*	17'355	16'930	17'289	15'030	19'549	0.379	0.618280027	1
ENSBTAG00000017370	*KIAA1244*	24	4	13	21	5	-2.170	0.58681328	1
ENSBTAG00000017371	*RELL2*	767	484	612	664	559	-0.249	0.77511712	1
ENSBTAG00000017373	*LACTB*	589	251	400	510	290	-0.816	0.376467756	1
ENSBTAG00000017375	*C6H4orf32*	893	353	590	773	408	-0.924	0.290484212	1

Ensembl gene ID	geneName	counts wildtype horn bud	counts polled horn bud	baseMean	baseMean wildtype horn bud	baseMean polled horn bud	log2FoldChange	pval	padj
ENSBTAG00000017376	*BT.61067*	975	253	568	844	292	-1.531	0.084951504	1
ENSBTAG00000017378	*GALNT8*	194	240	223	168	277	0.722	0.484289006	1
ENSBTAG00000017379	*GCN1L1*	5'992	11'812	9'414	5'189	13'639	1.394	0.073038716	1
ENSBTAG00000017380	*MYO18A*	4'417	7'095	6'009	3'825	8'193	1.099	0.157186494	1
ENSBTAG00000017381	*CLDN19*	124	10	59	107	12	-3.217	0.064968505	1
ENSBTAG00000017382	*LEPRE1*	9'520	8'719	9'156	8'245	10'068	0.288	0.70594089	1
ENSBTAG00000017385	*protein_coding*	493	373	429	427	431	0.013	0.991142775	1
ENSBTAG00000017386	*BT.49310*	1'355	1'226	1'295	1'173	1'416	0.271	0.7380029	1
ENSBTAG00000017387	*protein_coding*	76	38	55	66	44	-0.585	0.732051026	1
ENSBTAG00000017388	*CCDC23*	4'646	3'534	4'052	4'024	4'081	0.020	0.978706557	1
ENSBTAG00000017389	*RPLP0*	105'620	127'338	119'253	91'470	147'037	0.685	0.367928038	1
ENSBTAG00000017390	*LGI2*	208	116	157	180	134	-0.427	0.70885072	1
ENSBTAG00000017391	*HS3ST2*	60	29	43	52	33	-0.634	0.742530228	1
ENSBTAG00000017393	*L3MBTL3*	1'240	936	1'077	1'074	1'081	0.009	0.991581214	1
ENSBTAG00000017395	*LASS5*	2'462	2'599	2'567	2'132	3'001	0.493	0.529659632	1
ENSBTAG00000017397	*ZNF423*	2'499	864	1'581	2'164	998	-1.117	0.16716199	1
ENSBTAG00000017401	*TBK1*	1'395	575	936	1'208	664	-0.864	0.3003826	1
ENSBTAG00000017402	*BT.20496*	2'448	1'975	2'200	2'120	2'281	0.105	0.893593736	1
ENSBTAG00000017404	*SEC14L2*	199	182	191	172	210	0.286	0.791518345	1
ENSBTAG00000017405	*BT.49360*	405	407	410	351	470	0.422	0.644428393	1
ENSBTAG00000017407	*HSF2BP*	235	191	212	204	221	0.116	0.915008665	1
ENSBTAG00000017408	*KRT40*	10	4	7	9	5	-0.907	0.905171387	1
ENSBTAG00000017409	*DLX3*	1'608	906	1'219	1'393	1'046	-0.413	0.612858135	1
ENSBTAG00000017412	*SOCS6*	159	69	109	138	80	-0.789	0.537261606	1
ENSBTAG00000017416	*BT.92115*	1'304	1'439	1'395	1'129	1'662	0.557	0.489834668	1
ENSBTAG00000017418	*RRP1B*	996	791	888	863	913	0.083	0.921597112	1
ENSBTAG00000017419	*ZZZ3*	3'726	2'361	2'977	3'227	2'726	-0.243	0.756295307	1
ENSBTAG00000017420	*ZMAT4*	26	33	30	23	38	0.759	0.739377436	1
ENSBTAG00000017421	*SLC3A1*	790	488	624	684	563	-0.280	0.747349405	1
ENSBTAG00000017422	*TAOK2*	3'662	5'199	4'587	3'171	6'003	0.921	0.236460554	1
ENSBTAG00000017423	*TMEM214*	7'234	7'868	7'675	6'265	9'085	0.536	0.484726917	1
ENSBTAG00000017424	*VAPB*	2'372	2'262	2'333	2'054	2'612	0.347	0.659416044	1
ENSBTAG00000017425	*GJB2*	358	328	344	310	379	0.289	0.760021493	1
ENSBTAG00000017426	*PDCD6IP*	7'515	4'466	5'833	6'508	5'157	-0.336	0.663419801	1
ENSBTAG00000017429	*PREPL*	1'188	861	1'012	1'029	994	-0.049	0.95414264	1
ENSBTAG00000017434	*HEATR7B1*	3	4	4	3	5	0.830	0.962490925	1
ENSBTAG00000017435	*BT.35761*	1'320	878	1'078	1'143	1'014	-0.173	0.834187341	1
ENSBTAG00000017436	*BT.71016*	9	4	6	8	5	-0.755	0.931866093	1
ENSBTAG00000017437	*TMEM237*	1'824	1'183	1'473	1'580	1'366	-0.210	0.795408258	1
ENSBTAG00000017438	*C17H5ORF52*	54	21	36	47	24	-0.948	0.651110446	1
ENSBTAG00000017439	*SMCR7L*	1'781	1'360	1'556	1'542	1'570	0.026	0.974343868	1
ENSBTAG00000017440	*METT10D*	320	397	368	277	458	0.726	0.435898977	1
ENSBTAG00000017441	*RPL27*	35'043	34'159	34'896	30'348	39'443	0.378	0.618354186	1
ENSBTAG00000017442	*BT.90608*	3'367	2'777	3'061	2'916	3'207	0.137	0.860106712	1
ENSBTAG00000017443	*BT.42344*	187	73	123	162	84	-0.942	0.442314673	1
ENSBTAG00000017446	*BT.30900*	194	228	216	168	263	0.648	0.533269513	1
ENSBTAG00000017448	*EFEMP1*	7'147	3'813	5'296	6'189	4'403	-0.491	0.524936881	1
ENSBTAG00000017450	*KLHL24*	414	347	380	359	401	0.160	0.864024688	1
ENSBTAG00000017451	*TSPAN17*	948	900	930	821	1'039	0.340	0.681758408	1
ENSBTAG00000017452	*RCAN3*	495	511	509	429	590	0.461	0.602724656	1
ENSBTAG00000017455	*ADAM9*	2'260	1'799	2'017	1'957	2'077	0.086	0.913433921	1
ENSBTAG00000017456	*NYAP1*	759	805	793	657	930	0.500	0.552497871	1
ENSBTAG00000017457	*STK10*	904	620	749	783	716	-0.129	0.880847638	1
ENSBTAG00000017458	*BT.96872*	15	0	6	13	0		0.491308095	1
ENSBTAG00000017459	*DUSP18*	213	243	233	184	281	0.605	0.553642724	1
ENSBTAG00000017460	*PRORSD1*	1'320	607	922	1'143	701	-0.706	0.3971836	1
ENSBTAG00000017461	*SLC16A3*	445	536	502	385	619	0.683	0.441577297	1
ENSBTAG00000017462	*BT.76048*	23'027	13'535	17'785	19'942	15'629	-0.352	0.64511805	1
ENSBTAG00000017463	*RPS19BP1*	1'130	729	910	979	842	-0.217	0.79523947	1
ENSBTAG00000017465	*BT.20235*	8'899	4'850	6'654	7'707	5'600	-0.461	0.549774157	1
ENSBTAG00000017466	*HCN4*	44	133	96	38	154	2.011	0.139241185	1
ENSBTAG00000017468	*NUCB2*	4'899	1'964	3'255	4'243	2'268	-0.904	0.249186541	1
ENSBTAG00000017469	*CCDC88A*	2'286	1'001	1'568	1'980	1'156	-0.776	0.334820765	1
ENSBTAG00000017471	*KBTBD13*	1	0	0	1	0		1	1
ENSBTAG00000017475	*GNAS*	80'686	43'172	59'863	69'876	49'851	-0.487	0.522041983	1
ENSBTAG00000017477	*pseudogene*	7	3	5	6	3	-0.807	0.947428618	1
ENSBTAG00000017478	*GSDMC*	660	209	406	572	241	-1.244	0.178990231	1
ENSBTAG00000017480	*PDE6B*	0	1	1	0	1	Inf	0.993540919	1
ENSBTAG00000017482	*ISYNA1*	3'526	7'101	5'627	3'054	8'200	1.425	0.068840609	1
ENSBTAG00000017483	*BT.28437*	39	48	45	34	55	0.715	0.700460469	1
ENSBTAG00000017486	*ADARB1*	325	317	324	281	366	0.379	0.691202224	1
ENSBTAG00000017488	*KLF3*	575	267	403	498	308	-0.692	0.452255817	1

Ensembl gene ID	geneName	counts wildtype horn bud	counts polled horn bud	baseMean	baseMean wildtype horn bud	baseMean polled horn bud	log2FoldChange	pval	padj
ENSBTAG00000017489	*TSHR*	0	1	1	0	1	Inf	0.993540919	1
ENSBTAG00000017490	*PRKCSH*	613	265	418	531	306	-0.795	0.385190473	1
ENSBTAG00000017492	*PCMTD1*	6'462	4'099	5'165	5'596	4'733	-0.242	0.754716876	1
ENSBTAG00000017493	*BT.95514*	802	408	583	695	471	-0.560	0.521260796	1
ENSBTAG00000017496	*ATP5I*	1'278	1'490	1'414	1'107	1'721	0.636	0.430224526	1
ENSBTAG00000017500	*BT.26879*	374	522	463	324	603	0.896	0.319648309	1
ENSBTAG00000017502	*RIMKLA*	34	23	28	29	27	-0.149	0.966657603	1
ENSBTAG00000017504	*FAIM2*	366	265	311	317	306	-0.051	0.962148681	1
ENSBTAG00000017505	*BT.21228*	1'185	900	1'033	1'026	1'039	0.018	0.98302825	1
ENSBTAG00000017507	*SV2C*	1	0	0	1	0		1	1
ENSBTAG00000017508	*CYSLTR2*	174	39	98	151	45	-1.743	0.195363506	1
ENSBTAG00000017514	*CCND1*	1'020	1'192	1'130	883	1'376	0.640	0.434342056	1
ENSBTAG00000017515	*CCDC168*	528	129	303	457	149	-1.618	0.099908093	1
ENSBTAG00000017517	*ZNF236*	1'455	983	1'198	1'260	1'135	-0.151	0.854557085	1
ENSBTAG00000017521	*ATOH7*	2	0	1	2	0		0.974934741	1
ENSBTAG00000017524	*CBLN4*	35	46	42	30	53	0.809	0.672422683	1
ENSBTAG00000017525	*BT.49134*	2'550	1'618	2'038	2'208	1'868	-0.241	0.761399927	1
ENSBTAG00000017527	*AIM1*	2'129	2'343	2'275	1'844	2'705	0.553	0.482902451	1
ENSBTAG00000017528	*SNAI3*	0	11	6	0	13	Inf	0.464170207	1
ENSBTAG00000017529	*CA8*	149	23	78	129	27	-2.281	0.126805778	1
ENSBTAG00000017530	*C8H9ORF46*	1'527	795	1'120	1'322	918	-0.527	0.520849722	1
ENSBTAG00000017533	*LRRC1*	1'022	653	820	885	754	-0.231	0.784403695	1
ENSBTAG00000017536	*MPP3*	268	158	207	232	182	-0.347	0.743956099	1
ENSBTAG00000017537	*PTGIS*	1'469	574	967	1'272	663	-0.941	0.258522125	1
ENSBTAG00000017540	*BT.42471*	2'801	1'179	1'894	2'426	1'361	-0.833	0.296496594	1
ENSBTAG00000017542	*PPARD*	507	517	518	439	597	0.443	0.615936362	1
ENSBTAG00000017543	*XRCC6BP1*	1'125	783	939	974	904	-0.108	0.898358577	1
ENSBTAG00000017545	*BT.47551*	1'500	689	1'047	1'299	796	-0.707	0.391322249	1
ENSBTAG00000017547	*DBNL*	5'263	6'418	5'984	4'558	7'411	0.701	0.363411282	1
ENSBTAG00000017549	*KITLG*	237	211	224	205	244	0.247	0.812172321	1
ENSBTAG00000017550	*BT.26664*	5'172	2'894	3'910	4'479	3'342	-0.423	0.586657127	1
ENSBTAG00000017551	*DROSHA*	1'868	848	1'298	1'618	979	-0.724	0.373108845	1
ENSBTAG00000017554	*RTN4IP1*	806	455	612	698	525	-0.410	0.637021985	1
ENSBTAG00000017557	*BT.64411*	702	752	738	608	868	0.514	0.544030353	1
ENSBTAG00000017560	*CEBPG*	810	711	761	701	821	0.227	0.788429844	1
ENSBTAG00000017561	*HHIPL2*	4	0	2	3	0		0.89545886	1
ENSBTAG00000017564	*CGN*	1'600	1'316	1'453	1'386	1'520	0.133	0.868510669	1
ENSBTAG00000017565	*SCFD1*	4'904	2'486	3'559	4'247	2'871	-0.565	0.468502961	1
ENSBTAG00000017566	*TUFT1*	2'571	1'673	2'079	2'227	1'932	-0.205	0.796541635	1
ENSBTAG00000017567	*BT.88312*	2'236	1'454	1'808	1'936	1'679	-0.206	0.796845035	1
ENSBTAG00000017569	*C20H5ORF22*	934	486	685	809	561	-0.527	0.538228197	1
ENSBTAG00000017570	*pseudogene*	244	119	174	211	137	-0.621	0.574010093	1
ENSBTAG00000017571	*PLEKHA5*	6'283	3'918	4'983	5'441	4'524	-0.266	0.730749109	1
ENSBTAG00000017573	*RBM26*	2'058	1'246	1'611	1'782	1'439	-0.309	0.700362749	1
ENSBTAG00000017574	*LMNA*	16'719	24'649	21'471	14'479	28'462	0.975	0.203494869	1
ENSBTAG00000017576	*GPR144*	5	1	3	4	1	-1.907	0.9188325	1
ENSBTAG00000017580	*RFX5*	321	367	351	278	424	0.608	0.517143356	1
ENSBTAG00000017582	*CHEK1*	723	717	727	626	828	0.403	0.634890989	1
ENSBTAG00000017584	*PLCG1*	5'660	7'721	6'909	4'902	8'915	0.863	0.263578493	1
ENSBTAG00000017592	*AGPAT9*	391	276	329	339	319	-0.087	0.930571857	1
ENSBTAG00000017593	*TREM1*	3	1	2	3	1	-1.170	0.985253077	1
ENSBTAG00000017594	*ZHX3*	374	816	633	324	942	1.541	0.078917499	1
ENSBTAG00000017595	*HS2ST1*	60	101	84	52	117	1.166	0.404202114	1
ENSBTAG00000017597	*ESCO1*	920	474	672	797	547	-0.542	0.52814409	1
ENSBTAG00000017599	*NR2F1*	2'377	638	1'398	2'059	737	-1.482	0.070874923	1
ENSBTAG00000017602	*TMEM45B*	562	185	350	487	214	-1.188	0.210163776	1
ENSBTAG00000017604	*RAB13*	3'089	2'164	2'587	2'675	2'499	-0.098	0.901230646	1
ENSBTAG00000017605	*AK2*	2'850	2'979	2'954	2'468	3'440	0.479	0.539794857	1
ENSBTAG00000017610	*NFRKB*	1'128	2'152	1'731	977	2'485	1.347	0.095719413	1
ENSBTAG00000017611	*FAM190B*	1'550	1'218	1'374	1'342	1'406	0.067	0.933723586	1
ENSBTAG00000017613	*BT.30862*	785	360	548	680	416	-0.710	0.420189595	1
ENSBTAG00000017616	*ADSSL1*	318	424	382	275	490	0.830	0.370410046	1
ENSBTAG00000017617	*C6ORF222*	35	19	26	30	22	-0.466	0.860711567	1
ENSBTAG00000017618	*MAP3K10*	790	1'152	1'007	684	1'330	0.959	0.247237022	1
ENSBTAG00000017622	*SIVA1*	436	437	441	378	505	0.418	0.643666594	1
ENSBTAG00000017624	*RAB17*	5	2	3	4	2	-0.907	0.965043111	1
ENSBTAG00000017626	*BT.54106*	715	457	573	619	528	-0.231	0.793257158	1
ENSBTAG00000017628	*MAP7D2*	43	2	20	37	2	-4.011	0.219995324	1
ENSBTAG00000017629	*protein_coding*	605	482	540	524	557	0.087	0.922276349	1
ENSBTAG00000017631	*GFER*	846	958	919	733	1'106	0.594	0.474496538	1
ENSBTAG00000017632	*AEN*	72	84	80	62	97	0.637	0.65640914	1
ENSBTAG00000017633	*EIF1AX*	6'079	4'474	5'215	5'265	5'166	-0.027	0.972767706	1

Ensembl gene ID	geneName	counts wildtype horn bud	counts polled horn bud	baseMean	baseMean wildtype horn bud	baseMean polled horn bud	log2FoldChange	pval	padj
ENSBTAG00000017636	*AKT1*	8'234	10'173	9'439	7'131	11'747	0.720	0.348195025	1
ENSBTAG00000017639	*RPS6KA3*	317	137	216	275	158	-0.795	0.445933546	1
ENSBTAG00000017642	*BMP3*	157	125	140	136	144	0.086	0.946723568	1
ENSBTAG00000017644	*BT.24244*	121	125	125	105	144	0.462	0.705438106	1
ENSBTAG00000017645	*pseudogene*	17	4	10	15	5	-1.672	0.733083131	1
ENSBTAG00000017648	*TIFAB*	6	3	4	5	3	-0.585	0.976586913	1
ENSBTAG00000017649	*SCML1*	384	211	288	333	244	-0.449	0.647108203	1
ENSBTAG00000017650	*TMPRSS5*	229	41	123	198	47	-2.067	0.100491042	1
ENSBTAG00000017651	*protein_coding*	530	255	377	459	294	-0.640	0.491275288	1
ENSBTAG00000017652	*BT.37218*	1'136	1'172	1'169	984	1'353	0.460	0.572568873	1
ENSBTAG00000017655	*PALMD*	2'486	1'699	2'057	2'153	1'962	-0.134	0.866488435	1
ENSBTAG00000017656	*SIAT6*	1'126	1'526	1'369	975	1'762	0.854	0.292393449	1
ENSBTAG00000017659	*BFSP2*	20	8	13	17	9	-0.907	0.820299743	1
ENSBTAG00000017660	*BT.70368*	259	392	338	224	453	1.013	0.285740286	1
ENSBTAG00000017661	*RFX2*	459	1'024	790	398	1'182	1.573	0.066746208	1
ENSBTAG00000017662	*EEF2K*	2'257	1'606	1'905	1'955	1'854	-0.076	0.925103095	1
ENSBTAG00000017663	*GAST*	1	3	2	1	3	2.000	0.933774563	1
ENSBTAG00000017664	*HGF*	107	32	65	93	37	-1.326	0.397390753	1
ENSBTAG00000017665	*DHRS4*	1'290	394	786	1'117	455	-1.296	0.128782063	1
ENSBTAG00000017666	*BT.104466*	1'921	1'052	1'439	1'664	1'215	-0.454	0.574033032	1
ENSBTAG00000017670	*BT.60918*	70	33	49	61	38	-0.670	0.707968507	1
ENSBTAG00000017672	*HAS1*	45	18	30	39	21	-0.907	0.694702049	1
ENSBTAG00000017674	*SCNN1D*	84	115	103	73	133	0.868	0.503416149	1
ENSBTAG00000017676	*TPK1*	759	411	566	657	475	-0.470	0.591881564	1
ENSBTAG00000017677	*SCG3*	775	192	446	671	222	-1.598	0.082404859	1
ENSBTAG00000017678	*SPRED3*	39	27	32	34	31	-0.115	0.974021001	1
ENSBTAG00000017679	*PAK7*	112	195	161	97	225	1.215	0.28179171	1
ENSBTAG00000017680	*NOS3*	958	569	743	830	657	-0.337	0.692423773	1
ENSBTAG00000017681	*RIN1*	120	234	187	104	270	1.379	0.205004749	1
ENSBTAG00000017682	*TET2*	1'123	611	839	973	706	-0.463	0.581350543	1
ENSBTAG00000017683	*LYSMD2*	85	118	105	74	136	0.888	0.490416475	1
ENSBTAG00000017684	*BT.87196*	1	1	1	1	1	0.415	1	1
ENSBTAG00000017685	*JUP*	10'390	16'859	14'233	8'998	19'467	1.113	0.148429115	1
ENSBTAG00000017689	*BRMS1*	1'000	1'020	1'022	866	1'178	0.444	0.589980146	1
ENSBTAG00000017690	*BT.24269*	225	356	303	195	411	1.077	0.266739361	1
ENSBTAG00000017691	*pseudogene*	111	65	86	96	75	-0.357	0.803168812	1
ENSBTAG00000017694	*TRPS1*	977	1'512	1'296	846	1'746	1.045	0.200299526	1
ENSBTAG00000017695	*CCDC150*	449	242	334	389	279	-0.477	0.616432182	1
ENSBTAG00000017697	*CTF1*	92	53	70	80	61	-0.381	0.806541232	1
ENSBTAG00000017698	*PSMA8*	5	0	2	4	0		0.853268594	1
ENSBTAG00000017701	*CYP2W1*	580	543	565	502	627	0.320	0.714400779	1
ENSBTAG00000017704	*ABCG2*	2'264	1'815	2'028	1'961	2'096	0.096	0.90315187	1
ENSBTAG00000017706	*SLC39A3*	376	491	446	326	567	0.800	0.376287588	1
ENSBTAG00000017709	*ATRNL1*	202	165	183	175	191	0.123	0.913496763	1
ENSBTAG00000017710	*ECHS1*	4'801	3'816	4'282	4'158	4'406	0.084	0.913362666	1
ENSBTAG00000017711	*WHAMM*	927	741	829	803	856	0.092	0.913249234	1
ENSBTAG00000017713	*BT.64023*	6'154	2'498	4'107	5'330	2'884	-0.886	0.255961579	1
ENSBTAG00000017714	*C4BPB*	2	0	1	2	0		0.974934741	1
ENSBTAG00000017715	*HOMER2*	728	585	653	630	675	0.100	0.908620893	1
ENSBTAG00000017716	*BEGAIN*	406	592	518	352	684	0.959	0.279843333	1
ENSBTAG00000017717	*BT.51837*	81	11	41	70	13	-2.465	0.21493338	1
ENSBTAG00000017718	*CCL22*	7	0	3	6	0		0.769774961	1
ENSBTAG00000017721	*METTL13*	476	680	599	412	785	0.930	0.28591915	1
ENSBTAG00000017722	*BT.23666*	85	39	59	74	45	-0.709	0.664692061	1
ENSBTAG00000017727	*KIFAP3*	4'693	2'782	3'638	4'064	3'212	-0.339	0.662983099	1
ENSBTAG00000017729	*BT.28215*	1'175	685	904	1'018	791	-0.363	0.6632742	1
ENSBTAG00000017731	*ZFAT*	425	471	456	368	544	0.563	0.531271575	1
ENSBTAG00000017734	*BT.106071*	329	155	232	285	179	-0.671	0.513298438	1
ENSBTAG00000017738	*BT.62212*	4'210	3'808	4'022	3'646	4'397	0.270	0.726791166	1
ENSBTAG00000017739	*BT.76175*	310	558	456	268	644	1.263	0.164351071	1
ENSBTAG00000017740	*BT.54364*	0	1	1	0	1	Inf	0.993540919	1
ENSBTAG00000017741	*BT.45949*	884	461	649	766	532	-0.524	0.543112027	1
ENSBTAG00000017743	*XIRP2*	229	399	330	198	461	1.216	0.203813379	1
ENSBTAG00000017744	*FAF2*	1'057	972	1'019	915	1'122	0.294	0.720907897	1
ENSBTAG00000017745	*BT.66498*	1'644	1'031	1'307	1'424	1'190	-0.258	0.750940979	1
ENSBTAG00000017746	*SLIT3*	8'456	9'748	9'290	7'323	11'256	0.620	0.418591057	1
ENSBTAG00000017747	*PLSCR3*	5'077	6'345	5'862	4'397	7'327	0.737	0.340082907	1
ENSBTAG00000017748	*RNF44*	2'196	1'866	2'028	1'902	2'155	0.180	0.819670594	1
ENSBTAG00000017750	*RHBDD3*	780	707	746	675	816	0.273	0.746965444	1
ENSBTAG00000017751	*RGS9*	506	534	527	438	617	0.493	0.576150746	1
ENSBTAG00000017752	*DYNLRB2*	151	83	113	131	96	-0.448	0.724751657	1
ENSBTAG00000017753	*APP*	94'621	100'244	98'848	81'944	115'752	0.498	0.511409724	1

Ensembl gene ID	geneName	counts wildtype horn bud	counts polled horn bud	baseMean	baseMean wildtype horn bud	baseMean polled horn bud	log2FoldChange	pval	padj
ENSBTAG00000017755	*EWSR1*	14'757	8'788	11'464	12'780	10'148	-0.333	0.663828494	1
ENSBTAG00000017759	*BT.48087*	7'211	8'963	8'297	6'245	10'350	0.729	0.343082528	1
ENSBTAG00000017761	*BT.91789*	1'046	1'138	1'110	906	1'314	0.537	0.512140943	1
ENSBTAG00000017762	*GNG8*	22	13	17	19	15	-0.344	0.931546014	1
ENSBTAG00000017763	*NFIL3*	2'371	1'379	1'823	2'053	1'592	-0.367	0.645488338	1
ENSBTAG00000017764	*HDAC4*	586	778	703	507	898	0.824	0.334928376	1
ENSBTAG00000017765	*GSTM1*	1'529	1'042	1'264	1'324	1'203	-0.138	0.866160004	1
ENSBTAG00000017766	*pseudogene*	80	33	54	69	38	-0.862	0.612960782	1
ENSBTAG00000017767	*GPR173*	621	716	682	538	827	0.620	0.468203882	1
ENSBTAG00000017769	*BT.55972*	203	146	172	176	169	-0.060	0.962761944	1
ENSBTAG00000017770	*LUC7L2*	5'325	3'925	4'572	4'612	4'532	-0.025	0.975126608	1
ENSBTAG00000017771	*EPN2*	1'158	1'231	1'212	1'003	1'421	0.503	0.536191696	1
ENSBTAG00000017775	*SDCCAG8*	328	160	234	284	185	-0.621	0.54442187	1
ENSBTAG00000017776	*BT.89866*	5'467	3'654	4'477	4'735	4'219	-0.166	0.830568918	1
ENSBTAG00000017779	*HSD17B10*	2'173	2'402	2'328	1'882	2'774	0.560	0.47753547	1
ENSBTAG00000017781	*BCDIN3D*	455	350	399	394	404	0.037	0.970525397	1
ENSBTAG00000017783	*TSPYL2*	3'001	1'650	2'252	2'599	1'905	-0.448	0.57080927	1
ENSBTAG00000017785	*FRMPD4*	79	65	72	68	75	0.134	0.936796956	1
ENSBTAG00000017786	*FCRL6*	15	41	30	13	47	1.866	0.411278196	1
ENSBTAG00000017788	*AKT3*	822	503	646	712	581	-0.294	0.734324001	1
ENSBTAG00000017790	*B9D1*	516	615	579	447	710	0.668	0.443441688	1
ENSBTAG00000017793	*KLRG2*	23	28	26	20	32	0.699	0.780285198	1
ENSBTAG00000017794	*CCDC153*	96	18	52	83	21	-2.000	0.254542327	1
ENSBTAG00000017797	*BT.59869*	107	13	54	93	15	-2.626	0.137824231	1
ENSBTAG00000017798	*ERH*	1'841	1'461	1'641	1'594	1'687	0.082	0.918765391	1
ENSBTAG00000017799	*PITPNB*	3'924	3'156	3'521	3'398	3'644	0.101	0.89640528	1
ENSBTAG00000017801	*ATP6AP2*	5'739	2'842	4'126	4'970	3'282	-0.599	0.44092287	1
ENSBTAG00000017802	*THRB*	245	291	274	212	336	0.663	0.500766947	1
ENSBTAG00000017803	*RAB14*	1'601	1'197	1'384	1'387	1'382	-0.005	0.997290137	1
ENSBTAG00000017804	*BNIP3*	1'466	1'036	1'233	1'270	1'196	-0.086	0.917491097	1
ENSBTAG00000017805	*EFCAB4A*	510	279	382	442	322	-0.455	0.624273159	1
ENSBTAG00000017808	*ARSJ*	263	383	335	228	442	0.957	0.313521986	1
ENSBTAG00000017809	*PDS5A*	1'681	1'143	1'388	1'456	1'320	-0.141	0.86212614	1
ENSBTAG00000017810	*EFHC1*	383	162	259	332	187	-0.826	0.409021736	1
ENSBTAG00000017811	*BT.27129*	1'942	1'671	1'806	1'682	1'930	0.198	0.802954909	1
ENSBTAG00000017812	*BT.24359*	915	755	832	792	872	0.138	0.869831613	1
ENSBTAG00000017814	*LGI4*	1'212	1'093	1'156	1'050	1'262	0.266	0.744348619	1
ENSBTAG00000017815	*STX8*	1'191	808	982	1'031	933	-0.145	0.862567553	1
ENSBTAG00000017816	*FXYD1*	2'242	2'063	2'162	1'942	2'382	0.295	0.708254701	1
ENSBTAG00000017818	*BT.104881*	698	361	511	604	417	-0.536	0.546070052	1
ENSBTAG00000017819	*BT.39186*	241	221	232	209	255	0.290	0.778125049	1
ENSBTAG00000017821	*pseudogene*	6	3	4	5	3	-0.585	0.976586913	1
ENSBTAG00000017823	*WDR16*	19	2	9	16	2	-2.833	0.582174212	1
ENSBTAG00000017824	*IRF8*	452	201	312	391	232	-0.754	0.433765131	1
ENSBTAG00000017825	*SEC13*	4'978	4'983	5'032	4'311	5'754	0.416	0.589126098	1
ENSBTAG00000017826	*SCLY*	347	454	412	301	524	0.803	0.380417057	1
ENSBTAG00000017827	*BT.97035*	9'965	3'759	6'485	8'630	4'341	-0.991	0.200712158	1
ENSBTAG00000017829	*protein_coding*	0	3	2	0	3	Inf	0.878646179	1
ENSBTAG00000017830	*RBMS2*	1'912	2'040	2'006	1'656	2'356	0.509	0.520843218	1
ENSBTAG00000017831	*ERLIN2*	11'039	7'330	9'012	9'560	8'464	-0.176	0.819156725	1
ENSBTAG00000017832	*PDK3*	571	276	407	495	319	-0.634	0.490326274	1
ENSBTAG00000017833	*RNF19A*	6'538	4'399	5'371	5'662	5'080	-0.157	0.839710576	1
ENSBTAG00000017834	*PRELP*	17'234	8'193	12'193	14'925	9'460	-0.658	0.390835758	1
ENSBTAG00000017835	*C5ORF63*	234	105	162	203	121	-0.741	0.510726553	1
ENSBTAG00000017836	*ZFP57*	436	340	385	378	393	0.056	0.953650102	1
ENSBTAG00000017837	*GABRB1*	32	1	14	28	1	-4.585	0.277113151	1
ENSBTAG00000017839	*BT.74139*	1'396	1'254	1'328	1'209	1'448	0.260	0.747345589	1
ENSBTAG00000017840	*BAZ2A*	5'725	4'456	5'052	4'958	5'145	0.054	0.944248989	1
ENSBTAG00000017843	*CD40LG*	15	4	9	13	5	-1.492	0.779648463	1
ENSBTAG00000017844	*STIL*	258	193	223	223	223	-0.004	1	1
ENSBTAG00000017845	*BAG1*	3'376	2'803	3'080	2'924	3'237	0.147	0.850419386	1
ENSBTAG00000017846	*F11R*	4'037	2'811	3'371	3'496	3'246	-0.107	0.89142745	1
ENSBTAG00000017847	*PPP1R12C*	1'764	1'765	1'783	1'528	2'038	0.416	0.601175914	1
ENSBTAG00000017850	*ARHGEF6*	2'566	1'662	2'071	2'222	1'919	-0.212	0.790028684	1
ENSBTAG00000017851	*BT.78456*	1'637	3'441	2'696	1'418	3'973	1.487	0.062275765	1
ENSBTAG00000017852	*ALDH9A1*	4'315	4'089	4'229	3'737	4'722	0.337	0.66251183	1
ENSBTAG00000017855	*ITM2C*	11'392	10'646	11'079	9'866	12'293	0.317	0.677430113	1
ENSBTAG00000017856	*TSTD1*	448	550	512	388	635	0.711	0.422372681	1
ENSBTAG00000017860	*BT.24840*	58	68	64	50	79	0.645	0.680900996	1
ENSBTAG00000017863	*BT.97928*	1'209	501	813	1'047	579	-0.856	0.310648856	1
ENSBTAG00000017864	*PRPH*	72	14	39	62	16	-1.948	0.331231258	1
ENSBTAG00000017867	*YPEL1*	1'409	1'212	1'310	1'220	1'399	0.198	0.806854714	1

Ensembl gene ID	geneName	counts wildtype horn bud	counts polled horn bud	baseMean	baseMean wildtype horn bud	baseMean polled horn bud	log2FoldChange	pval	padj
ENSBTAG00000017868	*DHX40*	4'055	3'232	3'622	3'512	3'732	0.088	0.909645134	1
ENSBTAG00000017869	*CAV1*	17'654	18'083	18'085	15'289	20'880	0.450	0.554881122	1
ENSBTAG00000017871	*GNA12*	1'200	2'380	1'894	1'039	2'748	1.403	0.081860542	1
ENSBTAG00000017872	*ZNF304*	737	367	531	638	424	-0.591	0.503667121	1
ENSBTAG00000017873	*USF1*	2'419	2'446	2'460	2'095	2'824	0.431	0.583085191	1
ENSBTAG00000017875	*ARHGAP30*	412	335	372	357	387	0.117	0.902032946	1
ENSBTAG00000017877	*PVRL4*	1'924	2'023	2'001	1'666	2'336	0.487	0.538245676	1
ENSBTAG00000017879	*C9H6orf163*	192	104	143	166	120	-0.469	0.689609419	1
ENSBTAG00000017882	*LRRC3*	201	391	313	174	451	1.375	0.15615523	1
ENSBTAG00000017883	*LRRC38*	7	5	6	6	6	-0.070	1	1
ENSBTAG00000017885	*BT.65753*	447	346	393	387	400	0.046	0.962772508	1
ENSBTAG00000017886	*CABP7*	43	41	42	37	47	0.346	0.862675507	1
ENSBTAG00000017889	*BT.68634*	5'612	5'072	5'358	4'860	5'857	0.269	0.726462843	1
ENSBTAG00000017890	*ZMAT5*	259	280	274	224	323	0.528	0.592462848	1
ENSBTAG00000017892	*protein_coding*	39	4	19	34	5	-2.870	0.355509969	1
ENSBTAG00000017894	*CAD*	4'643	6'901	5'995	4'021	7'969	0.987	0.202981987	1
ENSBTAG00000017895	*CLEC10A*	4	0	2	3	0		0.89545886	1
ENSBTAG00000017896	*ST8SIA2*	87	179	141	75	207	1.456	0.216966718	1
ENSBTAG00000017901	*LAYN*	393	229	302	340	264	-0.364	0.708233631	1
ENSBTAG00000017905	*WDR91*	1'728	1'708	1'734	1'496	1'972	0.398	0.617093006	1
ENSBTAG00000017907	*COG3*	1'735	1'173	1'429	1'503	1'354	-0.150	0.853820364	1
ENSBTAG00000017910	*pseudogene*	1	1	1	1	1	0.415	1	1
ENSBTAG00000017911	*BT.99181*	0	1	1	0	1	Inf	0.993540919	1
ENSBTAG00000017913	*BUB3*	2'797	1'787	2'243	2'422	2'063	-0.231	0.77011683	1
ENSBTAG00000017919	*BT.26972*	1'095	680	867	948	785	-0.272	0.745638642	1
ENSBTAG00000017921	*BT.97056*	1'955	2'038	2'023	1'693	2'353	0.475	0.548405886	1
ENSBTAG00000017922	*DOLPP1*	681	754	730	590	871	0.562	0.50795529	1
ENSBTAG00000017925	*LMF2*	4'836	4'347	4'604	4'188	5'019	0.261	0.734806462	1
ENSBTAG00000017927	*EGFL6*	2	0	1	2	0		0.974934741	1
ENSBTAG00000017932	*CCDC84*	642	321	463	556	371	-0.585	0.515953166	1
ENSBTAG00000017936	*C9ORF4*	83	40	59	72	46	-0.638	0.698040567	1
ENSBTAG00000017939	*BT.40032*	655	1'163	955	567	1'343	1.243	0.137455667	1
ENSBTAG00000017941	*NSUN5*	441	422	435	382	487	0.352	0.698368872	1
ENSBTAG00000017942	*CRTC2*	1'914	2'041	2'007	1'658	2'357	0.508	0.521484558	1
ENSBTAG00000017943	*GALNT6*	62	31	45	54	36	-0.585	0.757140162	1
ENSBTAG00000017945	*TCEANC*	254	228	242	220	263	0.259	0.79962572	1
ENSBTAG00000017946	*protein_coding*	105	22	58	91	25	-1.840	0.267941073	1
ENSBTAG00000017948	*SPIN4*	242	99	162	210	114	-0.874	0.437727019	1
ENSBTAG00000017949	*BT.64328*	2	0	1	2	0		0.974934741	1
ENSBTAG00000017950	*SLC4A8*	12	1	6	10	1	-3.170	0.692545189	1
ENSBTAG00000017951	*POLD4*	1'066	924	995	923	1'067	0.209	0.800272647	1
ENSBTAG00000017955	*KCNIP3*	21	13	17	18	15	-0.277	0.951008818	1
ENSBTAG00000017956	*KCTD15*	4'676	5'930	5'448	4'050	6'847	0.758	0.327119371	1
ENSBTAG00000017957	*SPATA24*	885	762	823	766	880	0.199	0.812513409	1
ENSBTAG00000017958	*AHI1*	1'072	561	788	928	648	-0.519	0.53878769	1
ENSBTAG00000017964	*LRRC51*	151	126	138	131	145	0.154	0.900496244	1
ENSBTAG00000017967	*PTGES3*	16'737	9'721	12'860	14'495	11'225	-0.369	0.629684689	1
ENSBTAG00000017969	*CA4*	12	5	8	10	6	-0.848	0.892845055	1
ENSBTAG00000017970	*ZYX*	6'192	13'691	10'586	5'362	15'809	1.560	0.045595086	1
ENSBTAG00000017971	*LRFN2*	44	26	34	38	30	-0.344	0.882655891	1
ENSBTAG00000017980	*BT.23584*	0	1	1	0	1	Inf	0.993540919	1
ENSBTAG00000017981	*ADRB3*	13	8	10	11	9	-0.285	0.973425765	1
ENSBTAG00000017982	*FIBCD1*	56	12	31	48	14	-1.807	0.422317673	1
ENSBTAG00000017983	*SLMO1*	55	33	43	48	38	-0.322	0.874633247	1
ENSBTAG00000017986	*GLIS1*	206	307	266	178	354	0.991	0.318921823	1
ENSBTAG00000017992	*CD164*	8'753	4'048	6'127	7'580	4'674	-0.698	0.366488117	1
ENSBTAG00000017994	*MAX*	1'142	960	1'049	989	1'109	0.165	0.841382998	1
ENSBTAG00000017996	*EGLN2*	4'159	4'113	4'176	3'602	4'749	0.399	0.60610288	1
ENSBTAG00000017999	*TNRC6A*	3'383	2'797	3'080	2'930	3'230	0.141	0.8565402	1
ENSBTAG00000018000	*CLN3*	1'441	1'339	1'397	1'248	1'546	0.309	0.701152991	1
ENSBTAG00000018002	*DTX3*	3'303	2'977	3'149	2'860	3'438	0.265	0.733347072	1
ENSBTAG00000018003	*GEFT*	8'349	9'274	8'970	7'230	10'709	0.567	0.459738431	1
ENSBTAG00000018004	*SH3GL3*	473	445	462	410	514	0.327	0.716050765	1
ENSBTAG00000018007	*NR2F2*	2'820	779	1'671	2'442	900	-1.441	0.076011365	1
ENSBTAG00000018009	*LEKR1*	9	3	6	8	3	-1.170	0.890813994	1
ENSBTAG00000018010	*ABCA4*	12	7	9	10	8	-0.363	0.964206651	1
ENSBTAG00000018011	*APOBR*	277	254	267	240	293	0.290	0.771030662	1
ENSBTAG00000018013	*EMP3*	6'968	5'573	6'235	6'034	6'435	0.093	0.903448438	1
ENSBTAG00000018015	*BT.101510*	61	41	50	53	47	-0.158	0.940064916	1
ENSBTAG00000018016	*NUPR1*	2'082	836	1'384	1'803	965	-0.901	0.266875527	1
ENSBTAG00000018019	*BCL11B*	281	599	468	243	692	1.507	0.09784589	1
ENSBTAG00000018020	*GNAT1*	2'512	3'311	2'999	2'175	3'823	0.813	0.29948017	1

Ensembl gene ID	geneName	counts wildtype horn bud	counts polled horn bud	baseMean	baseMean wildtype horn bud	baseMean polled horn bud	log2FoldChange	pval	padj
ENSBTAG00000018022	*C11ORF84*	1'620	1'802	1'742	1'403	2'081	0.569	0.47583689	1
ENSBTAG00000018024	*NR1D2*	290	256	273	251	296	0.235	0.813046127	1
ENSBTAG00000018025	*BT.56204*	61	31	44	53	36	-0.562	0.76827261	1
ENSBTAG00000018026	*LANCL2*	1'312	1'546	1'461	1'136	1'785	0.652	0.418409303	1
ENSBTAG00000018028	*TTLL10*	16	22	20	14	25	0.874	0.767703817	1
ENSBTAG00000018031	*PODXL2*	674	691	691	584	798	0.451	0.597166301	1
ENSBTAG00000018033	*PIP4K2A*	1'176	989	1'080	1'018	1'142	0.165	0.84043016	1
ENSBTAG00000018035	*TGFBR1*	673	764	733	583	882	0.598	0.481095002	1
ENSBTAG00000018036	*CNOT7*	5'925	4'844	5'362	5'131	5'593	0.124	0.871206937	1
ENSBTAG00000018037	*PSKH1*	655	1'318	1'045	567	1'522	1.424	0.088087902	1
ENSBTAG00000018039	*BT.100618*	1	0	0	1	0		1	1
ENSBTAG00000018040	*PSMB10*	1'672	1'189	1'410	1'448	1'373	-0.077	0.925599357	1
ENSBTAG00000018041	*ACADSB*	1'080	1'348	1'246	935	1'557	0.735	0.366681738	1
ENSBTAG00000018043	*LCAT*	4'742	4'495	4'649	4'107	5'190	0.338	0.661500368	1
ENSBTAG00000018044	*POLR3E*	1'443	891	1'139	1'250	1'029	-0.281	0.732438384	1
ENSBTAG00000018045	*SLC12A4*	6'954	11'741	9'790	6'022	13'557	1.171	0.130169409	1
ENSBTAG00000018046	*B3GNT2*	1'373	758	1'032	1'189	875	-0.442	0.592229666	1
ENSBTAG00000018048	*MED15*	2'577	2'530	2'577	2'232	2'921	0.388	0.620147314	1
ENSBTAG00000018049	*MKNK2*	1'377	1'696	1'575	1'193	1'958	0.716	0.372740352	1
ENSBTAG00000018050	*CDR2*	2'078	1'315	1'659	1'800	1'518	-0.245	0.759986524	1
ENSBTAG00000018052	*PTPRS*	11'661	16'615	14'642	10'099	19'185	0.926	0.227753667	1
ENSBTAG00000018053	*BT.19047*	1'116	1'006	1'064	966	1'162	0.265	0.746416494	1
ENSBTAG00000018057	*BT.74376*	320	277	298	277	320	0.207	0.83242257	1
ENSBTAG00000018058	*C2ORF82*	1	2	2	1	2	1.415	0.981979269	1
ENSBTAG00000018059	*BT.27986*	11	3	6	10	3	-1.459	0.83622464	1
ENSBTAG00000018060	*WDR13*	4'291	3'726	4'009	3'716	4'302	0.211	0.784553696	1
ENSBTAG00000018061	*BT.50207*	1'321	750	1'005	1'144	866	-0.402	0.627281232	1
ENSBTAG00000018062	*KIAA1522*	845	1'845	1'431	732	2'130	1.542	0.060242614	1
ENSBTAG00000018063	*SPATA2*	1'479	1'294	1'388	1'281	1'494	0.222	0.782697824	1
ENSBTAG00000018064	*MGC134574*	16	15	16	14	17	0.322	0.940397404	1
ENSBTAG00000018065	*YARS*	2'266	2'551	2'454	1'962	2'946	0.586	0.456320846	1
ENSBTAG00000018067	*S100PBP*	2'666	1'609	2'083	2'309	1'858	-0.313	0.6926145	1
ENSBTAG00000018069	*TNFSF15*	8	2	5	7	2	-1.585	0.87391615	1
ENSBTAG00000018070	*SCNM1*	854	743	799	740	858	0.214	0.799194216	1
ENSBTAG00000018071	*TMOD4*	148	130	139	128	150	0.228	0.849651928	1
ENSBTAG00000018072	*FARS2*	453	591	537	392	682	0.799	0.364775763	1
ENSBTAG00000018073	*TSPO*	1'084	956	1'021	939	1'104	0.234	0.776474434	1
ENSBTAG00000018074	*VPS72*	1'385	1'393	1'404	1'199	1'608	0.423	0.599265454	1
ENSBTAG00000018077	*BT.64740*	3'505	5'061	4'440	3'035	5'844	0.945	0.224752442	1
ENSBTAG00000018080	*pseudogene*	7	2	4	6	2	-1.392	0.903936064	1
ENSBTAG00000018082	*BT.27138*	132	52	87	114	60	-0.929	0.502849844	1
ENSBTAG00000018084	*GATSL3*	1'187	1'151	1'179	1'028	1'329	0.371	0.64908868	1
ENSBTAG00000018086	*TBC1D10A*	1'101	1'983	1'622	953	2'290	1.264	0.118457001	1
ENSBTAG00000018088	*SETBP1*	308	397	363	267	458	0.781	0.40323867	1
ENSBTAG00000018090	*ZNF449*	507	424	464	439	490	0.157	0.86211767	1
ENSBTAG00000018093	*MLL*	861	721	789	746	833	0.159	0.850597899	1
ENSBTAG00000018094	*ZNF689*	310	324	321	268	374	0.479	0.615987219	1
ENSBTAG00000018095	*protein_coding*	1	0	0	1	0		1	1
ENSBTAG00000018097	*PARN*	1'429	1'217	1'321	1'238	1'405	0.183	0.820584296	1
ENSBTAG00000018098	*pseudogene*	792	760	782	686	878	0.356	0.673049814	1
ENSBTAG00000018101	*AHCY*	5'900	10'971	8'889	5'110	12'668	1.310	0.0916557	1
ENSBTAG00000018103	*HMGB1*	7'071	8'154	7'770	6'124	9'415	0.621	0.419114942	1
ENSBTAG00000018105	*ACVR2B*	68	81	76	59	94	0.667	0.647057737	1
ENSBTAG00000018106	*BT.51755*	13	1	6	11	1	-3.285	0.663144098	1
ENSBTAG00000018108	*PXN*	1'016	1'848	1'507	880	2'134	1.278	0.115874699	1
ENSBTAG00000018112	*TBL1X*	2'049	2'482	2'320	1'774	2'866	0.692	0.380821168	1
ENSBTAG00000018114	*ACVR2A*	3'140	2'299	2'687	2'719	2'655	-0.035	0.965830607	1
ENSBTAG00000018115	*PEBP1*	10'374	7'507	8'826	8'984	8'668	-0.052	0.947039357	1
ENSBTAG00000018116	*MTP18*	412	415	418	357	479	0.426	0.640799444	1
ENSBTAG00000018119	*BT.64791*	171	103	134	148	119	-0.316	0.794859618	1
ENSBTAG00000018123	*FBLN5*	9'629	8'595	9'132	8'339	9'925	0.251	0.74223091	1
ENSBTAG00000018126	*VPS53*	1'185	761	952	1'026	879	-0.224	0.788289677	1
ENSBTAG00000018127	*PPM1A*	1'762	1'019	1'351	1'526	1'177	-0.375	0.643496828	1
ENSBTAG00000018130	*MAP2*	874	487	660	757	562	-0.429	0.618515508	1
ENSBTAG00000018131	*ATF1*	2'665	2'037	2'330	2'308	2'352	0.027	0.972182083	1
ENSBTAG00000018133	*SEMA3A*	1'191	283	679	1'031	327	-1.658	0.057723951	1
ENSBTAG00000018134	*BT.28934*	115	39	72	100	45	-1.145	0.44349636	1
ENSBTAG00000018138	*BT.91577*	302	191	241	262	221	-0.246	0.811663486	1
ENSBTAG00000018142	*DTL*	611	593	607	529	685	0.372	0.667629616	1
ENSBTAG00000018143	*SARG*	219	280	256	190	323	0.770	0.441448845	1
ENSBTAG00000018146	*VAT1L*	291	273	284	252	315	0.323	0.742193729	1
ENSBTAG00000018147	*NXPH2*	231	48	128	200	55	-1.852	0.133373449	1

Ensembl gene ID	geneName	counts wildtype horn bud	counts polled horn bud	baseMean	baseMean wildtype horn bud	baseMean polled horn bud	log2FoldChange	pval	padj
ENSBTAG00000018151	*HELQ*	127	80	101	110	92	-0.252	0.853671678	1
ENSBTAG00000018152	*MYADM*	2'984	4'783	4'054	2'584	5'523	1.096	0.161224304	1
ENSBTAG00000018153	*NRBP1*	8'252	7'844	8'102	7'146	9'057	0.342	0.655095738	1
ENSBTAG00000018155	*MRPS18C*	704	387	528	610	447	-0.448	0.612391314	1
ENSBTAG00000018156	*BT.49091*	450	284	359	390	328	-0.249	0.792587185	1
ENSBTAG00000018157	*IFT172*	3'082	1'978	2'477	2'669	2'284	-0.225	0.775628705	1
ENSBTAG00000018158	*TMEM139*	25	19	22	22	22	0.019	1	1
ENSBTAG00000018159	*BT.103474*	862	1'577	1'284	747	1'821	1.286	0.116932877	1
ENSBTAG00000018160	*LAMA1*	2'048	1'374	1'680	1'774	1'587	-0.161	0.841532476	1
ENSBTAG00000018161	*TBX18*	586	1'055	863	507	1'218	1.263	0.134705544	1
ENSBTAG00000018162	*BOSTAUV1R416*	2	0	1	2	0		0.974934741	1
ENSBTAG00000018164	*BT.32315*	891	875	891	772	1'010	0.389	0.640363176	1
ENSBTAG00000018165	*FAM65B*	605	216	387	524	249	-1.071	0.249646699	1
ENSBTAG00000018167	*KLHL31*	7	35	23	6	40	2.737	0.31146065	1
ENSBTAG00000018169	*ITGB4*	3'822	8'642	6'644	3'310	9'979	1.592	0.042483577	1
ENSBTAG00000018170	*BT.53506*	0	4	2	0	5	Inf	0.817359038	1
ENSBTAG00000018172	*C17ORF101*	241	282	267	209	326	0.642	0.517053452	1
ENSBTAG00000018175	*HEXDC*	1'438	1'187	1'308	1'245	1'371	0.138	0.864354828	1
ENSBTAG00000018176	*C19H17orf62*	2'486	2'687	2'628	2'153	3'103	0.527	0.501453582	1
ENSBTAG00000018178	*NARF*	2'331	2'294	2'334	2'019	2'649	0.392	0.618256587	1
ENSBTAG00000018180	*BT.54238*	4	4	4	3	5	0.415	0.994759363	1
ENSBTAG00000018181	*CCDC8*	5'174	8'866	7'359	4'481	10'238	1.192	0.12458734	1
ENSBTAG00000018185	*ASB4*	1	8	5	1	9	3.415	0.697043054	1
ENSBTAG00000018186	*PDXK*	381	736	590	330	850	1.365	0.120942145	1
ENSBTAG00000018188	*NDUFC2*	3'976	2'704	3'283	3'443	3'122	-0.141	0.857043629	1
ENSBTAG00000018189	*CSTB*	555	935	780	481	1'080	1.168	0.169895735	1
ENSBTAG00000018192	*BT.104280*	1'245	805	1'004	1'078	930	-0.214	0.796582928	1
ENSBTAG00000018193	*BT.32041*	5	6	6	4	7	0.678	0.946418607	1
ENSBTAG00000018196	*BT.69492*	3'688	2'303	2'927	3'194	2'659	-0.264	0.735970016	1
ENSBTAG00000018198	*TLR9*	21	36	30	18	42	1.193	0.59898772	1
ENSBTAG00000018199	*BT.63811*	506	403	452	438	465	0.087	0.924965316	1
ENSBTAG00000018200	*IL15*	32	8	18	28	9	-1.585	0.604898832	1
ENSBTAG00000018201	*DAK*	711	1'178	988	616	1'360	1.143	0.169892892	1
ENSBTAG00000018202	*CYBASC3*	788	705	748	682	814	0.254	0.763824256	1
ENSBTAG00000018203	*CD101*	44	22	32	38	25	-0.585	0.798419738	1
ENSBTAG00000018205	*PHC1*	2'143	2'639	2'452	1'856	3'047	0.715	0.363881424	1
ENSBTAG00000018206	*NIPAL2*	1'430	1'077	1'241	1'238	1'244	0.006	0.994523392	1
ENSBTAG00000018207	*M6PR*	3'783	2'949	3'341	3'276	3'405	0.056	0.94259404	1
ENSBTAG00000018211	*pseudogene*	3	0	1	3	0		0.936647693	1
ENSBTAG00000018213	*LRRC35*	1'327	691	974	1'149	798	-0.526	0.52548856	1
ENSBTAG00000018214	*SHISA2*	574	581	584	497	671	0.433	0.619083772	1
ENSBTAG00000018216	*SKA1*	205	161	182	178	186	0.066	0.955744711	1
ENSBTAG00000018218	*BT.104311*	2	0	1	2	0		0.974934741	1
ENSBTAG00000018220	*RGSL1*	51	10	28	44	12	-1.935	0.420820066	1
ENSBTAG00000018223	*BT.60417*	596	256	406	516	296	-0.804	0.382009134	1
ENSBTAG00000018224	*GLP1R*	5	6	6	4	7	0.678	0.946418607	1
ENSBTAG00000018227	*BT.36774*	2'137	734	1'349	1'851	848	-1.127	0.167391415	1
ENSBTAG00000018229	*NFIX*	3'402	4'859	4'278	2'946	5'611	0.929	0.232779069	1
ENSBTAG00000018232	*BT.37393*	126	97	111	109	112	0.038	0.98350404	1
ENSBTAG00000018235	*SLC45A2*	141	112	126	122	129	0.083	0.95146597	1
ENSBTAG00000018236	*EPB41L5*	675	616	648	585	711	0.283	0.742053602	1
ENSBTAG00000018238	*ABTB1*	5'068	3'083	3'974	4'389	3'560	-0.302	0.69755074	1
ENSBTAG00000018239	*DNAJC27*	580	287	417	502	331	-0.600	0.51208747	1
ENSBTAG00000018240	*BT.65407*	154	435	318	133	502	1.913	0.051187957	1
ENSBTAG00000018242	*protein_coding*	30	34	33	26	39	0.596	0.788374291	1
ENSBTAG00000018244	*VSTM2B*	47	10	26	41	12	-1.818	0.46480839	1
ENSBTAG00000018246	*RAB39A*	21	11	15	18	13	-0.518	0.894018059	1
ENSBTAG00000018247	*OCRL*	2'342	1'832	2'072	2'028	2'115	0.061	0.938688967	1
ENSBTAG00000018248	*MGLL*	411	252	323	356	291	-0.291	0.762842647	1
ENSBTAG00000018249	*KCNN3*	94	44	66	81	51	-0.680	0.662834413	1
ENSBTAG00000018250	*CLEC19A*	10	10	10	9	12	0.415	0.946590905	1
ENSBTAG00000018252	*ARRDC4*	3'492	1'385	2'312	3'024	1'599	-0.919	0.246320004	1
ENSBTAG00000018254	*CDKN1B*	4'207	2'383	3'198	3'643	2'752	-0.405	0.604190581	1
ENSBTAG00000018255	*ACTN1*	5'725	7'521	6'821	4'958	8'685	0.809	0.29449	1
ENSBTAG00000018256	*SMC6*	1'519	1'101	1'293	1'315	1'271	-0.049	0.953181986	1
ENSBTAG00000018257	*APIP*	737	878	826	638	1'014	0.668	0.426506184	1
ENSBTAG00000018258	*SRSF2*	22'150	9'268	14'942	19'182	10'702	-0.842	0.272435571	1
ENSBTAG00000018260	*BT.74349*	595	329	448	515	380	-0.440	0.627389324	1
ENSBTAG00000018261	*PDHX*	1'025	701	849	888	809	-0.133	0.875395457	1
ENSBTAG00000018263	*WAC*	5'772	2'898	4'173	4'999	3'346	-0.579	0.456064315	1
ENSBTAG00000018268	*UCN*	4	3	3	3	3	0.000	1	1
ENSBTAG00000018269	*MPV17*	2'240	1'135	1'625	1'940	1'311	-0.566	0.480551369	1

Ensembl gene ID	geneName	counts wildtype horn bud	counts polled horn bud	baseMean	baseMean wildtype horn bud	baseMean polled horn bud	log2FoldChange	pval	padj
ENSBTAG00000018270	*NFATC2*	250	143	191	217	165	-0.391	0.71849002	1
ENSBTAG00000018271	*BT.103064*	4'984	7'217	6'325	4'316	8'333	0.949	0.22009172	1
ENSBTAG00000018272	*RERE*	1'550	1'978	1'813	1'342	2'284	0.767	0.336568409	1
ENSBTAG00000018274	*FADD*	380	448	423	329	517	0.653	0.473501727	1
ENSBTAG00000018275	*pseudogene*	327	423	386	283	488	0.786	0.395215035	1
ENSBTAG00000018278	*ATP5O*	7'183	6'213	6'697	6'221	7'174	0.206	0.788229044	1
ENSBTAG00000018279	*SPATA5L1*	1'183	558	834	1'025	644	-0.669	0.426080123	1
ENSBTAG00000018280	*SLC28A3*	191	86	132	165	99	-0.736	0.538926471	1
ENSBTAG00000018281	*GPX7*	12'975	6'582	9'418	11'237	7'600	-0.564	0.462386731	1
ENSBTAG00000018282	*NIF3L1*	495	320	399	429	370	-0.214	0.818057436	1
ENSBTAG00000018283	*ACAA1*	2'401	2'890	2'708	2'079	3'337	0.682	0.384614101	1
ENSBTAG00000018284	*KBTBD11*	135	68	98	117	79	-0.574	0.667223292	1
ENSBTAG00000018285	*VAMP5*	945	441	664	818	509	-0.684	0.426232464	1
ENSBTAG00000018286	*RNF181*	1'180	748	943	1'022	864	-0.243	0.771063092	1
ENSBTAG00000018287	*SLC25A48*	5	1	3	4	1	-1.907	0.9188325	1
ENSBTAG00000018289	*TMEM150A*	643	598	624	557	691	0.310	0.719325477	1
ENSBTAG00000018290	*IL9*	1	0	0	1	0		1	1
ENSBTAG00000018291	*C11H2orf68*	1'425	977	1'181	1'234	1'128	-0.129	0.875246507	1
ENSBTAG00000018292	*MOSPD2*	737	426	565	638	492	-0.376	0.668538074	1
ENSBTAG00000018294	*DNASE1L3*	264	86	164	229	99	-1.203	0.285711582	1
ENSBTAG00000018295	*SEC23B*	2'601	1'959	2'257	2'253	2'262	0.006	0.993798748	1
ENSBTAG00000018297	*PLEKHH1*	81	32	54	70	37	-0.925	0.587593244	1
ENSBTAG00000018299	*BT.61756*	312	149	221	270	172	-0.651	0.530410863	1
ENSBTAG00000018300	*TNFRSF19*	425	535	493	368	618	0.747	0.401719337	1
ENSBTAG00000018310	*SETD3*	2'148	1'276	1'667	1'860	1'473	-0.336	0.674588131	1
ENSBTAG00000018312	*PFN1*	2'805	4'751	3'958	2'429	5'486	1.175	0.133750561	1
ENSBTAG00000018313	*MBNL2*	4'437	2'960	3'630	3'843	3'418	-0.169	0.828658866	1
ENSBTAG00000018314	*MED11*	1'111	699	885	962	807	-0.253	0.762382458	1
ENSBTAG00000018315	*PELP1*	1'937	2'824	2'469	1'677	3'261	0.959	0.225197166	1
ENSBTAG00000018316	*ZMYND15*	349	355	356	302	410	0.440	0.638896044	1
ENSBTAG00000018317	*PPP2R2A*	3'168	1'700	2'353	2'744	1'963	-0.483	0.540416619	1
ENSBTAG00000018318	*DNAJB5*	542	518	534	469	598	0.350	0.691237916	1
ENSBTAG00000018320	*RPLP1*	60'776	52'310	56'518	52'634	60'402	0.199	0.792884504	1
ENSBTAG00000018321	*BT.68053*	1'362	1'354	1'371	1'180	1'563	0.407	0.614298629	1
ENSBTAG00000018322	*SEC24A*	5'652	2'573	3'933	4'895	2'971	-0.720	0.355035274	1
ENSBTAG00000018324	*BT.47704*	74	47	59	64	54	-0.240	0.891898233	1
ENSBTAG00000018329	*EXOSC7*	1'424	1'222	1'322	1'233	1'411	0.194	0.810025248	1
ENSBTAG00000018330	*BT.25691*	55	45	50	48	52	0.126	0.953639922	1
ENSBTAG00000018331	*CLEC3B*	4'591	1'557	2'887	3'976	1'798	-1.145	0.147269527	1
ENSBTAG00000018332	*PPTC7*	128	123	126	111	142	0.358	0.770054691	1
ENSBTAG00000018334	*PLAGL2*	184	288	246	159	333	1.061	0.294434241	1
ENSBTAG00000018339	*ATP5G1*	2'307	1'727	1'996	1'998	1'994	-0.003	0.998707442	1
ENSBTAG00000018340	*processed_pseudogene*	387	561	491	335	648	0.951	0.287404151	1
ENSBTAG00000018342	*BT.59823*	210	216	216	182	249	0.456	0.661859431	1
ENSBTAG00000018343	*NCR3*	113	59	83	98	68	-0.522	0.714371262	1
ENSBTAG00000018345	*FBXO39*	1	0	0	1	0		1	1
ENSBTAG00000018347	*IL33*	1'437	972	1'183	1'244	1'122	-0.149	0.856348633	1
ENSBTAG00000018348	*CBLL1*	568	295	416	492	341	-0.530	0.562561046	1
ENSBTAG00000018349	*IFI30*	857	632	736	742	730	-0.024	0.979623596	1
ENSBTAG00000018352	*ABRA*	2	32	19	2	37	4.415	0.183803022	1
ENSBTAG00000018356	*SYT15*	16	4	9	14	5	-1.585	0.756102726	1
ENSBTAG00000018358	*STAC3*	146	285	228	126	329	1.380	0.183018573	1
ENSBTAG00000018360	*MRPL1*	1'223	707	938	1'059	816	-0.376	0.651680879	1
ENSBTAG00000018361	*BT.61431*	3'260	2'290	2'734	2'823	2'644	-0.094	0.904995263	1
ENSBTAG00000018362	*TMEM109*	4'610	5'128	4'957	3'992	5'921	0.569	0.461709467	1
ENSBTAG00000018363	*RBM48*	727	554	635	630	640	0.023	0.980065897	1
ENSBTAG00000018364	*TMEM132A*	2'337	2'917	2'696	2'024	3'368	0.735	0.349592279	1
ENSBTAG00000018366	*SLC15A3*	196	181	189	170	209	0.300	0.781956938	1
ENSBTAG00000018367	*CD6*	9	7	8	8	8	0.052	1	1
ENSBTAG00000018369	*BT.105634*	157	465	336	136	537	1.982	0.041723328	1
ENSBTAG00000018372	*STARD7*	4'225	5'287	4'882	3'659	6'105	0.739	0.340325622	1
ENSBTAG00000018373	*DPYSL2*	12'117	5'382	8'354	10'494	6'215	-0.756	0.326285131	1
ENSBTAG00000018374	*WASF2*	8'117	10'811	9'756	7'030	12'483	0.829	0.281090052	1
ENSBTAG00000018379	*BT.102972*	1'056	1'189	1'144	915	1'373	0.586	0.473253057	1
ENSBTAG00000018381	*CRLF3*	1'316	909	1'095	1'140	1'050	-0.119	0.886345454	1
ENSBTAG00000018382	*BT.87402*	682	499	583	591	576	-0.036	0.970169614	1
ENSBTAG00000018383	*ATAD5*	488	294	381	423	339	-0.316	0.73483545	1
ENSBTAG00000018385	*DNAJC8*	1'233	1'154	1'200	1'068	1'333	0.320	0.69444841	1
ENSBTAG00000018386	*BT.22404*	516	435	475	447	502	0.169	0.851430574	1
ENSBTAG00000018387	*BT.38451*	672	1'024	882	582	1'182	1.023	0.222805268	1
ENSBTAG00000018390	*GLB1L2*	37	40	39	32	46	0.528	0.793946889	1
ENSBTAG00000018394	*BT.35781*	95	109	104	82	126	0.613	0.635725307	1

Ensembl gene ID	geneName	counts wildtype horn bud	counts polled horn bud	baseMean	baseMean wildtype horn bud	baseMean polled horn bud	log2FoldChange	pval	padj
ENSBTAG00000018395	*KIAA1217*	2'408	1'900	2'140	2'085	2'194	0.073	0.925995135	1
ENSBTAG00000018397	*HHLA2*	14	24	20	12	28	1.193	0.679489814	1
ENSBTAG00000018398	*GPR4*	98	67	81	85	77	-0.134	0.934423334	1
ENSBTAG00000018399	*MYH15*	52	45	48	45	52	0.206	0.916729313	1
ENSBTAG00000018400	*BRMS1L*	1'624	926	1'238	1'406	1'069	-0.395	0.627438968	1
ENSBTAG00000018401	*APLF*	894	480	664	774	554	-0.482	0.57496358	1
ENSBTAG00000018402	*BT.70150*	2'393	912	1'563	2'072	1'053	-0.977	0.226352189	1
ENSBTAG00000018403	*ARHGAP9*	54	61	59	47	70	0.591	0.718490951	1
ENSBTAG00000018404	*PRKG1*	244	62	141	211	72	-1.562	0.187808739	1
ENSBTAG00000018405	*MARS*	3'355	2'744	3'037	2'906	3'168	0.125	0.872348203	1
ENSBTAG00000018406	*CSTF2T*	2'810	2'046	2'398	2'434	2'363	-0.043	0.957898106	1
ENSBTAG00000018408	*SNX14*	4'352	2'111	3'103	3'769	2'438	-0.629	0.42182463	1
ENSBTAG00000018410	*EBPL*	1'165	564	830	1'009	651	-0.632	0.452615644	1
ENSBTAG00000018413	*FAM46B*	417	487	462	361	562	0.639	0.476992497	1
ENSBTAG00000018415	*TTLL4*	1'251	1'448	1'378	1'083	1'672	0.626	0.438461728	1
ENSBTAG00000018416	*CCDC42*	3	4	4	3	5	0.830	0.962490925	1
ENSBTAG00000018417	*PSMF1*	1'146	1'573	1'404	992	1'816	0.872	0.281550785	1
ENSBTAG00000018418	*TMEM74B*	0	1	1	0	1	Inf	0.993540919	1
ENSBTAG00000018419	*ADCY4*	1'323	1'147	1'235	1'146	1'324	0.209	0.796787148	1
ENSBTAG00000018420	*POLG2*	867	382	596	751	441	-0.767	0.378594009	1
ENSBTAG00000018421	*WWP2*	8'687	5'979	7'214	7'523	6'904	-0.124	0.872469735	1
ENSBTAG00000018422	*BT.27723*	469	187	311	406	216	-0.912	0.344874756	1
ENSBTAG00000018423	*BT.64934*	38'065	20'949	28'578	32'965	24'190	-0.447	0.557916712	1
ENSBTAG00000018424	*CXCR7*	2'773	1'192	1'889	2'401	1'376	-0.803	0.31428481	1
ENSBTAG00000018425	*PSMD7*	3'290	2'397	2'809	2'849	2'768	-0.042	0.95851198	1
ENSBTAG00000018426	*BT.21335*	655	510	578	567	589	0.054	0.951873523	1
ENSBTAG00000018427	*NCK1*	2'162	1'335	1'707	1'872	1'542	-0.280	0.726072281	1
ENSBTAG00000018430	*PION*	314	99	193	272	114	-1.250	0.246681137	1
ENSBTAG00000018431	*NAV2*	947	823	885	820	950	0.213	0.798744565	1
ENSBTAG00000018433	*FAM116A*	773	599	681	669	692	0.047	0.957127995	1
ENSBTAG00000018435	*BT.74488*	1	1	1	1	1	0.415	1	1
ENSBTAG00000018436	*UBE2J1*	867	437	628	751	505	-0.573	0.507612143	1
ENSBTAG00000018437	*SMC5*	2'656	1'272	1'884	2'300	1'469	-0.647	0.416725791	1
ENSBTAG00000018438	*RRAGD*	267	188	224	231	217	-0.091	0.934685024	1
ENSBTAG00000018440	*BT.76061*	1	0	0	1	0		1	1
ENSBTAG00000018442	*PRSS37*	2	0	1	2	0		0.974934741	1
ENSBTAG00000018445	*GRIN3A*	147	21	76	127	24	-2.392	0.114548682	1
ENSBTAG00000018446	*GCA*	70	11	37	61	13	-2.255	0.282094046	1
ENSBTAG00000018447	*BT.20263*	3'685	1'698	2'576	3'191	1'961	-0.703	0.372276588	1
ENSBTAG00000018448	*MSH4*	65	32	47	56	37	-0.607	0.742470823	1
ENSBTAG00000018449	*NUMA1*	12'846	24'051	19'448	11'125	27'772	1.320	0.087595057	1
ENSBTAG00000018451	*PTMS*	6'709	7'240	7'085	5'810	8'360	0.525	0.494345688	1
ENSBTAG00000018452	*ANKRA2*	1'144	570	824	991	658	-0.590	0.483062275	1
ENSBTAG00000018453	*ANKRD37*	245	148	192	212	171	-0.312	0.774372491	1
ENSBTAG00000018454	*BT.20434*	1'345	889	1'096	1'165	1'027	-0.182	0.825293568	1
ENSBTAG00000018455	*COMMD5*	1'158	981	1'068	1'003	1'133	0.176	0.830521312	1
ENSBTAG00000018456	*ZNF7*	981	859	921	850	992	0.223	0.787923177	1
ENSBTAG00000018460	*ADAMTS16*	260	422	356	225	487	1.114	0.237041944	1
ENSBTAG00000018463	*VIM*	167'643	187'607	180'907	145'183	216'630	0.577	0.447034569	1
ENSBTAG00000018464	*ZDHHC14*	352	577	486	305	666	1.128	0.209000943	1
ENSBTAG00000018467	*C14ORF45*	34	28	31	29	32	0.135	0.966128827	1
ENSBTAG00000018469	*BT.98765*	717	639	679	621	738	0.249	0.771212337	1
ENSBTAG00000018471	*SSR3*	5'809	3'107	4'309	5'031	3'588	-0.488	0.529587109	1
ENSBTAG00000018473	*MARCO*	3	2	2	3	2	-0.170	1	1
ENSBTAG00000018474	*BT.68623*	345	292	318	299	337	0.174	0.857004737	1
ENSBTAG00000018478	*RPL7L1*	5'111	3'855	4'439	4'426	4'451	0.008	0.991227214	1
ENSBTAG00000018479	*PTPMT1*	815	858	848	706	991	0.489	0.558583998	1
ENSBTAG00000018481	*protein_coding*	58	11	31	50	13	-1.984	0.378313094	1
ENSBTAG00000018482	*BT.68679*	1'045	718	867	905	829	-0.126	0.881472597	1
ENSBTAG00000018483	*NDUFS3*	2'176	1'792	1'977	1'884	2'069	0.135	0.864542747	1
ENSBTAG00000018487	*CCDC19*	47	3	22	41	3	-3.555	0.230250583	1
ENSBTAG00000018488	*AFF1*	743	497	609	643	574	-0.165	0.850915688	1
ENSBTAG00000018489	*OTOL1*	2	0	1	2	0		0.974934741	1
ENSBTAG00000018493	*C14H8orf47*	44	159	111	38	184	2.268	0.082084923	1
ENSBTAG00000018495	*NKX6-3*	1	0	0	1	0		1	1
ENSBTAG00000018496	*BT.79452*	1'318	1'304	1'324	1'141	1'506	0.400	0.621071102	1
ENSBTAG00000018497	*SDPR*	1'896	557	1'143	1'642	643	-1.352	0.102744809	1
ENSBTAG00000018498	*PQBP1*	1'610	1'817	1'746	1'394	2'098	0.590	0.459824197	1
ENSBTAG00000018499	*ANXA8*	1'456	1'626	1'569	1'261	1'878	0.574	0.473749373	1
ENSBTAG00000018501	*KIAA0947*	1'423	1'379	1'412	1'232	1'592	0.370	0.64609428	1
ENSBTAG00000018502	*ARMC9*	795	667	729	688	770	0.162	0.849290568	1
ENSBTAG00000018506	*protein_coding*	879	1'322	1'144	761	1'527	1.004	0.222032571	1

Ensembl gene ID	geneName	counts wildtype horn bud	counts polled horn bud	baseMean	baseMean wildtype horn bud	baseMean polled horn bud	log2FoldChange	pval	padj
ENSBTAG00000018508	*TMEM108*	315	119	205	273	137	-0.989	0.35006263	1
ENSBTAG00000018509	*ETNK2*	280	389	346	242	449	0.889	0.345907452	1
ENSBTAG00000018513	*BT.23401*	8'202	4'903	6'382	7'103	5'661	-0.327	0.671007049	1
ENSBTAG00000018517	*VLDLR*	2'654	959	1'703	2'298	1'107	-1.054	0.190537756	1
ENSBTAG00000018518	*RASL10B*	204	200	204	177	231	0.386	0.715121937	1
ENSBTAG00000018519	*GAS2L2*	28	11	18	24	13	-0.933	0.765131313	1
ENSBTAG00000018520	*BT.35472*	235	70	142	204	81	-1.332	0.25795869	1
ENSBTAG00000018522	*BT.97611*	18'404	14'933	16'591	15'938	17'243	0.114	0.880904631	1
ENSBTAG00000018523	*TRIM38*	820	625	716	710	722	0.023	0.979291415	1
ENSBTAG00000018524	*AMHR2*	88	38	60	76	44	-0.796	0.623435436	1
ENSBTAG00000018527	*BT.20582*	1'533	1'055	1'273	1'328	1'218	-0.124	0.879845598	1
ENSBTAG00000018528	*BT.61674*	100	56	76	87	65	-0.421	0.778359268	1
ENSBTAG00000018530	*TUBA8*	106	122	116	92	141	0.618	0.619772571	1
ENSBTAG00000018531	*IGJ*	471	168	301	408	194	-1.072	0.270599269	1
ENSBTAG00000018534	*DGCR14*	580	989	822	502	1'142	1.185	0.161709999	1
ENSBTAG00000018535	*CDK5RAP1*	1'263	593	889	1'094	685	-0.676	0.418894101	1
ENSBTAG00000018538	*CEP70*	974	457	686	844	528	-0.677	0.429883762	1
ENSBTAG00000018539	*KLK1*	35	35	35	30	40	0.415	0.849137879	1
ENSBTAG00000018540	*NOX4*	89	112	103	77	129	0.747	0.564595938	1
ENSBTAG00000018542	*COX5B*	4'900	3'539	4'165	4'244	4'086	-0.054	0.944958295	1
ENSBTAG00000018543	*VIL1*	14	11	12	12	13	0.067	1	1
ENSBTAG00000018545	*KLK15*	0	2	1	0	2	Inf	0.939077559	1
ENSBTAG00000018546	*LRBA*	1'800	1'919	1'887	1'559	2'216	0.507	0.52285584	1
ENSBTAG00000018548	*INTS7*	1'535	1'581	1'577	1'329	1'826	0.458	0.567620127	1
ENSBTAG00000018549	*SPAG16*	24	9	16	21	10	-1.000	0.775206422	1
ENSBTAG00000018552	*VWC2L*	3	0	1	3	0		0.936647693	1
ENSBTAG00000018554	*protein_coding*	0	1	1	0	1	Inf	0.993540919	1
ENSBTAG00000018555	*SFXN5*	263	115	180	228	133	-0.778	0.476544573	1
ENSBTAG00000018557	*BT.75826*	6'784	4'770	5'692	5'875	5'508	-0.093	0.904576048	1
ENSBTAG00000018559	*LYRM1*	193	115	150	167	133	-0.332	0.776448351	1
ENSBTAG00000018560	*BT.63231*	119	98	108	103	113	0.135	0.921695645	1
ENSBTAG00000018562	*TMEM159*	2'043	1'204	1'580	1'769	1'390	-0.348	0.664997229	1
ENSBTAG00000018564	*ZFYVE27*	1'980	1'719	1'850	1'715	1'985	0.211	0.790185937	1
ENSBTAG00000018566	*SFRP5*	271	57	150	235	66	-1.834	0.118012988	1
ENSBTAG00000018569	*CUL4B*	1'698	1'841	1'798	1'471	2'126	0.532	0.504182986	1
ENSBTAG00000018570	*SDR16C5*	2'305	1'681	1'969	1'996	1'941	-0.040	0.960690062	1
ENSBTAG00000018571	*IL1RL1*	31	9	19	27	10	-1.369	0.653625596	1
ENSBTAG00000018572	*BT.28173*	50	6	25	43	7	-2.644	0.312062188	1
ENSBTAG00000018575	*TRHDE*	151	105	126	131	121	-0.109	0.935591428	1
ENSBTAG00000018577	*SLC7A1*	364	503	448	315	581	0.882	0.329755881	1
ENSBTAG00000018578	*LOXL3*	1'225	265	683	1'061	306	-1.794	0.04074039	1
ENSBTAG00000018579	*DOK1*	489	366	423	423	423	-0.003	1	1
ENSBTAG00000018581	*BT.29459*	3'206	1'956	2'518	2'776	2'259	-0.298	0.705238751	1
ENSBTAG00000018585	*GABRB2*	42	43	43	36	50	0.449	0.816926186	1
ENSBTAG00000018588	*TMBIM6*	22'352	13'689	17'582	19'357	15'807	-0.292	0.701800415	1
ENSBTAG00000018589	*RFC2*	1'647	1'672	1'679	1'426	1'931	0.437	0.584159649	1
ENSBTAG00000018590	*STRN*	346	275	309	300	318	0.084	0.933201302	1
ENSBTAG00000018593	*PSENEN*	2'783	2'402	2'592	2'410	2'774	0.203	0.795696297	1
ENSBTAG00000018594	*LIN37*	589	478	531	510	552	0.114	0.89834915	1
ENSBTAG00000018596	*PTPN21*	1'796	1'518	1'654	1'555	1'753	0.172	0.828915742	1
ENSBTAG00000018598	*HSPB6*	7'175	5'051	6'023	6'214	5'832	-0.091	0.906272472	1
ENSBTAG00000018599	*C19ORF55*	296	349	330	256	403	0.653	0.492003679	1
ENSBTAG00000018600	*VGLL4*	1'851	1'652	1'755	1'603	1'908	0.251	0.752414639	1
ENSBTAG00000018604	*SEMA4G*	612	861	762	530	994	0.908	0.284861276	1
ENSBTAG00000018607	*TSPEAR*	414	849	669	359	980	1.451	0.095106477	1
ENSBTAG00000018613	*BT.25395*	3'478	1'951	2'632	3'012	2'253	-0.419	0.593891818	1
ENSBTAG00000018616	*IPO11*	2'833	1'987	2'374	2'453	2'294	-0.097	0.903282455	1
ENSBTAG00000018622	*PCBP4*	5'832	6'357	6'196	5'051	7'340	0.539	0.483385632	1
ENSBTAG00000018625	*ZNF263*	1'578	911	1'209	1'367	1'052	-0.378	0.643651068	1
ENSBTAG00000018628	*RPL29*	31'913	39'062	36'371	27'637	45'105	0.707	0.353824142	1
ENSBTAG00000018629	*BT.65669*	1'693	892	1'248	1'466	1'030	-0.509	0.531526346	1
ENSBTAG00000018631	*BT.48904*	4'759	3'932	4'331	4'121	4'540	0.140	0.85626975	1
ENSBTAG00000018633	*RABAC1*	8'031	6'031	6'960	6'955	6'964	0.002	0.997610006	1
ENSBTAG00000018634	*SH3BGRL2*	195	279	246	169	322	0.932	0.356661234	1
ENSBTAG00000018636	*BT.21198*	1'082	826	945	937	954	0.026	0.976107238	1
ENSBTAG00000018637	*HDDC3*	348	356	356	301	411	0.448	0.632608186	1
ENSBTAG00000018638	*CC2D2A*	1'109	877	987	960	1'013	0.076	0.926714896	1
ENSBTAG00000018639	*UNC45A*	3'068	4'633	4'003	2'657	5'350	1.010	0.196090128	1
ENSBTAG00000018642	*RCCD1*	295	253	274	255	292	0.193	0.846270534	1
ENSBTAG00000018643	*BT.20428*	1'237	1'343	1'311	1'071	1'551	0.534	0.509828438	1
ENSBTAG00000018644	*PDZRN3*	4'394	3'281	3'797	3'805	3'789	-0.006	0.99453764	1
ENSBTAG00000018646	*ATP6V1B2*	4'053	3'271	3'644	3'510	3'777	0.106	0.891242901	1

Ensembl gene ID	geneName	counts wildtype horn bud	counts polled horn bud	baseMean	baseMean wildtype horn bud	baseMean polled horn bud	log2FoldChange	pval	padj
ENSBTAG00000018647	*BT.40271*	107	39	69	93	45	-1.041	0.494672737	1
ENSBTAG00000018652	*CXCL17*	5	16	11	4	18	2.093	0.61950395	1
ENSBTAG00000018653	*OBFC2A*	512	201	338	443	232	-0.934	0.325813746	1
ENSBTAG00000018655	*C7H19orf10*	2'613	2'861	2'783	2'263	3'304	0.546	0.485738335	1
ENSBTAG00000018656	*HP1BP3*	10'481	8'296	9'328	9'077	9'579	0.078	0.918482537	1
ENSBTAG00000018657	*ATP13A5*	56	28	40	48	32	-0.585	0.769556011	1
ENSBTAG00000018658	*TNRC6C*	1'490	1'026	1'238	1'290	1'185	-0.123	0.880899965	1
ENSBTAG00000018660	*PPP6R2*	2'431	2'806	2'673	2'105	3'240	0.622	0.427975277	1
ENSBTAG00000018661	*TMC6*	555	809	707	481	934	0.959	0.262518996	1
ENSBTAG00000018663	*GPRC5C*	1'189	1'151	1'179	1'030	1'329	0.368	0.651216477	1
ENSBTAG00000018667	*RCL1*	465	477	477	403	551	0.452	0.613246879	1
ENSBTAG00000018669	*PLB1*	156	74	110	135	85	-0.661	0.604229883	1
ENSBTAG00000018671	*DCUN1D2*	558	201	358	483	232	-1.058	0.261574175	1
ENSBTAG00000018673	*BT.91974*	154	106	128	133	122	-0.124	0.925298432	1
ENSBTAG00000018674	*BT.48999*	1'335	1'126	1'228	1'156	1'300	0.169	0.83486358	1
ENSBTAG00000018680	*BT.44977*	790	463	609	684	535	-0.356	0.682467875	1
ENSBTAG00000018682	*SETD4*	374	268	317	324	309	-0.066	0.949396309	1
ENSBTAG00000018689	*BT.25483*	1'552	1'623	1'609	1'344	1'874	0.480	0.548859642	1
ENSBTAG00000018690	*RPAP3*	2'088	1'218	1'607	1'808	1'406	-0.363	0.651376899	1
ENSBTAG00000018691	*RHOU*	3'245	1'402	2'215	2'810	1'619	-0.796	0.315519951	1
ENSBTAG00000018694	*BT.104098*	2'036	1'178	1'562	1'763	1'360	-0.374	0.641315711	1
ENSBTAG00000018697	*BT.90677*	1'268	800	1'011	1'098	924	-0.249	0.763435416	1
ENSBTAG00000018699	*MYST2*	3'657	2'614	3'093	3'167	3'018	-0.069	0.930192897	1
ENSBTAG00000018704	*BTG3*	3'414	1'715	2'468	2'957	1'980	-0.578	0.463087883	1
ENSBTAG00000018705	*BT.46702*	1'752	2'313	2'094	1'517	2'671	0.816	0.303986792	1
ENSBTAG00000018706	*PCTP*	580	400	482	502	462	-0.121	0.894906154	1
ENSBTAG00000018708	*UTP14A*	1'216	1'726	1'523	1'053	1'993	0.920	0.253952771	1
ENSBTAG00000018709	*CATSPERB*	46	13	27	40	15	-1.408	0.558417298	1
ENSBTAG00000018710	*TTF1*	1'654	853	1'209	1'432	985	-0.540	0.507879195	1
ENSBTAG00000018711	*DENND4B*	2'415	2'428	2'448	2'091	2'804	0.423	0.590369602	1
ENSBTAG00000018713	*protein_coding*	15	34	26	13	39	1.596	0.514464627	1
ENSBTAG00000018715	*C4H7ORF11*	574	338	444	497	390	-0.349	0.700977184	1
ENSBTAG00000018717	*DDX20*	2'925	1'565	2'170	2'533	1'807	-0.487	0.538124418	1
ENSBTAG00000018718	*DDI2*	109	79	93	94	91	-0.049	0.980668122	1
ENSBTAG00000018722	*PLEKHM2*	3'201	4'421	3'939	2'772	5'105	0.881	0.25847233	1
ENSBTAG00000018723	*SLC25A34*	281	338	317	243	390	0.681	0.476440123	1
ENSBTAG00000018724	*TMEM82*	26	35	31	23	40	0.844	0.704200107	1
ENSBTAG00000018725	*FBLIM1*	4'250	3'666	3'957	3'681	4'233	0.202	0.794123652	1
ENSBTAG00000018726	*RIMS3*	56	35	44	48	40	-0.263	0.898081272	1
ENSBTAG00000018727	*GOLIM4*	8'590	5'840	7'091	7'439	6'743	-0.142	0.854320488	1
ENSBTAG00000018729	*SPEN*	3'184	2'338	2'729	2'757	2'700	-0.031	0.970082817	1
ENSBTAG00000018730	*ZBTB17*	1'257	1'656	1'500	1'089	1'912	0.813	0.313290054	1
ENSBTAG00000018731	*FAM172A*	66	58	62	57	67	0.229	0.892760157	1
ENSBTAG00000018735	*C13H20orf27*	2'290	2'134	2'224	1'983	2'464	0.313	0.690810438	1
ENSBTAG00000018737	*GLMN*	1'306	778	1'015	1'131	898	-0.332	0.687874081	1
ENSBTAG00000018739	*RPAP2*	635	338	470	550	390	-0.495	0.582077952	1
ENSBTAG00000018742	*MTCH2*	2'145	1'503	1'797	1'858	1'736	-0.098	0.903161936	1
ENSBTAG00000018743	*C5H12orf29*	1'126	656	866	975	757	-0.364	0.6636488	1
ENSBTAG00000018744	*MGAT5*	241	346	304	209	400	0.937	0.332660185	1
ENSBTAG00000018745	*CEP290*	2'378	946	1'576	2'059	1'092	-0.915	0.256493243	1
ENSBTAG00000018746	*BT.73998*	2'354	1'214	1'720	2'039	1'402	-0.540	0.499254151	1
ENSBTAG00000018747	*PRKAA2*	43	46	45	37	53	0.512	0.78450387	1
ENSBTAG00000018749	*NUDT16*	1'052	829	934	911	957	0.071	0.931990268	1
ENSBTAG00000018752	*SUZ12*	1'079	635	834	934	733	-0.350	0.677452923	1
ENSBTAG00000018753	*TMEM163*	233	88	152	202	102	-0.990	0.389162212	1
ENSBTAG00000018759	*pseudogene*	1	3	2	1	3	2.000	0.933774563	1
ENSBTAG00000018761	*MGC139367*	1'423	1'433	1'444	1'232	1'655	0.425	0.597135408	1
ENSBTAG00000018762	*SLC22A12*	1	0	0	1	0		1	1
ENSBTAG00000018765	*SEMA5B*	905	622	751	784	718	-0.126	0.883677604	1
ENSBTAG00000018767	*RTP3*	61	55	58	53	64	0.266	0.877792824	1
ENSBTAG00000018768	*HMGN1*	16'489	11'328	13'680	14'280	13'080	-0.127	0.868899314	1
ENSBTAG00000018770	*HSDL1*	3'477	2'004	2'663	3'011	2'314	-0.380	0.628642903	1
ENSBTAG00000018771	*BT.53537*	15	6	10	13	7	-0.907	0.858439879	1
ENSBTAG00000018772	*BT.66017*	1'115	1'287	1'226	966	1'486	0.622	0.444677654	1
ENSBTAG00000018773	*RND1*	125	15	63	108	17	-2.644	0.110296954	1
ENSBTAG00000018774	*NUAK2*	148	119	133	128	137	0.100	0.938278442	1
ENSBTAG00000018775	*BT.28741*	1'994	1'812	1'910	1'727	2'092	0.277	0.726712649	1
ENSBTAG00000018777	*ADCY5*	445	709	602	385	819	1.087	0.213026663	1
ENSBTAG00000018778	*TMC1*	4	3	3	3	3	0.000	1	1
ENSBTAG00000018783	*TH1L*	3'388	2'859	3'118	2'934	3'301	0.170	0.826873044	1
ENSBTAG00000018784	*CTSZ*	2'707	1'014	1'758	2'344	1'171	-1.002	0.212236282	1
ENSBTAG00000018785	*BT.29383*	68	35	50	59	40	-0.543	0.762940501	1

Ensembl gene ID	geneName	counts wildtype horn bud	counts polled horn bud	baseMean	baseMean wildtype horn bud	baseMean polled horn bud	log2FoldChange	pval	padj
ENSBTAG00000018787	*GCM2*	0	1	1	0	1	Inf	0.993540919	1
ENSBTAG00000018792	*SLC22A11*	0	5	3	0	6	Inf	0.757530434	1
ENSBTAG00000018795	*BT.20671*	939	428	654	813	494	-0.718	0.404543463	1
ENSBTAG00000018796	*RABGGTA*	826	867	858	715	1'001	0.485	0.561617094	1
ENSBTAG00000018799	*GPSM1*	3'966	4'565	4'353	3'435	5'271	0.618	0.425209959	1
ENSBTAG00000018801	*SNX3*	8'756	6'150	7'342	7'583	7'101	-0.095	0.902598793	1
ENSBTAG00000018802	*UBE2Z*	4'838	4'547	4'720	4'190	5'250	0.326	0.672989961	1
ENSBTAG00000018803	*SNF8*	2'180	1'935	2'061	1'888	2'234	0.243	0.758252936	1
ENSBTAG00000018804	*BT.105016*	1'250	2'418	1'937	1'083	2'792	1.367	0.089426667	1
ENSBTAG00000018805	*ASB13*	102	101	102	88	117	0.401	0.760386163	1
ENSBTAG00000018806	*PSRC1*	1'176	1'337	1'281	1'018	1'544	0.600	0.459476184	1
ENSBTAG00000018807	*MYBPHL*	62	28	43	54	32	-0.732	0.701650784	1
ENSBTAG00000018808	*SLC25A35*	83	85	85	72	98	0.449	0.749656328	1
ENSBTAG00000018809	*BT.26835*	335	343	343	290	396	0.449	0.633959385	1
ENSBTAG00000018810	*THBS2*	22'748	18'003	20'244	19'700	20'788	0.078	0.918212298	1
ENSBTAG00000018811	*BT.61953*	9	8	9	8	9	0.245	0.986825286	1
ENSBTAG00000018812	*BT.49005*	5'403	3'992	4'644	4'679	4'610	-0.022	0.978669584	1
ENSBTAG00000018813	*IDH3B*	3'758	3'290	3'527	3'255	3'799	0.223	0.77354725	1
ENSBTAG00000018817	*BT.106485*	20	20	20	17	23	0.415	0.896464675	1
ENSBTAG00000018819	*ODZ1*	200	116	154	173	134	-0.371	0.748469768	1
ENSBTAG00000018823	*GRN*	6'687	6'840	6'845	5'791	7'898	0.448	0.559840653	1
ENSBTAG00000018824	*BT.55630*	12'528	8'118	10'112	10'850	9'374	-0.211	0.783332373	1
ENSBTAG00000018825	*SSH1*	310	448	393	268	517	0.946	0.305818019	1
ENSBTAG00000018828	*ATN1*	10'846	13'514	12'499	9'393	15'605	0.732	0.339055786	1
ENSBTAG00000018829	*SPRYD7*	352	213	275	305	246	-0.310	0.755656847	1
ENSBTAG00000018831	*DAO*	2	0	1	2	0		0.974934741	1
ENSBTAG00000018833	*BT.25377*	662	226	417	573	261	-1.135	0.217104902	1
ENSBTAG00000018834	*PPP1R37*	2'487	3'168	2'906	2'154	3'658	0.764	0.329789992	1
ENSBTAG00000018837	*SLC39A11*	536	271	389	464	313	-0.569	0.538866651	1
ENSBTAG00000018838	*protein_coding*	42	36	39	36	42	0.193	0.933528612	1
ENSBTAG00000018841	*PTPN3*	433	336	381	375	388	0.049	0.95992568	1
ENSBTAG00000018843	*SERPINA1*	23	9	15	20	10	-0.939	0.794031165	1
ENSBTAG00000018845	*DND1*	100	70	84	87	81	-0.100	0.9531452	1
ENSBTAG00000018846	*CLN5*	738	458	584	639	529	-0.273	0.755210771	1
ENSBTAG00000018847	*HARS*	2'153	1'346	1'709	1'865	1'554	-0.263	0.742928749	1
ENSBTAG00000018848	*FBXL3*	4'673	2'142	3'260	4'047	2'473	-0.710	0.363835066	1
ENSBTAG00000018851	*MYCBP2*	5'544	2'492	3'839	4'801	2'878	-0.739	0.343292885	1
ENSBTAG00000018852	*BT.25770*	5'397	2'083	3'540	4'674	2'405	-0.958	0.221030585	1
ENSBTAG00000018854	*SOD1*	2'327	2'637	2'530	2'015	3'045	0.595	0.448620655	1
ENSBTAG00000018855	*SCAF4*	2'051	1'224	1'595	1'776	1'413	-0.330	0.681366717	1
ENSBTAG00000018857	*RAB4A*	683	488	577	591	563	-0.070	0.938717117	1
ENSBTAG00000018859	*BT.56371*	205	266	242	178	307	0.791	0.434762278	1
ENSBTAG00000018861	*C12ORF56*	84	48	64	73	55	-0.392	0.808740024	1
ENSBTAG00000018862	*BT.39759*	408	315	359	353	364	0.042	0.966822801	1
ENSBTAG00000018863	*CCDC111*	758	310	507	656	358	-0.875	0.32597	1
ENSBTAG00000018864	*MLF1IP*	706	336	500	611	388	-0.656	0.461443245	1
ENSBTAG00000018868	*METTL9*	2'814	1'942	2'340	2'437	2'242	-0.120	0.879855759	1
ENSBTAG00000018869	*IGSF6*	90	30	56	78	35	-1.170	0.482236639	1
ENSBTAG00000018870	*OTOA*	61	53	57	53	61	0.212	0.905645201	1
ENSBTAG00000018872	*BT.89433*	30	24	27	26	28	0.093	0.98570819	1
ENSBTAG00000018873	*TTPAL*	224	150	184	194	173	-0.163	0.885185394	1
ENSBTAG00000018877	*PDIA5*	2'208	1'328	1'723	1'912	1'533	-0.318	0.690529432	1
ENSBTAG00000018879	*ZNF451*	505	345	418	437	398	-0.135	0.885570303	1
ENSBTAG00000018881	*SYT6*	44	1	20	38	1	-5.044	0.157825826	1
ENSBTAG00000018883	*DCTN3*	2'909	1'938	2'379	2'519	2'238	-0.171	0.828961077	1
ENSBTAG00000018884	*RING1*	4'084	3'975	4'063	3'537	4'590	0.376	0.627133587	1
ENSBTAG00000018887	*IPO13*	1'741	2'405	2'142	1'508	2'777	0.881	0.266939145	1
ENSBTAG00000018888	*DPH2*	907	762	833	785	880	0.164	0.845379476	1
ENSBTAG00000018889	*ATP6V0B*	3'729	2'634	3'135	3'229	3'041	-0.086	0.912685834	1
ENSBTAG00000018893	*BT.37868*	6'917	3'837	5'210	5'990	4'431	-0.435	0.573470011	1
ENSBTAG00000018894	*PEX11G*	839	650	739	727	751	0.047	0.956921616	1
ENSBTAG00000018897	*PSMD11*	1'895	1'693	1'798	1'641	1'955	0.252	0.750727391	1
ENSBTAG00000018898	*ARID4A*	1'789	769	1'219	1'549	888	-0.803	0.325770127	1
ENSBTAG00000018900	*RLTPR*	93	47	67	81	54	-0.570	0.714188625	1
ENSBTAG00000018902	*BT.63240*	460	304	375	398	351	-0.183	0.846995876	1
ENSBTAG00000018903	*ACD*	692	777	748	599	897	0.582	0.491798651	1
ENSBTAG00000018904	*BT.30029*	270	315	299	234	364	0.637	0.510170968	1
ENSBTAG00000018905	*MAN2A2*	1'639	1'924	1'821	1'419	2'222	0.646	0.417138587	1
ENSBTAG00000018906	*C16ORF86*	59	74	68	51	85	0.742	0.626306468	1
ENSBTAG00000018908	*GFOD2*	638	823	751	553	950	0.782	0.356352715	1
ENSBTAG00000018909	*BT.41832*	66	189	138	57	218	1.933	0.108232802	1
ENSBTAG00000018910	*WDR6*	7'054	10'651	9'204	6'109	12'299	1.010	0.19069492	1

Ensembl gene ID	geneName	counts wildtype horn bud	counts polled horn bud	baseMean	baseMean wildtype horn bud	baseMean polled horn bud	log2FoldChange	pval	padj
ENSBTAG00000018912	*ARHGEF1*	3'676	5'019	4'489	3'184	5'795	0.864	0.26612886	1
ENSBTAG00000018913	*DALRD3*	629	612	626	545	707	0.376	0.663451079	1
ENSBTAG00000018914	*RAB25*	694	565	627	601	652	0.118	0.891754531	1
ENSBTAG00000018915	*BT.20323*	5'330	3'509	4'334	4'616	4'052	-0.188	0.808762917	1
ENSBTAG00000018918	*NDUFAF3*	1'243	948	1'086	1'076	1'095	0.024	0.977020535	1
ENSBTAG00000018920	*TRIM47*	1'166	611	858	1'010	706	-0.517	0.537075722	1
ENSBTAG00000018921	*USP19*	4'553	5'604	5'207	3'943	6'471	0.715	0.355454992	1
ENSBTAG00000018922	*RG9MTD3*	1'123	604	835	973	697	-0.480	0.568014222	1
ENSBTAG00000018924	*QRICH1*	4'937	4'302	4'622	4'276	4'968	0.216	0.778891924	1
ENSBTAG00000018925	*EXOSC3*	1'070	468	734	927	540	-0.778	0.361117542	1
ENSBTAG00000018928	*QARS*	7'948	10'537	9'525	6'883	12'167	0.822	0.285042493	1
ENSBTAG00000018935	*SETMAR*	992	704	836	859	813	-0.080	0.926236474	1
ENSBTAG00000018936	*LSS*	1'879	1'822	1'866	1'627	2'104	0.371	0.640541339	1
ENSBTAG00000018937	*HMOX2*	1'547	1'582	1'583	1'340	1'827	0.447	0.576285709	1
ENSBTAG00000018938	*C25H16orf5*	1'523	2'016	1'823	1'319	2'328	0.820	0.304599742	1
ENSBTAG00000018940	*EFCAB4B*	8	36	24	7	42	2.585	0.324256329	1
ENSBTAG00000018941	*CPXM2*	1'559	2'808	2'296	1'350	3'242	1.264	0.113009589	1
ENSBTAG00000018942	*SSX2IP*	1'000	802	896	866	926	0.097	0.907990793	1
ENSBTAG00000018945	*AMOTL1*	1'655	1'666	1'679	1'433	1'924	0.425	0.59465195	1
ENSBTAG00000018946	*FAM129C*	215	156	183	186	180	-0.048	0.971238584	1
ENSBTAG00000018947	*SYT16*	2	2	2	2	2	0.415	1	1
ENSBTAG00000018948	*HIF3A*	3'413	3'528	3'515	2'956	4'074	0.463	0.551547474	1
ENSBTAG00000018951	*TBC1D9*	843	778	814	730	898	0.299	0.721454268	1
ENSBTAG00000018954	*BT.104357*	2'795	1'787	2'242	2'421	2'063	-0.230	0.771122108	1
ENSBTAG00000018955	*SOHLH2*	5	3	4	4	3	-0.322	1	1
ENSBTAG00000018959	*RAB11A*	4'060	2'553	3'232	3'516	2'948	-0.254	0.744962726	1
ENSBTAG00000018960	*protein_coding*	122	69	93	106	80	-0.407	0.767652997	1
ENSBTAG00000018965	*PRDM16*	124	23	67	107	27	-2.016	0.199788846	1
ENSBTAG00000018966	*PLCE1*	514	92	276	445	106	-2.067	0.041989825	1
ENSBTAG00000018967	*YIPF3*	7'368	7'621	7'590	6'381	8'800	0.464	0.545394006	1
ENSBTAG00000018969	*POLR1C*	1'024	880	951	887	1'016	0.196	0.812634975	1
ENSBTAG00000018970	*XPO5*	1'718	1'704	1'728	1'488	1'968	0.403	0.61276617	1
ENSBTAG00000018971	*RAD51B*	301	241	269	261	278	0.094	0.926853058	1
ENSBTAG00000018972	*GARS*	5'581	3'074	4'191	4'833	3'550	-0.445	0.566107219	1
ENSBTAG00000018973	*COPS3*	3'740	3'072	3'393	3'239	3'547	0.131	0.865681751	1
ENSBTAG00000018975	*KCNT1*	214	151	180	185	174	-0.088	0.941621877	1
ENSBTAG00000018978	*CSRP2BP*	1'204	1'841	1'584	1'043	2'126	1.028	0.202623785	1
ENSBTAG00000018979	*PUM1*	6'670	3'792	5'078	5'776	4'379	-0.400	0.605225322	1
ENSBTAG00000018981	*BT.78544*	5	4	4	4	5	0.093	1	1
ENSBTAG00000018984	*PIK3CD*	259	174	213	224	201	-0.159	0.883679847	1
ENSBTAG00000018986	*ACSL4*	532	367	442	461	424	-0.121	0.896787394	1
ENSBTAG00000018987	*RPS25*	43'915	46'046	45'600	38'032	53'169	0.483	0.524507255	1
ENSBTAG00000018991	*TSGA10*	201	81	134	174	94	-0.896	0.45274622	1
ENSBTAG00000018993	*ITGB7*	603	312	441	522	360	-0.536	0.554959941	1
ENSBTAG00000018994	*BT.20790*	82	31	53	71	36	-0.988	0.56240051	1
ENSBTAG00000018996	*BT.61689*	45	33	39	39	38	-0.032	1	1
ENSBTAG00000018999	*MGRN1*	6'855	7'551	7'328	5'937	8'719	0.555	0.47028018	1
ENSBTAG00000019001	*PRDM10*	258	128	186	223	148	-0.596	0.583030415	1
ENSBTAG00000019002	*BT.37186*	378	231	297	327	267	-0.295	0.763029908	1
ENSBTAG00000019006	*WDR24*	850	1'241	1'085	736	1'433	0.961	0.24379455	1
ENSBTAG00000019007	*protein_coding*	2	0	1	2	0		0.974934741	1
ENSBTAG00000019010	*BT.10192*	1'125	840	972	974	970	-0.006	0.995966546	1
ENSBTAG00000019011	*PGM1*	2'095	2'153	2'150	1'814	2'486	0.454	0.564842463	1
ENSBTAG00000019012	*KCTD16*	5	5	5	4	6	0.415	0.985023983	1
ENSBTAG00000019013	*BT.32841*	3'038	3'329	3'237	2'631	3'844	0.547	0.482926486	1
ENSBTAG00000019014	*BT.66399*	2'473	1'392	1'875	2'142	1'607	-0.414	0.60305857	1
ENSBTAG00000019015	*IFITM3*	2'829	2'606	2'730	2'450	3'009	0.297	0.704408513	1
ENSBTAG00000019017	*IFITM2*	1'652	707	1'124	1'431	816	-0.809	0.324679353	1
ENSBTAG00000019018	*BT.88795*	1'439	2'070	1'818	1'246	2'390	0.940	0.240196631	1
ENSBTAG00000019020	*TAX1BP1*	10'717	4'619	7'307	9'281	5'334	-0.799	0.300249681	1
ENSBTAG00000019023	*NANS*	1'170	1'330	1'275	1'013	1'536	0.600	0.459766812	1
ENSBTAG00000019024	*JAZF1*	946	1'690	1'385	819	1'951	1.252	0.125005386	1
ENSBTAG00000019025	*NDUFA11*	1'964	1'781	1'879	1'701	2'057	0.274	0.729768981	1
ENSBTAG00000019026	*BT.55356*	1'625	634	1'070	1'407	732	-0.943	0.253780103	1
ENSBTAG00000019027	*SLC30A7*	596	355	463	516	410	-0.332	0.712847496	1
ENSBTAG00000019028	*STOX1*	88	14	46	76	16	-2.237	0.231454343	1
ENSBTAG00000019029	*DPH5*	1'530	1'117	1'307	1'325	1'290	-0.039	0.963412243	1
ENSBTAG00000019031	*BT.51728*	79	8	39	68	9	-2.889	0.167492805	1
ENSBTAG00000019036	*ARHGAP28*	910	911	920	788	1'052	0.417	0.615771769	1
ENSBTAG00000019039	*ZNF239*	914	492	680	792	568	-0.478	0.57693352	1
ENSBTAG00000019040	*PLBD2*	2'390	3'265	2'920	2'070	3'770	0.865	0.270604097	1
ENSBTAG00000019041	*BT.66729*	48	14	29	42	16	-1.363	0.559836341	1

Ensembl gene ID	geneName	counts wildtype horn bud	counts polled horn bud	baseMean	baseMean wildtype horn bud	baseMean polled horn bud	log2FoldChange	pval	padj
ENSBTAG00000019043	*GABPA*	1'017	598	786	881	691	-0.351	0.678118527	1
ENSBTAG00000019044	*BAIAP2*	2'785	3'673	3'327	2'412	4'241	0.814	0.297627421	1
ENSBTAG00000019045	*COMMD9*	1'118	1'219	1'188	968	1'408	0.540	0.507676348	1
ENSBTAG00000019048	*processed_pseudogene*	97	228	174	84	263	1.648	0.139984792	1
ENSBTAG00000019049	*AATK*	635	530	581	550	612	0.154	0.860147032	1
ENSBTAG00000019051	*NAT14*	797	722	762	690	834	0.272	0.747165954	1
ENSBTAG00000019052	*ANK3*	3'170	1'897	2'468	2'745	2'190	-0.326	0.679251846	1
ENSBTAG00000019053	*MAP9*	185	73	122	160	84	-0.927	0.450890333	1
ENSBTAG00000019054	*EPSTI1*	309	262	285	268	303	0.177	0.858222075	1
ENSBTAG00000019058	*IQSEC3*	21	1	10	18	1	-3.977	0.462631882	1
ENSBTAG00000019059	*ATG16L2*	399	492	457	346	568	0.717	0.42563802	1
ENSBTAG00000019061	*BT.53461*	1'190	1'817	1'564	1'031	2'098	1.026	0.203806932	1
ENSBTAG00000019062	*FCHSD2*	1'072	660	845	928	762	-0.285	0.734955964	1
ENSBTAG00000019065	*EEPD1*	759	1'112	971	657	1'284	0.966	0.245328567	1
ENSBTAG00000019067	*TMC8*	306	488	414	265	563	1.088	0.235996137	1
ENSBTAG00000019069	*SYNGR2*	836	997	938	724	1'151	0.669	0.420355138	1
ENSBTAG00000019070	*PMP22*	11'678	11'438	11'660	10'113	13'207	0.385	0.613830699	1
ENSBTAG00000019071	*COMMD10*	1'417	980	1'179	1'227	1'132	-0.117	0.887443323	1
ENSBTAG00000019072	*PSD4*	193	365	294	167	421	1.334	0.173403671	1
ENSBTAG00000019073	*TRAF7*	5'685	5'978	5'913	4'923	6'903	0.488	0.52645217	1
ENSBTAG00000019075	*MAP1D*	573	309	427	496	357	-0.476	0.601961138	1
ENSBTAG00000019076	*PAK6*	835	958	915	723	1'106	0.613	0.460847527	1
ENSBTAG00000019077	*DAD1*	7'594	6'404	6'986	6'577	7'395	0.169	0.825015123	1
ENSBTAG00000019078	*BT.76072*	9	11	10	8	13	0.705	0.889330664	1
ENSBTAG00000019079	*PLCB2*	227	135	176	197	156	-0.335	0.763595183	1
ENSBTAG00000019080	*PTH*	3	0	1	3	0		0.936647693	1
ENSBTAG00000019082	*RAD51D*	779	905	860	675	1'045	0.631	0.45026044	1
ENSBTAG00000019085	*SRP54*	3'348	2'020	2'616	2'899	2'332	-0.314	0.689706815	1
ENSBTAG00000019086	*TELO2*	749	583	661	649	673	0.054	0.951300627	1
ENSBTAG00000019090	*PLEKHH3*	2'097	1'887	1'997	1'816	2'179	0.263	0.739642971	1
ENSBTAG00000019091	*RNPC3*	2'498	906	1'605	2'163	1'046	-1.048	0.194083198	1
ENSBTAG00000019092	*BT.54236*	2	1	1	2	1	-0.585	1	1
ENSBTAG00000019093	*BT.20890*	3'287	925	1'957	2'847	1'068	-1.414	0.079232813	1
ENSBTAG00000019094	*NLE1*	666	787	743	577	909	0.656	0.43927484	1
ENSBTAG00000019095	*CCR10*	13	23	19	11	27	1.238	0.677916987	1
ENSBTAG00000019096	*BT.23164*	4'209	6'820	5'760	3'645	7'875	1.111	0.152857698	1
ENSBTAG00000019097	*CNTNAP1*	1'095	939	1'016	948	1'084	0.193	0.814536671	1
ENSBTAG00000019099	*SLC25A37*	850	827	846	736	955	0.375	0.653468347	1
ENSBTAG00000019104	*C17ORF70*	505	1'024	810	437	1'182	1.435	0.09245415	1
ENSBTAG00000019105	*NPLOC4*	1'961	2'935	2'544	1'698	3'389	0.997	0.207270847	1
ENSBTAG00000019106	*EIF1B*	3'012	3'196	3'149	2'608	3'690	0.501	0.520936982	1
ENSBTAG00000019107	*GAS7*	1'149	1'414	1'314	995	1'633	0.714	0.378420263	1
ENSBTAG00000019110	*BT.70581*	3'034	2'070	2'509	2'628	2'390	-0.137	0.862938435	1
ENSBTAG00000019115	*CDC42EP4*	3'145	3'930	3'631	2'724	4'538	0.737	0.344590098	1
ENSBTAG00000019116	*MOBKL3*	2'414	1'114	1'688	2'091	1'286	-0.701	0.381918592	1
ENSBTAG00000019119	*ZCRB1*	449	505	486	389	583	0.585	0.512028288	1
ENSBTAG00000019120	*WDHD1*	1'332	1'132	1'230	1'154	1'307	0.180	0.824348886	1
ENSBTAG00000019121	*IFT122*	2'215	2'406	2'348	1'918	2'778	0.534	0.49735401	1
ENSBTAG00000019122	*EFCAB5*	108	33	66	94	38	-1.295	0.405228733	1
ENSBTAG00000019123	*MEFV*	37	18	26	32	21	-0.624	0.806604612	1
ENSBTAG00000019124	*EIF4EBP2*	565	648	619	489	748	0.613	0.478476995	1
ENSBTAG00000019125	*SLC1A1*	564	130	319	488	150	-1.702	0.081176376	1
ENSBTAG00000019129	*BT.19064*	211	207	211	183	239	0.387	0.712039453	1
ENSBTAG00000019130	*FAM149B1*	1'459	1'007	1'213	1'264	1'163	-0.120	0.884349184	1
ENSBTAG00000019133	*ZNF326*	2'985	1'301	2'044	2'585	1'502	-0.783	0.324722527	1
ENSBTAG00000019134	*NEK5*	2	2	2	2	2	0.415	1	1
ENSBTAG00000019136	*BT.76143*	4'497	4'482	4'535	3'895	5'175	0.410	0.595431128	1
ENSBTAG00000019137	*SCN7A*	159	22	82	138	25	-2.438	0.09847453	1
ENSBTAG00000019138	*KIF3C*	1'758	994	1'335	1'522	1'148	-0.408	0.615175189	1
ENSBTAG00000019139	*PHF3*	4'613	3'638	4'098	3'995	4'201	0.072	0.925034913	1
ENSBTAG00000019140	*BT.43608*	4'625	1'793	3'038	4'005	2'070	-0.952	0.225952854	1
ENSBTAG00000019143	*BT.87682*	1	0	0	1	0		1	1
ENSBTAG00000019146	*BT.37641*	123	30	71	107	35	-1.621	0.286638718	1
ENSBTAG00000019147	*RPS20*	62'273	32'895	45'957	53'930	37'984	-0.506	0.506592731	1
ENSBTAG00000019150	*PLD3*	13'014	12'062	12'599	11'270	13'928	0.305	0.688534422	1
ENSBTAG00000019152	*NYAP2*	5	0	2	4	0		0.853268594	1
ENSBTAG00000019153	*JMJD6*	938	921	938	812	1'063	0.389	0.639104076	1
ENSBTAG00000019155	*FRS2*	331	277	303	287	320	0.158	0.871846597	1
ENSBTAG00000019156	*CCT2*	6'732	5'367	6'014	5'830	6'197	0.088	0.908277525	1
ENSBTAG00000019157	*LRRC10*	0	4	2	0	5	Inf	0.817359038	1
ENSBTAG00000019158	*MEX3A*	246	476	381	213	550	1.367	0.144082292	1
ENSBTAG00000019159	*PVALB*	34	0	15	29	0		0.163610325	1

Ensembl gene ID	geneName	counts wildtype horn bud	counts polled horn bud	baseMean	baseMean wildtype horn bud	baseMean polled horn bud	log2FoldChange	pval	padj
ENSBTAG00000019160	*GLOD4*	2'693	1'619	2'101	2'332	1'869	-0.319	0.687295368	1
ENSBTAG00000019161	*NPRL2*	2'008	1'567	1'774	1'739	1'809	0.057	0.942641481	1
ENSBTAG00000019162	*RNMTL1*	333	282	307	288	326	0.175	0.857392228	1
ENSBTAG00000019163	*CYB561D2*	262	191	224	227	221	-0.041	0.973918134	1
ENSBTAG00000019164	*BT.103435*	1'381	1'161	1'268	1'196	1'341	0.165	0.839052205	1
ENSBTAG00000019166	*NSMCE4A*	2'059	1'174	1'569	1'783	1'356	-0.395	0.622502638	1
ENSBTAG00000019167	*INPPL1*	8'515	14'685	12'165	7'374	16'957	1.201	0.12000814	1
ENSBTAG00000019168	*PHOX2A*	5	14	10	4	16	1.900	0.676791734	1
ENSBTAG00000019174	*ZNF710*	676	1'008	875	585	1'164	0.991	0.237367225	1
ENSBTAG00000019177	*BT.54746*	2'716	3'639	3'277	2'352	4'202	0.837	0.284676622	1
ENSBTAG00000019179	*BT.51172*	34	23	28	29	27	-0.149	0.966657603	1
ENSBTAG00000019180	*BT.44458*	3'259	5'814	4'768	2'822	6'713	1.250	0.109972797	1
ENSBTAG00000019181	*BT.105147*	307	402	365	266	464	0.804	0.389242613	1
ENSBTAG00000019182	*TIA1*	6'244	2'705	4'265	5'407	3'123	-0.792	0.308855216	1
ENSBTAG00000019183	*FUZ*	520	411	462	450	475	0.076	0.934474349	1
ENSBTAG00000019184	*TRAPPC2L*	3'296	1'610	2'357	2'854	1'859	-0.619	0.433316258	1
ENSBTAG00000019187	*ZNF462*	1'719	860	1'241	1'489	993	-0.584	0.473402303	1
ENSBTAG00000019188	*BT.59584*	3'753	5'040	4'535	3'250	5'820	0.840	0.279289284	1
ENSBTAG00000019190	*CPO*	1	2	2	1	2	1.415	0.981979269	1
ENSBTAG00000019192	*LHX8*	59	16	35	51	18	-1.468	0.487110667	1
ENSBTAG00000019193	*SETD5*	5'989	4'702	5'308	5'187	5'429	0.066	0.931289356	1
ENSBTAG00000019194	*FAM35B*	697	335	495	604	387	-0.642	0.471734396	1
ENSBTAG00000019197	*FAM208B*	4'901	2'668	3'663	4'244	3'081	-0.462	0.552683517	1
ENSBTAG00000019198	*LRRC42*	2'046	2'132	2'117	1'772	2'462	0.474	0.548156958	1
ENSBTAG00000019202	*EAF1*	1'079	1'170	1'143	934	1'351	0.532	0.515046869	1
ENSBTAG00000019203	*S100A4*	11'504	7'386	9'246	9'963	8'529	-0.224	0.770187494	1
ENSBTAG00000019204	*RTN3*	2'275	1'909	2'087	1'970	2'204	0.162	0.837339228	1
ENSBTAG00000019208	*protein_coding*	672	497	578	582	574	-0.020	0.984514576	1
ENSBTAG00000019209	*RAD1*	1'660	907	1'242	1'438	1'047	-0.457	0.574727493	1
ENSBTAG00000019210	*BT.62494*	1'160	597	847	1'005	689	-0.543	0.517300416	1
ENSBTAG00000019211	*BRIX1*	2'411	1'133	1'698	2'088	1'308	-0.674	0.399723049	1
ENSBTAG00000019212	*KCNS3*	52	30	40	45	35	-0.379	0.856258861	1
ENSBTAG00000019213	*CNNM4*	440	655	569	381	756	0.989	0.259639893	1
ENSBTAG00000019214	*BT.20599*	1'900	1'288	1'566	1'645	1'487	-0.146	0.856810755	1
ENSBTAG00000019215	*THOC1*	3'539	1'488	2'392	3'065	1'718	-0.835	0.291125301	1
ENSBTAG00000019216	*TUFM*	2'739	4'017	3'505	2'372	4'638	0.968	0.216478809	1
ENSBTAG00000019217	*KIF13A*	1'487	1'511	1'516	1'288	1'745	0.438	0.584978174	1
ENSBTAG00000019218	*BT.68276*	3'497	2'236	2'805	3'028	2'582	-0.230	0.769438153	1
ENSBTAG00000019219	*SH2B1*	2'123	2'310	2'253	1'839	2'667	0.537	0.496045869	1
ENSBTAG00000019220	*bta-mir-2882*	4'146	4'553	4'424	3'591	5'257	0.550	0.477277043	1
ENSBTAG00000019221	*protein_coding*	1	1	1	1	1	0.415	1	1
ENSBTAG00000019225	*SLC39A14*	2'862	1'021	1'829	2'479	1'179	-1.072	0.181560012	1
ENSBTAG00000019226	*PAG8*	6	8	7	5	9	0.830	0.903753758	1
ENSBTAG00000019227	*BT.58908*	17	22	20	15	25	0.787	0.789040458	1
ENSBTAG00000019229	*TMEM144*	88	45	64	76	52	-0.553	0.728617583	1
ENSBTAG00000019230	*PTGER3*	213	199	207	184	230	0.317	0.764425489	1
ENSBTAG00000019231	*C2H2orf47*	489	269	367	423	311	-0.447	0.632775049	1
ENSBTAG00000019232	*TYW5*	221	172	195	191	199	0.053	0.964460739	1
ENSBTAG00000019234	*BMP6*	673	413	530	583	477	-0.289	0.744258178	1
ENSBTAG00000019235	*MAGOHB*	1'769	1'412	1'581	1'532	1'630	0.090	0.910661729	1
ENSBTAG00000019237	*TXNDC5*	4'081	3'798	3'960	3'534	4'386	0.311	0.687501668	1
ENSBTAG00000019238	*C8H9ORF156*	566	409	481	490	472	-0.054	0.955252763	1
ENSBTAG00000019241	*FGF11*	1'404	862	1'106	1'216	995	-0.289	0.725459507	1
ENSBTAG00000019242	*CHRNB1*	573	366	459	496	423	-0.232	0.798797115	1
ENSBTAG00000019244	*P2RX6*	116	24	64	100	28	-1.858	0.24363199	1
ENSBTAG00000019246	*SC5DL*	3'249	1'310	2'163	2'814	1'513	-0.895	0.259760087	1
ENSBTAG00000019247	*pseudogene*	6	0	3	5	0		0.81115604	1
ENSBTAG00000019249	*HS3ST6*	174	339	271	151	391	1.377	0.167398221	1
ENSBTAG00000019250	*BTK*	349	133	228	302	154	-0.977	0.343861864	1
ENSBTAG00000019251	*EPB41L3*	5'077	4'001	4'508	4'397	4'620	0.071	0.925948429	1
ENSBTAG00000019252	*MGC159903*	271	381	337	235	440	0.907	0.338949622	1
ENSBTAG00000019254	*C9ORF171*	0	1	1	0	1	Inf	0.993540919	1
ENSBTAG00000019255	*BT.17324*	6'517	3'732	4'977	5'644	4'309	-0.389	0.614833174	1
ENSBTAG00000019256	*GLA*	456	294	367	395	339	-0.218	0.817492339	1
ENSBTAG00000019260	*GLIPR1L2*	2	0	1	2	0		0.974934741	1
ENSBTAG00000019262	*TOP2A*	5'627	5'367	5'535	4'873	6'197	0.347	0.652167034	1
ENSBTAG00000019264	*EDEM3*	472	241	344	409	278	-0.555	0.557619628	1
ENSBTAG00000019265	*AGK*	783	489	621	678	565	-0.264	0.761407537	1
ENSBTAG00000019266	*TDRD12*	36	15	24	31	17	-0.848	0.747030072	1
ENSBTAG00000019267	*BT.69347*	91'089	203'560	156'968	78'885	235'051	1.575	0.041973008	1
ENSBTAG00000019268	*CHMP3*	1	0	0	1	0		1	1
ENSBTAG00000019269	*COL6A2*	58'151	126'624	98'287	50'360	146'213	1.538	0.046948669	1

Ensembl gene ID	geneName	counts wildtype horn bud	counts polled horn bud	baseMean	baseMean wildtype horn bud	baseMean polled horn bud	log2FoldChange	pval	padj
ENSBTAG00000019271	*BT.51668*	6'515	4'394	5'358	5'642	5'074	-0.153	0.843214303	1
ENSBTAG00000019272	*LPCAT2*	244	302	280	211	349	0.723	0.461311755	1
ENSBTAG00000019274	*ATIC*	8'207	4'377	6'081	7'107	5'054	-0.492	0.523614731	1
ENSBTAG00000019275	*SSBIP1*	425	263	336	368	304	-0.277	0.77195098	1
ENSBTAG00000019276	*pseudogene*	246	397	336	213	458	1.106	0.245407847	1
ENSBTAG00000019277	*KCNH3*	208	435	341	180	502	1.479	0.121976463	1
ENSBTAG00000019278	*KNTC1*	485	399	440	420	461	0.133	0.884059048	1
ENSBTAG00000019279	*MLH3*	910	424	639	788	490	-0.687	0.426697178	1
ENSBTAG00000019280	*CRYBB1*	74	77	76	64	89	0.472	0.74771472	1
ENSBTAG00000019281	*MCRS1*	1'736	2'064	1'943	1'503	2'383	0.665	0.402762164	1
ENSBTAG00000019282	*CRYBA4*	42	23	31	36	27	-0.454	0.847674521	1
ENSBTAG00000019283	*FAM186B*	255	159	202	221	184	-0.266	0.804735522	1
ENSBTAG00000019284	*processed_pseudogene*	1'687	1'368	1'520	1'461	1'580	0.113	0.888325631	1
ENSBTAG00000019285	*FBXL17*	1'671	730	1'145	1'447	843	-0.780	0.341939926	1
ENSBTAG00000019287	*FAM81B*	1	0	0	1	0		1	1
ENSBTAG00000019289	*ITGA2*	701	614	658	607	709	0.224	0.794507054	1
ENSBTAG00000019290	*PACSIN2*	2'640	2'169	2'395	2'286	2'505	0.132	0.866851488	1
ENSBTAG00000019291	*BT.36371*	193	56	116	167	65	-1.370	0.276014791	1
ENSBTAG00000019293	*EGR3*	212	201	208	184	232	0.338	0.748686524	1
ENSBTAG00000019294	*ABCC9*	1'601	1'156	1'361	1'387	1'335	-0.055	0.947517775	1
ENSBTAG00000019295	*MDH1*	6'699	6'074	6'408	5'802	7'014	0.274	0.721192858	1
ENSBTAG00000019297	*GFRA1*	3'680	1'374	2'387	3'187	1'587	-1.006	0.204445363	1
ENSBTAG00000019298	*STRADB*	734	483	597	636	558	-0.189	0.829897312	1
ENSBTAG00000019299	*GRHPR*	196	297	256	170	343	1.015	0.311492845	1
ENSBTAG00000019300	*TPMT*	1'411	604	960	1'222	697	-0.809	0.330635361	1
ENSBTAG00000019302	*BT.48090*	116	133	127	100	154	0.612	0.612632103	1
ENSBTAG00000019303	*BT.105395*	161	162	163	139	187	0.424	0.706084003	1
ENSBTAG00000019304	*BTBD3*	680	408	530	589	471	-0.322	0.71641195	1
ENSBTAG00000019305	*EXOG*	77	120	103	67	139	1.055	0.416755834	1
ENSBTAG00000019309	*BT.53810*	64	20	39	55	23	-1.263	0.523664001	1
ENSBTAG00000019310	*FOXK1*	265	647	488	229	747	1.703	0.061227144	1
ENSBTAG00000019312	*TFCP2*	3'788	2'322	2'981	3'281	2'681	-0.291	0.710170375	1
ENSBTAG00000019313	*ZMIZ1*	1'366	1'695	1'570	1'183	1'957	0.726	0.365773469	1
ENSBTAG00000019314	*BT.41700*	1'512	718	1'069	1'309	829	-0.659	0.423355611	1
ENSBTAG00000019315	*PLA2G7*	310	102	193	268	118	-1.189	0.27019629	1
ENSBTAG00000019317	*GPS1*	2'140	2'717	2'495	1'853	3'137	0.759	0.335101349	1
ENSBTAG00000019321	*CCDC57*	668	573	620	579	662	0.194	0.823084618	1
ENSBTAG00000019322	*HABP2*	1	1	1	1	1	0.415	1	1
ENSBTAG00000019325	*BT.21089*	820	1'171	1'031	710	1'352	0.929	0.261328331	1
ENSBTAG00000019329	*LYL1*	135	88	109	117	102	-0.202	0.880322005	1
ENSBTAG00000019330	*BT.23613*	14	3	8	12	3	-1.807	0.758305361	1
ENSBTAG00000019331	*KATNAL2*	64	29	44	55	33	-0.727	0.698783445	1
ENSBTAG00000019332	*SLC9A10*	6	2	4	5	2	-1.170	0.934316631	1
ENSBTAG00000019334	*KLC2*	1'090	1'329	1'239	944	1'535	0.701	0.389082294	1
ENSBTAG00000019336	*DVL3*	3'795	3'854	3'868	3'287	4'450	0.437	0.572760087	1
ENSBTAG00000019337	*ACVR1C*	6	0	3	5	0		0.81115604	1
ENSBTAG00000019338	*IL13RA1*	2'049	3'432	2'869	1'774	3'963	1.159	0.142271688	1
ENSBTAG00000019340	*PCDH9*	176	29	93	152	33	-2.186	0.116022362	1
ENSBTAG00000019341	*BT.31987*	3'558	1'131	2'194	3'081	1'306	-1.238	0.121107884	1
ENSBTAG00000019342	*OSBPL3*	156	233	202	135	269	0.994	0.348150223	1
ENSBTAG00000019343	*MDFI*	2'147	2'209	2'205	1'859	2'551	0.456	0.563032895	1
ENSBTAG00000019345	*RFK*	1'323	623	933	1'146	719	-0.671	0.419931973	1
ENSBTAG00000019348	*protein_coding*	3	25	16	3	29	3.474	0.329808061	1
ENSBTAG00000019349	*SLC6A4*	324	108	203	281	125	-1.170	0.271798891	1
ENSBTAG00000019350	*SLC25A21*	61	31	44	53	36	-0.562	0.76827261	1
ENSBTAG00000019353	*STIM2*	1'272	813	1'020	1'102	939	-0.231	0.780686491	1
ENSBTAG00000019354	*BT.24636*	32	60	48	28	69	1.322	0.455544714	1
ENSBTAG00000019355	*KCNK10*	65	10	34	56	12	-2.285	0.295562758	1
ENSBTAG00000019357	*BT.36519*	188	144	165	163	166	0.030	0.983673682	1
ENSBTAG00000019358	*RNF214*	921	718	813	798	829	0.056	0.947775606	1
ENSBTAG00000019359	*protein_coding*	286	440	378	248	508	1.037	0.26571386	1
ENSBTAG00000019362	*BT.21058*	1'154	936	1'040	999	1'081	0.113	0.891004685	1
ENSBTAG00000019365	*BACE1*	3'584	2'655	3'085	3'104	3'066	-0.018	0.982922852	1
ENSBTAG00000019366	*POLR2B*	7'157	5'329	6'176	6'198	6'153	-0.010	0.990074574	1
ENSBTAG00000019368	*IGFBP7*	11'748	5'611	8'327	10'174	6'479	-0.651	0.397263399	1
ENSBTAG00000019369	*TSPAN4*	2'851	1'929	2'348	2'469	2'227	-0.149	0.851271246	1
ENSBTAG00000019370	*BT.34284*	642	398	508	556	460	-0.275	0.758336297	1
ENSBTAG00000019371	*BT.44383*	4	1	2	3	1	-1.585	0.952247636	1
ENSBTAG00000019373	*CAPRIN2*	1'942	821	1'315	1'682	948	-0.827	0.309330632	1
ENSBTAG00000019375	*SCARF2*	3'454	4'078	3'850	2'991	4'709	0.655	0.399711706	1
ENSBTAG00000019376	*FNDC3B*	2'949	2'432	2'681	2'554	2'808	0.137	0.860851028	1
ENSBTAG00000019378	*KIAA1755*	583	253	399	505	292	-0.789	0.392180472	1

Ensembl gene ID	geneName	counts wildtype horn bud	counts polled horn bud	baseMean	baseMean wildtype horn bud	baseMean polled horn bud	log2FoldChange	pval	padj
ENSBTAG00000019379	*TUSC5*	1	0	0	1	0		1	1
ENSBTAG00000019382	*PKDCC*	3'505	2'657	3'052	3'035	3'068	0.015	0.984014565	1
ENSBTAG00000019386	*BT.91164*	723	397	542	626	458	-0.450	0.609863859	1
ENSBTAG00000019387	*MKI67IP*	2'585	1'784	2'149	2'239	2'060	-0.120	0.880316182	1
ENSBTAG00000019388	*KIAA2013*	1'740	2'405	2'142	1'507	2'777	0.882	0.266499647	1
ENSBTAG00000019390	*CA12*	648	565	607	561	652	0.217	0.8023448	1
ENSBTAG00000019399	*BT.27870*	94	163	135	81	188	1.209	0.30945402	1
ENSBTAG00000019401	*BT.73689*	6	1	3	5	1	-2.170	0.885240772	1
ENSBTAG00000019402	*BT.55869*	96	167	138	83	193	1.214	0.304195299	1
ENSBTAG00000019403	*C4H7ORF30*	583	249	396	505	288	-0.812	0.379078406	1
ENSBTAG00000019404	*IL10RB*	1'014	829	918	878	957	0.124	0.881289431	1
ENSBTAG00000019406	*IGF2BP3*	2'062	1'171	1'569	1'786	1'352	-0.401	0.617414227	1
ENSBTAG00000019407	*NAP1L3*	279	196	234	242	226	-0.094	0.931214666	1
ENSBTAG00000019409	*GNPTAB*	2'589	1'238	1'836	2'242	1'430	-0.649	0.415681169	1
ENSBTAG00000019410	*CEP57L1*	241	93	158	209	107	-0.959	0.398439721	1
ENSBTAG00000019412	*BT.20737*	2'523	2'163	2'341	2'185	2'498	0.193	0.805969873	1
ENSBTAG00000019414	*CLK4*	7'906	2'346	4'778	6'847	2'709	-1.338	0.087928676	1
ENSBTAG00000019417	*ARMCX2*	2'415	1'733	2'046	2'091	2'001	-0.064	0.937091443	1
ENSBTAG00000019418	*UBE3D*	268	126	189	232	145	-0.674	0.53283309	1
ENSBTAG00000019419	*NDUFS7*	1'162	1'261	1'231	1'006	1'456	0.533	0.512028896	1
ENSBTAG00000019421	*DACT1*	2'206	1'382	1'753	1'910	1'596	-0.260	0.745461078	1
ENSBTAG00000019423	*ACSM2A*	0	1	1	0	1	Inf	0.993540919	1
ENSBTAG00000019426	*RNF175*	107	23	60	93	27	-1.803	0.272011329	1
ENSBTAG00000019427	*KIAA0232*	1'193	1'135	1'172	1'033	1'311	0.343	0.673632399	1
ENSBTAG00000019428	*CCR1*	348	147	236	301	170	-0.828	0.418025589	1
ENSBTAG00000019429	*BT.71090*	15	6	10	13	7	-0.907	0.858439879	1
ENSBTAG00000019432	*ICAM2*	150	75	108	130	87	-0.585	0.649205108	1
ENSBTAG00000019434	*PDCD4*	7'253	2'767	4'738	6'281	3'195	-0.975	0.210348407	1
ENSBTAG00000019436	*SLC39A13*	3'984	4'064	4'071	3'450	4'693	0.444	0.56671926	1
ENSBTAG00000019437	*SLFN11*	203	220	215	176	254	0.531	0.610167305	1
ENSBTAG00000019440	*BT.17776*	80	46	61	69	53	-0.383	0.817322998	1
ENSBTAG00000019441	*BT.29898*	877	1'202	1'074	760	1'388	0.870	0.291081545	1
ENSBTAG00000019443	*CTDNEP1*	4'417	4'727	4'642	3'825	5'458	0.513	0.507026442	1
ENSBTAG00000019445	*C7ORF31*	18	7	12	16	8	-0.948	0.827785041	1
ENSBTAG00000019446	*C17ORF81*	3'865	3'876	3'911	3'347	4'476	0.419	0.588642107	1
ENSBTAG00000019447	*NPVF*	11	1	5	10	1	-3.044	0.722857941	1
ENSBTAG00000019448	*CLDN7*	168	138	152	145	159	0.131	0.912961535	1
ENSBTAG00000019451	*TAPT1*	716	629	673	620	726	0.228	0.790112911	1
ENSBTAG00000019453	*PTGES*	728	1'028	909	630	1'187	0.913	0.27443043	1
ENSBTAG00000019454	*ANGEL1*	520	778	674	450	898	0.996	0.246930364	1
ENSBTAG00000019455	*MYO5B*	627	218	397	543	252	-1.109	0.231355797	1
ENSBTAG00000019456	*SPRED2*	849	570	697	735	658	-0.160	0.853350311	1
ENSBTAG00000019457	*MRPL22*	994	521	731	861	602	-0.517	0.543582153	1
ENSBTAG00000019458	*ASB3*	2'732	1'314	1'942	2'366	1'517	-0.641	0.420546435	1
ENSBTAG00000019459	*CHAC2*	159	127	142	138	147	0.091	0.943176261	1
ENSBTAG00000019460	*MOXD1*	1'096	1'948	1'599	949	2'249	1.245	0.124236253	1
ENSBTAG00000019461	*NUMBL*	2'769	3'155	3'021	2'398	3'643	0.603	0.440201993	1
ENSBTAG00000019462	*ADCK4*	523	487	508	453	562	0.312	0.724882575	1
ENSBTAG00000019463	*SLC25A39*	3'640	4'301	4'059	3'152	4'966	0.656	0.39835107	1
ENSBTAG00000019464	*pseudogene*	775	863	834	671	997	0.570	0.496276187	1
ENSBTAG00000019465	*BT.21658*	1'437	1'838	1'683	1'244	2'122	0.770	0.336204905	1
ENSBTAG00000019467	*ACTL7B*	5	1	3	4	1	-1.907	0.9188325	1
ENSBTAG00000019468	*ACTL7A*	4	4	4	3	5	0.415	0.994759363	1
ENSBTAG00000019470	*NEK6*	5'347	2'157	3'561	4'631	2'491	-0.895	0.252784857	1
ENSBTAG00000019471	*CNIH4*	2'884	2'164	2'498	2'498	2'499	0.001	0.99923658	1
ENSBTAG00000019472	*BT.22888*	1'319	1'120	1'218	1'142	1'293	0.179	0.825648867	1
ENSBTAG00000019473	*IGLON5*	501	478	493	434	552	0.347	0.696489184	1
ENSBTAG00000019474	*BT.103700*	4'785	2'416	3'467	4'144	2'790	-0.571	0.464285056	1
ENSBTAG00000019478	*BT.64229*	141	136	140	122	157	0.363	0.759053889	1
ENSBTAG00000019479	*TATDN2*	632	1'067	890	547	1'232	1.171	0.163790563	1
ENSBTAG00000019480	*PDE6D*	1'040	891	965	901	1'029	0.192	0.816599988	1
ENSBTAG00000019486	*CLDN12*	941	800	869	815	924	0.181	0.828665058	1
ENSBTAG00000019488	*USP6NL*	731	427	563	633	493	-0.361	0.681395857	1
ENSBTAG00000019490	*protein_coding*	284	506	415	246	584	1.248	0.175301873	1
ENSBTAG00000019492	*FANCE*	509	322	406	441	372	-0.246	0.790935322	1
ENSBTAG00000019494	*RPL10A*	14'960	25'029	20'928	12'956	28'901	1.158	0.132567284	1
ENSBTAG00000019495	*TEAD3*	3'398	6'106	4'997	2'943	7'051	1.261	0.106825737	1
ENSBTAG00000019496	*CDKN3*	164	97	127	142	112	-0.343	0.781271342	1
ENSBTAG00000019497	*ILDR2*	190	53	113	165	61	-1.427	0.261376728	1
ENSBTAG00000019500	*CNIH*	2'987	2'193	2'560	2'587	2'532	-0.031	0.969951485	1
ENSBTAG00000019501	*NUDT15*	708	508	600	613	587	-0.064	0.943963206	1
ENSBTAG00000019502	*MED4*	3'551	1'644	2'487	3'075	1'898	-0.696	0.377464079	1

Ensembl gene ID	geneName	counts wildtype horn bud	counts polled horn bud	baseMean	baseMean wildtype horn bud	baseMean polled horn bud	log2FoldChange	pval	padj
ENSBTAG00000019504	*ADRA1D*	121	75	96	105	87	-0.275	0.84297519	1
ENSBTAG00000019506	*ODF3L1*	1	0	0	1	0		1	1
ENSBTAG00000019508	*SEC61A2*	4'863	3'262	3'989	4'211	3'767	-0.161	0.836222453	1
ENSBTAG00000019509	*NUDT5*	1'098	720	891	951	831	-0.194	0.817562491	1
ENSBTAG00000019510	*BT.48888*	2'835	1'923	2'338	2'455	2'220	-0.145	0.854915158	1
ENSBTAG00000019511	*CDC123*	1'755	1'574	1'669	1'520	1'817	0.258	0.746316777	1
ENSBTAG00000019512	*BT.65227*	1'798	2'990	2'505	1'557	3'453	1.149	0.147515425	1
ENSBTAG00000019513	*C9ORF50*	58	57	58	50	66	0.390	0.816360604	1
ENSBTAG00000019514	*OPA1*	1'533	815	1'134	1'328	941	-0.496	0.544645394	1
ENSBTAG00000019515	*SNX17*	3'333	4'660	4'134	2'886	5'381	0.899	0.248699174	1
ENSBTAG00000019516	*BT.33191*	414	446	437	359	515	0.522	0.563958704	1
ENSBTAG00000019517	*ELN*	15'132	5'629	9'802	13'105	6'500	-1.012	0.189823421	1
ENSBTAG00000019518	*ZNF513*	750	894	841	650	1'032	0.668	0.425187355	1
ENSBTAG00000019519	*PWWP2B*	127	394	282	110	455	2.048	0.042204108	1
ENSBTAG00000019520	*ZC3H18*	1'898	2'137	2'056	1'644	2'468	0.586	0.45917131	1
ENSBTAG00000019522	*PPM1G*	3'983	3'937	3'998	3'449	4'546	0.398	0.607081896	1
ENSBTAG00000019524	*ITGAD*	6	1	3	5	1	-2.170	0.885240772	1
ENSBTAG00000019525	*BT.18214*	1'278	976	1'117	1'107	1'127	0.026	0.975048757	1
ENSBTAG00000019526	*BT.93597*	9'070	7'073	8'011	7'855	8'167	0.056	0.940938523	1
ENSBTAG00000019529	*ATP8A2*	120	29	69	104	33	-1.634	0.288211559	1
ENSBTAG00000019530	*KDM8*	306	278	293	265	321	0.277	0.777056436	1
ENSBTAG00000019532	*HTRA4*	26	3	13	23	3	-2.700	0.499863335	1
ENSBTAG00000019533	*C23H6ORF105*	12	3	7	10	3	-1.585	0.809704871	1
ENSBTAG00000019534	*TRNT1*	1'946	1'009	1'425	1'685	1'165	-0.533	0.509657651	1
ENSBTAG00000019535	*pseudogene*	6	17	12	5	20	1.918	0.627759625	1
ENSBTAG00000019536	*CRBN*	2'481	1'753	2'086	2'149	2'024	-0.086	0.914526315	1
ENSBTAG00000019537	*Mrz 08*	2'811	1'941	2'338	2'434	2'241	-0.119	0.880658713	1
ENSBTAG00000019538	*FBXO28*	619	496	554	536	573	0.095	0.914397713	1
ENSBTAG00000019539	*MFAP3L*	27	20	23	23	23	-0.018	1	1
ENSBTAG00000019540	*CRNN*	100	4	46	87	5	-4.229	0.043388663	1
ENSBTAG00000019542	*MRPL39*	1'082	695	870	937	803	-0.224	0.790252144	1
ENSBTAG00000019545	*SPATA13*	330	200	258	286	231	-0.307	0.760772454	1
ENSBTAG00000019546	*CXORF48*	1	0	0	1	0		1	1
ENSBTAG00000019547	*protein_coding*	725	432	563	628	499	-0.332	0.705666645	1
ENSBTAG00000019548	*BT.106330*	32	37	35	28	43	0.624	0.768108452	1
ENSBTAG00000019550	*TBC1D2*	314	347	336	272	401	0.559	0.55450989	1
ENSBTAG00000019552	*PGRMC1*	7'821	5'254	6'420	6'773	6'067	-0.159	0.836946226	1
ENSBTAG00000019553	*AKAP17A*	1	2	2	1	2	1.415	0.981979269	1
ENSBTAG00000019554	*FBP2*	22	11	16	19	13	-0.585	0.874518804	1
ENSBTAG00000019555	*ZSCAN20*	140	177	163	121	204	0.753	0.501229362	1
ENSBTAG00000019556	*MIIP*	1'305	957	1'118	1'130	1'105	-0.032	0.970280477	1
ENSBTAG00000019557	*NR1I2*	10	4	7	9	5	-0.907	0.905171387	1
ENSBTAG00000019563	*C5H22ORF23*	85	47	64	74	54	-0.440	0.784938281	1
ENSBTAG00000019564	*SMU1*	3'540	2'786	3'141	3'066	3'217	0.069	0.928663009	1
ENSBTAG00000019565	*NCOA4*	7'141	4'418	5'643	6'184	5'101	-0.278	0.719101013	1
ENSBTAG00000019567	*IL21R*	42	27	34	36	31	-0.222	0.930745495	1
ENSBTAG00000019568	*IFT74*	1'495	508	941	1'295	587	-1.142	0.172493653	1
ENSBTAG00000019569	*CD151*	5'034	7'067	6'260	4'360	8'160	0.904	0.242296058	1
ENSBTAG00000019574	*MAPK12*	292	117	194	253	135	-0.904	0.399281489	1
ENSBTAG00000019577	*CCRL1*	92	50	69	80	58	-0.465	0.765080336	1
ENSBTAG00000019579	*BT.86779*	571	551	565	495	636	0.364	0.677317207	1
ENSBTAG00000019581	*PLEKHF2*	2'215	1'753	1'971	1'918	2'024	0.078	0.92191569	1
ENSBTAG00000019585	*MYOM1*	165	283	235	143	327	1.193	0.244426181	1
ENSBTAG00000019587	*PI15*	122	96	108	106	111	0.069	0.963574073	1
ENSBTAG00000019588	*BOLA-DQB*	1'067	293	631	924	338	-1.450	0.098012193	1
ENSBTAG00000019589	*C1H21ORF2*	660	630	650	572	727	0.348	0.685564156	1
ENSBTAG00000019591	*DCX*	49	22	34	42	25	-0.740	0.733014257	1
ENSBTAG00000019592	*MBD5*	1'026	607	795	889	701	-0.342	0.685489751	1
ENSBTAG00000019593	*TSG118*	872	1'015	964	755	1'172	0.634	0.443902974	1
ENSBTAG00000019595	*EGFLAM*	1'231	1'955	1'662	1'066	2'257	1.082	0.179033951	1
ENSBTAG00000019596	*GPRC5B*	1'219	1'065	1'143	1'056	1'230	0.220	0.787375988	1
ENSBTAG00000019597	*pseudogene*	1	1	1	1	1	0.415	1	1
ENSBTAG00000019600	*OOEP*	244	194	218	211	224	0.084	0.938969422	1
ENSBTAG00000019601	*TMOD3*	265	251	260	229	290	0.337	0.736401906	1
ENSBTAG00000019603	*LDHB*	7'286	12'093	10'137	6'310	13'964	1.146	0.138136505	1
ENSBTAG00000019604	*VASP*	3'087	3'389	3'293	2'673	3'913	0.550	0.480593644	1
ENSBTAG00000019605	*OPA3*	36	59	50	31	68	1.128	0.519346108	1
ENSBTAG00000019606	*IFT52*	1'404	790	1'064	1'216	912	-0.415	0.61463386	1
ENSBTAG00000019608	*DDX47*	2'029	1'786	1'910	1'757	2'062	0.231	0.770623048	1
ENSBTAG00000019611	*MAGI3*	3'086	1'969	2'473	2'673	2'274	-0.233	0.767404687	1
ENSBTAG00000019612	*RNASE4*	6'046	5'279	5'666	5'236	6'096	0.219	0.77520638	1
ENSBTAG00000019614	*BT.64974*	356	292	323	308	337	0.129	0.894333261	1

Ensembl gene ID	geneName	counts wildtype horn bud	counts polled horn bud	baseMean	baseMean wildtype horn bud	baseMean polled horn bud	log2FoldChange	pval	padj
ENSBTAG00000019615	*PHTF1*	545	295	406	472	341	-0.471	0.60889788	1
ENSBTAG00000019617	*PTPN22*	22	6	13	19	7	-1.459	0.70932461	1
ENSBTAG00000019621	*NDRG3*	5'847	3'735	4'688	5'064	4'313	-0.232	0.765122589	1
ENSBTAG00000019622	*protein_coding*	1	3	2	1	3	2.000	0.933774563	1
ENSBTAG00000019623	*GRID1*	682	403	528	591	465	-0.344	0.697914601	1
ENSBTAG00000019625	*EHHADH*	761	710	739	659	820	0.315	0.710147653	1
ENSBTAG00000019627	*THY1*	15'285	7'785	11'113	13'237	8'989	-0.558	0.466325947	1
ENSBTAG00000019630	*RGL1*	2'383	2'511	2'482	2'064	2'899	0.491	0.532324984	1
ENSBTAG00000019633	*SHROOM3*	256	322	297	222	372	0.746	0.441743236	1
ENSBTAG00000019634	*PIDD*	573	795	707	496	918	0.887	0.299124176	1
ENSBTAG00000019636	*SCARA5*	3'901	3'069	3'461	3'378	3'544	0.069	0.928978861	1
ENSBTAG00000019639	*SNX16*	1'299	613	916	1'125	708	-0.668	0.422727648	1
ENSBTAG00000019642	*ANKDD1A*	7	4	5	6	5	-0.392	0.98714753	1
ENSBTAG00000019644	*EXT2*	5'501	5'581	5'604	4'764	6'444	0.436	0.571298061	1
ENSBTAG00000019645	*TFDP1*	3'928	2'927	3'391	3'402	3'380	-0.009	0.991549624	1
ENSBTAG00000019646	*TRIM41*	1'716	1'361	1'529	1'486	1'572	0.081	0.919956956	1
ENSBTAG00000019648	*GNB2L1*	107'488	126'016	119'299	93'087	145'511	0.644	0.39655784	1
ENSBTAG00000019651	*TRIM52*	503	247	360	436	285	-0.611	0.51465165	1
ENSBTAG00000019652	*MST4*	1'173	1'138	1'165	1'016	1'314	0.371	0.64870728	1
ENSBTAG00000019658	*ASB16*	15	17	16	13	20	0.596	0.865938341	1
ENSBTAG00000019660	*BT.31436*	2'072	1'500	1'763	1'794	1'732	-0.051	0.950326982	1
ENSBTAG00000019662	*ATXN7L3*	3'902	5'596	4'920	3'379	6'462	0.935	0.228641954	1
ENSBTAG00000019663	*DECR1*	1'567	675	1'068	1'357	779	-0.800	0.332027304	1
ENSBTAG00000019665	*IL1RN*	441	101	249	382	117	-1.711	0.095588911	1
ENSBTAG00000019667	*RNF187*	1'897	1'664	1'782	1'643	1'921	0.226	0.776187947	1
ENSBTAG00000019669	*CD163*	1'158	617	858	1'003	712	-0.493	0.556154944	1
ENSBTAG00000019670	*SUCNR1*	12	10	11	10	12	0.152	0.995696634	1
ENSBTAG00000019672	*BT.91865*	500	481	494	433	555	0.359	0.686474757	1
ENSBTAG00000019674	*CLIP3*	2'192	2'875	2'609	1'898	3'320	0.806	0.305723439	1
ENSBTAG00000019675	*ATXN1*	140	129	135	121	149	0.297	0.804971657	1
ENSBTAG00000019676	*MC4R*	35	3	17	30	3	-3.129	0.359989613	1
ENSBTAG00000019678	*ALKBH6*	258	194	224	223	224	0.004	1	1
ENSBTAG00000019679	*C19ORF46*	29	3	14	25	3	-2.858	0.448507214	1
ENSBTAG00000019680	*ZYG11B*	702	630	668	608	727	0.259	0.762670562	1
ENSBTAG00000019682	*PRRC2A*	11'246	21'310	17'173	9'739	24'607	1.337	0.083823466	1
ENSBTAG00000019683	*LYPD6B*	92	80	86	80	92	0.213	0.883602938	1
ENSBTAG00000019684	*NUDT3*	3'635	3'366	3'517	3'148	3'887	0.304	0.695194417	1
ENSBTAG00000019685	*BT.23547*	9'301	13'653	11'910	8'055	15'765	0.969	0.20787505	1
ENSBTAG00000019686	*BT.30694*	586	189	363	507	218	-1.217	0.196534362	1
ENSBTAG00000019687	*C9ORF95*	365	174	259	316	201	-0.654	0.513762084	1
ENSBTAG00000019689	*C11ORF80*	514	290	390	445	335	-0.411	0.657658852	1
ENSBTAG00000019690	*PNLIPRP3*	170	77	118	147	89	-0.728	0.55866901	1
ENSBTAG00000019692	*BCL2L2*	3'129	1'546	2'247	2'710	1'785	-0.602	0.446421864	1
ENSBTAG00000019695	*RLF*	2'909	1'643	2'208	2'519	1'897	-0.409	0.604883717	1
ENSBTAG00000019696	*RCE1*	1'074	860	962	930	993	0.094	0.909538949	1
ENSBTAG00000019697	*BT.55456*	156	71	109	135	82	-0.721	0.573720233	1
ENSBTAG00000019698	*TMCO2*	39	17	27	34	20	-0.783	0.753120629	1
ENSBTAG00000019700	*PC*	1'454	2'167	1'881	1'259	2'502	0.991	0.215294598	1
ENSBTAG00000019701	*RPL31*	76'992	50'790	62'662	66'677	58'647	-0.185	0.807874563	1
ENSBTAG00000019703	*PRPS1*	1'406	690	1'007	1'218	797	-0.612	0.459406248	1
ENSBTAG00000019704	*HLTF*	1'964	937	1'391	1'701	1'082	-0.653	0.420025564	1
ENSBTAG00000019707	*GATA2*	310	171	233	268	197	-0.443	0.666437305	1
ENSBTAG00000019711	*LRMP*	262	58	147	227	67	-1.760	0.135247064	1
ENSBTAG00000019712	*ST14*	659	1'437	1'115	571	1'659	1.540	0.064654132	1
ENSBTAG00000019714	*DAZAP1*	1'969	1'869	1'932	1'705	2'158	0.340	0.66796608	1
ENSBTAG00000019718	*RPS15*	45'583	43'028	44'580	39'476	49'684	0.332	0.661676351	1
ENSBTAG00000019721	*REXO4*	443	587	531	384	678	0.821	0.352384819	1
ENSBTAG00000019722	*GUCY2C*	718	485	591	622	560	-0.151	0.864311599	1
ENSBTAG00000019723	*PEX12*	453	392	422	392	453	0.206	0.821606015	1
ENSBTAG00000019725	*UBE4A*	1'622	1'000	1'280	1'405	1'155	-0.283	0.728324574	1
ENSBTAG00000019726	*ANKRD5*	19	1	9	16	1	-3.833	0.507017893	1
ENSBTAG00000019728	*BT.50945*	39	25	31	34	29	-0.227	0.933100084	1
ENSBTAG00000019729	*BT.18733*	897	608	739	777	702	-0.146	0.865173743	1
ENSBTAG00000019730	*BT.75868*	15'123	4'435	9'109	13'097	5'121	-1.355	0.081400629	1
ENSBTAG00000019733	*BT.23705*	1'198	322	705	1'037	372	-1.480	0.087468467	1
ENSBTAG00000019734	*CD276*	2'693	3'907	3'422	2'332	4'511	0.952	0.224136827	1
ENSBTAG00000019735	*CPSF3*	1'935	1'754	1'851	1'676	2'025	0.273	0.730496075	1
ENSBTAG00000019738	*MDM1*	675	422	536	585	487	-0.263	0.767141921	1
ENSBTAG00000019739	*pseudogene*	1'708	1'255	1'464	1'479	1'449	-0.030	0.972276545	1
ENSBTAG00000019741	*C3AR1*	389	223	297	337	257	-0.388	0.691119986	1
ENSBTAG00000019742	*FOXJ2*	1'263	1'626	1'486	1'094	1'878	0.780	0.333524335	1
ENSBTAG00000019743	*CHTF18*	559	909	767	484	1'050	1.116	0.189620239	1

Ensembl gene ID	geneName	counts wildtype horn bud	counts polled horn bud	baseMean	baseMean wildtype horn bud	baseMean polled horn bud	log2FoldChange	pval	padj
ENSBTAG00000019745	*LMF1*	827	766	800	716	885	0.304	0.71724316	1
ENSBTAG00000019746	*CCDC66*	2'700	883	1'679	2'338	1'020	-1.197	0.138135598	1
ENSBTAG00000019748	*FAM208A*	5'599	2'572	3'909	4'849	2'970	-0.707	0.363804891	1
ENSBTAG00000019750	*TMEM106B*	1'133	697	893	981	805	-0.286	0.732622747	1
ENSBTAG00000019754	*PRKCDBP*	1'986	1'422	1'681	1'720	1'642	-0.067	0.934606929	1
ENSBTAG00000019755	*REEP3*	1'724	2'074	1'944	1'493	2'395	0.682	0.390946972	1
ENSBTAG00000019757	*SUDS3*	461	296	371	399	342	-0.224	0.812189926	1
ENSBTAG00000019759	*IDE*	664	493	572	575	569	-0.015	0.98972859	1
ENSBTAG00000019761	*MANBA*	3'072	1'652	2'284	2'660	1'908	-0.480	0.543438519	1
ENSBTAG00000019762	*processed_pseudogene*	1	0	0	1	0		1	1
ENSBTAG00000019767	*MIER2*	2'640	3'381	3'095	2'286	3'904	0.772	0.324097485	1
ENSBTAG00000019770	*APOA4*	10	9	10	9	10	0.263	0.978917997	1
ENSBTAG00000019771	*PALM*	1'983	2'715	2'426	1'717	3'135	0.868	0.271686558	1
ENSBTAG00000019772	*OXTR*	13	8	10	11	9	-0.285	0.973425765	1
ENSBTAG00000019777	*CDCA3*	600	784	712	520	905	0.801	0.347764344	1
ENSBTAG00000019779	*RAB39B*	59	1	26	51	1	-5.468	0.078724721	1
ENSBTAG00000019780	*USP5*	2'771	4'686	3'905	2'400	5'411	1.173	0.134600777	1
ENSBTAG00000019781	*CLASP1*	1'827	1'922	1'901	1'582	2'219	0.488	0.538528912	1
ENSBTAG00000019782	*TPI1*	10'543	13'977	12'635	9'131	16'139	0.822	0.283959433	1
ENSBTAG00000019783	*SPSB2*	170	162	167	147	187	0.345	0.757838757	1
ENSBTAG00000019784	*PPP2R5E*	884	676	773	766	781	0.028	0.974492657	1
ENSBTAG00000019785	*CIC*	3'989	8'245	6'488	3'455	9'521	1.463	0.061563837	1
ENSBTAG00000019787	*PAFAH1B3*	1'840	2'659	2'332	1'593	3'070	0.946	0.232248612	1
ENSBTAG00000019788	*TEAD4*	74	147	117	64	170	1.405	0.260777186	1
ENSBTAG00000019790	*BT.24814*	265	76	159	229	88	-1.387	0.224411267	1
ENSBTAG00000019791	*GRHL3*	656	407	519	568	470	-0.274	0.758592187	1
ENSBTAG00000019792	*SLC16A9*	102	28	60	88	32	-1.450	0.370615913	1
ENSBTAG00000019793	*RGS22*	201	237	224	174	274	0.653	0.526573549	1
ENSBTAG00000019794	*SYPL1*	9'272	7'454	8'318	8'030	8'607	0.100	0.895381147	1
ENSBTAG00000019795	*FBXO43*	13	19	17	11	22	0.963	0.770262643	1
ENSBTAG00000019796	*FAIM3*	17	2	9	15	2	-2.672	0.628971544	1
ENSBTAG00000019798	*BT.105276*	26	2	12	23	2	-3.285	0.441095735	1
ENSBTAG00000019800	*RUNX3*	110	105	108	95	121	0.348	0.787970043	1
ENSBTAG00000019801	*FAM151A*	96	28	58	83	32	-1.363	0.408640262	1
ENSBTAG00000019803	*POLR3K*	1'853	1'426	1'626	1'605	1'647	0.037	0.963059653	1
ENSBTAG00000019804	*SNRNP25*	1'859	1'686	1'778	1'610	1'947	0.274	0.730262365	1
ENSBTAG00000019805	*RHBDF1*	3'453	4'438	4'057	2'990	5'125	0.777	0.317731868	1
ENSBTAG00000019806	*YPEL2*	295	301	302	255	348	0.444	0.645999054	1
ENSBTAG00000019807	*COL27A1*	3'746	3'248	3'497	3'244	3'750	0.209	0.787340814	1
ENSBTAG00000019808	*BT.60938*	226	170	196	196	196	0.004	1	1
ENSBTAG00000019810	*AARS*	6'290	5'891	6'125	5'447	6'802	0.320	0.676433949	1
ENSBTAG00000019812	*BT.44539*	3'813	3'941	3'926	3'302	4'551	0.463	0.550660497	1
ENSBTAG00000019813	*ADIPOQ*	16	11	13	14	13	-0.126	0.9977793	1
ENSBTAG00000019814	*PGBD2*	158	189	178	137	218	0.673	0.537974214	1
ENSBTAG00000019818	*BT.104317*	1'121	1'797	1'523	971	2'075	1.096	0.175719954	1
ENSBTAG00000019819	*FUT8*	2'022	1'633	1'818	1'751	1'886	0.107	0.893056668	1
ENSBTAG00000019820	*RAB9B*	121	88	103	105	102	-0.044	0.982011749	1
ENSBTAG00000019821	*DHX9*	10'972	10'575	10'856	9'502	12'211	0.362	0.635467502	1
ENSBTAG00000019823	*ADAMTS17*	1'345	879	1'090	1'165	1'015	-0.199	0.809851291	1
ENSBTAG00000019824	*pseudogene*	13	64	43	11	74	2.715	0.166408427	1
ENSBTAG00000019828	*MAP3K7IP1*	676	1'256	1'018	585	1'450	1.309	0.116645945	1
ENSBTAG00000019830	*BT.52114*	446	421	436	386	486	0.332	0.714427875	1
ENSBTAG00000019831	*BT.48989*	530	518	529	459	598	0.382	0.664722751	1
ENSBTAG00000019832	*BT.66001*	11'948	4'924	8'017	10'347	5'686	-0.864	0.262765173	1
ENSBTAG00000019834	*ARL15*	172	251	219	149	290	0.960	0.354804897	1
ENSBTAG00000019836	*GSKIP*	1'545	1'116	1'313	1'338	1'289	-0.054	0.948222528	1
ENSBTAG00000019838	*SHC1*	17'081	16'448	16'893	14'793	18'993	0.361	0.635679747	1
ENSBTAG00000019839	*BT.89253*	19'312	11'869	15'215	16'725	13'705	-0.287	0.706982602	1
ENSBTAG00000019840	*pseudogene*	4	1	2	3	1	-1.585	0.952247636	1
ENSBTAG00000019841	*BT.57532*	255	120	180	221	139	-0.672	0.539071712	1
ENSBTAG00000019843	*HAUS6*	868	491	659	752	567	-0.407	0.636512243	1
ENSBTAG00000019844	*ZNF467*	111	168	145	96	194	1.013	0.382691327	1
ENSBTAG00000019845	*USP30*	1'567	1'565	1'582	1'357	1'807	0.413	0.6056464	1
ENSBTAG00000019846	*BT.65140*	1'196	929	1'054	1'036	1'073	0.051	0.951394964	1
ENSBTAG00000019847	*CPEB2*	310	114	200	268	132	-1.028	0.334719833	1
ENSBTAG00000019848	*UNG*	2'452	2'539	2'528	2'123	2'932	0.465	0.553236766	1
ENSBTAG00000019851	*PPP2R1A*	10'463	14'689	13'011	9'061	16'961	0.904	0.238842307	1
ENSBTAG00000019852	*PDHA1*	2'229	2'347	2'320	1'930	2'710	0.489	0.534185803	1
ENSBTAG00000019853	*SMURF2*	1'616	869	1'201	1'399	1'003	-0.480	0.556517411	1
ENSBTAG00000019854	*ABCE1*	1'525	1'127	1'311	1'321	1'301	-0.021	0.980777694	1
ENSBTAG00000019855	*MAP3K15*	14	10	12	12	12	-0.070	1	1
ENSBTAG00000019857	*OTUD4*	1'807	1'113	1'425	1'565	1'285	-0.284	0.725290536	1

Ensembl gene ID	geneName	counts wildtype horn bud	counts polled horn bud	baseMean	baseMean wildtype horn bud	baseMean polled horn bud	log2FoldChange	pval	padj
ENSBTAG00000019859	*B3GALT2*	1'188	574	846	1'029	663	-0.634	0.449810156	1
ENSBTAG00000019864	*BT.41192*	386	175	268	334	202	-0.726	0.464687015	1
ENSBTAG00000019866	*NRP1*	5'828	5'872	5'914	5'047	6'780	0.426	0.579786728	1
ENSBTAG00000019867	*BT.58045*	1'771	1'577	1'677	1'534	1'821	0.248	0.756092565	1
ENSBTAG00000019868	*RUFY1*	628	745	702	544	860	0.662	0.438023287	1
ENSBTAG00000019869	*bta-mir-1306*	3'709	4'759	4'354	3'212	5'495	0.775	0.318528476	1
ENSBTAG00000019870	*SLC14A1*	133	51	87	115	59	-0.968	0.485397065	1
ENSBTAG00000019871	*TRMT2A*	1'050	1'248	1'175	909	1'441	0.664	0.415879342	1
ENSBTAG00000019872	*PIK3AP1*	361	119	225	313	137	-1.186	0.253279145	1
ENSBTAG00000019876	*DSC1*	6'784	2'534	4'401	5'875	2'926	-1.006	0.19730442	1
ENSBTAG00000019877	*DOCK7*	4'986	2'621	3'672	4'318	3'026	-0.513	0.510254257	1
ENSBTAG00000019880	*protein_coding*	1	0	0	1	0		1	1
ENSBTAG00000019881	*BT.89943*	25	14	19	22	16	-0.421	0.90258764	1
ENSBTAG00000019883	*BT.49487*	8'369	6'614	7'442	7'248	7'637	0.076	0.921036067	1
ENSBTAG00000019884	*protein_coding*	907	450	653	785	520	-0.596	0.488993686	1
ENSBTAG00000019885	*ZNF280C*	441	238	328	382	275	-0.475	0.618997397	1
ENSBTAG00000019886	*BORA*	892	440	640	772	508	-0.605	0.483868108	1
ENSBTAG00000019887	*protein_coding*	212	177	194	184	204	0.155	0.888125338	1
ENSBTAG00000019888	*HIRA*	1'675	2'311	2'060	1'451	2'669	0.879	0.26863298	1
ENSBTAG00000019889	*DIS3*	1'533	740	1'091	1'328	854	-0.636	0.439418545	1
ENSBTAG00000019891	*MRPL40*	538	534	541	466	617	0.404	0.645392199	1
ENSBTAG00000019892	*BT.63577*	1'637	1'105	1'347	1'418	1'276	-0.152	0.85215438	1
ENSBTAG00000019894	*C17H22orf39*	243	186	213	210	215	0.029	0.98184748	1
ENSBTAG00000019895	*PIBF1*	494	210	335	428	242	-0.819	0.388942364	1
ENSBTAG00000019900	*LNPEP*	58	64	62	50	74	0.557	0.727712959	1
ENSBTAG00000019901	*RAD54L*	857	628	734	742	725	-0.033	0.970981265	1
ENSBTAG00000019903	*RAMP2*	3'589	1'948	2'679	3'108	2'249	-0.467	0.552511793	1
ENSBTAG00000019904	*BT.37162*	241	454	366	209	524	1.329	0.158155125	1
ENSBTAG00000019906	*VPS25*	4'434	4'496	4'516	3'840	5'192	0.435	0.573457931	1
ENSBTAG00000019907	*WNK4*	688	1'065	913	596	1'230	1.045	0.211578959	1
ENSBTAG00000019908	*CUL9*	2'659	3'447	3'142	2'303	3'980	0.789	0.313150059	1
ENSBTAG00000019909	*SIRT6*	452	552	514	391	637	0.703	0.426959906	1
ENSBTAG00000019910	*SDCBP*	6'575	6'082	6'359	5'694	7'023	0.303	0.693348363	1
ENSBTAG00000019911	*CCDC56*	2'101	2'046	2'091	1'820	2'363	0.377	0.633336799	1
ENSBTAG00000019912	*CNTD1*	243	70	146	210	81	-1.380	0.2381584	1
ENSBTAG00000019913	*C6ORF108*	751	757	762	650	874	0.427	0.613591859	1
ENSBTAG00000019914	*BECN1*	2'546	1'637	2'048	2'205	1'890	-0.222	0.779839395	1
ENSBTAG00000019915	*BT.53493*	55'083	56'039	56'206	47'703	64'708	0.440	0.562180536	1
ENSBTAG00000019916	*CLMN*	239	402	336	207	464	1.165	0.221378565	1
ENSBTAG00000019918	*PSME3*	1'220	1'453	1'367	1'057	1'678	0.667	0.409374134	1
ENSBTAG00000019919	*TTC40*	193	382	304	167	441	1.400	0.151185363	1
ENSBTAG00000019920	*CCDC64B*	199	178	189	172	206	0.254	0.815555188	1
ENSBTAG00000019924	*BT.91786*	977	474	697	846	547	-0.628	0.462614406	1
ENSBTAG00000019926	*BT.44370*	5	2	3	4	2	-0.907	0.965043111	1
ENSBTAG00000019927	*CYB5R1*	1'253	1'430	1'368	1'085	1'651	0.606	0.453578809	1
ENSBTAG00000019928	*AQP11*	50	36	42	43	42	-0.059	0.990467387	1
ENSBTAG00000019929	*ITGAV*	2'439	1'422	1'877	2'112	1'642	-0.363	0.648207672	1
ENSBTAG00000019930	*CLNS1A*	1'527	1'239	1'377	1'322	1'431	0.114	0.888168366	1
ENSBTAG00000019933	*BT.21055*	1'532	1'213	1'364	1'327	1'401	0.078	0.922984351	1
ENSBTAG00000019937	*WDR3*	2'767	2'382	2'573	2'396	2'750	0.199	0.799434227	1
ENSBTAG00000019938	*N4BP1*	618	549	585	535	634	0.244	0.779295364	1
ENSBTAG00000019939	*SLC35D2*	292	71	167	253	82	-1.625	0.150719199	1
ENSBTAG00000019940	*RASGRF1*	44	11	25	38	13	-1.585	0.529046878	1
ENSBTAG00000019942	*PABPC5*	157	138	148	136	159	0.229	0.845980006	1
ENSBTAG00000019944	*BT.49424*	5'959	3'722	4'729	5'161	4'298	-0.264	0.733285806	1
ENSBTAG00000019946	*BT.36835*	31	20	25	27	23	-0.217	0.94785322	1
ENSBTAG00000019947	*SYT7*	180	50	107	156	58	-1.433	0.268437239	1
ENSBTAG00000019948	*AFAP1L1*	610	267	418	528	308	-0.777	0.395957237	1
ENSBTAG00000019949	*DBR1*	758	436	580	656	503	-0.383	0.661609303	1
ENSBTAG00000019950	*GRPEL2*	2'217	1'460	1'803	1'920	1'686	-0.188	0.814657597	1
ENSBTAG00000019951	*PCYOX1L*	495	455	477	429	525	0.293	0.743037507	1
ENSBTAG00000019952	*ARMC8*	1'644	1'123	1'360	1'424	1'297	-0.135	0.868791231	1
ENSBTAG00000019953	*CYBB*	438	352	393	379	406	0.100	0.915625133	1
ENSBTAG00000019954	*ABHD2*	212	257	240	184	297	0.693	0.494526269	1
ENSBTAG00000019956	*CMPK1*	2'707	1'473	2'023	2'344	1'701	-0.463	0.559717127	1
ENSBTAG00000019958	*NXNL2*	31	4	16	27	5	-2.539	0.465876696	1
ENSBTAG00000019960	*TM4SF18*	706	152	393	611	176	-1.801	0.056404334	1
ENSBTAG00000019961	*BT.29440*	5	1	3	4	1	-1.907	0.9188325	1
ENSBTAG00000019964	*GAS6*	2'387	1'011	1'617	2'067	1'167	-0.824	0.305279488	1
ENSBTAG00000019965	*FAM70B*	5	8	7	4	9	1.093	0.872919157	1
ENSBTAG00000019966	*BT.47460*	305	470	403	264	543	1.039	0.259564314	1
ENSBTAG00000019967	*RASA3*	5'921	3'087	4'346	5'128	3'565	-0.525	0.498947368	1

Ensembl gene ID	geneName	counts wildtype horn bud	counts polled horn bud	baseMean	baseMean wildtype horn bud	baseMean polled horn bud	log2FoldChange	pval	padj
ENSBTAG00000019971	*CDC16*	2'373	1'293	1'774	2'055	1'493	-0.461	0.563660767	1
ENSBTAG00000019972	*BT.21446*	1'039	1'638	1'396	900	1'891	1.072	0.18731674	1
ENSBTAG00000019975	*IL7R*	21	17	19	18	20	0.110	0.989855944	1
ENSBTAG00000019979	*CMPK2*	228	89	150	197	103	-0.942	0.413658052	1
ENSBTAG00000019980	*DGKI*	120	32	70	104	37	-1.492	0.325682317	1
ENSBTAG00000019983	*ASXL2*	532	416	471	461	480	0.060	0.948112037	1
ENSBTAG00000019984	*GINS1*	1'970	2'254	2'154	1'706	2'603	0.609	0.440865382	1
ENSBTAG00000019986	*CSNK1D*	5'504	7'176	6'526	4'767	8'286	0.798	0.301275096	1
ENSBTAG00000019987	*RPP14*	1'427	1'016	1'204	1'236	1'173	-0.075	0.928192455	1
ENSBTAG00000019988	*BT.27662*	117	103	110	101	119	0.231	0.859754983	1
ENSBTAG00000019989	*PXK*	820	649	730	710	749	0.078	0.927944194	1
ENSBTAG00000019993	*APLN*	77	70	74	67	81	0.278	0.856319363	1
ENSBTAG00000019994	*pseudogene*	19'827	11'691	15'335	17'171	13'500	-0.347	0.64968362	1
ENSBTAG00000019995	*C29H11orf73*	1'936	1'500	1'704	1'677	1'732	0.047	0.953149281	1
ENSBTAG00000019998	*HAGH*	1'560	1'423	1'497	1'351	1'643	0.282	0.724801189	1
ENSBTAG00000020000	*RAP1GAP2*	66	29	45	57	33	-0.771	0.677772397	1
ENSBTAG00000020001	*C16ORF73*	176	68	115	152	79	-0.957	0.444932833	1
ENSBTAG00000020003	*KLHL14*	27	21	24	23	24	0.052	1	1
ENSBTAG00000020004	*ZNF800*	614	350	468	532	404	-0.396	0.660253429	1
ENSBTAG00000020007	*protein_coding*	1	0	0	1	0		1	1
ENSBTAG00000020009	*CCDC81*	6	5	5	5	6	0.152	1	1
ENSBTAG00000020010	*SPATA1*	1	1	1	1	1	0.415	1	1
ENSBTAG00000020012	*CDC5L*	2'103	1'577	1'821	1'821	1'821	0.000	1	1
ENSBTAG00000020014	*CEP104*	1'204	953	1'072	1'043	1'100	0.078	0.924872596	1
ENSBTAG00000020015	*BT.22809*	889	577	718	770	666	-0.209	0.807895451	1
ENSBTAG00000020017	*SLC25A41*	158	121	138	137	140	0.030	0.985760374	1
ENSBTAG00000020018	*FRK*	55	24	38	48	28	-0.781	0.702128407	1
ENSBTAG00000020019	*BT.106550*	0	1	1	0	1	Inf	0.993540919	1
ENSBTAG00000020022	*IL13RA2*	18	6	11	16	7	-1.170	0.791784766	1
ENSBTAG00000020023	*MTHFS*	824	612	710	714	707	-0.014	0.989433038	1
ENSBTAG00000020026	*BT.69212*	4'459	4'155	4'330	3'862	4'798	0.313	0.685213608	1
ENSBTAG00000020027	*NHLH1*	14	15	15	12	17	0.515	0.896151294	1
ENSBTAG00000020028	*RBP1*	1'092	1'472	1'323	946	1'700	0.846	0.297718377	1
ENSBTAG00000020030	*FITM2*	1'296	1'115	1'205	1'122	1'287	0.198	0.807672401	1
ENSBTAG00000020031	*PKD2*	9'720	5'151	7'183	8'418	5'948	-0.501	0.514958268	1
ENSBTAG00000020032	*KIAA1377*	763	152	418	661	176	-1.913	0.041430767	1
ENSBTAG00000020034	*LYN*	362	156	247	314	180	-0.799	0.429445956	1
ENSBTAG00000020035	*RCAN1*	3'792	943	2'186	3'284	1'089	-1.593	0.048094356	1
ENSBTAG00000020036	*CDCP2*	1	3	2	1	3	2.000	0.933774563	1
ENSBTAG00000020037	*CYB5RL*	141	70	101	122	81	-0.595	0.651085767	1
ENSBTAG00000020040	*LPCAT4*	2'630	2'721	2'710	2'278	3'142	0.464	0.553374599	1
ENSBTAG00000020041	*FAM13B*	1'576	792	1'140	1'365	915	-0.578	0.48082576	1
ENSBTAG00000020042	*KLHL28*	491	304	388	425	351	-0.277	0.766586955	1
ENSBTAG00000020043	*ARHGAP17*	1'828	1'230	1'502	1'583	1'420	-0.157	0.846650722	1
ENSBTAG00000020046	*ASAM*	3'630	1'941	2'692	3'144	2'241	-0.488	0.534246223	1
ENSBTAG00000020047	*MON1A*	431	713	598	373	823	1.141	0.191856559	1
ENSBTAG00000020049	*CABP1*	55	30	41	48	35	-0.459	0.819454556	1
ENSBTAG00000020050	*MLEC*	905	1'912	1'496	784	2'208	1.494	0.067579146	1
ENSBTAG00000020053	*ZEB1*	2'590	1'917	2'228	2'243	2'214	-0.019	0.982037068	1
ENSBTAG00000020054	*TNFRSF21*	2'632	1'730	2'139	2'279	1'998	-0.190	0.81052823	1
ENSBTAG00000020055	*KIAA1712*	941	486	688	815	561	-0.538	0.529763021	1
ENSBTAG00000020056	*COL12A1*	68'182	37'556	51'207	59'047	43'366	-0.445	0.558448921	1
ENSBTAG00000020059	*GEN1*	450	229	327	390	264	-0.560	0.557793219	1
ENSBTAG00000020060	*TXNIP*	24'776	18'842	21'607	21'457	21'757	0.020	0.978361398	1
ENSBTAG00000020061	*KCNJ12*	128	313	236	111	361	1.705	0.099969846	1
ENSBTAG00000020062	*BT.20401*	1'674	1'747	1'733	1'450	2'017	0.477	0.549838378	1
ENSBTAG00000020066	*ZW10*	2'471	1'757	2'084	2'140	2'029	-0.077	0.923701765	1
ENSBTAG00000020067	*BT.52154*	732	927	852	634	1'070	0.756	0.367148296	1
ENSBTAG00000020070	*ABCC3*	461	376	417	399	434	0.121	0.896016884	1
ENSBTAG00000020072	*CATHL4*	4	1	2	3	1	-1.585	0.952247636	1
ENSBTAG00000020073	*NCEH1*	497	244	356	430	282	-0.611	0.515331722	1
ENSBTAG00000020074	*BT.88257*	135	241	198	117	278	1.251	0.242168509	1
ENSBTAG00000020076	*CATHL5*	10	4	7	9	5	-0.907	0.905171387	1
ENSBTAG00000020077	*CATHL6*	2	0	1	2	0		0.974934741	1
ENSBTAG00000020079	*MAP3K4*	2'922	2'502	2'710	2'531	2'889	0.191	0.806724458	1
ENSBTAG00000020080	*MYBPC2*	153	26	81	133	30	-2.142	0.142613676	1
ENSBTAG00000020082	*FAM71E1*	29	33	32	25	38	0.601	0.789992796	1
ENSBTAG00000020084	*LONRF1*	1'676	1'419	1'545	1'451	1'639	0.175	0.82714301	1
ENSBTAG00000020087	*CAMK2A*	120	72	94	104	83	-0.322	0.816641348	1
ENSBTAG00000020089	*USP32*	905	585	730	784	675	-0.214	0.802193114	1
ENSBTAG00000020090	*EPHX3*	323	281	302	280	324	0.214	0.826117595	1
ENSBTAG00000020093	*CUL4A*	4'494	4'370	4'469	3'892	5'046	0.375	0.627677114	1

Ensembl gene ID	geneName	counts wildtype horn bud	counts polled horn bud	baseMean	baseMean wildtype horn bud	baseMean polled horn bud	log2FoldChange	pval	padj
ENSBTAG00000020096	*MS4A2*	22	12	16	19	14	-0.459	0.903495485	1
ENSBTAG00000020097	*PERP*	24'247	11'022	16'863	20'999	12'727	-0.722	0.345328197	1
ENSBTAG00000020100	*ARHGAP11A*	1'102	703	883	954	812	-0.233	0.780794156	1
ENSBTAG00000020102	*EPHA8*	19	2	9	16	2	-2.833	0.582174212	1
ENSBTAG00000020103	*pseudogene*	140	139	141	121	161	0.405	0.730965543	1
ENSBTAG00000020105	*BT.64996*	123	111	117	107	128	0.267	0.833348308	1
ENSBTAG00000020106	*AP2M1*	7'002	7'987	7'643	6'064	9'223	0.605	0.43097825	1
ENSBTAG00000020107	*DNASE1*	1'528	693	1'062	1'323	800	-0.726	0.378755981	1
ENSBTAG00000020108	*BT.27057*	1'198	2'218	1'799	1'037	2'561	1.304	0.105885583	1
ENSBTAG00000020109	*TRAP1*	1'214	1'412	1'341	1'051	1'630	0.633	0.434139537	1
ENSBTAG00000020115	*UBE2O*	2'239	3'352	2'905	1'939	3'871	0.997	0.205219852	1
ENSBTAG00000020116	*BT.102070*	1'282	416	795	1'110	480	-1.209	0.155408126	1
ENSBTAG00000020117	*ZBTB7A*	517	531	530	448	613	0.454	0.606559159	1
ENSBTAG00000020119	*RNF43*	271	388	341	235	448	0.933	0.324272488	1
ENSBTAG00000020120	*SEL1L2*	1	2	2	1	2	1.415	0.981979269	1
ENSBTAG00000020121	*RWDD2B*	552	384	461	478	443	-0.109	0.906779137	1
ENSBTAG00000020122	*BT.51616*	4'470	2'499	3'378	3'871	2'886	-0.424	0.586867289	1
ENSBTAG00000020123	*AUP1*	5'349	4'858	5'121	4'632	5'610	0.276	0.71982111	1
ENSBTAG00000020124	*HTRA2*	2'238	1'790	2'003	1'938	2'067	0.093	0.906564771	1
ENSBTAG00000020125	*RBFOX2*	1'942	1'414	1'657	1'682	1'633	-0.043	0.958827466	1
ENSBTAG00000020127	*TKTL1*	70	5	33	61	6	-3.392	0.145551138	1
ENSBTAG00000020130	*CLCN3*	530	423	474	459	488	0.090	0.921634653	1
ENSBTAG00000020132	*C8H4orf27*	425	546	499	368	630	0.776	0.382789434	1
ENSBTAG00000020133	*PPP3CC*	1'607	944	1'241	1'392	1'090	-0.352	0.665339673	1
ENSBTAG00000020136	*CASK*	3'158	2'468	2'792	2'735	2'850	0.059	0.939253456	1
ENSBTAG00000020139	*RPL7*	16'902	10'863	13'591	14'638	12'544	-0.223	0.770962452	1
ENSBTAG00000020140	*CER1*	6	6	6	5	7	0.415	0.976119138	1
ENSBTAG00000020141	*FREM1*	1'735	1'681	1'722	1'503	1'941	0.369	0.642854025	1
ENSBTAG00000020142	*LMBRD2*	80	22	47	69	25	-1.447	0.423593136	1
ENSBTAG00000020143	*RDH10*	773	505	626	669	583	-0.199	0.819409993	1
ENSBTAG00000020144	*BT.92137*	378	179	267	327	207	-0.663	0.504583505	1
ENSBTAG00000020147	*BT.42601*	400	561	497	346	648	0.903	0.311161445	1
ENSBTAG00000020148	*TEK*	2'488	1'214	1'778	2'155	1'402	-0.620	0.437507695	1
ENSBTAG00000020149	*RNASEH2B*	1'762	686	1'159	1'526	792	-0.946	0.24957581	1
ENSBTAG00000020150	*KLHDC9*	22	14	18	19	16	-0.237	0.958766648	1
ENSBTAG00000020151	*protein_coding*	126	42	79	109	48	-1.170	0.417682045	1
ENSBTAG00000020152	*PFDN2*	2'118	1'625	1'855	1'834	1'876	0.033	0.9671251	1
ENSBTAG00000020153	*BT.96669*	869	610	728	753	704	-0.096	0.912606807	1
ENSBTAG00000020154	*DEDD*	1'923	1'817	1'882	1'665	2'098	0.333	0.6743904	1
ENSBTAG00000020155	*RP1L1*	12	25	20	10	29	1.474	0.611572134	1
ENSBTAG00000020157	*BT.62777*	51	32	41	44	37	-0.257	0.906140921	1
ENSBTAG00000020159	*NKAIN1*	217	241	233	188	278	0.566	0.579169156	1
ENSBTAG00000020160	*RNF138*	519	269	380	449	311	-0.533	0.566245012	1
ENSBTAG00000020161	*KLHL22*	2'419	2'709	2'611	2'095	3'128	0.578	0.461181999	1
ENSBTAG00000020164	*BAZ1A*	2'264	1'882	2'067	1'961	2'173	0.148	0.850845323	1
ENSBTAG00000020166	*ZNFX1*	1'480	1'329	1'408	1'282	1'535	0.260	0.74693712	1
ENSBTAG00000020169	*BT.99383*	2'717	1'420	1'996	2'353	1'640	-0.521	0.511758386	1
ENSBTAG00000020172	*BT.26797*	160	106	130	139	122	-0.179	0.887546009	1
ENSBTAG00000020173	*BT.26676*	666	336	482	577	388	-0.572	0.522864336	1
ENSBTAG00000020174	*BT.36640*	4'062	1'866	2'836	3'518	2'155	-0.707	0.367842696	1
ENSBTAG00000020175	*UBE2K*	1'324	896	1'091	1'147	1'035	-0.148	0.857914545	1
ENSBTAG00000020177	*C5H12orf10*	673	723	709	583	835	0.518	0.542497264	1
ENSBTAG00000020178	*ARSK*	339	167	243	294	193	-0.606	0.550304482	1
ENSBTAG00000020179	*AAAS*	847	990	938	734	1'143	0.640	0.440627128	1
ENSBTAG00000020180	*TMED5*	1'882	966	1'373	1'630	1'115	-0.547	0.499102145	1
ENSBTAG00000020185	*protein_coding*	318	286	303	275	330	0.262	0.787239041	1
ENSBTAG00000020186	*EIF1AD*	757	671	715	656	775	0.241	0.777015354	1
ENSBTAG00000020190	*KREMEN2*	287	597	469	249	689	1.472	0.105498179	1
ENSBTAG00000020192	*PPP2R5C*	8'517	4'793	6'455	7'376	5'534	-0.414	0.590640067	1
ENSBTAG00000020193	*DCLRE1A*	1'192	864	1'015	1'032	998	-0.049	0.954289452	1
ENSBTAG00000020194	*FGF13*	1'367	539	903	1'184	622	-0.928	0.267814022	1
ENSBTAG00000020196	*ENPP3*	387	111	232	335	128	-1.387	0.180414946	1
ENSBTAG00000020199	*BT.69975*	2'277	1'319	1'747	1'972	1'523	-0.373	0.640925139	1
ENSBTAG00000020202	*CRHBP*	25	1	11	22	1	-4.229	0.384445569	1
ENSBTAG00000020203	*BT.34601*	29	3	14	25	3	-2.858	0.448507214	1
ENSBTAG00000020205	*PDE8B*	579	639	620	501	738	0.557	0.518985244	1
ENSBTAG00000020209	*TIGD7*	476	83	254	412	96	-2.105	0.042115662	1
ENSBTAG00000020212	*WDR41*	2'108	1'064	1'527	1'826	1'229	-0.571	0.477696903	1
ENSBTAG00000020213	*NIPAL4*	30	26	28	26	30	0.209	0.943045033	1
ENSBTAG00000020214	*LRRC8B*	192	441	338	166	509	1.615	0.093174211	1
ENSBTAG00000020215	*EXOC2*	2'677	2'253	2'460	2'318	2'602	0.166	0.832023391	1
ENSBTAG00000020218	*ANXA7*	1'310	853	1'060	1'134	985	-0.204	0.80527106	1

Ensembl gene ID	geneName	counts wildtype horn bud	counts polled horn bud	baseMean	baseMean wildtype horn bud	baseMean polled horn bud	log2FoldChange	pval	padj
ENSBTAG00000020219	*ZMYND17*	1'365	546	906	1'182	630	-0.907	0.278371341	1
ENSBTAG00000020221	*BT.88484*	897	667	774	777	770	-0.012	0.990779303	1
ENSBTAG00000020225	*BT.29646*	44	21	31	38	24	-0.652	0.776423402	1
ENSBTAG00000020226	*CHRAC1*	955	814	883	827	940	0.185	0.824919133	1
ENSBTAG00000020227	*FAM72A*	185	126	153	160	145	-0.139	0.90910389	1
ENSBTAG00000020229	*ATP8B3*	42	22	31	36	25	-0.518	0.825975862	1
ENSBTAG00000020232	*REXO1*	2'206	3'425	2'933	1'910	3'955	1.050	0.182668969	1
ENSBTAG00000020233	*C10ORF118*	1'034	532	755	895	614	-0.544	0.521565657	1
ENSBTAG00000020236	*NECAB2*	707	451	567	612	521	-0.234	0.791070469	1
ENSBTAG00000020237	*NECAP1*	3'153	1'572	2'273	2'731	1'815	-0.589	0.456096125	1
ENSBTAG00000020238	*RIMS1*	180	18	88	156	21	-2.907	0.046771881	1
ENSBTAG00000020242	*CRLF2*	2	0	1	2	0		0.974934741	1
ENSBTAG00000020243	*SVEP1*	7'688	9'766	8'967	6'658	11'277	0.760	0.322544819	1
ENSBTAG00000020244	*EFNA1*	2'436	2'330	2'400	2'110	2'690	0.351	0.655119377	1
ENSBTAG00000020247	*ADCYAP1R1*	502	204	335	435	236	-0.884	0.352705551	1
ENSBTAG00000020250	*BT.42063*	16	29	24	14	33	1.273	0.623253784	1
ENSBTAG00000020252	*NLGN2*	7'620	8'891	8'433	6'599	10'266	0.638	0.406199887	1
ENSBTAG00000020253	*BT.34275*	224	239	235	194	276	0.509	0.618006025	1
ENSBTAG00000020257	*PTPN5*	310	49	163	268	57	-2.246	0.053544787	1
ENSBTAG00000020260	*BT.43903*	377	220	290	326	254	-0.362	0.712219703	1
ENSBTAG00000020261	*CDH26*	8	2	5	7	2	-1.585	0.87391615	1
ENSBTAG00000020262	*PSME4*	3'751	2'132	2'855	3'248	2'462	-0.400	0.60979	1
ENSBTAG00000020263	*TIMP4*	198	113	151	171	130	-0.394	0.73419797	1
ENSBTAG00000020264	*C1ORF114*	63	17	37	55	20	-1.475	0.470234323	1
ENSBTAG00000020266	*SLC19A2*	236	172	201	204	199	-0.041	0.974850584	1
ENSBTAG00000020267	*WNT9A*	115	137	129	100	158	0.668	0.578883502	1
ENSBTAG00000020268	*RSF1*	1'224	796	990	1'060	919	-0.206	0.804632459	1
ENSBTAG00000020269	*BT.50219*	60	82	73	52	95	0.866	0.557587244	1
ENSBTAG00000020270	*BT.60919*	1'738	1'390	1'555	1'505	1'605	0.093	0.907924698	1
ENSBTAG00000020272	*SIK2*	864	865	874	748	999	0.417	0.617285213	1
ENSBTAG00000020273	*YOD1*	61	38	48	53	44	-0.268	0.890679042	1
ENSBTAG00000020277	*PPP2R1B*	1'075	841	951	931	971	0.061	0.941961594	1
ENSBTAG00000020279	*ALG9*	450	424	440	390	490	0.329	0.716276309	1
ENSBTAG00000020281	*NIN*	1'648	743	1'143	1'427	858	-0.734	0.370672094	1
ENSBTAG00000020282	*DCTD*	807	533	657	699	615	-0.183	0.832719107	1
ENSBTAG00000020283	*DUSP5*	60	40	49	52	46	-0.170	0.935382741	1
ENSBTAG00000020285	*PRG3*	5	2	3	4	2	-0.907	0.965043111	1
ENSBTAG00000020286	*FAM59A*	337	222	274	292	256	-0.187	0.852915628	1
ENSBTAG00000020289	*ZNF214*	75	41	56	65	47	-0.456	0.789763756	1
ENSBTAG00000020292	*C5H12ORF57*	5'183	5'574	5'462	4'489	6'436	0.520	0.500005366	1
ENSBTAG00000020294	*PTPN6*	584	503	543	506	581	0.200	0.8208102	1
ENSBTAG00000020296	*UBR3*	3'013	1'671	2'269	2'609	1'930	-0.435	0.581480184	1
ENSBTAG00000020297	*PQLC3*	171	137	153	148	158	0.095	0.938344086	1
ENSBTAG00000020298	*BT.99191*	10	1	5	9	1	-2.907	0.754028015	1
ENSBTAG00000020301	*BLM*	684	576	629	592	665	0.167	0.846991016	1
ENSBTAG00000020303	*FBXO38*	3'547	2'266	2'844	3'072	2'617	-0.231	0.76811344	1
ENSBTAG00000020304	*MTG1*	2'279	1'777	2'013	1'974	2'052	0.056	0.94344908	1
ENSBTAG00000020305	*TOR1A*	1'273	1'355	1'334	1'102	1'565	0.505	0.532207659	1
ENSBTAG00000020307	*C11H9ORF78*	2'380	1'924	2'141	2'061	2'222	0.108	0.890812381	1
ENSBTAG00000020308	*BT.68997*	48'704	33'273	40'300	42'179	38'420	-0.135	0.859905388	1
ENSBTAG00000020309	*G3BP*	3'953	3'115	3'510	3'423	3'597	0.071	0.926533618	1
ENSBTAG00000020311	*USP20*	2'273	2'089	2'190	1'968	2'412	0.293	0.709753176	1
ENSBTAG00000020312	*NCOA2*	718	529	616	622	611	-0.026	0.979137458	1
ENSBTAG00000020313	*FNBP1*	1'164	1'100	1'139	1'008	1'270	0.333	0.682927513	1
ENSBTAG00000020314	*COG2*	2'837	1'548	2'122	2'457	1'787	-0.459	0.562319467	1
ENSBTAG00000020315	*DENND5B*	103	52	75	89	60	-0.571	0.701150513	1
ENSBTAG00000020316	*BT.88309*	2'464	2'933	2'760	2'134	3'387	0.666	0.39552113	1
ENSBTAG00000020318	*DBNDD1*	941	1'610	1'337	815	1'859	1.190	0.145208098	1
ENSBTAG00000020319	*ALOX5*	316	183	242	274	211	-0.373	0.714866391	1
ENSBTAG00000020321	*SNX11*	752	1'069	943	651	1'234	0.922	0.268021238	1
ENSBTAG00000020327	*ZNF410*	3'525	2'206	2'800	3'053	2'547	-0.261	0.739343293	1
ENSBTAG00000020329	*BT.42682*	264	115	181	229	133	-0.784	0.473163024	1
ENSBTAG00000020330	*BTG1*	13'433	6'721	9'697	11'633	7'761	-0.584	0.446695062	1
ENSBTAG00000020331	*BT.52419*	1'450	456	891	1'256	527	-1.254	0.136859718	1
ENSBTAG00000020332	*OSBP*	1'331	1'833	1'635	1'153	2'117	0.877	0.275050567	1
ENSBTAG00000020334	*BT.103949*	879	575	713	761	664	-0.197	0.81841179	1
ENSBTAG00000020335	*RNF32*	12	10	11	10	12	0.152	0.995696634	1
ENSBTAG00000020338	*BT.53009*	78	37	55	68	43	-0.661	0.696762961	1
ENSBTAG00000020340	*ZNF830*	1'313	796	1'028	1'137	919	-0.307	0.71033287	1
ENSBTAG00000020341	*MOV10L1*	53	0	23	46	0		0.056788891	1
ENSBTAG00000020342	*MYOC*	254	293	279	220	338	0.621	0.526775135	1
ENSBTAG00000020344	*SLC44A3*	184	62	115	159	72	-1.154	0.357635614	1

Ensembl gene ID	geneName	counts wildtype horn bud	counts polled horn bud	baseMean	baseMean wildtype horn bud	baseMean polled horn bud	log2FoldChange	pval	padj
ENSBTAG00000020345	*CNN3*	11'520	8'409	9'843	9'977	9'710	-0.039	0.96002768	1
ENSBTAG00000020346	*CCDC92*	496	340	411	430	393	-0.130	0.890157216	1
ENSBTAG00000020348	*BT.43194*	1	1	1	1	1	0.415	1	1
ENSBTAG00000020350	*BT.44392*	21	10	15	18	12	-0.655	0.864027356	1
ENSBTAG00000020352	*PAQR5*	56	13	32	48	15	-1.692	0.446973729	1
ENSBTAG00000020353	*PFN3*	2	2	2	2	2	0.415	1	1
ENSBTAG00000020355	*KLF4*	4'588	1'956	3'116	3'973	2'259	-0.815	0.298704425	1
ENSBTAG00000020356	*BT.45280*	2'574	3'554	3'166	2'229	4'104	0.880	0.261224957	1
ENSBTAG00000020357	*GOLPH3L*	1'420	1'308	1'370	1'230	1'510	0.297	0.713110526	1
ENSBTAG00000020359	*BT.88368*	459	690	597	398	797	1.003	0.250227801	1
ENSBTAG00000020361	*SLC35F3*	51	48	50	44	55	0.328	0.858630251	1
ENSBTAG00000020363	*BT.55737*	3	3	3	3	3	0.415	1	1
ENSBTAG00000020366	*BT.20483*	1'580	1'927	1'797	1'368	2'225	0.701	0.379137506	1
ENSBTAG00000020367	*SLC30A9*	1'286	773	1'003	1'114	893	-0.319	0.699762342	1
ENSBTAG00000020368	*protein_coding*	251	565	435	217	652	1.586	0.085457398	1
ENSBTAG00000020369	*NOXO1*	89	26	54	77	30	-1.360	0.425217196	1
ENSBTAG00000020371	*BT.43859*	610	754	699	528	871	0.721	0.398563079	1
ENSBTAG00000020373	*C3ORF67*	102	79	90	88	91	0.046	0.981185554	1
ENSBTAG00000020374	*THSD1*	766	517	630	663	597	-0.152	0.862089372	1
ENSBTAG00000020375	*protein_coding*	3	2	2	3	2	-0.170	1	1
ENSBTAG00000020376	*PAIP1*	1'923	782	1'284	1'665	903	-0.883	0.278753528	1
ENSBTAG00000020378	*DLG1*	3'317	2'032	2'609	2'873	2'346	-0.292	0.710476303	1
ENSBTAG00000020379	*BT.96759*	1'021	1'309	1'198	884	1'512	0.774	0.343472873	1
ENSBTAG00000020381	*FCF1*	462	336	394	400	388	-0.044	0.965270046	1
ENSBTAG00000020382	*RIN2*	2'334	1'285	1'753	2'021	1'484	-0.446	0.576597682	1
ENSBTAG00000020384	*WDR47*	2'251	1'163	1'646	1'949	1'343	-0.538	0.502273894	1
ENSBTAG00000020385	*ALDH1B1*	7	17	13	6	20	1.695	0.660205265	1
ENSBTAG00000020387	*MARF1*	1'033	1'311	1'204	895	1'514	0.759	0.352430421	1
ENSBTAG00000020389	*BT.48227*	2'089	1'446	1'739	1'809	1'670	-0.116	0.885813479	1
ENSBTAG00000020390	*ARL13A*	30	10	19	26	12	-1.170	0.701297322	1
ENSBTAG00000020391	*BT.29198*	7	1	4	6	1	-2.392	0.851750348	1
ENSBTAG00000020393	*METTL21C*	3	5	4	3	6	1.152	0.920866718	1
ENSBTAG00000020394	*TNFAIP8L2*	126	93	108	109	107	-0.023	0.994899361	1
ENSBTAG00000020395	*BT.68733*	918	654	775	795	755	-0.074	0.932144528	1
ENSBTAG00000020397	*APCDD1L*	897	1'636	1'333	777	1'889	1.282	0.117322324	1
ENSBTAG00000020398	*TRMT12*	594	384	479	514	443	-0.214	0.812631857	1
ENSBTAG00000020399	*RNF139*	2'270	1'464	1'828	1'966	1'690	-0.218	0.785206313	1
ENSBTAG00000020400	*GJA3*	3	3	3	3	3	0.415	1	1
ENSBTAG00000020402	*TDRD9*	16	34	27	14	39	1.503	0.535161027	1
ENSBTAG00000020403	*ZMYM2*	5'882	4'510	5'151	5'094	5'208	0.032	0.966620391	1
ENSBTAG00000020405	*NDUFB9*	2'131	2'065	2'115	1'846	2'384	0.370	0.639596984	1
ENSBTAG00000020406	*GPC3*	33'555	49'672	43'208	29'059	57'356	0.981	0.199898398	1
ENSBTAG00000020407	*MTSS1*	7'239	2'646	4'662	6'269	3'055	-1.037	0.183481407	1
ENSBTAG00000020409	*C1H21ORF56*	0	3	2	0	3	Inf	0.878646179	1
ENSBTAG00000020410	*UFL1*	1'733	829	1'229	1'501	957	-0.649	0.426317791	1
ENSBTAG00000020412	*RAP2A*	961	402	648	832	464	-0.842	0.32950013	1
ENSBTAG00000020413	*APBA2*	998	1'059	1'044	864	1'223	0.501	0.542589755	1
ENSBTAG00000020414	*DHX37*	1'347	1'632	1'526	1'167	1'884	0.692	0.389452277	1
ENSBTAG00000020415	*KLK4*	47	15	29	41	17	-1.233	0.596986546	1
ENSBTAG00000020416	*PGAP1*	38	20	28	33	23	-0.511	0.838636924	1
ENSBTAG00000020417	*DAPK3*	2'757	3'289	3'093	2'388	3'798	0.670	0.391718467	1
ENSBTAG00000020418	*CA5B*	381	350	367	330	404	0.293	0.754142879	1
ENSBTAG00000020419	*BT.21842*	840	303	539	727	350	-1.056	0.233233495	1
ENSBTAG00000020420	*AP1S2*	1'119	933	1'023	969	1'077	0.153	0.852961306	1
ENSBTAG00000020421	*SUPT16H*	6'594	5'505	6'034	5'711	6'357	0.155	0.840119383	1
ENSBTAG00000020422	*CHD8*	3'718	3'500	3'631	3'220	4'041	0.328	0.672545566	1
ENSBTAG00000020423	*EP400*	1'499	1'399	1'457	1'298	1'615	0.315	0.694650949	1
ENSBTAG00000020425	*TFAP2D*	39	1	17	34	1	-4.870	0.199492463	1
ENSBTAG00000020426	*TFAP2B*	774	1'409	1'149	670	1'627	1.279	0.121600977	1
ENSBTAG00000020427	*DNTT*	5	4	4	4	5	0.093	1	1
ENSBTAG00000020428	*BT.97093*	1'296	1'242	1'278	1'122	1'434	0.354	0.662425302	1
ENSBTAG00000020429	*MED29*	781	899	857	676	1'038	0.618	0.459922488	1
ENSBTAG00000020430	*GLT1D1*	90	6	42	78	7	-3.492	0.091486521	1
ENSBTAG00000020431	*DERL2*	2'019	1'803	1'915	1'749	2'082	0.252	0.750616542	1
ENSBTAG00000020432	*MIS12*	871	608	728	754	702	-0.104	0.905054286	1
ENSBTAG00000020433	*NLRP1*	81	16	44	70	18	-1.925	0.307763662	1
ENSBTAG00000020434	*C18H19ORF12*	1'407	1'225	1'317	1'218	1'415	0.215	0.790114312	1
ENSBTAG00000020436	*BT.38287*	1'327	586	913	1'149	677	-0.764	0.359929019	1
ENSBTAG00000020439	*BUD31*	2'714	2'071	2'371	2'350	2'391	0.025	0.974596359	1
ENSBTAG00000020440	*BT.62782*	808	854	843	700	986	0.495	0.55424636	1
ENSBTAG00000020441	*HMG20A*	4'006	3'410	3'703	3'469	3'938	0.183	0.8135175	1
ENSBTAG00000020444	*MAST2*	7'650	6'230	6'909	6'625	7'194	0.119	0.876451233	1

Ensembl gene ID	geneName	counts wildtype horn bud	counts polled horn bud	baseMean	baseMean wildtype horn bud	baseMean polled horn bud	log2FoldChange	pval	padj
ENSBTAG00000020445	*ZNF398*	168	207	192	145	239	0.716	0.503507064	1
ENSBTAG00000020446	*THOP1*	1'071	1'575	1'373	928	1'819	0.971	0.23163013	1
ENSBTAG00000020447	*VSIG10L*	223	174	197	193	201	0.057	0.961516409	1
ENSBTAG00000020448	*ZNF282*	392	571	499	339	659	0.958	0.282882092	1
ENSBTAG00000020449	*ETFB*	2'498	2'945	2'782	2'163	3'401	0.653	0.40524165	1
ENSBTAG00000020450	*CLDND2*	20	12	16	17	14	-0.322	0.942838359	1
ENSBTAG00000020451	*BT.40485*	3'387	2'266	2'775	2'933	2'617	-0.165	0.834108409	1
ENSBTAG00000020453	*CLEC5A*	21	9	14	18	10	-0.807	0.832834108	1
ENSBTAG00000020454	*BT.41549*	1'848	2'467	2'225	1'600	2'849	0.832	0.293571248	1
ENSBTAG00000020455	*BT.22305*	2'131	842	1'409	1'846	972	-0.925	0.254409101	1
ENSBTAG00000020456	*AGPAT3*	721	765	754	624	883	0.500	0.55402412	1
ENSBTAG00000020457	*TGFB1*	1'667	1'628	1'662	1'444	1'880	0.381	0.633200985	1
ENSBTAG00000020458	*STX6*	950	1'253	1'135	823	1'447	0.814	0.320608417	1
ENSBTAG00000020459	*TIMELESS*	952	1'106	1'051	824	1'277	0.631	0.442822756	1
ENSBTAG00000020465	*FERMT1*	381	366	376	330	423	0.357	0.700629044	1
ENSBTAG00000020466	*BT.103992*	24	3	12	21	3	-2.585	0.53693803	1
ENSBTAG00000020467	*HRH4*	7	5	6	6	6	-0.070	1	1
ENSBTAG00000020468	*EFHA2*	389	104	228	337	120	-1.488	0.152877435	1
ENSBTAG00000020469	*TMED8*	16	14	15	14	16	0.222	0.967786569	1
ENSBTAG00000020471	*SAMD15*	34	9	20	29	10	-1.503	0.606713166	1
ENSBTAG00000020472	*KDM5A*	608	485	543	527	560	0.089	0.920557299	1
ENSBTAG00000020474	*NOXRED1*	4	1	2	3	1	-1.585	0.952247636	1
ENSBTAG00000020475	*SPE39*	2'223	2'055	2'149	1'925	2'373	0.302	0.702046464	1
ENSBTAG00000020477	*AHSA1*	4'666	3'751	4'186	4'041	4'331	0.100	0.896647015	1
ENSBTAG00000020480	*SPTLC2*	613	535	574	531	618	0.219	0.802456703	1
ENSBTAG00000020481	*GXYLT2*	1'042	690	850	902	797	-0.180	0.831564689	1
ENSBTAG00000020482	*ASCC3*	1'902	1'204	1'519	1'647	1'390	-0.245	0.761532075	1
ENSBTAG00000020484	*ADCK2*	511	807	687	443	932	1.074	0.211574168	1
ENSBTAG00000020485	*ARRB1*	1'006	1'405	1'247	871	1'622	0.897	0.271604529	1
ENSBTAG00000020489	*SEMA6A*	2'072	1'861	1'972	1'794	2'149	0.260	0.742373466	1
ENSBTAG00000020490	*NACC2*	104	95	100	90	110	0.284	0.832503166	1
ENSBTAG00000020491	*SOX30*	4	1	2	3	1	-1.585	0.952247636	1
ENSBTAG00000020494	*RPE65*	2	0	1	2	0		0.974934741	1
ENSBTAG00000020495	*BT.54567*	1'934	1'092	1'468	1'675	1'261	-0.410	0.61144503	1
ENSBTAG00000020496	*C23H6ORF62*	9'917	3'317	6'209	8'588	3'830	-1.165	0.134179304	1
ENSBTAG00000020498	*ACP1*	1'697	999	1'312	1'470	1'154	-0.349	0.66697485	1
ENSBTAG00000020499	*PLA2G2D3*	169	117	141	146	135	-0.115	0.928122423	1
ENSBTAG00000020503	*C12ORF65*	437	325	377	378	375	-0.012	0.993633533	1
ENSBTAG00000020504	*CDK2AP1*	3'159	2'641	2'893	2'736	3'050	0.157	0.840719445	1
ENSBTAG00000020505	*SBNO1*	772	496	621	669	573	-0.223	0.79792145	1
ENSBTAG00000020508	*pseudogene*	14	1	7	12	1	-3.392	0.634690491	1
ENSBTAG00000020510	*EPHA10*	33	7	18	29	8	-1.822	0.554890505	1
ENSBTAG00000020512	*GJB1*	13	8	10	11	9	-0.285	0.973425765	1
ENSBTAG00000020514	*FAM169B*	20	31	27	17	36	1.047	0.666651314	1
ENSBTAG00000020515	*FAM160A2*	665	683	682	576	789	0.454	0.595553547	1
ENSBTAG00000020516	*C9ORF153*	2	0	1	2	0		0.974934741	1
ENSBTAG00000020517	*MED9*	616	812	736	533	938	0.814	0.338713225	1
ENSBTAG00000020518	*PARK7*	6'964	6'063	6'516	6'031	7'001	0.215	0.778898836	1
ENSBTAG00000020520	*BT.48977*	190	137	161	165	158	-0.057	0.966506003	1
ENSBTAG00000020522	*BT.24806*	2	0	1	2	0		0.974934741	1
ENSBTAG00000020523	*SLC10A5*	273	189	227	236	218	-0.115	0.915296905	1
ENSBTAG00000020524	*BT.34351*	690	664	682	598	767	0.360	0.673881924	1
ENSBTAG00000020525	*SEC31A*	14'719	10'045	12'173	12'747	11'599	-0.136	0.859133356	1
ENSBTAG00000020527	*IDH1*	4'052	5'127	4'715	3'509	5'920	0.755	0.330395984	1
ENSBTAG00000020528	*PCOLCE*	73'773	35'228	52'284	63'889	40'678	-0.651	0.392768694	1
ENSBTAG00000020530	*MTIF3*	1'012	574	770	876	663	-0.403	0.634210067	1
ENSBTAG00000020532	*BT.35939*	165	163	166	143	188	0.397	0.722881193	1
ENSBTAG00000020533	*FBXO34*	797	729	766	690	842	0.286	0.734551969	1
ENSBTAG00000020535	*PYCARD*	216	121	163	187	140	-0.421	0.709854918	1
ENSBTAG00000020536	*HERC6*	489	125	284	423	144	-1.553	0.118463668	1
ENSBTAG00000020537	*ACCN4*	81	41	59	70	47	-0.567	0.731918912	1
ENSBTAG00000020538	*HERC5*	381	240	304	330	277	-0.252	0.797025654	1
ENSBTAG00000020539	*CHPF*	8'042	9'751	9'112	6'965	11'259	0.693	0.366602923	1
ENSBTAG00000020540	*TMEM198*	102	54	75	88	62	-0.503	0.735670952	1
ENSBTAG00000020541	*BT.67700*	2'466	1'357	1'851	2'136	1'567	-0.447	0.575004354	1
ENSBTAG00000020542	*BT.44067*	228	156	189	197	180	-0.132	0.907335645	1
ENSBTAG00000020543	*UPK1A*	24	18	21	21	21	0.000	1	1
ENSBTAG00000020544	*BCL7B*	807	714	762	699	824	0.238	0.7779938	1
ENSBTAG00000020546	*TRDMT1*	1'203	669	907	1'042	772	-0.432	0.604899784	1
ENSBTAG00000020547	*BCAP31*	6'179	7'248	6'860	5'351	8'369	0.645	0.401766018	1
ENSBTAG00000020548	*ADC*	215	98	150	186	113	-0.718	0.533331101	1
ENSBTAG00000020551	*ABCD1*	432	722	604	374	834	1.156	0.185819722	1

Ensembl gene ID	geneName	counts wildtype horn bud	counts polled horn bud	baseMean	baseMean wildtype horn bud	baseMean polled horn bud	log2FoldChange	pval	padj
ENSBTAG00000020552	*PRDM2*	1'451	1'371	1'420	1'257	1'583	0.333	0.678841348	1
ENSBTAG00000020554	*AIF1*	637	247	418	552	285	-0.952	0.299264245	1
ENSBTAG00000020555	*BT.54136*	0	3	2	0	3	Inf	0.878646179	1
ENSBTAG00000020556	*APOC4*	1	0	0	1	0		1	1
ENSBTAG00000020559	*STMND1*	4	1	2	3	1	-1.585	0.952247636	1
ENSBTAG00000020560	*BT.91082*	2'060	2'800	2'509	1'784	3'233	0.858	0.276888858	1
ENSBTAG00000020561	*KLHL15*	242	182	210	210	210	0.004	1	1
ENSBTAG00000020563	*DDX59*	712	495	594	617	572	-0.109	0.902181379	1
ENSBTAG00000020566	*BCR*	1'086	1'728	1'468	941	1'995	1.085	0.180759903	1
ENSBTAG00000020567	*HNRPLL*	2'992	1'566	2'200	2'591	1'808	-0.519	0.511796556	1
ENSBTAG00000020568	*NKX2-5*	1	10	6	1	12	3.737	0.613387923	1
ENSBTAG00000020569	*CACNA2D1*	442	209	312	383	241	-0.666	0.489507222	1
ENSBTAG00000020570	*PRDM8*	1'133	579	825	981	669	-0.553	0.510507483	1
ENSBTAG00000020575	*IFITM5*	2'931	1'070	1'887	2'538	1'236	-1.039	0.194602681	1
ENSBTAG00000020578	*PRDM6*	105	17	55	91	20	-2.212	0.198338736	1
ENSBTAG00000020580	*TCN1*	2	0	1	2	0		0.974934741	1
ENSBTAG00000020582	*BT.29351*	151	169	163	131	195	0.578	0.606392055	1
ENSBTAG00000020583	*AASDH*	1'016	506	732	880	584	-0.591	0.487643764	1
ENSBTAG00000020584	*IFT81*	1'046	473	726	906	546	-0.730	0.391831617	1
ENSBTAG00000020589	*ENTPD1*	104	183	151	90	211	1.230	0.285114333	1
ENSBTAG00000020590	*FZD2*	2'042	3'511	2'911	1'768	4'054	1.197	0.129862766	1
ENSBTAG00000020591	*VAMP4*	586	274	412	507	316	-0.682	0.457240809	1
ENSBTAG00000020592	*BT.63808*	31	0	13	27	0		0.194281724	1
ENSBTAG00000020594	*ELF2*	3'317	1'906	2'537	2'873	2'201	-0.384	0.625258713	1
ENSBTAG00000020595	*KCTD20*	6'970	5'660	6'286	6'036	6'536	0.115	0.880861716	1
ENSBTAG00000020596	*TMEM126B*	564	260	394	488	300	-0.702	0.447103531	1
ENSBTAG00000020597	*FMO3*	80	15	43	69	17	-2.000	0.295736437	1
ENSBTAG00000020600	*BT.45701*	727	329	505	630	380	-0.729	0.412875636	1
ENSBTAG00000020601	*ZNF366*	83	21	48	72	24	-1.568	0.383684453	1
ENSBTAG00000020602	*BT.19792*	8	0	3	7	0		0.72953145	1
ENSBTAG00000020605	*SMTNL2*	2'026	2'311	2'212	1'755	2'669	0.605	0.443695535	1
ENSBTAG00000020606	*ZFAND2B*	1'727	1'360	1'533	1'496	1'570	0.070	0.930150736	1
ENSBTAG00000020607	*ABCB6*	1'079	923	1'000	934	1'066	0.190	0.818136836	1
ENSBTAG00000020608	*ATG9A*	2'437	3'531	3'094	2'111	4'077	0.950	0.226262914	1
ENSBTAG00000020609	*pseudogene*	80	278	195	69	321	2.212	0.044865759	1
ENSBTAG00000020610	*ANKZF1*	1'545	1'398	1'476	1'338	1'614	0.271	0.735900757	1
ENSBTAG00000020611	*BT.44278*	3'146	2'497	2'804	2'725	2'883	0.082	0.916506292	1
ENSBTAG00000020612	*NEK3*	1'006	466	705	871	538	-0.695	0.416081315	1
ENSBTAG00000020613	*STK16*	1'434	1'627	1'560	1'242	1'879	0.597	0.45657652	1
ENSBTAG00000020614	*BT.50439*	439	262	341	380	303	-0.330	0.729005677	1
ENSBTAG00000020616	*DGKH*	1'765	2'291	2'087	1'529	2'645	0.791	0.318600941	1
ENSBTAG00000020617	*TSC2*	6'229	9'980	8'459	5'394	11'524	1.095	0.156805276	1
ENSBTAG00000020619	*PKD1*	7'050	12'900	10'501	6'105	14'896	1.287	0.096871023	1
ENSBTAG00000020620	*processed_pseudogene*	259	397	341	224	458	1.031	0.276539893	1
ENSBTAG00000020622	*BT.4235*	293	287	293	254	331	0.385	0.692392658	1
ENSBTAG00000020626	*BT.26212*	2'849	1'530	2'117	2'467	1'767	-0.482	0.542988037	1
ENSBTAG00000020629	*UTP18*	1'245	696	941	1'078	804	-0.424	0.610153219	1
ENSBTAG00000020630	*BT.22536*	1'507	1'111	1'294	1'305	1'283	-0.025	0.977352221	1
ENSBTAG00000020633	*BT.49199*	1'822	1'603	1'714	1'578	1'851	0.230	0.772456823	1
ENSBTAG00000020634	*BT.21932*	1'872	1'213	1'511	1'621	1'401	-0.211	0.793815235	1
ENSBTAG00000020635	*VPS45*	2'360	1'830	2'078	2'044	2'113	0.048	0.951414161	1
ENSBTAG00000020636	*SYN3*	24	5	13	21	6	-1.848	0.630822808	1
ENSBTAG00000020638	*TIMP3*	5'677	2'779	4'063	4'916	3'209	-0.616	0.428524017	1
ENSBTAG00000020641	*PSMA5*	3'143	2'418	2'757	2'722	2'792	0.037	0.962380686	1
ENSBTAG00000020642	*ZDHHC13*	1'142	655	873	989	756	-0.387	0.643927537	1
ENSBTAG00000020643	*BT.27698*	2'009	2'703	2'431	1'740	3'121	0.843	0.285611363	1
ENSBTAG00000020644	*BT.19508*	2'210	2'057	2'145	1'914	2'375	0.312	0.692832961	1
ENSBTAG00000020645	*GNAI2*	15'436	16'839	16'406	13'368	19'444	0.541	0.478532555	1
ENSBTAG00000020646	*ZC3H6*	636	406	510	551	469	-0.233	0.794941197	1
ENSBTAG00000020647	*RASL11B*	2'869	786	1'696	2'485	908	-1.453	0.073483424	1
ENSBTAG00000020648	*SCFD2*	985	728	847	853	841	-0.021	0.982140116	1
ENSBTAG00000020649	*CIITA*	185	127	153	160	147	-0.128	0.917043716	1
ENSBTAG00000020650	*ADCYAP1*	6	7	7	5	8	0.637	0.939319729	1
ENSBTAG00000020652	*TSSK3*	72	83	79	62	96	0.620	0.666239587	1
ENSBTAG00000020653	*FIP1L1*	3'694	2'545	3'069	3'199	2'939	-0.122	0.876215733	1
ENSBTAG00000020654	*BAZ2B*	1'812	685	1'180	1'569	791	-0.988	0.228679784	1
ENSBTAG00000020655	*BSDC1*	3'026	2'856	2'959	2'621	3'298	0.332	0.670734232	1
ENSBTAG00000020657	*FAT1*	7'526	4'706	5'976	6'518	5'434	-0.262	0.73382252	1
ENSBTAG00000020658	*LNX1*	609	162	357	527	187	-1.495	0.115890813	1
ENSBTAG00000020661	*BT.89249*	1'245	840	1'024	1'078	970	-0.153	0.854479742	1
ENSBTAG00000020662	*CIP29*	2'511	1'721	2'081	2'175	1'987	-0.130	0.870534783	1
ENSBTAG00000020663	*ORMDL2*	2'033	1'422	1'701	1'761	1'642	-0.101	0.900909263	1

Ensembl gene ID	geneName	counts wildtype horn bud	counts polled horn bud	baseMean	baseMean wildtype horn bud	baseMean polled horn bud	log2FoldChange	pval	padj
ENSBTAG00000020664	*DNAJC14*	3'923	3'885	3'942	3'397	4'486	0.401	0.604754912	1
ENSBTAG00000020665	*BT.37170*	680	1'163	966	589	1'343	1.189	0.15453687	1
ENSBTAG00000020667	*KCNQ3*	85	40	60	74	46	-0.672	0.680117767	1
ENSBTAG00000020668	*pseudogene*	4'739	1'747	3'061	4'104	2'017	-1.025	0.192933697	1
ENSBTAG00000020669	*PROKR1*	27	9	17	23	10	-1.170	0.720958498	1
ENSBTAG00000020671	*TRIM62*	147	252	209	127	291	1.193	0.257647279	1
ENSBTAG00000020674	*LTB*	261	89	164	226	103	-1.137	0.312092039	1
ENSBTAG00000020679	*CNTN1*	622	141	351	539	163	-1.726	0.072210048	1
ENSBTAG00000020681	*FNDC3A*	2'559	1'358	1'892	2'216	1'568	-0.499	0.530689563	1
ENSBTAG00000020684	*processed_pseudogene*	354	480	430	307	554	0.854	0.347698843	1
ENSBTAG00000020685	*protein_coding*	2'050	3'129	2'694	1'775	3'613	1.025	0.194065925	1
ENSBTAG00000020688	*ARHGEF15*	493	359	421	427	415	-0.043	0.966305205	1
ENSBTAG00000020689	*PPP1R13L*	3'031	3'951	3'594	2'625	4'562	0.797	0.306689662	1
ENSBTAG00000020693	*BT.8261*	1'364	991	1'163	1'181	1'144	-0.046	0.956982505	1
ENSBTAG00000020696	*HECW2*	511	253	367	443	292	-0.599	0.521469925	1
ENSBTAG00000020698	*BT.26830*	3'169	3'262	3'256	2'744	3'767	0.457	0.557540599	1
ENSBTAG00000020699	*TSPAN11*	1'182	1'290	1'257	1'024	1'490	0.541	0.505040295	1
ENSBTAG00000020700	*CLCN6*	355	644	526	307	744	1.274	0.152696144	1
ENSBTAG00000020701	*MEF2C*	1'157	1'113	1'144	1'002	1'285	0.359	0.659940034	1
ENSBTAG00000020704	*RAMP3*	132	248	200	114	286	1.325	0.214626199	1
ENSBTAG00000020705	*PRSS12*	1'216	1'933	1'643	1'053	2'232	1.084	0.178748531	1
ENSBTAG00000020707	*GPR126*	435	361	397	377	417	0.146	0.875286538	1
ENSBTAG00000020709	*BT.53370*	470	361	412	407	417	0.034	0.972175603	1
ENSBTAG00000020710	*CENPQ*	482	220	336	417	254	-0.716	0.450659706	1
ENSBTAG00000020713	*BT.79862*	44	84	68	38	97	1.348	0.378618205	1
ENSBTAG00000020714	*BT.37736*	1'133	808	957	981	933	-0.073	0.931961804	1
ENSBTAG00000020715	*PIK3C2G*	348	187	259	301	216	-0.481	0.631471201	1
ENSBTAG00000020717	*BT.31511*	823	520	657	713	600	-0.247	0.77487871	1
ENSBTAG00000020720	*MMTAG2*	1'444	1'089	1'254	1'251	1'257	0.008	0.992604596	1
ENSBTAG00000020721	*MRPL55*	1'312	1'112	1'210	1'136	1'284	0.176	0.828283218	1
ENSBTAG00000020726	*ARHGEF7*	2'724	3'456	3'175	2'359	3'991	0.758	0.332223589	1
ENSBTAG00000020729	*DHRS7*	454	230	329	393	266	-0.566	0.552697757	1
ENSBTAG00000020731	*PRRG4*	49	64	58	42	74	0.800	0.62390206	1
ENSBTAG00000020733	*RPS15A*	1'783	1'485	1'629	1'544	1'715	0.151	0.849845954	1
ENSBTAG00000020734	*BT.64555*	2'460	1'508	1'936	2'130	1'741	-0.291	0.714589368	1
ENSBTAG00000020735	*SMG1*	1'631	828	1'184	1'412	956	-0.563	0.490839731	1
ENSBTAG00000020736	*BT.42498*	298	185	236	258	214	-0.273	0.792101531	1
ENSBTAG00000020737	*SOX8*	245	106	167	212	122	-0.794	0.47708752	1
ENSBTAG00000020739	*NXT2*	945	1'070	1'027	818	1'236	0.594	0.47068748	1
ENSBTAG00000020742	*FZD9*	123	147	138	107	170	0.672	0.567811423	1
ENSBTAG00000020745	*HIVEP3*	160	68	109	139	79	-0.819	0.521965618	1
ENSBTAG00000020747	*ASS1*	362	605	506	314	699	1.156	0.195657955	1
ENSBTAG00000020748	*FAM76B*	656	414	523	568	478	-0.249	0.779735907	1
ENSBTAG00000020749	*AAED1*	1'041	504	742	902	582	-0.631	0.457569538	1
ENSBTAG00000020750	*AFF2*	43	17	28	37	20	-0.924	0.697473507	1
ENSBTAG00000020751	*HSF1*	2'239	2'945	2'670	1'939	3'401	0.810	0.302944311	1
ENSBTAG00000020752	*CEP78*	760	492	613	658	568	-0.212	0.808026276	1
ENSBTAG00000020754	*ZNF526*	515	766	665	446	885	0.988	0.251605021	1
ENSBTAG00000020755	*SELP*	1'182	388	736	1'024	448	-1.192	0.164215905	1
ENSBTAG00000020756	*GSK3A*	2'510	3'075	2'862	2'174	3'551	0.708	0.36647874	1
ENSBTAG00000020757	*PCBP2*	20'719	16'854	18'702	17'943	19'461	0.117	0.877040841	1
ENSBTAG00000020758	*MAP3K12*	3'438	2'860	3'140	2'977	3'302	0.149	0.847528749	1
ENSBTAG00000020759	*NCLN*	1'373	2'091	1'802	1'189	2'414	1.022	0.202317587	1
ENSBTAG00000020760	*SNAP29*	636	589	615	551	680	0.304	0.725040409	1
ENSBTAG00000020761	*USP33*	5'096	1'966	3'342	4'413	2'270	-0.959	0.221408947	1
ENSBTAG00000020764	*CNN2*	4'672	5'503	5'200	4'046	6'354	0.651	0.399390383	1
ENSBTAG00000020766	*ABCA7*	597	237	395	517	274	-0.918	0.321006412	1
ENSBTAG00000020769	*OPCML*	91	101	98	79	117	0.565	0.669964536	1
ENSBTAG00000020772	*HMHA1*	328	257	290	284	297	0.063	0.951212177	1
ENSBTAG00000020773	*CTSW*	85	27	52	74	31	-1.239	0.471403281	1
ENSBTAG00000020774	*processed_pseudogene*	37	49	44	32	57	0.820	0.658516845	1
ENSBTAG00000020775	*FIBP*	1'431	1'499	1'485	1'239	1'731	0.482	0.548547035	1
ENSBTAG00000020776	*POLR2E*	4'474	5'453	5'086	3'875	6'297	0.701	0.365161264	1
ENSBTAG00000020777	*ERCC3*	3'114	2'189	2'612	2'697	2'528	-0.093	0.906196002	1
ENSBTAG00000020780	*SBNO2*	2'366	2'924	2'713	2'049	3'376	0.721	0.358884532	1
ENSBTAG00000020781	*SETD6*	1'563	707	1'085	1'354	816	-0.729	0.375509878	1
ENSBTAG00000020782	*CNOT1*	8'646	6'394	7'435	7'488	7'383	-0.020	0.979778318	1
ENSBTAG00000020783	*MAPK14*	5'490	3'019	4'120	4'754	3'486	-0.448	0.564222928	1
ENSBTAG00000020784	*pseudogene*	98	162	136	85	187	1.140	0.336087948	1
ENSBTAG00000020787	*PIK3R4*	4'479	2'551	3'412	3'879	2'946	-0.397	0.61064371	1
ENSBTAG00000020788	*SLC18A1*	0	1	1	0	1	Inf	0.993540919	1
ENSBTAG00000020789	*STS*	24	26	25	21	30	0.531	0.839166484	1

Ensembl gene ID	geneName	counts wildtype horn bud	counts polled horn bud	baseMean	baseMean wildtype horn bud	baseMean polled horn bud	log2FoldChange	pval	padj
ENSBTAG00000020790	*pseudogene*	579	723	668	501	835	0.735	0.391423521	1
ENSBTAG00000020791	*RAPGEF1*	1'629	3'340	2'634	1'411	3'857	1.451	0.068816302	1
ENSBTAG00000020792	*TPH2*	2	1	1	2	1	-0.585	1	1
ENSBTAG00000020793	*KCNA4*	113	13	56	98	15	-2.705	0.119858038	1
ENSBTAG00000020795	*RPS21*	25'693	20'717	23'086	22'251	23'922	0.104	0.890096392	1
ENSBTAG00000020796	*UBE2D1*	976	694	823	845	801	-0.077	0.929046479	1
ENSBTAG00000020797	*CABLES2*	496	579	549	430	669	0.638	0.466971212	1
ENSBTAG00000020798	*C1ORF94*	2	0	1	2	0		0.974934741	1
ENSBTAG00000020799	*BT.64101*	753	1'116	970	652	1'289	0.983	0.237440827	1
ENSBTAG00000020800	*BEND7*	95	30	58	82	35	-1.248	0.446094408	1
ENSBTAG00000020801	*FAM49B*	1'446	756	1'063	1'252	873	-0.521	0.527252621	1
ENSBTAG00000020802	*BT.66235*	410	111	242	355	128	-1.470	0.152478182	1
ENSBTAG00000020803	*protein_coding*	71	110	94	61	127	1.047	0.434605542	1
ENSBTAG00000020806	*HELB*	807	397	579	699	458	-0.608	0.486319323	1
ENSBTAG00000020807	*FAU*	10'340	4'398	7'017	8'955	5'078	-0.818	0.289227277	1
ENSBTAG00000020810	*CYB561*	356	201	270	308	232	-0.410	0.680544765	1
ENSBTAG00000020813	*BT.28262*	1	0	0	1	0		1	1
ENSBTAG00000020814	*C2CD4A*	20	16	18	17	18	0.093	0.996214168	1
ENSBTAG00000020815	*UHRF2*	3'411	1'486	2'335	2'954	1'716	-0.784	0.321834061	1
ENSBTAG00000020819	*YY1*	2'437	1'427	1'879	2'111	1'648	-0.357	0.653852713	1
ENSBTAG00000020824	*KRT10*	30'711	14'824	21'857	26'597	17'117	-0.636	0.40522631	1
ENSBTAG00000020825	*IRAK1BP1*	298	181	234	258	209	-0.304	0.768659995	1
ENSBTAG00000020826	*CHIC1*	109	94	101	94	109	0.201	0.882544764	1
ENSBTAG00000020828	*SH2D6*	13	4	8	11	5	-1.285	0.828302954	1
ENSBTAG00000020829	*PTPRK*	3'942	1'765	2'726	3'414	2'038	-0.744	0.344084017	1
ENSBTAG00000020831	*BT.18335*	1'865	822	1'282	1'615	949	-0.767	0.346242709	1
ENSBTAG00000020835	*KIAA0513*	194	364	294	168	420	1.323	0.177044153	1
ENSBTAG00000020836	*FAM92B*	13	0	6	11	0		0.551229012	1
ENSBTAG00000020837	*ARHGEF16*	310	327	323	268	378	0.492	0.605822056	1
ENSBTAG00000020839	*MEGF6*	2'508	4'363	3'605	2'172	5'038	1.214	0.122561297	1
ENSBTAG00000020843	*PCSK1*	43	15	27	37	17	-1.104	0.648166995	1
ENSBTAG00000020844	*TMEM246*	847	356	572	734	411	-0.835	0.340497734	1
ENSBTAG00000020848	*PIK3CG*	195	62	120	169	72	-1.238	0.317899105	1
ENSBTAG00000020850	*MUL1*	665	1'208	985	576	1'395	1.276	0.126684858	1
ENSBTAG00000020852	*SLC16A11*	344	251	294	298	290	-0.040	0.972043553	1
ENSBTAG00000020853	*SLC16A13*	519	250	369	449	289	-0.639	0.493940647	1
ENSBTAG00000020854	*BCL6B*	419	255	329	363	294	-0.301	0.753463418	1
ENSBTAG00000020855	*PTPN4*	145	60	97	126	69	-0.858	0.51887617	1
ENSBTAG00000020856	*HFM1*	76	10	39	66	12	-2.511	0.223047709	1
ENSBTAG00000020858	*UPF3A*	710	413	546	615	477	-0.367	0.677557179	1
ENSBTAG00000020859	*SERPINF2*	29	17	22	25	20	-0.355	0.909573651	1
ENSBTAG00000020860	*CHD1*	3'816	1'553	2'549	3'305	1'793	-0.882	0.264054256	1
ENSBTAG00000020861	*CHAMP1*	1'566	935	1'218	1'356	1'080	-0.329	0.686836347	1
ENSBTAG00000020865	*PELI1*	2'045	1'905	1'985	1'771	2'200	0.313	0.692656154	1
ENSBTAG00000020872	*BT.34208*	42	86	68	36	99	1.449	0.343884644	1
ENSBTAG00000020873	*MAT2A*	19'151	8'727	13'331	16'585	10'077	-0.719	0.348369538	1
ENSBTAG00000020878	*DMTF1*	4'279	2'270	3'163	3'706	2'621	-0.500	0.522758035	1
ENSBTAG00000020880	*BT.51579*	1'924	1'594	1'753	1'666	1'841	0.144	0.856779266	1
ENSBTAG00000020884	*CASP4*	1'414	382	833	1'225	441	-1.473	0.08388708	1
ENSBTAG00000020886	*DIEXF*	3'152	2'096	2'575	2'730	2'420	-0.174	0.825804349	1
ENSBTAG00000020887	*BT.22875*	49	19	32	42	22	-0.952	0.666879029	1
ENSBTAG00000020889	*CA7*	51	136	101	44	157	1.830	0.168607069	1
ENSBTAG00000020890	*TRIM66*	62	109	90	54	126	1.229	0.368451004	1
ENSBTAG00000020892	*IL2RA*	21	14	17	18	16	-0.170	0.978224233	1
ENSBTAG00000020893	*MATN3*	86	78	82	74	90	0.274	0.851115455	1
ENSBTAG00000020894	*LAPTM4A*	28'306	24'059	26'147	24'514	27'781	0.181	0.811693353	1
ENSBTAG00000020896	*GRIFIN*	1	0	0	1	0		1	1
ENSBTAG00000020898	*BT.32313*	1'013	1'033	1'035	877	1'193	0.443	0.589918957	1
ENSBTAG00000020900	*RPUSD4*	721	719	727	624	830	0.411	0.628160958	1
ENSBTAG00000020904	*BT.44422*	336	215	270	291	248	-0.229	0.820031365	1
ENSBTAG00000020905	*RPL11*	62'544	57'699	60'395	54'165	66'625	0.299	0.693348176	1
ENSBTAG00000020907	*PSMC3IP*	572	506	540	495	584	0.238	0.786876605	1
ENSBTAG00000020908	*NAP1L1*	1'764	1'660	1'722	1'528	1'917	0.327	0.681053494	1
ENSBTAG00000020911	*FNBP4*	5'747	2'798	4'104	4'977	3'231	-0.623	0.422588616	1
ENSBTAG00000020914	*CPNE8*	1'507	631	1'017	1'305	729	-0.841	0.30991868	1
ENSBTAG00000020919	*BT.106438*	2	0	1	2	0		0.974934741	1
ENSBTAG00000020921	*PRKCB*	180	41	102	156	47	-1.719	0.195086551	1
ENSBTAG00000020922	*UPF1*	4'514	8'030	6'591	3'909	9'272	1.246	0.109320877	1
ENSBTAG00000020923	*BT.45969*	375	414	401	325	478	0.558	0.54330627	1
ENSBTAG00000020924	*BT.43176*	497	360	423	430	416	-0.050	0.959513044	1
ENSBTAG00000020927	*BT.64601*	2'229	1'378	1'761	1'930	1'591	-0.279	0.727274322	1
ENSBTAG00000020928	*ADPRHL1*	27	93	65	23	107	2.199	0.165737647	1

Ensembl gene ID	geneName	counts wildtype horn bud	counts polled horn bud	baseMean	baseMean wildtype horn bud	baseMean polled horn bud	log2FoldChange	pval	padj
ENSBTAG00000020929	*BT.34113*	28	0	12	24	0		0.230947873	1
ENSBTAG00000020930	*GEMIN2*	707	351	509	612	405	-0.595	0.502988976	1
ENSBTAG00000020931	*CHN2*	371	62	196	321	72	-2.166	0.049504949	1
ENSBTAG00000020933	*HCFC2*	1'481	976	1'205	1'283	1'127	-0.187	0.819979315	1
ENSBTAG00000020934	*SHF*	1'093	932	1'011	947	1'076	0.185	0.822323128	1
ENSBTAG00000020935	*HIF1A*	5'853	3'862	4'764	5'069	4'459	-0.185	0.811658361	1
ENSBTAG00000020936	*NOD2*	572	562	572	495	649	0.390	0.655154413	1
ENSBTAG00000020937	*SNAPC1*	574	504	540	497	582	0.227	0.796361122	1
ENSBTAG00000020938	*TWF1*	1'233	699	937	1'068	807	-0.404	0.627407343	1
ENSBTAG00000020939	*PLAC9*	6'021	9'965	8'360	5'214	11'507	1.142	0.140250275	1
ENSBTAG00000020940	*ANXA11*	8'198	6'595	7'357	7'100	7'615	0.101	0.894547306	1
ENSBTAG00000020942	*RNF166*	900	765	831	779	883	0.181	0.829638396	1
ENSBTAG00000020943	*BT.88215*	981	1'173	1'102	850	1'354	0.673	0.411969639	1
ENSBTAG00000020944	*PIEZO1*	3'989	4'832	4'517	3'455	5'580	0.692	0.372252283	1
ENSBTAG00000020947	*RNF103*	2'149	947	1'477	1'861	1'094	-0.767	0.342047032	1
ENSBTAG00000020953	*pseudogene*	1'391	1'209	1'300	1'205	1'396	0.213	0.792630317	1
ENSBTAG00000020954	*TRIM7*	171	56	106	148	65	-1.195	0.354561734	1
ENSBTAG00000020956	*MPPED2*	944	725	827	818	837	0.034	0.968279944	1
ENSBTAG00000020957	*FZD5*	48	8	25	42	9	-2.170	0.395000906	1
ENSBTAG00000020958	*LRRC8D*	1'066	744	891	923	859	-0.104	0.902703097	1
ENSBTAG00000020959	*BT.64623*	3'400	2'583	2'964	2'944	2'983	0.019	0.980839492	1
ENSBTAG00000020961	*DUSP19*	857	483	650	742	558	-0.412	0.632662756	1
ENSBTAG00000020962	*TUSC2*	296	227	259	256	262	0.032	0.977842677	1
ENSBTAG00000020963	*BT.56096*	2'515	2'388	2'468	2'178	2'757	0.340	0.664531494	1
ENSBTAG00000020964	*FAM40A*	2'466	3'474	3'074	2'136	4'011	0.909	0.246458759	1
ENSBTAG00000020965	*BT.51460*	44	12	26	38	14	-1.459	0.556675321	1
ENSBTAG00000020966	*BT.61185*	186	446	338	161	515	1.677	0.081737977	1
ENSBTAG00000020968	*BRP44*	1'338	1'045	1'183	1'159	1'207	0.058	0.943131456	1
ENSBTAG00000020969	*BT.92050*	3'046	1'545	2'211	2'638	1'784	-0.564	0.475685065	1
ENSBTAG00000020973	*RFT1*	308	325	321	267	375	0.493	0.605895501	1
ENSBTAG00000020975	*SYNGAP1*	1'443	1'653	1'579	1'250	1'909	0.611	0.445986556	1
ENSBTAG00000020979	*NGFR*	3'808	4'647	4'332	3'298	5'366	0.702	0.365383458	1
ENSBTAG00000020980	*CAT*	6'973	5'994	6'480	6'039	6'921	0.197	0.797315927	1
ENSBTAG00000020981	*BT.106043*	707	603	654	612	696	0.185	0.82945397	1
ENSBTAG00000020983	*RRAS*	800	879	854	693	1'015	0.551	0.50999932	1
ENSBTAG00000020984	*RAPGEF4*	774	172	434	670	199	-1.755	0.058594498	1
ENSBTAG00000020987	*PTCHD1*	16	26	22	14	30	1.115	0.681992165	1
ENSBTAG00000020988	*PLK1S1*	704	470	576	610	543	-0.168	0.849457473	1
ENSBTAG00000020989	*BT.31679*	3'092	1'540	2'228	2'678	1'778	-0.591	0.455305073	1
ENSBTAG00000020990	*P2RY14*	25	19	22	22	22	0.019	1	1
ENSBTAG00000020991	*COL19A1*	34	18	25	29	21	-0.503	0.852460785	1
ENSBTAG00000020992	*RHBDL3*	47	180	124	41	208	2.352	0.062072395	1
ENSBTAG00000020994	*C17ORF75*	1'127	667	873	976	770	-0.342	0.683314984	1
ENSBTAG00000020995	*pseudogene*	1	0	0	1	0		1	1
ENSBTAG00000020996	*BT.25222*	7'392	3'971	5'493	6'402	4'585	-0.481	0.533096206	1
ENSBTAG00000020997	*GPR15*	3	0	1	3	0		0.936647693	1
ENSBTAG00000020999	*DDB2*	1'555	1'527	1'555	1'347	1'763	0.389	0.627341131	1
ENSBTAG00000021000	*KIAA2018*	38	22	29	33	25	-0.373	0.883598383	1
ENSBTAG00000021002	*ACP2*	509	790	677	441	912	1.049	0.222981532	1
ENSBTAG00000021003	*BT.106515*	6	13	10	5	15	1.531	0.740992914	1
ENSBTAG00000021007	*BT.62067*	6	0	3	5	0		0.81115604	1
ENSBTAG00000021008	*ZNF219*	735	1'012	903	637	1'169	0.876	0.294049408	1
ENSBTAG00000021009	*TRIM55*	4	53	32	3	61	4.143	0.088377225	1
ENSBTAG00000021011	*pseudogene*	426	661	566	369	763	1.049	0.232669188	1
ENSBTAG00000021015	*FAM196A*	61	52	56	53	60	0.185	0.919842786	1
ENSBTAG00000021016	*GTF2F1*	1'830	2'243	2'087	1'585	2'590	0.709	0.371260097	1
ENSBTAG00000021018	*KHSRP*	3'459	5'949	4'932	2'996	6'869	1.197	0.125154319	1
ENSBTAG00000021019	*SLC30A4*	277	196	233	240	226	-0.084	0.939470692	1
ENSBTAG00000021020	*RIF1*	1'051	560	778	910	647	-0.493	0.559765351	1
ENSBTAG00000021021	*C15ORF39*	685	1'051	903	593	1'214	1.033	0.217509963	1
ENSBTAG00000021023	*ZMYM4*	1'640	1'340	1'484	1'420	1'547	0.124	0.8777276	1
ENSBTAG00000021024	*MACF1*	12'050	9'388	10'638	10'436	10'840	0.055	0.942156708	1
ENSBTAG00000021025	*LEPREL1*	2'606	2'001	2'284	2'257	2'311	0.034	0.965546812	1
ENSBTAG00000021026	*pseudogene*	1'350	1'034	1'182	1'169	1'194	0.030	0.970751308	1
ENSBTAG00000021029	*BT.19824*	786	378	559	681	436	-0.641	0.465206185	1
ENSBTAG00000021033	*BT.32803*	9	3	6	8	3	-1.170	0.890813994	1
ENSBTAG00000021035	*CTSK*	43'573	37'889	40'743	37'735	43'750	0.213	0.778082891	1
ENSBTAG00000021036	*Mrz 02*	1'797	1'538	1'666	1'556	1'776	0.191	0.811218977	1
ENSBTAG00000021037	*BT.87256*	2'456	2'185	2'325	2'127	2'523	0.246	0.753916951	1
ENSBTAG00000021039	*HNRNPM*	16'921	9'962	13'079	14'654	11'503	-0.349	0.647914675	1
ENSBTAG00000021041	*PRAM1*	39	15	26	34	17	-0.963	0.703159579	1
ENSBTAG00000021045	*E2F3*	171	217	199	148	251	0.759	0.474463177	1

Ensembl gene ID	geneName	counts wildtype horn bud	counts polled horn bud	baseMean	baseMean wildtype horn bud	baseMean polled horn bud	log2FoldChange	pval	padj
ENSBTAG00000021048	*bta-mir-6518*	489	91	264	423	105	-2.011	0.049452275	1
ENSBTAG00000021050	*ECEL1*	22	41	33	19	47	1.313	0.539653082	1
ENSBTAG00000021051	*ZNHIT6*	667	398	519	578	460	-0.330	0.710486188	1
ENSBTAG00000021052	*GNB1L*	830	888	872	719	1'025	0.512	0.539080653	1
ENSBTAG00000021057	*TMEM190*	125	258	203	108	298	1.460	0.171172585	1
ENSBTAG00000021058	*DDX42*	4'834	3'861	4'322	4'186	4'458	0.091	0.906131133	1
ENSBTAG00000021059	*BT.103569*	1'274	560	875	1'103	647	-0.771	0.357538537	1
ENSBTAG00000021060	*FTSJ3*	974	785	875	844	906	0.104	0.901441375	1
ENSBTAG00000021061	*PSMC5*	3'303	3'551	3'480	2'860	4'100	0.519	0.504292143	1
ENSBTAG00000021062	*SMARCD2*	3'137	5'041	4'269	2'717	5'821	1.099	0.159414313	1
ENSBTAG00000021064	*protein_coding*	116	248	193	100	286	1.511	0.162389678	1
ENSBTAG00000021065	*TMEM180*	786	748	772	681	864	0.344	0.683839134	1
ENSBTAG00000021066	*CAPN11*	44	11	25	38	13	-1.585	0.529046878	1
ENSBTAG00000021067	*BT.48228*	7'356	10'037	8'980	6'370	11'590	0.863	0.261987311	1
ENSBTAG00000021068	*BT.91956*	450	875	700	390	1'010	1.374	0.111565528	1
ENSBTAG00000021069	*PBK*	980	744	854	849	859	0.018	0.984120657	1
ENSBTAG00000021071	*TRIM8*	1'238	1'141	1'195	1'072	1'318	0.297	0.714779163	1
ENSBTAG00000021072	*DTNB*	697	545	616	604	629	0.060	0.945728718	1
ENSBTAG00000021073	*KIAA1549*	374	491	445	324	567	0.808	0.37189437	1
ENSBTAG00000021076	*TNRC6B*	426	305	361	369	352	-0.067	0.94664499	1
ENSBTAG00000021078	*PYROXD1*	1'062	637	828	920	736	-0.322	0.701820745	1
ENSBTAG00000021079	*C3H1ORF210*	87	65	75	75	75	-0.006	1	1
ENSBTAG00000021082	*TMEM125*	174	178	178	151	206	0.448	0.682775515	1
ENSBTAG00000021083	*RECQL*	1'169	579	840	1'012	669	-0.599	0.475951588	1
ENSBTAG00000021087	*UBR1*	986	589	767	854	680	-0.328	0.698741285	1
ENSBTAG00000021090	*BT.88646*	943	1'754	1'421	817	2'025	1.310	0.108353408	1
ENSBTAG00000021091	*ANKFY1*	1'692	1'952	1'860	1'465	2'254	0.621	0.434861385	1
ENSBTAG00000021093	*RPS16*	7'740	7'141	7'474	6'703	8'246	0.299	0.696346396	1
ENSBTAG00000021096	*protein_coding*	232	346	300	201	400	0.992	0.306686794	1
ENSBTAG00000021097	*PELI3*	33	15	23	29	17	-0.722	0.793259482	1
ENSBTAG00000021099	*CLVS2*	79	5	37	68	6	-3.567	0.108598236	1
ENSBTAG00000021100	*PPP1R3F*	265	227	246	229	262	0.192	0.851465578	1
ENSBTAG00000021101	*GPR88*	8	5	6	7	6	-0.263	0.996869191	1
ENSBTAG00000021102	*GALM*	1'531	1'076	1'284	1'326	1'242	-0.094	0.909443742	1
ENSBTAG00000021103	*SLC35F5*	5'247	2'731	3'849	4'544	3'153	-0.527	0.498093092	1
ENSBTAG00000021105	*IRAK4*	3'521	1'587	2'441	3'049	1'833	-0.735	0.352038548	1
ENSBTAG00000021106	*CASP14*	19	11	15	16	13	-0.373	0.934208892	1
ENSBTAG00000021107	*STXBP5*	639	288	443	553	333	-0.735	0.417892778	1
ENSBTAG00000021109	*pseudogene*	3	0	1	3	0		0.936647693	1
ENSBTAG00000021111	*BT.92603*	1	0	0	1	0		1	1
ENSBTAG00000021113	*ALDH3A1*	5	2	3	4	2	-0.907	0.965043111	1
ENSBTAG00000021115	*ZNF692*	1'597	1'116	1'336	1'383	1'289	-0.102	0.901094751	1
ENSBTAG00000021116	*BT.47793*	986	438	680	854	506	-0.756	0.378787176	1
ENSBTAG00000021118	*CYP26A1*	6	0	3	5	0		0.81115604	1
ENSBTAG00000021119	*BT.22336*	14	3	8	12	3	-1.807	0.758305361	1
ENSBTAG00000021121	*PPIL2*	2'278	2'614	2'496	1'973	3'018	0.614	0.435286104	1
ENSBTAG00000021125	*NUP160*	1'847	1'836	1'860	1'600	2'120	0.406	0.608732893	1
ENSBTAG00000021126	*TAF5L*	0	1	1	0	1	Inf	0.993540919	1
ENSBTAG00000021128	*ADD1*	6'516	10'094	8'649	5'643	11'656	1.046	0.17552625	1
ENSBTAG00000021130	*MFSD10*	2'905	1'804	2'299	2'516	2'083	-0.272	0.730401678	1
ENSBTAG00000021131	*HNRNPK*	15'971	17'492	17'015	13'831	20'198	0.546	0.473823883	1
ENSBTAG00000021132	*SYNPO2L*	8	86	53	7	99	3.841	0.040454797	1
ENSBTAG00000021133	*BT.91898*	3'394	1'050	2'076	2'939	1'212	-1.278	0.110860064	1
ENSBTAG00000021134	*DPP9*	841	1'021	954	728	1'179	0.695	0.402204834	1
ENSBTAG00000021135	*pseudogene*	606	316	445	525	365	-0.524	0.56281487	1
ENSBTAG00000021139	*BT.53910*	4	1	2	3	1	-1.585	0.952247636	1
ENSBTAG00000021140	*RMND5A*	464	565	527	402	652	0.699	0.428228472	1
ENSBTAG00000021141	*CD8A*	24	2	12	21	2	-3.170	0.47791533	1
ENSBTAG00000021143	*DNMT3A*	2'293	2'345	2'347	1'986	2'708	0.447	0.56959065	1
ENSBTAG00000021144	*PSTPIP1*	280	138	201	242	159	-0.606	0.56897256	1
ENSBTAG00000021145	*MAP4K1*	98	65	80	85	75	-0.177	0.909956318	1
ENSBTAG00000021151	*MYH10*	10'898	16'291	14'125	9'438	18'811	0.995	0.195512604	1
ENSBTAG00000021156	*WDR67*	2'320	1'314	1'763	2'009	1'517	-0.405	0.611932457	1
ENSBTAG00000021157	*BT.21710*	3'981	4'815	4'504	3'448	5'560	0.689	0.373780032	1
ENSBTAG00000021158	*SATB1*	3'053	2'265	2'630	2'644	2'615	-0.016	0.985277002	1
ENSBTAG00000021160	*AP1M2*	384	649	541	333	749	1.172	0.185770419	1
ENSBTAG00000021161	*CREG2*	48	14	29	42	16	-1.363	0.559836341	1
ENSBTAG00000021162	*BT.65454*	3'237	1'830	2'458	2'803	2'113	-0.408	0.604665603	1
ENSBTAG00000021164	*SLMAP*	2'807	1'321	1'978	2'431	1'525	-0.672	0.397892741	1
ENSBTAG00000021165	*C16ORF7*	897	809	855	777	934	0.266	0.750212645	1
ENSBTAG00000021166	*FAM71A*	0	3	2	0	3	Inf	0.878646179	1
ENSBTAG00000021168	*SIRT4*	244	166	201	211	192	-0.141	0.899213739	1

Ensembl gene ID	geneName	counts wildtype horn bud	counts polled horn bud	baseMean	baseMean wildtype horn bud	baseMean polled horn bud	log2FoldChange	pval	padj
ENSBTAG00000021170	*BT.31540*	5'004	2'904	3'843	4'334	3'353	-0.370	0.634205616	1
ENSBTAG00000021172	*IWS1*	1'912	1'349	1'607	1'656	1'558	-0.088	0.913645584	1
ENSBTAG00000021173	*CCDC134*	200	231	220	173	267	0.623	0.547326213	1
ENSBTAG00000021174	*BT.44915*	6'078	3'948	4'911	5'264	4'559	-0.207	0.788868006	1
ENSBTAG00000021176	*CRISPLD2*	6'138	2'406	4'047	5'316	2'778	-0.936	0.230400332	1
ENSBTAG00000021180	*SPATIAL*	2	0	1	2	0		0.974934741	1
ENSBTAG00000021181	*BUB1*	1'921	1'526	1'713	1'664	1'762	0.083	0.917135383	1
ENSBTAG00000021182	*DOCK4*	538	224	362	466	259	-0.849	0.365282196	1
ENSBTAG00000021183	*protein_coding*	330	219	269	286	253	-0.176	0.862056859	1
ENSBTAG00000021187	*ID2*	3'712	3'746	3'770	3'215	4'326	0.428	0.580965719	1
ENSBTAG00000021188	*MRPL16*	1'417	1'246	1'333	1'227	1'439	0.230	0.776336881	1
ENSBTAG00000021192	*GLTSCR2*	3'943	8'461	6'592	3'415	9'770	1.517	0.052840335	1
ENSBTAG00000021193	*FBXO5*	322	244	280	279	282	0.015	0.991361633	1
ENSBTAG00000021196	*C5H12orf45*	392	336	364	339	388	0.193	0.837746611	1
ENSBTAG00000021199	*APOPT1*	654	376	500	566	434	-0.384	0.667253565	1
ENSBTAG00000021200	*KISS1*	0	1	1	0	1	Inf	0.993540919	1
ENSBTAG00000021201	*FAM126B*	146	95	118	126	110	-0.205	0.874925784	1
ENSBTAG00000021202	*ACOT7*	857	683	765	742	789	0.088	0.918084948	1
ENSBTAG00000021205	*PPAPDC3*	628	619	629	544	715	0.394	0.647554492	1
ENSBTAG00000021208	*RRM2B*	138	89	111	120	103	-0.218	0.869701592	1
ENSBTAG00000021209	*UBR5*	9'506	4'488	6'707	8'232	5'182	-0.668	0.386582659	1
ENSBTAG00000021210	*HES2*	524	302	401	454	349	-0.380	0.68055052	1
ENSBTAG00000021211	*DPT*	31'510	56'552	46'295	27'288	65'301	1.259	0.101868227	1
ENSBTAG00000021214	*BT.17790*	3'824	3'487	3'669	3'312	4'026	0.282	0.716085841	1
ENSBTAG00000021215	*VWA5A*	2'063	2'543	2'362	1'787	2'936	0.717	0.363563581	1
ENSBTAG00000021216	*HECW1*	176	27	92	152	31	-2.290	0.102704075	1
ENSBTAG00000021219	*Sep 01*	107	129	121	93	149	0.685	0.577205739	1
ENSBTAG00000021220	*TMEM72*	4	1	2	3	1	-1.585	0.952247636	1
ENSBTAG00000021222	*BT.100459*	957	870	917	829	1'005	0.278	0.738252358	1
ENSBTAG00000021223	*CRY2*	764	1'066	946	662	1'231	0.896	0.281875218	1
ENSBTAG00000021224	*ZSWIM6*	1'007	458	700	872	529	-0.722	0.398980183	1
ENSBTAG00000021225	*DCAF5*	2'106	1'992	2'062	1'824	2'300	0.335	0.671776621	1
ENSBTAG00000021226	*SAR1B*	631	438	526	546	506	-0.112	0.901881081	1
ENSBTAG00000021227	*ATP5H*	7'072	5'856	6'443	6'125	6'762	0.143	0.851980697	1
ENSBTAG00000021230	*SFTPB*	0	1	1	0	1	Inf	0.993540919	1
ENSBTAG00000021231	*KCTD2*	784	1'379	1'136	679	1'592	1.230	0.136600959	1
ENSBTAG00000021232	*TPD52L2*	5'890	4'513	5'156	5'101	5'211	0.031	0.967656205	1
ENSBTAG00000021235	*protein_coding*	0	1	1	0	1	Inf	0.993540919	1
ENSBTAG00000021237	*DST*	9'601	8'938	9'318	8'315	10'321	0.312	0.683183017	1
ENSBTAG00000021240	*TM7SF4*	13	1	6	11	1	-3.285	0.663144098	1
ENSBTAG00000021242	*COQ7*	494	397	443	428	458	0.100	0.913734979	1
ENSBTAG00000021245	*SPRY1*	4'266	3'048	3'607	3'694	3'520	-0.070	0.929213474	1
ENSBTAG00000021246	*BT.88689*	1'657	1'023	1'308	1'435	1'181	-0.281	0.729816727	1
ENSBTAG00000021248	*C25H7ORF26*	1'421	1'535	1'502	1'231	1'772	0.526	0.512277249	1
ENSBTAG00000021249	*BT.26847*	144	87	113	125	100	-0.312	0.809438252	1
ENSBTAG00000021250	*RALBP1*	2'432	2'468	2'478	2'106	2'850	0.436	0.578480964	1
ENSBTAG00000021251	*RIPK4*	1'130	1'952	1'616	979	2'254	1.204	0.136598523	1
ENSBTAG00000021252	*TMEM35*	921	233	533	798	269	-1.568	0.080453201	1
ENSBTAG00000021253	*PRDM15*	246	282	269	213	326	0.612	0.535878825	1
ENSBTAG00000021254	*CENPI*	1'093	745	903	947	860	-0.138	0.870026697	1
ENSBTAG00000021257	*OTOP2*	20	5	12	17	6	-1.585	0.710402816	1
ENSBTAG00000021260	*GALNT11*	2'753	1'837	2'253	2'384	2'121	-0.169	0.831603787	1
ENSBTAG00000021262	*BT.28460*	3'038	2'244	2'611	2'631	2'591	-0.022	0.97882923	1
ENSBTAG00000021263	*BT.52602*	2'389	1'318	1'795	2'069	1'522	-0.443	0.578710481	1
ENSBTAG00000021270	*UMODL1*	95	4	43	82	5	-4.155	0.05141429	1
ENSBTAG00000021272	*BT.29337*	219	97	151	190	112	-0.760	0.508947514	1
ENSBTAG00000021273	*SEMA4B*	1'137	1'258	1'219	985	1'453	0.561	0.490556992	1
ENSBTAG00000021275	*CIB1*	2'212	1'807	2'001	1'916	2'087	0.123	0.876068275	1
ENSBTAG00000021276	*BT.56560*	1	0	0	1	0		1	1
ENSBTAG00000021282	*BT.30480*	5'270	5'704	5'575	4'564	6'586	0.529	0.492318344	1
ENSBTAG00000021283	*KCNF1*	46	21	32	40	24	-0.716	0.749794005	1
ENSBTAG00000021284	*BT.37492*	49	1	22	42	1	-5.200	0.124985357	1
ENSBTAG00000021286	*SLC37A1*	176	68	115	152	79	-0.957	0.444932833	1
ENSBTAG00000021287	*SLC16A7*	354	421	396	307	486	0.665	0.469696531	1
ENSBTAG00000021288	*PSMB4*	6'593	4'722	5'581	5'710	5'452	-0.066	0.932062997	1
ENSBTAG00000021289	*BT.28081*	6'713	4'035	5'236	5'814	4'659	-0.319	0.679469549	1
ENSBTAG00000021291	*BT.88441*	355	708	562	307	818	1.411	0.111351265	1
ENSBTAG00000021292	*ANKFN1*	20	1	9	17	1	-3.907	0.484363729	1
ENSBTAG00000021293	*SNUPN*	1'381	852	1'090	1'196	984	-0.282	0.732164922	1
ENSBTAG00000021294	*VWA1*	2'954	2'450	2'694	2'558	2'829	0.145	0.852605256	1
ENSBTAG00000021301	*ACSF2*	5'148	4'235	4'674	4'458	4'890	0.133	0.862405178	1
ENSBTAG00000021303	*BT.29418*	1'095	823	949	948	950	0.003	0.997833649	1

Ensembl gene ID	geneName	counts wildtype horn bud	counts polled horn bud	baseMean	baseMean wildtype horn bud	baseMean polled horn bud	log2FoldChange	pval	padj
ENSBTAG00000021304	*TP53BP1*	5'913	4'552	5'189	5'121	5'256	0.038	0.960622108	1
ENSBTAG00000021306	*CHRDL2*	65	51	58	56	59	0.065	0.979198771	1
ENSBTAG00000021307	*BNIP3L*	2'968	3'447	3'275	2'570	3'980	0.631	0.418717456	1
ENSBTAG00000021308	*IRS1*	1'421	1'329	1'383	1'231	1'535	0.318	0.692757191	1
ENSBTAG00000021310	*COL4A4*	44	4	21	38	5	-3.044	0.299531593	1
ENSBTAG00000021316	*COL4A3*	33	4	17	29	5	-2.629	0.435665511	1
ENSBTAG00000021318	*SCAMP5*	550	175	339	476	202	-1.237	0.194901876	1
ENSBTAG00000021319	*C2H2ORF33*	3'771	2'216	2'912	3'266	2'559	-0.352	0.653240312	1
ENSBTAG00000021321	*SDCBP2*	66	25	43	57	29	-0.986	0.602910743	1
ENSBTAG00000021322	*BT.21850*	1	0	0	1	0		1	1
ENSBTAG00000021323	*AGFG1*	2'085	1'340	1'676	1'806	1'547	-0.223	0.781261106	1
ENSBTAG00000021325	*SLC19A3*	251	211	231	217	244	0.165	0.874917497	1
ENSBTAG00000021326	*CCL20*	7	6	6	6	7	0.193	1	1
ENSBTAG00000021327	*BT.25265*	40	1	18	35	1	-4.907	0.190349621	1
ENSBTAG00000021328	*SPHKAP*	50	7	26	43	8	-2.421	0.343745578	1
ENSBTAG00000021329	*ZBTB3*	63	74	70	55	85	0.647	0.668468268	1
ENSBTAG00000021333	*BT.17940*	14	10	12	12	12	-0.070	1	1
ENSBTAG00000021334	*C24H18orf32*	1'849	974	1'363	1'601	1'125	-0.510	0.529031388	1
ENSBTAG00000021337	*SBF1*	4'023	6'112	5'271	3'484	7'058	1.018	0.190020708	1
ENSBTAG00000021338	*OAF*	3'070	3'515	3'359	2'659	4'059	0.610	0.433628344	1
ENSBTAG00000021339	*SCN8A*	102	16	53	88	18	-2.257	0.196891509	1
ENSBTAG00000021340	*BT.88070*	228	235	234	197	271	0.459	0.653285628	1
ENSBTAG00000021341	*BT.103144*	1'747	1'013	1'341	1'513	1'170	-0.371	0.647032773	1
ENSBTAG00000021342	*TMEM136*	878	409	616	760	472	-0.687	0.42839838	1
ENSBTAG00000021343	*BT.52588*	837	928	898	725	1'072	0.564	0.498182455	1
ENSBTAG00000021344	*USP26*	0	1	1	0	1	Inf	0.993540919	1
ENSBTAG00000021345	*OXGR1*	1	0	0	1	0		1	1
ENSBTAG00000021346	*CCDC34*	440	197	304	381	227	-0.744	0.441926119	1
ENSBTAG00000021347	*CWH43*	2	0	1	2	0		0.974934741	1
ENSBTAG00000021351	*MED12*	6'325	7'592	7'122	5'478	8'766	0.678	0.377968651	1
ENSBTAG00000021357	*ZNF518A*	1'215	544	840	1'052	628	-0.744	0.375966208	1
ENSBTAG00000021358	*BT.24520*	404	173	275	350	200	-0.809	0.413360223	1
ENSBTAG00000021359	*BT.106074*	423	149	269	366	172	-1.090	0.273598058	1
ENSBTAG00000021360	*RASGEF1C*	58	4	27	50	5	-3.443	0.184475246	1
ENSBTAG00000021361	*BT.47533*	2	1	1	2	1	-0.585	1	1
ENSBTAG00000021364	*BT.64988*	2'516	2'442	2'499	2'179	2'820	0.372	0.635396824	1
ENSBTAG00000021365	*CDHR3*	11	19	16	10	22	1.204	0.721377641	1
ENSBTAG00000021367	*ANP32B*	3'948	2'896	3'382	3'419	3'344	-0.032	0.96825661	1
ENSBTAG00000021368	*AKR1C3*	0	1	1	0	1	Inf	0.993540919	1
ENSBTAG00000021370	*BLMH*	1'928	1'552	1'731	1'670	1'792	0.102	0.89802607	1
ENSBTAG00000021372	*Sep 11*	1'711	2'948	2'443	1'482	3'404	1.200	0.131059068	1
ENSBTAG00000021373	*GTF3C5*	1'275	1'588	1'469	1'104	1'834	0.732	0.363976479	1
ENSBTAG00000021374	*FARSA*	1'109	1'338	1'253	960	1'545	0.686	0.399031782	1
ENSBTAG00000021375	*OR10Q1*	1	0	0	1	0		1	1
ENSBTAG00000021377	*S100A14*	8'364	7'159	7'755	7'243	8'267	0.191	0.803069388	1
ENSBTAG00000021378	*S100A13*	1'349	881	1'093	1'168	1'017	-0.200	0.808859695	1
ENSBTAG00000021381	*DAAM2*	1'785	3'248	2'648	1'546	3'750	1.279	0.107286264	1
ENSBTAG00000021386	*ANGPTL3*	15	2	8	13	2	-2.492	0.678711229	1
ENSBTAG00000021390	*BT.54311*	16	8	12	14	9	-0.585	0.904936474	1
ENSBTAG00000021392	*BT.85442*	2'093	1'941	2'027	1'813	2'241	0.306	0.698428627	1
ENSBTAG00000021393	*FLRT2*	1'445	986	1'195	1'251	1'139	-0.136	0.868464784	1
ENSBTAG00000021394	*FITM1*	190	142	164	165	164	-0.005	1	1
ENSBTAG00000021395	*PSME1*	5'364	5'179	5'313	4'645	5'980	0.364	0.636037569	1
ENSBTAG00000021396	*HEG1*	1'179	1'724	1'506	1'021	1'991	0.963	0.233106201	1
ENSBTAG00000021397	*NKX2-3*	0	1	1	0	1	Inf	0.993540919	1
ENSBTAG00000021398	*C9H6ORF203*	584	423	497	506	488	-0.050	0.958022017	1
ENSBTAG00000021407	*CASP16*	4	11	8	3	13	1.874	0.733957485	1
ENSBTAG00000021408	*FMO1*	53	14	31	46	16	-1.506	0.502769511	1
ENSBTAG00000021410	*C1GALT1C1*	1'666	886	1'233	1'443	1'023	-0.496	0.542738495	1
ENSBTAG00000021413	*ZIC3*	109	113	112	94	130	0.467	0.712143096	1
ENSBTAG00000021414	*BT.50635*	3	5	4	3	6	1.152	0.920866718	1
ENSBTAG00000021415	*MGC148542*	5	1	3	4	1	-1.907	0.9188325	1
ENSBTAG00000021416	*FAM213A*	2'606	1'918	2'236	2'257	2'215	-0.027	0.973787123	1
ENSBTAG00000021417	*DMWD*	1'453	1'878	1'713	1'258	2'169	0.785	0.326514861	1
ENSBTAG00000021421	*SSR4*	15'481	14'050	14'815	13'407	16'224	0.275	0.717678203	1
ENSBTAG00000021422	*PDZD4*	588	807	721	509	932	0.872	0.306691733	1
ENSBTAG00000021424	*CSK*	2'941	3'138	3'085	2'547	3'623	0.509	0.51457862	1
ENSBTAG00000021425	*LMAN1L*	1	0	0	1	0		1	1
ENSBTAG00000021427	*HOXB3*	12	3	7	10	3	-1.585	0.809704871	1
ENSBTAG00000021428	*BT.64331*	1	3	2	1	3	2.000	0.933774563	1
ENSBTAG00000021430	*UPK3B*	291	165	221	252	191	-0.404	0.698761315	1
ENSBTAG00000021432	*TEDDM1*	8	1	4	7	1	-2.585	0.818599781	1

Ensembl gene ID	geneName	counts wildtype horn bud	counts polled horn bud	baseMean	baseMean wildtype horn bud	baseMean polled horn bud	log2FoldChange	pval	padj
ENSBTAG00000021433	*ZNF836*	672	348	492	582	402	-0.534	0.549539732	1
ENSBTAG00000021434	*EDN2*	204	292	257	177	337	0.932	0.351557354	1
ENSBTAG00000021435	*MAFF*	371	263	312	321	304	-0.081	0.936553443	1
ENSBTAG00000021437	*GPR174*	10	0	4	9	0		0.653360153	1
ENSBTAG00000021438	*ZNF821*	1'239	933	1'075	1'073	1'077	0.006	0.994970288	1
ENSBTAG00000021442	*CRTAP*	2'373	3'140	2'840	2'055	3'626	0.819	0.296960936	1
ENSBTAG00000021443	*PGLYRP3*	54	34	43	47	39	-0.252	0.90478782	1
ENSBTAG00000021445	*BT.17789*	438	234	325	379	270	-0.489	0.608934161	1
ENSBTAG00000021447	*SIM2*	0	2	1	0	2	Inf	0.939077559	1
ENSBTAG00000021449	*DCAF13*	943	705	815	817	814	-0.005	0.998069142	1
ENSBTAG00000021452	*TRANK1*	4'379	3'997	4'204	3'792	4'615	0.283	0.71388273	1
ENSBTAG00000021454	*SNX32*	86	53	68	74	61	-0.283	0.860885841	1
ENSBTAG00000021455	*CFL1*	23'071	23'986	23'838	19'980	27'697	0.471	0.535750666	1
ENSBTAG00000021456	*BT.26546*	1'564	1'638	1'623	1'354	1'891	0.482	0.546891821	1
ENSBTAG00000021457	*EFEMP2*	10'001	9'673	9'915	8'661	11'169	0.367	0.63103723	1
ENSBTAG00000021461	*PSMD3*	4'879	6'579	5'911	4'225	7'597	0.846	0.273827195	1
ENSBTAG00000021462	*CSF3*	4	2	3	3	2	-0.585	0.996239345	1
ENSBTAG00000021464	*SPRYD3*	1'245	1'181	1'221	1'078	1'364	0.339	0.676577738	1
ENSBTAG00000021466	*BT.64714*	2'428'525	3'647'191	3'157'289	2'103'164	4'211'413	1.002	0.189793212	1
ENSBTAG00000021467	*IGFBP6*	7'275	3'375	5'099	6'300	3'897	-0.693	0.370961035	1
ENSBTAG00000021468	*MED24*	2'613	2'668	2'672	2'263	3'081	0.445	0.569857282	1
ENSBTAG00000021469	*CTTNBP2*	4'123	1'749	2'795	3'571	2'020	-0.822	0.296023453	1
ENSBTAG00000021471	*GIN1*	289	213	248	250	246	-0.025	0.985406958	1
ENSBTAG00000021472	*ZC3H3*	648	951	830	561	1'098	0.968	0.250428809	1
ENSBTAG00000021474	*GSDMD*	717	651	686	621	752	0.276	0.746960903	1
ENSBTAG00000021480	*BT.32317*	7	4	5	6	5	-0.392	0.98714753	1
ENSBTAG00000021481	*CA14*	24	21	23	21	24	0.222	0.948915741	1
ENSBTAG00000021482	*APH1A*	4'238	4'961	4'699	3'670	5'728	0.642	0.40667293	1
ENSBTAG00000021483	*BT.25836*	4'423	4'949	4'773	3'830	5'715	0.577	0.455408384	1
ENSBTAG00000021484	*BT.24405*	504	1'080	842	436	1'247	1.515	0.075299356	1
ENSBTAG00000021485	*C3H1orf54*	630	270	429	546	312	-0.807	0.376018177	1
ENSBTAG00000021487	*C1ORF51*	245	153	194	212	177	-0.264	0.808432074	1
ENSBTAG00000021490	*WDR4*	366	420	401	317	485	0.614	0.50387634	1
ENSBTAG00000021491	*BT.43863*	1'994	1'928	1'977	1'727	2'226	0.366	0.643402815	1
ENSBTAG00000021492	*FAM163A*	15	0	6	13	0		0.491308095	1
ENSBTAG00000021494	*EN1*	452	383	417	391	442	0.176	0.848130863	1
ENSBTAG00000021499	*BT.42764*	22'010	27'621	25'478	19'061	31'894	0.743	0.330613712	1
ENSBTAG00000021501	*BT.66156*	27	5	15	23	6	-2.018	0.576089911	1
ENSBTAG00000021504	*TIRAP*	120	49	80	104	57	-0.877	0.54030931	1
ENSBTAG00000021505	*USP36*	1'032	1'647	1'398	894	1'902	1.089	0.180262965	1
ENSBTAG00000021506	*ARL6IP5*	5'429	3'126	4'156	4'702	3'610	-0.381	0.623255458	1
ENSBTAG00000021508	*LMOD3*	20	113	74	17	130	2.913	0.060899964	1
ENSBTAG00000021512	*ZBTB7B*	920	1'374	1'192	797	1'587	0.994	0.225360831	1
ENSBTAG00000021514	*DNPEP*	2'069	2'831	2'530	1'792	3'269	0.867	0.271485682	1
ENSBTAG00000021515	*ZDHHC19*	12	13	13	10	15	0.531	0.905259543	1
ENSBTAG00000021516	*GSTA1*	45	17	29	39	20	-0.989	0.671023777	1
ENSBTAG00000021517	*TMEM216*	685	379	515	593	438	-0.439	0.621018124	1
ENSBTAG00000021518	*TMPRSS4*	1'236	942	1'079	1'070	1'088	0.023	0.978026158	1
ENSBTAG00000021519	*CAMLG*	1'107	662	862	959	764	-0.327	0.69690812	1
ENSBTAG00000021520	*BT.60112*	3'657	1'773	2'607	3'167	2'047	-0.629	0.423747471	1
ENSBTAG00000021521	*BFAR*	2'934	1'880	2'356	2'541	2'171	-0.227	0.773781104	1
ENSBTAG00000021522	*BT.22381*	4	3	3	3	3	0.000	1	1
ENSBTAG00000021523	*STAT3*	7'443	4'137	5'611	6'446	4'777	-0.432	0.575528951	1
ENSBTAG00000021525	*BT.87211*	58	70	66	50	81	0.686	0.658503257	1
ENSBTAG00000021527	*IGF1R*	702	1'194	993	608	1'379	1.181	0.156345736	1
ENSBTAG00000021529	*protein_coding*	1	0	0	1	0		1	1
ENSBTAG00000021531	*PGPEP1L*	1	0	0	1	0		1	1
ENSBTAG00000021535	*BT.65661*	1'386	787	1'055	1'200	909	-0.401	0.626143163	1
ENSBTAG00000021537	*RWDD3*	250	98	165	217	113	-0.936	0.40396332	1
ENSBTAG00000021538	*MYO1E*	1'591	806	1'154	1'378	931	-0.566	0.489282562	1
ENSBTAG00000021540	*BRWD2*	3'465	2'154	2'744	3'001	2'487	-0.271	0.730221571	1
ENSBTAG00000021543	*MDFIC*	4'035	1'586	2'663	3'494	1'831	-0.932	0.237518159	1
ENSBTAG00000021544	*EBNA1BP2*	1'730	1'301	1'500	1'498	1'502	0.004	0.996415712	1
ENSBTAG00000021547	*WDR65*	133	229	190	115	264	1.199	0.266793873	1
ENSBTAG00000021549	*PHPT1*	1'392	1'522	1'481	1'206	1'757	0.544	0.498757873	1
ENSBTAG00000021550	*BT.54874*	273	271	275	236	313	0.404	0.681764226	1
ENSBTAG00000021553	*PTPN14*	411	589	518	356	680	0.934	0.292323427	1
ENSBTAG00000021554	*NOL7*	1'504	783	1'103	1'303	904	-0.527	0.521267949	1
ENSBTAG00000021556	*RANBP9*	3'106	1'443	2'178	2'690	1'666	-0.691	0.383200096	1
ENSBTAG00000021557	*BT.21655*	12	6	9	10	7	-0.585	0.929512138	1
ENSBTAG00000021558	*LSM14B*	4'104	4'036	4'107	3'554	4'660	0.391	0.613495067	1
ENSBTAG00000021565	*PRSS2*	510	491	504	442	567	0.360	0.684700445	1

Ensembl gene ID	geneName	counts wildtype horn bud	counts polled horn bud	baseMean	baseMean wildtype horn bud	baseMean polled horn bud	log2FoldChange	pval	padj
ENSBTAG00000021568	*VWCE*	374	530	468	324	612	0.918	0.307403748	1
ENSBTAG00000021569	*DDB1*	13'329	18'299	16'337	11'543	21'130	0.872	0.255082173	1
ENSBTAG00000021570	*PLEKHG4*	1'444	342	823	1'251	395	-1.663	0.052442238	1
ENSBTAG00000021573	*DCAF12*	5'037	3'421	4'156	4'362	3'950	-0.143	0.854223574	1
ENSBTAG00000021574	*TCFL2*	952	726	831	824	838	0.024	0.977996506	1
ENSBTAG00000021575	*BRD7*	5'664	3'930	4'722	4'905	4'538	-0.112	0.885299214	1
ENSBTAG00000021576	*LMOD1*	417	1'045	784	361	1'207	1.740	0.043598395	1
ENSBTAG00000021577	*ZFYVE16*	3'566	1'875	2'627	3'088	2'165	-0.512	0.514515528	1
ENSBTAG00000021580	*BT.94621*	2'974	2'873	2'947	2'576	3'317	0.365	0.639792736	1
ENSBTAG00000021581	*FHOD3*	3'409	2'508	2'924	2'952	2'896	-0.028	0.972798247	1
ENSBTAG00000021582	*BT.100379*	2'190	1'230	1'658	1'897	1'420	-0.417	0.602422884	1
ENSBTAG00000021583	*C24H18ORF10*	1'674	1'413	1'541	1'450	1'632	0.170	0.831446801	1
ENSBTAG00000021586	*FAM122A*	929	576	735	805	665	-0.275	0.747578595	1
ENSBTAG00000021587	*BT.97078*	196	176	186	170	203	0.260	0.812134512	1
ENSBTAG00000021590	*OR52B2*	1	0	0	1	0		1	1
ENSBTAG00000021591	*OAZ3*	18	0	8	16	0		0.412867528	1
ENSBTAG00000021592	*WRN*	1'044	919	983	904	1'061	0.231	0.779673021	1
ENSBTAG00000021594	*TDRKH*	237	86	152	205	99	-1.047	0.361842269	1
ENSBTAG00000021595	*HIP1R*	1'180	2'363	1'875	1'022	2'729	1.417	0.079107727	1
ENSBTAG00000021596	*ICOS*	1	2	2	1	2	1.415	0.981979269	1
ENSBTAG00000021600	*C2ORF67*	1'225	667	916	1'061	770	-0.462	0.579310865	1
ENSBTAG00000021601	*BT.42541*	186	143	163	161	165	0.036	0.979873214	1
ENSBTAG00000021602	*CTTNBP2NL*	923	1'309	1'155	799	1'512	0.919	0.262518717	1
ENSBTAG00000021604	*TMEM138*	1'816	969	1'346	1'573	1'119	-0.491	0.544431454	1
ENSBTAG00000021606	*ERCC8*	556	283	404	482	327	-0.559	0.54318051	1
ENSBTAG00000021607	*BT.17287*	210	115	157	182	133	-0.454	0.691310297	1
ENSBTAG00000021609	*GPR110*	13	2	7	11	2	-2.285	0.731320841	1
ENSBTAG00000021611	*C15H11ORF74*	2'129	1'665	1'883	1'844	1'923	0.060	0.939330354	1
ENSBTAG00000021614	*TASP1*	641	353	481	555	408	-0.446	0.619017718	1
ENSBTAG00000021615	*LGALS3*	0	1	1	0	1	Inf	0.993540919	1
ENSBTAG00000021616	*BT.43153*	3	2	2	3	2	-0.170	1	1
ENSBTAG00000021617	*ZC3HAV1*	948	866	910	821	1'000	0.285	0.732058864	1
ENSBTAG00000021620	*MGC159500*	2'740	1'523	2'066	2'373	1'759	-0.432	0.585682098	1
ENSBTAG00000021630	*MTFMT*	599	327	448	519	378	-0.458	0.61285951	1
ENSBTAG00000021632	*MSRA*	748	571	654	648	659	0.025	0.977586988	1
ENSBTAG00000021633	*TDG*	2'417	1'719	2'039	2'093	1'985	-0.077	0.924119733	1
ENSBTAG00000021634	*PIAS3*	2'125	2'509	2'369	1'840	2'897	0.655	0.406204489	1
ENSBTAG00000021635	*NUDT17*	621	361	477	538	417	-0.368	0.682455439	1
ENSBTAG00000021636	*GPR155*	311	166	231	269	192	-0.491	0.633680522	1
ENSBTAG00000021637	*POLR3C*	1'648	1'332	1'483	1'427	1'538	0.108	0.893160642	1
ENSBTAG00000021639	*ARC*	16	27	23	14	31	1.170	0.661901171	1
ENSBTAG00000021643	*C3ORF25*	29	4	15	25	5	-2.443	0.497961729	1
ENSBTAG00000021645	*BT.49400*	730	666	701	632	769	0.283	0.740229298	1
ENSBTAG00000021647	*FCAR*	1	0	0	1	0		1	1
ENSBTAG00000021651	*UQCRC2*	960	706	823	831	815	-0.028	0.97537557	1
ENSBTAG00000021652	*PDZD9*	55	14	32	48	16	-1.559	0.481455921	1
ENSBTAG00000021653	*TRIP12*	9'401	5'651	7'333	8'142	6'525	-0.319	0.678041058	1
ENSBTAG00000021654	*GAS2*	1'632	371	921	1'413	428	-1.722	0.042871581	1
ENSBTAG00000021656	*SPECC1L*	3'021	4'660	3'999	2'616	5'381	1.040	0.183116174	1
ENSBTAG00000021657	*CACNG6*	3	13	9	3	15	2.531	0.625618481	1
ENSBTAG00000021658	*GRASP*	1'720	1'852	1'814	1'490	2'139	0.522	0.512006328	1
ENSBTAG00000021660	*IKBIP*	2'528	1'233	1'807	2'189	1'424	-0.621	0.436725073	1
ENSBTAG00000021661	*APAF1*	331	276	303	287	319	0.153	0.876199105	1
ENSBTAG00000021663	*C9H6orf162*	291	199	241	252	230	-0.133	0.899866216	1
ENSBTAG00000021664	*TACR2*	48	35	41	42	40	-0.041	0.999082338	1
ENSBTAG00000021667	*BT.66660*	47	2	22	41	2	-4.140	0.186502445	1
ENSBTAG00000021668	*METTL4*	60	36	47	52	42	-0.322	0.868233214	1
ENSBTAG00000021671	*METTL18*	946	581	745	819	671	-0.288	0.734975175	1
ENSBTAG00000021672	*RGS1*	248	36	128	215	42	-2.369	0.059115884	1
ENSBTAG00000021673	*NDC80*	596	413	497	516	477	-0.114	0.900569952	1
ENSBTAG00000021675	*PJA2*	3'791	3'278	3'534	3'283	3'785	0.205	0.791211748	1
ENSBTAG00000021677	*BT.24470*	999	398	662	865	460	-0.913	0.28996162	1
ENSBTAG00000021678	*SLC31A1*	3'231	2'678	2'945	2'798	3'092	0.144	0.853117689	1
ENSBTAG00000021680	*BT.89449*	1'856	1'483	1'660	1'607	1'712	0.091	0.908898623	1
ENSBTAG00000021681	*BT.83549*	102	81	91	88	94	0.082	0.959238225	1
ENSBTAG00000021684	*protein_coding*	7	9	8	6	10	0.778	0.89868577	1
ENSBTAG00000021686	*MELK*	402	363	384	348	419	0.268	0.772844553	1
ENSBTAG00000021687	*PHF15*	757	946	874	656	1'092	0.737	0.378261798	1
ENSBTAG00000021688	*FH*	2'909	2'244	2'555	2'519	2'591	0.041	0.958564596	1
ENSBTAG00000021691	*PSMD14*	2'140	1'717	1'918	1'853	1'983	0.097	0.902229012	1
ENSBTAG00000021693	*RPP25*	17	8	12	15	9	-0.672	0.883107327	1
ENSBTAG00000021694	*SPAST*	3'004	1'407	2'113	2'602	1'625	-0.679	0.391836229	1

Ensembl gene ID	geneName	counts wildtype horn bud	counts polled horn bud	baseMean	baseMean wildtype horn bud	baseMean polled horn bud	log2FoldChange	pval	padj
ENSBTAG00000021695	*GLYCTK*	406	359	383	352	415	0.238	0.798235144	1
ENSBTAG00000021696	*CCIN*	10	22	17	9	25	1.553	0.625261719	1
ENSBTAG00000021697	*PDGFB*	1'608	1'422	1'517	1'393	1'642	0.238	0.766831699	1
ENSBTAG00000021699	*RORB*	17	1	8	15	1	-3.672	0.555170792	1
ENSBTAG00000021700	*MADD*	1'161	1'715	1'493	1'005	1'980	0.978	0.226413315	1
ENSBTAG00000021703	*MED12L*	79	57	67	68	66	-0.056	0.982840956	1
ENSBTAG00000021705	*UFC1*	5'330	3'682	4'434	4'616	4'252	-0.119	0.878963216	1
ENSBTAG00000021706	*TBX3*	283	107	184	245	124	-0.988	0.363871762	1
ENSBTAG00000021707	*MYBPC3*	8	25	18	7	29	2.059	0.506096367	1
ENSBTAG00000021708	*BT.63656*	118	101	109	102	117	0.191	0.885806274	1
ENSBTAG00000021709	*BT.58934*	370	181	265	320	209	-0.616	0.536002667	1
ENSBTAG00000021713	*MAMLD1*	57	16	34	49	18	-1.418	0.507501707	1
ENSBTAG00000021714	*TEKT2*	100	69	83	87	80	-0.120	0.941270491	1
ENSBTAG00000021715	*BT.51810*	1'235	1'458	1'377	1'070	1'684	0.655	0.418073129	1
ENSBTAG00000021717	*BT.72991*	88	138	118	76	159	1.064	0.39069497	1
ENSBTAG00000021720	*MRPL27*	2'181	2'031	2'117	1'889	2'345	0.312	0.69232734	1
ENSBTAG00000021723	*BT.53032*	1'332	830	1'056	1'154	958	-0.267	0.746022719	1
ENSBTAG00000021724	*PDHB*	2'847	2'120	2'457	2'466	2'448	-0.010	0.990784265	1
ENSBTAG00000021725	*BT.45537*	3'960	2'821	3'343	3'429	3'257	-0.074	0.92500991	1
ENSBTAG00000021726	*CWC22*	1'713	1'034	1'339	1'484	1'194	-0.313	0.69939329	1
ENSBTAG00000021727	*NTSR1*	30	1	14	26	1	-4.492	0.304362174	1
ENSBTAG00000021728	*OGDHL*	38	9	22	33	10	-1.663	0.548722021	1
ENSBTAG00000021729	*C4ORF50*	3	0	1	3	0		0.936647693	1
ENSBTAG00000021730	*GJD2*	6	3	4	5	3	-0.585	0.976586913	1
ENSBTAG00000021731	*TGFBRAP1*	1'216	1'734	1'528	1'053	2'002	0.927	0.250503226	1
ENSBTAG00000021735	*GALNT14*	549	1'065	853	475	1'230	1.371	0.105741241	1
ENSBTAG00000021739	*NPPB*	2	0	1	2	0		0.974934741	1
ENSBTAG00000021741	*RPS6KA2*	789	1'023	932	683	1'181	0.790	0.342504687	1
ENSBTAG00000021742	*ZNF568*	1'488	514	941	1'289	594	-1.118	0.181368513	1
ENSBTAG00000021743	*ZNF704*	44	27	35	38	31	-0.290	0.902842027	1
ENSBTAG00000021744	*BT.49089*	7'637	7'165	7'444	6'614	8'273	0.323	0.673252428	1
ENSBTAG00000021745	*RAPSN*	32	109	77	28	126	2.183	0.14186726	1
ENSBTAG00000021746	*BT.49277*	14'372	10'255	12'144	12'447	11'841	-0.072	0.925713776	1
ENSBTAG00000021747	*protein_coding*	8	0	3	7	0		0.72953145	1
ENSBTAG00000021751	*RASEF*	38	13	24	33	15	-1.132	0.664786809	1
ENSBTAG00000021752	*DNAJB4*	2'010	1'046	1'474	1'741	1'208	-0.527	0.513040291	1
ENSBTAG00000021754	*DUSP12*	1'312	733	991	1'136	846	-0.425	0.607864161	1
ENSBTAG00000021755	*processed_pseudogene*	3'876	7'383	5'941	3'357	8'525	1.345	0.085137522	1
ENSBTAG00000021756	*ZDHHC17*	1'442	811	1'093	1'249	936	-0.415	0.613352182	1
ENSBTAG00000021759	*NDUFB2*	891	770	830	772	889	0.204	0.807420419	1
ENSBTAG00000021761	*BRAF*	552	386	462	478	446	-0.101	0.913400162	1
ENSBTAG00000021762	*ZC3H15*	2'540	1'109	1'740	2'200	1'281	-0.781	0.329724172	1
ENSBTAG00000021764	*GLRB*	153	55	98	133	64	-1.061	0.424198181	1
ENSBTAG00000021766	*HBEGF*	345	141	231	299	163	-0.876	0.394158172	1
ENSBTAG00000021767	*UBE2N*	4'577	3'610	4'066	3'964	4'168	0.073	0.924889229	1
ENSBTAG00000021768	*BT.87531*	2'613	1'984	2'277	2'263	2'291	0.018	0.981964349	1
ENSBTAG00000021769	*BT.69487*	3'824	2'393	3'037	3'312	2'763	-0.261	0.738645154	1
ENSBTAG00000021770	*CRYGB*	1	0	0	1	0		1	1
ENSBTAG00000021771	*PTTG1IP*	9'915	7'168	8'432	8'587	8'277	-0.053	0.945638462	1
ENSBTAG00000021772	*BT.69069*	4'831	3'084	3'872	4'184	3'561	-0.232	0.765144774	1
ENSBTAG00000021773	*ERCC4*	275	202	236	238	233	-0.030	0.981980543	1
ENSBTAG00000021775	*SLC4A9*	27	6	15	23	7	-1.755	0.616167115	1
ENSBTAG00000021776	*NDUFV1*	2'159	2'861	2'587	1'870	3'304	0.821	0.297151117	1
ENSBTAG00000021777	*DOC2G*	160	53	100	139	61	-1.179	0.371814412	1
ENSBTAG00000021778	*BT.106328*	9'239	14'890	12'597	8'001	17'193	1.104	0.152279343	1
ENSBTAG00000021779	*MGST2*	2'329	2'387	2'387	2'017	2'756	0.451	0.566663019	1
ENSBTAG00000021780	*SCO1*	1'702	1'538	1'625	1'474	1'776	0.269	0.736384601	1
ENSBTAG00000021781	*BT.49482*	2'691	1'931	2'280	2'330	2'230	-0.064	0.936710153	1
ENSBTAG00000021785	*TMEM219*	1'663	1'477	1'573	1'440	1'705	0.244	0.760445863	1
ENSBTAG00000021786	*MGC157229*	199	134	164	172	155	-0.155	0.895109016	1
ENSBTAG00000021787	*PAQR7*	67	11	35	58	13	-2.192	0.303685275	1
ENSBTAG00000021789	*ZNF574*	1'065	1'252	1'184	922	1'446	0.648	0.426776228	1
ENSBTAG00000021790	*APOL*	1'033	522	749	895	603	-0.570	0.502234786	1
ENSBTAG00000021791	*PARP9*	1'066	700	866	923	808	-0.192	0.819943805	1
ENSBTAG00000021795	*BT.104940*	4	1	2	3	1	-1.585	0.952247636	1
ENSBTAG00000021796	*BT.26729*	722	951	862	625	1'098	0.812	0.33217434	1
ENSBTAG00000021798	*ERBB2*	4'968	8'824	7'246	4'302	10'189	1.244	0.109512721	1
ENSBTAG00000021799	*RCN3*	17'639	16'194	16'988	15'276	18'699	0.292	0.701252327	1
ENSBTAG00000021801	*INPP5F*	1'735	886	1'263	1'503	1'023	-0.555	0.495566902	1
ENSBTAG00000021802	*MIEN1*	876	609	731	759	703	-0.109	0.899491788	1
ENSBTAG00000021803	*GRAMD4*	1'582	1'609	1'614	1'370	1'858	0.439	0.582609627	1
ENSBTAG00000021805	*RIP5*	739	816	791	640	942	0.558	0.507559365	1

Ensembl gene ID	geneName	counts wildtype horn bud	counts polled horn bud	baseMean	baseMean wildtype horn bud	baseMean polled horn bud	log2FoldChange	pval	padj
ENSBTAG00000021807	*TAC3*	71	7	35	61	8	-2.927	0.186874684	1
ENSBTAG00000021808	*CACNB3*	2'720	3'746	3'341	2'356	4'326	0.877	0.262527442	1
ENSBTAG00000021809	*DEPDC7*	286	166	220	248	192	-0.370	0.723729201	1
ENSBTAG00000021810	*DDX23*	4'105	3'462	3'776	3'555	3'998	0.169	0.826849606	1
ENSBTAG00000021811	*ANGPTL4*	1'601	796	1'153	1'387	919	-0.593	0.468856902	1
ENSBTAG00000021813	*AMACR*	414	258	328	359	298	-0.267	0.781183974	1
ENSBTAG00000021814	*DDX43*	9	0	4	8	0		0.690676647	1
ENSBTAG00000021815	*STARD13*	2'142	835	1'410	1'855	964	-0.944	0.24466493	1
ENSBTAG00000021818	*CD97*	1'856	1'484	1'660	1'607	1'714	0.092	0.907929405	1
ENSBTAG00000021819	*IFNAR1*	1'227	1'088	1'159	1'063	1'256	0.242	0.767025441	1
ENSBTAG00000021820	*DDX39*	2'060	2'524	2'349	1'784	2'914	0.708	0.369462708	1
ENSBTAG00000021821	*pseudogene*	14	39	29	12	45	1.893	0.418506366	1
ENSBTAG00000021822	*BT.48907*	1'941	866	1'340	1'681	1'000	-0.749	0.356019049	1
ENSBTAG00000021827	*TCF21*	3	0	1	3	0		0.936647693	1
ENSBTAG00000021829	*GM2A*	945	870	911	818	1'005	0.296	0.721881454	1
ENSBTAG00000021830	*ENPP1*	1'422	1'307	1'370	1'231	1'509	0.293	0.716005913	1
ENSBTAG00000021831	*pseudogene*	111	171	147	96	197	1.038	0.369257001	1
ENSBTAG00000021832	*HMGCL*	1'233	904	1'056	1'068	1'044	-0.033	0.970168683	1
ENSBTAG00000021833	*GJA8*	2	3	3	2	3	1.000	0.97171833	1
ENSBTAG00000021835	*PRICKLE2*	129	122	126	112	141	0.335	0.784953401	1
ENSBTAG00000021836	*TCEA2*	1'707	1'195	1'429	1'478	1'380	-0.099	0.903178528	1
ENSBTAG00000021837	*RGS19*	651	680	674	564	785	0.478	0.576459364	1
ENSBTAG00000021838	*ESRP1*	2'577	1'286	1'858	2'232	1'485	-0.588	0.46082864	1
ENSBTAG00000021841	*CHD7*	716	164	405	620	189	-1.711	0.067804925	1
ENSBTAG00000021842	*FCGR2*	76	21	45	66	24	-1.441	0.436818231	1
ENSBTAG00000021843	*UBQLN2*	1'789	1'323	1'538	1'549	1'528	-0.020	0.981407528	1
ENSBTAG00000021844	*BT.27005*	148	29	81	128	33	-1.936	0.182848928	1
ENSBTAG00000021845	*STRN3*	3'771	2'220	2'915	3'266	2'563	-0.349	0.6556328	1
ENSBTAG00000021846	*CELSR3*	222	30	113	192	35	-2.472	0.059467602	1
ENSBTAG00000021849	*DUS4L*	341	208	268	295	240	-0.298	0.766083887	1
ENSBTAG00000021850	*NCKIPSD*	342	448	407	296	517	0.805	0.380426458	1
ENSBTAG00000021851	*BCAP29*	1'428	917	1'148	1'237	1'059	-0.224	0.785114264	1
ENSBTAG00000021853	*IP6K2*	3'073	2'397	2'715	2'661	2'768	0.057	0.942118228	1
ENSBTAG00000021855	*ANO3*	22	38	31	19	44	1.204	0.585044473	1
ENSBTAG00000021856	*C10ORF28*	1'016	662	822	880	764	-0.203	0.810413548	1
ENSBTAG00000021857	*ARHGAP21*	4'723	1'676	3'013	4'090	1'935	-1.080	0.170725798	1
ENSBTAG00000021858	*SLC5A12*	3	0	1	3	0		0.936647693	1
ENSBTAG00000021859	*PDSS2*	308	275	292	267	318	0.252	0.797183328	1
ENSBTAG00000021861	*BT.50723*	2'537	3'923	3'363	2'197	4'530	1.044	0.183434458	1
ENSBTAG00000021862	*SLC27A3*	1'147	1'378	1'292	993	1'591	0.680	0.402290718	1
ENSBTAG00000021864	*BT.27268*	1'394	643	975	1'207	742	-0.701	0.397984328	1
ENSBTAG00000021865	*MED31*	666	468	559	577	540	-0.094	0.917113484	1
ENSBTAG00000021867	*BFSP1*	170	177	176	147	204	0.473	0.666764002	1
ENSBTAG00000021869	*BT.25372*	419	288	348	363	333	-0.126	0.897015403	1
ENSBTAG00000021870	*FAM98B*	1'123	1'029	1'080	973	1'188	0.289	0.724423549	1
ENSBTAG00000021872	*BT.40228*	186	67	119	161	77	-1.058	0.393601508	1
ENSBTAG00000021874	*KIF14*	336	334	338	291	386	0.406	0.667469488	1
ENSBTAG00000021875	*SLC41A2*	550	236	374	476	273	-0.806	0.387476918	1
ENSBTAG00000021876	*WDR72*	1	0	0	1	0		1	1
ENSBTAG00000021879	*BT.102875*	10'073	9'117	9'625	8'723	10'527	0.271	0.722423946	1
ENSBTAG00000021880	*ADCK3*	658	394	512	570	455	-0.325	0.715199572	1
ENSBTAG00000021882	*protein_coding*	10	11	11	9	13	0.553	0.915317076	1
ENSBTAG00000021883	*BT.27280*	6'098	3'405	4'606	5'281	3'932	-0.426	0.582693677	1
ENSBTAG00000021884	*CXXC1*	3'350	3'177	3'285	2'901	3'668	0.339	0.663370279	1
ENSBTAG00000021885	*RSPO4*	12	1	6	10	1	-3.170	0.692545189	1
ENSBTAG00000021887	*DPYS*	2	2	2	2	2	0.415	1	1
ENSBTAG00000021893	*BT.24990*	2'140	2'304	2'257	1'853	2'660	0.522	0.508278258	1
ENSBTAG00000021894	*PPOX*	2'420	2'000	2'203	2'096	2'309	0.140	0.858812424	1
ENSBTAG00000021896	*NUP214*	2'203	3'650	3'061	1'908	4'215	1.143	0.146871795	1
ENSBTAG00000021897	*B4GALT3*	1'564	2'087	1'882	1'354	2'410	0.831	0.297202023	1
ENSBTAG00000021899	*PDE9A*	548	535	546	475	618	0.380	0.664678224	1
ENSBTAG00000021900	*PKN2*	1'461	1'022	1'223	1'265	1'180	-0.101	0.903165105	1
ENSBTAG00000021901	*TRPM2*	43	19	30	37	22	-0.763	0.744681388	1
ENSBTAG00000021902	*BT.105580*	361	455	419	313	525	0.749	0.411787925	1
ENSBTAG00000021905	*DGKB*	69	18	40	60	21	-1.524	0.436965169	1
ENSBTAG00000021906	*BT.52702*	59	4	28	51	5	-3.468	0.178173654	1
ENSBTAG00000021910	*TBL1XR1*	1'139	817	965	986	943	-0.064	0.9399751	1
ENSBTAG00000021911	*BT.92027*	765	552	650	663	637	-0.056	0.950843021	1
ENSBTAG00000021912	*DUSP7*	1'346	1'421	1'403	1'166	1'641	0.493	0.540592619	1
ENSBTAG00000021913	*MMD2*	25	2	12	22	2	-3.229	0.459170232	1
ENSBTAG00000021918	*EZH1*	3'945	2'542	3'176	3'416	2'935	-0.219	0.779538079	1
ENSBTAG00000021920	*SEMA4C*	4'075	5'248	4'794	3'529	6'060	0.780	0.314355715	1

Ensembl gene ID	geneName	counts wildtype horn bud	counts polled horn bud	baseMean	baseMean wildtype horn bud	baseMean polled horn bud	log2FoldChange	pval	padj
ENSBTAG00000021921	*STRBP*	1'465	1'225	1'342	1'269	1'415	0.157	0.84599989	1
ENSBTAG00000021922	*SMAP2*	1'014	689	837	878	796	-0.142	0.866749233	1
ENSBTAG00000021923	*DSG2*	1'227	1'775	1'556	1'063	2'050	0.948	0.239776397	1
ENSBTAG00000021924	*PLA2G4E*	197	78	130	171	90	-0.922	0.443858782	1
ENSBTAG00000021927	*AATF*	806	844	836	698	975	0.482	0.565290851	1
ENSBTAG00000021928	*FAM69B*	606	970	822	525	1'120	1.094	0.195676511	1
ENSBTAG00000021931	*BAP18*	2'094	1'956	2'036	1'813	2'259	0.317	0.688660879	1
ENSBTAG00000021932	*RNASEK*	3'105	3'647	3'450	2'689	4'211	0.647	0.406292994	1
ENSBTAG00000021933	*BT.92152*	736	807	785	637	932	0.548	0.515599233	1
ENSBTAG00000021934	*RETSAT*	730	360	524	632	416	-0.605	0.494377765	1
ENSBTAG00000021935	*ELMOD3*	1'598	1'648	1'643	1'384	1'903	0.459	0.565241158	1
ENSBTAG00000021938	*ARHGAP44*	173	114	141	150	132	-0.187	0.879089204	1
ENSBTAG00000021939	*ELAC2*	1'271	1'409	1'364	1'101	1'627	0.564	0.485334313	1
ENSBTAG00000021940	*CCDC149*	252	256	257	218	296	0.438	0.6616193	1
ENSBTAG00000021941	*BT.87382*	795	914	872	688	1'055	0.616	0.460520196	1
ENSBTAG00000021942	*HPS6*	330	600	489	286	693	1.278	0.155610371	1
ENSBTAG00000021943	*NFYC*	3'067	2'918	3'013	2'656	3'369	0.343	0.659820851	1
ENSBTAG00000021944	*C10H14ORF166*	15'571	13'836	14'731	13'485	15'976	0.245	0.747763823	1
ENSBTAG00000021945	*BT.96645*	21'376	5'889	12'656	18'512	6'800	-1.445	0.062921278	1
ENSBTAG00000021948	*ZMYM6*	1'148	572	827	994	660	-0.590	0.482933799	1
ENSBTAG00000021951	*RBM17*	1'547	1'258	1'396	1'340	1'453	0.117	0.884939267	1
ENSBTAG00000021953	*LARGE*	1'877	1'459	1'655	1'626	1'685	0.052	0.948573645	1
ENSBTAG00000021955	*NPC2*	11'493	6'644	8'813	9'953	7'672	-0.376	0.624545213	1
ENSBTAG00000021956	*ISCA2*	359	348	356	311	402	0.370	0.692995106	1
ENSBTAG00000021957	*LTBP2*	1'546	2'211	1'946	1'339	2'553	0.931	0.242956623	1
ENSBTAG00000021958	*BT.58970*	1	0	0	1	0		1	1
ENSBTAG00000021959	*CDC20B*	1	1	1	1	1	0.415	1	1
ENSBTAG00000021960	*GPX8*	11'880	7'152	9'273	10'288	8'258	-0.317	0.679340828	1
ENSBTAG00000021961	*SPR*	1'928	2'217	2'115	1'670	2'560	0.617	0.435849168	1
ENSBTAG00000021962	*BT.37525*	1'517	563	982	1'314	650	-1.015	0.22277646	1
ENSBTAG00000021963	*SLAIN2*	610	447	522	528	516	-0.033	0.972858986	1
ENSBTAG00000021964	*CDH17*	13	9	11	11	10	-0.115	1	1
ENSBTAG00000021965	*SUB1*	17'900	9'897	13'465	15'502	11'428	-0.440	0.565211469	1
ENSBTAG00000021967	*TMEM168*	1'130	910	1'015	979	1'051	0.103	0.901180528	1
ENSBTAG00000021968	*BT.89078*	156	92	121	135	106	-0.347	0.782408771	1
ENSBTAG00000021969	*BT.40893*	870	479	653	753	553	-0.446	0.604787575	1
ENSBTAG00000021970	*EYA4*	109	94	101	94	109	0.201	0.882544764	1
ENSBTAG00000021971	*SNCAIP*	1'930	871	1'339	1'671	1'006	-0.733	0.366673188	1
ENSBTAG00000021972	*DNAH5*	85	9	42	74	10	-2.824	0.159262936	1
ENSBTAG00000021974	*SMUG1*	707	780	756	612	901	0.557	0.510334191	1
ENSBTAG00000021975	*INADL*	430	394	414	372	455	0.289	0.752308805	1
ENSBTAG00000021976	*NDUFS1*	3'866	3'307	3'583	3'348	3'819	0.190	0.806609999	1
ENSBTAG00000021977	*PRRC1*	2'334	1'325	1'776	2'021	1'530	-0.402	0.614759993	1
ENSBTAG00000021978	*PARVB*	396	347	372	343	401	0.224	0.810243226	1
ENSBTAG00000021979	*EEF1B*	20'999	18'481	19'763	18'186	21'340	0.231	0.761174373	1
ENSBTAG00000021980	*C12ORF2*	1'061	712	870	919	822	-0.160	0.849236791	1
ENSBTAG00000021981	*TMEM18*	338	609	498	293	703	1.264	0.158768564	1
ENSBTAG00000021984	*C21ORF58*	25	29	28	22	33	0.629	0.796761755	1
ENSBTAG00000021986	*MAPK1IP1L*	1'212	739	951	1'050	853	-0.299	0.719559462	1
ENSBTAG00000021988	*SOCS4*	533	374	447	462	432	-0.096	0.918312769	1
ENSBTAG00000021991	*BT.52359*	350	275	310	303	318	0.067	0.946981626	1
ENSBTAG00000021992	*MURC*	621	462	536	538	533	-0.012	0.992624758	1
ENSBTAG00000021993	*SPDEF*	17	6	11	15	7	-1.087	0.813534849	1
ENSBTAG00000021994	*CACNA2D4*	1	1	1	1	1	0.415	1	1
ENSBTAG00000021995	*LRTM2*	34	110	78	29	127	2.109	0.151887019	1
ENSBTAG00000021996	*GATAD1*	152	118	134	132	136	0.050	0.97267962	1
ENSBTAG00000021997	*ITSN1*	2'188	2'336	2'296	1'895	2'697	0.509	0.517849435	1
ENSBTAG00000021999	*CPT1A*	1'233	788	989	1'068	910	-0.231	0.781109841	1
ENSBTAG00000022000	*CACNB2*	857	275	530	742	318	-1.225	0.169003372	1
ENSBTAG00000022003	*NSUN6*	534	454	493	462	524	0.181	0.83974714	1
ENSBTAG00000022004	*FLNB*	10'596	22'357	17'496	9'176	25'816	1.492	0.054572831	1
ENSBTAG00000022005	*C13H20orf118*	42	4	20	36	5	-2.977	0.320832869	1
ENSBTAG00000022006	*TRIM65*	1'431	997	1'195	1'239	1'151	-0.106	0.897679935	1
ENSBTAG00000022007	*BT.92388*	336	196	259	291	226	-0.363	0.718660933	1
ENSBTAG00000022009	*MRPL38*	1'690	1'608	1'660	1'464	1'857	0.343	0.667063833	1
ENSBTAG00000022013	*CBR4*	1'369	970	1'153	1'186	1'120	-0.082	0.92163927	1
ENSBTAG00000022019	*protein_coding*	23	10	16	20	12	-0.787	0.825334133	1
ENSBTAG00000022020	*CLDN5*	866	575	707	750	664	-0.176	0.838326981	1
ENSBTAG00000022022	*BT.22853*	1'912	1'960	1'960	1'656	2'263	0.451	0.569420077	1
ENSBTAG00000022027	*DDT*	507	497	506	439	574	0.386	0.663017582	1
ENSBTAG00000022028	*BT.21056*	52	86	72	45	99	1.141	0.442722887	1
ENSBTAG00000022032	*CHCHD10*	474	682	599	410	788	0.940	0.280657864	1

Ensembl gene ID	geneName	counts wildtype horn bud	counts polled horn bud	baseMean	baseMean wildtype horn bud	baseMean polled horn bud	log2FoldChange	pval	padj
ENSBTAG00000022036	*ZNRF3*	183	273	237	158	315	0.992	0.330693368	1
ENSBTAG00000022039	*CMLKR1*	142	193	173	123	223	0.858	0.436326681	1
ENSBTAG00000022044	*BT.26843*	2'504	2'584	2'576	2'169	2'984	0.460	0.557178956	1
ENSBTAG00000022058	*BT.31457*	120	73	94	104	84	-0.302	0.827974332	1
ENSBTAG00000022067	*FAM216A*	104	48	73	90	55	-0.700	0.63961974	1
ENSBTAG00000022069	*ATXN2*	3'699	1'667	2'564	3'203	1'925	-0.735	0.351101853	1
ENSBTAG00000022083	*REP15*	5	14	10	4	16	1.900	0.676791734	1
ENSBTAG00000022084	*protein_coding*	318	462	404	275	533	0.954	0.299737252	1
ENSBTAG00000022109	*SCOC*	2'931	1'713	2'258	2'538	1'978	-0.360	0.648849152	1
ENSBTAG00000022114	*TMEM65*	974	394	649	844	455	-0.891	0.302674133	1
ENSBTAG00000022120	*FGB*	38	12	23	33	14	-1.248	0.637252626	1
ENSBTAG00000022128	*processed_pseudogene*	2'188	2'586	2'440	1'895	2'986	0.656	0.404674342	1
ENSBTAG00000022137	*pseudogene*	2	5	4	2	6	1.737	0.883335552	1
ENSBTAG00000022142	*SCGB1C1*	17	6	11	15	7	-1.087	0.813534849	1
ENSBTAG00000022147	*EPS8L2*	1'247	1'289	1'284	1'080	1'488	0.463	0.567857328	1
ENSBTAG00000022150	*MXRA5*	10'494	13'034	12'069	9'088	15'050	0.728	0.342172776	1
ENSBTAG00000022155	*FSTL1*	73'559	114'335	97'863	63'704	132'023	1.051	0.16957146	1
ENSBTAG00000022160	*BT.46958*	5'583	5'031	5'322	4'835	5'809	0.265	0.730617473	1
ENSBTAG00000022161	*TLR7*	141	88	112	122	102	-0.265	0.839353255	1
ENSBTAG00000022167	*DERA*	1'378	961	1'152	1'193	1'110	-0.105	0.899331002	1
ENSBTAG00000022169	*PREX2*	195	125	157	169	144	-0.227	0.846551682	1
ENSBTAG00000022185	*IGHMBP2*	1'210	1'385	1'324	1'048	1'599	0.610	0.451336723	1
ENSBTAG00000022188	*PPFIA1*	1'575	1'055	1'291	1'364	1'218	-0.163	0.84181109	1
ENSBTAG00000022199	*pseudogene*	5'232	4'538	4'886	4'531	5'240	0.210	0.785305413	1
ENSBTAG00000022204	*TCEAL4*	3'489	2'097	2'721	3'022	2'421	-0.319	0.684119215	1
ENSBTAG00000022209	*CABP2*	3	2	2	3	2	-0.170	1	1
ENSBTAG00000022227	*PLSCR2*	910	597	739	788	689	-0.193	0.821537034	1
ENSBTAG00000022238	*B3GNT1*	1'912	1'633	1'771	1'656	1'886	0.187	0.813591465	1
ENSBTAG00000022242	*BT.49074*	1'082	1'236	1'182	937	1'427	0.607	0.456743265	1
ENSBTAG00000022255	*AR*	24	38	32	21	44	1.078	0.620102398	1
ENSBTAG00000022275	*RPL10*	13'145	25'667	20'511	11'384	29'638	1.380	0.074336651	1
ENSBTAG00000022288	*AMMECR1*	150	82	112	130	95	-0.456	0.720828299	1
ENSBTAG00000022292	*TIMM8A*	934	475	679	809	548	-0.560	0.513517034	1
ENSBTAG00000022293	*METTL12*	347	408	386	301	471	0.649	0.482643696	1
ENSBTAG00000022294	*FADS1*	860	387	596	745	447	-0.737	0.397699986	1
ENSBTAG00000022314	*EMD*	2'674	2'819	2'785	2'316	3'255	0.491	0.530195228	1
ENSBTAG00000022329	*SLCO1B3*	3	0	1	3	0		0.936647693	1
ENSBTAG00000022360	*SOX5*	99	57	76	86	66	-0.381	0.799792486	1
ENSBTAG00000022372	*pseudogene*	4'134	3'453	3'784	3'580	3'987	0.155	0.840844627	1
ENSBTAG00000022373	*BT.25030*	171	120	143	148	139	-0.096	0.941192948	1
ENSBTAG00000022379	*VSIG2*	56	32	43	48	37	-0.392	0.844443139	1
ENSBTAG00000022381	*TET3*	240	229	236	208	264	0.347	0.734000261	1
ENSBTAG00000022382	*TMEM218*	497	379	434	430	438	0.024	0.981012565	1
ENSBTAG00000022394	*SAA1*	1	0	0	1	0		1	1
ENSBTAG00000022396	*SAA3*	237	142	185	205	164	-0.324	0.768186711	1
ENSBTAG00000022399	*pseudogene*	15	8	11	13	9	-0.492	0.927244877	1
ENSBTAG00000022427	*protein_coding*	26	93	65	23	107	2.254	0.157454387	1
ENSBTAG00000022449	*SCD5*	2'883	829	1'727	2'497	957	-1.383	0.087613992	1
ENSBTAG00000022450	*THAP9*	1'205	915	1'050	1'044	1'057	0.018	0.983277547	1
ENSBTAG00000022460	*processed_pseudogene*	98	22	55	85	25	-1.740	0.304866026	1
ENSBTAG00000022461	*BT.63999*	4'662	3'163	3'845	4'037	3'652	-0.145	0.852964896	1
ENSBTAG00000022471	*TTPA*	3	2	2	3	2	-0.170	1	1
ENSBTAG00000022484	*SOX21*	117	91	103	101	105	0.052	0.975130719	1
ENSBTAG00000022489	*BT.63387*	1'310	737	993	1'134	851	-0.415	0.616359127	1
ENSBTAG00000022498	*BT.87344*	33	24	28	29	28	-0.044	1	1
ENSBTAG00000022509	*DNAH9*	36	9	21	31	10	-1.585	0.577080965	1
ENSBTAG00000022514	*BT.9455*	2	0	1	2	0		0.974934741	1
ENSBTAG00000022520	*BRCA1*	393	421	413	340	486	0.514	0.573356585	1
ENSBTAG00000022529	*protein_coding*	1	1	1	1	1	0.415	1	1
ENSBTAG00000022530	*TMEM186*	188	182	186	163	210	0.368	0.734315719	1
ENSBTAG00000022535	*GPR139*	3	0	1	3	0		0.936647693	1
ENSBTAG00000022539	*POLR2K*	2'285	1'106	1'628	1'979	1'277	-0.632	0.430995693	1
ENSBTAG00000022562	*pseudogene*	1	12	7	1	14	4.000	0.538008997	1
ENSBTAG00000022564	*BT.63212*	7	6	6	6	7	0.193	1	1
ENSBTAG00000022575	*TPRA1*	1'071	1'003	1'043	928	1'158	0.320	0.696589428	1
ENSBTAG00000022580	*FAM212B*	266	246	257	230	284	0.302	0.763342724	1
ENSBTAG00000022583	*SHC4*	70	27	46	61	31	-0.959	0.601097868	1
ENSBTAG00000022588	*C8ORF34*	84	21	48	73	24	-1.585	0.376610146	1
ENSBTAG00000022598	*NRADD*	1'320	1'609	1'501	1'143	1'858	0.701	0.383968678	1
ENSBTAG00000022613	*CYCS*	664	658	667	575	760	0.402	0.638882132	1
ENSBTAG00000022622	*CISH*	457	695	599	396	803	1.020	0.242367542	1
ENSBTAG00000022632	*TCTA*	978	831	903	847	960	0.180	0.828810398	1

Ensembl gene ID	geneName	counts wildtype horn bud	counts polled horn bud	baseMean	baseMean wildtype horn bud	baseMean polled horn bud	log2FoldChange	pval	padj
ENSBTAG00000022635	*BT.106077*	14'806	17'271	16'383	12'822	19'943	0.637	0.404076894	1
ENSBTAG00000022647	*processed_pseudogene*	997	2'353	1'790	863	2'717	1.654	0.042090674	1
ENSBTAG00000022656	*KCTD6*	965	429	666	836	495	-0.755	0.380582377	1
ENSBTAG00000022671	*WNT4*	168	359	280	145	415	1.511	0.128634311	1
ENSBTAG00000022681	*EOGT*	1'181	503	802	1'023	581	-0.816	0.333929935	1
ENSBTAG00000022689	*RYBP*	635	664	658	550	767	0.479	0.576248126	1
ENSBTAG00000022714	*HSPB3*	10	26	19	9	30	1.794	0.541131366	1
ENSBTAG00000022715	*DMBT1*	7	0	3	6	0		0.769774961	1
ENSBTAG00000022721	*SLC25A38*	1'488	1'571	1'551	1'289	1'814	0.493	0.538263799	1
ENSBTAG00000022731	*SSR1*	17'385	9'988	13'295	15'056	11'533	-0.385	0.615092685	1
ENSBTAG00000022733	*EPM2AIP1*	635	547	591	550	632	0.200	0.818662124	1
ENSBTAG00000022736	*pseudogene*	2	2	2	2	2	0.415	1	1
ENSBTAG00000022741	*MGC134040*	1	0	0	1	0		1	1
ENSBTAG00000022744	*TRIM71*	31	17	23	27	20	-0.452	0.876164144	1
ENSBTAG00000022751	*ZCWPW2*	143	55	94	124	64	-0.963	0.475183524	1
ENSBTAG00000022762	*pseudogene*	74	187	140	64	216	1.752	0.141125103	1
ENSBTAG00000022775	*C14ORF180*	12	20	17	10	23	1.152	0.722217561	1
ENSBTAG00000022777	*CDC42BPA*	1'197	1'043	1'120	1'037	1'204	0.216	0.79129774	1
ENSBTAG00000022779	*OLFM4*	3	0	1	3	0		0.936647693	1
ENSBTAG00000022783	*BT.37690*	182	23	92	158	27	-2.569	0.069917636	1
ENSBTAG00000022796	*pseudogene*	7'858	7'885	7'955	6'805	9'105	0.420	0.583572083	1
ENSBTAG00000022799	*NOTCH1*	4'197	8'573	6'767	3'635	9'899	1.445	0.064392829	1
ENSBTAG00000022801	*BT.37836*	542	398	464	469	460	-0.030	0.976311915	1
ENSBTAG00000022807	*BT.89096*	0	1	1	0	1	Inf	0.993540919	1
ENSBTAG00000022808	*C10ORF46*	129	103	115	112	119	0.090	0.94855574	1
ENSBTAG00000022813	*TMEM150B*	167	140	153	145	162	0.161	0.892074047	1
ENSBTAG00000022819	*BT.88609*	8	1	4	7	1	-2.585	0.818599781	1
ENSBTAG00000022825	*BRI3*	1'517	1'651	1'610	1'314	1'906	0.537	0.502136842	1
ENSBTAG00000022829	*protein_coding*	8	10	9	7	12	0.737	0.893887116	1
ENSBTAG00000022837	*ZNF75*	371	220	288	321	254	-0.339	0.730625933	1
ENSBTAG00000022847	*HEATR5A*	2'846	1'525	2'113	2'465	1'761	-0.485	0.540346545	1
ENSBTAG00000022880	*pseudogene*	55	57	57	48	66	0.467	0.78133014	1
ENSBTAG00000022887	*ZNF407*	648	659	661	561	761	0.439	0.608399364	1
ENSBTAG00000022893	*CD1B3*	6	2	4	5	2	-1.170	0.934316631	1
ENSBTAG00000022895	*PQLC1*	220	238	233	191	275	0.528	0.60505855	1
ENSBTAG00000022917	*BT.34203*	98	23	56	85	27	-1.676	0.320005403	1
ENSBTAG00000022920	*BT.29416*	1'078	489	749	934	565	-0.725	0.393255808	1
ENSBTAG00000022922	*BT.87961*	149	74	107	129	85	-0.595	0.644790419	1
ENSBTAG00000022927	*RAC3*	1'102	817	949	954	943	-0.017	0.986098192	1
ENSBTAG00000022931	*BT.91662*	30	8	18	26	9	-1.492	0.637063859	1
ENSBTAG00000022938	*ITLN2*	1	0	0	1	0		1	1
ENSBTAG00000022942	*protein_coding*	1	0	0	1	0		1	1
ENSBTAG00000022954	*protein_coding*	203	209	209	176	241	0.457	0.663535914	1
ENSBTAG00000022960	*HEPHL1*	23	1	11	20	1	-4.109	0.421841907	1
ENSBTAG00000022962	*DGKK*	0	1	1	0	1	Inf	0.993540919	1
ENSBTAG00000022986	*BT.66476*	76	9	38	66	10	-2.663	0.202668001	1
ENSBTAG00000022989	*BT.22149*	143	278	222	124	321	1.374	0.187279028	1
ENSBTAG00000022991	*NBEA*	1'633	608	1'058	1'414	702	-1.010	0.222214781	1
ENSBTAG00000023002	*JPH2*	29	39	35	25	45	0.842	0.687420457	1
ENSBTAG00000023007	*protein_coding*	6	3	4	5	3	-0.585	0.976586913	1
ENSBTAG00000023018	*BT.65122*	2'434	1'561	1'955	2'108	1'802	-0.226	0.776712904	1
ENSBTAG00000023026	*BT.58893*	178	359	284	154	415	1.427	0.14901638	1
ENSBTAG00000023028	*WDFY4*	419	183	287	363	211	-0.780	0.425756366	1
ENSBTAG00000023032	*MBL-A*	1	0	0	1	0		1	1
ENSBTAG00000023039	*BT.44881*	4	39	24	3	45	3.700	0.181797448	1
ENSBTAG00000023054	*SLC25A16*	723	415	553	626	479	-0.386	0.661065779	1
ENSBTAG00000023064	*protein_coding*	9	32	22	8	37	2.245	0.408315219	1
ENSBTAG00000023073	*FAM89A*	349	194	263	302	224	-0.432	0.665688422	1
ENSBTAG00000023074	*C1ORF198*	757	1'067	944	656	1'232	0.910	0.274298774	1
ENSBTAG00000023078	*pseudogene*	38	90	68	33	104	1.659	0.278986699	1
ENSBTAG00000023106	*SETMAR*	1'405	865	1'108	1'217	999	-0.285	0.729081289	1
ENSBTAG00000023143	*pseudogene*	3	0	1	3	0		0.936647693	1
ENSBTAG00000023144	*PTPRC*	490	105	273	424	121	-1.807	0.073561611	1
ENSBTAG00000023146	*ADAM32*	2	6	4	2	7	2.000	0.840116992	1
ENSBTAG00000023147	*LINGO1*	486	591	552	421	682	0.697	0.426781098	1
ENSBTAG00000023157	*protein_coding*	0	2	1	0	2	Inf	0.939077559	1
ENSBTAG00000023163	*protein_coding*	4	5	5	3	6	0.737	0.954105567	1
ENSBTAG00000023169	*protein_coding*	851	549	685	737	634	-0.217	0.800931282	1
ENSBTAG00000023172	*ITSN2*	1'460	875	1'137	1'264	1'010	-0.324	0.693156265	1
ENSBTAG00000023177	*BT.106251*	9	1	4	8	1	-2.755	0.785977687	1
ENSBTAG00000023179	*TRIB1*	542	555	555	469	641	0.449	0.607894931	1
ENSBTAG00000023182	*WNK3*	15	9	12	13	10	-0.322	0.959295425	1

Ensembl gene ID	geneName	counts wildtype horn bud	counts polled horn bud	baseMean	baseMean wildtype horn bud	baseMean polled horn bud	log2FoldChange	pval	padj
ENSBTAG00000023186	*MRPS17*	73	230	164	63	266	2.071	0.07133436	1
ENSBTAG00000023187	*protein_coding*	0	4	2	0	5	Inf	0.817359038	1
ENSBTAG00000023198	*BT.53705*	505	209	339	437	241	-0.858	0.366024529	1
ENSBTAG00000023206	*pseudogene*	0	2	1	0	2	Inf	0.939077559	1
ENSBTAG00000023207	*FAM3D*	3	4	4	3	5	0.830	0.962490925	1
ENSBTAG00000023216	*C10ORF112*	1	1	1	1	1	0.415	1	1
ENSBTAG00000023218	*UBE2V2*	2'116	1'255	1'641	1'833	1'449	-0.339	0.672747235	1
ENSBTAG00000023249	*processed_pseudogene*	1	0	0	1	0		1	1
ENSBTAG00000023259	*OSBPL1A*	1'082	510	763	937	589	-0.670	0.429305809	1
ENSBTAG00000023270	*CDH19*	617	118	335	534	136	-1.971	0.043249532	1
ENSBTAG00000023274	*protein_coding*	40'765	61'592	53'212	35'304	71'120	1.010	0.186771929	1
ENSBTAG00000023277	*processed_pseudogene*	2	2	2	2	2	0.415	1	1
ENSBTAG00000023279	*N4BP2L2*	2'242	1'341	1'745	1'942	1'548	-0.326	0.682967711	1
ENSBTAG00000023283	*AMICA1*	56	13	32	48	15	-1.692	0.446973729	1
ENSBTAG00000023289	*BT.30449*	200	221	214	173	255	0.559	0.591661594	1
ENSBTAG00000023309	*BT.82323*	1	2	2	1	2	1.415	0.981979269	1
ENSBTAG00000023318	*processed_pseudogene*	57	49	53	49	57	0.197	0.916904578	1
ENSBTAG00000023333	*LENG1*	364	321	343	315	371	0.234	0.805415838	1
ENSBTAG00000023338	*bta-mir-2900*	2'597	1'166	1'798	2'249	1'346	-0.740	0.354350012	1
ENSBTAG00000023343	*RPL28*	17'835	19'105	18'753	15'446	22'061	0.514	0.499738662	1
ENSBTAG00000023365	*protein_coding*	30	29	30	26	33	0.366	0.882165907	1
ENSBTAG00000023369	*GRIN2D*	386	77	212	334	89	-1.911	0.075000839	1
ENSBTAG00000023372	*MYCN*	273	234	253	236	270	0.193	0.849666855	1
ENSBTAG00000023374	*FUT1*	253	280	271	219	323	0.561	0.569682658	1
ENSBTAG00000023375	*METTL6*	1'196	643	889	1'036	742	-0.480	0.565345862	1
ENSBTAG00000023377	*SH3BP5*	1'744	946	1'301	1'510	1'092	-0.467	0.564830716	1
ENSBTAG00000023384	*CBR1*	199	146	170	172	169	-0.032	0.983948895	1
ENSBTAG00000023398	*UBASH3A*	4	3	3	3	3	0.000	1	1
ENSBTAG00000023407	*B3GALT5*	6	1	3	5	1	-2.170	0.885240772	1
ENSBTAG00000023411	*BT.52938*	148	62	100	128	72	-0.840	0.523841641	1
ENSBTAG00000023415	*LIN7B*	456	367	409	395	424	0.102	0.913123445	1
ENSBTAG00000023416	*BT.104689*	2'390	1'684	2'007	2'070	1'945	-0.090	0.910649569	1
ENSBTAG00000023417	*RUVBL2*	1'627	2'308	2'037	1'409	2'665	0.919	0.247948247	1
ENSBTAG00000023419	*SULT2A1*	1	1	1	1	1	0.415	1	1
ENSBTAG00000023426	*FAIM*	915	558	718	792	644	-0.298	0.726906979	1
ENSBTAG00000023439	*protein_coding*	0	1	1	0	1	Inf	0.993540919	1
ENSBTAG00000023445	*SIAH2*	1'172	905	1'030	1'015	1'045	0.042	0.959782734	1
ENSBTAG00000023452	*B9D2*	468	550	520	405	635	0.648	0.463411247	1
ENSBTAG00000023453	*C18H19orf54*	428	582	521	371	672	0.858	0.332273766	1
ENSBTAG00000023462	*protein_coding*	15'848	19'067	17'871	13'725	22'017	0.682	0.372051992	1
ENSBTAG00000023464	*ZFP82*	641	470	549	555	543	-0.033	0.97335056	1
ENSBTAG00000023471	*RPL36*	10'030	15'182	13'108	8'686	17'531	1.013	0.187901194	1
ENSBTAG00000023472	*PPP1R14A*	226	157	189	196	181	-0.111	0.923768947	1
ENSBTAG00000023487	*COX6B1*	3'414	1'916	2'585	2'957	2'212	-0.418	0.59471505	1
ENSBTAG00000023513	*BT.59126*	184	238	217	159	275	0.786	0.449095496	1
ENSBTAG00000023523	*GKAP1*	781	362	547	676	418	-0.694	0.430340387	1
ENSBTAG00000023529	*SPEF2*	65	54	59	56	62	0.148	0.936455599	1
ENSBTAG00000023530	*miRNA*	26	10	17	23	12	-0.963	0.770075479	1
ENSBTAG00000023550	*pseudogene*	11	12	12	10	14	0.541	0.910156842	1
ENSBTAG00000023551	*MOB3C*	1'014	1'314	1'198	878	1'517	0.789	0.334041704	1
ENSBTAG00000023562	*KCNK6*	105	111	110	91	128	0.495	0.697927076	1
ENSBTAG00000023563	*BT.28475*	2	0	1	2	0		0.974934741	1
ENSBTAG00000023593	*GMNC*	1	0	0	1	0		1	1
ENSBTAG00000023600	*APOD*	748	301	498	648	348	-0.898	0.31461387	1
ENSBTAG00000023601	*CD3EAP*	760	1'030	924	658	1'189	0.854	0.305696873	1
ENSBTAG00000023607	*PTPLB*	1'138	849	983	986	980	-0.008	0.994781798	1
ENSBTAG00000023610	*U2AF1L4*	1'466	1'224	1'341	1'270	1'413	0.155	0.84810164	1
ENSBTAG00000023611	*ZBTB32*	53	24	37	46	28	-0.728	0.72581296	1
ENSBTAG00000023614	*BT.52325*	358	224	284	310	259	-0.261	0.792075558	1
ENSBTAG00000023617	*protein_coding*	2	2	2	2	2	0.415	1	1
ENSBTAG00000023628	*C23H6ORF47*	615	646	639	533	746	0.486	0.572352256	1
ENSBTAG00000023629	*protein_coding*	198	116	153	171	134	-0.356	0.758663199	1
ENSBTAG00000023632	*BT.29334*	423	403	416	366	465	0.345	0.705555161	1
ENSBTAG00000023635	*CLEC6A*	27	10	17	23	12	-1.018	0.752367523	1
ENSBTAG00000023648	*BT.91045*	5	2	3	4	2	-0.907	0.965043111	1
ENSBTAG00000023652	*PROS1*	11'221	6'676	8'713	9'718	7'709	-0.334	0.663343084	1
ENSBTAG00000023659	*MT2*	180	24	92	156	28	-2.492	0.078128696	1
ENSBTAG00000023666	*protein_coding*	5	6	6	4	7	0.678	0.946418607	1
ENSBTAG00000023670	*protein_coding*	614	560	589	532	647	0.282	0.745638239	1
ENSBTAG00000023675	*protein_coding*	795	360	552	688	416	-0.728	0.407915756	1
ENSBTAG00000023697	*protein_coding*	27	13	19	23	15	-0.639	0.839610081	1
ENSBTAG00000023718	*MRAP*	9	5	7	8	6	-0.433	0.969858938	1

Ensembl gene ID	geneName	counts wildtype horn bud	counts polled horn bud	baseMean	baseMean wildtype horn bud	baseMean polled horn bud	log2FoldChange	pval	padj
ENSBTAG00000023726	*protein_coding*	1	1	1	1	1	0.415	1	1
ENSBTAG00000023730	*TUBB3*	5'273	1'410	3'097	4'567	1'628	-1.488	0.061095625	1
ENSBTAG00000023731	*MC1-R*	82	49	64	71	57	-0.328	0.842083948	1
ENSBTAG00000023734	*BT.20795*	102	87	94	88	100	0.186	0.895655363	1
ENSBTAG00000023736	*CDC42EP3*	2'510	1'761	2'104	2'174	2'033	-0.096	0.904243226	1
ENSBTAG00000023744	*YPEL5*	2'588	1'071	1'739	2'241	1'237	-0.858	0.284554451	1
ENSBTAG00000023745	*BANP*	770	868	835	667	1'002	0.588	0.483051303	1
ENSBTAG00000023752	*PABPN1L*	3	7	5	3	8	1.637	0.840440358	1
ENSBTAG00000023765	*C16ORF46*	84	71	77	73	82	0.172	0.9121644	1
ENSBTAG00000023776	*C11H9orf116*	261	268	268	226	309	0.453	0.6474052	1
ENSBTAG00000023780	*SYCE1L*	133	54	89	115	62	-0.885	0.520268787	1
ENSBTAG00000023784	*TMEM88B*	75	45	58	65	52	-0.322	0.851641124	1
ENSBTAG00000023787	*C9ORF167*	16	15	16	14	17	0.322	0.940397404	1
ENSBTAG00000023788	*EXD3*	256	352	314	222	406	0.874	0.362478799	1
ENSBTAG00000023792	*protein_coding*	23'203	33'790	29'556	20'094	39'017	0.957	0.211182856	1
ENSBTAG00000023795	*BT.62229*	1'633	2'570	2'191	1'414	2'968	1.069	0.179008127	1
ENSBTAG00000023806	*COBL*	111	198	162	96	229	1.250	0.267395534	1
ENSBTAG00000023814	*BT.28366*	1'331	1'006	1'157	1'153	1'162	0.011	0.989607732	1
ENSBTAG00000023823	*CYCS*	731	547	632	633	632	-0.003	0.999851164	1
ENSBTAG00000023831	*BT.89060*	406	320	361	352	370	0.072	0.941135863	1
ENSBTAG00000023832	*ADAM8*	377	534	472	326	617	0.917	0.307227601	1
ENSBTAG00000023840	*BT.19472*	275	358	326	238	413	0.796	0.403779689	1
ENSBTAG00000023843	*RALGPS1*	150	81	112	130	94	-0.474	0.710696552	1
ENSBTAG00000023845	*protein_coding*	284	305	299	246	352	0.518	0.592528165	1
ENSBTAG00000023846	*BT.76461*	716	404	543	620	466	-0.411	0.641463989	1
ENSBTAG00000023847	*IKZF5*	1'377	989	1'167	1'193	1'142	-0.062	0.9407053	1
ENSBTAG00000023851	*BT.44867*	667	1'217	991	578	1'405	1.283	0.124673437	1
ENSBTAG00000023860	*protein_coding*	80	81	81	69	94	0.433	0.762923363	1
ENSBTAG00000023867	*BT.43222*	318	217	263	275	251	-0.136	0.895067668	1
ENSBTAG00000023885	*ZNF79*	884	567	710	766	655	-0.226	0.792556632	1
ENSBTAG00000023891	*RBM20*	75	58	66	65	67	0.044	0.987489804	1
ENSBTAG00000023907	*BT.11942*	14'999	12'169	13'521	12'990	14'052	0.113	0.881210398	1
ENSBTAG00000023920	*GPR158*	5	1	3	4	1	-1.907	0.9188325	1
ENSBTAG00000023928	*RDH14*	595	324	445	515	374	-0.462	0.610456787	1
ENSBTAG00000023929	*FOSL2*	4'367	4'742	4'629	3'782	5'476	0.534	0.489920363	1
ENSBTAG00000023933	*SEC16A*	4'104	8'467	6'666	3'554	9'777	1.460	0.061928928	1
ENSBTAG00000023938	*BT.66169*	828	388	583	717	448	-0.679	0.437240261	1
ENSBTAG00000023939	*BT.55395*	21	9	14	18	10	-0.807	0.832834108	1
ENSBTAG00000023947	*AVPI1*	212	465	360	184	537	1.548	0.102901348	1
ENSBTAG00000023954	*protein_coding*	19	4	11	16	5	-1.833	0.688634464	1
ENSBTAG00000023955	*BT.42112*	2	5	4	2	6	1.737	0.883335552	1
ENSBTAG00000023963	*RHBDD1*	1'014	974	1'001	878	1'125	0.357	0.664973026	1
ENSBTAG00000023964	*protein_coding*	1	0	0	1	0		1	1
ENSBTAG00000023967	*pseudogene*	3	0	1	3	0		0.936647693	1
ENSBTAG00000023970	*protein_coding*	0	21	12	0	24	Inf	0.20026095	1
ENSBTAG00000023971	*pseudogene*	17	29	24	15	33	1.186	0.6441502	1
ENSBTAG00000023976	*ARHGAP25*	538	493	518	466	569	0.289	0.743958549	1
ENSBTAG00000023978	*protein_coding*	67	71	70	58	82	0.499	0.743262561	1
ENSBTAG00000023986	*protein_coding*	82	54	67	71	62	-0.188	0.91262763	1
ENSBTAG00000023989	*MSANTD4*	1'708	1'280	1'479	1'479	1'478	-0.001	1	1
ENSBTAG00000023997	*VAMP8*	1'526	976	1'224	1'322	1'127	-0.230	0.778659337	1
ENSBTAG00000023998	*protein_coding*	1	0	0	1	0		1	1
ENSBTAG00000023999	*BT.104044*	15	9	12	13	10	-0.322	0.959295425	1
ENSBTAG00000024000	*ATOH8*	578	891	765	501	1'029	1.039	0.221489889	1
ENSBTAG00000024015	*PTPRM*	3'541	3'138	3'345	3'067	3'623	0.241	0.756630761	1
ENSBTAG00000024027	*RG9MTD1*	1'251	741	970	1'083	856	-0.340	0.681650025	1
ENSBTAG00000024042	*MORN2*	613	293	435	531	338	-0.650	0.474722619	1
ENSBTAG00000024043	*pseudogene*	1	0	0	1	0		1	1
ENSBTAG00000024044	*BT.38522*	44	22	32	38	25	-0.585	0.798419738	1
ENSBTAG00000024058	*BT.29719*	1	0	0	1	0		1	1
ENSBTAG00000024075	*SLC9A4*	4	1	2	3	1	-1.585	0.952247636	1
ENSBTAG00000024081	*BT.42321*	6'444	7'139	6'912	5'581	8'243	0.563	0.464067339	1
ENSBTAG00000024086	*ZMYM6NB*	397	322	358	344	372	0.113	0.90585066	1
ENSBTAG00000024088	*pseudogene*	3	1	2	3	1	-1.170	0.985253077	1
ENSBTAG00000024091	*MALL*	1'012	835	920	876	964	0.138	0.868636114	1
ENSBTAG00000024095	*THRAP3*	8'575	9'123	8'980	7'426	10'534	0.504	0.510155758	1
ENSBTAG00000024096	*LSM10*	336	323	332	291	373	0.358	0.706248115	1
ENSBTAG00000024097	*MRPS15*	1'512	984	1'223	1'309	1'136	-0.205	0.802480367	1
ENSBTAG00000024105	*BCL2L11*	61	82	74	53	95	0.842	0.567634128	1
ENSBTAG00000024107	*INPP5B*	1'993	1'216	1'565	1'726	1'404	-0.298	0.711135661	1
ENSBTAG00000024125	*RPS27*	8	5	6	7	6	-0.263	0.996869191	1
ENSBTAG00000024132	*SMC1B*	1	0	0	1	0		1	1

Ensembl gene ID	geneName	counts wildtype horn bud	counts polled horn bud	baseMean	baseMean wildtype horn bud	baseMean polled horn bud	log2FoldChange	pval	padj
ENSBTAG00000024144	*C1ORF190*	13	17	15	11	20	0.802	0.820295848	1
ENSBTAG00000024157	*WDR60*	792	681	736	686	786	0.197	0.816409934	1
ENSBTAG00000024162	*BT.27229*	562	717	657	487	828	0.766	0.372813973	1
ENSBTAG00000024175	*HIST1H3H*	0	1	1	0	1	Inf	0.993540919	1
ENSBTAG00000024176	*HIST1H2AG*	8	8	8	7	9	0.415	0.960311941	1
ENSBTAG00000024177	*H2B*	8	2	5	7	2	-1.585	0.87391615	1
ENSBTAG00000024178	*H4*	2	1	1	2	1	-0.585	1	1
ENSBTAG00000024179	*H4*	0	3	2	0	3	Inf	0.878646179	1
ENSBTAG00000024180	*HIST1H2AD*	4	2	3	3	2	-0.585	0.996239345	1
ENSBTAG00000024182	*H2B*	30	19	24	26	22	-0.244	0.941091264	1
ENSBTAG00000024183	*HIST1H2AG*	5	6	6	4	7	0.678	0.946418607	1
ENSBTAG00000024184	*protein_coding*	0	1	1	0	1	Inf	0.993540919	1
ENSBTAG00000024185	*H4*	5	2	3	4	2	-0.907	0.965043111	1
ENSBTAG00000024186	*protein_coding*	6	0	3	5	0		0.81115604	1
ENSBTAG00000024187	*HIST1H2AG*	5	2	3	4	2	-0.907	0.965043111	1
ENSBTAG00000024188	*H2B*	1	6	4	1	7	3.000	0.788284548	1
ENSBTAG00000024195	*LDLRAD1*	2	0	1	2	0		0.974934741	1
ENSBTAG00000024199	*MLL3*	4'254	3'329	3'764	3'684	3'844	0.061	0.936660945	1
ENSBTAG00000024201	*protein_coding*	2	1	1	2	1	-0.585	1	1
ENSBTAG00000024204	*protein_coding*	0	4	2	0	5	Inf	0.817359038	1
ENSBTAG00000024206	*pseudogene*	7	4	5	6	5	-0.392	0.98714753	1
ENSBTAG00000024219	*TRPV6*	173	257	223	150	297	0.986	0.340210227	1
ENSBTAG00000024233	*BT.64943*	46	10	26	40	12	-1.787	0.476442628	1
ENSBTAG00000024240	*ACADM*	1'558	491	958	1'349	567	-1.251	0.135431662	1
ENSBTAG00000024245	*protein_coding*	2	0	1	2	0		0.974934741	1
ENSBTAG00000024255	*UOX*	1	0	0	1	0		1	1
ENSBTAG00000024272	*BT.37579*	61	13	34	53	15	-1.815	0.399255255	1
ENSBTAG00000024275	*XRCC4*	608	520	563	527	600	0.189	0.829023165	1
ENSBTAG00000024280	*pseudogene*	264	463	382	229	535	1.226	0.189028088	1
ENSBTAG00000024288	*BT.26742*	5	4	4	4	5	0.093	1	1
ENSBTAG00000024291	*BT.95168*	4	4	4	3	5	0.415	0.994759363	1
ENSBTAG00000024311	*protein_coding*	979	1'123	1'072	848	1'297	0.613	0.455428101	1
ENSBTAG00000024318	*BT.25501*	10	4	7	9	5	-0.907	0.905171387	1
ENSBTAG00000024319	*pseudogene*	1	0	0	1	0		1	1
ENSBTAG00000024326	*processed_pseudogene*	11	9	10	10	10	0.126	1	1
ENSBTAG00000024334	*processed_pseudogene*	31	41	37	27	47	0.818	0.687402498	1
ENSBTAG00000024340	*BT.87351*	466	379	421	404	438	0.117	0.899430945	1
ENSBTAG00000024361	*protein_coding*	0	1	1	0	1	Inf	0.993540919	1
ENSBTAG00000024378	*MRPS21*	2'395	1'887	2'127	2'074	2'179	0.071	0.928128598	1
ENSBTAG00000024379	*LRRC17*	10'624	14'707	13'091	9'201	16'982	0.884	0.249345713	1
ENSBTAG00000024381	*MAN1A1*	1'234	440	788	1'069	508	-1.073	0.206582704	1
ENSBTAG00000024387	*PNPLA8*	1'559	903	1'196	1'350	1'043	-0.373	0.64808005	1
ENSBTAG00000024394	*SEMA3D*	1'102	1'578	1'388	954	1'822	0.933	0.250000798	1
ENSBTAG00000024406	*BT.66886*	45	51	49	39	59	0.596	0.738768112	1
ENSBTAG00000024407	*processed_pseudogene*	8'435	8'254	8'418	7'305	9'531	0.384	0.616108357	1
ENSBTAG00000024426	*PPP1R9A*	110	48	75	95	55	-0.781	0.595381328	1
ENSBTAG00000024431	*PEX1*	1'127	702	893	976	811	-0.268	0.748955219	1
ENSBTAG00000024443	*TATDN3*	1'963	682	1'244	1'700	788	-1.110	0.175743857	1
ENSBTAG00000024449	*CENPF*	1'818	1'875	1'870	1'574	2'165	0.460	0.562768999	1
ENSBTAG00000024450	*MAP2K2*	2'899	3'540	3'299	2'511	4'088	0.703	0.367746767	1
ENSBTAG00000024453	*RD3*	1	0	0	1	0		1	1
ENSBTAG00000024470	*SH2D2A*	228	271	255	197	313	0.664	0.506652855	1
ENSBTAG00000024476	*CKS1B*	1'323	1'005	1'153	1'146	1'160	0.018	0.982490446	1
ENSBTAG00000024477	*protein_coding*	2	0	1	2	0		0.974934741	1
ENSBTAG00000024482	*MGC157043*	116	79	96	100	91	-0.139	0.925157915	1
ENSBTAG00000024485	*KLHL20*	2'396	1'350	1'817	2'075	1'559	-0.413	0.604834213	1
ENSBTAG00000024487	*processed_pseudogene*	3'267	4'182	3'829	2'829	4'829	0.771	0.321950181	1
ENSBTAG00000024490	*BT.89019*	2	6	4	2	7	2.000	0.840116992	1
ENSBTAG00000024492	*protein_coding*	40	36	38	35	42	0.263	0.905226184	1
ENSBTAG00000024493	*DHRS3*	1'365	2'426	1'992	1'182	2'801	1.245	0.120485751	1
ENSBTAG00000024497	*CTRC*	1	0	0	1	0		1	1
ENSBTAG00000024503	*FCER1G*	639	483	556	553	558	0.011	0.991409442	1
ENSBTAG00000024507	*BT.106112*	16	0	7	14	0		0.463690882	1
ENSBTAG00000024508	*CALML6*	1	0	0	1	0		1	1
ENSBTAG00000024509	*TMEM52*	1	4	3	1	5	2.415	0.884888347	1
ENSBTAG00000024510	*C1ORF222*	4	1	2	3	1	-1.585	0.952247636	1
ENSBTAG00000024520	*C16H1orf174*	863	599	720	747	692	-0.112	0.89751278	1
ENSBTAG00000024525	*HES3*	5	22	15	4	25	2.553	0.471401507	1
ENSBTAG00000024526	*BT.100543*	179	177	180	155	204	0.399	0.715644932	1
ENSBTAG00000024534	*BT.39352*	31	31	31	27	36	0.415	0.859812303	1
ENSBTAG00000024539	*BT.24841*	1'429	1'093	1'250	1'238	1'262	0.028	0.972543037	1
ENSBTAG00000024542	*PIGC*	3'216	2'463	2'815	2'785	2'844	0.030	0.969006933	1

Ensembl gene ID	geneName	counts wildtype horn bud	counts polled horn bud	baseMean	baseMean wildtype horn bud	baseMean polled horn bud	log2FoldChange	pval	padj
ENSBTAG00000024548	*pseudogene*	6	3	4	5	3	-0.585	0.976586913	1
ENSBTAG00000024549	*SNRPC*	2'037	2'033	2'056	1'764	2'348	0.412	0.60208888	1
ENSBTAG00000024552	*SHH*	44	170	117	38	196	2.365	0.065798362	1
ENSBTAG00000024555	*EFCAB2*	267	125	188	231	144	-0.680	0.529701671	1
ENSBTAG00000024560	*MGC151592*	41	20	29	36	23	-0.621	0.795344536	1
ENSBTAG00000024561	*H3F3A*	14'207	14'700	14'639	12'304	16'974	0.464	0.542667451	1
ENSBTAG00000024563	*pseudogene*	1	0	0	1	0		1	1
ENSBTAG00000024578	*pseudogene*	2'277	3'756	3'154	1'972	4'337	1.137	0.148747764	1
ENSBTAG00000024585	*ZNF33B*	62	26	42	54	30	-0.839	0.663546469	1
ENSBTAG00000024595	*HECA*	451	278	356	391	321	-0.283	0.764878742	1
ENSBTAG00000024603	*NCOR2*	5'020	11'269	8'680	4'347	13'012	1.582	0.04315035	1
ENSBTAG00000024605	*DYNLL1*	986	960	981	854	1'109	0.376	0.648411978	1
ENSBTAG00000024608	*BT.105281*	19'079	21'190	20'496	16'523	24'468	0.566	0.457371037	1
ENSBTAG00000024623	*PIGP*	196	238	222	170	275	0.695	0.500800892	1
ENSBTAG00000024632	*LRP1B*	91	21	52	79	24	-1.700	0.330447843	1
ENSBTAG00000024633	*PIK3C2B*	1'826	2'103	2'005	1'581	2'428	0.619	0.435180355	1
ENSBTAG00000024639	*protein_coding*	4	51	31	3	59	4.087	0.097938988	1
ENSBTAG00000024641	*ZBED1*	345	866	649	299	1'000	1.743	0.04746795	1
ENSBTAG00000024643	*VEPH1*	7	7	7	6	8	0.415	0.967915805	1
ENSBTAG00000024647	*BT.33303*	66	30	46	57	35	-0.722	0.695986124	1
ENSBTAG00000024648	*PGR*	1	0	0	1	0		1	1
ENSBTAG00000024657	*ZFP91*	1'050	951	1'004	909	1'098	0.272	0.741255558	1
ENSBTAG00000024662	*CXORF30*	11	2	6	10	2	-2.044	0.786625039	1
ENSBTAG00000024663	*LAD1*	938	1'396	1'212	812	1'612	0.989	0.22715904	1
ENSBTAG00000024675	*CYSLTR1*	12	10	11	10	12	0.152	0.995696634	1
ENSBTAG00000024688	*PHIP*	680	411	532	589	475	-0.311	0.725305995	1
ENSBTAG00000024701	*TIMM10*	786	852	832	681	984	0.531	0.526027201	1
ENSBTAG00000024708	*CABIN1*	2'760	4'982	4'071	2'390	5'753	1.267	0.106445169	1
ENSBTAG00000024715	*PTPRJ*	478	294	377	414	339	-0.286	0.759885687	1
ENSBTAG00000024723	*CHCHD3*	4'123	4'655	4'473	3'571	5'375	0.590	0.445967397	1
ENSBTAG00000024726	*HJURP*	613	732	688	531	845	0.671	0.432507218	1
ENSBTAG00000024751	*protein_coding*	44	53	50	38	61	0.684	0.69844292	1
ENSBTAG00000024756	*FAM116B*	2'285	2'271	2'301	1'979	2'622	0.406	0.605808084	1
ENSBTAG00000024765	*pseudogene*	1	3	2	1	3	2.000	0.933774563	1
ENSBTAG00000024772	*TRIP6*	2'269	3'100	2'772	1'965	3'580	0.865	0.271270096	1
ENSBTAG00000024781	*MRPL17*	2'558	1'489	1'967	2'215	1'719	-0.366	0.645434722	1
ENSBTAG00000024787	*BT.72777*	1'815	884	1'296	1'572	1'021	-0.623	0.443436831	1
ENSBTAG00000024801	*RANBP17*	302	123	202	262	142	-0.881	0.406885158	1
ENSBTAG00000024803	*ENDOD1*	2'596	2'230	2'412	2'248	2'575	0.196	0.802940452	1
ENSBTAG00000024814	*B4GALNT4*	553	694	640	479	801	0.743	0.38907345	1
ENSBTAG00000024815	*ANKRD28*	1'759	1'286	1'504	1'523	1'485	-0.037	0.96499432	1
ENSBTAG00000024822	*SETX*	1'837	1'503	1'663	1'591	1'736	0.126	0.874987817	1
ENSBTAG00000024826	*TECRL*	6	23	16	5	27	2.354	0.485077849	1
ENSBTAG00000024839	*protein_coding*	708	1'169	981	613	1'350	1.138	0.171932056	1
ENSBTAG00000024849	*BT.72972*	3	5	4	3	6	1.152	0.920866718	1
ENSBTAG00000024851	*TRIM14*	14	9	11	12	10	-0.222	0.982048045	1
ENSBTAG00000024852	*CATHL2*	4	0	2	3	0		0.89545886	1
ENSBTAG00000024869	*CX3CL1*	427	458	449	370	529	0.516	0.567006931	1
ENSBTAG00000024874	*BT.56334*	2	4	3	2	5	1.415	0.927250049	1
ENSBTAG00000024878	*ANKRD31*	137	34	79	119	39	-1.596	0.272207665	1
ENSBTAG00000024884	*C16ORF59*	857	793	829	742	916	0.303	0.717612686	1
ENSBTAG00000024888	*PDE5A*	398	248	316	345	286	-0.267	0.782731707	1
ENSBTAG00000024889	*HSBP1*	7'229	5'526	6'321	6'260	6'381	0.027	0.971014751	1
ENSBTAG00000024901	*protein_coding*	0	1	1	0	1	Inf	0.993540919	1
ENSBTAG00000024903	*KCNJ16*	4	0	2	3	0		0.89545886	1
ENSBTAG00000024904	*KIAA1683*	97	199	157	84	230	1.452	0.203976004	1
ENSBTAG00000024909	*H3F3A*	18'927	12'211	15'246	16'391	14'100	-0.217	0.776322955	1
ENSBTAG00000024910	*BT.86629*	0	1	1	0	1	Inf	0.993540919	1
ENSBTAG00000024911	*LIPN*	7	1	4	6	1	-2.392	0.851750348	1
ENSBTAG00000024914	*pseudogene*	3	4	4	3	5	0.830	0.962490925	1
ENSBTAG00000024915	*BT.62450*	0	12	7	0	14	Inf	0.42678658	1
ENSBTAG00000024918	*BIRC3*	1'800	492	1'063	1'559	568	-1.456	0.081157113	1
ENSBTAG00000024926	*TMEM235*	0	1	1	0	1	Inf	0.993540919	1
ENSBTAG00000024928	*TNFRSF1B*	283	297	294	245	343	0.485	0.617785502	1
ENSBTAG00000024932	*FSCN2*	9	8	9	8	9	0.245	0.986825286	1
ENSBTAG00000024934	*protein_coding*	1	1	1	1	1	0.415	1	1
ENSBTAG00000024942	*RPRML*	25	2	12	22	2	-3.229	0.459170232	1
ENSBTAG00000024943	*protein_coding*	12	7	9	10	8	-0.363	0.964206651	1
ENSBTAG00000024947	*TCAM1*	60	15	35	52	17	-1.585	0.454660426	1
ENSBTAG00000024950	*ACE*	163	111	135	141	128	-0.139	0.913178377	1
ENSBTAG00000024958	*FAM214A*	3'244	998	1'981	2'809	1'152	-1.286	0.109368878	1
ENSBTAG00000024960	*BT.61154*	39	9	22	34	10	-1.700	0.535008866	1

Ensembl gene ID	geneName	counts wildtype horn bud	counts polled horn bud	baseMean	baseMean wildtype horn bud	baseMean polled horn bud	log2FoldChange	pval	padj
ENSBTAG00000024964	*pseudogene*	2'806	5'855	4'595	2'430	6'761	1.476	0.060671685	1
ENSBTAG00000024974	*HEXIM2*	171	120	143	148	139	-0.096	0.941192948	1
ENSBTAG00000024975	*BT.71183*	3	2	2	3	2	-0.170	1	1
ENSBTAG00000024979	*HSPB9*	1	3	2	1	3	2.000	0.933774563	1
ENSBTAG00000024980	*VIT*	711	282	471	616	326	-0.919	0.307408345	1
ENSBTAG00000024983	*FBXO47*	15	12	13	13	14	0.093	1	1
ENSBTAG00000024984	*BT.91076*	169	65	111	146	75	-0.963	0.448386516	1
ENSBTAG00000024991	*MKRN2-AS1*	0	3	2	0	3	Inf	0.878646179	1
ENSBTAG00000024995	*BT.62407*	1	0	0	1	0		1	1
ENSBTAG00000024999	*UTF1*	0	7	4	0	8	Inf	0.646259203	1
ENSBTAG00000025001	*BT.36663*	2'314	2'308	2'335	2'004	2'665	0.411	0.601090157	1
ENSBTAG00000025003	*INTS10*	2'725	2'422	2'578	2'360	2'797	0.245	0.754365501	1
ENSBTAG00000025005	*METTL7A*	103	52	75	89	60	-0.571	0.701150513	1
ENSBTAG00000025009	*HOXB9*	0	1	1	0	1	Inf	0.993540919	1
ENSBTAG00000025021	*protein_coding*	185	34	100	160	39	-2.029	0.132481368	1
ENSBTAG00000025023	*ZNF8*	120	133	129	104	154	0.563	0.640127485	1
ENSBTAG00000025028	*MZT1*	503	338	413	436	390	-0.158	0.865092878	1
ENSBTAG00000025029	*MAN2A1*	3'100	2'167	2'593	2'685	2'502	-0.102	0.898056708	1
ENSBTAG00000025035	*PRLR*	11	2	6	10	2	-2.044	0.786625039	1
ENSBTAG00000025042	*pseudogene*	5	2	3	4	2	-0.907	0.965043111	1
ENSBTAG00000025046	*ALKBH5*	1'891	2'233	2'108	1'638	2'578	0.655	0.408133658	1
ENSBTAG00000025062	*MAGEE2*	738	610	672	639	704	0.140	0.870548576	1
ENSBTAG00000025071	*ODZ2*	964	1'086	1'044	835	1'254	0.587	0.475567049	1
ENSBTAG00000025078	*FAM211A*	407	487	457	352	562	0.674	0.453911303	1
ENSBTAG00000025088	*RCVRN*	10	10	10	9	12	0.415	0.946590905	1
ENSBTAG00000025099	*RNF145*	5'446	4'023	4'681	4'716	4'645	-0.022	0.978378152	1
ENSBTAG00000025101	*BT.18330*	0	1	1	0	1	Inf	0.993540919	1
ENSBTAG00000025108	*pseudogene*	0	1	1	0	1	Inf	0.993540919	1
ENSBTAG00000025121	*BT.68438*	663	435	538	574	502	-0.193	0.828449344	1
ENSBTAG00000025124	*ZNF300*	283	101	181	245	117	-1.071	0.327780584	1
ENSBTAG00000025126	*protein_coding*	1'784	1'006	1'353	1'545	1'162	-0.411	0.611524069	1
ENSBTAG00000025129	*MNT*	349	530	457	302	612	1.018	0.260120327	1
ENSBTAG00000025130	*HIC1*	725	1'114	957	628	1'286	1.035	0.214415622	1
ENSBTAG00000025136	*MYOZ3*	59	150	112	51	173	1.761	0.168233906	1
ENSBTAG00000025140	*DOC2B*	151	184	172	131	212	0.700	0.525825453	1
ENSBTAG00000025146	*BT.101703*	192	150	170	166	173	0.059	0.96247094	1
ENSBTAG00000025148	*BT.53859*	948	575	742	821	664	-0.306	0.719058999	1
ENSBTAG00000025149	*RPAIN*	477	214	330	413	247	-0.741	0.436592865	1
ENSBTAG00000025161	*BT.22590*	1'209	2'117	1'746	1'047	2'445	1.223	0.129060337	1
ENSBTAG00000025165	*pseudogene*	44	106	80	38	122	1.684	0.242004344	1
ENSBTAG00000025167	*ABHD15*	74	93	86	64	107	0.745	0.591780202	1
ENSBTAG00000025181	*BT.61572*	577	605	599	500	699	0.483	0.577292439	1
ENSBTAG00000025182	*FBXL21*	176	79	122	152	91	-0.741	0.547388091	1
ENSBTAG00000025183	*ANKRD40*	4	1	2	3	1	-1.585	0.952247636	1
ENSBTAG00000025191	*SKP1*	2'939	1'694	2'251	2'545	1'956	-0.380	0.630750387	1
ENSBTAG00000025200	*ACCN1*	71	7	35	61	8	-2.927	0.186874684	1
ENSBTAG00000025210	*COL4A2*	20'416	32'280	27'477	17'681	37'274	1.076	0.160965817	1
ENSBTAG00000025211	*BT.20117*	872	1'017	965	755	1'174	0.637	0.441833946	1
ENSBTAG00000025212	*BT.28199*	1'951	904	1'367	1'690	1'044	-0.695	0.391310693	1
ENSBTAG00000025219	*protein_coding*	12	3	7	10	3	-1.585	0.809704871	1
ENSBTAG00000025221	*TEX30*	617	378	485	534	436	-0.292	0.745258258	1
ENSBTAG00000025226	*IGIP*	490	127	285	424	147	-1.533	0.122653487	1
ENSBTAG00000025233	*C19ORF26*	139	104	120	120	120	-0.003	1	1
ENSBTAG00000025242	*C19H17orf78*	3	0	1	3	0		0.936647693	1
ENSBTAG00000025250	*CCL3*	52	24	36	45	28	-0.700	0.737915867	1
ENSBTAG00000025257	*CCL4*	20	3	10	17	3	-2.322	0.618209332	1
ENSBTAG00000025258	*protein_coding*	11	1	5	10	1	-3.044	0.722857941	1
ENSBTAG00000025260	*EXPI*	2'441	1'557	1'956	2'114	1'798	-0.234	0.769127412	1
ENSBTAG00000025263	*FAM160A1*	559	452	503	484	522	0.109	0.903919528	1
ENSBTAG00000025274	*TUBB4B*	6'966	10'726	9'209	6'033	12'385	1.038	0.178814724	1
ENSBTAG00000025277	*BT.51825*	2'398	2'624	2'553	2'077	3'030	0.545	0.487639166	1
ENSBTAG00000025280	*SERPINB6*	3'218	2'732	2'971	2'787	3'155	0.179	0.818427727	1
ENSBTAG00000025283	*processed_pseudogene*	363	640	527	314	739	1.233	0.165893219	1
ENSBTAG00000025297	*MMGT1*	80	51	64	69	59	-0.234	0.890059166	1
ENSBTAG00000025308	*BT.106520*	46	31	38	40	36	-0.154	0.953061737	1
ENSBTAG00000025310	*protein_coding*	12	49	33	10	57	2.445	0.263728469	1
ENSBTAG00000025311	*GMFB*	4'082	2'418	3'164	3'535	2'792	-0.340	0.663141218	1
ENSBTAG00000025313	*DYNLL2*	6'133	7'233	6'832	5'311	8'352	0.653	0.396179812	1
ENSBTAG00000025320	*AKAP1*	1'495	1'627	1'587	1'295	1'879	0.537	0.502499019	1
ENSBTAG00000025324	*NRXN3*	43	1	19	37	1	-5.011	0.165386276	1
ENSBTAG00000025329	*IRF2BPL*	1'123	1'013	1'071	973	1'170	0.266	0.745384191	1
ENSBTAG00000025335	*pseudogene*	496	1'226	923	430	1'416	1.721	0.042868725	1

Ensembl gene ID	geneName	counts wildtype horn bud	counts polled horn bud	baseMean	baseMean wildtype horn bud	baseMean polled horn bud	log2FoldChange	pval	padj
ENSBTAG00000025337	*MYH13*	1	31	18	1	36	5.369	0.147221188	1
ENSBTAG00000025340	*CDHR2*	26	45	37	23	52	1.206	0.549526597	1
ENSBTAG00000025345	*ARL10*	435	640	558	377	739	0.972	0.268893756	1
ENSBTAG00000025358	*BT.26946*	4'860	2'663	3'642	4'209	3'075	-0.453	0.56084263	1
ENSBTAG00000025372	*C10H14ORF169*	795	766	786	688	885	0.361	0.667765468	1
ENSBTAG00000025379	*pseudogene*	262	316	296	227	365	0.685	0.479788481	1
ENSBTAG00000025383	*processed_pseudogene*	715	435	561	619	502	-0.302	0.731584519	1
ENSBTAG00000025385	*RPL12*	23'018	26'440	25'232	19'934	30'530	0.615	0.419631051	1
ENSBTAG00000025391	*H4*	21	6	13	18	7	-1.392	0.729267326	1
ENSBTAG00000025398	*protein_coding*	1	0	0	1	0		1	1
ENSBTAG00000025400	*PARP4*	1'410	1'232	1'322	1'221	1'423	0.220	0.785156103	1
ENSBTAG00000025401	*DOK3*	36	20	27	31	23	-0.433	0.86863557	1
ENSBTAG00000025402	*IL17D*	530	415	469	459	479	0.062	0.946386695	1
ENSBTAG00000025403	*TTLL5*	790	693	742	684	800	0.226	0.789818904	1
ENSBTAG00000025405	*BATF*	20	27	24	17	31	0.848	0.743425918	1
ENSBTAG00000025410	*EGFL8*	1'274	362	761	1'103	418	-1.400	0.102677317	1
ENSBTAG00000025413	*RNF5*	1'526	1'578	1'572	1'322	1'822	0.463	0.562859743	1
ENSBTAG00000025424	*NOTCH4*	514	433	473	445	500	0.168	0.852450158	1
ENSBTAG00000025425	*BT.56132*	3'378	2'000	2'617	2'925	2'309	-0.341	0.664319423	1
ENSBTAG00000025426	*NSD1*	3'349	3'074	3'225	2'900	3'550	0.291	0.707923808	1
ENSBTAG00000025428	*protein_coding*	0	1	1	0	1	Inf	0.993540919	1
ENSBTAG00000025434	*ZFP36L1*	22'214	9'259	14'965	19'238	10'691	-0.848	0.269311789	1
ENSBTAG00000025441	*HSPA1A*	19'885	19'777	20'029	17'221	22'837	0.407	0.592526855	1
ENSBTAG00000025442	*HSPA1L*	80	50	64	69	58	-0.263	0.875777074	1
ENSBTAG00000025443	*FNIP1*	1'603	888	1'207	1'388	1'025	-0.437	0.592196184	1
ENSBTAG00000025445	*processed_pseudogene*	1'042	839	936	902	969	0.102	0.902125805	1
ENSBTAG00000025448	*BT.40226*	41	17	28	36	20	-0.855	0.724831058	1
ENSBTAG00000025449	*LY6G5C*	0	1	1	0	1	Inf	0.993540919	1
ENSBTAG00000025450	*SYNE2*	7'492	5'514	6'428	6'488	6'367	-0.027	0.972641799	1
ENSBTAG00000025452	*BT.27293*	1'209	1'639	1'470	1'047	1'893	0.854	0.290185098	1
ENSBTAG00000025458	*DPF3*	11	2	6	10	2	-2.044	0.786625039	1
ENSBTAG00000025462	*GADD45B*	895	489	670	775	565	-0.457	0.594794344	1
ENSBTAG00000025471	*TNF*	29	12	19	25	14	-0.858	0.777103274	1
ENSBTAG00000025477	*BT.70921*	928	550	719	804	635	-0.340	0.690734626	1
ENSBTAG00000025485	*protein_coding*	3	4	4	3	5	0.830	0.962490925	1
ENSBTAG00000025494	*PAQR8*	681	586	633	590	677	0.198	0.818503621	1
ENSBTAG00000025496	*SORD*	671	247	433	581	285	-1.027	0.260750057	1
ENSBTAG00000025502	*LECT1*	148	37	85	128	43	-1.585	0.260650593	1
ENSBTAG00000025516	*BT.38185*	1'335	1'833	1'636	1'156	2'117	0.872	0.277369462	1
ENSBTAG00000025522	*UNC13C*	114	50	78	99	58	-0.774	0.593250657	1
ENSBTAG00000025526	*MDC1*	4'370	4'569	4'530	3'785	5'276	0.479	0.535303821	1
ENSBTAG00000025535	*BT.93015*	33	4	17	29	5	-2.629	0.435665511	1
ENSBTAG00000025540	*ALKBH7*	414	478	455	359	552	0.622	0.489300316	1
ENSBTAG00000025550	*PRR22*	118	78	96	102	90	-0.182	0.898805405	1
ENSBTAG00000025554	*LRRC73*	193	64	121	167	74	-1.177	0.341377147	1
ENSBTAG00000025564	*RPL36A*	10'413	6'456	8'236	9'018	7'455	-0.275	0.720703687	1
ENSBTAG00000025571	*BT.21792*	934	903	926	809	1'043	0.366	0.658806728	1
ENSBTAG00000025589	*CDKAL1*	1'340	1'021	1'170	1'160	1'179	0.023	0.978169604	1
ENSBTAG00000025593	*CCDC32*	1'545	995	1'243	1'338	1'149	-0.220	0.787872272	1
ENSBTAG00000025595	*BT.86815*	7'737	5'130	6'312	6'700	5'924	-0.178	0.817843764	1
ENSBTAG00000025597	*GRIN3B*	99	96	98	86	111	0.371	0.781878332	1
ENSBTAG00000025604	*pseudogene*	3	0	1	3	0		0.936647693	1
ENSBTAG00000025606	*YIPF2*	3'373	4'077	3'814	2'921	4'708	0.689	0.37607984	1
ENSBTAG00000025612	*URM1*	1'594	2'447	2'103	1'380	2'826	1.033	0.194443507	1
ENSBTAG00000025617	*RPS4Y1*	2'219	2'288	2'282	1'922	2'642	0.459	0.559878977	1
ENSBTAG00000025621	*HNRNPA1L2*	3'254	4'518	4'017	2'818	5'217	0.889	0.254231887	1
ENSBTAG00000025622	*DEF6*	357	420	397	309	485	0.650	0.480011157	1
ENSBTAG00000025632	*NOP10*	1'795	1'213	1'478	1'555	1'401	-0.150	0.852861101	1
ENSBTAG00000025634	*FMN1*	25	11	17	22	13	-0.769	0.818249294	1
ENSBTAG00000025642	*RYR3*	1'843	528	1'103	1'596	610	-1.388	0.094944208	1
ENSBTAG00000025644	*CALM*	14'614	9'983	12'092	12'656	11'527	-0.135	0.860578282	1
ENSBTAG00000025652	*BEST2*	1	6	4	1	7	3.000	0.788284548	1
ENSBTAG00000025659	*ZNF618*	182	300	252	158	346	1.136	0.25981855	1
ENSBTAG00000025664	*BT.58446*	15	11	13	13	13	-0.032	1	1
ENSBTAG00000025666	*RPS29*	35'554	10'576	21'501	30'791	12'212	-1.334	0.084218657	1
ENSBTAG00000025667	*BT.38344*	133	369	271	115	426	1.887	0.062167216	1
ENSBTAG00000025669	*B3GALT4*	583	667	638	505	770	0.609	0.479513091	1
ENSBTAG00000025718	*HMGCLL1*	59	0	26	51	0		0.041133735	1
ENSBTAG00000025752	*LEPREL4*	4'758	6'431	5'773	4'121	7'426	0.850	0.272096934	1
ENSBTAG00000025755	*C14ORF164*	23	21	22	20	24	0.284	0.93047699	1
ENSBTAG00000025756	*FBXW12*	2'077	1'099	1'534	1'799	1'269	-0.503	0.531484149	1
ENSBTAG00000025760	*BAAT*	52	25	37	45	29	-0.642	0.758368091	1

Ensembl gene ID	geneName	counts wildtype horn bud	counts polled horn bud	baseMean	baseMean wildtype horn bud	baseMean polled horn bud	log2FoldChange	pval	padj
ENSBTAG00000025762	*CNP*	4'128	4'908	4'621	3'575	5'667	0.665	0.390760625	1
ENSBTAG00000025764	*pseudogene*	128	313	236	111	361	1.705	0.099969846	1
ENSBTAG00000025767	*TMEM221*	24	13	18	21	15	-0.469	0.89373307	1
ENSBTAG00000025775	*INSL3*	1	0	0	1	0		1	1
ENSBTAG00000025778	*FAM158A*	233	243	241	202	281	0.476	0.639013271	1
ENSBTAG00000025782	*BT.44261*	56	67	63	48	77	0.674	0.670052381	1
ENSBTAG00000025788	*MPV17L2*	191	143	165	165	165	-0.003	1	1
ENSBTAG00000025792	*ARID3C*	13	14	14	11	16	0.522	0.900598582	1
ENSBTAG00000025803	*BT.34437*	864	1'365	1'162	748	1'576	1.075	0.191134378	1
ENSBTAG00000025809	*ABHD8*	841	1'062	977	728	1'226	0.752	0.364235126	1
ENSBTAG00000025812	*pseudogene*	48	19	32	42	22	-0.922	0.679327723	1
ENSBTAG00000025814	*TMEM202*	10	7	8	9	8	-0.100	1	1
ENSBTAG00000025817	*MEF2BNB*	1'316	863	1'068	1'140	997	-0.194	0.814831767	1
ENSBTAG00000025822	*CALML4*	301	128	204	261	148	-0.819	0.439305314	1
ENSBTAG00000025826	*SLC24A1*	34	12	22	29	14	-1.087	0.696466331	1
ENSBTAG00000025830	*R3HCC1*	997	1'154	1'098	863	1'333	0.626	0.445203331	1
ENSBTAG00000025848	*RBM3*	6'947	2'490	4'446	6'016	2'875	-1.065	0.172425915	1
ENSBTAG00000025853	*HOMER1*	777	459	601	673	530	-0.344	0.69266376	1
ENSBTAG00000025856	*JMY*	150	73	107	130	84	-0.624	0.628470288	1
ENSBTAG00000025859	*BT.21210*	871	659	758	754	761	0.013	0.989151124	1
ENSBTAG00000025868	*TLN1*	15'602	30'300	24'250	13'512	34'987	1.373	0.075740657	1
ENSBTAG00000025893	*BT.41071*	53	0	23	46	0		0.056788891	1
ENSBTAG00000025898	*TBC1D8*	677	478	569	586	552	-0.087	0.923161055	1
ENSBTAG00000025920	*BT.59968*	2	0	1	2	0		0.974934741	1
ENSBTAG00000025928	*protein_coding*	1	2	2	1	2	1.415	0.981979269	1
ENSBTAG00000025929	*MTAP*	399	468	443	346	540	0.645	0.47542562	1
ENSBTAG00000025931	*NEU3*	237	263	254	205	304	0.565	0.572340653	1
ENSBTAG00000025942	*BT.43717*	5'984	2'418	3'987	5'182	2'792	-0.892	0.252811631	1
ENSBTAG00000025951	*protein_coding*	1	1	1	1	1	0.415	1	1
ENSBTAG00000025953	*pseudogene*	1	21	13	1	24	4.807	0.292435339	1
ENSBTAG00000025964	*MAST4*	806	409	585	698	472	-0.564	0.518348618	1
ENSBTAG00000025968	*pseudogene*	1'555	1'825	1'727	1'347	2'107	0.646	0.418506785	1
ENSBTAG00000026003	*GUCY2F*	1	0	0	1	0		1	1
ENSBTAG00000026008	*METTL5*	795	571	674	688	659	-0.062	0.94433294	1
ENSBTAG00000026011	*processed_pseudogene*	35	79	61	30	91	1.590	0.322923726	1
ENSBTAG00000026025	*protein_coding*	24	22	23	21	25	0.290	0.925977597	1
ENSBTAG00000026032	*ZYG11A*	7	5	6	6	6	-0.070	1	1
ENSBTAG00000026063	*pseudogene*	34	143	97	29	165	2.487	0.070732255	1
ENSBTAG00000026067	*SH2D1A*	12	6	9	10	7	-0.585	0.929512138	1
ENSBTAG00000026070	*protein_coding*	2	0	1	2	0		0.974934741	1
ENSBTAG00000026080	*LAIR1*	67	75	72	58	87	0.578	0.699098277	1
ENSBTAG00000026088	*LYZ2*	26	3	13	23	3	-2.700	0.499863335	1
ENSBTAG00000026097	*LIMD1*	426	528	489	369	610	0.725	0.416346262	1
ENSBTAG00000026102	*PAG9*	1	0	0	1	0		1	1
ENSBTAG00000026114	*UBE2F*	1'032	829	925	894	957	0.099	0.905470397	1
ENSBTAG00000026119	*PTI*	307	149	219	266	172	-0.628	0.546248116	1
ENSBTAG00000026122	*ZNF804A*	90	15	48	78	17	-2.170	0.237773185	1
ENSBTAG00000026123	*protein_coding*	0	1	1	0	1	Inf	0.993540919	1
ENSBTAG00000026139	*ARMCX1*	3'178	906	1'899	2'752	1'046	-1.395	0.083550719	1
ENSBTAG00000026141	*protein_coding*	347	218	276	301	252	-0.256	0.798022921	1
ENSBTAG00000026156	*VGLL3*	359	191	266	311	221	-0.495	0.619147482	1
ENSBTAG00000026172	*FAM84B*	710	322	493	615	372	-0.726	0.416331498	1
ENSBTAG00000026181	*UGT1A1*	60	11	32	52	13	-2.032	0.360366107	1
ENSBTAG00000026191	*BT.29993*	540	585	572	468	675	0.531	0.54314423	1
ENSBTAG00000026192	*C25H7ORF50*	1'245	1'318	1'300	1'078	1'522	0.497	0.539214659	1
ENSBTAG00000026194	*AMZ1*	106	13	53	92	15	-2.612	0.141071946	1
ENSBTAG00000026199	*BT.97896*	71'663	89'861	82'912	62'062	103'763	0.742	0.330170389	1
ENSBTAG00000026232	*TP53INP1*	274	297	290	237	343	0.531	0.585294225	1
ENSBTAG00000026233	*VPS13B*	38	32	35	33	37	0.167	0.948891151	1
ENSBTAG00000026234	*STK3*	2'745	2'744	2'773	2'377	3'168	0.415	0.596118482	1
ENSBTAG00000026236	*AZGP1*	201	47	114	174	54	-1.681	0.186706637	1
ENSBTAG00000026242	*BT.35965*	327	108	204	283	125	-1.183	0.265731147	1
ENSBTAG00000026243	*PPP1R35*	644	560	602	558	647	0.213	0.806018513	1
ENSBTAG00000026246	*MOSPD3*	1'163	1'232	1'215	1'007	1'423	0.498	0.540209329	1
ENSBTAG00000026247	*protein_coding*	4	0	2	3	0		0.89545886	1
ENSBTAG00000026248	*POP7*	1'666	2'059	1'910	1'443	2'378	0.721	0.36508721	1
ENSBTAG00000026249	*protein_coding*	100	132	120	87	152	0.816	0.507987395	1
ENSBTAG00000026254	*MED30*	834	583	698	722	673	-0.102	0.907461818	1
ENSBTAG00000026260	*KCNE2*	3	2	2	3	2	-0.170	1	1
ENSBTAG00000026263	*ZNF45*	1'924	996	1'408	1'666	1'150	-0.535	0.508125895	1
ENSBTAG00000026266	*MYL12B*	2'433	1'674	2'020	2'107	1'933	-0.124	0.876271639	1
ENSBTAG00000026273	*MYL10*	0	3	2	0	3	Inf	0.878646179	1

Ensembl gene ID	geneName	counts wildtype horn bud	counts polled horn bud	baseMean	baseMean wildtype horn bud	baseMean polled horn bud	log2FoldChange	pval	padj
ENSBTAG00000026275	*BT.32408*	58	80	71	50	92	0.879	0.556081548	1
ENSBTAG00000026278	*CLDN4*	374	276	321	324	319	-0.023	0.984982211	1
ENSBTAG00000026283	*ASPH*	3'466	2'807	3'121	3'002	3'241	0.111	0.886633814	1
ENSBTAG00000026286	*BT.22655*	2'009	1'643	1'819	1'740	1'897	0.125	0.87503774	1
ENSBTAG00000026290	*BT.88926*	2'702	1'516	2'045	2'340	1'751	-0.419	0.597599593	1
ENSBTAG00000026306	*protein_coding*	144	151	150	125	174	0.484	0.674658044	1
ENSBTAG00000026307	*ZNF629*	289	518	424	250	598	1.257	0.170982462	1
ENSBTAG00000026309	*BT.88023*	708	1'075	927	613	1'241	1.018	0.223171032	1
ENSBTAG00000026319	*BT.25685*	46	1	20	40	1	-5.109	0.143743703	1
ENSBTAG00000026320	*VPS28*	3'861	3'174	3'504	3'344	3'665	0.132	0.864361183	1
ENSBTAG00000026326	*SPN*	137	61	95	119	70	-0.752	0.576324706	1
ENSBTAG00000026327	*RPL8*	30'637	52'457	43'552	26'532	60'572	1.191	0.121204938	1
ENSBTAG00000026340	*BT.44756*	79	45	60	68	52	-0.397	0.812033994	1
ENSBTAG00000026344	*MAFA*	2	5	4	2	6	1.737	0.883335552	1
ENSBTAG00000026350	*SPATC1*	24	1	11	21	1	-4.170	0.402732934	1
ENSBTAG00000026356	*DGAT1*	1'848	1'380	1'597	1'600	1'593	-0.006	0.995372228	1
ENSBTAG00000026365	*protein_coding*	3	0	1	3	0		0.936647693	1
ENSBTAG00000026369	*BT.53943*	4'717	1'178	2'723	4'085	1'360	-1.586	0.047218518	1
ENSBTAG00000026371	*BT.53906*	170	303	249	147	350	1.249	0.217835481	1
ENSBTAG00000026375	*BT.17992*	1'042	1'222	1'157	902	1'411	0.645	0.429982418	1
ENSBTAG00000026376	*SNN*	589	396	484	510	457	-0.158	0.862191854	1
ENSBTAG00000026381	*TMEM114*	27	38	34	23	44	0.908	0.670889335	1
ENSBTAG00000026383	*BT.88635*	0	2	1	0	2	Inf	0.939077559	1
ENSBTAG00000026384	*ZAR1L*	26	19	22	23	22	-0.037	1	1
ENSBTAG00000026385	*protein_coding*	3	0	1	3	0		0.936647693	1
ENSBTAG00000026394	*MGC142811*	483	591	550	418	682	0.706	0.421059316	1
ENSBTAG00000026400	*pseudogene*	37	70	56	32	81	1.335	0.419488985	1
ENSBTAG00000026403	*CREBBP*	7'153	4'885	5'918	6'195	5'641	-0.135	0.861349724	1
ENSBTAG00000026408	*ZNF213*	395	524	474	342	605	0.823	0.358874182	1
ENSBTAG00000026415	*BT.49027*	1'727	2'114	1'968	1'496	2'441	0.707	0.37367332	1
ENSBTAG00000026417	*HBA*	1'116	1'290	1'228	966	1'490	0.624	0.443124355	1
ENSBTAG00000026418	*HBA*	1'741	2'005	1'911	1'508	2'315	0.619	0.436169977	1
ENSBTAG00000026422	*MPG*	995	907	955	862	1'047	0.281	0.733815248	1
ENSBTAG00000026428	*ATP6V0C*	5'103	4'334	4'712	4'419	5'004	0.179	0.815811622	1
ENSBTAG00000026429	*DECR2*	770	679	725	667	784	0.234	0.783453085	1
ENSBTAG00000026467	*MGC128071*	44	31	37	38	36	-0.090	0.980378975	1
ENSBTAG00000026481	*RGS8*	15	34	26	13	39	1.596	0.514464627	1
ENSBTAG00000026489	*TNFRSF13C*	0	1	1	0	1	Inf	0.993540919	1
ENSBTAG00000026495	*BT.29582*	168	172	172	145	199	0.449	0.684899006	1
ENSBTAG00000026497	*A4GALT*	173	346	275	150	400	1.415	0.1552752	1
ENSBTAG00000026501	*CYP2D14*	627	835	754	543	964	0.828	0.328955769	1
ENSBTAG00000026502	*protein_coding*	158	155	158	137	179	0.387	0.73335208	1
ENSBTAG00000026519	*DEADC1*	770	444	590	667	513	-0.379	0.663928186	1
ENSBTAG00000026522	*protein_coding*	24	1	11	21	1	-4.170	0.402732934	1
ENSBTAG00000026523	*BT.37603*	4'330	1'547	2'768	3'750	1'786	-1.070	0.175641613	1
ENSBTAG00000026527	*AHRR*	177	237	213	153	274	0.836	0.422893928	1
ENSBTAG00000026531	*BT.64205*	1	0	0	1	0		1	1
ENSBTAG00000026578	*protein_coding*	25	6	14	22	7	-1.644	0.652134139	1
ENSBTAG00000026580	*BT.101029*	19	2	9	16	2	-2.833	0.582174212	1
ENSBTAG00000026585	*TCEB3*	2'098	1'341	1'683	1'817	1'548	-0.231	0.773631483	1
ENSBTAG00000026586	*PPP1R14C*	1'638	1'420	1'529	1'419	1'640	0.209	0.794226445	1
ENSBTAG00000026604	*LYRM2*	834	399	591	722	461	-0.649	0.456814319	1
ENSBTAG00000026610	*C12ORF69*	19	8	13	16	9	-0.833	0.840803998	1
ENSBTAG00000026611	*protein_coding*	14	2	7	12	2	-2.392	0.704665469	1
ENSBTAG00000026613	*MSTO1*	866	1'197	1'066	750	1'382	0.882	0.284710758	1
ENSBTAG00000026624	*LYRM5*	791	415	582	685	479	-0.516	0.554959431	1
ENSBTAG00000026626	*ANHX*	7	4	5	6	5	-0.392	0.98714753	1
ENSBTAG00000026637	*BT.103816*	60	28	42	52	32	-0.684	0.723678078	1
ENSBTAG00000026638	*CATSPER4*	20	16	18	17	18	0.093	0.996214168	1
ENSBTAG00000026645	*protein_coding*	4	6	5	3	7	1.000	0.91481797	1
ENSBTAG00000026650	*protein_coding*	1'236	475	809	1'070	548	-0.965	0.254054026	1
ENSBTAG00000026657	*IFT27*	489	392	438	423	453	0.096	0.917097377	1
ENSBTAG00000026660	*TAF12*	639	461	543	553	532	-0.056	0.952061904	1
ENSBTAG00000026672	*BT.56246*	2	0	1	2	0		0.974934741	1
ENSBTAG00000026673	*KPNA5*	368	173	259	319	200	-0.674	0.500609348	1
ENSBTAG00000026676	*BT.105251*	2'887	4'096	3'615	2'500	4'730	0.920	0.239313024	1
ENSBTAG00000026684	*SYNC*	825	977	921	714	1'128	0.659	0.42804356	1
ENSBTAG00000026696	*DCDC2B*	41	28	34	36	32	-0.135	0.964886952	1
ENSBTAG00000026704	*C6ORF173*	246	232	240	213	268	0.331	0.745550309	1
ENSBTAG00000026710	*C2ORF72*	19	0	8	16	0		0.389538827	1
ENSBTAG00000026716	*IBTK*	3'287	1'470	2'272	2'847	1'697	-0.746	0.346004139	1
ENSBTAG00000026732	*PLA2G1B*	1	2	2	1	2	1.415	0.981979269	1

Ensembl gene ID	geneName	counts wildtype horn bud	counts polled horn bud	baseMean	baseMean wildtype horn bud	baseMean polled horn bud	log2FoldChange	pval	padj
ENSBTAG00000026748	*GULO*	124	34	73	107	39	-1.452	0.330565123	1
ENSBTAG00000026751	*CCDC108*	15	0	6	13	0		0.491308095	1
ENSBTAG00000026753	*BT.66847*	4	4	4	3	5	0.415	0.994759363	1
ENSBTAG00000026754	*BT.87427*	668	845	777	579	976	0.754	0.372243258	1
ENSBTAG00000026758	*protein_coding*	126	251	199	109	290	1.409	0.188110071	1
ENSBTAG00000026768	*TH*	758	1'119	974	656	1'292	0.977	0.239966157	1
ENSBTAG00000026769	*ASCL2*	65	83	76	56	96	0.768	0.59796416	1
ENSBTAG00000026772	*ZNF142*	1'606	2'756	2'287	1'391	3'182	1.194	0.133790246	1
ENSBTAG00000026779	*LYS*	56	19	35	48	22	-1.144	0.585245408	1
ENSBTAG00000026788	*SHOX*	1	3	2	1	3	2.000	0.933774563	1
ENSBTAG00000026792	*BT.141*	5	1	3	4	1	-1.907	0.9188325	1
ENSBTAG00000026796	*BT.86891*	2	5	4	2	6	1.737	0.883335552	1
ENSBTAG00000026809	*pseudogene*	12'509	9'808	11'079	10'833	11'325	0.064	0.932538659	1
ENSBTAG00000026812	*BT.42973*	37	3	18	32	3	-3.209	0.334323923	1
ENSBTAG00000026813	*AQP5*	379	71	205	328	82	-2.001	0.065012397	1
ENSBTAG00000026819	*HDAC7*	6'290	8'279	7'504	5'447	9'560	0.811	0.292308141	1
ENSBTAG00000026825	*TMEM37*	175	121	146	152	140	-0.117	0.925862322	1
ENSBTAG00000026829	*TMEM185B*	1'137	831	972	985	960	-0.037	0.966087623	1
ENSBTAG00000026836	*ABI3BP*	2'394	1'833	2'095	2'073	2'117	0.030	0.969858983	1
ENSBTAG00000026842	*ZRANB3*	913	597	740	791	689	-0.198	0.817131338	1
ENSBTAG00000026848	*processed_pseudogene*	3'730	2'954	3'321	3'230	3'411	0.079	0.919290517	1
ENSBTAG00000026851	*FMNL2*	1'425	1'180	1'298	1'234	1'363	0.143	0.859976809	1
ENSBTAG00000026880	*KRT85*	53	105	84	46	121	1.401	0.319546923	1
ENSBTAG00000026882	*BT.55894*	179	255	225	155	294	0.926	0.369564999	1
ENSBTAG00000026885	*ABCB11*	26	9	16	23	10	-1.115	0.73867587	1
ENSBTAG00000026886	*MP68*	847	649	741	734	749	0.031	0.971943253	1
ENSBTAG00000026893	*EXOC3L4*	31	22	26	27	25	-0.080	0.993095314	1
ENSBTAG00000026896	*FOXF2*	21	5	12	18	6	-1.655	0.689790335	1
ENSBTAG00000026909	*protein_coding*	9'182	11'163	10'421	7'952	12'890	0.697	0.36338742	1
ENSBTAG00000026913	*HHIPL1*	346	120	219	300	139	-1.113	0.286271981	1
ENSBTAG00000026915	*NEK1*	3'298	1'591	2'347	2'856	1'837	-0.637	0.420191198	1
ENSBTAG00000026916	*PRR13*	1'068	632	827	925	730	-0.342	0.68463983	1
ENSBTAG00000026919	*BT.89412*	1'945	1'613	1'773	1'684	1'863	0.145	0.855280986	1
ENSBTAG00000026936	*BT.56445*	272	142	200	236	164	-0.523	0.62405045	1
ENSBTAG00000026944	*BT.103911*	3	5	4	3	6	1.152	0.920866718	1
ENSBTAG00000026953	*TRIP11*	148	127	137	128	147	0.194	0.873146982	1
ENSBTAG00000026962	*XPOT*	8'662	4'637	6'428	7'502	5'354	-0.486	0.52784869	1
ENSBTAG00000026963	*NCALD*	1'493	585	984	1'293	675	-0.937	0.259878089	1
ENSBTAG00000026966	*RASSF9*	92	87	90	80	100	0.334	0.81006563	1
ENSBTAG00000026971	*BT.91218*	139	215	184	120	248	1.044	0.335937587	1
ENSBTAG00000026972	*MYF5*	2	21	13	2	24	3.807	0.357738515	1
ENSBTAG00000026977	*PTPRQ*	21	9	14	18	10	-0.807	0.832834108	1
ENSBTAG00000026992	*pseudogene*	2	0	1	2	0		0.974934741	1
ENSBTAG00000026993	*CGI-119*	5'862	3'731	4'692	5'077	4'308	-0.237	0.759945574	1
ENSBTAG00000026994	*C2H2orf88*	40	27	33	35	31	-0.152	0.959402546	1
ENSBTAG00000026995	*BT.49763*	14'803	6'507	10'167	12'820	7'514	-0.771	0.315939529	1
ENSBTAG00000027013	*pseudogene*	1	0	0	1	0		1	1
ENSBTAG00000027015	*RPL17*	419	863	680	363	997	1.457	0.093204248	1
ENSBTAG00000027017	*SIX3*	1	0	0	1	0		1	1
ENSBTAG00000027020	*COL5A2*	175'679	128'452	150'233	152'142	148'324	-0.037	0.962084972	1
ENSBTAG00000027024	*ARHGAP5*	2'490	1'566	1'982	2'156	1'808	-0.254	0.749414682	1
ENSBTAG00000027033	*CMA1*	1	0	0	1	0		1	1
ENSBTAG00000027049	*MGC137021*	287	207	244	249	239	-0.056	0.960643817	1
ENSBTAG00000027051	*PTAFR*	145	60	97	126	69	-0.858	0.51887617	1
ENSBTAG00000027052	*protein_coding*	0	1	1	0	1	Inf	0.993540919	1
ENSBTAG00000027058	*MESP2*	304	272	289	263	314	0.255	0.795265099	1
ENSBTAG00000027059	*C21H15orf38*	2'024	1'745	1'884	1'753	2'015	0.201	0.799787342	1
ENSBTAG00000027064	*BTBD11*	427	374	401	370	432	0.224	0.808295178	1
ENSBTAG00000027069	*MGC127733*	694	474	574	601	547	-0.135	0.879322322	1
ENSBTAG00000027074	*SV2B*	617	266	421	534	307	-0.799	0.382439075	1
ENSBTAG00000027075	*BT.62501*	32'615	30'794	31'902	28'245	35'558	0.332	0.661618356	1
ENSBTAG00000027118	*KCNU1*	1	0	0	1	0		1	1
ENSBTAG00000027126	*MUC15*	558	423	486	483	488	0.015	0.988110721	1
ENSBTAG00000027151	*ARHGAP26*	202	179	191	175	207	0.241	0.824965393	1
ENSBTAG00000027159	*ZBTB8OS*	1'397	850	1'096	1'210	981	-0.302	0.713738236	1
ENSBTAG00000027172	*protein_coding*	23	71	51	20	82	2.041	0.245921366	1
ENSBTAG00000027173	*CHP*	3'418	2'836	3'117	2'960	3'275	0.146	0.851323754	1
ENSBTAG00000027181	*LAMA3*	4'926	3'177	3'967	4'266	3'668	-0.218	0.779596787	1
ENSBTAG00000027182	*NR3C2*	155	95	122	134	110	-0.291	0.817230502	1
ENSBTAG00000027197	*KIF6*	6	3	4	5	3	-0.585	0.976586913	1
ENSBTAG00000027201	*RAPH1*	309	137	213	268	158	-0.758	0.469026672	1
ENSBTAG00000027204	*BT.65432*	38	13	24	33	15	-1.132	0.664786809	1

Ensembl gene ID	geneName	counts wildtype horn bud	counts polled horn bud	baseMean	baseMean wildtype horn bud	baseMean polled horn bud	log2FoldChange	pval	padj
ENSBTAG00000027205	*PGBD5*	197	103	145	171	119	-0.521	0.656190236	1
ENSBTAG00000027221	*protein_coding*	44	39	42	38	45	0.241	0.909470319	1
ENSBTAG00000027225	*LAP*	5	3	4	4	3	-0.322	1	1
ENSBTAG00000027246	*BT.49039*	75	20	44	65	23	-1.492	0.426126997	1
ENSBTAG00000027274	*protein_coding*	42	77	63	36	89	1.290	0.414156661	1
ENSBTAG00000027279	*processed_pseudogene*	491	583	549	425	673	0.663	0.450085379	1
ENSBTAG00000027312	*ECT2L*	13	5	9	11	6	-0.963	0.868297704	1
ENSBTAG00000027316	*UBE2V1*	5'080	2'898	3'873	4'399	3'346	-0.395	0.611654194	1
ENSBTAG00000027317	*RNF114*	3'404	3'313	3'387	2'948	3'826	0.376	0.628679853	1
ENSBTAG00000027319	*protein_coding*	1'341	1'703	1'564	1'161	1'966	0.760	0.344435202	1
ENSBTAG00000027320	*KCNB1*	61	24	40	53	28	-0.931	0.635111532	1
ENSBTAG00000027321	*CCDC18*	405	113	241	351	130	-1.427	0.164939157	1
ENSBTAG00000027326	*TP53RK*	816	516	651	707	596	-0.246	0.776147847	1
ENSBTAG00000027327	*C20ORF123*	8	0	3	7	0		0.72953145	1
ENSBTAG00000027337	*CTSA*	4'865	5'560	5'317	4'213	6'420	0.608	0.431221664	1
ENSBTAG00000027341	*BT.56249*	3	0	1	3	0		0.936647693	1
ENSBTAG00000027348	*OOSP1*	3	2	2	3	2	-0.170	1	1
ENSBTAG00000027357	*FGF22*	9	3	6	8	3	-1.170	0.890813994	1
ENSBTAG00000027361	*ROMO1*	2'701	2'174	2'425	2'339	2'510	0.102	0.896586336	1
ENSBTAG00000027375	*DUSP22*	195	271	241	169	313	0.890	0.380537159	1
ENSBTAG00000027386	*pseudogene*	555	731	662	481	844	0.812	0.344694023	1
ENSBTAG00000027387	*NRSN2*	116	18	61	100	21	-2.273	0.169635695	1
ENSBTAG00000027390	*C13H20ORF43*	1'849	1'724	1'796	1'601	1'991	0.314	0.692748279	1
ENSBTAG00000027396	*pseudogene*	0	5	3	0	6	Inf	0.757530434	1
ENSBTAG00000027397	*PPP1R3D*	39	16	26	34	18	-0.870	0.728438954	1
ENSBTAG00000027405	*C13H20orf195*	12	3	7	10	3	-1.585	0.809704871	1
ENSBTAG00000027407	*TNFRSF6B*	189	165	177	164	191	0.219	0.844205248	1
ENSBTAG00000027409	*SLC4A11*	233	73	143	202	84	-1.259	0.283374293	1
ENSBTAG00000027412	*SOD1*	2'546	2'833	2'738	2'205	3'271	0.569	0.467662987	1
ENSBTAG00000027416	*CALML3*	2	1	1	2	1	-0.585	1	1
ENSBTAG00000027420	*protein_coding*	31	18	24	27	21	-0.369	0.900607694	1
ENSBTAG00000027425	*C3H1orf110*	260	127	186	225	147	-0.619	0.568652437	1
ENSBTAG00000027426	*processed_pseudogene*	4'206	7'519	6'162	3'643	8'682	1.253	0.107694824	1
ENSBTAG00000027431	*ZNF227*	4'486	2'158	3'188	3'885	2'492	-0.641	0.412732016	1
ENSBTAG00000027434	*SORCS2*	803	682	741	695	788	0.179	0.832673777	1
ENSBTAG00000027438	*protein_coding*	6	4	5	5	5	-0.170	1	1
ENSBTAG00000027442	*BT.91925*	2'202	2'184	2'214	1'907	2'522	0.403	0.608960226	1
ENSBTAG00000027444	*BT.106587*	3'889	2'778	3'288	3'368	3'208	-0.070	0.929077536	1
ENSBTAG00000027446	*RSU1*	3'827	2'796	3'271	3'314	3'229	-0.038	0.962363828	1
ENSBTAG00000027453	*RNF4*	7'101	3'978	5'372	6'150	4'593	-0.421	0.585828797	1
ENSBTAG00000027464	*FGFRL1*	416	367	392	360	424	0.234	0.800226599	1
ENSBTAG00000027477	*BT.88113*	535	169	329	463	195	-1.247	0.193671318	1
ENSBTAG00000027506	*SNRPB2*	3'124	2'123	2'578	2'705	2'451	-0.142	0.8570835	1
ENSBTAG00000027513	*CXCL2*	2	0	1	2	0		0.974934741	1
ENSBTAG00000027516	*APLNR*	2'785	2'315	2'543	2'412	2'673	0.148	0.849677876	1
ENSBTAG00000027534	*processed_pseudogene*	223	263	248	193	304	0.653	0.516370003	1
ENSBTAG00000027547	*protein_coding*	0	1	1	0	1	Inf	0.993540919	1
ENSBTAG00000027557	*ZNF408*	955	1'313	1'172	827	1'516	0.874	0.285681748	1
ENSBTAG00000027562	*C15H11orf94*	16	22	20	14	25	0.874	0.767703817	1
ENSBTAG00000027563	*BT.47446*	37	128	90	32	148	2.206	0.115433451	1
ENSBTAG00000027569	*BT.24324*	2'448	1'323	1'824	2'120	1'528	-0.473	0.553212327	1
ENSBTAG00000027577	*C15H11orf91*	3	1	2	3	1	-1.170	0.985253077	1
ENSBTAG00000027578	*protein_coding*	2	0	1	2	0		0.974934741	1
ENSBTAG00000027610	*RPL36A*	20'779	15'396	17'886	17'995	17'778	-0.018	0.982385615	1
ENSBTAG00000027612	*PPME1*	1'645	1'026	1'305	1'425	1'185	-0.266	0.74357743	1
ENSBTAG00000027626	*GOLGA7*	5'114	3'939	4'489	4'429	4'548	0.038	0.959974337	1
ENSBTAG00000027629	*ANK1*	30	96	68	26	111	2.093	0.177382565	1
ENSBTAG00000027630	*CISD2*	1'205	813	991	1'044	939	-0.153	0.854861374	1
ENSBTAG00000027635	*PPAPDC1B*	1'944	1'884	1'930	1'684	2'175	0.370	0.640746984	1
ENSBTAG00000027642	*C11ORF42*	93	26	55	81	30	-1.424	0.397568907	1
ENSBTAG00000027654	*EIF4EBP1*	1'406	1'214	1'310	1'218	1'402	0.203	0.801632233	1
ENSBTAG00000027655	*TIFA*	773	357	541	669	412	-0.700	0.427598858	1
ENSBTAG00000027665	*BRF2*	647	763	721	560	881	0.653	0.44269967	1
ENSBTAG00000027676	*IL18BP*	801	517	645	694	597	-0.217	0.802953219	1
ENSBTAG00000027684	*FOLR2*	1'066	840	947	923	970	0.071	0.931953438	1
ENSBTAG00000027694	*TTC30A*	701	376	521	607	434	-0.484	0.585200274	1
ENSBTAG00000027696	*TTC30B*	108	78	92	94	90	-0.054	0.97778452	1
ENSBTAG00000027711	*YIF1B*	1'057	1'047	1'062	915	1'209	0.401	0.624822508	1
ENSBTAG00000027713	*BT.56524*	753	556	647	652	642	-0.023	0.981851056	1
ENSBTAG00000027715	*processed_pseudogene*	287	276	284	249	319	0.359	0.714549507	1
ENSBTAG00000027716	*C7H19orf53*	346	471	422	300	544	0.860	0.346046671	1
ENSBTAG00000027722	*IPO7*	5'330	5'525	5'498	4'616	6'380	0.467	0.544491778	1

Ensembl gene ID	geneName	counts wildtype horn bud	counts polled horn bud	baseMean	baseMean wildtype horn bud	baseMean polled horn bud	log2FoldChange	pval	padj
ENSBTAG00000027727	*BT.94335*	2	0	1	2	0		0.974934741	1
ENSBTAG00000027728	*NUDT12*	1'349	422	828	1'168	487	-1.262	0.137098649	1
ENSBTAG00000027759	*protein_coding*	1	1	1	1	1	0.415	1	1
ENSBTAG00000027760	*pseudogene*	261	366	324	226	423	0.903	0.344378303	1
ENSBTAG00000027764	*EID1*	3'515	2'215	2'801	3'044	2'558	-0.251	0.748986365	1
ENSBTAG00000027770	*protein_coding*	792	205	461	686	237	-1.535	0.093149393	1
ENSBTAG00000027772	*RPS25*	3'863	7'837	6'197	3'345	9'049	1.436	0.066489002	1
ENSBTAG00000027775	*BT.102578*	1	0	0	1	0		1	1
ENSBTAG00000027786	*pseudogene*	2	2	2	2	2	0.415	1	1
ENSBTAG00000027787	*TCEB2*	2'622	2'861	2'787	2'271	3'304	0.541	0.489669531	1
ENSBTAG00000027789	*PPIG*	3'604	1'267	2'292	3'121	1'463	-1.093	0.169307638	1
ENSBTAG00000027795	*BT.21913*	1'997	1'148	1'528	1'729	1'326	-0.384	0.633435698	1
ENSBTAG00000027803	*processed_pseudogene*	6'243	2'980	4'424	5'407	3'441	-0.652	0.401074805	1
ENSBTAG00000027809	*RABGAP1L*	195	129	159	169	149	-0.181	0.877951877	1
ENSBTAG00000027832	*BT.87378*	82	97	92	71	112	0.657	0.62795525	1
ENSBTAG00000027841	*protein_coding*	82	85	85	71	98	0.467	0.74047423	1
ENSBTAG00000027843	*TCEAL8*	5'422	4'570	4'986	4'696	5'277	0.168	0.826691384	1
ENSBTAG00000027854	*BT.24416*	111	27	64	96	31	-1.624	0.306393761	1
ENSBTAG00000027863	*processed_pseudogene*	11	32	23	10	37	1.956	0.457962514	1
ENSBTAG00000027867	*protein_coding*	73	75	75	63	87	0.454	0.759606276	1
ENSBTAG00000027868	*DLG2*	333	73	186	288	84	-1.775	0.107788223	1
ENSBTAG00000027875	*CCDC141*	207	104	150	179	120	-0.578	0.61692401	1
ENSBTAG00000027878	*RPL12*	7	4	5	6	5	-0.392	0.98714753	1
ENSBTAG00000027879	*BRP44L*	1'112	750	915	963	866	-0.153	0.855434568	1
ENSBTAG00000027900	*LRRIQ1*	11	2	6	10	2	-2.044	0.786625039	1
ENSBTAG00000027916	*MALT1*	867	657	755	751	759	0.015	0.987023427	1
ENSBTAG00000027924	*SNX18*	5'347	2'530	3'776	4'631	2'921	-0.665	0.393648481	1
ENSBTAG00000027932	*BIRC6*	5'456	2'954	4'068	4'725	3'411	-0.470	0.545001389	1
ENSBTAG00000027936	*C3H1orf146*	21	3	11	18	3	-2.392	0.596985337	1
ENSBTAG00000027937	*PRP*	3'975	2'299	3'049	3'442	2'655	-0.375	0.631763382	1
ENSBTAG00000027962	*processed_pseudogene*	1'570	2'516	2'132	1'360	2'905	1.095	0.169282424	1
ENSBTAG00000027974	*KERIA*	5	1	3	4	1	-1.907	0.9188325	1
ENSBTAG00000027980	*TAF9*	4'116	2'086	2'987	3'565	2'409	-0.565	0.470291213	1
ENSBTAG00000027983	*BT.58967*	1'012	546	753	876	630	-0.475	0.57545645	1
ENSBTAG00000027991	*ANAPC1*	1'839	1'361	1'582	1'593	1'572	-0.019	0.982437546	1
ENSBTAG00000028094	*U2*	0	1	1	0	1	Inf	0.993540919	1
ENSBTAG00000028153	*5S_rRNA*	2	0	1	2	0		0.974934741	1
ENSBTAG00000028229	*U1*	1	1	1	1	1	0.415	1	1
ENSBTAG00000028273	*5S_rRNA*	19	7	12	16	8	-1.026	0.806704154	1
ENSBTAG00000028324	*5S_rRNA*	1	0	0	1	0		1	1
ENSBTAG00000028330	*U3*	1	1	1	1	1	0.415	1	1
ENSBTAG00000028355	*5S_rRNA*	2	0	1	2	0		0.974934741	1
ENSBTAG00000028359	*U3*	62	7	31	54	8	-2.732	0.242973678	1
ENSBTAG00000028362	*5S_rRNA*	1	0	0	1	0		1	1
ENSBTAG00000028419	*U1*	0	1	1	0	1	Inf	0.993540919	1
ENSBTAG00000028421	*U1*	18	2	9	16	2	-2.755	0.605203757	1
ENSBTAG00000028430	*5S_rRNA*	1	0	0	1	0		1	1
ENSBTAG00000028443	*5S_rRNA*	2	6	4	2	7	2.000	0.840116992	1
ENSBTAG00000028616	*5S_rRNA*	1	2	2	1	2	1.415	0.981979269	1
ENSBTAG00000028669	*5S_rRNA*	1	0	0	1	0		1	1
ENSBTAG00000028675	*5S_rRNA*	27	16	21	23	18	-0.340	0.919037185	1
ENSBTAG00000028692	*5S_rRNA*	1	0	0	1	0		1	1
ENSBTAG00000028809	*U1*	17	19	18	15	22	0.576	0.859294388	1
ENSBTAG00000028923	*U1*	10	21	16	9	24	1.485	0.64796492	1
ENSBTAG00000028980	*5S_rRNA*	1	0	0	1	0		1	1
ENSBTAG00000028988	*5S_rRNA*	1	0	0	1	0		1	1
ENSBTAG00000029007	*U2*	1	0	0	1	0		1	1
ENSBTAG00000029055	*U1*	1	1	1	1	1	0.415	1	1
ENSBTAG00000029069	*U2*	0	1	1	0	1	Inf	0.993540919	1
ENSBTAG00000029075	*5S_rRNA*	0	1	1	0	1	Inf	0.993540919	1
ENSBTAG00000029076	*5S_rRNA*	3	10	7	3	12	2.152	0.727540088	1
ENSBTAG00000029119	*5S_rRNA*	1	0	0	1	0		1	1
ENSBTAG00000029173	*5S_rRNA*	3	0	1	3	0		0.936647693	1
ENSBTAG00000029206	*5S_rRNA*	4	2	3	3	2	-0.585	0.996239345	1
ENSBTAG00000029237	*5S_rRNA*	296	221	256	256	255	-0.007	1	1
ENSBTAG00000029283	*5S_rRNA*	1	0	0	1	0		1	1
ENSBTAG00000029287	*U1*	1	0	0	1	0		1	1
ENSBTAG00000029291	*5S_rRNA*	0	1	1	0	1	Inf	0.993540919	1
ENSBTAG00000029295	*5S_rRNA*	4	1	2	3	1	-1.585	0.952247636	1
ENSBTAG00000029317	*5S_rRNA*	19	21	20	16	24	0.559	0.85309117	1
ENSBTAG00000029452	*5S_rRNA*	1	0	0	1	0		1	1
ENSBTAG00000029512	*U1*	6	0	3	5	0		0.81115604	1

Ensembl gene ID	geneName	counts wildtype horn bud	counts polled horn bud	baseMean	baseMean wildtype horn bud	baseMean polled horn bud	log2FoldChange	pval	padj
ENSBTAG00000029540	*5S_rRNA*	1	0	0	1	0		1	1
ENSBTAG00000029651	*5S_rRNA*	2	0	1	2	0		0.974934741	1
ENSBTAG00000029765	*bta-mir-29b-2*	12	2	6	10	2	-2.170	0.758651977	1
ENSBTAG00000029768	*bta-mir-99a*	1	0	0	1	0		1	1
ENSBTAG00000029769	*bta-mir-185*	3	1	2	3	1	-1.170	0.985253077	1
ENSBTAG00000029770	*bta-mir-380*	1	0	0	1	0		1	1
ENSBTAG00000029771	*bta-mir-140*	3	2	2	3	2	-0.170	1	1
ENSBTAG00000029772	*bta-let-7a-3*	16	19	18	14	22	0.663	0.837933827	1
ENSBTAG00000029775	*bta-mir-338*	0	1	1	0	1	Inf	0.993540919	1
ENSBTAG00000029776	*bta-mir-124a-1*	1	0	0	1	0		1	1
ENSBTAG00000029777	*bta-mir-340*	1	0	0	1	0		1	1
ENSBTAG00000029778	*bta-mir-32*	0	1	1	0	1	Inf	0.993540919	1
ENSBTAG00000029782	*bta-mir-370*	3	3	3	3	3	0.415	1	1
ENSBTAG00000029790	*bta-mir-1-1*	0	2	1	0	2	Inf	0.939077559	1
ENSBTAG00000029791	*bta-mir-3578*	2	3	3	2	3	1.000	0.97171833	1
ENSBTAG00000029792	*bta-mir-493*	2	10	7	2	12	2.737	0.678653301	1
ENSBTAG00000029794	*bta-mir-369*	0	1	1	0	1	Inf	0.993540919	1
ENSBTAG00000029796	*bta-mir-187*	1	0	0	1	0		1	1
ENSBTAG00000029797	*bta-mir-186*	61	14	34	53	16	-1.708	0.422380737	1
ENSBTAG00000029798	*bta-mir-532*	2	0	1	2	0		0.974934741	1
ENSBTAG00000029800	*bta-mir-335*	1	0	0	1	0		1	1
ENSBTAG00000029802	*bta-mir-92a-1*	17	5	10	15	6	-1.350	0.775139346	1
ENSBTAG00000029803	*miRNA*	51	31	40	44	36	-0.303	0.887623927	1
ENSBTAG00000029805	*bta-mir-30c*	1	5	3	1	6	2.737	0.836148221	1
ENSBTAG00000029807	*bta-mir-450a-1*	276	161	212	239	186	-0.363	0.731212653	1
ENSBTAG00000029811	*bta-mir-378-1*	3	0	1	3	0		0.936647693	1
ENSBTAG00000029812	*bta-mir-324*	10	15	13	9	17	1.000	0.799125949	1
ENSBTAG00000029817	*bta-mir-93*	2	1	1	2	1	-0.585	1	1
ENSBTAG00000029818	*miRNA*	152	100	124	132	115	-0.189	0.883216309	1
ENSBTAG00000029820	*bta-mir-323*	0	1	1	0	1	Inf	0.993540919	1
ENSBTAG00000029822	*bta-mir-423*	12	6	9	10	7	-0.585	0.929512138	1
ENSBTAG00000029826	*bta-mir-133b*	0	7	4	0	8	Inf	0.646259203	1
ENSBTAG00000029827	*bta-mir-125b-2*	13	3	7	11	3	-1.700	0.783725709	1
ENSBTAG00000029828	*bta-mir-421*	415	176	281	359	203	-0.822	0.403105583	1
ENSBTAG00000029830	*bta-mir-146a*	1	0	0	1	0		1	1
ENSBTAG00000029836	*bta-mir-30f*	13	2	7	11	2	-2.285	0.731320841	1
ENSBTAG00000029839	*bta-mir-181c*	1	0	0	1	0		1	1
ENSBTAG00000029841	*bta-mir-181a-2*	6	4	5	5	5	-0.170	1	1
ENSBTAG00000029843	*bta-mir-107*	2	0	1	2	0		0.974934741	1
ENSBTAG00000029844	*bta-mir-125a*	10	7	8	9	8	-0.100	1	1
ENSBTAG00000029845	*bta-mir-411a*	1	0	0	1	0		1	1
ENSBTAG00000029846	*bta-mir-27b*	14	17	16	12	20	0.695	0.84345254	1
ENSBTAG00000029849	*bta-mir-181a-1*	0	1	1	0	1	Inf	0.993540919	1
ENSBTAG00000029850	*bta-mir-26b*	5	1	3	4	1	-1.907	0.9188325	1
ENSBTAG00000029852	*bta-mir-148b*	1	0	0	1	0		1	1
ENSBTAG00000029853	*bta-let-7d*	48	9	26	42	10	-2.000	0.42475096	1
ENSBTAG00000029855	*bta-mir-101-1*	3	0	1	3	0		0.936647693	1
ENSBTAG00000029857	*bta-mir-145*	32	9	19	28	10	-1.415	0.637653774	1
ENSBTAG00000029862	*miRNA*	6	3	4	5	3	-0.585	0.976586913	1
ENSBTAG00000029863	*bta-mir-208a*	0	11	6	0	13	Inf	0.464170207	1
ENSBTAG00000029869	*bta-mir-26a-2*	30	54	44	26	62	1.263	0.495108628	1
ENSBTAG00000029880	*bta-let-7e*	4	2	3	3	2	-0.585	0.996239345	1
ENSBTAG00000029881	*bta-let-7a-2*	0	1	1	0	1	Inf	0.993540919	1
ENSBTAG00000029884	*bta-mir-29a*	0	1	1	0	1	Inf	0.993540919	1
ENSBTAG00000029885	*bta-mir-450a-2*	157	84	116	136	97	-0.487	0.698525409	1
ENSBTAG00000029886	*miRNA*	396	66	210	343	76	-2.170	0.04555734	1
ENSBTAG00000029887	*bta-mir-345*	1	1	1	1	1	0.415	1	1
ENSBTAG00000029889	*bta-mir-452*	83	53	67	72	61	-0.232	0.889176645	1
ENSBTAG00000029890	*bta-mir-3604-1*	12	1	6	10	1	-3.170	0.692545189	1
ENSBTAG00000029893	*bta-mir-9-2*	17	1	8	15	1	-3.672	0.555170792	1
ENSBTAG00000029895	*bta-mir-218-2*	1	0	0	1	0		1	1
ENSBTAG00000029896	*bta-mir-181b-2*	18	6	11	16	7	-1.170	0.791784766	1
ENSBTAG00000029897	*bta-mir-21*	201	72	129	174	83	-1.066	0.378307524	1
ENSBTAG00000029901	*bta-mir-23a*	2	1	1	2	1	-0.585	1	1
ENSBTAG00000029902	*bta-mir-455*	1	0	0	1	0		1	1
ENSBTAG00000029903	*bta-mir-216a*	3	1	2	3	1	-1.170	0.985253077	1
ENSBTAG00000029905	*bta-mir-132*	1	0	0	1	0		1	1
ENSBTAG00000029906	*bta-mir-133a-2*	0	4	2	0	5	Inf	0.817359038	1
ENSBTAG00000029909	*bta-mir-7-2*	181	51	108	157	59	-1.412	0.273603554	1
ENSBTAG00000029912	*bta-let-7c*	1	0	0	1	0		1	1
ENSBTAG00000029913	*bta-mir-425*	72	53	62	62	61	-0.027	0.99943772	1
ENSBTAG00000029914	*bta-mir-365-2*	4	1	2	3	1	-1.585	0.952247636	1

Ensembl gene ID	geneName	counts wildtype horn bud	counts polled horn bud	baseMean	baseMean wildtype horn bud	baseMean polled horn bud	log2FoldChange	pval	padj
ENSBTAG00000029917	*bta-mir-135a-2*	5	0	2	4	0		0.853268594	1
ENSBTAG00000029918	*bta-mir-99b*	8	2	5	7	2	-1.585	0.87391615	1
ENSBTAG00000029920	*bta-mir-25*	102	24	58	88	28	-1.672	0.312300572	1
ENSBTAG00000029921	*bta-mir-197*	10	4	7	9	5	-0.907	0.905171387	1
ENSBTAG00000029922	*bta-mir-9-1*	2	0	1	2	0		0.974934741	1
ENSBTAG00000029924	*bta-mir-34a*	3	0	1	3	0		0.936647693	1
ENSBTAG00000029925	*bta-mir-125b-1*	2	0	1	2	0		0.974934741	1
ENSBTAG00000029926	*bta-mir-1-2*	1	3	2	1	3	2.000	0.933774563	1
ENSBTAG00000029927	*bta-mir-10b*	20	42	33	17	48	1.485	0.490311949	1
ENSBTAG00000029930	*bta-mir-22*	49	29	38	42	33	-0.342	0.875360027	1
ENSBTAG00000029931	*bta-mir-199a-1*	109	29	64	94	33	-1.495	0.344233309	1
ENSBTAG00000029932	*bta-mir-101-2*	1	0	0	1	0		1	1
ENSBTAG00000029933	*bta-mir-330*	2	0	1	2	0		0.974934741	1
ENSBTAG00000029935	*bta-let-7f-1*	3	0	1	3	0		0.936647693	1
ENSBTAG00000029937	*bta-mir-26c*	0	1	1	0	1	Inf	0.993540919	1
ENSBTAG00000029938	*bta-mir-138-2*	2	0	1	2	0		0.974934741	1
ENSBTAG00000029940	*bta-mir-192*	2	1	1	2	1	-0.585	1	1
ENSBTAG00000029942	*bta-mir-29c*	25	2	12	22	2	-3.229	0.459170232	1
ENSBTAG00000029954	*bta-mir-24-1*	16	17	17	14	20	0.503	0.887822469	1
ENSBTAG00000029955	*bta-mir-193a*	2	0	1	2	0		0.974934741	1
ENSBTAG00000029957	*bta-mir-191*	30	7	17	26	8	-1.684	0.602356086	1
ENSBTAG00000029969	*bta-mir-181d*	3	3	3	3	3	0.415	1	1
ENSBTAG00000029974	*bta-mir-199b*	1	4	3	1	5	2.415	0.884888347	1
ENSBTAG00000029976	*bta-mir-484*	2	1	1	2	1	-0.585	1	1
ENSBTAG00000029982	*bta-mir-142*	2	0	1	2	0		0.974934741	1
ENSBTAG00000029988	*bta-mir-331*	98	38	64	85	44	-0.952	0.544186719	1
ENSBTAG00000029989	*bta-mir-23b*	3	5	4	3	6	1.152	0.920866718	1
ENSBTAG00000029993	*bta-mir-181b-1*	2	2	2	2	2	0.415	1	1
ENSBTAG00000029994	*bta-let-7i*	14	12	13	12	14	0.193	0.980949072	1
ENSBTAG00000029995	*bta-mir-487a*	1	3	2	1	3	2.000	0.933774563	1
ENSBTAG00000029996	*bta-mir-27a*	0	1	1	0	1	Inf	0.993540919	1
ENSBTAG00000029999	*bta-mir-328*	0	1	1	0	1	Inf	0.993540919	1
ENSBTAG00000030010	*bta-mir-92b*	2	2	2	2	2	0.415	1	1
ENSBTAG00000030016	*bta-mir-656*	45	68	59	39	79	1.011	0.533061897	1
ENSBTAG00000030017	*bta-mir-654*	1	0	0	1	0		1	1
ENSBTAG00000030019	*bta-mir-545*	1	0	0	1	0		1	1
ENSBTAG00000030024	*bta-mir-542*	220	104	155	191	120	-0.666	0.559567895	1
ENSBTAG00000030026	*bta-mir-487b*	3	6	5	3	7	1.415	0.880191876	1
ENSBTAG00000030035	*bta-mir-544a*	8	5	6	7	6	-0.263	0.996869191	1
ENSBTAG00000030049	*bta-mir-190a*	1	0	0	1	0		1	1
ENSBTAG00000030052	*bta-mir-631*	5	9	7	4	10	1.263	0.837733706	1
ENSBTAG00000030060	*bta-mir-584-2*	12	7	9	10	8	-0.363	0.964206651	1
ENSBTAG00000030062	*bta-mir-200b*	1	0	0	1	0		1	1
ENSBTAG00000030071	*bta-mir-24-2*	1	1	1	1	1	0.415	1	1
ENSBTAG00000030082	*bta-mir-584-4*	2	0	1	2	0		0.974934741	1
ENSBTAG00000030084	*bta-mir-503*	56	39	47	48	45	-0.107	0.966395922	1
ENSBTAG00000030089	*bta-mir-149*	4	0	2	3	0		0.89545886	1
ENSBTAG00000030090	*bta-mir-544b-1*	0	2	1	0	2	Inf	0.939077559	1
ENSBTAG00000030095	*bta-mir-584-5*	3	0	1	3	0		0.936647693	1
ENSBTAG00000030100	*bta-mir-562*	0	1	1	0	1	Inf	0.993540919	1
ENSBTAG00000030111	*bta-mir-763*	3	1	2	3	1	-1.170	0.985253077	1
ENSBTAG00000030113	*bta-mir-195*	1	0	0	1	0		1	1
ENSBTAG00000030114	*bta-mir-143*	0	1	1	0	1	Inf	0.993540919	1
ENSBTAG00000030117	*miRNA*	9	2	5	8	2	-1.755	0.844314416	1
ENSBTAG00000030119	*miRNA*	7	6	6	6	7	0.193	1	1
ENSBTAG00000030162	*AXIN2*	2'913	5'102	4'207	2'523	5'891	1.224	0.118400308	1
ENSBTAG00000030164	*RPL38*	12'610	13'369	13'179	10'921	15'437	0.499	0.513038054	1
ENSBTAG00000030166	*BTBD17*	6	6	6	5	7	0.415	0.976119138	1
ENSBTAG00000030168	*MIF4GD*	875	602	726	758	695	-0.124	0.88550393	1
ENSBTAG00000030169	*BT.106572*	6'967	5'457	6'167	6'034	6'301	0.063	0.934604846	1
ENSBTAG00000030170	*HN1*	2'809	1'810	2'261	2'433	2'090	-0.219	0.781988455	1
ENSBTAG00000030172	*PRPSAP1*	4'365	4'302	4'374	3'780	4'968	0.394	0.61020043	1
ENSBTAG00000030173	*QRICH2*	144	207	182	125	239	0.939	0.388209347	1
ENSBTAG00000030174	*ACOX1*	986	637	795	854	736	-0.215	0.799696965	1
ENSBTAG00000030175	*FBF1*	1'172	2'103	1'722	1'015	2'428	1.259	0.118913552	1
ENSBTAG00000030180	*SHANK3*	1'279	1'275	1'290	1'108	1'472	0.411	0.612185893	1
ENSBTAG00000030182	*BT.40394*	354	137	232	307	158	-0.955	0.352654429	1
ENSBTAG00000030186	*USMG5*	209	55	122	181	64	-1.511	0.222875347	1
ENSBTAG00000030189	*BT.38812*	140	67	99	121	77	-0.648	0.624615291	1
ENSBTAG00000030190	*COL6A3*	136'562	174'529	159'897	118'266	201'529	0.769	0.312532953	1
ENSBTAG00000030193	*GPR35*	16	8	12	14	9	-0.585	0.904936474	1
ENSBTAG00000030198	*TMEM232*	213	99	149	184	114	-0.690	0.549802117	1

Ensembl gene ID	geneName	counts wildtype horn bud	counts polled horn bud	baseMean	baseMean wildtype horn bud	baseMean polled horn bud	log2FoldChange	pval	padj
ENSBTAG00000030199	*protein_coding*	1	10	6	1	12	3.737	0.613387923	1
ENSBTAG00000030200	*ARL16*	350	442	407	303	510	0.752	0.412228433	1
ENSBTAG00000030208	*FAM132B*	27	27	27	23	31	0.415	0.87171026	1
ENSBTAG00000030209	*ARHGDIA*	10'278	13'420	12'199	8'901	15'496	0.800	0.296969682	1
ENSBTAG00000030210	*SLCO6A1*	4	2	3	3	2	-0.585	0.996239345	1
ENSBTAG00000030222	*L3MBTL2*	604	1'278	999	523	1'476	1.496	0.074558256	1
ENSBTAG00000030225	*RBX1*	2'282	1'900	2'085	1'976	2'194	0.151	0.848497161	1
ENSBTAG00000030227	*BT.58387*	1'204	353	725	1'043	408	-1.355	0.115612899	1
ENSBTAG00000030230	*processed_pseudogene*	85	137	116	74	158	1.104	0.376088359	1
ENSBTAG00000030246	*BT.42111*	3	3	3	3	3	0.415	1	1
ENSBTAG00000030247	*NELF*	3'374	3'853	3'686	2'922	4'449	0.607	0.435401791	1
ENSBTAG00000030255	*BT.45290*	761	1'244	1'048	659	1'436	1.124	0.17515991	1
ENSBTAG00000030257	*SH3BP1*	1'098	1'625	1'414	951	1'876	0.981	0.226550437	1
ENSBTAG00000030258	*CDC42EP1*	4'191	2'600	3'316	3'630	3'002	-0.274	0.725919278	1
ENSBTAG00000030259	*RASGRF2*	114	7	53	99	8	-3.610	0.053461107	1
ENSBTAG00000030269	*BT.87230*	1	2	2	1	2	1.415	0.981979269	1
ENSBTAG00000030274	*CMC2*	499	712	627	432	822	0.928	0.284215169	1
ENSBTAG00000030278	*EGFL7*	330	158	234	286	182	-0.648	0.527162034	1
ENSBTAG00000030281	*processed_pseudogene*	21'734	19'897	20'899	18'822	22'975	0.288	0.704947331	1
ENSBTAG00000030282	*C7H5orf54*	2'928	1'397	2'074	2'536	1'613	-0.653	0.410940235	1
ENSBTAG00000030285	*BT.57890*	1'035	937	989	896	1'082	0.272	0.742135803	1
ENSBTAG00000030286	*GABRA1*	49	11	28	42	13	-1.740	0.469847408	1
ENSBTAG00000030296	*LCNL1*	58	88	76	50	102	1.016	0.484828757	1
ENSBTAG00000030301	*MAP7D1*	3'246	3'790	3'594	2'811	4'376	0.639	0.412027731	1
ENSBTAG00000030302	*EDF1*	3'556	4'542	4'162	3'080	5'245	0.768	0.323037223	1
ENSBTAG00000030305	*BT.93739*	1'237	651	911	1'071	752	-0.511	0.539808397	1
ENSBTAG00000030308	*U2*	1	0	0	1	0		1	1
ENSBTAG00000030314	*U2*	0	1	1	0	1	Inf	0.993540919	1
ENSBTAG00000030317	*PPP1R26*	683	677	687	591	782	0.402	0.637530313	1
ENSBTAG00000030319	*GBGT1*	560	546	558	485	630	0.379	0.6653939	1
ENSBTAG00000030322	*KLHDC7A*	2	10	7	2	12	2.737	0.678653301	1
ENSBTAG00000030333	*BT.105258*	4'056	1'930	2'871	3'513	2'229	-0.656	0.402835649	1
ENSBTAG00000030334	*AOC2*	295	178	231	255	206	-0.314	0.76222559	1
ENSBTAG00000030335	*ALDH4A1*	1'262	2'223	1'830	1'093	2'567	1.232	0.125647959	1
ENSBTAG00000030337	*MYCBP*	478	309	385	414	357	-0.214	0.819126671	1
ENSBTAG00000030338	*GJA9*	50	22	34	43	25	-0.769	0.72051121	1
ENSBTAG00000030340	*BT.46227*	459	722	616	398	834	1.069	0.219554678	1
ENSBTAG00000030341	*U1*	4	6	5	3	7	1.000	0.91481797	1
ENSBTAG00000030343	*FGF23*	2	0	1	2	0		0.974934741	1
ENSBTAG00000030347	*TUBG2*	1'402	1'026	1'199	1'214	1'185	-0.035	0.96710094	1
ENSBTAG00000030348	*BT.76574*	904	608	742	783	702	-0.157	0.854669154	1
ENSBTAG00000030360	*BOSTAUV1R430*	1	0	0	1	0		1	1
ENSBTAG00000030366	*BT.89338*	3'185	2'490	2'817	2'758	2'875	0.060	0.938702391	1
ENSBTAG00000030369	*IFFO1*	869	704	783	753	813	0.111	0.895557204	1
ENSBTAG00000030371	*protein_coding*	15	1	7	13	1	-3.492	0.607205542	1
ENSBTAG00000030384	*SURF1*	5'778	6'452	6'227	5'004	7'450	0.574	0.455752856	1
ENSBTAG00000030393	*RDH13*	773	677	726	669	782	0.224	0.792420937	1
ENSBTAG00000030403	*BT.44430*	1'951	2'206	2'118	1'690	2'547	0.592	0.453986092	1
ENSBTAG00000030404	*pseudogene*	3	0	1	3	0		0.936647693	1
ENSBTAG00000030413	*LACTBL1*	61	62	62	53	72	0.438	0.785883263	1
ENSBTAG00000030416	*processed_pseudogene*	1	1	1	1	1	0.415	1	1
ENSBTAG00000030423	*CLEC1B*	2	2	2	2	2	0.415	1	1
ENSBTAG00000030424	*CLEC1A*	455	111	261	394	128	-1.620	0.110053896	1
ENSBTAG00000030425	*ID3*	2'457	1'320	1'826	2'128	1'524	-0.481	0.546026848	1
ENSBTAG00000030426	*protein_coding*	5	0	2	4	0		0.853268594	1
ENSBTAG00000030431	*pseudogene*	4	8	6	3	9	1.415	0.839396723	1
ENSBTAG00000030432	*TTC8*	1'044	578	786	904	667	-0.438	0.604321926	1
ENSBTAG00000030434	*FUCA1*	7'106	4'666	5'771	6'154	5'388	-0.192	0.803951043	1
ENSBTAG00000030435	*PNRC2*	11'577	7'031	9'072	10'026	8'119	-0.304	0.691544103	1
ENSBTAG00000030453	*BT.55792*	3'223	2'205	2'669	2'791	2'546	-0.133	0.866617256	1
ENSBTAG00000030454	*protein_coding*	4	0	2	3	0		0.89545886	1
ENSBTAG00000030456	*BT.38371*	1'101	512	772	953	591	-0.690	0.41552573	1
ENSBTAG00000030470	*protein_coding*	287	402	356	249	464	0.901	0.337067389	1
ENSBTAG00000030474	*BT.63171*	103	66	83	89	76	-0.227	0.879801135	1
ENSBTAG00000030482	*KLK6*	16	10	13	14	12	-0.263	0.967957861	1
ENSBTAG00000030483	*KLK7*	257	111	175	223	128	-0.796	0.469888833	1
ENSBTAG00000030484	*BT.106540*	46	42	44	40	48	0.284	0.886975646	1
ENSBTAG00000030490	*RPL17*	301	305	306	261	352	0.434	0.652440522	1
ENSBTAG00000030493	*RERGL*	17	4	10	15	5	-1.672	0.733083131	1
ENSBTAG00000030499	*DIAPH1*	2'074	2'096	2'108	1'796	2'420	0.430	0.58594476	1
ENSBTAG00000030502	*BT.51104*	7	1	4	6	1	-2.392	0.851750348	1
ENSBTAG00000030503	*H2AFJ*	1'085	857	965	940	990	0.075	0.928528777	1

Ensembl gene ID	geneName	counts wildtype horn bud	counts polled horn bud	baseMean	baseMean wildtype horn bud	baseMean polled horn bud	log2FoldChange	pval	padj
ENSBTAG00000030504	*H4*	7	4	5	6	5	-0.392	0.98714753	1
ENSBTAG00000030505	*BT.82341*	1	1	1	1	1	0.415	1	1
ENSBTAG00000030507	*protein_coding*	68	25	44	59	29	-1.029	0.583192446	1
ENSBTAG00000030517	*protein_coding*	12	4	8	10	5	-1.170	0.853405557	1
ENSBTAG00000030518	*ECSCR*	1'619	551	1'019	1'402	636	-1.140	0.170611218	1
ENSBTAG00000030519	*BT.102521*	0	2	1	0	2	Inf	0.939077559	1
ENSBTAG00000030520	*C5ORF65*	225	279	259	195	322	0.725	0.46722072	1
ENSBTAG00000030521	*IER5L*	466	644	574	404	744	0.882	0.313593016	1
ENSBTAG00000030523	*ZPBP2*	104	62	81	90	72	-0.331	0.822229197	1
ENSBTAG00000030529	*FAM53C*	2'869	3'318	3'158	2'485	3'831	0.625	0.423636141	1
ENSBTAG00000030532	*KNCN*	1	0	0	1	0		1	1
ENSBTAG00000030533	*protein_coding*	1	1	1	1	1	0.415	1	1
ENSBTAG00000030539	*KRT222*	67	7	33	58	8	-2.844	0.210032222	1
ENSBTAG00000030540	*ZDHHC12*	151	184	172	131	212	0.700	0.525825453	1
ENSBTAG00000030542	*KRT25*	2	1	1	2	1	-0.585	1	1
ENSBTAG00000030543	*SLC6A16*	244	121	176	211	140	-0.597	0.588368337	1
ENSBTAG00000030544	*pseudogene*	0	1	1	0	1	Inf	0.993540919	1
ENSBTAG00000030546	*KRT26*	2	0	1	2	0		0.974934741	1
ENSBTAG00000030548	*KRT27*	4	0	2	3	0		0.89545886	1
ENSBTAG00000030553	*protein_coding*	4	7	6	3	8	1.222	0.876605346	1
ENSBTAG00000030555	*KRT28*	3	0	1	3	0		0.936647693	1
ENSBTAG00000030556	*ZNF217*	356	260	304	308	300	-0.038	0.97283702	1
ENSBTAG00000030557	*LIN52*	1'146	801	959	992	925	-0.102	0.904025769	1
ENSBTAG00000030563	*PRRG2*	130	120	126	113	139	0.300	0.80801811	1
ENSBTAG00000030564	*protein_coding*	1	0	0	1	0		1	1
ENSBTAG00000030566	*GLE1*	1'261	1'083	1'171	1'092	1'251	0.196	0.810445304	1
ENSBTAG00000030567	*ODF2*	1'994	1'332	1'632	1'727	1'538	-0.167	0.835666863	1
ENSBTAG00000030575	*BT.74188*	317	211	259	275	244	-0.172	0.866755077	1
ENSBTAG00000030578	*PTOV1*	6'858	8'906	8'111	5'939	10'284	0.792	0.303433387	1
ENSBTAG00000030581	*TBC1D5*	634	504	566	549	582	0.084	0.924621839	1
ENSBTAG00000030584	*SLC35A4*	1'678	2'048	1'909	1'453	2'365	0.703	0.377143097	1
ENSBTAG00000030587	*LASP1*	16'094	16'008	16'211	13'938	18'484	0.407	0.592806152	1
ENSBTAG00000030589	*TMCO6*	1'817	1'476	1'639	1'574	1'704	0.115	0.885335991	1
ENSBTAG00000030591	*BT.103404*	447	410	430	387	473	0.290	0.749525901	1
ENSBTAG00000030592	*UBL5*	2'285	1'649	1'941	1'979	1'904	-0.056	0.945464385	1
ENSBTAG00000030593	*RFTN1*	450	413	433	390	477	0.291	0.748526078	1
ENSBTAG00000030595	*COX16*	1'318	908	1'095	1'141	1'048	-0.123	0.882689459	1
ENSBTAG00000030599	*SMOC1*	1'966	1'619	1'786	1'703	1'869	0.135	0.865254709	1
ENSBTAG00000030600	*P97BCNT2*	1'759	1'462	1'606	1'523	1'688	0.148	0.852880278	1
ENSBTAG00000030608	*SWI5*	2'952	1'974	2'418	2'557	2'279	-0.166	0.834204721	1
ENSBTAG00000030616	*ZCCHC10*	645	360	487	559	416	-0.426	0.633824307	1
ENSBTAG00000030622	*MANSC4*	59	41	49	51	47	-0.110	0.963371241	1
ENSBTAG00000030623	*BT.56900*	4	0	2	3	0		0.89545886	1
ENSBTAG00000030624	*WFDC5*	42	2	19	36	2	-3.977	0.229264678	1
ENSBTAG00000030629	*MGC127133*	3	3	3	3	3	0.415	1	1
ENSBTAG00000030632	*ALG10*	2'109	1'273	1'648	1'826	1'470	-0.313	0.695953445	1
ENSBTAG00000030646	*KCTD17*	386	296	338	334	342	0.032	0.975692435	1
ENSBTAG00000030648	*MPST*	755	838	811	654	968	0.566	0.500920841	1
ENSBTAG00000030650	*TST*	314	360	344	272	416	0.612	0.515912174	1
ENSBTAG00000030652	*MGC133880*	0	1	1	0	1	Inf	0.993540919	1
ENSBTAG00000030667	*CCDC114*	392	218	296	339	252	-0.431	0.658291065	1
ENSBTAG00000030669	*FGGY*	527	284	392	456	328	-0.477	0.606147693	1
ENSBTAG00000030670	*PCNT*	2'253	1'679	1'945	1'951	1'939	-0.009	0.992177979	1
ENSBTAG00000030671	*HOOK1*	196	131	161	170	151	-0.166	0.888100939	1
ENSBTAG00000030672	*pseudogene*	8	31	21	7	36	2.369	0.397763997	1
ENSBTAG00000030674	*AK4*	259	144	195	224	166	-0.432	0.688061617	1
ENSBTAG00000030675	*PDCL*	1'418	1'140	1'272	1'228	1'316	0.100	0.901778266	1
ENSBTAG00000030683	*protein_coding*	2	0	1	2	0		0.974934741	1
ENSBTAG00000030686	*RAVER2*	335	167	241	290	193	-0.589	0.562316029	1
ENSBTAG00000030690	*RADIL*	542	207	354	469	239	-0.974	0.301787762	1
ENSBTAG00000030704	*EEF1A1*	9	6	7	8	7	-0.170	1	1
ENSBTAG00000030705	*DACT3*	873	1'164	1'050	756	1'344	0.830	0.314198526	1
ENSBTAG00000030706	*BT.25021*	1'022	539	754	885	622	-0.508	0.549335456	1
ENSBTAG00000030710	*ITGB3BP*	466	275	361	404	318	-0.346	0.713276835	1
ENSBTAG00000030711	*BT.69052*	5	5	5	4	6	0.415	0.985023983	1
ENSBTAG00000030721	*UNCX*	3	5	4	3	6	1.152	0.920866718	1
ENSBTAG00000030735	*COX7B*	541	841	720	469	971	1.052	0.219008109	1
ENSBTAG00000030744	*NFYB*	1'084	238	607	939	275	-1.772	0.045902336	1
ENSBTAG00000030749	*BT.65042*	936	194	517	811	224	-1.855	0.041002127	1
ENSBTAG00000030757	*ELFN1*	121	173	152	105	200	0.931	0.415412427	1
ENSBTAG00000030764	*5S_rRNA*	1	0	0	1	0		1	1
ENSBTAG00000030769	*KPTN*	950	1'017	999	823	1'174	0.513	0.533802114	1

Ensembl gene ID	geneName	counts wildtype horn bud	counts polled horn bud	baseMean	baseMean wildtype horn bud	baseMean polled horn bud	log2FoldChange	pval	padj
ENSBTAG00000030775	*PCBP3*	119	191	162	103	221	1.098	0.329363468	1
ENSBTAG00000030784	*APPL2*	3'989	2'611	3'235	3'455	3'015	-0.196	0.801820355	1
ENSBTAG00000030792	*protein_coding*	28	1	13	24	1	-4.392	0.334236041	1
ENSBTAG00000030801	*MNAT1*	927	536	711	803	619	-0.375	0.660446071	1
ENSBTAG00000030805	*SHISA4*	664	815	758	575	941	0.711	0.401341073	1
ENSBTAG00000030809	*BT.50351*	2	0	1	2	0		0.974934741	1
ENSBTAG00000030814	*TFF2*	1	0	0	1	0		1	1
ENSBTAG00000030817	*LMBR1*	1'506	1'093	1'283	1'304	1'262	-0.047	0.955066795	1
ENSBTAG00000030820	*NT5M*	1'025	878	951	888	1'014	0.192	0.817056835	1
ENSBTAG00000030824	*C10H14orf149*	339	110	210	294	127	-1.209	0.252168722	1
ENSBTAG00000030834	*protein_coding*	3	8	6	3	9	1.830	0.801688691	1
ENSBTAG00000030836	*ZNF295*	326	245	283	282	283	0.003	1	1
ENSBTAG00000030838	*pseudogene*	4	2	3	3	2	-0.585	0.996239345	1
ENSBTAG00000030839	*MUM1*	3'397	2'867	3'126	2'942	3'311	0.170	0.826655461	1
ENSBTAG00000030845	*pseudogene*	1'031	483	725	893	558	-0.679	0.425643919	1
ENSBTAG00000030847	*GLYATL2*	5	0	2	4	0		0.853268594	1
ENSBTAG00000030852	*MGC137454*	1	0	0	1	0		1	1
ENSBTAG00000030857	*5S_rRNA*	13	21	18	11	24	1.107	0.722840811	1
ENSBTAG00000030862	*protein_coding*	0	2	1	0	2	Inf	0.939077559	1
ENSBTAG00000030864	*FAM228B*	186	46	107	161	53	-1.601	0.217779623	1
ENSBTAG00000030876	*MNX1*	1	1	1	1	1	0.415	1	1
ENSBTAG00000030881	*C10H14ORF101*	2'086	1'095	1'535	1'807	1'264	-0.515	0.52213765	1
ENSBTAG00000030882	*protein_coding*	117	47	78	101	54	-0.901	0.534510121	1
ENSBTAG00000030885	*SSC5D*	12'654	19'226	16'579	10'959	22'200	1.019	0.184971195	1
ENSBTAG00000030890	*protein_coding*	21	53	40	18	61	1.751	0.372958201	1
ENSBTAG00000030892	*pseudogene*	4	4	4	3	5	0.415	0.994759363	1
ENSBTAG00000030897	*XRCC2*	211	162	185	183	187	0.034	0.979903474	1
ENSBTAG00000030898	*BT.53756*	14	7	10	12	8	-0.585	0.916699259	1
ENSBTAG00000030908	*OR9Q2*	1	0	0	1	0		1	1
ENSBTAG00000030910	*CD46*	3	0	1	3	0		0.936647693	1
ENSBTAG00000030913	*MX1*	1'020	246	584	883	284	-1.637	0.065433798	1
ENSBTAG00000030915	*BT.41862*	1'570	1'509	1'551	1'360	1'742	0.358	0.654994189	1
ENSBTAG00000030920	*BT.55275*	7	2	4	6	2	-1.392	0.903936064	1
ENSBTAG00000030921	*FAM3B*	4	3	3	3	3	0.000	1	1
ENSBTAG00000030922	*BT.56248*	4	4	4	3	5	0.415	0.994759363	1
ENSBTAG00000030927	*protein_coding*	14	10	12	12	12	-0.070	1	1
ENSBTAG00000030929	*ZNF558*	214	185	199	185	214	0.205	0.849444922	1
ENSBTAG00000030930	*BT.62601*	1'237	1'415	1'353	1'071	1'634	0.609	0.45142547	1
ENSBTAG00000030932	*IFI44L*	170	34	93	147	39	-1.907	0.165878045	1
ENSBTAG00000030933	*ZNF576*	1'023	497	730	886	574	-0.626	0.461851152	1
ENSBTAG00000030939	*ZNF575*	517	558	546	448	644	0.525	0.549546966	1
ENSBTAG00000030940	*GIMAP7*	28	15	21	24	17	-0.485	0.875876678	1
ENSBTAG00000030941	*protein_coding*	4	40	25	3	46	3.737	0.172659135	1
ENSBTAG00000030942	*MRPL3*	2'488	2'102	2'291	2'155	2'427	0.172	0.826976192	1
ENSBTAG00000030951	*ZNF655*	309	166	230	268	192	-0.481	0.640502862	1
ENSBTAG00000030956	*ZSCAN21*	2'791	1'939	2'328	2'417	2'239	-0.110	0.889536079	1
ENSBTAG00000030960	*DNAJC25*	746	326	511	646	376	-0.779	0.380654686	1
ENSBTAG00000030962	*EEFSEC*	843	1'271	1'099	730	1'468	1.007	0.221716038	1
ENSBTAG00000030965	*MCM7*	4'744	6'904	6'040	4'108	7'972	0.956	0.216954197	1
ENSBTAG00000030966	*TAF6*	2'824	3'449	3'214	2'446	3'983	0.703	0.367911691	1
ENSBTAG00000030974	*TUBA4A*	1'380	1'568	1'503	1'195	1'811	0.599	0.455917372	1
ENSBTAG00000030977	*ULK2*	3'840	2'134	2'895	3'326	2'464	-0.433	0.580926239	1
ENSBTAG00000030979	*BT.36464*	70	95	85	61	110	0.856	0.538498731	1
ENSBTAG00000030990	*BT.61969*	1'353	953	1'136	1'172	1'100	-0.091	0.913405681	1
ENSBTAG00000030993	*LCA5L*	3	0	1	3	0		0.936647693	1
ENSBTAG00000030994	*BT.43548*	253	43	134	219	50	-2.142	0.080704201	1
ENSBTAG00000030999	*DUSP8*	294	376	344	255	434	0.770	0.414249757	1
ENSBTAG00000031001	*MEGF8*	3'251	5'864	4'793	2'815	6'771	1.266	0.105614463	1
ENSBTAG00000031004	*CNFN*	496	109	278	430	126	-1.771	0.078114669	1
ENSBTAG00000031010	*ACAD11*	706	670	693	611	774	0.340	0.690679561	1
ENSBTAG00000031012	*PIGK*	1'743	1'320	1'517	1'509	1'524	0.014	0.986316798	1
ENSBTAG00000031014	*C2ORF43*	220	242	235	191	279	0.553	0.587805321	1
ENSBTAG00000031017	*protein_coding*	1	0	0	1	0		1	1
ENSBTAG00000031018	*BT.81092*	35	33	34	30	38	0.330	0.885279245	1
ENSBTAG00000031020	*pseudogene*	30	22	26	26	25	-0.032	1	1
ENSBTAG00000031041	*NHEJ1*	484	583	546	419	673	0.684	0.436457319	1
ENSBTAG00000031044	*FAM73A*	404	251	320	350	290	-0.272	0.77871469	1
ENSBTAG00000031052	*CDK5R2*	33	0	14	29	0		0.17323257	1
ENSBTAG00000031059	*BT.34530*	180	97	134	156	112	-0.477	0.691200198	1
ENSBTAG00000031061	*POLR2L*	1'253	992	1'115	1'085	1'145	0.078	0.924307943	1
ENSBTAG00000031065	*protein_coding*	1	1	1	1	1	0.415	1	1
ENSBTAG00000031069	*SNX24*	1'148	1'099	1'132	994	1'269	0.352	0.666389626	1

Ensembl gene ID	geneName	counts wildtype horn bud	counts polled horn bud	baseMean	baseMean wildtype horn bud	baseMean polled horn bud	log2FoldChange	pval	padj
ENSBTAG00000031071	*ZNF624*	1'591	623	1'049	1'378	719	-0.938	0.257099886	1
ENSBTAG00000031076	*processed_pseudogene*	97	208	162	84	240	1.516	0.181425798	1
ENSBTAG00000031082	*protein_coding*	37'640	35'975	37'069	32'597	41'540	0.350	0.64481327	1
ENSBTAG00000031084	*BT.51392*	26	28	27	23	32	0.522	0.834125412	1
ENSBTAG00000031088	*protein_coding*	378	386	387	327	446	0.445	0.629750998	1
ENSBTAG00000031106	*BT.40980*	384	440	420	333	508	0.611	0.502170178	1
ENSBTAG00000031107	*HS3ST3A1*	22	17	19	19	20	0.043	1	1
ENSBTAG00000031115	*protein_coding*	4	21	14	3	24	2.807	0.454483345	1
ENSBTAG00000031134	*BT.104932*	3'616	2'716	3'134	3'132	3'136	0.002	0.997618804	1
ENSBTAG00000031135	*CTXN3*	37	18	26	32	21	-0.624	0.806604612	1
ENSBTAG00000031145	*RS1*	1	1	1	1	1	0.415	1	1
ENSBTAG00000031146	*WIBG*	863	864	873	747	998	0.417	0.617319707	1
ENSBTAG00000031150	*C11H2orf70*	1	0	0	1	0		1	1
ENSBTAG00000031160	*BT.29726*	4	0	2	3	0		0.89545886	1
ENSBTAG00000031165	*TRPM7*	3'970	1'802	2'759	3'438	2'081	-0.725	0.356733984	1
ENSBTAG00000031166	*ZDBF2*	11	0	5	10	0		0.617662983	1
ENSBTAG00000031171	*BT.91312*	1'304	963	1'121	1'129	1'112	-0.022	0.980176797	1
ENSBTAG00000031178	*BT.87114*	1'607	1'363	1'483	1'392	1'574	0.177	0.825056604	1
ENSBTAG00000031184	*CDKN1C*	3'718	2'166	2'860	3'220	2'501	-0.364	0.641938912	1
ENSBTAG00000031185	*C2ORF28*	1'406	903	1'130	1'218	1'043	-0.224	0.785545657	1
ENSBTAG00000031186	*GBP6*	2	0	1	2	0		0.974934741	1
ENSBTAG00000031188	*ADIG*	1	0	0	1	0		1	1
ENSBTAG00000031194	*PHLDA2*	7	2	4	6	2	-1.392	0.903936064	1
ENSBTAG00000031197	*HMX1*	2	50	30	2	58	5.059	0.062024634	1
ENSBTAG00000031198	*BEND2*	1	0	0	1	0		1	1
ENSBTAG00000031205	*RPS4X*	9'419	15'177	12'841	8'157	17'525	1.103	0.152331719	1
ENSBTAG00000031209	*bta-mir-2457*	66	35	49	57	40	-0.500	0.78416675	1
ENSBTAG00000031210	*MYPOP*	307	329	323	266	380	0.515	0.589198436	1
ENSBTAG00000031214	*protein_coding*	14	9	11	12	10	-0.222	0.982048045	1
ENSBTAG00000031217	*MYL6B*	532	390	456	461	450	-0.033	0.974269283	1
ENSBTAG00000031231	*IRF1*	686	378	515	594	436	-0.445	0.616311398	1
ENSBTAG00000031232	*BT.29750*	0	1	1	0	1	Inf	0.993540919	1
ENSBTAG00000031234	*BT.89203*	1	2	2	1	2	1.415	0.981979269	1
ENSBTAG00000031235	*IL5*	5	1	3	4	1	-1.907	0.9188325	1
ENSBTAG00000031238	*BT.51827*	38	12	23	33	14	-1.248	0.637252626	1
ENSBTAG00000031242	*CDCA4*	218	288	261	189	333	0.817	0.412458982	1
ENSBTAG00000031246	*BT.54629*	234	164	196	203	189	-0.098	0.932418039	1
ENSBTAG00000031249	*PSMG4*	2'993	2'972	3'012	2'592	3'432	0.405	0.603757712	1
ENSBTAG00000031250	*pseudogene*	1	0	0	1	0		1	1
ENSBTAG00000031252	*CD82*	2'232	2'203	2'238	1'933	2'544	0.396	0.615049154	1
ENSBTAG00000031256	*protein_coding*	1	49	29	1	57	6.030	0.043859303	1
ENSBTAG00000031261	*REPS2*	136	72	100	118	83	-0.503	0.704790475	1
ENSBTAG00000031265	*SVOPL*	12	6	9	10	7	-0.585	0.929512138	1
ENSBTAG00000031267	*SERPINB9*	81	23	48	70	27	-1.401	0.43365345	1
ENSBTAG00000031279	*BT.30373*	2'956	2'739	2'861	2'560	3'163	0.305	0.695982914	1
ENSBTAG00000031287	*TMEM158*	1'094	821	948	947	948	0.001	0.999956375	1
ENSBTAG00000031295	*HSDL2*	1'443	678	1'016	1'250	783	-0.675	0.414515546	1
ENSBTAG00000031299	*TTC17*	1'967	1'838	1'913	1'703	2'122	0.317	0.688999453	1
ENSBTAG00000031301	*pseudogene*	1	0	0	1	0		1	1
ENSBTAG00000031306	*PLSCR1*	2	0	1	2	0		0.974934741	1
ENSBTAG00000031309	*RAG2*	9	4	6	8	5	-0.755	0.931866093	1
ENSBTAG00000031327	*BT.88936*	623	875	775	540	1'010	0.905	0.285358191	1
ENSBTAG00000031332	*RBKS*	259	145	196	224	167	-0.422	0.694792675	1
ENSBTAG00000031335	*BRE*	1'001	963	989	867	1'112	0.359	0.663313482	1
ENSBTAG00000031340	*SLC6A20*	60	59	60	52	68	0.391	0.812939707	1
ENSBTAG00000031346	*ANKUB1*	10	6	8	9	7	-0.322	0.979718036	1
ENSBTAG00000031347	*SPDYA*	243	44	131	210	51	-2.050	0.096357108	1
ENSBTAG00000031348	*BT.25697*	3	0	1	3	0		0.936647693	1
ENSBTAG00000031351	*BT.62216*	33	60	49	29	69	1.278	0.468794487	1
ENSBTAG00000031352	*ZNF554*	157	83	116	136	96	-0.505	0.688655587	1
ENSBTAG00000031354	*protein_coding*	22	20	21	19	23	0.278	0.935151797	1
ENSBTAG00000031355	*BT.101019*	22	8	14	19	9	-1.044	0.780532613	1
ENSBTAG00000031358	*BT.66173*	899	549	706	779	634	-0.296	0.729150744	1
ENSBTAG00000031359	*protein_coding*	103	20	56	89	23	-1.950	0.249354316	1
ENSBTAG00000031361	*BPIFB5*	2	2	2	2	2	0.415	1	1
ENSBTAG00000031362	*FAM55B*	1	1	1	1	1	0.415	1	1
ENSBTAG00000031363	*AES*	7'905	8'043	8'067	6'846	9'287	0.440	0.565784957	1
ENSBTAG00000031364	*NAT8L*	19	19	19	16	22	0.415	0.900529004	1
ENSBTAG00000031376	*BSP30C*	66	1	29	57	1	-5.629	0.0572057	1
ENSBTAG00000031377	*SYCP2L*	6	2	4	5	2	-1.170	0.934316631	1
ENSBTAG00000031383	*C19ORF77*	16	22	20	14	25	0.874	0.767703817	1
ENSBTAG00000031385	*RFWD2*	3'783	2'005	2'796	3'276	2'315	-0.501	0.523142417	1

Ensembl gene ID	geneName	counts wildtype horn bud	counts polled horn bud	baseMean	baseMean wildtype horn bud	baseMean polled horn bud	log2FoldChange	pval	padj
ENSBTAG00000031387	*FZR1*	1'530	2'416	2'057	1'325	2'790	1.074	0.178154615	1
ENSBTAG00000031388	*DYX1C1-CCPG1*	170	64	111	147	74	-0.994	0.434250986	1
ENSBTAG00000031395	*C4orf48*	104	71	86	90	82	-0.136	0.931130381	1
ENSBTAG00000031396	*protein_coding*	193	152	171	167	176	0.071	0.953807481	1
ENSBTAG00000031397	*P2RY13*	165	60	106	143	69	-1.044	0.41833261	1
ENSBTAG00000031402	*ASXL1*	3'188	4'699	4'093	2'761	5'426	0.975	0.211513174	1
ENSBTAG00000031430	*CD83*	278	115	187	241	133	-0.858	0.428152535	1
ENSBTAG00000031432	*LYRM4*	458	539	510	397	622	0.650	0.463272979	1
ENSBTAG00000031433	*BT.65767*	131	67	95	113	77	-0.552	0.682265796	1
ENSBTAG00000031435	*SELT*	3'115	1'684	2'321	2'698	1'945	-0.472	0.549613367	1
ENSBTAG00000031439	*CDC14A*	179	83	125	155	96	-0.694	0.569503251	1
ENSBTAG00000031441	*FXYD5*	1'920	1'644	1'781	1'663	1'898	0.191	0.809968468	1
ENSBTAG00000031444	*LY86*	93	74	83	81	85	0.085	0.959760885	1
ENSBTAG00000031447	*BT.62588*	2	0	1	2	0		0.974934741	1
ENSBTAG00000031453	*C20ORF160*	84	60	71	73	69	-0.070	0.973782068	1
ENSBTAG00000031454	*BT.88497*	39	20	28	34	23	-0.548	0.823982265	1
ENSBTAG00000031458	*pseudogene*	606	578	596	525	667	0.347	0.689540914	1
ENSBTAG00000031461	*SNX7*	1'888	691	1'216	1'635	798	-1.035	0.206870651	1
ENSBTAG00000031462	*FAM209B*	5	4	4	4	5	0.093	1	1
ENSBTAG00000031468	*HIST3H2A*	0	1	1	0	1	Inf	0.993540919	1
ENSBTAG00000031473	*BT.64115*	2'188	1'093	1'578	1'895	1'262	-0.586	0.465509318	1
ENSBTAG00000031476	*DLL1*	879	537	691	761	620	-0.296	0.730295733	1
ENSBTAG00000031481	*TTLL2*	1	0	0	1	0		1	1
ENSBTAG00000031483	*processed_pseudogene*	2'313	3'084	2'782	2'003	3'561	0.830	0.29091364	1
ENSBTAG00000031497	*FGFBP1*	166	195	184	144	225	0.647	0.549813957	1
ENSBTAG00000031500	*SHMT2*	3'022	2'546	2'778	2'617	2'940	0.168	0.829823815	1
ENSBTAG00000031503	*NDUFA4L2*	845	197	480	732	227	-1.686	0.064778444	1
ENSBTAG00000031509	*PFAS*	703	862	802	609	995	0.709	0.399749489	1
ENSBTAG00000031519	*NGP*	1	0	0	1	0		1	1
ENSBTAG00000031523	*BT.88844*	129	89	107	112	103	-0.120	0.933013677	1
ENSBTAG00000031524	*BT.102954*	769	540	645	666	624	-0.095	0.914448108	1
ENSBTAG00000031532	*DACT2*	104	149	131	90	172	0.934	0.435063508	1
ENSBTAG00000031538	*protein_coding*	1'305	2'540	2'032	1'130	2'933	1.376	0.086805054	1
ENSBTAG00000031544	*DDIT3*	1'417	689	1'011	1'227	796	-0.625	0.449567721	1
ENSBTAG00000031548	*BT.89244*	274	52	149	237	60	-1.983	0.093682713	1
ENSBTAG00000031551	*BT.45454*	719	403	544	623	465	-0.420	0.633582149	1
ENSBTAG00000031553	*MFSD7*	920	415	638	797	479	-0.733	0.396150987	1
ENSBTAG00000031558	*protein_coding*	8	32	22	7	37	2.415	0.381922776	1
ENSBTAG00000031561	*RSRC1*	1'720	927	1'280	1'490	1'070	-0.477	0.557544391	1
ENSBTAG00000031564	*GNL3L*	5'839	3'800	4'722	5'057	4'388	-0.205	0.791769849	1
ENSBTAG00000031567	*SMARCC1*	2'483	4'065	3'422	2'150	4'694	1.126	0.151720537	1
ENSBTAG00000031569	*GOLM1*	3'782	3'996	3'945	3'275	4'614	0.494	0.523729535	1
ENSBTAG00000031572	*IFT80*	1'135	599	837	983	692	-0.507	0.546057114	1
ENSBTAG00000031573	*ITGB1BP3*	8	76	47	7	88	3.663	0.061106254	1
ENSBTAG00000031575	*VAV3*	693	427	547	600	493	-0.284	0.74827479	1
ENSBTAG00000031579	*SGOL2*	1'436	607	972	1'244	701	-0.827	0.319475754	1
ENSBTAG00000031582	*protein_coding*	3	2	2	3	2	-0.170	1	1
ENSBTAG00000031583	*BT.29464*	79	52	64	68	60	-0.188	0.914045228	1
ENSBTAG00000031590	*SSH3*	2'357	1'925	2'132	2'041	2'223	0.123	0.876055948	1
ENSBTAG00000031593	*CATHL1*	6	1	3	5	1	-2.170	0.885240772	1
ENSBTAG00000031595	*BT.55206*	33	7	18	29	8	-1.822	0.554890505	1
ENSBTAG00000031598	*GBAS*	3'575	2'454	2'965	3'096	2'834	-0.128	0.871007022	1
ENSBTAG00000031599	*CATHL3*	3	0	1	3	0		0.936647693	1
ENSBTAG00000031600	*processed_pseudogene*	137	297	231	119	343	1.531	0.139776196	1
ENSBTAG00000031609	*PRKRIR*	1'116	1'202	1'177	966	1'388	0.522	0.521868441	1
ENSBTAG00000031612	*SPINK8*	1	2	2	1	2	1.415	0.981979269	1
ENSBTAG00000031614	*BT.25777*	15	0	6	13	0		0.491308095	1
ENSBTAG00000031618	*WDR49*	1	0	0	1	0		1	1
ENSBTAG00000031625	*HRH3*	46	17	30	40	20	-1.021	0.658132137	1
ENSBTAG00000031631	*FAM132A*	112	141	130	97	163	0.747	0.533206401	1
ENSBTAG00000031632	*ADRA1A*	6	1	3	5	1	-2.170	0.885240772	1
ENSBTAG00000031641	*BT.91216*	4'296	4'994	4'744	3'720	5'767	0.632	0.413910639	1
ENSBTAG00000031647	*LRG1*	16	4	9	14	5	-1.585	0.756102726	1
ENSBTAG00000031648	*C19ORF33*	713	246	451	617	284	-1.120	0.217790538	1
ENSBTAG00000031654	*KCND3*	25	13	18	22	15	-0.528	0.875356394	1
ENSBTAG00000031655	*pseudogene*	4	2	3	3	2	-0.585	0.996239345	1
ENSBTAG00000031656	*TAF13*	2'353	1'181	1'701	2'038	1'364	-0.579	0.469023765	1
ENSBTAG00000031658	*SEMA6B*	323	332	332	280	383	0.455	0.631885681	1
ENSBTAG00000031673	*BT.50759*	31	12	20	27	14	-0.954	0.743935879	1
ENSBTAG00000031679	*HSD17B13*	35	4	17	30	5	-2.714	0.407253132	1
ENSBTAG00000031682	*ZNF484*	2'418	999	1'624	2'094	1'154	-0.860	0.28485691	1
ENSBTAG00000031686	*ZNF566*	282	146	206	244	169	-0.535	0.613040778	1

Ensembl gene ID	geneName	counts wildtype horn bud	counts polled horn bud	baseMean	baseMean wildtype horn bud	baseMean polled horn bud	log2FoldChange	pval	padj
ENSBTAG00000031687	*protein_coding*	2'043	1'590	1'803	1'769	1'836	0.053	0.946507839	1
ENSBTAG00000031688	*RBM4*	5'381	4'917	5'169	4'660	5'678	0.285	0.71125562	1
ENSBTAG00000031693	*BT.98313*	18	29	25	16	33	1.103	0.664570586	1
ENSBTAG00000031696	*ANKRD44*	189	83	130	164	96	-0.772	0.521796358	1
ENSBTAG00000031697	*KLHDC1*	1'045	555	773	905	641	-0.498	0.556318139	1
ENSBTAG00000031701	*TAGAP*	91	33	58	79	38	-1.048	0.521813507	1
ENSBTAG00000031707	*FRMD6*	8'029	6'006	6'944	6'953	6'935	-0.004	0.996973906	1
ENSBTAG00000031709	*PDCD10*	2'636	1'476	1'994	2'283	1'704	-0.422	0.59546254	1
ENSBTAG00000031711	*protein_coding*	452	792	653	391	915	1.224	0.15806895	1
ENSBTAG00000031715	*PCMTD2*	3'288	1'751	2'435	2'847	2'022	-0.494	0.530751784	1
ENSBTAG00000031716	*C20ORF20*	800	627	708	693	724	0.064	0.941476895	1
ENSBTAG00000031717	*protein_coding*	4	0	2	3	0		0.89545886	1
ENSBTAG00000031718	*OGFR*	1'165	1'396	1'310	1'009	1'612	0.676	0.404460213	1
ENSBTAG00000031719	*H4*	2	3	3	2	3	1.000	0.97171833	1
ENSBTAG00000031724	*MAS1*	6	1	3	5	1	-2.170	0.885240772	1
ENSBTAG00000031725	*TNFSF12*	677	651	669	586	752	0.359	0.675469581	1
ENSBTAG00000031731	*HIGD1D*	144	95	117	125	110	-0.185	0.888177973	1
ENSBTAG00000031737	*TMEM102*	136	217	184	118	251	1.089	0.316011692	1
ENSBTAG00000031738	*HIST1H2AB*	7	4	5	6	5	-0.392	0.98714753	1
ENSBTAG00000031741	*MGC157324*	349	478	427	302	552	0.869	0.340238359	1
ENSBTAG00000031746	*pseudogene*	566	838	729	490	968	0.981	0.250215029	1
ENSBTAG00000031747	*HMGN4*	1'815	1'031	1'381	1'572	1'190	-0.401	0.620278566	1
ENSBTAG00000031749	*SLC35E2B*	768	834	814	665	963	0.534	0.524844711	1
ENSBTAG00000031752	*C19H17orf61*	2'193	1'390	1'752	1'899	1'605	-0.243	0.761550591	1
ENSBTAG00000031756	*H4*	28	10	18	24	12	-1.070	0.735005286	1
ENSBTAG00000031757	*H2B*	9	7	8	8	8	0.052	1	1
ENSBTAG00000031758	*HIST1H2AG*	8	3	5	7	3	-1.000	0.918861955	1
ENSBTAG00000031759	*protein_coding*	2	1	1	2	1	-0.585	1	1
ENSBTAG00000031760	*H2B*	19	7	12	16	8	-1.026	0.806704154	1
ENSBTAG00000031761	*protein_coding*	4	4	4	3	5	0.415	0.994759363	1
ENSBTAG00000031762	*H4*	4	3	3	3	3	0.000	1	1
ENSBTAG00000031763	*H4*	3	3	3	3	3	0.415	1	1
ENSBTAG00000031764	*pseudogene*	230	293	269	199	338	0.764	0.439968807	1
ENSBTAG00000031765	*C16ORF93*	284	215	247	246	248	0.013	0.993226092	1
ENSBTAG00000031766	*protein_coding*	13	5	9	11	6	-0.963	0.868297704	1
ENSBTAG00000031767	*pseudogene*	9	3	6	8	3	-1.170	0.890813994	1
ENSBTAG00000031768	*HIST1H2AC*	15	3	8	13	3	-1.907	0.733459861	1
ENSBTAG00000031769	*H2B*	23	8	15	20	9	-1.109	0.761253883	1
ENSBTAG00000031770	*H2B*	3	2	2	3	2	-0.170	1	1
ENSBTAG00000031771	*HIST1H2AG*	18	7	12	16	8	-0.948	0.827785041	1
ENSBTAG00000031773	*HIST1H3A*	13	4	8	11	5	-1.285	0.828302954	1
ENSBTAG00000031776	*H2B*	4	6	5	3	7	1.000	0.91481797	1
ENSBTAG00000031777	*GDPD5*	972	557	742	842	643	-0.388	0.647960482	1
ENSBTAG00000031778	*HIST1H2BI*	263	116	181	228	134	-0.766	0.483269356	1
ENSBTAG00000031780	*HIST1H2BB*	3	0	1	3	0		0.936647693	1
ENSBTAG00000031786	*BT.37203*	25'803	15'844	20'321	22'346	18'295	-0.289	0.705283116	1
ENSBTAG00000031788	*GSTM1*	128	161	148	111	186	0.746	0.516880074	1
ENSBTAG00000031789	*FBRS*	2'139	2'237	2'218	1'852	2'583	0.480	0.543042612	1
ENSBTAG00000031792	*ZNRD1*	1'481	758	1'079	1'283	875	-0.551	0.502738845	1
ENSBTAG00000031793	*NUDT2*	839	646	736	727	746	0.038	0.965353085	1
ENSBTAG00000031794	*PSPN*	7	1	4	6	1	-2.392	0.851750348	1
ENSBTAG00000031795	*BT.102615*	783	1'567	1'244	678	1'809	1.416	0.085992152	1
ENSBTAG00000031797	*MANF*	3'153	2'547	2'836	2'731	2'941	0.107	0.890709545	1
ENSBTAG00000031800	*BT.53925*	5'469	3'850	4'591	4'736	4'446	-0.091	0.906759289	1
ENSBTAG00000031802	*SPATA16*	13	8	10	11	9	-0.285	0.973425765	1
ENSBTAG00000031806	*BT.21907*	497	797	675	430	920	1.096	0.203352199	1
ENSBTAG00000031809	*DENND1C*	347	278	311	301	321	0.095	0.92348711	1
ENSBTAG00000031810	*WDR88*	19	3	10	16	3	-2.248	0.640043503	1
ENSBTAG00000031814	*SDS*	16	5	10	14	6	-1.263	0.797689913	1
ENSBTAG00000031824	*BT.27424*	1'139	1'718	1'485	986	1'984	1.008	0.212805523	1
ENSBTAG00000031828	*BT.51566*	840	645	736	727	745	0.034	0.969090118	1
ENSBTAG00000031829	*FTSJ1*	4'100	2'515	3'227	3'551	2'904	-0.290	0.71049497	1
ENSBTAG00000031834	*protein_coding*	221	390	321	191	450	1.234	0.199429069	1
ENSBTAG00000031837	*IMPDH2*	4'635	4'762	4'756	4'014	5'499	0.454	0.556550777	1
ENSBTAG00000031843	*protein_coding*	0	1	1	0	1	Inf	0.993540919	1
ENSBTAG00000031845	*BT.43709*	22	6	13	19	7	-1.459	0.70932461	1
ENSBTAG00000031846	*BT.22766*	2'028	2'126	2'106	1'756	2'455	0.483	0.540977464	1
ENSBTAG00000031849	*BT.20234*	11'560	9'084	10'250	10'011	10'489	0.067	0.929268534	1
ENSBTAG00000031851	*SELPLG*	210	139	171	182	161	-0.180	0.875551076	1
ENSBTAG00000031852	*TBC1D10B*	1'730	2'688	2'301	1'498	3'104	1.051	0.185680503	1
ENSBTAG00000031861	*BT.106570*	1'254	1'143	1'203	1'086	1'320	0.281	0.729390048	1
ENSBTAG00000031863	*HIG2*	37	35	36	32	40	0.335	0.879194107	1

Ensembl gene ID	geneName	counts wildtype horn bud	counts polled horn bud	baseMean	baseMean wildtype horn bud	baseMean polled horn bud	log2FoldChange	pval	padj
ENSBTAG00000031866	*protein_coding*	6	9	8	5	10	1.000	0.869259965	1
ENSBTAG00000031869	*BT.21072*	1'338	716	993	1'159	827	-0.487	0.556289843	1
ENSBTAG00000031871	*NKAPL*	763	267	485	661	308	-1.100	0.221314423	1
ENSBTAG00000031873	*ZSCAN16*	446	231	326	386	267	-0.534	0.576029748	1
ENSBTAG00000031874	*ZNF165*	153	97	122	133	112	-0.242	0.848732372	1
ENSBTAG00000031875	*BANF1*	6'387	6'767	6'673	5'531	7'814	0.498	0.516604761	1
ENSBTAG00000031885	*TMEM251*	708	490	589	613	566	-0.116	0.896319821	1
ENSBTAG00000031886	*H2B*	682	447	553	591	516	-0.194	0.826449854	1
ENSBTAG00000031888	*HIST1H2AJ*	5	6	6	4	7	0.678	0.946418607	1
ENSBTAG00000031889	*H2B*	11	9	10	10	10	0.126	1	1
ENSBTAG00000031890	*DOCK1*	4'674	4'010	4'339	4'048	4'630	0.194	0.801498365	1
ENSBTAG00000031891	*NLGN1*	118	22	64	102	25	-2.008	0.210603088	1
ENSBTAG00000031895	*BT.103496*	1'474	634	1'004	1'277	732	-0.802	0.333007892	1
ENSBTAG00000031898	*CNTNAP5*	5	0	2	4	0		0.853268594	1
ENSBTAG00000031905	*IFITM3*	0	1	1	0	1	Inf	0.993540919	1
ENSBTAG00000031906	*C13H20ORF135*	52	66	61	45	76	0.759	0.636035486	1
ENSBTAG00000031913	*processed_pseudogene*	1'133	1'239	1'206	981	1'431	0.544	0.503926288	1
ENSBTAG00000031916	*BT.88914*	194	168	181	168	194	0.207	0.851734278	1
ENSBTAG00000031917	*SCAF8*	3'361	2'629	2'973	2'911	3'036	0.061	0.937793506	1
ENSBTAG00000031919	*SLC35E3*	2'357	1'267	1'752	2'041	1'463	-0.480	0.547493466	1
ENSBTAG00000031933	*ALOX12E*	1'205	806	987	1'044	931	-0.165	0.843036013	1
ENSBTAG00000031940	*RSBN1*	363	224	287	314	259	-0.281	0.775860412	1
ENSBTAG00000031941	*BT.53798*	2'719	3'052	2'939	2'355	3'524	0.582	0.456981025	1
ENSBTAG00000031950	*RAB3IP*	611	486	545	529	561	0.085	0.924274698	1
ENSBTAG00000031955	*GPR26*	8	0	3	7	0		0.72953145	1
ENSBTAG00000031962	*RAB20*	45	64	56	39	74	0.923	0.576178879	1
ENSBTAG00000031965	*BT.90060*	4	0	2	3	0		0.89545886	1
ENSBTAG00000031967	*AMPH*	607	1'022	853	526	1'180	1.167	0.166727233	1
ENSBTAG00000031981	*BT.22674*	1'757	766	1'203	1'522	885	-0.783	0.338509176	1
ENSBTAG00000031993	*C12ORF34*	34	33	34	29	38	0.372	0.869906503	1
ENSBTAG00000031998	*CXCL16*	1'351	998	1'161	1'170	1'152	-0.022	0.980493264	1
ENSBTAG00000032003	*ZCCHC13*	12	4	8	10	5	-1.170	0.853405557	1
ENSBTAG00000032007	*LRP11*	376	353	367	326	408	0.324	0.728601483	1
ENSBTAG00000032017	*BT.45028*	308	538	444	267	621	1.220	0.18066798	1
ENSBTAG00000032018	*ZNF19*	826	449	617	715	518	-0.464	0.592362079	1
ENSBTAG00000032021	*RALB*	4'964	6'043	5'638	4'299	6'978	0.699	0.365522572	1
ENSBTAG00000032024	*BT.41985*	2'669	2'121	2'380	2'311	2'449	0.083	0.915244472	1
ENSBTAG00000032026	*BT.74381*	1'149	1'852	1'567	995	2'139	1.104	0.172053675	1
ENSBTAG00000032031	*ZBTB12*	796	875	850	689	1'010	0.552	0.509681128	1
ENSBTAG00000032034	*DYNLL1*	1'426	2'159	1'864	1'235	2'493	1.013	0.205322975	1
ENSBTAG00000032038	*processed_pseudogene*	1'702	2'266	2'045	1'474	2'617	0.828	0.297386197	1
ENSBTAG00000032047	*BT.22184*	327	151	229	283	174	-0.700	0.496676362	1
ENSBTAG00000032051	*BT.76987*	112	174	149	97	201	1.051	0.361672619	1
ENSBTAG00000032055	*VPS39*	1'467	1'341	1'409	1'270	1'548	0.285	0.722880317	1
ENSBTAG00000032057	*protein_coding*	2'613	4'722	3'858	2'263	5'452	1.269	0.106415847	1
ENSBTAG00000032059	*BT.56855*	24	12	17	21	14	-0.585	0.865660962	1
ENSBTAG00000032068	*PLA2G4F*	21	13	17	18	15	-0.277	0.951008818	1
ENSBTAG00000032071	*SYCP1*	20	1	9	17	1	-3.907	0.484363729	1
ENSBTAG00000032074	*U1*	1	0	0	1	0		1	1
ENSBTAG00000032077	*KCND2*	35	4	17	30	5	-2.714	0.407253132	1
ENSBTAG00000032079	*SYT9*	176	136	155	152	157	0.043	0.975273516	1
ENSBTAG00000032083	*DHX33*	321	367	351	278	424	0.608	0.517143356	1
ENSBTAG00000032084	*GPR183*	72	11	38	62	13	-2.295	0.26852928	1
ENSBTAG00000032087	*ATXN2L*	7'422	7'498	7'543	6'428	8'658	0.430	0.575140944	1
ENSBTAG00000032089	*CDC42EP2*	1'219	1'078	1'150	1'056	1'245	0.238	0.770797897	1
ENSBTAG00000032092	*CDH11*	26'360	17'932	21'767	22'828	20'706	-0.141	0.853874672	1
ENSBTAG00000032093	*SPINK4*	1	2	2	1	2	1.415	0.981979269	1
ENSBTAG00000032097	*FBXO21*	783	844	826	678	975	0.523	0.532596458	1
ENSBTAG00000032106	*PPAPDC1A*	275	343	317	238	396	0.734	0.443321693	1
ENSBTAG00000032114	*processed_pseudogene*	2	0	1	2	0		0.974934741	1
ENSBTAG00000032121	*C7orf10*	129	101	114	112	117	0.062	0.967207541	1
ENSBTAG00000032122	*CD19*	291	404	359	252	466	0.888	0.343223957	1
ENSBTAG00000032125	*protein_coding*	1	2	2	1	2	1.415	0.981979269	1
ENSBTAG00000032130	*CATSPER2*	58	34	45	50	39	-0.355	0.856644463	1
ENSBTAG00000032132	*BT.45294*	70	42	55	61	48	-0.322	0.856791327	1
ENSBTAG00000032136	*pseudogene*	656	554	604	568	640	0.171	0.844020483	1
ENSBTAG00000032137	*PNPLA6*	1'576	2'163	1'931	1'365	2'498	0.872	0.274012158	1
ENSBTAG00000032140	*SBK1*	330	367	355	286	424	0.568	0.544180818	1
ENSBTAG00000032148	*TMEM117*	701	237	440	607	274	-1.149	0.207813688	1
ENSBTAG00000032151	*CCDC73*	36	42	40	31	48	0.637	0.747307132	1
ENSBTAG00000032152	*BT.63399*	22	4	12	19	5	-2.044	0.62595401	1
ENSBTAG00000032156	*protein_coding*	2	0	1	2	0		0.974934741	1

Ensembl gene ID	geneName	counts wildtype horn bud	counts polled horn bud	baseMean	baseMean wildtype horn bud	baseMean polled horn bud	log2FoldChange	pval	padj
ENSBTAG00000032163	*LTV1*	2'242	1'191	1'658	1'942	1'375	-0.498	0.534471616	1
ENSBTAG00000032166	*GTPBP10*	701	449	563	607	518	-0.228	0.796464683	1
ENSBTAG00000032178	*C11ORF85*	1	0	0	1	0		1	1
ENSBTAG00000032183	*NELL2*	3'785	932	2'177	3'278	1'076	-1.607	0.046245151	1
ENSBTAG00000032187	*ZNF697*	151	98	122	131	113	-0.209	0.87092498	1
ENSBTAG00000032193	*REG4*	2	2	2	2	2	0.415	1	1
ENSBTAG00000032198	*protein_coding*	172	91	127	149	105	-0.503	0.679972518	1
ENSBTAG00000032200	*DNAJC24*	1'410	683	1'005	1'221	789	-0.631	0.445852283	1
ENSBTAG00000032206	*BT.67346*	23	9	15	20	10	-0.939	0.794031165	1
ENSBTAG00000032217	*processed_pseudogene*	4'746	8'493	6'959	4'110	9'807	1.255	0.106725981	1
ENSBTAG00000032223	*COG7*	1'161	892	1'018	1'005	1'030	0.035	0.966895523	1
ENSBTAG00000032227	*C14H8orf59*	157	125	140	136	144	0.086	0.946723568	1
ENSBTAG00000032233	*protein_coding*	45	22	32	39	25	-0.617	0.784945429	1
ENSBTAG00000032234	*BT.105466*	1'372	1'197	1'285	1'188	1'382	0.218	0.787595975	1
ENSBTAG00000032236	*CA1*	4	0	2	3	0		0.89545886	1
ENSBTAG00000032237	*BT.27517*	0	1	1	0	1	Inf	0.993540919	1
ENSBTAG00000032247	*PPP1R11*	1'927	1'599	1'758	1'669	1'846	0.146	0.854520462	1
ENSBTAG00000032253	*RAD54L2*	544	1'165	908	471	1'345	1.514	0.073565614	1
ENSBTAG00000032260	*DCUN1D3*	302	231	264	262	267	0.028	0.980722401	1
ENSBTAG00000032265	*BT.24813*	108	32	65	94	37	-1.340	0.391378791	1
ENSBTAG00000032277	*BT.79081*	26	7	15	23	8	-1.478	0.671006397	1
ENSBTAG00000032279	*IQCF2*	2	0	1	2	0		0.974934741	1
ENSBTAG00000032281	*pseudogene*	184	464	348	159	536	1.749	0.068561838	1
ENSBTAG00000032288	*GCK*	304	225	262	263	260	-0.019	0.98990132	1
ENSBTAG00000032289	*ARHGAP15*	114	24	63	99	28	-1.833	0.252452407	1
ENSBTAG00000032292	*CCDC159*	48	25	35	42	29	-0.526	0.808801103	1
ENSBTAG00000032293	*TSPAN16*	0	3	2	0	3	Inf	0.878646179	1
ENSBTAG00000032301	*SLC26A7*	189	107	144	164	124	-0.406	0.730598974	1
ENSBTAG00000032304	*BT.89482*	2'064	1'667	1'856	1'787	1'925	0.107	0.892875665	1
ENSBTAG00000032308	*U1*	4	4	4	3	5	0.415	0.994759363	1
ENSBTAG00000032331	*SLC48A1*	790	982	909	684	1'134	0.729	0.381604983	1
ENSBTAG00000032340	*C10H15ORF23*	703	676	695	609	781	0.359	0.674203843	1
ENSBTAG00000032347	*pseudogene*	666	588	628	577	679	0.235	0.785341828	1
ENSBTAG00000032353	*CCDC63*	2	0	1	2	0		0.974934741	1
ENSBTAG00000032360	*processed_pseudogene*	7'043	12'950	10'526	6'099	14'953	1.294	0.095110918	1
ENSBTAG00000032364	*protein_coding*	205	155	178	178	179	0.012	0.996700294	1
ENSBTAG00000032366	*SLC23A2*	376	441	417	326	509	0.645	0.479491451	1
ENSBTAG00000032369	*BT.87159*	1'095	658	854	948	760	-0.320	0.703336156	1
ENSBTAG00000032372	*BT.59500*	120	114	118	104	132	0.341	0.785909161	1
ENSBTAG00000032373	*BT.62752*	38	14	25	33	16	-1.026	0.691518589	1
ENSBTAG00000032374	*SELK*	2'999	1'996	2'451	2'597	2'305	-0.172	0.827363421	1
ENSBTAG00000032396	*KCTD19*	14	4	8	12	5	-1.392	0.803716462	1
ENSBTAG00000032402	*U1*	5	1	3	4	1	-1.907	0.9188325	1
ENSBTAG00000032405	*protein_coding*	79	98	91	68	113	0.726	0.593215658	1
ENSBTAG00000032424	*FSHR*	3	0	1	3	0		0.936647693	1
ENSBTAG00000032427	*FHOD1*	1'763	1'949	1'889	1'527	2'251	0.560	0.48110622	1
ENSBTAG00000032428	*protein_coding*	3	3	3	3	3	0.415	1	1
ENSBTAG00000032429	*protein_coding*	0	2	1	0	2	Inf	0.939077559	1
ENSBTAG00000032433	*protein_coding*	334	267	299	289	308	0.092	0.926818065	1
ENSBTAG00000032436	*BT.106367*	38'059	24'164	30'431	32'960	27'902	-0.240	0.752464633	1
ENSBTAG00000032446	*protein_coding*	2'468	2'026	2'238	2'137	2'339	0.130	0.868409562	1
ENSBTAG00000032450	*PPP1R14B*	229	312	279	198	360	0.861	0.380795036	1
ENSBTAG00000032451	*H2B*	3	1	2	3	1	-1.170	0.985253077	1
ENSBTAG00000032452	*HIST2H3A*	1	0	0	1	0		1	1
ENSBTAG00000032453	*H4*	14	5	9	12	6	-1.070	0.844263455	1
ENSBTAG00000032455	*H2B*	30	27	29	26	31	0.263	0.922347889	1
ENSBTAG00000032456	*BT.12047*	190	57	115	165	66	-1.322	0.293806986	1
ENSBTAG00000032458	*protein_coding*	5	0	2	4	0		0.853268594	1
ENSBTAG00000032477	*HECTD1*	7'950	4'756	6'188	6'885	5'492	-0.326	0.672191502	1
ENSBTAG00000032481	*DAPL1*	1'107	643	851	959	742	-0.369	0.660423121	1
ENSBTAG00000032485	*LHFPL1*	6	0	3	5	0		0.81115604	1
ENSBTAG00000032493	*FAM134C*	3'046	3'025	3'065	2'638	3'493	0.405	0.60341718	1
ENSBTAG00000032503	*ITPRIPL2*	159	102	128	138	118	-0.225	0.857568945	1
ENSBTAG00000032509	*FAM166B*	39	6	20	34	7	-2.285	0.434300363	1
ENSBTAG00000032515	*PLA2R1*	293	63	163	254	73	-1.802	0.115343961	1
ENSBTAG00000032517	*BCL7A*	162	168	167	140	194	0.468	0.675021498	1
ENSBTAG00000032518	*SPRED1*	5'284	2'933	3'981	4'576	3'387	-0.434	0.57628619	1
ENSBTAG00000032519	*CAMKMT*	156	119	136	135	137	0.024	0.989827386	1
ENSBTAG00000032521	*PLEKHH2*	927	661	783	803	763	-0.073	0.933277806	1
ENSBTAG00000032527	*ERCC6*	672	510	585	582	589	0.017	0.985857962	1
ENSBTAG00000032531	*MUSTN1*	259	298	284	224	344	0.617	0.527720465	1
ENSBTAG00000032534	*RHOF*	1'614	1'247	1'419	1'398	1'440	0.043	0.957780385	1

Ensembl gene ID	geneName	counts wildtype horn bud	counts polled horn bud	baseMean	baseMean wildtype horn bud	baseMean polled horn bud	log2FoldChange	pval	padj
ENSBTAG00000032538	*TPRG1*	24	1	11	21	1	-4.170	0.402732934	1
ENSBTAG00000032544	*SPAG1*	571	309	426	495	357	-0.471	0.605951118	1
ENSBTAG00000032548	*SYBU*	1'133	351	693	981	405	-1.276	0.139716095	1
ENSBTAG00000032557	*C29H11orf10*	2'885	2'034	2'424	2'498	2'349	-0.089	0.910762389	1
ENSBTAG00000032558	*TTC7A*	331	583	480	287	673	1.232	0.171712723	1
ENSBTAG00000032567	*C1QL4*	2	0	1	2	0		0.974934741	1
ENSBTAG00000032588	*TMEM150C*	679	208	414	588	240	-1.292	0.162018911	1
ENSBTAG00000032591	*CTHRC1*	10'096	13'758	12'315	8'743	15'886	0.862	0.261723964	1
ENSBTAG00000032598	*RP9*	601	374	476	520	432	-0.269	0.765224952	1
ENSBTAG00000032603	*BT.93156*	174	112	140	151	129	-0.221	0.856204857	1
ENSBTAG00000032609	*pseudogene*	895	1'522	1'266	775	1'757	1.181	0.149476277	1
ENSBTAG00000032613	*SCG5*	3'301	1'033	2'026	2'859	1'193	-1.261	0.11578959	1
ENSBTAG00000032617	*FBXO44*	4'219	2'498	3'269	3'654	2'884	-0.341	0.662236149	1
ENSBTAG00000032623	*KCNG3*	0	6	3	0	7	Inf	0.700309343	1
ENSBTAG00000032631	*protein_coding*	1	2	2	1	2	1.415	0.981979269	1
ENSBTAG00000032637	*EXOC1*	1'431	741	1'047	1'239	856	-0.534	0.516772624	1
ENSBTAG00000032640	*FOPNL*	2'358	1'290	1'766	2'042	1'490	-0.455	0.568658851	1
ENSBTAG00000032642	*BT.33009*	1	1	1	1	1	0.415	1	1
ENSBTAG00000032650	*DPY19L2*	12	0	5	10	0		0.583618942	1
ENSBTAG00000032651	*protein_coding*	1	1	1	1	1	0.415	1	1
ENSBTAG00000032656	*CPN2*	4	1	2	3	1	-1.585	0.952247636	1
ENSBTAG00000032657	*TEAD1*	140	94	115	121	109	-0.160	0.90545032	1
ENSBTAG00000032660	*CHIC2*	1'021	597	787	884	689	-0.359	0.671060796	1
ENSBTAG00000032669	*protein_coding*	1	0	0	1	0		1	1
ENSBTAG00000032674	*SENP5*	658	660	666	570	762	0.419	0.62445185	1
ENSBTAG00000032680	*TMEM63B*	2'949	3'152	3'097	2'554	3'640	0.511	0.512478126	1
ENSBTAG00000032684	*TCTEX1D2*	368	247	302	319	285	-0.160	0.871853383	1
ENSBTAG00000032686	*HOMEZ*	717	696	712	621	804	0.372	0.661722115	1
ENSBTAG00000032687	*CST9L*	1	0	0	1	0		1	1
ENSBTAG00000032704	*NAPB*	1'626	691	1'103	1'408	798	-0.820	0.31934656	1
ENSBTAG00000032705	*BT.63542*	4'964	3'954	4'432	4'299	4'566	0.087	0.910127701	1
ENSBTAG00000032709	*CMTM5*	108	112	111	94	129	0.468	0.712736625	1
ENSBTAG00000032711	*HTATSF1*	215	85	142	186	98	-0.924	0.430339121	1
ENSBTAG00000032719	*TFRC*	417	280	342	361	323	-0.160	0.86889057	1
ENSBTAG00000032727	*protein_coding*	1	0	0	1	0		1	1
ENSBTAG00000032733	*RIIAD1*	25	4	13	22	5	-2.229	0.568026072	1
ENSBTAG00000032738	*C8ORF85*	4	0	2	3	0		0.89545886	1
ENSBTAG00000032761	*CALHM3*	5	0	2	4	0		0.853268594	1
ENSBTAG00000032763	*RANBP10*	2'355	2'815	2'645	2'039	3'250	0.672	0.391930408	1
ENSBTAG00000032764	*pseudogene*	4'530	6'297	5'597	3'923	7'271	0.890	0.250458723	1
ENSBTAG00000032774	*C28H10ORF116*	674	517	590	584	597	0.032	0.971624152	1
ENSBTAG00000032777	*TOMM5*	1'707	1'028	1'333	1'478	1'187	-0.317	0.696422035	1
ENSBTAG00000032790	*protein_coding*	1	1	1	1	1	0.415	1	1
ENSBTAG00000032806	*protein_coding*	145	150	149	126	173	0.464	0.68737513	1
ENSBTAG00000032808	*FASLG*	4	2	3	3	2	-0.585	0.996239345	1
ENSBTAG00000032819	*MUC20*	317	53	168	275	61	-2.165	0.059642856	1
ENSBTAG00000032821	*SCEL*	3'614	1'595	2'486	3'130	1'842	-0.765	0.332355733	1
ENSBTAG00000032827	*5S_rRNA*	1	3	2	1	3	2.000	0.933774563	1
ENSBTAG00000032829	*GHITM*	4'572	2'681	3'528	3'959	3'096	-0.355	0.648686391	1
ENSBTAG00000032831	*MRPL33*	752	354	530	651	409	-0.672	0.447165096	1
ENSBTAG00000032833	*protein_coding*	2	0	1	2	0		0.974934741	1
ENSBTAG00000032839	*LRCH3*	1'311	678	959	1'135	783	-0.536	0.518244975	1
ENSBTAG00000032846	*MGC152346*	24	12	17	21	14	-0.585	0.865660962	1
ENSBTAG00000032848	*GOLPH3*	6'084	2'810	4'257	5'269	3'245	-0.699	0.368227703	1
ENSBTAG00000032852	*BT.58452*	1'481	575	973	1'283	664	-0.950	0.253720047	1
ENSBTAG00000032858	*DYDC1*	2	0	1	2	0		0.974934741	1
ENSBTAG00000032859	*protein_coding*	188	174	182	163	201	0.303	0.782045899	1
ENSBTAG00000032862	*DYDC2*	33	3	16	29	3	-3.044	0.387507238	1
ENSBTAG00000032869	*RNASE10*	92	39	62	80	45	-0.823	0.605600391	1
ENSBTAG00000032872	*protein_coding*	284	210	244	246	242	-0.020	0.989304073	1
ENSBTAG00000032881	*SLCO5A1*	3	0	1	3	0		0.936647693	1
ENSBTAG00000032884	*TNP2*	2	7	5	2	8	2.222	0.797845137	1
ENSBTAG00000032887	*BT.64136*	1'298	654	940	1'124	755	-0.574	0.490111841	1
ENSBTAG00000032893	*protein_coding*	89	174	139	77	201	1.382	0.242422778	1
ENSBTAG00000032899	*protein_coding*	10	10	10	9	12	0.415	0.946590905	1
ENSBTAG00000032902	*SLC11A2*	862	481	651	747	555	-0.427	0.620733913	1
ENSBTAG00000032905	*FAM82A1*	539	197	347	467	227	-1.037	0.273525898	1
ENSBTAG00000032908	*C10H14orf176*	8	2	5	7	2	-1.585	0.87391615	1
ENSBTAG00000032914	*SLC11A2*	2'670	1'399	1'964	2'312	1'615	-0.517	0.515032274	1
ENSBTAG00000032923	*protein_coding*	2	1	1	2	1	-0.585	1	1
ENSBTAG00000032933	*TTC23L*	11	2	6	10	2	-2.044	0.786625039	1
ENSBTAG00000032944	*pseudogene*	0	1	1	0	1	Inf	0.993540919	1

Ensembl gene ID	geneName	counts wildtype horn bud	counts polled horn bud	baseMean	baseMean wildtype horn bud	baseMean polled horn bud	log2FoldChange	pval	padj
ENSBTAG00000032947	*GRXCR1*	1	0	0	1	0		1	1
ENSBTAG00000032951	*FAM108C1*	1'007	289	603	872	334	-1.386	0.115047316	1
ENSBTAG00000032954	*ATP5E*	4'750	4'176	4'468	4'114	4'822	0.229	0.76634991	1
ENSBTAG00000032961	*IER3IP1*	2'950	1'672	2'243	2'555	1'931	-0.404	0.609135567	1
ENSBTAG00000032962	*SKOR2*	1	0	0	1	0		1	1
ENSBTAG00000032964	*C24H18orf25*	263	171	213	228	197	-0.206	0.847692806	1
ENSBTAG00000032979	*protein_coding*	345	365	360	299	421	0.496	0.5954134	1
ENSBTAG00000032982	*BT.23286*	2'886	1'303	2'002	2'499	1'505	-0.732	0.357284071	1
ENSBTAG00000032996	*P4HA1*	14'168	8'154	10'843	12'270	9'415	-0.382	0.617954974	1
ENSBTAG00000033007	*BT.61161*	2'358	864	1'520	2'042	998	-1.033	0.201592123	1
ENSBTAG00000033008	*BT.45893*	85	288	203	74	333	2.176	0.04602536	1
ENSBTAG00000033010	*TTC27*	1'784	1'319	1'534	1'545	1'523	-0.021	0.981083467	1
ENSBTAG00000033015	*FAHD1*	586	371	468	507	428	-0.244	0.787175985	1
ENSBTAG00000033021	*pseudogene*	1	0	0	1	0		1	1
ENSBTAG00000033030	*protein_coding*	2	0	1	2	0		0.974934741	1
ENSBTAG00000033032	*BT.49606*	3'652	2'766	3'178	3'163	3'194	0.014	0.985279931	1
ENSBTAG00000033041	*YIPF4*	1'969	874	1'357	1'705	1'009	-0.757	0.35096943	1
ENSBTAG00000033055	*protein_coding*	1	1	1	1	1	0.415	1	1
ENSBTAG00000033056	*PLD5*	554	106	301	480	122	-1.971	0.047696943	1
ENSBTAG00000033076	*TRPM6*	53	17	33	46	20	-1.225	0.573855536	1
ENSBTAG00000033077	*NUFIP2*	479	352	411	415	406	-0.029	0.978070997	1
ENSBTAG00000033078	*CNEP1R1*	1'948	1'258	1'570	1'687	1'453	-0.216	0.788696384	1
ENSBTAG00000033080	*RPL15*	17'817	15'844	16'863	15'430	18'295	0.246	0.746460518	1
ENSBTAG00000033089	*PTPLA*	484	220	337	419	254	-0.722	0.446687911	1
ENSBTAG00000033095	*WDR70*	1'472	1'351	1'417	1'275	1'560	0.291	0.717392673	1
ENSBTAG00000033107	*BT.103407*	464	430	449	402	497	0.305	0.735370361	1
ENSBTAG00000033122	*FOXD4L1*	0	1	1	0	1	Inf	0.993540919	1
ENSBTAG00000033127	*C16H1orf100*	1	1	1	1	1	0.415	1	1
ENSBTAG00000033128	*CRH*	5	0	2	4	0		0.853268594	1
ENSBTAG00000033136	*FAM36A*	3'195	1'019	1'972	2'767	1'177	-1.234	0.12412924	1
ENSBTAG00000033137	*PSD3*	218	417	335	189	482	1.351	0.158015216	1
ENSBTAG00000033140	*LRRC3B*	1'596	481	969	1'382	555	-1.315	0.116607435	1
ENSBTAG00000033160	*FAM114A1*	3'572	2'474	2'975	3'093	2'857	-0.115	0.884058774	1
ENSBTAG00000033166	*SLC15A2*	227	120	168	197	139	-0.505	0.652184763	1
ENSBTAG00000033167	*protein_coding*	83	77	80	72	89	0.307	0.834209106	1
ENSBTAG00000033169	*LAMB4*	5	2	3	4	2	-0.907	0.965043111	1
ENSBTAG00000033170	*LRRC58*	115	51	79	100	59	-0.758	0.599109985	1
ENSBTAG00000033173	*BHLHE22*	67	3	31	58	3	-4.066	0.108759842	1
ENSBTAG00000033174	*TFAP4*	562	948	791	487	1'095	1.169	0.168700564	1
ENSBTAG00000033180	*SMYD3*	451	551	513	391	636	0.704	0.42670336	1
ENSBTAG00000033182	*HGSNAT*	2'566	2'678	2'657	2'222	3'092	0.477	0.542990047	1
ENSBTAG00000033186	*OXCT1*	3'984	2'622	3'239	3'450	3'028	-0.189	0.809644583	1
ENSBTAG00000033190	*PGM5*	3'845	4'002	3'975	3'330	4'621	0.473	0.541924325	1
ENSBTAG00000033195	*protein_coding*	15	0	6	13	0		0.491308095	1
ENSBTAG00000033197	*EPDR1*	954	310	592	826	358	-1.207	0.169295461	1
ENSBTAG00000033214	*TBC1D19*	470	169	301	407	195	-1.061	0.275699264	1
ENSBTAG00000033217	*TPM3*	7'563	6'261	6'890	6'550	7'230	0.142	0.852210613	1
ENSBTAG00000033218	*BT.67847*	13	29	22	11	33	1.573	0.557224015	1
ENSBTAG00000033220	*C3H1orf189*	71	25	45	61	29	-1.091	0.554703862	1
ENSBTAG00000033221	*CCDC152*	13'037	7'469	9'957	11'290	8'624	-0.389	0.612194438	1
ENSBTAG00000033222	*BT.54234*	0	1	1	0	1	Inf	0.993540919	1
ENSBTAG00000033237	*protein_coding*	1	0	0	1	0		1	1
ENSBTAG00000033248	*CDH3*	3'096	7'053	5'413	2'681	8'144	1.603	0.041766926	1
ENSBTAG00000033252	*BT.88134*	304	329	322	263	380	0.529	0.579265351	1
ENSBTAG00000033254	*DFNB59*	152	63	102	132	73	-0.856	0.51276102	1
ENSBTAG00000033255	*BT.69450*	1'964	1'189	1'537	1'701	1'373	-0.309	0.700983758	1
ENSBTAG00000033267	*LIN9*	398	341	369	345	394	0.192	0.837810903	1
ENSBTAG00000033269	*U1*	0	1	1	0	1	Inf	0.993540919	1
ENSBTAG00000033270	*SEBOX*	1	0	0	1	0		1	1
ENSBTAG00000033278	*BT.61519*	6	12	10	5	14	1.415	0.771639311	1
ENSBTAG00000033284	*CHCHD7*	1'198	718	933	1'037	829	-0.324	0.697697744	1
ENSBTAG00000033289	*pseudogene*	7	13	11	6	15	1.308	0.772290598	1
ENSBTAG00000033290	*TMEM60*	751	521	626	650	602	-0.112	0.898634134	1
ENSBTAG00000033291	*SPAG6*	20	0	9	17	0		0.367514426	1
ENSBTAG00000033298	*C19H17orf108*	1'018	1'041	1'042	882	1'202	0.447	0.586366428	1
ENSBTAG00000033299	*BT.24635*	243	50	134	210	58	-1.866	0.124793185	1
ENSBTAG00000033304	*C27H8orf4*	2'884	848	1'738	2'498	979	-1.351	0.094895779	1
ENSBTAG00000033308	*pseudogene*	55	85	73	48	98	1.043	0.480771568	1
ENSBTAG00000033312	*BT.54180*	11	0	5	10	0		0.617662983	1
ENSBTAG00000033313	*SLC38A9*	1'124	669	873	973	772	-0.334	0.690527054	1
ENSBTAG00000033315	*DNAJC1*	1'197	710	928	1'037	820	-0.338	0.684525948	1
ENSBTAG00000033316	*PDCD1LG2*	4	0	2	3	0		0.89545886	1

Ensembl gene ID	geneName	counts wildtype horn bud	counts polled horn bud	baseMean	baseMean wildtype horn bud	baseMean polled horn bud	log2FoldChange	pval	padj
ENSBTAG00000033322	*SRP9*	1'409	1'086	1'237	1'220	1'254	0.039	0.961676934	1
ENSBTAG00000033330	*HOXD11*	8	21	16	7	24	1.807	0.592169817	1
ENSBTAG00000033331	*CHMP1A*	2'503	2'726	2'658	2'168	3'148	0.538	0.492494225	1
ENSBTAG00000033333	*CDK10*	1'274	975	1'115	1'103	1'126	0.029	0.972077512	1
ENSBTAG00000033334	*SPATA2L*	182	142	161	158	164	0.057	0.964721653	1
ENSBTAG00000033335	*NDUFB4*	51	39	45	44	45	0.028	1	1
ENSBTAG00000033339	*ANKLE2*	2'733	2'549	2'655	2'367	2'943	0.314	0.687786885	1
ENSBTAG00000033344	*BT.88035*	183	150	166	158	173	0.128	0.912612028	1
ENSBTAG00000033345	*STAR*	393	145	254	340	167	-1.023	0.309725539	1
ENSBTAG00000033346	*BT.61462*	31	24	27	27	28	0.046	1	1
ENSBTAG00000033351	*PACRGL*	622	367	481	539	424	-0.346	0.699876042	1
ENSBTAG00000033360	*processed_pseudogene*	34	55	46	29	64	1.109	0.539171582	1
ENSBTAG00000033365	*C6ORF186*	215	50	122	186	58	-1.689	0.175253986	1
ENSBTAG00000033367	*BT.25930*	6	4	5	5	5	-0.170	1	1
ENSBTAG00000033377	*protein_coding*	5	1	3	4	1	-1.907	0.9188325	1
ENSBTAG00000033381	*protein_coding*	3	9	6	3	10	2.000	0.764028622	1
ENSBTAG00000033384	*BT.54399*	2	3	3	2	3	1.000	0.97171833	1
ENSBTAG00000033386	*C12H13ORF26*	16	0	7	14	0		0.463690882	1
ENSBTAG00000033395	*CCSMST1*	1'633	962	1'263	1'414	1'111	-0.348	0.668651721	1
ENSBTAG00000033396	*C8H9orf135*	5	1	3	4	1	-1.907	0.9188325	1
ENSBTAG00000033398	*HKDC1*	83	30	53	72	35	-1.053	0.53740072	1
ENSBTAG00000033412	*B3GALTL*	2'540	1'818	2'149	2'200	2'099	-0.067	0.933174146	1
ENSBTAG00000033413	*BT.55841*	3'152	1'866	2'442	2'730	2'155	-0.341	0.664936803	1
ENSBTAG00000033422	*HHATL*	6	33	22	5	38	2.874	0.310686182	1
ENSBTAG00000033423	*BT.35532*	546	893	752	473	1'031	1.125	0.187248603	1
ENSBTAG00000033429	*C9H6orf225*	515	468	493	446	540	0.277	0.755956809	1
ENSBTAG00000033441	*SHCBP1*	733	540	629	635	624	-0.026	0.978912703	1
ENSBTAG00000033446	*TATDN1*	2'465	2'016	2'231	2'135	2'328	0.125	0.873812831	1
ENSBTAG00000033449	*SLC25A40*	220	67	134	191	77	-1.300	0.278054761	1
ENSBTAG00000033453	*MTCH1*	15'440	22'177	19'490	13'371	25'608	0.937	0.221379649	1
ENSBTAG00000033457	*BT.65444*	1'345	625	943	1'165	722	-0.691	0.406411168	1
ENSBTAG00000033460	*FAM162B*	82	59	70	71	68	-0.060	0.979964086	1
ENSBTAG00000033464	*BT.41234*	356	252	300	308	291	-0.083	0.93548936	1
ENSBTAG00000033470	*SPINK6*	94	101	99	81	117	0.519	0.694921158	1
ENSBTAG00000033476	*protein_coding*	1	0	0	1	0		1	1
ENSBTAG00000033486	*C17H4orf46*	1'621	1'226	1'410	1'404	1'416	0.012	0.988317825	1
ENSBTAG00000033504	*MAK16*	1'287	952	1'107	1'115	1'099	-0.020	0.982516189	1
ENSBTAG00000033509	*OR4D9*	1	0	0	1	0		1	1
ENSBTAG00000033510	*MPZL2*	1'236	1'184	1'219	1'070	1'367	0.353	0.663955333	1
ENSBTAG00000033515	*BT.21896*	15'138	7'499	10'884	13'110	8'659	-0.598	0.435181005	1
ENSBTAG00000033523	*BT.18403*	182	115	145	158	133	-0.247	0.836080385	1
ENSBTAG00000033526	*GNG13*	4	2	3	3	2	-0.585	0.996239345	1
ENSBTAG00000033529	*SCN2B*	167	318	256	145	367	1.344	0.182792809	1
ENSBTAG00000033531	*protein_coding*	1	2	2	1	2	1.415	0.981979269	1
ENSBTAG00000033535	*FAM26D*	1	0	0	1	0		1	1
ENSBTAG00000033543	*RRAGA*	5'327	4'209	4'737	4'613	4'860	0.075	0.921984478	1
ENSBTAG00000033545	*EBD*	198	55	117	171	64	-1.433	0.253033554	1
ENSBTAG00000033563	*ZNF529*	1'532	528	968	1'327	610	-1.122	0.179121549	1
ENSBTAG00000033565	*pseudogene*	3'764	1'615	2'562	3'260	1'865	-0.806	0.307023118	1
ENSBTAG00000033580	*MSLNL*	13	12	13	11	14	0.300	0.958035022	1
ENSBTAG00000033603	*MNF1*	851	907	892	737	1'047	0.507	0.542634549	1
ENSBTAG00000033608	*pseudogene*	90	68	78	78	79	0.011	1	1
ENSBTAG00000033621	*C11orf75*	435	383	409	377	442	0.231	0.801200337	1
ENSBTAG00000033642	*protein_coding*	2'054	486	1'170	1'779	561	-1.664	0.045945892	1
ENSBTAG00000033643	*protein_coding*	1	2	2	1	2	1.415	0.981979269	1
ENSBTAG00000033648	*INTS12*	1'019	743	870	882	858	-0.041	0.963304193	1
ENSBTAG00000033657	*SLC35F1*	224	38	119	194	44	-2.144	0.092606809	1
ENSBTAG00000033662	*NCKAP1*	5'502	3'756	4'551	4'765	4'337	-0.136	0.861445779	1
ENSBTAG00000033669	*BT.42400*	1'598	1'819	1'742	1'384	2'100	0.602	0.45056441	1
ENSBTAG00000033672	*JMJD8*	608	623	623	527	719	0.450	0.602024116	1
ENSBTAG00000033677	*WDR90*	1'409	1'927	1'723	1'220	2'225	0.867	0.279262524	1
ENSBTAG00000033680	*BT.32707*	647	389	505	560	449	-0.319	0.720765413	1
ENSBTAG00000033685	*BT.88687*	122	95	108	106	110	0.054	0.973332148	1
ENSBTAG00000033690	*BARD1*	187	196	194	162	226	0.483	0.651710585	1
ENSBTAG00000033695	*IFNB2*	2	3	3	2	3	1.000	0.97171833	1
ENSBTAG00000033699	*CHRM4*	24	13	18	21	15	-0.469	0.89373307	1
ENSBTAG00000033702	*STARD8*	268	137	195	232	158	-0.553	0.6060623	1
ENSBTAG00000033721	*RLBP1*	247	69	147	214	80	-1.425	0.222778516	1
ENSBTAG00000033726	*GRIP1*	471	414	443	408	478	0.229	0.800766178	1
ENSBTAG00000033727	*BT.48519*	467	533	510	404	615	0.606	0.494073555	1
ENSBTAG00000033731	*BT.55896*	3'718	2'173	2'865	3'220	2'509	-0.360	0.646187667	1
ENSBTAG00000033735	*KIAA1033*	1'457	831	1'111	1'262	960	-0.395	0.630359708	1

Ensembl gene ID	geneName	counts wildtype horn bud	counts polled horn bud	baseMean	baseMean wildtype horn bud	baseMean polled horn bud	log2FoldChange	pval	padj
ENSBTAG00000033739	*C16ORF11*	0	1	1	0	1	Inf	0.993540919	1
ENSBTAG00000033740	*processed_pseudogene*	182	458	343	158	529	1.746	0.069630632	1
ENSBTAG00000033747	*SIL1*	1'564	1'593	1'597	1'354	1'839	0.442	0.581030098	1
ENSBTAG00000033748	*IL18RAP*	7	1	4	6	1	-2.392	0.851750348	1
ENSBTAG00000033749	*processed_pseudogene*	605	421	505	524	486	-0.108	0.905741004	1
ENSBTAG00000033759	*protein_coding*	1	0	0	1	0		1	1
ENSBTAG00000033766	*KRT16*	303	510	426	262	589	1.166	0.202922282	1
ENSBTAG00000033801	*BT.61819*	357	380	374	309	439	0.505	0.586464132	1
ENSBTAG00000033802	*pseudogene*	3	0	1	3	0		0.936647693	1
ENSBTAG00000033806	*BT.43626*	54	158	115	47	182	1.964	0.124011709	1
ENSBTAG00000033837	*protein_coding*	16	6	10	14	7	-1.000	0.835750236	1
ENSBTAG00000033884	*CCDC72*	1'700	715	1'149	1'472	826	-0.834	0.309298766	1
ENSBTAG00000033886	*BT.28198*	15	73	49	13	84	2.698	0.14288081	1
ENSBTAG00000033890	*BT.88201*	1	3	2	1	3	2.000	0.933774563	1
ENSBTAG00000033891	*MGAT2*	1'232	958	1'087	1'067	1'106	0.052	0.949728569	1
ENSBTAG00000033894	*protein_coding*	1	0	0	1	0		1	1
ENSBTAG00000033902	*BT.91032*	1'131	565	816	979	652	-0.586	0.4862836	1
ENSBTAG00000033961	*RNF217*	149	65	102	129	75	-0.782	0.550207119	1
ENSBTAG00000033983	*MAP2K1*	3'276	1'795	2'455	2'837	2'073	-0.453	0.565316891	1
ENSBTAG00000033994	*U2*	0	1	1	0	1	Inf	0.993540919	1
ENSBTAG00000033998	*RFX8*	23	14	18	20	16	-0.301	0.939684769	1
ENSBTAG00000034005	*ZNF35*	228	335	292	197	387	0.970	0.319756146	1
ENSBTAG00000034024	*HBZ*	3	2	2	3	2	-0.170	1	1
ENSBTAG00000034033	*LHFP*	3'645	2'356	2'939	3'157	2'720	-0.215	0.784443286	1
ENSBTAG00000034045	*BT.17305*	2	0	1	2	0		0.974934741	1
ENSBTAG00000034069	*MAB21L1*	577	452	511	500	522	0.063	0.944975326	1
ENSBTAG00000034075	*BT.104135*	1	2	2	1	2	1.415	0.981979269	1
ENSBTAG00000034089	*protein_coding*	4'127	3'847	4'008	3'574	4'442	0.314	0.685220141	1
ENSBTAG00000034090	*protein_coding*	288	111	189	249	128	-0.960	0.374315271	1
ENSBTAG00000034091	*LRRC72*	44	17	29	38	20	-0.957	0.684136623	1
ENSBTAG00000034106	*CYP8B1*	9	2	5	8	2	-1.755	0.844314416	1
ENSBTAG00000034113	*GALNT10*	557	396	470	482	457	-0.077	0.934467989	1
ENSBTAG00000034138	*protein_coding*	569	264	399	493	305	-0.693	0.45228805	1
ENSBTAG00000034140	*MEGF11*	147	38	86	127	44	-1.537	0.274596191	1
ENSBTAG00000034145	*protein_coding*	15	5	9	13	6	-1.170	0.820729911	1
ENSBTAG00000034147	*OLFML2B*	14'326	11'594	12'897	12'407	13'388	0.110	0.884979176	1
ENSBTAG00000034154	*AGMO*	140	39	83	121	45	-1.429	0.313989807	1
ENSBTAG00000034159	*FCRLA*	15	7	11	13	8	-0.684	0.893749952	1
ENSBTAG00000034170	*DYNLL1*	700	1'364	1'091	606	1'575	1.377	0.097504654	1
ENSBTAG00000034174	*protein_coding*	17	6	11	15	7	-1.087	0.813534849	1
ENSBTAG00000034182	*processed_pseudogene*	3'444	4'758	4'238	2'983	5'494	0.881	0.257511125	1
ENSBTAG00000034185	*BT.63960*	135'433	228'037	190'301	117'288	263'314	1.167	0.12814354	1
ENSBTAG00000034192	*BT.47932*	34	13	22	29	15	-0.972	0.724312717	1
ENSBTAG00000034193	*BT.64006*	3'083	2'908	3'014	2'670	3'358	0.331	0.67139922	1
ENSBTAG00000034196	*HHLA1*	9	0	4	8	0		0.690676647	1
ENSBTAG00000034206	*BT.65771*	3'598	1'336	2'329	3'116	1'543	-1.014	0.201344524	1
ENSBTAG00000034220	*CDKN2A*	8	12	10	7	14	1.000	0.83142557	1
ENSBTAG00000034222	*CAB39L*	338	258	295	293	298	0.025	0.982312309	1
ENSBTAG00000034225	*BT.92467*	1'282	1'116	1'199	1'110	1'289	0.215	0.79159905	1
ENSBTAG00000034246	*ACRBP*	176	170	174	152	196	0.365	0.741339666	1
ENSBTAG00000034255	*BT.81994*	2'256	1'329	1'744	1'954	1'535	-0.348	0.662880604	1
ENSBTAG00000034261	*protein_coding*	224	519	397	194	599	1.627	0.08219412	1
ENSBTAG00000034269	*RCBTB1*	962	1'042	1'018	833	1'203	0.530	0.519820576	1
ENSBTAG00000034281	*protein_coding*	8	19	14	7	22	1.663	0.639717134	1
ENSBTAG00000034284	*pseudogene*	0	13	8	0	15	Inf	0.392334779	1
ENSBTAG00000034287	*TULP1*	123	111	117	107	128	0.267	0.833348308	1
ENSBTAG00000034294	*pseudogene*	1	2	2	1	2	1.415	0.981979269	1
ENSBTAG00000034295	*BT.18959*	28	35	32	24	40	0.737	0.737898558	1
ENSBTAG00000034302	*BT.96720*	25	3	13	22	3	-2.644	0.518110331	1
ENSBTAG00000034315	*pseudogene*	1	0	0	1	0		1	1
ENSBTAG00000034320	*pseudogene*	2	0	1	2	0		0.974934741	1
ENSBTAG00000034323	*BT.19805*	15	14	15	13	16	0.316	0.945977687	1
ENSBTAG00000034325	*IFNAG*	1	1	1	1	1	0.415	1	1
ENSBTAG00000034326	*protein_coding*	2	1	1	2	1	-0.585	1	1
ENSBTAG00000034332	*BT.86243*	3	0	1	3	0		0.936647693	1
ENSBTAG00000034337	*U3*	6	13	10	5	15	1.531	0.740992914	1
ENSBTAG00000034338	*C15H11orf88*	12	0	5	10	0		0.583618942	1
ENSBTAG00000034346	*BTG4*	2	0	1	2	0		0.974934741	1
ENSBTAG00000034347	*BT.48829*	212	113	157	184	130	-0.493	0.666030099	1
ENSBTAG00000034349	*IFI44*	51	13	30	44	15	-1.557	0.499888893	1
ENSBTAG00000034360	*SERF1*	1'273	919	1'082	1'102	1'061	-0.055	0.948316061	1
ENSBTAG00000034366	*RGS2*	379	237	301	328	274	-0.262	0.788900879	1

Ensembl gene ID	geneName	counts wildtype horn bud	counts polled horn bud	baseMean	baseMean wildtype horn bud	baseMean polled horn bud	log2FoldChange	pval	padj
ENSBTAG00000034368	*PRSS33*	10	8	9	9	9	0.093	1	1
ENSBTAG00000034373	*CDH13*	3'266	4'747	4'155	2'828	5'481	0.955	0.220827102	1
ENSBTAG00000034376	*pseudogene*	5	9	7	4	10	1.263	0.837733706	1
ENSBTAG00000034384	*VWA3B*	41	10	24	36	12	-1.621	0.538651466	1
ENSBTAG00000034385	*BT.44382*	470	115	270	407	133	-1.616	0.108459376	1
ENSBTAG00000034391	*C9ORF11*	37	14	24	32	16	-0.987	0.706068506	1
ENSBTAG00000034393	*BT.46670*	678	338	489	587	390	-0.589	0.509654228	1
ENSBTAG00000034396	*BT.106321*	743	641	692	643	740	0.202	0.81324826	1
ENSBTAG00000034398	*processed_pseudogene*	6	24	16	5	28	2.415	0.464285575	1
ENSBTAG00000034402	*ACE2*	149	38	86	129	44	-1.556	0.266939431	1
ENSBTAG00000034411	*SHISA3*	1'163	509	797	1'007	588	-0.777	0.357746568	1
ENSBTAG00000034430	*BT.41271*	389	328	358	337	379	0.169	0.858227094	1
ENSBTAG00000034435	*NKD2*	1'487	1'078	1'266	1'288	1'245	-0.049	0.953524233	1
ENSBTAG00000034436	*PDPK1*	316	359	344	274	415	0.599	0.524907688	1
ENSBTAG00000034441	*BT.50280*	39	32	35	34	37	0.130	0.963531861	1
ENSBTAG00000034442	*LNP1*	318	142	220	275	164	-0.748	0.471690648	1
ENSBTAG00000034449	*GNG11*	670	1'324	1'055	580	1'529	1.398	0.093634075	1
ENSBTAG00000034493	*C6ORF106*	347	525	453	301	606	1.012	0.263179862	1
ENSBTAG00000034495	*GTF2F2*	548	405	471	475	468	-0.021	0.984537975	1
ENSBTAG00000034496	*SHFM1*	5'991	2'112	3'814	5'188	2'439	-1.089	0.164525418	1
ENSBTAG00000034498	*LY6D*	845	548	682	732	633	-0.210	0.807922472	1
ENSBTAG00000034501	*BT.64790*	213	66	130	184	76	-1.275	0.291304148	1
ENSBTAG00000034503	*protein_coding*	4	1	2	3	1	-1.585	0.952247636	1
ENSBTAG00000034506	*SNRPG*	1'755	1'757	1'774	1'520	2'029	0.417	0.600538354	1
ENSBTAG00000034514	*protein_coding*	0	5	3	0	6	Inf	0.757530434	1
ENSBTAG00000034519	*protein_coding*	210	110	154	182	127	-0.518	0.651467181	1
ENSBTAG00000034522	*SLC10A7*	159	132	145	138	152	0.147	0.903850913	1
ENSBTAG00000034531	*RNF146B*	2'702	1'615	2'102	2'340	1'865	-0.327	0.679502542	1
ENSBTAG00000034536	*GDPD4*	1	0	0	1	0		1	1
ENSBTAG00000034560	*processed_pseudogene*	157	210	189	136	242	0.835	0.437720943	1
ENSBTAG00000034580	*TMSB4*	6'041	13'477	10'397	5'232	15'562	1.573	0.043942007	1
ENSBTAG00000034586	*BT.73167*	783	159	431	678	184	-1.885	0.043301525	1
ENSBTAG00000034597	*protein_coding*	106	102	105	92	118	0.360	0.783397485	1
ENSBTAG00000034598	*BT.88393*	2'174	1'285	1'683	1'883	1'484	-0.344	0.667844938	1
ENSBTAG00000034609	*BT.72554*	25	12	18	22	14	-0.644	0.847278186	1
ENSBTAG00000034611	*BT.91405*	1	0	0	1	0		1	1
ENSBTAG00000034613	*C8ORF73*	143	37	83	124	43	-1.535	0.280024176	1
ENSBTAG00000034626	*FBXO16*	21	15	18	18	17	-0.070	1	1
ENSBTAG00000034633	*ERO1LB*	419	202	298	363	233	-0.638	0.511766633	1
ENSBTAG00000034643	*protein_coding*	53	22	36	46	25	-0.853	0.684118027	1
ENSBTAG00000034644	*protein_coding*	1	0	0	1	0		1	1
ENSBTAG00000034645	*PON3*	555	522	542	481	603	0.327	0.710203762	1
ENSBTAG00000034656	*protein_coding*	1'622	817	1'174	1'405	943	-0.574	0.482451003	1
ENSBTAG00000034657	*BT.50731*	1	0	0	1	0		1	1
ENSBTAG00000034659	*UBTD2*	1'354	1'437	1'416	1'173	1'659	0.501	0.534180971	1
ENSBTAG00000034662	*processed_pseudogene*	1'115	1'594	1'403	966	1'841	0.931	0.250894047	1
ENSBTAG00000034665	*PRSS55*	1	0	0	1	0		1	1
ENSBTAG00000034674	*BT.77727*	3	7	5	3	8	1.637	0.840440358	1
ENSBTAG00000034676	*HIGD1D*	123	93	107	107	107	0.012	1	1
ENSBTAG00000034680	*FAM131C*	34	39	37	29	45	0.613	0.765582117	1
ENSBTAG00000034681	*CA10*	141	32	80	122	37	-1.725	0.235531633	1
ENSBTAG00000034689	*ZNRF1*	1'355	1'305	1'340	1'173	1'507	0.361	0.655080794	1
ENSBTAG00000034691	*TSTA3*	1'102	1'202	1'171	954	1'388	0.540	0.507665892	1
ENSBTAG00000034700	*NAF1*	1'071	650	839	928	751	-0.305	0.716564451	1
ENSBTAG00000034711	*protein_coding*	2	0	1	2	0		0.974934741	1
ENSBTAG00000034712	*BT.35757*	165	90	123	143	104	-0.459	0.70988053	1
ENSBTAG00000034753	*CCDC122*	4	1	2	3	1	-1.585	0.952247636	1
ENSBTAG00000034766	*U1*	3	6	5	3	7	1.415	0.880191876	1
ENSBTAG00000034767	*U1*	3	9	6	3	10	2.000	0.764028622	1
ENSBTAG00000034768	*U1*	6	0	3	5	0		0.81115604	1
ENSBTAG00000034769	*U1*	5	4	4	4	5	0.093	1	1
ENSBTAG00000034770	*U3*	2	6	4	2	7	2.000	0.840116992	1
ENSBTAG00000034776	*DDAH1*	2'314	1'125	1'652	2'004	1'299	-0.625	0.435308343	1
ENSBTAG00000034785	*DNAJC15*	530	388	454	459	448	-0.035	0.972529974	1
ENSBTAG00000034796	*TEX264*	1'721	2'324	2'087	1'490	2'684	0.848	0.285375883	1
ENSBTAG00000034823	*BT.26680*	1'762	1'022	1'353	1'526	1'180	-0.371	0.647236231	1
ENSBTAG00000034824	*processed_pseudogene*	2'766	2'285	2'517	2'395	2'638	0.139	0.858681117	1
ENSBTAG00000034827	*PDGFD*	1'452	1'623	1'566	1'257	1'874	0.576	0.472802541	1
ENSBTAG00000034844	*RPL39*	2'497	1'028	1'675	2'162	1'187	-0.865	0.281302486	1
ENSBTAG00000034845	*protein_coding*	1	0	0	1	0		1	1
ENSBTAG00000034848	*BT.28745*	244	344	304	211	397	0.911	0.346157668	1
ENSBTAG00000034850	*DENR*	1'734	1'113	1'393	1'502	1'285	-0.225	0.781738939	1

Ensembl gene ID	geneName	counts wildtype horn bud	counts polled horn bud	baseMean	baseMean wildtype horn bud	baseMean polled horn bud	log2FoldChange	pval	padj
ENSBTAG00000034867	*TMEM38B*	425	350	386	368	404	0.135	0.885454672	1
ENSBTAG00000034871	*protein_coding*	670	1'201	984	580	1'387	1.257	0.13238693	1
ENSBTAG00000034875	*ALPK1*	699	362	512	605	418	-0.534	0.547406377	1
ENSBTAG00000034879	*pseudogene*	4'507	4'649	4'636	3'903	5'368	0.460	0.551775131	1
ENSBTAG00000034883	*CCR4*	68	36	50	59	42	-0.503	0.779914791	1
ENSBTAG00000034901	*HIST3H2BB*	20	15	17	17	17	0.000	1	1
ENSBTAG00000034905	*BT.54259*	18	12	15	16	14	-0.170	0.983826208	1
ENSBTAG00000034918	*IFIT2*	20	19	20	17	22	0.341	0.920476198	1
ENSBTAG00000034925	*C6ORF221*	7	2	4	6	2	-1.392	0.903936064	1
ENSBTAG00000034936	*ANKIB1*	3'508	1'995	2'671	3'038	2'304	-0.399	0.611264741	1
ENSBTAG00000034949	*protein_coding*	8	2	5	7	2	-1.585	0.87391615	1
ENSBTAG00000034952	*SPAG11*	7	3	5	6	3	-0.807	0.947428618	1
ENSBTAG00000034954	*DEFB5*	84	79	82	73	91	0.327	0.821677003	1
ENSBTAG00000034963	*MKS1*	456	390	423	395	450	0.189	0.836168091	1
ENSBTAG00000034978	*FAM168B*	7'483	4'835	6'032	6'480	5'583	-0.215	0.780519545	1
ENSBTAG00000034985	*PHLDA1*	656	372	499	568	430	-0.403	0.651179523	1
ENSBTAG00000034987	*MKKS*	1'690	1'028	1'325	1'464	1'187	-0.302	0.709776257	1
ENSBTAG00000034991	*C20ORF94*	152	142	148	132	164	0.317	0.785781693	1
ENSBTAG00000034992	*MCTS1*	820	430	603	710	497	-0.516	0.552673298	1
ENSBTAG00000034995	*ITPRIPL1*	412	361	387	357	417	0.224	0.808988665	1
ENSBTAG00000034998	*C5ORF25*	1'034	691	847	895	798	-0.166	0.844000315	1
ENSBTAG00000035001	*protein_coding*	6	12	10	5	14	1.415	0.771639311	1
ENSBTAG00000035005	*pseudogene*	247	356	312	214	411	0.942	0.327277976	1
ENSBTAG00000035007	*GALNTL6*	21	0	9	18	0		0.346730876	1
ENSBTAG00000035012	*protein_coding*	0	2	1	0	2	Inf	0.939077559	1
ENSBTAG00000035013	*protein_coding*	57	58	58	49	67	0.440	0.79160047	1
ENSBTAG00000035018	*ZCCHC12*	137	154	148	119	178	0.584	0.612487254	1
ENSBTAG00000035030	*TMED7*	5'141	3'197	4'072	4'452	3'692	-0.270	0.727930768	1
ENSBTAG00000035041	*5S_rRNA*	0	1	1	0	1	Inf	0.993540919	1
ENSBTAG00000035054	*COL9A1*	757	677	719	656	782	0.254	0.765307609	1
ENSBTAG00000035064	*BT.22367*	268	113	181	232	130	-0.831	0.446691347	1
ENSBTAG00000035076	*protein_coding*	3	0	1	3	0		0.936647693	1
ENSBTAG00000035081	*SERINC2*	326	655	519	282	756	1.422	0.112379918	1
ENSBTAG00000035083	*ATXN7L3B*	10'358	7'653	8'904	8'970	8'837	-0.022	0.978311742	1
ENSBTAG00000035084	*BT.79602*	360	397	385	312	458	0.556	0.547204621	1
ENSBTAG00000035110	*CCDC67*	13	0	6	11	0		0.551229012	1
ENSBTAG00000035122	*PLCXD1*	0	1	1	0	1	Inf	0.993540919	1
ENSBTAG00000035129	*BT.56334*	97	75	85	84	87	0.044	0.983462394	1
ENSBTAG00000035144	*BT.96703*	197	126	158	171	145	-0.230	0.843833392	1
ENSBTAG00000035147	*C17ORF67*	49	16	30	42	18	-1.200	0.597010955	1
ENSBTAG00000035158	*TMEM249*	107	49	75	93	57	-0.712	0.630494596	1
ENSBTAG00000035171	*SERPINB12*	40	48	45	35	55	0.678	0.714268671	1
ENSBTAG00000035172	*pseudogene*	638	787	731	553	909	0.718	0.398363666	1
ENSBTAG00000035174	*THOC3*	627	627	633	543	724	0.415	0.630018537	1
ENSBTAG00000035175	*DONSON*	2'022	1'108	1'515	1'751	1'279	-0.453	0.573683341	1
ENSBTAG00000035182	*SERPINB13*	15	1	7	13	1	-3.492	0.607205542	1
ENSBTAG00000035183	*BT.26980*	5	1	3	4	1	-1.907	0.9188325	1
ENSBTAG00000035199	*MRPL33*	405	374	391	351	432	0.300	0.745216866	1
ENSBTAG00000035206	*pseudogene*	0	1	1	0	1	Inf	0.993540919	1
ENSBTAG00000035224	*protein_coding*	20	6	12	17	7	-1.322	0.749655668	1
ENSBTAG00000035226	*TOR1AIP1*	1'703	1'025	1'329	1'475	1'184	-0.317	0.69570851	1
ENSBTAG00000035230	*TOR1AIP2*	268	284	280	232	328	0.499	0.611226299	1
ENSBTAG00000035244	*ANKRD39*	759	714	741	657	824	0.327	0.699640222	1
ENSBTAG00000035247	*MCEE*	174	132	152	151	152	0.016	0.9944201	1
ENSBTAG00000035254	*BT.44030*	2'205	2'845	2'597	1'910	3'285	0.783	0.320036529	1
ENSBTAG00000035265	*TMEM192*	316	237	274	274	274	0.000	1	1
ENSBTAG00000035286	*BT.48986*	923	730	821	799	843	0.077	0.927920306	1
ENSBTAG00000035293	*protein_coding*	57	114	90	49	132	1.415	0.300523134	1
ENSBTAG00000035319	*BT.87489*	855	551	688	740	636	-0.219	0.799457579	1
ENSBTAG00000035323	*TMX4*	4'396	4'444	4'469	3'807	5'131	0.431	0.577371448	1
ENSBTAG00000035370	*TBPL1*	986	665	811	854	768	-0.153	0.857076619	1
ENSBTAG00000035373	*protein_coding*	3	2	2	3	2	-0.170	1	1
ENSBTAG00000035379	*pseudogene*	21'810	22'576	22'478	18'888	26'069	0.465	0.541292316	1
ENSBTAG00000035396	*OR7D2*	2	0	1	2	0		0.974934741	1
ENSBTAG00000035437	*STAG1*	2'677	2'100	2'372	2'318	2'425	0.065	0.934148804	1
ENSBTAG00000035480	*miRNA*	0	1	1	0	1	Inf	0.993540919	1
ENSBTAG00000035544	*BT.34882*	88	8	43	76	9	-3.044	0.130246179	1
ENSBTAG00000035556	*BT.56566*	10'875	5'180	7'700	9'418	5'981	-0.655	0.394866983	1
ENSBTAG00000035572	*pseudogene*	308	439	387	267	507	0.926	0.31721321	1
ENSBTAG00000035584	*BT.104452*	1'553	1'084	1'298	1'345	1'252	-0.104	0.899661794	1
ENSBTAG00000035587	*ARRB2*	1'259	1'135	1'200	1'090	1'311	0.265	0.744149421	1
ENSBTAG00000035615	*UPF3B*	1'350	522	886	1'169	603	-0.956	0.254521498	1

Ensembl gene ID	geneName	counts wildtype horn bud	counts polled horn bud	baseMean	baseMean wildtype horn bud	baseMean polled horn bud	log2FoldChange	pval	padj
ENSBTAG00000035641	*pseudogene*	11	43	30	10	50	2.382	0.306653802	1
ENSBTAG00000035660	*protein_coding*	46	18	30	40	21	-0.939	0.681717925	1
ENSBTAG00000035662	*CAMK4*	38	19	27	33	22	-0.585	0.815468474	1
ENSBTAG00000035697	*GNG12*	263	286	279	228	330	0.536	0.585030291	1
ENSBTAG00000035701	*protein_coding*	1	0	0	1	0		1	1
ENSBTAG00000035705	*MTMR8*	113	77	93	98	89	-0.138	0.926581054	1
ENSBTAG00000035706	*MYOCD*	36	48	43	31	55	0.830	0.658317507	1
ENSBTAG00000035710	*ZBBX*	9	9	9	8	10	0.415	0.953225767	1
ENSBTAG00000035726	*processed_pseudogene*	556	301	415	482	348	-0.470	0.607900422	1
ENSBTAG00000035735	*BIRC2*	412	419	420	357	484	0.439	0.629651094	1
ENSBTAG00000035744	*TRIM26*	803	765	789	695	883	0.345	0.681838121	1
ENSBTAG00000035764	*BT.71247*	3	0	1	3	0		0.936647693	1
ENSBTAG00000035776	*C6H4orf22*	11	5	8	10	6	-0.722	0.917926652	1
ENSBTAG00000035777	*protein_coding*	2	1	1	2	1	-0.585	1	1
ENSBTAG00000035782	*BT.103808*	1'163	1'122	1'151	1'007	1'296	0.363	0.656104856	1
ENSBTAG00000035785	*BT.104275*	1	0	0	1	0		1	1
ENSBTAG00000035827	*BT.64311*	504	497	505	436	574	0.395	0.656121555	1
ENSBTAG00000035836	*GLT25D2*	155	214	191	134	247	0.880	0.412277889	1
ENSBTAG00000035844	*BT.89248*	119	75	95	103	87	-0.251	0.857958286	1
ENSBTAG00000035855	*pseudogene*	3'607	7'986	6'173	3'124	9'221	1.562	0.046624989	1
ENSBTAG00000035858	*MCC*	1'931	1'456	1'677	1'672	1'681	0.008	0.992475868	1
ENSBTAG00000035868	*SIGLEC14*	15	14	15	13	16	0.316	0.945977687	1
ENSBTAG00000035882	*processed_pseudogene*	156	209	188	135	241	0.837	0.437089674	1
ENSBTAG00000035907	*DDX3X*	13'667	8'426	10'783	11'836	9'730	-0.283	0.712082347	1
ENSBTAG00000035915	*protein_coding*	3	1	2	3	1	-1.170	0.985253077	1
ENSBTAG00000035926	*protein_coding*	39'592	30'543	34'778	34'288	35'268	0.041	0.956647988	1
ENSBTAG00000035945	*protein_coding*	3	1	2	3	1	-1.170	0.985253077	1
ENSBTAG00000035958	*protein_coding*	414	570	508	359	658	0.876	0.324020146	1
ENSBTAG00000035959	*pseudogene*	47'131	35'539	40'927	40'817	41'037	0.008	0.991197523	1
ENSBTAG00000035966	*GRXCR2*	6	2	4	5	2	-1.170	0.934316631	1
ENSBTAG00000035975	*SCIMP*	5	0	2	4	0		0.853268594	1
ENSBTAG00000035990	*protein_coding*	1	0	0	1	0		1	1
ENSBTAG00000035995	*TNFAIP2*	299	188	238	259	217	-0.254	0.805743815	1
ENSBTAG00000035998	*BT.5084*	4'218	2'178	3'084	3'653	2'515	-0.539	0.491239518	1
ENSBTAG00000036009	*TRAF6*	1'081	596	812	936	688	-0.444	0.598202317	1
ENSBTAG00000036012	*OR13C3*	1	0	0	1	0		1	1
ENSBTAG00000036016	*FAM111B*	376	345	362	326	398	0.291	0.756128907	1
ENSBTAG00000036019	*DSCR6*	10	5	7	9	6	-0.585	0.94357809	1
ENSBTAG00000036028	*BEX2*	1'457	794	1'089	1'262	917	-0.461	0.575071645	1
ENSBTAG00000036061	*BT.66312*	5	9	7	4	10	1.263	0.837733706	1
ENSBTAG00000036078	*EMP1*	4'245	3'171	3'669	3'676	3'662	-0.006	0.995146037	1
ENSBTAG00000036087	*ARMC2*	218	142	176	189	164	-0.203	0.857541288	1
ENSBTAG00000036099	*protein_coding*	69	79	75	60	91	0.610	0.677211926	1
ENSBTAG00000036101	*BT.89187*	567	516	543	491	596	0.279	0.750940932	1
ENSBTAG00000036111	*pseudogene*	5'187	6'516	6'008	4'492	7'524	0.744	0.335102893	1
ENSBTAG00000036113	*BT.51155*	7	18	13	6	21	1.778	0.63434103	1
ENSBTAG00000036116	*BT.74823*	3	0	1	3	0		0.936647693	1
ENSBTAG00000036127	*AS3MT*	701	311	483	607	359	-0.757	0.397736106	1
ENSBTAG00000036154	*BT.89819*	7	1	4	6	1	-2.392	0.851750348	1
ENSBTAG00000036183	*FBXW8*	3'342	2'557	2'923	2'894	2'953	0.029	0.970394243	1
ENSBTAG00000036222	*protein_coding*	39	13	24	34	15	-1.170	0.65058657	1
ENSBTAG00000036224	*BT.86138*	21	2	10	18	2	-2.977	0.538317156	1
ENSBTAG00000036242	*RFESD*	60	49	54	52	57	0.123	0.952029459	1
ENSBTAG00000036257	*protein_coding*	35	34	35	30	39	0.373	0.867044582	1
ENSBTAG00000036258	*protein_coding*	364	143	240	315	165	-0.933	0.359936804	1
ENSBTAG00000036260	*BT.37348*	145	70	103	126	81	-0.636	0.626613477	1
ENSBTAG00000036262	*BT.52690*	1'445	930	1'163	1'251	1'074	-0.221	0.787974256	1
ENSBTAG00000036282	*CCDC59*	1'606	984	1'264	1'391	1'136	-0.292	0.720246152	1
ENSBTAG00000036287	*SAMD5*	10	0	4	9	0		0.653360153	1
ENSBTAG00000036297	*BT.62681*	525	578	561	455	667	0.554	0.526600913	1
ENSBTAG00000036298	*BT.42671*	173	61	110	150	70	-1.089	0.392843467	1
ENSBTAG00000036304	*CNGB3*	5	1	3	4	1	-1.907	0.9188325	1
ENSBTAG00000036310	*RNF146A*	364	372	372	315	430	0.446	0.63104166	1
ENSBTAG00000036349	*CCDC160*	101	33	63	87	38	-1.199	0.449925168	1
ENSBTAG00000036356	*bta-mir-329a*	1	0	0	1	0		1	1
ENSBTAG00000036358	*miRNA*	0	1	1	0	1	Inf	0.993540919	1
ENSBTAG00000036361	*bta-mir-152*	28	11	18	24	13	-0.933	0.765131313	1
ENSBTAG00000036362	*bta-mir-106b*	1	1	1	1	1	0.415	1	1
ENSBTAG00000036363	*bta-mir-133a-1*	0	2	1	0	2	Inf	0.939077559	1
ENSBTAG00000036364	*bta-mir-410*	0	3	2	0	3	Inf	0.878646179	1
ENSBTAG00000036365	*bta-mir-29e*	0	1	1	0	1	Inf	0.993540919	1
ENSBTAG00000036367	*bta-mir-374b*	281	163	216	243	188	-0.371	0.724295893	1

Ensembl gene ID	geneName	counts wildtype horn bud	counts polled horn bud	baseMean	baseMean wildtype horn bud	baseMean polled horn bud	log2FoldChange	pval	padj
ENSBTAG00000036368	*bta-mir-381*	1	2	2	1	2	1.415	0.981979269	1
ENSBTAG00000036369	*bta-mir-100*	1	0	0	1	0		1	1
ENSBTAG00000036373	*bta-mir-568*	108	20	58	94	23	-2.018	0.226372657	1
ENSBTAG00000036375	*bta-mir-582*	0	1	1	0	1	Inf	0.993540919	1
ENSBTAG00000036379	*bta-mir-379*	1	0	0	1	0		1	1
ENSBTAG00000036388	*bta-mir-483*	32	15	23	28	17	-0.678	0.809212694	1
ENSBTAG00000036396	*bta-mir-494*	1	0	0	1	0		1	1
ENSBTAG00000036397	*bta-mir-302b*	1	1	1	1	1	0.415	1	1
ENSBTAG00000036398	*bta-mir-655*	3	8	6	3	9	1.830	0.801688691	1
ENSBTAG00000036400	*miRNA*	0	1	1	0	1	Inf	0.993540919	1
ENSBTAG00000036404	*bta-mir-155*	9	2	5	8	2	-1.755	0.844314416	1
ENSBTAG00000036410	*bta-mir-141*	1	1	1	1	1	0.415	1	1
ENSBTAG00000036417	*bta-mir-3596*	18	3	10	16	3	-2.170	0.662488565	1
ENSBTAG00000036419	*bta-mir-424*	5	1	3	4	1	-1.907	0.9188325	1
ENSBTAG00000036421	*bta-mir-124a-2*	1	0	0	1	0		1	1
ENSBTAG00000036426	*bta-mir-154a*	0	1	1	0	1	Inf	0.993540919	1
ENSBTAG00000036428	*bta-mir-302a*	1	0	0	1	0		1	1
ENSBTAG00000036430	*bta-mir-29d*	5	0	2	4	0		0.853268594	1
ENSBTAG00000036433	*bta-mir-539*	4	3	3	3	3	0.000	1	1
ENSBTAG00000036435	*miRNA*	0	1	1	0	1	Inf	0.993540919	1
ENSBTAG00000036437	*bta-mir-33b*	6	2	4	5	2	-1.170	0.934316631	1
ENSBTAG00000036438	*bta-mir-708*	2	1	1	2	1	-0.585	1	1
ENSBTAG00000036444	*bta-mir-758*	1	0	0	1	0		1	1
ENSBTAG00000036445	*bta-mir-453*	1	0	0	1	0		1	1
ENSBTAG00000036488	*5S_rRNA*	2	0	1	2	0		0.974934741	1
ENSBTAG00000036578	*5S_rRNA*	5	0	2	4	0		0.853268594	1
ENSBTAG00000036625	*U2*	0	1	1	0	1	Inf	0.993540919	1
ENSBTAG00000036666	*5S_rRNA*	1	0	0	1	0		1	1
ENSBTAG00000036732	*5S_rRNA*	1	0	0	1	0		1	1
ENSBTAG00000036743	*5S_rRNA*	0	1	1	0	1	Inf	0.993540919	1
ENSBTAG00000036744	*5S_rRNA*	1	0	0	1	0		1	1
ENSBTAG00000036766	*U1*	1	0	0	1	0		1	1
ENSBTAG00000036858	*U3*	0	1	1	0	1	Inf	0.993540919	1
ENSBTAG00000036929	*U3*	0	1	1	0	1	Inf	0.993540919	1
ENSBTAG00000036993	*U2*	3	0	1	3	0		0.936647693	1
ENSBTAG00000036997	*U1*	1	0	0	1	0		1	1
ENSBTAG00000037013	*U1*	0	5	3	0	6	Inf	0.757530434	1
ENSBTAG00000037087	*5S_rRNA*	3	10	7	3	12	2.152	0.727540088	1
ENSBTAG00000037108	*5S_rRNA*	1	0	0	1	0		1	1
ENSBTAG00000037110	*U12*	7	2	4	6	2	-1.392	0.903936064	1
ENSBTAG00000037179	*U3*	5	8	7	4	9	1.093	0.872919157	1
ENSBTAG00000037187	*U1*	1	0	0	1	0		1	1
ENSBTAG00000037229	*5S_rRNA*	1	0	0	1	0		1	1
ENSBTAG00000037261	*miRNA*	0	1	1	0	1	Inf	0.993540919	1
ENSBTAG00000037265	*bta-mir-665*	3	1	2	3	1	-1.170	0.985253077	1
ENSBTAG00000037291	*bta-mir-875*	0	1	1	0	1	Inf	0.993540919	1
ENSBTAG00000037292	*bta-mir-873*	1	0	0	1	0		1	1
ENSBTAG00000037304	*miRNA*	3	0	1	3	0		0.936647693	1
ENSBTAG00000037319	*miRNA*	41	15	26	36	17	-1.036	0.67517422	1
ENSBTAG00000037339	*bta-mir-208b*	1	1	1	1	1	0.415	1	1
ENSBTAG00000037348	*bta-mir-940*	13	5	9	11	6	-0.963	0.868297704	1
ENSBTAG00000037354	*miRNA*	1	1	1	1	1	0.415	1	1
ENSBTAG00000037375	*BT.62337*	2'143	990	1'500	1'856	1'143	-0.699	0.385886106	1
ENSBTAG00000037377	*ABHD14B*	4'122	4'143	4'177	3'570	4'784	0.422	0.585247836	1
ENSBTAG00000037381	*BT.41777*	2	0	1	2	0		0.974934741	1
ENSBTAG00000037383	*AKAP13*	842	1'656	1'321	729	1'912	1.391	0.090305118	1
ENSBTAG00000037389	*TRIM44*	337	249	290	292	288	-0.022	0.987176004	1
ENSBTAG00000037393	*ZNF639*	1'596	764	1'132	1'382	882	-0.648	0.429608718	1
ENSBTAG00000037397	*PRSS36*	16	27	23	14	31	1.170	0.661901171	1
ENSBTAG00000037399	*protein_coding*	378	99	221	327	114	-1.518	0.148378081	1
ENSBTAG00000037400	*TNRC18*	6'856	14'301	11'225	5'937	16'513	1.476	0.057904358	1
ENSBTAG00000037404	*LRFN4*	300	376	347	260	434	0.741	0.431409114	1
ENSBTAG00000037410	*protein_coding*	3	2	2	3	2	-0.170	1	1
ENSBTAG00000037413	*BT.37166*	553	633	605	479	731	0.610	0.481657298	1
ENSBTAG00000037415	*COX7B*	930	1'221	1'108	805	1'410	0.808	0.325336475	1
ENSBTAG00000037418	*KISS1R*	6	4	5	5	5	-0.170	1	1
ENSBTAG00000037421	*protein_coding*	3	0	1	3	0		0.936647693	1
ENSBTAG00000037424	*protein_coding*	0	1	1	0	1	Inf	0.993540919	1
ENSBTAG00000037426	*protein_coding*	1	0	0	1	0		1	1
ENSBTAG00000037429	*HTR1B*	27	13	19	23	15	-0.639	0.839610081	1
ENSBTAG00000037434	*processed_pseudogene*	377	441	418	326	509	0.641	0.482024323	1
ENSBTAG00000037440	*protein_coding*	1'723	925	1'280	1'492	1'068	-0.482	0.552899149	1

Ensembl gene ID	geneName	counts wildtype horn bud	counts polled horn bud	baseMean	baseMean wildtype horn bud	baseMean polled horn bud	log2FoldChange	pval	padj
ENSBTAG00000037449	*ACSM5*	9	1	4	8	1	-2.755	0.785977687	1
ENSBTAG00000037452	*BT.73361*	98	48	70	85	55	-0.615	0.68676514	1
ENSBTAG00000037456	*AHDC1*	2'369	3'746	3'189	2'052	4'326	1.076	0.171130176	1
ENSBTAG00000037457	*MBD2*	3'126	1'229	2'063	2'707	1'419	-0.932	0.242021773	1
ENSBTAG00000037461	*SDHAF1*	402	427	421	348	493	0.502	0.581470581	1
ENSBTAG00000037465	*BT.51503*	380	106	226	329	122	-1.427	0.17107849	1
ENSBTAG00000037483	*BT.61395*	80	38	57	69	44	-0.659	0.694125098	1
ENSBTAG00000037488	*JRKL*	757	505	619	656	583	-0.169	0.847036413	1
ENSBTAG00000037489	*CRCP*	313	320	320	271	370	0.447	0.639921436	1
ENSBTAG00000037490	*protein_coding*	7	8	8	6	9	0.608	0.932722577	1
ENSBTAG00000037493	*BT.56130*	5'203	7'504	6'585	4'506	8'665	0.943	0.222613262	1
ENSBTAG00000037494	*RGS21*	1	2	2	1	2	1.415	0.981979269	1
ENSBTAG00000037500	*miRNA*	60	35	46	52	40	-0.363	0.850925472	1
ENSBTAG00000037508	*EBF1*	985	798	887	853	921	0.111	0.894158334	1
ENSBTAG00000037510	*BT.41393*	16	4	9	14	5	-1.585	0.756102726	1
ENSBTAG00000037516	*pseudogene*	13'318	11'658	12'498	11'534	13'461	0.223	0.769580824	1
ENSBTAG00000037523	*B3GALT6*	120	183	158	104	211	1.024	0.366025841	1
ENSBTAG00000037527	*BT.106205*	127	9	60	110	10	-3.404	0.052036406	1
ENSBTAG00000037531	*pseudogene*	352	390	378	305	450	0.563	0.543687242	1
ENSBTAG00000037533	*BT.105154*	1'836	1'124	1'444	1'590	1'298	-0.293	0.716910985	1
ENSBTAG00000037534	*KLRF1*	4	0	2	3	0		0.89545886	1
ENSBTAG00000037537	*protein_coding*	32	3	16	28	3	-3.000	0.401992648	1
ENSBTAG00000037539	*VCAM1*	2'312	1'046	1'605	2'002	1'208	-0.729	0.364158757	1
ENSBTAG00000037549	*BT.59351*	1'315	956	1'121	1'139	1'104	-0.045	0.958022633	1
ENSBTAG00000037552	*protein_coding*	9	4	6	8	5	-0.755	0.931866093	1
ENSBTAG00000037553	*KRT33A*	4	11	8	3	13	1.874	0.733957485	1
ENSBTAG00000037554	*BT.89063*	98	19	53	85	22	-1.952	0.25951471	1
ENSBTAG00000037555	*FAM183A*	27	0	12	23	0		0.244698433	1
ENSBTAG00000037558	*CXCL2*	18	9	13	16	10	-0.585	0.894065104	1
ENSBTAG00000037559	*BT.68523*	10	10	10	9	12	0.415	0.946590905	1
ENSBTAG00000037560	*BT.93348*	0	2	1	0	2	Inf	0.939077559	1
ENSBTAG00000037565	*MGC152321*	76	23	46	66	27	-1.309	0.473710244	1
ENSBTAG00000037566	*BT.43579*	741	923	854	642	1'066	0.732	0.382257195	1
ENSBTAG00000037571	*EBAG9*	1'627	1'237	1'419	1'409	1'428	0.020	0.980801512	1
ENSBTAG00000037576	*Mrz 11*	49	4	24	42	5	-3.200	0.252071399	1
ENSBTAG00000037578	*BT.62519*	22	40	33	19	46	1.278	0.554419977	1
ENSBTAG00000037580	*BT.103550*	168	75	116	145	87	-0.748	0.549689916	1
ENSBTAG00000037581	*MZF1*	3'279	2'899	3'094	2'840	3'347	0.237	0.760450107	1
ENSBTAG00000037582	*ZNF865*	85	86	86	74	99	0.432	0.757802271	1
ENSBTAG00000037586	*pseudogene*	4	0	2	3	0		0.89545886	1
ENSBTAG00000037595	*ZSCAN22*	141	100	119	122	115	-0.081	0.955977447	1
ENSBTAG00000037596	*5S_rRNA*	1	0	0	1	0		1	1
ENSBTAG00000037600	*RPS15A*	22'114	11'616	16'282	19'151	13'413	-0.514	0.501367298	1
ENSBTAG00000037604	*EFNB3*	354	327	342	307	378	0.301	0.750738773	1
ENSBTAG00000037605	*BOLA-DQA1*	1'859	492	1'089	1'610	568	-1.503	0.071739521	1
ENSBTAG00000037608	*BT.66080*	105	109	108	91	126	0.469	0.714551921	1
ENSBTAG00000037616	*KCNJ6*	8	1	4	7	1	-2.585	0.818599781	1
ENSBTAG00000037624	*miRNA*	1	0	0	1	0		1	1
ENSBTAG00000037625	*protein_coding*	69	91	82	60	105	0.814	0.563372626	1
ENSBTAG00000037626	*5_8S_rRNA*	1	0	0	1	0		1	1
ENSBTAG00000037632	*BT.103492*	215	206	212	186	238	0.353	0.736347718	1
ENSBTAG00000037634	*protein_coding*	10	4	7	9	5	-0.907	0.905171387	1
ENSBTAG00000037640	*PID1*	3'007	2'664	2'840	2'604	3'076	0.240	0.758155094	1
ENSBTAG00000037644	*BT.102944*	5'324	3'584	4'375	4'611	4'138	-0.156	0.841091387	1
ENSBTAG00000037649	*VIPR2*	1'075	1'755	1'479	931	2'026	1.122	0.166459019	1
ENSBTAG00000037650	*RGAG1*	50	42	46	43	48	0.163	0.93907155	1
ENSBTAG00000037651	*S100A2*	2'057	1'606	1'818	1'781	1'854	0.058	0.941873525	1
ENSBTAG00000037657	*protein_coding*	2	2	2	2	2	0.415	1	1
ENSBTAG00000037661	*DENND1B*	55	26	39	48	30	-0.666	0.742322803	1
ENSBTAG00000037662	*protein_coding*	0	3	2	0	3	Inf	0.878646179	1
ENSBTAG00000037665	*protein_coding*	0	2	1	0	2	Inf	0.939077559	1
ENSBTAG00000037673	*GSTM4*	137	139	140	119	161	0.436	0.711510597	1
ENSBTAG00000037679	*PPP1R3E*	84	58	70	73	67	-0.119	0.947511193	1
ENSBTAG00000037686	*SRPX2*	3'313	3'119	3'235	2'869	3'602	0.328	0.673350466	1
ENSBTAG00000037687	*GPR111*	46	42	44	40	48	0.284	0.886975646	1
ENSBTAG00000037693	*MUC16*	3	0	1	3	0		0.936647693	1
ENSBTAG00000037699	*protein_coding*	27	6	15	23	7	-1.755	0.616167115	1
ENSBTAG00000037702	*SP140L*	198	138	165	171	159	-0.106	0.930581471	1
ENSBTAG00000037703	*MICALL1*	2'312	4'013	3'318	2'002	4'634	1.211	0.124307308	1
ENSBTAG00000037709	*pseudogene*	119	167	148	103	193	0.904	0.432834123	1
ENSBTAG00000037717	*PCDHGA7*	135	147	143	117	170	0.538	0.644404831	1
ENSBTAG00000037721	*protein_coding*	287	101	183	249	117	-1.092	0.317621643	1

Ensembl gene ID	geneName	counts wildtype horn bud	counts polled horn bud	baseMean	baseMean wildtype horn bud	baseMean polled horn bud	log2FoldChange	pval	padj
ENSBTAG00000037726	*PLCD1*	5'109	3'303	4'119	4'425	3'814	-0.214	0.782870818	1
ENSBTAG00000037729	*LASS1*	696	1'037	900	603	1'197	0.990	0.23675251	1
ENSBTAG00000037735	*GPR77*	197	65	123	171	75	-1.185	0.335466315	1
ENSBTAG00000037743	*BT.99192*	25	12	18	22	14	-0.644	0.847278186	1
ENSBTAG00000037746	*SNRNP27*	1'475	790	1'095	1'277	912	-0.486	0.554357573	1
ENSBTAG00000037756	*TET1*	260	186	220	225	215	-0.068	0.952894919	1
ENSBTAG00000037757	*EBF4*	788	1'010	924	682	1'166	0.773	0.352993485	1
ENSBTAG00000037765	*TNFAIP8L1*	512	580	557	443	670	0.595	0.496869507	1
ENSBTAG00000037773	*BT.74563*	6	0	3	5	0		0.81115604	1
ENSBTAG00000037775	*protein_coding*	6'989	4'480	5'613	6'053	5'173	-0.227	0.769340593	1
ENSBTAG00000037778	*CXCL2*	126	38	76	109	44	-1.314	0.369096642	1
ENSBTAG00000037781	*BT.24583*	147	142	146	127	164	0.365	0.754424556	1
ENSBTAG00000037786	*NALCN*	271	133	194	235	154	-0.612	0.568518162	1
ENSBTAG00000037791	*protein_coding*	3	0	1	3	0		0.936647693	1
ENSBTAG00000037794	*MYH4*	9	89	55	8	103	3.721	0.041924403	1
ENSBTAG00000037795	*CYP2C87*	32	23	27	28	27	-0.061	0.998655064	1
ENSBTAG00000037799	*BT.93022*	74	13	40	64	15	-2.094	0.296592536	1
ENSBTAG00000037800	*BT.48025*	2	1	1	2	1	-0.585	1	1
ENSBTAG00000037803	*ZNF197*	1'031	649	821	893	749	-0.253	0.764669345	1
ENSBTAG00000037804	*BT.104763*	42	9	23	36	10	-1.807	0.495680066	1
ENSBTAG00000037811	*CCL2*	826	222	486	715	256	-1.481	0.102295668	1
ENSBTAG00000037813	*IFRG15*	3'689	2'077	2'797	3'195	2'398	-0.414	0.597871364	1
ENSBTAG00000037815	*HBE1*	0	1	1	0	1	Inf	0.993540919	1
ENSBTAG00000037818	*pseudogene*	1	0	0	1	0		1	1
ENSBTAG00000037819	*SFT2D2*	339	392	373	294	453	0.625	0.501396686	1
ENSBTAG00000037821	*protein_coding*	5	0	2	4	0		0.853268594	1
ENSBTAG00000037824	*protein_coding*	1	0	0	1	0		1	1
ENSBTAG00000037826	*BT.44982*	1	0	0	1	0		1	1
ENSBTAG00000037830	*protein_coding*	2	1	1	2	1	-0.585	1	1
ENSBTAG00000037832	*ASPHD2*	328	419	384	284	484	0.768	0.406485053	1
ENSBTAG00000037844	*CDH18*	113	14	57	98	16	-2.598	0.131594725	1
ENSBTAG00000037846	*miRNA*	0	3	2	0	3	Inf	0.878646179	1
ENSBTAG00000037856	*MGC152281*	14	12	13	12	14	0.193	0.980949072	1
ENSBTAG00000037858	*BT.63162*	176	28	92	152	32	-2.237	0.109293073	1
ENSBTAG00000037868	*miRNA*	21	13	17	18	15	-0.277	0.951008818	1
ENSBTAG00000037875	*protein_coding*	1	0	0	1	0		1	1
ENSBTAG00000037882	*ZNF584*	95	187	149	82	216	1.392	0.229405263	1
ENSBTAG00000037885	*PCDHB1*	1	1	1	1	1	0.415	1	1
ENSBTAG00000037888	*LYPD5*	31	23	27	27	27	-0.016	1	1
ENSBTAG00000037896	*protein_coding*	16	13	14	14	15	0.115	0.995982734	1
ENSBTAG00000037899	*BT.65303*	33'817	9'564	20'165	29'286	11'044	-1.407	0.069148484	1
ENSBTAG00000037902	*BT.38978*	168	160	165	145	185	0.345	0.759264431	1
ENSBTAG00000037905	*bta-mir-1271*	2	7	5	2	8	2.222	0.797845137	1
ENSBTAG00000037906	*BT.35363*	105	68	85	91	79	-0.212	0.887355897	1
ENSBTAG00000037907	*DSEL*	3'139	2'049	2'542	2'718	2'366	-0.200	0.799404434	1
ENSBTAG00000037913	*pseudogene*	1'797	1'584	1'693	1'556	1'829	0.233	0.769980287	1
ENSBTAG00000037917	*SLC17A1*	0	1	1	0	1	Inf	0.993540919	1
ENSBTAG00000037921	*OVCH2*	23	13	17	20	15	-0.408	0.912458883	1
ENSBTAG00000037925	*pseudogene*	1'229	2'524	1'989	1'064	2'914	1.453	0.071246971	1
ENSBTAG00000037929	*ADAM28*	6	1	3	5	1	-2.170	0.885240772	1
ENSBTAG00000037931	*RPL39*	2'367	1'936	2'143	2'050	2'236	0.125	0.873917193	1
ENSBTAG00000037932	*protein_coding*	2	1	1	2	1	-0.585	1	1
ENSBTAG00000037935	*BT.87407*	880	1'263	1'110	762	1'458	0.936	0.255130776	1
ENSBTAG00000037937	*BT.97476*	64	4	30	55	5	-3.585	0.149745064	1
ENSBTAG00000037938	*MGC142695*	625	305	447	541	352	-0.620	0.493480724	1
ENSBTAG00000037941	*QTRT1*	1'579	1'197	1'375	1'367	1'382	0.015	0.985050236	1
ENSBTAG00000037942	*BT.88006*	403	690	573	349	797	1.191	0.175918818	1
ENSBTAG00000037945	*CASKIN1*	657	365	495	569	421	-0.433	0.627670104	1
ENSBTAG00000037951	*BT.86196*	43	19	30	37	22	-0.763	0.744681388	1
ENSBTAG00000037953	*bta-mir-1282*	103	62	80	89	72	-0.317	0.830441261	1
ENSBTAG00000037962	*BT.102365*	439	271	347	380	313	-0.281	0.767730371	1
ENSBTAG00000037964	*HIST3H2A*	131	24	71	113	28	-2.033	0.186494096	1
ENSBTAG00000037965	*ZSCAN23*	76	43	58	66	50	-0.407	0.811011549	1
ENSBTAG00000037966	*pseudogene*	1'442	1'016	1'211	1'249	1'173	-0.090	0.913391145	1
ENSBTAG00000037968	*PCDHGB2*	66	76	72	57	88	0.619	0.678225161	1
ENSBTAG00000037971	*protein_coding*	51	38	44	44	44	-0.009	1	1
ENSBTAG00000037972	*BT.88477*	1'357	539	899	1'175	622	-0.917	0.27344113	1
ENSBTAG00000037980	*TSEN2*	696	487	583	603	562	-0.100	0.910951755	1
ENSBTAG00000037981	*ZNF416*	274	218	245	237	252	0.085	0.936016062	1
ENSBTAG00000037983	*CEACAM16*	16	5	10	14	6	-1.263	0.797689913	1
ENSBTAG00000037984	*pseudogene*	1	0	0	1	0		1	1
ENSBTAG00000037985	*CHRM5*	16	12	14	14	14	0.000	1	1

Ensembl gene ID	geneName	counts wildtype horn bud	counts polled horn bud	baseMean	baseMean wildtype horn bud	baseMean polled horn bud	log2FoldChange	pval	padj
ENSBTAG00000037988	*ZNF323*	336	411	383	291	475	0.706	0.445754718	1
ENSBTAG00000037989	*protein_coding*	33	12	21	29	14	-1.044	0.711991345	1
ENSBTAG00000037991	*BT.64113*	1'658	487	999	1'436	562	-1.352	0.106093119	1
ENSBTAG00000037996	*ARMCX3*	6'190	3'151	4'500	5'361	3'638	-0.559	0.470940601	1
ENSBTAG00000038011	*SH3RF3*	596	832	738	516	961	0.896	0.292269573	1
ENSBTAG00000038020	*BT.41720*	25	28	27	22	32	0.579	0.816473743	1
ENSBTAG00000038025	*BT.76000*	8'334	2'690	5'162	7'217	3'106	-1.216	0.119280197	1
ENSBTAG00000038033	*BT.91778*	2'861	3'020	2'982	2'478	3'487	0.493	0.527817032	1
ENSBTAG00000038034	*ZNF628*	205	303	264	178	350	0.979	0.325749851	1
ENSBTAG00000038042	*BT.87187*	7	7	7	6	8	0.415	0.967915805	1
ENSBTAG00000038043	*BT.87418*	17	1	8	15	1	-3.672	0.555170792	1
ENSBTAG00000038045	*FLT3LG*	295	255	275	255	294	0.205	0.837009804	1
ENSBTAG00000038047	*H2AFX*	809	1'181	1'032	701	1'364	0.961	0.245593785	1
ENSBTAG00000038048	*BT.41060*	1'627	1'139	1'362	1'409	1'315	-0.099	0.903498756	1
ENSBTAG00000038050	*BT.35008*	958	570	744	830	658	-0.334	0.694612125	1
ENSBTAG00000038051	*BT.30326*	485	847	699	420	978	1.219	0.156636858	1
ENSBTAG00000038054	*protein_coding*	1	0	0	1	0		1	1
ENSBTAG00000038055	*KLF16*	183	309	258	158	357	1.171	0.243568199	1
ENSBTAG00000038062	*FUT4*	252	296	280	218	342	0.647	0.50929716	1
ENSBTAG00000038063	*LIPJ*	35	20	27	30	23	-0.392	0.883987423	1
ENSBTAG00000038067	*MT1A*	5	0	2	4	0		0.853268594	1
ENSBTAG00000038073	*bta-mir-1247*	1	0	0	1	0		1	1
ENSBTAG00000038074	*GJC3*	337	55	178	292	64	-2.200	0.052243954	1
ENSBTAG00000038079	*C16H1ORF21*	1'686	1'662	1'690	1'460	1'919	0.394	0.620973134	1
ENSBTAG00000038084	*NAT6*	362	256	305	314	296	-0.085	0.934060898	1
ENSBTAG00000038085	*BT.63195*	268	179	219	232	207	-0.167	0.876174748	1
ENSBTAG00000038088	*ZNF814*	184	146	164	159	169	0.081	0.946801199	1
ENSBTAG00000038090	*pseudogene*	1	1	1	1	1	0.415	1	1
ENSBTAG00000038093	*BT.41908*	3'558	3'678	3'664	3'081	4'247	0.463	0.551106626	1
ENSBTAG00000038104	*BT.90489*	21'400	18'621	20'017	18'533	21'502	0.214	0.777616822	1
ENSBTAG00000038107	*MAPKAPK2*	2'044	3'212	2'740	1'770	3'709	1.067	0.176544098	1
ENSBTAG00000038111	*protein_coding*	61	177	129	53	204	1.952	0.112467617	1
ENSBTAG00000038112	*BT.27399*	329	307	320	285	354	0.315	0.742189295	1
ENSBTAG00000038115	*FLYWCH2*	122	90	105	106	104	-0.024	0.994772279	1
ENSBTAG00000038116	*TGOLN2*	1'060	942	1'003	918	1'088	0.245	0.766552759	1
ENSBTAG00000038117	*MGC138057*	428	371	400	371	428	0.209	0.821160828	1
ENSBTAG00000038124	*BT.102404*	19	26	23	16	30	0.868	0.744004518	1
ENSBTAG00000038125	*protein_coding*	1	0	0	1	0		1	1
ENSBTAG00000038126	*WIPF2*	500	573	547	433	662	0.612	0.485845289	1
ENSBTAG00000038131	*ABCC5*	2'517	725	1'508	2'180	837	-1.381	0.090362061	1
ENSBTAG00000038132	*BT.51483*	276	99	177	239	114	-1.064	0.334083539	1
ENSBTAG00000038134	*ZDHHC1*	2'154	2'285	2'252	1'865	2'638	0.500	0.525742794	1
ENSBTAG00000038138	*BT.82239*	0	1	1	0	1	Inf	0.993540919	1
ENSBTAG00000038139	*BT.76302*	3'804	2'967	3'360	3'294	3'426	0.057	0.941771428	1
ENSBTAG00000038141	*protein_coding*	1'416	2'426	2'014	1'226	2'801	1.192	0.136460493	1
ENSBTAG00000038146	*MGC152344*	76	59	67	66	68	0.050	0.984253269	1
ENSBTAG00000038148	*ANKRD56*	51	25	37	44	29	-0.614	0.770711955	1
ENSBTAG00000038149	*NLRP12*	5	0	2	4	0		0.853268594	1
ENSBTAG00000038151	*ELOF1*	1'729	1'605	1'675	1'497	1'853	0.308	0.699646353	1
ENSBTAG00000038154	*HSH2D*	38	11	23	33	13	-1.373	0.608817883	1
ENSBTAG00000038155	*U1*	3	7	5	3	8	1.637	0.840440358	1
ENSBTAG00000038156	*DYNLT3*	4'925	2'330	3'478	4'265	2'690	-0.665	0.394440049	1
ENSBTAG00000038159	*protein_coding*	1	0	0	1	0		1	1
ENSBTAG00000038171	*CFHR5*	2	0	1	2	0		0.974934741	1
ENSBTAG00000038173	*BT.88561*	20	11	15	17	13	-0.447	0.913909355	1
ENSBTAG00000038174	*processed_pseudogene*	56	80	70	48	92	0.930	0.535520483	1
ENSBTAG00000038178	*ZNF25*	2'093	1'012	1'491	1'813	1'169	-0.633	0.432044702	1
ENSBTAG00000038181	*KIAA1797*	1'733	1'017	1'338	1'501	1'174	-0.354	0.662527893	1
ENSBTAG00000038183	*protein_coding*	11	8	9	10	9	-0.044	1	1
ENSBTAG00000038186	*GLRX*	997	1'051	1'039	863	1'214	0.491	0.550391884	1
ENSBTAG00000038189	*BT.92874*	33	10	20	29	12	-1.307	0.653206662	1
ENSBTAG00000038190	*SMAP1*	1'123	859	982	973	992	0.028	0.973210989	1
ENSBTAG00000038195	*BT.81816*	4'878	2'661	3'649	4'224	3'073	-0.459	0.555293478	1
ENSBTAG00000038204	*protein_coding*	7	1	4	6	1	-2.392	0.851750348	1
ENSBTAG00000038207	*miRNA*	0	1	1	0	1	Inf	0.993540919	1
ENSBTAG00000038215	*CLCA2*	449	243	335	389	281	-0.471	0.620756309	1
ENSBTAG00000038221	*EFNA2*	1'079	641	837	934	740	-0.336	0.689243082	1
ENSBTAG00000038224	*GDF7*	2	0	1	2	0		0.974934741	1
ENSBTAG00000038228	*FAM127A*	1'796	2'256	2'080	1'555	2'605	0.744	0.348143796	1
ENSBTAG00000038232	*protein_coding*	6	2	4	5	2	-1.170	0.934316631	1
ENSBTAG00000038234	*BHMT2*	11	7	9	10	8	-0.237	0.988863286	1
ENSBTAG00000038235	*NOD1*	244	265	259	211	306	0.534	0.59236473	1

Ensembl gene ID	geneName	counts wildtype horn bud	counts polled horn bud	baseMean	baseMean wildtype horn bud	baseMean polled horn bud	log2FoldChange	pval	padj
ENSBTAG00000038237	*KPRP*	9	1	4	8	1	-2.755	0.785977687	1
ENSBTAG00000038238	*BT.106027*	13	92	59	11	106	3.238	0.062165281	1
ENSBTAG00000038240	*ZNF134*	1'053	537	766	912	620	-0.556	0.511223297	1
ENSBTAG00000038241	*REPIN1*	543	240	374	470	277	-0.763	0.413156931	1
ENSBTAG00000038242	*bta-mir-1296*	1	0	0	1	0		1	1
ENSBTAG00000038249	*OR5C1*	1	0	0	1	0		1	1
ENSBTAG00000038251	*NAA16*	620	449	528	537	518	-0.051	0.957306872	1
ENSBTAG00000038258	*protein_coding*	95	29	58	82	33	-1.297	0.430732995	1
ENSBTAG00000038260	*AKR1E2*	87	47	65	75	54	-0.473	0.766708291	1
ENSBTAG00000038262	*MUC5AC*	3	4	4	3	5	0.830	0.962490925	1
ENSBTAG00000038263	*BT.60932*	1	1	1	1	1	0.415	1	1
ENSBTAG00000038272	*SOX1*	17	0	7	15	0		0.437564044	1
ENSBTAG00000038275	*BT.91845*	431	371	401	373	428	0.199	0.829670591	1
ENSBTAG00000038277	*HIST1H2BJ*	16	2	8	14	2	-2.585	0.65347607	1
ENSBTAG00000038283	*C4H7orf23*	604	247	404	523	285	-0.875	0.34213979	1
ENSBTAG00000038284	*ZNF496*	459	515	496	398	595	0.581	0.513335955	1
ENSBTAG00000038286	*BT.72838*	90	28	55	78	32	-1.269	0.450322313	1
ENSBTAG00000038288	*BT.79356*	4	1	2	3	1	-1.585	0.952247636	1
ENSBTAG00000038292	*C1ORF216*	157	112	133	136	129	-0.072	0.959251943	1
ENSBTAG00000038298	*C22H3ORF19*	1'096	866	975	949	1'000	0.075	0.927956664	1
ENSBTAG00000038306	*BT.45582*	415	878	687	359	1'014	1.496	0.084804651	1
ENSBTAG00000038309	*protein_coding*	1	0	0	1	0		1	1
ENSBTAG00000038312	*MS4A18*	1	0	0	1	0		1	1
ENSBTAG00000038316	*GPATCH8*	2'655	2'136	2'383	2'299	2'466	0.101	0.897321511	1
ENSBTAG00000038317	*protein_coding*	1	2	2	1	2	1.415	0.981979269	1
ENSBTAG00000038318	*FAM123C*	40	0	17	35	0		0.116452486	1
ENSBTAG00000038321	*BT.28410*	155	177	169	134	204	0.607	0.584247876	1
ENSBTAG00000038322	*BT.46254*	104	69	85	90	80	-0.177	0.907547238	1
ENSBTAG00000038323	*BT.62101*	35	103	75	30	119	1.972	0.186395814	1
ENSBTAG00000038325	*CES4A*	105	40	69	91	46	-0.977	0.52224875	1
ENSBTAG00000038326	*protein_coding*	24	14	18	21	16	-0.363	0.920962882	1
ENSBTAG00000038327	*protein_coding*	104	61	80	90	70	-0.355	0.809697517	1
ENSBTAG00000038330	*protein_coding*	1'153	806	965	999	931	-0.102	0.904154694	1
ENSBTAG00000038333	*BT.91499*	3'158	2'819	2'995	2'735	3'255	0.251	0.747172706	1
ENSBTAG00000038335	*IGBP1*	350	596	496	303	688	1.183	0.186887537	1
ENSBTAG00000038337	*PACAP*	34	26	30	29	30	0.028	1	1
ENSBTAG00000038340	*CLNK*	8	2	5	7	2	-1.585	0.87391615	1
ENSBTAG00000038351	*protein_coding*	487	277	371	422	320	-0.399	0.66959988	1
ENSBTAG00000038361	*SERPINA11*	2	0	1	2	0		0.974934741	1
ENSBTAG00000038366	*protein_coding*	0	1	1	0	1	Inf	0.993540919	1
ENSBTAG00000038368	*protein_coding*	1'034	671	835	895	775	-0.209	0.804695628	1
ENSBTAG00000038370	*miRNA*	1	0	0	1	0		1	1
ENSBTAG00000038375	*BT.87711*	3'891	3'565	3'743	3'370	4'117	0.289	0.70937401	1
ENSBTAG00000038377	*ADAM20*	30	23	26	26	27	0.032	1	1
ENSBTAG00000038379	*protein_coding*	35'041	19'736	26'568	30'346	22'789	-0.413	0.58775727	1
ENSBTAG00000038381	*BT.103276*	1'271	1'422	1'371	1'101	1'642	0.577	0.475048561	1
ENSBTAG00000038384	*KRT5*	45'959	66'831	58'486	39'802	77'170	0.955	0.211493519	1
ENSBTAG00000038385	*protein_coding*	1	0	0	1	0		1	1
ENSBTAG00000038407	*processed_pseudogene*	267	629	479	231	726	1.651	0.069850093	1
ENSBTAG00000038409	*MBD3*	1'028	1'534	1'331	890	1'771	0.992	0.222693571	1
ENSBTAG00000038411	*processed_pseudogene*	1'250	1'444	1'375	1'083	1'667	0.623	0.440579248	1
ENSBTAG00000038412	*BPIFB4*	4	0	2	3	0		0.89545886	1
ENSBTAG00000038415	*SLC6A12*	286	73	166	248	84	-1.555	0.169355245	1
ENSBTAG00000038421	*NPY5R*	31	18	24	27	21	-0.369	0.900607694	1
ENSBTAG00000038422	*processed_pseudogene*	6	0	3	5	0		0.81115604	1
ENSBTAG00000038423	*bta-mir-376a*	0	1	1	0	1	Inf	0.993540919	1
ENSBTAG00000038428	*RELB*	469	565	529	406	652	0.684	0.438203884	1
ENSBTAG00000038430	*processed_pseudogene*	4'378	4'152	4'293	3'791	4'794	0.339	0.661340476	1
ENSBTAG00000038433	*protein_coding*	0	11	6	0	13	Inf	0.464170207	1
ENSBTAG00000038434	*ATRX*	1'844	1'356	1'581	1'597	1'566	-0.028	0.973222643	1
ENSBTAG00000038438	*protein_coding*	820	1'147	1'017	710	1'324	0.899	0.27723769	1
ENSBTAG00000038439	*H1F0*	10'667	10'635	10'759	9'238	12'280	0.411	0.590789942	1
ENSBTAG00000038440	*protein_coding*	5	0	2	4	0		0.853268594	1
ENSBTAG00000038442	*WDR87*	78	39	56	68	45	-0.585	0.72883628	1
ENSBTAG00000038444	*protein_coding*	0	2	1	0	2	Inf	0.939077559	1
ENSBTAG00000038449	*protein_coding*	1	0	0	1	0		1	1
ENSBTAG00000038461	*protein_coding*	1	0	0	1	0		1	1
ENSBTAG00000038462	*TMEM182*	29	87	63	25	100	2.000	0.212239346	1
ENSBTAG00000038464	*PLIN5*	14	4	8	12	5	-1.392	0.803716462	1
ENSBTAG00000038476	*HIST2H2AB*	2	2	2	2	2	0.415	1	1
ENSBTAG00000038477	*GGCX*	11'930	7'118	9'275	10'332	8'219	-0.330	0.66702301	1
ENSBTAG00000038480	*FPGT*	1'317	518	869	1'141	598	-0.931	0.267571139	1

Ensembl gene ID	geneName	counts wildtype horn bud	counts polled horn bud	baseMean	baseMean wildtype horn bud	baseMean polled horn bud	log2FoldChange	pval	padj
ENSBTAG00000038486	*BT.102765*	18	11	14	16	13	-0.295	0.954936444	1
ENSBTAG00000038487	*ZNF613*	938	387	630	812	447	-0.862	0.319903052	1
ENSBTAG00000038488	*BT.65336*	14'421	13'262	13'901	12'489	15'314	0.294	0.699230631	1
ENSBTAG00000038494	*BT.73383*	29	15	21	25	17	-0.536	0.858771285	1
ENSBTAG00000038495	*DCLK1*	5'021	5'079	5'107	4'348	5'865	0.432	0.575636922	1
ENSBTAG00000038496	*CR2*	9	57	37	8	66	3.078	0.150824532	1
ENSBTAG00000038498	*ZBTB39*	180	276	237	156	319	1.032	0.311819058	1
ENSBTAG00000038500	*protein_coding*	41	25	32	36	29	-0.299	0.904119937	1
ENSBTAG00000038504	*BT.105623*	0	1	1	0	1	Inf	0.993540919	1
ENSBTAG00000038510	*protein_coding*	9	5	7	8	6	-0.433	0.969858938	1
ENSBTAG00000038512	*LIPI*	17	1	8	15	1	-3.672	0.555170792	1
ENSBTAG00000038520	*TMPRSS11E*	313	105	196	271	121	-1.161	0.279467903	1
ENSBTAG00000038523	*BT.106410*	27	18	22	23	21	-0.170	0.968634064	1
ENSBTAG00000038527	*C14H8orf59*	783	484	618	678	559	-0.279	0.748454465	1
ENSBTAG00000038532	*FOLR1*	1	0	0	1	0		1	1
ENSBTAG00000038534	*ZNF446*	25	76	55	22	88	2.019	0.235723283	1
ENSBTAG00000038536	*BT.43571*	1'293	703	966	1'120	812	-0.464	0.575882013	1
ENSBTAG00000038540	*BT.88696*	313	85	185	271	98	-1.466	0.181535956	1
ENSBTAG00000038541	*ZNF879*	346	180	254	300	208	-0.528	0.599992071	1
ENSBTAG00000038544	*BT.101620*	2	0	1	2	0		0.974934741	1
ENSBTAG00000038548	*SLC47A2*	6	3	4	5	3	-0.585	0.976586913	1
ENSBTAG00000038551	*protein_coding*	0	2	1	0	2	Inf	0.939077559	1
ENSBTAG00000038562	*protein_coding*	4	8	6	3	9	1.415	0.839396723	1
ENSBTAG00000038565	*BT.67468*	1	0	0	1	0		1	1
ENSBTAG00000038568	*protein_coding*	12	0	5	10	0		0.583618942	1
ENSBTAG00000038576	*BT.106439*	1	0	0	1	0		1	1
ENSBTAG00000038577	*TSC22D4*	218	131	170	189	151	-0.320	0.776457503	1
ENSBTAG00000038584	*OLFM1*	651	192	393	564	222	-1.347	0.148865629	1
ENSBTAG00000038589	*ZNF157*	33	20	26	29	23	-0.307	0.915418315	1
ENSBTAG00000038598	*protein_coding*	48	15	29	42	17	-1.263	0.584764123	1
ENSBTAG00000038600	*LRRC15*	592	155	346	513	179	-1.518	0.112668676	1
ENSBTAG00000038604	*H2B*	110	36	68	95	42	-1.196	0.434126415	1
ENSBTAG00000038605	*BT.105105*	1	0	0	1	0		1	1
ENSBTAG00000038606	*BT.26282*	3	2	2	3	2	-0.170	1	1
ENSBTAG00000038610	*ZBTB26*	170	144	157	147	166	0.176	0.880625274	1
ENSBTAG00000038613	*protein_coding*	64	12	35	55	14	-2.000	0.350097889	1
ENSBTAG00000038616	*PRSS46*	1	0	0	1	0		1	1
ENSBTAG00000038617	*SH3D21*	514	588	562	445	679	0.609	0.486199603	1
ENSBTAG00000038619	*BT.62638*	352	270	308	305	312	0.032	0.976105408	1
ENSBTAG00000038620	*CIR1*	1'661	1'032	1'315	1'438	1'192	-0.272	0.738261604	1
ENSBTAG00000038625	*BT.93168*	407	254	323	352	293	-0.265	0.783550838	1
ENSBTAG00000038627	*protein_coding*	6	7	7	5	8	0.637	0.939319729	1
ENSBTAG00000038630	*KLHL34*	4	1	2	3	1	-1.585	0.952247636	1
ENSBTAG00000038635	*BT.88280*	1'767	1'206	1'461	1'530	1'393	-0.136	0.866992549	1
ENSBTAG00000038639	*CXCL9*	42	4	20	36	5	-2.977	0.320832869	1
ENSBTAG00000038640	*HIGD1D*	516	331	415	447	382	-0.226	0.807317223	1
ENSBTAG00000038649	*protein_coding*	1	0	0	1	0		1	1
ENSBTAG00000038650	*MS4A7*	37	2	17	32	2	-3.794	0.281715942	1
ENSBTAG00000038652	*protein_coding*	80	26	50	69	30	-1.206	0.494131632	1
ENSBTAG00000038659	*KCTD8*	17	18	18	15	21	0.497	0.883909407	1
ENSBTAG00000038660	*MGC134232*	1	0	0	1	0		1	1
ENSBTAG00000038662	*GJB6*	237	111	167	205	128	-0.679	0.543403657	1
ENSBTAG00000038674	*BT.73479*	264	134	192	229	155	-0.563	0.601065071	1
ENSBTAG00000038680	*processed_pseudogene*	1	0	0	1	0		1	1
ENSBTAG00000038682	*FAM83H*	656	1'560	1'185	568	1'801	1.665	0.045591693	1
ENSBTAG00000038691	*WDR85*	1'776	1'758	1'784	1'538	2'030	0.400	0.614767429	1
ENSBTAG00000038696	*RAB8A*	2'085	2'538	2'368	1'806	2'931	0.699	0.375670619	1
ENSBTAG00000038698	*protein_coding*	2	2	2	2	2	0.415	1	1
ENSBTAG00000038700	*FAM124B*	676	198	407	585	229	-1.356	0.143660586	1
ENSBTAG00000038702	*protein_coding*	113	59	83	98	68	-0.522	0.714371262	1
ENSBTAG00000038706	*MT1E*	3	1	2	3	1	-1.170	0.985253077	1
ENSBTAG00000038710	*protein_coding*	317	123	208	275	142	-0.951	0.367141039	1
ENSBTAG00000038711	*TMEM91*	79	78	79	68	90	0.397	0.785365238	1
ENSBTAG00000038715	*protein_coding*	156	27	83	135	31	-2.115	0.143686799	1
ENSBTAG00000038716	*SKI*	2'255	3'108	2'771	1'953	3'589	0.878	0.264431899	1
ENSBTAG00000038733	*EFS*	5'120	8'222	6'964	4'434	9'494	1.098	0.156504702	1
ENSBTAG00000038735	*LHB*	48	59	55	42	68	0.713	0.671545805	1
ENSBTAG00000038737	*P2RY6*	48	33	40	42	38	-0.126	0.963287273	1
ENSBTAG00000038738	*PXMP4*	78	53	64	68	61	-0.142	0.938013204	1
ENSBTAG00000038739	*CPLX4*	5	0	2	4	0		0.853268594	1
ENSBTAG00000038745	*CHMP6*	1'218	1'511	1'400	1'055	1'745	0.726	0.369008452	1
ENSBTAG00000038748	*HBB*	10	9	10	9	10	0.263	0.978917997	1

Ensembl gene ID	geneName	counts wildtype horn bud	counts polled horn bud	baseMean	baseMean wildtype horn bud	baseMean polled horn bud	log2FoldChange	pval	padj
ENSBTAG00000038755	*protein_coding*	273	162	212	236	187	-0.338	0.749454485	1
ENSBTAG00000038756	*GLB1L3*	53	28	39	46	32	-0.506	0.805607882	1
ENSBTAG00000038757	*HBM*	9	4	6	8	5	-0.755	0.931866093	1
ENSBTAG00000038770	*protein_coding*	116	64	87	100	74	-0.443	0.753005906	1
ENSBTAG00000038777	*MSMP*	711	537	618	616	620	0.010	0.992118776	1
ENSBTAG00000038779	*pseudogene*	4	3	3	3	3	0.000	1	1
ENSBTAG00000038782	*ZSCAN30*	10	3	6	9	3	-1.322	0.863266279	1
ENSBTAG00000038794	*TMEM245*	119	95	106	103	110	0.090	0.950701795	1
ENSBTAG00000038795	*STARD3NL*	876	379	598	759	438	-0.794	0.362396367	1
ENSBTAG00000038797	*protein_coding*	3	4	4	3	5	0.830	0.962490925	1
ENSBTAG00000038806	*ZNF891*	75	52	62	65	60	-0.113	0.954264319	1
ENSBTAG00000038810	*SFTA2*	41	30	35	36	35	-0.036	1	1
ENSBTAG00000038815	*BT.71216*	21	26	24	18	30	0.723	0.783160023	1
ENSBTAG00000038831	*BT.53448*	1'368	1'043	1'195	1'185	1'204	0.024	0.977209858	1
ENSBTAG00000038842	*NEDD8*	3'193	1'954	2'511	2'765	2'256	-0.293	0.709418392	1
ENSBTAG00000038844	*ANKRD35*	171	96	129	148	111	-0.418	0.731571745	1
ENSBTAG00000038845	*TYSND1*	304	280	293	263	323	0.296	0.761307256	1
ENSBTAG00000038848	*bta-mir-1301*	2	0	1	2	0		0.974934741	1
ENSBTAG00000038849	*TRDN*	23	18	20	20	21	0.061	1	1
ENSBTAG00000038854	*CLEC2L*	55	2	25	48	2	-4.366	0.13408164	1
ENSBTAG00000038865	*BT.28947*	1'305	454	827	1'130	524	-1.108	0.190240044	1
ENSBTAG00000038866	*UBE2I*	2'717	1'590	2'094	2'353	1'836	-0.358	0.651538915	1
ENSBTAG00000038869	*ZNF383*	1'414	974	1'175	1'225	1'125	-0.123	0.881841423	1
ENSBTAG00000038873	*U3*	6	0	3	5	0		0.81115604	1
ENSBTAG00000038878	*BT.28692*	694	801	763	601	925	0.622	0.462013944	1
ENSBTAG00000038879	*bta-mir-1307*	11	1	5	10	1	-3.044	0.722857941	1
ENSBTAG00000038882	*bta-mir-1249*	3	3	3	3	3	0.415	1	1
ENSBTAG00000038888	*ZBTB9*	965	882	927	836	1'018	0.285	0.730956552	1
ENSBTAG00000038889	*RILP*	64	60	62	55	69	0.322	0.84457756	1
ENSBTAG00000038891	*protein_coding*	128	58	89	111	67	-0.727	0.597984376	1
ENSBTAG00000038893	*BT.87408*	526	635	594	456	733	0.687	0.429506642	1
ENSBTAG00000038896	*BT.67830*	21'406	18'767	20'104	18'538	21'670	0.225	0.766701437	1
ENSBTAG00000038900	*BT.65765*	47	176	122	41	203	2.320	0.067056327	1
ENSBTAG00000038903	*VN1R2*	34	15	23	29	17	-0.766	0.777580933	1
ENSBTAG00000038904	*BT.104269*	321	131	215	278	151	-0.878	0.401211312	1
ENSBTAG00000038910	*SECTM1*	0	1	1	0	1	Inf	0.993540919	1
ENSBTAG00000038916	*PRICKLE4*	247	261	258	214	301	0.495	0.620485752	1
ENSBTAG00000038920	*VPS13C*	1'605	1'304	1'448	1'390	1'506	0.115	0.885940953	1
ENSBTAG00000038926	*protein_coding*	293	169	224	254	195	-0.379	0.715687858	1
ENSBTAG00000038928	*protein_coding*	6	4	5	5	5	-0.170	1	1
ENSBTAG00000038929	*BCAS4*	1'334	899	1'097	1'155	1'038	-0.154	0.852055684	1
ENSBTAG00000038931	*protein_coding*	88	76	82	76	88	0.204	0.891762585	1
ENSBTAG00000038933	*protein_coding*	1	0	0	1	0		1	1
ENSBTAG00000038938	*BT.93593*	3	55	33	3	64	4.611	0.062877152	1
ENSBTAG00000038945	*PADI6*	5	0	2	4	0		0.853268594	1
ENSBTAG00000038949	*MRPL30*	587	403	487	508	465	-0.128	0.888910752	1
ENSBTAG00000038951	*ZNF461*	424	184	290	367	212	-0.789	0.419413474	1
ENSBTAG00000038955	*protein_coding*	4	7	6	3	8	1.222	0.876605346	1
ENSBTAG00000038960	*BT.92696*	2	1	1	2	1	-0.585	1	1
ENSBTAG00000038969	*pseudogene*	1	0	0	1	0		1	1
ENSBTAG00000038970	*ZFP161*	419	273	339	363	315	-0.203	0.832819177	1
ENSBTAG00000038972	*LCN9*	24	0	10	21	0		0.291206645	1
ENSBTAG00000038978	*protein_coding*	71	115	97	61	133	1.111	0.40211296	1
ENSBTAG00000038979	*TMEM167B*	2'906	2'225	2'543	2'517	2'569	0.030	0.969543692	1
ENSBTAG00000038981	*EMR4P*	13	4	8	11	5	-1.285	0.828302954	1
ENSBTAG00000038982	*BT.51219*	7	3	5	6	3	-0.807	0.947428618	1
ENSBTAG00000038985	*protein_coding*	15	19	17	13	22	0.756	0.816016334	1
ENSBTAG00000038990	*TRAF4*	2'639	2'707	2'706	2'285	3'126	0.452	0.563972465	1
ENSBTAG00000038992	*STK11IP*	737	829	798	638	957	0.585	0.487196439	1
ENSBTAG00000038993	*protein_coding*	0	1	1	0	1	Inf	0.993540919	1
ENSBTAG00000039012	*BT.88331*	52	19	33	45	22	-1.037	0.630732115	1
ENSBTAG00000039014	*BT.39404*	352	468	423	305	540	0.826	0.365110239	1
ENSBTAG00000039015	*BT.25702*	285	70	164	247	81	-1.610	0.156759007	1
ENSBTAG00000039016	*protein_coding*	4	0	2	3	0		0.89545886	1
ENSBTAG00000039021	*protein_coding*	0	1	1	0	1	Inf	0.993540919	1
ENSBTAG00000039023	*ZNF664*	1'013	857	933	877	990	0.174	0.834237795	1
ENSBTAG00000039028	*PI3*	2	1	1	2	1	-0.585	1	1
ENSBTAG00000039035	*HSPA6*	64	58	61	55	67	0.273	0.870835727	1
ENSBTAG00000039037	*SERPINB4*	1'038	312	630	899	360	-1.319	0.131082041	1
ENSBTAG00000039041	*BT.53519*	6	2	4	5	2	-1.170	0.934316631	1
ENSBTAG00000039046	*MGC127538*	10'136	3'999	6'698	8'778	4'618	-0.927	0.231025157	1
ENSBTAG00000039049	*RAI2*	579	508	544	501	587	0.226	0.797115677	1

Ensembl gene ID	geneName	counts wildtype horn bud	counts polled horn bud	baseMean	baseMean wildtype horn bud	baseMean polled horn bud	log2FoldChange	pval	padj
ENSBTAG00000039050	*P2RY2*	69	48	58	60	55	-0.109	0.959275434	1
ENSBTAG00000039052	*protein_coding*	2	3	3	2	3	1.000	0.97171833	1
ENSBTAG00000039055	*ANKRD65*	19	79	54	16	91	2.471	0.156195666	1
ENSBTAG00000039059	*GP1BB*	15	5	9	13	6	-1.170	0.820729911	1
ENSBTAG00000039065	*HS6ST3*	2	3	3	2	3	1.000	0.97171833	1
ENSBTAG00000039066	*BT.36560*	9	0	4	8	0		0.690676647	1
ENSBTAG00000039067	*PCDHB15*	60	57	59	52	66	0.341	0.839306073	1
ENSBTAG00000039069	*IFNE*	0	1	1	0	1	Inf	0.993540919	1
ENSBTAG00000039071	*BT.89347*	845	905	888	732	1'045	0.514	0.537224445	1
ENSBTAG00000039075	*ZNF527*	967	533	726	837	615	-0.444	0.601918457	1
ENSBTAG00000039077	*protein_coding*	2	0	1	2	0		0.974934741	1
ENSBTAG00000039080	*SLC22A3*	1'081	351	671	936	405	-1.208	0.162899049	1
ENSBTAG00000039090	*MAGEH1*	1'481	987	1'211	1'283	1'140	-0.170	0.835438581	1
ENSBTAG00000039091	*CSRNP3*	10	1	5	9	1	-2.907	0.754028015	1
ENSBTAG00000039093	*protein_coding*	1	0	0	1	0		1	1
ENSBTAG00000039105	*S100A3*	8	1	4	7	1	-2.585	0.818599781	1
ENSBTAG00000039111	*ZNF577*	6	1	3	5	1	-2.170	0.885240772	1
ENSBTAG00000039117	*FAM179B*	2'407	1'111	1'684	2'085	1'283	-0.700	0.382186568	1
ENSBTAG00000039118	*bta-mir-376d*	0	1	1	0	1	Inf	0.993540919	1
ENSBTAG00000039119	*AANAT*	4	0	2	3	0		0.89545886	1
ENSBTAG00000039121	*PTP4A2*	4'217	3'769	4'002	3'652	4'352	0.253	0.743580777	1
ENSBTAG00000039129	*BT.43313*	136	100	117	118	115	-0.029	0.990502693	1
ENSBTAG00000039130	*BT.63981*	5'625	5'179	5'426	4'871	5'980	0.296	0.700514056	1
ENSBTAG00000039132	*CC2D2B*	749	201	440	649	232	-1.483	0.106725351	1
ENSBTAG00000039133	*C3ORF49*	9	2	5	8	2	-1.755	0.844314416	1
ENSBTAG00000039138	*MRPL41*	652	723	700	565	835	0.564	0.508166869	1
ENSBTAG00000039141	*TMEM74*	40	1	18	35	1	-4.907	0.190349621	1
ENSBTAG00000039151	*BT.27991*	136	65	96	118	75	-0.650	0.627380358	1
ENSBTAG00000039153	*RPS6KA4*	1'195	1'698	1'498	1'035	1'961	0.922	0.253598532	1
ENSBTAG00000039154	*pseudogene*	16	3	9	14	3	-2.000	0.709202358	1
ENSBTAG00000039157	*GPR182*	27	31	30	23	36	0.614	0.793316703	1
ENSBTAG00000039160	*VAV1*	282	194	234	244	224	-0.125	0.90740567	1
ENSBTAG00000039161	*ALDH3A2*	4'883	5'132	5'077	4'229	5'926	0.487	0.528099268	1
ENSBTAG00000039162	*PCDHGA2*	290	193	237	251	223	-0.172	0.869539607	1
ENSBTAG00000039163	*HIST1H1D*	287	79	170	249	91	-1.446	0.197166938	1
ENSBTAG00000039169	*processed_pseudogene*	0	4	2	0	5	Inf	0.817359038	1
ENSBTAG00000039172	*RABGAP1*	6'313	5'528	5'925	5'467	6'383	0.223	0.770925895	1
ENSBTAG00000039186	*MGC151949*	1	0	0	1	0		1	1
ENSBTAG00000039190	*SLC9A5*	1'806	929	1'318	1'564	1'073	-0.544	0.502597436	1
ENSBTAG00000039194	*C3H3orf22*	129	45	82	112	52	-1.104	0.43722316	1
ENSBTAG00000039195	*NHS*	482	344	407	417	397	-0.072	0.941135074	1
ENSBTAG00000039196	*protein_coding*	6	8	7	5	9	0.830	0.903753758	1
ENSBTAG00000039197	*SLC2A9*	14	2	7	12	2	-2.392	0.704665469	1
ENSBTAG00000039206	*SLFN14*	4	3	3	3	3	0.000	1	1
ENSBTAG00000039208	*ATP5E*	354	639	522	307	738	1.267	0.155297307	1
ENSBTAG00000039212	*CTU1*	137	144	142	119	166	0.487	0.677044824	1
ENSBTAG00000039215	*KIR2DL5A*	4	0	2	3	0		0.89545886	1
ENSBTAG00000039218	*pseudogene*	194	249	228	168	288	0.775	0.450375151	1
ENSBTAG00000039223	*WDR38*	6	5	5	5	6	0.152	1	1
ENSBTAG00000039231	*C4H7orf41*	5'561	2'878	4'070	4'816	3'323	-0.535	0.490903571	1
ENSBTAG00000039234	*LIPK*	107	53	77	93	61	-0.599	0.683277951	1
ENSBTAG00000039241	*OR6S1*	1	0	0	1	0		1	1
ENSBTAG00000039242	*ZNF10*	267	175	217	231	202	-0.194	0.855760743	1
ENSBTAG00000039246	*TRIM11*	1'729	1'595	1'670	1'497	1'842	0.299	0.70807773	1
ENSBTAG00000039255	*CLDN17*	84	29	53	73	33	-1.119	0.512612419	1
ENSBTAG00000039261	*KRT2*	4	4	4	3	5	0.415	0.994759363	1
ENSBTAG00000039263	*protein_coding*	191	371	297	165	428	1.373	0.160950799	1
ENSBTAG00000039274	*protein_coding*	1	4	3	1	5	2.415	0.884888347	1
ENSBTAG00000039275	*ERAP2*	437	282	352	378	326	-0.217	0.819985826	1
ENSBTAG00000039276	*MUC17*	1	0	0	1	0		1	1
ENSBTAG00000039283	*pseudogene*	9	8	9	8	9	0.245	0.986825286	1
ENSBTAG00000039287	*ZNF642*	1'023	534	751	886	617	-0.523	0.537847596	1
ENSBTAG00000039292	*pseudogene*	411	366	389	356	423	0.248	0.789092912	1
ENSBTAG00000039296	*BT.103770*	148	86	114	128	99	-0.368	0.773603533	1
ENSBTAG00000039299	*RNF222*	7	2	4	6	2	-1.392	0.903936064	1
ENSBTAG00000039307	*BT.28580*	1'493	843	1'133	1'293	973	-0.410	0.617299866	1
ENSBTAG00000039313	*ZNF341*	602	577	594	521	666	0.354	0.683690359	1
ENSBTAG00000039316	*protein_coding*	1'004	442	690	869	510	-0.769	0.369949263	1
ENSBTAG00000039321	*protein_coding*	12	25	20	10	29	1.474	0.611572134	1
ENSBTAG00000039324	*FGFBP3*	8	1	4	7	1	-2.585	0.818599781	1
ENSBTAG00000039325	*GPA*	8	4	6	7	5	-0.585	0.95915875	1
ENSBTAG00000039326	*BT.38428*	80	84	83	69	97	0.485	0.73204905	1

Ensembl gene ID	geneName	counts wildtype horn bud	counts polled horn bud	baseMean	baseMean wildtype horn bud	baseMean polled horn bud	log2FoldChange	pval	padj
ENSBTAG00000039328	*PURG*	146	105	124	126	121	-0.061	0.968455998	1
ENSBTAG00000039329	*BT.22952*	75	19	43	65	22	-1.566	0.407148693	1
ENSBTAG00000039334	*protein_coding*	6	8	7	5	9	0.830	0.903753758	1
ENSBTAG00000039335	*ARRDC2*	404	539	486	350	622	0.831	0.35237426	1
ENSBTAG00000039340	*SCN4B*	93	242	180	81	279	1.795	0.106101415	1
ENSBTAG00000039341	*protein_coding*	632	292	442	547	337	-0.699	0.440954274	1
ENSBTAG00000039343	*ZBTB34*	132	145	141	114	167	0.551	0.638350329	1
ENSBTAG00000039346	*protein_coding*	4	1	2	3	1	-1.585	0.952247636	1
ENSBTAG00000039347	*CLEC4D*	9	0	4	8	0		0.690676647	1
ENSBTAG00000039353	*ISM2*	13	2	7	11	2	-2.285	0.731320841	1
ENSBTAG00000039355	*BT.46202*	0	3	2	0	3	Inf	0.878646179	1
ENSBTAG00000039356	*protein_coding*	2	0	1	2	0		0.974934741	1
ENSBTAG00000039366	*CD1A*	3	3	3	3	3	0.415	1	1
ENSBTAG00000039372	*ODF3L2*	14	8	11	12	9	-0.392	0.950061617	1
ENSBTAG00000039374	*RANBP6*	11'291	15'732	13'972	9'778	18'166	0.894	0.244246631	1
ENSBTAG00000039377	*SLITRK2*	709	1'103	944	614	1'274	1.053	0.207260529	1
ENSBTAG00000039380	*protein_coding*	2	1	1	2	1	-0.585	1	1
ENSBTAG00000039384	*MGC157237*	13	4	8	11	5	-1.285	0.828302954	1
ENSBTAG00000039391	*BT.88841*	386	261	318	334	301	-0.150	0.879264104	1
ENSBTAG00000039397	*BT.71501*	65	53	59	56	61	0.121	0.950532365	1
ENSBTAG00000039401	*TRB@*	1	1	1	1	1	0.415	1	1
ENSBTAG00000039402	*protein_coding*	1	0	0	1	0		1	1
ENSBTAG00000039406	*processed_pseudogene*	992	1'601	1'354	859	1'849	1.106	0.174820401	1
ENSBTAG00000039409	*protein_coding*	1	0	0	1	0		1	1
ENSBTAG00000039413	*protein_coding*	6	8	7	5	9	0.830	0.903753758	1
ENSBTAG00000039415	*CHMP4A*	2'480	1'661	2'033	2'148	1'918	-0.163	0.837630837	1
ENSBTAG00000039425	*BT.99723*	3'147	1'338	2'135	2'725	1'545	-0.819	0.302459237	1
ENSBTAG00000039426	*DPEP2*	19	13	16	16	15	-0.132	0.991133154	1
ENSBTAG00000039433	*PCDHB7*	182	120	148	158	139	-0.186	0.877496043	1
ENSBTAG00000039434	*FAM127A*	1'323	1'746	1'581	1'146	2'016	0.815	0.310472821	1
ENSBTAG00000039435	*LEMD3*	1'266	820	1'022	1'096	947	-0.212	0.798648797	1
ENSBTAG00000039437	*protein_coding*	19	10	14	16	12	-0.511	0.904299589	1
ENSBTAG00000039440	*COX7C*	15	11	13	13	13	-0.032	1	1
ENSBTAG00000039442	*SEC62*	7'062	3'433	5'040	6'116	3'964	-0.626	0.41911871	1
ENSBTAG00000039453	*ZNF446*	109	134	125	94	155	0.713	0.557603248	1
ENSBTAG00000039456	*BT.87860*	2'579	5'771	4'449	2'233	6'664	1.577	0.045769378	1
ENSBTAG00000039462	*PAF*	1'435	1'135	1'277	1'243	1'311	0.077	0.924848224	1
ENSBTAG00000039466	*CDR2L*	809	1'124	999	701	1'298	0.889	0.283061803	1
ENSBTAG00000039470	*WC1*	3	1	2	3	1	-1.170	0.985253077	1
ENSBTAG00000039476	*protein_coding*	3	0	1	3	0		0.936647693	1
ENSBTAG00000039477	*TPBG*	3'332	1'801	2'483	2'886	2'080	-0.473	0.548438878	1
ENSBTAG00000039483	*BT.56359*	2'779	2'039	2'381	2'407	2'354	-0.032	0.969144274	1
ENSBTAG00000039485	*C17ORF104*	22	13	17	19	15	-0.344	0.931546014	1
ENSBTAG00000039486	*protein_coding*	28	22	25	24	25	0.067	0.996573573	1
ENSBTAG00000039491	*protein_coding*	8	9	9	7	10	0.585	0.926558417	1
ENSBTAG00000039492	*HIST1H2AG*	1	1	1	1	1	0.415	1	1
ENSBTAG00000039493	*BT.67889*	1'442	669	1'011	1'249	772	-0.693	0.402255793	1
ENSBTAG00000039499	*protein_coding*	210	32	109	182	37	-2.299	0.08127342	1
ENSBTAG00000039509	*BT.18782*	440	255	338	381	294	-0.372	0.695958087	1
ENSBTAG00000039512	*ZNF498*	270	348	318	234	402	0.781	0.414440497	1
ENSBTAG00000039513	*GCNT2*	77	121	103	67	140	1.067	0.410616896	1
ENSBTAG00000039520	*BT.39727*	178	467	347	154	539	1.807	0.060552725	1
ENSBTAG00000039522	*CYCT*	4	0	2	3	0		0.89545886	1
ENSBTAG00000039523	*ZNF266*	287	133	201	249	154	-0.695	0.51333555	1
ENSBTAG00000039524	*BT.87446*	6	1	3	5	1	-2.170	0.885240772	1
ENSBTAG00000039525	*pseudogene*	10	2	5	9	2	-1.907	0.815196829	1
ENSBTAG00000039529	*BTBD19*	451	86	245	391	99	-1.976	0.057319685	1
ENSBTAG00000039531	*LCN12*	7	0	3	6	0		0.769774961	1
ENSBTAG00000039550	*U2*	2	2	2	2	2	0.415	1	1
ENSBTAG00000039552	*PLK4*	1'604	856	1'189	1'389	988	-0.491	0.54780472	1
ENSBTAG00000039555	*COX7C*	2'297	2'176	2'251	1'989	2'513	0.337	0.668648819	1
ENSBTAG00000039556	*BT.89318*	2'049	1'714	1'877	1'774	1'979	0.157	0.842553079	1
ENSBTAG00000039563	*protein_coding*	20	64	46	17	74	2.093	0.259084143	1
ENSBTAG00000039571	*C13H10orf47*	1'982	1'145	1'519	1'716	1'322	-0.377	0.639846253	1
ENSBTAG00000039573	*PARF*	2'051	3'387	2'844	1'776	3'911	1.139	0.149378524	1
ENSBTAG00000039574	*NEPN*	1	0	0	1	0		1	1
ENSBTAG00000039581	*HOXD4*	555	777	689	481	897	0.900	0.29350519	1
ENSBTAG00000039582	*NDUFC1*	222	281	258	192	324	0.755	0.449333041	1
ENSBTAG00000039588	*BT.49529*	4	0	2	3	0		0.89545886	1
ENSBTAG00000039591	*TMEM167A*	4'742	3'027	3'801	4'107	3'495	-0.233	0.765159424	1
ENSBTAG00000039593	*FAM98C*	331	402	375	287	464	0.695	0.453868522	1
ENSBTAG00000039594	*ZNF862*	298	492	413	258	568	1.138	0.215785459	1

Ensembl gene ID	geneName	counts wildtype horn bud	counts polled horn bud	baseMean	baseMean wildtype horn bud	baseMean polled horn bud	log2FoldChange	pval	padj
ENSBTAG00000039599	*BT.46279*	5	0	2	4	0		0.853268594	1
ENSBTAG00000039601	*SAP25*	425	309	362	368	357	-0.045	0.965673821	1
ENSBTAG00000039607	*PRCD*	0	1	1	0	1	Inf	0.993540919	1
ENSBTAG00000039618	*C10H11ORF46*	93	184	147	81	212	1.399	0.229429357	1
ENSBTAG00000039620	*MGC151586*	148	31	82	128	36	-1.840	0.201737593	1
ENSBTAG00000039630	*TMED2*	3'920	3'474	3'703	3'395	4'011	0.241	0.755963419	1
ENSBTAG00000039634	*NDNL2*	1'509	1'084	1'279	1'307	1'252	-0.062	0.940497197	1
ENSBTAG00000039635	*CCDC97*	1'493	2'227	1'932	1'293	2'572	0.992	0.2142057	1
ENSBTAG00000039644	*protein_coding*	0	2	1	0	2	Inf	0.939077559	1
ENSBTAG00000039652	*GPR44*	12	9	10	10	10	0.000	1	1
ENSBTAG00000039658	*PDCD7*	1'269	709	959	1'099	819	-0.425	0.608876246	1
ENSBTAG00000039662	*RSC1A1*	583	298	424	505	344	-0.553	0.544441521	1
ENSBTAG00000039664	*processed_pseudogene*	435	303	363	377	350	-0.107	0.912498894	1
ENSBTAG00000039671	*processed_pseudogene*	4'414	4'207	4'340	3'823	4'858	0.346	0.654577643	1
ENSBTAG00000039674	*protein_coding*	0	2	1	0	2	Inf	0.939077559	1
ENSBTAG00000039676	*protein_coding*	1	0	0	1	0		1	1
ENSBTAG00000039682	*MTUS1*	2'899	1'640	2'202	2'511	1'894	-0.407	0.606982076	1
ENSBTAG00000039684	*PTRF*	20'415	25'720	23'689	17'680	29'699	0.748	0.327113472	1
ENSBTAG00000039686	*BT.59650*	36	31	33	31	36	0.199	0.93798045	1
ENSBTAG00000039688	*FAM101A*	993	1'046	1'034	860	1'208	0.490	0.551401077	1
ENSBTAG00000039695	*KIAA1949*	2'430	2'873	2'711	2'104	3'317	0.657	0.402693383	1
ENSBTAG00000039696	*SHD*	35	13	23	30	15	-1.014	0.709014988	1
ENSBTAG00000039702	*FCRL3*	2	2	2	2	2	0.415	1	1
ENSBTAG00000039705	*protein_coding*	596	868	759	516	1'002	0.957	0.25976068	1
ENSBTAG00000039708	*C9ORF172*	45	23	33	39	27	-0.553	0.806500404	1
ENSBTAG00000039709	*PARPBP*	259	202	229	224	233	0.056	0.959863072	1
ENSBTAG00000039711	*ZFP62*	1'322	508	866	1'145	587	-0.965	0.251099284	1
ENSBTAG00000039714	*BT.59018*	10	5	7	9	6	-0.585	0.94357809	1
ENSBTAG00000039718	*BT.23479*	4	7	6	3	8	1.222	0.876605346	1
ENSBTAG00000039719	*PHGDH*	6'978	8'593	7'983	6'043	9'922	0.715	0.352161441	1
ENSBTAG00000039720	*protein_coding*	952	463	680	824	535	-0.625	0.46631576	1
ENSBTAG00000039722	*protein_coding*	1	1	1	1	1	0.415	1	1
ENSBTAG00000039727	*DCLK3*	51	34	42	44	39	-0.170	0.942391534	1
ENSBTAG00000039728	*protein_coding*	45'326	29'379	36'589	39'253	33'924	-0.211	0.782305008	1
ENSBTAG00000039731	*RND3*	6'576	3'199	4'694	5'695	3'694	-0.625	0.420469369	1
ENSBTAG00000039732	*BT.22851*	1'377	1'302	1'348	1'193	1'503	0.334	0.678859067	1
ENSBTAG00000039738	*ADORA3*	27	23	25	23	27	0.184	0.95691183	1
ENSBTAG00000039740	*LY6G5B*	1'083	835	951	938	964	0.040	0.962263182	1
ENSBTAG00000039757	*protein_coding*	0	1	1	0	1	Inf	0.993540919	1
ENSBTAG00000039764	*IER5*	385	188	275	333	217	-0.619	0.530860553	1
ENSBTAG00000039766	*FBRSL1*	1'312	1'735	1'570	1'136	2'003	0.818	0.308936097	1
ENSBTAG00000039770	*BT.73256*	7'199	3'388	5'073	6'235	3'912	-0.672	0.385360649	1
ENSBTAG00000039772	*BT.75542*	16	3	9	14	3	-2.000	0.709202358	1
ENSBTAG00000039782	*ZNF12*	462	255	347	400	294	-0.442	0.639829686	1
ENSBTAG00000039784	*PRR5*	1'161	1'180	1'184	1'005	1'363	0.438	0.590341197	1
ENSBTAG00000039787	*CSN3*	3	2	2	3	2	-0.170	1	1
ENSBTAG00000039789	*protein_coding*	135	368	271	117	425	1.862	0.065501558	1
ENSBTAG00000039793	*HDGF*	2'706	4'765	3'923	2'343	5'502	1.231	0.116686977	1
ENSBTAG00000039794	*CSNK1B*	1'338	541	892	1'159	625	-0.891	0.287260997	1
ENSBTAG00000039803	*MYO7B*	502	131	293	435	151	-1.523	0.123019863	1
ENSBTAG00000039807	*BT.28004*	1	0	0	1	0		1	1
ENSBTAG00000039812	*H4*	103	22	57	89	25	-1.812	0.278023157	1
ENSBTAG00000039813	*BT.21347*	42	9	23	36	10	-1.807	0.495680066	1
ENSBTAG00000039815	*ZBTB6*	155	132	143	134	152	0.183	0.878844375	1
ENSBTAG00000039819	*BT.25326*	359	311	335	311	359	0.208	0.82753303	1
ENSBTAG00000039820	*CLDN8*	564	583	581	488	673	0.463	0.594978622	1
ENSBTAG00000039823	*KBTBD7*	529	395	457	458	456	-0.006	0.997952595	1
ENSBTAG00000039834	*protein_coding*	45	15	28	39	17	-1.170	0.622112607	1
ENSBTAG00000039847	*protein_coding*	3	0	1	3	0		0.936647693	1
ENSBTAG00000039849	*protein_coding*	1	1	1	1	1	0.415	1	1
ENSBTAG00000039850	*protein_coding*	0	1	1	0	1	Inf	0.993540919	1
ENSBTAG00000039851	*UBAC1*	3'739	4'030	3'946	3'238	4'653	0.523	0.500032954	1
ENSBTAG00000039855	*SUMF1*	4'000	3'266	3'618	3'464	3'771	0.123	0.874191509	1
ENSBTAG00000039856	*BT.86212*	1	7	4	1	8	3.222	0.741805782	1
ENSBTAG00000039861	*BT.41610*	545	126	309	472	145	-1.698	0.083879973	1
ENSBTAG00000039870	*BT.71121*	1	0	0	1	0		1	1
ENSBTAG00000039871	*ZNF518B*	505	754	654	437	871	0.993	0.249896529	1
ENSBTAG00000039875	*protein_coding*	5	2	3	4	2	-0.907	0.965043111	1
ENSBTAG00000039886	*BT.32293*	122	142	135	106	164	0.634	0.59295415	1
ENSBTAG00000039888	*MAGED2*	18'093	15'096	16'550	15'669	17'431	0.154	0.839403122	1
ENSBTAG00000039892	*protein_coding*	2	10	7	2	12	2.737	0.678653301	1
ENSBTAG00000039904	*protein_coding*	1	2	2	1	2	1.415	0.981979269	1

Ensembl gene ID	geneName	counts wildtype horn bud	counts polled horn bud	baseMean	baseMean wildtype horn bud	baseMean polled horn bud	log2FoldChange	pval	padj
ENSBTAG00000039914	*CTRB1*	3	1	2	3	1	-1.170	0.985253077	1
ENSBTAG00000039916	*SMAD2*	2'750	2'003	2'347	2'382	2'313	-0.042	0.958445834	1
ENSBTAG00000039922	*ARAP2*	589	280	417	510	323	-0.658	0.472317051	1
ENSBTAG00000039928	*protein_coding*	72	11	38	62	13	-2.295	0.26852928	1
ENSBTAG00000039929	*RXFP3*	3	0	1	3	0		0.936647693	1
ENSBTAG00000039932	*protein_coding*	5	14	10	4	16	1.900	0.676791734	1
ENSBTAG00000039935	*KCNC3*	29	6	16	25	7	-1.858	0.581888091	1
ENSBTAG00000039937	*TNFRSF8*	28	55	44	24	64	1.389	0.455012999	1
ENSBTAG00000039943	*NARS2*	728	269	471	630	311	-1.021	0.257434299	1
ENSBTAG00000039946	*ZNF154*	88	65	76	76	75	-0.022	0.999588782	1
ENSBTAG00000039950	*protein_coding*	59	128	99	51	148	1.532	0.24722328	1
ENSBTAG00000039951	*PRMT6*	528	463	496	457	535	0.226	0.800377122	1
ENSBTAG00000039954	*COPZ2*	8'395	6'814	7'569	7'270	7'868	0.114	0.881240066	1
ENSBTAG00000039955	*FERD3L*	2	3	3	2	3	1.000	0.97171833	1
ENSBTAG00000039956	*protein_coding*	1	0	0	1	0		1	1
ENSBTAG00000039958	*GYS1*	1'304	2'193	1'831	1'129	2'532	1.165	0.146921246	1
ENSBTAG00000039962	*DCTPP1*	699	655	681	605	756	0.321	0.707102064	1
ENSBTAG00000039963	*IFNA16*	3	0	1	3	0		0.936647693	1
ENSBTAG00000039968	*TMEM55A*	206	103	149	178	119	-0.585	0.613374518	1
ENSBTAG00000039969	*protein_coding*	240	95	159	208	110	-0.922	0.416006163	1
ENSBTAG00000039971	*BT.60204*	1	0	0	1	0		1	1
ENSBTAG00000039974	*SSTR5*	3	14	9	3	16	2.637	0.594242955	1
ENSBTAG00000039978	*protein_coding*	76	59	67	66	68	0.050	0.984253269	1
ENSBTAG00000039980	*protein_coding*	113	36	70	98	42	-1.235	0.415882902	1
ENSBTAG00000039992	*TUBA1C*	3'832	5'166	4'642	3'319	5'965	0.846	0.275945909	1
ENSBTAG00000039993	*protein_coding*	140	45	87	121	52	-1.222	0.379993227	1
ENSBTAG00000039994	*protein_coding*	0	3	2	0	3	Inf	0.878646179	1
ENSBTAG00000040000	*BT.62233*	125	23	67	108	27	-2.027	0.196187829	1
ENSBTAG00000040001	*MARVELD2*	123	172	153	107	199	0.899	0.431268717	1
ENSBTAG00000040005	*FXYD3*	863	1'072	993	747	1'238	0.728	0.378854643	1
ENSBTAG00000040006	*SRSF3*	3'941	4'471	4'288	3'413	5'163	0.597	0.441052629	1
ENSBTAG00000040018	*processed_pseudogene*	180	213	201	156	246	0.658	0.534272553	1
ENSBTAG00000040019	*protein_coding*	3'940	4'435	4'267	3'412	5'121	0.586	0.449729514	1
ENSBTAG00000040026	*protein_coding*	17	9	13	15	10	-0.503	0.915336184	1
ENSBTAG00000040031	*ZNF148*	5'345	3'323	4'233	4'629	3'837	-0.271	0.727347101	1
ENSBTAG00000040034	*MGC157266*	2	4	3	2	5	1.415	0.927250049	1
ENSBTAG00000040038	*BT.85810*	31	1	14	27	1	-4.539	0.290422853	1
ENSBTAG00000040040	*TAS2R40*	1	1	1	1	1	0.415	1	1
ENSBTAG00000040042	*GRIN2C*	56	29	41	48	33	-0.534	0.788650998	1
ENSBTAG00000040043	*ARMCX6*	2'726	1'311	1'937	2'361	1'514	-0.641	0.420500729	1
ENSBTAG00000040046	*protein_coding*	573	174	349	496	201	-1.304	0.170187732	1
ENSBTAG00000040051	*protein_coding*	1'880	1'950	1'940	1'628	2'252	0.468	0.555203728	1
ENSBTAG00000040053	*BT.102832*	91	109	102	79	126	0.675	0.603711184	1
ENSBTAG00000040055	*CAB39*	4'280	2'204	3'126	3'707	2'545	-0.542	0.48792695	1
ENSBTAG00000040056	*LTB4R2*	71	87	81	61	100	0.708	0.618232306	1
ENSBTAG00000040058	*ORC5L*	1'504	541	964	1'303	625	-1.060	0.203915221	1
ENSBTAG00000040060	*ARTN*	14	11	12	12	13	0.067	1	1
ENSBTAG00000040061	*ZBTB38*	452	525	499	391	606	0.631	0.477620448	1
ENSBTAG00000040063	*ZNF585B*	305	127	205	264	147	-0.849	0.42193267	1
ENSBTAG00000040065	*RAD51AP1*	203	205	206	176	237	0.429	0.683942262	1
ENSBTAG00000040067	*HIPK4*	225	165	193	195	191	-0.032	0.982135313	1
ENSBTAG00000040070	*SCGB1D*	12	3	7	10	3	-1.585	0.809704871	1
ENSBTAG00000040072	*ZNF140*	371	350	363	321	404	0.331	0.723439295	1
ENSBTAG00000040076	*ILF3*	6'168	6'046	6'161	5'342	6'981	0.386	0.615226318	1
ENSBTAG00000040078	*FLVCR2*	189	134	159	164	155	-0.081	0.949256905	1
ENSBTAG00000040083	*BT.50811*	3	1	2	3	1	-1.170	0.985253077	1
ENSBTAG00000040086	*SLC38A8*	5	11	9	4	13	1.553	0.770343785	1
ENSBTAG00000040088	*SLC9B2*	42	33	37	36	38	0.067	0.987719351	1
ENSBTAG00000040093	*FOXI3*	286	468	394	248	540	1.126	0.224369459	1
ENSBTAG00000040098	*protein_coding*	16	15	16	14	17	0.322	0.940397404	1
ENSBTAG00000040106	*CHADL*	41	3	19	36	3	-3.358	0.288139309	1
ENSBTAG00000040108	*ZNF829*	503	354	422	436	409	-0.092	0.922935888	1
ENSBTAG00000040111	*OTOP3*	8	8	8	7	9	0.415	0.960311941	1
ENSBTAG00000040116	*BT.51509*	5'127	7'678	6'653	4'440	8'866	0.998	0.197495047	1
ENSBTAG00000040126	*BT.62689*	6	0	3	5	0		0.81115604	1
ENSBTAG00000040128	*BT.76547*	220	155	185	191	179	-0.090	0.939424017	1
ENSBTAG00000040131	*CD58*	1'496	1'553	1'544	1'296	1'793	0.469	0.558529879	1
ENSBTAG00000040132	*MUM1L1*	722	339	508	625	391	-0.676	0.447198362	1
ENSBTAG00000040133	*C3ORF20*	4	5	5	3	6	0.737	0.954105567	1
ENSBTAG00000040134	*BT.20057*	0	1	1	0	1	Inf	0.993540919	1
ENSBTAG00000040144	*BT.42147*	3	6	5	3	7	1.415	0.880191876	1
ENSBTAG00000040146	*pseudogene*	0	1	1	0	1	Inf	0.993540919	1

Ensembl gene ID	geneName	counts wildtype horn bud	counts polled horn bud	baseMean	baseMean wildtype horn bud	baseMean polled horn bud	log2FoldChange	pval	padj
ENSBTAG00000040147	*SYNDIG1*	67	51	58	58	59	0.021	1	1
ENSBTAG00000040151	*GCH1*	111	23	61	96	27	-1.856	0.252999001	1
ENSBTAG00000040155	*H4*	0	2	1	0	2	Inf	0.939077559	1
ENSBTAG00000040161	*TM4SF19*	43	13	26	37	15	-1.311	0.596402998	1
ENSBTAG00000040169	*BT.51450*	396	324	359	343	374	0.126	0.8950802	1
ENSBTAG00000040177	*KLK9*	17	11	14	15	13	-0.213	0.976116423	1
ENSBTAG00000040187	*protein_coding*	1	0	0	1	0		1	1
ENSBTAG00000040190	*CCDC88B*	161	390	295	139	450	1.691	0.087118995	1
ENSBTAG00000040193	*COLQ*	449	257	343	389	297	-0.390	0.681080599	1
ENSBTAG00000040199	*POLR2H*	3'998	3'842	3'949	3'462	4'436	0.358	0.64423014	1
ENSBTAG00000040206	*ZNF770*	281	177	224	243	204	-0.252	0.810635485	1
ENSBTAG00000040209	*ZNF285*	397	319	356	344	368	0.099	0.917478806	1
ENSBTAG00000040215	*EIF4G3*	6'034	4'281	5'084	5'226	4'943	-0.080	0.918139418	1
ENSBTAG00000040226	*BT.71205*	3	6	5	3	7	1.415	0.880191876	1
ENSBTAG00000040228	*protein_coding*	1	0	0	1	0		1	1
ENSBTAG00000040229	*BT.80000*	0	1	1	0	1	Inf	0.993540919	1
ENSBTAG00000040244	*APOL3*	65	31	46	56	36	-0.653	0.72458185	1
ENSBTAG00000040248	*BT.50959*	0	1	1	0	1	Inf	0.993540919	1
ENSBTAG00000040253	*BT.27996*	48	41	44	42	47	0.188	0.929560875	1
ENSBTAG00000040254	*bta-mir-1193*	2	0	1	2	0		0.974934741	1
ENSBTAG00000040261	*protein_coding*	1	0	0	1	0		1	1
ENSBTAG00000040268	*BT.53394*	11	2	6	10	2	-2.044	0.786625039	1
ENSBTAG00000040280	*OR2J3*	1	1	1	1	1	0.415	1	1
ENSBTAG00000040281	*BT.29751*	0	1	1	0	1	Inf	0.993540919	1
ENSBTAG00000040282	*NOG*	123	106	114	107	122	0.200	0.877422699	1
ENSBTAG00000040290	*GSTO2*	4	1	2	3	1	-1.585	0.952247636	1
ENSBTAG00000040291	*KRTAP10-8*	3	0	1	3	0		0.936647693	1
ENSBTAG00000040293	*RAG1*	0	1	1	0	1	Inf	0.993540919	1
ENSBTAG00000040295	*CHCHD9*	2'013	1'632	1'814	1'743	1'884	0.112	0.887541159	1
ENSBTAG00000040296	*GPR21*	238	108	165	206	125	-0.725	0.517496989	1
ENSBTAG00000040298	*GSTT1*	2'090	2'385	2'282	1'810	2'754	0.606	0.442707509	1
ENSBTAG00000040302	*bta-mir-1281*	6	9	8	5	10	1.000	0.869259965	1
ENSBTAG00000040304	*BT.87103*	40	20	29	35	23	-0.585	0.809552579	1
ENSBTAG00000040308	*RPS13*	987	1'990	1'576	855	2'298	1.427	0.079585143	1
ENSBTAG00000040313	*BT.87304*	130	116	123	113	134	0.251	0.841022415	1
ENSBTAG00000040316	*TMEM92*	276	328	309	239	379	0.664	0.489869318	1
ENSBTAG00000040318	*protein_coding*	13	2	7	11	2	-2.285	0.731320841	1
ENSBTAG00000040321	*SDR16C6*	626	226	402	542	261	-1.055	0.253777912	1
ENSBTAG00000040323	*SECTM1*	615	821	740	533	948	0.832	0.327799206	1
ENSBTAG00000040329	*HTR1D*	4	6	5	3	7	1.000	0.91481797	1
ENSBTAG00000040331	*GIMAP1*	7	4	5	6	5	-0.392	0.98714753	1
ENSBTAG00000040333	*QDPR*	3'393	3'519	3'501	2'938	4'063	0.468	0.547492159	1
ENSBTAG00000040334	*pseudogene*	1'447	1'351	1'407	1'253	1'560	0.316	0.694722988	1
ENSBTAG00000040336	*ZNF268*	146	89	115	126	103	-0.299	0.816337784	1
ENSBTAG00000040338	*BT.43804*	5'388	6'451	6'058	4'666	7'449	0.675	0.381543857	1
ENSBTAG00000040339	*protein_coding*	1	0	0	1	0		1	1
ENSBTAG00000040347	*GPC6*	25	9	16	22	10	-1.059	0.75675668	1
ENSBTAG00000040350	*XIAP*	471	340	400	408	393	-0.055	0.955716746	1
ENSBTAG00000040351	*BOSTAUV1R-PS402*	2	0	1	2	0		0.974934741	1
ENSBTAG00000040356	*SPIRE2*	70	24	44	61	28	-1.129	0.545261509	1
ENSBTAG00000040358	*ZNF772*	39	22	30	34	25	-0.411	0.868871187	1
ENSBTAG00000040360	*BT.99310*	19	9	13	16	10	-0.663	0.873235283	1
ENSBTAG00000040361	*LFNG*	1'647	2'374	2'084	1'426	2'741	0.943	0.236044409	1
ENSBTAG00000040367	*RPL15*	3'733	7'384	5'880	3'233	8'526	1.399	0.073663951	1
ENSBTAG00000040368	*MGC152585*	1'208	924	1'057	1'046	1'067	0.028	0.972994658	1
ENSBTAG00000040378	*HIST1H2AG*	4	5	5	3	6	0.737	0.954105567	1
ENSBTAG00000040380	*pseudogene*	1'186	1'150	1'178	1'027	1'328	0.371	0.64914155	1
ENSBTAG00000040381	*ZNF75A*	474	265	358	410	306	-0.424	0.652169808	1
ENSBTAG00000040384	*POLR2M*	1'417	1'274	1'349	1'227	1'471	0.262	0.745902425	1
ENSBTAG00000040388	*BT.89204*	1	0	0	1	0		1	1
ENSBTAG00000040390	*BT.59602*	182	73	121	158	84	-0.903	0.46404952	1
ENSBTAG00000040392	*BT.25102*	434	235	324	376	271	-0.470	0.623529835	1
ENSBTAG00000040394	*PRSS27*	194	48	112	168	55	-1.600	0.211336944	1
ENSBTAG00000040398	*BT.31332*	581	117	319	503	135	-1.897	0.053369174	1
ENSBTAG00000040399	*VWA5B2*	130	61	92	113	70	-0.677	0.62012827	1
ENSBTAG00000040406	*processed_pseudogene*	199	366	297	172	423	1.294	0.185418526	1
ENSBTAG00000040409	*BT.104754*	0	2	1	0	2	Inf	0.939077559	1
ENSBTAG00000040411	*protein_coding*	1'264	903	1'069	1'095	1'043	-0.070	0.933683038	1
ENSBTAG00000040413	*CES3*	5	1	3	4	1	-1.907	0.9188325	1
ENSBTAG00000040416	*5_8S_rRNA*	1	0	0	1	0		1	1
ENSBTAG00000040422	*BT.88040*	609	423	508	527	488	-0.111	0.903252939	1
ENSBTAG00000040427	*ARHGAP19*	386	595	511	334	687	1.039	0.243064309	1

Ensembl gene ID	geneName	counts wildtype horn bud	counts polled horn bud	baseMean	baseMean wildtype horn bud	baseMean polled horn bud	log2FoldChange	pval	padj
ENSBTAG00000040432	*CLDN3*	23	27	26	20	31	0.646	0.800334419	1
ENSBTAG00000040435	*RPS27L*	1'296	821	1'035	1'122	948	-0.244	0.768455486	1
ENSBTAG00000040437	*protein_coding*	0	1	1	0	1	Inf	0.993540919	1
ENSBTAG00000040439	*BT.27993*	11	0	5	10	0		0.617662983	1
ENSBTAG00000040442	*protein_coding*	1'067	726	881	924	838	-0.140	0.867930826	1
ENSBTAG00000040457	*protein_coding*	0	2	1	0	2	Inf	0.939077559	1
ENSBTAG00000040460	*KRT23*	1'515	948	1'203	1'312	1'095	-0.261	0.749293708	1
ENSBTAG00000040461	*BT.99193*	0	1	1	0	1	Inf	0.993540919	1
ENSBTAG00000040473	*PCDHGA3*	142	131	137	123	151	0.299	0.802867402	1
ENSBTAG00000040477	*CHD5*	808	999	927	700	1'154	0.721	0.385876732	1
ENSBTAG00000040485	*THEG*	1	1	1	1	1	0.415	1	1
ENSBTAG00000040490	*CCDC3*	22'724	13'395	17'573	19'680	15'467	-0.347	0.649005892	1
ENSBTAG00000040494	*UBL5*	1'312	1'029	1'162	1'136	1'188	0.065	0.937295821	1
ENSBTAG00000040496	*BT.26994*	36	14	24	31	16	-0.948	0.72088335	1
ENSBTAG00000040504	*TRAV16*	1	0	0	1	0		1	1
ENSBTAG00000040507	*ARHGEF18*	786	1'390	1'143	681	1'605	1.238	0.13403105	1
ENSBTAG00000040512	*PCP4L1*	381	81	212	330	94	-1.819	0.089123611	1
ENSBTAG00000040521	*SAMD14*	548	557	559	475	643	0.439	0.616164027	1
ENSBTAG00000040536	*processed_pseudogene*	593	757	694	514	874	0.767	0.36949917	1
ENSBTAG00000040541	*P2RY4*	0	1	1	0	1	Inf	0.993540919	1
ENSBTAG00000040543	*TMEM200A*	153	27	82	133	31	-2.087	0.151131437	1
ENSBTAG00000040547	*UBXN10*	5	0	2	4	0		0.853268594	1
ENSBTAG00000040550	*NKX6-2*	1	1	1	1	1	0.415	1	1
ENSBTAG00000040551	*ZBTB1*	3'196	1'640	2'331	2'768	1'894	-0.548	0.487969678	1
ENSBTAG00000040555	*FBXL19*	1'024	1'350	1'223	887	1'559	0.814	0.318560327	1
ENSBTAG00000040559	*PTPRH*	70	59	64	61	68	0.168	0.922330916	1
ENSBTAG00000040564	*COX7C*	2'817	1'426	2'043	2'440	1'647	-0.567	0.47483922	1
ENSBTAG00000040567	*BT.62033*	3'620	1'251	2'290	3'135	1'445	-1.118	0.160072671	1
ENSBTAG00000040568	*protein_coding*	1'633	664	1'090	1'414	767	-0.883	0.283964643	1
ENSBTAG00000040569	*NLRX1*	920	1'682	1'369	797	1'942	1.286	0.115777419	1
ENSBTAG00000040573	*TSPAN10*	20	3	10	17	3	-2.322	0.618209332	1
ENSBTAG00000040575	*KCTD5*	1'351	1'351	1'365	1'170	1'560	0.415	0.607034491	1
ENSBTAG00000040580	*BT.88435*	68	11	36	59	13	-2.213	0.296317709	1
ENSBTAG00000040584	*DSC2*	1'316	811	1'038	1'140	936	-0.283	0.731667084	1
ENSBTAG00000040585	*NR6A1*	50	60	56	43	69	0.678	0.683445197	1
ENSBTAG00000040596	*LINGO4*	2	0	1	2	0		0.974934741	1
ENSBTAG00000040597	*GSK3B*	136	106	120	118	122	0.055	0.970588656	1
ENSBTAG00000040598	*CXXC11*	30	0	13	26	0		0.205783873	1
ENSBTAG00000040600	*U2*	2	3	3	2	3	1.000	0.97171833	1
ENSBTAG00000040602	*protein_coding*	6'140	4'411	5'205	5'317	5'093	-0.062	0.936706618	1
ENSBTAG00000040603	*ZNF175*	1'289	643	929	1'116	742	-0.588	0.479689204	1
ENSBTAG00000040605	*FOXL1*	14	7	10	12	8	-0.585	0.916699259	1
ENSBTAG00000040607	*SLA2*	154	101	125	133	117	-0.194	0.879720128	1
ENSBTAG00000042084	*U6*	1	0	0	1	0		1	1
ENSBTAG00000042092	*U6*	4	5	5	3	6	0.737	0.954105567	1
ENSBTAG00000042094	*SNORD66*	5	4	4	4	5	0.093	1	1
ENSBTAG00000042098	*SNORD49*	108	43	72	94	50	-0.914	0.54257279	1
ENSBTAG00000042099	*SNORD12*	148	53	95	128	61	-1.066	0.427653664	1
ENSBTAG00000042100	*SNORD102*	19	6	12	16	7	-1.248	0.770493406	1
ENSBTAG00000042102	*U6*	0	2	1	0	2	Inf	0.939077559	1
ENSBTAG00000042115	*SNORD116*	4	1	2	3	1	-1.585	0.952247636	1
ENSBTAG00000042117	*SNORD19*	22	8	14	19	9	-1.044	0.780532613	1
ENSBTAG00000042124	*U6atac*	5	3	4	4	3	-0.322	1	1
ENSBTAG00000042125	*SNORD115*	0	1	1	0	1	Inf	0.993540919	1
ENSBTAG00000042126	*SNORA66*	2	0	1	2	0		0.974934741	1
ENSBTAG00000042134	*SNORD14*	62	35	47	54	40	-0.410	0.828151054	1
ENSBTAG00000042135	*SNORA48*	0	5	3	0	6	Inf	0.757530434	1
ENSBTAG00000042142	*SNORD103*	0	4	2	0	5	Inf	0.817359038	1
ENSBTAG00000042143	*SNORD105*	1	6	4	1	7	3.000	0.788284548	1
ENSBTAG00000042154	*SNORA77*	24	16	20	21	18	-0.170	0.97319525	1
ENSBTAG00000042156	*U6*	3	2	2	3	2	-0.170	1	1
ENSBTAG00000042157	*U6*	1	0	0	1	0		1	1
ENSBTAG00000042166	*SNORA2*	83	27	52	72	31	-1.205	0.487069052	1
ENSBTAG00000042168	*U6*	1	1	1	1	1	0.415	1	1
ENSBTAG00000042169	*snoU2_19*	8	7	8	7	8	0.222	0.99532173	1
ENSBTAG00000042171	*SNORA70*	1	0	0	1	0		1	1
ENSBTAG00000042179	*U6*	4	0	2	3	0		0.89545886	1
ENSBTAG00000042180	*SNORD47*	533	118	299	462	136	-1.760	0.075436206	1
ENSBTAG00000042181	*SNORD70*	1	3	2	1	3	2.000	0.933774563	1
ENSBTAG00000042182	*U6*	1	0	0	1	0		1	1
ENSBTAG00000042183	*SNORA20*	1	2	2	1	2	1.415	0.981979269	1
ENSBTAG00000042185	*SNORD61*	1	0	0	1	0		1	1

Ensembl gene ID	geneName	counts wildtype horn bud	counts polled horn bud	baseMean	baseMean wildtype horn bud	baseMean polled horn bud	log2FoldChange	pval	padj
ENSBTAG00000042186	*SNORD22*	3	0	1	3	0		0.936647693	1
ENSBTAG00000042192	*U6*	0	2	1	0	2	Inf	0.939077559	1
ENSBTAG00000042198	*SNORD57*	14	10	12	12	12	-0.070	1	1
ENSBTAG00000042199	*SNORD113*	6	2	4	5	2	-1.170	0.934316631	1
ENSBTAG00000042201	*SNORA72*	1	0	0	1	0		1	1
ENSBTAG00000042206	*SNORD58*	57	27	40	49	31	-0.663	0.738564931	1
ENSBTAG00000042216	*U6*	2	1	1	2	1	-0.585	1	1
ENSBTAG00000042217	*SNORD77*	217	54	125	188	62	-1.592	0.196651769	1
ENSBTAG00000042224	*SNORA19*	13	1	6	11	1	-3.285	0.663144098	1
ENSBTAG00000042226	*SNORA62*	3	2	2	3	2	-0.170	1	1
ENSBTAG00000042233	*SNORD18*	2	1	1	2	1	-0.585	1	1
ENSBTAG00000042234	*SNORA70*	2	0	1	2	0		0.974934741	1
ENSBTAG00000042235	*SNORD30*	296	134	206	256	155	-0.728	0.4906383	1
ENSBTAG00000042237	*SNORA25*	1	0	0	1	0		1	1
ENSBTAG00000042240	*U6*	2	1	1	2	1	-0.585	1	1
ENSBTAG00000042245	*U6*	1	0	0	1	0		1	1
ENSBTAG00000042250	*SNORA72*	87	14	46	75	16	-2.221	0.236724535	1
ENSBTAG00000042254	*U6*	1	0	0	1	0		1	1
ENSBTAG00000042256	*SNORA74*	4	0	2	3	0		0.89545886	1
ENSBTAG00000042257	*U6*	0	2	1	0	2	Inf	0.939077559	1
ENSBTAG00000042264	*snoU2_19*	1	0	0	1	0		1	1
ENSBTAG00000042272	*SNORA79*	3	1	2	3	1	-1.170	0.985253077	1
ENSBTAG00000042273	*SNORA57*	6	2	4	5	2	-1.170	0.934316631	1
ENSBTAG00000042275	*SNORD101*	21	22	22	18	25	0.482	0.86964816	1
ENSBTAG00000042276	*SNORA30*	2	1	1	2	1	-0.585	1	1
ENSBTAG00000042278	*U6*	21	4	11	18	5	-1.977	0.646315739	1
ENSBTAG00000042279	*U6*	0	1	1	0	1	Inf	0.993540919	1
ENSBTAG00000042280	*SNORA40*	46	25	34	40	29	-0.465	0.835095566	1
ENSBTAG00000042283	*snoU2_19*	13	12	13	11	14	0.300	0.958035022	1
ENSBTAG00000042289	*SNORA38*	15	3	8	13	3	-1.907	0.733459861	1
ENSBTAG00000042292	*SNORD38*	33	45	40	29	52	0.862	0.657530334	1
ENSBTAG00000042293	*SNORA72*	145	46	89	126	53	-1.241	0.36713729	1
ENSBTAG00000042297	*SNORD72*	45	10	25	39	12	-1.755	0.48834034	1
ENSBTAG00000042298	*U6*	12	5	8	10	6	-0.848	0.892845055	1
ENSBTAG00000042304	*U6*	1	0	0	1	0		1	1
ENSBTAG00000042305	*snoZ30*	10	4	7	9	5	-0.907	0.905171387	1
ENSBTAG00000042310	*SNORA67*	3	22	14	3	25	3.290	0.388411129	1
ENSBTAG00000042312	*U6*	2	2	2	2	2	0.415	1	1
ENSBTAG00000042319	*SNORA57*	0	1	1	0	1	Inf	0.993540919	1
ENSBTAG00000042322	*SNORA71*	26	17	21	23	20	-0.198	0.961875311	1
ENSBTAG00000042335	*SNORA3*	68	38	51	59	44	-0.424	0.813334901	1
ENSBTAG00000042339	*snosnR60_Z15*	412	98	235	357	113	-1.657	0.110790355	1
ENSBTAG00000042343	*U6*	1	1	1	1	1	0.415	1	1
ENSBTAG00000042346	*SNORD113*	5	8	7	4	9	1.093	0.872919157	1
ENSBTAG00000042349	*U6*	1	0	0	1	0		1	1
ENSBTAG00000042350	*SNORD62*	59	109	88	51	126	1.301	0.344369498	1
ENSBTAG00000042353	*SNORD45*	7	4	5	6	5	-0.392	0.98714753	1
ENSBTAG00000042354	*SNORA3*	133	123	129	115	142	0.302	0.804685168	1
ENSBTAG00000042357	*SNORD21*	5	3	4	4	3	-0.322	1	1
ENSBTAG00000042358	*snoU2_19*	33	24	28	29	28	-0.044	1	1
ENSBTAG00000042359	*U6*	1	0	0	1	0		1	1
ENSBTAG00000042363	*SNORA18*	0	1	1	0	1	Inf	0.993540919	1
ENSBTAG00000042365	*SNORD113*	13	4	8	11	5	-1.285	0.828302954	1
ENSBTAG00000042366	*SNORA22*	1	1	1	1	1	0.415	1	1
ENSBTAG00000042368	*SNORD58*	40	22	30	35	25	-0.447	0.854360569	1
ENSBTAG00000042370	*SNORA56*	9	11	10	8	13	0.705	0.889330664	1
ENSBTAG00000042372	*SNORA71*	2	0	1	2	0		0.974934741	1
ENSBTAG00000042373	*U6*	0	2	1	0	2	Inf	0.939077559	1
ENSBTAG00000042377	*SNORD33*	17	26	22	15	30	1.028	0.703147919	1
ENSBTAG00000042378	*SNORA70*	3	0	1	3	0		0.936647693	1
ENSBTAG00000042381	*SNORD36*	52	48	50	45	55	0.300	0.871091584	1
ENSBTAG00000042382	*U6*	3	1	2	3	1	-1.170	0.985253077	1
ENSBTAG00000042383	*SNORD37*	12	18	16	10	21	1.000	0.77098114	1
ENSBTAG00000042385	*U6*	1	1	1	1	1	0.415	1	1
ENSBTAG00000042387	*SNORD91*	43	11	25	37	13	-1.552	0.541661086	1
ENSBTAG00000042388	*U6atac*	1	0	0	1	0		1	1
ENSBTAG00000042391	*SNORA26*	7	1	4	6	1	-2.392	0.851750348	1
ENSBTAG00000042394	*SNORA64*	16	27	23	14	31	1.170	0.661901171	1
ENSBTAG00000042398	*SNORA50*	7	1	4	6	1	-2.392	0.851750348	1
ENSBTAG00000042401	*SNORD113*	8	5	6	7	6	-0.263	0.996869191	1
ENSBTAG00000042402	*U6*	2	0	1	2	0		0.974934741	1
ENSBTAG00000042406	*SNORD18*	42	18	29	36	21	-0.807	0.734963231	1

Ensembl gene ID	geneName	counts wildtype horn bud	counts polled horn bud	baseMean	baseMean wildtype horn bud	baseMean polled horn bud	log2FoldChange	pval	padj
ENSBTAG00000042408	*SNORA27*	43	11	25	37	13	-1.552	0.541661086	1
ENSBTAG00000042411	*U6*	0	1	1	0	1	Inf	0.993540919	1
ENSBTAG00000042412	*SNORD113*	4	1	2	3	1	-1.585	0.952247636	1
ENSBTAG00000042414	*SNORA23*	11	5	8	10	6	-0.722	0.917926652	1
ENSBTAG00000042415	*U6*	3	2	2	3	2	-0.170	1	1
ENSBTAG00000042417	*SNORA53*	43	22	31	37	25	-0.552	0.812095655	1
ENSBTAG00000042418	*U6*	11	1	5	10	1	-3.044	0.722857941	1
ENSBTAG00000042432	*U6*	0	1	1	0	1	Inf	0.993540919	1
ENSBTAG00000042434	*SNORA66*	25	9	16	22	10	-1.059	0.75675668	1
ENSBTAG00000042435	*SNORA75*	8	6	7	7	7	0.000	1	1
ENSBTAG00000042438	*SNORD113*	3	3	3	3	3	0.415	1	1
ENSBTAG00000042439	*snoU2-30*	0	1	1	0	1	Inf	0.993540919	1
ENSBTAG00000042440	*SNORA9*	17	3	9	15	3	-2.087	0.685542914	1
ENSBTAG00000042442	*SNORD113*	98	15	51	85	17	-2.293	0.19953639	1
ENSBTAG00000042445	*U6*	1	0	0	1	0		1	1
ENSBTAG00000042447	*SNORD49*	262	131	189	227	151	-0.585	0.588355948	1
ENSBTAG00000042450	*SNORA40*	1	0	0	1	0		1	1
ENSBTAG00000042453	*U6*	1	0	0	1	0		1	1
ENSBTAG00000042456	*SNORD43*	12	9	10	10	10	0.000	1	1
ENSBTAG00000042458	*RNase_MRP*	234	95	156	203	110	-0.885	0.436823882	1
ENSBTAG00000042461	*snoR38*	2	0	1	2	0		0.974934741	1
ENSBTAG00000042464	*snR56*	33	14	22	29	16	-0.822	0.766955725	1
ENSBTAG00000042466	*SNORA12*	0	3	2	0	3	Inf	0.878646179	1
ENSBTAG00000042471	*SNORA25*	151	41	89	131	47	-1.466	0.289370569	1
ENSBTAG00000042475	*SNORA63*	73	21	44	63	24	-1.382	0.461629122	1
ENSBTAG00000042478	*SNORA41*	0	3	2	0	3	Inf	0.878646179	1
ENSBTAG00000042479	*SNORD26*	32	13	21	28	15	-0.885	0.755764694	1
ENSBTAG00000042482	*SNORA17*	201	225	217	174	260	0.578	0.578101589	1
ENSBTAG00000042484	*SNORD22*	172	60	109	149	69	-1.104	0.387731449	1
ENSBTAG00000042493	*SNORA70*	40	38	39	35	44	0.341	0.870633653	1
ENSBTAG00000042498	*SNORA2*	1	0	0	1	0		1	1
ENSBTAG00000042500	*U6*	2	1	1	2	1	-0.585	1	1
ENSBTAG00000042502	*SNORA1*	1	0	0	1	0		1	1
ENSBTAG00000042504	*SNORD78*	213	49	121	184	57	-1.705	0.173138251	1
ENSBTAG00000042507	*SNORA41*	90	51	68	78	59	-0.404	0.796656518	1
ENSBTAG00000042508	*SNORD113*	6	2	4	5	2	-1.170	0.934316631	1
ENSBTAG00000042518	*SNORA33*	8	3	5	7	3	-1.000	0.918861955	1
ENSBTAG00000042519	*SNORD113*	13	1	6	11	1	-3.285	0.663144098	1
ENSBTAG00000042521	*U6*	1	3	2	1	3	2.000	0.933774563	1
ENSBTAG00000042522	*SNORA11*	0	1	1	0	1	Inf	0.993540919	1
ENSBTAG00000042524	*SNORD22*	409	135	255	354	156	-1.184	0.240604921	1
ENSBTAG00000042526	*SNORD28*	43	17	28	37	20	-0.924	0.697473507	1
ENSBTAG00000042536	*SNORA2*	173	64	112	150	74	-1.020	0.420888033	1
ENSBTAG00000042538	*U6*	1	0	0	1	0		1	1
ENSBTAG00000042543	*SNORD113*	212	103	151	184	119	-0.626	0.586240929	1
ENSBTAG00000042545	*snosnR61*	149	118	133	129	136	0.079	0.953215518	1
ENSBTAG00000042546	*U6atac*	0	1	1	0	1	Inf	0.993540919	1
ENSBTAG00000042549	*SNORA67*	0	1	1	0	1	Inf	0.993540919	1
ENSBTAG00000042553	*SNORD99*	80	38	57	69	44	-0.659	0.694125098	1
ENSBTAG00000042555	*U6*	2	0	1	2	0		0.974934741	1
ENSBTAG00000042557	*U6*	1	1	1	1	1	0.415	1	1
ENSBTAG00000042559	*SNORA42*	8	6	7	7	7	0.000	1	1
ENSBTAG00000042563	*U6*	1	0	0	1	0		1	1
ENSBTAG00000042564	*U6*	3	0	1	3	0		0.936647693	1
ENSBTAG00000042565	*U6*	1	0	0	1	0		1	1
ENSBTAG00000042579	*snoU6-53*	7	6	6	6	7	0.193	1	1
ENSBTAG00000042588	*SNORD113*	3	0	1	3	0		0.936647693	1
ENSBTAG00000042594	*U5*	1	0	0	1	0		1	1
ENSBTAG00000042595	*U6*	2	0	1	2	0		0.974934741	1
ENSBTAG00000042600	*SNORD94*	66	22	41	57	25	-1.170	0.544557466	1
ENSBTAG00000042601	*7SK*	21	59	43	18	68	1.905	0.314308018	1
ENSBTAG00000042603	*SNORD113*	2	1	1	2	1	-0.585	1	1
ENSBTAG00000042604	*U6*	1	1	1	1	1	0.415	1	1
ENSBTAG00000042605	*SNORA7*	2	6	4	2	7	2.000	0.840116992	1
ENSBTAG00000042607	*SNORD56*	103	113	110	89	130	0.549	0.666277686	1
ENSBTAG00000042610	*snoU2-30*	7	3	5	6	3	-0.807	0.947428618	1
ENSBTAG00000042613	*SNORD88*	9	5	7	8	6	-0.433	0.969858938	1
ENSBTAG00000042614	*SNORA11*	6	8	7	5	9	0.830	0.903753758	1
ENSBTAG00000042624	*SNORA36*	2	0	1	2	0		0.974934741	1
ENSBTAG00000042625	*snoU2-30*	59	16	35	51	18	-1.468	0.487110667	1
ENSBTAG00000042626	*SNORD22*	45	39	42	39	45	0.209	0.923029511	1
ENSBTAG00000042629	*U6*	1	0	0	1	0		1	1

Ensembl gene ID	geneName	counts wildtype horn bud	counts polled horn bud	baseMean	baseMean wildtype horn bud	baseMean polled horn bud	log2FoldChange	pval	padj
ENSBTAG00000042630	*SNORA1*	2	0	1	2	0		0.974934741	1
ENSBTAG00000042631	*SNORD86*	364	260	308	315	300	-0.070	0.94592431	1
ENSBTAG00000042638	*SNORA11*	1	0	0	1	0		1	1
ENSBTAG00000042639	*U6*	0	1	1	0	1	Inf	0.993540919	1
ENSBTAG00000042644	*snoU6-53*	1	1	1	1	1	0.415	1	1
ENSBTAG00000042645	*SNORD46*	84	67	75	73	77	0.089	0.960316182	1
ENSBTAG00000042649	*SNORD15*	8	2	5	7	2	-1.585	0.87391615	1
ENSBTAG00000042652	*SNORA71*	17	6	11	15	7	-1.087	0.813534849	1
ENSBTAG00000042654	*SNORA11*	88	23	51	76	27	-1.521	0.382869066	1
ENSBTAG00000042660	*U6*	1	0	0	1	0		1	1
ENSBTAG00000042661	*SNORA55*	6	3	4	5	3	-0.585	0.976586913	1
ENSBTAG00000042662	*SNORD17*	13	1	6	11	1	-3.285	0.663144098	1
ENSBTAG00000042663	*SNORA73*	0	1	1	0	1	Inf	0.993540919	1
ENSBTAG00000042667	*SNORA70*	1	1	1	1	1	0.415	1	1
ENSBTAG00000042668	*U6*	0	3	2	0	3	Inf	0.878646179	1
ENSBTAG00000042669	*SNORA1*	4	0	2	3	0		0.89545886	1
ENSBTAG00000042677	*SNORD59*	35	45	41	30	52	0.778	0.687032236	1
ENSBTAG00000042678	*SNORA71*	38	18	27	33	21	-0.663	0.791807555	1
ENSBTAG00000042679	*SNORD113*	107	50	75	93	58	-0.683	0.64378463	1
ENSBTAG00000042680	*SNORA54*	5	0	2	4	0		0.853268594	1
ENSBTAG00000042681	*U6*	11	15	13	10	17	0.862	0.824700399	1
ENSBTAG00000042684	*SNORD14*	76	14	41	66	16	-2.026	0.302971628	1
ENSBTAG00000042688	*SNORA64*	48	88	72	42	102	1.290	0.387936682	1
ENSBTAG00000042690	*SNORD113*	62	45	53	54	52	-0.047	0.991646618	1
ENSBTAG00000042691	*SNORA28*	2	3	3	2	3	1.000	0.97171833	1
ENSBTAG00000042696	*SNORD73*	201	48	115	174	55	-1.651	0.193672474	1
ENSBTAG00000042697	*U6*	0	1	1	0	1	Inf	0.993540919	1
ENSBTAG00000042699	*SNORA63*	4	0	2	3	0		0.89545886	1
ENSBTAG00000042702	*SNORA4*	54	27	39	47	31	-0.585	0.773963247	1
ENSBTAG00000042705	*U6*	8	2	5	7	2	-1.585	0.87391615	1
ENSBTAG00000042706	*snoR38*	2	0	1	2	0		0.974934741	1
ENSBTAG00000042707	*U6*	2	1	1	2	1	-0.585	1	1
ENSBTAG00000042711	*snoU2_19*	25	29	28	22	33	0.629	0.796761755	1
ENSBTAG00000042713	*SNORA28*	35	12	22	30	14	-1.129	0.681233895	1
ENSBTAG00000042718	*SNORA58*	1	1	1	1	1	0.415	1	1
ENSBTAG00000042723	*U11*	0	1	1	0	1	Inf	0.993540919	1
ENSBTAG00000042726	*U6*	1	1	1	1	1	0.415	1	1
ENSBTAG00000042727	*SNORD113*	14	8	11	12	9	-0.392	0.950061617	1
ENSBTAG00000042733	*SNORD14*	2	0	1	2	0		0.974934741	1
ENSBTAG00000042734	*U6*	4	1	2	3	1	-1.585	0.952247636	1
ENSBTAG00000042735	*SNORA25*	41	18	28	36	21	-0.773	0.748829362	1
ENSBTAG00000042736	*U6*	4	10	8	3	12	1.737	0.768040958	1
ENSBTAG00000042738	*U6*	1	0	0	1	0		1	1
ENSBTAG00000042739	*SNORD113*	27	6	15	23	7	-1.755	0.616167115	1
ENSBTAG00000042741	*SNORND104*	118	32	70	102	37	-1.468	0.335842452	1
ENSBTAG00000042742	*SNORD88*	7	5	6	6	6	-0.070	1	1
ENSBTAG00000042743	*SNORA70*	6	0	3	5	0		0.81115604	1
ENSBTAG00000042744	*SNORA71*	16	16	16	14	18	0.415	0.913757539	1
ENSBTAG00000042750	*U6atac*	1	0	0	1	0		1	1
ENSBTAG00000042751	*SNORD60*	1	0	0	1	0		1	1
ENSBTAG00000042752	*SNORA3*	0	1	1	0	1	Inf	0.993540919	1
ENSBTAG00000042753	*SNORA62*	5	0	2	4	0		0.853268594	1
ENSBTAG00000042757	*SNORA20*	165	114	137	143	132	-0.118	0.926839683	1
ENSBTAG00000042758	*SNORD29*	178	91	130	154	105	-0.553	0.647749537	1
ENSBTAG00000042761	*Y_RNA*	13	7	10	11	8	-0.478	0.940171262	1
ENSBTAG00000042771	*SNORA72*	4	1	2	3	1	-1.585	0.952247636	1
ENSBTAG00000042775	*SNORD103*	22	12	16	19	14	-0.459	0.903495485	1
ENSBTAG00000042780	*SNORD91*	9	5	7	8	6	-0.433	0.969858938	1
ENSBTAG00000042784	*SNORD88*	3	0	1	3	0		0.936647693	1
ENSBTAG00000042785	*U6*	0	5	3	0	6	Inf	0.757530434	1
ENSBTAG00000042786	*snoRNA*	17	12	14	15	14	-0.087	1	1
ENSBTAG00000042787	*SNORA30*	1	2	2	1	2	1.415	0.981979269	1
ENSBTAG00000042789	*U6*	0	1	1	0	1	Inf	0.993540919	1
ENSBTAG00000042792	*Telomerase-vert*	6	3	4	5	3	-0.585	0.976586913	1
ENSBTAG00000042801	*SNORD100*	57	37	46	49	43	-0.208	0.920652352	1
ENSBTAG00000042803	*SNORA11*	2	4	3	2	5	1.415	0.927250049	1
ENSBTAG00000042805	*SNORD113*	0	1	1	0	1	Inf	0.993540919	1
ENSBTAG00000042812	*SNORD116*	4	2	3	3	2	-0.585	0.996239345	1
ENSBTAG00000042818	*U6*	0	1	1	0	1	Inf	0.993540919	1
ENSBTAG00000042819	*snoZ39*	22	5	12	19	6	-1.722	0.669657938	1
ENSBTAG00000042827	*U6*	0	1	1	0	1	Inf	0.993540919	1
ENSBTAG00000042830	*U6*	1	0	0	1	0		1	1

Ensembl gene ID	geneName	counts wildtype horn bud	counts polled horn bud	baseMean	baseMean wildtype horn bud	baseMean polled horn bud	log2FoldChange	pval	padj
ENSBTAG00000042832	*U6*	2	1	1	2	1	-0.585	1	1
ENSBTAG00000042833	*SNORA33*	146	48	91	126	55	-1.190	0.383896213	1
ENSBTAG00000042836	*SNORA48*	25	20	22	22	23	0.093	0.990498651	1
ENSBTAG00000042837	*snoU2_19*	132	45	83	114	52	-1.138	0.420851388	1
ENSBTAG00000042842	*SNORA2*	2	0	1	2	0		0.974934741	1
ENSBTAG00000042845	*SNORD113*	5	1	3	4	1	-1.907	0.9188325	1
ENSBTAG00000042846	*snoZ40*	123	35	73	107	40	-1.398	0.34784108	1
ENSBTAG00000042853	*miRNA*	7	1	4	6	1	-2.392	0.851750348	1
ENSBTAG00000042863	*SNORA7*	12	20	17	10	23	1.152	0.722217561	1
ENSBTAG00000042867	*U6*	3	4	4	3	5	0.830	0.962490925	1
ENSBTAG00000042868	*SNORA67*	28	51	42	24	59	1.280	0.502128104	1
ENSBTAG00000042871	*SNORA44*	22	28	26	19	32	0.763	0.761718009	1
ENSBTAG00000042875	*SNORA62*	54	38	45	47	44	-0.092	0.974142025	1
ENSBTAG00000042878	*SNORD22*	20	7	13	17	8	-1.100	0.786059398	1
ENSBTAG00000042879	*U6*	3	0	1	3	0		0.936647693	1
ENSBTAG00000042880	*SNORA42*	2	1	1	2	1	-0.585	1	1
ENSBTAG00000042882	*SNORD113*	41	28	34	36	32	-0.135	0.964886952	1
ENSBTAG00000042883	*SNORD35*	64	50	57	55	58	0.059	0.98268546	1
ENSBTAG00000042885	*U6*	1	0	0	1	0		1	1
ENSBTAG00000042891	*U6*	1	1	1	1	1	0.415	1	1
ENSBTAG00000042898	*snoU2-30*	15	4	9	13	5	-1.492	0.779648463	1
ENSBTAG00000042916	*SNORD15*	52	13	30	45	15	-1.585	0.48887018	1
ENSBTAG00000042917	*U6*	1	0	0	1	0		1	1
ENSBTAG00000042923	*SNORA62*	2	0	1	2	0		0.974934741	1
ENSBTAG00000042924	*SNORA70*	5	0	2	4	0		0.853268594	1
ENSBTAG00000042926	*U6*	2	0	1	2	0		0.974934741	1
ENSBTAG00000042928	*U6*	2	2	2	2	2	0.415	1	1
ENSBTAG00000042930	*SNORA17*	303	210	252	262	242	-0.114	0.914050948	1
ENSBTAG00000042931	*SNORD75*	41	15	26	36	17	-1.036	0.67517422	1
ENSBTAG00000042936	*SNORD96*	485	326	398	420	376	-0.158	0.866328719	1
ENSBTAG00000042937	*U6*	1	0	0	1	0		1	1
ENSBTAG00000042941	*SNORA1*	1	0	0	1	0		1	1
ENSBTAG00000042946	*snoU83B*	42	29	35	36	33	-0.119	0.970205342	1
ENSBTAG00000042949	*U6*	1	0	0	1	0		1	1
ENSBTAG00000042950	*SNORD45*	40	10	23	35	12	-1.585	0.551930545	1
ENSBTAG00000042953	*SNORD113*	4	3	3	3	3	0.000	1	1
ENSBTAG00000042956	*U5*	2	0	1	2	0		0.974934741	1
ENSBTAG00000042961	*U6*	2	1	1	2	1	-0.585	1	1
ENSBTAG00000042963	*SNORA8*	460	118	267	398	136	-1.548	0.124272831	1
ENSBTAG00000042970	*U6*	70	52	60	61	60	-0.014	1	1
ENSBTAG00000042974	*SNORD24*	308	93	187	267	107	-1.313	0.228298124	1
ENSBTAG00000042981	*U6*	1	1	1	1	1	0.415	1	1
ENSBTAG00000042982	*U6*	1	0	0	1	0		1	1
ENSBTAG00000042984	*SNORA38*	9	20	15	8	23	1.567	0.644190163	1
ENSBTAG00000042986	*SNORA16*	28	17	22	24	20	-0.305	0.926707752	1
ENSBTAG00000042989	*SNORD52*	65	35	48	56	40	-0.478	0.794969834	1
ENSBTAG00000043000	*SNORA1*	185	72	122	160	83	-0.946	0.441974238	1
ENSBTAG00000043003	*7SK*	1	0	0	1	0		1	1
ENSBTAG00000043006	*SNORD31*	3	1	2	3	1	-1.170	0.985253077	1
ENSBTAG00000043014	*U6*	1	0	0	1	0		1	1
ENSBTAG00000043016	*snR47*	24	36	31	21	42	1.000	0.652683277	1
ENSBTAG00000043023	*U6*	2	1	1	2	1	-0.585	1	1
ENSBTAG00000043024	*U6*	1	0	0	1	0		1	1
ENSBTAG00000043032	*U6*	1	0	0	1	0		1	1
ENSBTAG00000043034	*SNORD113*	1	1	1	1	1	0.415	1	1
ENSBTAG00000043036	*SNORD12*	406	118	244	352	136	-1.368	0.181158083	1
ENSBTAG00000043038	*U6*	1	0	0	1	0		1	1
ENSBTAG00000043040	*SNORD113*	11	3	6	10	3	-1.459	0.83622464	1
ENSBTAG00000043045	*U6*	3	0	1	3	0		0.936647693	1
ENSBTAG00000043047	*SNORA65*	2	7	5	2	8	2.222	0.797845137	1
ENSBTAG00000043048	*SNORD59*	8	7	8	7	8	0.222	0.99532173	1
ENSBTAG00000043049	*SNORD111*	14	11	12	12	13	0.067	1	1
ENSBTAG00000043066	*U6*	11	4	7	10	5	-1.044	0.879025484	1
ENSBTAG00000043068	*SNORA2*	1	0	0	1	0		1	1
ENSBTAG00000043071	*SNORA8*	1	0	0	1	0		1	1
ENSBTAG00000043073	*U6*	1	0	0	1	0		1	1
ENSBTAG00000043077	*SNORA51*	22	14	18	19	16	-0.237	0.958766648	1
ENSBTAG00000043081	*SNORD98*	25	7	15	22	8	-1.421	0.689157104	1
ENSBTAG00000043086	*Y_RNA*	13	0	6	11	0		0.551229012	1
ENSBTAG00000043090	*U6*	1	0	0	1	0		1	1
ENSBTAG00000043093	*SNORA11*	166	62	108	144	72	-1.006	0.433232357	1
ENSBTAG00000043098	*SNORD67*	2	0	1	2	0		0.974934741	1

Ensembl gene ID	geneName	counts wildtype horn bud	counts polled horn bud	baseMean	baseMean wildtype horn bud	baseMean polled horn bud	log2FoldChange	pval	padj
ENSBTAG00000043099	*U6*	2	3	3	2	3	1.000	0.97171833	1
ENSBTAG00000043100	*U5*	11	1	5	10	1	-3.044	0.722857941	1
ENSBTAG00000043101	*SNORD24*	12	18	16	10	21	1.000	0.77098114	1
ENSBTAG00000043103	*SNORA8*	2	3	3	2	3	1.000	0.97171833	1
ENSBTAG00000043105	*SNORA65*	4	2	3	3	2	-0.585	0.996239345	1
ENSBTAG00000043107	*U6*	1	0	0	1	0		1	1
ENSBTAG00000043112	*U6*	0	1	1	0	1	Inf	0.993540919	1
ENSBTAG00000043116	*U6*	1	0	0	1	0		1	1
ENSBTAG00000043121	*SNORA5*	0	1	1	0	1	Inf	0.993540919	1
ENSBTAG00000043122	*SNORA61*	1	1	1	1	1	0.415	1	1
ENSBTAG00000043123	*U6*	2	0	1	2	0		0.974934741	1
ENSBTAG00000043125	*U11*	3	0	1	3	0		0.936647693	1
ENSBTAG00000043126	*SNORA56*	2	1	1	2	1	-0.585	1	1
ENSBTAG00000043128	*SNORA69*	13	4	8	11	5	-1.285	0.828302954	1
ENSBTAG00000043133	*U6*	0	1	1	0	1	Inf	0.993540919	1
ENSBTAG00000043135	*U4atac*	0	1	1	0	1	Inf	0.993540919	1
ENSBTAG00000043140	*SNORD69*	18	7	12	16	8	-0.948	0.827785041	1
ENSBTAG00000043144	*U6*	2	0	1	2	0		0.974934741	1
ENSBTAG00000043145	*U6*	1	1	1	1	1	0.415	1	1
ENSBTAG00000043151	*SNORD48*	14	11	12	12	13	0.067	1	1
ENSBTAG00000043152	*U6*	1	0	0	1	0		1	1
ENSBTAG00000043156	*U6*	0	1	1	0	1	Inf	0.993540919	1
ENSBTAG00000043157	*Telomerase-vert*	3	6	5	3	7	1.415	0.880191876	1
ENSBTAG00000043158	*SNORD89*	22	9	15	19	10	-0.874	0.813237758	1
ENSBTAG00000043162	*SNORA74*	3	1	2	3	1	-1.170	0.985253077	1
ENSBTAG00000043165	*SNORA70*	5	3	4	4	3	-0.322	1	1
ENSBTAG00000043166	*snoMe28S-Am982*	33	21	26	29	24	-0.237	0.93825302	1
ENSBTAG00000043167	*SNORA52*	62	46	53	54	53	-0.016	1	1
ENSBTAG00000043168	*SNORD35*	15	26	22	13	30	1.209	0.660290471	1
ENSBTAG00000043171	*7SK*	31	2	15	27	2	-3.539	0.360208319	1
ENSBTAG00000043177	*SNORA16*	6	3	4	5	3	-0.585	0.976586913	1
ENSBTAG00000043179	*SNORD113*	10	3	6	9	3	-1.322	0.863266279	1
ENSBTAG00000043187	*SNORA3*	40	23	31	35	27	-0.383	0.876135313	1
ENSBTAG00000043192	*SNORA50*	0	1	1	0	1	Inf	0.993540919	1
ENSBTAG00000043193	*SNORA70*	3	2	2	3	2	-0.170	1	1
ENSBTAG00000043194	*SNORD103*	13	2	7	11	2	-2.285	0.731320841	1
ENSBTAG00000043196	*snoU6-53*	3	1	2	3	1	-1.170	0.985253077	1
ENSBTAG00000043197	*SNORD33*	43	42	43	37	48	0.381	0.846526887	1
ENSBTAG00000043199	*U6*	3	1	2	3	1	-1.170	0.985253077	1
ENSBTAG00000043202	*SNORD50*	280	56	154	242	65	-1.907	0.102733395	1
ENSBTAG00000043207	*SNORD74*	57	17	34	49	20	-1.330	0.530152091	1
ENSBTAG00000043214	*SNORA62*	36	15	24	31	17	-0.848	0.747030072	1
ENSBTAG00000043217	*SNORA29*	33	15	23	29	17	-0.722	0.793259482	1
ENSBTAG00000043222	*SNORA12*	67	25	43	58	29	-1.007	0.592977924	1
ENSBTAG00000043223	*U6*	1	0	0	1	0		1	1
ENSBTAG00000043224	*SNORD95*	40	19	28	35	22	-0.659	0.786478102	1
ENSBTAG00000043226	*SNORA33*	4	7	6	3	8	1.222	0.876605346	1
ENSBTAG00000043227	*U6*	6	1	3	5	1	-2.170	0.885240772	1
ENSBTAG00000043232	*U6*	2	0	1	2	0		0.974934741	1
ENSBTAG00000043233	*SNORA27*	31	15	22	27	17	-0.632	0.825445441	1
ENSBTAG00000043234	*SNORD87*	86	24	51	74	28	-1.426	0.413638792	1
ENSBTAG00000043237	*SNORA11*	391	254	316	339	293	-0.207	0.831650825	1
ENSBTAG00000043240	*SNORD23*	24	14	18	21	16	-0.363	0.920962882	1
ENSBTAG00000043243	*SNORA31*	72	29	48	62	33	-0.897	0.618058565	1
ENSBTAG00000043249	*SNORA2*	5	2	3	4	2	-0.907	0.965043111	1
ENSBTAG00000043257	*U6*	0	1	1	0	1	Inf	0.993540919	1
ENSBTAG00000043258	*SNORA18*	114	49	78	99	57	-0.803	0.580368027	1
ENSBTAG00000043260	*SNORD88*	7	3	5	6	3	-0.807	0.947428618	1
ENSBTAG00000043261	*SNORD70*	0	2	1	0	2	Inf	0.939077559	1
ENSBTAG00000043262	*U6*	1	0	0	1	0		1	1
ENSBTAG00000043263	*SNORD58*	12	6	9	10	7	-0.585	0.929512138	1
ENSBTAG00000043268	*SNORD79*	173	23	88	150	27	-2.496	0.082170283	1
ENSBTAG00000043271	*SNORA20*	2	0	1	2	0		0.974934741	1
ENSBTAG00000043272	*U5*	9	1	4	8	1	-2.755	0.785977687	1
ENSBTAG00000043278	*U6*	1	0	0	1	0		1	1
ENSBTAG00000043282	*SNORA70*	8	12	10	7	14	1.000	0.83142557	1
ENSBTAG00000043284	*U6*	2	0	1	2	0		0.974934741	1
ENSBTAG00000043287	*SNORD56*	1	1	1	1	1	0.415	1	1
ENSBTAG00000043291	*U6atac*	0	1	1	0	1	Inf	0.993540919	1
ENSBTAG00000043295	*SNORA13*	93	15	49	81	17	-2.217	0.222657265	1
ENSBTAG00000043296	*U6*	12	3	7	10	3	-1.585	0.809704871	1
ENSBTAG00000043297	*SNORD20*	44	16	28	38	18	-1.044	0.659856401	1

Ensembl gene ID	geneName	counts wildtype horn bud	counts polled horn bud	baseMean	baseMean wildtype horn bud	baseMean polled horn bud	log2FoldChange	pval	padj
ENSBTAG00000043301	*SNORD58*	84	37	58	73	43	-0.768	0.642224065	1
ENSBTAG00000043302	*SNORD31*	396	123	242	343	142	-1.272	0.213379865	1
ENSBTAG00000043306	*SNORD110*	14	6	10	12	7	-0.807	0.881616426	1
ENSBTAG00000043313	*U6*	0	1	1	0	1	Inf	0.993540919	1
ENSBTAG00000043315	*SNORA61*	62	36	48	54	42	-0.369	0.845389321	1
ENSBTAG00000043316	*U6*	1	0	0	1	0		1	1
ENSBTAG00000043323	*U6*	1	0	0	1	0		1	1
ENSBTAG00000043325	*SNORD14*	11	9	10	10	10	0.126	1	1
ENSBTAG00000043327	*SNORA72*	31	6	17	27	7	-1.954	0.549257798	1
ENSBTAG00000043329	*SNORA49*	7	0	3	6	0		0.769774961	1
ENSBTAG00000043333	*U6*	2	0	1	2	0		0.974934741	1
ENSBTAG00000043335	*U6*	1	0	0	1	0		1	1
ENSBTAG00000043341	*SNORD36*	58	87	75	50	100	1.000	0.493249242	1
ENSBTAG00000043342	*SNORD113*	3	6	5	3	7	1.415	0.880191876	1
ENSBTAG00000043344	*SNORA70*	1	0	0	1	0		1	1
ENSBTAG00000043347	*SNORA36*	102	75	87	88	87	-0.029	0.994031325	1
ENSBTAG00000043348	*U6*	2	0	1	2	0		0.974934741	1
ENSBTAG00000043349	*snoU2-30*	28	6	16	24	7	-1.807	0.598818952	1
ENSBTAG00000043350	*SNORD12*	332	76	188	288	88	-1.712	0.11940199	1
ENSBTAG00000043354	*SNORD113*	6	7	7	5	8	0.637	0.939319729	1
ENSBTAG00000043359	*SNORA21*	4	3	3	3	3	0.000	1	1
ENSBTAG00000043360	*U4*	2	1	1	2	1	-0.585	1	1
ENSBTAG00000043361	*U6*	6	1	3	5	1	-2.170	0.885240772	1
ENSBTAG00000043364	*U6*	3	1	2	3	1	-1.170	0.985253077	1
ENSBTAG00000043369	*U6*	3	0	1	3	0		0.936647693	1
ENSBTAG00000043371	*7SK*	0	1	1	0	1	Inf	0.993540919	1
ENSBTAG00000043372	*U6*	1	0	0	1	0		1	1
ENSBTAG00000043373	*SNORD62*	3	3	3	3	3	0.415	1	1
ENSBTAG00000043376	*snoU2_19*	14	12	13	12	14	0.193	0.980949072	1
ENSBTAG00000043377	*SNORD16*	20	16	18	17	18	0.093	0.996214168	1
ENSBTAG00000043378	*SNORD81*	418	110	245	362	127	-1.511	0.140721954	1
ENSBTAG00000043380	*U6*	3	0	1	3	0		0.936647693	1
ENSBTAG00000043382	*CPEB3_ribozyme*	0	1	1	0	1	Inf	0.993540919	1
ENSBTAG00000043383	*SNORD24*	15	13	14	13	15	0.209	0.974190924	1
ENSBTAG00000043392	*SNORD83*	27	17	22	23	20	-0.252	0.944138581	1
ENSBTAG00000043394	*SNORA32*	112	22	61	97	25	-1.933	0.235387921	1
ENSBTAG00000043395	*SNORA30*	1	0	0	1	0		1	1
ENSBTAG00000043396	*U6*	2	1	1	2	1	-0.585	1	1
ENSBTAG00000043398	*SNORA5*	3	1	2	3	1	-1.170	0.985253077	1
ENSBTAG00000043399	*SNORA20*	5	4	4	4	5	0.093	1	1
ENSBTAG00000043400	*U6*	30	13	20	26	15	-0.791	0.788388247	1
ENSBTAG00000043402	*SNORA43*	29	69	52	25	80	1.666	0.332728963	1
ENSBTAG00000043412	*SNORA71*	1	1	1	1	1	0.415	1	1
ENSBTAG00000043413	*SNORD113*	3	2	2	3	2	-0.170	1	1
ENSBTAG00000043414	*snoR38*	46	21	32	40	24	-0.716	0.749794005	1
ENSBTAG00000043415	*U6*	0	1	1	0	1	Inf	0.993540919	1
ENSBTAG00000043417	*SNORD113*	7	3	5	6	3	-0.807	0.947428618	1
ENSBTAG00000043425	*SNORA30*	1	0	0	1	0		1	1
ENSBTAG00000043430	*SNORA11*	0	1	1	0	1	Inf	0.993540919	1
ENSBTAG00000043432	*SNORD44*	178	44	102	154	51	-1.601	0.22468456	1
ENSBTAG00000043438	*U6*	2	0	1	2	0		0.974934741	1
ENSBTAG00000043440	*U6*	4	0	2	3	0		0.89545886	1
ENSBTAG00000043442	*SNORD65*	198	79	131	171	91	-0.911	0.448217748	1
ENSBTAG00000043444	*SNORD14*	3	0	1	3	0		0.936647693	1
ENSBTAG00000043446	*SNORA51*	5	8	7	4	9	1.093	0.872919157	1
ENSBTAG00000043447	*SNORD45*	9	2	5	8	2	-1.755	0.844314416	1
ENSBTAG00000043453	*SNORD34*	14	23	19	12	27	1.131	0.701095973	1
ENSBTAG00000043462	*SNORD14*	83	47	63	72	54	-0.405	0.803536913	1
ENSBTAG00000043465	*U6*	16	4	9	14	5	-1.585	0.756102726	1
ENSBTAG00000043468	*SNORD50*	205	65	126	178	75	-1.242	0.308634936	1
ENSBTAG00000043472	*SNORD71*	3	3	3	3	3	0.415	1	1
ENSBTAG00000043476	*SNORA70*	1	6	4	1	7	3.000	0.788284548	1
ENSBTAG00000043478	*SNORA71*	1	0	0	1	0		1	1
ENSBTAG00000043480	*SNORD14*	61	28	43	53	32	-0.708	0.712589693	1
ENSBTAG00000043481	*SNORA70*	2	0	1	2	0		0.974934741	1
ENSBTAG00000043482	*7SK*	92	74	83	80	85	0.101	0.950822221	1
ENSBTAG00000043483	*SNORD83*	4	6	5	3	7	1.000	0.91481797	1
ENSBTAG00000043486	*U6*	0	2	1	0	2	Inf	0.939077559	1
ENSBTAG00000043487	*U6*	1	1	1	1	1	0.415	1	1
ENSBTAG00000043490	*SNORD113*	273	126	191	236	145	-0.700	0.515423194	1
ENSBTAG00000043502	*snoU83B*	59	43	50	51	50	-0.041	0.995333574	1
ENSBTAG00000043508	*U8*	182	77	123	158	89	-0.826	0.500325832	1

Ensembl gene ID	geneName	counts wildtype horn bud	counts polled horn bud	baseMean	baseMean wildtype horn bud	baseMean polled horn bud	log2FoldChange	pval	padj
ENSBTAG00000043509	*SNORD27*	51	22	35	44	25	-0.798	0.708195583	1
ENSBTAG00000043512	*SNORA68*	13	17	15	11	20	0.802	0.820295848	1
ENSBTAG00000043514	*SNORA62*	2	0	1	2	0		0.974934741	1
ENSBTAG00000043519	*SNORD93*	26	2	12	23	2	-3.285	0.441095735	1
ENSBTAG00000043521	*SNORA70*	12	11	12	10	13	0.290	0.964579659	1
ENSBTAG00000043528	*SNORA46*	4	0	2	3	0		0.89545886	1
ENSBTAG00000043530	*SNORA24*	156	51	97	135	59	-1.198	0.369356693	1
ENSBTAG00000043531	*SNORD18*	12	12	12	10	14	0.415	0.934466298	1
ENSBTAG00000043541	*SNORA5*	3	8	6	3	9	1.830	0.801688691	1
ENSBTAG00000043542	*U6*	2	0	1	2	0		0.974934741	1
ENSBTAG00000043546	*ND6*	20'612	7'790	13'423	17'851	8'995	-0.989	0.198755892	1
ENSBTAG00000043548	*Mt_tRNA*	1	0	0	1	0		1	1
ENSBTAG00000043550	*MT-CYB*	185'847	126'781	153'671	160'948	146'394	-0.137	0.857488384	1
ENSBTAG00000043553	*GPX3*	568	239	384	492	276	-0.834	0.369151051	1
ENSBTAG00000043556	*MT-CO2*	130'868	96'695	112'494	113'335	111'654	-0.022	0.977995106	1
ENSBTAG00000043558	*ND1*	167'019	96'016	127'756	144'643	110'870	-0.384	0.613740089	1
ENSBTAG00000043559	*ND4L*	12'709	13'938	13'550	11'006	16'094	0.548	0.472867835	1
ENSBTAG00000043560	*MT-CO3*	234'386	152'321	189'435	202'984	175'885	-0.207	0.785689305	1
ENSBTAG00000043561	*COX1*	710'375	549'872	625'070	615'203	634'937	0.046	0.951370667	1
ENSBTAG00000043563	*ND5*	60'278	29'983	43'412	52'202	34'621	-0.592	0.436868277	1
ENSBTAG00000043564	*MT-ATP8*	23'074	49'683	38'676	19'983	57'369	1.522	0.049537676	1
ENSBTAG00000043567	*Mt_rRNA*	14'763	4'787	9'156	12'785	5'528	-1.210	0.118496898	1
ENSBTAG00000043568	*MT-ND3*	100'123	40'122	66'519	86'709	46'329	-0.904	0.236744423	1
ENSBTAG00000043570	*Mt_rRNA*	30'683	8'019	17'916	26'572	9'260	-1.521	0.050228767	1
ENSBTAG00000043571	*ND2*	96'263	56'420	74'257	83'366	65'148	-0.356	0.6399115	1
ENSBTAG00000043573	*Mt_tRNA*	1	1	1	1	1	0.415	1	1
ENSBTAG00000043577	*MT-ND4*	145'393	86'196	112'722	125'914	99'531	-0.339	0.655377054	1
ENSBTAG00000043578	*protein_coding*	2	0	1	2	0		0.974934741	1
ENSBTAG00000043581	*TXNRD2*	747	830	803	647	958	0.567	0.500171056	1
ENSBTAG00000043582	*Mt_tRNA*	18'367	10'123	13'798	15'906	11'689	-0.444	0.561109607	1
ENSBTAG00000043584	*ATP6*	241'593	120'188	174'003	209'226	138'781	-0.592	0.436302586	1
ENSBTAG00000043589	*SNORA79*	13	3	7	11	3	-1.700	0.783725709	1
ENSBTAG00000043590	*SNORA58*	14	14	14	12	16	0.415	0.923601843	1
ENSBTAG00000043602	*7SK*	27	8	16	23	9	-1.340	0.688031079	1
ENSBTAG00000043634	*SNORA42*	3	2	2	3	2	-0.170	1	1
ENSBTAG00000043637	*5S_rRNA*	0	1	1	0	1	Inf	0.993540919	1
ENSBTAG00000043638	*7SK*	0	1	1	0	1	Inf	0.993540919	1
ENSBTAG00000043639	*7SK*	54	24	37	47	28	-0.755	0.713884439	1
ENSBTAG00000043641	*SNORA19*	18	11	14	16	13	-0.295	0.954936444	1
ENSBTAG00000043642	*miRNA*	0	1	1	0	1	Inf	0.993540919	1
ENSBTAG00000043643	*U2*	2	1	1	2	1	-0.585	1	1
ENSBTAG00000043654	*U6*	12	3	7	10	3	-1.585	0.809704871	1
ENSBTAG00000043655	*5S_rRNA*	1	1	1	1	1	0.415	1	1
ENSBTAG00000043656	*SNORA19*	4	0	2	3	0		0.89545886	1
ENSBTAG00000043657	*7SK*	0	1	1	0	1	Inf	0.993540919	1
ENSBTAG00000043661	*7SK*	1	0	0	1	0		1	1
ENSBTAG00000043663	*U4*	1	0	0	1	0		1	1
ENSBTAG00000043670	*SNORD113*	3	2	2	3	2	-0.170	1	1
ENSBTAG00000043671	*7SK*	3	1	2	3	1	-1.170	0.985253077	1
ENSBTAG00000043676	*U2*	1	0	0	1	0		1	1
ENSBTAG00000043683	*miRNA*	2	0	1	2	0		0.974934741	1
ENSBTAG00000043694	*SNORD113*	2	1	1	2	1	-0.585	1	1
ENSBTAG00000043695	*bta-mir-450b*	196	80	131	170	92	-0.878	0.465008102	1
ENSBTAG00000043706	*miRNA*	233	106	162	202	122	-0.721	0.522045624	1
ENSBTAG00000043708	*U6*	4	2	3	3	2	-0.585	0.996239345	1
ENSBTAG00000043716	*U6*	3	2	2	3	2	-0.170	1	1
ENSBTAG00000043717	*SNORA22*	1	3	2	1	3	2.000	0.933774563	1
ENSBTAG00000043719	*SNORA19*	1	0	0	1	0		1	1
ENSBTAG00000043729	*miRNA*	12	5	8	10	6	-0.848	0.892845055	1
ENSBTAG00000043732	*U2*	1	0	0	1	0		1	1
ENSBTAG00000043738	*7SK*	11	0	5	10	0		0.617662983	1
ENSBTAG00000043747	*U2*	1	1	1	1	1	0.415	1	1
ENSBTAG00000043775	*miRNA*	37	3	18	32	3	-3.209	0.334323923	1
ENSBTAG00000043778	*SNORD41*	1	4	3	1	5	2.415	0.884888347	1
ENSBTAG00000043784	*7SK*	0	1	1	0	1	Inf	0.993540919	1
ENSBTAG00000043786	*U6*	1	0	0	1	0		1	1
ENSBTAG00000043795	*SNORD31*	2	1	1	2	1	-0.585	1	1
ENSBTAG00000043803	*miRNA*	8	5	6	7	6	-0.263	0.996869191	1
ENSBTAG00000043814	*5S_rRNA*	1	0	0	1	0		1	1
ENSBTAG00000043819	*U6*	0	1	1	0	1	Inf	0.993540919	1
ENSBTAG00000043822	*U4*	1	0	0	1	0		1	1
ENSBTAG00000043823	*U6*	2	0	1	2	0		0.974934741	1

Ensembl gene ID	geneName	counts wildtype horn bud	counts polled horn bud	baseMean	baseMean wildtype horn bud	baseMean polled horn bud	log2FoldChange	pval	padj
ENSBTAG00000043826	*U6*	4	8	6	3	9	1.415	0.839396723	1
ENSBTAG00000043829	*miRNA*	1	2	2	1	2	1.415	0.981979269	1
ENSBTAG00000043830	*7SK*	2	0	1	2	0		0.974934741	1
ENSBTAG00000043833	*5S_rRNA*	2	0	1	2	0		0.974934741	1
ENSBTAG00000043834	*miRNA*	1	0	0	1	0		1	1
ENSBTAG00000043836	*5S_rRNA*	0	1	1	0	1	Inf	0.993540919	1
ENSBTAG00000043846	*5S_rRNA*	1	1	1	1	1	0.415	1	1
ENSBTAG00000043848	*5S_rRNA*	23	6	13	20	7	-1.524	0.689823929	1
ENSBTAG00000043853	*5S_rRNA*	3	1	2	3	1	-1.170	0.985253077	1
ENSBTAG00000043859	*SNORD113*	15	7	11	13	8	-0.684	0.893749952	1
ENSBTAG00000043867	*SNORA11*	10	8	9	9	9	0.093	1	1
ENSBTAG00000043869	*bta-mir-1468*	1	0	0	1	0		1	1
ENSBTAG00000043870	*U6*	6	7	7	5	8	0.637	0.939319729	1
ENSBTAG00000043875	*U2*	1	0	0	1	0		1	1
ENSBTAG00000043879	*SNORA57*	1	0	0	1	0		1	1
ENSBTAG00000043880	*U6*	1	0	0	1	0		1	1
ENSBTAG00000043885	*7SK*	0	4	2	0	5	Inf	0.817359038	1
ENSBTAG00000043888	*SNORA35*	2	1	1	2	1	-0.585	1	1
ENSBTAG00000043893	*5S_rRNA*	2	0	1	2	0		0.974934741	1
ENSBTAG00000043900	*7SK*	1	0	0	1	0		1	1
ENSBTAG00000043904	*SNORA57*	3	1	2	3	1	-1.170	0.985253077	1
ENSBTAG00000043908	*7SK*	1	0	0	1	0		1	1
ENSBTAG00000043911	*miRNA*	5	0	2	4	0		0.853268594	1
ENSBTAG00000043914	*SNORD19*	19	5	11	16	6	-1.511	0.7314974	1
ENSBTAG00000043917	*U6*	1	0	0	1	0		1	1
ENSBTAG00000043918	*5S_rRNA*	12	12	12	10	14	0.415	0.934466298	1
ENSBTAG00000043932	*SNORA25*	1	0	0	1	0		1	1
ENSBTAG00000043933	*SNORD113*	5	1	3	4	1	-1.907	0.9188325	1
ENSBTAG00000043935	*7SK*	8	9	9	7	10	0.585	0.926558417	1
ENSBTAG00000043940	*U4*	2	1	1	2	1	-0.585	1	1
ENSBTAG00000043946	*7SK*	1	1	1	1	1	0.415	1	1
ENSBTAG00000043948	*HCN1*	147	244	205	127	282	1.146	0.27892753	1
ENSBTAG00000043949	*PAWR*	191	165	178	165	191	0.204	0.855025871	1
ENSBTAG00000043950	*LCA5*	327	144	225	283	166	-0.768	0.457436896	1
ENSBTAG00000043951	*IMPA2*	401	323	360	347	373	0.103	0.914274561	1
ENSBTAG00000043953	*SPIB*	23	23	23	20	27	0.415	0.885135718	1
ENSBTAG00000043954	*BT.53896*	2	0	1	2	0		0.974934741	1
ENSBTAG00000043955	*C7ORF72*	2	0	1	2	0		0.974934741	1
ENSBTAG00000043956	*BT.55273*	3'326	1'877	2'524	2'880	2'167	-0.410	0.602078655	1
ENSBTAG00000043957	*C8ORF42*	57	42	49	49	48	-0.026	1	1
ENSBTAG00000043958	*TMEM33*	384	330	357	333	381	0.196	0.835170835	1
ENSBTAG00000043959	*PDGFC*	859	496	658	744	573	-0.377	0.661454947	1
ENSBTAG00000043960	*BT.26545*	254	175	211	220	202	-0.122	0.911804937	1
ENSBTAG00000043961	*MPDZ*	3'744	2'624	3'136	3'242	3'030	-0.098	0.901203624	1
ENSBTAG00000043962	*BT.63103*	120	87	102	104	100	-0.049	0.979333552	1
ENSBTAG00000043963	*FHDC1*	377	361	372	326	417	0.352	0.70494722	1
ENSBTAG00000043964	*BT.38599*	61	64	63	53	74	0.484	0.761174977	1
ENSBTAG00000043969	*CALN1*	31	7	17	27	8	-1.732	0.586159122	1
ENSBTAG00000043970	*C8orf37*	773	378	553	669	436	-0.617	0.482654798	1
ENSBTAG00000043971	*NOTCH3*	5'853	13'224	10'169	5'069	15'270	1.591	0.041684198	1
ENSBTAG00000043972	*BT.74453*	0	4	2	0	5	Inf	0.817359038	1
ENSBTAG00000043974	*BT.61029*	1'061	1'226	1'167	919	1'416	0.624	0.445025347	1
ENSBTAG00000043975	*MYCT1*	363	167	254	314	193	-0.705	0.483107356	1
ENSBTAG00000043976	*RNFT2*	168	130	148	145	150	0.045	0.974470355	1
ENSBTAG00000043978	*CLVS1*	12	0	5	10	0		0.583618942	1
ENSBTAG00000043979	*BT.88345*	218	69	134	189	80	-1.245	0.298328575	1
ENSBTAG00000043981	*SBF2*	2'891	2'129	2'481	2'504	2'458	-0.026	0.974477015	1
ENSBTAG00000043983	*LRRC19*	3	2	2	3	2	-0.170	1	1
ENSBTAG00000043985	*DACH1*	227	181	203	197	209	0.088	0.937195567	1
ENSBTAG00000043987	*KDM4C*	712	435	559	617	502	-0.296	0.736904218	1
ENSBTAG00000043989	*EYA3*	592	633	622	513	731	0.512	0.553545119	1
ENSBTAG00000043990	*KHDRBS2*	34	0	15	29	0		0.163610325	1
ENSBTAG00000043992	*RNF180*	103	22	57	89	25	-1.812	0.278023157	1
ENSBTAG00000043994	*GRK6*	609	1'146	925	527	1'323	1.327	0.114467849	1
ENSBTAG00000043996	*SAMD12*	61	53	57	53	61	0.212	0.905645201	1
ENSBTAG00000043999	*REV1*	2'132	1'192	1'611	1'846	1'376	-0.424	0.597276396	1
ENSBTAG00000044000	*PRIM1*	2'194	1'112	1'592	1'900	1'284	-0.565	0.481327469	1
ENSBTAG00000044001	*LPP*	175	118	144	152	136	-0.154	0.901065278	1
ENSBTAG00000044003	*TEX10*	1'969	1'500	1'719	1'705	1'732	0.023	0.977563495	1
ENSBTAG00000044004	*C9ORF84*	11	8	9	10	9	-0.044	1	1
ENSBTAG00000044006	*BT.88212*	376	679	555	326	784	1.268	0.151919072	1
ENSBTAG00000044007	*KLF12*	197	66	123	171	76	-1.163	0.343643802	1

Ensembl gene ID	geneName	counts wildtype horn bud	counts polled horn bud	baseMean	baseMean wildtype horn bud	baseMean polled horn bud	log2FoldChange	pval	padj
ENSBTAG00000044009	*PPP1R1C*	3	0	1	3	0		0.936647693	1
ENSBTAG00000044010	*EMB*	109	40	70	94	46	-1.031	0.495004027	1
ENSBTAG00000044011	*PRTFDC1*	30	13	20	26	15	-0.791	0.788388247	1
ENSBTAG00000044012	*TMEM196*	1	0	0	1	0		1	1
ENSBTAG00000044015	*RBM12*	1'554	1'696	1'652	1'346	1'958	0.541	0.4983912	1
ENSBTAG00000044017	*MSRB3*	243	127	179	210	147	-0.521	0.635444363	1
ENSBTAG00000044018	*BT.87573*	245	214	230	212	247	0.220	0.832236008	1
ENSBTAG00000044019	*KAT2A*	1'775	2'540	2'235	1'537	2'933	0.932	0.239932071	1
ENSBTAG00000044021	*FAM179A*	2	3	3	2	3	1.000	0.97171833	1
ENSBTAG00000044022	*protein_coding*	283	568	450	245	656	1.420	0.119949365	1
ENSBTAG00000044023	*CDK6*	77	114	99	67	132	0.981	0.455459137	1
ENSBTAG00000044025	*IL1RAPL2*	90	5	42	78	6	-3.755	0.076043088	1
ENSBTAG00000044027	*ITGBL1*	8'116	4'439	6'077	7'029	5'126	-0.455	0.554720083	1
ENSBTAG00000044029	*AVEN*	1'028	513	741	890	592	-0.588	0.489211648	1
ENSBTAG00000044032	*KDM2A*	3'046	2'693	2'874	2'638	3'110	0.237	0.760963279	1
ENSBTAG00000044037	*DACH2*	1	0	0	1	0		1	1
ENSBTAG00000044038	*TEN1*	783	898	858	678	1'037	0.613	0.463741375	1
ENSBTAG00000044040	*PDAP1*	4'385	3'871	4'134	3'798	4'470	0.235	0.760878418	1
ENSBTAG00000044043	*FGF20*	1	4	3	1	5	2.415	0.884888347	1
ENSBTAG00000044044	*CLOCK*	78	52	64	68	60	-0.170	0.923969051	1
ENSBTAG00000044046	*SGCD*	555	586	579	481	677	0.493	0.571047219	1
ENSBTAG00000044047	*C10ORF140*	85	41	60	74	47	-0.637	0.695432617	1
ENSBTAG00000044048	*LMBR1L*	1'071	1'076	1'085	928	1'242	0.422	0.606766485	1
ENSBTAG00000044049	*ABHD12B*	33	6	18	29	7	-2.044	0.51823266	1
ENSBTAG00000044050	*XKR4*	70	76	74	61	88	0.534	0.718748325	1
ENSBTAG00000044053	*SLC17A5*	2'392	1'345	1'812	2'072	1'553	-0.416	0.602302903	1
ENSBTAG00000044055	*THSD7B*	237	96	158	205	111	-0.889	0.433543069	1
ENSBTAG00000044056	*C17ORF98*	5	1	3	4	1	-1.907	0.9188325	1
ENSBTAG00000044058	*MFSD8*	1'293	420	802	1'120	485	-1.207	0.155558788	1
ENSBTAG00000044059	*CHRNA10*	20	13	16	17	15	-0.206	0.970864367	1
ENSBTAG00000044061	*C6ORF168*	413	255	326	358	294	-0.281	0.770611687	1
ENSBTAG00000044062	*RBMS3*	92	110	103	80	127	0.673	0.603839653	1
ENSBTAG00000044063	*B4GALT6*	574	441	503	497	509	0.035	0.970461263	1
ENSBTAG00000044064	*RNF144A*	631	364	483	546	420	-0.379	0.672766437	1
ENSBTAG00000044065	*PITPNC1*	142	64	98	123	74	-0.735	0.579690131	1
ENSBTAG00000044066	*C1ORF101*	2	0	1	2	0		0.974934741	1
ENSBTAG00000044067	*PLAC8L1*	17	12	14	15	14	-0.087	1	1
ENSBTAG00000044068	*VWC2*	46	133	97	40	154	1.947	0.150143288	1
ENSBTAG00000044070	*SNX30*	197	229	218	171	264	0.632	0.542475413	1
ENSBTAG00000044071	*HRH2*	131	161	150	113	186	0.713	0.534803626	1
ENSBTAG00000044074	*MLLT10*	1'898	1'095	1'454	1'644	1'264	-0.379	0.638949619	1
ENSBTAG00000044075	*SRPRB*	3'797	2'810	3'267	3'288	3'245	-0.019	0.981399646	1
ENSBTAG00000044077	*POLR3G*	65	82	75	56	95	0.750	0.607547106	1
ENSBTAG00000044078	*TESC*	63	14	35	55	16	-1.755	0.404229	1
ENSBTAG00000044079	*C3ORF78*	1'025	744	873	888	859	-0.047	0.95702291	1
ENSBTAG00000044080	*CPA6*	1	3	2	1	3	2.000	0.933774563	1
ENSBTAG00000044081	*AUH*	226	100	156	196	115	-0.761	0.504220147	1
ENSBTAG00000044082	*Mrz 01*	33	4	17	29	5	-2.629	0.435665511	1
ENSBTAG00000044083	*LIMK1*	730	1'329	1'083	632	1'535	1.279	0.12309918	1
ENSBTAG00000044087	*KIAA1328*	34	22	27	29	25	-0.213	0.944749153	1
ENSBTAG00000044091	*PTK6*	56	66	62	48	76	0.652	0.681555184	1
ENSBTAG00000044092	*IQCK*	364	342	355	315	395	0.325	0.72919487	1
ENSBTAG00000044093	*protein_coding*	0	1	1	0	1	Inf	0.993540919	1
ENSBTAG00000044097	*KLF7*	251	245	250	217	283	0.380	0.705943706	1
ENSBTAG00000044099	*ZSWIM7*	1'033	505	739	895	583	-0.617	0.467742697	1
ENSBTAG00000044100	*HEATR1*	2'513	1'841	2'151	2'176	2'126	-0.034	0.967085554	1
ENSBTAG00000044101	*C14ORF159*	172	190	184	149	219	0.559	0.606176694	1
ENSBTAG00000044105	*FOXO1*	497	436	467	430	503	0.226	0.801603619	1
ENSBTAG00000044106	*KIAA0146*	755	702	732	654	811	0.310	0.714769755	1
ENSBTAG00000044107	*FAM159A*	33	35	34	29	40	0.500	0.818346205	1
ENSBTAG00000044111	*EPHA6*	33	8	19	29	9	-1.629	0.589346764	1
ENSBTAG00000044112	*MSRB2*	497	568	543	430	656	0.608	0.489092282	1
ENSBTAG00000044113	*C18H19ORF41*	30	15	22	26	17	-0.585	0.841963028	1
ENSBTAG00000044115	*RIPPLY1*	1	1	1	1	1	0.415	1	1
ENSBTAG00000044117	*ANKRD45*	15	15	15	13	17	0.415	0.918564425	1
ENSBTAG00000044118	*HMGA2*	38	61	52	33	70	1.098	0.522811961	1
ENSBTAG00000044119	*KSR2*	3	2	2	3	2	-0.170	1	1
ENSBTAG00000044121	*NUBPL*	135	86	108	117	99	-0.236	0.859953915	1
ENSBTAG00000044123	*HAND2*	2	0	1	2	0		0.974934741	1
ENSBTAG00000044125	*TMEM56*	30	9	18	26	10	-1.322	0.669937554	1
ENSBTAG00000044126	*SNTB1*	466	317	385	404	366	-0.141	0.882000448	1
ENSBTAG00000044129	*ST6GALNAC6*	1'276	1'276	1'289	1'105	1'473	0.415	0.608297137	1

Ensembl gene ID	geneName	counts wildtype horn bud	counts polled horn bud	baseMean	baseMean wildtype horn bud	baseMean polled horn bud	log2FoldChange	pval	padj
ENSBTAG00000044132	*NTN5*	11	4	7	10	5	-1.044	0.879025484	1
ENSBTAG00000044135	*PIGO*	3'660	2'753	3'174	3'170	3'179	0.004	0.995487217	1
ENSBTAG00000044137	*C10ORF67*	1	0	0	1	0		1	1
ENSBTAG00000044138	*METTL23*	158	217	194	137	251	0.873	0.414382673	1
ENSBTAG00000044141	*HEATR8*	4	1	2	3	1	-1.585	0.952247636	1
ENSBTAG00000044144	*GUCY1A2*	8	1	4	7	1	-2.585	0.818599781	1
ENSBTAG00000044148	*UTS2D*	5	0	2	4	0		0.853268594	1
ENSBTAG00000044150	*BT.28506*	459	370	412	398	427	0.104	0.91100501	1
ENSBTAG00000044151	*ANKDD1B*	12	6	9	10	7	-0.585	0.929512138	1
ENSBTAG00000044153	*SH3PXD2A*	2'406	3'870	3'276	2'084	4'469	1.101	0.161447856	1
ENSBTAG00000044155	*BSPRY*	69	49	58	60	57	-0.079	0.974010988	1
ENSBTAG00000044157	*FAM71E2*	7	4	5	6	5	-0.392	0.98714753	1
ENSBTAG00000044158	*LDLRAD3*	406	204	294	352	236	-0.578	0.553375719	1
ENSBTAG00000044159	*C4ORF33*	172	68	114	149	79	-0.924	0.463121077	1
ENSBTAG00000044160	*MCCC2*	303	299	304	262	345	0.396	0.681958364	1
ENSBTAG00000044161	*NMU*	14	2	7	12	2	-2.392	0.704665469	1
ENSBTAG00000044167	*C3ORF77*	5	7	6	4	8	0.900	0.909121035	1
ENSBTAG00000044169	*PRELID2*	236	295	273	204	341	0.737	0.455104633	1
ENSBTAG00000044171	*CNIH3*	87	50	67	75	58	-0.384	0.809676283	1
ENSBTAG00000044172	*LMLN*	84	65	74	73	75	0.045	0.985125963	1
ENSBTAG00000044173	*EHBP1*	2'561	1'920	2'217	2'218	2'217	-0.001	1	1
ENSBTAG00000044175	*CENPK*	386	204	285	334	236	-0.505	0.606975965	1
ENSBTAG00000044176	*C12ORF40*	3	0	1	3	0		0.936647693	1
ENSBTAG00000044177	*C11ORF53*	0	3	2	0	3	Inf	0.878646179	1
ENSBTAG00000044178	*ZMPSTE24*	1'106	704	885	958	813	-0.237	0.777810475	1
ENSBTAG00000044179	*BT.106453*	32	24	28	28	28	0.000	1	1
ENSBTAG00000044181	*GRM1*	16	7	11	14	8	-0.778	0.871294281	1
ENSBTAG00000044183	*SPINK7*	4	3	3	3	3	0.000	1	1
ENSBTAG00000044184	*TUBGCP3*	2'272	1'928	2'097	1'968	2'226	0.178	0.821314907	1
ENSBTAG00000044185	*SOX6*	49	14	29	42	16	-1.392	0.54797818	1
ENSBTAG00000044190	*TMEM67*	802	426	593	695	492	-0.498	0.567841269	1
ENSBTAG00000044191	*ABCC12*	5	10	8	4	12	1.415	0.803541841	1
ENSBTAG00000044192	*MAF*	1'374	606	945	1'190	700	-0.766	0.357436112	1
ENSBTAG00000044194	*ZDHHC2*	672	442	546	582	510	-0.189	0.831297952	1
ENSBTAG00000044195	*SDK2*	1'034	1'003	1'027	895	1'158	0.371	0.651918435	1
ENSBTAG00000044197	*EDARADD*	60	126	99	52	145	1.485	0.262736521	1
ENSBTAG00000044198	*THAP7*	513	405	456	444	468	0.074	0.936124641	1
ENSBTAG00000044204	*CD79B*	19	3	10	16	3	-2.248	0.640043503	1
ENSBTAG00000044207	*BT.53717*	3'140	2'158	2'606	2'719	2'492	-0.126	0.873323751	1
ENSBTAG00000044208	*DUSP4*	68	54	61	59	62	0.082	0.969149343	1
ENSBTAG00000044210	*SPO11*	1	0	0	1	0		1	1
ENSBTAG00000044212	*BT.82319*	25	72	52	22	83	1.941	0.262365964	1
ENSBTAG00000044214	*bta-mir-2399*	2	0	1	2	0		0.974934741	1
ENSBTAG00000044221	*SNORA9*	1	0	0	1	0		1	1
ENSBTAG00000044222	*bta-mir-2448*	2	0	1	2	0		0.974934741	1
ENSBTAG00000044224	*bta-mir-2467*	1	0	0	1	0		1	1
ENSBTAG00000044225	*bta-mir-2329-2*	0	1	1	0	1	Inf	0.993540919	1
ENSBTAG00000044236	*bta-mir-2299*	285	115	190	247	133	-0.894	0.407215	1
ENSBTAG00000044240	*SNORD64*	9	2	5	8	2	-1.755	0.844314416	1
ENSBTAG00000044248	*snoU13*	1	1	1	1	1	0.415	1	1
ENSBTAG00000044260	*SCARNA8*	3	1	2	3	1	-1.170	0.985253077	1
ENSBTAG00000044262	*bta-mir-2407*	5	18	13	4	21	2.263	0.566248339	1
ENSBTAG00000044273	*U6*	1	0	0	1	0		1	1
ENSBTAG00000044275	*SNORA31*	1	0	0	1	0		1	1
ENSBTAG00000044279	*U6*	0	1	1	0	1	Inf	0.993540919	1
ENSBTAG00000044280	*bta-mir-2324*	3	8	6	3	9	1.830	0.801688691	1
ENSBTAG00000044282	*miRNA*	1	0	0	1	0		1	1
ENSBTAG00000044284	*bta-mir-2357*	4	0	2	3	0		0.89545886	1
ENSBTAG00000044289	*SNORA72*	1	0	0	1	0		1	1
ENSBTAG00000044291	*7SK*	2	4	3	2	5	1.415	0.927250049	1
ENSBTAG00000044294	*bta-mir-2418*	1	0	0	1	0		1	1
ENSBTAG00000044304	*bta-mir-2303*	11	3	6	10	3	-1.459	0.83622464	1
ENSBTAG00000044308	*SCARNA3*	6	0	3	5	0		0.81115604	1
ENSBTAG00000044311	*SNORD2*	62	39	49	54	45	-0.254	0.89590076	1
ENSBTAG00000044315	*NRON*	1	0	0	1	0		1	1
ENSBTAG00000044316	*bta-mir-2367*	2	0	1	2	0		0.974934741	1
ENSBTAG00000044318	*5S_rRNA*	3	1	2	3	1	-1.170	0.985253077	1
ENSBTAG00000044326	*SNORD112*	10	5	7	9	6	-0.585	0.94357809	1
ENSBTAG00000044330	*SNORD112*	1	0	0	1	0		1	1
ENSBTAG00000044335	*U6*	1	0	0	1	0		1	1
ENSBTAG00000044340	*U6*	2	3	3	2	3	1.000	0.97171833	1
ENSBTAG00000044345	*SNORD112*	2	0	1	2	0		0.974934741	1

Ensembl gene ID	geneName	counts wildtype horn bud	counts polled horn bud	baseMean	baseMean wildtype horn bud	baseMean polled horn bud	log2FoldChange	pval	padj
ENSBTAG00000044348	*bta-mir-2466*	1	1	1	1	1	0.415	1	1
ENSBTAG00000044350	*SNORA31*	1	0	0	1	0		1	1
ENSBTAG00000044352	*SNORA31*	1	1	1	1	1	0.415	1	1
ENSBTAG00000044363	*bta-mir-2430*	0	1	1	0	1	Inf	0.993540919	1
ENSBTAG00000044364	*5S_rRNA*	1	0	0	1	0		1	1
ENSBTAG00000044375	*SNORA70*	17	20	19	15	23	0.650	0.835291049	1
ENSBTAG00000044376	*SNORA31*	2	1	1	2	1	-0.585	1	1
ENSBTAG00000044379	*5S_rRNA*	5	4	4	4	5	0.093	1	1
ENSBTAG00000044381	*U6*	18	1	8	16	1	-3.755	0.530614628	1
ENSBTAG00000044384	*U6*	1	0	0	1	0		1	1
ENSBTAG00000044391	*snoU109*	0	1	1	0	1	Inf	0.993540919	1
ENSBTAG00000044398	*bta-mir-2291*	1	0	0	1	0		1	1
ENSBTAG00000044405	*U6*	1	0	0	1	0		1	1
ENSBTAG00000044406	*SCARNA15*	2	1	1	2	1	-0.585	1	1
ENSBTAG00000044417	*5S_rRNA*	10	5	7	9	6	-0.585	0.94357809	1
ENSBTAG00000044419	*5S_rRNA*	5	1	3	4	1	-1.907	0.9188325	1
ENSBTAG00000044420	*U6*	3	3	3	3	3	0.415	1	1
ENSBTAG00000044422	*bta-mir-2459*	0	1	1	0	1	Inf	0.993540919	1
ENSBTAG00000044425	*SCARNA14*	21	6	13	18	7	-1.392	0.729267326	1
ENSBTAG00000044426	*miRNA*	1	0	0	1	0		1	1
ENSBTAG00000044427	*RNaseP_nuc*	307	85	182	266	98	-1.438	0.191370256	1
ENSBTAG00000044429	*bta-mir-2284j*	1	0	0	1	0		1	1
ENSBTAG00000044430	*SNORA70*	2	0	1	2	0		0.974934741	1
ENSBTAG00000044441	*SCARNA1*	4	1	2	3	1	-1.585	0.952247636	1
ENSBTAG00000044443	*SCARNA16*	47	17	30	41	20	-1.052	0.645458921	1
ENSBTAG00000044450	*bta-mir-199c*	1	0	0	1	0		1	1
ENSBTAG00000044457	*U6*	2	0	1	2	0		0.974934741	1
ENSBTAG00000044465	*miRNA*	1	0	0	1	0		1	1
ENSBTAG00000044466	*SCARNA20*	16	8	12	14	9	-0.585	0.904936474	1
ENSBTAG00000044469	*bta-mir-2296*	0	1	1	0	1	Inf	0.993540919	1
ENSBTAG00000044472	*5S_rRNA*	3	2	2	3	2	-0.170	1	1
ENSBTAG00000044478	*bta-mir-2442*	20	13	16	17	15	-0.206	0.970864367	1
ENSBTAG00000044481	*miRNA*	1	0	0	1	0		1	1
ENSBTAG00000044492	*protein_coding*	7	12	10	6	14	1.193	0.802511113	1
ENSBTAG00000044499	*bta-mir-2423*	0	1	1	0	1	Inf	0.993540919	1
ENSBTAG00000044500	*SNORD123*	25	7	15	22	8	-1.421	0.689157104	1
ENSBTAG00000044508	*U6*	1	1	1	1	1	0.415	1	1
ENSBTAG00000044509	*bta-mir-2317*	8	9	9	7	10	0.585	0.926558417	1
ENSBTAG00000044514	*SNORA70*	5	1	3	4	1	-1.907	0.9188325	1
ENSBTAG00000044515	*miRNA*	4	3	3	3	3	0.000	1	1
ENSBTAG00000044521	*ACA59*	1	3	2	1	3	2.000	0.933774563	1
ENSBTAG00000044524	*SNORD112*	2	0	1	2	0		0.974934741	1
ENSBTAG00000044526	*bta-mir-1584*	14	5	9	12	6	-1.070	0.844263455	1
ENSBTAG00000044536	*SNORA30*	5	6	6	4	7	0.678	0.946418607	1
ENSBTAG00000044537	*snoU109*	1	0	0	1	0		1	1
ENSBTAG00000044539	*U6*	3	0	1	3	0		0.936647693	1
ENSBTAG00000044542	*7SK*	0	1	1	0	1	Inf	0.993540919	1
ENSBTAG00000044545	*miRNA*	1	0	0	1	0		1	1
ENSBTAG00000044548	*bta-mir-2284b*	2	2	2	2	2	0.415	1	1
ENSBTAG00000044552	*7SK*	1	0	0	1	0		1	1
ENSBTAG00000044555	*7SK*	1	0	0	1	0		1	1
ENSBTAG00000044563	*U5*	0	1	1	0	1	Inf	0.993540919	1
ENSBTAG00000044566	*miRNA*	0	1	1	0	1	Inf	0.993540919	1
ENSBTAG00000044567	*SNORA18*	2	1	1	2	1	-0.585	1	1
ENSBTAG00000044568	*U6*	1	5	3	1	6	2.737	0.836148221	1
ENSBTAG00000044580	*bta-mir-2388*	1	0	0	1	0		1	1
ENSBTAG00000044581	*miRNA*	5	1	3	4	1	-1.907	0.9188325	1
ENSBTAG00000044591	*SNORA70*	6	1	3	5	1	-2.170	0.885240772	1
ENSBTAG00000044600	*RNaseP_nuc*	2	0	1	2	0		0.974934741	1
ENSBTAG00000044604	*snoU89*	4	0	2	3	0		0.89545886	1
ENSBTAG00000044606	*bta-mir-2382*	96	76	85	83	88	0.078	0.963357539	1
ENSBTAG00000044614	*SCARNA23*	10	19	15	9	22	1.341	0.695457515	1
ENSBTAG00000044615	*SNORA70*	1	0	0	1	0		1	1
ENSBTAG00000044616	*U6*	1	0	0	1	0		1	1
ENSBTAG00000044617	*5S_rRNA*	0	2	1	0	2	Inf	0.939077559	1
ENSBTAG00000044620	*bta-mir-2397*	0	1	1	0	1	Inf	0.993540919	1
ENSBTAG00000044621	*U6*	0	1	1	0	1	Inf	0.993540919	1
ENSBTAG00000044622	*bta-mir-2295*	1	0	0	1	0		1	1
ENSBTAG00000044625	*bta-mir-2401*	1	0	0	1	0		1	1
ENSBTAG00000044630	*bta-mir-2356*	4	4	4	3	5	0.415	0.994759363	1
ENSBTAG00000044631	*SCARNA6*	26	18	22	23	21	-0.115	0.986375687	1
ENSBTAG00000044633	*bta-mir-2469*	144	85	111	125	98	-0.345	0.78926373	1

Ensembl gene ID	geneName	counts wildtype horn bud	counts polled horn bud	baseMean	baseMean wildtype horn bud	baseMean polled horn bud	log2FoldChange	pval	padj
ENSBTAG00000044637	*U6*	1	0	0	1	0		1	1
ENSBTAG00000044643	*bta-mir-2346*	1	19	11	1	22	4.663	0.335365735	1
ENSBTAG00000044645	*SNORD113*	1	1	1	1	1	0.415	1	1
ENSBTAG00000044649	*bta-mir-2451*	10	11	11	9	13	0.553	0.915317076	1
ENSBTAG00000044659	*SCARNA13*	151	52	95	131	60	-1.123	0.402522928	1
ENSBTAG00000044674	*miRNA*	0	1	1	0	1	Inf	0.993540919	1
ENSBTAG00000044680	*bta-mir-2428*	0	1	1	0	1	Inf	0.993540919	1
ENSBTAG00000044681	*U6*	1	4	3	1	5	2.415	0.884888347	1
ENSBTAG00000044687	*U2*	1	0	0	1	0		1	1
ENSBTAG00000044690	*snoU109*	8	1	4	7	1	-2.585	0.818599781	1
ENSBTAG00000044699	*U6*	2	0	1	2	0		0.974934741	1
ENSBTAG00000044702	*bta-mir-2396*	12	5	8	10	6	-0.848	0.892845055	1
ENSBTAG00000044712	*bta-mir-2383*	48	43	46	42	50	0.256	0.897244349	1
ENSBTAG00000044714	*miRNA*	1	1	1	1	1	0.415	1	1
ENSBTAG00000044716	*U6*	0	1	1	0	1	Inf	0.993540919	1
ENSBTAG00000044719	*SNORA79*	13	3	7	11	3	-1.700	0.783725709	1
ENSBTAG00000044720	*miRNA*	1	0	0	1	0		1	1
ENSBTAG00000044722	*miRNA*	0	1	1	0	1	Inf	0.993540919	1
ENSBTAG00000044726	*7SK*	9	6	7	8	7	-0.170	1	1
ENSBTAG00000044741	*SNORD97*	78	46	60	68	53	-0.347	0.836798561	1
ENSBTAG00000044744	*SNORA79*	3	0	1	3	0		0.936647693	1
ENSBTAG00000044748	*SNORA81*	6	13	10	5	15	1.531	0.740992914	1
ENSBTAG00000044752	*U5*	0	1	1	0	1	Inf	0.993540919	1
ENSBTAG00000044753	*U1*	0	1	1	0	1	Inf	0.993540919	1
ENSBTAG00000044756	*5S_rRNA*	5	1	3	4	1	-1.907	0.9188325	1
ENSBTAG00000044758	*bta-mir-2362*	16	3	9	14	3	-2.000	0.709202358	1
ENSBTAG00000044760	*SNORD112*	12	7	9	10	8	-0.363	0.964206651	1
ENSBTAG00000044764	*SNORA25*	0	1	1	0	1	Inf	0.993540919	1
ENSBTAG00000044766	*U6*	2	14	9	2	16	3.222	0.540921467	1
ENSBTAG00000044771	*5S_rRNA*	1	0	0	1	0		1	1
ENSBTAG00000044774	*U6*	3	1	2	3	1	-1.170	0.985253077	1
ENSBTAG00000044775	*bta-mir-2387*	1	1	1	1	1	0.415	1	1
ENSBTAG00000044776	*snoU109*	0	1	1	0	1	Inf	0.993540919	1
ENSBTAG00000044778	*U6*	17	6	11	15	7	-1.087	0.813534849	1
ENSBTAG00000044779	*5S_rRNA*	10	4	7	9	5	-0.907	0.905171387	1
ENSBTAG00000044786	*U6*	1	0	0	1	0		1	1
ENSBTAG00000044787	*miRNA*	1	2	2	1	2	1.415	0.981979269	1
ENSBTAG00000044788	*SNORA81*	13	25	20	11	29	1.358	0.635396707	1
ENSBTAG00000044794	*U6atac*	1	1	1	1	1	0.415	1	1
ENSBTAG00000044796	*SNORA70*	52	36	43	45	42	-0.115	0.964921862	1
ENSBTAG00000044800	*bta-mir-2450c*	2	2	2	2	2	0.415	1	1
ENSBTAG00000044802	*SCARNA11*	3	4	4	3	5	0.830	0.962490925	1
ENSBTAG00000044807	*snoU13*	0	1	1	0	1	Inf	0.993540919	1
ENSBTAG00000044812	*snoU82P*	37	95	71	32	110	1.775	0.241251169	1
ENSBTAG00000044817	*bta-mir-2415*	4	0	2	3	0		0.89545886	1
ENSBTAG00000044830	*bta-mir-339a*	4	3	3	3	3	0.000	1	1
ENSBTAG00000044833	*bta-mir-2392*	15	15	15	13	17	0.415	0.918564425	1
ENSBTAG00000044837	*SCARNA21*	24	10	16	21	12	-0.848	0.806552039	1
ENSBTAG00000044844	*U6*	2	0	1	2	0		0.974934741	1
ENSBTAG00000044845	*miRNA*	2	0	1	2	0		0.974934741	1
ENSBTAG00000044848	*SNORA18*	1	0	0	1	0		1	1
ENSBTAG00000044853	*snoU85*	51	13	30	44	15	-1.557	0.499888893	1
ENSBTAG00000044854	*U6*	1	0	0	1	0		1	1
ENSBTAG00000044855	*SNORA9*	1	0	0	1	0		1	1
ENSBTAG00000044856	*SNORD10*	61	42	51	53	48	-0.123	0.956129754	1
ENSBTAG00000044859	*bta-mir-2408*	4	8	6	3	9	1.415	0.839396723	1
ENSBTAG00000044861	*SCARNA6*	11	4	7	10	5	-1.044	0.879025484	1
ENSBTAG00000044862	*bta-mir-2338*	8	18	14	7	21	1.585	0.664662352	1
ENSBTAG00000044864	*ACA64*	1	1	1	1	1	0.415	1	1
ENSBTAG00000044868	*bta-mir-1843*	14	17	16	12	20	0.695	0.84345254	1
ENSBTAG00000044872	*7SK*	0	1	1	0	1	Inf	0.993540919	1
ENSBTAG00000044875	*U6*	3	0	1	3	0		0.936647693	1
ENSBTAG00000044880	*bta-mir-2385*	68	32	48	59	37	-0.672	0.710947762	1
ENSBTAG00000044884	*bta-mir-2343*	3	0	1	3	0		0.936647693	1
ENSBTAG00000044887	*SCARNA18*	0	1	1	0	1	Inf	0.993540919	1
ENSBTAG00000044888	*bta-mir-2403*	14	30	23	12	35	1.515	0.561526757	1
ENSBTAG00000044890	*5S_rRNA*	3	0	1	3	0		0.936647693	1
ENSBTAG00000044891	*snoU109*	3	1	2	3	1	-1.170	0.985253077	1
ENSBTAG00000044897	*bta-mir-2438*	1	2	2	1	2	1.415	0.981979269	1
ENSBTAG00000044898	*SNORA74*	4	0	2	3	0		0.89545886	1
ENSBTAG00000044899	*bta-mir-2460*	1	0	0	1	0		1	1
ENSBTAG00000044900	*bta-mir-2364*	0	1	1	0	1	Inf	0.993540919	1

Ensembl gene ID	geneName	counts wildtype horn bud	counts polled horn bud	baseMean	baseMean wildtype horn bud	baseMean polled horn bud	log2FoldChange	pval	padj
ENSBTAG00000044905	*bta-mir-449d*	0	1	1	0	1	Inf	0.993540919	1
ENSBTAG00000044906	*snoU2_19*	1	0	0	1	0		1	1
ENSBTAG00000044908	*7SK*	1	0	0	1	0		1	1
ENSBTAG00000044911	*U6*	6	1	3	5	1	-2.170	0.885240772	1
ENSBTAG00000044913	*SNORD125*	0	2	1	0	2	Inf	0.939077559	1
ENSBTAG00000044918	*U6*	0	1	1	0	1	Inf	0.993540919	1
ENSBTAG00000044920	*miRNA*	0	1	1	0	1	Inf	0.993540919	1
ENSBTAG00000044921	*bta-mir-2292*	1	1	1	1	1	0.415	1	1
ENSBTAG00000044922	*U6*	0	1	1	0	1	Inf	0.993540919	1
ENSBTAG00000044924	*snoU105B*	1	1	1	1	1	0.415	1	1
ENSBTAG00000044928	*bta-mir-2373*	1	2	2	1	2	1.415	0.981979269	1
ENSBTAG00000044942	*bta-mir-2454*	16	11	13	14	13	-0.126	0.9977793	1
ENSBTAG00000044944	*U6*	2	1	1	2	1	-0.585	1	1
ENSBTAG00000044951	*bta-mir-2477*	29	10	18	25	12	-1.121	0.717983518	1
ENSBTAG00000044952	*U6*	3	0	1	3	0		0.936647693	1
ENSBTAG00000044972	*SNORD19B*	22	11	16	19	13	-0.585	0.874518804	1
ENSBTAG00000044977	*SNORD113*	2	0	1	2	0		0.974934741	1
ENSBTAG00000044983	*miRNA*	1	0	0	1	0		1	1
ENSBTAG00000044987	*U6*	2	1	1	2	1	-0.585	1	1
ENSBTAG00000044998	*miRNA*	2	1	1	2	1	-0.585	1	1
ENSBTAG00000044999	*ACA59*	1	1	1	1	1	0.415	1	1
ENSBTAG00000045008	*U6*	1	0	0	1	0		1	1
ENSBTAG00000045010	*bta-mir-2350*	12	11	12	10	13	0.290	0.964579659	1
ENSBTAG00000045016	*miRNA*	0	1	1	0	1	Inf	0.993540919	1
ENSBTAG00000045022	*SNORA21*	17	4	10	15	5	-1.672	0.733083131	1
ENSBTAG00000045023	*bta-mir-2425*	0	2	1	0	2	Inf	0.939077559	1
ENSBTAG00000045024	*U6*	6	0	3	5	0		0.81115604	1
ENSBTAG00000045026	*SNORA70*	31	15	22	27	17	-0.632	0.825445441	1
ENSBTAG00000045033	*bta-mir-2485*	2	2	2	2	2	0.415	1	1
ENSBTAG00000045039	*SCARNA17*	24	25	25	21	29	0.474	0.860158361	1
ENSBTAG00000045040	*bta-mir-2410*	1	3	2	1	3	2.000	0.933774563	1
ENSBTAG00000045044	*bta-mir-2380*	1	0	0	1	0		1	1
ENSBTAG00000045047	*7SK*	1	0	0	1	0		1	1
ENSBTAG00000045062	*SNORD11B*	1	2	2	1	2	1.415	0.981979269	1
ENSBTAG00000045067	*SNORD124*	39	56	49	34	65	0.937	0.594321393	1
ENSBTAG00000045070	*U6atac*	2	0	1	2	0		0.974934741	1
ENSBTAG00000045071	*SNORA64*	1	0	0	1	0		1	1
ENSBTAG00000045074	*bta-mir-2355*	1	0	0	1	0		1	1
ENSBTAG00000045080	*SNORD5*	91	36	60	79	42	-0.923	0.568148886	1
ENSBTAG00000045086	*7SK*	52	19	33	45	22	-1.037	0.630732115	1
ENSBTAG00000045087	*miRNA*	3	0	1	3	0		0.936647693	1
ENSBTAG00000045094	*bta-mir-2323*	12	10	11	10	12	0.152	0.995696634	1
ENSBTAG00000045097	*snoU54*	55	54	55	48	62	0.389	0.821722806	1
ENSBTAG00000045099	*SNORA61*	2	1	1	2	1	-0.585	1	1
ENSBTAG00000045102	*snoU89*	54	60	58	47	69	0.567	0.730957534	1
ENSBTAG00000045103	*bta-mir-2917*	2	1	1	2	1	-0.585	1	1
ENSBTAG00000045114	*5S_rRNA*	5	6	6	4	7	0.678	0.946418607	1
ENSBTAG00000045116	*U2*	1	2	2	1	2	1.415	0.981979269	1
ENSBTAG00000045117	*bta-mir-2287*	0	1	1	0	1	Inf	0.993540919	1
ENSBTAG00000045118	*miRNA*	1	0	0	1	0		1	1
ENSBTAG00000045119	*miRNA*	1	0	0	1	0		1	1
ENSBTAG00000045124	*miRNA*	1	0	0	1	0		1	1
ENSBTAG00000045134	*SNORA47*	1	0	0	1	0		1	1
ENSBTAG00000045139	*5S_rRNA*	15	5	9	13	6	-1.170	0.820729911	1
ENSBTAG00000045142	*U6*	4	4	4	3	5	0.415	0.994759363	1
ENSBTAG00000045147	*bta-mir-2325c*	1	0	0	1	0		1	1
ENSBTAG00000045149	*bta-mir-2333*	1	0	0	1	0		1	1
ENSBTAG00000045154	*U6*	0	1	1	0	1	Inf	0.993540919	1
ENSBTAG00000045156	*SNORA48*	10	3	6	9	3	-1.322	0.863266279	1
ENSBTAG00000045159	*SNORD112*	6	3	4	5	3	-0.585	0.976586913	1
ENSBTAG00000045163	*U4*	0	2	1	0	2	Inf	0.939077559	1
ENSBTAG00000045166	*SNORD121A*	1	2	2	1	2	1.415	0.981979269	1
ENSBTAG00000045167	*SNORD112*	1	0	0	1	0		1	1
ENSBTAG00000045168	*U5*	1	0	0	1	0		1	1
ENSBTAG00000045172	*bta-mir-2358*	414	499	467	359	576	0.684	0.445513572	1
ENSBTAG00000045181	*bta-mir-2379*	1	1	1	1	1	0.415	1	1
ENSBTAG00000045183	*miRNA*	2	1	1	2	1	-0.585	1	1
ENSBTAG00000045184	*SNORD127*	0	3	2	0	3	Inf	0.878646179	1
ENSBTAG00000045185	*7SK*	2	0	1	2	0		0.974934741	1
ENSBTAG00000045197	*SNORD125*	20	18	19	17	21	0.263	0.945084087	1
ENSBTAG00000045203	*SNORA84*	3	1	2	3	1	-1.170	0.985253077	1
ENSBTAG00000045206	*U6*	1	0	0	1	0		1	1

Ensembl gene ID	geneName	counts wildtype horn bud	counts polled horn bud	baseMean	baseMean wildtype horn bud	baseMean polled horn bud	log2FoldChange	pval	padj
ENSBTAG00000045209	*SCARNA15*	4	1	2	3	1	-1.585	0.952247636	1
ENSBTAG00000045218	*bta-mir-2431*	19	9	13	16	10	-0.663	0.873235283	1
ENSBTAG00000045220	*bta-mir-2305*	1	0	0	1	0		1	1
ENSBTAG00000045223	*bta-mir-2419*	1	2	2	1	2	1.415	0.981979269	1
ENSBTAG00000045228	*miRNA*	2	3	3	2	3	1.000	0.97171833	1
ENSBTAG00000045230	*miRNA*	0	5	3	0	6	Inf	0.757530434	1
ENSBTAG00000045234	*U6*	10	5	7	9	6	-0.585	0.94357809	1
ENSBTAG00000045235	*SCARNA18*	18	16	17	16	18	0.245	0.955905935	1
ENSBTAG00000045238	*bta-mir-2462*	1	0	0	1	0		1	1
ENSBTAG00000045243	*snoU109*	3	1	2	3	1	-1.170	0.985253077	1
ENSBTAG00000045248	*bta-mir-2429*	2	1	1	2	1	-0.585	1	1
ENSBTAG00000045249	*SNORA48*	0	3	2	0	3	Inf	0.878646179	1
ENSBTAG00000045252	*miRNA*	0	1	1	0	1	Inf	0.993540919	1
ENSBTAG00000045263	*bta-mir-2284t*	0	1	1	0	1	Inf	0.993540919	1
ENSBTAG00000045265	*bta-mir-2284g*	5	0	2	4	0		0.853268594	1
ENSBTAG00000045275	*SCARNA7*	39	12	24	34	14	-1.285	0.623151531	1
ENSBTAG00000045281	*bta-mir-2400*	8	3	5	7	3	-1.000	0.918861955	1
ENSBTAG00000045290	*bta-mir-2447*	12	6	9	10	7	-0.585	0.929512138	1
ENSBTAG00000045291	*bta-mir-2285e-2*	10	7	8	9	8	-0.100	1	1
ENSBTAG00000045293	*SNORA48*	30	15	22	26	17	-0.585	0.841963028	1
ENSBTAG00000045303	*bta-mir-2417*	1	0	0	1	0		1	1
ENSBTAG00000045306	*SNORD63*	22	8	14	19	9	-1.044	0.780532613	1
ENSBTAG00000045307	*bta-mir-2330*	1	0	0	1	0		1	1
ENSBTAG00000045308	*SNORD116*	15	2	8	13	2	-2.492	0.678711229	1
ENSBTAG00000045309	*bta-mir-2443*	41	23	31	36	27	-0.419	0.861801859	1
ENSBTAG00000045310	*SNORA31*	0	1	1	0	1	Inf	0.993540919	1
ENSBTAG00000045316	*SNORD11*	4	0	2	3	0		0.89545886	1
ENSBTAG00000045323	*SNORD112*	1	0	0	1	0		1	1
ENSBTAG00000045326	*SNORD113*	9	8	9	8	9	0.245	0.986825286	1
ENSBTAG00000045327	*ACA64*	47	23	34	41	27	-0.616	0.780031245	1
ENSBTAG00000045330	*bta-mir-2284u*	10	14	12	9	16	0.900	0.826937278	1
ENSBTAG00000045332	*bta-mir-2449*	3	4	4	3	5	0.830	0.962490925	1
ENSBTAG00000045337	*bta-mir-2376*	2	0	1	2	0		0.974934741	1
ENSBTAG00000045341	*U6*	3	5	4	3	6	1.152	0.920866718	1
ENSBTAG00000045350	*5S_rRNA*	1	0	0	1	0		1	1
ENSBTAG00000045354	*ACA64*	1	0	0	1	0		1	1
ENSBTAG00000045355	*5S_rRNA*	1	0	0	1	0		1	1
ENSBTAG00000045370	*U1*	2	0	1	2	0		0.974934741	1
ENSBTAG00000045371	*5S_rRNA*	0	1	1	0	1	Inf	0.993540919	1
ENSBTAG00000045372	*SNORD23*	1	2	2	1	2	1.415	0.981979269	1
ENSBTAG00000045373	*bta-mir-2320*	1	0	0	1	0		1	1
ENSBTAG00000045374	*7SK*	1	0	0	1	0		1	1
ENSBTAG00000045376	*SNORD116*	4	1	2	3	1	-1.585	0.952247636	1
ENSBTAG00000045379	*U1*	2	1	1	2	1	-0.585	1	1
ENSBTAG00000045380	*bta-mir-2473*	1	0	0	1	0		1	1
ENSBTAG00000045385	*SCARNA11*	0	1	1	0	1	Inf	0.993540919	1
ENSBTAG00000045392	*miRNA*	0	1	1	0	1	Inf	0.993540919	1
ENSBTAG00000045397	*SNORA40*	0	1	1	0	1	Inf	0.993540919	1
ENSBTAG00000045400	*miRNA*	0	1	1	0	1	Inf	0.993540919	1
ENSBTAG00000045402	*SNORA31*	1	0	0	1	0		1	1
ENSBTAG00000045404	*SNORA79*	5	2	3	4	2	-0.907	0.965043111	1
ENSBTAG00000045409	*bta-mir-2487*	0	1	1	0	1	Inf	0.993540919	1
ENSBTAG00000045414	*U5*	1	0	0	1	0		1	1
ENSBTAG00000045417	*U6*	5	4	4	4	5	0.093	1	1
ENSBTAG00000045418	*bta-mir-2461*	0	3	2	0	3	Inf	0.878646179	1
ENSBTAG00000045422	*SNORA79*	7	1	4	6	1	-2.392	0.851750348	1
ENSBTAG00000045425	*snoU109*	0	1	1	0	1	Inf	0.993540919	1
ENSBTAG00000045426	*SNORD116*	2	0	1	2	0		0.974934741	1
ENSBTAG00000045427	*7SK*	0	1	1	0	1	Inf	0.993540919	1
ENSBTAG00000045429	*bta-mir-2481*	0	1	1	0	1	Inf	0.993540919	1
ENSBTAG00000045434	*U6*	1	2	2	1	2	1.415	0.981979269	1
ENSBTAG00000045445	*U6atac*	1	0	0	1	0		1	1
ENSBTAG00000045449	*bta-mir-2349*	2	6	4	2	7	2.000	0.840116992	1
ENSBTAG00000045458	*bta-mir-2458*	1	0	0	1	0		1	1
ENSBTAG00000045459	*SNORD109A*	1	1	1	1	1	0.415	1	1
ENSBTAG00000045464	*U6*	4	0	2	3	0		0.89545886	1
ENSBTAG00000045469	*bta-mir-2439*	1	0	0	1	0		1	1
ENSBTAG00000045475	*U6*	0	1	1	0	1	Inf	0.993540919	1
ENSBTAG00000045476	*miRNA*	0	5	3	0	6	Inf	0.757530434	1
ENSBTAG00000045478	*bta-mir-2308*	16	22	20	14	25	0.874	0.767703817	1
ENSBTAG00000045482	*U6*	5	2	3	4	2	-0.907	0.965043111	1
ENSBTAG00000045484	*5S_rRNA*	2	1	1	2	1	-0.585	1	1

Ensembl gene ID	geneName	counts wildtype horn bud	counts polled horn bud	baseMean	baseMean wildtype horn bud	baseMean polled horn bud	log2FoldChange	pval	padj
ENSBTAG00000045486	*U2*	1	0	0	1	0		1	1
ENSBTAG00000045487	*protein_coding*	3	5	4	3	6	1.152	0.920866718	1
ENSBTAG00000045492	*ANG*	1'916	1'416	1'647	1'659	1'635	-0.021	0.980339306	1
ENSBTAG00000045493	*protein_coding*	262	595	457	227	687	1.598	0.080849203	1
ENSBTAG00000045496	*miRNA*	1	0	0	1	0		1	1
ENSBTAG00000045497	*protein_coding*	526	1'022	818	456	1'180	1.373	0.106482392	1
ENSBTAG00000045498	*SULT2B1*	174	238	213	151	275	0.867	0.406553628	1
ENSBTAG00000045499	*protein_coding*	13	0	6	11	0		0.551229012	1
ENSBTAG00000045500	*SGSM2*	2'344	2'478	2'446	2'030	2'861	0.495	0.528614144	1
ENSBTAG00000045504	*RPS4XP21*	21'726	48'470	37'392	18'815	55'968	1.573	0.042652978	1
ENSBTAG00000045505	*HDGF*	2	1	1	2	1	-0.585	1	1
ENSBTAG00000045509	*C20ORF177*	106	71	87	92	82	-0.163	0.914583634	1
ENSBTAG00000045510	*EMC10*	8'314	8'315	8'401	7'200	9'601	0.415	0.58761618	1
ENSBTAG00000045513	*protein_coding*	374	262	313	324	303	-0.098	0.922199361	1
ENSBTAG00000045515	*protein_coding*	6	1	3	5	1	-2.170	0.885240772	1
ENSBTAG00000045516	*miRNA*	2	0	1	2	0		0.974934741	1
ENSBTAG00000045517	*pseudogene*	14	103	66	12	119	3.294	0.047519799	1
ENSBTAG00000045519	*miRNA*	0	3	2	0	3	Inf	0.878646179	1
ENSBTAG00000045520	*BT.87983*	93	62	76	81	72	-0.170	0.916325377	1
ENSBTAG00000045523	*processed_pseudogene*	1'888	2'157	2'063	1'635	2'491	0.607	0.443237604	1
ENSBTAG00000045526	*BT.34523*	89	7	43	77	8	-3.253	0.110394788	1
ENSBTAG00000045529	*NCR1*	4	0	2	3	0		0.89545886	1
ENSBTAG00000045530	*SCARNA2*	28	13	20	24	15	-0.692	0.822223488	1
ENSBTAG00000045531	*protein_coding*	167	379	291	145	438	1.597	0.106142729	1
ENSBTAG00000045535	*BT.50843*	2	3	3	2	3	1.000	0.97171833	1
ENSBTAG00000045536	*miRNA*	1	0	0	1	0		1	1
ENSBTAG00000045538	*protein_coding*	651	499	570	564	576	0.031	0.972769598	1
ENSBTAG00000045539	*PCDHA5*	47	15	29	41	17	-1.233	0.596986546	1
ENSBTAG00000045543	*processed_pseudogene*	35	46	42	30	53	0.809	0.672422683	1
ENSBTAG00000045547	*AMIGO3*	1'043	1'428	1'276	903	1'649	0.868	0.286306193	1
ENSBTAG00000045548	*CRKL*	1'053	2'070	1'651	912	2'390	1.390	0.086576528	1
ENSBTAG00000045550	*TSPAN6*	5'641	3'604	4'523	4'885	4'162	-0.231	0.765518991	1
ENSBTAG00000045551	*protein_coding*	28	51	42	24	59	1.280	0.502128104	1
ENSBTAG00000045552	*protein_coding*	9	3	6	8	3	-1.170	0.890813994	1
ENSBTAG00000045555	*protein_coding*	76	39	55	66	45	-0.547	0.748130048	1
ENSBTAG00000045563	*pseudogene*	0	6	3	0	7	Inf	0.700309343	1
ENSBTAG00000045565	*NHSL2*	115	98	106	100	113	0.184	0.891086787	1
ENSBTAG00000045566	*protein_coding*	1'932	1'960	1'968	1'673	2'263	0.436	0.582228811	1
ENSBTAG00000045567	*PRR15*	43	33	38	37	38	0.033	1	1
ENSBTAG00000045568	*pseudogene*	38'080	54'301	47'840	32'978	62'701	0.927	0.225227586	1
ENSBTAG00000045571	*protein_coding*	7	62	39	6	72	3.562	0.095911104	1
ENSBTAG00000045572	*ZNF319*	148	232	198	128	268	1.064	0.318282851	1
ENSBTAG00000045573	*miRNA*	261	168	210	226	194	-0.221	0.837222419	1
ENSBTAG00000045575	*processed_pseudogene*	125	281	216	108	324	1.584	0.133042775	1
ENSBTAG00000045577	*MCCD1*	26	32	30	23	37	0.715	0.757535358	1
ENSBTAG00000045578	*AGR3*	0	2	1	0	2	Inf	0.939077559	1
ENSBTAG00000045579	*TMEM60*	89	98	95	77	113	0.554	0.679444446	1
ENSBTAG00000045580	*protein_coding*	0	1	1	0	1	Inf	0.993540919	1
ENSBTAG00000045581	*protein_coding*	221	101	154	191	117	-0.715	0.532021336	1
ENSBTAG00000045582	*ARPC5*	9'324	7'130	8'154	8'075	8'233	0.028	0.97033918	1
ENSBTAG00000045583	*protein_coding*	11	7	9	10	8	-0.237	0.988863286	1
ENSBTAG00000045584	*CAV2*	907	616	748	785	711	-0.143	0.867667274	1
ENSBTAG00000045588	*protein_coding*	579	181	355	501	209	-1.263	0.182483787	1
ENSBTAG00000045589	*protein_coding*	1	0	0	1	0		1	1
ENSBTAG00000045590	*protein_coding*	2	0	1	2	0		0.974934741	1
ENSBTAG00000045592	*BT.67234*	41	57	51	36	66	0.890	0.60816053	1
ENSBTAG00000045593	*EARS2*	36	37	37	31	43	0.455	0.829323627	1
ENSBTAG00000045594	*TMEM121*	208	346	290	180	400	1.149	0.240663476	1
ENSBTAG00000045596	*YY1AP1*	309	297	305	268	343	0.358	0.710974716	1
ENSBTAG00000045598	*ATP4A*	0	1	1	0	1	Inf	0.993540919	1
ENSBTAG00000045601	*KCTD21*	170	222	202	147	256	0.800	0.449421746	1
ENSBTAG00000045603	*PCDHB4*	111	144	131	96	166	0.791	0.508366005	1
ENSBTAG00000045604	*TTC9*	572	223	376	495	257	-0.944	0.311510252	1
ENSBTAG00000045608	*EGLN1*	415	219	306	359	253	-0.507	0.600131699	1
ENSBTAG00000045609	*protein_coding*	370	251	305	320	290	-0.145	0.884234276	1
ENSBTAG00000045610	*CACNA1I*	3	3	3	3	3	0.415	1	1
ENSBTAG00000045611	*bta-mir-411b*	0	1	1	0	1	Inf	0.993540919	1
ENSBTAG00000045617	*MTERF*	573	353	452	496	408	-0.284	0.754690785	1
ENSBTAG00000045619	*RPL39*	5'487	6'419	6'082	4'752	7'412	0.641	0.405349042	1
ENSBTAG00000045621	*C17ORF50*	1	2	2	1	2	1.415	0.981979269	1
ENSBTAG00000045622	*protein_coding*	2'041	2'172	2'138	1'768	2'508	0.505	0.522819444	1
ENSBTAG00000045625	*NTN3*	497	929	752	430	1'073	1.317	0.123980667	1

Ensembl gene ID	geneName	counts wildtype horn bud	counts polled horn bud	baseMean	baseMean wildtype horn bud	baseMean polled horn bud	log2FoldChange	pval	padj
ENSBTAG00000045626	*DEFB300*	369	62	196	320	72	-2.158	0.050524004	1
ENSBTAG00000045628	*C15ORF61*	105	34	65	91	39	-1.212	0.437994379	1
ENSBTAG00000045630	*protein_coding*	1	1	1	1	1	0.415	1	1
ENSBTAG00000045631	*protein_coding*	1	0	0	1	0		1	1
ENSBTAG00000045633	*AGTR1*	79	123	105	68	142	1.054	0.41326853	1
ENSBTAG00000045637	*PRR23A*	1	0	0	1	0		1	1
ENSBTAG00000045641	*SNORD42*	32	15	23	28	17	-0.678	0.809212694	1
ENSBTAG00000045643	*FOXD2*	61	132	103	53	152	1.529	0.243112024	1
ENSBTAG00000045645	*protein_coding*	0	2	1	0	2	Inf	0.939077559	1
ENSBTAG00000045646	*pseudogene*	1	1	1	1	1	0.415	1	1
ENSBTAG00000045647	*ARL4C*	287	237	261	249	274	0.139	0.891570384	1
ENSBTAG00000045648	*protein_coding*	4	5	5	3	6	0.737	0.954105567	1
ENSBTAG00000045649	*DEFB*	116	39	73	100	45	-1.158	0.437459989	1
ENSBTAG00000045650	*pseudogene*	2	0	1	2	0		0.974934741	1
ENSBTAG00000045652	*PYY2*	5	1	3	4	1	-1.907	0.9188325	1
ENSBTAG00000045655	*protein_coding*	1	1	1	1	1	0.415	1	1
ENSBTAG00000045658	*BT.88077*	987	985	996	855	1'137	0.412	0.617307279	1
ENSBTAG00000045661	*Metazoa_SRP*	764	169	428	662	195	-1.762	0.058233114	1
ENSBTAG00000045662	*KRT3*	2	13	8	2	15	3.115	0.57299014	1
ENSBTAG00000045664	*LRRC41*	4'289	4'016	4'176	3'714	4'637	0.320	0.67882631	1
ENSBTAG00000045665	*protein_coding*	207	333	282	179	385	1.101	0.263262899	1
ENSBTAG00000045668	*SNORA73*	45	16	29	39	18	-1.077	0.646840141	1
ENSBTAG00000045672	*protein_coding*	1	2	2	1	2	1.415	0.981979269	1
ENSBTAG00000045673	*protein_coding*	13	59	40	11	68	2.597	0.198456182	1
ENSBTAG00000045674	*protein_coding*	2	3	3	2	3	1.000	0.97171833	1
ENSBTAG00000045678	*protein_coding*	938	1'622	1'343	812	1'873	1.205	0.140128117	1
ENSBTAG00000045679	*protein_coding*	22	12	16	19	14	-0.459	0.903495485	1
ENSBTAG00000045680	*protein_coding*	1	0	0	1	0		1	1
ENSBTAG00000045682	*U6*	2	1	1	2	1	-0.585	1	1
ENSBTAG00000045683	*PCDHGA5*	73	190	141	63	219	1.795	0.131120223	1
ENSBTAG00000045685	*PER2*	129	118	124	112	136	0.286	0.817354636	1
ENSBTAG00000045686	*bta-mir-2450b*	1	2	2	1	2	1.415	0.981979269	1
ENSBTAG00000045687	*miRNA*	2	0	1	2	0		0.974934741	1
ENSBTAG00000045689	*processed_pseudogene*	2'231	3'618	3'055	1'932	4'178	1.113	0.157878614	1
ENSBTAG00000045690	*protein_coding*	2	1	1	2	1	-0.585	1	1
ENSBTAG00000045692	*BT.64739*	488	357	417	423	412	-0.036	0.97222734	1
ENSBTAG00000045694	*protein_coding*	41	48	45	36	55	0.642	0.727959403	1
ENSBTAG00000045695	*SLC6A19*	2	1	1	2	1	-0.585	1	1
ENSBTAG00000045697	*MTCP1*	264	92	167	229	106	-1.106	0.32292232	1
ENSBTAG00000045699	*BT.102825*	17	11	14	15	13	-0.213	0.976116423	1
ENSBTAG00000045701	*OPRL1*	60	5	29	52	6	-3.170	0.201573083	1
ENSBTAG00000045702	*ZBED6*	93	55	72	81	64	-0.343	0.824837836	1
ENSBTAG00000045703	*COX15*	553	476	514	479	550	0.199	0.822966759	1
ENSBTAG00000045704	*BT.90639*	129	23	69	112	27	-2.073	0.182418334	1
ENSBTAG00000045705	*pseudogene*	1	0	0	1	0		1	1
ENSBTAG00000045706	*protein_coding*	1	0	0	1	0		1	1
ENSBTAG00000045708	*MYPOP*	84	99	94	73	114	0.652	0.627953642	1
ENSBTAG00000045710	*RN28S1*	2'548	2'464	2'526	2'207	2'845	0.367	0.640081301	1
ENSBTAG00000045711	*C11H9orf169*	77	24	47	67	28	-1.267	0.483529855	1
ENSBTAG00000045713	*SNORA41*	0	3	2	0	3	Inf	0.878646179	1
ENSBTAG00000045715	*processed_pseudogene*	32	68	53	28	79	1.503	0.377719308	1
ENSBTAG00000045717	*ADO*	176	122	147	152	141	-0.114	0.928218017	1
ENSBTAG00000045718	*miRNA*	1	0	0	1	0		1	1
ENSBTAG00000045726	*protein_coding*	0	1	1	0	1	Inf	0.993540919	1
ENSBTAG00000045728	*BT.65021*	479	691	606	415	798	0.944	0.278069173	1
ENSBTAG00000045729	*C6H4orf52*	466	439	455	404	507	0.329	0.715026274	1
ENSBTAG00000045730	*miRNA*	0	1	1	0	1	Inf	0.993540919	1
ENSBTAG00000045732	*C17ORF105*	3	0	1	3	0		0.936647693	1
ENSBTAG00000045734	*LRRC26*	1	0	0	1	0		1	1
ENSBTAG00000045737	*BT.23579*	7	0	3	6	0		0.769774961	1
ENSBTAG00000045740	*pseudogene*	1	0	0	1	0		1	1
ENSBTAG00000045741	*U1*	6	1	3	5	1	-2.170	0.885240772	1
ENSBTAG00000045742	*C5H12orf75*	212	164	186	184	189	0.045	0.971649284	1
ENSBTAG00000045743	*PCDHB10*	302	170	229	262	196	-0.414	0.688777058	1
ENSBTAG00000045744	*NKRF*	502	279	378	435	322	-0.432	0.642387235	1
ENSBTAG00000045746	*BT.43562*	68	4	32	59	5	-3.672	0.130312442	1
ENSBTAG00000045747	*5_8S_rRNA*	529	276	388	458	319	-0.524	0.571813294	1
ENSBTAG00000045748	*RAF1*	2'696	2'424	2'567	2'335	2'799	0.262	0.738352098	1
ENSBTAG00000045751	*BT.63792*	5	2	3	4	2	-0.907	0.965043111	1
ENSBTAG00000045753	*protein_coding*	20	14	17	17	16	-0.100	0.998076173	1
ENSBTAG00000045754	*BT.29911*	557	546	556	482	630	0.386	0.659054996	1
ENSBTAG00000045756	*protein_coding*	216	352	297	187	406	1.120	0.250543845	1

Ensembl gene ID	geneName	counts wildtype horn bud	counts polled horn bud	baseMean	baseMean wildtype horn bud	baseMean polled horn bud	log2FoldChange	pval	padj
ENSBTAG00000045757	*TNNC1*	67	209	150	58	241	2.056	0.081060117	1
ENSBTAG00000045758	*protein_coding*	1	0	0	1	0		1	1
ENSBTAG00000045759	*pseudogene*	7	24	17	6	28	2.193	0.49644578	1
ENSBTAG00000045760	*protein_coding*	7	1	4	6	1	-2.392	0.851750348	1
ENSBTAG00000045762	*SCAMP4*	1'877	2'596	2'312	1'626	2'998	0.883	0.264643676	1
ENSBTAG00000045763	*pseudogene*	3	0	1	3	0		0.936647693	1
ENSBTAG00000045765	*processed_pseudogene*	99	197	157	86	227	1.408	0.217812606	1
ENSBTAG00000045767	*BT.18889*	328	161	235	284	186	-0.612	0.550062382	1
ENSBTAG00000045768	*protein_coding*	1	0	0	1	0		1	1
ENSBTAG00000045769	*protein_coding*	5	2	3	4	2	-0.907	0.965043111	1
ENSBTAG00000045770	*KRT77*	1'577	1'912	1'787	1'366	2'208	0.693	0.385032558	1
ENSBTAG00000045772	*protein_coding*	953	1'208	1'110	825	1'395	0.757	0.356208192	1
ENSBTAG00000045776	*C1ORF122*	880	712	792	762	822	0.109	0.897164905	1
ENSBTAG00000045779	*UBE2A*	771	661	715	668	763	0.193	0.820853028	1
ENSBTAG00000045781	*Metazoa_SRP*	2	7	5	2	8	2.222	0.797845137	1
ENSBTAG00000045783	*processed_pseudogene*	2'058	597	1'236	1'782	689	-1.370	0.096577362	1
ENSBTAG00000045784	*CENPB*	1'078	1'748	1'476	934	2'018	1.112	0.17014659	1
ENSBTAG00000045785	*TOM1*	1'907	2'299	2'153	1'652	2'655	0.685	0.386871412	1
ENSBTAG00000045786	*KLRC1*	0	2	1	0	2	Inf	0.939077559	1
ENSBTAG00000045787	*CDS1*	190	142	164	165	164	-0.005	1	1
ENSBTAG00000045788	*protein_coding*	7	8	8	6	9	0.608	0.932722577	1
ENSBTAG00000045789	*TNNT2*	139	74	103	120	85	-0.494	0.706918737	1
ENSBTAG00000045791	*ZNF623*	645	619	637	559	715	0.356	0.67962736	1
ENSBTAG00000045792	*SNORD39*	40	47	44	35	54	0.648	0.728718344	1
ENSBTAG00000045793	*miRNA*	5	1	3	4	1	-1.907	0.9188325	1
ENSBTAG00000045794	*BT.87362*	10'385	7'727	8'958	8'994	8'922	-0.011	0.988873387	1
ENSBTAG00000045795	*BT.36112*	7	1	4	6	1	-2.392	0.851750348	1
ENSBTAG00000045798	*protein_coding*	1	0	0	1	0		1	1
ENSBTAG00000045800	*protein_coding*	1	0	0	1	0		1	1
ENSBTAG00000045801	*5_8S_rRNA*	0	1	1	0	1	Inf	0.993540919	1
ENSBTAG00000045805	*BT.33157*	20	37	30	17	43	1.303	0.564512232	1
ENSBTAG00000045808	*IL17REL*	19	50	37	16	58	1.811	0.373626938	1
ENSBTAG00000045812	*GSX2*	19	2	9	16	2	-2.833	0.582174212	1
ENSBTAG00000045813	*protein_coding*	4	5	5	3	6	0.737	0.954105567	1
ENSBTAG00000045814	*processed_pseudogene*	13	83	54	11	96	3.090	0.085192323	1
ENSBTAG00000045817	*BT.20209*	2'103	3'489	2'925	1'821	4'029	1.145	0.146725278	1
ENSBTAG00000045818	*Metazoa_SRP*	205	74	131	178	85	-1.055	0.379874586	1
ENSBTAG00000045819	*pseudogene*	1	0	0	1	0		1	1
ENSBTAG00000045821	*protein_coding*	2	4	3	2	5	1.415	0.927250049	1
ENSBTAG00000045822	*pseudogene*	659	426	531	571	492	-0.214	0.809746399	1
ENSBTAG00000045823	*miRNA*	2	0	1	2	0		0.974934741	1
ENSBTAG00000045824	*SOX9*	178	188	186	154	217	0.494	0.648161456	1
ENSBTAG00000045826	*C10ORF105*	7	1	4	6	1	-2.392	0.851750348	1
ENSBTAG00000045828	*PTBP1*	11'845	14'960	13'766	10'258	17'274	0.752	0.326147807	1
ENSBTAG00000045829	*BT.96677*	40	45	43	35	52	0.585	0.758385712	1
ENSBTAG00000045830	*protein_coding*	0	1	1	0	1	Inf	0.993540919	1
ENSBTAG00000045832	*PHLPP1*	830	714	772	719	824	0.198	0.814905008	1
ENSBTAG00000045834	*RASSF7*	239	295	274	207	341	0.719	0.465841067	1
ENSBTAG00000045836	*miRNA*	2	0	1	2	0		0.974934741	1
ENSBTAG00000045847	*CTXN2*	6	0	3	5	0		0.81115604	1
ENSBTAG00000045849	*KCNK13*	4	2	3	3	2	-0.585	0.996239345	1
ENSBTAG00000045852	*TAL2*	13	4	8	11	5	-1.285	0.828302954	1
ENSBTAG00000045854	*BT.97257*	16	3	9	14	3	-2.000	0.709202358	1
ENSBTAG00000045857	*FAM195A*	245	185	213	212	214	0.010	0.997028602	1
ENSBTAG00000045859	*PCDHB13*	246	163	201	213	188	-0.179	0.870588102	1
ENSBTAG00000045862	*BT.106173*	147	95	119	127	110	-0.215	0.868358553	1
ENSBTAG00000045864	*ZNF581*	192	174	184	166	201	0.273	0.803349625	1
ENSBTAG00000045867	*RPGR*	242	208	225	210	240	0.197	0.850987001	1
ENSBTAG00000045868	*ZFP37*	472	237	341	409	274	-0.579	0.54104922	1
ENSBTAG00000045870	*bta-mir-2888-2*	1	0	0	1	0		1	1
ENSBTAG00000045871	*protein_coding*	1	3	2	1	3	2.000	0.933774563	1
ENSBTAG00000045873	*protein_coding*	13	1	6	11	1	-3.285	0.663144098	1
ENSBTAG00000045875	*miRNA*	4	1	2	3	1	-1.585	0.952247636	1
ENSBTAG00000045876	*BT.104395*	198	223	214	171	257	0.587	0.573364835	1
ENSBTAG00000045877	*TSC22D3*	587	144	337	508	166	-1.612	0.094361554	1
ENSBTAG00000045879	*FAM26E*	320	322	324	277	372	0.424	0.656456122	1
ENSBTAG00000045880	*protein_coding*	55	19	35	48	22	-1.118	0.596333837	1
ENSBTAG00000045881	*bta-mir-1260b*	13	4	8	11	5	-1.285	0.828302954	1
ENSBTAG00000045884	*BT.62029*	40	4	20	35	5	-2.907	0.343574329	1
ENSBTAG00000045885	*miRNA*	913	364	605	791	420	-0.912	0.295438714	1
ENSBTAG00000045886	*STARD4*	124	94	108	107	109	0.015	0.998059926	1
ENSBTAG00000045887	*EPB49*	297	74	171	257	85	-1.590	0.156737936	1

Ensembl gene ID	geneName	counts wildtype horn bud	counts polled horn bud	baseMean	baseMean wildtype horn bud	baseMean polled horn bud	log2FoldChange	pval	padj
ENSBTAG00000045888	*BT.37779*	322	174	240	279	201	-0.473	0.643018235	1
ENSBTAG00000045889	*protein_coding*	3	28	17	3	32	3.637	0.279687657	1
ENSBTAG00000045892	*protein_coding*	589	680	648	510	785	0.622	0.469390838	1
ENSBTAG00000045893	*BT.42384*	233	249	245	202	288	0.511	0.613113071	1
ENSBTAG00000045895	*NFASC*	370	225	290	320	260	-0.303	0.75857481	1
ENSBTAG00000045898	*pseudogene*	7	3	5	6	3	-0.807	0.947428618	1
ENSBTAG00000045899	*protein_coding*	1	0	0	1	0		1	1
ENSBTAG00000045901	*CLDN6*	9	39	26	8	45	2.531	0.310153229	1
ENSBTAG00000045902	*EPHB2*	2'882	4'288	3'724	2'496	4'951	0.988	0.206297567	1
ENSBTAG00000045904	*PIM3*	816	727	773	707	839	0.248	0.768538175	1
ENSBTAG00000045905	*PCDH15*	74	4	34	64	5	-3.794	0.105823801	1
ENSBTAG00000045907	*BT.24855*	1	0	0	1	0		1	1
ENSBTAG00000045908	*U6*	2	0	1	2	0		0.974934741	1
ENSBTAG00000045909	*FAM174A*	737	348	520	638	402	-0.668	0.451282335	1
ENSBTAG00000045910	*BT.60386*	98	109	105	85	126	0.569	0.659568848	1
ENSBTAG00000045912	*protein_coding*	1	0	0	1	0		1	1
ENSBTAG00000045913	*U6*	1	2	2	1	2	1.415	0.981979269	1
ENSBTAG00000045914	*H3F3C*	4'686	8'344	6'847	4'058	9'635	1.247	0.108762292	1
ENSBTAG00000045915	*SCT*	12	5	8	10	6	-0.848	0.892845055	1
ENSBTAG00000045916	*U2*	3	11	8	3	13	2.290	0.692284022	1
ENSBTAG00000045917	*protein_coding*	1	2	2	1	2	1.415	0.981979269	1
ENSBTAG00000045921	*protein_coding*	0	3	2	0	3	Inf	0.878646179	1
ENSBTAG00000045923	*BOLA-DIB*	16	1	8	14	1	-3.585	0.580698833	1
ENSBTAG00000045925	*CITED1*	171	150	161	148	173	0.226	0.843873903	1
ENSBTAG00000045926	*miRNA*	2	0	1	2	0		0.974934741	1
ENSBTAG00000045928	*protein_coding*	8	0	3	7	0		0.72953145	1
ENSBTAG00000045929	*BT.29090*	54	5	26	47	6	-3.018	0.244932489	1
ENSBTAG00000045931	*MAGT1*	1'416	1'127	1'264	1'226	1'301	0.086	0.916046166	1
ENSBTAG00000045932	*protein_coding*	0	3	2	0	3	Inf	0.878646179	1
ENSBTAG00000045934	*GPR82*	8	0	3	7	0		0.72953145	1
ENSBTAG00000045936	*protein_coding*	1	0	0	1	0		1	1
ENSBTAG00000045937	*processed_pseudogene*	3'229	5'253	4'431	2'796	6'066	1.117	0.152635433	1
ENSBTAG00000045939	*MPEG1*	1	1	1	1	1	0.415	1	1
ENSBTAG00000045940	*BT.54183*	12	0	5	10	0		0.583618942	1
ENSBTAG00000045941	*C7H5orf30*	771	289	501	668	334	-1.001	0.26288994	1
ENSBTAG00000045946	*VMO1*	6	5	5	5	6	0.152	1	1
ENSBTAG00000045947	*protein_coding*	56	64	61	48	74	0.608	0.705083022	1
ENSBTAG00000045948	*CSF2RA*	148	263	216	128	304	1.245	0.234374235	1
ENSBTAG00000045952	*5_8S_rRNA*	335	190	255	290	219	-0.403	0.68930539	1
ENSBTAG00000045953	*protein_coding*	1	0	0	1	0		1	1
ENSBTAG00000045954	*LRRC14B*	2	12	8	2	14	3.000	0.606634849	1
ENSBTAG00000045955	*BT.94964*	39	138	97	34	159	2.238	0.101572392	1
ENSBTAG00000045956	*SNORD53_SNORD92*	3	1	2	3	1	-1.170	0.985253077	1
ENSBTAG00000045957	*SAMM50*	2'607	3'072	2'902	2'258	3'547	0.652	0.405147857	1
ENSBTAG00000045963	*5S_rRNA*	0	1	1	0	1	Inf	0.993540919	1
ENSBTAG00000045965	*bta-mir-2904-3*	30	0	13	26	0		0.205783873	1
ENSBTAG00000045966	*GPRIN3*	22	11	16	19	13	-0.585	0.874518804	1
ENSBTAG00000045967	*LENEP*	23	13	17	20	15	-0.408	0.912458883	1
ENSBTAG00000045970	*protein_coding*	6	3	4	5	3	-0.585	0.976586913	1
ENSBTAG00000045971	*RTN3*	4'876	2'914	3'794	4'223	3'365	-0.328	0.673616666	1
ENSBTAG00000045973	*U2*	12	21	17	10	24	1.222	0.698825514	1
ENSBTAG00000045974	*pseudogene*	40	10	23	35	12	-1.585	0.551930545	1
ENSBTAG00000045976	*BT.57034*	1'016	295	610	880	341	-1.369	0.118850896	1
ENSBTAG00000045977	*Metazoa_SRP*	1	0	0	1	0		1	1
ENSBTAG00000045978	*protein_coding*	1	0	0	1	0		1	1
ENSBTAG00000045980	*ARF6*	1'669	1'448	1'559	1'445	1'672	0.210	0.792929858	1
ENSBTAG00000045981	*DIRAS1*	73	21	44	63	24	-1.382	0.461629122	1
ENSBTAG00000045984	*protein_coding*	1	2	2	1	2	1.415	0.981979269	1
ENSBTAG00000045985	*protein_coding*	109	25	62	94	29	-1.709	0.289496672	1
ENSBTAG00000045986	*MIA2*	1	2	2	1	2	1.415	0.981979269	1
ENSBTAG00000045987	*protein_coding*	113	176	151	98	203	1.054	0.358525174	1
ENSBTAG00000045989	*CDC42EP5*	1'001	915	962	867	1'057	0.285	0.73001468	1
ENSBTAG00000045991	*protein_coding*	0	8	5	0	9	Inf	0.59561973	1
ENSBTAG00000045993	*protein_coding*	0	4	2	0	5	Inf	0.817359038	1
ENSBTAG00000045994	*pseudogene*	42	37	40	36	43	0.232	0.916043754	1
ENSBTAG00000045996	*pseudogene*	0	1	1	0	1	Inf	0.993540919	1
ENSBTAG00000045997	*pseudogene*	5	10	8	4	12	1.415	0.803541841	1
ENSBTAG00000045998	*MAGEL2*	1'063	1'175	1'139	921	1'357	0.560	0.493607677	1
ENSBTAG00000046000	*processed_pseudogene*	115	145	134	100	167	0.749	0.52840848	1
ENSBTAG00000046001	*U6*	1	1	1	1	1	0.415	1	1
ENSBTAG00000046003	*BT.12047*	111	77	93	96	89	-0.113	0.942464941	1
ENSBTAG00000046004	*processed_pseudogene*	1'628	1'159	1'374	1'410	1'338	-0.075	0.927323838	1

Ensembl gene ID	geneName	counts wildtype horn bud	counts polled horn bud	baseMean	baseMean wildtype horn bud	baseMean polled horn bud	log2FoldChange	pval	padj
ENSBTAG00000046005	*RANBP1*	1'220	1'090	1'158	1'057	1'259	0.252	0.756850912	1
ENSBTAG00000046006	*CNR1*	33	3	16	29	3	-3.044	0.387507238	1
ENSBTAG00000046007	*CCDC27*	4	0	2	3	0		0.89545886	1
ENSBTAG00000046008	*protein_coding*	594	437	510	514	505	-0.028	0.978184357	1
ENSBTAG00000046010	*BHLHA15*	2	0	1	2	0		0.974934741	1
ENSBTAG00000046012	*ANKRD58*	36	15	24	31	17	-0.848	0.747030072	1
ENSBTAG00000046013	*BT.103350*	8	2	5	7	2	-1.585	0.87391615	1
ENSBTAG00000046014	*GPR146*	51	42	46	44	48	0.135	0.951775683	1
ENSBTAG00000046017	*POPDC3*	23	61	45	20	70	1.822	0.324267434	1
ENSBTAG00000046019	*BT.24449*	5'578	6'958	6'433	4'831	8'034	0.734	0.341208722	1
ENSBTAG00000046022	*PYY*	30	27	29	26	31	0.263	0.922347889	1
ENSBTAG00000046024	*protein_coding*	1'548	657	1'050	1'341	759	-0.821	0.320011263	1
ENSBTAG00000046025	*miRNA*	0	1	1	0	1	Inf	0.993540919	1
ENSBTAG00000046026	*SLC39A4*	8	7	8	7	8	0.222	0.99532173	1
ENSBTAG00000046029	*protein_coding*	54	36	44	47	42	-0.170	0.939935238	1
ENSBTAG00000046030	*protein_coding*	31	19	24	27	22	-0.291	0.924493582	1
ENSBTAG00000046031	*C14H8orf82*	384	418	408	333	483	0.537	0.557123415	1
ENSBTAG00000046033	*UBFD1*	1'409	1'475	1'462	1'220	1'703	0.481	0.549671576	1
ENSBTAG00000046034	*protein_coding*	19'030	18'539	18'944	16'480	21'407	0.377	0.619911669	1
ENSBTAG00000046035	*protein_coding*	1	1	1	1	1	0.415	1	1
ENSBTAG00000046036	*pseudogene*	8	2	5	7	2	-1.585	0.87391615	1
ENSBTAG00000046037	*BT.102599*	2'205	2'066	2'148	1'910	2'386	0.321	0.683903468	1
ENSBTAG00000046041	*BT.61919*	6	1	3	5	1	-2.170	0.885240772	1
ENSBTAG00000046044	*protein_coding*	5	6	6	4	7	0.678	0.946418607	1
ENSBTAG00000046046	*MGC160046*	0	23	13	0	27	Inf	0.169548618	1
ENSBTAG00000046047	*protein_coding*	30	20	25	26	23	-0.170	0.964462122	1
ENSBTAG00000046048	*protein_coding*	5	1	3	4	1	-1.907	0.9188325	1
ENSBTAG00000046049	*pseudogene*	84	34	56	73	39	-0.890	0.594419392	1
ENSBTAG00000046050	*SNORA73*	115	19	61	100	22	-2.183	0.18533089	1
ENSBTAG00000046051	*protein_coding*	64	30	45	55	35	-0.678	0.71714262	1
ENSBTAG00000046052	*ANKRD63*	2	1	1	2	1	-0.585	1	1
ENSBTAG00000046053	*SNORD116*	0	1	1	0	1	Inf	0.993540919	1
ENSBTAG00000046054	*RHOB*	6'954	4'413	5'559	6'022	5'096	-0.241	0.755033191	1
ENSBTAG00000046057	*PPP1R3G*	5	1	3	4	1	-1.907	0.9188325	1
ENSBTAG00000046058	*RNASE11*	1	1	1	1	1	0.415	1	1
ENSBTAG00000046060	*protein_coding*	14	5	9	12	6	-1.070	0.844263455	1
ENSBTAG00000046062	*protein_coding*	33	91	67	29	105	1.878	0.227451544	1
ENSBTAG00000046063	*SNORA3*	10	39	27	9	45	2.379	0.333035083	1
ENSBTAG00000046064	*miRNA*	2	0	1	2	0		0.974934741	1
ENSBTAG00000046066	*pseudogene*	21	64	46	18	74	2.023	0.272263475	1
ENSBTAG00000046067	*bta-mir-2893*	4	0	2	3	0		0.89545886	1
ENSBTAG00000046068	*pseudogene*	2	0	1	2	0		0.974934741	1
ENSBTAG00000046069	*SPACA5*	2	1	1	2	1	-0.585	1	1
ENSBTAG00000046070	*pseudogene*	2	0	1	2	0		0.974934741	1
ENSBTAG00000046073	*C16H1orf70*	193	167	180	167	193	0.206	0.852822328	1
ENSBTAG00000046076	*protein_coding*	3	0	1	3	0		0.936647693	1
ENSBTAG00000046079	*processed_pseudogene*	673	859	787	583	992	0.767	0.363566729	1
ENSBTAG00000046080	*GNG5P2*	917	1'354	1'179	794	1'563	0.977	0.233332191	1
ENSBTAG00000046081	*U1*	2	2	2	2	2	0.415	1	1
ENSBTAG00000046088	*pseudogene*	287	499	412	249	576	1.213	0.187944571	1
ENSBTAG00000046089	*KRT1*	86'145	45'541	63'595	74'604	52'586	-0.505	0.507312419	1
ENSBTAG00000046090	*protein_coding*	1	0	0	1	0		1	1
ENSBTAG00000046092	*BT.90254*	670	514	587	580	594	0.033	0.971479862	1
ENSBTAG00000046095	*protein_coding*	2'093	487	1'187	1'813	562	-1.689	0.042813021	1
ENSBTAG00000046096	*protein_coding*	7	3	5	6	3	-0.807	0.947428618	1
ENSBTAG00000046098	*ACSM4*	4	1	2	3	1	-1.585	0.952247636	1
ENSBTAG00000046100	*pseudogene*	6'151	13'150	10'256	5'327	15'184	1.511	0.052523259	1
ENSBTAG00000046101	*protein_coding*	1'558	794	1'133	1'349	917	-0.557	0.496449696	1
ENSBTAG00000046103	*protein_coding*	24	31	28	21	36	0.784	0.740807928	1
ENSBTAG00000046104	*BT.30969*	1'301	511	858	1'127	590	-0.933	0.267096659	1
ENSBTAG00000046105	*PRTN3*	1	1	1	1	1	0.415	1	1
ENSBTAG00000046107	*protein_coding*	11	0	5	10	0		0.617662983	1
ENSBTAG00000046108	*protein_coding*	2	3	3	2	3	1.000	0.97171833	1
ENSBTAG00000046110	*CLCF1*	161	78	115	139	90	-0.630	0.61634153	1
ENSBTAG00000046111	*ZNF444*	324	225	270	281	260	-0.111	0.914818197	1
ENSBTAG00000046112	*miRNA*	1	0	0	1	0		1	1
ENSBTAG00000046113	*U1*	15	15	15	13	17	0.415	0.918564425	1
ENSBTAG00000046115	*protein_coding*	12	3	7	10	3	-1.585	0.809704871	1
ENSBTAG00000046117	*TMSB4X*	4'284	9'635	7'418	3'710	11'126	1.584	0.043164488	1
ENSBTAG00000046118	*pseudogene*	28	20	24	24	23	-0.070	0.998498845	1
ENSBTAG00000046119	*protein_coding*	1	0	0	1	0		1	1
ENSBTAG00000046121	*protein_coding*	47	44	46	41	51	0.320	0.868408185	1

Ensembl gene ID	geneName	counts wildtype horn bud	counts polled horn bud	baseMean	baseMean wildtype horn bud	baseMean polled horn bud	log2FoldChange	pval	padj
ENSBTAG00000046122	*NRARP*	96	81	88	83	94	0.170	0.907933942	1
ENSBTAG00000046124	*KRT36*	4	1	2	3	1	-1.585	0.952247636	1
ENSBTAG00000046127	*PAG5*	1	0	0	1	0		1	1
ENSBTAG00000046129	*RAB34*	4'209	4'969	4'691	3'645	5'738	0.655	0.39788583	1
ENSBTAG00000046131	*SHROOM2*	0	37	21	0	43	Inf	0.054546593	1
ENSBTAG00000046133	*bta-mir-2887-2*	439	196	303	380	226	-0.748	0.439747354	1
ENSBTAG00000046134	*miRNA*	1	0	0	1	0		1	1
ENSBTAG00000046135	*protein_coding*	6'026	3'302	4'516	5'219	3'813	-0.453	0.559037922	1
ENSBTAG00000046138	*C1ORF68*	4	2	3	3	2	-0.585	0.996239345	1
ENSBTAG00000046139	*protein_coding*	0	1	1	0	1	Inf	0.993540919	1
ENSBTAG00000046140	*NPDC1*	692	201	416	599	232	-1.369	0.138930367	1
ENSBTAG00000046142	*PRKAR1B*	887	874	889	768	1'009	0.394	0.636287074	1
ENSBTAG00000046143	*protein_coding*	1	0	0	1	0		1	1
ENSBTAG00000046144	*DEFB7*	1	1	1	1	1	0.415	1	1
ENSBTAG00000046148	*C17ORF97*	7	2	4	6	2	-1.392	0.903936064	1
ENSBTAG00000046150	*protein_coding*	12	4	8	10	5	-1.170	0.853405557	1
ENSBTAG00000046151	*pseudogene*	2	0	1	2	0		0.974934741	1
ENSBTAG00000046152	*MGAM*	3	3	3	3	3	0.415	1	1
ENSBTAG00000046153	*processed_pseudogene*	337	795	605	292	918	1.653	0.061484839	1
ENSBTAG00000046154	*miRNA*	0	1	1	0	1	Inf	0.993540919	1
ENSBTAG00000046155	*RGN*	1'005	424	680	870	490	-0.830	0.333996472	1
ENSBTAG00000046156	*SIAT4B*	1'018	1'186	1'126	882	1'369	0.635	0.43764545	1
ENSBTAG00000046158	*CFB*	141	231	194	122	267	1.127	0.292897931	1
ENSBTAG00000046159	*ITM2A*	1	3	2	1	3	2.000	0.933774563	1
ENSBTAG00000046160	*BT.33915*	1'789	694	1'175	1'549	801	-0.951	0.246564236	1
ENSBTAG00000046161	*BT.101554*	1	0	0	1	0		1	1
ENSBTAG00000046162	*ZNF385C*	50	35	42	43	40	-0.100	0.972970357	1
ENSBTAG00000046165	*RPL31*	2	2	2	2	2	0.415	1	1
ENSBTAG00000046166	*protein_coding*	13	7	10	11	8	-0.478	0.940171262	1
ENSBTAG00000046168	*protein_coding*	1	0	0	1	0		1	1
ENSBTAG00000046169	*CPA1*	22	2	11	19	2	-3.044	0.517475779	1
ENSBTAG00000046172	*protein_coding*	2'388	785	1'487	2'068	906	-1.190	0.143040713	1
ENSBTAG00000046173	*BT.96691*	976	1'080	1'046	845	1'247	0.561	0.495061082	1
ENSBTAG00000046174	*GNA13*	273	313	299	236	361	0.612	0.52693175	1
ENSBTAG00000046175	*protein_coding*	5	4	4	4	5	0.093	1	1
ENSBTAG00000046176	*BT.39893*	1'337	1'701	1'561	1'158	1'964	0.762	0.342841435	1
ENSBTAG00000046177	*IGFN1*	18	24	22	16	28	0.830	0.765788213	1
ENSBTAG00000046178	*processed_pseudogene*	424	301	357	367	348	-0.079	0.936247924	1
ENSBTAG00000046180	*pseudogene*	234	255	249	203	294	0.539	0.592267068	1
ENSBTAG00000046181	*protein_coding*	0	1	1	0	1	Inf	0.993540919	1
ENSBTAG00000046182	*miRNA*	1	1	1	1	1	0.415	1	1
ENSBTAG00000046183	*U2*	9	4	6	8	5	-0.755	0.931866093	1
ENSBTAG00000046184	*pseudogene*	2'637	3'071	2'915	2'284	3'546	0.635	0.417334421	1
ENSBTAG00000046185	*BTBD7*	605	846	750	524	977	0.899	0.29020035	1
ENSBTAG00000046186	*protein_coding*	48	14	29	42	16	-1.363	0.559836341	1
ENSBTAG00000046188	*ELA2*	4	3	3	3	3	0.000	1	1
ENSBTAG00000046189	*RNASE13*	45	17	29	39	20	-0.989	0.671023777	1
ENSBTAG00000046190	*PCDHA3*	13	0	6	11	0		0.551229012	1
ENSBTAG00000046191	*ADAD1*	0	1	1	0	1	Inf	0.993540919	1
ENSBTAG00000046192	*TAF9*	831	299	532	720	345	-1.060	0.232359222	1
ENSBTAG00000046193	*COL21A1*	18'874	6'265	11'790	16'345	7'234	-1.176	0.128047286	1
ENSBTAG00000046194	*miRNA*	6	6	6	5	7	0.415	0.976119138	1
ENSBTAG00000046199	*BT.65826*	409	269	332	354	311	-0.189	0.844733336	1
ENSBTAG00000046204	*BT.29226*	838	408	598	726	471	-0.623	0.47388012	1
ENSBTAG00000046208	*SCRT1*	24	0	10	21	0		0.291206645	1
ENSBTAG00000046211	*protein_coding*	1	3	2	1	3	2.000	0.933774563	1
ENSBTAG00000046213	*SNORD116*	10	2	5	9	2	-1.907	0.815196829	1
ENSBTAG00000046214	*bta-mir-3613*	10	0	4	9	0		0.653360153	1
ENSBTAG00000046215	*TMEM203*	832	858	856	721	991	0.459	0.582429489	1
ENSBTAG00000046216	*protein_coding*	15	5	9	13	6	-1.170	0.820729911	1
ENSBTAG00000046218	*TIEG2*	4'183	2'316	3'148	3'623	2'674	-0.438	0.575326124	1
ENSBTAG00000046220	*protein_coding*	45	20	31	39	23	-0.755	0.740668018	1
ENSBTAG00000046222	*SNORA81*	1	0	0	1	0		1	1
ENSBTAG00000046223	*ANAPC2*	2'146	2'736	2'509	1'858	3'159	0.765	0.331240961	1
ENSBTAG00000046225	*BT.23406*	1	0	0	1	0		1	1
ENSBTAG00000046229	*protein_coding*	499	166	312	432	192	-1.173	0.225749034	1
ENSBTAG00000046231	*protein_coding*	575	292	418	498	337	-0.563	0.53866052	1
ENSBTAG00000046235	*SRSF12*	287	184	231	249	212	-0.226	0.828785721	1
ENSBTAG00000046237	*pseudogene*	325	455	403	281	525	0.900	0.32745419	1
ENSBTAG00000046238	*miRNA*	2	0	1	2	0		0.974934741	1
ENSBTAG00000046239	*C10ORF12*	107	73	88	93	84	-0.137	0.929564765	1
ENSBTAG00000046244	*miRNA*	27	18	22	23	21	-0.170	0.968634064	1

Ensembl gene ID	geneName	counts wildtype horn bud	counts polled horn bud	baseMean	baseMean wildtype horn bud	baseMean polled horn bud	log2FoldChange	pval	padj
ENSBTAG00000046245	*protein_coding*	50	16	31	43	18	-1.229	0.585098432	1
ENSBTAG00000046246	*U3*	6	6	6	5	7	0.415	0.976119138	1
ENSBTAG00000046248	*ARPC1B*	2'200	1'858	2'025	1'905	2'145	0.171	0.82837177	1
ENSBTAG00000046250	*MIOX*	2	3	3	2	3	1.000	0.97171833	1
ENSBTAG00000046255	*ATXN1L*	310	503	425	268	581	1.113	0.223868813	1
ENSBTAG00000046256	*TMEM132C*	3'334	3'343	3'374	2'887	3'860	0.419	0.590156046	1
ENSBTAG00000046257	*GIMAP4*	264	38	136	229	44	-2.381	0.053269459	1
ENSBTAG00000046258	*SOX7*	255	168	207	221	194	-0.187	0.863105574	1
ENSBTAG00000046259	*pseudogene*	274	160	211	237	185	-0.361	0.732757493	1
ENSBTAG00000046260	*protein_coding*	3	4	4	3	5	0.830	0.962490925	1
ENSBTAG00000046261	*pseudogene*	1	0	0	1	0		1	1
ENSBTAG00000046262	*PCDHB7*	263	155	203	228	179	-0.348	0.744836878	1
ENSBTAG00000046263	*ADAMTSL5*	505	380	438	437	439	0.005	0.998046439	1
ENSBTAG00000046264	*protein_coding*	288	91	177	249	105	-1.247	0.258569989	1
ENSBTAG00000046265	*protein_coding*	770	331	525	667	382	-0.803	0.364783069	1
ENSBTAG00000046271	*ZIC5*	29	1	13	25	1	-4.443	0.318957573	1
ENSBTAG00000046273	*MGC155170*	811	768	795	702	887	0.336	0.689216331	1
ENSBTAG00000046276	*protein_coding*	89	53	69	77	61	-0.333	0.833343624	1
ENSBTAG00000046277	*RGS4*	1'630	2'255	2'008	1'412	2'604	0.883	0.26704428	1
ENSBTAG00000046282	*VGLL2*	1	16	10	1	18	4.415	0.411390385	1
ENSBTAG00000046283	*protein_coding*	9	3	6	8	3	-1.170	0.890813994	1
ENSBTAG00000046286	*CCNB3*	385	259	316	333	299	-0.157	0.873284285	1
ENSBTAG00000046289	*U6*	1	0	0	1	0		1	1
ENSBTAG00000046290	*PIRT*	5	6	6	4	7	0.678	0.946418607	1
ENSBTAG00000046291	*FAM83E*	1	3	2	1	3	2.000	0.933774563	1
ENSBTAG00000046293	*ADAM30*	1	0	0	1	0		1	1
ENSBTAG00000046294	*protein_coding*	2	1	1	2	1	-0.585	1	1
ENSBTAG00000046295	*BT.88245*	17'254	7'781	11'964	14'942	8'985	-0.734	0.338829473	1
ENSBTAG00000046301	*ZNF445*	919	942	942	796	1'088	0.451	0.5865125	1
ENSBTAG00000046303	*ENG*	4'325	3'391	3'831	3'746	3'916	0.064	0.933814991	1
ENSBTAG00000046304	*U1*	6	5	5	5	6	0.152	1	1
ENSBTAG00000046305	*BT.64458*	1	0	0	1	0		1	1
ENSBTAG00000046307	*pseudogene*	1'121	825	962	971	953	-0.027	0.97579924	1
ENSBTAG00000046308	*BT.105888*	10	93	58	9	107	3.632	0.041511785	1
ENSBTAG00000046309	*BT.87814*	24	10	16	21	12	-0.848	0.806552039	1
ENSBTAG00000046311	*processed_pseudogene*	53	97	79	46	112	1.287	0.370231723	1
ENSBTAG00000046312	*U6*	2	0	1	2	0		0.974934741	1
ENSBTAG00000046313	*CCDC54*	3	0	1	3	0		0.936647693	1
ENSBTAG00000046314	*protein_coding*	254	172	209	220	199	-0.147	0.892934956	1
ENSBTAG00000046316	*processed_pseudogene*	250	266	262	217	307	0.505	0.612175101	1
ENSBTAG00000046319	*NDUFA7*	1'767	1'580	1'677	1'530	1'824	0.254	0.750376697	1
ENSBTAG00000046320	*BLR1*	2	2	2	2	2	0.415	1	1
ENSBTAG00000046321	*BZRAP1*	1'408	875	1'115	1'219	1'010	-0.271	0.741414869	1
ENSBTAG00000046323	*protein_coding*	488	703	617	423	812	0.942	0.278094998	1
ENSBTAG00000046324	*protein_coding*	396	135	249	343	156	-1.138	0.261572997	1
ENSBTAG00000046325	*protein_coding*	822	839	840	712	969	0.445	0.595270331	1
ENSBTAG00000046328	*XG*	156	151	155	135	174	0.368	0.747941637	1
ENSBTAG00000046333	*BT.87502*	3	46	28	3	53	4.354	0.103161893	1
ENSBTAG00000046335	*processed_pseudogene*	962	1'283	1'157	833	1'481	0.830	0.31063756	1
ENSBTAG00000046336	*BT.91670*	7	8	8	6	9	0.608	0.932722577	1
ENSBTAG00000046337	*TUBB6*	2'545	2'938	2'798	2'204	3'393	0.622	0.427163467	1
ENSBTAG00000046338	*PPP2R3B*	402	423	418	348	488	0.488	0.592059166	1
ENSBTAG00000046339	*BT.69318*	4'215	4'182	4'240	3'650	4'829	0.404	0.601761503	1
ENSBTAG00000046343	*CCNJL*	103	151	132	89	174	0.967	0.418235618	1
ENSBTAG00000046344	*pseudogene*	165	220	198	143	254	0.830	0.434611746	1
ENSBTAG00000046345	*protein_coding*	25	26	26	22	30	0.472	0.857186998	1
ENSBTAG00000046346	*PACS2*	2'545	4'480	3'689	2'204	5'173	1.231	0.117334328	1
ENSBTAG00000046350	*PKP1*	5'712	9'590	8'010	4'947	11'074	1.163	0.133557502	1
ENSBTAG00000046351	*RN28S1*	12'539	8'107	10'110	10'859	9'361	-0.214	0.780097219	1
ENSBTAG00000046352	*pseudogene*	0	1	1	0	1	Inf	0.993540919	1
ENSBTAG00000046357	*SLC29A3*	160	244	210	139	282	1.024	0.329291505	1
ENSBTAG00000046358	*PABPC1*	34'205	26'323	30'009	29'622	30'395	0.037	0.96036046	1
ENSBTAG00000046359	*PHRF1*	2'499	3'322	3'000	2'164	3'836	0.826	0.292337271	1
ENSBTAG00000046362	*BBIP1*	738	220	447	639	254	-1.331	0.145310265	1
ENSBTAG00000046364	*protein_coding*	444	126	265	385	145	-1.402	0.163082299	1
ENSBTAG00000046365	*LGALS7B*	1'698	2'578	2'224	1'471	2'977	1.017	0.200316501	1
ENSBTAG00000046367	*protein_coding*	110	67	86	95	77	-0.300	0.835040434	1
ENSBTAG00000046372	*protein_coding*	1	0	0	1	0		1	1
ENSBTAG00000046374	*processed_pseudogene*	6	0	3	5	0		0.81115604	1
ENSBTAG00000046375	*BT.65427*	1	1	1	1	1	0.415	1	1
ENSBTAG00000046376	*protein_coding*	14	4	8	12	5	-1.392	0.803716462	1
ENSBTAG00000046379	*SLC34A3*	85	79	82	74	91	0.309	0.830951248	1

Ensembl gene ID	geneName	counts wildtype horn bud	counts polled horn bud	baseMean	baseMean wildtype horn bud	baseMean polled horn bud	log2FoldChange	pval	padj
ENSBTAG00000046380	*protein_coding*	1	0	0	1	0		1	1
ENSBTAG00000046383	*BT.97089*	20	3	10	17	3	-2.322	0.618209332	1
ENSBTAG00000046385	*BT.24043*	2'381	1'603	1'956	2'062	1'851	-0.156	0.845318839	1
ENSBTAG00000046386	*SERTM1*	161	28	86	139	32	-2.109	0.139977079	1
ENSBTAG00000046389	*KLRD1*	9	0	4	8	0		0.690676647	1
ENSBTAG00000046391	*GRAMD2*	71	72	72	61	83	0.435	0.773032403	1
ENSBTAG00000046393	*U4*	1	2	2	1	2	1.415	0.981979269	1
ENSBTAG00000046396	*TMEM200C*	50	1	22	43	1	-5.229	0.119305888	1
ENSBTAG00000046399	*BT.86404*	12	13	13	10	15	0.531	0.905259543	1
ENSBTAG00000046400	*miRNA*	3	0	1	3	0		0.936647693	1
ENSBTAG00000046402	*ASCL4*	3	2	2	3	2	-0.170	1	1
ENSBTAG00000046406	*R3HDM4*	1'873	2'177	2'068	1'622	2'514	0.632	0.424909831	1
ENSBTAG00000046407	*NYX*	1	0	0	1	0		1	1
ENSBTAG00000046408	*FAM124A*	27	95	67	23	110	2.230	0.157343159	1
ENSBTAG00000046409	*BT.96766*	183	113	144	158	130	-0.280	0.813730906	1
ENSBTAG00000046411	*5S_rRNA*	1	1	1	1	1	0.415	1	1
ENSBTAG00000046412	*protein_coding*	1	0	0	1	0		1	1
ENSBTAG00000046413	*GRID2*	51	1	23	44	1	-5.257	0.113891005	1
ENSBTAG00000046415	*SSNA1*	1'452	1'092	1'259	1'257	1'261	0.004	0.996546293	1
ENSBTAG00000046416	*protein_coding*	1'174	788	963	1'017	910	-0.160	0.848128738	1
ENSBTAG00000046418	*RNF224*	5	14	10	4	16	1.900	0.676791734	1
ENSBTAG00000046419	*TPC3*	1'181	1'193	1'200	1'023	1'378	0.430	0.5974958	1
ENSBTAG00000046420	*SNORD113*	396	135	249	343	156	-1.138	0.261572997	1
ENSBTAG00000046425	*LEUTX*	0	1	1	0	1	Inf	0.993540919	1
ENSBTAG00000046428	*bta-mir-376e*	1	1	1	1	1	0.415	1	1
ENSBTAG00000046429	*pseudogene*	0	2	1	0	2	Inf	0.939077559	1
ENSBTAG00000046430	*ZNF804B*	13	6	9	11	7	-0.700	0.90529819	1
ENSBTAG00000046431	*TNFRSF10A*	10	5	7	9	6	-0.585	0.94357809	1
ENSBTAG00000046432	*protein_coding*	123	291	221	107	336	1.657	0.114712931	1
ENSBTAG00000046435	*protein_coding*	21	7	13	18	8	-1.170	0.765843478	1
ENSBTAG00000046437	*miRNA*	0	1	1	0	1	Inf	0.993540919	1
ENSBTAG00000046438	*processed_pseudogene*	52	204	140	45	236	2.387	0.04963207	1
ENSBTAG00000046440	*TRAM1L1*	158	72	110	137	83	-0.719	0.572907116	1
ENSBTAG00000046442	*protein_coding*	1	0	0	1	0		1	1
ENSBTAG00000046443	*BT.45033*	6	1	3	5	1	-2.170	0.885240772	1
ENSBTAG00000046444	*pseudogene*	1'451	983	1'196	1'257	1'135	-0.147	0.858412067	1
ENSBTAG00000046447	*CLIC6*	1	0	0	1	0		1	1
ENSBTAG00000046448	*protein_coding*	1	0	0	1	0		1	1
ENSBTAG00000046449	*DOCK2*	10	3	6	9	3	-1.322	0.863266279	1
ENSBTAG00000046450	*EIF1*	7'414	6'722	7'091	6'421	7'762	0.274	0.720887941	1
ENSBTAG00000046452	*protein_coding*	4	0	2	3	0		0.89545886	1
ENSBTAG00000046454	*DYTN*	2	0	1	2	0		0.974934741	1
ENSBTAG00000046457	*protein_coding*	4	3	3	3	3	0.000	1	1
ENSBTAG00000046461	*protein_coding*	8	6	7	7	7	0.000	1	1
ENSBTAG00000046463	*pseudogene*	7	15	12	6	17	1.515	0.71450562	1
ENSBTAG00000046467	*PTP4A3*	581	803	715	503	927	0.882	0.301555576	1
ENSBTAG00000046468	*processed_pseudogene*	1	2	2	1	2	1.415	0.981979269	1
ENSBTAG00000046472	*BOSTAUV1R417*	1	0	0	1	0		1	1
ENSBTAG00000046476	*IGSF1*	205	306	265	178	353	0.993	0.318197281	1
ENSBTAG00000046480	*bta-mir-411c*	4	2	3	3	2	-0.585	0.996239345	1
ENSBTAG00000046481	*BT.38521*	503	466	487	436	538	0.305	0.732663868	1
ENSBTAG00000046482	*RNASE7*	3	1	2	3	1	-1.170	0.985253077	1
ENSBTAG00000046484	*AIF1L*	601	304	436	520	351	-0.568	0.531817223	1
ENSBTAG00000046485	*BT.59545*	2'156	2'350	2'290	1'867	2'714	0.539	0.493775877	1
ENSBTAG00000046486	*BT.89752*	3'693	6'644	5'435	3'198	7'672	1.262	0.105873733	1
ENSBTAG00000046489	*protein_coding*	0	3	2	0	3	Inf	0.878646179	1
ENSBTAG00000046491	*protein_coding*	0	1	1	0	1	Inf	0.993540919	1
ENSBTAG00000046493	*PDCD6*	3'603	2'607	3'065	3'120	3'010	-0.052	0.948184519	1
ENSBTAG00000046494	*CORT*	49	1	22	42	1	-5.200	0.124985357	1
ENSBTAG00000046495	*pseudogene*	3	5	4	3	6	1.152	0.920866718	1
ENSBTAG00000046498	*SUN1*	956	825	890	828	953	0.202	0.808093112	1
ENSBTAG00000046502	*BT.17458*	513	321	407	444	371	-0.261	0.77751102	1
ENSBTAG00000046503	*BT.44795*	188	229	214	163	264	0.700	0.502145553	1
ENSBTAG00000046506	*pseudogene*	42	23	31	36	27	-0.454	0.847674521	1
ENSBTAG00000046507	*bta-mir-3432-2*	1	0	0	1	0		1	1
ENSBTAG00000046509	*FAM46C*	131	288	223	113	333	1.552	0.137942068	1
ENSBTAG00000046514	*BT.65226*	1'273	1'369	1'342	1'102	1'581	0.520	0.520136075	1
ENSBTAG00000046516	*FUT7*	16	6	10	14	7	-1.000	0.835750236	1
ENSBTAG00000046517	*protein_coding*	21	13	17	18	15	-0.277	0.951008818	1
ENSBTAG00000046519	*C5H12ORF4*	102	68	83	88	79	-0.170	0.912337467	1
ENSBTAG00000046520	*TCFL5*	825	441	612	714	509	-0.489	0.573554446	1
ENSBTAG00000046521	*protein_coding*	12	7	9	10	8	-0.363	0.964206651	1

Ensembl gene ID	geneName	counts wildtype horn bud	counts polled horn bud	baseMean	baseMean wildtype horn bud	baseMean polled horn bud	log2FoldChange	pval	padj
ENSBTAG00000046524	*snoZ17*	24	27	26	21	31	0.585	0.818627771	1
ENSBTAG00000046526	*ISCA1*	1'586	1'333	1'456	1'374	1'539	0.164	0.838001143	1
ENSBTAG00000046528	*SNORD53_SNORD92*	2	0	1	2	0		0.974934741	1
ENSBTAG00000046529	*miRNA*	45	17	29	39	20	-0.989	0.671023777	1
ENSBTAG00000046531	*RPS26*	25'241	18'451	21'582	21'859	21'305	-0.037	0.961907374	1
ENSBTAG00000046532	*U3*	3	1	2	3	1	-1.170	0.985253077	1
ENSBTAG00000046533	*BT.64758*	681	1'119	941	590	1'292	1.132	0.175963036	1
ENSBTAG00000046542	*C19ORF24*	627	576	604	543	665	0.293	0.735769728	1
ENSBTAG00000046543	*SNORD109A*	3	5	4	3	6	1.152	0.920866718	1
ENSBTAG00000046544	*BT.97125*	2'394	2'559	2'514	2'073	2'955	0.511	0.515087613	1
ENSBTAG00000046545	*MEF2D*	1'271	1'704	1'534	1'101	1'968	0.838	0.298103732	1
ENSBTAG00000046547	*protein_coding*	38	16	26	33	18	-0.833	0.742877625	1
ENSBTAG00000046548	*SIAT7B*	985	1'101	1'062	853	1'271	0.576	0.483510987	1
ENSBTAG00000046549	*BICD2*	2'161	1'796	1'973	1'871	2'074	0.148	0.851456007	1
ENSBTAG00000046551	*SNORA32*	18	12	15	16	14	-0.170	0.983826208	1
ENSBTAG00000046552	*A2LD1*	240	131	180	208	151	-0.458	0.676714829	1
ENSBTAG00000046553	*SECISBP2L*	560	271	399	485	313	-0.632	0.492817875	1
ENSBTAG00000046554	*pseudogene*	2	0	1	2	0		0.974934741	1
ENSBTAG00000046555	*WAS*	103	40	68	89	46	-0.950	0.536383382	1
ENSBTAG00000046556	*SOX4*	991	802	892	858	926	0.110	0.89557768	1
ENSBTAG00000046557	*miRNA*	2	2	2	2	2	0.415	1	1
ENSBTAG00000046558	*U6*	1	0	0	1	0		1	1
ENSBTAG00000046560	*pseudogene*	25	21	23	22	24	0.163	0.967026967	1
ENSBTAG00000046561	*LCORL*	736	374	535	637	432	-0.562	0.524624191	1
ENSBTAG00000046566	*pseudogene*	613	691	664	531	798	0.588	0.493019847	1
ENSBTAG00000046569	*CISD3*	145	136	141	126	157	0.323	0.784943301	1
ENSBTAG00000046570	*pseudogene*	48	169	118	42	195	2.231	0.080151634	1
ENSBTAG00000046573	*PGBD1*	1'072	629	827	928	726	-0.354	0.673917674	1
ENSBTAG00000046574	*FRMPD1*	44	56	51	38	65	0.763	0.659238996	1
ENSBTAG00000046579	*pseudogene*	5	1	3	4	1	-1.907	0.9188325	1
ENSBTAG00000046580	*BT.27564*	523	538	537	453	621	0.456	0.60418307	1
ENSBTAG00000046583	*TMEM61*	67	39	52	58	45	-0.366	0.840505885	1
ENSBTAG00000046584	*protein_coding*	3	2	2	3	2	-0.170	1	1
ENSBTAG00000046585	*bta-mir-433*	144	318	246	125	367	1.558	0.127777162	1
ENSBTAG00000046586	*BT.98128*	40	163	111	35	188	2.442	0.062531796	1
ENSBTAG00000046587	*BT.88773*	973	663	804	843	766	-0.138	0.871101396	1
ENSBTAG00000046588	*DNAJB9*	1'779	1'033	1'367	1'541	1'193	-0.369	0.648450685	1
ENSBTAG00000046590	*FAM181A*	19	0	8	16	0		0.389538827	1
ENSBTAG00000046591	*pseudogene*	3	1	2	3	1	-1.170	0.985253077	1
ENSBTAG00000046593	*protein_coding*	2	0	1	2	0		0.974934741	1
ENSBTAG00000046594	*protein_coding*	103	171	143	89	197	1.146	0.325755463	1
ENSBTAG00000046595	*BT.102975*	4	10	8	3	12	1.737	0.768040958	1
ENSBTAG00000046597	*GPRIN2*	17	23	21	15	27	0.851	0.766760787	1
ENSBTAG00000046598	*protein_coding*	29	10	18	25	12	-1.121	0.717983518	1
ENSBTAG00000046600	*pseudogene*	1	0	0	1	0		1	1
ENSBTAG00000046601	*protein_coding*	45	73	62	39	84	1.113	0.483324034	1
ENSBTAG00000046604	*GREM1*	376	290	330	326	335	0.040	0.968816773	1
ENSBTAG00000046607	*BT.45180*	384	500	455	333	577	0.796	0.377406325	1
ENSBTAG00000046608	*pseudogene*	463	278	361	401	321	-0.321	0.733351409	1
ENSBTAG00000046609	*STELLA*	1	0	0	1	0		1	1
ENSBTAG00000046612	*ZNF292*	3'510	1'889	2'610	3'040	2'181	-0.479	0.542475826	1
ENSBTAG00000046614	*BT.56877*	40	10	23	35	12	-1.585	0.551930545	1
ENSBTAG00000046615	*protein_coding*	2	0	1	2	0		0.974934741	1
ENSBTAG00000046620	*protein_coding*	870	993	950	753	1'147	0.606	0.464895493	1
ENSBTAG00000046621	*HIST1H1A*	2	1	1	2	1	-0.585	1	1
ENSBTAG00000046622	*Metazoa_SRP*	44	0	19	38	0		0.093105778	1
ENSBTAG00000046623	*RPL26*	44'747	47'839	46'996	38'752	55'240	0.511	0.500860605	1
ENSBTAG00000046625	*PURB*	95	70	82	82	81	-0.026	0.996684558	1
ENSBTAG00000046626	*CNTD2*	0	1	1	0	1	Inf	0.993540919	1
ENSBTAG00000046628	*LYZ3*	2	10	7	2	12	2.737	0.678653301	1
ENSBTAG00000046631	*protein_coding*	61	40	50	53	46	-0.194	0.923802521	1
ENSBTAG00000046633	*processed_pseudogene*	136	216	184	118	249	1.082	0.319333546	1
ENSBTAG00000046637	*ZBED2*	4	0	2	3	0		0.89545886	1
ENSBTAG00000046640	*miRNA*	6	9	8	5	10	1.000	0.869259965	1
ENSBTAG00000046644	*HRAS*	958	1'253	1'138	830	1'447	0.802	0.327664709	1
ENSBTAG00000046646	*miRNA*	1	0	0	1	0		1	1
ENSBTAG00000046648	*CPA4*	79	35	54	68	40	-0.759	0.654919857	1
ENSBTAG00000046649	*SGPP2*	28	4	14	24	5	-2.392	0.514729066	1
ENSBTAG00000046654	*CERK*	1'053	1'325	1'221	912	1'530	0.747	0.359803131	1
ENSBTAG00000046655	*BT.88132*	640	1'430	1'103	554	1'651	1.575	0.05921429	1
ENSBTAG00000046657	*protein_coding*	807	393	576	699	454	-0.623	0.476148273	1
ENSBTAG00000046662	*TRPM5*	26	23	25	23	27	0.238	0.939424562	1

Ensembl gene ID	geneName	counts wildtype horn bud	counts polled horn bud	baseMean	baseMean wildtype horn bud	baseMean polled horn bud	log2FoldChange	pval	padj
ENSBTAG00000046663	*BT.97971*	0	2	1	0	2	Inf	0.939077559	1
ENSBTAG00000046664	*BT.10953*	1'092	855	966	946	987	0.062	0.940723649	1
ENSBTAG00000046666	*TTC9B*	38	2	18	33	2	-3.833	0.270360231	1
ENSBTAG00000046668	*ANG2*	237	165	198	205	191	-0.107	0.92492145	1
ENSBTAG00000046670	*MLX*	3'197	2'471	2'811	2'769	2'853	0.043	0.955506712	1
ENSBTAG00000046671	*WFS1*	727	1'113	957	630	1'285	1.029	0.216704229	1
ENSBTAG00000046672	*ZDHHC18*	670	735	714	580	849	0.549	0.518969515	1
ENSBTAG00000046675	*miRNA*	1	0	0	1	0		1	1
ENSBTAG00000046676	*protein_coding*	1'123	592	828	973	684	-0.509	0.545183549	1
ENSBTAG00000046677	*RTN4R*	111	94	102	96	109	0.175	0.898443572	1
ENSBTAG00000046678	*protein_coding*	17	40	30	15	46	1.650	0.463393835	1
ENSBTAG00000046679	*pseudogene*	3	2	2	3	2	-0.170	1	1
ENSBTAG00000046680	*5_8S_rRNA*	470	174	304	407	201	-1.019	0.293995504	1
ENSBTAG00000046681	*SNORA73*	82	28	52	71	32	-1.135	0.512076458	1
ENSBTAG00000046684	*FOXN3*	175	225	206	152	260	0.778	0.460048493	1
ENSBTAG00000046688	*protein_coding*	40'695	19'004	28'593	35'243	21'944	-0.684	0.370654161	1
ENSBTAG00000046689	*miRNA*	27	4	14	23	5	-2.340	0.531990604	1
ENSBTAG00000046690	*protein_coding*	111	102	107	96	118	0.293	0.822735112	1
ENSBTAG00000046694	*FNDC9*	1	0	0	1	0		1	1
ENSBTAG00000046695	*miRNA*	245	104	166	212	120	-0.821	0.462854206	1
ENSBTAG00000046699	*STAT4*	10	14	12	9	16	0.900	0.826937278	1
ENSBTAG00000046700	*pseudogene*	231	612	453	200	707	1.821	0.048421943	1
ENSBTAG00000046701	*FRAT2*	24	13	18	21	15	-0.469	0.89373307	1
ENSBTAG00000046702	*SNORA67*	13	4	8	11	5	-1.285	0.828302954	1
ENSBTAG00000046703	*NPW*	12	17	15	10	20	0.918	0.796381602	1
ENSBTAG00000046705	*U6*	1	3	2	1	3	2.000	0.933774563	1
ENSBTAG00000046706	*U6*	0	1	1	0	1	Inf	0.993540919	1
ENSBTAG00000046708	*PDP2*	188	171	180	163	197	0.278	0.800596853	1
ENSBTAG00000046711	*DEFB112*	4	0	2	3	0		0.89545886	1
ENSBTAG00000046712	*PSMC2*	7'081	5'042	5'977	6'132	5'822	-0.075	0.923268505	1
ENSBTAG00000046713	*pseudogene*	429	273	343	372	315	-0.237	0.804144431	1
ENSBTAG00000046714	*pseudogene*	16	5	10	14	6	-1.263	0.797689913	1
ENSBTAG00000046716	*UBE2D3*	51	41	46	44	47	0.100	0.96809477	1
ENSBTAG00000046717	*BT.87618*	23	10	16	20	12	-0.787	0.825334133	1
ENSBTAG00000046720	*BT.76551*	0	3	2	0	3	Inf	0.878646179	1
ENSBTAG00000046722	*BT.88628*	2	4	3	2	5	1.415	0.927250049	1
ENSBTAG00000046723	*SYT14*	13	1	6	11	1	-3.285	0.663144098	1
ENSBTAG00000046724	*CBX6*	1'331	2'834	2'213	1'153	3'272	1.505	0.060937992	1
ENSBTAG00000046726	*CLDN20*	15	7	11	13	8	-0.684	0.893749952	1
ENSBTAG00000046727	*protein_coding*	1	1	1	1	1	0.415	1	1
ENSBTAG00000046728	*GPR152*	6	1	3	5	1	-2.170	0.885240772	1
ENSBTAG00000046730	*protein_coding*	250	325	296	217	375	0.794	0.413613375	1
ENSBTAG00000046732	*MMP8*	1	0	0	1	0		1	1
ENSBTAG00000046733	*protein_coding*	5	5	5	4	6	0.415	0.985023983	1
ENSBTAG00000046739	*HEATR7A*	1	0	0	1	0		1	1
ENSBTAG00000046742	*CLEC2A*	10	1	5	9	1	-2.907	0.754028015	1
ENSBTAG00000046744	*PALM3*	89	59	73	77	68	-0.178	0.91387605	1
ENSBTAG00000046746	*BT.88700*	1'080	997	1'043	935	1'151	0.300	0.715332285	1
ENSBTAG00000046747	*protein_coding*	3	1	2	3	1	-1.170	0.985253077	1
ENSBTAG00000046749	*protein_coding*	60	5	29	52	6	-3.170	0.201573083	1
ENSBTAG00000046750	*B3GNT3*	351	168	249	304	194	-0.648	0.520999298	1
ENSBTAG00000046752	*PRRT2*	40	46	44	35	53	0.617	0.743422959	1
ENSBTAG00000046753	*protein_coding*	11	1	5	10	1	-3.044	0.722857941	1
ENSBTAG00000046755	*protein_coding*	56	117	92	48	135	1.478	0.277615839	1
ENSBTAG00000046757	*GTF2H4*	828	1'050	965	717	1'212	0.758	0.360898628	1
ENSBTAG00000046758	*RNF223*	8	11	10	7	13	0.874	0.862196486	1
ENSBTAG00000046759	*snoU13*	1	0	0	1	0		1	1
ENSBTAG00000046760	*BRD1*	1'613	1'860	1'772	1'397	2'148	0.621	0.436360285	1
ENSBTAG00000046763	*MGC143103*	44	25	33	38	29	-0.401	0.862131833	1
ENSBTAG00000046765	*BT.64620*	4'340	8'683	6'892	3'759	10'026	1.416	0.06984507	1
ENSBTAG00000046766	*protein_coding*	49	36	42	42	42	-0.030	1	1
ENSBTAG00000046768	*IGFBP1*	7	0	3	6	0		0.769774961	1
ENSBTAG00000046769	*protein_coding*	95	49	69	82	57	-0.540	0.725450651	1
ENSBTAG00000046773	*MCOLN2*	16	3	9	14	3	-2.000	0.709202358	1
ENSBTAG00000046774	*ZNF182*	351	147	237	304	170	-0.841	0.410541291	1
ENSBTAG00000046775	*MAN1B1*	1'062	1'742	1'466	920	2'011	1.129	0.16415901	1
ENSBTAG00000046776	*protein_coding*	100	49	72	87	57	-0.614	0.684456163	1
ENSBTAG00000046783	*protein_coding*	52	52	53	45	60	0.415	0.813238401	1
ENSBTAG00000046785	*miRNA*	0	1	1	0	1	Inf	0.993540919	1
ENSBTAG00000046786	*UQCRFS1*	2'638	1'924	2'253	2'285	2'222	-0.040	0.960493935	1
ENSBTAG00000046790	*protein_coding*	28	0	12	24	0		0.230947873	1
ENSBTAG00000046794	*processed_pseudogene*	149	217	190	129	251	0.957	0.373511193	1

Ensembl gene ID	geneName	counts wildtype horn bud	counts polled horn bud	baseMean	baseMean wildtype horn bud	baseMean polled horn bud	log2FoldChange	pval	padj
ENSBTAG00000046795	*miRNA*	5	3	4	4	3	-0.322	1	1
ENSBTAG00000046797	*NRAS*	6'494	4'807	5'587	5'624	5'551	-0.019	0.981321411	1
ENSBTAG00000046803	*GAS1*	1'588	1'671	1'652	1'375	1'930	0.489	0.540897435	1
ENSBTAG00000046805	*CYP2F1*	68	24	43	59	28	-1.087	0.564173626	1
ENSBTAG00000046807	*protein_coding*	36	0	16	31	0		0.14599784	1
ENSBTAG00000046808	*protein_coding*	550	709	647	476	819	0.781	0.364446907	1
ENSBTAG00000046809	*TBP*	314	182	241	272	210	-0.372	0.716182752	1
ENSBTAG00000046810	*protein_coding*	3	5	4	3	6	1.152	0.920866718	1
ENSBTAG00000046812	*ATPAF1-AS1*	7	2	4	6	2	-1.392	0.903936064	1
ENSBTAG00000046814	*ACOT2*	192	113	148	166	130	-0.350	0.765193089	1
ENSBTAG00000046815	*protein_coding*	0	1	1	0	1	Inf	0.993540919	1
ENSBTAG00000046817	*protein_coding*	2	4	3	2	5	1.415	0.927250049	1
ENSBTAG00000046821	*pseudogene*	831	1'000	937	720	1'155	0.682	0.411475454	1
ENSBTAG00000046823	*BT.65803*	1'515	551	974	1'312	636	-1.044	0.210266629	1
ENSBTAG00000046824	*BT.66153*	1	0	0	1	0		1	1
ENSBTAG00000046828	*protein_coding*	67	245	170	58	283	2.286	0.046036459	1
ENSBTAG00000046830	*pseudogene*	1	0	0	1	0		1	1
ENSBTAG00000046833	*protein_coding*	20	0	9	17	0		0.367514426	1
ENSBTAG00000046834	*bta-mir-2450a*	1	0	0	1	0		1	1
ENSBTAG00000046836	*protein_coding*	19	8	13	16	9	-0.833	0.840803998	1
ENSBTAG00000046837	*ZNF358*	1'869	2'194	2'076	1'619	2'533	0.646	0.414509661	1
ENSBTAG00000046838	*protein_coding*	2	3	3	2	3	1.000	0.97171833	1
ENSBTAG00000046839	*protein_coding*	1'930	1'661	1'795	1'671	1'918	0.198	0.802737044	1
ENSBTAG00000046840	*DEXI*	937	1'001	984	811	1'156	0.510	0.536668938	1
ENSBTAG00000046841	*IRF2BP2*	2'830	1'767	2'246	2'451	2'040	-0.264	0.738132484	1
ENSBTAG00000046842	*U4*	9	3	6	8	3	-1.170	0.890813994	1
ENSBTAG00000046844	*pseudogene*	34	43	40	29	50	0.754	0.702255028	1
ENSBTAG00000046846	*FAM32A*	5'027	3'080	3'955	4'354	3'556	-0.292	0.707444687	1
ENSBTAG00000046848	*protein_coding*	102	19	55	88	22	-2.009	0.239790211	1
ENSBTAG00000046852	*processed_pseudogene*	6	0	3	5	0		0.81115604	1
ENSBTAG00000046854	*BT.89235*	55	54	55	48	62	0.389	0.821722806	1
ENSBTAG00000046855	*protein_coding*	111	119	117	96	137	0.515	0.679310861	1
ENSBTAG00000046857	*C14ORF80*	13	59	40	11	68	2.597	0.198456182	1
ENSBTAG00000046862	*MARCKSL1*	2'335	3'419	2'985	2'022	3'948	0.965	0.219515211	1
ENSBTAG00000046864	*pseudogene*	11	3	6	10	3	-1.459	0.83622464	1
ENSBTAG00000046865	*protein_coding*	7	0	3	6	0		0.769774961	1
ENSBTAG00000046866	*ZNF707*	277	226	250	240	261	0.121	0.906565151	1
ENSBTAG00000046869	*BT.49065*	1'417	1'425	1'436	1'227	1'645	0.423	0.598945227	1
ENSBTAG00000046874	*bta-mir-2887-2*	289	178	228	250	206	-0.284	0.785193458	1
ENSBTAG00000046875	*AP5B1*	607	827	740	526	955	0.861	0.311195384	1
ENSBTAG00000046876	*miRNA*	3	5	4	3	6	1.152	0.920866718	1
ENSBTAG00000046878	*protein_coding*	52	32	41	45	37	-0.285	0.893501315	1
ENSBTAG00000046879	*pseudogene*	856	651	747	741	752	0.020	0.98214627	1
ENSBTAG00000046880	*U6*	1	2	2	1	2	1.415	0.981979269	1
ENSBTAG00000046881	*miRNA*	0	2	1	0	2	Inf	0.939077559	1
ENSBTAG00000046882	*5_8S_rRNA*	702	345	503	608	398	-0.610	0.493214267	1
ENSBTAG00000046885	*RXFP4*	27	37	33	23	43	0.870	0.687294594	1
ENSBTAG00000046886	*BT.105119*	8'144	3'825	5'735	7'053	4'417	-0.675	0.382363528	1
ENSBTAG00000046888	*Metazoa_SRP*	42	11	25	36	13	-1.518	0.554542507	1
ENSBTAG00000046891	*U2*	1	0	0	1	0		1	1
ENSBTAG00000046893	*miRNA*	3	1	2	3	1	-1.170	0.985253077	1
ENSBTAG00000046896	*Metazoa_SRP*	0	1	1	0	1	Inf	0.993540919	1
ENSBTAG00000046899	*miRNA*	2	0	1	2	0		0.974934741	1
ENSBTAG00000046900	*BT.28253*	232	58	134	201	67	-1.585	0.188876658	1
ENSBTAG00000046901	*protein_coding*	52	20	34	45	23	-0.963	0.6528709	1
ENSBTAG00000046902	*processed_pseudogene*	331	290	311	287	335	0.224	0.816782673	1
ENSBTAG00000046903	*pseudogene*	3	1	2	3	1	-1.170	0.985253077	1
ENSBTAG00000046905	*protein_coding*	3	0	1	3	0		0.936647693	1
ENSBTAG00000046906	*protein_coding*	0	1	1	0	1	Inf	0.993540919	1
ENSBTAG00000046908	*protein_coding*	35	54	46	30	62	1.041	0.565137451	1
ENSBTAG00000046910	*processed_pseudogene*	130	209	177	113	241	1.100	0.316408951	1
ENSBTAG00000046911	*SCN10A*	0	1	1	0	1	Inf	0.993540919	1
ENSBTAG00000046912	*Metazoa_SRP*	190	152	170	165	176	0.093	0.937428928	1
ENSBTAG00000046918	*BT.99122*	19	24	22	16	28	0.752	0.786083273	1
ENSBTAG00000046919	*C17ORF48*	369	310	339	320	358	0.164	0.864155329	1
ENSBTAG00000046922	*TWIST1*	1'908	2'202	2'098	1'652	2'543	0.622	0.432130543	1
ENSBTAG00000046924	*SEMA3G*	1'333	2'829	2'211	1'154	3'267	1.501	0.061728501	1
ENSBTAG00000046928	*processed_pseudogene*	380	638	533	329	737	1.163	0.190152336	1
ENSBTAG00000046931	*pseudogene*	702	718	719	608	829	0.448	0.59843156	1
ENSBTAG00000046932	*protein_coding*	1	0	0	1	0		1	1
ENSBTAG00000046933	*pseudogene*	11	0	5	10	0		0.617662983	1
ENSBTAG00000046936	*MRFAP1*	18'491	13'409	15'749	16'014	15'483	-0.049	0.949908657	1

Ensembl gene ID	geneName	counts wildtype horn bud	counts polled horn bud	baseMean	baseMean wildtype horn bud	baseMean polled horn bud	log2FoldChange	pval	padj
ENSBTAG00000046938	*BT.102686*	0	3	2	0	3	Inf	0.878646179	1
ENSBTAG00000046939	*C9ORF69*	1'642	2'338	2'061	1'422	2'700	0.925	0.245006177	1
ENSBTAG00000046941	*processed_pseudogene*	113	259	198	98	299	1.612	0.134716437	1
ENSBTAG00000046942	*protein_coding*	4	0	2	3	0		0.89545886	1
ENSBTAG00000046951	*FGF17*	2	1	1	2	1	-0.585	1	1
ENSBTAG00000046952	*protein_coding*	1'877	2'108	2'030	1'626	2'434	0.582	0.46219533	1
ENSBTAG00000046956	*STYX*	675	409	528	585	472	-0.308	0.728617733	1
ENSBTAG00000046957	*DOK6*	47	43	45	41	50	0.287	0.884179607	1
ENSBTAG00000046959	*BT.85663*	3'317	1'811	2'482	2'873	2'091	-0.458	0.560765526	1
ENSBTAG00000046961	*BT.61239*	1	0	0	1	0		1	1
ENSBTAG00000046966	*GTLF3B*	183	307	256	158	354	1.161	0.247736782	1
ENSBTAG00000046967	*protein_coding*	3	2	2	3	2	-0.170	1	1
ENSBTAG00000046968	*miRNA*	1	2	2	1	2	1.415	0.981979269	1
ENSBTAG00000046971	*protein_coding*	3'334	1'723	2'438	2'887	1'990	-0.537	0.495420954	1
ENSBTAG00000046973	*pseudogene*	1	0	0	1	0		1	1
ENSBTAG00000046974	*pseudogene*	2'075	3'933	3'169	1'797	4'541	1.338	0.090787509	1
ENSBTAG00000046975	*protein_coding*	6	3	4	5	3	-0.585	0.976586913	1
ENSBTAG00000046977	*BT.37581*	5	7	6	4	8	0.900	0.909121035	1
ENSBTAG00000046979	*LGMN*	4'761	3'368	4'006	4'123	3'889	-0.084	0.914279383	1
ENSBTAG00000046980	*KLRF2*	42	0	18	36	0		0.104095408	1
ENSBTAG00000046981	*BT.64122*	9'268	3'590	6'086	8'026	4'145	-0.953	0.218760078	1
ENSBTAG00000046984	*ZNF672*	265	268	269	229	309	0.431	0.663059619	1
ENSBTAG00000046987	*CENPBD1*	30	23	26	26	27	0.032	1	1
ENSBTAG00000046988	*FAM43B*	21	2	10	18	2	-2.977	0.538317156	1
ENSBTAG00000046990	*C21H14orf142*	693	522	601	600	603	0.006	0.995790452	1
ENSBTAG00000046996	*BT.4091*	4'030	5'115	4'698	3'490	5'906	0.759	0.327599563	1
ENSBTAG00000046999	*miRNA*	2	0	1	2	0		0.974934741	1
ENSBTAG00000047002	*FANCM*	71	35	51	61	40	-0.605	0.732035955	1
ENSBTAG00000047003	*ASXL3*	162	88	121	140	102	-0.465	0.708132048	1
ENSBTAG00000047004	*protein_coding*	0	1	1	0	1	Inf	0.993540919	1
ENSBTAG00000047009	*BT.71827*	6	0	3	5	0		0.81115604	1
ENSBTAG00000047019	*pseudogene*	33	2	15	29	2	-3.629	0.331956808	1
ENSBTAG00000047022	*PSCA*	6	0	3	5	0		0.81115604	1
ENSBTAG00000047023	*U2*	3	2	2	3	2	-0.170	1	1
ENSBTAG00000047024	*C6ORF183*	3	4	4	3	5	0.830	0.962490925	1
ENSBTAG00000047027	*C14ORF119*	2'477	1'598	1'995	2'145	1'845	-0.217	0.784793281	1
ENSBTAG00000047029	*protein_coding*	19'313	26'507	23'667	16'726	30'608	0.872	0.254492991	1
ENSBTAG00000047030	*BT.41962*	23	59	44	20	68	1.774	0.342582973	1
ENSBTAG00000047031	*BT.39594*	1'718	2'015	1'907	1'488	2'327	0.645	0.417061897	1
ENSBTAG00000047035	*BT.12647*	1'388	1'643	1'550	1'202	1'897	0.658	0.412286601	1
ENSBTAG00000047036	*GPR39*	7	20	15	6	23	1.930	0.58513434	1
ENSBTAG00000047038	*protein_coding*	12	0	5	10	0		0.583618942	1
ENSBTAG00000047039	*protein_coding*	1	1	1	1	1	0.415	1	1
ENSBTAG00000047043	*DCXR*	0	1	1	0	1	Inf	0.993540919	1
ENSBTAG00000047045	*pseudogene*	2	1	1	2	1	-0.585	1	1
ENSBTAG00000047046	*RN28S1*	10'407	8'244	9'266	9'013	9'519	0.079	0.917294228	1
ENSBTAG00000047048	*protein_coding*	8	27	19	7	31	2.170	0.467342609	1
ENSBTAG00000047051	*processed_pseudogene*	12'292	14'690	13'804	10'645	16'963	0.672	0.379504152	1
ENSBTAG00000047053	*ZNF534*	34	29	31	29	33	0.186	0.946165179	1
ENSBTAG00000047054	*CLRN3*	1	0	0	1	0		1	1
ENSBTAG00000047055	*protein_coding*	44	56	51	38	65	0.763	0.659238996	1
ENSBTAG00000047059	*TIMM17A*	751	826	802	650	954	0.552	0.511310992	1
ENSBTAG00000047061	*BT.27888*	1'102	1'264	1'207	954	1'460	0.613	0.451784103	1
ENSBTAG00000047062	*LRP3*	1'367	1'829	1'648	1'184	2'112	0.835	0.297997106	1
ENSBTAG00000047064	*pseudogene*	3'511	5'229	4'539	3'041	6'038	0.990	0.203776589	1
ENSBTAG00000047069	*pseudogene*	9	4	6	8	5	-0.755	0.931866093	1
ENSBTAG00000047070	*U6*	6	1	3	5	1	-2.170	0.885240772	1
ENSBTAG00000047073	*protein_coding*	84	39	59	73	45	-0.692	0.673539669	1
ENSBTAG00000047074	*U6*	1	0	0	1	0		1	1
ENSBTAG00000047077	*ENTPD7*	378	239	302	327	276	-0.246	0.801622555	1
ENSBTAG00000047078	*NTF4*	66	109	92	57	126	1.139	0.400772379	1
ENSBTAG00000047080	*protein_coding*	299	246	271	259	284	0.134	0.894846363	1
ENSBTAG00000047083	*FJX1*	222	179	199	192	207	0.104	0.925294813	1
ENSBTAG00000047084	*protein_coding*	0	1	1	0	1	Inf	0.993540919	1
ENSBTAG00000047086	*protein_coding*	0	1	1	0	1	Inf	0.993540919	1
ENSBTAG00000047087	*MCI*	1	0	0	1	0		1	1
ENSBTAG00000047088	*BT.91459*	44	8	24	38	9	-2.044	0.440136748	1
ENSBTAG00000047089	*processed_pseudogene*	6'801	6'198	6'523	5'890	7'157	0.281	0.713980319	1
ENSBTAG00000047092	*protein_coding*	91	80	86	79	92	0.229	0.874679914	1
ENSBTAG00000047098	*pseudogene*	4	10	8	3	12	1.737	0.768040958	1
ENSBTAG00000047101	*protein_coding*	4	0	2	3	0		0.89545886	1
ENSBTAG00000047102	*protein_coding*	29	17	22	25	20	-0.355	0.909573651	1

Ensembl gene ID	geneName	counts wildtype horn bud	counts polled horn bud	baseMean	baseMean wildtype horn bud	baseMean polled horn bud	log2FoldChange	pval	padj
ENSBTAG00000047103	*C9ORF103*	266	381	335	230	440	0.933	0.325566124	1
ENSBTAG00000047107	*TNIP3*	1	0	0	1	0		1	1
ENSBTAG00000047108	*pseudogene*	181	167	175	157	193	0.299	0.787635033	1
ENSBTAG00000047111	*protein_coding*	6	1	3	5	1	-2.170	0.885240772	1
ENSBTAG00000047113	*protein_coding*	11	7	9	10	8	-0.237	0.988863286	1
ENSBTAG00000047115	*5S_rRNA*	1	0	0	1	0		1	1
ENSBTAG00000047116	*TPPP*	68	127	103	59	147	1.316	0.312530901	1
ENSBTAG00000047118	*protein_coding*	72	7	35	62	8	-2.948	0.181491191	1
ENSBTAG00000047119	*protein_coding*	71	159	123	61	184	1.578	0.201480227	1
ENSBTAG00000047121	*protein_coding*	32	16	23	28	18	-0.585	0.834862636	1
ENSBTAG00000047124	*ASMT*	19	4	11	16	5	-1.833	0.688634464	1
ENSBTAG00000047127	*C7H19orf25*	255	265	263	221	306	0.471	0.636024698	1
ENSBTAG00000047128	*IL17C*	1	2	2	1	2	1.415	0.981979269	1
ENSBTAG00000047129	*B3GNT6*	37	36	37	32	42	0.376	0.861537192	1
ENSBTAG00000047133	*protein_coding*	727	299	487	630	345	-0.867	0.333082405	1
ENSBTAG00000047134	*BT.87655*	7	1	4	6	1	-2.392	0.851750348	1
ENSBTAG00000047135	*BT.74375*	955	883	923	827	1'020	0.302	0.715980297	1
ENSBTAG00000047136	*RPL39*	32'105	35'481	34'387	27'804	40'970	0.559	0.462235893	1
ENSBTAG00000047138	*AVPR2*	3	2	2	3	2	-0.170	1	1
ENSBTAG00000047139	*C9H12orf49*	796	658	725	689	760	0.140	0.869289642	1
ENSBTAG00000047140	*Metazoa_SRP*	2	0	1	2	0		0.974934741	1
ENSBTAG00000047142	*IFNB1*	2	7	5	2	8	2.222	0.797845137	1
ENSBTAG00000047144	*protein_coding*	7	3	5	6	3	-0.807	0.947428618	1
ENSBTAG00000047146	*pseudogene*	10	5	7	9	6	-0.585	0.94357809	1
ENSBTAG00000047147	*BT.41849*	73	79	77	63	91	0.529	0.716673878	1
ENSBTAG00000047148	*CNKSR1*	118	131	127	102	151	0.566	0.640426579	1
ENSBTAG00000047150	*RTEL*	917	768	840	794	887	0.159	0.849469529	1
ENSBTAG00000047153	*pseudogene*	2	12	8	2	14	3.000	0.606634849	1
ENSBTAG00000047154	*RN28S1*	20'126	14'970	17'358	17'430	17'286	-0.012	0.988244013	1
ENSBTAG00000047155	*C10ORF71*	9	56	36	8	65	3.052	0.157024589	1
ENSBTAG00000047156	*TRDV2*	2	1	1	2	1	-0.585	1	1
ENSBTAG00000047157	*bta-mir-2887-1*	1'059	322	644	917	372	-1.303	0.134835702	1
ENSBTAG00000047158	*LRFN1*	293	550	444	254	635	1.324	0.147157371	1
ENSBTAG00000047161	*ARSH*	174	110	139	151	127	-0.247	0.839003612	1
ENSBTAG00000047164	*BT.63070*	186	42	105	161	48	-1.732	0.187228159	1
ENSBTAG00000047165	*KRT9*	23	10	16	20	12	-0.787	0.825334133	1
ENSBTAG00000047166	*SH2D7*	82	48	63	71	55	-0.358	0.827593087	1
ENSBTAG00000047169	*CXHXorf56*	2'144	1'418	1'747	1'857	1'637	-0.181	0.82099508	1
ENSBTAG00000047171	*U6*	0	1	1	0	1	Inf	0.993540919	1
ENSBTAG00000047174	*protein_coding*	70	172	130	61	199	1.712	0.159843141	1
ENSBTAG00000047176	*protein_coding*	1	0	0	1	0		1	1
ENSBTAG00000047183	*protein_coding*	13	3	7	11	3	-1.700	0.783725709	1
ENSBTAG00000047186	*NME2*	991	1'877	1'513	858	2'167	1.337	0.100523142	1
ENSBTAG00000047191	*KCNA6*	245	293	275	212	338	0.673	0.494041402	1
ENSBTAG00000047194	*protein_coding*	0	1	1	0	1	Inf	0.993540919	1
ENSBTAG00000047196	*protein_coding*	877	1'544	1'271	760	1'783	1.231	0.133306746	1
ENSBTAG00000047199	*protein_coding*	151	130	140	131	150	0.199	0.868944296	1
ENSBTAG00000047200	*HYAL1*	566	430	493	490	497	0.019	0.98520885	1
ENSBTAG00000047203	*TSPAN19*	2	2	2	2	2	0.415	1	1
ENSBTAG00000047205	*protein_coding*	1	0	0	1	0		1	1
ENSBTAG00000047206	*HIST1H1E*	17	7	11	15	8	-0.865	0.849311167	1
ENSBTAG00000047207	*RRAGA*	349	875	656	302	1'010	1.741	0.047417196	1
ENSBTAG00000047208	*protein_coding*	224	267	251	194	308	0.668	0.505548168	1
ENSBTAG00000047209	*IFNAA*	0	1	1	0	1	Inf	0.993540919	1
ENSBTAG00000047210	*processed_pseudogene*	313	332	327	271	383	0.500	0.599072088	1
ENSBTAG00000047213	*protein_coding*	1'033	459	712	895	530	-0.755	0.376669639	1
ENSBTAG00000047214	*TRAPPC1*	1'423	1'211	1'315	1'232	1'398	0.182	0.821658196	1
ENSBTAG00000047216	*NMNAT3*	29	11	19	25	13	-0.984	0.748093269	1
ENSBTAG00000047217	*MED16*	1'053	1'919	1'564	912	2'216	1.281	0.11439621	1
ENSBTAG00000047218	*BT.53973*	57	29	41	49	33	-0.560	0.776893239	1
ENSBTAG00000047219	*ZNF793*	42	24	32	36	28	-0.392	0.868987631	1
ENSBTAG00000047221	*miRNA*	0	1	1	0	1	Inf	0.993540919	1
ENSBTAG00000047223	*GNAS*	59	22	38	51	25	-1.008	0.616166781	1
ENSBTAG00000047225	*BCL2L14*	5	0	2	4	0		0.853268594	1
ENSBTAG00000047226	*MRRF*	341	390	373	295	450	0.609	0.512358849	1
ENSBTAG00000047227	*protein_coding*	112	72	90	97	83	-0.222	0.877895477	1
ENSBTAG00000047228	*pseudogene*	2	13	8	2	15	3.115	0.57299014	1
ENSBTAG00000047229	*CRIP1*	1'430	3'083	2'399	1'238	3'560	1.523	0.0572995	1
ENSBTAG00000047238	*BT.35830*	80	62	70	69	72	0.047	0.984704316	1
ENSBTAG00000047240	*DYNLL1*	580	1'233	963	502	1'424	1.503	0.074137957	1
ENSBTAG00000047242	*Metazoa_SRP*	84	121	106	73	140	0.942	0.462912938	1
ENSBTAG00000047243	*miRNA*	1	0	0	1	0		1	1

Ensembl gene ID	geneName	counts wildtype horn bud	counts polled horn bud	baseMean	baseMean wildtype horn bud	baseMean polled horn bud	log2FoldChange	pval	padj
ENSBTAG00000047247	*C3ORF80*	45	7	24	39	8	-2.269	0.396514456	1
ENSBTAG00000047248	*protein_coding*	199	421	329	172	486	1.496	0.120372256	1
ENSBTAG00000047249	*WBSCR1*	15'531	13'855	14'724	13'450	15'998	0.250	0.742122225	1
ENSBTAG00000047250	*SPTSSB*	48	18	31	42	21	-1.000	0.656387797	1
ENSBTAG00000047254	*ABCA2*	2'467	3'641	3'170	2'136	4'204	0.977	0.213436774	1
ENSBTAG00000047255	*protein_coding*	8	1	4	7	1	-2.585	0.818599781	1
ENSBTAG00000047256	*FMO6P*	8	5	6	7	6	-0.263	0.996869191	1
ENSBTAG00000047260	*protein_coding*	82	32	54	71	37	-0.943	0.578950579	1
ENSBTAG00000047262	*protein_coding*	64	59	62	55	68	0.298	0.857622926	1
ENSBTAG00000047264	*protein_coding*	9	0	4	8	0		0.690676647	1
ENSBTAG00000047265	*protein_coding*	1'030	893	962	892	1'031	0.209	0.800523855	1
ENSBTAG00000047266	*protein_coding*	154	224	196	133	259	0.956	0.370457308	1
ENSBTAG00000047268	*WT1*	2	0	1	2	0		0.974934741	1
ENSBTAG00000047270	*protein_coding*	1	0	0	1	0		1	1
ENSBTAG00000047273	*5S_rRNA*	1	0	0	1	0		1	1
ENSBTAG00000047274	*KIF21B*	981	947	972	850	1'094	0.364	0.659441915	1
ENSBTAG00000047275	*pseudogene*	2	0	1	2	0		0.974934741	1
ENSBTAG00000047277	*PNKD*	307	407	368	266	470	0.822	0.37825921	1
ENSBTAG00000047278	*NDUFB11*	3'209	2'344	2'743	2'779	2'707	-0.038	0.962335395	1
ENSBTAG00000047281	*C15ORF62*	90	103	98	78	119	0.610	0.644536266	1
ENSBTAG00000047282	*pseudogene*	8	1	4	7	1	-2.585	0.818599781	1
ENSBTAG00000047284	*DEFB4A*	166	216	197	144	249	0.795	0.455333236	1
ENSBTAG00000047287	*Mrz 01*	132	74	100	114	85	-0.420	0.75344639	1
ENSBTAG00000047290	*protein_coding*	1	0	0	1	0		1	1
ENSBTAG00000047293	*NRIP1*	436	301	363	378	348	-0.120	0.901523356	1
ENSBTAG00000047299	*BT.21248*	1'704	1'837	1'798	1'476	2'121	0.523	0.510766637	1
ENSBTAG00000047301	*protein_coding*	4	1	2	3	1	-1.585	0.952247636	1
ENSBTAG00000047302	*protein_coding*	3	2	2	3	2	-0.170	1	1
ENSBTAG00000047303	*BT.63389*	2	0	1	2	0		0.974934741	1
ENSBTAG00000047305	*protein_coding*	4	10	8	3	12	1.737	0.768040958	1
ENSBTAG00000047309	*protein_coding*	2	0	1	2	0		0.974934741	1
ENSBTAG00000047314	*BT.21742*	1'806	974	1'344	1'564	1'125	-0.476	0.557134933	1
ENSBTAG00000047315	*PTF1A*	0	2	1	0	2	Inf	0.939077559	1
ENSBTAG00000047316	*BT.106075*	55	35	44	48	40	-0.237	0.910211497	1
ENSBTAG00000047319	*BT.65221*	3'205	2'033	2'562	2'776	2'348	-0.242	0.758916206	1
ENSBTAG00000047320	*UBE2D3*	109	76	91	94	88	-0.105	0.947375009	1
ENSBTAG00000047321	*pseudogene*	4	0	2	3	0		0.89545886	1
ENSBTAG00000047322	*PTTG1*	120	248	195	104	286	1.462	0.174929197	1
ENSBTAG00000047323	*BT.6202*	56	43	49	48	50	0.034	0.997313264	1
ENSBTAG00000047324	*protein_coding*	0	1	1	0	1	Inf	0.993540919	1
ENSBTAG00000047325	*GNGT2*	45	21	32	39	24	-0.684	0.763007177	1
ENSBTAG00000047326	*EDA2R*	1	3	2	1	3	2.000	0.933774563	1
ENSBTAG00000047330	*FABP5*	7'547	2'631	4'787	6'536	3'038	-1.105	0.15657184	1
ENSBTAG00000047331	*GYPB*	21	6	13	18	7	-1.392	0.729267326	1
ENSBTAG00000047338	*DCBLD1*	385	177	269	333	204	-0.706	0.476894425	1
ENSBTAG00000047339	*BCOR*	2'792	1'971	2'347	2'418	2'276	-0.087	0.912781123	1
ENSBTAG00000047340	*protein_coding*	10	2	5	9	2	-1.907	0.815196829	1
ENSBTAG00000047341	*protein_coding*	132	166	153	114	192	0.746	0.513214615	1
ENSBTAG00000047342	*protein_coding*	69	62	66	60	72	0.261	0.872740131	1
ENSBTAG00000047343	*pseudogene*	594	738	683	514	852	0.728	0.394945009	1
ENSBTAG00000047344	*BT.88090*	8	3	5	7	3	-1.000	0.918861955	1
ENSBTAG00000047345	*protein_coding*	314	439	389	272	507	0.898	0.331258916	1
ENSBTAG00000047347	*RAD51AP1*	121	108	115	105	125	0.251	0.844827416	1
ENSBTAG00000047349	*BT.30011*	2	0	1	2	0		0.974934741	1
ENSBTAG00000047354	*pseudogene*	1'965	2'491	2'289	1'702	2'876	0.757	0.337962865	1
ENSBTAG00000047355	*protein_coding*	1	1	1	1	1	0.415	1	1
ENSBTAG00000047356	*protein_coding*	348	240	289	301	277	-0.121	0.905137725	1
ENSBTAG00000047357	*RNF208*	90	94	93	78	109	0.478	0.724491433	1
ENSBTAG00000047361	*pseudogene*	5'250	4'789	5'038	4'547	5'530	0.282	0.713790769	1
ENSBTAG00000047362	*BT.96665*	37'407	18'045	26'616	32'395	20'837	-0.637	0.404227043	1
ENSBTAG00000047363	*protein_coding*	38	5	19	33	6	-2.511	0.40921164	1
ENSBTAG00000047364	*processed_pseudogene*	199	423	330	172	488	1.503	0.118514958	1
ENSBTAG00000047365	*pseudogene*	545	352	439	472	406	-0.216	0.813988986	1
ENSBTAG00000047366	*protein_coding*	4	3	3	3	3	0.000	1	1
ENSBTAG00000047367	*FTSJD1*	960	700	820	831	808	-0.041	0.963639421	1
ENSBTAG00000047370	*bta-mir-2887-2*	483	220	336	418	254	-0.719	0.448669579	1
ENSBTAG00000047374	*SLC26A10*	260	232	247	225	268	0.251	0.805336477	1
ENSBTAG00000047375	*protein_coding*	10	3	6	9	3	-1.322	0.863266279	1
ENSBTAG00000047376	*PIN4*	836	556	683	724	642	-0.173	0.84115556	1
ENSBTAG00000047378	*miRNA*	1	0	0	1	0		1	1
ENSBTAG00000047379	*BT.88669*	142	96	117	123	111	-0.150	0.91121715	1
ENSBTAG00000047380	*CT*	31	11	20	27	13	-1.080	0.714986343	1

Ensembl gene ID	geneName	counts wildtype horn bud	counts polled horn bud	baseMean	baseMean wildtype horn bud	baseMean polled horn bud	log2FoldChange	pval	padj
ENSBTAG00000047382	*miRNA*	1	0	0	1	0		1	1
ENSBTAG00000047383	*protein_coding*	22	3	11	19	3	-2.459	0.576368753	1
ENSBTAG00000047389	*BT.88916*	494	223	343	428	257	-0.732	0.43897344	1
ENSBTAG00000047391	*processed_pseudogene*	0	4	2	0	5	Inf	0.817359038	1
ENSBTAG00000047400	*IL11*	17	12	14	15	14	-0.087	1	1
ENSBTAG00000047401	*5_8S_rRNA*	1'034	348	649	895	402	-1.156	0.182849108	1
ENSBTAG00000047402	*FAM170B*	7	4	5	6	5	-0.392	0.98714753	1
ENSBTAG00000047404	*protein_coding*	4	1	2	3	1	-1.585	0.952247636	1
ENSBTAG00000047405	*protein_coding*	260	153	201	225	177	-0.350	0.744053619	1
ENSBTAG00000047406	*protein_coding*	3'071	1'049	1'935	2'660	1'211	-1.135	0.156786408	1
ENSBTAG00000047409	*processed_pseudogene*	1	0	0	1	0		1	1
ENSBTAG00000047410	*CXORF59*	2	0	1	2	0		0.974934741	1
ENSBTAG00000047411	*protein_coding*	2	1	1	2	1	-0.585	1	1
ENSBTAG00000047412	*TNFSF18*	187	56	113	162	65	-1.325	0.295559577	1
ENSBTAG00000047413	*BT.87655*	1	0	0	1	0		1	1
ENSBTAG00000047414	*protein_coding*	0	1	1	0	1	Inf	0.993540919	1
ENSBTAG00000047416	*HEPH*	316	175	238	274	202	-0.438	0.668985271	1
ENSBTAG00000047418	*SLC25A6*	30'440	51'644	42'998	26'362	59'633	1.178	0.125296847	1
ENSBTAG00000047420	*BT.96681*	190	74	125	165	85	-0.945	0.438446635	1
ENSBTAG00000047424	*TRIP10*	1'161	1'082	1'127	1'005	1'249	0.313	0.701278141	1
ENSBTAG00000047425	*protein_coding*	4'651	10'404	8'021	4'028	12'014	1.577	0.043971035	1
ENSBTAG00000047426	*LYPD6*	96	75	85	83	87	0.059	0.974748949	1
ENSBTAG00000047428	*ZNF281*	679	411	531	588	475	-0.309	0.727150931	1
ENSBTAG00000047431	*SNORA42*	10	6	8	9	7	-0.322	0.979718036	1
ENSBTAG00000047432	*processed_pseudogene*	1'009	2'204	1'709	874	2'545	1.542	0.057816418	1
ENSBTAG00000047434	*BT.42177*	1'751	848	1'248	1'516	979	-0.631	0.438598915	1
ENSBTAG00000047436	*protein_coding*	4	1	2	3	1	-1.585	0.952247636	1
ENSBTAG00000047441	*processed_pseudogene*	4'934	4'707	4'854	4'273	5'435	0.347	0.652622182	1
ENSBTAG00000047442	*protein_coding*	4	1	2	3	1	-1.585	0.952247636	1
ENSBTAG00000047443	*BT.104862*	3	0	1	3	0		0.936647693	1
ENSBTAG00000047446	*miRNA*	1	1	1	1	1	0.415	1	1
ENSBTAG00000047448	*C25H16orf54*	35	36	36	30	42	0.456	0.831553697	1
ENSBTAG00000047449	*GNLY*	49	15	30	42	17	-1.293	0.572764807	1
ENSBTAG00000047450	*SFR1*	914	539	707	792	622	-0.347	0.685032127	1
ENSBTAG00000047457	*protein_coding*	3	0	1	3	0		0.936647693	1
ENSBTAG00000047459	*protein_coding*	8	16	13	7	18	1.415	0.716950431	1
ENSBTAG00000047461	*DYNC2H1*	1'000	495	719	866	572	-0.599	0.48201545	1
ENSBTAG00000047462	*protein_coding*	356	461	420	308	532	0.788	0.387811952	1
ENSBTAG00000047467	*LYPLA2*	265	376	332	229	434	0.920	0.333466053	1
ENSBTAG00000047470	*U3*	3	4	4	3	5	0.830	0.962490925	1
ENSBTAG00000047471	*protein_coding*	1'122	1'472	1'336	972	1'700	0.807	0.320047902	1
ENSBTAG00000047473	*protein_coding*	6	0	3	5	0		0.81115604	1
ENSBTAG00000047474	*SPRN*	37	14	24	32	16	-0.987	0.706068506	1
ENSBTAG00000047478	*PCDHB6*	77	72	75	67	83	0.318	0.832988896	1
ENSBTAG00000047481	*bta-mir-2904-1*	21	0	9	18	0		0.346730876	1
ENSBTAG00000047482	*PRRT4*	28	11	18	24	13	-0.933	0.765131313	1
ENSBTAG00000047483	*protein_coding*	59	81	72	51	94	0.872	0.556845763	1
ENSBTAG00000047484	*HRK*	1	1	1	1	1	0.415	1	1
ENSBTAG00000047486	*CARTPT*	6	0	3	5	0		0.81115604	1
ENSBTAG00000047488	*KCNK9*	1	0	0	1	0		1	1
ENSBTAG00000047490	*protein_coding*	4	0	2	3	0		0.89545886	1
ENSBTAG00000047494	*miRNA*	37	12	23	32	14	-1.209	0.651630884	1
ENSBTAG00000047495	*BT.51424*	18'471	25'177	22'534	15'996	29'072	0.862	0.259969395	1
ENSBTAG00000047496	*protein_coding*	117	199	166	101	230	1.181	0.291376203	1
ENSBTAG00000047499	*IRX2*	2'396	2'335	2'386	2'075	2'696	0.378	0.630654631	1
ENSBTAG00000047501	*bta-mir-2887-2*	499	224	345	432	259	-0.741	0.433299494	1
ENSBTAG00000047504	*U1*	9	2	5	8	2	-1.755	0.844314416	1
ENSBTAG00000047505	*NANOS2*	1	0	0	1	0		1	1
ENSBTAG00000047507	*protein_coding*	1	1	1	1	1	0.415	1	1
ENSBTAG00000047508	*ZNF580*	151	129	140	131	149	0.188	0.876750115	1
ENSBTAG00000047509	*KIAA1257*	2	1	1	2	1	-0.585	1	1
ENSBTAG00000047510	*SMIM6*	4	1	2	3	1	-1.585	0.952247636	1
ENSBTAG00000047511	*protein_coding*	147	330	254	127	381	1.582	0.119647755	1
ENSBTAG00000047514	*SLC10A6*	11	5	8	10	6	-0.722	0.917926652	1
ENSBTAG00000047515	*KRTAP3-3*	2	0	1	2	0		0.974934741	1
ENSBTAG00000047518	*protein_coding*	40	42	42	35	48	0.485	0.804855476	1
ENSBTAG00000047520	*MPV17L*	19	6	12	16	7	-1.248	0.770493406	1
ENSBTAG00000047524	*TMEM200B*	112	85	98	97	98	0.017	0.997974141	1
ENSBTAG00000047525	*miRNA*	2	11	7	2	13	2.874	0.64186083	1
ENSBTAG00000047526	*GSC2*	8	0	3	7	0		0.72953145	1
ENSBTAG00000047528	*C2CD4D*	8	5	6	7	6	-0.263	0.996869191	1
ENSBTAG00000047531	*BT.96719*	264	194	226	229	224	-0.029	0.982827951	1

Ensembl gene ID	geneName	counts wildtype horn bud	counts polled horn bud	baseMean	baseMean wildtype horn bud	baseMean polled horn bud	log2FoldChange	pval	padj
ENSBTAG00000047532	*C8ORF83*	471	341	401	408	394	-0.051	0.959408586	1
ENSBTAG00000047534	*BT.37863*	24	26	25	21	30	0.531	0.839166484	1
ENSBTAG00000047536	*PRRG1*	112	118	117	97	136	0.490	0.694509483	1
ENSBTAG00000047537	*BT.65444*	6'748	1'869	4'001	5'844	2'158	-1.437	0.068363571	1
ENSBTAG00000047538	*BT.40996*	14	10	12	12	12	-0.070	1	1
ENSBTAG00000047540	*miRNA*	1	0	0	1	0		1	1
ENSBTAG00000047543	*NTRK3*	200	355	292	173	410	1.243	0.204749556	1
ENSBTAG00000047545	*RGAG4*	1'175	1'626	1'448	1'018	1'878	0.884	0.274382692	1
ENSBTAG00000047550	*KIAA2026*	1'637	753	1'144	1'418	869	-0.705	0.389677369	1
ENSBTAG00000047551	*BT.49252*	2	14	9	2	16	3.222	0.540921467	1
ENSBTAG00000047559	*HIST1H2BL*	1	0	0	1	0		1	1
ENSBTAG00000047560	*C2CD4C*	8	10	9	7	12	0.737	0.893887116	1
ENSBTAG00000047561	*protein_coding*	563	275	403	488	318	-0.619	0.501423562	1
ENSBTAG00000047563	*CLDN9*	0	3	2	0	3	Inf	0.878646179	1
ENSBTAG00000047567	*VEGFB*	2'645	2'618	2'657	2'291	3'023	0.400	0.609278879	1
ENSBTAG00000047568	*VN1R1*	17	11	14	15	13	-0.213	0.976116423	1
ENSBTAG00000047570	*protein_coding*	13	5	9	11	6	-0.963	0.868297704	1
ENSBTAG00000047572	*BT.72504*	169	97	129	146	112	-0.386	0.752110818	1
ENSBTAG00000047575	*protein_coding*	629	582	608	545	672	0.303	0.726523316	1
ENSBTAG00000047579	*processed_pseudogene*	3	1	2	3	1	-1.170	0.985253077	1
ENSBTAG00000047580	*protein_coding*	1	0	0	1	0		1	1
ENSBTAG00000047582	*CSNK1E*	601	670	647	520	774	0.572	0.506070351	1
ENSBTAG00000047584	*U1*	1	0	0	1	0		1	1
ENSBTAG00000047585	*protein_coding*	2	2	2	2	2	0.415	1	1
ENSBTAG00000047586	*NPY1R*	115	114	116	100	132	0.402	0.749002063	1
ENSBTAG00000047587	*protein_coding*	87	109	101	75	126	0.740	0.571480885	1
ENSBTAG00000047590	*SNORA63*	10	1	5	9	1	-2.907	0.754028015	1
ENSBTAG00000047591	*BT.29619*	276	281	282	239	324	0.441	0.652884149	1
ENSBTAG00000047592	*pseudogene*	6	2	4	5	2	-1.170	0.934316631	1
ENSBTAG00000047594	*CCNO*	15	16	16	13	18	0.508	0.891898205	1
ENSBTAG00000047595	*FSTL5*	101	4	46	87	5	-4.243	0.041945544	1
ENSBTAG00000047596	*C2ORF53*	15	1	7	13	1	-3.492	0.607205542	1
ENSBTAG00000047598	*NPR2*	4'527	3'106	3'753	3'920	3'586	-0.128	0.869429976	1
ENSBTAG00000047600	*ROS1*	3	1	2	3	1	-1.170	0.985253077	1
ENSBTAG00000047601	*MGC140151*	100	4	46	87	5	-4.229	0.043388663	1
ENSBTAG00000047602	*protein_coding*	9	3	6	8	3	-1.170	0.890813994	1
ENSBTAG00000047604	*HSD17B1*	119	87	102	103	100	-0.037	0.98696433	1
ENSBTAG00000047605	*protein_coding*	42	20	30	36	23	-0.655	0.781354987	1
ENSBTAG00000047606	*MTA1*	5'976	6'685	6'447	5'175	7'719	0.577	0.453533166	1
ENSBTAG00000047608	*MRPS22*	1'042	860	948	902	993	0.138	0.867892443	1
ENSBTAG00000047609	*CIDEA*	50	22	34	43	25	-0.769	0.72051121	1
ENSBTAG00000047611	*protein_coding*	33	72	56	29	83	1.541	0.355282107	1
ENSBTAG00000047616	*ZNF114*	11	0	5	10	0		0.617662983	1
ENSBTAG00000047618	*miRNA*	2	0	1	2	0		0.974934741	1
ENSBTAG00000047620	*miRNA*	3	0	1	3	0		0.936647693	1
ENSBTAG00000047621	*BT.30402*	104	142	127	90	164	0.864	0.474236309	1
ENSBTAG00000047625	*protein_coding*	31	101	72	27	117	2.119	0.164275356	1
ENSBTAG00000047626	*protein_coding*	153	54	97	133	62	-1.087	0.413829143	1
ENSBTAG00000047627	*miRNA*	1	0	0	1	0		1	1
ENSBTAG00000047628	*XPO7*	3'950	3'114	3'508	3'421	3'596	0.072	0.925887856	1
ENSBTAG00000047633	*MAML1*	714	790	765	618	912	0.561	0.506704063	1
ENSBTAG00000047635	*BT.31951*	53	18	33	46	21	-1.143	0.596664274	1
ENSBTAG00000047636	*pseudogene*	1	0	0	1	0		1	1
ENSBTAG00000047637	*processed_pseudogene*	2'113	1'844	1'980	1'830	2'129	0.219	0.782284383	1
ENSBTAG00000047638	*protein_coding*	41	26	33	36	30	-0.242	0.924725965	1
ENSBTAG00000047640	*bta-mir-3956*	1	2	2	1	2	1.415	0.981979269	1
ENSBTAG00000047642	*BT.85102*	9	6	7	8	7	-0.170	1	1
ENSBTAG00000047643	*BT.103343*	125	97	110	108	112	0.049	0.976156496	1
ENSBTAG00000047645	*protein_coding*	3	0	1	3	0		0.936647693	1
ENSBTAG00000047647	*protein_coding*	4	6	5	3	7	1.000	0.91481797	1
ENSBTAG00000047648	*SYT2*	194	83	132	168	96	-0.810	0.49935579	1
ENSBTAG00000047649	*AUH*	149	101	123	129	117	-0.146	0.911928382	1
ENSBTAG00000047650	*NXPH4*	71	48	58	61	55	-0.150	0.937965657	1
ENSBTAG00000047651	*U1*	1	0	0	1	0		1	1
ENSBTAG00000047653	*protein_coding*	96	71	83	83	82	-0.020	0.999649572	1
ENSBTAG00000047654	*CMTM3*	3'092	4'396	3'877	2'678	5'076	0.923	0.237051364	1
ENSBTAG00000047658	*PHF7*	69	37	51	60	43	-0.484	0.786188552	1
ENSBTAG00000047661	*FABP12*	4	0	2	3	0		0.89545886	1
ENSBTAG00000047663	*bta-mir-2887-2*	609	291	432	527	336	-0.650	0.474879378	1
ENSBTAG00000047664	*GNAZ*	119	121	121	103	140	0.439	0.722007041	1
ENSBTAG00000047665	*BT.88853*	1	0	0	1	0		1	1
ENSBTAG00000047667	*protein_coding*	0	2	1	0	2	Inf	0.939077559	1

Ensembl gene ID	geneName	counts wildtype horn bud	counts polled horn bud	baseMean	baseMean wildtype horn bud	baseMean polled horn bud	log2FoldChange	pval	padj
ENSBTAG00000047670	*CHST8*	47	93	74	41	107	1.400	0.343000248	1
ENSBTAG00000047672	*protein_coding*	0	3	2	0	3	Inf	0.878646179	1
ENSBTAG00000047673	*THUMPD1*	593	713	668	514	823	0.681	0.427224025	1
ENSBTAG00000047675	*SIGLEC6*	74	41	56	64	47	-0.437	0.799777174	1
ENSBTAG00000047676	*BT.20369*	291	402	358	252	464	0.881	0.347353083	1
ENSBTAG00000047677	*miRNA*	0	3	2	0	3	Inf	0.878646179	1
ENSBTAG00000047678	*miRNA*	1	0	0	1	0		1	1
ENSBTAG00000047679	*DNTTIP2*	2'074	1'483	1'754	1'796	1'712	-0.069	0.932482809	1
ENSBTAG00000047680	*BT.96672*	394	382	391	341	441	0.370	0.688012175	1
ENSBTAG00000047684	*protein_coding*	1	0	0	1	0		1	1
ENSBTAG00000047685	*bta-mir-2416*	50	159	113	43	184	2.084	0.105162625	1
ENSBTAG00000047686	*miRNA*	2	7	5	2	8	2.222	0.797845137	1
ENSBTAG00000047689	*protein_coding*	0	2	1	0	2	Inf	0.939077559	1
ENSBTAG00000047694	*SERF2*	7'027	8'802	8'125	6'086	10'164	0.740	0.335943457	1
ENSBTAG00000047697	*protein_coding*	156	233	202	135	269	0.994	0.348150223	1
ENSBTAG00000047698	*BT.106625*	1	0	0	1	0		1	1
ENSBTAG00000047702	*NAA10*	1'038	1'093	1'081	899	1'262	0.490	0.550517293	1
ENSBTAG00000047703	*pseudogene*	11	4	7	10	5	-1.044	0.879025484	1
ENSBTAG00000047706	*ING2*	753	707	734	652	816	0.324	0.702326301	1
ENSBTAG00000047707	*BOLA-DOB*	40	8	22	35	9	-1.907	0.489943449	1
ENSBTAG00000047708	*BT.64579*	598	769	703	518	888	0.778	0.362345086	1
ENSBTAG00000047713	*C11ORF35*	17	12	14	15	14	-0.087	1	1
ENSBTAG00000047715	*TPRN*	598	915	787	518	1'057	1.029	0.22492058	1
ENSBTAG00000047717	*BT.657*	401	482	452	347	557	0.680	0.45037052	1
ENSBTAG00000047718	*ZNF70*	231	322	286	200	372	0.894	0.36071002	1
ENSBTAG00000047719	*DRD1*	117	22	63	101	25	-1.996	0.21454715	1
ENSBTAG00000047720	*U6*	1	1	1	1	1	0.415	1	1
ENSBTAG00000047722	*BT.64391*	1	0	0	1	0		1	1
ENSBTAG00000047727	*NUAK2*	177	115	143	153	133	-0.207	0.864383663	1
ENSBTAG00000047729	*protein_coding*	2	1	1	2	1	-0.585	1	1
ENSBTAG00000047731	*FAM189B*	1'027	1'326	1'210	889	1'531	0.784	0.336909375	1
ENSBTAG00000047734	*C16ORF58*	1'404	1'429	1'433	1'216	1'650	0.441	0.584148633	1
ENSBTAG00000047736	*protein_coding*	1	2	2	1	2	1.415	0.981979269	1
ENSBTAG00000047739	*TSC22D1*	11'432	4'431	7'508	9'900	5'116	-0.952	0.217924631	1
ENSBTAG00000047740	*protein_coding*	4	0	2	3	0		0.89545886	1
ENSBTAG00000047743	*KCNIP4*	34	3	16	29	3	-3.087	0.373510501	1
ENSBTAG00000047744	*pseudogene*	1'380	2'835	2'234	1'195	3'274	1.454	0.069877319	1
ENSBTAG00000047745	*protein_coding*	0	1	1	0	1	Inf	0.993540919	1
ENSBTAG00000047747	*NDFIP1*	8'505	4'065	6'030	7'366	4'694	-0.650	0.399922016	1
ENSBTAG00000047749	*BT.91898*	27	15	20	23	17	-0.433	0.893286441	1
ENSBTAG00000047752	*OTUD1*	268	125	188	232	144	-0.685	0.526176255	1
ENSBTAG00000047754	*bta-mir-2904-3*	13	0	6	11	0		0.551229012	1
ENSBTAG00000047755	*C1orf233*	153	160	159	133	185	0.480	0.67176186	1
ENSBTAG00000047756	*CNTROB*	2'079	1'643	1'849	1'800	1'897	0.075	0.924254393	1
ENSBTAG00000047757	*protein_coding*	26	103	71	23	119	2.401	0.120892061	1
ENSBTAG00000047760	*BT.41320*	1'707	1'848	1'806	1'478	2'134	0.530	0.505814429	1
ENSBTAG00000047761	*protein_coding*	80	40	58	69	46	-0.585	0.725701763	1
ENSBTAG00000047764	*BT.63582*	3	10	7	3	12	2.152	0.727540088	1
ENSBTAG00000047765	*pseudogene*	32	33	33	28	38	0.459	0.838567134	1
ENSBTAG00000047766	*G0S2*	311	212	257	269	245	-0.138	0.894457274	1
ENSBTAG00000047767	*MUC3A*	4	4	4	3	5	0.415	0.994759363	1
ENSBTAG00000047768	*COX7B*	272	325	305	236	375	0.672	0.485752435	1
ENSBTAG00000047771	*protein_coding*	1	0	0	1	0		1	1
ENSBTAG00000047772	*FBXO48*	48	19	32	42	22	-0.922	0.679327723	1
ENSBTAG00000047774	*AMGY*	0	4	2	0	5	Inf	0.817359038	1
ENSBTAG00000047775	*U6*	0	1	1	0	1	Inf	0.993540919	1
ENSBTAG00000047776	*protein_coding*	46	6	23	40	7	-2.524	0.352195564	1
ENSBTAG00000047777	*protein_coding*	3	0	1	3	0		0.936647693	1
ENSBTAG00000047780	*CCDC127*	1'459	827	1'109	1'264	955	-0.404	0.622656322	1
ENSBTAG00000047781	*BT.103428*	1	1	1	1	1	0.415	1	1
ENSBTAG00000047783	*protein_coding*	44	71	60	38	82	1.105	0.491260044	1
ENSBTAG00000047785	*miRNA*	2	1	1	2	1	-0.585	1	1
ENSBTAG00000047786	*LRRC10B*	37	66	54	32	76	1.250	0.457701866	1
ENSBTAG00000047787	*RPS15A*	6'942	4'275	5'474	6'012	4'936	-0.284	0.712750987	1
ENSBTAG00000047788	*protein_coding*	6	1	3	5	1	-2.170	0.885240772	1
ENSBTAG00000047789	*Metazoa_SRP*	13	32	24	11	37	1.715	0.504274581	1
ENSBTAG00000047790	*bta-mir-2887-1*	670	274	448	580	316	-0.875	0.334421636	1
ENSBTAG00000047791	*BOSTAUV1R421*	0	1	1	0	1	Inf	0.993540919	1
ENSBTAG00000047793	*TSSC4*	552	701	644	478	809	0.760	0.378046692	1
ENSBTAG00000047794	*DNAH1*	195	334	277	169	386	1.191	0.228354183	1
ENSBTAG00000047795	*miRNA*	21	94	63	18	109	2.577	0.114079669	1
ENSBTAG00000047797	*miRNA*	5	0	2	4	0		0.853268594	1

Ensembl gene ID	geneName	counts wildtype horn bud	counts polled horn bud	baseMean	baseMean wildtype horn bud	baseMean polled horn bud	log2FoldChange	pval	padj
ENSBTAG00000047801	*CRELD2*	948	1'226	1'118	821	1'416	0.786	0.338066119	1
ENSBTAG00000047805	*protein_coding*	22	36	30	19	42	1.126	0.617166584	1
ENSBTAG00000047806	*RPE*	319	251	283	276	290	0.069	0.946576893	1
ENSBTAG00000047807	*C9ORF140*	114	166	145	99	192	0.957	0.409030944	1
ENSBTAG00000047809	*HDHD2*	223	252	242	193	291	0.591	0.559058103	1
ENSBTAG00000047810	*BT.102766*	12	0	5	10	0		0.583618942	1
ENSBTAG00000047815	*protein_coding*	20	17	18	17	20	0.181	0.970317963	1
ENSBTAG00000047816	*protein_coding*	6	11	9	5	13	1.290	0.803227255	1
ENSBTAG00000047818	*FAM19A2*	67	77	73	58	89	0.616	0.677887512	1
ENSBTAG00000047820	*miRNA*	0	1	1	0	1	Inf	0.993540919	1
ENSBTAG00000047821	*protein_coding*	5	0	2	4	0		0.853268594	1
ENSBTAG00000047822	*protein_coding*	59	31	43	51	36	-0.513	0.790964638	1
ENSBTAG00000047823	*pseudogene*	30	0	13	26	0		0.205783873	1
ENSBTAG00000047824	*pseudogene*	2'376	2'018	2'194	2'058	2'330	0.179	0.819750262	1
ENSBTAG00000047827	*RENBP*	656	761	723	568	879	0.629	0.45917452	1
ENSBTAG00000047828	*BT.102184*	265	163	209	229	188	-0.286	0.788443194	1
ENSBTAG00000047830	*protein_coding*	4	2	3	3	2	-0.585	0.996239345	1
ENSBTAG00000047834	*BT.65438*	2'093	1'168	1'581	1'813	1'349	-0.426	0.595305974	1
ENSBTAG00000047836	*protein_coding*	174	277	235	151	320	1.086	0.288450717	1
ENSBTAG00000047837	*BT.30186*	1'271	769	994	1'101	888	-0.310	0.708469708	1
ENSBTAG00000047838	*bta-mir-2887-2*	3	11	8	3	13	2.290	0.692284022	1
ENSBTAG00000047839	*P2RY8*	1	2	2	1	2	1.415	0.981979269	1
ENSBTAG00000047845	*protein_coding*	2'590	2'429	2'524	2'243	2'805	0.322	0.680844038	1
ENSBTAG00000047847	*protein_coding*	591	464	524	512	536	0.066	0.941815816	1
ENSBTAG00000047850	*protein_coding*	1	2	2	1	2	1.415	0.981979269	1
ENSBTAG00000047855	*MECP2*	552	663	622	478	766	0.679	0.43199394	1
ENSBTAG00000047856	*PAK2*	1'204	985	1'090	1'043	1'137	0.125	0.878573141	1
ENSBTAG00000047858	*protein_coding*	6	2	4	5	2	-1.170	0.934316631	1
ENSBTAG00000047861	*protein_coding*	7	2	4	6	2	-1.392	0.903936064	1
ENSBTAG00000047862	*protein_coding*	142	97	117	123	112	-0.135	0.920791955	1
ENSBTAG00000047863	*pseudogene*	2	1	1	2	1	-0.585	1	1
ENSBTAG00000047865	*SNORD107*	118	72	93	102	83	-0.298	0.831616465	1
ENSBTAG00000047866	*BT.62430*	0	3	2	0	3	Inf	0.878646179	1
ENSBTAG00000047868	*BT.65683*	138	130	135	120	150	0.329	0.784018163	1
ENSBTAG00000047869	*protein_coding*	4	15	10	3	17	2.322	0.608798021	1
ENSBTAG00000047870	*OR4D2*	1	1	1	1	1	0.415	1	1
ENSBTAG00000047871	*BT.104733*	2	0	1	2	0		0.974934741	1
ENSBTAG00000047872	*KRT84*	9	2	5	8	2	-1.755	0.844314416	1
ENSBTAG00000047873	*protein_coding*	7	10	9	6	12	0.930	0.865678914	1
ENSBTAG00000047874	*PRPF38B*	5'417	2'860	3'997	4'691	3'302	-0.506	0.514624715	1
ENSBTAG00000047875	*miRNA*	3'837	926	2'196	3'323	1'069	-1.636	0.042537659	1
ENSBTAG00000047876	*SH3TC1*	233	164	196	202	189	-0.092	0.937139972	1
ENSBTAG00000047877	*TRIM67*	28	1	13	24	1	-4.392	0.334236041	1
ENSBTAG00000047879	*BT.87277*	39	11	23	34	13	-1.411	0.594828841	1
ENSBTAG00000047880	*protein_coding*	461	134	277	399	155	-1.367	0.16941543	1
ENSBTAG00000047881	*BT.87943*	4	8	6	3	9	1.415	0.839396723	1
ENSBTAG00000047882	*U6*	1	1	1	1	1	0.415	1	1
ENSBTAG00000047883	*BT.103970*	464	220	328	402	254	-0.662	0.487961381	1
ENSBTAG00000047884	*pseudogene*	2	3	3	2	3	1.000	0.97171833	1
ENSBTAG00000047885	*ACTL10*	4	14	10	3	16	2.222	0.638389235	1
ENSBTAG00000047889	*miRNA*	56	43	49	48	50	0.034	0.997313264	1
ENSBTAG00000047891	*protein_coding*	3	7	5	3	8	1.637	0.840440358	1
ENSBTAG00000047893	*protein_coding*	4	18	12	3	21	2.585	0.526766351	1
ENSBTAG00000047894	*SLC32A1*	56	0	24	48	0		0.048297215	1
ENSBTAG00000047896	*protein_coding*	236	249	246	204	288	0.492	0.625647823	1
ENSBTAG00000047899	*pseudogene*	1	4	3	1	5	2.415	0.884888347	1
ENSBTAG00000047900	*BT.71247*	1	0	0	1	0		1	1
ENSBTAG00000047901	*miRNA*	2	3	3	2	3	1.000	0.97171833	1
ENSBTAG00000047902	*BT.104971*	54	13	31	47	15	-1.639	0.467492948	1
ENSBTAG00000047903	*SNORA5*	0	1	1	0	1	Inf	0.993540919	1
ENSBTAG00000047904	*protein_coding*	60	79	72	52	91	0.812	0.586222918	1
ENSBTAG00000047906	*MRPL36*	873	855	872	756	987	0.385	0.644371717	1
ENSBTAG00000047907	*protein_coding*	0	1	1	0	1	Inf	0.993540919	1
ENSBTAG00000047908	*Metazoa_SRP*	98	92	96	85	106	0.324	0.811858319	1
ENSBTAG00000047910	*protein_coding*	1	0	0	1	0		1	1
ENSBTAG00000047912	*protein_coding*	0	1	1	0	1	Inf	0.993540919	1
ENSBTAG00000047914	*BT.97581*	15	6	10	13	7	-0.907	0.858439879	1
ENSBTAG00000047915	*miRNA*	0	1	1	0	1	Inf	0.993540919	1
ENSBTAG00000047917	*protein_coding*	0	1	1	0	1	Inf	0.993540919	1
ENSBTAG00000047918	*bta-mir-2887-2*	359	140	236	311	162	-0.944	0.35633788	1
ENSBTAG00000047924	*MAB21L2*	1	1	1	1	1	0.415	1	1
ENSBTAG00000047925	*pseudogene*	564	1'008	826	488	1'164	1.253	0.139420952	1

Ensembl gene ID	geneName	counts wildtype horn bud	counts polled horn bud	baseMean	baseMean wildtype horn bud	baseMean polled horn bud	log2FoldChange	pval	padj
ENSBTAG00000047926	*OVCA2*	20	30	26	17	35	1.000	0.685166323	1
ENSBTAG00000047928	*miRNA*	1	0	0	1	0		1	1
ENSBTAG00000047930	*UBE2E1*	942	911	934	816	1'052	0.367	0.65820481	1
ENSBTAG00000047932	*protein_coding*	2	1	1	2	1	-0.585	1	1
ENSBTAG00000047933	*CYP4F22*	51	19	33	44	22	-1.009	0.642583639	1
ENSBTAG00000047935	*protein_coding*	1	7	4	1	8	3.222	0.741805782	1
ENSBTAG00000047936	*processed_pseudogene*	212	375	308	184	433	1.238	0.201611244	1
ENSBTAG00000047937	*protein_coding*	14	10	12	12	12	-0.070	1	1
ENSBTAG00000047939	*protein_coding*	39	11	23	34	13	-1.411	0.594828841	1
ENSBTAG00000047940	*processed_pseudogene*	18	96	63	16	111	2.830	0.086401115	1
ENSBTAG00000047942	*PPPDE2*	343	483	427	297	558	0.909	0.318666162	1
ENSBTAG00000047943	*protein_coding*	1	0	0	1	0		1	1
ENSBTAG00000047946	*protein_coding*	12'642	7'276	9'675	10'948	8'402	-0.382	0.618355923	1
ENSBTAG00000047947	*Metazoa_SRP*	119	119	120	103	137	0.415	0.737800417	1
ENSBTAG00000047948	*pseudogene*	1	0	0	1	0		1	1
ENSBTAG00000047951	*U1*	1	0	0	1	0		1	1
ENSBTAG00000047954	*BT.66539*	1'152	812	968	998	938	-0.090	0.915625742	1
ENSBTAG00000047955	*ZNF774*	92	76	84	80	88	0.139	0.927983452	1
ENSBTAG00000047956	*NAGLU*	1'786	1'631	1'715	1'547	1'883	0.284	0.72136008	1
ENSBTAG00000047957	*SCD*	197	254	232	171	293	0.782	0.444648587	1
ENSBTAG00000047958	*BT.96685*	1'941	1'658	1'798	1'681	1'914	0.188	0.813257943	1
ENSBTAG00000047961	*protein_coding*	1	0	0	1	0		1	1
ENSBTAG00000047962	*protein_coding*	76	66	71	66	76	0.212	0.894603615	1
ENSBTAG00000047963	*pseudogene*	4	15	10	3	17	2.322	0.608798021	1
ENSBTAG00000047964	*TMEM210*	1	2	2	1	2	1.415	0.981979269	1
ENSBTAG00000047965	*protein_coding*	1	0	0	1	0		1	1
ENSBTAG00000047966	*processed_pseudogene*	258	418	353	223	483	1.111	0.238847622	1
ENSBTAG00000047968	*BTBD18*	4	3	3	3	3	0.000	1	1
ENSBTAG00000047969	*NAT8*	12	11	12	10	13	0.290	0.964579659	1
ENSBTAG00000047970	*FDPS*	503	864	717	436	998	1.196	0.163631686	1
ENSBTAG00000047972	*C1ORF106*	75	178	135	65	206	1.662	0.166214492	1
ENSBTAG00000047973	*ASPSCR1*	337	455	409	292	525	0.848	0.355035049	1
ENSBTAG00000047975	*BT.19222*	439	589	530	380	680	0.839	0.342112413	1
ENSBTAG00000047977	*protein_coding*	5	4	4	4	5	0.093	1	1
ENSBTAG00000047979	*protein_coding*	139	220	187	120	254	1.077	0.319075116	1
ENSBTAG00000047988	*protein_coding*	91	54	71	79	62	-0.338	0.829039312	1
ENSBTAG00000047990	*protein_coding*	7	3	5	6	3	-0.807	0.947428618	1
ENSBTAG00000047991	*TAF4*	1'524	734	1'084	1'320	848	-0.639	0.437318931	1
ENSBTAG00000047992	*BT.71103*	19	14	16	16	16	-0.026	1	1
ENSBTAG00000047993	*miRNA*	1	0	0	1	0		1	1
ENSBTAG00000047994	*protein_coding*	2	1	1	2	1	-0.585	1	1
ENSBTAG00000047996	*IFITM10*	31	24	27	27	28	0.046	1	1
ENSBTAG00000047997	*BT.34369*	151	200	181	131	231	0.820	0.450926409	1
ENSBTAG00000047998	*COL5A1*	89'068	198'625	153'244	77'135	229'352	1.572	0.042356182	1
ENSBTAG00000048002	*pseudogene*	51	13	30	44	15	-1.557	0.499888893	1
ENSBTAG00000048005	*MGC157368*	135	22	71	117	25	-2.202	0.153590429	1
ENSBTAG00000048006	*pseudogene*	2	4	3	2	5	1.415	0.927250049	1
ENSBTAG00000048007	*protein_coding*	5	3	4	4	3	-0.322	1	1
ENSBTAG00000048008	*pseudogene*	12	0	5	10	0		0.583618942	1
ENSBTAG00000048010	*NUDT14*	352	347	353	305	401	0.394	0.674449884	1
ENSBTAG00000048011	*GPR160*	38	21	29	33	24	-0.441	0.861339366	1
ENSBTAG00000048014	*pseudogene*	1	1	1	1	1	0.415	1	1
ENSBTAG00000048016	*BT.86229*	2	0	1	2	0		0.974934741	1
ENSBTAG00000048017	*PRR16*	627	552	590	543	637	0.231	0.790579205	1
ENSBTAG00000048020	*protein_coding*	201	111	151	174	128	-0.442	0.702793426	1
ENSBTAG00000048021	*PNMAL2*	100	69	83	87	80	-0.120	0.941270491	1
ENSBTAG00000048022	*protein_coding*	259	215	236	224	248	0.146	0.888367748	1
ENSBTAG00000048023	*protein_coding*	133	114	123	115	132	0.193	0.87903319	1
ENSBTAG00000048025	*protein_coding*	1	0	0	1	0		1	1
ENSBTAG00000048029	*BT.3417*	6	0	3	5	0		0.81115604	1
ENSBTAG00000048032	*protein_coding*	0	1	1	0	1	Inf	0.993540919	1
ENSBTAG00000048033	*C5ORF43*	920	606	748	797	700	-0.187	0.826665189	1
ENSBTAG00000048034	*GLTPD2*	29	23	26	25	27	0.081	0.991041129	1
ENSBTAG00000048038	*bta-mir-2284w*	1	0	0	1	0		1	1
ENSBTAG00000048040	*protein_coding*	0	1	1	0	1	Inf	0.993540919	1
ENSBTAG00000048041	*ACTR1B*	1'541	1'580	1'579	1'335	1'824	0.451	0.573124205	1
ENSBTAG00000048042	*protein_coding*	625	284	435	541	328	-0.723	0.426705749	1
ENSBTAG00000048043	*protein_coding*	0	1	1	0	1	Inf	0.993540919	1
ENSBTAG00000048044	*IQCF3*	1	0	0	1	0		1	1
ENSBTAG00000048046	*pseudogene*	1	0	0	1	0		1	1
ENSBTAG00000048047	*protein_coding*	1	1	1	1	1	0.415	1	1
ENSBTAG00000048049	*protein_coding*	99	47	70	86	54	-0.660	0.664833487	1

Ensembl gene ID	geneName	counts wildtype horn bud	counts polled horn bud	baseMean	baseMean wildtype horn bud	baseMean polled horn bud	log2FoldChange	pval	padj
ENSBTAG00000048052	*OR52I2*	3	1	2	3	1	-1.170	0.985253077	1
ENSBTAG00000048053	*SLBP2*	0	1	1	0	1	Inf	0.993540919	1
ENSBTAG00000048054	*UAP1L1*	1'185	1'655	1'469	1'026	1'911	0.897	0.266955755	1
ENSBTAG00000048057	*BT.48740*	382	313	346	331	361	0.128	0.894047708	1
ENSBTAG00000048058	*CDO1*	631	574	605	546	663	0.278	0.748161962	1
ENSBTAG00000048059	*BT.25695*	428	281	348	371	324	-0.192	0.841222668	1
ENSBTAG00000048060	*TBX22*	9	33	23	8	38	2.290	0.392709147	1
ENSBTAG00000048061	*GRM5*	30	0	13	26	0		0.205783873	1
ENSBTAG00000048062	*KDM6B*	1'755	2'767	2'357	1'520	3'195	1.072	0.176805021	1
ENSBTAG00000048067	*C17ORF96*	5	15	11	4	17	2.000	0.647640022	1
ENSBTAG00000048070	*SNORD53_SNORD92*	2	0	1	2	0		0.974934741	1
ENSBTAG00000048071	*EXOSC2*	1'191	996	1'091	1'031	1'150	0.157	0.848078664	1
ENSBTAG00000048076	*bta-mir-3432-1*	1	1	1	1	1	0.415	1	1
ENSBTAG00000048077	*BT.96666*	8'222	8'491	8'463	7'120	9'805	0.461	0.54686037	1
ENSBTAG00000048078	*BT.62001*	4	0	2	3	0		0.89545886	1
ENSBTAG00000048080	*protein_coding*	33	25	29	29	29	0.014	1	1
ENSBTAG00000048081	*TOMM20*	241	266	258	209	307	0.557	0.576519137	1
ENSBTAG00000048082	*BT.103662*	4	1	2	3	1	-1.585	0.952247636	1
ENSBTAG00000048083	*BT.23580*	2	0	1	2	0		0.974934741	1
ENSBTAG00000048085	*KLF5*	3	2	2	3	2	-0.170	1	1
ENSBTAG00000048086	*TEX13A*	13	17	15	11	20	0.802	0.820295848	1
ENSBTAG00000048087	*protein_coding*	22	5	12	19	6	-1.722	0.669657938	1
ENSBTAG00000048089	*protein_coding*	83	45	62	72	52	-0.468	0.774000712	1
ENSBTAG00000048090	*BT.64954*	828	847	848	717	978	0.448	0.592340795	1
ENSBTAG00000048096	*protein_coding*	63	6	31	55	7	-2.977	0.209781139	1
ENSBTAG00000048097	*protein_coding*	7	12	10	6	14	1.193	0.802511113	1
ENSBTAG00000048098	*PKP3*	2'007	3'585	2'939	1'738	4'140	1.252	0.113463316	1
ENSBTAG00000048101	*SNORD113*	35	8	20	30	9	-1.714	0.559280423	1
ENSBTAG00000048103	*Metazoa_SRP*	0	1	1	0	1	Inf	0.993540919	1
ENSBTAG00000048107	*CANX*	21'941	18'197	20'007	19'001	21'012	0.145	0.848149994	1
ENSBTAG00000048110	*miRNA*	0	1	1	0	1	Inf	0.993540919	1
ENSBTAG00000048112	*BT.29621*	15	18	17	13	21	0.678	0.840652913	1
ENSBTAG00000048114	*protein_coding*	44	22	32	38	25	-0.585	0.798419738	1
ENSBTAG00000048116	*miRNA*	0	3	2	0	3	Inf	0.878646179	1
ENSBTAG00000048117	*miRNA*	0	1	1	0	1	Inf	0.993540919	1
ENSBTAG00000048118	*LGI1*	76	7	37	66	8	-3.026	0.161453911	1
ENSBTAG00000048119	*protein_coding*	195	206	203	169	238	0.494	0.64000064	1
ENSBTAG00000048120	*7SK*	60	20	38	52	23	-1.170	0.563919696	1
ENSBTAG00000048121	*SNORD116*	3	4	4	3	5	0.830	0.962490925	1
ENSBTAG00000048122	*CFD*	516	175	324	447	202	-1.145	0.233236577	1
ENSBTAG00000048123	*TSSK2*	71	180	135	61	208	1.757	0.144843829	1
ENSBTAG00000048125	*ENTPD2*	1'672	1'542	1'614	1'448	1'781	0.298	0.708924479	1
ENSBTAG00000048127	*SPACA4*	1	1	1	1	1	0.415	1	1
ENSBTAG00000048132	*U6*	3	1	2	3	1	-1.170	0.985253077	1
ENSBTAG00000048135	*BT.97040*	8	1	4	7	1	-2.585	0.818599781	1
ENSBTAG00000048136	*Metazoa_SRP*	1	0	0	1	0		1	1
ENSBTAG00000048137	*protein_coding*	126	138	134	109	159	0.546	0.646043043	1
ENSBTAG00000048138	*XKR9*	1	0	0	1	0		1	1
ENSBTAG00000048140	*HEATR2*	604	407	497	523	470	-0.154	0.86452302	1
ENSBTAG00000048142	*GPR45*	8	17	13	7	20	1.503	0.690403323	1
ENSBTAG00000048143	*WBP5*	638	146	361	553	169	-1.713	0.072986551	1
ENSBTAG00000048145	*protein_coding*	0	2	1	0	2	Inf	0.939077559	1
ENSBTAG00000048146	*bta-mir-154c*	15	7	11	13	8	-0.684	0.893749952	1
ENSBTAG00000048148	*5_8S_rRNA*	793	291	511	687	336	-1.031	0.247423142	1
ENSBTAG00000048150	*protein_coding*	1	0	0	1	0		1	1
ENSBTAG00000048151	*PRPF40A*	3'192	1'762	2'399	2'764	2'035	-0.442	0.574847484	1
ENSBTAG00000048152	*TUBG1*	1'662	1'918	1'827	1'439	2'215	0.622	0.434891002	1
ENSBTAG00000048153	*protein_coding*	30	12	20	26	14	-0.907	0.760364313	1
ENSBTAG00000048154	*GPER*	40	17	27	35	20	-0.819	0.738857766	1
ENSBTAG00000048155	*FCN2*	181	186	186	157	215	0.454	0.674840547	1
ENSBTAG00000048156	*pseudogene*	30	69	53	26	80	1.617	0.344813545	1
ENSBTAG00000048157	*BT.104783*	14'422	9'718	11'856	12'490	11'221	-0.155	0.840336024	1
ENSBTAG00000048158	*pseudogene*	10	4	7	9	5	-0.907	0.905171387	1
ENSBTAG00000048159	*TAB3*	245	204	224	212	236	0.151	0.886544035	1
ENSBTAG00000048160	*TIGD5*	427	901	705	370	1'040	1.492	0.084709103	1
ENSBTAG00000048161	*protein_coding*	2	0	1	2	0		0.974934741	1
ENSBTAG00000048162	*C5H12ORF4*	156	79	113	135	91	-0.567	0.654671409	1
ENSBTAG00000048166	*protein_coding*	0	1	1	0	1	Inf	0.993540919	1
ENSBTAG00000048167	*U4*	42	4	20	36	5	-2.977	0.320832869	1
ENSBTAG00000048170	*PCDHA11*	18	4	10	16	5	-1.755	0.710592951	1
ENSBTAG00000048171	*TAP*	1	0	0	1	0		1	1
ENSBTAG00000048173	*BT.83918*	628	179	375	544	207	-1.396	0.137948932	1

Ensembl gene ID	geneName	counts wildtype horn bud	counts polled horn bud	baseMean	baseMean wildtype horn bud	baseMean polled horn bud	log2FoldChange	pval	padj
ENSBTAG00000048174	*bta-mir-2887-2*	421	136	261	365	157	-1.215	0.226496896	1
ENSBTAG00000048177	*protein_coding*	1	0	0	1	0		1	1
ENSBTAG00000048178	*miRNA*	1	0	0	1	0		1	1
ENSBTAG00000048179	*protein_coding*	0	1	1	0	1	Inf	0.993540919	1
ENSBTAG00000048184	*BT.96733*	38	28	33	33	32	-0.026	1	1
ENSBTAG00000048185	*Metazoa_SRP*	68	52	59	59	60	0.028	0.997510019	1
ENSBTAG00000048188	*protein_coding*	9	20	15	8	23	1.567	0.644190163	1
ENSBTAG00000048191	*bta-mir-2887-2*	4	3	3	3	3	0.000	1	1
ENSBTAG00000048195	*PIGX*	371	214	284	321	247	-0.379	0.700633654	1
ENSBTAG00000048199	*protein_coding*	0	1	1	0	1	Inf	0.993540919	1
ENSBTAG00000048203	*SAMD13*	12	17	15	10	20	0.918	0.796381602	1
ENSBTAG00000048204	*protein_coding*	937	415	645	811	479	-0.760	0.378848948	1
ENSBTAG00000048205	*bta-mir-2887-2*	349	211	273	302	244	-0.311	0.755144209	1
ENSBTAG00000048206	*protein_coding*	23	12	17	20	14	-0.524	0.884396421	1
ENSBTAG00000048207	*C2ORF15*	1	0	0	1	0		1	1
ENSBTAG00000048209	*pseudogene*	13	4	8	11	5	-1.285	0.828302954	1
ENSBTAG00000048211	*miRNA*	8	8	8	7	9	0.415	0.960311941	1
ENSBTAG00000048212	*protein_coding*	10	1	5	9	1	-2.907	0.754028015	1
ENSBTAG00000048217	*snoZ17*	47	50	49	41	58	0.504	0.778733205	1
ENSBTAG00000048219	*protein_coding*	1	0	0	1	0		1	1
ENSBTAG00000048225	*BT.87090*	6'416	8'747	7'828	5'556	10'100	0.862	0.263341256	1
ENSBTAG00000048226	*protein_coding*	81	46	62	70	53	-0.401	0.807729815	1
ENSBTAG00000048228	*SNHG12*	2'335	1'641	1'959	2'022	1'895	-0.094	0.907021505	1
ENSBTAG00000048229	*TPT1*	77'800	107'026	95'480	67'377	123'583	0.875	0.25136959	1
ENSBTAG00000048230	*C6ORF132*	414	826	656	359	954	1.412	0.104900928	1
ENSBTAG00000048234	*BT.97025*	1'383	1'185	1'283	1'198	1'368	0.192	0.812510028	1
ENSBTAG00000048237	*FGF9*	81	36	56	70	42	-0.755	0.652962904	1
ENSBTAG00000048241	*bta-mir-3431*	561	200	358	486	231	-1.073	0.254847376	1
ENSBTAG00000048246	*DPP4*	492	270	369	426	312	-0.451	0.629811938	1
ENSBTAG00000048249	*BT.25659*	1	0	0	1	0		1	1
ENSBTAG00000048253	*CCDC101*	223	169	194	193	195	0.015	0.99360941	1
ENSBTAG00000048255	*5_8S_rRNA*	305	174	233	264	201	-0.395	0.701705168	1
ENSBTAG00000048256	*protein_coding*	1	0	0	1	0		1	1
ENSBTAG00000048257	*CYP4F3*	66	137	108	57	158	1.469	0.253567276	1
ENSBTAG00000048261	*BOSTAUV1R434*	1	0	0	1	0		1	1
ENSBTAG00000048262	*ACTR5*	485	545	525	420	629	0.583	0.508543809	1
ENSBTAG00000048263	*LRRC56*	55	23	37	48	27	-0.843	0.68159758	1
ENSBTAG00000048264	*FLRT1*	203	338	283	176	390	1.151	0.242359284	1
ENSBTAG00000048265	*ERI1*	68	131	105	59	151	1.361	0.293032315	1
ENSBTAG00000048267	*protein_coding*	26	11	18	23	13	-0.826	0.800203209	1
ENSBTAG00000048268	*BT.98069*	2	0	1	2	0		0.974934741	1
ENSBTAG00000048269	*CTXN1*	93	29	57	81	33	-1.266	0.444649684	1
ENSBTAG00000048271	*MICAL3*	1'596	2'148	1'931	1'382	2'480	0.844	0.289638742	1
ENSBTAG00000048273	*C4ORF3*	6'163	4'038	5'000	5'337	4'663	-0.195	0.801282741	1
ENSBTAG00000048274	*pseudogene*	1	0	0	1	0		1	1
ENSBTAG00000048278	*C17ORF47*	3	0	1	3	0		0.936647693	1
ENSBTAG00000048280	*BT.69141*	836	507	655	724	585	-0.306	0.72258963	1
ENSBTAG00000048285	*bta-mir-2894*	1	0	0	1	0		1	1
ENSBTAG00000048286	*E2F6*	1'108	723	897	960	835	-0.201	0.810825959	1
ENSBTAG00000048287	*BT.20005*	12	15	14	10	17	0.737	0.84931155	1
ENSBTAG00000048289	*7SK*	0	1	1	0	1	Inf	0.993540919	1
ENSBTAG00000048290	*protein_coding*	15	4	9	13	5	-1.492	0.779648463	1
ENSBTAG00000048291	*protein_coding*	53	41	47	46	47	0.045	0.993138314	1
ENSBTAG00000048293	*U2*	3	5	4	3	6	1.152	0.920866718	1
ENSBTAG00000048296	*BT.62996*	397	239	310	344	276	-0.317	0.743796816	1
ENSBTAG00000048304	*protein_coding*	2	3	3	2	3	1.000	0.97171833	1
ENSBTAG00000048306	*protein_coding*	122	75	96	106	87	-0.287	0.835563996	1
ENSBTAG00000048308	*ICMT*	642	816	749	556	942	0.761	0.369641371	1
ENSBTAG00000048309	*KIAA1024L*	58	48	53	50	55	0.142	0.94362114	1
ENSBTAG00000048314	*protein_coding*	1'506	736	1'077	1'304	850	-0.618	0.4527249	1
no_feature	*MISSING*	24'612'651	########	20'987'187	21'315'181	20'659'193	-0.045	0.953190909	1
alignment_not_unique	*MISSING*	0	0	0	0	0	NA	NA	NA
ENSBTAG00000000059	*RPL22*	0	0	0	0	0	NA	NA	NA
ENSBTAG00000000060	*PRDM14*	0	0	0	0	0	NA	NA	NA
ENSBTAG00000000070	*F13B*	0	0	0	0	0	NA	NA	NA
ENSBTAG00000000085	*BT.45907*	0	0	0	0	0	NA	NA	NA
ENSBTAG00000000118	*protein_coding*	0	0	0	0	0	NA	NA	NA
ENSBTAG00000000119	*pseudogene*	0	0	0	0	0	NA	NA	NA
ENSBTAG00000000145	*OR13D1*	0	0	0	0	0	NA	NA	NA
ENSBTAG00000000168	*protein_coding*	0	0	0	0	0	NA	NA	NA
ENSBTAG00000000171	*pseudogene*	0	0	0	0	0	NA	NA	NA
ENSBTAG00000000226	*DKK4*	0	0	0	0	0	NA	NA	NA

Ensembl gene ID	geneName	counts wildtype horn bud	counts polled horn bud	baseMean	baseMean wildtype horn bud	baseMean polled horn bud	log2FoldChange	pval	padj
ENSBTAG00000000228	*protein_coding*	0	0	0	0	0	NA	NA	NA
ENSBTAG00000000229	*protein_coding*	0	0	0	0	0	NA	NA	NA
ENSBTAG00000000259	*BT.66344*	0	0	0	0	0	NA	NA	NA
ENSBTAG00000000273	*IL5RA*	0	0	0	0	0	NA	NA	NA
ENSBTAG00000000280	*protein_coding*	0	0	0	0	0	NA	NA	NA
ENSBTAG00000000335	*HMGB4*	0	0	0	0	0	NA	NA	NA
ENSBTAG00000000344	*BT.54326*	0	0	0	0	0	NA	NA	NA
ENSBTAG00000000351	*protein_coding*	0	0	0	0	0	NA	NA	NA
ENSBTAG00000000360	*pseudogene*	0	0	0	0	0	NA	NA	NA
ENSBTAG00000000368	*OR56A1*	0	0	0	0	0	NA	NA	NA
ENSBTAG00000000410	*PAX4*	0	0	0	0	0	NA	NA	NA
ENSBTAG00000000426	*pseudogene*	0	0	0	0	0	NA	NA	NA
ENSBTAG00000000438	*GNRHR*	0	0	0	0	0	NA	NA	NA
ENSBTAG00000000487	*protein_coding*	0	0	0	0	0	NA	NA	NA
ENSBTAG00000000502	*DAZL*	0	0	0	0	0	NA	NA	NA
ENSBTAG00000000567	*protein_coding*	0	0	0	0	0	NA	NA	NA
ENSBTAG00000000574	*OR10Z1*	0	0	0	0	0	NA	NA	NA
ENSBTAG00000000617	*protein_coding*	0	0	0	0	0	NA	NA	NA
ENSBTAG00000000688	*protein_coding*	0	0	0	0	0	NA	NA	NA
ENSBTAG00000000726	*OR1J1*	0	0	0	0	0	NA	NA	NA
ENSBTAG00000000763	*protein_coding*	0	0	0	0	0	NA	NA	NA
ENSBTAG00000000765	*protein_coding*	0	0	0	0	0	NA	NA	NA
ENSBTAG00000000805	*IQCF1*	0	0	0	0	0	NA	NA	NA
ENSBTAG00000000872	*protein_coding*	0	0	0	0	0	NA	NA	NA
ENSBTAG00000000926	*protein_coding*	0	0	0	0	0	NA	NA	NA
ENSBTAG00000000929	*pseudogene*	0	0	0	0	0	NA	NA	NA
ENSBTAG00000000953	*pseudogene*	0	0	0	0	0	NA	NA	NA
ENSBTAG00000000963	*BT.82078*	0	0	0	0	0	NA	NA	NA
ENSBTAG00000000966	*BT.59291*	0	0	0	0	0	NA	NA	NA
ENSBTAG00000000992	*BT.106443*	0	0	0	0	0	NA	NA	NA
ENSBTAG00000000997	*pseudogene*	0	0	0	0	0	NA	NA	NA
ENSBTAG00000001006	*HMX3*	0	0	0	0	0	NA	NA	NA
ENSBTAG00000001084	*BT.76106*	0	0	0	0	0	NA	NA	NA
ENSBTAG00000001191	*CCL17*	0	0	0	0	0	NA	NA	NA
ENSBTAG00000001210	*protein_coding*	0	0	0	0	0	NA	NA	NA
ENSBTAG00000001214	*pseudogene*	0	0	0	0	0	NA	NA	NA
ENSBTAG00000001221	*NKX3-1*	0	0	0	0	0	NA	NA	NA
ENSBTAG00000001265	*HRG*	0	0	0	0	0	NA	NA	NA
ENSBTAG00000001291	*OR8H3*	0	0	0	0	0	NA	NA	NA
ENSBTAG00000001317	*pseudogene*	0	0	0	0	0	NA	NA	NA
ENSBTAG00000001336	*TAS2R46*	0	0	0	0	0	NA	NA	NA
ENSBTAG00000001383	*pseudogene*	0	0	0	0	0	NA	NA	NA
ENSBTAG00000001397	*protein_coding*	0	0	0	0	0	NA	NA	NA
ENSBTAG00000001416	*BT.106502*	0	0	0	0	0	NA	NA	NA
ENSBTAG00000001423	*protein_coding*	0	0	0	0	0	NA	NA	NA
ENSBTAG00000001442	*protein_coding*	0	0	0	0	0	NA	NA	NA
ENSBTAG00000001448	*BT.106433*	0	0	0	0	0	NA	NA	NA
ENSBTAG00000001451	*A4GNT*	0	0	0	0	0	NA	NA	NA
ENSBTAG00000001455	*BT.99285*	0	0	0	0	0	NA	NA	NA
ENSBTAG00000001488	*OTC*	0	0	0	0	0	NA	NA	NA
ENSBTAG00000001495	*SPINLW1*	0	0	0	0	0	NA	NA	NA
ENSBTAG00000001515	*pseudogene*	0	0	0	0	0	NA	NA	NA
ENSBTAG00000001516	*CRSP1*	0	0	0	0	0	NA	NA	NA
ENSBTAG00000001549	*OR1L1*	0	0	0	0	0	NA	NA	NA
ENSBTAG00000001606	*OR2C1*	0	0	0	0	0	NA	NA	NA
ENSBTAG00000001638	*FGA*	0	0	0	0	0	NA	NA	NA
ENSBTAG00000001681	*pseudogene*	0	0	0	0	0	NA	NA	NA
ENSBTAG00000001726	*CDX4*	0	0	0	0	0	NA	NA	NA
ENSBTAG00000001761	*protein_coding*	0	0	0	0	0	NA	NA	NA
ENSBTAG00000001809	*DUSP21*	0	0	0	0	0	NA	NA	NA
ENSBTAG00000001819	*BT.46244*	0	0	0	0	0	NA	NA	NA
ENSBTAG00000001873	*protein_coding*	0	0	0	0	0	NA	NA	NA
ENSBTAG00000001893	*TM4SF4*	0	0	0	0	0	NA	NA	NA
ENSBTAG00000001925	*TMPRSS11D*	0	0	0	0	0	NA	NA	NA
ENSBTAG00000002008	*pseudogene*	0	0	0	0	0	NA	NA	NA
ENSBTAG00000002016	*pseudogene*	0	0	0	0	0	NA	NA	NA
ENSBTAG00000002043	*protein_coding*	0	0	0	0	0	NA	NA	NA
ENSBTAG00000002071	*protein_coding*	0	0	0	0	0	NA	NA	NA
ENSBTAG00000002088	*BT.99282*	0	0	0	0	0	NA	NA	NA
ENSBTAG00000002099	*BT.104599*	0	0	0	0	0	NA	NA	NA
ENSBTAG00000002134	*FAM196B*	0	0	0	0	0	NA	NA	NA
ENSBTAG00000002150	*IL17*	0	0	0	0	0	NA	NA	NA

Ensembl gene ID	geneName	counts wildtype horn bud	counts polled horn bud	baseMean	baseMean wildtype horn bud	baseMean polled horn bud	log2FoldChange	pval	padj
ENSBTAG00000002158	*protein_coding*	0	0	0	0	0	NA	NA	NA
ENSBTAG00000002245	*BT.105410*	0	0	0	0	0	NA	NA	NA
ENSBTAG00000002247	*protein_coding*	0	0	0	0	0	NA	NA	NA
ENSBTAG00000002253	*FKBP6*	0	0	0	0	0	NA	NA	NA
ENSBTAG00000002261	*BT.54498*	0	0	0	0	0	NA	NA	NA
ENSBTAG00000002273	*ARGFX*	0	0	0	0	0	NA	NA	NA
ENSBTAG00000002284	*BT.87578*	0	0	0	0	0	NA	NA	NA
ENSBTAG00000002288	*NT5DC4*	0	0	0	0	0	NA	NA	NA
ENSBTAG00000002289	*NLRP14*	0	0	0	0	0	NA	NA	NA
ENSBTAG00000002330	*protein_coding*	0	0	0	0	0	NA	NA	NA
ENSBTAG00000002335	*HAND1*	0	0	0	0	0	NA	NA	NA
ENSBTAG00000002380	*pseudogene*	0	0	0	0	0	NA	NA	NA
ENSBTAG00000002441	*BT.98647*	0	0	0	0	0	NA	NA	NA
ENSBTAG00000002446	*pseudogene*	0	0	0	0	0	NA	NA	NA
ENSBTAG00000002489	*BT.50400*	0	0	0	0	0	NA	NA	NA
ENSBTAG00000002498	*pseudogene*	0	0	0	0	0	NA	NA	NA
ENSBTAG00000002545	*protein_coding*	0	0	0	0	0	NA	NA	NA
ENSBTAG00000002559	*BT.27866*	0	0	0	0	0	NA	NA	NA
ENSBTAG00000002632	*CSN2*	0	0	0	0	0	NA	NA	NA
ENSBTAG00000002642	*SLC17A2*	0	0	0	0	0	NA	NA	NA
ENSBTAG00000002675	*CCDC7*	0	0	0	0	0	NA	NA	NA
ENSBTAG00000002679	*protein_coding*	0	0	0	0	0	NA	NA	NA
ENSBTAG00000002686	*OR1I1*	0	0	0	0	0	NA	NA	NA
ENSBTAG00000002700	*PLA2G2A*	0	0	0	0	0	NA	NA	NA
ENSBTAG00000002741	*processed_pseudogene*	0	0	0	0	0	NA	NA	NA
ENSBTAG00000002757	*IL20*	0	0	0	0	0	NA	NA	NA
ENSBTAG00000002803	*SULT1C3*	0	0	0	0	0	NA	NA	NA
ENSBTAG00000002805	*protein_coding*	0	0	0	0	0	NA	NA	NA
ENSBTAG00000002913	*protein_coding*	0	0	0	0	0	NA	NA	NA
ENSBTAG00000002919	*UNC5CL*	0	0	0	0	0	NA	NA	NA
ENSBTAG00000002963	*SAA4*	0	0	0	0	0	NA	NA	NA
ENSBTAG00000003006	*RNASE9*	0	0	0	0	0	NA	NA	NA
ENSBTAG00000003023	*protein_coding*	0	0	0	0	0	NA	NA	NA
ENSBTAG00000003056	*protein_coding*	0	0	0	0	0	NA	NA	NA
ENSBTAG00000003096	*OR6K3*	0	0	0	0	0	NA	NA	NA
ENSBTAG00000003107	*LHX5*	0	0	0	0	0	NA	NA	NA
ENSBTAG00000003118	*processed_pseudogene*	0	0	0	0	0	NA	NA	NA
ENSBTAG00000003134	*CRYAA*	0	0	0	0	0	NA	NA	NA
ENSBTAG00000003179	*RTP2*	0	0	0	0	0	NA	NA	NA
ENSBTAG00000003236	*BT.64180*	0	0	0	0	0	NA	NA	NA
ENSBTAG00000003250	*protein_coding*	0	0	0	0	0	NA	NA	NA
ENSBTAG00000003273	*pseudogene*	0	0	0	0	0	NA	NA	NA
ENSBTAG00000003278	*BT.65682*	0	0	0	0	0	NA	NA	NA
ENSBTAG00000003281	*protein_coding*	0	0	0	0	0	NA	NA	NA
ENSBTAG00000003297	*BT.27720*	0	0	0	0	0	NA	NA	NA
ENSBTAG00000003331	*NEUROG1*	0	0	0	0	0	NA	NA	NA
ENSBTAG00000003335	*VSX1*	0	0	0	0	0	NA	NA	NA
ENSBTAG00000003357	*CLRN2*	0	0	0	0	0	NA	NA	NA
ENSBTAG00000003364	*DEFB119*	0	0	0	0	0	NA	NA	NA
ENSBTAG00000003377	*TMEM174*	0	0	0	0	0	NA	NA	NA
ENSBTAG00000003380	*KCNK15*	0	0	0	0	0	NA	NA	NA
ENSBTAG00000003430	*EPO*	0	0	0	0	0	NA	NA	NA
ENSBTAG00000003442	*LRIT1*	0	0	0	0	0	NA	NA	NA
ENSBTAG00000003445	*ODF1*	0	0	0	0	0	NA	NA	NA
ENSBTAG00000003493	*protein_coding*	0	0	0	0	0	NA	NA	NA
ENSBTAG00000003523	*BT.63207*	0	0	0	0	0	NA	NA	NA
ENSBTAG00000003549	*protein_coding*	0	0	0	0	0	NA	NA	NA
ENSBTAG00000003551	*OR2T11*	0	0	0	0	0	NA	NA	NA
ENSBTAG00000003582	*OR51I1*	0	0	0	0	0	NA	NA	NA
ENSBTAG00000003621	*protein_coding*	0	0	0	0	0	NA	NA	NA
ENSBTAG00000003628	*PRSS58*	0	0	0	0	0	NA	NA	NA
ENSBTAG00000003645	*BT.87141*	0	0	0	0	0	NA	NA	NA
ENSBTAG00000003656	*pseudogene*	0	0	0	0	0	NA	NA	NA
ENSBTAG00000003664	*protein_coding*	0	0	0	0	0	NA	NA	NA
ENSBTAG00000003739	*pseudogene*	0	0	0	0	0	NA	NA	NA
ENSBTAG00000003818	*PGC*	0	0	0	0	0	NA	NA	NA
ENSBTAG00000003838	*pseudogene*	0	0	0	0	0	NA	NA	NA
ENSBTAG00000003850	*INSL5*	0	0	0	0	0	NA	NA	NA
ENSBTAG00000003867	*protein_coding*	0	0	0	0	0	NA	NA	NA
ENSBTAG00000003886	*BSP3*	0	0	0	0	0	NA	NA	NA
ENSBTAG00000003930	*OR4C15*	0	0	0	0	0	NA	NA	NA
ENSBTAG00000004003	*F9*	0	0	0	0	0	NA	NA	NA

Ensembl gene ID	geneName	counts wildtype horn bud	counts polled horn bud	baseMean	baseMean wildtype horn bud	baseMean polled horn bud	log2FoldChange	pval	padj
ENSBTAG00000004032	*pseudogene*	0	0	0	0	0	NA	NA	NA
ENSBTAG00000004040	*BT.68473*	0	0	0	0	0	NA	NA	NA
ENSBTAG00000004105	*TMEM247*	0	0	0	0	0	NA	NA	NA
ENSBTAG00000004116	*PAG10*	0	0	0	0	0	NA	NA	NA
ENSBTAG00000004134	*BT.99406*	0	0	0	0	0	NA	NA	NA
ENSBTAG00000004151	*protein_coding*	0	0	0	0	0	NA	NA	NA
ENSBTAG00000004181	*protein_coding*	0	0	0	0	0	NA	NA	NA
ENSBTAG00000004217	*protein_coding*	0	0	0	0	0	NA	NA	NA
ENSBTAG00000004219	*RPL27*	0	0	0	0	0	NA	NA	NA
ENSBTAG00000004241	*G6PC2*	0	0	0	0	0	NA	NA	NA
ENSBTAG00000004293	*BT.54108*	0	0	0	0	0	NA	NA	NA
ENSBTAG00000004314	*HOXD12*	0	0	0	0	0	NA	NA	NA
ENSBTAG00000004324	*protein_coding*	0	0	0	0	0	NA	NA	NA
ENSBTAG00000004369	*protein_coding*	0	0	0	0	0	NA	NA	NA
ENSBTAG00000004418	*PLA2G2E*	0	0	0	0	0	NA	NA	NA
ENSBTAG00000004474	*BANF2*	0	0	0	0	0	NA	NA	NA
ENSBTAG00000004487	*pseudogene*	0	0	0	0	0	NA	NA	NA
ENSBTAG00000004519	*ALPI*	0	0	0	0	0	NA	NA	NA
ENSBTAG00000004530	*pseudogene*	0	0	0	0	0	NA	NA	NA
ENSBTAG00000004563	*BT.50916*	0	0	0	0	0	NA	NA	NA
ENSBTAG00000004569	*GLIPR1L1*	0	0	0	0	0	NA	NA	NA
ENSBTAG00000004583	*MS4A5*	0	0	0	0	0	NA	NA	NA
ENSBTAG00000004596	*CYP11B2*	0	0	0	0	0	NA	NA	NA
ENSBTAG00000004611	*CEACAM18*	0	0	0	0	0	NA	NA	NA
ENSBTAG00000004667	*protein_coding*	0	0	0	0	0	NA	NA	NA
ENSBTAG00000004702	*protein_coding*	0	0	0	0	0	NA	NA	NA
ENSBTAG00000004710	*BT.41992*	0	0	0	0	0	NA	NA	NA
ENSBTAG00000004712	*MC3R*	0	0	0	0	0	NA	NA	NA
ENSBTAG00000004725	*BT.32473*	0	0	0	0	0	NA	NA	NA
ENSBTAG00000004765	*protein_coding*	0	0	0	0	0	NA	NA	NA
ENSBTAG00000004778	*BT.71529*	0	0	0	0	0	NA	NA	NA
ENSBTAG00000004821	*OR4K14*	0	0	0	0	0	NA	NA	NA
ENSBTAG00000004830	*ADAM18*	0	0	0	0	0	NA	NA	NA
ENSBTAG00000004890	*protein_coding*	0	0	0	0	0	NA	NA	NA
ENSBTAG00000004944	*ANXA10*	0	0	0	0	0	NA	NA	NA
ENSBTAG00000004955	*SYTL5*	0	0	0	0	0	NA	NA	NA
ENSBTAG00000005005	*CSN1S2*	0	0	0	0	0	NA	NA	NA
ENSBTAG00000005041	*BT.36386*	0	0	0	0	0	NA	NA	NA
ENSBTAG00000005051	*protein_coding*	0	0	0	0	0	NA	NA	NA
ENSBTAG00000005054	*protein_coding*	0	0	0	0	0	NA	NA	NA
ENSBTAG00000005060	*OR52K2*	0	0	0	0	0	NA	NA	NA
ENSBTAG00000005070	*protein_coding*	0	0	0	0	0	NA	NA	NA
ENSBTAG00000005087	*OR5T1*	0	0	0	0	0	NA	NA	NA
ENSBTAG00000005101	*pseudogene*	0	0	0	0	0	NA	NA	NA
ENSBTAG00000005103	*FEZF1*	0	0	0	0	0	NA	NA	NA
ENSBTAG00000005112	*OR8G5*	0	0	0	0	0	NA	NA	NA
ENSBTAG00000005122	*KNG1*	0	0	0	0	0	NA	NA	NA
ENSBTAG00000005128	*VPREB3*	0	0	0	0	0	NA	NA	NA
ENSBTAG00000005199	*protein_coding*	0	0	0	0	0	NA	NA	NA
ENSBTAG00000005200	*WDR64*	0	0	0	0	0	NA	NA	NA
ENSBTAG00000005228	*CNGA4*	0	0	0	0	0	NA	NA	NA
ENSBTAG00000005248	*OFCC1*	0	0	0	0	0	NA	NA	NA
ENSBTAG00000005255	*protein_coding*	0	0	0	0	0	NA	NA	NA
ENSBTAG00000005262	*pseudogene*	0	0	0	0	0	NA	NA	NA
ENSBTAG00000005267	*protein_coding*	0	0	0	0	0	NA	NA	NA
ENSBTAG00000005270	*BT.50661*	0	0	0	0	0	NA	NA	NA
ENSBTAG00000005287	*BT.48294*	0	0	0	0	0	NA	NA	NA
ENSBTAG00000005319	*BT.63279*	0	0	0	0	0	NA	NA	NA
ENSBTAG00000005386	*SLC2A2*	0	0	0	0	0	NA	NA	NA
ENSBTAG00000005425	*GATA4*	0	0	0	0	0	NA	NA	NA
ENSBTAG00000005435	*protein_coding*	0	0	0	0	0	NA	NA	NA
ENSBTAG00000005449	*MCHR2*	0	0	0	0	0	NA	NA	NA
ENSBTAG00000005491	*protein_coding*	0	0	0	0	0	NA	NA	NA
ENSBTAG00000005520	*OR7C2*	0	0	0	0	0	NA	NA	NA
ENSBTAG00000005546	*HOXB7*	0	0	0	0	0	NA	NA	NA
ENSBTAG00000005570	*BT.106446*	0	0	0	0	0	NA	NA	NA
ENSBTAG00000005606	*BT.105875*	0	0	0	0	0	NA	NA	NA
ENSBTAG00000005624	*protein_coding*	0	0	0	0	0	NA	NA	NA
ENSBTAG00000005648	*POU4F2*	0	0	0	0	0	NA	NA	NA
ENSBTAG00000005702	*CNGB1*	0	0	0	0	0	NA	NA	NA
ENSBTAG00000005749	*MAGEB16*	0	0	0	0	0	NA	NA	NA
ENSBTAG00000005759	*PLA2G2A*	0	0	0	0	0	NA	NA	NA

Ensembl gene ID	geneName	counts wildtype horn bud	counts polled horn bud	baseMean	baseMean wildtype horn bud	baseMean polled horn bud	log2FoldChange	pval	padj
ENSBTAG00000005774	*VRTN*	0	0	0	0	0	NA	NA	NA
ENSBTAG00000005864	*BT.50682*	0	0	0	0	0	NA	NA	NA
ENSBTAG00000005874	*protein_coding*	0	0	0	0	0	NA	NA	NA
ENSBTAG00000005885	*protein_coding*	0	0	0	0	0	NA	NA	NA
ENSBTAG00000005914	*protein_coding*	0	0	0	0	0	NA	NA	NA
ENSBTAG00000005930	*protein_coding*	0	0	0	0	0	NA	NA	NA
ENSBTAG00000005938	*BT.87288*	0	0	0	0	0	NA	NA	NA
ENSBTAG00000005941	*protein_coding*	0	0	0	0	0	NA	NA	NA
ENSBTAG00000005953	*BT.54351*	0	0	0	0	0	NA	NA	NA
ENSBTAG00000006096	*TKDP1*	0	0	0	0	0	NA	NA	NA
ENSBTAG00000006128	*TCTEX1D4*	0	0	0	0	0	NA	NA	NA
ENSBTAG00000006179	*DNAJB8*	0	0	0	0	0	NA	NA	NA
ENSBTAG00000006198	*protein_coding*	0	0	0	0	0	NA	NA	NA
ENSBTAG00000006216	*protein_coding*	0	0	0	0	0	NA	NA	NA
ENSBTAG00000006264	*BT.65955*	0	0	0	0	0	NA	NA	NA
ENSBTAG00000006267	*protein_coding*	0	0	0	0	0	NA	NA	NA
ENSBTAG00000006294	*pseudogene*	0	0	0	0	0	NA	NA	NA
ENSBTAG00000006304	*PAG6*	0	0	0	0	0	NA	NA	NA
ENSBTAG00000006313	*OR6C76*	0	0	0	0	0	NA	NA	NA
ENSBTAG00000006380	*protein_coding*	0	0	0	0	0	NA	NA	NA
ENSBTAG00000006421	*OR5AR1*	0	0	0	0	0	NA	NA	NA
ENSBTAG00000006431	*NEUROG3*	0	0	0	0	0	NA	NA	NA
ENSBTAG00000006445	*LIPF*	0	0	0	0	0	NA	NA	NA
ENSBTAG00000006473	*pseudogene*	0	0	0	0	0	NA	NA	NA
ENSBTAG00000006484	*pseudogene*	0	0	0	0	0	NA	NA	NA
ENSBTAG00000006488	*protein_coding*	0	0	0	0	0	NA	NA	NA
ENSBTAG00000006514	*TRAV3*	0	0	0	0	0	NA	NA	NA
ENSBTAG00000006597	*pseudogene*	0	0	0	0	0	NA	NA	NA
ENSBTAG00000006599	*BT.56181*	0	0	0	0	0	NA	NA	NA
ENSBTAG00000006600	*protein_coding*	0	0	0	0	0	NA	NA	NA
ENSBTAG00000006605	*protein_coding*	0	0	0	0	0	NA	NA	NA
ENSBTAG00000006621	*protein_coding*	0	0	0	0	0	NA	NA	NA
ENSBTAG00000006629	*protein_coding*	0	0	0	0	0	NA	NA	NA
ENSBTAG00000006634	*OR4D5*	0	0	0	0	0	NA	NA	NA
ENSBTAG00000006648	*protein_coding*	0	0	0	0	0	NA	NA	NA
ENSBTAG00000006655	*PIP*	0	0	0	0	0	NA	NA	NA
ENSBTAG00000006674	*SLC13A1*	0	0	0	0	0	NA	NA	NA
ENSBTAG00000006709	*NPPA*	0	0	0	0	0	NA	NA	NA
ENSBTAG00000006717	*protein_coding*	0	0	0	0	0	NA	NA	NA
ENSBTAG00000006725	*pseudogene*	0	0	0	0	0	NA	NA	NA
ENSBTAG00000006753	*MBD3L1*	0	0	0	0	0	NA	NA	NA
ENSBTAG00000006761	*CSH1*	0	0	0	0	0	NA	NA	NA
ENSBTAG00000006802	*C13H20ORF71*	0	0	0	0	0	NA	NA	NA
ENSBTAG00000006810	*BT.91028*	0	0	0	0	0	NA	NA	NA
ENSBTAG00000006847	*pseudogene*	0	0	0	0	0	NA	NA	NA
ENSBTAG00000006855	*TDRD1*	0	0	0	0	0	NA	NA	NA
ENSBTAG00000006858	*pseudogene*	0	0	0	0	0	NA	NA	NA
ENSBTAG00000006879	*BT.30875*	0	0	0	0	0	NA	NA	NA
ENSBTAG00000006965	*NLRP8*	0	0	0	0	0	NA	NA	NA
ENSBTAG00000006975	*SI*	0	0	0	0	0	NA	NA	NA
ENSBTAG00000006986	*BT.62533*	0	0	0	0	0	NA	NA	NA
ENSBTAG00000007009	*RPL9*	0	0	0	0	0	NA	NA	NA
ENSBTAG00000007029	*OR4E2*	0	0	0	0	0	NA	NA	NA
ENSBTAG00000007041	*BT.92787*	0	0	0	0	0	NA	NA	NA
ENSBTAG00000007043	*SERPINA3*	0	0	0	0	0	NA	NA	NA
ENSBTAG00000007135	*REG3G*	0	0	0	0	0	NA	NA	NA
ENSBTAG00000007140	*protein_coding*	0	0	0	0	0	NA	NA	NA
ENSBTAG00000007194	*GUCA2B*	0	0	0	0	0	NA	NA	NA
ENSBTAG00000007238	*protein_coding*	0	0	0	0	0	NA	NA	NA
ENSBTAG00000007248	*protein_coding*	0	0	0	0	0	NA	NA	NA
ENSBTAG00000007297	*OR10T2*	0	0	0	0	0	NA	NA	NA
ENSBTAG00000007392	*SERPINA14*	0	0	0	0	0	NA	NA	NA
ENSBTAG00000007400	*OR2A5*	0	0	0	0	0	NA	NA	NA
ENSBTAG00000007411	*F7*	0	0	0	0	0	NA	NA	NA
ENSBTAG00000007518	*TRIM69*	0	0	0	0	0	NA	NA	NA
ENSBTAG00000007532	*TRIM15*	0	0	0	0	0	NA	NA	NA
ENSBTAG00000007557	*OR2AK2*	0	0	0	0	0	NA	NA	NA
ENSBTAG00000007601	*pseudogene*	0	0	0	0	0	NA	NA	NA
ENSBTAG00000007603	*HOXB1*	0	0	0	0	0	NA	NA	NA
ENSBTAG00000007627	*protein_coding*	0	0	0	0	0	NA	NA	NA
ENSBTAG00000007690	*BT.55194*	0	0	0	0	0	NA	NA	NA
ENSBTAG00000007706	*protein_coding*	0	0	0	0	0	NA	NA	NA

Ensembl gene ID	geneName	counts wildtype horn bud	counts polled horn bud	baseMean	baseMean wildtype horn bud	baseMean polled horn bud	log2FoldChange	pval	padj
ENSBTAG00000007729	*pseudogene*	0	0	0	0	0	NA	NA	NA
ENSBTAG00000007810	*pseudogene*	0	0	0	0	0	NA	NA	NA
ENSBTAG00000007839	*OTUD6A*	0	0	0	0	0	NA	NA	NA
ENSBTAG00000007849	*protein_coding*	0	0	0	0	0	NA	NA	NA
ENSBTAG00000007873	*OR6C2*	0	0	0	0	0	NA	NA	NA
ENSBTAG00000007944	*OR5I1*	0	0	0	0	0	NA	NA	NA
ENSBTAG00000007997	*protein_coding*	0	0	0	0	0	NA	NA	NA
ENSBTAG00000008029	*SPADH2*	0	0	0	0	0	NA	NA	NA
ENSBTAG00000008039	*ACMSD*	0	0	0	0	0	NA	NA	NA
ENSBTAG00000008085	*CRISP1*	0	0	0	0	0	NA	NA	NA
ENSBTAG00000008117	*SLC5A4*	0	0	0	0	0	NA	NA	NA
ENSBTAG00000008127	*RBP5*	0	0	0	0	0	NA	NA	NA
ENSBTAG00000008152	*protein_coding*	0	0	0	0	0	NA	NA	NA
ENSBTAG00000008155	*BT.64065*	0	0	0	0	0	NA	NA	NA
ENSBTAG00000008177	*pseudogene*	0	0	0	0	0	NA	NA	NA
ENSBTAG00000008226	*BT.71251*	0	0	0	0	0	NA	NA	NA
ENSBTAG00000008244	*protein_coding*	0	0	0	0	0	NA	NA	NA
ENSBTAG00000008312	*protein_coding*	0	0	0	0	0	NA	NA	NA
ENSBTAG00000008322	*KCNA10*	0	0	0	0	0	NA	NA	NA
ENSBTAG00000008326	*ZCCHC5*	0	0	0	0	0	NA	NA	NA
ENSBTAG00000008335	*protein_coding*	0	0	0	0	0	NA	NA	NA
ENSBTAG00000008357	*pseudogene*	0	0	0	0	0	NA	NA	NA
ENSBTAG00000008359	*protein_coding*	0	0	0	0	0	NA	NA	NA
ENSBTAG00000008379	*pseudogene*	0	0	0	0	0	NA	NA	NA
ENSBTAG00000008477	*protein_coding*	0	0	0	0	0	NA	NA	NA
ENSBTAG00000008481	*OR1D5*	0	0	0	0	0	NA	NA	NA
ENSBTAG00000008500	*CTRB1*	0	0	0	0	0	NA	NA	NA
ENSBTAG00000008502	*protein_coding*	0	0	0	0	0	NA	NA	NA
ENSBTAG00000008505	*APOB*	0	0	0	0	0	NA	NA	NA
ENSBTAG00000008511	*protein_coding*	0	0	0	0	0	NA	NA	NA
ENSBTAG00000008534	*protein_coding*	0	0	0	0	0	NA	NA	NA
ENSBTAG00000008540	*SLC22A1*	0	0	0	0	0	NA	NA	NA
ENSBTAG00000008588	*pseudogene*	0	0	0	0	0	NA	NA	NA
ENSBTAG00000008623	*FGF3*	0	0	0	0	0	NA	NA	NA
ENSBTAG00000008637	*TRIML2*	0	0	0	0	0	NA	NA	NA
ENSBTAG00000008678	*protein_coding*	0	0	0	0	0	NA	NA	NA
ENSBTAG00000008749	*OLIG3*	0	0	0	0	0	NA	NA	NA
ENSBTAG00000008786	*pseudogene*	0	0	0	0	0	NA	NA	NA
ENSBTAG00000008870	*KCNS1*	0	0	0	0	0	NA	NA	NA
ENSBTAG00000008894	*pseudogene*	0	0	0	0	0	NA	NA	NA
ENSBTAG00000008905	*FIGLA*	0	0	0	0	0	NA	NA	NA
ENSBTAG00000009017	*NR5A1*	0	0	0	0	0	NA	NA	NA
ENSBTAG00000009039	*OR4K5*	0	0	0	0	0	NA	NA	NA
ENSBTAG00000009044	*OR52N1*	0	0	0	0	0	NA	NA	NA
ENSBTAG00000009069	*protein_coding*	0	0	0	0	0	NA	NA	NA
ENSBTAG00000009073	*protein_coding*	0	0	0	0	0	NA	NA	NA
ENSBTAG00000009128	*PIT-1*	0	0	0	0	0	NA	NA	NA
ENSBTAG00000009143	*MC5R*	0	0	0	0	0	NA	NA	NA
ENSBTAG00000009197	*TRIM43*	0	0	0	0	0	NA	NA	NA
ENSBTAG00000009212	*APOA2*	0	0	0	0	0	NA	NA	NA
ENSBTAG00000009220	*pseudogene*	0	0	0	0	0	NA	NA	NA
ENSBTAG00000009249	*OTP*	0	0	0	0	0	NA	NA	NA
ENSBTAG00000009268	*pseudogene*	0	0	0	0	0	NA	NA	NA
ENSBTAG00000009280	*pseudogene*	0	0	0	0	0	NA	NA	NA
ENSBTAG00000009285	*OR2V1*	0	0	0	0	0	NA	NA	NA
ENSBTAG00000009365	*FSCN3*	0	0	0	0	0	NA	NA	NA
ENSBTAG00000009408	*ADAM2*	0	0	0	0	0	NA	NA	NA
ENSBTAG00000009440	*protein_coding*	0	0	0	0	0	NA	NA	NA
ENSBTAG00000009444	*SLC15A5*	0	0	0	0	0	NA	NA	NA
ENSBTAG00000009474	*protein_coding*	0	0	0	0	0	NA	NA	NA
ENSBTAG00000009497	*OR2M5*	0	0	0	0	0	NA	NA	NA
ENSBTAG00000009558	*BT.29765*	0	0	0	0	0	NA	NA	NA
ENSBTAG00000009626	*SPINT4*	0	0	0	0	0	NA	NA	NA
ENSBTAG00000009692	*pseudogene*	0	0	0	0	0	NA	NA	NA
ENSBTAG00000009722	*protein_coding*	0	0	0	0	0	NA	NA	NA
ENSBTAG00000009753	*protein_coding*	0	0	0	0	0	NA	NA	NA
ENSBTAG00000009763	*protein_coding*	0	0	0	0	0	NA	NA	NA
ENSBTAG00000009894	*SERPINA10*	0	0	0	0	0	NA	NA	NA
ENSBTAG00000009932	*protein_coding*	0	0	0	0	0	NA	NA	NA
ENSBTAG00000009980	*protein_coding*	0	0	0	0	0	NA	NA	NA
ENSBTAG00000010005	*protein_coding*	0	0	0	0	0	NA	NA	NA
ENSBTAG00000010057	*BT.19365*	0	0	0	0	0	NA	NA	NA

Ensembl gene ID	geneName	counts wildtype horn bud	counts polled horn bud	baseMean	baseMean wildtype horn bud	baseMean polled horn bud	log2FoldChange	pval	padj
ENSBTAG00000010072	*pseudogene*	0	0	0	0	0	NA	NA	NA
ENSBTAG00000010168	*protein_coding*	0	0	0	0	0	NA	NA	NA
ENSBTAG00000010184	*G6PC*	0	0	0	0	0	NA	NA	NA
ENSBTAG00000010197	*TSPO2*	0	0	0	0	0	NA	NA	NA
ENSBTAG00000010233	*SLC22A20*	0	0	0	0	0	NA	NA	NA
ENSBTAG00000010248	*BT.88716*	0	0	0	0	0	NA	NA	NA
ENSBTAG00000010283	*protein_coding*	0	0	0	0	0	NA	NA	NA
ENSBTAG00000010314	*ICAM4*	0	0	0	0	0	NA	NA	NA
ENSBTAG00000010317	*IMP5*	0	0	0	0	0	NA	NA	NA
ENSBTAG00000010385	*protein_coding*	0	0	0	0	0	NA	NA	NA
ENSBTAG00000010461	*protein_coding*	0	0	0	0	0	NA	NA	NA
ENSBTAG00000010486	*OR9G1*	0	0	0	0	0	NA	NA	NA
ENSBTAG00000010530	*IAPP*	0	0	0	0	0	NA	NA	NA
ENSBTAG00000010535	*TXNDC8*	0	0	0	0	0	NA	NA	NA
ENSBTAG00000010553	*pseudogene*	0	0	0	0	0	NA	NA	NA
ENSBTAG00000010560	*BT.49633*	0	0	0	0	0	NA	NA	NA
ENSBTAG00000010686	*OR2K2*	0	0	0	0	0	NA	NA	NA
ENSBTAG00000010690	*BT.87929*	0	0	0	0	0	NA	NA	NA
ENSBTAG00000010752	*pseudogene*	0	0	0	0	0	NA	NA	NA
ENSBTAG00000010797	*BT.56247*	0	0	0	0	0	NA	NA	NA
ENSBTAG00000010828	*GZMH*	0	0	0	0	0	NA	NA	NA
ENSBTAG00000010855	*ANKK1*	0	0	0	0	0	NA	NA	NA
ENSBTAG00000010874	*GHSR*	0	0	0	0	0	NA	NA	NA
ENSBTAG00000010889	*FSHB*	0	0	0	0	0	NA	NA	NA
ENSBTAG00000010930	*BT.106517*	0	0	0	0	0	NA	NA	NA
ENSBTAG00000011023	*C5H12orf59*	0	0	0	0	0	NA	NA	NA
ENSBTAG00000011119	*BOSTAUV1R404*	0	0	0	0	0	NA	NA	NA
ENSBTAG00000011144	*protein_coding*	0	0	0	0	0	NA	NA	NA
ENSBTAG00000011160	*BT.59526*	0	0	0	0	0	NA	NA	NA
ENSBTAG00000011191	*protein_coding*	0	0	0	0	0	NA	NA	NA
ENSBTAG00000011233	*GTSF1L*	0	0	0	0	0	NA	NA	NA
ENSBTAG00000011240	*pseudogene*	0	0	0	0	0	NA	NA	NA
ENSBTAG00000011254	*OR2Z1*	0	0	0	0	0	NA	NA	NA
ENSBTAG00000011255	*ACTL9*	0	0	0	0	0	NA	NA	NA
ENSBTAG00000011312	*NKX2-4*	0	0	0	0	0	NA	NA	NA
ENSBTAG00000011441	*pseudogene*	0	0	0	0	0	NA	NA	NA
ENSBTAG00000011457	*pseudogene*	0	0	0	0	0	NA	NA	NA
ENSBTAG00000011508	*H3F3C*	0	0	0	0	0	NA	NA	NA
ENSBTAG00000011510	*OR4K2*	0	0	0	0	0	NA	NA	NA
ENSBTAG00000011521	*protein_coding*	0	0	0	0	0	NA	NA	NA
ENSBTAG00000011631	*YWHAH*	0	0	0	0	0	NA	NA	NA
ENSBTAG00000011660	*MSMB*	0	0	0	0	0	NA	NA	NA
ENSBTAG00000011664	*BT.33471*	0	0	0	0	0	NA	NA	NA
ENSBTAG00000011707	*OR5AS1*	0	0	0	0	0	NA	NA	NA
ENSBTAG00000011740	*OPALIN*	0	0	0	0	0	NA	NA	NA
ENSBTAG00000011871	*BTLA*	0	0	0	0	0	NA	NA	NA
ENSBTAG00000011874	*C9ORF71*	0	0	0	0	0	NA	NA	NA
ENSBTAG00000011925	*protein_coding*	0	0	0	0	0	NA	NA	NA
ENSBTAG00000012084	*BT.104732*	0	0	0	0	0	NA	NA	NA
ENSBTAG00000012116	*HNF4A*	0	0	0	0	0	NA	NA	NA
ENSBTAG00000012149	*BT.93761*	0	0	0	0	0	NA	NA	NA
ENSBTAG00000012166	*TMC3*	0	0	0	0	0	NA	NA	NA
ENSBTAG00000012171	*BT.87463*	0	0	0	0	0	NA	NA	NA
ENSBTAG00000012209	*CLDN24*	0	0	0	0	0	NA	NA	NA
ENSBTAG00000012281	*AWAT2*	0	0	0	0	0	NA	NA	NA
ENSBTAG00000012301	*pseudogene*	0	0	0	0	0	NA	NA	NA
ENSBTAG00000012325	*protein_coding*	0	0	0	0	0	NA	NA	NA
ENSBTAG00000012326	*protein_coding*	0	0	0	0	0	NA	NA	NA
ENSBTAG00000012328	*GHRL*	0	0	0	0	0	NA	NA	NA
ENSBTAG00000012339	*protein_coding*	0	0	0	0	0	NA	NA	NA
ENSBTAG00000012368	*BT.23583*	0	0	0	0	0	NA	NA	NA
ENSBTAG00000012389	*protein_coding*	0	0	0	0	0	NA	NA	NA
ENSBTAG00000012407	*FOXA2*	0	0	0	0	0	NA	NA	NA
ENSBTAG00000012437	*OR6N2*	0	0	0	0	0	NA	NA	NA
ENSBTAG00000012461	*PRSS16*	0	0	0	0	0	NA	NA	NA
ENSBTAG00000012502	*protein_coding*	0	0	0	0	0	NA	NA	NA
ENSBTAG00000012529	*IFNG*	0	0	0	0	0	NA	NA	NA
ENSBTAG00000012540	*GAT*	0	0	0	0	0	NA	NA	NA
ENSBTAG00000012546	*protein_coding*	0	0	0	0	0	NA	NA	NA
ENSBTAG00000012549	*OR4C3*	0	0	0	0	0	NA	NA	NA
ENSBTAG00000012553	*pseudogene*	0	0	0	0	0	NA	NA	NA
ENSBTAG00000012559	*SLC6A18*	0	0	0	0	0	NA	NA	NA

Ensembl gene ID	geneName	counts wildtype horn bud	counts polled horn bud	baseMean	baseMean wildtype horn bud	baseMean polled horn bud	log2FoldChange	pval	padj
ENSBTAG00000012563	*BT.62944*	0	0	0	0	0	NA	NA	NA
ENSBTAG00000012652	*TINAG*	0	0	0	0	0	NA	NA	NA
ENSBTAG00000012710	*GHRH*	0	0	0	0	0	NA	NA	NA
ENSBTAG00000012775	*SUBH2BV*	0	0	0	0	0	NA	NA	NA
ENSBTAG00000012835	*pseudogene*	0	0	0	0	0	NA	NA	NA
ENSBTAG00000012884	*MPTX*	0	0	0	0	0	NA	NA	NA
ENSBTAG00000012886	*OR10J3*	0	0	0	0	0	NA	NA	NA
ENSBTAG00000012956	*protein_coding*	0	0	0	0	0	NA	NA	NA
ENSBTAG00000012971	*protein_coding*	0	0	0	0	0	NA	NA	NA
ENSBTAG00000013003	*INS*	0	0	0	0	0	NA	NA	NA
ENSBTAG00000013026	*SNTN*	0	0	0	0	0	NA	NA	NA
ENSBTAG00000013035	*SIX6*	0	0	0	0	0	NA	NA	NA
ENSBTAG00000013039	*protein_coding*	0	0	0	0	0	NA	NA	NA
ENSBTAG00000013051	*PRM3*	0	0	0	0	0	NA	NA	NA
ENSBTAG00000013055	*BT.76321*	0	0	0	0	0	NA	NA	NA
ENSBTAG00000013077	*SATL1*	0	0	0	0	0	NA	NA	NA
ENSBTAG00000013079	*BORIS*	0	0	0	0	0	NA	NA	NA
ENSBTAG00000013130	*pseudogene*	0	0	0	0	0	NA	NA	NA
ENSBTAG00000013149	*NAA11*	0	0	0	0	0	NA	NA	NA
ENSBTAG00000013156	*protein_coding*	0	0	0	0	0	NA	NA	NA
ENSBTAG00000013170	*CTLA-4*	0	0	0	0	0	NA	NA	NA
ENSBTAG00000013174	*WBP2NL*	0	0	0	0	0	NA	NA	NA
ENSBTAG00000013207	*CAPZA3*	0	0	0	0	0	NA	NA	NA
ENSBTAG00000013214	*BT.88769*	0	0	0	0	0	NA	NA	NA
ENSBTAG00000013255	*protein_coding*	0	0	0	0	0	NA	NA	NA
ENSBTAG00000013262	*BT.39107*	0	0	0	0	0	NA	NA	NA
ENSBTAG00000013345	*protein_coding*	0	0	0	0	0	NA	NA	NA
ENSBTAG00000013349	*pseudogene*	0	0	0	0	0	NA	NA	NA
ENSBTAG00000013352	*BT.74366*	0	0	0	0	0	NA	NA	NA
ENSBTAG00000013384	*protein_coding*	0	0	0	0	0	NA	NA	NA
ENSBTAG00000013398	*protein_coding*	0	0	0	0	0	NA	NA	NA
ENSBTAG00000013415	*CST11*	0	0	0	0	0	NA	NA	NA
ENSBTAG00000013417	*GLYCAM1*	0	0	0	0	0	NA	NA	NA
ENSBTAG00000013445	*OR13F1*	0	0	0	0	0	NA	NA	NA
ENSBTAG00000013450	*FAM71B*	0	0	0	0	0	NA	NA	NA
ENSBTAG00000013481	*CYP4A22*	0	0	0	0	0	NA	NA	NA
ENSBTAG00000013499	*LHX9*	0	0	0	0	0	NA	NA	NA
ENSBTAG00000013503	*protein_coding*	0	0	0	0	0	NA	NA	NA
ENSBTAG00000013551	*NOX3*	0	0	0	0	0	NA	NA	NA
ENSBTAG00000013567	*OR5D13*	0	0	0	0	0	NA	NA	NA
ENSBTAG00000013625	*protein_coding*	0	0	0	0	0	NA	NA	NA
ENSBTAG00000013654	*OR14J1*	0	0	0	0	0	NA	NA	NA
ENSBTAG00000013660	*processed_pseudogene*	0	0	0	0	0	NA	NA	NA
ENSBTAG00000013673	*protein_coding*	0	0	0	0	0	NA	NA	NA
ENSBTAG00000013683	*protein_coding*	0	0	0	0	0	NA	NA	NA
ENSBTAG00000013685	*KRT31*	0	0	0	0	0	NA	NA	NA
ENSBTAG00000013691	*OR2L13*	0	0	0	0	0	NA	NA	NA
ENSBTAG00000013720	*BT.93347*	0	0	0	0	0	NA	NA	NA
ENSBTAG00000013768	*NPSR1*	0	0	0	0	0	NA	NA	NA
ENSBTAG00000013789	*OR6K6*	0	0	0	0	0	NA	NA	NA
ENSBTAG00000013809	*protein_coding*	0	0	0	0	0	NA	NA	NA
ENSBTAG00000013950	*TKDP4*	0	0	0	0	0	NA	NA	NA
ENSBTAG00000013995	*MAGEB3*	0	0	0	0	0	NA	NA	NA
ENSBTAG00000013997	*ART1*	0	0	0	0	0	NA	NA	NA
ENSBTAG00000014008	*NKX2-6*	0	0	0	0	0	NA	NA	NA
ENSBTAG00000014012	*CLUL1*	0	0	0	0	0	NA	NA	NA
ENSBTAG00000014045	*pseudogene*	0	0	0	0	0	NA	NA	NA
ENSBTAG00000014056	*CHRNA6*	0	0	0	0	0	NA	NA	NA
ENSBTAG00000014094	*BT.63318*	0	0	0	0	0	NA	NA	NA
ENSBTAG00000014141	*SPATA4*	0	0	0	0	0	NA	NA	NA
ENSBTAG00000014157	*BT.61788*	0	0	0	0	0	NA	NA	NA
ENSBTAG00000014234	*C10H14orf53*	0	0	0	0	0	NA	NA	NA
ENSBTAG00000014236	*NR2E3*	0	0	0	0	0	NA	NA	NA
ENSBTAG00000014274	*protein_coding*	0	0	0	0	0	NA	NA	NA
ENSBTAG00000014287	*BT.50949*	0	0	0	0	0	NA	NA	NA
ENSBTAG00000014305	*protein_coding*	0	0	0	0	0	NA	NA	NA
ENSBTAG00000014309	*protein_coding*	0	0	0	0	0	NA	NA	NA
ENSBTAG00000014345	*TKDP3*	0	0	0	0	0	NA	NA	NA
ENSBTAG00000014457	*BT.88960*	0	0	0	0	0	NA	NA	NA
ENSBTAG00000014513	*pseudogene*	0	0	0	0	0	NA	NA	NA
ENSBTAG00000014532	*C7ORF34*	0	0	0	0	0	NA	NA	NA
ENSBTAG00000014572	*protein_coding*	0	0	0	0	0	NA	NA	NA

Ensembl gene ID	geneName	counts wildtype horn bud	counts polled horn bud	baseMean	baseMean wildtype horn bud	baseMean polled horn bud	log2FoldChange	pval	padj
ENSBTAG00000014584	*BT.62924*	0	0	0	0	0	NA	NA	NA
ENSBTAG00000014589	*CLDN18*	0	0	0	0	0	NA	NA	NA
ENSBTAG00000014594	*protein_coding*	0	0	0	0	0	NA	NA	NA
ENSBTAG00000014606	*FOXR1*	0	0	0	0	0	NA	NA	NA
ENSBTAG00000014632	*VSX2*	0	0	0	0	0	NA	NA	NA
ENSBTAG00000014634	*pseudogene*	0	0	0	0	0	NA	NA	NA
ENSBTAG00000014657	*C14ORF39*	0	0	0	0	0	NA	NA	NA
ENSBTAG00000014663	*BT.62402*	0	0	0	0	0	NA	NA	NA
ENSBTAG00000014678	*LGB*	0	0	0	0	0	NA	NA	NA
ENSBTAG00000014683	*APOBEC1*	0	0	0	0	0	NA	NA	NA
ENSBTAG00000014704	*HELT*	0	0	0	0	0	NA	NA	NA
ENSBTAG00000014720	*CATSPER1*	0	0	0	0	0	NA	NA	NA
ENSBTAG00000014738	*HOXA11*	0	0	0	0	0	NA	NA	NA
ENSBTAG00000014741	*BT.87811*	0	0	0	0	0	NA	NA	NA
ENSBTAG00000014770	*GPR119*	0	0	0	0	0	NA	NA	NA
ENSBTAG00000014783	*CRYGC*	0	0	0	0	0	NA	NA	NA
ENSBTAG00000014800	*pseudogene*	0	0	0	0	0	NA	NA	NA
ENSBTAG00000014810	*BT.51020*	0	0	0	0	0	NA	NA	NA
ENSBTAG00000014848	*NR0B2*	0	0	0	0	0	NA	NA	NA
ENSBTAG00000014853	*protein_coding*	0	0	0	0	0	NA	NA	NA
ENSBTAG00000014911	*OB*	0	0	0	0	0	NA	NA	NA
ENSBTAG00000014987	*protein_coding*	0	0	0	0	0	NA	NA	NA
ENSBTAG00000015033	*pseudogene*	0	0	0	0	0	NA	NA	NA
ENSBTAG00000015047	*protein_coding*	0	0	0	0	0	NA	NA	NA
ENSBTAG00000015054	*CRYGD*	0	0	0	0	0	NA	NA	NA
ENSBTAG00000015081	*C11H9ORF142*	0	0	0	0	0	NA	NA	NA
ENSBTAG00000015084	*MT4*	0	0	0	0	0	NA	NA	NA
ENSBTAG00000015137	*OR9A4*	0	0	0	0	0	NA	NA	NA
ENSBTAG00000015143	*pseudogene*	0	0	0	0	0	NA	NA	NA
ENSBTAG00000015244	*protein_coding*	0	0	0	0	0	NA	NA	NA
ENSBTAG00000015298	*TNFRSF13B*	0	0	0	0	0	NA	NA	NA
ENSBTAG00000015299	*protein_coding*	0	0	0	0	0	NA	NA	NA
ENSBTAG00000015315	*protein_coding*	0	0	0	0	0	NA	NA	NA
ENSBTAG00000015353	*BT.56220*	0	0	0	0	0	NA	NA	NA
ENSBTAG00000015357	*OR52B4*	0	0	0	0	0	NA	NA	NA
ENSBTAG00000015407	*IL-22*	0	0	0	0	0	NA	NA	NA
ENSBTAG00000015461	*pseudogene*	0	0	0	0	0	NA	NA	NA
ENSBTAG00000015475	*MORC1*	0	0	0	0	0	NA	NA	NA
ENSBTAG00000015493	*BT.33457*	0	0	0	0	0	NA	NA	NA
ENSBTAG00000015517	*protein_coding*	0	0	0	0	0	NA	NA	NA
ENSBTAG00000015558	*SPINK1*	0	0	0	0	0	NA	NA	NA
ENSBTAG00000015574	*RBP7*	0	0	0	0	0	NA	NA	NA
ENSBTAG00000015600	*protein_coding*	0	0	0	0	0	NA	NA	NA
ENSBTAG00000015608	*CYLC2*	0	0	0	0	0	NA	NA	NA
ENSBTAG00000015674	*pseudogene*	0	0	0	0	0	NA	NA	NA
ENSBTAG00000015729	*ADH7*	0	0	0	0	0	NA	NA	NA
ENSBTAG00000015735	*CYP4F2*	0	0	0	0	0	NA	NA	NA
ENSBTAG00000015791	*protein_coding*	0	0	0	0	0	NA	NA	NA
ENSBTAG00000015797	*pseudogene*	0	0	0	0	0	NA	NA	NA
ENSBTAG00000015812	*KRTAP8-1*	0	0	0	0	0	NA	NA	NA
ENSBTAG00000015827	*PRP1*	0	0	0	0	0	NA	NA	NA
ENSBTAG00000015876	*OR10AG1*	0	0	0	0	0	NA	NA	NA
ENSBTAG00000015949	*protein_coding*	0	0	0	0	0	NA	NA	NA
ENSBTAG00000015983	*protein_coding*	0	0	0	0	0	NA	NA	NA
ENSBTAG00000016019	*protein_coding*	0	0	0	0	0	NA	NA	NA
ENSBTAG00000016059	*pseudogene*	0	0	0	0	0	NA	NA	NA
ENSBTAG00000016065	*protein_coding*	0	0	0	0	0	NA	NA	NA
ENSBTAG00000016082	*protein_coding*	0	0	0	0	0	NA	NA	NA
ENSBTAG00000016090	*OR4C46*	0	0	0	0	0	NA	NA	NA
ENSBTAG00000016114	*CABP5*	0	0	0	0	0	NA	NA	NA
ENSBTAG00000016119	*protein_coding*	0	0	0	0	0	NA	NA	NA
ENSBTAG00000016144	*LCN10*	0	0	0	0	0	NA	NA	NA
ENSBTAG00000016146	*protein_coding*	0	0	0	0	0	NA	NA	NA
ENSBTAG00000016153	*protein_coding*	0	0	0	0	0	NA	NA	NA
ENSBTAG00000016159	*NPBWR1*	0	0	0	0	0	NA	NA	NA
ENSBTAG00000016173	*protein_coding*	0	0	0	0	0	NA	NA	NA
ENSBTAG00000016216	*pseudogene*	0	0	0	0	0	NA	NA	NA
ENSBTAG00000016270	*protein_coding*	0	0	0	0	0	NA	NA	NA
ENSBTAG00000016317	*protein_coding*	0	0	0	0	0	NA	NA	NA
ENSBTAG00000016405	*OR4C6*	0	0	0	0	0	NA	NA	NA
ENSBTAG00000016434	*PDILT*	0	0	0	0	0	NA	NA	NA
ENSBTAG00000016624	*HTR3C*	0	0	0	0	0	NA	NA	NA

Ensembl gene ID	geneName	counts wildtype horn bud	counts polled horn bud	baseMean	baseMean wildtype horn bud	baseMean polled horn bud	log2FoldChange	pval	padj
ENSBTAG00000016647	*OR10D3*	0	0	0	0	0	NA	NA	NA
ENSBTAG00000016653	*protein_coding*	0	0	0	0	0	NA	NA	NA
ENSBTAG00000016789	*OR4A16*	0	0	0	0	0	NA	NA	NA
ENSBTAG00000016807	*protein_coding*	0	0	0	0	0	NA	NA	NA
ENSBTAG00000016835	*IL17F*	0	0	0	0	0	NA	NA	NA
ENSBTAG00000016859	*pseudogene*	0	0	0	0	0	NA	NA	NA
ENSBTAG00000016864	*BT.24181*	0	0	0	0	0	NA	NA	NA
ENSBTAG00000016905	*protein_coding*	0	0	0	0	0	NA	NA	NA
ENSBTAG00000016940	*protein_coding*	0	0	0	0	0	NA	NA	NA
ENSBTAG00000016989	*protein_coding*	0	0	0	0	0	NA	NA	NA
ENSBTAG00000016996	*C5H12orf54*	0	0	0	0	0	NA	NA	NA
ENSBTAG00000017000	*IL22RA2*	0	0	0	0	0	NA	NA	NA
ENSBTAG00000017013	*C5ORF50*	0	0	0	0	0	NA	NA	NA
ENSBTAG00000017042	*HAMP*	0	0	0	0	0	NA	NA	NA
ENSBTAG00000017045	*FABP2*	0	0	0	0	0	NA	NA	NA
ENSBTAG00000017054	*protein_coding*	0	0	0	0	0	NA	NA	NA
ENSBTAG00000017098	*BPIFA1*	0	0	0	0	0	NA	NA	NA
ENSBTAG00000017131	*AFP*	0	0	0	0	0	NA	NA	NA
ENSBTAG00000017145	*C1ORF177*	0	0	0	0	0	NA	NA	NA
ENSBTAG00000017166	*C6ORF58*	0	0	0	0	0	NA	NA	NA
ENSBTAG00000017173	*BOSTAUV1R437*	0	0	0	0	0	NA	NA	NA
ENSBTAG00000017199	*GKN2*	0	0	0	0	0	NA	NA	NA
ENSBTAG00000017220	*GH1*	0	0	0	0	0	NA	NA	NA
ENSBTAG00000017227	*BT.88548*	0	0	0	0	0	NA	NA	NA
ENSBTAG00000017301	*AVPR1B*	0	0	0	0	0	NA	NA	NA
ENSBTAG00000017316	*protein_coding*	0	0	0	0	0	NA	NA	NA
ENSBTAG00000017322	*ARL14*	0	0	0	0	0	NA	NA	NA
ENSBTAG00000017345	*BT.88292*	0	0	0	0	0	NA	NA	NA
ENSBTAG00000017396	*protein_coding*	0	0	0	0	0	NA	NA	NA
ENSBTAG00000017431	*BT.87199*	0	0	0	0	0	NA	NA	NA
ENSBTAG00000017481	*pseudogene*	0	0	0	0	0	NA	NA	NA
ENSBTAG00000017495	*OR8D2*	0	0	0	0	0	NA	NA	NA
ENSBTAG00000017531	*FETUB*	0	0	0	0	0	NA	NA	NA
ENSBTAG00000017538	*pseudogene*	0	0	0	0	0	NA	NA	NA
ENSBTAG00000017541	*TBX20*	0	0	0	0	0	NA	NA	NA
ENSBTAG00000017568	*WFDC13*	0	0	0	0	0	NA	NA	NA
ENSBTAG00000017587	*WFDC9*	0	0	0	0	0	NA	NA	NA
ENSBTAG00000017588	*pseudogene*	0	0	0	0	0	NA	NA	NA
ENSBTAG00000017615	*PRLH*	0	0	0	0	0	NA	NA	NA
ENSBTAG00000017647	*protein_coding*	0	0	0	0	0	NA	NA	NA
ENSBTAG00000017725	*OR11L1*	0	0	0	0	0	NA	NA	NA
ENSBTAG00000017735	*protein_coding*	0	0	0	0	0	NA	NA	NA
ENSBTAG00000017778	*OR10R2*	0	0	0	0	0	NA	NA	NA
ENSBTAG00000017800	*DMRT3*	0	0	0	0	0	NA	NA	NA
ENSBTAG00000017884	*KCNV2*	0	0	0	0	0	NA	NA	NA
ENSBTAG00000017912	*OR7G2*	0	0	0	0	0	NA	NA	NA
ENSBTAG00000017916	*pseudogene*	0	0	0	0	0	NA	NA	NA
ENSBTAG00000017924	*protein_coding*	0	0	0	0	0	NA	NA	NA
ENSBTAG00000017929	*ACTRT2*	0	0	0	0	0	NA	NA	NA
ENSBTAG00000017931	*pseudogene*	0	0	0	0	0	NA	NA	NA
ENSBTAG00000017934	*protein_coding*	0	0	0	0	0	NA	NA	NA
ENSBTAG00000017938	*protein_coding*	0	0	0	0	0	NA	NA	NA
ENSBTAG00000017953	*pseudogene*	0	0	0	0	0	NA	NA	NA
ENSBTAG00000017978	*protein_coding*	0	0	0	0	0	NA	NA	NA
ENSBTAG00000017993	*protein_coding*	0	0	0	0	0	NA	NA	NA
ENSBTAG00000018012	*BT.106467*	0	0	0	0	0	NA	NA	NA
ENSBTAG00000018054	*FABP1*	0	0	0	0	0	NA	NA	NA
ENSBTAG00000018085	*GTSF1*	0	0	0	0	0	NA	NA	NA
ENSBTAG00000018122	*protein_coding*	0	0	0	0	0	NA	NA	NA
ENSBTAG00000018182	*protein_coding*	0	0	0	0	0	NA	NA	NA
ENSBTAG00000018195	*protein_coding*	0	0	0	0	0	NA	NA	NA
ENSBTAG00000018221	*protein_coding*	0	0	0	0	0	NA	NA	NA
ENSBTAG00000018228	*SAMD7*	0	0	0	0	0	NA	NA	NA
ENSBTAG00000018259	*protein_coding*	0	0	0	0	0	NA	NA	NA
ENSBTAG00000018262	*DNAJC5G*	0	0	0	0	0	NA	NA	NA
ENSBTAG00000018277	*TBPL2*	0	0	0	0	0	NA	NA	NA
ENSBTAG00000018311	*protein_coding*	0	0	0	0	0	NA	NA	NA
ENSBTAG00000018323	*MAGEA11*	0	0	0	0	0	NA	NA	NA
ENSBTAG00000018346	*TRIM42*	0	0	0	0	0	NA	NA	NA
ENSBTAG00000018365	*CYP2E1*	0	0	0	0	0	NA	NA	NA
ENSBTAG00000018391	*BT.51107*	0	0	0	0	0	NA	NA	NA
ENSBTAG00000018490	*OR2D2*	0	0	0	0	0	NA	NA	NA

Ensembl gene ID	geneName	counts wildtype horn bud	counts polled horn bud	baseMean	baseMean wildtype horn bud	baseMean polled horn bud	log2FoldChange	pval	padj
ENSBTAG00000018503	*protein_coding*	0	0	0	0	0	NA	NA	NA
ENSBTAG00000018507	*protein_coding*	0	0	0	0	0	NA	NA	NA
ENSBTAG00000018556	*MGC137211*	0	0	0	0	0	NA	NA	NA
ENSBTAG00000018580	*SPERT*	0	0	0	0	0	NA	NA	NA
ENSBTAG00000018583	*processed_pseudogene*	0	0	0	0	0	NA	NA	NA
ENSBTAG00000018601	*BT.74372*	0	0	0	0	0	NA	NA	NA
ENSBTAG00000018610	*KRTAP12-2*	0	0	0	0	0	NA	NA	NA
ENSBTAG00000018681	*T*	0	0	0	0	0	NA	NA	NA
ENSBTAG00000018736	*protein_coding*	0	0	0	0	0	NA	NA	NA
ENSBTAG00000018756	*DNAJB3*	0	0	0	0	0	NA	NA	NA
ENSBTAG00000018839	*AWAT1*	0	0	0	0	0	NA	NA	NA
ENSBTAG00000018840	*protein_coding*	0	0	0	0	0	NA	NA	NA
ENSBTAG00000018849	*BT.56893*	0	0	0	0	0	NA	NA	NA
ENSBTAG00000018882	*BSP5*	0	0	0	0	0	NA	NA	NA
ENSBTAG00000018885	*pseudogene*	0	0	0	0	0	NA	NA	NA
ENSBTAG00000018890	*pseudogene*	0	0	0	0	0	NA	NA	NA
ENSBTAG00000018917	*EN2*	0	0	0	0	0	NA	NA	NA
ENSBTAG00000018919	*OR5AU1*	0	0	0	0	0	NA	NA	NA
ENSBTAG00000019003	*TNP1*	0	0	0	0	0	NA	NA	NA
ENSBTAG00000019009	*protein_coding*	0	0	0	0	0	NA	NA	NA
ENSBTAG00000019038	*OR10J5*	0	0	0	0	0	NA	NA	NA
ENSBTAG00000019084	*INHBC*	0	0	0	0	0	NA	NA	NA
ENSBTAG00000019087	*C16orf95*	0	0	0	0	0	NA	NA	NA
ENSBTAG00000019103	*protein_coding*	0	0	0	0	0	NA	NA	NA
ENSBTAG00000019114	*FOXB1*	0	0	0	0	0	NA	NA	NA
ENSBTAG00000019144	*OR6A2*	0	0	0	0	0	NA	NA	NA
ENSBTAG00000019145	*C-MOS*	0	0	0	0	0	NA	NA	NA
ENSBTAG00000019173	*PAG12*	0	0	0	0	0	NA	NA	NA
ENSBTAG00000019199	*MGC133647*	0	0	0	0	0	NA	NA	NA
ENSBTAG00000019200	*BPIFB2*	0	0	0	0	0	NA	NA	NA
ENSBTAG00000019273	*BT.50646*	0	0	0	0	0	NA	NA	NA
ENSBTAG00000019352	*OR5D14*	0	0	0	0	0	NA	NA	NA
ENSBTAG00000019391	*protein_coding*	0	0	0	0	0	NA	NA	NA
ENSBTAG00000019425	*BT.59505*	0	0	0	0	0	NA	NA	NA
ENSBTAG00000019435	*protein_coding*	0	0	0	0	0	NA	NA	NA
ENSBTAG00000019469	*BT.55305*	0	0	0	0	0	NA	NA	NA
ENSBTAG00000019544	*protein_coding*	0	0	0	0	0	NA	NA	NA
ENSBTAG00000019549	*OR51M1*	0	0	0	0	0	NA	NA	NA
ENSBTAG00000019580	*TCL1A*	0	0	0	0	0	NA	NA	NA
ENSBTAG00000019584	*protein_coding*	0	0	0	0	0	NA	NA	NA
ENSBTAG00000019616	*APCS*	0	0	0	0	0	NA	NA	NA
ENSBTAG00000019618	*OR10J3*	0	0	0	0	0	NA	NA	NA
ENSBTAG00000019705	*protein_coding*	0	0	0	0	0	NA	NA	NA
ENSBTAG00000019709	*protein_coding*	0	0	0	0	0	NA	NA	NA
ENSBTAG00000019716	*IL-8*	0	0	0	0	0	NA	NA	NA
ENSBTAG00000019719	*OPRM1*	0	0	0	0	0	NA	NA	NA
ENSBTAG00000019732	*GLRA3*	0	0	0	0	0	NA	NA	NA
ENSBTAG00000019752	*BPIFA2B*	0	0	0	0	0	NA	NA	NA
ENSBTAG00000019764	*APOA5*	0	0	0	0	0	NA	NA	NA
ENSBTAG00000019768	*OR5P3*	0	0	0	0	0	NA	NA	NA
ENSBTAG00000019799	*FCAMR*	0	0	0	0	0	NA	NA	NA
ENSBTAG00000019811	*BT.103776*	0	0	0	0	0	NA	NA	NA
ENSBTAG00000019849	*CABS1*	0	0	0	0	0	NA	NA	NA
ENSBTAG00000019863	*CCDC70*	0	0	0	0	0	NA	NA	NA
ENSBTAG00000019879	*pseudogene*	0	0	0	0	0	NA	NA	NA
ENSBTAG00000019890	*BT.99275*	0	0	0	0	0	NA	NA	NA
ENSBTAG00000019925	*OR2M3*	0	0	0	0	0	NA	NA	NA
ENSBTAG00000019931	*protein_coding*	0	0	0	0	0	NA	NA	NA
ENSBTAG00000019963	*GRK1*	0	0	0	0	0	NA	NA	NA
ENSBTAG00000019997	*TRHR*	0	0	0	0	0	NA	NA	NA
ENSBTAG00000020033	*protein_coding*	0	0	0	0	0	NA	NA	NA
ENSBTAG00000020051	*BT.82181*	0	0	0	0	0	NA	NA	NA
ENSBTAG00000020078	*KRT39*	0	0	0	0	0	NA	NA	NA
ENSBTAG00000020095	*MS4A3*	0	0	0	0	0	NA	NA	NA
ENSBTAG00000020183	*TSGA13*	0	0	0	0	0	NA	NA	NA
ENSBTAG00000020184	*AQP8*	0	0	0	0	0	NA	NA	NA
ENSBTAG00000020191	*FEV*	0	0	0	0	0	NA	NA	NA
ENSBTAG00000020195	*ANKRD34C*	0	0	0	0	0	NA	NA	NA
ENSBTAG00000020201	*S100Z*	0	0	0	0	0	NA	NA	NA
ENSBTAG00000020249	*BT.54390*	0	0	0	0	0	NA	NA	NA
ENSBTAG00000020251	*LYPD4*	0	0	0	0	0	NA	NA	NA
ENSBTAG00000020299	*OPN1LW*	0	0	0	0	0	NA	NA	NA

Ensembl gene ID	geneName	counts wildtype horn bud	counts polled horn bud	baseMean	baseMean wildtype horn bud	baseMean polled horn bud	log2FoldChange	pval	padj
ENSBTAG00000020300	*TEX28*	0	0	0	0	0	NA	NA	NA
ENSBTAG00000020351	*SLC34A1*	0	0	0	0	0	NA	NA	NA
ENSBTAG00000020364	*protein_coding*	0	0	0	0	0	NA	NA	NA
ENSBTAG00000020404	*FTCD*	0	0	0	0	0	NA	NA	NA
ENSBTAG00000020460	*BT.104772*	0	0	0	0	0	NA	NA	NA
ENSBTAG00000020513	*protein_coding*	0	0	0	0	0	NA	NA	NA
ENSBTAG00000020550	*protein_coding*	0	0	0	0	0	NA	NA	NA
ENSBTAG00000020558	*APOC2*	0	0	0	0	0	NA	NA	NA
ENSBTAG00000020564	*BT.105675*	0	0	0	0	0	NA	NA	NA
ENSBTAG00000020615	*CCDC105*	0	0	0	0	0	NA	NA	NA
ENSBTAG00000020660	*protein_coding*	0	0	0	0	0	NA	NA	NA
ENSBTAG00000020672	*NEU2*	0	0	0	0	0	NA	NA	NA
ENSBTAG00000020673	*protein_coding*	0	0	0	0	0	NA	NA	NA
ENSBTAG00000020723	*pseudogene*	0	0	0	0	0	NA	NA	NA
ENSBTAG00000020725	*BT.28014*	0	0	0	0	0	NA	NA	NA
ENSBTAG00000020746	*protein_coding*	0	0	0	0	0	NA	NA	NA
ENSBTAG00000020765	*BT.87236*	0	0	0	0	0	NA	NA	NA
ENSBTAG00000020767	*SH2D1B*	0	0	0	0	0	NA	NA	NA
ENSBTAG00000020794	*protein_coding*	0	0	0	0	0	NA	NA	NA
ENSBTAG00000020838	*protein_coding*	0	0	0	0	0	NA	NA	NA
ENSBTAG00000020849	*pseudogene*	0	0	0	0	0	NA	NA	NA
ENSBTAG00000020883	*IL2*	0	0	0	0	0	NA	NA	NA
ENSBTAG00000020916	*NANOG*	0	0	0	0	0	NA	NA	NA
ENSBTAG00000020918	*NKX1-2*	0	0	0	0	0	NA	NA	NA
ENSBTAG00000020952	*OR52E6*	0	0	0	0	0	NA	NA	NA
ENSBTAG00000021098	*protein_coding*	0	0	0	0	0	NA	NA	NA
ENSBTAG00000021104	*protein_coding*	0	0	0	0	0	NA	NA	NA
ENSBTAG00000021122	*protein_coding*	0	0	0	0	0	NA	NA	NA
ENSBTAG00000021147	*protein_coding*	0	0	0	0	0	NA	NA	NA
ENSBTAG00000021150	*protein_coding*	0	0	0	0	0	NA	NA	NA
ENSBTAG00000021175	*IMPG1*	0	0	0	0	0	NA	NA	NA
ENSBTAG00000021185	*CRX*	0	0	0	0	0	NA	NA	NA
ENSBTAG00000021195	*A1CF*	0	0	0	0	0	NA	NA	NA
ENSBTAG00000021233	*OR5B17*	0	0	0	0	0	NA	NA	NA
ENSBTAG00000021280	*TMPRSS3*	0	0	0	0	0	NA	NA	NA
ENSBTAG00000021298	*METTL11B*	0	0	0	0	0	NA	NA	NA
ENSBTAG00000021423	*C20ORF201*	0	0	0	0	0	NA	NA	NA
ENSBTAG00000021439	*protein_coding*	0	0	0	0	0	NA	NA	NA
ENSBTAG00000021446	*protein_coding*	0	0	0	0	0	NA	NA	NA
ENSBTAG00000021460	*protein_coding*	0	0	0	0	0	NA	NA	NA
ENSBTAG00000021493	*PRM1*	0	0	0	0	0	NA	NA	NA
ENSBTAG00000021566	*PAX2*	0	0	0	0	0	NA	NA	NA
ENSBTAG00000021603	*pseudogene*	0	0	0	0	0	NA	NA	NA
ENSBTAG00000021621	*protein_coding*	0	0	0	0	0	NA	NA	NA
ENSBTAG00000021624	*protein_coding*	0	0	0	0	0	NA	NA	NA
ENSBTAG00000021816	*TMEM239*	0	0	0	0	0	NA	NA	NA
ENSBTAG00000021817	*C20orf141*	0	0	0	0	0	NA	NA	NA
ENSBTAG00000021886	*GRK7*	0	0	0	0	0	NA	NA	NA
ENSBTAG00000021914	*pseudogene*	0	0	0	0	0	NA	NA	NA
ENSBTAG00000021954	*pseudogene*	0	0	0	0	0	NA	NA	NA
ENSBTAG00000021987	*BT.46904*	0	0	0	0	0	NA	NA	NA
ENSBTAG00000022030	*C22ORF15*	0	0	0	0	0	NA	NA	NA
ENSBTAG00000022183	*pseudogene*	0	0	0	0	0	NA	NA	NA
ENSBTAG00000022205	*BT.19825*	0	0	0	0	0	NA	NA	NA
ENSBTAG00000022229	*BT.60935*	0	0	0	0	0	NA	NA	NA
ENSBTAG00000022246	*C29H11orf86*	0	0	0	0	0	NA	NA	NA
ENSBTAG00000022306	*pseudogene*	0	0	0	0	0	NA	NA	NA
ENSBTAG00000022339	*protein_coding*	0	0	0	0	0	NA	NA	NA
ENSBTAG00000022348	*PAG3*	0	0	0	0	0	NA	NA	NA
ENSBTAG00000022368	*protein_coding*	0	0	0	0	0	NA	NA	NA
ENSBTAG00000022376	*protein_coding*	0	0	0	0	0	NA	NA	NA
ENSBTAG00000022378	*protein_coding*	0	0	0	0	0	NA	NA	NA
ENSBTAG00000022384	*protein_coding*	0	0	0	0	0	NA	NA	NA
ENSBTAG00000022395	*protein_coding*	0	0	0	0	0	NA	NA	NA
ENSBTAG00000022466	*C18ORF63*	0	0	0	0	0	NA	NA	NA
ENSBTAG00000022501	*BT.74574*	0	0	0	0	0	NA	NA	NA
ENSBTAG00000022502	*protein_coding*	0	0	0	0	0	NA	NA	NA
ENSBTAG00000022504	*protein_coding*	0	0	0	0	0	NA	NA	NA
ENSBTAG00000022528	*protein_coding*	0	0	0	0	0	NA	NA	NA
ENSBTAG00000022534	*RPS4XP21*	0	0	0	0	0	NA	NA	NA
ENSBTAG00000022556	*OR10K1*	0	0	0	0	0	NA	NA	NA
ENSBTAG00000022570	*protein_coding*	0	0	0	0	0	NA	NA	NA

Ensembl gene ID	geneName	counts wildtype horn bud	counts polled horn bud	baseMean	baseMean wildtype horn bud	baseMean polled horn bud	log2FoldChange	pval	padj
ENSBTAG00000022571	*BT.92662*	0	0	0	0	0	NA	NA	NA
ENSBTAG00000022603	*pseudogene*	0	0	0	0	0	NA	NA	NA
ENSBTAG00000022640	*OR7G1*	0	0	0	0	0	NA	NA	NA
ENSBTAG00000022663	*protein_coding*	0	0	0	0	0	NA	NA	NA
ENSBTAG00000022684	*protein_coding*	0	0	0	0	0	NA	NA	NA
ENSBTAG00000022690	*protein_coding*	0	0	0	0	0	NA	NA	NA
ENSBTAG00000022759	*BT.65105*	0	0	0	0	0	NA	NA	NA
ENSBTAG00000022767	*protein_coding*	0	0	0	0	0	NA	NA	NA
ENSBTAG00000022787	*protein_coding*	0	0	0	0	0	NA	NA	NA
ENSBTAG00000022809	*protein_coding*	0	0	0	0	0	NA	NA	NA
ENSBTAG00000022826	*RAX*	0	0	0	0	0	NA	NA	NA
ENSBTAG00000022858	*protein_coding*	0	0	0	0	0	NA	NA	NA
ENSBTAG00000022861	*KLRJ1*	0	0	0	0	0	NA	NA	NA
ENSBTAG00000022908	*protein_coding*	0	0	0	0	0	NA	NA	NA
ENSBTAG00000022915	*protein_coding*	0	0	0	0	0	NA	NA	NA
ENSBTAG00000022937	*BT.97877*	0	0	0	0	0	NA	NA	NA
ENSBTAG00000022947	*processed_pseudogene*	0	0	0	0	0	NA	NA	NA
ENSBTAG00000022971	*protein_coding*	0	0	0	0	0	NA	NA	NA
ENSBTAG00000022990	*pseudogene*	0	0	0	0	0	NA	NA	NA
ENSBTAG00000023000	*protein_coding*	0	0	0	0	0	NA	NA	NA
ENSBTAG00000023023	*ANTXRL*	0	0	0	0	0	NA	NA	NA
ENSBTAG00000023079	*protein_coding*	0	0	0	0	0	NA	NA	NA
ENSBTAG00000023125	*OR56A4*	0	0	0	0	0	NA	NA	NA
ENSBTAG00000023192	*GDF3*	0	0	0	0	0	NA	NA	NA
ENSBTAG00000023202	*protein_coding*	0	0	0	0	0	NA	NA	NA
ENSBTAG00000023247	*protein_coding*	0	0	0	0	0	NA	NA	NA
ENSBTAG00000023258	*BT.76070*	0	0	0	0	0	NA	NA	NA
ENSBTAG00000023300	*protein_coding*	0	0	0	0	0	NA	NA	NA
ENSBTAG00000023301	*protein_coding*	0	0	0	0	0	NA	NA	NA
ENSBTAG00000023310	*protein_coding*	0	0	0	0	0	NA	NA	NA
ENSBTAG00000023349	*BOSTAUV1R426*	0	0	0	0	0	NA	NA	NA
ENSBTAG00000023393	*AIRE*	0	0	0	0	0	NA	NA	NA
ENSBTAG00000023412	*IZUMO3*	0	0	0	0	0	NA	NA	NA
ENSBTAG00000023431	*protein_coding*	0	0	0	0	0	NA	NA	NA
ENSBTAG00000023434	*BSP1*	0	0	0	0	0	NA	NA	NA
ENSBTAG00000023447	*protein_coding*	0	0	0	0	0	NA	NA	NA
ENSBTAG00000023507	*protein_coding*	0	0	0	0	0	NA	NA	NA
ENSBTAG00000023511	*protein_coding*	0	0	0	0	0	NA	NA	NA
ENSBTAG00000023535	*protein_coding*	0	0	0	0	0	NA	NA	NA
ENSBTAG00000023541	*C6ORF10*	0	0	0	0	0	NA	NA	NA
ENSBTAG00000023542	*protein_coding*	0	0	0	0	0	NA	NA	NA
ENSBTAG00000023546	*FAM186A*	0	0	0	0	0	NA	NA	NA
ENSBTAG00000023549	*CYP2C19*	0	0	0	0	0	NA	NA	NA
ENSBTAG00000023556	*OR10K2*	0	0	0	0	0	NA	NA	NA
ENSBTAG00000023608	*pseudogene*	0	0	0	0	0	NA	NA	NA
ENSBTAG00000023623	*protein_coding*	0	0	0	0	0	NA	NA	NA
ENSBTAG00000023668	*protein_coding*	0	0	0	0	0	NA	NA	NA
ENSBTAG00000023748	*protein_coding*	0	0	0	0	0	NA	NA	NA
ENSBTAG00000023797	*PRDM12*	0	0	0	0	0	NA	NA	NA
ENSBTAG00000023801	*BT.61873*	0	0	0	0	0	NA	NA	NA
ENSBTAG00000023820	*protein_coding*	0	0	0	0	0	NA	NA	NA
ENSBTAG00000023912	*protein_coding*	0	0	0	0	0	NA	NA	NA
ENSBTAG00000023918	*protein_coding*	0	0	0	0	0	NA	NA	NA
ENSBTAG00000023987	*LRIT2*	0	0	0	0	0	NA	NA	NA
ENSBTAG00000024003	*protein_coding*	0	0	0	0	0	NA	NA	NA
ENSBTAG00000024019	*protein_coding*	0	0	0	0	0	NA	NA	NA
ENSBTAG00000024041	*protein_coding*	0	0	0	0	0	NA	NA	NA
ENSBTAG00000024061	*NOTO*	0	0	0	0	0	NA	NA	NA
ENSBTAG00000024065	*TAS2R38*	0	0	0	0	0	NA	NA	NA
ENSBTAG00000024070	*pseudogene*	0	0	0	0	0	NA	NA	NA
ENSBTAG00000024080	*protein_coding*	0	0	0	0	0	NA	NA	NA
ENSBTAG00000024085	*pseudogene*	0	0	0	0	0	NA	NA	NA
ENSBTAG00000024139	*OR4S2*	0	0	0	0	0	NA	NA	NA
ENSBTAG00000024141	*pseudogene*	0	0	0	0	0	NA	NA	NA
ENSBTAG00000024153	*protein_coding*	0	0	0	0	0	NA	NA	NA
ENSBTAG00000024169	*BT.99247*	0	0	0	0	0	NA	NA	NA
ENSBTAG00000024210	*protein_coding*	0	0	0	0	0	NA	NA	NA
ENSBTAG00000024230	*protein_coding*	0	0	0	0	0	NA	NA	NA
ENSBTAG00000024231	*protein_coding*	0	0	0	0	0	NA	NA	NA
ENSBTAG00000024242	*protein_coding*	0	0	0	0	0	NA	NA	NA
ENSBTAG00000024341	*HOXA6*	0	0	0	0	0	NA	NA	NA
ENSBTAG00000024397	*protein_coding*	0	0	0	0	0	NA	NA	NA

Ensembl gene ID	geneName	counts wildtype horn bud	counts polled horn bud	baseMean	baseMean wildtype horn bud	baseMean polled horn bud	log2FoldChange	pval	padj
ENSBTAG00000024423	*protein_coding*	0	0	0	0	0	NA	NA	NA
ENSBTAG00000024437	*BT.54514*	0	0	0	0	0	NA	NA	NA
ENSBTAG00000024445	*pseudogene*	0	0	0	0	0	NA	NA	NA
ENSBTAG00000024496	*ELA2A*	0	0	0	0	0	NA	NA	NA
ENSBTAG00000024530	*pseudogene*	0	0	0	0	0	NA	NA	NA
ENSBTAG00000024582	*RPL10L*	0	0	0	0	0	NA	NA	NA
ENSBTAG00000024593	*SPZ1*	0	0	0	0	0	NA	NA	NA
ENSBTAG00000024607	*OR6C75*	0	0	0	0	0	NA	NA	NA
ENSBTAG00000024611	*BT.83780*	0	0	0	0	0	NA	NA	NA
ENSBTAG00000024620	*pseudogene*	0	0	0	0	0	NA	NA	NA
ENSBTAG00000024653	*OR2T1*	0	0	0	0	0	NA	NA	NA
ENSBTAG00000024659	*protein_coding*	0	0	0	0	0	NA	NA	NA
ENSBTAG00000024668	*pseudogene*	0	0	0	0	0	NA	NA	NA
ENSBTAG00000024683	*STATH*	0	0	0	0	0	NA	NA	NA
ENSBTAG00000024691	*OR6C1*	0	0	0	0	0	NA	NA	NA
ENSBTAG00000024700	*BT.88685*	0	0	0	0	0	NA	NA	NA
ENSBTAG00000024713	*protein_coding*	0	0	0	0	0	NA	NA	NA
ENSBTAG00000024731	*processed_pseudogene*	0	0	0	0	0	NA	NA	NA
ENSBTAG00000024752	*OR10G3*	0	0	0	0	0	NA	NA	NA
ENSBTAG00000024769	*protein_coding*	0	0	0	0	0	NA	NA	NA
ENSBTAG00000024771	*BT.105910*	0	0	0	0	0	NA	NA	NA
ENSBTAG00000024788	*protein_coding*	0	0	0	0	0	NA	NA	NA
ENSBTAG00000024791	*BT.88149*	0	0	0	0	0	NA	NA	NA
ENSBTAG00000024798	*protein_coding*	0	0	0	0	0	NA	NA	NA
ENSBTAG00000024806	*protein_coding*	0	0	0	0	0	NA	NA	NA
ENSBTAG00000024809	*MGC137030*	0	0	0	0	0	NA	NA	NA
ENSBTAG00000024811	*protein_coding*	0	0	0	0	0	NA	NA	NA
ENSBTAG00000024841	*protein_coding*	0	0	0	0	0	NA	NA	NA
ENSBTAG00000024891	*protein_coding*	0	0	0	0	0	NA	NA	NA
ENSBTAG00000024895	*protein_coding*	0	0	0	0	0	NA	NA	NA
ENSBTAG00000024899	*BT.50537*	0	0	0	0	0	NA	NA	NA
ENSBTAG00000024952	*OR5AK2*	0	0	0	0	0	NA	NA	NA
ENSBTAG00000025068	*protein_coding*	0	0	0	0	0	NA	NA	NA
ENSBTAG00000025109	*pseudogene*	0	0	0	0	0	NA	NA	NA
ENSBTAG00000025110	*pseudogene*	0	0	0	0	0	NA	NA	NA
ENSBTAG00000025113	*protein_coding*	0	0	0	0	0	NA	NA	NA
ENSBTAG00000025192	*MGC134473*	0	0	0	0	0	NA	NA	NA
ENSBTAG00000025202	*pseudogene*	0	0	0	0	0	NA	NA	NA
ENSBTAG00000025266	*BT.56258*	0	0	0	0	0	NA	NA	NA
ENSBTAG00000025288	*protein_coding*	0	0	0	0	0	NA	NA	NA
ENSBTAG00000025293	*OR2W3*	0	0	0	0	0	NA	NA	NA
ENSBTAG00000025302	*PRP6*	0	0	0	0	0	NA	NA	NA
ENSBTAG00000025304	*PRP5*	0	0	0	0	0	NA	NA	NA
ENSBTAG00000025307	*pseudogene*	0	0	0	0	0	NA	NA	NA
ENSBTAG00000025314	*PRP8*	0	0	0	0	0	NA	NA	NA
ENSBTAG00000025317	*PRP7*	0	0	0	0	0	NA	NA	NA
ENSBTAG00000025430	*pseudogene*	0	0	0	0	0	NA	NA	NA
ENSBTAG00000025651	*protein_coding*	0	0	0	0	0	NA	NA	NA
ENSBTAG00000025663	*SRN*	0	0	0	0	0	NA	NA	NA
ENSBTAG00000025720	*KLF17*	0	0	0	0	0	NA	NA	NA
ENSBTAG00000025748	*BT.102568*	0	0	0	0	0	NA	NA	NA
ENSBTAG00000025847	*protein_coding*	0	0	0	0	0	NA	NA	NA
ENSBTAG00000025882	*HIST1H2BA*	0	0	0	0	0	NA	NA	NA
ENSBTAG00000025908	*protein_coding*	0	0	0	0	0	NA	NA	NA
ENSBTAG00000025952	*BT.72861*	0	0	0	0	0	NA	NA	NA
ENSBTAG00000025954	*ADAM20*	0	0	0	0	0	NA	NA	NA
ENSBTAG00000026016	*PAG17*	0	0	0	0	0	NA	NA	NA
ENSBTAG00000026030	*protein_coding*	0	0	0	0	0	NA	NA	NA
ENSBTAG00000026034	*pseudogene*	0	0	0	0	0	NA	NA	NA
ENSBTAG00000026065	*OR4P4*	0	0	0	0	0	NA	NA	NA
ENSBTAG00000026068	*protein_coding*	0	0	0	0	0	NA	NA	NA
ENSBTAG00000026078	*protein_coding*	0	0	0	0	0	NA	NA	NA
ENSBTAG00000026085	*ZP4*	0	0	0	0	0	NA	NA	NA
ENSBTAG00000026133	*protein_coding*	0	0	0	0	0	NA	NA	NA
ENSBTAG00000026148	*protein_coding*	0	0	0	0	0	NA	NA	NA
ENSBTAG00000026157	*pseudogene*	0	0	0	0	0	NA	NA	NA
ENSBTAG00000026163	*protein_coding*	0	0	0	0	0	NA	NA	NA
ENSBTAG00000026180	*protein_coding*	0	0	0	0	0	NA	NA	NA
ENSBTAG00000026185	*protein_coding*	0	0	0	0	0	NA	NA	NA
ENSBTAG00000026231	*pseudogene*	0	0	0	0	0	NA	NA	NA
ENSBTAG00000026250	*PDHA2*	0	0	0	0	0	NA	NA	NA
ENSBTAG00000026259	*protein_coding*	0	0	0	0	0	NA	NA	NA

Ensembl gene ID	geneName	counts wildtype horn bud	counts polled horn bud	baseMean	baseMean wildtype horn bud	baseMean polled horn bud	log2FoldChange	pval	padj
ENSBTAG00000026322	*protein_coding*	0	0	0	0	0	NA	NA	NA
ENSBTAG00000026323	*LYSB*	0	0	0	0	0	NA	NA	NA
ENSBTAG00000026325	*ZG16*	0	0	0	0	0	NA	NA	NA
ENSBTAG00000026441	*protein_coding*	0	0	0	0	0	NA	NA	NA
ENSBTAG00000026451	*processed_pseudogene*	0	0	0	0	0	NA	NA	NA
ENSBTAG00000026515	*protein_coding*	0	0	0	0	0	NA	NA	NA
ENSBTAG00000026543	*protein_coding*	0	0	0	0	0	NA	NA	NA
ENSBTAG00000026548	*protein_coding*	0	0	0	0	0	NA	NA	NA
ENSBTAG00000026633	*pseudogene*	0	0	0	0	0	NA	NA	NA
ENSBTAG00000026666	*MGC137014*	0	0	0	0	0	NA	NA	NA
ENSBTAG00000026822	*protein_coding*	0	0	0	0	0	NA	NA	NA
ENSBTAG00000026826	*protein_coding*	0	0	0	0	0	NA	NA	NA
ENSBTAG00000026859	*OR3A1*	0	0	0	0	0	NA	NA	NA
ENSBTAG00000026861	*RPL3*	0	0	0	0	0	NA	NA	NA
ENSBTAG00000026917	*BT.88451*	0	0	0	0	0	NA	NA	NA
ENSBTAG00000026922	*protein_coding*	0	0	0	0	0	NA	NA	NA
ENSBTAG00000026924	*protein_coding*	0	0	0	0	0	NA	NA	NA
ENSBTAG00000026929	*protein_coding*	0	0	0	0	0	NA	NA	NA
ENSBTAG00000027113	*protein_coding*	0	0	0	0	0	NA	NA	NA
ENSBTAG00000027162	*BT.47403*	0	0	0	0	0	NA	NA	NA
ENSBTAG00000027170	*protein_coding*	0	0	0	0	0	NA	NA	NA
ENSBTAG00000027223	*protein_coding*	0	0	0	0	0	NA	NA	NA
ENSBTAG00000027241	*protein_coding*	0	0	0	0	0	NA	NA	NA
ENSBTAG00000027245	*protein_coding*	0	0	0	0	0	NA	NA	NA
ENSBTAG00000027251	*protein_coding*	0	0	0	0	0	NA	NA	NA
ENSBTAG00000027275	*protein_coding*	0	0	0	0	0	NA	NA	NA
ENSBTAG00000027328	*protein_coding*	0	0	0	0	0	NA	NA	NA
ENSBTAG00000027373	*pseudogene*	0	0	0	0	0	NA	NA	NA
ENSBTAG00000027383	*DEFB122*	0	0	0	0	0	NA	NA	NA
ENSBTAG00000027384	*DEFB122A*	0	0	0	0	0	NA	NA	NA
ENSBTAG00000027419	*protein_coding*	0	0	0	0	0	NA	NA	NA
ENSBTAG00000027430	*protein_coding*	0	0	0	0	0	NA	NA	NA
ENSBTAG00000027443	*OR52L1*	0	0	0	0	0	NA	NA	NA
ENSBTAG00000027489	*OR4D6*	0	0	0	0	0	NA	NA	NA
ENSBTAG00000027490	*OR5A1*	0	0	0	0	0	NA	NA	NA
ENSBTAG00000027531	*protein_coding*	0	0	0	0	0	NA	NA	NA
ENSBTAG00000027637	*protein_coding*	0	0	0	0	0	NA	NA	NA
ENSBTAG00000027651	*OR51I2*	0	0	0	0	0	NA	NA	NA
ENSBTAG00000027653	*pseudogene*	0	0	0	0	0	NA	NA	NA
ENSBTAG00000027699	*protein_coding*	0	0	0	0	0	NA	NA	NA
ENSBTAG00000027723	*protein_coding*	0	0	0	0	0	NA	NA	NA
ENSBTAG00000027768	*pseudogene*	0	0	0	0	0	NA	NA	NA
ENSBTAG00000027782	*OR5K1*	0	0	0	0	0	NA	NA	NA
ENSBTAG00000027825	*protein_coding*	0	0	0	0	0	NA	NA	NA
ENSBTAG00000027865	*GZMA*	0	0	0	0	0	NA	NA	NA
ENSBTAG00000027889	*OR52N4*	0	0	0	0	0	NA	NA	NA
ENSBTAG00000027911	*OR2G3*	0	0	0	0	0	NA	NA	NA
ENSBTAG00000027923	*pseudogene*	0	0	0	0	0	NA	NA	NA
ENSBTAG00000027942	*protein_coding*	0	0	0	0	0	NA	NA	NA
ENSBTAG00000027949	*protein_coding*	0	0	0	0	0	NA	NA	NA
ENSBTAG00000027955	*protein_coding*	0	0	0	0	0	NA	NA	NA
ENSBTAG00000027978	*BT.89092*	0	0	0	0	0	NA	NA	NA
ENSBTAG00000028022	*5S_rRNA*	0	0	0	0	0	NA	NA	NA
ENSBTAG00000028036	*U1*	0	0	0	0	0	NA	NA	NA
ENSBTAG00000028043	*U1*	0	0	0	0	0	NA	NA	NA
ENSBTAG00000028061	*5S_rRNA*	0	0	0	0	0	NA	NA	NA
ENSBTAG00000028065	*5S_rRNA*	0	0	0	0	0	NA	NA	NA
ENSBTAG00000028103	*5S_rRNA*	0	0	0	0	0	NA	NA	NA
ENSBTAG00000028105	*U2*	0	0	0	0	0	NA	NA	NA
ENSBTAG00000028119	*U1*	0	0	0	0	0	NA	NA	NA
ENSBTAG00000028219	*5S_rRNA*	0	0	0	0	0	NA	NA	NA
ENSBTAG00000028255	*5S_rRNA*	0	0	0	0	0	NA	NA	NA
ENSBTAG00000028336	*5S_rRNA*	0	0	0	0	0	NA	NA	NA
ENSBTAG00000028358	*Vault*	0	0	0	0	0	NA	NA	NA
ENSBTAG00000028373	*U1*	0	0	0	0	0	NA	NA	NA
ENSBTAG00000028375	*5S_rRNA*	0	0	0	0	0	NA	NA	NA
ENSBTAG00000028457	*5S_rRNA*	0	0	0	0	0	NA	NA	NA
ENSBTAG00000028463	*5S_rRNA*	0	0	0	0	0	NA	NA	NA
ENSBTAG00000028470	*5S_rRNA*	0	0	0	0	0	NA	NA	NA
ENSBTAG00000028471	*5S_rRNA*	0	0	0	0	0	NA	NA	NA
ENSBTAG00000028498	*5S_rRNA*	0	0	0	0	0	NA	NA	NA
ENSBTAG00000028556	*U1*	0	0	0	0	0	NA	NA	NA

Ensembl gene ID	geneName	counts wildtype horn bud	counts polled horn bud	baseMean	baseMean wildtype horn bud	baseMean polled horn bud	log2FoldChange	pval	padj
ENSBTAG00000028572	*5S_rRNA*	0	0	0	0	0	NA	NA	NA
ENSBTAG00000028575	*U1*	0	0	0	0	0	NA	NA	NA
ENSBTAG00000028582	*U1*	0	0	0	0	0	NA	NA	NA
ENSBTAG00000028588	*5S_rRNA*	0	0	0	0	0	NA	NA	NA
ENSBTAG00000028620	*5S_rRNA*	0	0	0	0	0	NA	NA	NA
ENSBTAG00000028628	*U1*	0	0	0	0	0	NA	NA	NA
ENSBTAG00000028631	*5S_rRNA*	0	0	0	0	0	NA	NA	NA
ENSBTAG00000028641	*5S_rRNA*	0	0	0	0	0	NA	NA	NA
ENSBTAG00000028668	*5S_rRNA*	0	0	0	0	0	NA	NA	NA
ENSBTAG00000028682	*5S_rRNA*	0	0	0	0	0	NA	NA	NA
ENSBTAG00000028683	*5S_rRNA*	0	0	0	0	0	NA	NA	NA
ENSBTAG00000028696	*U3*	0	0	0	0	0	NA	NA	NA
ENSBTAG00000028700	*5S_rRNA*	0	0	0	0	0	NA	NA	NA
ENSBTAG00000028704	*5S_rRNA*	0	0	0	0	0	NA	NA	NA
ENSBTAG00000028707	*U3*	0	0	0	0	0	NA	NA	NA
ENSBTAG00000028794	*5S_rRNA*	0	0	0	0	0	NA	NA	NA
ENSBTAG00000028811	*U3*	0	0	0	0	0	NA	NA	NA
ENSBTAG00000028813	*5S_rRNA*	0	0	0	0	0	NA	NA	NA
ENSBTAG00000028814	*5S_rRNA*	0	0	0	0	0	NA	NA	NA
ENSBTAG00000028847	*5S_rRNA*	0	0	0	0	0	NA	NA	NA
ENSBTAG00000028857	*5S_rRNA*	0	0	0	0	0	NA	NA	NA
ENSBTAG00000028860	*5S_rRNA*	0	0	0	0	0	NA	NA	NA
ENSBTAG00000028880	*5S_rRNA*	0	0	0	0	0	NA	NA	NA
ENSBTAG00000028889	*U1*	0	0	0	0	0	NA	NA	NA
ENSBTAG00000028898	*5S_rRNA*	0	0	0	0	0	NA	NA	NA
ENSBTAG00000028919	*5S_rRNA*	0	0	0	0	0	NA	NA	NA
ENSBTAG00000028938	*5S_rRNA*	0	0	0	0	0	NA	NA	NA
ENSBTAG00000028976	*5S_rRNA*	0	0	0	0	0	NA	NA	NA
ENSBTAG00000028990	*U1*	0	0	0	0	0	NA	NA	NA
ENSBTAG00000029040	*5S_rRNA*	0	0	0	0	0	NA	NA	NA
ENSBTAG00000029080	*5S_rRNA*	0	0	0	0	0	NA	NA	NA
ENSBTAG00000029086	*5S_rRNA*	0	0	0	0	0	NA	NA	NA
ENSBTAG00000029106	*5S_rRNA*	0	0	0	0	0	NA	NA	NA
ENSBTAG00000029114	*5S_rRNA*	0	0	0	0	0	NA	NA	NA
ENSBTAG00000029115	*5S_rRNA*	0	0	0	0	0	NA	NA	NA
ENSBTAG00000029126	*5S_rRNA*	0	0	0	0	0	NA	NA	NA
ENSBTAG00000029127	*5S_rRNA*	0	0	0	0	0	NA	NA	NA
ENSBTAG00000029159	*5S_rRNA*	0	0	0	0	0	NA	NA	NA
ENSBTAG00000029168	*U3*	0	0	0	0	0	NA	NA	NA
ENSBTAG00000029179	*5S_rRNA*	0	0	0	0	0	NA	NA	NA
ENSBTAG00000029209	*5S_rRNA*	0	0	0	0	0	NA	NA	NA
ENSBTAG00000029250	*5S_rRNA*	0	0	0	0	0	NA	NA	NA
ENSBTAG00000029292	*5S_rRNA*	0	0	0	0	0	NA	NA	NA
ENSBTAG00000029303	*5S_rRNA*	0	0	0	0	0	NA	NA	NA
ENSBTAG00000029316	*5S_rRNA*	0	0	0	0	0	NA	NA	NA
ENSBTAG00000029343	*5S_rRNA*	0	0	0	0	0	NA	NA	NA
ENSBTAG00000029403	*U1*	0	0	0	0	0	NA	NA	NA
ENSBTAG00000029461	*5S_rRNA*	0	0	0	0	0	NA	NA	NA
ENSBTAG00000029474	*5S_rRNA*	0	0	0	0	0	NA	NA	NA
ENSBTAG00000029491	*5S_rRNA*	0	0	0	0	0	NA	NA	NA
ENSBTAG00000029544	*U2*	0	0	0	0	0	NA	NA	NA
ENSBTAG00000029569	*5S_rRNA*	0	0	0	0	0	NA	NA	NA
ENSBTAG00000029576	*5S_rRNA*	0	0	0	0	0	NA	NA	NA
ENSBTAG00000029583	*5S_rRNA*	0	0	0	0	0	NA	NA	NA
ENSBTAG00000029592	*5S_rRNA*	0	0	0	0	0	NA	NA	NA
ENSBTAG00000029625	*5S_rRNA*	0	0	0	0	0	NA	NA	NA
ENSBTAG00000029640	*U1*	0	0	0	0	0	NA	NA	NA
ENSBTAG00000029663	*5S_rRNA*	0	0	0	0	0	NA	NA	NA
ENSBTAG00000029683	*U1*	0	0	0	0	0	NA	NA	NA
ENSBTAG00000029713	*5S_rRNA*	0	0	0	0	0	NA	NA	NA
ENSBTAG00000029760	*bta-mir-320a-2*	0	0	0	0	0	NA	NA	NA
ENSBTAG00000029761	*bta-mir-320a-1*	0	0	0	0	0	NA	NA	NA
ENSBTAG00000029762	*bta-mir-183*	0	0	0	0	0	NA	NA	NA
ENSBTAG00000029763	*bta-mir-375*	0	0	0	0	0	NA	NA	NA
ENSBTAG00000029764	*bta-mir-19a*	0	0	0	0	0	NA	NA	NA
ENSBTAG00000029774	*bta-let-7f-2*	0	0	0	0	0	NA	NA	NA
ENSBTAG00000029781	*bta-mir-106a*	0	0	0	0	0	NA	NA	NA
ENSBTAG00000029783	*bta-mir-218-1*	0	0	0	0	0	NA	NA	NA
ENSBTAG00000029785	*bta-mir-19b-2*	0	0	0	0	0	NA	NA	NA
ENSBTAG00000029787	*bta-mir-301a*	0	0	0	0	0	NA	NA	NA
ENSBTAG00000029788	*bta-mir-196a-2*	0	0	0	0	0	NA	NA	NA
ENSBTAG00000029789	*bta-mir-134*	0	0	0	0	0	NA	NA	NA

Ensembl gene ID	geneName	counts wildtype horn bud	counts polled horn bud	baseMean	baseMean wildtype horn bud	baseMean polled horn bud	log2FoldChange	pval	padj
ENSBTAG00000029793	*bta-mir-103-2*	0	0	0	0	0	NA	NA	NA
ENSBTAG00000029804	*bta-mir-30a*	0	0	0	0	0	NA	NA	NA
ENSBTAG00000029808	*bta-mir-412*	0	0	0	0	0	NA	NA	NA
ENSBTAG00000029809	*bta-mir-137*	0	0	0	0	0	NA	NA	NA
ENSBTAG00000029810	*bta-mir-361*	0	0	0	0	0	NA	NA	NA
ENSBTAG00000029814	*bta-mir-138-1*	0	0	0	0	0	NA	NA	NA
ENSBTAG00000029815	*bta-mir-135b*	0	0	0	0	0	NA	NA	NA
ENSBTAG00000029825	*bta-mir-135a-1*	0	0	0	0	0	NA	NA	NA
ENSBTAG00000029829	*bta-mir-136*	0	0	0	0	0	NA	NA	NA
ENSBTAG00000029833	*bta-mir-326*	0	0	0	0	0	NA	NA	NA
ENSBTAG00000029842	*bta-mir-346*	0	0	0	0	0	NA	NA	NA
ENSBTAG00000029847	*bta-mir-18a*	0	0	0	0	0	NA	NA	NA
ENSBTAG00000029848	*bta-mir-365-1*	0	0	0	0	0	NA	NA	NA
ENSBTAG00000029851	*bta-mir-122*	0	0	0	0	0	NA	NA	NA
ENSBTAG00000029854	*miRNA*	0	0	0	0	0	NA	NA	NA
ENSBTAG00000029856	*bta-mir-34b*	0	0	0	0	0	NA	NA	NA
ENSBTAG00000029859	*bta-mir-139*	0	0	0	0	0	NA	NA	NA
ENSBTAG00000029860	*bta-mir-20a*	0	0	0	0	0	NA	NA	NA
ENSBTAG00000029861	*bta-let-7g*	0	0	0	0	0	NA	NA	NA
ENSBTAG00000029864	*bta-mir-504*	0	0	0	0	0	NA	NA	NA
ENSBTAG00000029865	*bta-mir-151*	0	0	0	0	0	NA	NA	NA
ENSBTAG00000029867	*bta-mir-15a*	0	0	0	0	0	NA	NA	NA
ENSBTAG00000029868	*bta-mir-497*	0	0	0	0	0	NA	NA	NA
ENSBTAG00000029870	*bta-mir-98*	0	0	0	0	0	NA	NA	NA
ENSBTAG00000029871	*bta-mir-30e*	0	0	0	0	0	NA	NA	NA
ENSBTAG00000029875	*bta-mir-105a*	0	0	0	0	0	NA	NA	NA
ENSBTAG00000029877	*bta-mir-153-2*	0	0	0	0	0	NA	NA	NA
ENSBTAG00000029878	*bta-mir-188*	0	0	0	0	0	NA	NA	NA
ENSBTAG00000029891	*bta-mir-130b*	0	0	0	0	0	NA	NA	NA
ENSBTAG00000029892	*bta-mir-10a*	0	0	0	0	0	NA	NA	NA
ENSBTAG00000029898	*bta-mir-488*	0	0	0	0	0	NA	NA	NA
ENSBTAG00000029904	*miRNA*	0	0	0	0	0	NA	NA	NA
ENSBTAG00000029907	*bta-mir-377*	0	0	0	0	0	NA	NA	NA
ENSBTAG00000029910	*bta-mir-20b*	0	0	0	0	0	NA	NA	NA
ENSBTAG00000029911	*bta-mir-432*	0	0	0	0	0	NA	NA	NA
ENSBTAG00000029915	*bta-mir-129-1*	0	0	0	0	0	NA	NA	NA
ENSBTAG00000029916	*bta-mir-16b*	0	0	0	0	0	NA	NA	NA
ENSBTAG00000029929	*bta-mir-15b*	0	0	0	0	0	NA	NA	NA
ENSBTAG00000029939	*bta-mir-129-2*	0	0	0	0	0	NA	NA	NA
ENSBTAG00000029945	*bta-mir-148a*	0	0	0	0	0	NA	NA	NA
ENSBTAG00000029948	*bta-mir-19b*	0	0	0	0	0	NA	NA	NA
ENSBTAG00000029949	*bta-mir-499*	0	0	0	0	0	NA	NA	NA
ENSBTAG00000029950	*bta-mir-505*	0	0	0	0	0	NA	NA	NA
ENSBTAG00000029951	*bta-mir-17*	0	0	0	0	0	NA	NA	NA
ENSBTAG00000029953	*bta-mir-144*	0	0	0	0	0	NA	NA	NA
ENSBTAG00000029956	*bta-mir-491*	0	0	0	0	0	NA	NA	NA
ENSBTAG00000029959	*bta-mir-200c*	0	0	0	0	0	NA	NA	NA
ENSBTAG00000029960	*bta-mir-383*	0	0	0	0	0	NA	NA	NA
ENSBTAG00000029961	*bta-mir-409b*	0	0	0	0	0	NA	NA	NA
ENSBTAG00000029962	*bta-mir-124b*	0	0	0	0	0	NA	NA	NA
ENSBTAG00000029965	*bta-mir-490*	0	0	0	0	0	NA	NA	NA
ENSBTAG00000029967	*bta-mir-103-1*	0	0	0	0	0	NA	NA	NA
ENSBTAG00000029968	*bta-mir-7-3*	0	0	0	0	0	NA	NA	NA
ENSBTAG00000029970	*bta-mir-196b*	0	0	0	0	0	NA	NA	NA
ENSBTAG00000029972	*bta-mir-30b*	0	0	0	0	0	NA	NA	NA
ENSBTAG00000029973	*bta-mir-18b*	0	0	0	0	0	NA	NA	NA
ENSBTAG00000029975	*bta-mir-33a*	0	0	0	0	0	NA	NA	NA
ENSBTAG00000029977	*bta-mir-96*	0	0	0	0	0	NA	NA	NA
ENSBTAG00000029978	*bta-mir-363*	0	0	0	0	0	NA	NA	NA
ENSBTAG00000029979	*bta-mir-126*	0	0	0	0	0	NA	NA	NA
ENSBTAG00000029980	*bta-mir-29b-1*	0	0	0	0	0	NA	NA	NA
ENSBTAG00000029981	*bta-let-7a-1*	0	0	0	0	0	NA	NA	NA
ENSBTAG00000029983	*bta-mir-367*	0	0	0	0	0	NA	NA	NA
ENSBTAG00000029987	*bta-mir-30d*	0	0	0	0	0	NA	NA	NA
ENSBTAG00000029990	*bta-mir-128-1*	0	0	0	0	0	NA	NA	NA
ENSBTAG00000029991	*bta-mir-34c*	0	0	0	0	0	NA	NA	NA
ENSBTAG00000029998	*bta-mir-200a*	0	0	0	0	0	NA	NA	NA
ENSBTAG00000030002	*bta-mir-431*	0	0	0	0	0	NA	NA	NA
ENSBTAG00000030003	*bta-mir-127*	0	0	0	0	0	NA	NA	NA
ENSBTAG00000030004	*bta-mir-433*	0	0	0	0	0	NA	NA	NA
ENSBTAG00000030013	*bta-mir-660*	0	0	0	0	0	NA	NA	NA
ENSBTAG00000030014	*bta-mir-628*	0	0	0	0	0	NA	NA	NA

Ensembl gene ID	geneName	counts wildtype horn bud	counts polled horn bud	baseMean	baseMean wildtype horn bud	baseMean polled horn bud	log2FoldChange	pval	padj
ENSBTAG00000030020	*bta-mir-599*	0	0	0	0	0	NA	NA	NA
ENSBTAG00000030027	*bta-mir-449b*	0	0	0	0	0	NA	NA	NA
ENSBTAG00000030029	*bta-mir-551b*	0	0	0	0	0	NA	NA	NA
ENSBTAG00000030030	*bta-mir-592*	0	0	0	0	0	NA	NA	NA
ENSBTAG00000030038	*miRNA*	0	0	0	0	0	NA	NA	NA
ENSBTAG00000030039	*miRNA*	0	0	0	0	0	NA	NA	NA
ENSBTAG00000030040	*bta-mir-1277*	0	0	0	0	0	NA	NA	NA
ENSBTAG00000030046	*bta-mir-216b*	0	0	0	0	0	NA	NA	NA
ENSBTAG00000030047	*miRNA*	0	0	0	0	0	NA	NA	NA
ENSBTAG00000030051	*bta-mir-449c*	0	0	0	0	0	NA	NA	NA
ENSBTAG00000030053	*bta-mir-194-1*	0	0	0	0	0	NA	NA	NA
ENSBTAG00000030055	*miRNA*	0	0	0	0	0	NA	NA	NA
ENSBTAG00000030056	*miRNA*	0	0	0	0	0	NA	NA	NA
ENSBTAG00000030059	*bta-mir-92a-2*	0	0	0	0	0	NA	NA	NA
ENSBTAG00000030064	*bta-mir-584-6*	0	0	0	0	0	NA	NA	NA
ENSBTAG00000030066	*bta-mir-454*	0	0	0	0	0	NA	NA	NA
ENSBTAG00000030067	*bta-mir-584-1*	0	0	0	0	0	NA	NA	NA
ENSBTAG00000030068	*bta-mir-150*	0	0	0	0	0	NA	NA	NA
ENSBTAG00000030073	*bta-mir-342*	0	0	0	0	0	NA	NA	NA
ENSBTAG00000030083	*bta-mir-670*	0	0	0	0	0	NA	NA	NA
ENSBTAG00000030085	*bta-mir-584-3*	0	0	0	0	0	NA	NA	NA
ENSBTAG00000030086	*bta-mir-544b-2*	0	0	0	0	0	NA	NA	NA
ENSBTAG00000030097	*bta-mir-584-7*	0	0	0	0	0	NA	NA	NA
ENSBTAG00000030099	*bta-mir-551a*	0	0	0	0	0	NA	NA	NA
ENSBTAG00000030102	*bta-mir-760*	0	0	0	0	0	NA	NA	NA
ENSBTAG00000030104	*bta-mir-767*	0	0	0	0	0	NA	NA	NA
ENSBTAG00000030105	*miRNA*	0	0	0	0	0	NA	NA	NA
ENSBTAG00000030106	*bta-mir-105b*	0	0	0	0	0	NA	NA	NA
ENSBTAG00000030107	*miRNA*	0	0	0	0	0	NA	NA	NA
ENSBTAG00000030108	*bta-mir-769*	0	0	0	0	0	NA	NA	NA
ENSBTAG00000030109	*bta-mir-744*	0	0	0	0	0	NA	NA	NA
ENSBTAG00000030110	*bta-mir-759*	0	0	0	0	0	NA	NA	NA
ENSBTAG00000030118	*bta-mir-182*	0	0	0	0	0	NA	NA	NA
ENSBTAG00000030121	*bta-mir-449a*	0	0	0	0	0	NA	NA	NA
ENSBTAG00000030122	*bta-mir-130a*	0	0	0	0	0	NA	NA	NA
ENSBTAG00000030123	*bta-mir-194-2*	0	0	0	0	0	NA	NA	NA
ENSBTAG00000030124	*miRNA*	0	0	0	0	0	NA	NA	NA
ENSBTAG00000030185	*protein_coding*	0	0	0	0	0	NA	NA	NA
ENSBTAG00000030204	*EPB49*	0	0	0	0	0	NA	NA	NA
ENSBTAG00000030221	*protein_coding*	0	0	0	0	0	NA	NA	NA
ENSBTAG00000030239	*protein_coding*	0	0	0	0	0	NA	NA	NA
ENSBTAG00000030297	*MGC137099*	0	0	0	0	0	NA	NA	NA
ENSBTAG00000030306	*U2*	0	0	0	0	0	NA	NA	NA
ENSBTAG00000030325	*protein_coding*	0	0	0	0	0	NA	NA	NA
ENSBTAG00000030344	*protein_coding*	0	0	0	0	0	NA	NA	NA
ENSBTAG00000030367	*BT.48834*	0	0	0	0	0	NA	NA	NA
ENSBTAG00000030385	*protein_coding*	0	0	0	0	0	NA	NA	NA
ENSBTAG00000030386	*protein_coding*	0	0	0	0	0	NA	NA	NA
ENSBTAG00000030439	*protein_coding*	0	0	0	0	0	NA	NA	NA
ENSBTAG00000030440	*protein_coding*	0	0	0	0	0	NA	NA	NA
ENSBTAG00000030444	*protein_coding*	0	0	0	0	0	NA	NA	NA
ENSBTAG00000030461	*BT.76068*	0	0	0	0	0	NA	NA	NA
ENSBTAG00000030463	*BT.76065*	0	0	0	0	0	NA	NA	NA
ENSBTAG00000030466	*BT.76067*	0	0	0	0	0	NA	NA	NA
ENSBTAG00000030468	*BT.76064*	0	0	0	0	0	NA	NA	NA
ENSBTAG00000030471	*BT.76069*	0	0	0	0	0	NA	NA	NA
ENSBTAG00000030472	*BT.95801*	0	0	0	0	0	NA	NA	NA
ENSBTAG00000030476	*protein_coding*	0	0	0	0	0	NA	NA	NA
ENSBTAG00000030494	*protein_coding*	0	0	0	0	0	NA	NA	NA
ENSBTAG00000030559	*KRTAP1-1*	0	0	0	0	0	NA	NA	NA
ENSBTAG00000030585	*CLDN14*	0	0	0	0	0	NA	NA	NA
ENSBTAG00000030621	*C12ORF70*	0	0	0	0	0	NA	NA	NA
ENSBTAG00000030663	*U1*	0	0	0	0	0	NA	NA	NA
ENSBTAG00000030676	*OR1K1*	0	0	0	0	0	NA	NA	NA
ENSBTAG00000030677	*protein_coding*	0	0	0	0	0	NA	NA	NA
ENSBTAG00000030714	*protein_coding*	0	0	0	0	0	NA	NA	NA
ENSBTAG00000030718	*SPATA3*	0	0	0	0	0	NA	NA	NA
ENSBTAG00000030719	*pseudogene*	0	0	0	0	0	NA	NA	NA
ENSBTAG00000030722	*OR2M4*	0	0	0	0	0	NA	NA	NA
ENSBTAG00000030725	*protein_coding*	0	0	0	0	0	NA	NA	NA
ENSBTAG00000030796	*WFDC10A*	0	0	0	0	0	NA	NA	NA
ENSBTAG00000030798	*WFDC11*	0	0	0	0	0	NA	NA	NA

Ensembl gene ID	geneName	counts wildtype horn bud	counts polled horn bud	baseMean	baseMean wildtype horn bud	baseMean polled horn bud	log2FoldChange	pval	padj
ENSBTAG00000030800	*WFDC10B*	0	0	0	0	0	NA	NA	NA
ENSBTAG00000030802	*protein_coding*	0	0	0	0	0	NA	NA	NA
ENSBTAG00000030841	*GCSH*	0	0	0	0	0	NA	NA	NA
ENSBTAG00000030909	*protein_coding*	0	0	0	0	0	NA	NA	NA
ENSBTAG00000030925	*pseudogene*	0	0	0	0	0	NA	NA	NA
ENSBTAG00000030953	*protein_coding*	0	0	0	0	0	NA	NA	NA
ENSBTAG00000030976	*C20ORF173*	0	0	0	0	0	NA	NA	NA
ENSBTAG00000031025	*protein_coding*	0	0	0	0	0	NA	NA	NA
ENSBTAG00000031026	*BT.76076*	0	0	0	0	0	NA	NA	NA
ENSBTAG00000031027	*protein_coding*	0	0	0	0	0	NA	NA	NA
ENSBTAG00000031028	*protein_coding*	0	0	0	0	0	NA	NA	NA
ENSBTAG00000031029	*protein_coding*	0	0	0	0	0	NA	NA	NA
ENSBTAG00000031030	*OR4C5*	0	0	0	0	0	NA	NA	NA
ENSBTAG00000031031	*OR10P1*	0	0	0	0	0	NA	NA	NA
ENSBTAG00000031032	*protein_coding*	0	0	0	0	0	NA	NA	NA
ENSBTAG00000031096	*protein_coding*	0	0	0	0	0	NA	NA	NA
ENSBTAG00000031097	*protein_coding*	0	0	0	0	0	NA	NA	NA
ENSBTAG00000031099	*protein_coding*	0	0	0	0	0	NA	NA	NA
ENSBTAG00000031100	*pseudogene*	0	0	0	0	0	NA	NA	NA
ENSBTAG00000031111	*OR6C68*	0	0	0	0	0	NA	NA	NA
ENSBTAG00000031119	*OR4S1*	0	0	0	0	0	NA	NA	NA
ENSBTAG00000031162	*pseudogene*	0	0	0	0	0	NA	NA	NA
ENSBTAG00000031212	*protein_coding*	0	0	0	0	0	NA	NA	NA
ENSBTAG00000031216	*BT.91405*	0	0	0	0	0	NA	NA	NA
ENSBTAG00000031236	*BT.75235*	0	0	0	0	0	NA	NA	NA
ENSBTAG00000031247	*MUC12*	0	0	0	0	0	NA	NA	NA
ENSBTAG00000031254	*BT.100199*	0	0	0	0	0	NA	NA	NA
ENSBTAG00000031266	*5S_rRNA*	0	0	0	0	0	NA	NA	NA
ENSBTAG00000031271	*MRGPRG*	0	0	0	0	0	NA	NA	NA
ENSBTAG00000031302	*GCKR*	0	0	0	0	0	NA	NA	NA
ENSBTAG00000031320	*protein_coding*	0	0	0	0	0	NA	NA	NA
ENSBTAG00000031330	*HTR3B*	0	0	0	0	0	NA	NA	NA
ENSBTAG00000031373	*BT.73263*	0	0	0	0	0	NA	NA	NA
ENSBTAG00000031375	*BSP30D*	0	0	0	0	0	NA	NA	NA
ENSBTAG00000031412	*OR6F1*	0	0	0	0	0	NA	NA	NA
ENSBTAG00000031420	*protein_coding*	0	0	0	0	0	NA	NA	NA
ENSBTAG00000031507	*protein_coding*	0	0	0	0	0	NA	NA	NA
ENSBTAG00000031517	*TSPY-M2*	0	0	0	0	0	NA	NA	NA
ENSBTAG00000031594	*protein_coding*	0	0	0	0	0	NA	NA	NA
ENSBTAG00000031640	*PRP3*	0	0	0	0	0	NA	NA	NA
ENSBTAG00000031677	*5S_rRNA*	0	0	0	0	0	NA	NA	NA
ENSBTAG00000031721	*protein_coding*	0	0	0	0	0	NA	NA	NA
ENSBTAG00000031727	*5S_rRNA*	0	0	0	0	0	NA	NA	NA
ENSBTAG00000031753	*BTN1A1*	0	0	0	0	0	NA	NA	NA
ENSBTAG00000031772	*PRODH2*	0	0	0	0	0	NA	NA	NA
ENSBTAG00000031774	*HIST1H1E*	0	0	0	0	0	NA	NA	NA
ENSBTAG00000031825	*BT.19997*	0	0	0	0	0	NA	NA	NA
ENSBTAG00000031832	*protein_coding*	0	0	0	0	0	NA	NA	NA
ENSBTAG00000031835	*protein_coding*	0	0	0	0	0	NA	NA	NA
ENSBTAG00000031850	*protein_coding*	0	0	0	0	0	NA	NA	NA
ENSBTAG00000031881	*protein_coding*	0	0	0	0	0	NA	NA	NA
ENSBTAG00000031903	*protein_coding*	0	0	0	0	0	NA	NA	NA
ENSBTAG00000031907	*protein_coding*	0	0	0	0	0	NA	NA	NA
ENSBTAG00000031915	*protein_coding*	0	0	0	0	0	NA	NA	NA
ENSBTAG00000031918	*PPIA*	0	0	0	0	0	NA	NA	NA
ENSBTAG00000031923	*OR52K1*	0	0	0	0	0	NA	NA	NA
ENSBTAG00000031924	*OR52M1*	0	0	0	0	0	NA	NA	NA
ENSBTAG00000031934	*BT.54156*	0	0	0	0	0	NA	NA	NA
ENSBTAG00000031937	*protein_coding*	0	0	0	0	0	NA	NA	NA
ENSBTAG00000031943	*protein_coding*	0	0	0	0	0	NA	NA	NA
ENSBTAG00000031958	*BT.32541*	0	0	0	0	0	NA	NA	NA
ENSBTAG00000032014	*BT.71274*	0	0	0	0	0	NA	NA	NA
ENSBTAG00000032049	*BT.76066*	0	0	0	0	0	NA	NA	NA
ENSBTAG00000032050	*protein_coding*	0	0	0	0	0	NA	NA	NA
ENSBTAG00000032052	*protein_coding*	0	0	0	0	0	NA	NA	NA
ENSBTAG00000032056	*OR10A4*	0	0	0	0	0	NA	NA	NA
ENSBTAG00000032142	*OR7G3*	0	0	0	0	0	NA	NA	NA
ENSBTAG00000032209	*CXHXorf65*	0	0	0	0	0	NA	NA	NA
ENSBTAG00000032224	*protein_coding*	0	0	0	0	0	NA	NA	NA
ENSBTAG00000032255	*protein_coding*	0	0	0	0	0	NA	NA	NA
ENSBTAG00000032256	*protein_coding*	0	0	0	0	0	NA	NA	NA
ENSBTAG00000032259	*protein_coding*	0	0	0	0	0	NA	NA	NA

Ensembl gene ID	geneName	counts wildtype horn bud	counts polled horn bud	baseMean	baseMean wildtype horn bud	baseMean polled horn bud	log2FoldChange	pval	padj
ENSBTAG00000032262	*protein_coding*	0	0	0	0	0	NA	NA	NA
ENSBTAG00000032264	*protein_coding*	0	0	0	0	0	NA	NA	NA
ENSBTAG00000032274	*OR52E4*	0	0	0	0	0	NA	NA	NA
ENSBTAG00000032276	*protein_coding*	0	0	0	0	0	NA	NA	NA
ENSBTAG00000032280	*OR56A3*	0	0	0	0	0	NA	NA	NA
ENSBTAG00000032299	*BT.56227*	0	0	0	0	0	NA	NA	NA
ENSBTAG00000032307	*pseudogene*	0	0	0	0	0	NA	NA	NA
ENSBTAG00000032339	*ASCL3*	0	0	0	0	0	NA	NA	NA
ENSBTAG00000032350	*BT.66319*	0	0	0	0	0	NA	NA	NA
ENSBTAG00000032382	*protein_coding*	0	0	0	0	0	NA	NA	NA
ENSBTAG00000032393	*C7ORF62*	0	0	0	0	0	NA	NA	NA
ENSBTAG00000032406	*U1*	0	0	0	0	0	NA	NA	NA
ENSBTAG00000032407	*U1*	0	0	0	0	0	NA	NA	NA
ENSBTAG00000032425	*PPBP*	0	0	0	0	0	NA	NA	NA
ENSBTAG00000032442	*BT.72855*	0	0	0	0	0	NA	NA	NA
ENSBTAG00000032457	*protein_coding*	0	0	0	0	0	NA	NA	NA
ENSBTAG00000032467	*protein_coding*	0	0	0	0	0	NA	NA	NA
ENSBTAG00000032508	*BT.76062*	0	0	0	0	0	NA	NA	NA
ENSBTAG00000032560	*SPINK2*	0	0	0	0	0	NA	NA	NA
ENSBTAG00000032576	*CMTM2*	0	0	0	0	0	NA	NA	NA
ENSBTAG00000032615	*5S_rRNA*	0	0	0	0	0	NA	NA	NA
ENSBTAG00000032624	*5S_rRNA*	0	0	0	0	0	NA	NA	NA
ENSBTAG00000032643	*protein_coding*	0	0	0	0	0	NA	NA	NA
ENSBTAG00000032655	*ZNF485*	0	0	0	0	0	NA	NA	NA
ENSBTAG00000032668	*protein_coding*	0	0	0	0	0	NA	NA	NA
ENSBTAG00000032670	*OR2S2*	0	0	0	0	0	NA	NA	NA
ENSBTAG00000032671	*protein_coding*	0	0	0	0	0	NA	NA	NA
ENSBTAG00000032694	*CST8*	0	0	0	0	0	NA	NA	NA
ENSBTAG00000032696	*BT.50520*	0	0	0	0	0	NA	NA	NA
ENSBTAG00000032701	*CSTL1*	0	0	0	0	0	NA	NA	NA
ENSBTAG00000032728	*5S_rRNA*	0	0	0	0	0	NA	NA	NA
ENSBTAG00000032730	*BT.56257*	0	0	0	0	0	NA	NA	NA
ENSBTAG00000032781	*pseudogene*	0	0	0	0	0	NA	NA	NA
ENSBTAG00000032783	*pseudogene*	0	0	0	0	0	NA	NA	NA
ENSBTAG00000032787	*protein_coding*	0	0	0	0	0	NA	NA	NA
ENSBTAG00000032789	*OR4N2*	0	0	0	0	0	NA	NA	NA
ENSBTAG00000032792	*OR4Q3*	0	0	0	0	0	NA	NA	NA
ENSBTAG00000032798	*protein_coding*	0	0	0	0	0	NA	NA	NA
ENSBTAG00000032799	*protein_coding*	0	0	0	0	0	NA	NA	NA
ENSBTAG00000032800	*OPN4*	0	0	0	0	0	NA	NA	NA
ENSBTAG00000032801	*OR11H6*	0	0	0	0	0	NA	NA	NA
ENSBTAG00000032853	*pseudogene*	0	0	0	0	0	NA	NA	NA
ENSBTAG00000032873	*protein_coding*	0	0	0	0	0	NA	NA	NA
ENSBTAG00000032878	*protein_coding*	0	0	0	0	0	NA	NA	NA
ENSBTAG00000032880	*PRM2*	0	0	0	0	0	NA	NA	NA
ENSBTAG00000032894	*protein_coding*	0	0	0	0	0	NA	NA	NA
ENSBTAG00000032895	*protein_coding*	0	0	0	0	0	NA	NA	NA
ENSBTAG00000032896	*protein_coding*	0	0	0	0	0	NA	NA	NA
ENSBTAG00000032918	*SCGB1A1*	0	0	0	0	0	NA	NA	NA
ENSBTAG00000032926	*pseudogene*	0	0	0	0	0	NA	NA	NA
ENSBTAG00000032983	*FOXB2*	0	0	0	0	0	NA	NA	NA
ENSBTAG00000032984	*protein_coding*	0	0	0	0	0	NA	NA	NA
ENSBTAG00000032995	*LELP1*	0	0	0	0	0	NA	NA	NA
ENSBTAG00000033034	*protein_coding*	0	0	0	0	0	NA	NA	NA
ENSBTAG00000033036	*BT.86117*	0	0	0	0	0	NA	NA	NA
ENSBTAG00000033045	*protein_coding*	0	0	0	0	0	NA	NA	NA
ENSBTAG00000033061	*protein_coding*	0	0	0	0	0	NA	NA	NA
ENSBTAG00000033083	*protein_coding*	0	0	0	0	0	NA	NA	NA
ENSBTAG00000033092	*protein_coding*	0	0	0	0	0	NA	NA	NA
ENSBTAG00000033096	*PAG2*	0	0	0	0	0	NA	NA	NA
ENSBTAG00000033117	*HIST1H1E*	0	0	0	0	0	NA	NA	NA
ENSBTAG00000033146	*protein_coding*	0	0	0	0	0	NA	NA	NA
ENSBTAG00000033187	*protein_coding*	0	0	0	0	0	NA	NA	NA
ENSBTAG00000033275	*5S_rRNA*	0	0	0	0	0	NA	NA	NA
ENSBTAG00000033309	*5S_rRNA*	0	0	0	0	0	NA	NA	NA
ENSBTAG00000033327	*protein_coding*	0	0	0	0	0	NA	NA	NA
ENSBTAG00000033352	*DMRT1*	0	0	0	0	0	NA	NA	NA
ENSBTAG00000033359	*protein_coding*	0	0	0	0	0	NA	NA	NA
ENSBTAG00000033375	*PATE2*	0	0	0	0	0	NA	NA	NA
ENSBTAG00000033397	*SIRPD*	0	0	0	0	0	NA	NA	NA
ENSBTAG00000033437	*protein_coding*	0	0	0	0	0	NA	NA	NA
ENSBTAG00000033445	*BT.54325*	0	0	0	0	0	NA	NA	NA

Ensembl gene ID	geneName	counts wildtype horn bud	counts polled horn bud	baseMean	baseMean wildtype horn bud	baseMean polled horn bud	log2FoldChange	pval	padj
ENSBTAG00000033477	*protein_coding*	0	0	0	0	0	NA	NA	NA
ENSBTAG00000033480	*OR8B4*	0	0	0	0	0	NA	NA	NA
ENSBTAG00000033481	*C1QTNF8*	0	0	0	0	0	NA	NA	NA
ENSBTAG00000033502	*BT.54138*	0	0	0	0	0	NA	NA	NA
ENSBTAG00000033506	*OR4D11*	0	0	0	0	0	NA	NA	NA
ENSBTAG00000033508	*OR10S1*	0	0	0	0	0	NA	NA	NA
ENSBTAG00000033511	*pseudogene*	0	0	0	0	0	NA	NA	NA
ENSBTAG00000033513	*PRP9*	0	0	0	0	0	NA	NA	NA
ENSBTAG00000033519	*protein_coding*	0	0	0	0	0	NA	NA	NA
ENSBTAG00000033547	*C17H4orf45*	0	0	0	0	0	NA	NA	NA
ENSBTAG00000033554	*BET3L*	0	0	0	0	0	NA	NA	NA
ENSBTAG00000033555	*protein_coding*	0	0	0	0	0	NA	NA	NA
ENSBTAG00000033558	*protein_coding*	0	0	0	0	0	NA	NA	NA
ENSBTAG00000033562	*LHX1*	0	0	0	0	0	NA	NA	NA
ENSBTAG00000033568	*pseudogene*	0	0	0	0	0	NA	NA	NA
ENSBTAG00000033594	*C12ORF74*	0	0	0	0	0	NA	NA	NA
ENSBTAG00000033600	*protein_coding*	0	0	0	0	0	NA	NA	NA
ENSBTAG00000033604	*protein_coding*	0	0	0	0	0	NA	NA	NA
ENSBTAG00000033620	*protein_coding*	0	0	0	0	0	NA	NA	NA
ENSBTAG00000033622	*U1*	0	0	0	0	0	NA	NA	NA
ENSBTAG00000033627	*protein_coding*	0	0	0	0	0	NA	NA	NA
ENSBTAG00000033656	*TRIM49*	0	0	0	0	0	NA	NA	NA
ENSBTAG00000033671	*protein_coding*	0	0	0	0	0	NA	NA	NA
ENSBTAG00000033708	*protein_coding*	0	0	0	0	0	NA	NA	NA
ENSBTAG00000033772	*5S_rRNA*	0	0	0	0	0	NA	NA	NA
ENSBTAG00000033839	*5S_rRNA*	0	0	0	0	0	NA	NA	NA
ENSBTAG00000033841	*protein_coding*	0	0	0	0	0	NA	NA	NA
ENSBTAG00000033874	*protein_coding*	0	0	0	0	0	NA	NA	NA
ENSBTAG00000033881	*processed_pseudogene*	0	0	0	0	0	NA	NA	NA
ENSBTAG00000033954	*BT.87335*	0	0	0	0	0	NA	NA	NA
ENSBTAG00000033967	*protein_coding*	0	0	0	0	0	NA	NA	NA
ENSBTAG00000033979	*protein_coding*	0	0	0	0	0	NA	NA	NA
ENSBTAG00000033993	*PRP14*	0	0	0	0	0	NA	NA	NA
ENSBTAG00000034032	*processed_pseudogene*	0	0	0	0	0	NA	NA	NA
ENSBTAG00000034064	*BT.50889*	0	0	0	0	0	NA	NA	NA
ENSBTAG00000034139	*BT.19521*	0	0	0	0	0	NA	NA	NA
ENSBTAG00000034176	*protein_coding*	0	0	0	0	0	NA	NA	NA
ENSBTAG00000034184	*NMS*	0	0	0	0	0	NA	NA	NA
ENSBTAG00000034187	*5S_rRNA*	0	0	0	0	0	NA	NA	NA
ENSBTAG00000034189	*protein_coding*	0	0	0	0	0	NA	NA	NA
ENSBTAG00000034238	*ZSCAN10*	0	0	0	0	0	NA	NA	NA
ENSBTAG00000034244	*protein_coding*	0	0	0	0	0	NA	NA	NA
ENSBTAG00000034266	*pseudogene*	0	0	0	0	0	NA	NA	NA
ENSBTAG00000034283	*ADAD2*	0	0	0	0	0	NA	NA	NA
ENSBTAG00000034308	*pseudogene*	0	0	0	0	0	NA	NA	NA
ENSBTAG00000034328	*5S_rRNA*	0	0	0	0	0	NA	NA	NA
ENSBTAG00000034372	*BT.45760*	0	0	0	0	0	NA	NA	NA
ENSBTAG00000034431	*protein_coding*	0	0	0	0	0	NA	NA	NA
ENSBTAG00000034437	*BT.53290*	0	0	0	0	0	NA	NA	NA
ENSBTAG00000034444	*protein_coding*	0	0	0	0	0	NA	NA	NA
ENSBTAG00000034463	*pseudogene*	0	0	0	0	0	NA	NA	NA
ENSBTAG00000034551	*U1*	0	0	0	0	0	NA	NA	NA
ENSBTAG00000034555	*AP3S1*	0	0	0	0	0	NA	NA	NA
ENSBTAG00000034577	*protein_coding*	0	0	0	0	0	NA	NA	NA
ENSBTAG00000034582	*H2B*	0	0	0	0	0	NA	NA	NA
ENSBTAG00000034601	*OR6N1*	0	0	0	0	0	NA	NA	NA
ENSBTAG00000034632	*LEMD1*	0	0	0	0	0	NA	NA	NA
ENSBTAG00000034678	*protein_coding*	0	0	0	0	0	NA	NA	NA
ENSBTAG00000034724	*protein_coding*	0	0	0	0	0	NA	NA	NA
ENSBTAG00000034743	*protein_coding*	0	0	0	0	0	NA	NA	NA
ENSBTAG00000034761	*protein_coding*	0	0	0	0	0	NA	NA	NA
ENSBTAG00000034841	*protein_coding*	0	0	0	0	0	NA	NA	NA
ENSBTAG00000034849	*protein_coding*	0	0	0	0	0	NA	NA	NA
ENSBTAG00000034869	*KRTAP11-1*	0	0	0	0	0	NA	NA	NA
ENSBTAG00000034891	*protein_coding*	0	0	0	0	0	NA	NA	NA
ENSBTAG00000034909	*protein_coding*	0	0	0	0	0	NA	NA	NA
ENSBTAG00000034917	*protein_coding*	0	0	0	0	0	NA	NA	NA
ENSBTAG00000034921	*protein_coding*	0	0	0	0	0	NA	NA	NA
ENSBTAG00000034939	*BTNL2*	0	0	0	0	0	NA	NA	NA
ENSBTAG00000034940	*BT.106492*	0	0	0	0	0	NA	NA	NA
ENSBTAG00000034945	*BT.28255*	0	0	0	0	0	NA	NA	NA
ENSBTAG00000034948	*U1*	0	0	0	0	0	NA	NA	NA

Ensembl gene ID	geneName	counts wildtype horn bud	counts polled horn bud	baseMean	baseMean wildtype horn bud	baseMean polled horn bud	log2FoldChange	pval	padj
ENSBTAG00000034964	*pseudogene*	0	0	0	0	0	NA	NA	NA
ENSBTAG00000035006	*protein_coding*	0	0	0	0	0	NA	NA	NA
ENSBTAG00000035008	*protein_coding*	0	0	0	0	0	NA	NA	NA
ENSBTAG00000035021	*processed_pseudogene*	0	0	0	0	0	NA	NA	NA
ENSBTAG00000035027	*BT.76077*	0	0	0	0	0	NA	NA	NA
ENSBTAG00000035029	*protein_coding*	0	0	0	0	0	NA	NA	NA
ENSBTAG00000035031	*OR8K5*	0	0	0	0	0	NA	NA	NA
ENSBTAG00000035059	*OR8J1*	0	0	0	0	0	NA	NA	NA
ENSBTAG00000035072	*SYCE1*	0	0	0	0	0	NA	NA	NA
ENSBTAG00000035150	*5S_rRNA*	0	0	0	0	0	NA	NA	NA
ENSBTAG00000035184	*protein_coding*	0	0	0	0	0	NA	NA	NA
ENSBTAG00000035241	*protein_coding*	0	0	0	0	0	NA	NA	NA
ENSBTAG00000035268	*5S_rRNA*	0	0	0	0	0	NA	NA	NA
ENSBTAG00000035282	*OR5D18*	0	0	0	0	0	NA	NA	NA
ENSBTAG00000035309	*OR4F15*	0	0	0	0	0	NA	NA	NA
ENSBTAG00000035333	*protein_coding*	0	0	0	0	0	NA	NA	NA
ENSBTAG00000035349	*pseudogene*	0	0	0	0	0	NA	NA	NA
ENSBTAG00000035395	*protein_coding*	0	0	0	0	0	NA	NA	NA
ENSBTAG00000035399	*OR1M1*	0	0	0	0	0	NA	NA	NA
ENSBTAG00000035442	*protein_coding*	0	0	0	0	0	NA	NA	NA
ENSBTAG00000035485	*OR5D13*	0	0	0	0	0	NA	NA	NA
ENSBTAG00000035487	*protein_coding*	0	0	0	0	0	NA	NA	NA
ENSBTAG00000035489	*protein_coding*	0	0	0	0	0	NA	NA	NA
ENSBTAG00000035530	*protein_coding*	0	0	0	0	0	NA	NA	NA
ENSBTAG00000035606	*protein_coding*	0	0	0	0	0	NA	NA	NA
ENSBTAG00000035623	*protein_coding*	0	0	0	0	0	NA	NA	NA
ENSBTAG00000035631	*protein_coding*	0	0	0	0	0	NA	NA	NA
ENSBTAG00000035659	*protein_coding*	0	0	0	0	0	NA	NA	NA
ENSBTAG00000035675	*BT.64316*	0	0	0	0	0	NA	NA	NA
ENSBTAG00000035686	*protein_coding*	0	0	0	0	0	NA	NA	NA
ENSBTAG00000035696	*BT.60941*	0	0	0	0	0	NA	NA	NA
ENSBTAG00000035707	*protein_coding*	0	0	0	0	0	NA	NA	NA
ENSBTAG00000035708	*protein_coding*	0	0	0	0	0	NA	NA	NA
ENSBTAG00000035709	*protein_coding*	0	0	0	0	0	NA	NA	NA
ENSBTAG00000035736	*protein_coding*	0	0	0	0	0	NA	NA	NA
ENSBTAG00000035745	*BT.56170*	0	0	0	0	0	NA	NA	NA
ENSBTAG00000035758	*OR4K15*	0	0	0	0	0	NA	NA	NA
ENSBTAG00000035768	*protein_coding*	0	0	0	0	0	NA	NA	NA
ENSBTAG00000035803	*pseudogene*	0	0	0	0	0	NA	NA	NA
ENSBTAG00000035883	*pseudogene*	0	0	0	0	0	NA	NA	NA
ENSBTAG00000035952	*pseudogene*	0	0	0	0	0	NA	NA	NA
ENSBTAG00000035985	*protein_coding*	0	0	0	0	0	NA	NA	NA
ENSBTAG00000035986	*OR8K5*	0	0	0	0	0	NA	NA	NA
ENSBTAG00000035988	*protein_coding*	0	0	0	0	0	NA	NA	NA
ENSBTAG00000035989	*protein_coding*	0	0	0	0	0	NA	NA	NA
ENSBTAG00000036005	*protein_coding*	0	0	0	0	0	NA	NA	NA
ENSBTAG00000036008	*protein_coding*	0	0	0	0	0	NA	NA	NA
ENSBTAG00000036013	*OR13C8*	0	0	0	0	0	NA	NA	NA
ENSBTAG00000036025	*protein_coding*	0	0	0	0	0	NA	NA	NA
ENSBTAG00000036031	*protein_coding*	0	0	0	0	0	NA	NA	NA
ENSBTAG00000036042	*protein_coding*	0	0	0	0	0	NA	NA	NA
ENSBTAG00000036045	*5S_rRNA*	0	0	0	0	0	NA	NA	NA
ENSBTAG00000036058	*protein_coding*	0	0	0	0	0	NA	NA	NA
ENSBTAG00000036060	*protein_coding*	0	0	0	0	0	NA	NA	NA
ENSBTAG00000036082	*protein_coding*	0	0	0	0	0	NA	NA	NA
ENSBTAG00000036098	*protein_coding*	0	0	0	0	0	NA	NA	NA
ENSBTAG00000036102	*BT.64150*	0	0	0	0	0	NA	NA	NA
ENSBTAG00000036106	*protein_coding*	0	0	0	0	0	NA	NA	NA
ENSBTAG00000036115	*protein_coding*	0	0	0	0	0	NA	NA	NA
ENSBTAG00000036130	*protein_coding*	0	0	0	0	0	NA	NA	NA
ENSBTAG00000036131	*protein_coding*	0	0	0	0	0	NA	NA	NA
ENSBTAG00000036144	*BT.56221*	0	0	0	0	0	NA	NA	NA
ENSBTAG00000036172	*PAG20*	0	0	0	0	0	NA	NA	NA
ENSBTAG00000036175	*protein_coding*	0	0	0	0	0	NA	NA	NA
ENSBTAG00000036202	*protein_coding*	0	0	0	0	0	NA	NA	NA
ENSBTAG00000036215	*BOSTAUV1R433*	0	0	0	0	0	NA	NA	NA
ENSBTAG00000036271	*pseudogene*	0	0	0	0	0	NA	NA	NA
ENSBTAG00000036273	*protein_coding*	0	0	0	0	0	NA	NA	NA
ENSBTAG00000036277	*PAG4*	0	0	0	0	0	NA	NA	NA
ENSBTAG00000036321	*pseudogene*	0	0	0	0	0	NA	NA	NA
ENSBTAG00000036326	*protein_coding*	0	0	0	0	0	NA	NA	NA
ENSBTAG00000036328	*protein_coding*	0	0	0	0	0	NA	NA	NA

Ensembl gene ID	geneName	counts wildtype horn bud	counts polled horn bud	baseMean	baseMean wildtype horn bud	baseMean polled horn bud	log2FoldChange	pval	padj
ENSBTAG00000036331	*protein_coding*	0	0	0	0	0	NA	NA	NA
ENSBTAG00000036343	*BT.50632*	0	0	0	0	0	NA	NA	NA
ENSBTAG00000036355	*bta-mir-671*	0	0	0	0	0	NA	NA	NA
ENSBTAG00000036357	*bta-mir-128-2*	0	0	0	0	0	NA	NA	NA
ENSBTAG00000036359	*bta-mir-448*	0	0	0	0	0	NA	NA	NA
ENSBTAG00000036360	*bta-mir-495*	0	0	0	0	0	NA	NA	NA
ENSBTAG00000036366	*bta-mir-653*	0	0	0	0	0	NA	NA	NA
ENSBTAG00000036371	*bta-mir-193b*	0	0	0	0	0	NA	NA	NA
ENSBTAG00000036372	*bta-mir-28*	0	0	0	0	0	NA	NA	NA
ENSBTAG00000036374	*bta-mir-362*	0	0	0	0	0	NA	NA	NA
ENSBTAG00000036376	*bta-mir-502a-1*	0	0	0	0	0	NA	NA	NA
ENSBTAG00000036380	*bta-mir-184*	0	0	0	0	0	NA	NA	NA
ENSBTAG00000036381	*bta-mir-615*	0	0	0	0	0	NA	NA	NA
ENSBTAG00000036383	*bta-mir-329b*	0	0	0	0	0	NA	NA	NA
ENSBTAG00000036386	*bta-mir-196a-1*	0	0	0	0	0	NA	NA	NA
ENSBTAG00000036389	*bta-mir-16a*	0	0	0	0	0	NA	NA	NA
ENSBTAG00000036391	*miRNA*	0	0	0	0	0	NA	NA	NA
ENSBTAG00000036392	*miRNA*	0	0	0	0	0	NA	NA	NA
ENSBTAG00000036393	*bta-mir-658*	0	0	0	0	0	NA	NA	NA
ENSBTAG00000036394	*bta-mir-761*	0	0	0	0	0	NA	NA	NA
ENSBTAG00000036395	*bta-mir-429*	0	0	0	0	0	NA	NA	NA
ENSBTAG00000036399	*miRNA*	0	0	0	0	0	NA	NA	NA
ENSBTAG00000036401	*bta-mir-147*	0	0	0	0	0	NA	NA	NA
ENSBTAG00000036402	*bta-mir-485*	0	0	0	0	0	NA	NA	NA
ENSBTAG00000036403	*bta-mir-502a-2*	0	0	0	0	0	NA	NA	NA
ENSBTAG00000036406	*miRNA*	0	0	0	0	0	NA	NA	NA
ENSBTAG00000036407	*miRNA*	0	0	0	0	0	NA	NA	NA
ENSBTAG00000036408	*bta-mir-302d*	0	0	0	0	0	NA	NA	NA
ENSBTAG00000036411	*bta-mir-374a*	0	0	0	0	0	NA	NA	NA
ENSBTAG00000036414	*bta-mir-451*	0	0	0	0	0	NA	NA	NA
ENSBTAG00000036415	*bta-mir-496*	0	0	0	0	0	NA	NA	NA
ENSBTAG00000036418	*miRNA*	0	0	0	0	0	NA	NA	NA
ENSBTAG00000036420	*bta-mir-500*	0	0	0	0	0	NA	NA	NA
ENSBTAG00000036422	*bta-mir-95*	0	0	0	0	0	NA	NA	NA
ENSBTAG00000036423	*bta-mir-146b*	0	0	0	0	0	NA	NA	NA
ENSBTAG00000036425	*bta-mir-7-1*	0	0	0	0	0	NA	NA	NA
ENSBTAG00000036429	*bta-mir-302c*	0	0	0	0	0	NA	NA	NA
ENSBTAG00000036431	*bta-mir-543*	0	0	0	0	0	NA	NA	NA
ENSBTAG00000036436	*bta-mir-153-1*	0	0	0	0	0	NA	NA	NA
ENSBTAG00000036440	*miRNA*	0	0	0	0	0	NA	NA	NA
ENSBTAG00000036442	*miRNA*	0	0	0	0	0	NA	NA	NA
ENSBTAG00000036443	*bta-mir-301b*	0	0	0	0	0	NA	NA	NA
ENSBTAG00000036451	*5S_rRNA*	0	0	0	0	0	NA	NA	NA
ENSBTAG00000036475	*5S_rRNA*	0	0	0	0	0	NA	NA	NA
ENSBTAG00000036476	*5S_rRNA*	0	0	0	0	0	NA	NA	NA
ENSBTAG00000036480	*5S_rRNA*	0	0	0	0	0	NA	NA	NA
ENSBTAG00000036516	*5S_rRNA*	0	0	0	0	0	NA	NA	NA
ENSBTAG00000036542	*5S_rRNA*	0	0	0	0	0	NA	NA	NA
ENSBTAG00000036553	*5S_rRNA*	0	0	0	0	0	NA	NA	NA
ENSBTAG00000036562	*U1*	0	0	0	0	0	NA	NA	NA
ENSBTAG00000036587	*U3*	0	0	0	0	0	NA	NA	NA
ENSBTAG00000036603	*5S_rRNA*	0	0	0	0	0	NA	NA	NA
ENSBTAG00000036616	*5S_rRNA*	0	0	0	0	0	NA	NA	NA
ENSBTAG00000036618	*U2*	0	0	0	0	0	NA	NA	NA
ENSBTAG00000036621	*5S_rRNA*	0	0	0	0	0	NA	NA	NA
ENSBTAG00000036628	*5S_rRNA*	0	0	0	0	0	NA	NA	NA
ENSBTAG00000036635	*5S_rRNA*	0	0	0	0	0	NA	NA	NA
ENSBTAG00000036637	*5S_rRNA*	0	0	0	0	0	NA	NA	NA
ENSBTAG00000036644	*5S_rRNA*	0	0	0	0	0	NA	NA	NA
ENSBTAG00000036646	*5S_rRNA*	0	0	0	0	0	NA	NA	NA
ENSBTAG00000036649	*5S_rRNA*	0	0	0	0	0	NA	NA	NA
ENSBTAG00000036659	*U2*	0	0	0	0	0	NA	NA	NA
ENSBTAG00000036678	*5S_rRNA*	0	0	0	0	0	NA	NA	NA
ENSBTAG00000036754	*U12*	0	0	0	0	0	NA	NA	NA
ENSBTAG00000036777	*5S_rRNA*	0	0	0	0	0	NA	NA	NA
ENSBTAG00000036794	*5S_rRNA*	0	0	0	0	0	NA	NA	NA
ENSBTAG00000036807	*5S_rRNA*	0	0	0	0	0	NA	NA	NA
ENSBTAG00000036823	*5S_rRNA*	0	0	0	0	0	NA	NA	NA
ENSBTAG00000036841	*5S_rRNA*	0	0	0	0	0	NA	NA	NA
ENSBTAG00000036851	*U1*	0	0	0	0	0	NA	NA	NA
ENSBTAG00000036856	*5S_rRNA*	0	0	0	0	0	NA	NA	NA
ENSBTAG00000036889	*5S_rRNA*	0	0	0	0	0	NA	NA	NA

Ensembl gene ID	geneName	counts wildtype horn bud	counts polled horn bud	baseMean	baseMean wildtype horn bud	baseMean polled horn bud	log2FoldChange	pval	padj
ENSBTAG00000036902	*5S_rRNA*	0	0	0	0	0	NA	NA	NA
ENSBTAG00000036908	*5S_rRNA*	0	0	0	0	0	NA	NA	NA
ENSBTAG00000036921	*5S_rRNA*	0	0	0	0	0	NA	NA	NA
ENSBTAG00000036927	*U3*	0	0	0	0	0	NA	NA	NA
ENSBTAG00000036933	*5S_rRNA*	0	0	0	0	0	NA	NA	NA
ENSBTAG00000036942	*5S_rRNA*	0	0	0	0	0	NA	NA	NA
ENSBTAG00000036945	*5S_rRNA*	0	0	0	0	0	NA	NA	NA
ENSBTAG00000036946	*U1*	0	0	0	0	0	NA	NA	NA
ENSBTAG00000036950	*5S_rRNA*	0	0	0	0	0	NA	NA	NA
ENSBTAG00000036958	*U2*	0	0	0	0	0	NA	NA	NA
ENSBTAG00000036959	*U1*	0	0	0	0	0	NA	NA	NA
ENSBTAG00000036962	*5S_rRNA*	0	0	0	0	0	NA	NA	NA
ENSBTAG00000036968	*U3*	0	0	0	0	0	NA	NA	NA
ENSBTAG00000036970	*5S_rRNA*	0	0	0	0	0	NA	NA	NA
ENSBTAG00000036978	*5S_rRNA*	0	0	0	0	0	NA	NA	NA
ENSBTAG00000036982	*5S_rRNA*	0	0	0	0	0	NA	NA	NA
ENSBTAG00000036995	*5S_rRNA*	0	0	0	0	0	NA	NA	NA
ENSBTAG00000037006	*5S_rRNA*	0	0	0	0	0	NA	NA	NA
ENSBTAG00000037009	*U2*	0	0	0	0	0	NA	NA	NA
ENSBTAG00000037010	*5S_rRNA*	0	0	0	0	0	NA	NA	NA
ENSBTAG00000037026	*5S_rRNA*	0	0	0	0	0	NA	NA	NA
ENSBTAG00000037030	*U3*	0	0	0	0	0	NA	NA	NA
ENSBTAG00000037072	*U1*	0	0	0	0	0	NA	NA	NA
ENSBTAG00000037085	*5S_rRNA*	0	0	0	0	0	NA	NA	NA
ENSBTAG00000037096	*5S_rRNA*	0	0	0	0	0	NA	NA	NA
ENSBTAG00000037111	*5S_rRNA*	0	0	0	0	0	NA	NA	NA
ENSBTAG00000037147	*U3*	0	0	0	0	0	NA	NA	NA
ENSBTAG00000037153	*5S_rRNA*	0	0	0	0	0	NA	NA	NA
ENSBTAG00000037167	*5S_rRNA*	0	0	0	0	0	NA	NA	NA
ENSBTAG00000037174	*5S_rRNA*	0	0	0	0	0	NA	NA	NA
ENSBTAG00000037182	*5S_rRNA*	0	0	0	0	0	NA	NA	NA
ENSBTAG00000037199	*5S_rRNA*	0	0	0	0	0	NA	NA	NA
ENSBTAG00000037205	*5S_rRNA*	0	0	0	0	0	NA	NA	NA
ENSBTAG00000037206	*U1*	0	0	0	0	0	NA	NA	NA
ENSBTAG00000037226	*5S_rRNA*	0	0	0	0	0	NA	NA	NA
ENSBTAG00000037239	*5S_rRNA*	0	0	0	0	0	NA	NA	NA
ENSBTAG00000037243	*5S_rRNA*	0	0	0	0	0	NA	NA	NA
ENSBTAG00000037250	*miRNA*	0	0	0	0	0	NA	NA	NA
ENSBTAG00000037257	*miRNA*	0	0	0	0	0	NA	NA	NA
ENSBTAG00000037263	*bta-mir-541*	0	0	0	0	0	NA	NA	NA
ENSBTAG00000037264	*bta-mir-486*	0	0	0	0	0	NA	NA	NA
ENSBTAG00000037268	*miRNA*	0	0	0	0	0	NA	NA	NA
ENSBTAG00000037274	*miRNA*	0	0	0	0	0	NA	NA	NA
ENSBTAG00000037275	*bta-mir-190b*	0	0	0	0	0	NA	NA	NA
ENSBTAG00000037277	*miRNA*	0	0	0	0	0	NA	NA	NA
ENSBTAG00000037288	*miRNA*	0	0	0	0	0	NA	NA	NA
ENSBTAG00000037293	*miRNA*	0	0	0	0	0	NA	NA	NA
ENSBTAG00000037296	*miRNA*	0	0	0	0	0	NA	NA	NA
ENSBTAG00000037297	*miRNA*	0	0	0	0	0	NA	NA	NA
ENSBTAG00000037300	*miRNA*	0	0	0	0	0	NA	NA	NA
ENSBTAG00000037302	*miRNA*	0	0	0	0	0	NA	NA	NA
ENSBTAG00000037306	*miRNA*	0	0	0	0	0	NA	NA	NA
ENSBTAG00000037307	*bta-mir-874*	0	0	0	0	0	NA	NA	NA
ENSBTAG00000037311	*miRNA*	0	0	0	0	0	NA	NA	NA
ENSBTAG00000037316	*miRNA*	0	0	0	0	0	NA	NA	NA
ENSBTAG00000037322	*miRNA*	0	0	0	0	0	NA	NA	NA
ENSBTAG00000037325	*miRNA*	0	0	0	0	0	NA	NA	NA
ENSBTAG00000037328	*miRNA*	0	0	0	0	0	NA	NA	NA
ENSBTAG00000037330	*miRNA*	0	0	0	0	0	NA	NA	NA
ENSBTAG00000037332	*miRNA*	0	0	0	0	0	NA	NA	NA
ENSBTAG00000037336	*miRNA*	0	0	0	0	0	NA	NA	NA
ENSBTAG00000037337	*bta-mir-877*	0	0	0	0	0	NA	NA	NA
ENSBTAG00000037338	*bta-mir-935*	0	0	0	0	0	NA	NA	NA
ENSBTAG00000037350	*miRNA*	0	0	0	0	0	NA	NA	NA
ENSBTAG00000037353	*miRNA*	0	0	0	0	0	NA	NA	NA
ENSBTAG00000037357	*miRNA*	0	0	0	0	0	NA	NA	NA
ENSBTAG00000037358	*bta-mir-885*	0	0	0	0	0	NA	NA	NA
ENSBTAG00000037360	*miRNA*	0	0	0	0	0	NA	NA	NA
ENSBTAG00000037363	*miRNA*	0	0	0	0	0	NA	NA	NA
ENSBTAG00000037365	*miRNA*	0	0	0	0	0	NA	NA	NA
ENSBTAG00000037369	*bta-mir-876*	0	0	0	0	0	NA	NA	NA
ENSBTAG00000037384	*OR10AB1P*	0	0	0	0	0	NA	NA	NA

Ensembl gene ID	geneName	counts wildtype horn bud	counts polled horn bud	baseMean	baseMean wildtype horn bud	baseMean polled horn bud	log2FoldChange	pval	padj
ENSBTAG00000037398	*OR51T1*	0	0	0	0	0	NA	NA	NA
ENSBTAG00000037411	*protein_coding*	0	0	0	0	0	NA	NA	NA
ENSBTAG00000037416	*protein_coding*	0	0	0	0	0	NA	NA	NA
ENSBTAG00000037432	*protein_coding*	0	0	0	0	0	NA	NA	NA
ENSBTAG00000037436	*pseudogene*	0	0	0	0	0	NA	NA	NA
ENSBTAG00000037453	*BT.104867*	0	0	0	0	0	NA	NA	NA
ENSBTAG00000037454	*miRNA*	0	0	0	0	0	NA	NA	NA
ENSBTAG00000037458	*protein_coding*	0	0	0	0	0	NA	NA	NA
ENSBTAG00000037462	*BT.98278*	0	0	0	0	0	NA	NA	NA
ENSBTAG00000037464	*TGM6*	0	0	0	0	0	NA	NA	NA
ENSBTAG00000037467	*protein_coding*	0	0	0	0	0	NA	NA	NA
ENSBTAG00000037469	*protein_coding*	0	0	0	0	0	NA	NA	NA
ENSBTAG00000037474	*protein_coding*	0	0	0	0	0	NA	NA	NA
ENSBTAG00000037485	*OR6M1*	0	0	0	0	0	NA	NA	NA
ENSBTAG00000037492	*miRNA*	0	0	0	0	0	NA	NA	NA
ENSBTAG00000037496	*protein_coding*	0	0	0	0	0	NA	NA	NA
ENSBTAG00000037505	*protein_coding*	0	0	0	0	0	NA	NA	NA
ENSBTAG00000037509	*AKR1C4*	0	0	0	0	0	NA	NA	NA
ENSBTAG00000037511	*protein_coding*	0	0	0	0	0	NA	NA	NA
ENSBTAG00000037517	*pseudogene*	0	0	0	0	0	NA	NA	NA
ENSBTAG00000037529	*protein_coding*	0	0	0	0	0	NA	NA	NA
ENSBTAG00000037538	*protein_coding*	0	0	0	0	0	NA	NA	NA
ENSBTAG00000037542	*protein_coding*	0	0	0	0	0	NA	NA	NA
ENSBTAG00000037546	*protein_coding*	0	0	0	0	0	NA	NA	NA
ENSBTAG00000037550	*protein_coding*	0	0	0	0	0	NA	NA	NA
ENSBTAG00000037570	*BT.88767*	0	0	0	0	0	NA	NA	NA
ENSBTAG00000037575	*protein_coding*	0	0	0	0	0	NA	NA	NA
ENSBTAG00000037577	*protein_coding*	0	0	0	0	0	NA	NA	NA
ENSBTAG00000037597	*protein_coding*	0	0	0	0	0	NA	NA	NA
ENSBTAG00000037598	*protein_coding*	0	0	0	0	0	NA	NA	NA
ENSBTAG00000037602	*protein_coding*	0	0	0	0	0	NA	NA	NA
ENSBTAG00000037603	*protein_coding*	0	0	0	0	0	NA	NA	NA
ENSBTAG00000037611	*protein_coding*	0	0	0	0	0	NA	NA	NA
ENSBTAG00000037613	*BT.64372*	0	0	0	0	0	NA	NA	NA
ENSBTAG00000037623	*protein_coding*	0	0	0	0	0	NA	NA	NA
ENSBTAG00000037628	*protein_coding*	0	0	0	0	0	NA	NA	NA
ENSBTAG00000037629	*protein_coding*	0	0	0	0	0	NA	NA	NA
ENSBTAG00000037637	*5_8S_rRNA*	0	0	0	0	0	NA	NA	NA
ENSBTAG00000037638	*KRT73*	0	0	0	0	0	NA	NA	NA
ENSBTAG00000037645	*protein_coding*	0	0	0	0	0	NA	NA	NA
ENSBTAG00000037647	*protein_coding*	0	0	0	0	0	NA	NA	NA
ENSBTAG00000037660	*protein_coding*	0	0	0	0	0	NA	NA	NA
ENSBTAG00000037666	*protein_coding*	0	0	0	0	0	NA	NA	NA
ENSBTAG00000037684	*pseudogene*	0	0	0	0	0	NA	NA	NA
ENSBTAG00000037690	*bta-mir-1287*	0	0	0	0	0	NA	NA	NA
ENSBTAG00000037704	*protein_coding*	0	0	0	0	0	NA	NA	NA
ENSBTAG00000037710	*protein_coding*	0	0	0	0	0	NA	NA	NA
ENSBTAG00000037718	*TAAR1*	0	0	0	0	0	NA	NA	NA
ENSBTAG00000037728	*PAGE4*	0	0	0	0	0	NA	NA	NA
ENSBTAG00000037738	*SLC17A4*	0	0	0	0	0	NA	NA	NA
ENSBTAG00000037739	*protein_coding*	0	0	0	0	0	NA	NA	NA
ENSBTAG00000037751	*HBE2*	0	0	0	0	0	NA	NA	NA
ENSBTAG00000037758	*protein_coding*	0	0	0	0	0	NA	NA	NA
ENSBTAG00000037764	*BT.50381*	0	0	0	0	0	NA	NA	NA
ENSBTAG00000037767	*OR1N1*	0	0	0	0	0	NA	NA	NA
ENSBTAG00000037770	*protein_coding*	0	0	0	0	0	NA	NA	NA
ENSBTAG00000037779	*miRNA*	0	0	0	0	0	NA	NA	NA
ENSBTAG00000037782	*bta-mir-1225*	0	0	0	0	0	NA	NA	NA
ENSBTAG00000037783	*MUC19*	0	0	0	0	0	NA	NA	NA
ENSBTAG00000037784	*MGC157405*	0	0	0	0	0	NA	NA	NA
ENSBTAG00000037788	*miRNA*	0	0	0	0	0	NA	NA	NA
ENSBTAG00000037793	*protein_coding*	0	0	0	0	0	NA	NA	NA
ENSBTAG00000037807	*protein_coding*	0	0	0	0	0	NA	NA	NA
ENSBTAG00000037810	*protein_coding*	0	0	0	0	0	NA	NA	NA
ENSBTAG00000037812	*RAD51AP2*	0	0	0	0	0	NA	NA	NA
ENSBTAG00000037816	*protein_coding*	0	0	0	0	0	NA	NA	NA
ENSBTAG00000037820	*protein_coding*	0	0	0	0	0	NA	NA	NA
ENSBTAG00000037822	*protein_coding*	0	0	0	0	0	NA	NA	NA
ENSBTAG00000037831	*miRNA*	0	0	0	0	0	NA	NA	NA
ENSBTAG00000037833	*miRNA*	0	0	0	0	0	NA	NA	NA
ENSBTAG00000037842	*protein_coding*	0	0	0	0	0	NA	NA	NA
ENSBTAG00000037853	*protein_coding*	0	0	0	0	0	NA	NA	NA

Ensembl gene ID	geneName	counts wildtype horn bud	counts polled horn bud	baseMean	baseMean wildtype horn bud	baseMean polled horn bud	log2FoldChange	pval	padj
ENSBTAG00000037878	*protein_coding*	0	0	0	0	0	NA	NA	NA
ENSBTAG00000037881	*FAM32A*	0	0	0	0	0	NA	NA	NA
ENSBTAG00000037883	*protein_coding*	0	0	0	0	0	NA	NA	NA
ENSBTAG00000037886	*protein_coding*	0	0	0	0	0	NA	NA	NA
ENSBTAG00000037890	*BT.102219*	0	0	0	0	0	NA	NA	NA
ENSBTAG00000037897	*miRNA*	0	0	0	0	0	NA	NA	NA
ENSBTAG00000037903	*pseudogene*	0	0	0	0	0	NA	NA	NA
ENSBTAG00000037908	*PAG1*	0	0	0	0	0	NA	NA	NA
ENSBTAG00000037918	*protein_coding*	0	0	0	0	0	NA	NA	NA
ENSBTAG00000037924	*BT.29692*	0	0	0	0	0	NA	NA	NA
ENSBTAG00000037927	*protein_coding*	0	0	0	0	0	NA	NA	NA
ENSBTAG00000037928	*protein_coding*	0	0	0	0	0	NA	NA	NA
ENSBTAG00000037933	*BT.98298*	0	0	0	0	0	NA	NA	NA
ENSBTAG00000037943	*protein_coding*	0	0	0	0	0	NA	NA	NA
ENSBTAG00000037948	*miRNA*	0	0	0	0	0	NA	NA	NA
ENSBTAG00000037949	*BT.67381*	0	0	0	0	0	NA	NA	NA
ENSBTAG00000037959	*protein_coding*	0	0	0	0	0	NA	NA	NA
ENSBTAG00000037969	*MGC152301*	0	0	0	0	0	NA	NA	NA
ENSBTAG00000037977	*protein_coding*	0	0	0	0	0	NA	NA	NA
ENSBTAG00000037992	*OR51Q1*	0	0	0	0	0	NA	NA	NA
ENSBTAG00000037993	*protein_coding*	0	0	0	0	0	NA	NA	NA
ENSBTAG00000037994	*pseudogene*	0	0	0	0	0	NA	NA	NA
ENSBTAG00000038000	*protein_coding*	0	0	0	0	0	NA	NA	NA
ENSBTAG00000038022	*BT.106042*	0	0	0	0	0	NA	NA	NA
ENSBTAG00000038024	*C19ORF75*	0	0	0	0	0	NA	NA	NA
ENSBTAG00000038026	*miRNA*	0	0	0	0	0	NA	NA	NA
ENSBTAG00000038027	*protein_coding*	0	0	0	0	0	NA	NA	NA
ENSBTAG00000038030	*protein_coding*	0	0	0	0	0	NA	NA	NA
ENSBTAG00000038031	*protein_coding*	0	0	0	0	0	NA	NA	NA
ENSBTAG00000038035	*miRNA*	0	0	0	0	0	NA	NA	NA
ENSBTAG00000038049	*protein_coding*	0	0	0	0	0	NA	NA	NA
ENSBTAG00000038057	*protein_coding*	0	0	0	0	0	NA	NA	NA
ENSBTAG00000038058	*BT.88117*	0	0	0	0	0	NA	NA	NA
ENSBTAG00000038059	*OR1E1*	0	0	0	0	0	NA	NA	NA
ENSBTAG00000038060	*BT.101619*	0	0	0	0	0	NA	NA	NA
ENSBTAG00000038069	*FAM188B2*	0	0	0	0	0	NA	NA	NA
ENSBTAG00000038075	*protein_coding*	0	0	0	0	0	NA	NA	NA
ENSBTAG00000038080	*protein_coding*	0	0	0	0	0	NA	NA	NA
ENSBTAG00000038082	*protein_coding*	0	0	0	0	0	NA	NA	NA
ENSBTAG00000038083	*protein_coding*	0	0	0	0	0	NA	NA	NA
ENSBTAG00000038086	*protein_coding*	0	0	0	0	0	NA	NA	NA
ENSBTAG00000038094	*miRNA*	0	0	0	0	0	NA	NA	NA
ENSBTAG00000038098	*TRAV29DV5*	0	0	0	0	0	NA	NA	NA
ENSBTAG00000038103	*DPPA4*	0	0	0	0	0	NA	NA	NA
ENSBTAG00000038106	*miRNA*	0	0	0	0	0	NA	NA	NA
ENSBTAG00000038127	*protein_coding*	0	0	0	0	0	NA	NA	NA
ENSBTAG00000038160	*protein_coding*	0	0	0	0	0	NA	NA	NA
ENSBTAG00000038161	*protein_coding*	0	0	0	0	0	NA	NA	NA
ENSBTAG00000038166	*pseudogene*	0	0	0	0	0	NA	NA	NA
ENSBTAG00000038169	*protein_coding*	0	0	0	0	0	NA	NA	NA
ENSBTAG00000038170	*protein_coding*	0	0	0	0	0	NA	NA	NA
ENSBTAG00000038176	*miRNA*	0	0	0	0	0	NA	NA	NA
ENSBTAG00000038182	*protein_coding*	0	0	0	0	0	NA	NA	NA
ENSBTAG00000038188	*protein_coding*	0	0	0	0	0	NA	NA	NA
ENSBTAG00000038201	*OR2A12*	0	0	0	0	0	NA	NA	NA
ENSBTAG00000038205	*bta-mir-1298*	0	0	0	0	0	NA	NA	NA
ENSBTAG00000038206	*bta-mir-1185*	0	0	0	0	0	NA	NA	NA
ENSBTAG00000038209	*protein_coding*	0	0	0	0	0	NA	NA	NA
ENSBTAG00000038214	*protein_coding*	0	0	0	0	0	NA	NA	NA
ENSBTAG00000038222	*protein_coding*	0	0	0	0	0	NA	NA	NA
ENSBTAG00000038227	*protein_coding*	0	0	0	0	0	NA	NA	NA
ENSBTAG00000038245	*protein_coding*	0	0	0	0	0	NA	NA	NA
ENSBTAG00000038252	*miRNA*	0	0	0	0	0	NA	NA	NA
ENSBTAG00000038257	*protein_coding*	0	0	0	0	0	NA	NA	NA
ENSBTAG00000038259	*miRNA*	0	0	0	0	0	NA	NA	NA
ENSBTAG00000038261	*protein_coding*	0	0	0	0	0	NA	NA	NA
ENSBTAG00000038264	*protein_coding*	0	0	0	0	0	NA	NA	NA
ENSBTAG00000038267	*protein_coding*	0	0	0	0	0	NA	NA	NA
ENSBTAG00000038268	*BT.86130*	0	0	0	0	0	NA	NA	NA
ENSBTAG00000038276	*miRNA*	0	0	0	0	0	NA	NA	NA
ENSBTAG00000038278	*protein_coding*	0	0	0	0	0	NA	NA	NA
ENSBTAG00000038290	*protein_coding*	0	0	0	0	0	NA	NA	NA

Ensembl gene ID	geneName	counts wildtype horn bud	counts polled horn bud	baseMean	baseMean wildtype horn bud	baseMean polled horn bud	log2FoldChange	pval	padj
ENSBTAG00000038291	*protein_coding*	0	0	0	0	0	NA	NA	NA
ENSBTAG00000038307	*protein_coding*	0	0	0	0	0	NA	NA	NA
ENSBTAG00000038308	*protein_coding*	0	0	0	0	0	NA	NA	NA
ENSBTAG00000038319	*OR5M9*	0	0	0	0	0	NA	NA	NA
ENSBTAG00000038345	*OR8B8*	0	0	0	0	0	NA	NA	NA
ENSBTAG00000038362	*protein_coding*	0	0	0	0	0	NA	NA	NA
ENSBTAG00000038364	*protein_coding*	0	0	0	0	0	NA	NA	NA
ENSBTAG00000038369	*SULT1C2*	0	0	0	0	0	NA	NA	NA
ENSBTAG00000038386	*KRTAP4-7*	0	0	0	0	0	NA	NA	NA
ENSBTAG00000038387	*SPIN2B*	0	0	0	0	0	NA	NA	NA
ENSBTAG00000038395	*protein_coding*	0	0	0	0	0	NA	NA	NA
ENSBTAG00000038397	*protein_coding*	0	0	0	0	0	NA	NA	NA
ENSBTAG00000038401	*miRNA*	0	0	0	0	0	NA	NA	NA
ENSBTAG00000038414	*TAAR2*	0	0	0	0	0	NA	NA	NA
ENSBTAG00000038416	*BOSTAUV1R427*	0	0	0	0	0	NA	NA	NA
ENSBTAG00000038418	*protein_coding*	0	0	0	0	0	NA	NA	NA
ENSBTAG00000038426	*protein_coding*	0	0	0	0	0	NA	NA	NA
ENSBTAG00000038437	*RLN3*	0	0	0	0	0	NA	NA	NA
ENSBTAG00000038447	*protein_coding*	0	0	0	0	0	NA	NA	NA
ENSBTAG00000038448	*protein_coding*	0	0	0	0	0	NA	NA	NA
ENSBTAG00000038452	*OR52E8*	0	0	0	0	0	NA	NA	NA
ENSBTAG00000038453	*protein_coding*	0	0	0	0	0	NA	NA	NA
ENSBTAG00000038463	*bta-mir-1251*	0	0	0	0	0	NA	NA	NA
ENSBTAG00000038472	*OR5V1*	0	0	0	0	0	NA	NA	NA
ENSBTAG00000038479	*protein_coding*	0	0	0	0	0	NA	NA	NA
ENSBTAG00000038483	*BT.64159*	0	0	0	0	0	NA	NA	NA
ENSBTAG00000038485	*protein_coding*	0	0	0	0	0	NA	NA	NA
ENSBTAG00000038489	*DCDC2C*	0	0	0	0	0	NA	NA	NA
ENSBTAG00000038497	*BT.88995*	0	0	0	0	0	NA	NA	NA
ENSBTAG00000038502	*BT.94719*	0	0	0	0	0	NA	NA	NA
ENSBTAG00000038506	*protein_coding*	0	0	0	0	0	NA	NA	NA
ENSBTAG00000038518	*OR2D3*	0	0	0	0	0	NA	NA	NA
ENSBTAG00000038521	*protein_coding*	0	0	0	0	0	NA	NA	NA
ENSBTAG00000038526	*protein_coding*	0	0	0	0	0	NA	NA	NA
ENSBTAG00000038531	*protein_coding*	0	0	0	0	0	NA	NA	NA
ENSBTAG00000038533	*protein_coding*	0	0	0	0	0	NA	NA	NA
ENSBTAG00000038537	*protein_coding*	0	0	0	0	0	NA	NA	NA
ENSBTAG00000038539	*protein_coding*	0	0	0	0	0	NA	NA	NA
ENSBTAG00000038546	*bta-mir-1284*	0	0	0	0	0	NA	NA	NA
ENSBTAG00000038549	*BT.48084*	0	0	0	0	0	NA	NA	NA
ENSBTAG00000038564	*protein_coding*	0	0	0	0	0	NA	NA	NA
ENSBTAG00000038567	*protein_coding*	0	0	0	0	0	NA	NA	NA
ENSBTAG00000038578	*pseudogene*	0	0	0	0	0	NA	NA	NA
ENSBTAG00000038579	*protein_coding*	0	0	0	0	0	NA	NA	NA
ENSBTAG00000038590	*protein_coding*	0	0	0	0	0	NA	NA	NA
ENSBTAG00000038593	*miRNA*	0	0	0	0	0	NA	NA	NA
ENSBTAG00000038595	*MAGEB16*	0	0	0	0	0	NA	NA	NA
ENSBTAG00000038597	*protein_coding*	0	0	0	0	0	NA	NA	NA
ENSBTAG00000038608	*protein_coding*	0	0	0	0	0	NA	NA	NA
ENSBTAG00000038623	*protein_coding*	0	0	0	0	0	NA	NA	NA
ENSBTAG00000038629	*OR2Y1*	0	0	0	0	0	NA	NA	NA
ENSBTAG00000038636	*protein_coding*	0	0	0	0	0	NA	NA	NA
ENSBTAG00000038637	*protein_coding*	0	0	0	0	0	NA	NA	NA
ENSBTAG00000038641	*OR10A3*	0	0	0	0	0	NA	NA	NA
ENSBTAG00000038643	*OR11H4*	0	0	0	0	0	NA	NA	NA
ENSBTAG00000038648	*protein_coding*	0	0	0	0	0	NA	NA	NA
ENSBTAG00000038651	*protein_coding*	0	0	0	0	0	NA	NA	NA
ENSBTAG00000038655	*BT.68446*	0	0	0	0	0	NA	NA	NA
ENSBTAG00000038665	*protein_coding*	0	0	0	0	0	NA	NA	NA
ENSBTAG00000038667	*pseudogene*	0	0	0	0	0	NA	NA	NA
ENSBTAG00000038668	*U3*	0	0	0	0	0	NA	NA	NA
ENSBTAG00000038669	*protein_coding*	0	0	0	0	0	NA	NA	NA
ENSBTAG00000038670	*OR8S1*	0	0	0	0	0	NA	NA	NA
ENSBTAG00000038672	*protein_coding*	0	0	0	0	0	NA	NA	NA
ENSBTAG00000038677	*pseudogene*	0	0	0	0	0	NA	NA	NA
ENSBTAG00000038679	*protein_coding*	0	0	0	0	0	NA	NA	NA
ENSBTAG00000038684	*BT.57843*	0	0	0	0	0	NA	NA	NA
ENSBTAG00000038687	*BPIFB3*	0	0	0	0	0	NA	NA	NA
ENSBTAG00000038690	*protein_coding*	0	0	0	0	0	NA	NA	NA
ENSBTAG00000038697	*miRNA*	0	0	0	0	0	NA	NA	NA
ENSBTAG00000038714	*protein_coding*	0	0	0	0	0	NA	NA	NA
ENSBTAG00000038718	*protein_coding*	0	0	0	0	0	NA	NA	NA

Ensembl gene ID	geneName	counts wildtype horn bud	counts polled horn bud	baseMean	baseMean wildtype horn bud	baseMean polled horn bud	log2FoldChange	pval	padj
ENSBTAG00000038722	*protein_coding*	0	0	0	0	0	NA	NA	NA
ENSBTAG00000038726	*protein_coding*	0	0	0	0	0	NA	NA	NA
ENSBTAG00000038727	*protein_coding*	0	0	0	0	0	NA	NA	NA
ENSBTAG00000038732	*protein_coding*	0	0	0	0	0	NA	NA	NA
ENSBTAG00000038751	*MAGEB18*	0	0	0	0	0	NA	NA	NA
ENSBTAG00000038759	*protein_coding*	0	0	0	0	0	NA	NA	NA
ENSBTAG00000038761	*protein_coding*	0	0	0	0	0	NA	NA	NA
ENSBTAG00000038765	*miRNA*	0	0	0	0	0	NA	NA	NA
ENSBTAG00000038787	*protein_coding*	0	0	0	0	0	NA	NA	NA
ENSBTAG00000038796	*protein_coding*	0	0	0	0	0	NA	NA	NA
ENSBTAG00000038798	*protein_coding*	0	0	0	0	0	NA	NA	NA
ENSBTAG00000038802	*OR8D4*	0	0	0	0	0	NA	NA	NA
ENSBTAG00000038813	*5S_rRNA*	0	0	0	0	0	NA	NA	NA
ENSBTAG00000038822	*pseudogene*	0	0	0	0	0	NA	NA	NA
ENSBTAG00000038823	*protein_coding*	0	0	0	0	0	NA	NA	NA
ENSBTAG00000038824	*pseudogene*	0	0	0	0	0	NA	NA	NA
ENSBTAG00000038835	*protein_coding*	0	0	0	0	0	NA	NA	NA
ENSBTAG00000038838	*miRNA*	0	0	0	0	0	NA	NA	NA
ENSBTAG00000038843	*BT.102908*	0	0	0	0	0	NA	NA	NA
ENSBTAG00000038858	*CKLF*	0	0	0	0	0	NA	NA	NA
ENSBTAG00000038868	*protein_coding*	0	0	0	0	0	NA	NA	NA
ENSBTAG00000038876	*protein_coding*	0	0	0	0	0	NA	NA	NA
ENSBTAG00000038890	*BOSTAUV1R431*	0	0	0	0	0	NA	NA	NA
ENSBTAG00000038894	*OR6Y1*	0	0	0	0	0	NA	NA	NA
ENSBTAG00000038899	*protein_coding*	0	0	0	0	0	NA	NA	NA
ENSBTAG00000038908	*protein_coding*	0	0	0	0	0	NA	NA	NA
ENSBTAG00000038911	*protein_coding*	0	0	0	0	0	NA	NA	NA
ENSBTAG00000038918	*protein_coding*	0	0	0	0	0	NA	NA	NA
ENSBTAG00000038919	*miRNA*	0	0	0	0	0	NA	NA	NA
ENSBTAG00000038921	*BT.103486*	0	0	0	0	0	NA	NA	NA
ENSBTAG00000038930	*protein_coding*	0	0	0	0	0	NA	NA	NA
ENSBTAG00000038934	*protein_coding*	0	0	0	0	0	NA	NA	NA
ENSBTAG00000038941	*protein_coding*	0	0	0	0	0	NA	NA	NA
ENSBTAG00000038943	*BT.92689*	0	0	0	0	0	NA	NA	NA
ENSBTAG00000038947	*ZSCAN4*	0	0	0	0	0	NA	NA	NA
ENSBTAG00000038958	*pseudogene*	0	0	0	0	0	NA	NA	NA
ENSBTAG00000038961	*protein_coding*	0	0	0	0	0	NA	NA	NA
ENSBTAG00000038963	*protein_coding*	0	0	0	0	0	NA	NA	NA
ENSBTAG00000038966	*BT.70110*	0	0	0	0	0	NA	NA	NA
ENSBTAG00000038968	*pseudogene*	0	0	0	0	0	NA	NA	NA
ENSBTAG00000038976	*ZGLP1*	0	0	0	0	0	NA	NA	NA
ENSBTAG00000038977	*OR52L1*	0	0	0	0	0	NA	NA	NA
ENSBTAG00000038980	*protein_coding*	0	0	0	0	0	NA	NA	NA
ENSBTAG00000038983	*protein_coding*	0	0	0	0	0	NA	NA	NA
ENSBTAG00000038986	*BOSTAUV1R403*	0	0	0	0	0	NA	NA	NA
ENSBTAG00000039005	*OR2A2*	0	0	0	0	0	NA	NA	NA
ENSBTAG00000039011	*HTR3D*	0	0	0	0	0	NA	NA	NA
ENSBTAG00000039018	*OR1G1*	0	0	0	0	0	NA	NA	NA
ENSBTAG00000039029	*pseudogene*	0	0	0	0	0	NA	NA	NA
ENSBTAG00000039031	*protein_coding*	0	0	0	0	0	NA	NA	NA
ENSBTAG00000039036	*PAG-18*	0	0	0	0	0	NA	NA	NA
ENSBTAG00000039038	*protein_coding*	0	0	0	0	0	NA	NA	NA
ENSBTAG00000039045	*protein_coding*	0	0	0	0	0	NA	NA	NA
ENSBTAG00000039054	*protein_coding*	0	0	0	0	0	NA	NA	NA
ENSBTAG00000039056	*protein_coding*	0	0	0	0	0	NA	NA	NA
ENSBTAG00000039058	*protein_coding*	0	0	0	0	0	NA	NA	NA
ENSBTAG00000039068	*CSMD3*	0	0	0	0	0	NA	NA	NA
ENSBTAG00000039074	*miRNA*	0	0	0	0	0	NA	NA	NA
ENSBTAG00000039079	*protein_coding*	0	0	0	0	0	NA	NA	NA
ENSBTAG00000039081	*protein_coding*	0	0	0	0	0	NA	NA	NA
ENSBTAG00000039086	*protein_coding*	0	0	0	0	0	NA	NA	NA
ENSBTAG00000039088	*protein_coding*	0	0	0	0	0	NA	NA	NA
ENSBTAG00000039099	*pseudogene*	0	0	0	0	0	NA	NA	NA
ENSBTAG00000039104	*miRNA*	0	0	0	0	0	NA	NA	NA
ENSBTAG00000039110	*OR8U1*	0	0	0	0	0	NA	NA	NA
ENSBTAG00000039113	*MSGN1*	0	0	0	0	0	NA	NA	NA
ENSBTAG00000039116	*protein_coding*	0	0	0	0	0	NA	NA	NA
ENSBTAG00000039120	*protein_coding*	0	0	0	0	0	NA	NA	NA
ENSBTAG00000039127	*miRNA*	0	0	0	0	0	NA	NA	NA
ENSBTAG00000039134	*protein_coding*	0	0	0	0	0	NA	NA	NA
ENSBTAG00000039136	*pseudogene*	0	0	0	0	0	NA	NA	NA
ENSBTAG00000039145	*protein_coding*	0	0	0	0	0	NA	NA	NA

Ensembl gene ID	geneName	counts wildtype horn bud	counts polled horn bud	baseMean	baseMean wildtype horn bud	baseMean polled horn bud	log2FoldChange	pval	padj
ENSBTAG00000039149	*CLDN25*	0	0	0	0	0	NA	NA	NA
ENSBTAG00000039152	*protein_coding*	0	0	0	0	0	NA	NA	NA
ENSBTAG00000039170	*protein_coding*	0	0	0	0	0	NA	NA	NA
ENSBTAG00000039171	*ADAM7*	0	0	0	0	0	NA	NA	NA
ENSBTAG00000039176	*U2*	0	0	0	0	0	NA	NA	NA
ENSBTAG00000039177	*5S_rRNA*	0	0	0	0	0	NA	NA	NA
ENSBTAG00000039178	*HBE4*	0	0	0	0	0	NA	NA	NA
ENSBTAG00000039182	*OR5M3*	0	0	0	0	0	NA	NA	NA
ENSBTAG00000039185	*pseudogene*	0	0	0	0	0	NA	NA	NA
ENSBTAG00000039189	*protein_coding*	0	0	0	0	0	NA	NA	NA
ENSBTAG00000039199	*miRNA*	0	0	0	0	0	NA	NA	NA
ENSBTAG00000039201	*protein_coding*	0	0	0	0	0	NA	NA	NA
ENSBTAG00000039213	*protein_coding*	0	0	0	0	0	NA	NA	NA
ENSBTAG00000039214	*protein_coding*	0	0	0	0	0	NA	NA	NA
ENSBTAG00000039219	*bta-mir-1197*	0	0	0	0	0	NA	NA	NA
ENSBTAG00000039222	*HIST1H2BI*	0	0	0	0	0	NA	NA	NA
ENSBTAG00000039225	*protein_coding*	0	0	0	0	0	NA	NA	NA
ENSBTAG00000039227	*5S_rRNA*	0	0	0	0	0	NA	NA	NA
ENSBTAG00000039235	*miRNA*	0	0	0	0	0	NA	NA	NA
ENSBTAG00000039236	*protein_coding*	0	0	0	0	0	NA	NA	NA
ENSBTAG00000039237	*protein_coding*	0	0	0	0	0	NA	NA	NA
ENSBTAG00000039245	*GK2*	0	0	0	0	0	NA	NA	NA
ENSBTAG00000039248	*protein_coding*	0	0	0	0	0	NA	NA	NA
ENSBTAG00000039253	*OR51F2*	0	0	0	0	0	NA	NA	NA
ENSBTAG00000039256	*protein_coding*	0	0	0	0	0	NA	NA	NA
ENSBTAG00000039257	*protein_coding*	0	0	0	0	0	NA	NA	NA
ENSBTAG00000039272	*protein_coding*	0	0	0	0	0	NA	NA	NA
ENSBTAG00000039281	*protein_coding*	0	0	0	0	0	NA	NA	NA
ENSBTAG00000039285	*processed_pseudogene*	0	0	0	0	0	NA	NA	NA
ENSBTAG00000039289	*BT.64294*	0	0	0	0	0	NA	NA	NA
ENSBTAG00000039298	*protein_coding*	0	0	0	0	0	NA	NA	NA
ENSBTAG00000039300	*OR5B2*	0	0	0	0	0	NA	NA	NA
ENSBTAG00000039304	*U3*	0	0	0	0	0	NA	NA	NA
ENSBTAG00000039306	*protein_coding*	0	0	0	0	0	NA	NA	NA
ENSBTAG00000039310	*SLC22A13*	0	0	0	0	0	NA	NA	NA
ENSBTAG00000039315	*protein_coding*	0	0	0	0	0	NA	NA	NA
ENSBTAG00000039319	*BT.25655*	0	0	0	0	0	NA	NA	NA
ENSBTAG00000039330	*protein_coding*	0	0	0	0	0	NA	NA	NA
ENSBTAG00000039331	*protein_coding*	0	0	0	0	0	NA	NA	NA
ENSBTAG00000039337	*protein_coding*	0	0	0	0	0	NA	NA	NA
ENSBTAG00000039348	*protein_coding*	0	0	0	0	0	NA	NA	NA
ENSBTAG00000039349	*bta-mir-1306*	0	0	0	0	0	NA	NA	NA
ENSBTAG00000039351	*miRNA*	0	0	0	0	0	NA	NA	NA
ENSBTAG00000039354	*protein_coding*	0	0	0	0	0	NA	NA	NA
ENSBTAG00000039362	*BT.47153*	0	0	0	0	0	NA	NA	NA
ENSBTAG00000039363	*pseudogene*	0	0	0	0	0	NA	NA	NA
ENSBTAG00000039369	*5S_rRNA*	0	0	0	0	0	NA	NA	NA
ENSBTAG00000039370	*OR4K1*	0	0	0	0	0	NA	NA	NA
ENSBTAG00000039388	*GPR101*	0	0	0	0	0	NA	NA	NA
ENSBTAG00000039390	*protein_coding*	0	0	0	0	0	NA	NA	NA
ENSBTAG00000039405	*protein_coding*	0	0	0	0	0	NA	NA	NA
ENSBTAG00000039411	*protein_coding*	0	0	0	0	0	NA	NA	NA
ENSBTAG00000039412	*miRNA*	0	0	0	0	0	NA	NA	NA
ENSBTAG00000039416	*protein_coding*	0	0	0	0	0	NA	NA	NA
ENSBTAG00000039422	*OR2B11*	0	0	0	0	0	NA	NA	NA
ENSBTAG00000039441	*RGS9BP*	0	0	0	0	0	NA	NA	NA
ENSBTAG00000039443	*protein_coding*	0	0	0	0	0	NA	NA	NA
ENSBTAG00000039444	*protein_coding*	0	0	0	0	0	NA	NA	NA
ENSBTAG00000039446	*BTRAPPIN-5*	0	0	0	0	0	NA	NA	NA
ENSBTAG00000039448	*protein_coding*	0	0	0	0	0	NA	NA	NA
ENSBTAG00000039449	*miRNA*	0	0	0	0	0	NA	NA	NA
ENSBTAG00000039461	*MS4A15*	0	0	0	0	0	NA	NA	NA
ENSBTAG00000039463	*protein_coding*	0	0	0	0	0	NA	NA	NA
ENSBTAG00000039467	*protein_coding*	0	0	0	0	0	NA	NA	NA
ENSBTAG00000039484	*OR2G2*	0	0	0	0	0	NA	NA	NA
ENSBTAG00000039487	*protein_coding*	0	0	0	0	0	NA	NA	NA
ENSBTAG00000039490	*WFDC8*	0	0	0	0	0	NA	NA	NA
ENSBTAG00000039494	*OR6P1*	0	0	0	0	0	NA	NA	NA
ENSBTAG00000039496	*protein_coding*	0	0	0	0	0	NA	NA	NA
ENSBTAG00000039497	*bta-mir-1256*	0	0	0	0	0	NA	NA	NA
ENSBTAG00000039503	*protein_coding*	0	0	0	0	0	NA	NA	NA
ENSBTAG00000039510	*miRNA*	0	0	0	0	0	NA	NA	NA

Ensembl gene ID	geneName	counts wildtype horn bud	counts polled horn bud	baseMean	baseMean wildtype horn bud	baseMean polled horn bud	log2FoldChange	pval	padj
ENSBTAG00000039511	*protein_coding*	0	0	0	0	0	NA	NA	NA
ENSBTAG00000039517	*protein_coding*	0	0	0	0	0	NA	NA	NA
ENSBTAG00000039533	*protein_coding*	0	0	0	0	0	NA	NA	NA
ENSBTAG00000039534	*protein_coding*	0	0	0	0	0	NA	NA	NA
ENSBTAG00000039540	*protein_coding*	0	0	0	0	0	NA	NA	NA
ENSBTAG00000039546	*protein_coding*	0	0	0	0	0	NA	NA	NA
ENSBTAG00000039554	*protein_coding*	0	0	0	0	0	NA	NA	NA
ENSBTAG00000039559	*BT.86147*	0	0	0	0	0	NA	NA	NA
ENSBTAG00000039562	*OR12D3*	0	0	0	0	0	NA	NA	NA
ENSBTAG00000039564	*pseudogene*	0	0	0	0	0	NA	NA	NA
ENSBTAG00000039565	*miRNA*	0	0	0	0	0	NA	NA	NA
ENSBTAG00000039566	*protein_coding*	0	0	0	0	0	NA	NA	NA
ENSBTAG00000039575	*5S_rRNA*	0	0	0	0	0	NA	NA	NA
ENSBTAG00000039587	*protein_coding*	0	0	0	0	0	NA	NA	NA
ENSBTAG00000039592	*protein_coding*	0	0	0	0	0	NA	NA	NA
ENSBTAG00000039597	*BT.75698*	0	0	0	0	0	NA	NA	NA
ENSBTAG00000039602	*KRTAP13-1*	0	0	0	0	0	NA	NA	NA
ENSBTAG00000039614	*protein_coding*	0	0	0	0	0	NA	NA	NA
ENSBTAG00000039615	*protein_coding*	0	0	0	0	0	NA	NA	NA
ENSBTAG00000039628	*protein_coding*	0	0	0	0	0	NA	NA	NA
ENSBTAG00000039631	*miRNA*	0	0	0	0	0	NA	NA	NA
ENSBTAG00000039633	*protein_coding*	0	0	0	0	0	NA	NA	NA
ENSBTAG00000039643	*BT.89075*	0	0	0	0	0	NA	NA	NA
ENSBTAG00000039645	*protein_coding*	0	0	0	0	0	NA	NA	NA
ENSBTAG00000039647	*BT.43270*	0	0	0	0	0	NA	NA	NA
ENSBTAG00000039651	*protein_coding*	0	0	0	0	0	NA	NA	NA
ENSBTAG00000039653	*protein_coding*	0	0	0	0	0	NA	NA	NA
ENSBTAG00000039654	*pseudogene*	0	0	0	0	0	NA	NA	NA
ENSBTAG00000039659	*miRNA*	0	0	0	0	0	NA	NA	NA
ENSBTAG00000039673	*pseudogene*	0	0	0	0	0	NA	NA	NA
ENSBTAG00000039675	*pseudogene*	0	0	0	0	0	NA	NA	NA
ENSBTAG00000039677	*OR2B6*	0	0	0	0	0	NA	NA	NA
ENSBTAG00000039683	*protein_coding*	0	0	0	0	0	NA	NA	NA
ENSBTAG00000039691	*protein_coding*	0	0	0	0	0	NA	NA	NA
ENSBTAG00000039693	*BT.55086*	0	0	0	0	0	NA	NA	NA
ENSBTAG00000039698	*miRNA*	0	0	0	0	0	NA	NA	NA
ENSBTAG00000039700	*miRNA*	0	0	0	0	0	NA	NA	NA
ENSBTAG00000039704	*miRNA*	0	0	0	0	0	NA	NA	NA
ENSBTAG00000039706	*protein_coding*	0	0	0	0	0	NA	NA	NA
ENSBTAG00000039707	*protein_coding*	0	0	0	0	0	NA	NA	NA
ENSBTAG00000039735	*protein_coding*	0	0	0	0	0	NA	NA	NA
ENSBTAG00000039745	*miRNA*	0	0	0	0	0	NA	NA	NA
ENSBTAG00000039753	*pseudogene*	0	0	0	0	0	NA	NA	NA
ENSBTAG00000039755	*protein_coding*	0	0	0	0	0	NA	NA	NA
ENSBTAG00000039756	*OR10A7*	0	0	0	0	0	NA	NA	NA
ENSBTAG00000039760	*protein_coding*	0	0	0	0	0	NA	NA	NA
ENSBTAG00000039765	*BT.72926*	0	0	0	0	0	NA	NA	NA
ENSBTAG00000039768	*pseudogene*	0	0	0	0	0	NA	NA	NA
ENSBTAG00000039769	*BT.90580*	0	0	0	0	0	NA	NA	NA
ENSBTAG00000039773	*protein_coding*	0	0	0	0	0	NA	NA	NA
ENSBTAG00000039785	*OR11H6*	0	0	0	0	0	NA	NA	NA
ENSBTAG00000039797	*protein_coding*	0	0	0	0	0	NA	NA	NA
ENSBTAG00000039801	*OR9G9*	0	0	0	0	0	NA	NA	NA
ENSBTAG00000039804	*protein_coding*	0	0	0	0	0	NA	NA	NA
ENSBTAG00000039805	*protein_coding*	0	0	0	0	0	NA	NA	NA
ENSBTAG00000039808	*BT.17574*	0	0	0	0	0	NA	NA	NA
ENSBTAG00000039809	*protein_coding*	0	0	0	0	0	NA	NA	NA
ENSBTAG00000039810	*pseudogene*	0	0	0	0	0	NA	NA	NA
ENSBTAG00000039817	*BT.66792*	0	0	0	0	0	NA	NA	NA
ENSBTAG00000039821	*OR4C13*	0	0	0	0	0	NA	NA	NA
ENSBTAG00000039825	*protein_coding*	0	0	0	0	0	NA	NA	NA
ENSBTAG00000039827	*protein_coding*	0	0	0	0	0	NA	NA	NA
ENSBTAG00000039837	*OR5L2*	0	0	0	0	0	NA	NA	NA
ENSBTAG00000039842	*U3*	0	0	0	0	0	NA	NA	NA
ENSBTAG00000039845	*protein_coding*	0	0	0	0	0	NA	NA	NA
ENSBTAG00000039858	*miRNA*	0	0	0	0	0	NA	NA	NA
ENSBTAG00000039864	*SERPINA4*	0	0	0	0	0	NA	NA	NA
ENSBTAG00000039868	*BT.88937*	0	0	0	0	0	NA	NA	NA
ENSBTAG00000039873	*BT.71330*	0	0	0	0	0	NA	NA	NA
ENSBTAG00000039877	*protein_coding*	0	0	0	0	0	NA	NA	NA
ENSBTAG00000039878	*C2ORF78*	0	0	0	0	0	NA	NA	NA
ENSBTAG00000039879	*protein_coding*	0	0	0	0	0	NA	NA	NA

Ensembl gene ID	geneName	counts wildtype horn bud	counts polled horn bud	baseMean	baseMean wildtype horn bud	baseMean polled horn bud	log2FoldChange	pval	padj
ENSBTAG00000039880	*protein_coding*	0	0	0	0	0	NA	NA	NA
ENSBTAG00000039884	*protein_coding*	0	0	0	0	0	NA	NA	NA
ENSBTAG00000039891	*KRT71*	0	0	0	0	0	NA	NA	NA
ENSBTAG00000039893	*OR9I1*	0	0	0	0	0	NA	NA	NA
ENSBTAG00000039901	*OR5V1*	0	0	0	0	0	NA	NA	NA
ENSBTAG00000039906	*miRNA*	0	0	0	0	0	NA	NA	NA
ENSBTAG00000039912	*U1*	0	0	0	0	0	NA	NA	NA
ENSBTAG00000039917	*OR5B3*	0	0	0	0	0	NA	NA	NA
ENSBTAG00000039921	*protein_coding*	0	0	0	0	0	NA	NA	NA
ENSBTAG00000039930	*OR2G2*	0	0	0	0	0	NA	NA	NA
ENSBTAG00000039933	*LRRC3C*	0	0	0	0	0	NA	NA	NA
ENSBTAG00000039944	*protein_coding*	0	0	0	0	0	NA	NA	NA
ENSBTAG00000039964	*protein_coding*	0	0	0	0	0	NA	NA	NA
ENSBTAG00000039970	*BT.66587*	0	0	0	0	0	NA	NA	NA
ENSBTAG00000039973	*BT.63095*	0	0	0	0	0	NA	NA	NA
ENSBTAG00000039983	*protein_coding*	0	0	0	0	0	NA	NA	NA
ENSBTAG00000039986	*protein_coding*	0	0	0	0	0	NA	NA	NA
ENSBTAG00000039989	*miRNA*	0	0	0	0	0	NA	NA	NA
[truncated: 218,980 more chars]
