# Supplementary material for: Independent Polled Mutations Leading to Complex Gene Expression Differences in Cattle
Source: PLoS One. 2014 Mar 26;9(3):e93435. doi: 10.1371/journal.pone.0093435 (PMC3966897; doi:10.1371/journal.pone.0093435)
Supplement: Table S6 — Primer used for genotyping of candidate variants. (PDF) [file pone.0093435.s015.pdf]

Table S6: Primer used for genotyping of candidate variants.

Simmental variant	Ref	Var	forward-Primer	reverse-Primer
Simmental InDel	TTGGTA GGCTGG TATTC	T	TGTAGTGAGAGCAGGCTGGA	GGCAGAGATGTTGGTCTTGG

Holstein variants	Ref	Var	forward-Primer	reverse-Primer
SNP_IFNGR2	G	A	TAGCAAATCACCCTCCCCTA	CGGCGTTTCAACATCTCTTT
SNP 1583414	A	G	CCACTGTGGTGATGTTGACC	TGTTGGAAAATGTATTTGTTGGA
SNP 1596777	G	T	CCCCCAACTCAACCTAAACA	TCCCTGTCCTGTCACTTTCC
SNP 1603747	C	T	TCAACTCACACTGCCTGGAG	GTTCTGTGTGGCCTTGGTTT
SNP 1606620	C	T	TGTGGATAAATACACGCACACAT	GAATTGTGGGACTGCGTGTT
SNP 1607675	G	A	GATGGAGTCTGGCAGAGAGC	ATGGGCTCTTTCTTCCTGGT
SNP 1618261	T	C	TCCCCTCTTTTTAAGACTTCTTTC	AGATCTGGGGGAGAGAAGGA
SNP 1623157	G	A	ATCTAGCACAGCCCTGGAGA	AGCTTTAGATCCTTCAAAAATTGAT
SNP 1623573	G	C	AATCAATTTTTGAAGGATCTAAAGCTA	TGCAGGAGACAAAGGAATCA
SNP 1624279	C	T	ACACCCGGTTAACTGTCCTG	TGTATCCGACCACCAGAAAA
SNP 1624522	T	G	ACACCCGGTTAACTGTCCTG	TGTATCCGACCACCAGAAAA
SNP 1624555	T	C	ACACCCGGTTAACTGTCCTG	TGTATCCGACCACCAGAAAA
SNP 1626030	T	C	TTTCCGGAACCAAATCTCAG	TACGGCCTTACAAACCCATC
SNP 1626065	G	C	TTTCCGGAACCAAATCTCAG	TACGGCCTTACAAACCCATC
SNP 1628090	A	G	CCGGGGCTTATACTTTACCC	TTGCATGAAATATTCCCTTGG
SNP 1631299	A	G	ACACCAGTTTGACCCAGCTC	CCAGGGAAGGCTGTCTCTTT
SNP 1631667	C	T	ACACCAGTTTGACCCAGCTC	CCAGGGAAGGCTGTCTCTTT
SNP 1631762	G	A	ACACCAGTTTGACCCAGCTC	CCAGGGAAGGCTGTCTCTTT
SNP 1631895	T	C	ACACCAGTTTGACCCAGCTC	CCAGGGAAGGCTGTCTCTTT
SNP 1633226	T	A	GAAAGCAGTGGGGATGTAGC	AAGTTTGCAGCTTGAAGGAGA
SNP 1633690	C	T	TGACACAGGCTGAGTTCCTG	CTCTGAAAAGTGCCCGCTAC
SNP 1635514	G	C	AGGGATGGGTATTTTTAAGATGG	CGTCTGGAAGCCTTTGACAT
SNP 1639632	A	G	TTAAGGTACCCAGGCTATGGTG	ATCCTCATGATGGAGGGTGA
SNP 1640658	G	T	ACCCAGGGACATGAAGACAG	CACCCTGGAAAACTTCCAAA
SNP 1640815	T	C	ACCCAGGGACATGAAGACAG	CACCCTGGAAAACTTCCAAA
SNP 1645083	G	T	GAGATTGGTCCCAAGGTCAG	CTGGGGCAAGTGGACTAGAG
deletion 1645296	AT	A	GAGATTGGTCCCAAGGTCAG	CTGGGGCAAGTGGACTAGAG
SNP 1645301	T	A	GAGATTGGTCCCAAGGTCAG	CTGGGGCAAGTGGACTAGAG
InDel 1649163	CGCATC A	TTCTCAG AATAG	GCAGAAATGAAACATCTGTCCA	CACAGCAGAGGGACAAGTCA
SNP 1651491	C	T	CCTCCGGACCCAGACTCTA	CCTGAAAAGAACAATCCTCTGC
SNP 1654405	G	A	CGCTCCAAATTCATACGTCA	GCATTGTTTTCAGGGCATTT
SNP 1655463	C	T	TTCCTATTGTGCCAGGTGTG	GGGTTGCCCTCTGTTTTGTA
SNP 1672807	G	A	TGATGACCCTCAGTGAGCAA	TGGAGAGAACAGGTGCTGTG
SNP 1676520	C	T	CTGCCCATCAAAACAAGACC	CAGCTTTACTGGTTGGAGCA
SNP 1679832	C	T	TTTGGAGAGTGGTCTCAGGAA	TCAGGCTCAGGGAGGTAATG
SNP 1680646	T	C	CTCCACCCACTCCATTCATC	CTCGCTGCTCATCTGCTATG
SNP 1684055	G	C	TGGCATCACTGACTTGATGG	GAGCCATATGAGGTGGTTCC
SNP 1764239	T	C	ATCTCCCAGTGGTGAGTTGC	CCTTGGGGATGAATGCTTTA
SNP 1768587	C	A	CACACACCTCCCTCACACAC	TCAGAGACAGTTATGGTCAGGAG
SNP 1855898	G	A	AGCAATGAGACAAAATGTCACC	TCCATCTAATCTCCCCCTGTT
80 kb Duplikation	ref	Dup	CCATCTTGGGTACAGCGTTT	TGTTCTGTGTGGGTTTGAGG
