# Supplementary material for: Independent Polled Mutations Leading to Complex Gene Expression Differences in Cattle
Source: PLoS One. 2014 Mar 26;9(3):e93435. doi: 10.1371/journal.pone.0093435 (PMC3966897; doi:10.1371/journal.pone.0093435)
Supplement: Table S7 — Primer used for qRT-PCR. (PDF) [file pone.0093435.s016.pdf]

Table S7: Primer used for qRT-PCR.

gene	forward primer	reverse primer
OLIG1	CATCATCGCGACAAAACATC	AATTCCCAGGTCGATGAGTG
OLIG1	GTGGCGATCTTGGAGAGC	GAGAGGAAGCGGATGCAC
OLIG2	CCTGGGTCCTCAGAGCTTC	CCGGTTCTCTGCCTCAGTC
LOC100848215	GGAAACCTGTTTGCTTTTCAA	CAGCAACTGTTTGGGTTCTG
C1H21orf62	ACCAGCTGTCAAGCCTGAGT	CAGAAGGAGGTGCCATTCTC
FOXL2	CCGGCATCTACCAGTACATTATAGC	GCACTCGTTGAGGCTGAGGT
RXFP2	AGAACCCCACAATCCAGATG	TGACCCTGGAGAAGTTCCTG
GART	GCCCGAGTACTTGTCATTGG	CCTGGGGTAACCAACACTTG
HPRT	GAACGGCTGGCTCGA	TCCAACAGGTCGGCAAAGAA
RPLP0	AGGAAGCGGGAATGCAGAGT	TCTCCTTCGGGCTGGTCAT
